# Nutritional Characterization and Untargeted Metabolomics of Oyster Mushroom Produced Using *Astragalus membranaceus* var. *mongolicus* Stems and Leaves as Substrates

**DOI:** 10.3389/fpls.2022.802801

**Published:** 2022-02-03

**Authors:** Xu Zeng, Jiaxue Li, Xinkai Lyu, Xiao-Mei Chen, Shunxing Guo

**Affiliations:** Institute of Medicinal Plant Development, Chinese Academy of Medical Sciences and Peking Union Medical College, Beijing, China

**Keywords:** untargeted metabolomics, nutrition, stems and leaves, *Astragalus membranaceus* var. *mongolicus*, *Pleurotus* spp.

## Abstract

*Astragalus membranaceus* var. *mongolicus* (AMM) is an edible and medicinal material and is commonly used in East Asia. According to the pharmacopeia of China, the dried root of AMM is medicinal. However, the aerial parts of AMM are always directly discarded after harvest. The stems and leaves are also rich in active compounds, including saponins, flavonoids, terpenoids, and polysaccharides. To rationally use resources, waste products from AMM stems and leaves are useful substrates for edible fungus cultivation. Here, oyster mushroom (*Pleurotus ostreatus* var. *florida*) was cultivated on a basal substrate supplemented with AMM stems and leaves (AMM group). The nutritional and chemical composition of the fruiting body were analyzed by metabolomics and chemometrics. Our results showed that AMM addition to the substrate affected the fresh weight, moisture, fat, protein, and element concentrations, and amino acid composition of oyster mushroom. Moreover, 2,156 metabolites were detected and annotated based on the metabolomics data, of which 680 were identified as differentially expressed metabolites. Many active phytometabolites previously identified in AMM herbs were also detected in the metabolomics of oyster mushroom from AMM group, including 46 terpenoids, 21 flavonoids, 17 alkaloids, 14 phenylpropanoids, and 3 fatty acids. In summary, our results imply that oyster mushroom cultured with AMM stems and leaves might have very high nutritional therapy health care value.

## Introduction

*Astragalus membranaceus* Bge. var. *mongolicus* (Bge.) Hsiao (AMM) is a perennial herbaceous plant from the legume family. The root of AMM has been known for thousands of years to be a traditional Chinese medicine (Astragali Radix). The main herbal material and its botanical product are widely used in hospitals (Chen et al., 2015). In addition, this herb is also a traditional food and flavoring in Supplementary Material. In recent years, the list of substances that are traditionally both food and traditional medicinal materials was formulated and published by the National Health Commission of China. AMM is one of 9 model substances on that list and is recommended for boiling, braising, and soaking in wine.

According to the Chinese pharmacopeia 2020 edition, the dried root of AMM is used to enhance people’s health and reinforce vital energy. Physiological functions such as immunomodulation, cardioprotective, antiperspirant, antidiuretic, antitumor, antioxidation, and anti-inflammatory effects have been widely reported (Cho and Leung, 2007; Kuo et al., 2009; Ye et al., 2009; Auyeung et al., 2016). Therefore, AMM is one of the major herbal materials with the largest market demand in East and South Asia. To date, approximately 150 components have been identified in AMM herbs (Mamedova and Isaev, 2004). The major bioactive metabolites are a variety of saponins, flavonoids, terpenoids, polysaccharides, amino acids, etc. Indeed, calycosin and astragaloside IV, types of flavonoids and triterpenoid saponins, were also used as standard compositions to evaluate the quality of AMM harvests. Calycosin-7-O-b-D-glucoside, astragaloside IV, and other natural ingredients are always considered the major bioactive constituents (Ma et al., 2003; Wu et al., 2005).

The annual output of AMM roots amounts to 50,000 tons, whereas its aerial parts, i.e., the stems and leaves, are always directly discarded after harvest. This leads to resource waste, enormous economic loss and serious environmental pollution. Previous study has shown that the aerial parts of AMM contain similar types of bioactive substances to those of the roots (Zhang, 2010). Therefore, it is reasonable and urgent to find methods of efficiently using AMM stems and leaves and generating value as food or health products. Some studies reported that AMM stems and leaves were used as a feed additive in the poultry and livestock industry (Xi et al., 2014; Adams et al., 2018). They suggested that feeding incorporated diets with appropriate AMM stems and leaves significantly increased growth performance, strengthened the immune system, improved antioxidative status, and regulated the intestinal microflora of quails. Here, our study was designed to evaluate the potential application of AMM stems and leaves in the edible fungus industry.

The mushrooms of the genus *Pleurotus* occupy the third position in the production of edible mushrooms, behind the species of the genera *Agaricus* and *Lentinula*. They have extremely high nutritional and medicinal values. Modern pharmacological study has shown that fungal polysaccharides in oyster mushrooms have biological properties such as improving immunity and antitumor, antioxidant and cholesterol-lowering activities (Jayakumar et al., 2011). Furthermore, these species have quick mycelial growth and fruiting and a low cost of culture, are slightly affected by diseases, and require minimal monitoring of the cultivation environment due to easy adaptation and maintenance. It has been reported that the total yield of oyster mushrooms in 2019 reached 300,000 tons (data from the China Edible Fungi Association).

The production of oyster mushrooms has been tested using different substrates, e.g., cotton waste textile, rice straw, husk of coffee, wheat straw, and sugarcane (Ramos et al., 2011; Naozuka et al., 2012; Fernandes et al., 2015). The adaptation of these mushrooms to new wastes represents one of the main methods for the bioconversion of agro-industrial waste into edible products with high nutritional value. In this study, a commercial strain of oyster mushrooms (*Pleurotus ostreatus* var. *florida*) was produced using a basal substrate supplement with AMM stems and leaves. Then, its nutritional and chemical composition were validated.

## Materials and Methods

### Standards and Reagents

Acetonitrile, methanol and chloroform were HPLC grade from Fisher Chemical Co., Inc. (Geel, Belgium). Reference standard astragaloside IV (CAS No. 84687-43-4) was purchased from Yuanye Bio-Technology Co., Ltd. (Shanghai, China). All other chemicals and solvents were analytical grade and purchased from common sources.

### Samples

**FIGURE 1:**
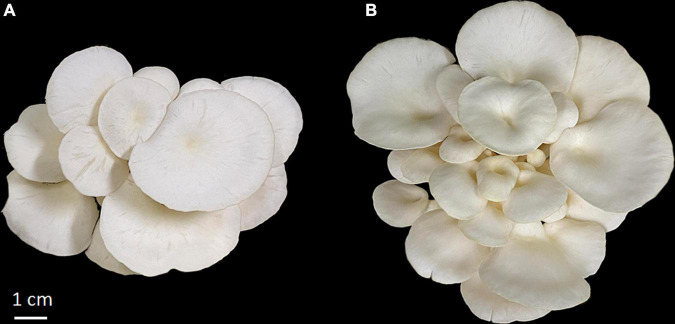
The fruiting body of *P. ostreatus* var. *florida* used in this study. **(A)** Sample from control group; **(B)** sample from AMM group.

The mycelium of *P. ostreatus* var. *florida* was a commercial strain (Xiaobaiping, ITS sequencing, Genbank accession OL905953) purchased from Zehai Edible Fungus Specialized Cooperative (Shandong, China) and further grown on basal substrates. The basal substrates used were 75% corn cob, 20% wheat bran, 2% gypsum powder for food, 1% soybean flour, 1% white sugar, and 1% calcium superphosphate. After harvesting AMM roots, the stems and leaves were collected from a planting base at Shanxi Zhendong Pharmaceutical Co., Ltd. (Changzhi, China). All materials were dried and crushed to coarse powder. The cultivation substrate in the AMM group was prepared from a mixture of 250 g of basal substrate and 250 g of coarse powder of AMM stems and leaves, with a moisture content of 65%. In the control group, 500 g of basal substrates were used without any addition. Each substrate (500 g) was poured into a bag of HDPE (high-density polyethylene) and autoclaved in a pressure cooker at approximately 120°C for 180 min. Physicochemical analysis of the substrates was performed by a qualified third institutions (Nanjing Convinced-Test Technology Co., Ltd). Referring to previous studies (Fernandes et al., 2015; Li et al., 2020), the substrate was mixed with the spawn (∼3 g of *P. ostreatus* var. *florida* grown on basal substrate) in a clean bench. Then, the inoculated bags were kept at 25°C in the dark to encourage mycelial growth. After the mycelium has grown throughout the substrate within 25 days, hole (one per bag) of about 3 cm diameter was made in the side of the bag, which was then placed in a room with a temperature of 10–15°C and a relative humidity of about 80–85%. After the first fruiting, the fruiting bodies of oyster mushrooms were collected over a total period of 75 days (Figure 1). Six replicates from each group were collected and immediately chilled in liquid nitrogen. All samples were stored at −80°C until metabolomic analysis.

### Nutrient Contents

A subset of 6 powder samples was used to determine the chemical composition of mushrooms by following the National Standards of the People’s Republic of China (NSPRC). The protein, fat and total saccharide contents in mushroom powders were determined by the Kjeldahl method (NSPRC GB 5009.5-2016), acid hydrolysis method (NSPRC GB 5009.6-2016), and phenol-sulfuric acid method (NSPRC GB/T 15627-2009), respectively. The amino acid component was estimated by an automatic amino acid analyzer (NSPRC GB/T 15627-2009). Se, Fe, Mn, and Zn determination were performed by inductively coupled plasma mass spectrometry (ICP-MS, NSPRC 5009.268-2016) and inductively coupled plasma optical emission spectrometry (ICP-OES, NSPRC 5009.13-2017).

### Metabolism Analysis

The groups were designated the AMM and control groups. From each group, six biological replicates were assayed for sample extraction and metabolomics analysis. Metabolite extraction and ultraperformance liquid chromatography and mass spectrometry (UPLC-MS) were performed according to the description in previous reports (Xu et al., 2020; Ma et al., 2021). In brief, 50 mg of powder from each sample was precisely weighed and ultrasonically extracted with methanol buffer (0.4 mL, 80% aqueous) with 20 μL of internal standard (0.3 mg/mL 2-chloro-D-phenylalanine) for 30 min and incubated for 30 min at −20°C. After centrifugation at 13,000 × g for 15 min, 200 μL of the supernatant was transferred to vial for UPLC-MS analysis. In addition to test samples, pooled quality control (QC) samples were prepared by combining 10 μL supernatant from each test sample. To monitor the stability and repeatability, QC samples were injected every six test samples throughout the analytical run to obtain a set of data to assess repeatability.

All chromatographic separations were performed using a UPLC system (Waters, Milford, United States). An Acquity BEH C18 column (100 mm × 2.1 mm i.d., 1.7 μm; Waters, Milford, United States) was used. Solvent A was aqueous formic acid (0.1%, v/v, formic acid), and solvent B was acetonitrile (0.1%, v/v, formic acid). The injection volume was 2.00 μL, and the column temperature was set at 40°C. Separation was achieved using the following gradient: 5–20% B over 0–3 min, 20–95% B over 3–9 min, and 95% B over 9–13 min, ramped linearly to 5% B at 13.1 min, and maintained at 5% B until 16 min at a flow rate of 0.40 mL/min. The MS data was collected using an Q Exactive HF-X mass spectrometer (Thermo Fisher Scientific, San Jose, CA) equipped with an electrospray ionization (ESI) source operating in either positive or negative ion mode. The conditions of mass spectrometry were as follows: spray voltage (+), 3,500 V; spray voltage (−), 2,800 V; capillary temperature, 320°C; sheath gas, 40 arb; auxiliary gas, 10 arb; probe heater temperature, 400°C; normalized collision energy (NCE), 20–40–60 V; and mass spectrum scanning range: 70–1,050 m/z.

According to the method described by previous studies (Xu et al., 2020; Ma et al., 2021), Progenesis QI (Waters Corporation, Milford, United States) was used to convert raw mass spectrum data for baseline filtration, peak identification, integration, retention time correction, peak alignment, and uniformization. Finally, we obtained a data matrix (.xls) that contained the retention time, mass charge ratio, and peak intensity. Subsequently, SIMCA-P + 14.0 was performed for cluster analysis by the PCA, PLS-DA, and OPLS-DA methods. Differential metabolites were identified by a statistically significant threshold of variable influence on projection (VIP) values obtained from the OPLS-DA and Student’s *t*-test (*P*-value) of the raw data. The metabolites with VIP > 1, *P* < 0.05 and fold change > 2 or < 0.5 were considered statistically significant. To explain the physical and chemical properties and biological functions of metabolites, the online METLIN,^1^ Human Metabolome,^2^ KEGG databases (using MetaboAnalyst)^3^ and metabolomic data (Wu et al., 2020) of *A. membranaceus* var. *mongolicus* (published data annotated by MassBank and Bio-PubChem) were used for identification and annotation.

### Determination of Astragaloside IV

Referring to the method recorded in the Chinese Pharmacopeia (2020 edition) and previous studies (Zu et al., 2015; Kafle et al., 2020), dried mushroom (10 g) were used for astragaloside IV extraction by the heat reflux method. Briefly, the powders were transferred to a round-bottom flask with 30 mL/g of methanol for samples. Extraction proceeded at 80°C. After 6 h, the solution was dried in a water bath and redissolved in 30 mL of water. Then, astragaloside IV was extracted by water-saturated n-butanol, supernatant extracts were combined and washed with 40% aqueous ammonia, and the butanol solvent was removed in a rotary evaporator at 55°C. The resulting dry extracts were diluted in 20 mL of methanol. The sample was further diluted 100-fold and filtered through a 0.22-μm membrane before injection into the UPLC-MS/MS system for analysis.

The reference standard astragaloside IV was applied to help identify the chromatography peaks. The standard chemicals were accurately weighed and dissolved in methanol to a concentration of 1 mg/mL. Stock solutions with final concentrations of 0.1 mg/mL were prepared in methanol, and the standard dilutions were 50, 125, 250, 500, 1,000, 2,500, and 5,000 ng/mL.

The UPLC system consisted of an LC-20A system (Shimadzu, Japan) equipped with an automatic degasser, a quaternary pump, and an autosampler. Chromatographic separation was achieved using a Waters XBridge Shield RP18 (4.6 mm × 150 mm; 5 μm) column at room temperature, and the flow rate was 0.4 mL/min. Then, 2 μL injections were eluted by a mixture of acetonitrile and 0.1% aqueous formic acid (60:40, v/v). The column effluent was monitored using a 5500 Q-TRAP^®^ LC-MS/MS system (AB Sciex, Canada). Astragaloside IV was achieved in positive ESI mode. Multiple reaction monitoring scanning was employed for quantification. The parameters were optimized and listed as follows: electrospray voltage, 5,500 V; curtain gas (CUR), 35 psi; nebulizer gas (GS1), 10 psi; heater gas (GS2) 0 psi; declustering potential (DP), 100 V; entrance potential (EP), 10 V; collision energy (CE), 30 eV; and collision cell exit potential (CXP), 13 V. The LC-MS/MS analysis of astragaloside IV produced a positive ion of m/z 785.6, corresponding to the [M + H]^+^ precursor ion. Likewise, the mass transition patterns m/z 785.6 + 473.5 and 785.6 + 143.2 were selected for the identification and quantification of astragaloside IV. Between-group comparisons were analyzed by Student’s *t*-test for unpaired data using SPSS 19.0. Differences at *p* ≤ 0.05 were considered significant.

## Results

### Nutrients

Nutritional values of oyster mushrooms cultivated on different substrates are shown in Table 1. After the first flush, the fruit bodies obtained in the AMM group gave higher fresh weight (75.33 ± 7.57 vs. 60.93 ± 13.12 g) and lower moisture level (86.69 ± 2.26 vs. 82.27 ± 1.48 g/100 g) than the control. The samples obtained from the AMM group gave lower fat contents than the control (2.31 ± 0.13 vs. 3.27 ± 0.35 g/100 g). The protein contents in the samples from the AMM group were obviously higher than those in the control (29.67 ± 3.55 vs. 16.08 ± 0.96 g/100 g). Nevertheless, the sample obtained from the AMM group gave similar carbohydrate contents to the control. No significant differences between the total saccharide levels of the samples were found for the two groups.

**TABLE 1:** Nutritional value, amino acid content and elemental concentrations of *P. ostreatus* var. *florida* (mean ± SD).

	Control	AMM group
**Nutritional value**		
Fresh weight (g)	60.93 ± 13.12	75.33 ± 7.57*
Moisture (g/100 g)	86.69 ± 2.26	82.27 ± 1.48*
Protein (g/100 g)	16.08 ± 0.96	29.67 ± 3.55*
Fat (g/100 g)	3.27 ± 0.35	2.31 ± 0.13*
Total saccharide (%)	51.9 ± 3.45	46.41 ± 7.74
**Amino acid (g/100 g)**		
Aspartic acid	1.81 ± 0.02	1.81 ± 0.02
Threonine	0.8 ± 0.02	0.81 ± 0.01
Serine	0.79 ± 0.05	0.75 ± 0.04
Glutamine	2.52 ± 0.07	2.84 ± 0.11*
Proline	0.71 ± 0.01	0.71 ± 0.01
Glycine	0.76 ± 0.01	0.81 ± 0.02*
Alanine	1.02 ± 0.01	1.07 ± 0.01*
Valine	0.76 ± 0.01	0.83 ± 0.01*
Methionine	0.26 ± 0.01	0.20 ± 0.01*
Isoleucine	0.64 ± 0.01	0.69 ± 0.01*
Leucine	1.23 ± 0.05	1.26 ± 0.06
Tyrosine	0.52 ± 0.04	0.53 ± 0.06
Phenylalanine	0.75 ± 0.02	0.79 ± 0.03
Lysine	1.08 ± 0.01	1.11 ± 0.01
Histidine	0.38 ± 0.01	0.43 ± 0.01*
Arginine	0.93 ± 0.01	1.09 ± 0.01*
Total amino acid	14.97 ± 0.06	15.7 ± 0.10*
**Mineral (mg/kg)**		
Se	0.2096 ± 0.0081	0.2654 ± 0.0056*
Fe	49.96 ± 0.81	45.04 ± 0.75*
Mn	9.54 ± 041	10.21 ± 0.24*
Zn	72.30 ± 3.86	79.60 ± 2.40*

The results are expressed on a dry weight basis from the first flush except for the fresh weight, amino acid content and moisture (* = statistically significant, p < 0.05).

In this study, a total of 16 amino acids were recorded in *P. ostreatus* var. *florida* (Table 1). Overall, the total amino acids of samples from the AMM group were higher than those of samples from the control group (15.7 ± 0.10 vs. 14.97 ± 0.06 g/100 g). Moreover, the amino acid profiles of the two groups were slightly different. In terms of individual amino acids, the three most abundant amino acids found in *P. ostreatus* var. *florida* mushrooms were glutamic acid, aspartic acid, and leucine.

AMM stem and leaf addition to the substrate affected the concentrations of elements in the mushrooms produced from the first flush. The Se, Mn, and Zn concentrations in the AMM group were higher than those in the control group (Table 1). However, the Fe values were lower than those found in the control group.

### Overview of Metabolomics

Mass spectral data were processed by alignment of all data sets from the samples in the AMM group (each with six biological replicates) and controls. After removing redundancy and noise signals, indicative of poor quality or non-biological origin, 5,653 and 7,680 spectral signals were retained for positive and negative ion modes, respectively (Supplementary Table 1). Annotation of metabolites from *P. ostreatus* var. *florida* samples was performed by comparison of the accurate m/z values. Taken together, 2,156 metabolites were annotated by our integrated bioinformatics pipeline (Supplementary Table 2).

**FIGURE 2:**
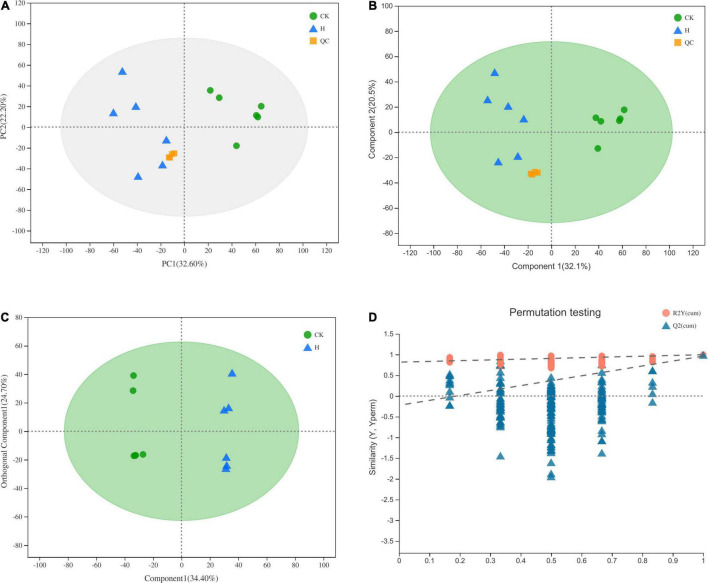
PAC **(A)**, PLS-DA **(B)**, OPLS-DA **(C)** score plots and OPLS-DA model permutation test **(D)** of metabolites for comparison between AMM and control group.

In the PCA and PLS-DA score plots (Figures 2A,B), quality control (QC) samples were clustered together, suggesting that this method has good stability and reproducibility. Typical base peak intensity chromatograms of QC samples were different for the two groups (Supplementary Figure 1). To understand the major differences between the metabolite levels of the two groups, we conducted OPLS-DA (Figure 2C) and determined the featured metabolites. For differences between the two groups, the tissue samples were well separated in the model (Figure 2D). The R^2^X, R^2^Y (goodness-of-fit parameter) and Q^2^ (predictive ability parameter) of the OPLS-DA model are 0.711, 0.996, and 0.962, respectively, indicating the good quality and high confidence of our model. The significantly differential metabolites in the AMM and control groups were well separated based on the criteria VIP > 1, *P* < 0.05 and fold change > 2 or < 0.5. In the comparison of the AMM and control groups (Supplementary Table 3), the relative peak areas of 520 and 160 annotated metabolites were significantly increased and decreased, respectively.

**FIGURE 3:**
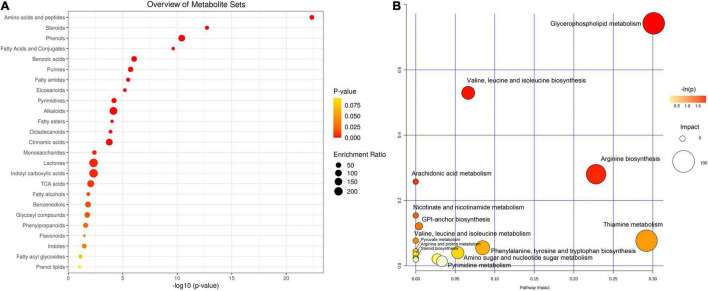
Functional classification and annotation of differential metabolites identified in the comparison between AMM and control group. **(A)** The KEGG classification of differential metabolites; **(B)** KEGG pathway enrichment of differential metabolites.

As shown in Figure 3A, most of the differential metabolites in the comparison were categorized into 20 classes. Judging from the proportion of metabolites in each class out of the total metabolites, amino acids and peptides, steroids, phenols, fatty acids, and benzoic acids were the most active metabolites. In addition, alkaloids, phenylpropanoids, and flavonoids were also widely detected in *P. ostreatus* var. *florida* from the AMM group. These metabolites were mainly identified in AMM herbs. Moreover, to understand the metabolic activities in *P. ostreatus* var. *florida* obtained from AMM group, we mapped the differential metabolites to KEGG pathways (Figure 3B). In comparing *P. ostreatus* var. *florida* from the AMM group to that from the control, most of the differential metabolites were involved in glycerophospholipid metabolism, arachidonic acid metabolism, thiamine metabolism, and amino acid metabolism, such as valine, leucine, and isoleucine biosynthesis, arginine biosynthesis, and phenylalanine, tyrosine and tryptophan biosynthesis. As shown in Supplementary Table 4, numerous metabolites identified as glutamine, glycine, alanine, leucine, isoleucine, histidine, and arginine significantly increased in *P. ostreatus* var. *florida* from the AMM group. Our metabolomics agree with the results described above from amino acid composition analysis. In addition, the “steroid biosynthesis” and “aminoacyl-tRNA biosynthesis” pathways of the AMM and control groups were also significantly different. In summary, differential clustering of AMM-group samples against the control sample suggested that AMM addition to the substrate could change metabolites in the fruiting bodies of *P. ostreatus* var. *florida*.

**FIGURE 4:**
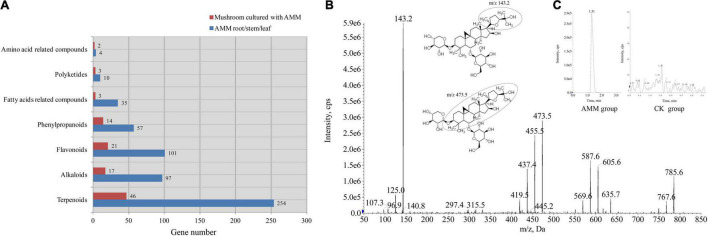
Classification and identification of differential phytometabolites. **(A)** Differential phytometabolites in our study and major phytometabolites in AMM herbs; **(B)** product ion mass spectra of [M + H]^+^ ion of astragaloside IV; and **(C)** chromatography peak of a representative AMM (1.31 min, astragaloside IV) and control sample.

In metabolomic studies (Li et al., 2019; Wu et al., 2020), a large number of pythometabolites were identified in AMM herbs. In our study, we also found an abundance of phytometabolites from *P. ostreatus* var. *florida* cultured with AMM stems and leaves. Most metabolites previously identified in AMM herbs were included in our mushroom data. In summary, the differential metabolites identified in the comparison mainly included terpenoids, flavonoids, alkaloids, and phenylpropanoids. Differential phytometabolites were shown in Figure 4 and Table 2.

**TABLE 2:** Differential pythometabolites for comparison between AMM and control group.

Metab_ID	Retention_Time	Apex_m/z	Mode	Adduct	VIP	Log2(Fold_Change)	*P*_Value	Putative_Metabolite	Putative_Formula	Pubchem_ID
**Terpenoids**										
metab_14941	0.53	783.11	neg	[M–H] −	1.91	3.87	0.03	Astragaloside IV or III	C41H68O14	441905
metab_7914	0.60	829.30	neg	[M + HCOO] −	1.25	3.81	0.00	Astragaloside IVor III	C41H68O14	13943297
metab_758	8.08	943.64	pos	[M + H] +	1.99	3.34	0.02	Soyasaponin I	C48H78O18	122097
metab_487	2.83	291.09	pos	[M + H] +	2.66	8.11	0.00	Plumericin	C15H14O6	5281545
metab_4742	4.91	235.17	pos	[M + H] +	1.38	3.08	0.01	Confertifolin	C15H22O2	442187
metab_5602	2.02	235.17	pos	[M + H] +	1.99	4.27	0.00	Confertifolin	C15H22O2	442187
metab_8507	1.82	301.18	neg	[M–H] −	1.14	2.57	0.00	Abietic acid	C20H30O2	10569
**Flavonoids**										
metab_2580	6.30	517.24	pos	[M + H] +	1.16	12.98	0.02	Formononetin 7-O-glucoside-6″-O-malonate	C25H24O12	23724663
metab_8081	1.08	515.20	neg	[M–H] −	1.07	4.57	0.02	Formononetin 7-O-glucoside-6″-O-malonate	C25H24O12	23724663
metab_14219	1.54	267.02	neg	[M–H] −	1.62	2.37	0.03	Formononetin	C16H12O4	5280378
metab_1163	0.51	287.06	pos	[M + H] +	1.07	1.64	0.02	Kaempferol	C15H10O6	5280863
metab_5123	3.24	449.28	pos	[M + H] +	1.89	12.32	0.00	Kaempferol-3-O-glucoside	C21H20O11	5282102
metab_8263	1.44	445.14	neg	[M–H] −	1.67	2.47	0.02	Calycosin 7-O-Glucoside	C22H22O10	5318267
metab_6222	0.84	285.14	pos	[M + H] +	1.70	3.71	0.03	Glycitein	C16H12O5	5317750
metab_6222	0.84	285.14	pos	[M + H] +	1.70	3.71	0.03	Biochanin A	C16H12O5	5280373
metab_14044	1.79	283.06	neg	[M–H] −	1.42	1.04	0.04	Biochanin A	C16H12O5	5280373
metab_9173	3.65	269.05	neg	[M–H] −	1.28	7.28	0.03	Genistein	C15H10O5	5280961
**Alkaloids**										
metab_6362	0.60	138.05	pos	[M + H] +	1.96	6.21	0.00	Trigonelline	C7H7NO2	5570
metab_1296	0.60	138.05	pos	[M + H] +	1.97	4.09	0.00	Trigonelline	C7H7NO2	5570
metab_1681	1.71	370.20	pos	[M + H] +	1.52	5.49	0.00	Corydaline	C22H27NO4	101301
metab_5698	1.80	180.14	pos	[M + H] +	2.46	14.91	0.00	(−)-Salsolinol	C10H13NO2	91588
metab_6048	1.19	352.15	pos	[M + H] +	1.51	4.52	0.00	Palmatine	C21H21NO4	19009
**Phenylpropanoids**										
metab_7983	0.78	145.04	neg	[M–H] −	1.30	2.61	0.04	Coumarin	C9H6O2	323
metab_5690	1.81	147.05	pos	[M + H] +	1.88	1.31	0.01	Coumarin	C9H6O2	323
metab_5244	2.87	193.09	pos	[M + H] +	1.62	3.47	0.00	Sinapyl alcohol	C11H14O4	5280507
metab_2362	4.64	225.11	pos	[M + H] +	1.39	11.86	0.03	Sinapic acid	C11H12O5	637775
**Polyketides**										
metab_6222	0.84	285.14	pos	[M + H] +	1.70	3.71	0.03	Acacetin	C16H12O5	5280442
**Amino acid related compounds**										
metab_4395	6.84	306.24	pos	[M + H] +	1.09	11.20	0.03	Capsaicin	C18H27NO3	1548943

### Detection of Astragaloside IV

As shown in Figures 4B,C and Supplementary Figure 2, astragaloside IV was identified from the standard reference by analyzing the ion fragmentation patterns. These components were not detected in any of the samples from the control group. However, the content of astragaloside IV in samples from the AMM group was 12.22 ± 0.54 mg/kg (∼0.0012%). As mentioned above, similar results were reported by our metabolomic data.

### Substrates

Physicochemical analyses of the substrates from AMM and control groups were shown in Supplementary Table 5. The results suggested that the basal substrate supplemented with AMM stems and leaves was more abundant in the amount of lignin, macronutrients C and N, micronutrients K, Ca, Mg, S, P, Fe, and Mo. However, there were no different in the amount of cellulose and hemicellulose, and most of physical parameter, such as water holding capacity, porosity, venting quality and total density between the substrates of AMM and control group.

## Discussion

### Nutrients

Oyster mushroom was cultured with a basal substrate supplement with AMM stems and leaves. The fruit bodies obtained in the AMM group presented a higher fresh weight and lower moisture than the control. The moisture values obtained from previous studies are as follows: 88.1 g/100 g for oyster mushroom cultivated in banana straw (Bonatti et al., 2004); 89.2 g/100 g in a commercial sample of oyster mushroom (Reis et al., 2012); and 90.3 g/100 g cultivated in blank paper scraps (Fernandes et al., 2015). Here, our results suggested that AMM stems and leaves were able to promote the growth of *P. ostreatus* var. *florida* and increase the dry weight.

The samples from the AMM group presented lower fat contents, higher protein contents. In our study, the total fat of oyster mushroom was lower than the values reported by Bonatti et al. (2004): 5.97 and 6.32 g/100 g in samples cultivated on banana and rice straw, respectively. However, the total fat of *P. ostreatus* cultivated in blank and printed paper scraps was as low as 1.18 and 1.68 g/100 g, respectively, as reported by Aguilar-Rivera et al. (2012). Moreover, the protein contents described in previous studies are as follows: 19.9–34.7 g/100 g in *P. ostreatus* cultivated on wheat straw supplemented with sugar beet (Manzi et al., 1999); 21 g/100 g in samples cultivated on wheat straw (Patil et al., 2010); 9.62 g/100 g in oyster mushrooms cultivated on coffee husks (Fan et al., 2000); and 9.29 g/100 g in fruiting bodies cultivated on paper scraps (Aguilar-Rivera et al., 2012). Here, our results revealed that substrates supplemented with AMM stems and leaves resulted in the accumulation of proteins in *P. ostreatus* var. *florida.*

The protein content and amino acid composition of mushrooms are widely reported in previous studies. Our result agreed with the reports of Manzi et al. (1999) and Mendez et al. (2005). Among them, comparing between the two groups shows that oyster mushroom from the AMM group contained more glutamine, glycine, alanine, valine, isoleucine, histidine, and arginine and less methionine. Interestingly, mushrooms from the AMM group contained significantly higher levels of glutamine than the control samples. Our results implied that AMM stem and leaf addition to the substrate could contribute significantly to the overall protein content and amino acid distribution of oyster mushrooms.

The concentrations of Se, Fe, Mn, and Zn (Table 1) in our study were similar to those found in *Pleurotus* spp. (Kalac, 2009; Naozuka et al., 2012). For example, *P. ostreatus* mushrooms were cultivated in coffee husks enriched with 70 mg/kg of Zn, 10 mg/kg of Mn, and 0.12 mg/kg of Se. Our study suggested that AMM stem and leaf addition to the substrate could improve the absorption and accumulation of nutrient elements in *P. ostreatus* var. *florida* mushrooms at the first flush.

### Overview of Metabolomics

Mass spectral data reveals multiple metabolites accumulated in *P. ostreatus* var. *florida* cultivated with AMM stems and leaves as one of organic culture substrates. Referring to previous studies (Zu et al., 2015; Wu et al., 2020), AMM herbs contain physiologically active substances with edible and nutritive value. Among them, the most abundant substances are terpenoids, followed by flavonoids, alkaloids, phenylpropanoids, and fatty acid-related compounds. Here, 46 terpenoids, 21 flavonoids, 17 alkaloids, 14 phenylpropanoids, and 3 fatty acids were identified as differential metabolites in *P. ostreatus* var. *florida* mushrooms from the AMM group (Figure 4A). Our results suggested that the fruiting body of *P. ostreatus* var. *florida* could absorb and accumulate active metabolites from substrates supplemented with AMM leaves and stems.

### Phytometabolites

In the AMM metabolomic study (Li et al., 2019; Wu et al., 2020), 254 terpenoids were identified. Among them, 46 terpenoids (18.11%) were detected in *P. ostreatus* var. *florida* cultured with AMM stems and leaves. Astragaloside IV (metab_7914 and 14941), the main active component of AMM herbs, has been reported to possess specific bioactive properties, such as antioxidant, antitumor, anti-inflammatory, nervous protection, diabetes treatment, and energy metabolism regulation properties (Kafle et al., 2020). Soyasaponin I (metab_758) plays a role in anticancer property by modulating the cell cycle and inducing apoptosis and anti-inflammatory effect by suppressing nitric oxide production (Li et al., 2019). Plumericin (metab_487) has antiparasitic and antimycobacterial activities against *Leishmania donovani* and *Mycobacterium tuberculosis* (Sharma et al., 2011). Confertifolin (metab_4742 and metab_5602), a natural mosquitocide, is an effective major substance against *Aedes aegypti* (Maheswaran and Ignacimuthu, 2015). In our study, astragaloside IV (same formula as astragaloside III), soyasaponin I, plumericin, and confertifolin were obviously detected in *P. ostreatus* var. *florida* from AMM group and were not found in control samples. The results suggested that some terpenoids in AMM herbs could transfer to the fruiting body of *P. ostreatus* var. *florida* from the substrate supplemented with AMM stems and leaves.

Flavonoids, especially their subclass of isoflavonoids, have been widely reported to possess antibacterial and antioxidant activities. In total, 101 flavonoids were annotated from AMM roots, stems and leaves, including flavones, flavanones, isoflavones, isoflavanones, flavonols, chalcones, and anthocyanins. Here, 21 flavonoids were also identified as differential metabolites in the comparison of the AMM and control groups. Formononetin (metab_14219, metab_2580, and metab_8081) has been reported to have antiangiogenic effect in the treatment of colon cancer cells *in vitro* and *in vivo* (Auyeung et al., 2012). Kaempferol (metab_1163 and metab_5123) was suggested to decrease the risk of cancers, such as liver, skin, and colon cancers. Calycosin (metab_8263), a typical component of AMM herbs, has multiple effects, including anticancer, antiallergic dermatitis, and neuroprotective abilities (Wu et al., 2020). Glycitein (metab_6222), an O-methylated isoflavone, could damage cell membranes by increasing membrane permeability and the suggested possible mechanisms of action of dietary phytoestrogens on human breast carcinoma SKBR-3 cells (Zhang et al., 2015). Genistein (metab_9173), a typical isoflavone in soy, has been shown to have multiple functions, including the promotion of apoptosis, inhibition of inflammation, and modulation of steroidal hormone receptors (Mannarapu et al., 2017). Biochanin A (metab_14044 and metab_6222) is a novel bioactive substance with multiple functions from nature. Here, formononetin and its derivative, formononetin 7-O-glucoside-6″-O-malonate, kaempferol and its derivative, kaempferol-3-O-glucoside, calycosin 7-O-glucoside, glycitein, genistein, and biochanin A were obviously detected in *P. ostreatus* var. *florida* from AMM group and not found in control samples. The results also suggested that some flavonoids in AMM herbs could transfer to the fruiting body of *P. ostreatus* var. *florida* from substrates supplemented with AMM stems and leaves.

There were 97 alkaloids found in fresh AMM roots, stems and leaves, including isoquinoline alkaloids, amines, pyridine alkaloids, and piperidine alkaloids. Trigonelline (metab_6362 and metab_1296) has therapeutic potential for diabetes and central nervous system diseases (Zhou et al., 2012). Corydaline (metab_1681) could be used to treat tachyarrhythmia (Zhang et al., 2012). As an isoquinoline alkaloid, palmatine (metab_6048) exerts multiple effects *in vivo*, including sedative effects, broad-spectrum antibacterial properties, antioxidant activities, immunity enhancement, and relief of diabetic neuropathic pain and depression (Wu et al., 2020). In our study, the relative contents of 17 alkaloids were significantly higher in the AMM group than in the control group. These results suggested that mushrooms cultured with AMM stems and leaves may have potential nutritive value in biofunctions such as glucose regulation and immunity enhancement.

Phenylpropanoids are key molecules upstream of flavonoid biosynthesis. In total, 57 metabolites from AMM herbs were identified as phenylpropanoids. Sinapic acid (metab_2362) is a precursor for coumarin synthesis (Wu et al., 2020). Coumarins (metab_7983 and metab_5690) are a large family of natural substances and are widely found in botanical medicines. These substances have been reported to have numerous biological activities, such as phytoalexin, antitumor, anti-inflammatory, antimicrobial, antioxidant, and anticoagulant activities (Kulharia et al., 2017). In our study, the contents of coumarin and sinapic acid were much more abundant in the fruiting body from the AMM group than in the control.

In addition, some polyketides and amino acid-related compounds in AMM herbs were also detected in mushrooms cultured with AMM stems and leaves. A recent study reported that acacetin (metab_6222) and capsaicin (metab_4395) isolated from plants could be potential drug candidates against COVID-19 (Adhikari et al., 2021).

### Astragaloside IV

Astragaloside IV, a major bioactive constituent, was used as the standard composition to evaluate the quality of AMM harvests. In the Chinese Pharmacopeia 2020 edition, the content of astragaloside IV in AMM roots must be higher than 0.08% based on the HPLC-ELSD method. LC-MS is a more sensitive and convenient method of quantifying astragaloside IV than LC-ELSD. Regarding previous studies, it is more common to increase sensitivity for astragaloside IV detection by applying electrospray ionization (ESI) in positive mode. Here, the content of astragaloside IV in *P. ostreatus* var. *florida* cultivated on a basal substrate supplemented with AMM stems and leaves was ∼12.22 mg/kg. Similarly, a recent HPTLC study showed that the content of astragaloside IV in oyster mushroom cultivated with AMM root as one of organic culture substrates was ∼233 mg/g (Li et al., 2020). As is well known, AMM root, the medicinal part, was rich in active compound astragaloside IV. Our results suggested that mushrooms cultured with AMM stems and leaves might absorb a certain amount of astragaloside IV and have potential health care value in immunity enhancement.

### Potential Bioactivity

The balance in the gut microbiota plays fundamental role in health and disease. In general, intestinal flora dysbiosis is a major reason for various diseases, such as cancer, diabetes, and colitis (Cheung et al., 2020). In recent decades, various natural products from medicinal plants and mushrooms were used for improving gut dysbiosis. For example, a study has illustrated that astragaloside IV could regulate gut microbiota and AMPK/SIRT1 and PI3K/AKT signaling pathways, suggesting it was an anti-diabetic botanical drug candidate (Gong et al., 2021). Moreover, numerous studies have also correlated the bioavailability and bioactivity of functional constituents through the action of the human microbiota (Chen et al., 2016; Vamanu and Gatea, 2020). One study implied that the therapeutic effects of astragaloside IV might probably be regulated by the intestinal microbiota (Jin et al., 2015).

Mushrooms were widely reported regulating gut microbiota via improving the levels of probiotics and reducing the growth of pathogens. A recent study reported that dietary fibers from oyster mushroom (*P. sajor-caju*) could increase the abundance of small-chain fatty acids (SCFAs) producing bacterial genera and inhibit the growth of intestinal pathogens (Maheshwari et al., 2021). Simultaneously, fibers impact on the bioavailability of functional constituents in the colon and are benefit for human health. A recent study investigated a long-term diet of quercetin-3-O-glucosides supplemented with soybean fibers and observed the increased levels of SCFAs production and the improved bioavailability of quercetin glycosides in rat (Trakooncharoenvit et al., 2019). Furthermore, the probiotic-ingredient relationship is a relatively new field in recent research. Some studies pointed out that the probiotics could promote the bioavailability of functional food or dietary supplements. For example, *Lactobacillus paracasei* A221 plays an important role in the functionality and bioavailability of kaempferol (Shimojo et al., 2018). This strain can convert kaempferol-3-o-sophroside into aglycone by its unique beta-glucosidase activity and increase the anti-aging activity *in vivo*. In addition, the presence of active ingredient can improve the physiological functionality of probiotics. Quercetin and resveratrol were proven to enhance the physiological functionality of *Lactobacillus* strain (Santos et al., 2019).

Various bioactive compounds from mushrooms cultured with AMM stems and leaves have been identified, such as caffeic acid (metab_7517), stilbenes (metab_4803), kaempferol (metab_5123), butyrate (metab_4652), flavonol (metab_1402), and cyanide (metab_5856). The reciprocal interactions between these phytometabolites and gut microbiota have been widely researched for the past few years (Williamson et al., 2018; Dey, 2019). Many studies have also shown that edible mushroom products, including *Pleurotus*, *Hericium*, and *Flammulina*, were rich in prebiotics for modulating the gut microbiota and could improve the bioactivity and bioavailability of functional compounds (Yin et al., 2020). Therefore, mushrooms cultured with different medicinal herbs are emerging strategies to treat diseases, and use as a basis for the carrying out of new field studies.

## Conclusion

In the present study, we analyzed and compared chemical constituents derived from the fruiting bodies of two groups, the AMM group and the control. AMM addition to the substrate affected the fresh weight, moisture, fat and element concentrations, protein content and amino acid composition of *P. ostreatus* var. *florida*. Based on the UPLC-MS data, metabolites were classified using constructed statistical models and identified by our integrated bioinformatics pipeline, especially annotated with AMM herb metabolome data published before. Differential metabolites and their related metabolic pathways in the two groups were successfully revealed. Compared with the control samples, metabolites exhibited significant differences in the fruiting bodies cultured with AMM stems and leaves. Many active compounds from AMM herbs can be absorbed and accumulate in the fruiting bodies of *P. ostreatus* var. *florida*. Our results imply that oyster mushroom cultured with AMM herbs might have very high nutritional value in therapy health care.

## Data Availability Statement

The original contributions presented in the study are included in the article/Supplementary Material, further inquiries can be directed to the corresponding author/s.

## Author Contributions

XZ, JL, X-MC, and SG discussed, plan the work, and wrote the manuscript. XZ, JL, and XL conducted the experiments. XZ carried out the data analysis, created the figures, and drafted the initial manuscript. All authors commented, made corrections, and approved the submitted version.

## Conflict of Interest

The authors declare that the research was conducted in the absence of any commercial or financial relationships that could be construed as a potential conflict of interest.

## Publisher’s Note

All claims expressed in this article are solely those of the authors and do not necessarily represent those of their affiliated organizations, or those of the publisher, the editors and the reviewers. Any product that may be evaluated in this article, or claim that may be made by its manufacturer, is not guaranteed or endorsed by the publisher.

## Footnotes

1
https://metlin.scripps.edu/

2
http://www.hmdb.ca/

3
https://www.metaboanalyst.ca/

## Supplementary Material

Supplementary Figure 1 Representative UPLC-MS typical base peak intensity chromatograms of QC samples.

Supplementary Figure 2 The standard curve of astragaloside IV.
