# Supplementary figures and images for: Nutritional Characterization and Untargeted Metabolomics of Oyster Mushroom Produced Using Astragalus membranaceus var. mongolicus Stems and Leaves as Substrates
Source: Front Plant Sci. 2022 Feb 3;13:802801. doi: 10.3389/fpls.2022.802801 (PMC8853653; doi:10.3389/fpls.2022.802801)

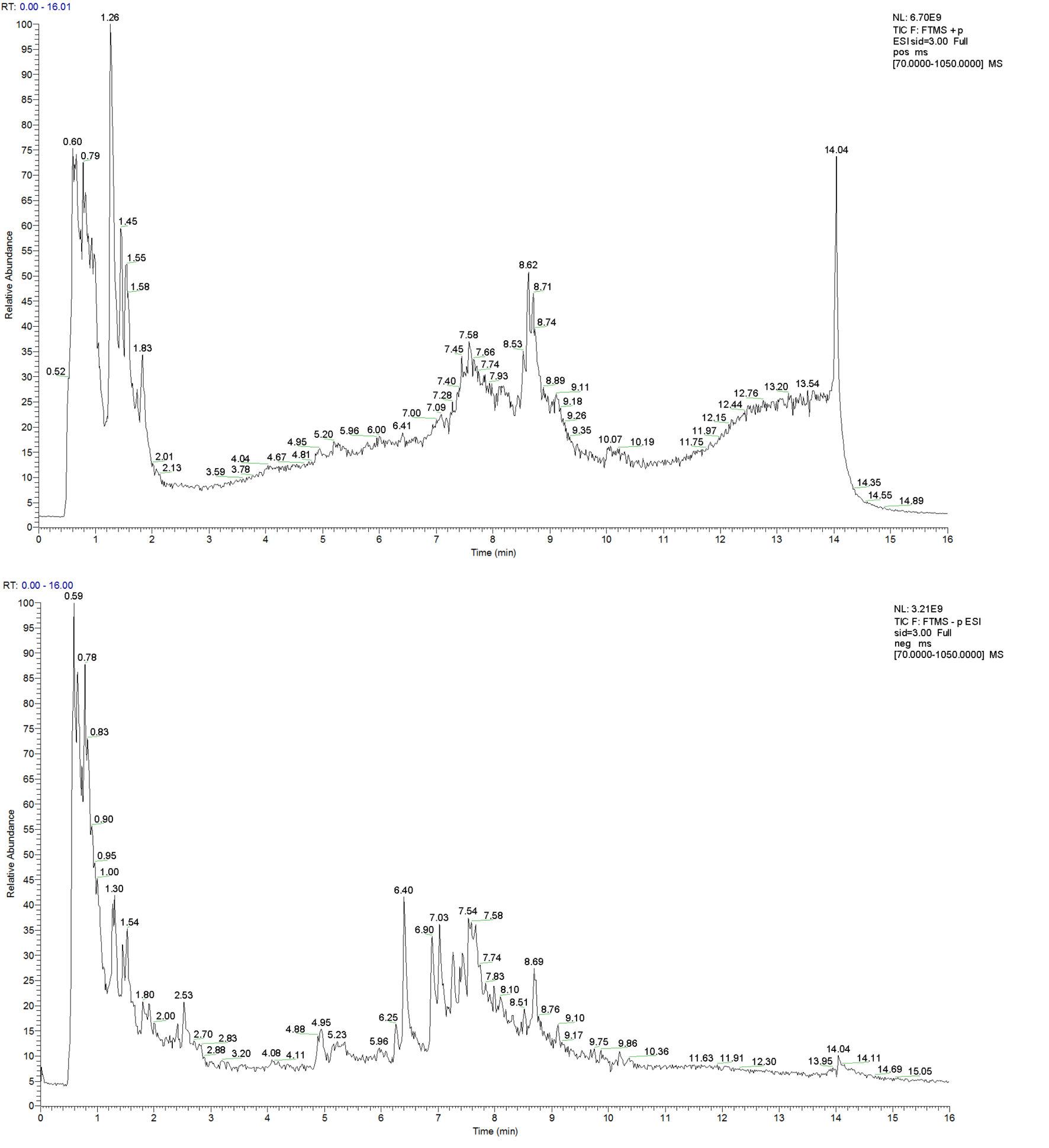

Supplement: Supplementary Figure 1 — Representative UPLC-MS typical base peak intensity chromatograms of QC samples. [file Image_1.TIF]

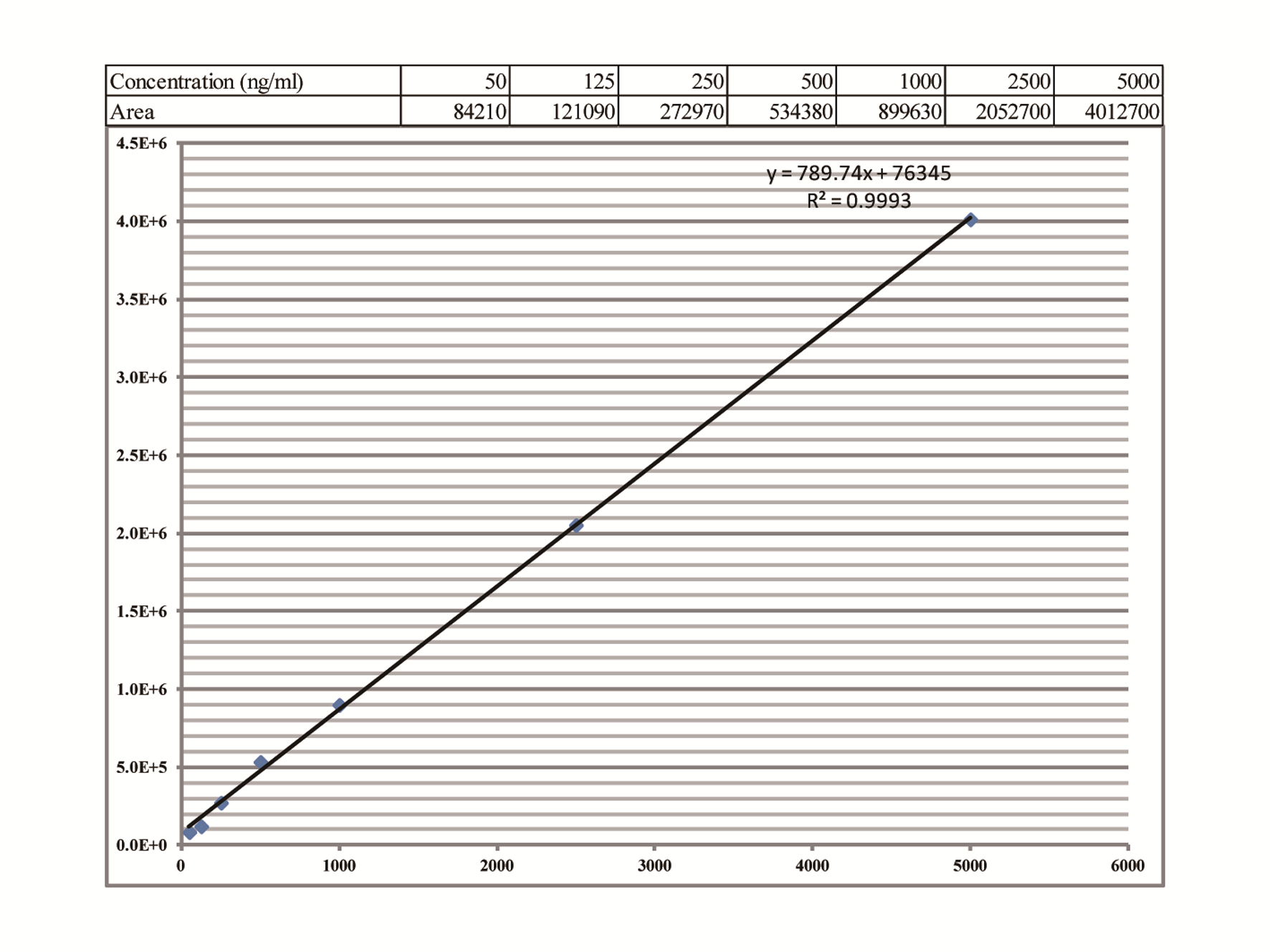

Supplement: Supplementary Figure 2 — The standard curve of astragaloside IV. [file Image_2.TIF]
