# Supplementary material for: Nutritional Characterization and Untargeted Metabolomics of Oyster Mushroom Produced Using Astragalus membranaceus var. mongolicus Stems and Leaves as Substrates
Source: Front Plant Sci. 2022 Feb 3;13:802801. doi: 10.3389/fpls.2022.802801 (PMC8853653; doi:10.3389/fpls.2022.802801)
Supplement: Supplementary file 3 [file Table_1.pdf]

Table S1 VIP value and fold change in metabolites botained from positive and negative ion modes.

metab_id	Mode	Apex_m/z	Retention_time	VIP_OPLS-DA	log2_FC	P_value	FDR
metab_6353	pos	70.0657	0.6123	0.4927	-0.8598	0.1788	0.2926
metab_1412	pos	70.0737	0.9200	0.0824	-0.4392	0.8561	0.9077
metab_3322	pos	70.9584	14.1820	0.2085	-0.1747	0.2114	0.3321
metab_7995	neg	71.0125	0.8240	0.0864	0.1166	0.7274	0.8038
metab_8196	neg	71.0483	1.3089	0.5271	-0.1327	0.0788	0.1635
metab_6078	pos	71.0497	1.1447	0.2861	0.0289	0.4554	0.5846
metab_1543	pos	71.0860	1.3293	1.9055	-3.5964	0.0000	0.0009
metab_3010	pos	71.0861	9.1302	0.0049	-0.0511	0.8229	0.8836
metab_1230	pos	72.0284	0.5140	0.5972	-0.8007	0.0244	0.0676
metab_6274	pos	72.0813	0.7661	0.3652	-0.5457	0.4371	0.5679
metab_6487	pos	72.0813	0.5140	0.2279	0.1526	0.2721	0.4003
metab_3412	pos	72.9377	14.4708	0.3589	-0.2901	0.0214	0.0612
metab_14765	neg	72.9918	0.6411	0.3601	-0.0274	0.0311	0.0848
metab_8339	neg	72.9918	1.5297	0.6792	-0.5156	0.0028	0.0166
metab_8813	neg	72.9918	2.5264	1.2612	-1.5991	0.0000	0.0004
metab_14682	neg	73.0282	0.8662	1.4155	-1.8252	0.0000	0.0012
metab_1567	pos	73.0653	1.4005	1.0572	-2.2081	0.0094	0.0344
metab_6271	pos	73.0846	0.7661	0.5252	-0.5150	0.4026	0.5361
metab_6484	pos	73.5322	0.5140	0.8192	-1.2695	0.0107	0.0375
metab_3659	pos	74.0605	10.1021	0.2716	0.0661	0.2581	0.3852
metab_3186	pos	74.0605	10.4092	0.2280	0.0008	0.3593	0.4934
metab_3392	pos	74.0605	15.1038	0.2169	-0.0121	0.1838	0.2985
metab_3274	pos	74.0969	14.0378	0.2894	-0.2110	0.0158	0.0490
metab_1413	pos	75.0445	0.9340	0.0407	0.4619	0.9794	0.9882
metab_6461	pos	75.0922	0.5140	0.6796	0.5963	0.0044	0.0200
metab_6239	pos	76.0397	0.7941	0.4615	-0.5769	0.3297	0.4640
metab_5720	pos	76.0397	1.7554	0.7204	0.6724	0.0609	0.1315
metab_1340	pos	76.0398	0.6822	0.6847	-1.2207	0.1757	0.2890
metab_9744	neg	78.4351	6.4073	0.4618	1.1636	0.2689	0.3988
metab_10851	neg	78.9577	14.1744	0.2223	0.0798	0.1800	0.2978
metab_1349	pos	79.0216	0.7381	0.6641	-0.6260	0.1139	0.2085
metab_3398	pos	79.0421	14.7524	0.1792	-0.1912	0.4706	0.5976
metab_1615	pos	79.0546	1.5284	1.0886	-1.3475	0.0004	0.0042
metab_1534	pos	79.0547	1.2725	0.4497	-0.5587	0.1417	0.2456
metab_4238	pos	79.0758	7.5743	0.4312	0.2820	0.0150	0.0474
metab_3984	pos	79.0758	8.6284	0.4043	-0.4014	0.0331	0.0844
metab_2831	pos	79.0758	8.1273	0.4348	0.3770	0.0373	0.0916
metab_6474	pos	80.0348	0.5140	0.3807	-0.5643	0.1414	0.2453
metab_10945	neg	80.9734	14.2070	0.2982	0.0315	0.0714	0.1519
metab_6216	pos	81.0339	0.8500	0.1933	0.2607	0.5538	0.6719
metab_3381	pos	81.0339	15.9629	0.2863	0.0730	0.0951	0.1825
metab_3425	pos	81.0703	14.3777	0.1059	-0.1573	0.5858	0.6991
metab_4736	pos	81.0703	4.9267	0.0176	-0.0974	0.8939	0.9319
metab_1169	pos	82.0142	0.5140	0.6602	-0.9955	0.0285	0.0757
metab_14792	neg	82.0285	0.6131	0.5031	1.6963	0.3874	0.5163
metab_11171	neg	82.0285	11.2769	0.3868	-0.0332	0.0179	0.0585

metab_1172	pos	82.5375	0.5140	0.7288	-1.0837	0.0150	0.0475
metab_8280	neg	82.9948	1.4568	0.0751	0.2884	0.7180	0.7976
metab_6483	pos	83.0149	0.5140	0.6350	-1.0005	0.0430	0.1020
metab_6486	pos	83.0391	0.5140	0.7332	-1.0590	0.0162	0.0499
metab_13890	neg	83.0489	2.0358	1.5721	4.0978	0.0027	0.0159
metab_14513	neg	83.0489	1.2377	0.4719	-0.5900	0.1659	0.2816
metab_5771	pos	83.0496	1.6693	0.1118	-0.0187	0.5860	0.6991
metab_4008	pos	83.0608	8.5989	0.4553	-0.3514	0.0036	0.0174
metab_221	pos	84.0447	0.7941	0.4618	0.4116	0.0870	0.1712
metab_3285	pos	84.9600	14.0525	0.0737	-0.0173	0.5120	0.6338
metab_14723	neg	85.0282	0.7818	0.7066	-0.5008	0.0056	0.0265
metab_935	pos	85.0287	14.3315	0.4378	0.3061	0.0316	0.0815
metab_5688	pos	85.0288	1.8277	0.5813	-0.6012	0.0219	0.0623
metab_8492	neg	85.0646	1.8046	1.8855	-2.9473	0.0006	0.0061
metab_5918	pos	85.0651	1.4005	0.7246	-0.7746	0.0350	0.0876
metab_6135	pos	85.0764	1.0180	1.4231	6.7135	0.0100	0.0359
metab_1259	pos	85.0764	0.5280	0.5116	-0.3849	0.1658	0.2761
metab_1297	pos	86.0603	0.5983	1.0213	1.5545	0.0007	0.0062
metab_1111	pos	86.0604	0.9480	0.5618	0.8364	0.0984	0.1871
metab_186	pos	86.0716	0.5140	0.9226	-0.9577	0.0382	0.0932
metab_1432	pos	86.0754	1.0040	0.4343	-0.6393	0.4447	0.5753
metab_1106	pos	86.0967	0.9761	0.3503	-0.6426	0.4627	0.5906
metab_1418	pos	86.1180	0.9480	0.5820	-0.7142	0.3820	0.5144
metab_7810	neg	86.9925	0.5126	0.7742	-0.6630	0.0057	0.0270
metab_14724	neg	87.0074	0.7818	0.6894	-0.3996	0.0077	0.0330
metab_14561	neg	87.0438	1.1212	0.0470	0.0228	0.8582	0.9020
metab_14416	neg	87.0438	1.3373	0.8892	-0.8633	0.0054	0.0258
metab_6375	pos	87.0443	0.5983	1.0965	1.6656	0.0002	0.0030
metab_1366	pos	87.0556	0.7941	0.1843	0.0308	0.5756	0.6903
metab_6432	pos	87.0556	0.5280	0.0109	0.1113	0.9481	0.9670
metab_5926	pos	87.0673	1.3865	0.2031	0.0000	0.3385	0.4727
metab_6155	pos	87.1000	0.9761	0.3628	-0.6552	0.4553	0.5846
metab_7181	neg	87.9240	14.1255	0.3766	-0.0399	0.0337	0.0896
metab_15114	neg	87.9240	0.0197	0.3364	-0.0219	0.0412	0.1029
metab_13626	neg	87.9240	2.5431	0.6863	-0.4328	0.0113	0.0428
metab_8878	neg	87.9240	2.7155	0.4070	-0.1956	0.1181	0.2186
metab_13682	neg	87.9240	2.4338	0.4945	-0.2291	0.0263	0.0760
metab_15070	neg	88.0027	0.3284	0.3340	-0.0327	0.0607	0.1355
metab_3558	pos	88.0903	11.6019	0.1934	-0.2561	0.2942	0.4246
metab_3518	pos	88.1123	14.0378	0.2998	-0.2161	0.0116	0.0399
metab_3559	pos	88.1345	11.5554	0.1991	-0.2363	0.3763	0.5096
metab_3331	pos	88.9687	14.2872	0.1333	-0.1691	0.5698	0.6854
metab_11174	neg	88.9867	11.2289	0.2849	0.0065	0.1409	0.2498
metab_10903	neg	88.9867	15.9128	0.2763	0.0306	0.1122	0.2115
metab_7781	neg	88.9867	0.4634	0.2381	0.0430	0.1749	0.2918
metab_10748	neg	89.0132	13.9952	0.5428	-0.3588	0.0778	0.1619
metab_14713	neg	89.0208	0.7818	0.2217	1.1259	0.4957	0.6169
metab_7972	neg	89.0231	0.7818	0.0091	0.5043	0.9430	0.9629

metab_6206	pos	89.0422	0.8640	1.9309	-12.5652	0.0001	0.0012
metab_7982	neg	89.0455	0.7818	0.0332	0.8265	0.9910	0.9933
metab_933	pos	89.0599	14.3470	0.3931	-0.3104	0.0013	0.0091
metab_1201	pos	89.0600	0.4387	0.3220	-0.2713	0.0373	0.0916
metab_6458	pos	89.1076	0.5140	0.2350	0.3081	0.3635	0.4971
metab_3516	pos	89.1156	14.0378	0.2892	-0.2105	0.0156	0.0487
metab_3469	pos	89.5069	14.0805	0.5997	-0.5607	0.0023	0.0131
metab_10794	neg	89.9244	14.0610	0.3199	-0.0066	0.0908	0.1810
metab_14210	neg	90.0085	1.5586	0.4915	-0.1515	0.0059	0.0274
metab_6292	pos	90.0552	0.6682	0.3375	0.1316	0.3424	0.4768
metab_932	pos	90.5069	14.0525	0.1607	-0.1160	0.5073	0.6295
metab_3505	pos	90.5260	14.0525	0.2532	-0.2134	0.0396	0.0957
metab_2032	pos	90.5261	2.9652	0.4398	-0.4415	0.0551	0.1225
metab_7755	neg	90.9322	0.0382	0.3651	-0.0931	0.0484	0.1150
metab_11058	neg	90.9322	14.0438	0.3731	-0.0466	0.0305	0.0838
metab_911	pos	90.9768	14.0228	0.0309	-0.1487	0.9132	0.9434
metab_13852	neg	91.0540	2.1153	1.5954	-3.5671	0.0021	0.0135
metab_13368	neg	91.0541	3.1115	1.6540	-3.2653	0.0052	0.0253
metab_5557	pos	91.0586	2.0973	1.0369	2.1162	0.0803	0.1615
metab_1228	pos	91.5426	0.5140	0.6518	-0.9447	0.0192	0.0565
metab_11047	neg	91.9199	14.0438	0.5800	-0.2639	0.0030	0.0173
metab_1868	pos	92.0497	2.3292	1.4672	9.3397	0.0181	0.0540
metab_15094	neg	92.9187	0.0320	0.2759	-0.0843	0.1852	0.3038
metab_11114	neg	92.9268	13.9952	0.4208	-0.0674	0.0145	0.0507
metab_14623	neg	93.0333	0.9649	1.3836	3.2546	0.0210	0.0651
metab_14060	neg	93.0333	1.7729	1.0737	3.2070	0.0682	0.1471
metab_14905	neg	93.0445	0.5431	0.6216	1.6399	0.2436	0.3699
metab_300	pos	93.0701	1.2725	0.1784	-0.1974	0.4147	0.5466
metab_12997	neg	94.0286	4.2203	0.5734	-0.3251	0.0029	0.0170
metab_8409	neg	94.0286	1.6491	0.1680	-0.0266	0.4530	0.5765
metab_9130	neg	94.0286	3.5141	0.5459	-0.3666	0.0201	0.0632
metab_8242	neg	94.0286	1.3965	0.1237	-0.0490	0.5565	0.6681
metab_3490	pos	94.0453	14.0525	0.1135	-0.2227	0.6571	0.7565
metab_1227	pos	94.0453	0.5140	0.8126	-1.1721	0.0062	0.0257
metab_3328	pos	94.0653	14.2561	0.0448	-0.1277	0.8765	0.9214
metab_6482	pos	94.5470	0.5140	0.7758	-1.1892	0.0101	0.0362
metab_10798	neg	94.9252	14.0610	0.3004	0.0420	0.2683	0.3984
metab_14803	neg	94.9765	0.5991	0.9700	4.0999	0.0263	0.0760
metab_14840	neg	94.9795	0.5991	1.1867	2.2562	0.0113	0.0428
metab_11724	neg	94.9795	8.7031	0.6981	-0.3904	0.0051	0.0248
metab_10743	neg	94.9795	13.9952	0.1571	0.1120	0.5781	0.6870
metab_13912	neg	94.9796	2.0055	1.4571	2.6413	0.0072	0.0315
metab_7742	neg	95.0238	0.0197	0.2249	0.0277	0.2958	0.4264
metab_10983	neg	95.0238	14.1090	0.2839	-0.0013	0.0984	0.1919
metab_9223	neg	95.0238	3.8001	0.4164	-0.0732	0.0171	0.0566
metab_13967	neg	95.0490	1.9125	0.8253	1.3663	0.0320	0.0865
metab_3450	pos	95.0493	14.1389	0.0911	-0.1984	0.5444	0.6638
metab_3456	pos	95.9736	14.1243	0.2668	-0.5478	0.3774	0.5107

metab_923	pos	96.5149	14.0805	0.2181	-0.2109	0.3260	0.4598
metab_10936	neg	96.9588	14.2720	0.5670	-0.2338	0.0102	0.0402
metab_7945	neg	96.9651	0.6411	0.0449	0.1122	0.9755	0.9826
metab_10981	neg	96.9683	14.1255	0.2035	0.0998	0.2001	0.3207
metab_7762	neg	96.9683	0.0589	0.1415	0.0901	0.4328	0.5572
metab_10701	neg	96.9683	11.9249	0.1638	0.0834	0.4076	0.5350
metab_6724	neg	96.9683	0.5571	0.2346	0.0683	0.2348	0.3602
metab_10417	neg	96.9683	9.1038	0.4846	-0.0799	0.0378	0.0971
metab_7097	neg	96.9683	8.7031	1.0680	-1.0316	0.0000	0.0001
metab_11461	neg	96.9683	9.4647	0.2496	0.1488	0.1865	0.3052
metab_14609	neg	97.0282	0.9933	0.5849	1.0472	0.1440	0.2539
metab_14426	neg	97.0282	1.3231	0.5034	0.7202	0.0794	0.1646
metab_8488	neg	97.0282	1.7885	0.6739	0.9941	0.0218	0.0668
metab_3310	pos	97.0285	14.1243	0.0519	-0.0497	0.8768	0.9214
metab_6217	pos	97.0286	0.8500	0.1627	0.1454	0.4116	0.5442
metab_3382	pos	97.0286	15.9471	0.3407	0.0853	0.0105	0.0371
metab_5781	pos	97.0286	1.6413	0.7745	0.5594	0.0020	0.0120
metab_6547	pos	97.0286	0.2545	0.2441	0.0052	0.0779	0.1576
metab_1832	pos	97.0286	2.2045	0.5438	0.2130	0.0003	0.0038
metab_3351	pos	97.0398	14.4708	0.4733	0.2778	0.0158	0.0492
metab_8108	neg	97.0646	1.1645	0.1764	-1.0081	0.7356	0.8091
metab_2913	pos	97.0649	8.5989	0.3130	0.1023	0.0404	0.0973
metab_1476	pos	97.0650	1.1447	0.8929	1.1906	0.0043	0.0197
metab_6454	pos	97.0762	0.5140	0.6220	-0.4249	0.0115	0.0394
metab_2736	pos	97.1013	7.3521	0.5897	-0.7716	0.0381	0.0930
metab_4093	pos	97.1013	8.2289	0.2979	-0.2612	0.0579	0.1269
metab_4528	pos	97.1013	6.0107	0.0763	-0.0328	0.8839	0.9262
metab_3995	pos	97.1013	8.6132	0.2817	-0.2310	0.0513	0.1162
metab_11784	neg	97.9301	8.6239	0.0670	0.2777	0.7110	0.7933
metab_8003	neg	98.0057	0.8522	1.0914	-3.2485	0.0250	0.0735
metab_8139	neg	98.0599	1.2377	0.4531	1.2982	0.4967	0.6175
metab_6106	pos	98.0602	1.1024	0.4836	0.5342	0.0885	0.1735
metab_4746	pos	98.0966	4.8961	0.2371	-0.2113	0.0706	0.1470
metab_6566	pos	98.5122	0.0160	0.5529	-0.4446	0.0011	0.0082
metab_3299	pos	98.5122	14.0805	0.5038	-0.4296	0.0027	0.0145
metab_10790	neg	98.9055	14.0438	0.0800	0.2572	0.7905	0.8498
metab_9839	neg	98.9378	6.9191	0.2668	0.0867	0.0589	0.1323
metab_10930	neg	98.9545	14.3373	0.5449	-0.1930	0.0107	0.0414
metab_10838	neg	98.9546	14.1255	0.5442	-0.4383	0.0490	0.1159
metab_14589	neg	98.9546	1.0357	0.6595	-0.3608	0.0180	0.0588
metab_3499	pos	98.9613	14.0525	0.2007	0.1728	0.3269	0.4609
metab_7870	neg	98.9725	0.5431	0.2374	-0.0410	0.4223	0.5483
metab_1386	pos	98.9843	0.8500	0.0264	0.2573	0.9543	0.9707
metab_3465	pos	99.0138	14.0948	0.3166	-0.2557	0.1806	0.2947
metab_6807	neg	99.0439	0.9227	0.4581	-0.1165	0.1296	0.2344
metab_14185	neg	99.0439	1.5885	0.4543	-0.1642	0.1762	0.2934
metab_6223	pos	99.0442	0.8361	0.1336	0.0088	0.7229	0.8090
metab_4340	pos	99.0806	7.0228	0.2494	-0.2143	0.4880	0.6130

metab_4668	pos	99.0806	5.2573	0.2855	0.5902	0.4973	0.6206
metab_2400	pos	99.0806	4.9267	0.1456	-0.0039	0.6558	0.7557
metab_3502	pos	99.5124	14.0525	0.0343	0.0180	0.8201	0.8820
metab_918	pos	99.5313	14.0525	0.1326	-0.1511	0.4635	0.5912
metab_11802	neg	99.9245	8.6093	0.1110	0.1532	0.5443	0.6574
metab_178	pos	100.0245	0.5140	0.7036	-1.0350	0.0176	0.0531
metab_14634	neg	100.0755	0.9508	0.0053	-0.0690	0.8944	0.9294
metab_1524	pos	100.0758	1.2585	0.8477	0.8482	0.0027	0.0146
metab_5683	pos	100.1113	1.8432	0.8215	-0.9913	0.0035	0.0173
metab_6561	pos	100.1122	0.0552	0.1726	-0.1655	0.1791	0.2928
metab_3400	pos	100.1122	14.6584	0.1452	-0.1517	0.2455	0.3700
metab_1642	pos	100.1122	1.6128	0.5688	0.3887	0.0161	0.0496
metab_3501	pos	100.5102	14.0525	0.0705	-0.1091	0.7265	0.8118
metab_11537	neg	100.9324	9.1858	0.4477	-0.0253	0.0131	0.0473
metab_7729	neg	100.9522	0.0197	0.4010	-0.1069	0.0284	0.0800
metab_3478	pos	100.9567	14.0665	0.0137	0.1032	0.8380	0.8938
metab_185	pos	101.0277	0.5140	0.6838	-1.0376	0.0220	0.0625
metab_14033	neg	101.0596	1.8205	1.0820	-1.2486	0.0013	0.0101
metab_14260	neg	101.0596	1.5297	0.0774	0.2787	0.9326	0.9561
metab_1498	pos	101.0598	1.1875	0.5665	0.4629	0.0439	0.1035
metab_237	pos	101.0599	0.7801	0.4300	0.8843	0.2633	0.3909
metab_7879	neg	101.0707	0.5571	1.3607	3.0329	0.0196	0.0619
metab_6300	pos	101.0711	0.6542	0.8420	1.2386	0.0207	0.0598
metab_1437	pos	101.1074	1.0180	0.4123	1.6548	0.4316	0.5630
metab_1243	pos	101.1075	0.5140	0.3170	-0.2983	0.3886	0.5208
metab_3287	pos	101.5089	14.0525	0.0756	0.0978	0.8753	0.9210
metab_3329	pos	102.0132	14.2561	0.0244	-0.0990	0.9859	0.9915
metab_3504	pos	102.0341	14.0525	0.3695	-0.3019	0.0161	0.0498
metab_14831	neg	102.0548	0.5991	0.7019	-0.6962	0.0165	0.0554
metab_3249	pos	102.1053	12.9248	0.2001	-0.3675	0.6580	0.7567
metab_3271	pos	102.1278	14.0378	0.2939	-0.2172	0.0177	0.0533
metab_4773	pos	102.1553	4.7302	0.4405	-0.3206	0.0635	0.1356
metab_3448	pos	102.1553	14.1674	0.2015	-0.2764	0.3160	0.4485
metab_4293	pos	102.1553	7.2314	0.4388	-0.4071	0.0104	0.0368
metab_3131	pos	102.1553	9.9154	0.3334	-0.2460	0.0496	0.1134
metab_3269	pos	102.1554	14.0228	0.3623	-0.2625	0.0072	0.0285
metab_4343	pos	102.1555	7.0082	0.4386	-0.3211	0.1803	0.2944
metab_10263	neg	102.9366	8.5937	0.2701	0.4235	0.3338	0.4667
metab_6675	neg	102.9475	0.0197	0.3750	-0.0478	0.0285	0.0802
metab_11018	neg	102.9476	14.0771	0.5938	-0.2409	0.0009	0.0076
metab_3413	pos	102.9477	14.4708	0.2253	-0.2206	0.3895	0.5219
metab_11110	neg	102.9556	14.0117	0.3549	0.0078	0.0420	0.1045
metab_14736	neg	103.0024	0.7537	0.1334	1.5897	0.9140	0.9428
metab_8064	neg	103.0388	1.0357	0.5810	-0.4718	0.0415	0.1035
metab_3455	pos	103.0389	14.1243	0.1883	0.1967	0.4132	0.5456
metab_6460	pos	103.0505	0.5140	0.8095	-1.1560	0.0188	0.0557
metab_5991	pos	103.0543	1.2725	0.5430	-0.6824	0.1255	0.2240
metab_1241	pos	103.1231	0.5140	1.2961	-2.2488	0.0040	0.0187

metab_3521	pos	103.1282	14.0228	0.0897	-0.4588	0.9257	0.9521
metab_3514	pos	103.1338	14.0378	0.2709	-0.2087	0.0335	0.0849
metab_10845	neg	103.9190	14.1744	0.4277	-0.0831	0.0152	0.0525
metab_7707	neg	103.9190	0.0197	0.3074	-0.0129	0.0820	0.1681
metab_3464	pos	103.9555	14.0948	0.2347	-0.1984	0.2445	0.3691
metab_8307	neg	104.0242	1.4856	0.3859	0.0131	0.3338	0.4667
metab_14898	neg	104.0340	0.5571	0.1823	-0.0015	0.5450	0.6580
metab_1116	pos	104.0530	0.7801	1.4368	-2.5543	0.0141	0.0455
metab_222	pos	104.0708	0.6542	0.9994	1.4472	0.0005	0.0050
metab_1324	pos	104.1042	0.6402	0.7904	-0.9581	0.0031	0.0158
metab_1147	pos	104.1070	0.5843	0.8246	-0.8917	0.0001	0.0014
metab_1305	pos	104.1354	0.5983	0.8544	-0.9628	0.0001	0.0015
metab_10971	neg	104.9268	14.1581	0.3837	-0.0608	0.0304	0.0835
metab_11101	neg	104.9527	14.0117	0.3641	0.0051	0.0733	0.1544
metab_1178	pos	105.0034	0.4999	0.7171	-1.0457	0.0221	0.0627
metab_7905	neg	105.0180	0.5991	1.3938	-1.9406	0.0000	0.0001
metab_2285	pos	105.0335	4.1698	0.5006	3.1142	0.5865	0.6994
metab_2152	pos	105.0335	3.4549	0.4275	4.0330	0.5985	0.7087
metab_1670	pos	105.0336	1.6983	0.9587	5.2029	0.2597	0.3875
metab_5606	pos	105.0336	2.0211	1.2261	4.4395	0.1203	0.2175
metab_5475	pos	105.0336	2.2519	0.9255	3.6491	0.1549	0.2622
metab_13567	neg	105.0698	2.6678	0.8194	8.0788	0.0308	0.0843
metab_5867	pos	105.0699	1.5144	1.3639	-2.0453	0.0001	0.0019
metab_6384	pos	105.1103	0.5843	0.8298	-0.9017	0.0001	0.0013
metab_6380	pos	105.1132	0.5843	0.9060	-0.9984	0.0000	0.0003
metab_12332	neg	105.6123	6.9354	0.6746	0.7009	0.2496	0.3770
metab_7168	neg	105.9346	14.1255	0.3695	-0.0439	0.0359	0.0933
metab_15104	neg	105.9456	0.0197	0.0339	0.3056	0.7352	0.8089
metab_14177	neg	106.0399	1.5885	0.2593	-0.2085	0.4931	0.6142
metab_5628	pos	106.0652	1.9918	0.5513	0.7888	0.1380	0.2407
metab_3337	pos	106.0862	14.3629	0.4355	0.2736	0.0719	0.1490
metab_15115	neg	106.9425	0.0197	0.2459	0.0263	0.1863	0.3050
metab_10870	neg	106.9425	14.2720	0.3182	-0.0138	0.0699	0.1498
metab_14032	neg	107.0490	1.8205	0.7517	-1.3538	0.1049	0.2011
metab_8682	neg	107.0490	2.2272	0.4209	-0.1619	0.2166	0.3402
metab_8316	neg	107.0490	1.5155	0.7464	-0.9717	0.0338	0.0897
metab_13118	neg	107.0491	3.8001	0.0110	0.0647	0.8501	0.8962
metab_5990	pos	107.0492	1.2725	0.5334	-0.6586	0.1097	0.2032
metab_2033	pos	107.0703	2.9652	0.6314	-0.5862	0.0074	0.0289
metab_6159	pos	107.0855	0.9620	0.9512	4.6635	0.1315	0.2321
metab_3530	pos	107.9668	13.9192	0.3196	-0.2397	0.0195	0.0571
metab_3291	pos	108.0227	14.0665	0.3804	-0.3545	0.0725	0.1500
metab_11966	neg	108.0442	8.0843	0.5057	0.8904	0.3484	0.4813
metab_9380	neg	108.0443	4.4722	0.9477	-0.9324	0.0003	0.0042
metab_8223	neg	108.0443	1.3669	0.4373	-0.6448	0.2427	0.3690
metab_8456	neg	108.0443	1.7419	0.1795	0.5916	0.7953	0.8531
metab_3367	pos	108.0444	14.9765	0.0533	-0.1199	0.7911	0.8597
metab_6554	pos	108.0444	0.2224	0.0599	-0.1239	0.7896	0.8586

metab_5909	pos	108.0808	1.4289	0.9796	1.0997	0.0002	0.0028
metab_5510	pos	108.0808	2.1897	0.2363	0.0673	0.3941	0.5268
metab_3496	pos	108.5175	14.0525	0.4045	-0.3434	0.0278	0.0747
metab_10796	neg	108.8985	14.0610	0.2008	0.0674	0.3497	0.4826
metab_8019	neg	109.0157	0.8946	0.9010	1.4236	0.0512	0.1193
metab_8532	neg	109.0157	1.8833	0.2618	0.6834	0.6261	0.7261
metab_8696	neg	109.0158	2.2595	0.2442	-0.0944	0.3943	0.5226
metab_14156	neg	109.0158	1.6335	0.2598	-0.0034	0.4926	0.6139
metab_8247	neg	109.0158	1.4115	0.0014	0.4908	0.8684	0.9097
metab_7769	neg	109.0283	0.1768	0.1931	0.0773	0.2738	0.4045
metab_8026	neg	109.0283	0.9227	0.9021	1.3923	0.0042	0.0215
metab_6871	neg	109.0283	1.3089	0.0801	0.2234	0.6903	0.7777
metab_5791	pos	109.0284	1.6272	0.8982	0.8614	0.0002	0.0030
metab_3379	pos	109.0284	15.9791	0.2159	-0.1977	0.1457	0.2508
metab_5472	pos	109.0285	2.2519	0.3045	0.0549	0.1121	0.2059
metab_7874	neg	109.0395	0.5571	0.6055	1.2315	0.1617	0.2766
metab_14109	neg	109.0647	1.6948	1.1586	1.6490	0.0015	0.0108
metab_5877	pos	109.0648	1.5004	1.3520	1.8227	0.0016	0.0099
metab_5280	pos	109.0648	2.7653	0.9304	0.8988	0.0328	0.0839
metab_4095	pos	109.1011	8.2289	0.0711	-0.1722	0.7144	0.8016
metab_4728	pos	109.1011	4.9413	0.2030	0.0511	0.5364	0.6568
metab_5439	pos	109.1012	2.3447	0.7986	0.6430	0.0007	0.0060
metab_3311	pos	109.9891	14.1243	0.4144	-0.3248	0.0072	0.0285
metab_3484	pos	110.0087	14.0525	0.3698	0.4275	0.2925	0.4228
metab_3471	pos	110.0200	14.0805	0.6484	-0.5821	0.0004	0.0039
metab_15045	neg	110.0235	0.4807	0.1127	0.1515	0.5769	0.6861
metab_8355	neg	110.0236	1.5586	0.6150	0.7801	0.0164	0.0551
metab_14582	neg	110.0600	1.0499	0.9654	2.5709	0.1201	0.2216
metab_6265	pos	110.0600	0.7801	0.6760	0.4351	0.0043	0.0198
metab_14925	neg	110.0711	0.5431	0.5928	1.3217	0.2411	0.3672
metab_1159	pos	110.0713	0.5280	0.6240	0.7471	0.0691	0.1447
metab_7917	neg	110.9840	0.5991	1.9099	4.0519	0.0000	0.0006
metab_6757	neg	111.0075	0.6411	0.9326	-0.7726	0.0077	0.0330
metab_6778	neg	111.0075	0.7818	0.8162	-0.6375	0.0039	0.0206
metab_14733	neg	111.0187	0.7678	0.7650	-1.4868	0.1312	0.2364
metab_3507	pos	111.0201	14.0525	0.2385	-0.1776	0.2047	0.3241
metab_3491	pos	111.0390	14.0525	0.3374	-0.3221	0.0908	0.1768
metab_14613	neg	111.0439	0.9933	1.1881	2.1001	0.0017	0.0120
metab_6224	pos	111.0440	0.8361	0.0117	0.1291	0.9656	0.9788
metab_13651	neg	111.0804	2.5113	0.1481	0.1919	0.7940	0.8524
metab_8366	neg	111.0804	1.5736	1.6963	3.7389	0.0001	0.0016
metab_2921	pos	111.0804	8.6132	0.3274	0.1715	0.1274	0.2266
metab_3991	pos	111.1168	8.6132	0.1986	-0.3525	0.4344	0.5656
metab_3500	pos	111.5218	14.0525	0.2588	-0.2230	0.0933	0.1803
metab_3345	pos	111.9683	14.4556	0.4239	-0.3188	0.0038	0.0182
metab_1184	pos	111.9683	0.0552	0.4931	-0.3752	0.0005	0.0050
metab_3467	pos	111.9844	14.0948	0.4256	-0.3256	0.0028	0.0150
metab_6556	pos	111.9844	0.1218	0.4558	-0.3576	0.0036	0.0176

metab_8012	neg	112.0392	0.8803	0.2818	0.0115	0.2825	0.4132
metab_1311	pos	112.0505	0.6123	0.8722	-1.1989	0.0060	0.0250
metab_1439	pos	112.0756	1.0321	0.5554	0.7004	0.0580	0.1269
metab_6490	pos	112.1121	0.5140	0.6828	0.5451	0.0016	0.0099
metab_6397	pos	112.8957	0.5560	0.3716	2.2601	0.4871	0.6121
metab_10922	neg	112.9551	14.4520	0.3258	-0.0124	0.0578	0.1307
metab_15069	neg	112.9551	0.3632	0.3071	-0.0174	0.0859	0.1738
metab_3426	pos	112.9558	14.3629	0.5510	-0.5870	0.0287	0.0763
metab_3362	pos	112.9558	14.7211	0.2146	-0.1812	0.2672	0.3948
metab_7207	neg	112.9844	14.0282	0.3135	0.0082	0.0724	0.1533
metab_3308	pos	112.9876	14.1090	0.4577	-0.3624	0.0025	0.0137
metab_11062	neg	113.0168	14.0282	0.5296	-0.5599	0.1545	0.2673
metab_6876	neg	113.0232	1.3231	0.9586	2.0917	0.0191	0.0610
metab_6195	pos	113.0232	0.8780	0.2797	0.4777	0.3117	0.4443
metab_3432	pos	113.0233	14.3019	0.3871	0.1350	0.0154	0.0484
metab_7554	neg	113.0344	0.5571	0.0448	0.2502	0.7384	0.8113
metab_6264	pos	113.0345	0.7801	1.0277	-1.8948	0.0074	0.0289
metab_3439	pos	113.0596	14.2127	0.1863	-0.1251	0.1725	0.2847
metab_8301	neg	113.0596	1.4856	0.4589	0.9376	0.2169	0.3406
metab_13828	neg	113.0596	2.1630	1.3905	-1.8897	0.0001	0.0020
metab_6117	pos	113.0596	1.0601	0.6048	-0.6363	0.0264	0.0716
metab_3326	pos	113.1073	14.2127	0.0408	-0.1186	0.8485	0.9023
metab_3008	pos	113.1324	9.1302	0.1539	0.0693	0.3385	0.4727
metab_10927	neg	113.9543	14.3862	0.3647	-0.0247	0.0358	0.0930
metab_15076	neg	113.9543	0.1093	0.2564	-0.0296	0.1565	0.2698
metab_3344	pos	113.9636	14.4556	0.4066	-0.3043	0.0043	0.0197
metab_165	pos	113.9636	0.0337	0.5059	-0.3866	0.0005	0.0048
metab_7873	neg	114.0184	0.5571	0.0625	0.2047	0.7168	0.7968
metab_14790	neg	114.0548	0.6131	1.0839	-1.4558	0.1001	0.1941
metab_8071	neg	114.0549	1.0499	0.9673	-1.7708	0.0203	0.0638
metab_2935	pos	114.0661	8.6717	0.7892	-0.7779	0.0000	0.0001
metab_3882	pos	114.0661	9.1140	0.7143	-0.7055	0.0000	0.0010
metab_1188	pos	114.0661	0.1727	0.0880	-0.2071	0.6545	0.7548
metab_6317	pos	114.0661	0.6262	0.6150	-1.3639	0.1980	0.3155
metab_7920	neg	114.0912	0.5991	0.0696	1.1760	0.9102	0.9410
metab_14572	neg	114.0912	1.0785	0.4164	2.5003	0.2830	0.4138
metab_13767	neg	114.0913	2.2754	0.3854	-0.3509	0.3810	0.5108
metab_1161	pos	114.1025	0.5280	0.9347	-0.9136	0.0014	0.0092
metab_6515	pos	114.1277	0.4999	1.8629	-3.5551	0.0000	0.0001
metab_1170	pos	114.5583	0.4999	1.0732	-1.3887	0.0230	0.0643
metab_10881	neg	114.9504	14.4185	0.3593	-0.0363	0.0402	0.1012
metab_15067	neg	114.9504	0.3798	0.3227	-0.0108	0.0594	0.1332
metab_3354	pos	114.9608	14.5331	0.1497	0.1204	0.4692	0.5962
metab_3342	pos	114.9669	14.4245	0.4293	-0.3272	0.0051	0.0221
metab_7963	neg	114.9695	0.7394	0.1503	-0.0034	0.5482	0.6603
metab_6563	pos	114.9714	0.0337	0.2872	-0.2254	0.0886	0.1736
metab_930	pos	114.9840	14.2414	0.3882	-0.2984	0.0104	0.0368
metab_7691	neg	114.9876	0.5126	0.6613	-0.5849	0.0222	0.0677

metab_7941	neg	115.0025	0.6411	0.1715	0.0601	0.3639	0.4960
metab_1074	pos	115.0389	1.3293	0.7352	0.9798	0.0156	0.0488
metab_10896	neg	115.0389	15.2315	0.0858	0.1419	0.7120	0.7937
metab_14472	neg	115.0389	1.2805	0.1753	0.2151	0.5868	0.6945
metab_7499	neg	115.0389	1.8046	1.2323	-1.4398	0.0001	0.0016
metab_5739	pos	115.0542	1.7271	0.2913	0.1500	0.3011	0.4322
metab_5855	pos	115.0542	1.5284	0.0613	0.0096	0.8036	0.8699
metab_8055	neg	115.0752	1.0074	1.2827	-3.3965	0.0701	0.1499
metab_5871	pos	115.0753	1.5004	1.1766	2.0007	0.0093	0.0341
metab_7521	neg	115.0753	1.9125	0.4507	0.9590	0.2782	0.4088
metab_5945	pos	115.0866	1.3579	0.8786	-1.4177	0.0073	0.0287
metab_5772	pos	115.0946	1.6553	0.8944	0.7484	0.0107	0.0375
metab_6433	pos	115.1059	0.5280	0.9561	-0.9476	0.0017	0.0107
metab_11173	neg	115.9195	11.2769	0.3794	-0.0522	0.0317	0.0859
metab_11539	neg	115.9195	9.1858	0.5134	-0.0622	0.0005	0.0055
metab_14999	neg	115.9909	0.5126	0.6190	-0.9521	0.0797	0.1649
metab_8691	neg	116.0494	2.2437	0.1840	0.3957	0.5540	0.6659
metab_14241	neg	116.0494	1.5297	0.3973	-0.1837	0.2974	0.4279
metab_1691	pos	116.0620	1.7271	0.2267	0.1409	0.5726	0.6881
metab_14734	neg	116.0705	0.7678	0.4132	-0.3270	0.4202	0.5463
metab_146	pos	116.0705	0.6123	0.5174	-0.8790	0.1684	0.2790
metab_8211	neg	116.0706	1.3373	0.3604	-0.3208	0.3953	0.5234
metab_7471	neg	116.0706	1.6491	0.2929	-0.0664	0.4738	0.5959
metab_1142	pos	116.1069	0.6123	0.8426	-0.9655	0.0016	0.0100
metab_3401	pos	116.1069	14.6428	0.0151	-0.1301	0.9928	0.9955
metab_3519	pos	116.1398	14.0228	0.5484	-0.7451	0.0710	0.1474
metab_3272	pos	116.1432	14.0378	0.2551	-0.1968	0.0395	0.0955
metab_11572	neg	116.9239	9.1038	0.9767	-0.4794	0.1441	0.2540
metab_11748	neg	116.9273	8.7031	0.7570	-0.3602	0.0000	0.0003
metab_3427	pos	116.9718	14.3470	0.3175	-0.2417	0.0455	0.1065
metab_1199	pos	116.9718	0.3721	0.4743	-0.3672	0.0021	0.0124
metab_10714	neg	117.0077	13.8489	0.4808	-0.1867	0.0092	0.0375
metab_6831	neg	117.0181	0.8662	1.4965	-2.0013	0.0001	0.0014
metab_3477	pos	117.0220	14.0665	0.2116	-0.2139	0.4667	0.5940
metab_8993	neg	117.0334	3.0101	1.7980	-3.0499	0.0004	0.0051
metab_8323	neg	117.0335	1.5155	1.9209	-4.8210	0.0000	0.0005
metab_177	pos	117.0396	0.4999	0.7507	-1.2518	0.0226	0.0636
metab_3374	pos	117.0545	15.7026	0.5364	0.4228	0.0573	0.1259
metab_3371	pos	117.0545	15.4267	0.4466	0.3146	0.0834	0.1657
metab_3372	pos	117.0545	15.5885	0.5480	0.3867	0.0247	0.0682
metab_10912	neg	117.0545	14.7832	0.3627	0.8001	0.4325	0.5570
metab_250	pos	117.0545	0.8780	0.0955	0.3420	0.8664	0.9147
metab_6543	pos	117.0545	0.3212	0.3988	0.2698	0.0966	0.1846
metab_14494	neg	117.0545	1.2663	0.2191	0.7252	0.6196	0.7212
metab_14172	neg	117.0546	1.6028	0.1245	0.3033	0.6994	0.7843
metab_1985	pos	117.0572	2.7959	0.6435	-0.6731	0.2386	0.3630
metab_5841	pos	117.0572	1.5424	0.0679	-0.0711	0.7655	0.8402
metab_2692	pos	117.0698	7.0670	0.3397	-0.4288	0.3108	0.4436

metab_4734	pos	117.0698	4.9267	0.2794	0.0007	0.5496	0.6678
metab_5740	pos	117.0698	1.7271	0.1266	0.0357	0.6933	0.7856
metab_14153	neg	117.0739	1.6335	0.3375	-0.0637	0.4560	0.5792
metab_2694	pos	117.0909	7.0670	0.6103	-0.5286	0.2066	0.3265
metab_6504	pos	117.1386	0.4999	1.6549	-2.3995	0.0009	0.0069
metab_3273	pos	117.1465	14.0378	0.2461	-0.1939	0.0481	0.1107
metab_3286	pos	117.1499	14.0525	0.4575	0.3307	0.0637	0.1360
metab_11164	neg	117.9351	11.5033	0.4558	-0.1238	0.0507	0.1186
metab_6562	pos	117.9595	0.0396	0.5030	-0.4072	0.0021	0.0124
metab_8519	neg	118.0035	1.8524	0.4015	-0.0820	0.0254	0.0742
metab_11163	neg	118.0286	11.5691	0.2923	0.0579	0.0453	0.1100
metab_13654	neg	118.0399	2.4970	0.2983	0.7011	0.2411	0.3672
metab_14858	neg	118.0498	0.5851	0.2475	-0.0093	0.4456	0.5693
metab_6289	pos	118.0609	0.6682	1.7258	3.3371	0.0000	0.0000
metab_5857	pos	118.0650	1.5284	0.0563	-0.0839	0.7649	0.8400
metab_8929	neg	118.0651	2.8440	0.3991	-0.2249	0.2534	0.3818
metab_1088	pos	118.0652	1.2725	0.5323	-0.6673	0.1151	0.2102
metab_6310	pos	118.0861	0.6402	0.9299	-1.5924	0.0755	0.1538
metab_3275	pos	118.1224	14.0378	0.1658	-0.1755	0.3779	0.5110
metab_6376	pos	118.1225	0.5983	1.1516	-1.9575	0.0026	0.0142
metab_11599	neg	118.8986	9.0557	0.5298	-0.1159	0.0121	0.0448
metab_10762	neg	118.9067	14.0282	0.2602	0.0035	0.2220	0.3461
metab_12394	neg	118.9231	6.7764	0.4432	-0.2287	0.0669	0.1451
metab_11800	neg	118.9231	8.6093	0.5140	-0.1868	0.0141	0.0498
metab_10942	neg	118.9426	14.2390	0.6308	-0.3254	0.0012	0.0096
metab_7977	neg	119.0338	0.7818	0.4798	-0.1232	0.0799	0.1652
metab_5888	pos	119.0351	1.4569	0.3560	-0.3371	0.5024	0.6249
metab_6228	pos	119.0352	0.8221	0.9831	-1.2263	0.0047	0.0209
metab_6233	pos	119.0490	0.8221	0.2991	-0.3836	0.3073	0.4394
metab_13644	neg	119.0491	2.5264	1.1052	-1.3764	0.0030	0.0172
metab_8314	neg	119.0491	1.5155	1.1755	-1.5464	0.0002	0.0035
metab_13737	neg	119.0491	2.3380	0.3905	0.5904	0.4353	0.5590
metab_6502	pos	119.0552	0.4999	0.6611	-1.1080	0.0809	0.1623
metab_8160	neg	119.0588	1.2663	0.0167	1.6275	0.9245	0.9508
metab_14164	neg	119.0588	1.6028	0.0690	0.3661	0.8327	0.8827
metab_1051	pos	119.0728	1.5705	0.2776	0.1134	0.0912	0.1774
metab_5357	pos	119.0729	2.5158	0.6520	-0.9425	0.1542	0.2614
metab_1211	pos	119.0835	0.4855	1.0736	-1.0811	0.0779	0.1576
metab_2518	pos	119.0854	5.7846	0.0432	-0.0940	0.9206	0.9486
metab_6306	pos	119.0894	0.6402	0.9739	-1.5701	0.1040	0.1952
metab_3255	pos	119.1064	13.3253	0.3012	-0.2845	0.0955	0.1832
metab_3549	pos	119.1064	12.4952	0.3539	-0.2504	0.0012	0.0083
metab_3544	pos	119.1064	12.7765	0.4648	-0.3644	0.0014	0.0093
metab_3541	pos	119.1064	13.0730	0.4118	-0.3757	0.0214	0.0612
metab_3300	pos	119.1064	14.0805	0.4251	-0.3605	0.0196	0.0574
metab_3945	pos	119.1065	8.8169	0.4625	-0.3483	0.0034	0.0169
metab_3434	pos	119.1065	14.2872	0.3682	-0.3842	0.0870	0.1712
metab_4812	pos	119.1065	4.5642	0.5662	-0.4182	0.0007	0.0061

metab_2415	pos	119.1065	5.0163	0.4323	-0.4125	0.0165	0.0506
metab_2385	pos	119.1065	4.8053	0.5555	-0.4665	0.0131	0.0432
metab_4888	pos	119.1065	4.2301	0.5753	-0.4334	0.0003	0.0037
metab_10803	neg	119.9225	14.0610	0.4681	-0.1205	0.0039	0.0207
metab_11122	neg	119.9458	13.9796	0.3369	0.0016	0.0508	0.1186
metab_11470	neg	119.9459	9.4322	0.2276	0.5580	0.7593	0.8273
metab_3459	pos	120.0234	14.1090	0.1870	-0.1713	0.2202	0.3425
metab_1867	pos	120.0443	2.3292	1.0660	2.6286	0.0263	0.0715
metab_13606	neg	120.0444	2.6046	0.9503	2.1955	0.0389	0.0988
metab_1525	pos	120.0454	1.2585	0.5822	-0.7415	0.0960	0.1838
metab_5398	pos	120.0556	2.4224	0.1137	0.0018	0.5065	0.6286
metab_1089	pos	120.0806	1.2725	0.5839	-0.7367	0.0934	0.1804
metab_5988	pos	120.1158	1.2725	0.6253	-0.7724	0.0744	0.1523
metab_10387	neg	120.8956	9.0391	0.6097	-0.2140	0.0070	0.0311
metab_14360	neg	120.9954	1.4427	1.1648	2.3905	0.0067	0.0300
metab_9343	neg	120.9954	4.2870	1.3416	3.1251	0.0081	0.0345
metab_13512	neg	120.9954	2.7792	1.8305	3.9864	0.0056	0.0266
metab_3877	pos	121.0282	9.1302	0.0313	-0.0850	0.8649	0.9136
metab_5076	pos	121.0283	3.4251	0.2410	2.1843	0.8203	0.8821
metab_1965	pos	121.0283	2.7029	0.3162	1.9324	0.6942	0.7864
metab_8023	neg	121.0283	0.9086	0.7005	1.1456	0.0447	0.1091
metab_8618	neg	121.0284	2.0670	0.1945	-0.2545	0.5140	0.6329
metab_13354	neg	121.0284	3.1626	0.2409	1.0119	0.6899	0.7775
metab_6244	pos	121.0395	0.7941	1.2120	8.6352	0.0930	0.1798
metab_1085	pos	121.0645	1.2725	0.1353	0.0033	0.6829	0.7773
metab_4684	pos	121.0646	5.1830	0.1049	-0.1612	0.5943	0.7054
metab_1397	pos	121.0646	0.8920	0.9765	-1.2284	0.0013	0.0089
metab_9184	neg	121.0648	3.6812	1.3499	3.5628	0.0034	0.0187
metab_6086	pos	121.0687	1.1306	0.8851	3.0598	0.1263	0.2250
metab_1084	pos	121.0838	1.2725	0.5894	-0.7491	0.0903	0.1760
metab_4650	pos	121.1010	5.3454	0.4122	0.4168	0.2225	0.3450
metab_4349	pos	121.1010	7.0082	0.0850	-0.0997	0.7991	0.8664
metab_4726	pos	121.1010	4.9413	0.3414	0.1883	0.3125	0.4453
metab_493	pos	121.1011	2.9028	1.1265	1.2226	0.0005	0.0050
metab_5648	pos	121.1011	1.9463	1.1899	1.6466	0.0000	0.0008
metab_6412	pos	121.6148	0.5420	1.1616	2.1210	0.0068	0.0273
metab_11121	neg	121.9429	13.9796	0.3336	-0.0006	0.0560	0.1274
metab_14726	neg	122.0236	0.7818	0.3590	0.0156	0.1526	0.2648
metab_3414	pos	122.0599	14.4708	0.2275	-0.0099	0.1123	0.2061
metab_5753	pos	122.0599	1.6983	0.5972	0.6227	0.1058	0.1976
metab_8904	neg	122.0600	2.7792	0.7660	1.1471	0.0492	0.1162
metab_14142	neg	122.0600	1.6491	0.0746	0.1677	0.9379	0.9595
metab_1925	pos	122.0874	2.5158	0.9722	-0.8986	0.1369	0.2394
metab_3515	pos	122.0962	14.0378	0.1955	-0.1779	0.1396	0.2430
metab_1058	pos	122.0962	1.5144	0.9515	-1.0583	0.0002	0.0028
metab_7748	neg	122.8930	0.0320	0.3284	-0.0237	0.0647	0.1419
metab_10972	neg	122.8930	14.1581	0.4309	-0.0764	0.0102	0.0402
metab_9725	neg	122.8930	6.3427	0.4046	-0.2046	0.2931	0.4245

metab_196	pos	122.9243	0.5560	0.6571	-0.6822	0.0061	0.0251
metab_10855	neg	123.0077	14.1904	0.3586	-0.0596	0.0659	0.1436
metab_8343	neg	123.0077	1.5439	0.5812	-0.3050	0.0028	0.0164
metab_10733	neg	123.0110	13.9633	0.3042	-0.0044	0.1530	0.2652
metab_916	pos	123.0401	14.0525	0.1020	-0.1212	0.5730	0.6882
metab_33	pos	123.0402	0.5140	0.7438	-1.0731	0.0113	0.0390
metab_1977	pos	123.0440	2.7497	0.4717	0.3803	0.1091	0.2023
metab_1892	pos	123.0440	2.3908	0.7676	0.8981	0.0205	0.0594
metab_6913	neg	123.0441	1.5012	0.0561	0.2743	0.9894	0.9920
metab_6248	pos	123.0515	0.7801	0.6059	2.9526	0.2899	0.4204
metab_6270	pos	123.0551	0.7801	0.1265	0.1472	0.8098	0.8741
metab_5650	pos	123.0803	1.9463	1.1179	1.5693	0.0002	0.0029
metab_8609	neg	123.0804	2.0358	1.7799	4.3871	0.0007	0.0068
metab_3229	pos	123.0914	11.4644	0.1433	-0.1409	0.2296	0.3532
metab_4087	pos	123.1166	8.2586	0.0822	-0.1388	0.6946	0.7866
metab_3993	pos	123.1166	8.6132	0.1371	-0.1532	0.3646	0.4981
metab_4733	pos	123.1167	4.9267	0.4446	0.2975	0.1448	0.2497
metab_10897	neg	123.8767	15.2980	0.3033	-0.0117	0.1476	0.2586
metab_10968	neg	123.8768	14.1581	0.4053	-0.0592	0.0227	0.0687
metab_15117	neg	123.9009	0.0197	0.4006	-0.0547	0.0148	0.0515
metab_7803	neg	123.9009	0.4975	0.2894	0.0847	0.3535	0.4864
metab_15139	neg	123.9453	0.0197	0.2928	-0.0433	0.2084	0.3308
metab_10868	neg	123.9453	14.2554	0.4218	-0.0793	0.0144	0.0505
metab_6251	pos	124.0021	0.7801	0.8778	1.3062	0.0335	0.0848
metab_1352	pos	124.0391	0.7801	0.0300	0.0957	0.8806	0.9240
metab_13984	neg	124.0393	1.8833	0.0159	0.1076	0.9847	0.9891
metab_13216	neg	124.0393	3.5641	0.8428	-0.9762	0.0064	0.0289
metab_6479	pos	124.0435	0.5140	0.7590	-1.0806	0.0125	0.0417
metab_1353	pos	124.0584	0.7801	0.1402	0.1669	0.7902	0.8590
metab_1076	pos	124.0755	1.3293	0.5430	0.5109	0.0392	0.0951
metab_14148	neg	124.0757	1.6335	0.2915	-0.0289	0.5262	0.6430
metab_3556	pos	124.0867	11.8118	0.0493	-0.1066	0.6990	0.7899
metab_7745	neg	124.8901	0.0259	0.3581	-0.0462	0.0449	0.1094
metab_10969	neg	124.8901	14.1581	0.4334	-0.0811	0.0112	0.0425
metab_1275	pos	124.9224	0.5560	0.6541	-0.5953	0.1384	0.2412
metab_14811	neg	124.9903	0.5991	1.4226	2.6435	0.0001	0.0025
metab_3148	pos	124.9996	10.0715	0.2752	-0.0222	0.2852	0.4154
metab_4260	pos	124.9996	7.4415	1.1148	1.5693	0.0001	0.0018
metab_7930	neg	124.9997	0.6131	1.8248	-13.8320	0.0015	0.0108
metab_2302	pos	125.0231	4.3060	0.1779	0.0412	0.4513	0.5808
metab_4838	pos	125.0231	4.4429	0.4099	0.1411	0.0166	0.0508
metab_4913	pos	125.0231	4.0788	0.2261	-0.0138	0.1885	0.3041
metab_3395	pos	125.0231	14.9127	0.4409	0.1582	0.0032	0.0162
metab_4561	pos	125.0231	5.8907	0.1971	-0.0381	0.3414	0.4756
metab_5756	pos	125.0231	1.6983	0.2982	0.2932	0.3144	0.4471
metab_2226	pos	125.0232	3.7908	0.2504	0.0711	0.2355	0.3596
metab_8042	neg	125.0233	0.9792	0.2923	0.0660	0.1432	0.2527
metab_5797	pos	125.0595	1.6272	1.1432	1.8018	0.0009	0.0069

metab_5186	pos	125.0596	3.0422	1.3963	2.3463	0.0001	0.0013
metab_6893	neg	125.0597	1.4115	1.2205	2.0679	0.0005	0.0053
metab_710	pos	125.0959	7.0670	0.0734	-0.1689	0.7931	0.8615
metab_4460	pos	125.0959	6.4035	0.3674	0.2734	0.1884	0.3041
metab_622	pos	125.0959	4.9267	0.2486	0.0270	0.5060	0.6283
metab_5654	pos	125.0959	1.9309	0.9457	1.0605	0.0001	0.0020
metab_5396	pos	125.0959	2.4370	0.8114	0.7930	0.0002	0.0029
metab_8644	neg	125.0961	2.1311	0.6219	-2.5364	0.3216	0.4539
metab_13340	neg	125.0961	3.1964	0.6048	0.6848	0.0783	0.1627
metab_6242	pos	125.1071	0.7941	1.2573	3.5827	0.0173	0.0525
metab_3964	pos	125.1322	8.7161	0.2732	0.0206	0.3391	0.4732
metab_6692	neg	125.8721	0.4307	0.4324	-0.0884	0.0110	0.0420
metab_7178	neg	125.8721	14.1744	0.4075	-0.0697	0.0230	0.0694
metab_15107	neg	125.8979	0.0197	0.1980	0.0288	0.3669	0.4985
metab_10859	neg	125.8980	14.1904	0.3906	-0.0819	0.0273	0.0782
metab_4961	pos	125.9861	3.8967	0.4050	-0.3353	0.0305	0.0796
metab_1193	pos	125.9861	0.2224	0.0082	-0.0791	0.9274	0.9533
metab_14796	neg	126.0185	0.6131	0.4677	1.6484	0.4186	0.5446
metab_233	pos	126.0547	0.7801	0.0237	-0.1218	0.9468	0.9665
metab_5974	pos	126.0911	1.3153	0.6509	0.8344	0.0463	0.1077
metab_8589	neg	126.0914	2.0055	0.0861	0.7644	0.9784	0.9853
metab_6456	pos	126.1025	0.5140	1.3989	-2.2415	0.0001	0.0012
metab_15101	neg	126.8799	0.0259	0.3702	-0.1141	0.0595	0.1335
metab_3320	pos	126.9552	14.1820	0.3154	-0.2505	0.0752	0.1534
metab_3418	pos	126.9718	14.4401	0.6372	-0.6778	0.0081	0.0310
metab_14340	neg	126.9849	1.4568	0.2441	0.0303	0.4001	0.5282
metab_9071	neg	126.9946	3.2801	0.6135	-0.3557	0.0153	0.0526
metab_8999	neg	126.9946	3.0260	0.8140	-0.6131	0.0127	0.0466
metab_7921	neg	127.0125	0.5991	1.5068	-3.4744	0.0005	0.0054
metab_10772	neg	127.0367	14.0282	0.1735	0.1279	0.5050	0.6246
metab_158	pos	127.0387	0.8361	0.0560	0.3015	0.7280	0.8123
metab_117	pos	127.0387	14.5961	0.0653	-0.1002	0.5656	0.6820
metab_28	pos	127.0387	0.2713	0.0985	-0.0654	0.3907	0.5227
metab_9060	neg	127.0390	3.2301	0.1547	0.3289	0.6498	0.7457
metab_14143	neg	127.0390	1.6491	0.1536	0.8967	0.7239	0.8020
metab_6073	pos	127.0751	1.1589	1.4837	3.5012	0.0024	0.0135
metab_5931	pos	127.0751	1.3865	0.1840	0.3376	0.7046	0.7940
metab_13427	neg	127.0754	2.9931	0.3164	0.5174	0.2607	0.3903
metab_8715	neg	127.0754	2.3078	0.0302	0.2192	0.8978	0.9318
metab_7482	neg	127.0754	1.7104	0.5524	0.9678	0.1138	0.2129
metab_6499	pos	127.1228	0.4999	0.4468	-0.2545	0.1150	0.2102
metab_6694	neg	127.8691	0.4307	0.4267	-0.0825	0.0125	0.0461
metab_7180	neg	127.8692	14.1581	0.4335	-0.0928	0.0146	0.0509
metab_11060	neg	127.8963	14.0282	0.4968	-0.1780	0.0240	0.0713
metab_3316	pos	127.9665	14.1530	0.3832	-0.3304	0.0399	0.0963
metab_164	pos	128.0191	0.4999	0.6964	-0.9989	0.0166	0.0508
metab_6763	neg	128.0303	0.6691	0.0729	0.8868	0.9980	0.9985
metab_7987	neg	128.0342	0.7958	0.4139	0.9949	0.3586	0.4915

metab_7470	neg	128.0706	1.6335	0.3012	-0.1224	0.3962	0.5241
metab_13410	neg	128.0787	3.0101	0.5850	1.1437	0.2228	0.3470
metab_13745	neg	128.0787	2.3078	0.3071	0.2332	0.5317	0.6479
metab_6254	pos	128.1068	0.7801	0.5020	-0.8257	0.2179	0.3399
metab_1075	pos	128.1068	1.3293	0.3381	0.1360	0.1854	0.3003
metab_5412	pos	128.1068	2.4071	0.2619	0.0417	0.4712	0.5981
metab_13703	neg	128.1070	2.4010	0.0280	0.3836	0.9714	0.9797
metab_14247	neg	128.1070	1.5297	1.3061	2.4803	0.0144	0.0506
metab_3295	pos	128.1431	14.0665	0.4458	0.2013	0.0549	0.1221
metab_3419	pos	128.1431	14.4245	0.1221	-0.1380	0.6106	0.7204
metab_2028	pos	128.1432	2.9501	0.3976	0.6380	0.1370	0.2395
metab_11097	neg	128.8802	14.0117	0.3104	0.3787	0.2947	0.4254
metab_11237	neg	128.9274	10.3355	0.4306	-0.0583	0.0168	0.0560
metab_10696	neg	128.9274	11.3577	0.2529	0.0380	0.1710	0.2875
metab_10536	neg	128.9274	9.6814	0.4337	0.0694	0.0422	0.1049
metab_11530	neg	128.9274	9.2025	0.4406	0.0010	0.0097	0.0391
metab_11143	neg	128.9274	13.8818	0.2026	0.0609	0.2395	0.3655
metab_11153	neg	128.9274	13.3240	0.1608	0.0987	0.3526	0.4859
metab_11436	neg	128.9274	9.5478	0.3393	0.1218	0.1014	0.1959
metab_927	pos	128.9505	14.1530	0.3884	-0.2935	0.0068	0.0272
metab_171	pos	128.9505	0.0552	0.4675	-0.3529	0.0008	0.0064
metab_1479	pos	128.9506	1.1447	0.3340	-0.2434	0.3069	0.4391
metab_6606	neg	128.9585	0.5571	0.8375	-0.6757	0.0094	0.0381
metab_6539	pos	128.9708	0.4387	0.6770	-0.9904	0.0600	0.1300
metab_3429	pos	128.9994	14.3315	0.3055	-0.2388	0.0644	0.1372
metab_14398	neg	129.0005	1.3669	0.5534	-0.1364	0.2734	0.4041
metab_1180	pos	129.0224	0.4999	0.7446	-1.0680	0.0124	0.0415
metab_14706	neg	129.0375	0.7958	0.4107	1.0305	0.3797	0.5096
metab_10902	neg	129.0546	15.9795	0.1171	0.0433	0.5798	0.6884
metab_7785	neg	129.0546	0.4634	0.0711	0.2145	0.7316	0.8067
metab_7539	neg	129.0546	2.0202	0.5602	-1.2670	0.2141	0.3369
metab_148	pos	129.0656	0.6262	0.4921	0.7277	0.1600	0.2685
metab_4326	pos	129.0696	7.0670	0.4046	-0.5740	0.4849	0.6103
metab_5734	pos	129.0907	1.7271	0.3805	-0.3885	0.3032	0.4349
metab_1442	pos	129.0908	1.0461	1.8574	8.0032	0.0023	0.0131
metab_1544	pos	129.0908	1.3293	1.5267	5.1232	0.0081	0.0308
metab_13574	neg	129.0910	2.6524	0.5661	0.9224	0.0910	0.1812
metab_9263	neg	129.0910	3.9508	0.9505	1.1341	0.0927	0.1836
metab_8966	neg	129.0910	2.9429	0.7382	1.0535	0.0232	0.0699
metab_3438	pos	129.1020	14.2269	0.4480	0.1810	0.0055	0.0235
metab_6491	pos	129.1384	0.5140	0.6494	0.4858	0.0022	0.0128
metab_15058	neg	129.8662	0.4307	0.3939	-0.0384	0.0149	0.0516
metab_11104	neg	129.8697	14.0117	0.8393	-0.8562	0.0037	0.0199
metab_3431	pos	129.9583	14.3167	0.3834	-0.2884	0.0693	0.1451
metab_3309	pos	129.9583	14.1090	0.6789	-0.7116	0.0007	0.0060
metab_10770	neg	129.9747	14.0282	0.3215	-0.0012	0.0727	0.1537
metab_6235	pos	130.0456	0.8081	0.9881	1.6797	0.0427	0.1015
metab_1325	pos	130.0456	0.6402	1.0734	1.8521	0.0065	0.0263

metab_220	pos	130.0496	0.7941	0.5037	0.5691	0.0776	0.1571
metab_13994	neg	130.0580	1.8682	0.1318	-0.1069	0.7371	0.8104
metab_6297	pos	130.0606	0.6542	2.2610	15.7830	0.0000	0.0003
metab_5842	pos	130.0649	1.5424	0.0705	-0.0312	0.7733	0.8467
metab_503	pos	130.0649	3.0114	0.1732	-0.3274	0.6148	0.7229
metab_9003	neg	130.0652	3.0260	0.0644	0.2793	0.7853	0.8468
metab_13676	neg	130.0653	2.4487	1.3341	-2.5614	0.0034	0.0185
metab_1136	pos	130.0860	0.6123	0.9308	1.2244	0.0054	0.0233
metab_1171	pos	130.0860	0.5140	0.4383	-0.3777	0.3164	0.4488
metab_14619	neg	130.0863	0.9792	0.7098	-1.2248	0.2190	0.3426
metab_8547	neg	130.0863	1.9280	0.4103	-0.6107	0.3547	0.4877
metab_13706	neg	130.0863	2.4010	0.2153	-0.1857	0.5974	0.7036
metab_6331	pos	130.0971	0.6262	1.5901	3.0553	0.0002	0.0024
metab_1378	pos	130.1224	0.8221	0.4968	-0.4389	0.2427	0.3675
metab_1309	pos	130.1224	0.6123	0.1035	-0.1038	0.8574	0.9084
metab_1461	pos	130.1224	1.1024	0.4721	-0.3105	0.1307	0.2311
metab_5234	pos	130.1224	2.8877	0.5456	-0.4537	0.0040	0.0189
metab_6485	pos	130.1417	0.5140	0.6416	0.4867	0.0025	0.0139
metab_3517	pos	130.1587	14.0378	0.2556	-0.2015	0.0472	0.1094
metab_12228	neg	130.1933	7.2701	0.9470	1.5176	0.1041	0.2000
metab_7740	neg	130.9427	0.0197	0.2126	0.0214	0.2631	0.3932
metab_14883	neg	130.9566	0.5571	0.7235	-0.7791	0.0663	0.1441
metab_3378	pos	130.9661	15.9629	0.2479	-0.1979	0.1119	0.2058
metab_6552	pos	130.9661	0.2224	0.3782	-0.2935	0.0070	0.0279
metab_3339	pos	130.9787	14.3928	0.4151	-0.3493	0.0123	0.0414
metab_6609	neg	130.9826	0.5126	0.7326	-0.6158	0.0057	0.0268
metab_10778	neg	130.9826	14.0438	0.3571	-0.0622	0.0633	0.1398
metab_14716	neg	130.9976	0.7818	1.0911	-1.0217	0.0076	0.0329
metab_14459	neg	130.9976	1.2805	1.0024	-1.0123	0.0027	0.0162
metab_8313	neg	131.0162	1.5012	0.3479	1.0019	0.2848	0.4159
metab_14417	neg	131.0339	1.3373	0.6680	-0.4539	0.0211	0.0654
metab_14897	neg	131.0451	0.5571	0.1099	0.1062	0.6101	0.7136
metab_13362	neg	131.0492	3.1286	1.2912	-1.9846	0.0043	0.0220
metab_230	pos	131.0529	0.7381	0.6103	0.8062	0.0542	0.1212
metab_3365	pos	131.0700	14.8646	0.6393	-0.7804	0.0092	0.0340
metab_7599	neg	131.0703	2.4175	0.2314	-0.0623	0.5578	0.6692
metab_14928	neg	131.0815	0.5431	1.1019	1.9462	0.0035	0.0190
metab_647	pos	131.0853	5.2573	0.5840	0.6542	0.1137	0.2081
metab_6427	pos	131.1250	0.5280	0.0097	-0.2114	0.9636	0.9781
metab_6450	pos	131.1289	0.5280	0.9402	-0.9130	0.0018	0.0111
metab_6478	pos	131.1539	0.5140	1.9316	-3.9917	0.0000	0.0001
metab_3356	pos	131.9739	14.5961	0.4926	-0.3810	0.0011	0.0080
metab_7805	neg	131.9858	0.5126	0.5888	-0.5755	0.0530	0.1226
metab_8650	neg	132.0208	2.1311	0.5140	-0.1337	0.0719	0.1526
metab_14843	neg	132.0292	0.5991	0.3418	0.5843	0.3883	0.5172
metab_10805	neg	132.0293	14.0610	0.3743	0.5655	0.1120	0.2113
metab_14560	neg	132.0373	1.1212	0.9091	-0.9182	0.0183	0.0593
metab_5843	pos	132.0441	1.5284	0.8184	1.1765	0.0843	0.1670

metab_2069	pos	132.0442	3.1191	1.5585	6.7006	0.0063	0.0258
metab_5501	pos	132.0442	2.1897	1.3904	1.9579	0.0000	0.0007
metab_10703	neg	132.0444	12.2652	0.4164	-0.0547	0.0076	0.0328
metab_6276	pos	132.0652	0.7381	0.3530	-0.9586	0.3787	0.5115
metab_6197	pos	132.0652	0.8780	0.0684	-0.1064	0.7507	0.8292
metab_1878	pos	132.0653	2.3600	0.4637	0.3759	0.1274	0.2266
metab_6344	pos	132.0764	0.6123	0.7829	-1.7734	0.0745	0.1523
metab_5856	pos	132.0805	1.5284	0.0105	0.0496	0.8933	0.9318
metab_8178	neg	132.0808	1.2805	1.3026	-3.4643	0.0001	0.0019
metab_1422	pos	132.1016	0.9761	0.3499	-0.6498	0.4588	0.5877
metab_6319	pos	132.1128	0.6262	0.1238	0.5899	0.6524	0.7533
metab_187	pos	132.1322	0.5280	0.8741	-0.8270	0.0027	0.0148
metab_3276	pos	132.1380	14.0378	0.0194	-0.0758	0.9880	0.9924
metab_3536	pos	132.1380	13.3993	0.1329	-0.1810	0.4592	0.5880
metab_3258	pos	132.1380	13.6662	0.2123	-0.2334	0.1828	0.2971
metab_3540	pos	132.1380	13.1619	0.0076	-0.0526	0.8819	0.9248
metab_1379	pos	132.1380	0.8221	1.3541	3.4011	0.0013	0.0089
metab_1430	pos	132.1421	0.9900	0.4737	-0.6214	0.4169	0.5488
metab_15097	neg	132.8668	0.0320	0.3116	0.0117	0.0632	0.1396
metab_10799	neg	132.9079	14.0610	0.4938	-0.1428	0.0658	0.1435
metab_6762	neg	133.0131	0.6411	0.4315	-0.1243	0.0207	0.0645
metab_8319	neg	133.0284	1.5155	1.1941	-1.4542	0.0082	0.0345
metab_1355	pos	133.0314	0.7801	1.5577	-2.8569	0.0162	0.0499
metab_7771	neg	133.0398	0.3632	0.3257	0.0279	0.0394	0.0998
metab_6151	pos	133.0491	0.9761	0.2736	0.0004	0.4812	0.6070
metab_7991	neg	133.0494	0.8098	0.3264	0.4943	0.4534	0.5767
metab_6261	pos	133.0604	0.7801	0.0205	-0.0965	0.9953	0.9970
metab_4575	pos	133.0645	5.7846	0.6217	0.4969	0.0123	0.0413
metab_8393	neg	133.0649	1.6028	1.0684	-2.5172	0.0266	0.0767
metab_8767	neg	133.0770	2.4175	0.2071	-0.0055	0.7270	0.8033
metab_1572	pos	133.0969	1.4146	0.6862	9.5341	0.1761	0.2893
metab_1160	pos	133.0969	0.5280	0.8379	1.4027	0.0698	0.1457
metab_4347	pos	133.1009	7.0082	0.0724	-0.0090	0.7841	0.8551
metab_5388	pos	133.1010	2.4530	1.1600	1.8204	0.0008	0.0064
metab_6150	pos	133.1049	0.9761	0.3525	-0.6511	0.4624	0.5904
metab_1258	pos	134.0268	0.5280	0.6702	0.8520	0.1106	0.2041
metab_8322	neg	134.0352	1.5155	0.6605	-0.3824	0.0012	0.0094
metab_1836	pos	134.0446	2.2045	0.3153	-0.3991	0.4565	0.5855
metab_14345	neg	134.0462	1.4568	0.5410	-0.6503	0.2112	0.3339
metab_8274	neg	134.0601	1.4568	0.2546	0.4195	0.5823	0.6906
metab_8933	neg	134.0601	2.8440	0.6190	0.8581	0.0594	0.1334
metab_5777	pos	134.0961	1.6553	0.4971	-0.8289	0.1793	0.2931
metab_6152	pos	134.1082	0.9761	0.5341	-0.7548	0.4070	0.5398
metab_11626	neg	134.8519	8.9574	0.2873	0.0006	0.1865	0.3052
metab_12469	neg	134.8641	6.4236	0.5158	-0.0794	0.0100	0.0398
metab_9833	neg	134.8641	6.8887	0.3343	-0.4084	0.4514	0.5748
metab_10608	neg	134.8642	10.0432	0.1813	0.0223	0.5316	0.6478
metab_10801	neg	134.8643	14.0610	0.3654	-0.0614	0.0364	0.0943

metab_11688	neg	134.8643	8.7488	0.5796	-0.2016	0.0092	0.0375
metab_7749	neg	134.8643	0.0320	0.3969	-0.0666	0.0227	0.0687
metab_7325	neg	134.8936	8.9574	0.4839	-0.0805	0.0036	0.0195
metab_11026	neg	134.9017	14.0610	0.4755	-0.2976	0.1840	0.3023
metab_7697	neg	134.9135	0.0197	0.3806	-0.0867	0.0680	0.1467
metab_10824	neg	134.9869	14.0927	0.2232	0.0851	0.1798	0.2977
metab_14159	neg	135.0111	1.6186	1.5349	3.9733	0.0130	0.0470
metab_14844	neg	135.0288	0.5991	1.1360	-1.3432	0.0001	0.0025
metab_7484	neg	135.0301	1.4856	0.3647	-0.2295	0.1275	0.2315
metab_5626	pos	135.0438	1.9918	0.5939	0.9105	0.1303	0.2307
metab_8270	neg	135.0495	1.4568	0.6769	-0.8779	0.1377	0.2458
metab_4756	pos	135.0801	4.8509	0.4834	0.4063	0.0902	0.1760
metab_4581	pos	135.0801	5.7846	0.0336	-0.0898	0.9730	0.9838
metab_1322	pos	135.0914	0.6262	1.1009	1.8755	0.0070	0.0279
metab_4350	pos	135.1165	7.0082	0.2630	-0.2681	0.4256	0.5569
metab_2917	pos	135.1166	8.6132	0.3006	-0.2849	0.1296	0.2297
metab_4737	pos	135.1166	4.9267	0.0940	-0.1185	0.8188	0.8812
metab_12189	neg	135.4080	7.4465	1.4364	10.1089	0.0000	0.0007
metab_11165	neg	135.9699	11.4384	0.3002	-0.0145	0.0920	0.1824
metab_10437	neg	135.9700	9.1693	0.3432	0.0230	0.0993	0.1928
metab_6051	pos	136.0212	1.1875	0.5177	0.7412	0.1628	0.2724
metab_3404	pos	136.0212	14.5961	0.1311	-0.1778	0.3372	0.4713
metab_6567	pos	136.0212	0.0098	0.3169	-0.2582	0.0538	0.1206
metab_8835	neg	136.0394	2.6046	0.7074	1.1111	0.0327	0.0879
metab_7586	neg	136.0394	2.3380	0.8838	1.5524	0.0210	0.0651
metab_14258	neg	136.0394	1.5297	0.7465	1.8617	0.0751	0.1574
metab_14084	neg	136.0394	1.7419	0.2891	0.4445	0.3743	0.5052
metab_7604	neg	136.0506	0.5431	0.5977	1.2343	0.2187	0.3424
metab_1372	pos	136.0615	0.8221	0.9430	-0.9848	0.0022	0.0128
metab_5896	pos	136.0615	1.4569	0.3299	-0.3458	0.4959	0.6195
metab_247	pos	136.0753	0.8221	0.3201	-0.3980	0.2689	0.3965
metab_8260	neg	136.0758	1.4427	0.1152	0.0395	0.7356	0.8091
metab_6526	pos	136.6200	0.4855	0.8515	1.2771	0.1278	0.2273
metab_11024	neg	136.8619	14.0610	0.3478	-0.0480	0.0426	0.1056
metab_7750	neg	136.8620	0.0320	0.2522	-0.0278	0.1953	0.3157
metab_14985	neg	136.8712	0.5126	1.0794	-1.6497	0.0031	0.0177
metab_11635	neg	136.8908	8.9574	0.4535	-0.0582	0.0074	0.0321
metab_7164	neg	136.9088	14.0771	0.5837	-0.2245	0.0006	0.0063
metab_14911	neg	136.9391	0.5431	0.6848	-0.6820	0.0385	0.0982
metab_7846	neg	136.9486	0.5286	1.2143	-1.5994	0.0005	0.0057
metab_14645	neg	136.9902	0.9227	1.3786	2.9727	0.0290	0.0811
metab_10551	neg	136.9904	9.7470	0.7712	-0.4807	0.0005	0.0056
metab_12889	neg	136.9904	4.6216	1.1981	2.0344	0.0164	0.0552
metab_350	pos	137.0149	1.5847	0.3200	-0.2795	0.1648	0.2747
metab_7496	neg	137.0234	1.7729	0.6304	2.1101	0.2818	0.4124
metab_6848	neg	137.0346	1.2084	0.1701	0.4787	0.5740	0.6836
metab_14924	neg	137.0346	0.5431	0.4547	1.0490	0.3643	0.4963
metab_10804	neg	137.0389	14.0610	0.4559	-0.1760	0.0492	0.1163

metab_267	pos	137.0454	1.0601	0.2173	-0.1867	0.5911	0.7031
metab_1380	pos	137.0454	0.8361	0.5255	-1.1598	0.2587	0.3861
metab_1181	pos	137.0455	0.0278	0.1516	-0.0373	0.2925	0.4228
metab_5382	pos	137.0455	2.4530	0.4035	-0.3392	0.0119	0.0406
metab_5636	pos	137.0455	1.9609	0.1560	-0.1842	0.3469	0.4817
metab_5247	pos	137.0455	2.8568	0.1727	-0.2188	0.4319	0.5632
metab_589	pos	137.0594	4.3371	1.0114	1.1442	0.0001	0.0015
metab_5482	pos	137.0595	2.2361	1.2428	1.9961	0.0008	0.0066
metab_13691	neg	137.0598	2.4175	0.1537	0.4216	0.7119	0.7937
metab_6229	pos	137.0648	0.8221	0.9967	-1.0957	0.0033	0.0167
metab_1587	pos	137.0648	1.4569	0.4095	-0.3535	0.4367	0.5677
metab_5486	pos	137.0649	2.2209	0.1189	-0.5382	0.7012	0.7915
metab_1373	pos	137.0787	0.8221	0.3420	-0.4335	0.2562	0.3830
metab_2401	pos	137.0958	4.9413	0.3385	0.1575	0.2121	0.3331
metab_5457	pos	137.0958	2.2978	0.8578	0.8960	0.0007	0.0058
metab_13711	neg	137.0962	2.3855	1.2019	1.7756	0.0034	0.0188
metab_6170	pos	137.1069	0.9340	0.2585	0.6211	0.6215	0.7286
metab_6098	pos	137.1069	1.1164	0.8894	-1.0555	0.0171	0.0520
metab_3312	pos	137.1070	14.1243	0.3529	0.0751	0.0923	0.1790
metab_385	pos	137.1070	1.7993	0.2165	0.0722	0.4729	0.5997
metab_4543	pos	137.1322	5.9504	0.8992	-0.2056	0.1938	0.3101
metab_3990	pos	137.1322	8.6132	0.0509	-0.1593	0.8760	0.9214
metab_4793	pos	137.1322	4.6096	1.2807	2.2984	0.0033	0.0164
metab_2012	pos	137.1322	2.8877	1.2065	1.3027	0.0000	0.0002
metab_6420	pos	137.6095	0.5420	1.2500	-1.7036	0.0104	0.0369
metab_3410	pos	137.9638	14.4708	0.5512	-0.4440	0.0019	0.0114
metab_7919	neg	137.9856	0.5991	0.3719	0.9187	0.5469	0.6593
metab_6557	pos	137.9866	0.1218	0.2240	-0.1911	0.1558	0.2634
metab_3451	pos	137.9869	14.1389	0.3356	-0.2740	0.0476	0.1098
metab_8038	neg	138.0186	0.9649	0.8268	1.3619	0.0172	0.0569
metab_8511	neg	138.0187	1.8371	0.6094	0.6593	0.0039	0.0206
metab_7683	neg	138.0187	3.1115	0.1240	0.1197	0.5418	0.6557
metab_1443	pos	138.0490	1.0601	0.0104	0.2748	0.9185	0.9468
metab_6362	pos	138.0503	0.5983	1.9558	6.2072	0.0000	0.0006
metab_1296	pos	138.0546	0.5983	1.9745	4.0936	0.0000	0.0000
metab_8400	neg	138.0551	1.6335	0.6832	1.1014	0.0383	0.0979
metab_8376	neg	138.0915	1.5736	0.9524	-1.1760	0.0315	0.0855
metab_3555	pos	138.1022	11.8118	0.0575	-0.1095	0.6427	0.7455
metab_10802	neg	138.8591	14.0610	0.3289	-0.0438	0.0625	0.1386
metab_7756	neg	138.8591	0.0382	0.2066	0.0229	0.2753	0.4056
metab_10197	neg	138.8949	8.3209	0.7817	-0.6589	0.0019	0.0127
metab_15113	neg	138.9059	0.0197	0.3558	-0.1256	0.0649	0.1422
metab_11015	neg	138.9059	14.0771	0.5791	-0.2537	0.0016	0.0112
metab_3408	pos	138.9856	14.5331	0.1028	-0.1406	0.6229	0.7295
metab_4399	pos	138.9949	6.8280	0.3115	0.0329	0.1952	0.3120
metab_922	pos	138.9949	14.0805	0.5422	-0.4349	0.0006	0.0054
metab_14092	neg	138.9962	1.7264	1.4560	-2.4778	0.0023	0.0144
metab_10382	neg	139.0027	9.0230	0.1939	0.3142	0.5146	0.6331

metab_939	pos	139.0175	14.0525	0.2128	-0.1833	0.1705	0.2820
metab_8734	neg	139.0265	2.3380	0.8029	-1.6614	0.1336	0.2399
metab_1769	pos	139.0387	2.0211	1.2618	2.3997	0.0040	0.0189
metab_14442	neg	139.0391	1.3089	0.1986	0.3889	0.5116	0.6306
metab_14048	neg	139.0391	1.7885	1.0993	1.7878	0.0004	0.0045
metab_6324	pos	139.0499	0.6262	0.8782	3.7009	0.1159	0.2113
metab_13901	neg	139.0503	2.0202	0.7109	-0.7029	0.0679	0.1467
metab_14369	neg	139.0503	1.4115	0.3523	0.0330	0.4096	0.5368
metab_3468	pos	139.0542	14.0805	0.3378	-0.2871	0.0967	0.1848
metab_2777	pos	139.0750	7.6776	0.1087	-0.4708	0.7269	0.8118
metab_4547	pos	139.0750	5.9361	0.5787	-0.6368	0.4001	0.5336
metab_4731	pos	139.0750	4.9267	0.8448	0.7151	0.0414	0.0992
metab_5015	pos	139.0751	3.6684	1.3348	2.2467	0.0016	0.0099
metab_1741	pos	139.0751	1.9463	1.1977	1.7246	0.0001	0.0019
metab_1793	pos	139.0751	2.0828	0.9804	1.0623	0.0001	0.0019
metab_2922	pos	139.1114	8.6132	0.2800	-0.2421	0.1416	0.2455
metab_4450	pos	139.1114	6.4639	1.3196	-2.0173	0.0005	0.0048
metab_1735	pos	139.1114	1.9309	1.1666	1.5557	0.0000	0.0004
metab_13103	neg	139.1119	3.8506	0.4892	-0.7853	0.2009	0.3218
metab_9161	neg	139.1119	3.5978	0.5003	-1.3485	0.4232	0.5490
metab_15046	neg	139.8718	0.4807	0.2785	-0.2071	0.4111	0.5384
metab_11065	neg	139.8960	14.0282	0.3294	-0.0927	0.1293	0.2340
metab_1189	pos	139.9818	0.2224	0.2044	-0.1837	0.1765	0.2896
metab_3347	pos	139.9818	14.4708	0.0922	-0.1355	0.6249	0.7311
metab_1918	pos	139.9819	2.4850	0.2801	-0.2317	0.2983	0.4294
metab_14808	neg	139.9835	0.5991	1.8583	-3.3506	0.0000	0.0000
metab_941	pos	139.9875	14.0525	0.1100	-0.0904	0.5790	0.6922
metab_2626	pos	139.9875	6.7825	0.3019	-0.2721	0.1031	0.1939
metab_8306	neg	139.9980	1.4856	0.0175	0.0285	0.9554	0.9707
metab_1365	pos	140.0338	0.7941	1.0134	1.5810	0.0035	0.0172
metab_8602	neg	140.0530	2.0202	0.0587	0.2470	0.9859	0.9899
metab_13433	neg	140.0530	2.9760	0.4590	0.6137	0.1267	0.2306
metab_3479	pos	140.0552	14.0665	0.0495	0.0852	0.8642	0.9132
metab_1242	pos	140.0554	0.5140	0.6982	-0.9612	0.0261	0.0712
metab_5967	pos	140.0702	1.3293	1.1046	1.4492	0.0001	0.0013
metab_8045	neg	140.0707	0.9933	0.0403	0.3519	0.9236	0.9507
metab_3364	pos	140.0814	14.8164	0.2378	0.1152	0.1871	0.3024
metab_6540	pos	140.0927	0.4220	0.0708	-0.0503	0.6927	0.7851
metab_3350	pos	140.1066	14.4708	0.2797	-0.0008	0.0859	0.1695
metab_358	pos	140.1067	1.6272	1.0027	1.4053	0.0012	0.0083
metab_13981	neg	140.1071	1.8833	0.0047	0.3885	0.9044	0.9366
metab_13573	neg	140.1071	2.6678	0.0702	0.5441	0.9794	0.9857
metab_8915	neg	140.1072	2.8123	0.1667	0.6110	0.8179	0.8708
metab_8985	neg	140.9820	2.9931	0.4493	-0.1974	0.0790	0.1639
metab_915	pos	140.9910	14.0525	0.2108	-0.1839	0.1821	0.2961
metab_3506	pos	140.9953	14.0525	0.0230	-0.0295	0.9395	0.9623
metab_4445	pos	140.9953	6.5096	0.0643	-0.0575	0.6612	0.7595
metab_2511	pos	140.9953	5.7086	0.5145	-0.4441	0.0080	0.0307

metab_3050	pos	140.9953	9.4049	0.3195	-0.2376	0.2157	0.3372
metab_2620	pos	140.9953	6.7217	0.0576	-0.0567	0.6280	0.7334
metab_14414	neg	141.0184	1.3373	0.6242	0.6851	0.0234	0.0702
metab_13475	neg	141.0193	2.8606	0.3913	-0.0678	0.0289	0.0809
metab_14192	neg	141.0371	1.5736	1.1080	2.1990	0.0483	0.1149
metab_13464	neg	141.0377	2.8768	0.2904	0.5048	0.4440	0.5677
metab_9191	neg	141.0449	3.7148	0.2951	0.5671	0.3645	0.4965
metab_354	pos	141.0543	1.6128	0.6080	0.5573	0.0260	0.0710
metab_6014	pos	141.0543	1.2306	0.5043	0.7487	0.2035	0.3226
metab_13674	neg	141.0548	2.4487	0.5277	-0.1817	0.0105	0.0409
metab_8224	neg	141.0548	1.3669	0.4535	-0.2170	0.0723	0.1532
metab_7479	neg	141.0548	1.6948	0.7144	0.7875	0.0263	0.0760
metab_13502	neg	141.0548	2.7961	0.1500	0.5119	0.7078	0.7908
metab_5524	pos	141.0655	2.1595	0.5169	0.3031	0.0955	0.1832
metab_707	pos	141.0906	7.0082	0.1294	-0.1614	0.7327	0.8154
metab_1866	pos	141.0907	2.3133	0.8262	0.8585	0.0015	0.0098
metab_5718	pos	141.0907	1.7554	1.1332	1.4539	0.0001	0.0019
metab_13442	neg	141.0912	2.9588	0.3161	0.6426	0.4259	0.5515
metab_642	pos	141.1270	5.3454	0.2212	0.0593	0.5175	0.6393
metab_687	pos	141.1270	6.5857	0.1389	-0.2882	0.7137	0.8011
metab_12553	neg	141.8671	6.0993	0.5515	-0.3147	0.0469	0.1126
metab_7165	neg	141.8671	14.0927	0.3952	-0.0529	0.0191	0.0610
metab_6688	neg	141.8671	0.0589	0.3387	-0.0177	0.0511	0.1193
metab_7787	neg	141.8671	0.4807	0.2348	0.3622	0.1353	0.2422
metab_10774	neg	141.8938	14.0282	0.2071	-0.0431	0.3981	0.5260
metab_6582	neg	141.9116	0.0197	0.2612	-0.0203	0.1860	0.3047
metab_3302	pos	141.9583	14.0948	0.4816	-0.3637	0.0008	0.0066
metab_4977	pos	141.9583	3.8063	0.4729	-0.4193	0.0553	0.1230
metab_5916	pos	141.9583	1.4005	0.0307	0.0436	0.8789	0.9230
metab_1183	pos	141.9583	0.0552	0.4927	-0.3779	0.0012	0.0086
metab_914	pos	141.9829	14.0525	0.1818	-0.1440	0.3047	0.4364
metab_6168	pos	142.0318	0.9340	1.1876	-1.2800	0.0411	0.0987
metab_14176	neg	142.0402	1.5885	0.3861	-0.0442	0.0492	0.1163
metab_235	pos	142.0495	0.7941	0.5459	-0.6515	0.0128	0.0425
metab_6826	neg	142.0500	1.0215	0.1124	0.5467	0.7962	0.8539
metab_14017	neg	142.0653	1.8205	0.6016	1.1252	0.3948	0.5230
metab_8350	neg	142.0654	1.5439	0.5242	-0.3371	0.2424	0.3686
metab_8893	neg	142.0654	2.7624	0.4317	-0.3822	0.3025	0.4330
metab_6519	pos	142.0711	0.4999	0.8205	-1.1972	0.0074	0.0290
metab_314	pos	142.0859	1.3865	0.8152	1.0181	0.0033	0.0166
metab_14573	neg	142.0863	1.0785	1.1013	2.0766	0.0284	0.0800
metab_7574	neg	142.0864	2.2595	0.2632	-0.1678	0.4729	0.5951
metab_7540	neg	142.0864	2.0055	0.1157	0.3818	0.8788	0.9174
metab_1510	pos	142.0972	1.2162	1.7682	3.8173	0.0000	0.0011
metab_3406	pos	142.1223	14.5638	0.2751	-0.4770	0.2167	0.3385
metab_3998	pos	142.1223	8.6132	0.2970	-0.3256	0.1598	0.2685
metab_4098	pos	142.1223	8.2143	0.1747	-0.2240	0.4570	0.5859
metab_1102	pos	142.1223	1.1875	0.3365	-0.8872	0.4129	0.5454

metab_2210	pos	142.1224	3.7148	0.0141	-0.2194	0.9614	0.9767
metab_433	pos	142.1224	2.2519	0.5749	0.2191	0.0219	0.0623
metab_8832	neg	142.1228	2.5893	1.1003	2.2596	0.0266	0.0766
metab_8525	neg	142.1228	1.8682	0.0800	0.2196	0.8905	0.9264
metab_13853	neg	142.1228	2.1153	1.2121	2.8616	0.0193	0.0614
metab_8889	neg	142.1228	2.7475	1.4650	3.1419	0.0081	0.0344
metab_3407	pos	142.1587	14.5331	0.1162	-0.5197	0.6727	0.7695
metab_7170	neg	142.8749	14.1744	0.4027	-0.0894	0.0266	0.0767
metab_10916	neg	142.9455	14.5349	0.3730	-0.0445	0.0317	0.0859
metab_3437	pos	142.9664	14.2414	0.3298	-0.3005	0.1307	0.2312
metab_3284	pos	142.9907	14.0525	0.1521	-0.1221	0.3778	0.5110
metab_1266	pos	143.0187	0.5420	0.5537	-1.7054	0.2791	0.4082
metab_14587	neg	143.0340	1.0499	0.2153	0.0665	0.2451	0.3717
metab_5873	pos	143.0700	1.5004	0.9073	1.5690	0.0166	0.0508
metab_13391	neg	143.0705	3.0604	0.9557	-2.3730	0.0620	0.1377
metab_14232	neg	143.0705	1.5439	0.9534	1.4840	0.0041	0.0212
metab_371	pos	143.0727	1.7271	0.5282	0.3395	0.0476	0.1099
metab_147	pos	143.0811	0.6123	0.4239	0.3135	0.1645	0.2745
metab_9298	neg	143.1068	4.0859	1.5923	-5.1737	0.0041	0.0210
metab_9048	neg	143.1069	3.1964	0.6322	0.7672	0.0982	0.1916
metab_1431	pos	143.1176	1.0040	1.3724	2.9133	0.0055	0.0235
metab_5913	pos	143.1176	1.4146	0.9800	0.8353	0.0548	0.1220
metab_1246	pos	143.1176	0.5140	0.5949	3.1630	0.2909	0.4213
metab_5467	pos	143.1257	2.2519	1.4094	2.6731	0.0001	0.0020
metab_12666	neg	143.8641	5.6292	0.5004	-0.2012	0.1750	0.2920
metab_9796	neg	143.8642	6.7439	0.6710	-0.6417	0.0501	0.1177
metab_6687	neg	143.8642	0.0589	0.3147	-0.0101	0.0709	0.1511
metab_12865	neg	143.8642	4.7046	0.7271	-0.4630	0.0294	0.0816
metab_9274	neg	143.8642	4.0020	0.6769	-0.3751	0.1065	0.2035
metab_6696	neg	143.8642	0.4807	0.3029	0.4181	0.0784	0.1628
metab_10820	neg	143.8642	14.0927	0.4850	-0.1249	0.0027	0.0163
metab_11042	neg	143.9227	14.0438	0.3749	-0.0771	0.0457	0.1106
metab_10807	neg	143.9380	14.0771	0.4784	-0.1293	0.0070	0.0309
metab_7705	neg	143.9380	0.0320	0.1958	-0.0106	0.4204	0.5465
metab_1196	pos	143.9587	0.2886	0.5254	-0.4063	0.0003	0.0033
metab_3493	pos	143.9805	14.0525	0.1633	-0.1251	0.3548	0.4894
metab_6542	pos	143.9965	0.3553	0.2356	-0.2259	0.1530	0.2599
metab_6555	pos	143.9965	0.1385	0.1856	-0.1842	0.2758	0.4044
metab_13503	neg	143.9984	2.7961	0.6431	-0.6060	0.2025	0.3239
metab_13634	neg	144.0194	2.5264	0.4629	-0.1049	0.0076	0.0326
metab_6593	neg	144.0292	0.6131	0.5785	1.6013	0.2469	0.3740
metab_13504	neg	144.0399	2.7961	0.6970	-1.1575	0.1378	0.2458
metab_5067	pos	144.0441	3.4549	0.9323	-0.0293	0.2318	0.3558
metab_5836	pos	144.0441	1.5424	0.2813	-0.1402	0.5176	0.6393
metab_8909	neg	144.0446	2.7961	0.5149	-0.6173	0.1964	0.3168
metab_1478	pos	144.0475	1.1447	0.2121	0.6462	0.6508	0.7520
metab_1435	pos	144.0652	1.0180	0.2398	0.4148	0.5031	0.6254
metab_14637	neg	144.0656	0.9508	0.1242	0.2141	0.7215	0.8003

metab_14236	neg	144.0657	1.5439	0.1490	-0.2141	0.6373	0.7352
metab_5742	pos	144.0804	1.7271	0.0477	-0.1372	0.9227	0.9500
metab_14478	neg	144.0810	1.2663	1.6740	-2.7374	0.0025	0.0154
metab_13506	neg	144.0906	2.7961	0.1418	-0.1941	0.6532	0.7482
metab_5668	pos	144.1015	1.9014	0.2446	-0.0588	0.4911	0.6152
metab_14603	neg	144.1020	1.0074	0.1222	-0.3325	0.7655	0.8319
metab_14354	neg	144.1021	1.4427	1.7327	-3.8267	0.0013	0.0101
metab_5731	pos	144.1263	1.7412	0.0562	0.0863	0.8523	0.9053
metab_2339	pos	144.1380	4.5027	0.1991	-0.2482	0.4637	0.5912
metab_3476	pos	144.1743	14.0665	0.0720	0.0691	0.9426	0.9640
metab_1709	pos	144.6173	1.7845	1.1316	3.1955	0.0194	0.0570
metab_6426	pos	144.6174	0.5420	0.6872	0.8847	0.0229	0.0641
metab_1655	pos	144.6174	1.6413	0.0852	0.7415	0.7419	0.8225
metab_2083	pos	144.6174	3.1799	0.6368	0.9372	0.0732	0.1510
metab_1840	pos	144.6174	2.2209	0.3905	0.4747	0.1761	0.2893
metab_2019	pos	144.6175	2.9192	0.6070	0.8431	0.1196	0.2164
metab_10853	neg	144.8721	14.1744	0.3468	-0.0282	0.0660	0.1437
metab_6677	neg	144.9423	0.0197	0.3883	-0.0681	0.0237	0.0707
metab_15116	neg	144.9584	0.0197	0.1328	0.1206	0.5342	0.6500
metab_3298	pos	144.9817	14.0805	0.4460	-0.3384	0.0041	0.0190
metab_3494	pos	144.9881	14.0525	0.5024	-0.4891	0.0348	0.0873
metab_14117	neg	144.9956	1.6797	0.4354	-0.0737	0.4182	0.5443
metab_6524	pos	145.0092	0.4855	0.4962	-0.5527	0.0773	0.1567
metab_14768	neg	145.0132	0.6411	1.0239	-2.8186	0.0668	0.1449
metab_917	pos	145.0167	14.0525	0.0017	-0.0668	0.9057	0.9398
metab_1588	pos	145.0315	1.4569	0.3117	-0.1120	0.5623	0.6789
metab_7983	neg	145.0397	0.7818	1.2966	2.6070	0.0438	0.0535
metab_14382	neg	145.0399	1.3817	1.2859	2.6713	0.0089	0.0367
metab_14088	neg	145.0400	1.7264	0.0828	0.0679	0.7951	0.8531
metab_8583	neg	145.0400	1.9904	0.6648	0.7423	0.0023	0.0145
metab_14000	neg	145.0450	1.8524	1.6698	-3.6355	0.0003	0.0042
metab_246	pos	145.0491	0.8500	0.0760	0.1202	0.9814	0.9887
metab_8516	neg	145.0497	1.8524	1.2035	-1.7005	0.0010	0.0084
metab_7438	neg	145.0497	1.5297	0.1378	0.3544	0.7626	0.8295
metab_7590	neg	145.0609	0.5571	0.6274	1.1913	0.1084	0.2061
metab_5599	pos	145.0645	2.0211	1.0266	1.1873	0.0221	0.0626
metab_5368	pos	145.0645	2.4850	1.3866	2.7337	0.0025	0.0141
metab_5306	pos	145.0645	2.6719	1.7750	3.7915	0.0000	0.0011
metab_9122	neg	145.0649	3.4800	1.3287	-7.9237	0.0124	0.0459
metab_8454	neg	145.0690	1.7264	0.3523	0.3370	0.4380	0.5619
metab_1685	pos	145.0837	1.7271	0.0205	-0.0774	0.9386	0.9616
metab_14293	neg	145.0861	1.4856	1.0236	1.7717	0.0022	0.0141
metab_13222	neg	145.0861	3.5475	0.1999	0.2745	0.6379	0.7356
metab_6016	pos	145.0968	1.2162	0.9308	-1.2818	0.0176	0.0532
metab_7858	neg	145.0972	0.5431	0.7197	-0.5153	0.1621	0.2769
metab_8744	neg	145.0973	2.3533	0.3190	0.5795	0.5105	0.6295
metab_6446	pos	145.1696	0.5280	1.0006	-1.6634	0.0060	0.0251
metab_1206	pos	145.6070	0.4693	0.9223	-0.9911	0.1193	0.2161

metab_11005	neg	145.8613	14.0771	0.4352	-0.2384	0.0455	0.1103
metab_10788	neg	145.9182	14.0438	0.0487	0.1776	0.7805	0.8436
metab_10863	neg	145.9299	14.2070	0.0992	0.1225	0.6356	0.7335
metab_7753	neg	145.9299	0.0382	0.0543	0.0751	0.7628	0.8296
metab_3335	pos	145.9657	14.3315	0.3406	-0.2864	0.0349	0.0875
metab_6537	pos	145.9657	0.4543	0.6845	-0.6135	0.0047	0.0209
metab_14303	neg	146.0239	1.4710	1.5740	3.7100	0.0015	0.0108
metab_8921	neg	146.0351	2.8123	0.3833	-0.1942	0.1136	0.2128
metab_14318	neg	146.0351	1.4568	0.6603	-0.3167	0.0007	0.0068
metab_13289	neg	146.0352	3.3144	0.3948	-0.1442	0.0637	0.1405
metab_6738	neg	146.0449	0.5991	0.5455	-0.5167	0.0688	0.1481
metab_7648	neg	146.0510	2.7961	0.2978	-0.3255	0.4451	0.5689
metab_5276	pos	146.0597	2.7804	0.4043	-0.6441	0.2625	0.3904
metab_13988	neg	146.0602	1.8682	0.5875	1.6675	0.3526	0.4859
metab_8658	neg	146.0603	2.1630	0.7687	1.4522	0.0885	0.1778
metab_8442	neg	146.0604	1.7104	1.0143	2.1661	0.0431	0.1063
metab_6732	neg	146.0641	0.5571	0.6596	1.2789	0.1011	0.1956
metab_1114	pos	146.0808	0.8640	0.1095	-0.0742	0.7004	0.7911
metab_8016	neg	146.0813	0.8946	1.2543	-2.2901	0.0031	0.0174
metab_9238	neg	146.0813	3.8506	0.1927	0.1447	0.2751	0.4053
metab_14445	neg	146.0813	1.3089	0.5422	-0.1850	0.0120	0.0446
metab_1133	pos	146.0920	0.6262	0.3520	1.4000	0.5128	0.6344
metab_1971	pos	146.0961	2.7188	0.8899	-1.1143	0.0057	0.0241
metab_1919	pos	146.0961	2.4850	1.2198	-1.9696	0.0003	0.0032
metab_14909	neg	146.1007	0.5431	0.5524	-0.2841	0.2359	0.3613
metab_6351	pos	146.1171	0.6123	1.2095	-2.2676	0.0015	0.0097
metab_5817	pos	146.1171	1.5847	0.2853	-0.0127	0.2855	0.4158
metab_1799	pos	146.1172	2.0973	0.8320	1.0222	0.0034	0.0170
metab_6126	pos	146.1648	1.0461	0.8538	1.0626	0.0129	0.0427
metab_6492	pos	146.1648	0.5140	0.6160	0.4435	0.0032	0.0164
metab_15096	neg	146.9226	0.0320	0.3101	-0.0067	0.0845	0.1716
metab_7724	neg	146.9376	0.0197	0.3698	-0.0509	0.0340	0.0900
metab_11019	neg	146.9376	14.0771	0.5379	-0.1738	0.0019	0.0128
metab_10776	neg	146.9602	14.0438	0.2333	0.0430	0.1731	0.2897
metab_15026	neg	146.9603	0.4975	0.1164	0.1265	0.5405	0.6548
metab_15044	neg	146.9648	0.4807	0.2786	0.3682	0.1691	0.2853
metab_10728	neg	146.9650	13.9301	0.2287	0.0693	0.1805	0.2985
metab_12429	neg	146.9650	6.5989	0.5502	-0.2653	0.0293	0.0816
metab_10262	neg	146.9650	8.5937	0.7166	-0.7200	0.0458	0.1107
metab_3361	pos	146.9799	14.6742	0.5966	-0.5066	0.0010	0.0076
metab_3472	pos	146.9799	14.0805	0.4456	-0.3377	0.0050	0.0219
metab_8094	neg	146.9844	1.1212	0.8958	11.7017	0.0339	0.0898
metab_13662	neg	147.0192	2.4813	0.2226	0.8442	0.5489	0.6609
metab_14729	neg	147.0289	0.7818	0.7289	-0.7128	0.0189	0.0606
metab_2235	pos	147.0437	3.8511	0.2760	0.1484	0.4796	0.6063
metab_5145	pos	147.0437	3.1799	0.0443	0.4519	0.7977	0.8652
metab_5530	pos	147.0437	2.1444	0.2791	0.5222	0.5373	0.6576
metab_14473	neg	147.0443	1.2805	0.9956	-1.1957	0.0151	0.0522

metab_6514	pos	147.0499	0.4999	0.7102	-1.0449	0.0141	0.0454
metab_5690	pos	147.0549	1.8134	1.8774	1.3112	0.0129	0.0427
metab_1707	pos	147.0647	1.7845	0.1033	0.1879	0.8310	0.8889
metab_8140	neg	147.0653	1.2521	0.2072	0.0189	0.4445	0.5682
metab_6301	pos	147.0760	0.6542	0.7980	1.1447	0.0242	0.0673
metab_1743	pos	147.0800	1.9463	1.5387	10.4956	0.0033	0.0166
metab_5334	pos	147.0801	2.5782	1.9346	4.8483	0.0002	0.0028
metab_2141	pos	147.0801	3.3793	1.9691	4.4920	0.0001	0.0018
metab_8936	neg	147.0806	2.8440	0.4041	0.0195	0.3507	0.4838
metab_1570	pos	147.0913	1.4146	0.2817	0.1067	0.1447	0.2496
metab_5458	pos	147.0913	2.2824	0.0785	-0.1523	0.5946	0.7054
metab_6488	pos	147.1124	0.5140	0.5107	-0.4756	0.2666	0.3943
metab_2390	pos	147.1164	4.8809	0.3097	0.2407	0.3652	0.4986
metab_562	pos	147.1164	3.8511	1.0779	1.1308	0.0011	0.0080
metab_4329	pos	147.1164	7.0670	0.3480	-0.3049	0.2623	0.3901
metab_1240	pos	147.1681	0.5140	0.5893	0.4207	0.0056	0.0238
metab_10789	neg	147.9257	14.0438	0.3154	0.0096	0.1197	0.2211
metab_11013	neg	147.9409	14.0771	0.6708	-0.3837	0.0006	0.0064
metab_15134	neg	147.9410	0.0197	0.4060	-0.0847	0.0183	0.0593
metab_7687	neg	147.9729	0.4975	0.5634	-0.3575	0.0156	0.0534
metab_5325	pos	148.0390	2.6090	0.8441	1.0481	0.0126	0.0420
metab_8278	neg	148.0396	1.4568	0.9615	1.4703	0.0128	0.0467
metab_14178	neg	148.0759	1.5885	0.0287	0.3742	0.9195	0.9471
metab_13934	neg	148.0759	1.9904	0.4123	0.9694	0.3316	0.4643
metab_1332	pos	148.0793	0.6542	0.8555	1.2872	0.0208	0.0599
metab_1298	pos	148.0964	0.5983	0.2435	-0.1573	0.0834	0.1657
metab_6335	pos	148.1076	0.6123	0.8823	9.8438	0.0288	0.0764
metab_5923	pos	148.1117	1.4005	0.7425	-1.4442	0.0704	0.1467
metab_6459	pos	148.1715	0.5140	0.6291	0.5134	0.0067	0.0272
metab_15088	neg	148.9179	0.0382	0.3202	-0.0372	0.0889	0.1785
metab_11022	neg	148.9664	14.0610	0.2179	-0.1491	0.3577	0.4904
metab_6700	neg	148.9682	0.4975	0.3080	-0.0510	0.1641	0.2793
metab_3433	pos	148.9766	14.2872	0.2982	-0.2532	0.2201	0.3425
metab_10980	neg	148.9897	14.1255	0.4036	-0.0519	0.0167	0.0558
metab_6550	pos	149.0230	0.2224	0.2327	-0.1903	0.0781	0.1578
metab_3357	pos	149.0230	14.6110	0.2265	-0.1862	0.0802	0.1614
metab_2635	pos	149.0230	6.8433	0.1820	-0.2119	0.2162	0.3378
metab_824	pos	149.0230	9.1302	0.2114	-0.1776	0.2780	0.4069
metab_4777	pos	149.0230	4.7001	0.1113	-0.1280	0.6123	0.7211
metab_5262	pos	149.0230	2.8259	0.7058	-0.6305	0.0004	0.0042
metab_8517	neg	149.0236	1.8524	0.1573	0.2857	0.5870	0.6946
metab_14798	neg	149.0445	0.6131	0.5787	0.8550	0.1235	0.2260
metab_70	pos	149.0593	1.2725	0.5653	-0.7035	0.1130	0.2072
metab_13487	neg	149.0599	2.8440	0.3656	-0.4735	0.2996	0.4300
metab_8730	neg	149.0599	2.3227	0.2074	0.0438	0.5890	0.6964
metab_6095	pos	149.0804	1.1164	0.0801	0.0680	0.9726	0.9838
metab_5565	pos	149.1070	2.0828	0.2852	-0.7028	0.3867	0.5189
metab_1555	pos	149.1070	1.3720	0.5667	0.6321	0.1282	0.2277

metab_2918	pos	149.1321	8.6132	0.1849	-0.2665	0.4154	0.5474
metab_4346	pos	149.1321	7.0082	0.2354	-0.2820	0.4528	0.5822
metab_132	pos	149.1321	6.2522	0.4863	-0.3959	0.1225	0.2205
metab_4675	pos	149.1321	5.2129	0.1799	-0.2280	0.7310	0.8142
metab_1010	pos	149.1322	4.9267	0.2064	-0.5052	0.6850	0.7791
metab_1250	pos	149.5483	0.5280	0.4524	-0.0045	0.4304	0.5619
metab_15145	neg	149.9122	0.0197	0.3694	-0.0436	0.0348	0.0913
metab_10856	neg	149.9122	14.1904	0.4890	-0.1317	0.0040	0.0210
metab_10793	neg	149.9359	14.0610	0.2137	0.0796	0.2018	0.3230
metab_6455	pos	149.9525	0.5140	1.0029	-1.4344	0.0018	0.0108
metab_7455	neg	150.0302	1.5586	1.1951	-2.4407	0.0249	0.0733
metab_6128	pos	150.0547	1.0461	0.6789	1.1145	0.0629	0.1347
metab_6111	pos	150.0910	1.0883	0.2245	-0.7054	0.4802	0.6065
metab_11185	neg	150.8886	11.0007	0.4947	-0.2306	0.0147	0.0513
metab_10678	neg	150.8886	10.7856	0.7798	-0.5009	0.0002	0.0033
metab_10591	neg	150.8886	9.9606	0.5981	-0.1609	0.0069	0.0306
metab_11606	neg	150.8886	9.0391	0.5485	-0.1632	0.0210	0.0651
metab_15091	neg	150.9153	0.0382	0.3769	-0.3358	0.2736	0.4043
metab_15039	neg	150.9461	0.4975	0.6665	-0.3865	0.0056	0.0265
metab_14346	neg	150.9962	1.4427	1.8934	-11.3234	0.0014	0.0106
metab_3463	pos	151.0152	14.0948	0.6631	-0.5656	0.0002	0.0030
metab_6523	pos	151.0349	0.4999	0.7516	-1.0527	0.0084	0.0320
metab_8683	neg	151.0392	2.2272	0.2268	0.0382	0.3028	0.4333
metab_14034	neg	151.0392	1.8205	0.5241	-0.7612	0.1956	0.3159
metab_7441	neg	151.0392	1.5297	0.3522	-0.7425	0.4347	0.5587
metab_8448	neg	151.0426	1.7104	1.0478	8.1916	0.0313	0.0852
metab_14838	neg	151.0603	0.5991	1.2609	-2.4977	0.0068	0.0302
metab_4750	pos	151.0750	4.8809	1.2642	2.0143	0.0100	0.0360
metab_1732	pos	151.0750	1.9169	0.7849	0.9403	0.0073	0.0287
metab_5092	pos	151.0751	3.3490	0.5101	0.6151	0.0806	0.1618
metab_9345	neg	151.0756	4.2870	0.2923	-0.0716	0.2618	0.3916
metab_5634	pos	151.0862	1.9758	0.6440	0.7951	0.0811	0.1626
metab_8299	neg	151.0868	1.4710	0.0510	-0.0374	0.9323	0.9560
metab_4603	pos	151.1114	5.6623	0.4172	-1.1236	0.4577	0.5865
metab_4331	pos	151.1114	7.0670	0.4481	-0.4708	0.1900	0.3054
metab_1014	pos	151.1114	4.8809	0.0629	-0.2778	0.8835	0.9261
metab_1986	pos	151.1114	2.8108	1.2387	2.0369	0.0006	0.0057
metab_5915	pos	151.1226	1.4146	0.5597	-0.5863	0.2511	0.3764
metab_2925	pos	151.1477	8.6132	0.0504	-0.2485	0.8952	0.9325
metab_15106	neg	151.9037	0.0197	0.2849	-0.0795	0.1300	0.2348
metab_14189	neg	152.0107	1.5736	0.1732	1.0512	0.7727	0.8374
metab_13902	neg	152.0167	2.0202	1.4697	3.7876	0.0172	0.0569
metab_13551	neg	152.0345	2.7155	0.0529	0.1730	0.9558	0.9709
metab_13061	neg	152.0345	4.0182	0.0812	0.2182	0.7183	0.7977
metab_6518	pos	152.0383	0.4999	0.8450	-1.1074	0.0017	0.0108
metab_6225	pos	152.0563	0.8361	0.8539	-1.0255	0.0127	0.0423
metab_1327	pos	152.0563	0.6402	1.1826	-1.7520	0.0057	0.0241
metab_6142	pos	152.0563	1.0180	0.2863	-0.2401	0.4840	0.6097

metab_1756	pos	152.0703	1.9918	0.2927	0.1086	0.2501	0.3754
metab_14358	neg	152.0708	1.4427	0.0751	0.2489	0.9679	0.9773
metab_2257	pos	152.1066	4.0028	0.2335	0.2418	0.6588	0.7573
metab_5684	pos	152.1066	1.8432	0.3504	0.0367	0.1691	0.2801
metab_6430	pos	152.1178	0.5280	1.0638	2.2583	0.0364	0.0900
metab_5050	pos	152.1430	3.5162	0.3838	0.1870	0.4633	0.5912
metab_10928	neg	152.8940	14.3693	0.6995	-0.3788	0.0011	0.0091
metab_7743	neg	152.9037	0.0197	0.1676	0.1175	0.4806	0.6029
metab_15057	neg	152.9503	0.4307	0.2998	0.0487	0.0554	0.1266
metab_10888	neg	152.9503	14.5179	0.3374	-0.0323	0.0683	0.1473
metab_10060	neg	152.9879	7.8193	1.3707	2.1699	0.0294	0.0816
metab_3323	pos	152.9945	14.1972	0.5213	-0.4282	0.0013	0.0089
metab_3416	pos	152.9945	14.4556	0.5377	-0.5959	0.0405	0.0975
metab_3366	pos	152.9945	14.9127	0.4721	-0.3867	0.0022	0.0129
metab_12589	neg	152.9946	5.9375	0.4061	-0.3043	0.4162	0.5423
metab_9987	neg	152.9947	7.5541	0.6037	1.0301	0.0988	0.1922
metab_9928	neg	152.9948	7.2701	0.9318	1.5845	0.0567	0.1289
metab_14810	neg	152.9949	0.5991	0.4279	0.8719	0.3728	0.5039
metab_3473	pos	153.0105	14.0805	0.5487	-0.4506	0.0009	0.0070
metab_2309	pos	153.0105	4.3512	0.6157	-0.5548	0.0043	0.0198
metab_4412	pos	153.0105	6.7366	0.2284	-0.0124	0.3052	0.4371
metab_5019	pos	153.0106	3.6535	0.6669	-0.6662	0.0020	0.0120
metab_4928	pos	153.0106	4.0338	0.6883	-0.6825	0.0013	0.0091
metab_4975	pos	153.0106	3.8208	0.6515	-0.5672	0.0039	0.0185
metab_14375	neg	153.0185	1.4115	0.0042	0.3285	0.9444	0.9637
metab_6177	pos	153.0403	0.9200	0.7339	-1.6160	0.3411	0.4753
metab_4847	pos	153.0403	4.3818	0.7027	2.2239	0.3376	0.4716
metab_1759	pos	153.0907	1.9918	0.8710	0.9590	0.0007	0.0058
metab_1849	pos	153.0907	2.2519	1.0091	1.0903	0.0001	0.0016
metab_13090	neg	153.0912	3.9005	0.3781	-0.0114	0.0532	0.1228
metab_13457	neg	153.0913	2.9092	1.5436	3.6456	0.0017	0.0119
metab_5673	pos	153.1018	1.8729	0.3858	0.4988	0.2498	0.3751
metab_8127	neg	153.1024	1.2084	0.7346	8.9999	0.2091	0.3316
metab_2923	pos	153.1270	8.6132	0.2168	-0.2811	0.2687	0.3963
metab_2856	pos	153.1270	8.2728	0.0714	-0.3154	0.7831	0.8544
metab_2284	pos	153.1270	4.1544	1.5593	3.5001	0.0054	0.0233
metab_1887	pos	153.1271	2.3908	1.6146	2.6855	0.0000	0.0000
metab_10808	neg	153.8991	14.0771	0.6654	-0.3354	0.0005	0.0054
metab_7775	neg	153.9495	0.3959	0.4544	-0.1767	0.0166	0.0557
metab_10926	neg	153.9495	14.4028	0.3662	-0.0290	0.0379	0.0972
metab_1630	pos	153.9905	1.5705	0.4500	-0.3840	0.1688	0.2795
metab_6818	neg	154.0136	0.9792	1.0713	1.6976	0.0035	0.0190
metab_14203	neg	154.0137	1.5736	1.2177	2.0081	0.0002	0.0028
metab_47	pos	154.0363	1.5705	0.5388	0.8756	0.1233	0.2215
metab_5828	pos	154.0414	1.5705	0.3170	-0.2850	0.1210	0.2184
metab_1411	pos	154.0437	0.9200	0.7497	-1.8446	0.4082	0.5410
metab_1069	pos	154.0494	1.4289	2.2130	5.4984	0.0000	0.0000
metab_8402	neg	154.0501	1.6335	0.1270	0.0395	0.5545	0.6663

metab_6584	neg	154.0613	0.5431	0.4151	0.9251	0.3769	0.5074
metab_5804	pos	154.0858	1.6128	0.6777	0.6879	0.0023	0.0132
metab_8417	neg	154.0865	1.6642	1.0458	-1.0971	0.0085	0.0354
metab_14408	neg	154.0865	1.3517	0.1497	0.0361	0.5035	0.6235
metab_2123	pos	154.1223	3.3183	0.9012	0.9611	0.0008	0.0063
metab_163	pos	154.1334	0.5280	0.0243	0.0851	0.8082	0.8732
metab_15075	neg	154.9456	0.1425	0.2952	0.0095	0.0869	0.1755
metab_10919	neg	154.9456	14.5008	0.3548	-0.0263	0.0413	0.1031
metab_6699	neg	154.9828	0.4975	0.5778	-0.2606	0.0056	0.0266
metab_3318	pos	154.9898	14.1820	0.5160	-0.3972	0.0005	0.0048
metab_2565	pos	154.9898	6.1167	0.3995	-0.3100	0.0492	0.1129
metab_690	pos	154.9898	6.7825	0.2973	0.0296	0.1785	0.2922
metab_5369	pos	154.9898	2.4850	0.6234	-0.5410	0.0018	0.0109
metab_8291	neg	154.9977	1.4710	0.6112	-0.3955	0.0298	0.0823
metab_3327	pos	155.0023	14.2269	0.3911	-0.2989	0.0102	0.0365
metab_977	pos	155.0100	7.6332	0.0658	0.0225	0.7849	0.8555
metab_5969	pos	155.0334	1.3153	1.0558	3.3198	0.0132	0.0434
metab_6079	pos	155.0334	1.1447	1.8190	11.9245	0.0000	0.0000
metab_14612	neg	155.0340	0.9933	0.8636	1.3871	0.0110	0.0420
metab_13911	neg	155.0341	2.0202	1.3356	3.4259	0.0087	0.0362
metab_5820	pos	155.0447	1.5705	0.3146	-0.2832	0.1425	0.2466
metab_14830	neg	155.0452	0.5991	0.5777	-0.1773	0.0645	0.1416
metab_5783	pos	155.0528	1.6413	1.7687	5.2487	0.0000	0.0000
metab_7865	neg	155.0646	0.5431	0.4539	1.0144	0.3448	0.4778
metab_7458	neg	155.0705	1.5736	1.0891	1.8198	0.0018	0.0121
metab_13171	neg	155.0705	3.6645	0.7849	0.9863	0.0245	0.0724
metab_9139	neg	155.0706	3.5310	0.6880	0.9785	0.0504	0.1181
metab_6033	pos	155.0810	1.2016	0.5428	-0.6817	0.0452	0.1059
metab_14655	neg	155.0816	0.8946	0.8312	5.5893	0.2205	0.3445
metab_2593	pos	155.1062	6.4035	0.3249	-0.3072	0.2702	0.3984
metab_5733	pos	155.1063	1.7271	1.1415	1.4973	0.0001	0.0018
metab_442	pos	155.1063	2.3447	0.1493	-0.0260	0.6796	0.7750
metab_6921	neg	155.1069	3.6142	1.2632	-2.0118	0.0144	0.0506
metab_1417	pos	155.1175	0.9480	0.6081	-0.0307	0.2350	0.3592
metab_1492	pos	155.1175	1.1875	0.3190	-0.1691	0.2869	0.4172
metab_10718	neg	155.8733	13.8818	0.2075	0.0693	0.2114	0.3341
metab_11044	neg	155.8733	14.0438	0.2580	0.0507	0.1813	0.2994
metab_15131	neg	155.8961	0.0197	0.3810	-0.0432	0.0270	0.0774
metab_15022	neg	155.8962	0.4975	0.5424	-0.1335	0.0538	0.1239
metab_3445	pos	155.9739	14.1820	0.4078	-0.2973	0.0079	0.0305
metab_3393	pos	155.9740	15.0719	0.3834	-0.2654	0.0182	0.0543
metab_1194	pos	155.9929	0.2389	0.6048	-0.4957	0.0001	0.0022
metab_3436	pos	155.9977	14.2561	0.1974	0.1243	0.4280	0.5597
metab_3466	pos	156.0101	14.0948	0.4753	-0.3657	0.0015	0.0096
metab_2953	pos	156.0102	8.8028	0.5169	1.1861	0.0731	0.1508
metab_4910	pos	156.0102	4.0788	0.5066	-0.4695	0.0403	0.0971
metab_2193	pos	156.0102	3.6535	0.6603	-0.6169	0.0003	0.0031
metab_5066	pos	156.0102	3.4549	0.6591	-0.6349	0.0030	0.0158

metab_2234	pos	156.0103	3.8511	0.4100	-0.3585	0.0495	0.1133
metab_5312	pos	156.0103	2.6402	0.5556	-0.4557	0.0034	0.0170
metab_8005	neg	156.0292	0.8662	0.4607	-0.0455	0.1647	0.2799
metab_920	pos	156.0328	14.0665	0.2144	-0.2002	0.4635	0.5912
metab_5827	pos	156.0384	1.5705	0.3248	-0.2874	0.1077	0.2002
metab_6383	pos	156.0417	0.5843	2.3031	-12.7109	0.0039	0.0185
metab_13559	neg	156.0559	2.6841	0.4427	-0.2041	0.0657	0.1434
metab_1384	pos	156.0651	0.8500	0.1969	0.2896	0.5477	0.6663
metab_7483	neg	156.0658	1.5012	0.2482	0.4708	0.3565	0.4893
metab_6309	pos	156.0764	0.6402	0.2193	0.2448	0.5893	0.7014
metab_6449	pos	156.0764	0.5280	0.3204	0.5104	0.4668	0.5940
metab_7867	neg	156.0769	0.5431	1.7025	6.1670	0.0001	0.0025
metab_8073	neg	156.0770	1.0499	1.0063	1.0991	0.1060	0.2027
metab_5579	pos	156.0804	2.0677	0.1083	0.0939	0.7825	0.8541
metab_5727	pos	156.1015	1.7554	1.0202	1.1546	0.0000	0.0008
metab_13499	neg	156.1021	2.8123	0.0373	0.0861	0.8493	0.8954
metab_7439	neg	156.1021	1.5297	0.6649	1.0081	0.1304	0.2354
metab_7633	neg	156.1021	2.6841	0.0784	0.2714	0.8089	0.8641
metab_13372	neg	156.1022	3.1115	0.3770	-0.6468	0.3862	0.5155
metab_8633	neg	156.1022	2.0987	0.3729	-0.4492	0.3317	0.4644
metab_8530	neg	156.1022	1.8682	0.1967	-0.0385	0.5482	0.6603
metab_3999	pos	156.1379	8.6132	0.3950	-0.4010	0.0478	0.1101
metab_763	pos	156.1379	8.2143	0.4666	-0.5363	0.0882	0.1730
metab_1557	pos	156.1380	1.3865	1.1961	1.3400	0.0135	0.0440
metab_1795	pos	156.1380	2.0973	0.4797	0.1638	0.0103	0.0366
metab_5307	pos	156.1380	2.6719	0.4350	0.1536	0.0464	0.1078
metab_5106	pos	156.1380	3.3183	0.5425	0.4475	0.1327	0.2336
metab_13300	neg	156.1386	3.2972	0.5977	1.9732	0.3045	0.4355
metab_1157	pos	156.1492	0.5280	0.3140	-0.3638	0.2899	0.4204
metab_1226	pos	156.5922	0.4999	1.0018	9.4234	0.0308	0.0800
metab_15019	neg	156.8940	0.4975	0.0391	0.2807	0.8331	0.8829
metab_11004	neg	156.8941	14.0771	0.2577	0.0077	0.1505	0.2618
metab_15042	neg	156.9621	0.4975	0.3281	0.0248	0.1170	0.2172
metab_10693	neg	156.9801	11.2769	0.3312	0.0016	0.0557	0.1270
metab_931	pos	156.9902	14.0525	0.1592	0.0901	0.5253	0.6463
metab_8102	neg	156.9956	1.1503	0.8114	2.2882	0.1150	0.2143
metab_10962	neg	156.9977	14.1744	0.2677	0.0678	0.1751	0.2920
metab_8997	neg	157.0054	3.0260	1.2603	-1.4452	0.0015	0.0108
metab_14690	neg	157.0133	0.8382	0.7529	1.0960	0.0261	0.0756
metab_7967	neg	157.0245	0.7537	0.0242	0.2114	0.9819	0.9873
metab_8773	neg	157.0399	2.4338	0.4489	-0.1156	0.1510	0.2627
metab_8914	neg	157.0399	2.7961	0.5530	-0.2133	0.0073	0.0319
metab_348	pos	157.0418	1.5705	0.6236	0.4226	0.0008	0.0065
metab_5947	pos	157.0491	1.3437	1.4053	4.4005	0.0050	0.0219
metab_1447	pos	157.0492	1.0601	1.7093	3.7787	0.0000	0.0003
metab_5469	pos	157.0492	2.2519	0.0453	-0.0664	0.8299	0.8882
metab_8656	neg	157.0498	2.1630	1.4154	-1.9080	0.0000	0.0013
metab_14299	neg	157.0498	1.4856	0.6393	0.9426	0.0381	0.0977

metab_1703	pos	157.0603	1.7705	0.9018	9.5741	0.0734	0.1512
metab_498	pos	157.0644	2.5003	0.6083	0.7723	0.1070	0.1994
metab_2504	pos	157.0855	5.6773	0.9782	-1.5572	0.1189	0.2154
metab_1006	pos	157.0855	5.9361	1.6266	-0.6895	0.0457	0.1067
metab_4694	pos	157.0856	5.1076	0.3254	0.3716	0.5453	0.6644
metab_5502	pos	157.0857	2.1897	1.1888	1.8230	0.0001	0.0020
metab_8765	neg	157.0862	2.4175	0.9525	1.5191	0.0026	0.0156
metab_8565	neg	157.0862	1.9596	0.8607	1.2369	0.0051	0.0249
metab_1320	pos	157.0968	0.6262	1.1188	1.3084	0.0000	0.0000
metab_1790	pos	157.0969	2.0828	1.3208	1.7917	0.0002	0.0026
metab_4585	pos	157.1220	5.7540	1.3158	4.4550	0.0060	0.0250
metab_15059	neg	157.8621	0.4307	0.4623	-0.1373	0.0162	0.0549
metab_10850	neg	157.8621	14.1744	0.4654	-0.1252	0.0076	0.0329
metab_10785	neg	157.8712	14.0438	0.2679	0.0293	0.1207	0.2223
metab_11292	neg	157.9000	10.0593	0.3611	0.3418	0.3082	0.4390
metab_11243	neg	157.9000	10.2875	0.9007	1.8988	0.0269	0.0772
metab_10800	neg	157.9303	14.0610	0.2143	-0.0875	0.3666	0.4985
metab_11791	neg	157.9304	8.6093	1.4934	-2.2427	0.0003	0.0043
metab_10985	neg	157.9417	14.1090	0.3182	-0.1355	0.1673	0.2835
metab_3348	pos	157.9734	14.4708	0.5411	-0.4461	0.0008	0.0064
metab_114	pos	158.0484	14.0525	0.1304	-0.1584	0.5032	0.6255
metab_1638	pos	158.0596	1.5987	0.4854	0.4187	0.1377	0.2405
metab_13604	neg	158.0603	2.6046	0.8054	-0.8309	0.0155	0.0531
metab_8463	neg	158.0604	1.7419	0.8662	1.3793	0.0949	0.1868
metab_5964	pos	158.0808	1.3293	0.9428	1.1787	0.0004	0.0039
metab_8708	neg	158.0814	2.2912	0.3995	-0.7350	0.3569	0.4896
metab_8408	neg	158.0814	1.6491	0.2756	0.5897	0.5461	0.6588
metab_1364	pos	158.0920	0.7941	0.4942	0.4792	0.2739	0.4020
metab_5627	pos	158.0961	1.9918	0.3247	0.3571	0.4134	0.5456
metab_1099	pos	158.1171	1.1731	0.0147	-0.3721	0.9617	0.9769
metab_5332	pos	158.1172	2.5938	0.2712	-0.1145	0.5083	0.6305
metab_13597	neg	158.1178	2.6206	0.6410	-0.0626	0.2264	0.3511
metab_1401	pos	158.1283	0.8920	1.0399	-1.2576	0.0002	0.0030
metab_3567	pos	158.1534	11.2827	0.1036	-0.1871	0.6351	0.7397
metab_2601	pos	158.1534	6.4948	0.0200	-0.0532	0.9114	0.9427
metab_3280	pos	158.1535	14.0378	0.2947	-0.2272	0.0233	0.0651
metab_3729	pos	158.1536	9.7899	0.4305	-0.2901	0.0812	0.1627
metab_4740	pos	158.1536	4.9267	0.3881	-0.2886	0.0078	0.0301
metab_11160	neg	158.8457	12.1016	0.6240	-0.2728	0.0025	0.0151
metab_7766	neg	158.8700	0.1093	0.1652	0.0994	0.5426	0.6563
metab_10844	neg	158.8700	14.1581	0.4295	-0.1247	0.0358	0.0930
metab_11120	neg	158.8915	13.9796	0.2959	0.0107	0.1119	0.2110
metab_11180	neg	158.9210	11.1147	0.2813	0.0454	0.1317	0.2372
metab_7754	neg	158.9380	0.0382	0.3066	-0.1204	0.1491	0.2601
metab_8352	neg	158.9846	1.5439	0.8739	11.5423	0.0350	0.0916
metab_13402	neg	159.0024	3.0260	1.2985	-1.5066	0.0026	0.0157
metab_13963	neg	159.0116	1.9280	0.9950	2.2803	0.0899	0.1798
metab_3333	pos	159.0128	14.3019	0.0252	-0.1512	0.9671	0.9801

metab_3336	pos	159.0184	14.3315	0.2169	-0.1945	0.2969	0.4276
metab_3483	pos	159.0184	14.0665	0.1076	-0.0153	0.7692	0.8435
metab_6788	neg	159.0290	0.7958	0.7156	1.0394	0.0372	0.0957
metab_4226	pos	159.0413	7.6332	0.4068	0.4763	0.1651	0.2751
metab_269	pos	159.0648	1.0601	0.5609	-0.6494	0.0665	0.1407
metab_7524	neg	159.0654	1.9125	0.4684	0.9736	0.2552	0.3836
metab_6327	pos	159.0760	0.6262	1.0000	1.0104	0.0000	0.0001
metab_6130	pos	159.0760	1.0461	1.1513	-2.7582	0.0121	0.0409
metab_7916	neg	159.0766	0.5991	0.2911	0.0053	0.3778	0.5079
metab_14320	neg	159.0767	1.4568	1.0684	-1.2571	0.0337	0.0896
metab_13393	neg	159.0807	3.0604	1.7065	-4.4347	0.0295	0.0818
metab_13588	neg	159.0808	2.6357	1.2198	-3.8947	0.1275	0.2315
metab_345	pos	159.0912	1.5284	0.1883	-0.0724	0.6171	0.7251
metab_8351	neg	159.0919	1.5439	0.9077	-0.8219	0.1202	0.2216
metab_239	pos	159.0953	0.7941	0.6421	0.6783	0.1723	0.2845
metab_13986	neg	159.1018	1.8682	1.6778	3.6701	0.0002	0.0035
metab_8845	neg	159.1019	2.6206	0.1342	0.0372	0.7074	0.7906
metab_9374	neg	159.1019	4.4386	0.2553	0.3971	0.2386	0.3646
metab_9240	neg	159.1019	3.8678	0.1217	-0.1392	0.8187	0.8713
metab_6149	pos	159.1124	0.9761	0.4527	1.1568	0.3019	0.4334
metab_12903	neg	159.1171	4.5884	1.4667	-5.7303	0.0628	0.1389
metab_6313	pos	159.1206	0.6262	2.3695	11.2103	0.0000	0.0000
metab_12935	neg	159.8591	4.4386	0.9459	-0.6409	0.0314	0.0855
metab_15065	neg	159.8592	0.3959	0.4774	-0.1645	0.0154	0.0531
metab_10858	neg	159.8592	14.1904	0.4683	-0.1200	0.0064	0.0291
metab_9239	neg	159.8592	3.8506	0.7360	-0.3951	0.1091	0.2069
metab_11007	neg	159.8860	14.0771	0.0192	0.2221	0.9519	0.9688
metab_10923	neg	159.8930	14.4520	0.3744	-0.0311	0.0241	0.0717
metab_11137	neg	159.9182	13.8980	0.0776	0.1128	0.7263	0.8030
metab_3415	pos	159.9687	14.4708	0.5126	-0.3957	0.0007	0.0061
metab_14986	neg	159.9784	0.5126	0.7564	-0.7112	0.0087	0.0362
metab_8748	neg	160.0218	2.3704	1.6210	11.3954	0.0048	0.0236
metab_14308	neg	160.0396	1.4710	0.4230	0.6156	0.1857	0.3043
metab_13469	neg	160.0396	2.8606	0.1173	0.2051	0.8586	0.9023
metab_13119	neg	160.0396	3.8001	0.1610	-0.0049	0.4969	0.6176
metab_1383	pos	160.0423	0.8361	1.1380	1.9185	0.0390	0.0948
metab_14295	neg	160.0508	1.4856	0.3981	-0.0599	0.0852	0.1727
metab_4310	pos	160.0600	7.1560	1.5820	3.0757	0.0073	0.0287
metab_6074	pos	160.0600	1.1589	0.8011	1.0853	0.0253	0.0696
metab_7491	neg	160.0606	0.5991	0.4002	-0.2015	0.2415	0.3676
metab_340	pos	160.0752	1.5284	0.0854	-0.0132	0.7509	0.8292
metab_6004	pos	160.0753	1.2446	1.0362	1.1532	0.0069	0.0274
metab_5509	pos	160.0754	2.1897	0.7867	0.8538	0.0142	0.0456
metab_1139	pos	160.0964	0.6123	0.1236	0.0880	0.6439	0.7466
metab_14841	neg	160.0970	0.5991	0.7093	0.9244	0.0877	0.1765
metab_6257	pos	160.1076	0.7801	0.6711	0.7870	0.0339	0.0857
metab_6268	pos	160.1328	0.7801	0.3491	-0.1995	0.1883	0.3040
metab_6204	pos	160.1439	0.8640	1.0961	-0.4886	0.0317	0.0817

metab_6457	pos	160.1805	0.5140	0.9538	2.4576	0.0120	0.0406
metab_10836	neg	160.8670	14.1255	0.4921	-0.2801	0.0922	0.1826
metab_10880	neg	160.9118	14.4028	0.4555	-0.0759	0.0105	0.0409
metab_10984	neg	160.9169	14.1090	0.4911	-0.2152	0.0482	0.1146
metab_15109	neg	160.9283	0.0197	0.1756	-0.2138	0.6777	0.7678
metab_7680	neg	160.9752	0.5126	0.7561	-0.6942	0.0073	0.0319
metab_6701	neg	160.9818	0.5126	0.7974	-0.9157	0.0138	0.0490
metab_14404	neg	161.0003	1.3517	2.0038	11.6810	0.0000	0.0001
metab_13338	neg	161.0236	3.1964	0.5006	-0.2601	0.0097	0.0391
metab_13113	neg	161.0351	3.8164	0.5916	1.1838	0.1140	0.2130
metab_14647	neg	161.0447	0.9227	0.7162	-0.4755	0.0139	0.0493
metab_5937	pos	161.0594	1.3720	1.0529	1.7283	0.0303	0.0791
metab_9138	neg	161.0599	3.5310	0.5009	-0.5885	0.1168	0.2170
metab_13529	neg	161.0599	2.7475	0.4441	-0.4703	0.1628	0.2777
metab_9098	neg	161.0600	3.3631	0.6152	-0.8338	0.0678	0.1465
metab_6036	pos	161.0706	1.2016	0.9620	2.3633	0.1280	0.2274
metab_6616	neg	161.0811	1.1940	0.3481	0.5604	0.3155	0.4474
metab_14134	neg	161.0811	1.6642	0.3445	0.5309	0.2634	0.3934
metab_14011	neg	161.0924	1.8371	1.2624	-1.7168	0.0077	0.0330
metab_1909	pos	161.0956	2.4530	1.2487	2.1622	0.0010	0.0075
metab_5167	pos	161.0957	3.1191	1.4237	2.9043	0.0013	0.0087
metab_373	pos	161.1069	1.7271	0.1236	0.0245	0.6901	0.7829
metab_1249	pos	161.1280	0.5280	0.4236	0.4218	0.2338	0.3578
metab_2860	pos	161.1320	8.2872	0.0542	-0.0425	0.7025	0.7923
metab_4651	pos	161.1320	5.3454	0.2041	0.2207	0.6556	0.7557
metab_4332	pos	161.1321	7.0670	0.5116	-0.5501	0.1462	0.2513
metab_2350	pos	161.1321	4.5785	0.8061	0.6779	0.0041	0.0191
metab_9565	neg	161.8489	5.4355	0.5677	-0.2385	0.0695	0.1491
metab_10909	neg	161.8749	15.0662	0.4349	-0.0860	0.0129	0.0470
metab_10878	neg	161.8749	14.3862	0.3670	-0.0251	0.0335	0.0894
metab_7746	neg	161.8838	0.0259	0.2537	0.0610	0.1353	0.2422
metab_11006	neg	161.8838	14.0771	0.0114	0.1764	0.9611	0.9736
metab_15025	neg	161.8931	0.4975	0.4643	-0.1650	0.0137	0.0489
metab_10724	neg	161.9127	13.9143	0.2825	0.0188	0.1257	0.2292
metab_11801	neg	161.9127	8.6093	0.2280	0.0718	0.3346	0.4675
metab_10910	neg	161.9247	14.9330	0.2005	0.0519	0.2988	0.4292
metab_6685	neg	161.9325	0.0197	0.3338	-0.0330	0.0616	0.1370
metab_6354	pos	161.9946	0.5983	1.0774	1.4064	0.0046	0.0206
metab_8515	neg	162.0189	1.8371	1.0901	1.5998	0.0000	0.0009
metab_1672	pos	162.0546	1.6983	0.9424	1.1064	0.0002	0.0029
metab_8135	neg	162.0552	1.2236	0.1195	0.4366	0.7415	0.8138
metab_14894	neg	162.0763	0.5571	0.2884	0.2087	0.2754	0.4056
metab_5354	pos	162.0910	2.5314	0.1131	-0.2805	0.8064	0.8717
metab_13905	neg	162.0916	2.0202	0.6950	3.6902	0.3641	0.4962
metab_8811	neg	162.0917	2.5264	0.0800	0.1845	0.8899	0.9260
metab_1303	pos	162.1064	0.5983	0.6928	-0.7528	0.0360	0.0892
metab_156	pos	162.1120	0.5983	0.6449	-0.5096	0.0089	0.0333
metab_3481	pos	162.1273	14.0665	0.0342	-0.2246	0.8223	0.8832

metab_4642	pos	162.1273	5.3913	0.4464	0.1743	0.3691	0.5029
metab_10854	neg	162.8779	14.1744	0.4654	-0.1490	0.0155	0.0532
metab_11116	neg	162.8925	13.9952	0.3855	-0.0667	0.0328	0.0882
metab_15095	neg	162.8926	0.0320	0.3151	-0.0390	0.1023	0.1973
metab_7794	neg	162.8926	0.4975	0.2999	-0.2197	0.3823	0.5120
metab_10876	neg	162.9200	14.3529	0.2117	0.0581	0.2561	0.3847
metab_7927	neg	163.0239	0.6131	0.5206	0.8879	0.1910	0.3108
metab_8731	neg	163.0393	2.3380	0.3914	0.0838	0.3880	0.5170
metab_325	pos	163.0419	1.4569	0.1999	0.0370	0.6419	0.7449
metab_1388	pos	163.0497	0.8500	0.9308	1.2203	0.0577	0.1265
metab_5694	pos	163.0750	1.7993	0.8141	1.2016	0.0374	0.0918
metab_6022	pos	163.0861	1.2162	0.3537	-0.6339	0.2842	0.4143
metab_1202	pos	163.0942	0.4387	0.0135	-0.2985	0.9793	0.9882
metab_709	pos	163.1114	7.0670	0.3780	-0.4200	0.2343	0.3584
metab_619	pos	163.1114	4.9267	0.1671	-0.0154	0.6675	0.7651
metab_13342	neg	163.1120	3.1797	0.0894	0.2880	0.9911	0.9933
metab_1503	pos	163.1225	1.2016	0.6562	0.8907	0.1213	0.2188
metab_5908	pos	163.1226	1.4289	0.6435	-0.6792	0.0597	0.1296
metab_2721	pos	163.1476	7.2614	0.0175	-0.3882	0.9824	0.9891
metab_792	pos	163.1476	8.6132	0.3703	-0.3755	0.0506	0.1149
metab_2851	pos	163.1476	8.2435	0.0081	-0.0919	0.9403	0.9630
metab_4495	pos	163.1476	6.2522	0.0169	0.2476	0.9406	0.9632
metab_4665	pos	163.1476	5.2717	1.3597	3.3537	0.0153	0.0481
metab_4531	pos	163.1477	5.9949	0.0124	0.7418	0.9950	0.9968
metab_9775	neg	163.8390	6.5989	0.5684	-0.2405	0.2187	0.3424
metab_7744	neg	163.8812	0.0197	0.1630	0.0879	0.3733	0.5045
metab_11094	neg	163.9126	14.0117	0.4591	-0.6628	0.1995	0.3202
metab_10817	neg	163.9127	14.0771	0.1713	0.1919	0.4314	0.5561
metab_6686	neg	163.9279	0.0197	0.3362	-0.0309	0.0553	0.1265
metab_10987	neg	163.9280	14.1090	0.5308	-0.1592	0.0021	0.0137
metab_10873	neg	163.9405	14.3039	0.2727	0.0257	0.1355	0.2423
metab_8510	neg	164.0167	1.8205	0.5045	0.5295	0.3440	0.4771
metab_13607	neg	164.0345	2.6046	1.0321	2.2043	0.0164	0.0551
metab_5886	pos	164.0549	1.4569	0.1612	0.3959	0.8260	0.8854
metab_9102	neg	164.0639	3.3971	0.3420	-0.1088	0.2023	0.3238
metab_13950	neg	164.0639	1.9596	0.5046	0.7293	0.1641	0.2793
metab_14483	neg	164.0652	1.2663	0.4727	0.7065	0.1596	0.2737
metab_6097	pos	164.0701	1.1164	0.6022	0.6289	0.0332	0.0845
metab_14475	neg	164.0709	1.2805	0.9890	-1.1805	0.0153	0.0526
metab_6298	pos	164.0913	0.6542	0.0545	0.2623	0.9337	0.9581
metab_1820	pos	164.0926	2.1595	0.2578	-0.4052	0.5742	0.6891
metab_1082	pos	164.1064	1.3153	0.5628	0.6816	0.0882	0.1730
metab_2100	pos	164.1067	3.2420	0.3121	-0.2366	0.0728	0.1505
metab_5231	pos	164.1067	2.9192	0.2147	-0.0067	0.6218	0.7289
metab_13234	neg	164.1073	3.5141	0.8069	-2.0393	0.2033	0.3249
metab_14124	neg	164.1074	1.6642	0.8389	1.0697	0.0298	0.0823
metab_8994	neg	164.1074	3.0101	0.8632	-1.4517	0.0768	0.1602
metab_10710	neg	164.8384	13.6682	0.7809	-0.5691	0.0018	0.0121

metab_11125	neg	164.8385	13.9796	0.5618	-0.1802	0.0015	0.0108
metab_15102	neg	164.9039	0.0259	0.3874	-0.0883	0.0416	0.1037
metab_7827	neg	164.9342	0.5286	0.7048	-0.5395	0.0091	0.0373
metab_14981	neg	164.9437	0.5286	1.1082	-1.2503	0.0008	0.0070
metab_14879	neg	164.9562	0.5571	1.0781	2.9361	0.0480	0.1142
metab_7692	neg	164.9631	0.4975	0.5185	-0.1822	0.0098	0.0392
metab_6342	pos	164.9879	0.6123	1.3723	-1.7107	0.0011	0.0082
metab_8541	neg	165.0185	1.9125	0.4268	0.7933	0.2227	0.3470
metab_14171	neg	165.0186	1.6028	0.2049	0.2687	0.4024	0.5306
metab_14845	neg	165.0396	0.5991	1.0645	-1.1464	0.0011	0.0091
metab_243	pos	165.0542	0.8221	0.3623	-0.4439	0.2171	0.3390
metab_7610	neg	165.0549	2.5264	1.3804	-2.0191	0.0001	0.0023
metab_1224	pos	165.0659	0.4999	0.3629	-0.3723	0.0694	0.1452
metab_162	pos	165.0753	0.8920	0.0207	-0.1700	0.9991	0.9994
metab_14791	neg	165.0759	0.6131	0.6920	-0.4765	0.0076	0.0328
metab_613	pos	165.0907	2.2045	1.1554	1.9976	0.0052	0.0226
metab_1956	pos	165.0907	2.6561	1.4482	2.6295	0.0001	0.0021
metab_13640	neg	165.1116	2.5264	1.6339	-2.8588	0.0003	0.0042
metab_4546	pos	165.1270	5.9504	0.3360	0.0430	0.5019	0.6246
metab_1541	pos	165.1381	1.3293	0.6912	-0.8141	0.0982	0.1868
metab_5785	pos	165.1382	1.6413	0.7166	-0.8414	0.1407	0.2444
metab_3983	pos	165.1634	8.6284	0.2520	-0.2433	0.2474	0.3727
metab_7778	neg	165.8431	0.4307	0.4917	-0.1702	0.0075	0.0324
metab_7169	neg	165.8431	14.1744	0.4475	-0.1100	0.0108	0.0418
metab_1280	pos	165.9663	0.5560	0.2523	-0.5999	0.5709	0.6862
metab_13166	neg	165.9782	3.6812	1.2674	-3.3113	0.0332	0.0889
metab_929	pos	165.9824	14.0805	0.3675	-0.2940	0.0461	0.1075
metab_10831	neg	165.9926	14.1090	0.3619	-0.0819	0.0811	0.1669
metab_14531	neg	166.0387	1.1940	0.1354	4.7512	0.4682	0.5906
metab_6059	pos	166.0495	1.1875	1.3292	2.2195	0.0001	0.0014
metab_11973	neg	166.0501	8.0843	0.7517	1.0776	0.0520	0.1208
metab_14357	neg	166.0502	1.4427	0.1545	0.2960	0.7531	0.8228
metab_8668	neg	166.0502	2.1956	0.0376	-0.0114	0.9489	0.9666
metab_6500	pos	166.0640	0.4999	0.9657	-1.8357	0.0195	0.0571
metab_5982	pos	166.0800	1.2725	0.2055	-0.1656	0.4664	0.5938
metab_72	pos	166.0858	1.2725	0.5881	-0.7359	0.0899	0.1756
metab_12946	neg	166.0865	4.3716	0.4237	-0.3960	0.3308	0.4636
metab_14254	neg	166.0865	1.5297	0.4204	-0.3890	0.2455	0.3721
metab_13776	neg	166.0866	2.2595	0.2376	-0.1292	0.5072	0.6266
metab_13943	neg	166.0866	1.9745	0.1286	0.0616	0.6678	0.7605
metab_1220	pos	166.1134	0.4999	1.2658	11.0414	0.0020	0.0119
metab_5716	pos	166.1222	1.7705	1.3866	1.9773	0.0005	0.0050
metab_5901	pos	166.1222	1.4429	1.9923	7.0942	0.0000	0.0005
metab_1960	pos	166.1223	2.6869	0.0036	-0.4015	0.9142	0.9438
metab_10992	neg	166.8324	14.0927	0.3326	-0.1667	0.1379	0.2460
metab_10979	neg	166.9325	14.1255	0.4487	-0.0657	0.0357	0.0928
metab_6900	neg	166.9979	1.4427	0.7734	0.9945	0.0128	0.0467
metab_7939	neg	167.0010	0.6271	0.7046	2.7874	0.2754	0.4056

metab_940	pos	167.0123	14.0525	0.2347	-0.1809	0.0473	0.1096
metab_381	pos	167.0335	1.7845	1.9193	5.9000	0.0000	0.0005
metab_6820	neg	167.0341	0.9933	0.1413	0.3363	0.6291	0.7279
metab_8831	neg	167.0342	2.5893	0.6389	0.8056	0.1911	0.3108
metab_14202	neg	167.0342	1.5736	0.3133	0.3983	0.2902	0.4215
metab_1750	pos	167.0699	1.9609	1.4762	2.5812	0.0000	0.0007
metab_5807	pos	167.0699	1.5987	1.6625	3.1792	0.0000	0.0004
metab_1671	pos	167.0810	1.6983	0.2550	0.1309	0.4865	0.6117
metab_6154	pos	167.0810	0.9761	0.2692	0.3260	0.5206	0.6422
metab_1533	pos	167.0891	1.2725	0.6043	-0.7616	0.0839	0.1664
metab_4664	pos	167.1062	5.2717	1.1531	1.6297	0.0074	0.0289
metab_367	pos	167.1062	1.6983	1.2369	1.8000	0.0000	0.0005
metab_579	pos	167.1062	4.0939	1.2594	1.9129	0.0138	0.0449
metab_1033	pos	167.1062	1.9169	1.1345	1.5072	0.0000	0.0006
metab_9217	neg	167.1071	3.8001	0.2744	0.3788	0.3927	0.5212
metab_6240	pos	167.1174	0.7941	1.0614	-1.7324	0.0056	0.0239
metab_257	pos	167.1174	0.9761	0.1153	0.3182	0.8642	0.9132
metab_6038	pos	167.1175	1.2016	0.3600	0.6261	0.4404	0.5710
metab_7452	neg	167.1182	1.5439	1.1760	3.7449	0.0298	0.0823
metab_4674	pos	167.1426	5.2129	0.1543	-0.2855	0.8220	0.8831
metab_2929	pos	167.1426	8.6284	0.1245	-0.1535	0.6747	0.7711
metab_4104	pos	167.1427	8.1857	0.2878	-0.3145	0.3139	0.4467
metab_12428	neg	167.8359	6.5989	0.6106	-0.2169	0.1707	0.2871
metab_11127	neg	167.8359	13.9633	0.1616	0.0769	0.5055	0.6251
metab_3338	pos	167.9816	14.3777	0.3895	-0.2823	0.0223	0.0631
metab_14660	neg	168.0294	0.8946	0.6097	0.8301	0.0798	0.1651
metab_6332	pos	168.0650	0.6262	1.7008	3.0884	0.0000	0.0000
metab_3368	pos	168.0651	15.0879	0.6357	0.3246	0.0000	0.0002
metab_1185	pos	168.0651	0.0552	0.6815	0.4423	0.0000	0.0007
metab_2499	pos	168.0651	5.6473	0.7235	0.4416	0.0003	0.0038
metab_8581	neg	168.0659	1.9904	0.2741	0.4731	0.4835	0.6056
metab_14927	neg	168.0770	0.5431	0.2780	0.6257	0.6000	0.7051
metab_5987	pos	168.0924	1.2725	0.5454	-0.5689	0.0277	0.0744
metab_6260	pos	168.1014	0.7801	1.8219	4.1173	0.0002	0.0025
metab_13992	neg	168.1021	1.8682	0.3819	-1.5188	0.4889	0.6105
metab_8468	neg	168.1022	1.7419	0.3093	-1.0466	0.5497	0.6618
metab_13730	neg	168.1022	2.3533	0.7492	-0.6040	0.0991	0.1925
metab_7641	neg	168.1022	2.7475	0.6814	0.9553	0.0855	0.1732
metab_4086	pos	168.1379	8.2586	0.3214	-0.5680	0.3662	0.4998
metab_7166	neg	168.8862	14.1090	0.4271	-0.0935	0.0164	0.0552
metab_7702	neg	168.9307	0.0197	0.1353	-0.0178	0.5458	0.6587
metab_15072	neg	168.9452	0.2104	0.3027	-0.0385	0.1132	0.2125
metab_10929	neg	168.9452	14.3693	0.3637	-0.0272	0.0338	0.0898
metab_14666	neg	168.9804	0.8803	1.0021	-0.8665	0.0149	0.0518
metab_9919	neg	169.0069	7.2069	0.2726	-0.8527	0.5438	0.6572
metab_10078	neg	169.0069	7.9137	0.8890	-1.0287	0.0072	0.0315
metab_11927	neg	169.0069	8.1936	1.2856	-1.8470	0.0004	0.0049
metab_8082	neg	169.0069	1.0785	1.1804	-1.3035	0.0003	0.0042

metab_8292	neg	169.0134	1.4710	1.1435	2.4007	0.0161	0.0545
metab_8387	neg	169.0135	1.6028	1.3627	3.6197	0.0113	0.0430
metab_14709	neg	169.0246	0.7818	0.5717	9.9477	0.2318	0.3571
metab_14524	neg	169.0328	1.2084	0.7159	1.6393	0.1288	0.2333
metab_14003	neg	169.0400	1.8524	1.9305	8.9598	0.0019	0.0128
metab_6521	pos	169.0454	0.4999	0.7968	-1.0974	0.0043	0.0197
metab_5507	pos	169.0491	2.1897	1.3460	2.7339	0.0060	0.0249
metab_8191	neg	169.0498	1.3089	0.8320	1.1296	0.0059	0.0274
metab_1281	pos	169.0579	0.5703	2.0362	4.9418	0.0199	0.0581
metab_5600	pos	169.0756	2.0211	0.8863	1.1417	0.0086	0.0324
metab_5611	pos	169.0855	2.0062	1.0287	1.2130	0.0001	0.0017
metab_8789	neg	169.0862	2.4651	0.1894	0.1390	0.6849	0.7735
metab_13843	neg	169.0863	2.1311	0.3833	-1.3093	0.4473	0.5708
metab_5818	pos	169.0966	1.5847	1.1225	-1.5625	0.0008	0.0065
metab_1883	pos	169.1220	2.3751	1.1429	1.9005	0.0011	0.0081
metab_5265	pos	169.1220	2.8108	1.1794	1.7305	0.0003	0.0032
metab_6946	neg	169.1226	4.2203	0.1684	-0.2326	0.7845	0.8462
metab_9093	neg	169.1227	3.3465	1.6318	3.1745	0.0000	0.0003
metab_12842	neg	169.1227	4.7881	0.0801	-0.3518	0.9444	0.9637
metab_13087	neg	169.1227	3.9005	1.3291	1.8813	0.0298	0.0823
metab_6102	pos	169.1331	1.1024	0.7402	-0.6866	0.0216	0.0617
metab_10871	neg	169.9066	14.2720	0.5075	-0.1872	0.0075	0.0325
metab_15092	neg	169.9066	0.0382	0.3251	-0.0304	0.1046	0.2006
metab_4764	pos	169.9769	4.8053	0.5986	-0.5485	0.0125	0.0418
metab_6037	pos	170.0443	1.2016	0.7886	1.0076	0.0132	0.0435
metab_5494	pos	170.0444	2.2045	0.0821	-0.1671	0.8267	0.8861
metab_14617	neg	170.0451	0.9792	0.2267	-0.0822	0.4025	0.5306
metab_8129	neg	170.0451	1.2236	0.8500	1.2583	0.0103	0.0404
metab_13219	neg	170.0603	3.5475	0.4433	-0.4381	0.3043	0.4352
metab_3492	pos	170.0657	14.0525	0.1134	-0.1091	0.6476	0.7496
metab_6520	pos	170.0658	0.4999	0.8011	-1.1220	0.0062	0.0256
metab_7468	neg	170.0815	1.6335	0.2169	-0.0125	0.4900	0.6114
metab_7534	neg	170.0815	2.0055	0.2967	0.7883	0.6160	0.7189
metab_1124	pos	170.0919	0.6262	0.0877	0.1000	0.8504	0.9038
metab_1771	pos	170.0960	2.0211	0.2838	0.1500	0.4138	0.5458
metab_1860	pos	170.0960	2.2824	0.2426	-0.3324	0.5178	0.6394
metab_5813	pos	170.1170	1.5987	1.1959	1.6978	0.0001	0.0019
metab_480	pos	170.1172	2.7497	0.8968	1.0349	0.0022	0.0127
metab_5477	pos	170.1172	2.2519	0.9496	0.9110	0.0013	0.0091
metab_8634	neg	170.1179	2.1153	1.3661	3.2241	0.0102	0.0402
metab_4000	pos	170.1534	8.6132	0.3318	-0.3516	0.1343	0.2360
metab_4937	pos	170.1535	3.9876	1.2514	2.8628	0.0270	0.0728
metab_2844	pos	170.1535	8.2143	0.3557	-0.3331	0.1023	0.1928
metab_7790	neg	170.8325	0.4975	0.8681	-0.9949	0.0041	0.0214
metab_11031	neg	170.8325	14.0610	0.1171	0.2629	0.6705	0.7623
metab_12423	neg	170.8325	6.6320	0.4867	-0.1831	0.1487	0.2598
metab_9542	neg	170.8325	5.3060	0.2621	-0.0290	0.2763	0.4067
metab_12567	neg	170.8325	6.0346	0.1336	0.1588	0.7506	0.8216

metab_12827	neg	170.8325	4.8541	0.3260	-0.0298	0.1698	0.2862
metab_10963	neg	170.8701	14.1744	0.4258	-0.1070	0.0187	0.0603
metab_15099	neg	170.8701	0.0320	0.3927	-0.0769	0.0253	0.0740
metab_10828	neg	170.8833	14.1090	0.4241	-0.1017	0.0086	0.0359
metab_14876	neg	170.9013	0.5571	0.3299	1.3175	0.5579	0.6692
metab_10885	neg	170.9406	14.5008	0.3592	-0.0316	0.0382	0.0977
metab_7772	neg	170.9406	0.3798	0.2916	0.0154	0.1019	0.1968
metab_11108	neg	170.9434	14.0117	0.5010	-0.1607	0.0204	0.0639
metab_8273	neg	170.9750	1.4568	0.3303	0.0133	0.3194	0.4518
metab_8883	neg	170.9848	2.7318	0.1854	1.9730	0.9091	0.9399
metab_9068	neg	170.9848	3.2801	1.0893	-1.4043	0.0305	0.0839
metab_921	pos	171.0051	14.0665	0.0047	0.1171	0.8589	0.9093
metab_7857	neg	171.0056	0.5431	0.0282	0.1695	0.9187	0.9465
metab_14170	neg	171.0113	1.6028	1.2425	4.2256	0.0496	0.1168
metab_11073	neg	171.0192	14.0282	0.1093	0.0643	0.6002	0.7051
metab_6115	pos	171.0218	1.0601	1.2133	-1.0209	0.0127	0.0423
metab_14491	neg	171.0291	1.2663	0.6427	-0.4535	0.0188	0.0604
metab_6040	pos	171.0647	1.2016	0.2161	-0.2133	0.4308	0.5621
metab_7587	neg	171.0656	2.3533	0.7122	1.1321	0.0623	0.1382
metab_8440	neg	171.0656	1.7104	1.2604	2.4457	0.0010	0.0083
metab_13428	neg	171.0656	2.9931	0.3006	0.4087	0.2948	0.4254
metab_413	pos	171.0761	2.0973	0.8046	0.7893	0.0089	0.0334
metab_7506	neg	171.0767	1.8371	0.5225	-0.4357	0.1505	0.2618
metab_8535	neg	171.0920	1.8833	0.0200	0.4488	0.7881	0.8484
metab_990	pos	171.1011	6.4035	0.5526	0.9557	0.2267	0.3499
metab_142	pos	171.1011	4.9267	0.1515	-0.2279	0.7766	0.8494
metab_5603	pos	171.1012	2.0211	0.9857	1.2595	0.0003	0.0033
metab_14037	neg	171.1019	1.8046	0.3028	-0.0535	0.3587	0.4916
metab_9752	neg	171.1019	6.4709	0.9228	1.3269	0.0004	0.0050
metab_8785	neg	171.1019	2.4487	0.2499	0.4129	0.7392	0.8119
metab_9604	neg	171.1019	5.6615	0.0335	0.1642	0.9970	0.9976
metab_9438	neg	171.1020	4.7217	0.1141	0.0597	0.7427	0.8146
metab_9314	neg	171.1020	4.1535	0.5039	0.5510	0.1370	0.2446
metab_13272	neg	171.1020	3.3803	0.0027	0.2971	0.9869	0.9904
metab_13157	neg	171.1020	3.7148	0.9247	1.3176	0.0001	0.0024
metab_7678	neg	171.1020	3.0432	0.7056	0.8023	0.0609	0.1358
metab_6266	pos	171.1123	0.7801	0.6721	0.7246	0.0151	0.0478
metab_9995	neg	171.9097	7.5855	0.7121	0.9776	0.0451	0.1097
metab_12195	neg	171.9097	7.4465	0.7183	0.9548	0.0103	0.0403
metab_15053	neg	171.9361	0.4634	0.3612	-0.0884	0.0475	0.1137
metab_9673	neg	171.9461	6.0346	0.0206	0.2391	0.9528	0.9694
metab_12850	neg	171.9461	4.7709	0.0784	0.2981	0.8450	0.8926
metab_13152	neg	171.9461	3.7317	0.1207	0.5282	0.5306	0.6470
metab_13349	neg	171.9462	3.1626	0.0204	0.3649	0.9631	0.9746
metab_6558	pos	171.9925	0.0720	0.5697	-0.4629	0.0002	0.0028
metab_3405	pos	171.9925	14.5638	0.5301	-0.4234	0.0011	0.0078
metab_3325	pos	172.0050	14.2127	0.5016	-0.3841	0.0012	0.0083
metab_14377	neg	172.0066	1.3965	0.7037	-0.9693	0.1624	0.2773

metab_3306	pos	172.0253	14.1090	0.4676	-0.4349	0.0123	0.0414
metab_8885	neg	172.0397	2.7475	0.2539	-0.0321	0.3649	0.4969
metab_3458	pos	172.0414	14.1090	0.5147	-0.4226	0.0110	0.0383
metab_14154	neg	172.0609	1.6335	0.0678	0.1470	0.7295	0.8055
metab_14212	neg	172.0689	1.5586	0.0486	0.2292	0.7153	0.7961
metab_5330	pos	172.0753	2.5938	0.8204	-0.9539	0.0007	0.0061
metab_1358	pos	172.0962	0.7801	0.2979	-0.7417	0.3733	0.5072
metab_336	pos	172.0963	1.5144	0.7483	0.9508	0.0090	0.0336
metab_13707	neg	172.0972	2.4010	0.1193	0.2896	0.8109	0.8654
metab_1594	pos	172.1326	1.4860	0.8458	0.7211	0.0002	0.0023
metab_6120	pos	172.1327	1.0601	0.0139	-0.0953	0.9799	0.9882
metab_1939	pos	172.1328	2.5938	0.8085	1.1490	0.0367	0.0904
metab_1874	pos	172.1328	2.3447	0.1473	-0.0497	0.6247	0.7309
metab_4573	pos	172.1691	5.8148	0.2719	-0.2530	0.1519	0.2585
metab_15040	neg	172.8295	0.4975	0.8731	-0.9648	0.0038	0.0204
metab_10795	neg	172.8296	14.0610	0.0603	0.2397	0.8984	0.9320
metab_7720	neg	172.8629	0.0382	0.4287	-0.1262	0.0214	0.0660
metab_15121	neg	172.8669	0.0197	0.2766	0.0138	0.1052	0.2015
metab_3460	pos	172.9559	14.1090	0.5570	-0.4423	0.0005	0.0047
metab_13307	neg	172.9818	3.2801	1.1531	-1.4479	0.0382	0.0978
metab_7971	neg	172.9849	0.7678	0.4020	-0.0404	0.5692	0.6798
metab_8239	neg	172.9907	1.3817	0.5114	-0.1410	0.2246	0.3492
metab_10754	neg	173.0021	14.0117	0.4442	-0.4257	0.3756	0.5063
metab_14727	neg	173.0084	0.7818	0.7177	-0.4801	0.0249	0.0732
metab_11049	neg	173.0299	14.0438	0.5298	-0.2072	0.0058	0.0271
metab_14536	neg	173.0448	1.1940	0.6548	0.9596	0.0450	0.1095
metab_6767	neg	173.0560	0.6131	0.0771	0.2317	0.6631	0.7568
metab_6887	neg	173.0561	1.3817	0.1542	-0.2946	0.7180	0.7976
metab_8707	neg	173.0600	2.2754	1.8189	-3.4654	0.0025	0.0151
metab_1578	pos	173.0804	1.4429	1.4018	-2.6477	0.0005	0.0048
metab_8078	neg	173.0812	1.0785	0.9779	2.5726	0.0324	0.0873
metab_8800	neg	173.0812	2.5113	0.2035	0.3206	0.6451	0.7414
metab_14839	neg	173.0923	0.5991	1.1765	-1.1192	0.0001	0.0018
metab_14929	neg	173.1036	0.5431	1.7875	3.8686	0.0006	0.0060
metab_2597	pos	173.1168	6.4639	1.0944	-1.4302	0.0196	0.0575
metab_621	pos	173.1168	4.9413	0.0093	-0.2701	0.9787	0.9881
metab_1871	pos	173.1169	2.3292	0.4984	0.3874	0.1569	0.2647
metab_13019	neg	173.1176	4.1364	0.4455	-0.5622	0.3210	0.4532
metab_9429	neg	173.1176	4.6722	0.2743	0.5609	0.5189	0.6370
metab_1958	pos	173.1283	2.6719	0.5453	-0.8016	0.1151	0.2102
metab_1369	pos	173.1392	0.7941	0.4379	7.7183	0.0754	0.1537
metab_10994	neg	173.8702	14.0927	0.3995	-0.1279	0.0838	0.1706
metab_15021	neg	173.9523	0.4975	0.1309	0.2058	0.5981	0.7038
metab_14392	neg	174.0118	1.3669	1.2177	-1.3025	0.0086	0.0357
metab_924	pos	174.0207	14.1090	0.4485	-0.3395	0.0017	0.0108
metab_3388	pos	174.0208	15.5562	0.4759	-0.3694	0.0037	0.0177
metab_14532	neg	174.0223	1.1940	0.6874	1.7992	0.2936	0.4247
metab_8192	neg	174.0224	1.3089	0.1100	0.2336	0.6791	0.7691

metab_6780	neg	174.0400	0.7818	0.8481	1.2302	0.0065	0.0292
metab_8451	neg	174.0401	1.7264	1.0999	-2.3370	0.0224	0.0680
metab_8161	neg	174.0401	1.2663	0.3289	0.6425	0.4918	0.6132
metab_5789	pos	174.0546	1.6272	1.3387	1.8016	0.0000	0.0010
metab_8784	neg	174.0553	2.4487	1.2799	-2.2721	0.0027	0.0163
metab_8536	neg	174.0667	1.8833	0.2835	-0.0919	0.3001	0.4303
metab_6823	neg	174.0764	0.9792	0.7899	1.2155	0.0104	0.0407
metab_13495	neg	174.0917	2.8269	0.2437	-0.0214	0.4586	0.5814
metab_7866	neg	174.1069	0.5431	1.9387	4.5154	0.0005	0.0057
metab_6027	pos	174.1120	1.2162	0.7973	0.8378	0.0003	0.0036
metab_6258	pos	174.1120	0.7801	0.3701	-0.2273	0.0818	0.1634
metab_5411	pos	174.1121	2.4071	0.8800	0.7591	0.0013	0.0087
metab_6208	pos	174.1232	0.8640	0.8003	0.9371	0.0061	0.0251
metab_6205	pos	174.1484	0.8640	1.7536	12.3818	0.0001	0.0011
metab_1455	pos	174.1484	1.0883	1.1751	-1.4308	0.0000	0.0001
metab_5302	pos	174.1485	2.6869	0.0760	0.4523	0.8934	0.9318
metab_7795	neg	174.8284	0.4975	0.9573	-1.0677	0.0021	0.0136
metab_10385	neg	174.8602	9.0230	0.4153	-0.0239	0.0614	0.1368
metab_6415	pos	174.8955	0.5420	0.3307	-0.2952	0.1407	0.2444
metab_7191	neg	174.9554	14.0438	0.3232	-0.0015	0.0521	0.1211
metab_115	pos	174.9559	14.0665	0.5320	-0.4088	0.0037	0.0177
metab_14019	neg	174.9983	1.8205	2.2358	-3.4513	0.0002	0.0034
metab_7944	neg	175.0240	0.6411	0.4659	0.7273	0.1739	0.2907
metab_8766	neg	175.0393	2.4175	0.7015	-0.8788	0.1354	0.2423
metab_13788	neg	175.0393	2.2272	0.4234	-0.5698	0.2304	0.3556
metab_8497	neg	175.0542	1.8046	2.2726	-4.6156	0.0000	0.0005
metab_7500	neg	175.0605	1.8046	1.6310	-2.3874	0.0000	0.0009
metab_7918	neg	175.0717	0.5991	0.1931	-0.0274	0.5989	0.7045
metab_13585	neg	175.0758	2.6357	1.0101	-1.2195	0.0013	0.0100
metab_13282	neg	175.0760	3.3304	1.4197	-3.2779	0.0073	0.0318
metab_1637	pos	175.0862	1.5987	0.0609	-0.0563	0.7499	0.8289
metab_8416	neg	175.0969	1.6642	0.7307	1.1316	0.0144	0.0506
metab_8972	neg	175.0969	2.9760	0.4012	0.5507	0.4297	0.5545
metab_4968	pos	175.1114	3.8511	1.4182	1.8681	0.0007	0.0060
metab_1900	pos	175.1151	2.4224	0.4137	0.1342	0.4335	0.5647
metab_1153	pos	175.1185	0.5420	1.8539	4.0089	0.0003	0.0035
metab_5205	pos	175.1186	2.9806	2.7495	6.9644	0.0004	0.0039
metab_8495	neg	175.1221	1.8046	1.9660	-3.3629	0.0001	0.0018
metab_1562	pos	175.1224	1.4005	0.9459	-1.7015	0.0502	0.1144
metab_1248	pos	175.1436	0.5280	1.2147	-1.9395	0.0008	0.0067
metab_4089	pos	175.1477	8.2435	0.2029	-0.2258	0.3089	0.4413
metab_4532	pos	175.1477	5.9949	0.2685	0.1127	0.5597	0.6764
metab_1146	pos	176.0103	0.5983	2.2748	5.3634	0.0000	0.0000
metab_8645	neg	176.0110	2.1311	0.3098	-0.0585	0.5064	0.6258
metab_13403	neg	176.0168	3.0260	0.7016	2.1598	0.1586	0.2725
metab_14794	neg	176.0557	0.6131	0.2104	-0.0946	0.4564	0.5798
metab_5597	pos	176.0702	2.0360	0.8079	0.7940	0.0009	0.0069
metab_5196	pos	176.0702	3.0114	0.1991	-0.3498	0.5638	0.6804

metab_8759	neg	176.0710	2.4010	0.5628	-0.3772	0.0644	0.1415
metab_14225	neg	176.0711	1.5439	0.0386	0.0291	0.7917	0.8508
metab_206	pos	176.0913	0.6123	0.3998	0.1741	0.0712	0.1479
metab_6536	pos	176.0913	0.4693	0.9827	-1.5172	0.0470	0.1091
metab_7899	neg	176.0920	0.5851	0.6418	0.7719	0.0598	0.1339
metab_1140	pos	176.1025	0.6123	1.0489	1.8999	0.0126	0.0421
metab_5910	pos	176.1066	1.4289	0.7229	-0.9760	0.0289	0.0765
metab_2068	pos	176.1066	3.1191	1.0916	1.5818	0.0002	0.0030
metab_6416	pos	176.1218	0.5420	2.1394	4.7183	0.0011	0.0077
metab_6318	pos	176.1275	0.6262	0.1863	-0.0337	0.5240	0.6450
metab_12179	neg	176.1437	7.4927	0.8312	-0.8409	0.0059	0.0273
metab_10658	neg	176.1438	10.4181	0.3740	-0.0506	0.0309	0.0845
metab_11177	neg	176.1438	11.1956	0.2916	0.0264	0.0729	0.1540
metab_10698	neg	176.1438	11.5033	0.2678	0.0732	0.0498	0.1171
metab_11157	neg	176.1438	12.3466	0.4093	-0.0558	0.0093	0.0380
metab_11156	neg	176.1438	12.5252	0.3635	-0.0377	0.0296	0.0818
metab_10712	neg	176.1438	13.7667	0.3476	-0.0078	0.0709	0.1511
metab_6346	pos	176.1639	0.6123	1.2849	2.1026	0.0005	0.0045
metab_15068	neg	176.8362	0.3798	0.3566	-0.0339	0.0394	0.0998
metab_10846	neg	176.8362	14.1744	0.3606	-0.0510	0.0452	0.1099
metab_1343	pos	176.8935	0.6962	0.3377	-0.2730	0.2016	0.3204
metab_6400	pos	176.8936	0.5560	0.3232	-0.2858	0.2246	0.3477
metab_7185	neg	176.9615	14.0771	0.3297	-0.0071	0.0519	0.1206
metab_15051	neg	176.9615	0.4634	0.0819	0.3279	0.7104	0.7928
metab_8227	neg	177.0188	1.3669	0.5334	0.5951	0.3798	0.5096
metab_14795	neg	177.0397	0.6131	0.2717	-0.0584	0.2599	0.3895
metab_1906	pos	177.0477	2.4370	1.0704	-1.2792	0.0014	0.0093
metab_3369	pos	177.0541	15.1520	0.0728	-0.1361	0.6531	0.7538
metab_170	pos	177.0542	0.2713	0.0941	-0.1424	0.6256	0.7316
metab_13738	neg	177.0551	2.3227	0.1148	-0.0452	0.7158	0.7962
metab_13380	neg	177.0551	3.0774	0.7197	-1.0580	0.0432	0.1065
metab_277	pos	177.0654	1.2016	0.9858	1.7021	0.0180	0.0539
metab_6817	neg	177.0761	0.9792	0.8902	1.3242	0.0146	0.0510
metab_5336	pos	177.0906	2.5782	1.4255	2.6229	0.0002	0.0026
metab_9435	neg	177.0913	4.7046	0.5552	0.7794	0.1048	0.2009
metab_4345	pos	177.1270	7.0082	0.3367	-0.3349	0.2492	0.3747
metab_633	pos	177.1270	5.2129	0.1027	-0.2487	0.8339	0.8909
metab_652	pos	177.1270	4.9267	0.1392	-0.4803	0.7717	0.8457
metab_461	pos	177.1270	2.5158	0.8770	1.1387	0.0173	0.0523
metab_790	pos	177.1633	8.6132	0.2608	-0.2957	0.2254	0.3487
metab_4542	pos	177.1633	5.9504	0.1491	0.1626	0.5705	0.6859
metab_4426	pos	177.1633	6.6148	0.7716	-0.8937	0.0003	0.0035
metab_12309	neg	177.8440	7.0162	0.9779	-3.4098	0.1058	0.2025
metab_15060	neg	177.8441	0.4307	0.4232	-0.1129	0.0208	0.0647
metab_10938	neg	177.8441	14.2554	0.4460	-0.1067	0.0096	0.0387
metab_12473	neg	177.8441	6.4073	0.0492	0.3667	0.8993	0.9328
metab_11605	neg	177.8593	9.0391	0.3919	-0.0230	0.0676	0.1461
metab_11113	neg	177.9050	13.9952	0.2432	0.0956	0.3055	0.4368

metab_14984	neg	177.9720	0.5126	0.4787	-0.4388	0.1537	0.2662
metab_13671	neg	178.0139	2.4651	1.6012	5.0918	0.0145	0.0508
metab_5300	pos	178.0495	2.6869	0.3006	0.7169	0.5002	0.6230
metab_13488	neg	178.0504	2.8440	0.5582	0.9938	0.0879	0.1770
metab_1016	pos	178.0859	2.1897	0.4961	0.4286	0.1036	0.1945
metab_6867	neg	178.0866	1.2805	0.1240	0.2166	0.8135	0.8674
metab_13176	neg	178.0868	3.6645	0.2382	0.7183	0.6978	0.7832
metab_332	pos	178.1222	1.4860	0.5448	-0.0928	0.2206	0.3431
metab_12877	neg	178.1232	4.6550	1.0626	-2.0307	0.0427	0.1056
metab_3538	pos	178.1585	13.2800	0.1752	-0.2134	0.3020	0.4334
metab_3168	pos	178.1585	10.2252	0.0997	-0.0498	0.4506	0.5804
metab_3570	pos	178.1586	11.2058	0.0997	-0.1401	0.5768	0.6907
metab_3193	pos	178.1586	10.6685	0.0424	-0.1393	0.9468	0.9665
metab_3676	pos	178.1586	9.9782	0.0431	-0.0887	0.9478	0.9669
metab_4636	pos	178.1586	5.4056	0.8826	-1.3866	0.0033	0.0166
metab_11020	neg	178.8146	14.0610	0.2931	0.0638	0.1706	0.2871
metab_10961	neg	178.8333	14.1744	0.3728	-0.0518	0.0334	0.0892
metab_11229	neg	178.8433	10.3687	1.2092	-1.3603	0.0000	0.0004
metab_10941	neg	178.8834	14.2390	0.4226	-0.1140	0.0232	0.0699
metab_6678	neg	178.9153	0.0197	0.2956	0.0024	0.0945	0.1862
metab_15127	neg	178.9229	0.0197	0.3070	-0.0211	0.1044	0.2003
metab_11021	neg	178.9483	14.0610	0.0958	0.2515	0.7207	0.7999
metab_10826	neg	178.9570	14.0927	0.1770	0.0763	0.4607	0.5833
metab_13046	neg	178.9771	4.0516	0.5098	-0.1451	0.0118	0.0440
metab_15048	neg	178.9771	0.4807	0.2240	0.3297	0.1353	0.2422
metab_7184	neg	178.9771	14.0927	0.2094	0.0609	0.1978	0.3185
metab_919	pos	178.9902	14.0805	0.5367	-0.4183	0.0013	0.0089
metab_3341	pos	178.9996	14.4245	0.5837	-0.5283	0.0147	0.0470
metab_8981	neg	179.0343	2.9931	0.0905	0.2624	0.9489	0.9666
metab_7517	neg	179.0343	1.8682	1.0909	-1.5844	0.0016	0.0113
metab_8190	neg	179.0345	1.3089	1.2772	-2.5270	0.0056	0.0265
metab_6894	neg	179.0376	1.4427	1.5251	-2.8992	0.0021	0.0134
metab_6744	neg	179.0554	0.6131	0.7419	-0.6803	0.0207	0.0646
metab_6069	pos	179.0698	1.1589	0.8674	2.0301	0.1219	0.2196
metab_5948	pos	179.0699	1.3437	1.0814	1.3532	0.0001	0.0018
metab_12988	neg	179.0706	4.2367	0.2798	-0.0027	0.1028	0.1979
metab_13271	neg	179.0707	3.3631	0.8611	-0.8582	0.0022	0.0139
metab_14187	neg	179.0707	1.5736	1.7316	4.2455	0.0000	0.0011
metab_8948	neg	179.0707	2.8768	0.2810	-0.5596	0.4718	0.5943
metab_313	pos	179.0810	1.3865	0.7220	-0.8665	0.0463	0.1077
metab_1501	pos	179.0811	1.2016	0.7777	0.8672	0.0063	0.0257
metab_14556	neg	179.0819	1.1355	0.0741	0.2683	0.9498	0.9672
metab_4513	pos	179.1062	6.1167	0.2395	0.0536	0.6813	0.7764
metab_4702	pos	179.1062	5.0451	1.0391	1.3840	0.0123	0.0414
metab_1846	pos	179.1063	2.2519	1.2553	1.9062	0.0001	0.0015
metab_12598	neg	179.1070	5.9217	0.2882	0.0234	0.0995	0.1932
metab_9202	neg	179.1071	3.7658	1.6218	4.5744	0.0039	0.0206
metab_1485	pos	179.1175	1.1589	0.4578	-0.5562	0.1573	0.2652

metab_5722	pos	179.1175	1.7554	1.0241	2.1275	0.0772	0.1566
metab_5880	pos	179.1175	1.4860	0.1682	2.1904	0.8668	0.9148
metab_4351	pos	179.1426	7.0082	0.3901	-0.3722	0.3168	0.4493
metab_662	pos	179.1426	5.9361	0.4011	-0.1440	0.3840	0.5166
metab_2894	pos	179.1426	8.4504	0.0040	0.1937	0.9919	0.9948
metab_651	pos	179.1426	4.9413	0.0602	-0.2388	0.8889	0.9298
metab_5567	pos	179.1538	2.0828	0.3407	-0.9727	0.4512	0.5808
metab_1751	pos	179.1538	1.9609	0.5554	-1.0081	0.2603	0.3878
metab_5426	pos	179.1539	2.3751	0.1596	-0.6620	0.7320	0.8149
metab_5515	pos	179.1539	2.1746	0.4570	-0.9045	0.3117	0.4443
metab_5839	pos	179.1539	1.5424	0.9779	-1.2986	0.0945	0.1821
metab_4085	pos	179.1789	8.2586	0.1441	-0.2069	0.5439	0.6635
metab_10398	neg	179.8408	9.0713	0.6662	-0.2119	0.0499	0.1173
metab_7777	neg	179.8410	0.4307	0.4057	-0.0929	0.0243	0.0722
metab_7173	neg	179.8411	14.2554	0.4379	-0.1023	0.0118	0.0441
metab_7752	neg	179.9356	0.0382	0.3951	-0.0688	0.0219	0.0671
metab_10932	neg	179.9356	14.3207	0.4453	-0.1045	0.0112	0.0427
metab_6442	pos	179.9902	0.5280	0.8720	-1.0776	0.0005	0.0049
metab_14252	neg	180.0058	1.5297	1.6463	4.1980	0.0026	0.0159
metab_8327	neg	180.0297	1.5297	1.0936	3.2823	0.0279	0.0791
metab_3391	pos	180.0650	15.2974	0.2080	0.0050	0.1567	0.2645
metab_6546	pos	180.0650	0.2713	0.1897	-0.0056	0.1791	0.2928
metab_42	pos	180.0651	1.3865	0.2703	-0.2599	0.2527	0.3788
metab_14696	neg	180.0659	0.8240	0.5536	-0.4012	0.0499	0.1173
metab_14376	neg	180.0854	1.3965	0.0354	0.0531	0.7434	0.8151
metab_3375	pos	180.0862	15.7352	0.3053	0.0493	0.0150	0.0474
metab_1086	pos	180.0873	1.2725	0.3658	-0.6192	0.3154	0.4481
metab_5747	pos	180.1015	1.7128	0.0059	-0.0879	0.9375	0.9611
metab_9361	neg	180.1024	4.3716	0.4458	-0.5449	0.4085	0.5357
metab_8628	neg	180.1024	2.0987	0.4839	-0.5412	0.1544	0.2671
metab_13466	neg	180.1024	2.8768	0.4782	-0.8908	0.3363	0.4694
metab_1071	pos	180.1377	1.3579	1.5805	3.4767	0.0249	0.0687
metab_4662	pos	180.1378	5.3014	1.6070	3.0334	0.0003	0.0032
metab_5698	pos	180.1378	1.7993	2.4619	14.9117	0.0000	0.0000
metab_5059	pos	180.1379	3.5011	0.5415	-0.1683	0.2760	0.4045
metab_1961	pos	180.1379	2.6869	0.0521	-0.6362	0.7976	0.8652
metab_1576	pos	180.1489	1.4289	0.2061	0.4652	0.6410	0.7442
metab_11040	neg	180.8123	14.0438	0.3272	-0.0594	0.0976	0.1908
metab_10958	neg	180.8304	14.1744	0.3681	-0.0426	0.0352	0.0921
metab_10652	neg	180.8414	10.3687	1.2921	-1.7485	0.0001	0.0023
metab_10695	neg	180.8588	11.3257	0.2518	0.0467	0.1135	0.2128
metab_10395	neg	180.8589	9.0557	0.3423	0.0434	0.1351	0.2420
metab_10806	neg	180.8990	14.0771	0.5856	-0.2153	0.0005	0.0057
metab_10861	neg	180.9120	14.1904	0.4383	-0.2034	0.1128	0.2121
metab_15143	neg	180.9183	0.0197	0.2718	0.0272	0.1113	0.2102
metab_10822	neg	180.9184	14.0927	0.4941	-0.1676	0.0059	0.0274
metab_7584	neg	180.9303	0.5571	0.8301	-0.5586	0.0001	0.0026
metab_6339	pos	180.9827	0.6123	1.6409	-2.3510	0.0014	0.0094

metab_926	pos	180.9893	14.0948	0.5629	-0.4570	0.0008	0.0064
metab_14183	neg	181.0059	1.5885	0.3064	0.0594	0.3097	0.4406
metab_8049	neg	181.0060	0.9933	0.1673	0.4101	0.6777	0.7678
metab_14396	neg	181.0137	1.3669	1.1289	1.6616	0.0012	0.0098
metab_8670	neg	181.0137	2.1956	0.9841	2.4052	0.0714	0.1519
metab_3282	pos	181.0265	14.0525	0.3192	-0.2602	0.2199	0.3423
metab_14267	neg	181.0436	1.5155	0.2030	-0.2337	0.9069	0.9381
metab_1203	pos	181.0491	0.4543	0.0455	-0.0218	0.9639	0.9781
metab_1711	pos	181.0491	1.7993	1.6422	2.7300	0.0009	0.0069
metab_7481	neg	181.0504	1.5155	1.1340	-1.4723	0.0015	0.0108
metab_3503	pos	181.0643	14.0525	0.3107	-0.2650	0.0420	0.1004
metab_14864	neg	181.0645	0.5711	0.5171	-0.2938	0.1408	0.2497
metab_6588	neg	181.0710	0.5711	0.4194	-0.1080	0.2804	0.4110
metab_6068	pos	181.0967	1.1731	0.8726	0.9413	0.0008	0.0064
metab_8453	neg	181.0972	1.7264	0.1030	-0.3770	0.7899	0.8493
metab_8169	neg	181.0974	1.2805	0.0976	0.9287	0.9214	0.9486
metab_8332	neg	181.1149	1.5297	1.3626	-2.2672	0.0010	0.0088
metab_4459	pos	181.1218	6.4035	0.7754	1.3604	0.0582	0.1271
metab_5272	pos	181.1219	2.7959	0.9226	1.2681	0.0047	0.0210
metab_2122	pos	181.1219	3.3183	1.0534	1.2476	0.0011	0.0079
metab_5670	pos	181.1219	1.8871	1.2791	2.0172	0.0001	0.0015
metab_9316	neg	181.1225	4.1699	0.2740	1.3330	0.7163	0.7965
metab_12461	neg	181.1225	6.4709	1.1202	1.7550	0.0000	0.0013
metab_12936	neg	181.1225	4.4386	0.0803	-0.4028	0.8454	0.8927
metab_1109	pos	181.1331	0.9620	0.7865	-1.1159	0.0590	0.1284
metab_8555	neg	181.1356	1.9280	0.0973	0.2449	0.8275	0.8788
metab_4083	pos	181.1582	8.2728	0.1648	-0.1838	0.6386	0.7424
metab_4892	pos	181.1583	4.1994	0.7527	1.0101	0.0316	0.0816
metab_7779	neg	181.8375	0.4307	0.3259	-0.0479	0.0992	0.1926
metab_10934	neg	181.8379	14.2876	0.3943	-0.0524	0.0177	0.0580
metab_8598	neg	182.0088	2.0202	2.0055	6.4121	0.0003	0.0040
metab_8297	neg	182.0275	1.4710	0.6393	0.9579	0.1387	0.2469
metab_6000	pos	182.0443	1.2446	1.5181	3.4897	0.0014	0.0095
metab_6885	neg	182.0453	1.3669	0.2979	-0.4628	0.3824	0.5120
metab_13968	neg	182.0453	1.9125	0.3641	0.3361	0.2730	0.4036
metab_7923	neg	182.0563	0.5991	0.1204	0.0841	0.6719	0.7631
metab_6370	pos	182.0574	0.5983	1.6150	-4.6141	0.0130	0.0430
metab_244	pos	182.0807	0.8221	0.1188	-0.1861	0.6611	0.7595
metab_14321	neg	182.0815	1.4568	0.6437	-0.6346	0.1990	0.3196
metab_13935	neg	182.0815	1.9745	1.0526	2.3568	0.0453	0.1100
metab_1330	pos	182.0917	0.6402	0.0756	0.2462	0.9549	0.9711
metab_1458	pos	182.0918	1.1024	0.8744	1.2085	0.0249	0.0686
metab_3359	pos	182.0920	14.6259	0.4232	0.1456	0.0053	0.0229
metab_166	pos	182.0920	0.1218	0.4934	0.3899	0.0511	0.1158
metab_4704	pos	182.1536	5.0451	1.4690	2.3961	0.0001	0.0014
metab_2853	pos	182.1536	8.2586	0.1522	-0.2118	0.6212	0.7285
metab_11546	neg	182.8562	9.1522	0.2722	0.1295	0.4183	0.5444
metab_10779	neg	182.8983	14.0438	0.4316	-0.0652	0.0107	0.0414

metab_3297	pos	182.9847	14.0805	0.5773	-0.4663	0.0002	0.0028
metab_11190	neg	182.9879	10.8354	0.1000	0.1357	0.5332	0.6490
metab_13284	neg	183.0042	3.3304	0.2429	0.3393	0.4470	0.5705
metab_352	pos	183.0204	1.5847	0.3073	-0.2566	0.2754	0.4039
metab_6765	neg	183.0291	0.6551	0.6198	1.2493	0.2473	0.3743
metab_8486	neg	183.0292	1.7885	1.4588	2.7213	0.0005	0.0058
metab_14636	neg	183.0292	0.9508	0.8746	1.5400	0.0160	0.0544
metab_3384	pos	183.0648	15.7513	0.2931	-0.2286	0.0142	0.0455
metab_6544	pos	183.0648	0.3055	0.0147	-0.1134	0.8432	0.8983
metab_5699	pos	183.0648	1.7993	1.8936	4.5319	0.0000	0.0009
metab_5421	pos	183.0649	2.3908	1.6608	3.6203	0.0003	0.0032
metab_5303	pos	183.0649	2.6869	1.2852	1.9595	0.0011	0.0079
metab_1617	pos	183.0760	1.5424	0.2053	-0.3573	0.6348	0.7395
metab_5	pos	183.0841	0.8361	0.2379	-0.0834	0.4427	0.5733
metab_5491	pos	183.0913	2.2209	0.5726	0.8054	0.1764	0.2895
metab_6498	pos	183.0975	0.4999	1.0590	-1.8188	0.0058	0.0245
metab_363	pos	183.1012	1.6983	1.2009	1.7263	0.0001	0.0018
metab_5632	pos	183.1012	1.9758	1.2530	1.8256	0.0000	0.0007
metab_1937	pos	183.1013	2.5782	1.1596	1.6706	0.0002	0.0026
metab_7570	neg	183.1020	2.2119	1.6493	3.2526	0.0019	0.0129
metab_8315	neg	183.1132	1.5155	0.1941	1.0018	0.6388	0.7364
metab_2594	pos	183.1375	6.4192	0.7979	0.8149	0.0010	0.0074
metab_4643	pos	183.1376	5.3913	0.6946	0.5338	0.0031	0.0160
metab_407	pos	183.1376	2.0828	0.7557	0.5560	0.0008	0.0066
metab_2652	pos	183.1376	6.8886	0.2825	0.0365	0.3257	0.4597
metab_555	pos	183.1376	3.7148	0.9834	1.0740	0.0068	0.0272
metab_5261	pos	183.1376	2.8259	0.9444	1.5298	0.0089	0.0333
metab_9702	neg	183.1384	6.2456	0.0263	0.5960	0.9284	0.9539
metab_12558	neg	183.1384	6.0831	1.0267	2.4220	0.0176	0.0578
metab_6963	neg	183.1384	4.6550	1.4072	2.9074	0.0030	0.0172
metab_10886	neg	183.8764	14.5008	0.5764	-0.2215	0.0107	0.0413
metab_10944	neg	183.8764	14.2070	0.5126	-0.2246	0.0261	0.0756
metab_6725	neg	184.0009	0.5711	0.8280	-0.9999	0.0146	0.0510
metab_3296	pos	184.0025	14.0665	0.4657	-0.5014	0.1305	0.2310
metab_6805	neg	184.0245	0.9086	0.3500	0.7716	0.3940	0.5224
metab_3495	pos	184.0276	14.0525	0.0425	-0.3541	0.9386	0.9616
metab_2035	pos	184.0423	2.9806	0.4267	-0.4667	0.1173	0.2132
metab_1778	pos	184.0423	2.0360	0.1626	0.7446	0.8093	0.8738
metab_13437	neg	184.0431	2.9760	0.0206	0.3607	0.9585	0.9725
metab_7537	neg	184.0431	2.0202	0.0972	0.4703	0.9242	0.9508
metab_6471	pos	184.0452	0.5140	0.3293	0.5125	0.2387	0.3630
metab_6191	pos	184.0601	0.8920	0.5937	0.9393	0.0948	0.1822
metab_14614	neg	184.0608	0.9933	0.3554	0.6912	0.4092	0.5365
metab_3145	pos	184.0728	10.0403	0.1380	-0.0690	0.5165	0.6384
metab_6308	pos	184.0729	0.6402	0.0377	-0.7191	0.9859	0.9915
metab_2743	pos	184.0729	7.4415	1.0687	1.4665	0.0001	0.0020
metab_2808	pos	184.0729	7.9695	1.2399	1.9535	0.0001	0.0011
metab_2683	pos	184.0730	7.0228	1.3383	3.0349	0.0012	0.0086

metab_1767	pos	184.0755	2.0062	1.2627	-3.0775	0.0093	0.0341
metab_1718	pos	184.0964	1.8432	0.9397	-1.2237	0.0001	0.0016
metab_13775	neg	184.0972	2.2595	0.1723	-0.0644	0.5957	0.7021
metab_8857	neg	184.0972	2.6678	0.0872	0.5455	0.9554	0.9707
metab_7650	neg	184.0972	2.8123	0.1874	0.5871	0.7794	0.8428
metab_1406	pos	184.1075	0.9060	0.7573	2.6919	0.0780	0.1578
metab_1496	pos	184.1076	1.1875	0.3774	-0.0366	0.4187	0.5504
metab_3409	pos	184.1328	14.5016	0.0064	-0.1715	0.9657	0.9788
metab_6551	pos	184.1328	0.2224	0.2142	-0.0011	0.1562	0.2638
metab_1092	pos	184.1328	1.2016	0.8101	0.9947	0.0105	0.0372
metab_5956	pos	184.1329	1.3293	1.0197	10.5881	0.0146	0.0466
metab_4840	pos	184.1329	4.4429	0.4661	0.1876	0.1112	0.2050
metab_4960	pos	184.1329	3.8967	0.5229	0.3066	0.0814	0.1629
metab_12874	neg	184.1336	4.6550	0.9520	-1.5171	0.1135	0.2128
metab_1377	pos	184.1440	0.8221	0.0980	0.1868	0.8442	0.8992
metab_4001	pos	184.1691	8.6132	0.4040	-0.3753	0.0433	0.1023
metab_4099	pos	184.1692	8.2143	0.2614	-0.3001	0.2851	0.4154
metab_10887	neg	184.9016	14.5179	0.5624	-0.2240	0.0017	0.0117
metab_7712	neg	184.9016	0.0259	0.3282	-0.0576	0.0688	0.1481
metab_10841	neg	184.9221	14.1424	0.3723	-0.1093	0.1236	0.2260
metab_7796	neg	184.9266	0.4975	0.1980	0.0389	0.4819	0.6039
metab_14674	neg	184.9850	0.8662	1.0495	-1.7138	0.0840	0.1709
metab_938	pos	184.9851	14.0525	0.0145	0.0316	0.8813	0.9243
metab_4408	pos	184.9851	6.7671	0.4589	-0.3936	0.0200	0.0583
metab_14662	neg	185.0020	0.8803	0.5131	0.4025	0.0964	0.1891
metab_14607	neg	185.0085	0.9933	1.0499	2.3660	0.0262	0.0759
metab_8222	neg	185.0086	1.3517	1.6296	3.2759	0.0003	0.0043
metab_11132	neg	185.0271	13.9301	0.3854	-0.0580	0.0320	0.0865
metab_11570	neg	185.0271	9.1200	0.7556	-0.3350	0.0000	0.0004
metab_11749	neg	185.0271	8.7031	0.7639	-0.4501	0.0000	0.0006
metab_6880	neg	185.0450	1.3517	0.3780	0.5645	0.3166	0.4487
metab_6792	neg	185.0561	0.8240	0.1191	0.7414	0.9358	0.9583
metab_4259	pos	185.0569	7.4415	1.6484	3.3236	0.0000	0.0011
metab_1253	pos	185.0683	0.5280	0.5719	-0.5766	0.1749	0.2878
metab_5780	pos	185.0804	1.6553	0.4162	-0.4355	0.2620	0.3898
metab_8597	neg	185.0814	2.0202	0.7774	0.9908	0.0018	0.0121
metab_7661	neg	185.0814	2.8922	0.3000	0.4839	0.3956	0.5236
metab_5462	pos	185.0999	2.2665	0.1334	0.0643	0.8059	0.8715
metab_13980	neg	185.1007	1.8833	0.5190	1.7826	0.4908	0.6122
metab_551	pos	185.1069	2.2361	0.9478	-1.2606	0.0729	0.1505
metab_2515	pos	185.1168	5.7385	1.5217	2.1875	0.0139	0.0452
metab_2590	pos	185.1168	6.4035	0.5965	0.9335	0.1541	0.2612
metab_635	pos	185.1168	5.2573	1.1627	1.5765	0.0044	0.0201
metab_5431	pos	185.1169	2.3600	1.4104	2.3093	0.0000	0.0006
metab_9476	neg	185.1176	4.8865	0.5409	0.5894	0.0855	0.1731
metab_12738	neg	185.1177	5.2419	0.2685	0.4139	0.4819	0.6039
metab_12971	neg	185.1177	4.3037	0.4274	0.6126	0.4827	0.6047
metab_9111	neg	185.1177	3.4470	0.1975	0.1637	0.6845	0.7733

metab_8823	neg	185.1178	2.5592	0.6877	-1.1332	0.1281	0.2323
metab_15047	neg	185.8651	0.4807	0.3825	0.5172	0.0348	0.0913
metab_7182	neg	185.8651	14.3207	0.4756	-0.1366	0.0085	0.0354
metab_15084	neg	185.8651	0.0589	0.2124	0.0258	0.2657	0.3958
metab_11141	neg	185.9105	13.8980	0.1514	0.1071	0.4061	0.5337
metab_11109	neg	185.9255	14.0117	0.4938	-0.1863	0.0141	0.0499
metab_10833	neg	185.9299	14.1255	0.3194	-0.0450	0.1199	0.2213
metab_14759	neg	186.0400	0.6551	0.0719	0.5643	0.9804	0.9864
metab_14722	neg	186.0401	0.7818	0.3555	0.8345	0.4406	0.5645
metab_4608	pos	186.0432	5.6168	0.4171	-0.3721	0.0281	0.0753
metab_3281	pos	186.0432	14.0525	0.2390	-0.1724	0.0424	0.1009
metab_2512	pos	186.0433	5.7086	0.5087	-0.4446	0.0372	0.0913
metab_4562	pos	186.0433	5.8753	0.3739	-0.2862	0.0822	0.1640
metab_2383	pos	186.0434	4.7758	0.4712	-0.3793	0.0346	0.0869
metab_4827	pos	186.0434	4.4881	0.5123	-0.3928	0.0055	0.0235
metab_408	pos	186.0435	2.0360	0.7730	0.7053	0.0027	0.0145
metab_14024	neg	186.0554	1.8205	1.8080	6.8423	0.0046	0.0231
metab_8753	neg	186.0555	2.3855	1.3992	3.0543	0.0076	0.0326
metab_1105	pos	186.0756	0.9900	0.7837	0.7967	0.0031	0.0160
metab_6865	neg	186.0766	1.2805	0.3708	0.5997	0.2421	0.3682
metab_7577	neg	186.0766	2.2595	0.0645	0.0416	0.7669	0.8331
metab_501	pos	186.0910	3.0114	0.7856	-1.0388	0.0240	0.0669
metab_13107	neg	186.1032	3.8333	0.4638	-0.1626	0.0129	0.0470
metab_6355	pos	186.1055	0.6123	1.1119	-1.3243	0.0006	0.0057
metab_3950	pos	186.1055	8.8169	0.8455	-1.0143	0.0009	0.0070
metab_316	pos	186.1119	1.3720	1.0251	1.3193	0.0002	0.0030
metab_431	pos	186.1121	2.2665	0.7420	-0.7857	0.0084	0.0317
metab_14535	neg	186.1129	1.1940	0.6163	-1.1209	0.1488	0.2598
metab_8830	neg	186.1129	2.5893	0.5428	0.7781	0.1879	0.3069
metab_8697	neg	186.1130	2.2595	0.8802	1.1133	0.0068	0.0304
metab_1444	pos	186.1232	1.0601	0.6747	1.5889	0.1457	0.2508
metab_1073	pos	186.1233	1.3437	1.2959	2.3138	0.0026	0.0143
metab_1103	pos	186.1484	1.1589	1.0006	1.2193	0.0037	0.0180
metab_4772	pos	186.1484	4.7458	0.9984	1.2747	0.0221	0.0627
metab_1599	pos	186.1485	1.5004	1.1314	1.3033	0.0058	0.0243
metab_5311	pos	186.1485	2.6561	0.5384	0.3025	0.0033	0.0166
metab_10740	neg	186.8635	13.9796	0.2896	0.0250	0.1096	0.2076
metab_10882	neg	186.8969	14.4520	0.4726	-0.1179	0.0057	0.0270
metab_7719	neg	186.8970	0.0259	0.3151	-0.0130	0.0697	0.1496
metab_15078	neg	186.9332	0.0925	0.1906	0.0345	0.2979	0.4284
metab_13724	neg	186.9797	2.3533	1.4882	6.3008	0.0028	0.0164
metab_6834	neg	187.0242	0.8522	0.3605	0.5765	0.2173	0.3409
metab_8552	neg	187.0242	1.9280	0.6612	0.8585	0.0118	0.0440
metab_13821	neg	187.0508	2.1630	1.4824	3.2812	0.0021	0.0134
metab_14443	neg	187.0606	1.3089	0.5713	0.6517	0.0626	0.1387
metab_1371	pos	187.0709	0.8081	1.4599	-2.4813	0.0001	0.0019
metab_7969	neg	187.0717	0.7678	0.8709	1.8995	0.0572	0.1296
metab_13336	neg	187.0901	3.1964	0.5910	0.5846	0.0984	0.1919

metab_1715	pos	187.0960	1.8277	0.9605	-1.3429	0.0267	0.0722
metab_9047	neg	187.0970	3.1964	0.6179	0.7225	0.0627	0.1388
metab_13430	neg	187.1082	2.9760	0.1830	0.3350	0.6432	0.7399
metab_8302	neg	187.1082	1.4856	0.1306	0.2390	0.7766	0.8405
metab_5136	pos	187.1111	3.2116	0.8317	1.1803	0.0983	0.1870
metab_6548	pos	187.1259	0.2389	0.2714	-0.2098	0.0562	0.1242
metab_1030	pos	187.1324	1.9309	1.8779	3.4666	0.0000	0.0000
metab_9551	neg	187.1334	5.3546	0.2873	0.1048	0.6056	0.7095
metab_9633	neg	187.1334	5.7913	0.4345	1.1649	0.2322	0.3574
metab_12922	neg	187.1334	4.5219	0.0288	0.6769	0.9074	0.9385
metab_6328	pos	187.1436	0.6262	0.3103	-0.2019	0.4320	0.5632
metab_2451	pos	187.1476	5.2717	1.0604	6.2162	0.1119	0.2058
metab_13100	neg	187.8602	3.8506	0.5865	-0.2331	0.1025	0.1975
metab_9180	neg	187.8602	3.6645	0.7298	-0.3399	0.0399	0.1007
metab_8987	neg	187.8603	2.9931	0.8792	-0.4925	0.1014	0.1959
metab_12930	neg	187.8603	4.4722	0.7939	-0.5026	0.0082	0.0346
metab_9458	neg	187.8603	4.8049	0.6424	-0.3601	0.0436	0.1071
metab_11001	neg	187.8603	14.0927	0.4095	-0.0921	0.0181	0.0590
metab_15086	neg	187.8604	0.0589	0.2830	0.0208	0.0936	0.1848
metab_15050	neg	187.8604	0.4807	0.3492	0.4326	0.0315	0.0855
metab_10940	neg	187.8730	14.2390	0.3036	0.0054	0.0917	0.1820
metab_15052	neg	187.8975	0.4634	0.5396	0.6747	0.0556	0.1269
metab_10986	neg	187.9042	14.1090	0.2962	0.0070	0.0916	0.1820
metab_11029	neg	187.9296	14.0610	0.4418	-0.0739	0.0139	0.0493
metab_10752	neg	187.9341	14.0117	0.3847	-0.2231	0.1997	0.3203
metab_15138	neg	187.9412	0.0197	0.1869	0.0649	0.2862	0.4177
metab_7162	neg	187.9412	14.0771	0.3826	-0.0238	0.0214	0.0660
metab_6730	neg	187.9960	0.5571	0.8464	-0.9155	0.0209	0.0650
metab_5840	pos	188.0010	1.5424	0.3205	-0.1602	0.5099	0.6320
metab_6888	neg	188.0276	1.3517	0.4101	-0.1884	0.3138	0.4456
metab_13660	neg	188.0348	2.4970	1.0483	2.2761	0.0396	0.1000
metab_8392	neg	188.0348	1.6028	0.0070	0.6401	0.8178	0.8707
metab_13782	neg	188.0348	2.2437	0.1649	0.6996	0.9031	0.9358
metab_8978	neg	188.0349	2.9760	0.6338	1.1800	0.2276	0.3526
metab_6185	pos	188.0549	0.8920	1.0007	-1.7738	0.0264	0.0717
metab_6786	neg	188.0558	0.8098	0.3873	0.5771	0.2448	0.3713
metab_14448	neg	188.0640	1.2948	1.8234	4.1205	0.0005	0.0053
metab_1055	pos	188.0701	1.5284	0.2418	-0.1230	0.5457	0.6647
metab_6565	pos	188.0701	0.0278	0.5490	0.4818	0.0225	0.0635
metab_13477	neg	188.0711	2.8440	0.9262	-1.2474	0.0142	0.0501
metab_8623	neg	188.0712	2.0670	0.9014	1.2956	0.0669	0.1451
metab_51	pos	188.0911	1.6413	0.0629	-0.2039	0.7706	0.8450
metab_13974	neg	188.0922	1.9125	0.2454	0.5734	0.6182	0.7203
metab_1934	pos	188.1066	2.5782	0.2108	-0.2195	0.4641	0.5914
metab_6061	pos	188.1276	1.1875	0.1333	-0.5596	0.7127	0.8004
metab_8648	neg	188.1286	2.1311	1.5627	3.3188	0.0000	0.0014
metab_1609	pos	188.1388	1.5284	0.2788	-0.0953	0.5387	0.6589
metab_6096	pos	188.1388	1.1164	0.9270	1.5702	0.0634	0.1355

metab_1686	pos	188.1640	1.7271	0.6989	-0.9065	0.0682	0.1433
metab_1156	pos	188.1753	0.5280	0.6485	-0.9925	0.0602	0.1304
metab_11119	neg	188.8605	13.9796	0.3895	-0.0586	0.0496	0.1169
metab_7711	neg	188.8941	0.0259	0.3241	-0.0152	0.0605	0.1351
metab_10921	neg	188.8941	14.4688	0.5008	-0.1332	0.0040	0.0210
metab_15081	neg	188.9012	0.0589	0.6295	0.9922	0.0877	0.1765
metab_15137	neg	188.9121	0.0197	0.3362	-0.0172	0.0525	0.1217
metab_10835	neg	188.9121	14.1255	0.4185	-0.0478	0.0126	0.0463
metab_6581	neg	188.9324	0.0136	0.3929	-0.0488	0.0199	0.0626
metab_3487	pos	188.9780	14.0525	0.0615	0.1434	0.9963	0.9977
metab_11059	neg	189.0075	14.0438	0.3056	0.0082	0.0652	0.1427
metab_6829	neg	189.0398	1.0499	0.5589	-0.3434	0.0371	0.0956
metab_9147	neg	189.0550	3.5641	0.0844	-0.0038	0.6667	0.7598
metab_13628	neg	189.0551	2.5431	0.7740	-1.6010	0.1787	0.2966
metab_5378	pos	189.0655	2.4690	1.6319	5.3559	0.0036	0.0176
metab_7608	neg	189.0763	2.5113	1.2542	-1.7511	0.0006	0.0059
metab_14400	neg	189.0763	1.3669	0.8540	1.2695	0.0063	0.0287
metab_241	pos	189.0865	0.8920	0.9467	1.2303	0.0065	0.0263
metab_13973	neg	189.0956	1.9125	0.2106	0.6372	0.6690	0.7614
metab_305	pos	189.1019	1.3293	0.3559	0.0381	0.3946	0.5273
metab_13320	neg	189.1126	3.2473	0.4666	0.5649	0.2416	0.3678
metab_7511	neg	189.1127	1.8682	0.6560	0.9965	0.0427	0.1056
metab_9246	neg	189.1127	3.8839	1.0186	1.8042	0.0075	0.0325
metab_13232	neg	189.1127	3.5141	0.3359	0.4082	0.3723	0.5036
metab_5874	pos	189.1227	1.5004	0.3659	0.1608	0.3579	0.4923
metab_1131	pos	189.1228	0.6262	0.9021	-1.2719	0.0054	0.0233
metab_6175	pos	189.1228	0.9200	0.3980	0.3772	0.2146	0.3358
metab_1800	pos	189.1230	2.1132	0.4529	-0.4627	0.0567	0.1252
metab_6382	pos	189.1341	0.5843	0.7235	-0.8645	0.0097	0.0353
metab_6448	pos	189.1593	0.5280	0.0191	0.0891	0.9799	0.9882
metab_4652	pos	189.1633	5.3454	0.6738	6.5865	0.3987	0.5320
metab_9800	neg	189.8574	6.7603	0.6872	-0.7122	0.0291	0.0812
metab_9635	neg	189.8574	5.8074	0.5595	-0.2886	0.1253	0.2285
metab_15049	neg	189.8574	0.4807	0.3751	0.4278	0.0191	0.0610
metab_9455	neg	189.8575	4.7881	0.7157	-0.4125	0.0364	0.0943
metab_13341	neg	189.8575	3.1797	0.9026	-0.4295	0.1131	0.2123
metab_11000	neg	189.8578	14.0927	0.4766	-0.1244	0.0051	0.0250
metab_10953	neg	189.8699	14.1904	0.3121	-0.0359	0.0974	0.1906
metab_10786	neg	189.9309	14.0438	0.4022	-0.0935	0.0290	0.0812
metab_12894	neg	190.0326	4.6056	0.1312	0.4018	0.8561	0.9002
metab_6756	neg	190.0351	0.6551	0.6919	0.9625	0.0611	0.1362
metab_14485	neg	190.0432	1.2663	0.7815	-0.6975	0.0208	0.0647
metab_351	pos	190.0494	1.5987	0.2145	0.2581	0.4846	0.6102
metab_1922	pos	190.0495	2.5003	1.0028	1.5436	0.0080	0.0307
metab_14014	neg	190.0502	1.8371	0.1557	-0.4650	0.6580	0.7524
metab_8371	neg	190.0504	1.5736	0.8059	1.3849	0.0515	0.1200
metab_499	pos	190.0859	2.5314	0.5934	-0.7036	0.0663	0.1404
metab_8764	neg	190.0868	2.4010	0.4078	-0.1702	0.1404	0.2493

metab_8452	neg	190.0868	1.7264	0.1917	0.5244	0.7658	0.8321
metab_13331	neg	190.0868	3.2130	0.8531	-0.9880	0.0498	0.1171
metab_7552	neg	190.0868	2.0987	0.2842	-0.0503	0.2373	0.3631
metab_8943	neg	190.0868	2.8606	0.4883	-0.3730	0.1686	0.2849
metab_14751	neg	190.0907	0.6831	0.1569	-0.7010	0.5964	0.7028
metab_1245	pos	190.0970	0.5140	1.8991	11.7344	0.0000	0.0003
metab_273	pos	190.1069	1.0601	0.3573	-0.4473	0.1216	0.2193
metab_1663	pos	190.1222	1.6838	0.5070	0.5695	0.1104	0.2040
metab_4895	pos	190.1222	4.1853	0.3980	3.9649	0.7053	0.7944
metab_5035	pos	190.1223	3.5771	0.8556	-0.9756	0.1302	0.2306
metab_1389	pos	190.1432	0.8500	1.0964	2.2463	0.0243	0.0674
metab_1134	pos	190.1432	0.6123	0.6103	1.0369	0.0998	0.1890
metab_6105	pos	190.1432	1.1024	0.6675	-0.6980	0.0032	0.0162
metab_4718	pos	190.1433	4.9698	0.2572	-0.2504	0.6559	0.7557
metab_5554	pos	190.1434	2.1132	1.1866	2.1476	0.0014	0.0093
metab_1850	pos	190.1586	2.2519	0.5027	-1.1135	0.2859	0.4162
metab_6674	neg	190.9278	0.0197	0.3584	-0.0494	0.0436	0.1071
metab_11036	neg	190.9278	14.0610	0.5664	-0.2107	0.0012	0.0096
metab_9448	neg	190.9480	4.7546	0.7644	-0.5698	0.0283	0.0800
metab_14712	neg	190.9485	0.7818	1.2140	-1.2541	0.0169	0.0562
metab_7952	neg	190.9489	0.6551	1.1668	-2.2460	0.0012	0.0098
metab_8568	neg	190.9570	1.9745	1.3352	3.1191	0.0058	0.0271
metab_8206	neg	191.0017	1.3231	0.3850	2.2767	0.7504	0.8215
metab_14686	neg	191.0120	0.8522	1.2701	-1.3783	0.0024	0.0150
metab_14770	neg	191.0191	0.6411	0.9687	-0.8559	0.0058	0.0271
metab_6779	neg	191.0191	0.7818	0.8740	-0.7350	0.0082	0.0345
metab_3462	pos	191.0267	14.0948	0.4796	-0.3583	0.0356	0.0886
metab_14006	neg	191.0343	1.8524	0.0851	0.0338	0.6055	0.7095
metab_7557	neg	191.0344	2.1153	0.1320	0.1972	0.8537	0.8985
metab_9074	neg	191.0554	3.2801	0.3370	0.4097	0.2094	0.3319
metab_9481	neg	191.0555	4.9183	0.3898	0.4575	0.1071	0.2042
metab_12504	neg	191.0555	6.3269	0.4040	0.4981	0.0782	0.1626
metab_5612	pos	191.0698	2.0062	1.4804	2.4578	0.0000	0.0007
metab_9150	neg	191.0707	3.5641	0.4076	-1.7670	0.5442	0.6574
metab_13443	neg	191.0708	2.9429	0.3387	-0.9147	0.3881	0.5170
metab_1483	pos	191.0811	1.1589	0.6586	1.4205	0.1905	0.3058
metab_1785	pos	191.0811	2.0677	1.4074	2.0440	0.0000	0.0000
metab_14411	neg	191.0821	1.3373	0.3933	0.2276	0.4156	0.5419
metab_14489	neg	191.0919	1.2663	1.0166	1.4426	0.0003	0.0042
metab_1130	pos	191.1021	0.6262	0.7400	-0.8120	0.0003	0.0036
metab_1607	pos	191.1173	1.5144	0.1376	-0.3060	0.7047	0.7940
metab_5386	pos	191.1426	2.4530	1.7119	4.3660	0.0017	0.0106
metab_9121	neg	191.1435	3.4800	0.2106	0.3228	0.5316	0.6478
metab_12860	neg	191.1436	4.7217	0.2702	0.2145	0.6511	0.7465
metab_5812	pos	191.1537	1.5987	1.3953	-1.5370	0.0183	0.0545
metab_791	pos	191.1789	8.6132	0.0821	-0.2170	0.7529	0.8307
metab_15082	neg	191.8545	0.0589	0.3795	-0.0515	0.0323	0.0870
metab_11152	neg	191.8575	13.4046	0.3676	-0.0417	0.0447	0.1091

metab_11148	neg	191.8576	13.7830	0.3006	-0.0356	0.1405	0.2493
metab_10736	neg	191.8576	13.9796	0.2381	0.0512	0.2586	0.3878
metab_7730	neg	191.9314	0.0197	0.3844	-0.0913	0.0358	0.0931
metab_9447	neg	191.9456	4.7546	0.4445	-0.0447	0.0377	0.0968
metab_7186	neg	191.9457	14.0610	0.2204	0.0516	0.1948	0.3150
metab_8398	neg	192.0496	1.6186	0.4812	-0.6639	0.2000	0.3207
metab_5892	pos	192.0650	1.4569	1.0691	-2.3104	0.0123	0.0414
metab_6092	pos	192.0650	1.1306	0.0082	-0.0375	0.9121	0.9430
metab_13718	neg	192.0661	2.3855	0.6658	-0.6656	0.0651	0.1427
metab_44	pos	192.1013	1.3720	0.1233	-0.2305	0.5657	0.6820
metab_1967	pos	192.1015	2.7188	0.6760	-0.5029	0.0546	0.1217
metab_6181	pos	192.1015	0.9060	0.5610	0.4519	0.0356	0.0886
metab_14119	neg	192.1023	1.6797	0.7150	-0.8765	0.2220	0.3461
metab_12776	neg	192.1024	5.0484	0.3606	-1.2367	0.6175	0.7199
metab_13248	neg	192.1024	3.4639	0.5236	1.2581	0.4312	0.5560
metab_13549	neg	192.1024	2.7155	0.8092	-0.2882	0.0353	0.0922
metab_13709	neg	192.1024	2.3855	0.1854	0.3021	0.7012	0.7858
metab_14371	neg	192.1025	1.4115	0.7755	-1.0349	0.0927	0.1837
metab_5097	pos	192.1095	3.3337	1.4664	3.6640	0.0140	0.0452
metab_6071	pos	192.1127	1.1589	0.0864	1.0897	0.9469	0.9665
metab_5493	pos	192.1743	2.2045	0.8125	-2.5899	0.1855	0.3003
metab_6642	neg	192.9283	14.0438	0.3749	-0.0294	0.0260	0.0755
metab_15093	neg	192.9284	0.0382	0.0532	0.1241	0.8793	0.9177
metab_8571	neg	192.9540	1.9745	0.7573	3.6336	0.0316	0.0857
metab_7696	neg	192.9583	0.4975	0.4783	-0.1550	0.0238	0.0710
metab_8363	neg	193.0138	1.5586	1.6243	5.2356	0.0007	0.0066
metab_13871	neg	193.0138	2.0670	1.6302	3.7346	0.0002	0.0035
metab_8121	neg	193.0171	1.1940	1.8559	-3.8163	0.0000	0.0012
metab_6853	neg	193.0347	0.5991	0.1864	0.2904	0.6191	0.7209
metab_13483	neg	193.0501	2.8440	0.0534	0.2578	0.9410	0.9617
metab_6030	pos	193.0605	1.2016	1.0226	3.0036	0.0208	0.0599
metab_14292	neg	193.0614	1.4856	0.7589	1.2530	0.0760	0.1588
metab_1816	pos	193.0686	2.1444	1.4003	9.5876	0.0055	0.0234
metab_5244	pos	193.0856	2.8725	1.6213	3.4697	0.0001	0.0013
metab_6978	neg	193.0865	5.1456	0.2348	0.3176	0.2947	0.4254
metab_13215	neg	193.0865	3.5641	0.3445	2.7815	0.7250	0.8023
metab_1029	pos	193.0968	1.9758	0.5635	0.6788	0.1024	0.1930
metab_9083	neg	193.0978	3.3144	0.2154	0.2294	0.6171	0.7196
metab_12786	neg	193.0993	5.0002	0.2681	0.0062	0.1976	0.3182
metab_2607	pos	193.1219	6.6001	0.7018	0.6488	0.0589	0.1284
metab_5522	pos	193.1321	2.1595	1.2389	-1.8499	0.0035	0.0172
metab_1539	pos	193.1330	1.3153	0.8679	-1.9983	0.1261	0.2250
metab_5598	pos	193.1331	2.0211	0.7111	-0.9568	0.0495	0.1133
metab_2550	pos	193.1583	5.9949	0.2047	1.2750	0.6223	0.7293
metab_145	pos	193.1583	4.8961	0.0387	-0.0889	0.5920	0.7038
metab_4476	pos	193.1583	6.3885	1.2114	1.8864	0.0001	0.0020
metab_1674	pos	193.1695	1.6983	1.0323	-1.7411	0.0181	0.0540
metab_5662	pos	193.1696	1.9169	0.8757	-1.1137	0.1132	0.2074

metab_5187	pos	193.1696	3.0422	0.8828	-0.8530	0.1533	0.2603
metab_1940	pos	193.1696	2.5938	0.4152	-0.1219	0.4941	0.6185
metab_5394	pos	193.1696	2.4370	0.5042	-0.7002	0.3507	0.4857
metab_5537	pos	193.1696	2.1444	1.5187	-2.8050	0.0012	0.0086
metab_15061	neg	193.8150	0.4307	0.3832	-0.0705	0.0385	0.0983
metab_10901	neg	193.8150	15.9795	0.4165	-0.0751	0.0156	0.0535
metab_12430	neg	193.8150	6.5989	0.5755	-0.2528	0.0674	0.1460
metab_11619	neg	193.8151	8.9903	0.5143	-0.1345	0.1261	0.2297
metab_10737	neg	193.8538	13.9796	0.1288	0.1284	0.6709	0.7624
metab_7738	neg	193.9102	0.0197	0.1689	0.0018	0.3900	0.5185
metab_11041	neg	193.9316	14.0438	0.3052	-0.1375	0.1326	0.2385
metab_10974	neg	193.9516	14.1424	0.2401	0.0600	0.1657	0.2814
metab_7582	neg	194.0276	2.3227	0.2581	0.1863	0.5169	0.6351
metab_14072	neg	194.0454	1.7419	0.0984	0.5639	0.9659	0.9763
metab_6192	pos	194.0807	0.8920	0.9271	1.0191	0.0002	0.0030
metab_389	pos	194.0808	1.8871	0.7485	0.6095	0.0030	0.0157
metab_12588	neg	194.0817	5.9542	0.2787	0.0041	0.1414	0.2503
metab_6147	pos	194.1032	0.9900	0.7119	0.3401	0.2129	0.3339
metab_5876	pos	194.1170	1.5004	1.0144	1.2440	0.0004	0.0045
metab_523	pos	194.1172	2.4071	0.1517	-0.3870	0.5471	0.6658
metab_9305	neg	194.1180	4.1023	0.7654	-2.2829	0.1676	0.2837
metab_13516	neg	194.1181	2.7792	0.8246	-1.3723	0.1725	0.2890
metab_9226	neg	194.1181	3.8164	0.9499	-1.8514	0.0819	0.1680
metab_5488	pos	194.1536	2.2209	1.0587	1.1457	0.0004	0.0039
metab_357	pos	194.1648	1.6128	1.3578	-1.9204	0.0168	0.0514
metab_11395	neg	194.9053	9.6814	0.6075	-0.2233	0.0178	0.0584
metab_7098	neg	194.9053	8.7191	0.7683	-0.4825	0.0003	0.0039
metab_11564	neg	194.9054	9.1200	0.5708	-0.1209	0.0063	0.0287
metab_7189	neg	194.9238	14.0438	0.3316	0.0049	0.0433	0.1066
metab_14802	neg	194.9778	0.5991	1.1997	-1.7789	0.0116	0.0435
metab_14259	neg	195.0293	1.5297	1.7665	4.4031	0.0003	0.0042
metab_3470	pos	195.0413	14.0805	0.6186	-0.5336	0.0002	0.0027
metab_7532	neg	195.0504	0.5991	0.7792	-0.4885	0.0014	0.0105
metab_11705	neg	195.0505	8.7191	1.0365	-1.0483	0.0003	0.0036
metab_5381	pos	195.0649	2.4690	1.1130	3.0145	0.0315	0.0814
metab_423	pos	195.0649	2.1897	0.2594	-0.1041	0.4689	0.5962
metab_7502	neg	195.0657	1.8046	0.4539	0.6339	0.1702	0.2866
metab_13252	neg	195.0658	3.4470	0.3691	-0.1865	0.1135	0.2128
metab_14275	neg	195.0771	1.5155	0.2162	0.5548	0.7040	0.7881
metab_14784	neg	195.0867	0.6131	1.0913	-1.1835	0.0115	0.0433
metab_14186	neg	195.0958	1.5736	0.8814	3.7361	0.0102	0.0402
metab_2510	pos	195.1011	5.6934	0.7769	1.9356	0.1362	0.2386
metab_5360	pos	195.1012	2.5158	1.3537	2.2363	0.0002	0.0027
metab_5508	pos	195.1013	2.1897	1.3504	2.3246	0.0004	0.0039
metab_12741	neg	195.1020	5.2257	0.1826	-0.0604	0.4255	0.5511
metab_9603	neg	195.1021	5.6615	1.0089	-1.0394	0.0098	0.0392
metab_12972	neg	195.1021	4.3037	0.0559	0.0497	0.7821	0.8446
metab_14107	neg	195.1021	1.6948	0.5226	1.9565	0.2239	0.3481

metab_6116	pos	195.1124	1.0741	0.8682	1.0149	0.0037	0.0178
metab_13701	neg	195.1134	2.4010	0.8167	-1.3874	0.1577	0.2714
metab_3376	pos	195.1223	15.7835	0.3367	-0.2539	0.0176	0.0531
metab_6560	pos	195.1223	0.0552	0.3544	-0.2684	0.0151	0.0477
metab_4490	pos	195.1375	6.2824	0.5091	-0.9820	0.1794	0.2931
metab_4738	pos	195.1375	4.9267	0.2087	-0.4221	0.6868	0.7805
metab_9867	neg	195.1385	7.0322	1.1133	-1.5198	0.0355	0.0926
metab_12797	neg	195.1385	4.9510	0.7804	-1.1941	0.2396	0.3656
metab_9581	neg	195.1385	5.5639	0.1235	0.0997	0.5187	0.6368
metab_5992	pos	195.1488	1.2585	1.5174	-2.0543	0.0155	0.0484
metab_10811	neg	195.8547	14.0771	0.0695	0.1197	0.7248	0.8022
metab_7723	neg	195.9054	0.0197	0.3149	-0.0185	0.0718	0.1524
metab_7175	neg	195.9055	14.4520	0.5419	-0.1949	0.0029	0.0167
metab_10869	neg	195.9591	14.2554	0.2346	0.0228	0.3371	0.4702
metab_1282	pos	195.9768	0.5703	0.5888	-0.7915	0.1101	0.2035
metab_13904	neg	196.0068	2.0202	1.3432	3.5988	0.0137	0.0489
metab_3315	pos	196.0163	14.1389	0.5777	-0.4816	0.0035	0.0172
metab_11294	neg	196.0246	10.0432	0.7527	0.7146	0.0781	0.1625
metab_8215	neg	196.0247	1.3373	0.4770	0.7584	0.1523	0.2643
metab_6399	pos	196.0478	0.5560	1.5863	5.3975	0.0001	0.0011
metab_1589	pos	196.0600	1.4569	0.6131	-0.7996	0.0681	0.1431
metab_7465	neg	196.0611	1.6028	0.2715	-0.1449	0.4232	0.5490
metab_6210	pos	196.0963	0.8640	1.9455	4.1230	0.0000	0.0000
metab_13603	neg	196.0974	2.6046	0.1484	0.1503	0.8423	0.8906
metab_7671	neg	196.0975	2.9931	0.5678	-0.8035	0.1092	0.2071
metab_5191	pos	196.1078	3.0114	0.1463	0.5122	0.7436	0.8241
metab_5746	pos	196.1116	1.7128	1.0022	-1.2949	0.0007	0.0060
metab_1459	pos	196.1327	1.1024	2.1641	5.4742	0.0000	0.0000
metab_458	pos	196.1329	2.4850	0.5543	-1.2449	0.1731	0.2854
metab_5004	pos	196.1329	3.7148	0.5034	-1.0043	0.1629	0.2725
metab_5355	pos	196.1409	2.5158	1.1976	8.7095	0.0157	0.0488
metab_4078	pos	196.1692	8.2872	0.1287	-0.2360	0.5963	0.7069
metab_15149	neg	196.8844	0.0136	0.4863	-0.2122	0.0133	0.0479
metab_10935	neg	196.8844	14.2720	0.6298	-0.2952	0.0017	0.0118
metab_10894	neg	196.9202	14.8990	0.2666	0.0255	0.1471	0.2580
metab_7773	neg	196.9202	0.3798	0.1475	0.0914	0.4412	0.5651
metab_10883	neg	196.9203	14.4688	0.0186	0.1716	0.9239	0.9508
metab_7764	neg	196.9404	0.0753	0.2976	0.0102	0.0841	0.1710
metab_10915	neg	196.9404	14.5349	0.3457	-0.0254	0.0538	0.1238
metab_11111	neg	196.9595	13.9952	0.4416	0.0062	0.0802	0.1656
metab_6849	neg	197.0087	1.2236	0.1817	-0.0022	0.5981	0.7038
metab_937	pos	197.0215	14.0525	0.1795	-0.1547	0.3600	0.4938
metab_3474	pos	197.0367	14.0805	0.5559	-0.4494	0.0004	0.0041
metab_4409	pos	197.0367	6.7671	0.1716	-0.0477	0.3623	0.4962
metab_6804	neg	197.0450	0.8946	0.2097	0.1819	0.4397	0.5637
metab_8906	neg	197.0451	2.7792	1.2859	1.9938	0.0002	0.0034
metab_8774	neg	197.0452	2.4338	0.0633	0.0860	0.7792	0.8426
metab_13608	neg	197.0656	2.5893	1.1344	-2.8520	0.0555	0.1267

metab_8757	neg	197.0661	2.3855	1.4953	-4.0327	0.0167	0.0558
metab_934	pos	197.0804	14.3928	0.2637	0.0195	0.0500	0.1141
metab_1192	pos	197.0804	0.2224	0.1314	-0.0489	0.3179	0.4505
metab_1851	pos	197.0805	2.2519	0.7990	0.8342	0.0019	0.0115
metab_5757	pos	197.0806	1.6838	1.2245	2.9722	0.0063	0.0257
metab_9248	neg	197.0814	3.9005	0.2613	0.1140	0.2975	0.4280
metab_13898	neg	197.0814	2.0358	1.2037	1.9269	0.0004	0.0049
metab_149	pos	197.0915	0.6262	0.7350	0.9616	0.0440	0.1035
metab_6129	pos	197.0917	1.0461	0.8966	1.1406	0.0014	0.0093
metab_1719	pos	197.0918	1.8432	0.2923	0.1242	0.5633	0.6800
metab_998	pos	197.1168	6.2978	0.2686	0.0956	0.5736	0.6885
metab_5363	pos	197.1169	2.5003	1.4063	2.3087	0.0000	0.0007
metab_1847	pos	197.1169	2.2519	1.3412	2.2257	0.0001	0.0020
metab_12698	neg	197.1178	5.4844	0.6884	1.0016	0.0587	0.1321
metab_8782	neg	197.1178	2.4487	0.6413	0.7994	0.0708	0.1510
metab_9219	neg	197.1178	3.8001	0.3114	0.3591	0.4614	0.5838
metab_5963	pos	197.1279	1.3293	0.8878	0.9195	0.0006	0.0057
metab_1027	pos	197.1281	2.0524	0.0811	0.1270	0.9509	0.9683
metab_13920	neg	197.1290	2.0055	0.0521	0.9111	0.9543	0.9702
metab_13827	neg	197.1290	2.1630	0.0088	0.6801	0.8678	0.9092
metab_2305	pos	197.1532	4.3371	0.8598	0.6530	0.0240	0.0668
metab_11002	neg	197.8517	14.0771	0.2242	0.3175	0.3125	0.4440
metab_7791	neg	197.8517	0.4975	0.8616	-0.8925	0.0021	0.0136
metab_10943	neg	197.9019	14.2390	0.3895	-0.0574	0.0267	0.0769
metab_7789	neg	197.9020	0.4807	0.3275	-0.0194	0.1270	0.2310
metab_10924	neg	197.9395	14.4185	0.3397	-0.0264	0.0605	0.1350
metab_10711	neg	197.9627	13.7337	0.2067	0.0585	0.2741	0.4046
metab_7456	neg	198.0323	1.5736	0.3297	-0.0218	0.1130	0.2123
metab_13668	neg	198.0323	2.4651	0.1573	0.1197	0.3881	0.5170
metab_14766	neg	198.0402	0.6411	0.5113	-1.4720	0.2773	0.4079
metab_14413	neg	198.0403	1.3373	0.1933	-0.1347	0.4763	0.5982
metab_6381	pos	198.0522	0.5843	0.9449	-1.1366	0.0001	0.0016
metab_5700	pos	198.0756	1.7993	0.0920	-0.1087	0.8207	0.8824
metab_14390	neg	198.0767	1.3817	0.4102	0.7599	0.3592	0.4919
metab_3293	pos	198.0797	14.0665	0.0924	-0.1535	0.6046	0.7145
metab_5531	pos	198.0910	2.1444	0.1326	-0.1096	0.6822	0.7768
metab_12993	neg	198.0920	4.2203	1.1050	-1.5749	0.0579	0.1309
metab_8947	neg	198.0921	2.8768	1.7654	-3.3901	0.0008	0.0072
metab_5833	pos	198.1120	1.5565	0.5518	0.5258	0.0218	0.0622
metab_5390	pos	198.1121	2.4530	0.9257	-1.2722	0.0002	0.0024
metab_1779	pos	198.1121	2.0524	0.1695	-0.1425	0.5139	0.6356
metab_13947	neg	198.1131	1.9596	0.0774	0.3909	0.9394	0.9606
metab_7689	neg	198.1132	3.1797	0.2653	-0.3575	0.4421	0.5660
metab_6447	pos	198.1232	0.5280	0.0996	0.0304	0.6261	0.7320
metab_4699	pos	198.1484	5.0610	0.7991	2.7431	0.2358	0.3597
metab_2337	pos	198.1484	4.5027	1.3211	2.7715	0.0170	0.0517
metab_2166	pos	198.1485	3.5311	0.8507	0.7271	0.0098	0.0354
metab_2064	pos	198.1485	3.1032	0.2259	0.1128	0.6339	0.7387

metab_6984	neg	198.1494	5.1939	0.8155	-1.4123	0.2256	0.3502
metab_6196	pos	198.1596	0.8780	0.5356	-0.5275	0.1638	0.2737
metab_2915	pos	198.1847	8.6132	0.4101	-0.3870	0.0494	0.1132
metab_603	pos	198.1848	4.6096	0.0501	-0.1162	0.8983	0.9345
metab_4100	pos	198.1848	8.2143	0.2911	-0.2788	0.2085	0.3283
metab_593	pos	198.1848	4.1994	0.1490	-0.1653	0.5280	0.6486
metab_5170	pos	198.1848	3.1032	0.2356	-0.6150	0.5235	0.6450
metab_5120	pos	198.1849	3.2573	0.0132	-0.3606	0.9728	0.9838
metab_10791	neg	198.8027	14.0438	0.7406	-0.3043	0.2175	0.3410
metab_11145	neg	198.8639	13.8818	0.3384	-0.0104	0.0404	0.1016
metab_10577	neg	198.8639	9.8947	0.5473	-0.1155	0.0091	0.0374
metab_10630	neg	198.8639	10.2225	0.5730	-0.2868	0.0275	0.0783
metab_11783	neg	198.8639	8.6239	0.7764	1.0793	0.0576	0.1302
metab_10884	neg	198.9358	14.5008	0.3302	-0.0073	0.0590	0.1325
metab_15079	neg	198.9358	0.0925	0.2914	0.0084	0.0990	0.1925
metab_8679	neg	198.9360	2.2119	0.3998	-0.0500	0.1122	0.2115
metab_12923	neg	198.9361	4.5054	0.5291	-0.1426	0.0162	0.0547
metab_14312	neg	198.9880	1.4710	0.7523	-0.5732	0.0183	0.0593
metab_3497	pos	199.0168	14.0525	0.3721	-0.2799	0.0983	0.1870
metab_6875	neg	199.0244	1.2948	1.1208	1.6941	0.0006	0.0059
metab_14205	neg	199.0608	1.5736	1.3071	2.4479	0.0005	0.0054
metab_14661	neg	199.0719	0.8946	0.6860	1.6513	0.1522	0.2642
metab_1204	pos	199.0760	0.4543	0.7100	0.9993	0.0458	0.1069
metab_13719	neg	199.0872	2.3704	1.6532	-7.5530	0.0224	0.0681
metab_5301	pos	199.0961	2.6869	0.5156	0.2944	0.0082	0.0312
metab_13201	neg	199.0971	3.5978	1.1407	-1.8132	0.0189	0.0607
metab_6076	pos	199.1073	1.1589	0.5938	-0.9983	0.0741	0.1520
metab_2037	pos	199.1074	2.9806	0.7555	-1.0397	0.0052	0.0227
metab_8736	neg	199.1084	2.3380	0.1197	-0.4666	0.7980	0.8554
metab_7512	neg	199.1084	1.8682	0.1046	1.2542	0.9448	0.9638
metab_6915	neg	199.1085	1.5012	0.6948	1.4667	0.1863	0.3050
metab_12747	neg	199.1334	5.2100	0.3055	-0.0787	0.5811	0.6895
metab_12876	neg	199.1335	4.6550	0.4198	-0.1550	0.0424	0.1052
metab_13323	neg	199.1335	3.2301	1.0917	1.6832	0.0225	0.0682
metab_13394	neg	199.1335	3.0432	0.1484	0.7404	0.8637	0.9065
metab_13151	neg	199.1336	3.7317	0.4976	0.4053	0.4392	0.5633
metab_1529	pos	199.1436	1.2585	1.3708	2.0966	0.0003	0.0035
metab_1872	pos	199.1437	2.3292	0.4321	-0.3944	0.4979	0.6211
metab_2693	pos	199.1476	7.0670	0.4941	-0.5845	0.2460	0.3707
metab_10995	neg	199.9163	14.0927	0.4050	-0.0599	0.0121	0.0448
metab_3305	pos	199.9748	14.1090	0.5253	-0.4212	0.0022	0.0127
metab_8364	neg	200.0293	1.5736	0.4101	-0.0941	0.0642	0.1412
metab_1053	pos	200.0468	1.5705	0.3474	-0.3091	0.0872	0.1715
metab_14725	neg	200.0559	0.7818	0.6181	-0.4592	0.0401	0.1011
metab_1145	pos	200.0678	0.5843	0.4275	0.2593	0.0495	0.1132
metab_522	pos	200.0702	2.4071	0.3573	-0.3923	0.3208	0.4538
metab_7594	neg	200.0713	2.4010	0.3480	-0.0849	0.1689	0.2851
metab_13839	neg	200.0713	2.1311	0.5131	-0.3209	0.0577	0.1305

metab_13024	neg	200.0713	4.1193	0.9599	-1.1291	0.0271	0.0776
metab_1556	pos	200.0912	1.3865	0.7815	1.1329	0.0284	0.0757
metab_1042	pos	200.0912	1.6693	0.8055	-0.8841	0.0075	0.0294
metab_14256	neg	200.0924	1.5297	0.7002	1.1171	0.0470	0.1128
metab_3498	pos	200.0953	14.0525	0.0728	-0.0829	0.7150	0.8020
metab_14074	neg	200.1005	1.7419	1.3227	5.1561	0.0089	0.0367
metab_6230	pos	200.1025	0.8221	0.2131	0.2025	0.6279	0.7334
metab_5845	pos	200.1063	1.5284	1.3180	-2.1504	0.0007	0.0060
metab_5641	pos	200.1065	1.9609	0.6096	0.5051	0.0155	0.0485
metab_5490	pos	200.1066	2.2209	0.8276	-1.2207	0.0201	0.0586
metab_9324	neg	200.1187	4.1869	0.6018	-0.3428	0.0030	0.0172
metab_6367	pos	200.1211	0.5983	0.5457	0.5244	0.1553	0.2627
metab_5732	pos	200.1276	1.7412	0.6328	0.7519	0.0265	0.0719
metab_5651	pos	200.1277	1.9463	0.2813	0.1457	0.2687	0.3963
metab_9075	neg	200.1287	3.2972	0.1622	0.6325	0.7973	0.8548
metab_4999	pos	200.1641	3.7297	0.3288	-0.0335	0.3464	0.4813
metab_5271	pos	200.1641	2.7959	0.0870	-0.2711	0.7470	0.8271
metab_1772	pos	200.1642	2.0211	0.6319	0.0385	0.2129	0.3339
metab_1292	pos	200.1753	0.5843	0.6548	-0.4443	0.1517	0.2583
metab_4391	pos	200.2004	6.8728	0.9266	-1.1148	0.0000	0.0005
metab_10715	neg	200.8583	13.8818	0.1824	0.0737	0.3074	0.4386
metab_10825	neg	200.9128	14.0927	0.5959	-0.1987	0.0079	0.0338
metab_3319	pos	200.9953	14.1820	0.4756	-0.3712	0.0045	0.0205
metab_8995	neg	200.9956	3.0260	1.2031	-1.3104	0.0014	0.0106
metab_8854	neg	200.9956	2.6524	1.6988	5.7060	0.0064	0.0290
metab_3446	pos	201.0078	14.1820	0.4575	-0.3450	0.0021	0.0125
metab_6559	pos	201.0078	0.0720	0.5541	-0.4591	0.0005	0.0048
metab_7862	neg	201.0164	0.5431	1.1635	-1.6238	0.0201	0.0633
metab_8040	neg	201.0221	0.9649	0.4852	3.0875	0.2288	0.3538
metab_8546	neg	201.0319	1.9125	1.0145	-7.8528	0.0082	0.0346
metab_14428	neg	201.0400	1.3231	0.0451	0.1626	0.6960	0.7819
metab_8529	neg	201.0666	1.8682	0.6941	0.9864	0.1139	0.2129
metab_30	pos	201.0716	0.5140	0.1143	0.1662	0.6752	0.7715
metab_14338	neg	201.0764	1.4568	1.1337	1.8937	0.0007	0.0065
metab_6188	pos	201.0865	0.8920	0.8744	1.4689	0.0302	0.0789
metab_14817	neg	201.0875	0.5991	0.3009	0.8606	0.5958	0.7022
metab_14441	neg	201.0876	1.3089	0.7689	1.1017	0.0157	0.0537
metab_8792	neg	201.0877	2.4651	0.3483	0.6704	0.6148	0.7177
metab_5322	pos	201.1018	2.6090	0.1056	0.1703	0.8244	0.8843
metab_1713	pos	201.1117	1.7993	2.0053	4.8403	0.0000	0.0001
metab_12487	neg	201.1127	6.4073	0.3582	0.8837	0.2951	0.4256
metab_14029	neg	201.1127	1.8205	1.0114	1.9908	0.0060	0.0279
metab_6935	neg	201.1128	3.8506	0.4107	-0.4210	0.1870	0.3059
metab_14298	neg	201.1240	1.4856	0.6313	0.7982	0.1172	0.2173
metab_3424	pos	201.1309	14.3777	0.1515	-0.1826	0.2838	0.4139
metab_1775	pos	201.1481	2.0360	1.8162	3.2616	0.0000	0.0001
metab_1606	pos	201.1594	1.5144	0.7966	1.7015	0.0828	0.1650
metab_10723	neg	201.9055	13.8980	0.2737	0.0337	0.1369	0.2445

metab_7163	neg	201.9250	14.0927	0.5061	-0.1448	0.0031	0.0176
metab_3303	pos	202.0155	14.0948	0.5603	-0.4531	0.0007	0.0058
metab_8846	neg	202.0311	2.6206	0.4876	-0.0076	0.3431	0.4763
metab_6755	neg	202.0352	0.6271	0.7526	-0.6205	0.0281	0.0794
metab_1052	pos	202.0438	1.5847	0.3442	-0.3076	0.0906	0.1765
metab_7714	neg	202.0505	3.4800	0.4906	-0.5556	0.1661	0.2819
metab_9022	neg	202.0505	3.0942	0.2025	-0.1729	0.4971	0.6178
metab_251	pos	202.0705	0.8780	0.2165	-0.3315	0.4493	0.5788
metab_13308	neg	202.0715	3.2801	0.1470	-0.0956	0.5890	0.6964
metab_12838	neg	202.0715	4.8049	0.6893	0.8554	0.0045	0.0226
metab_13054	neg	202.0715	4.0347	0.6469	0.6627	0.0004	0.0049
metab_14578	neg	202.0716	1.0785	0.2492	0.5428	0.5042	0.6241
metab_7514	neg	202.0716	1.8682	0.0349	0.1548	0.9658	0.9763
metab_13663	neg	202.0870	2.4813	1.4189	-1.7809	0.0006	0.0064
metab_13218	neg	202.0870	3.5475	1.0619	-1.6434	0.0675	0.1461
metab_13401	neg	202.0872	3.0260	1.2869	-1.8095	0.0019	0.0128
metab_5677	pos	202.1069	1.8729	0.0744	0.0784	0.9323	0.9573
metab_553	pos	202.1070	2.2209	0.2971	-0.4840	0.3651	0.4986
metab_8037	neg	202.1080	0.9649	0.8477	-1.6612	0.1145	0.2138
metab_7625	neg	202.1080	2.6206	0.5871	-0.0665	0.2346	0.3601
metab_14974	neg	202.1193	0.5286	0.2920	1.4454	0.5979	0.7038
metab_9332	neg	202.1232	4.2203	0.4067	0.8890	0.4572	0.5804
metab_5111	pos	202.1434	3.3032	0.8677	0.9494	0.0152	0.0480
metab_14940	neg	202.1557	0.5286	0.1770	0.2229	0.4734	0.5956
metab_5349	pos	202.1797	2.5314	0.0700	-0.2680	0.6871	0.7805
metab_13589	neg	202.1845	2.6206	0.5819	0.0378	0.2221	0.3462
metab_176	pos	202.1909	0.5140	0.6258	0.6257	0.0836	0.1660
metab_11175	neg	202.9285	11.2289	0.3279	-0.0650	0.1416	0.2507
metab_8996	neg	202.9926	3.0260	1.2283	-1.3720	0.0015	0.0110
metab_1155	pos	203.0062	0.5280	0.8694	-1.1547	0.0009	0.0070
metab_6884	neg	203.0557	1.4568	0.3934	-0.1346	0.1324	0.2382
metab_8608	neg	203.0557	2.0358	0.2038	0.2585	0.5018	0.6219
metab_10777	neg	203.0597	14.0438	0.3855	-0.0845	0.0350	0.0916
metab_6883	neg	203.0668	0.5991	0.9149	-1.0545	0.0280	0.0793
metab_8326	neg	203.0822	1.5297	0.4794	-0.1952	0.2899	0.4213
metab_14094	neg	203.0920	1.7264	0.9588	1.5073	0.0034	0.0188
metab_8657	neg	203.0920	2.1630	0.8756	1.2290	0.0025	0.0150
metab_1319	pos	203.1021	0.6262	1.1699	1.2163	0.0001	0.0020
metab_14821	neg	203.1031	0.5991	0.4728	0.7319	0.0558	0.1272
metab_6338	pos	203.1134	0.6123	1.0250	12.0484	0.0192	0.0566
metab_5211	pos	203.1175	2.9652	0.4251	1.6975	0.4551	0.5845
metab_9428	neg	203.1284	4.6722	1.4529	3.1097	0.0005	0.0057
metab_13558	neg	203.1284	2.6986	0.1038	-0.4139	0.7997	0.8565
metab_9114	neg	203.1284	3.4639	0.5746	-1.0493	0.3141	0.4457
metab_13165	neg	203.1284	3.6980	0.2247	-0.2164	0.6985	0.7837
metab_8635	neg	203.1285	2.1153	0.7658	1.3273	0.0219	0.0670
metab_274	pos	203.1386	1.1164	0.6452	0.7701	0.0642	0.1369
metab_479	pos	203.1386	2.7959	0.5128	-0.5707	0.0269	0.0726

metab_4864	pos	203.1427	4.3371	1.4218	1.9838	0.0002	0.0031
metab_6374	pos	203.1497	0.5983	0.5974	-0.5354	0.0106	0.0374
metab_1234	pos	203.2226	0.5140	1.0640	1.6828	0.0024	0.0133
metab_6424	pos	203.5629	0.5420	0.8770	-1.3825	0.1075	0.2001
metab_7768	neg	203.8553	0.1425	0.3621	-0.0806	0.0546	0.1252
metab_10950	neg	203.8554	14.1904	0.3948	-0.0880	0.0381	0.0977
metab_14291	neg	204.0299	1.4856	0.6098	1.1850	0.1793	0.2973
metab_8864	neg	204.0299	2.6678	0.9958	7.9176	0.0407	0.1019
metab_13756	neg	204.0300	2.2912	1.3416	2.2347	0.0004	0.0051
metab_8305	neg	204.0411	1.4856	0.4404	-0.0552	0.1489	0.2598
metab_6794	neg	204.0509	0.8946	0.5532	0.7304	0.0615	0.1369
metab_1791	pos	204.0651	2.0828	0.5482	0.6759	0.1099	0.2034
metab_7620	neg	204.0662	2.6046	1.0527	-1.2465	0.0036	0.0194
metab_14128	neg	204.0663	1.6642	0.2674	0.3083	0.5696	0.6801
metab_8290	neg	204.0782	1.4568	0.0187	-1.2610	0.9743	0.9818
metab_3396	pos	204.0861	14.8330	1.1286	1.3033	0.0000	0.0002
metab_2637	pos	204.0862	6.8433	1.6385	2.7719	0.0002	0.0023
metab_6481	pos	204.0862	0.5140	0.9644	0.9301	0.0000	0.0002
metab_2330	pos	204.0862	4.4580	1.0630	1.1897	0.0003	0.0031
metab_6816	neg	204.0873	0.9649	0.6778	1.1825	0.0314	0.0855
metab_5952	pos	204.1015	1.3437	0.6342	2.4736	0.3784	0.5113
metab_1964	pos	204.1015	2.7029	1.0137	-1.2915	0.0039	0.0183
metab_8482	neg	204.1025	1.7729	1.0705	-2.6603	0.0351	0.0919
metab_218	pos	204.1225	0.6542	0.6468	-2.3278	0.1981	0.3155
metab_6620	neg	204.1237	1.3231	1.4860	13.2006	0.1622	0.2770
metab_6329	pos	204.1334	0.6262	0.9461	1.9007	0.1469	0.2522
metab_6245	pos	204.1335	0.7941	0.6528	1.2787	0.1363	0.2387
metab_1610	pos	204.1488	1.5284	1.2057	-1.6273	0.0012	0.0086
metab_1931	pos	204.1591	2.5469	1.1878	2.2863	0.0064	0.0260
metab_5389	pos	204.1743	2.4370	0.1605	-1.2899	0.8246	0.8843
metab_15080	neg	204.8632	0.0753	0.2813	0.0483	0.1272	0.2313
metab_10891	neg	204.8924	14.5839	0.6340	-0.3183	0.0053	0.0256
metab_10734	neg	204.9240	13.9633	0.2881	0.0450	0.1067	0.2038
metab_11520	neg	204.9399	9.2185	0.4891	-0.0336	0.0001	0.0026
metab_10837	neg	204.9400	14.1255	0.1954	-0.2323	0.5350	0.6503
metab_11035	neg	204.9566	14.0610	0.2882	0.0366	0.0842	0.1711
metab_10973	neg	204.9799	14.1424	0.2989	0.0114	0.1332	0.2394
metab_13983	neg	205.0139	1.8833	0.5115	0.6181	0.4273	0.5526
metab_8015	neg	205.0349	0.8946	0.4528	-0.0368	0.0391	0.0991
metab_13183	neg	205.0350	3.6311	0.0159	0.2768	0.8010	0.8576
metab_13755	neg	205.0350	2.2912	0.3088	0.0189	0.1850	0.3036
metab_7583	neg	205.0502	2.3078	0.2721	-0.1011	0.4931	0.6142
metab_8624	neg	205.0615	2.0670	0.4782	-0.6508	0.1783	0.2960
metab_1144	pos	205.0677	0.5983	0.6894	2.0906	0.2913	0.4215
metab_14597	neg	205.0712	1.0215	1.2804	2.2196	0.0002	0.0028
metab_8433	neg	205.0713	1.6797	1.4131	2.7426	0.0000	0.0014
metab_3508	pos	205.0741	14.0378	0.2117	-0.2315	0.5703	0.6858
metab_1374	pos	205.0813	0.8221	1.1476	-2.0044	0.0366	0.0904

metab_1054	pos	205.0966	1.5284	0.1925	-0.0698	0.6070	0.7168
metab_7477	neg	205.0979	1.6797	0.0324	2.2235	0.8150	0.8685
metab_13535	neg	205.1078	2.7475	1.2103	2.0703	0.0026	0.0158
metab_8142	neg	205.1078	1.2521	1.0018	1.5429	0.0005	0.0057
metab_5326	pos	205.1257	2.6090	0.2033	-0.0102	0.5767	0.6907
metab_5309	pos	205.1695	2.6561	1.7701	-3.5112	0.0005	0.0050
metab_5660	pos	205.1696	1.9169	0.9271	-1.6717	0.0569	0.1256
metab_6691	neg	205.8392	0.4307	0.3917	-0.0630	0.0300	0.0827
metab_7179	neg	205.8392	14.1581	0.4465	-0.1121	0.0124	0.0459
metab_13178	neg	205.8393	3.6479	0.8516	-0.6756	0.0148	0.0514
metab_10860	neg	205.8523	14.1904	0.3777	-0.0925	0.0557	0.1270
metab_7734	neg	205.9229	0.0197	0.3548	-0.0975	0.0799	0.1652
metab_10839	neg	205.9878	14.1424	0.3628	-0.0441	0.0418	0.1041
metab_8721	neg	206.0276	2.3078	0.4631	1.7424	0.6168	0.7195
metab_9009	neg	206.0277	3.0432	1.7226	10.7088	0.0023	0.0141
metab_8285	neg	206.0455	1.4568	1.1628	2.2472	0.0068	0.0305
metab_14673	neg	206.0566	0.8662	0.3039	1.1000	0.6779	0.7680
metab_5955	pos	206.0806	1.3437	0.7676	1.8751	0.1514	0.2580
metab_8808	neg	206.0818	2.5264	0.1931	-0.0127	0.6953	0.7815
metab_6090	pos	206.0920	1.1306	0.5162	0.6836	0.1764	0.2895
metab_342	pos	206.0999	1.5284	0.1010	-0.0142	0.7303	0.8142
metab_16	pos	206.1017	1.0040	0.6001	0.8758	0.0562	0.1242
metab_1035	pos	206.1170	1.8577	0.1661	-0.5255	0.5582	0.6752
metab_12712	neg	206.1182	5.4034	0.2139	0.1118	0.7115	0.7934
metab_45	pos	206.1382	1.3153	1.4114	7.2501	0.0226	0.0636
metab_4816	pos	206.1535	4.5485	1.1679	2.3827	0.0053	0.0228
metab_1308	pos	206.1646	0.6123	0.3046	0.2158	0.3830	0.5156
metab_10867	neg	206.8603	14.2390	0.3689	-0.0220	0.0597	0.1338
metab_15087	neg	206.8604	0.0444	0.1263	0.0845	0.5351	0.6503
metab_195	pos	206.8854	0.5560	0.8450	-0.8579	0.0830	0.1652
metab_11115	neg	206.9212	13.9952	0.5019	-0.1085	0.0039	0.0208
metab_10908	neg	206.9722	15.2820	0.2364	0.0397	0.1927	0.3125
metab_10792	neg	206.9723	14.0610	0.2089	0.0556	0.2067	0.3287
metab_7784	neg	206.9723	0.4634	0.3414	0.4068	0.0267	0.0768
metab_6836	neg	206.9964	1.1069	0.2593	0.8945	0.7843	0.8462
metab_13693	neg	207.0119	2.4010	0.1701	0.6374	0.7981	0.8554
metab_6406	pos	207.0137	0.5560	1.9768	5.4105	0.0003	0.0038
metab_14182	neg	207.0294	1.5885	0.4618	0.4987	0.2472	0.3741
metab_13542	neg	207.0295	2.7318	1.3387	2.2693	0.0083	0.0348
metab_14718	neg	207.0505	0.7818	0.3974	0.6053	0.1521	0.2641
metab_387	pos	207.0648	1.8577	0.4512	0.7440	0.2601	0.3877
metab_7710	neg	207.0661	3.3803	0.0158	0.1147	0.8459	0.8929
metab_13781	neg	207.0772	2.2437	1.3071	-2.2286	0.0029	0.0167
metab_12793	neg	207.0811	4.9510	1.7982	-4.5009	0.0000	0.0008
metab_5437	pos	207.1011	2.3447	1.1587	1.9517	0.0042	0.0193
metab_12794	neg	207.1022	4.9510	0.4518	-0.1866	0.0285	0.0801
metab_6302	pos	207.1051	0.6402	1.6550	-5.7949	0.0000	0.0010
metab_5768	pos	207.1122	1.6693	0.4992	0.1915	0.0166	0.0508

metab_8626	neg	207.1136	2.0829	0.4057	-1.1491	0.4240	0.5497
metab_14098	neg	207.1137	1.7104	0.1259	-0.7389	0.7873	0.8480
metab_13554	neg	207.1387	2.6986	1.4573	2.9942	0.0008	0.0074
metab_1509	pos	207.1487	1.2162	1.5391	2.5053	0.0000	0.0008
metab_4981	pos	207.1586	3.7908	0.3255	-0.2923	0.1441	0.2487
metab_2078	pos	207.1587	3.1799	0.3158	-0.2603	0.1052	0.1968
metab_140	pos	207.1738	5.3454	0.3997	5.9824	0.6192	0.7266
metab_615	pos	207.1739	4.8509	0.9100	4.2950	0.1423	0.2464
metab_5571	pos	207.1851	2.0828	1.0120	-1.3080	0.1347	0.2367
metab_4982	pos	207.1851	3.7908	0.8592	-0.5921	0.0825	0.1645
metab_2149	pos	207.1851	3.4549	0.6001	-0.2459	0.3204	0.4535
metab_452	pos	207.1851	2.4071	1.2218	-2.1655	0.0072	0.0286
metab_6690	neg	207.8363	0.4307	0.4262	-0.1019	0.0167	0.0558
metab_7176	neg	207.8363	14.1744	0.4367	-0.1039	0.0140	0.0496
metab_9388	neg	207.8363	4.5219	0.7800	-0.5901	0.0177	0.0580
metab_6676	neg	207.9182	0.0197	0.3296	-0.0166	0.0555	0.1267
metab_10874	neg	207.9307	14.3207	0.3586	-0.0328	0.0444	0.1084
metab_7776	neg	207.9307	0.4307	0.3015	-0.0221	0.0947	0.1864
metab_6438	pos	207.9851	0.5280	0.9557	-1.2891	0.0005	0.0045
metab_6916	neg	208.0612	1.4856	0.4442	-0.3148	0.1447	0.2548
metab_14105	neg	208.0612	1.7104	0.3700	1.3631	0.5462	0.6588
metab_10745	neg	208.0645	13.9952	0.3434	-0.0200	0.0541	0.1244
metab_8807	neg	208.0875	2.5113	0.2934	0.1827	0.6613	0.7555
metab_6124	pos	208.0962	1.0601	0.6186	1.3028	0.1929	0.3089
metab_1658	pos	208.0963	1.6553	0.1447	0.3651	0.7068	0.7960
metab_1926	pos	208.0964	2.5314	0.0112	-0.1665	0.9847	0.9909
metab_9367	neg	208.0975	4.4055	0.6348	-1.2579	0.2444	0.3708
metab_13138	neg	208.0975	3.7658	0.1099	0.1739	0.8661	0.9081
metab_13235	neg	208.0975	3.5141	0.0383	-0.1195	0.8789	0.9174
metab_13741	neg	208.0975	2.3227	0.1875	0.2222	0.6671	0.7598
metab_1688	pos	208.1326	1.7271	0.8163	1.3304	0.0421	0.1004
metab_2464	pos	208.1327	5.3913	0.0991	0.0028	0.8327	0.8899
metab_5539	pos	208.1327	2.1444	0.3038	0.0510	0.1820	0.2960
metab_6136	pos	208.1438	1.0180	0.3934	0.4794	0.4250	0.5565
metab_4796	pos	208.1691	4.6096	0.2951	0.0354	0.1194	0.2161
metab_11123	neg	208.8445	13.9796	0.0247	0.2420	0.8623	0.9054
metab_10392	neg	208.8483	9.0391	0.1250	0.1544	0.7512	0.8218
metab_1279	pos	208.8835	0.5560	0.9246	-1.1315	0.0148	0.0470
metab_10849	neg	208.9359	14.1744	0.1996	0.0058	0.3200	0.4522
metab_36	pos	208.9720	0.5560	0.1715	-0.2468	0.5025	0.6250
metab_192	pos	209.0083	0.5420	0.6245	1.0589	0.1737	0.2863
metab_6882	neg	209.0087	1.3373	0.4909	0.6689	0.1133	0.2126
metab_13849	neg	209.0089	2.1153	1.0835	3.1069	0.0659	0.1436
metab_5593	pos	209.0440	2.0360	0.8100	11.5200	0.0093	0.0341
metab_7572	neg	209.0452	2.2119	0.2571	0.0738	0.1513	0.2630
metab_6776	neg	209.0662	0.6131	1.1920	1.7978	0.0002	0.0031
metab_9280	neg	209.0716	4.0182	0.3803	-0.1489	0.3818	0.5115
metab_819	pos	209.0803	9.1302	0.1712	-0.1617	0.3580	0.4923

metab_13768	neg	209.0814	2.2595	1.3395	2.6027	0.0027	0.0162
metab_13650	neg	209.0815	2.5113	0.6831	-0.8488	0.0453	0.1100
metab_8892	neg	209.0816	2.7624	1.1598	-1.8838	0.0056	0.0264
metab_12991	neg	209.0850	4.2367	1.0234	4.6514	0.1336	0.2400
metab_304	pos	209.0916	1.3293	1.0889	1.3967	0.0001	0.0020
metab_14470	neg	209.0927	1.2805	0.0931	0.4705	0.9414	0.9617
metab_2126	pos	209.1167	3.3337	0.8977	1.0868	0.0049	0.0215
metab_490	pos	209.1168	2.8725	1.2243	1.8240	0.0002	0.0028
metab_9510	neg	209.1179	5.1131	0.8235	1.0941	0.0258	0.0751
metab_5968	pos	209.1278	1.3293	0.5164	1.9090	0.3520	0.4871
metab_202	pos	209.1384	0.5983	0.7713	0.9924	0.0212	0.0607
metab_5680	pos	209.1391	1.8577	0.8655	-0.9439	0.0325	0.0833
metab_4720	pos	209.1531	4.9554	1.3587	1.8542	0.0001	0.0019
metab_12607	neg	209.1542	5.8731	1.7514	-4.5792	0.0041	0.0210
metab_3277	pos	209.2006	14.0378	0.1253	-0.1242	0.5240	0.6450
metab_3539	pos	209.2006	13.2207	0.3636	-0.2840	0.0196	0.0574
metab_3537	pos	209.2007	13.3095	0.0536	-0.0964	0.6467	0.7490
metab_5197	pos	209.2007	2.9954	0.1810	-0.3944	0.5088	0.6310
metab_7774	neg	209.8333	0.3798	0.4583	-0.1320	0.0105	0.0409
metab_10416	neg	209.8333	9.1038	0.5573	-0.0038	0.1384	0.2465
metab_10964	neg	209.8334	14.1744	0.3825	-0.0542	0.0308	0.0843
metab_11680	neg	209.8334	8.7640	1.0376	-0.8639	0.0124	0.0459
metab_11604	neg	209.8495	9.0391	0.2413	0.0847	0.4993	0.6199
metab_11051	neg	209.9186	14.0438	0.3576	-0.0647	0.0400	0.1008
metab_7751	neg	209.9186	0.0320	0.5387	-0.3978	0.0387	0.0986
metab_10988	neg	209.9486	14.1090	0.0788	0.0540	0.6758	0.7662
metab_6583	neg	209.9486	0.4807	0.6030	-0.4439	0.0215	0.0661
metab_1277	pos	209.9559	0.5560	0.6921	0.2394	0.2419	0.3670
metab_8341	neg	210.0404	1.5439	0.1057	0.0522	0.6836	0.7726
metab_64	pos	210.0755	2.1595	0.4720	-0.7709	0.1044	0.1958
metab_1564	pos	210.0755	1.4005	0.6957	0.7845	0.0129	0.0427
metab_13777	neg	210.0768	2.2595	0.2322	-0.1301	0.5021	0.6222
metab_8269	neg	210.0769	1.4568	0.0407	0.0669	0.7743	0.8390
metab_5288	pos	210.0909	2.7341	0.0667	-0.0867	0.7981	0.8655
metab_2060	pos	210.0910	3.0726	0.7056	-1.2871	0.0744	0.1523
metab_361	pos	210.1118	1.6553	0.9649	1.0737	0.0015	0.0095
metab_5925	pos	210.1119	1.4005	1.9592	4.2777	0.0000	0.0001
metab_259	pos	210.1119	1.0601	2.1259	4.8254	0.0000	0.0000
metab_8131	neg	210.1132	1.2236	0.2866	-0.7511	0.7080	0.7908
metab_8982	neg	210.1132	2.9931	0.0498	-0.1050	0.9575	0.9719
metab_388	pos	210.1271	1.8729	1.2806	-1.7119	0.0873	0.1716
metab_2509	pos	210.1275	5.6934	0.5040	-0.5116	0.4414	0.5721
metab_5527	pos	210.1343	2.1595	0.9328	-1.3927	0.0139	0.0451
metab_2847	pos	210.1846	8.2289	0.3080	-0.3214	0.2021	0.3210
metab_6285	pos	210.9399	0.6962	0.3408	-0.4051	0.3923	0.5245
metab_14783	neg	211.0008	0.6131	0.3463	0.3924	0.2474	0.3743
metab_14030	neg	211.0245	1.8205	1.7166	3.2688	0.0007	0.0069
metab_7451	neg	211.0245	1.5439	1.3522	2.3312	0.0005	0.0057

metab_6881	neg	211.0359	1.3517	0.1578	0.7973	0.6978	0.7832
metab_8567	neg	211.0510	1.9596	0.9151	2.4553	0.0936	0.1848
metab_8368	neg	211.0608	1.5736	1.0884	1.8495	0.0017	0.0118
metab_14870	neg	211.0819	0.5711	1.3117	-1.8713	0.0002	0.0033
metab_4829	pos	211.0861	4.4730	0.3802	-0.3432	0.4029	0.5361
metab_2133	pos	211.0960	3.3490	0.1343	0.5499	0.7962	0.8641
metab_8627	neg	211.0973	2.0987	1.9888	4.2540	0.0000	0.0004
metab_14368	neg	211.1085	1.4267	1.2727	-3.2108	0.0208	0.0649
metab_5391	pos	211.1225	2.4370	0.5751	-0.4847	0.1981	0.3155
metab_2517	pos	211.1323	5.7540	1.3003	2.0500	0.0085	0.0322
metab_4703	pos	211.1323	5.0451	1.0154	1.0320	0.0023	0.0129
metab_9483	neg	211.1335	4.9349	0.2677	-0.2775	0.6059	0.7096
metab_13364	neg	211.1336	3.1286	0.9483	1.7315	0.0980	0.1913
metab_13561	neg	211.1336	2.6841	0.2476	0.4672	0.6935	0.7802
metab_6034	pos	211.1436	1.2016	0.2234	0.3501	0.5953	0.7060
metab_2428	pos	211.1436	5.1076	0.6488	6.6708	0.3085	0.4409
metab_2467	pos	211.1687	5.3913	0.2341	-0.1890	0.2940	0.4245
metab_559	pos	211.1688	2.1897	0.9166	3.0103	0.1056	0.1974
metab_3527	pos	211.1929	13.9785	0.4416	-0.2872	0.0006	0.0055
metab_3548	pos	211.1929	12.5544	0.3552	-0.2606	0.0194	0.0570
metab_11027	neg	211.9141	14.0610	0.5082	-0.2927	0.0153	0.0527
metab_7747	neg	211.9209	0.0259	0.3127	-0.0933	0.1138	0.2129
metab_15034	neg	211.9527	0.4975	0.6062	-0.2917	0.0469	0.1126
metab_8425	neg	212.0199	1.6797	0.6802	1.3805	0.1872	0.3059
metab_14618	neg	212.0560	0.9792	0.2138	-0.4072	0.5358	0.6509
metab_6854	neg	212.0561	1.2521	0.0346	0.0610	0.7832	0.8454
metab_13196	neg	212.0713	3.5978	0.9094	-1.0661	0.0777	0.1618
metab_1129	pos	212.0911	0.6262	2.0712	4.9190	0.0000	0.0003
metab_231	pos	212.0911	0.8920	1.2741	1.8500	0.0000	0.0003
metab_1100	pos	212.0912	1.1731	0.9797	1.0837	0.0000	0.0006
metab_390	pos	212.0912	1.9169	0.0308	-0.0945	0.9527	0.9697
metab_8737	neg	212.0925	2.3533	1.0078	-0.8902	0.0265	0.0765
metab_14007	neg	212.0925	1.8524	0.0759	-0.0435	0.9415	0.9617
metab_284	pos	212.1024	1.1731	0.1966	-0.0856	0.3625	0.4963
metab_14403	neg	212.1038	1.3517	1.4146	-4.8997	0.0003	0.0044
metab_1828	pos	212.1065	2.1746	0.2663	-0.2592	0.4601	0.5885
metab_5315	pos	212.1066	2.6402	0.9918	-1.3130	0.0002	0.0028
metab_1580	pos	212.1066	1.4429	1.0240	1.4169	0.0454	0.1063
metab_1419	pos	212.1275	0.9480	0.0262	-0.4599	0.9117	0.9429
metab_53	pos	212.1276	1.7705	0.2598	-0.9775	0.5752	0.6900
metab_13076	neg	212.1289	3.9508	0.4310	-0.8681	0.3906	0.5191
metab_5399	pos	212.1429	2.4224	1.4646	-2.5485	0.0026	0.0142
metab_580	pos	212.1640	4.0939	0.3659	-0.0243	0.3504	0.4855
metab_2384	pos	212.1640	4.7907	0.4948	1.9531	0.4029	0.5361
metab_5418	pos	212.1640	2.3908	0.0177	-0.1647	0.9933	0.9959
metab_2914	pos	212.2003	8.6132	0.3688	-0.3674	0.1004	0.1895
metab_4084	pos	212.2004	8.2728	0.1512	-0.2129	0.5461	0.6649
metab_1269	pos	212.8418	0.5420	0.5394	-0.6121	0.1556	0.2630

metab_157	pos	212.8512	0.5420	0.3383	-0.3060	0.1565	0.2643
metab_15103	neg	212.8843	0.0259	0.2884	0.0049	0.1055	0.2020
metab_15055	neg	212.8843	0.4634	0.3679	0.3905	0.0342	0.0903
metab_10967	neg	212.8844	14.1581	0.5561	-0.2185	0.0019	0.0128
metab_10704	neg	212.9104	12.4115	0.2172	0.0539	0.1828	0.3010
metab_1346	pos	212.9381	0.7102	0.7335	-1.1532	0.1320	0.2328
metab_8000	neg	212.9801	0.8382	1.3335	2.5663	0.0109	0.0419
metab_10848	neg	212.9880	14.1744	0.3001	-0.0056	0.1127	0.2120
metab_8447	neg	213.0038	1.7104	1.0976	1.6772	0.0645	0.1417
metab_10726	neg	213.0128	13.9143	0.0642	0.3004	0.7919	0.8509
metab_10606	neg	213.0148	10.0432	0.7327	0.9848	0.0570	0.1293
metab_6293	pos	213.0267	0.6542	0.0224	0.5423	0.8592	0.9095
metab_279	pos	213.0356	1.1447	1.2635	-2.6207	0.0024	0.0133
metab_8241	neg	213.0402	1.3965	0.6290	0.9793	0.1007	0.1950
metab_7880	neg	213.0489	0.5571	1.3596	13.0817	0.0191	0.0610
metab_6349	pos	213.0629	0.6123	0.8640	-0.8569	0.0059	0.0249
metab_5538	pos	213.0654	2.1444	0.3959	0.7589	0.4386	0.5690
metab_5646	pos	213.0655	1.9463	1.0380	1.3565	0.0066	0.0268
metab_35	pos	213.0743	0.5420	1.9114	4.0876	0.0009	0.0067
metab_308	pos	213.0752	1.3437	1.7286	3.6957	0.0000	0.0006
metab_7478	neg	213.0765	1.6797	1.3849	3.2520	0.0021	0.0134
metab_13672	neg	213.0765	2.4651	0.2675	0.5104	0.5359	0.6509
metab_8642	neg	213.0765	2.1311	0.4594	-1.4298	0.3624	0.4949
metab_14874	neg	213.0860	0.5571	0.5628	-0.8450	0.2661	0.3962
metab_14616	neg	213.0877	0.9792	0.8467	1.8806	0.0347	0.0912
metab_13006	neg	213.1129	4.2040	0.1610	-0.1875	0.7758	0.8402
metab_14102	neg	213.1130	1.7104	0.8849	1.3452	0.0555	0.1267
metab_6209	pos	213.1227	0.8640	0.4989	0.3351	0.1595	0.2681
metab_13945	neg	213.1241	1.9596	0.1055	0.4087	0.8840	0.9216
metab_13679	neg	213.1241	2.4487	0.3638	0.3523	0.4288	0.5537
metab_13869	neg	213.1241	2.0670	0.1292	0.6187	0.7492	0.8208
metab_5665	pos	213.1380	1.9014	0.8481	-0.9754	0.0598	0.1297
metab_5441	pos	213.1381	2.3292	1.0141	-1.3388	0.0143	0.0458
metab_4739	pos	213.1480	4.9267	0.2413	-0.5033	0.6493	0.7511
metab_1915	pos	213.1480	2.4850	1.1407	1.3993	0.0003	0.0035
metab_5635	pos	213.1481	1.9609	1.4874	4.1031	0.0027	0.0148
metab_9528	neg	213.1492	5.2100	0.6477	-1.3420	0.4054	0.5334
metab_12593	neg	213.1493	5.9375	0.8630	-1.7002	0.2256	0.3502
metab_12846	neg	213.1493	4.7709	0.9968	1.6382	0.0071	0.0312
metab_1824	pos	213.1593	2.1595	0.1606	-0.1054	0.4292	0.5608
metab_15083	neg	213.8749	0.0589	0.3423	-0.2603	0.1614	0.2760
metab_11135	neg	213.8828	13.9143	0.5339	-0.2998	0.0337	0.0896
metab_10899	neg	213.8887	15.8292	0.1050	0.3347	0.5797	0.6884
metab_12830	neg	213.8889	4.8371	0.5545	0.1665	0.2672	0.3976
metab_8560	neg	213.9635	1.9439	0.1890	2.8719	0.8221	0.8740
metab_8770	neg	213.9935	2.4175	0.3309	-0.1783	0.4692	0.5916
metab_13552	neg	214.0177	2.6986	1.2786	10.4952	0.0485	0.1153
metab_13452	neg	214.0177	2.9263	1.6789	11.4700	0.0115	0.0433

metab_6806	neg	214.0353	0.9086	0.1878	0.4558	0.8161	0.8694
metab_7588	neg	214.0481	0.5571	1.0679	1.5900	0.0040	0.0210
metab_13520	neg	214.0506	2.7624	0.2270	-0.2753	0.5349	0.6503
metab_8694	neg	214.0506	2.2437	1.1150	-1.6326	0.0214	0.0660
metab_8998	neg	214.0507	3.0260	0.7155	-0.6620	0.1261	0.2297
metab_3452	pos	214.0519	14.1389	0.4866	-0.4057	0.0038	0.0182
metab_7550	neg	214.0718	2.0055	0.1761	0.8797	0.8471	0.8940
metab_5863	pos	214.0855	1.5144	1.8131	4.1684	0.0002	0.0024
metab_1757	pos	214.0856	1.9918	0.4656	0.6460	0.2663	0.3943
metab_2151	pos	214.0857	3.4549	1.5258	-2.8939	0.0000	0.0002
metab_12820	neg	214.0870	4.8865	1.0366	-1.5501	0.0134	0.0482
metab_13540	neg	214.0870	2.7318	0.4007	-0.3002	0.1826	0.3008
metab_13463	neg	214.0870	2.8922	0.8696	-1.1402	0.0474	0.1135
metab_13817	neg	214.0870	2.1798	0.3100	-0.1063	0.1884	0.3075
metab_5765	pos	214.1068	1.6838	0.9418	1.0623	0.0004	0.0043
metab_3387	pos	214.1068	15.6534	0.1746	-0.0312	0.1517	0.2583
metab_1186	pos	214.1068	0.0720	0.0573	-0.0722	0.5694	0.6852
metab_48	pos	214.1068	1.2725	0.9164	0.8645	0.0000	0.0002
metab_5435	pos	214.1069	2.3600	0.8648	-1.0328	0.0092	0.0340
metab_13854	neg	214.1082	2.1153	0.9378	2.0208	0.0652	0.1427
metab_8396	neg	214.1082	1.6186	0.0544	0.2162	0.9362	0.9585
metab_278	pos	214.1180	1.1024	0.7457	0.6658	0.0037	0.0177
metab_1968	pos	214.1221	2.7188	1.4889	-3.3649	0.0009	0.0068
metab_1764	pos	214.1222	2.0062	2.7300	8.2321	0.0000	0.0000
metab_2046	pos	214.1334	3.0114	0.0373	0.1432	0.8621	0.9120
metab_11133	neg	214.1345	13.9143	0.3443	0.0292	0.0973	0.1905
metab_12859	neg	214.1347	4.7217	0.3294	-0.0767	0.1416	0.2506
metab_1208	pos	214.1395	0.4855	0.7551	0.9793	0.0179	0.0537
metab_469	pos	214.1433	2.5627	0.2229	0.1878	0.5884	0.7010
metab_12584	neg	214.1445	5.9542	0.9511	1.5031	0.0129	0.0470
metab_13033	neg	214.1446	4.1023	0.3789	1.4925	0.5454	0.6583
metab_4868	pos	214.1796	4.3371	0.2478	-0.1586	0.5116	0.6337
metab_2248	pos	214.1797	3.9270	0.2452	-0.3036	0.2645	0.3922
metab_2576	pos	214.2523	6.2226	0.2053	0.0856	0.1886	0.3041
metab_193	pos	214.8493	0.5560	0.2966	-0.3072	0.2682	0.3959
metab_10834	neg	214.8796	14.1255	0.4277	-0.0785	0.0127	0.0465
metab_15105	neg	214.8796	0.0259	0.2932	0.0018	0.0939	0.1852
metab_8533	neg	215.0016	1.8833	1.5422	3.6910	0.0026	0.0156
metab_14878	neg	215.0071	0.5571	0.7568	-0.7459	0.0146	0.0510
metab_10982	neg	215.0095	14.1255	0.3816	-0.1008	0.0420	0.1046
metab_9290	neg	215.0113	4.0685	1.1579	2.1118	0.0050	0.0245
metab_7872	neg	215.0227	0.5431	0.9638	-1.3439	0.0395	0.0998
metab_11687	neg	215.0324	8.7488	0.6890	-0.3555	0.0040	0.0210
metab_10431	neg	215.0325	9.1200	0.8351	-0.4420	0.0004	0.0048
metab_14933	neg	215.0437	0.5286	0.2277	0.0117	0.5565	0.6681
metab_1251	pos	215.0508	0.5280	0.0206	0.1293	0.8874	0.9286
metab_7459	neg	215.0559	1.5736	1.0401	-1.1358	0.0004	0.0045
metab_14731	neg	215.0669	0.7678	0.4863	2.5570	0.4994	0.6199

metab_1034	pos	215.0809	1.8871	0.9806	1.2343	0.0050	0.0220
metab_8534	neg	215.0823	1.8833	0.0944	0.4272	0.9894	0.9920
metab_179	pos	215.0872	0.5140	0.7293	-0.9667	0.1167	0.2124
metab_14042	neg	215.0922	1.8046	0.0918	0.4095	0.9117	0.9416
metab_9164	neg	215.0922	3.6142	1.3671	2.2525	0.0001	0.0016
metab_8380	neg	215.0923	1.5885	1.3751	2.5031	0.0002	0.0028
metab_258	pos	215.1020	0.9620	0.1740	-0.1846	0.4086	0.5413
metab_14538	neg	215.1034	1.1940	0.7429	1.3546	0.1103	0.2086
metab_6895	neg	215.1034	1.4267	0.2590	-0.1635	0.3776	0.5076
metab_2063	pos	215.1173	3.0877	1.0335	1.5482	0.0149	0.0472
metab_6957	neg	215.1286	4.4386	0.2417	0.3449	0.6267	0.7265
metab_13891	neg	215.1287	2.0358	0.3416	1.2175	0.5386	0.6533
metab_1333	pos	215.1384	0.6542	1.7225	5.4112	0.0000	0.0000
metab_1440	pos	215.1384	1.0461	0.6547	0.4142	0.0110	0.0383
metab_8136	neg	215.1398	1.2377	0.4977	-0.1794	0.3895	0.5182
metab_8556	neg	215.1398	1.9439	0.0208	1.4574	0.9670	0.9768
metab_8258	neg	215.1399	1.4427	0.2754	1.0283	0.5470	0.6593
metab_8457	neg	215.1399	1.7419	0.6413	1.7772	0.1386	0.2466
metab_13229	neg	215.1478	3.5141	0.2963	-0.1181	0.5837	0.6917
metab_4730	pos	215.1637	4.9267	0.0227	-0.3515	0.9855	0.9914
metab_4858	pos	215.1637	4.3512	0.5467	-0.0337	0.3090	0.4413
metab_9822	neg	215.1649	6.8565	0.3658	-0.0237	0.3491	0.4818
metab_15054	neg	215.8800	0.4634	0.2275	0.3404	0.3635	0.4958
metab_10913	neg	215.8855	14.6670	0.4207	0.6593	0.0691	0.1486
metab_10907	neg	215.8857	15.3818	0.2031	0.4748	0.3756	0.5063
metab_11070	neg	215.9214	14.0282	0.4520	-0.0814	0.0658	0.1435
metab_7782	neg	215.9261	0.4634	0.1367	0.0907	0.4868	0.6085
metab_10890	neg	215.9261	14.5507	0.3277	-0.0067	0.0701	0.1499
metab_14198	neg	215.9969	1.5736	1.4624	7.4986	0.0194	0.0615
metab_8762	neg	216.0092	2.4010	0.2737	-0.2378	0.5248	0.6422
metab_8769	neg	216.0486	2.4175	0.5937	-0.6621	0.1686	0.2849
metab_6227	pos	216.0625	0.8221	1.8046	3.8201	0.0002	0.0029
metab_8973	neg	216.0663	2.9760	0.1601	0.0009	0.5631	0.6742
metab_3307	pos	216.0676	14.1090	0.5724	-0.4871	0.0009	0.0069
metab_6819	neg	216.0874	0.9933	1.0403	1.8445	0.0029	0.0170
metab_7475	neg	216.0875	1.6642	0.9757	-0.9188	0.0073	0.0318
metab_14364	neg	216.0875	1.4427	0.4543	0.7747	0.2967	0.4274
metab_14819	neg	216.0986	0.5991	0.8144	3.0804	0.2088	0.3313
metab_4865	pos	216.1014	4.3371	1.1404	-2.0646	0.0325	0.0833
metab_5207	pos	216.1014	2.9806	0.4657	-0.0015	0.2772	0.4058
metab_5119	pos	216.1014	3.2727	1.1896	-1.9011	0.0028	0.0149
metab_526	pos	216.1015	2.4850	0.8546	-1.1616	0.0012	0.0083
metab_12975	neg	216.1027	4.2870	0.6049	-1.7241	0.2928	0.4243
metab_8550	neg	216.1139	1.9280	1.3635	-4.9365	0.0176	0.0579
metab_5805	pos	216.1225	1.6128	0.6491	0.6133	0.0025	0.0140
metab_57	pos	216.1226	2.1132	0.2789	0.2175	0.3542	0.4888
metab_13612	neg	216.1238	2.5893	0.7861	1.7222	0.1144	0.2137
metab_13785	neg	216.1238	2.2437	0.3074	0.8327	0.4750	0.5969

metab_7823	neg	216.1351	0.5286	0.3539	1.0151	0.5043	0.6241
metab_6533	pos	216.1371	0.4855	0.6239	0.7599	0.0311	0.0806
metab_5513	pos	216.1589	2.1746	1.2660	1.8870	0.0000	0.0006
metab_4911	pos	216.1589	4.0939	0.5997	1.4162	0.2767	0.4053
metab_7646	neg	216.1715	0.5286	0.7857	-0.5565	0.1384	0.2465
metab_6507	pos	216.2065	0.4999	1.6226	2.8911	0.0034	0.0170
metab_7722	neg	216.8531	0.0320	0.2487	-0.0187	0.2054	0.3274
metab_9885	neg	216.8534	7.0629	0.6101	-0.3114	0.0426	0.1056
metab_11170	neg	216.8534	11.2936	0.4888	-0.1277	0.0031	0.0176
metab_11565	neg	216.8535	9.1200	0.6207	-0.2136	0.0328	0.0881
metab_11735	neg	216.8535	8.7031	0.8645	-0.7852	0.0020	0.0134
metab_10918	neg	216.8765	14.5008	0.4552	-0.1080	0.0087	0.0361
metab_15141	neg	216.8766	0.0197	0.2994	-0.0021	0.0848	0.1721
metab_11126	neg	216.8799	13.9633	0.1772	0.1103	0.4590	0.5818
metab_14903	neg	216.9281	0.5431	1.2687	3.0991	0.0200	0.0630
metab_10735	neg	216.9337	13.9796	0.2897	0.0243	0.1113	0.2102
metab_11696	neg	216.9443	8.7346	0.5886	-0.3510	0.0105	0.0409
metab_13538	neg	216.9907	2.7318	0.8200	1.7716	0.1694	0.2857
metab_9291	neg	217.0085	4.0685	1.3580	2.7088	0.0042	0.0215
metab_6802	neg	217.0350	0.8662	0.3363	-0.3856	0.3165	0.4486
metab_8089	neg	217.0440	1.1069	0.0746	1.8520	0.7898	0.8493
metab_7497	neg	217.0479	0.6131	0.3563	2.0228	0.5145	0.6331
metab_11569	neg	217.0481	9.1200	0.5258	0.0171	0.1477	0.2587
metab_14235	neg	217.0715	1.5439	0.3770	0.7151	0.3738	0.5048
metab_7942	neg	217.0826	0.6411	0.2064	0.9213	0.5163	0.6346
metab_5367	pos	217.0854	2.5003	1.9227	4.5037	0.0001	0.0019
metab_5664	pos	217.0966	1.9169	0.5973	0.6714	0.0923	0.1790
metab_14010	neg	217.0981	1.8371	0.3365	-0.4258	0.4476	0.5710
metab_9475	neg	217.0996	4.8865	0.4756	-0.2435	0.0596	0.1336
metab_9549	neg	217.1078	5.3388	1.3090	2.9427	0.0062	0.0285
metab_13511	neg	217.1078	2.7961	0.8502	1.1104	0.0178	0.0584
metab_14278	neg	217.1079	1.5155	1.2865	2.2053	0.0000	0.0011
metab_6043	pos	217.1177	1.2016	0.5522	0.5882	0.1946	0.3112
metab_14361	neg	217.1190	1.4427	0.4651	0.5809	0.1909	0.3107
metab_5082	pos	217.1329	3.3943	1.5908	8.3147	0.0813	0.1627
metab_4334	pos	217.1429	7.0526	0.2077	-0.0817	0.4700	0.5970
metab_13740	neg	217.1442	2.3227	0.7991	2.1037	0.1261	0.2296
metab_13602	neg	217.1442	2.6046	0.8281	1.4538	0.0462	0.1114
metab_13125	neg	217.1442	3.8001	0.8305	-0.6078	0.0127	0.0464
metab_13426	neg	217.1443	2.9931	0.1403	0.2017	0.7350	0.8088
metab_13361	neg	217.1443	3.1455	0.5439	0.7115	0.0612	0.1364
metab_40	pos	217.1541	1.2162	1.0853	1.0381	0.0001	0.0011
metab_1738	pos	217.1541	1.9463	0.8366	1.6796	0.0699	0.1459
metab_5902	pos	217.1542	1.4429	0.9228	1.5077	0.0143	0.0458
metab_6480	pos	217.2018	0.5140	0.7998	1.4893	0.0875	0.1719
metab_6532	pos	217.6279	0.4855	1.1997	-2.3451	0.0643	0.1370
metab_10998	neg	217.8777	14.0927	0.3136	0.3218	0.3462	0.4793
metab_11139	neg	217.9006	13.8980	0.3348	-0.0076	0.0365	0.0945

metab_14126	neg	217.9948	1.6642	2.0088	-3.6527	0.0001	0.0018
metab_13415	neg	218.0459	3.0101	0.8859	1.6794	0.0347	0.0912
metab_14681	neg	218.0666	0.8662	0.2455	0.3076	0.5238	0.6414
metab_8460	neg	218.0749	1.7419	1.2037	2.3300	0.0142	0.0500
metab_1996	pos	218.0807	2.8411	0.2050	-0.2228	0.4374	0.5682
metab_8922	neg	218.0820	2.8269	0.1867	0.0358	0.5343	0.6501
metab_8790	neg	218.0854	2.4651	1.8668	-4.3768	0.0001	0.0020
metab_1482	pos	218.1017	1.1589	1.0802	1.2867	0.0000	0.0005
metab_6619	neg	218.1031	1.3089	0.5056	-0.1683	0.0242	0.0720
metab_13758	neg	218.1111	2.2912	1.1880	2.3024	0.0194	0.0616
metab_1469	pos	218.1129	1.1306	0.9044	1.2046	0.0102	0.0363
metab_5766	pos	218.1170	1.6693	1.1773	5.3940	0.0572	0.1258
metab_2287	pos	218.1170	4.1698	0.4611	2.4699	0.5661	0.6823
metab_2127	pos	218.1170	3.3337	1.1006	-1.7151	0.0129	0.0428
metab_9386	neg	218.1182	4.5054	1.2258	-2.0795	0.0295	0.0818
metab_13882	neg	218.1183	2.0516	0.0265	-0.3933	0.9721	0.9802
metab_280	pos	218.1381	1.0461	0.9815	-2.5899	0.0435	0.1026
metab_2651	pos	218.1381	6.8886	0.1381	-0.1608	0.5577	0.6749
metab_3373	pos	218.1381	15.6702	0.2288	-0.2479	0.1263	0.2250
metab_2537	pos	218.1381	5.9208	0.2016	-0.2828	0.1996	0.3176
metab_467	pos	218.1382	2.5938	0.8083	1.0296	0.0203	0.0589
metab_159	pos	218.1858	0.5280	0.1179	0.0412	0.6147	0.7229
metab_14422	neg	218.1890	1.3231	0.5988	-0.2926	0.0165	0.0553
metab_5134	pos	218.1898	3.2116	0.1060	-0.8668	0.8770	0.9214
metab_5041	pos	218.2110	3.5620	0.0648	-0.2308	0.7867	0.8567
metab_11129	neg	218.8762	13.9468	0.3854	-0.0276	0.0440	0.1077
metab_10708	neg	218.9309	13.3395	0.2673	0.0261	0.1628	0.2776
metab_4	pos	218.9831	0.4999	0.8769	-1.2879	0.0045	0.0205
metab_9021	neg	218.9887	3.0942	0.1036	0.0000	0.3592	0.4919
metab_11017	neg	219.0088	14.0771	0.1404	0.1510	0.4553	0.5785
metab_14829	neg	219.0452	0.5991	0.6997	2.1972	0.1457	0.2561
metab_11706	neg	219.0453	8.7191	0.4601	-0.1005	0.1559	0.2692
metab_10423	neg	219.0453	9.1200	0.4648	0.0929	0.1831	0.3014
metab_14401	neg	219.0507	1.3669	0.3547	0.5017	0.2220	0.3461
metab_6104	pos	219.0580	1.1024	0.4725	2.2086	0.5423	0.6621
metab_5192	pos	219.0646	3.0114	0.1249	-1.2283	0.7373	0.8197
metab_8763	neg	219.0860	2.4010	0.5147	-0.3105	0.1225	0.2246
metab_8155	neg	219.0871	1.2663	1.1229	1.9107	0.0009	0.0076
metab_14807	neg	219.0983	0.5991	0.3782	0.9053	0.4198	0.5460
metab_5769	pos	219.1121	1.6693	0.1244	-0.1731	0.5548	0.6724
metab_1575	pos	219.1124	1.4289	0.3218	0.2171	0.1967	0.3139
metab_14356	neg	219.1234	1.4427	1.2676	2.2447	0.0002	0.0028
metab_13470	neg	219.1235	2.8768	0.5700	1.0183	0.2522	0.3804
metab_2887	pos	219.2101	8.4074	0.7949	2.0029	0.1071	0.1994
metab_10904	neg	219.8450	15.8634	0.1738	0.0786	0.3291	0.4616
metab_7786	neg	219.8450	0.4634	0.2403	-0.0060	0.3055	0.4368
metab_10978	neg	219.8451	14.1255	0.0575	0.2591	0.7609	0.8286
metab_8970	neg	219.8451	2.9588	0.7820	0.1092	0.2412	0.3672

metab_7761	neg	219.8628	0.0589	0.1870	0.0620	0.2916	0.4228
metab_9614	neg	219.8913	5.7102	0.3260	-0.0556	0.2598	0.3895
metab_11016	neg	219.8914	14.0771	0.4350	-0.1060	0.0198	0.0626
metab_10402	neg	219.8914	9.0872	0.7964	-0.2228	0.0357	0.0928
metab_11719	neg	219.8914	8.7191	1.0934	-0.9955	0.0005	0.0057
metab_8563	neg	220.0249	1.9439	1.7425	4.2392	0.0003	0.0043
metab_1043	pos	220.0597	1.6553	1.0021	3.6182	0.1438	0.2483
metab_13899	neg	220.0612	2.0358	0.9137	1.4180	0.0019	0.0129
metab_8175	neg	220.0649	1.2805	1.7634	-2.2564	0.0009	0.0081
metab_14505	neg	220.0724	1.2521	0.3838	-0.0192	0.3536	0.4864
metab_1391	pos	220.0809	0.8640	0.7464	-1.3178	0.0573	0.1259
metab_7928	neg	220.0822	0.6131	0.1065	0.4958	0.9120	0.9418
metab_13409	neg	220.0977	3.0260	0.1044	0.2635	0.8952	0.9298
metab_13255	neg	220.0977	3.4470	0.2929	1.4230	0.6693	0.7615
metab_5701	pos	220.1074	1.7845	0.9006	1.0813	0.0757	0.1541
metab_0	pos	220.1173	1.3153	0.3864	-0.2621	0.0906	0.1765
metab_416	pos	220.1326	2.1132	0.0790	-0.4144	0.7283	0.8124
metab_2269	pos	220.1326	4.0479	0.9635	-1.2575	0.0068	0.0274
metab_7001	neg	220.1340	5.9542	0.4443	-0.3218	0.5425	0.6562
metab_6194	pos	220.1803	0.8780	0.2911	-0.4671	0.3330	0.4678
metab_6512	pos	220.4738	0.4999	1.5159	3.4084	0.0117	0.0400
metab_15073	neg	220.8342	0.1598	0.3372	-0.0129	0.0615	0.1368
metab_11128	neg	220.8720	13.9468	0.3742	-0.1262	0.1087	0.2065
metab_13360	neg	220.9545	3.1455	0.7025	2.8598	0.2832	0.4140
metab_6393	pos	221.0416	0.5703	0.2759	-0.1337	0.5287	0.6493
metab_7619	neg	221.0599	0.5431	0.4917	0.9577	0.2144	0.3373
metab_11154	neg	221.0663	13.2585	0.4774	-0.1442	0.0059	0.0273
metab_1994	pos	221.0803	2.8259	1.0461	-1.2759	0.0277	0.0744
metab_12867	neg	221.0816	4.7046	0.3091	0.5613	0.2605	0.3902
metab_14123	neg	221.0816	1.6642	1.6986	-5.0300	0.0000	0.0000
metab_14274	neg	221.0817	1.5155	2.1394	-5.6268	0.0001	0.0020
metab_1104	pos	221.0912	1.0321	1.0166	1.5500	0.0282	0.0754
metab_1393	pos	221.0913	0.8780	0.9721	1.6121	0.0951	0.1825
metab_1080	pos	221.0914	1.3153	0.8866	1.3948	0.0432	0.1022
metab_8378	neg	221.0930	1.5885	0.0640	0.2138	0.8669	0.9087
metab_8143	neg	221.1027	1.2521	0.6295	0.8645	0.0284	0.0800
metab_12790	neg	221.1180	4.9668	0.6213	-0.6577	0.0345	0.0909
metab_6029	pos	221.1278	1.2162	1.5963	2.6292	0.0000	0.0002
metab_1787	pos	221.1278	2.0677	0.1121	-1.0504	0.7877	0.8574
metab_5198	pos	221.1279	2.9954	0.7007	-0.9422	0.2330	0.3570
metab_13423	neg	221.1294	2.9931	0.4734	-0.1877	0.4137	0.5401
metab_8727	neg	221.1294	2.3227	0.6867	-2.0345	0.3625	0.4949
metab_13	pos	221.1530	4.8809	0.4371	1.3263	0.4255	0.5569
metab_5048	pos	221.1531	3.5311	1.5689	2.6957	0.0004	0.0045
metab_9860	neg	221.1543	7.0162	0.0137	0.1269	0.9793	0.9857
metab_6996	neg	221.1544	5.7102	0.7556	1.2223	0.0175	0.0575
metab_5292	pos	221.1643	2.7188	1.6545	-3.0357	0.0003	0.0032
metab_5243	pos	221.1643	2.8725	1.9274	-4.2896	0.0000	0.0002

metab_2281	pos	221.2006	4.1387	0.4136	-0.9493	0.5001	0.6230
metab_11011	neg	221.8761	14.0771	0.0276	0.2345	0.8294	0.8802
metab_10832	neg	221.8885	14.1090	0.6400	-0.2558	0.0004	0.0049
metab_11590	neg	221.8885	9.0872	0.7916	-0.2266	0.0343	0.0906
metab_10843	neg	221.9829	14.1581	0.4292	-0.0424	0.0321	0.0866
metab_1299	pos	222.0191	0.5983	1.3175	-3.5956	0.0334	0.0847
metab_1274	pos	222.0285	0.5560	0.1762	-1.3100	0.7096	0.7980
metab_7631	neg	222.0368	0.5286	0.4614	-0.3025	0.1430	0.2526
metab_8172	neg	222.0405	1.2805	1.1596	1.9347	0.0072	0.0317
metab_14327	neg	222.0405	1.4568	0.3483	0.5288	0.3598	0.4926
metab_13312	neg	222.0406	3.2801	1.3798	2.7429	0.0008	0.0071
metab_1773	pos	222.0754	2.0360	0.8738	0.9138	0.0004	0.0039
metab_8166	neg	222.0769	1.2805	0.9386	-1.0462	0.0055	0.0262
metab_14261	neg	222.0769	1.5297	0.2957	-0.1742	0.4813	0.6036
metab_1187	pos	222.0800	0.0894	0.2471	-0.1921	0.0841	0.1666
metab_3389	pos	222.0800	15.3788	0.1182	-0.1580	0.5772	0.6909
metab_297	pos	222.0867	1.2446	0.7042	1.5669	0.1896	0.3051
metab_14779	neg	222.0979	0.6131	0.7314	-0.3513	0.0126	0.0464
metab_12879	neg	222.1133	4.6550	0.9721	-1.6561	0.0986	0.1921
metab_9362	neg	222.1133	4.3716	0.4396	-0.7745	0.3080	0.4390
metab_14334	neg	222.1133	1.4568	1.1313	-0.9927	0.0052	0.0253
metab_13247	neg	222.1133	3.4639	0.3021	0.4962	0.6481	0.7439
metab_9252	neg	222.1133	3.9005	0.8756	1.0068	0.1268	0.2308
metab_13015	neg	222.1134	4.1699	0.9676	2.1433	0.1319	0.2374
metab_2528	pos	222.1482	5.8597	0.3547	-0.4687	0.5958	0.7065
metab_2089	pos	222.1483	3.2116	0.7577	0.4536	0.0710	0.1475
metab_6001	pos	222.1594	1.2446	0.0986	1.6661	0.7662	0.8409
metab_1873	pos	222.1596	2.3292	1.3194	-2.2082	0.0085	0.0320
metab_15063	neg	222.8295	0.4307	0.3222	-0.0125	0.0673	0.1458
metab_10857	neg	222.8296	14.1904	0.3820	-0.0574	0.0308	0.0843
metab_12681	neg	222.8296	5.5639	0.5849	-0.2771	0.0755	0.1580
metab_10716	neg	222.8404	13.8818	0.2859	0.0232	0.1182	0.2187
metab_7732	neg	222.9132	0.0197	0.2530	0.0550	0.1429	0.2524
metab_15	pos	222.9875	0.5560	0.5867	0.8535	0.1218	0.2196
metab_6443	pos	223.0240	0.5280	1.0012	-1.7422	0.0193	0.0567
metab_14025	neg	223.0245	1.8205	1.1660	2.0605	0.0109	0.0419
metab_8685	neg	223.0247	2.2272	1.2579	1.7844	0.0134	0.0482
metab_8030	neg	223.0279	0.9368	1.4169	-1.7773	0.0008	0.0071
metab_10660	neg	223.0280	10.5015	0.5828	-0.2609	0.0033	0.0182
metab_11196	neg	223.0281	10.7190	0.4772	-0.0391	0.0139	0.0493
metab_7929	neg	223.0456	0.6131	1.4233	2.9656	0.0042	0.0215
metab_8852	neg	223.0609	2.6524	0.8220	-1.4467	0.0457	0.1106
metab_9145	neg	223.0610	3.5641	1.1508	1.7603	0.0034	0.0188
metab_6178	pos	223.0631	0.9060	0.4094	0.1392	0.4890	0.6141
metab_6538	pos	223.0631	0.4543	0.3246	-0.2540	0.0076	0.0296
metab_3380	pos	223.0631	15.9959	0.2355	-0.2187	0.1405	0.2442
metab_1467	pos	223.0632	1.1164	0.1343	-0.1202	0.6091	0.7189
metab_13734	neg	223.0645	2.3380	0.5004	1.8593	0.4506	0.5741

metab_6890	neg	223.0722	1.3965	0.5300	0.7200	0.1196	0.2209
metab_6444	pos	223.0741	0.5280	0.6223	0.8286	0.1272	0.2264
metab_6280	pos	223.0741	0.7241	0.5028	0.6261	0.1641	0.2740
metab_9460	neg	223.0766	4.8210	1.4684	-3.7493	0.0418	0.1042
metab_12968	neg	223.0767	4.3207	0.1230	0.1384	0.8603	0.9036
metab_50	pos	223.0958	1.7128	0.4373	0.5847	0.2321	0.3560
metab_9212	neg	223.0974	3.7830	1.3089	3.1679	0.0125	0.0459
metab_5816	pos	223.1071	1.5847	0.1804	0.1474	0.5638	0.6804
metab_1526	pos	223.1071	1.2585	0.1605	-0.3551	0.5929	0.7044
metab_14133	neg	223.1086	1.6642	0.5802	-0.6752	0.1860	0.3047
metab_13432	neg	223.1086	2.9760	0.3097	-0.4872	0.4572	0.5804
metab_5546	pos	223.1324	2.1290	1.3488	2.2224	0.0001	0.0018
metab_307	pos	223.1434	1.3293	0.6129	0.8098	0.0975	0.1858
metab_5453	pos	223.1435	2.3133	0.7042	-1.8149	0.1397	0.2431
metab_1854	pos	223.1548	2.2665	1.8017	-1.6389	0.0433	0.1023
metab_2726	pos	223.1686	7.2763	0.0719	-0.4461	0.9108	0.9427
metab_2633	pos	223.1686	6.8280	0.0643	-0.0050	0.6682	0.7657
metab_10	pos	223.1686	5.2717	0.7360	3.3865	0.2210	0.3433
metab_10781	neg	223.7980	14.0438	0.3653	-0.0082	0.1188	0.2196
metab_9746	neg	223.8854	6.4073	0.2442	0.8726	0.4547	0.5779
metab_153	pos	223.9715	0.5703	0.8666	-1.1294	0.0003	0.0037
metab_6476	pos	224.0510	0.5140	0.0232	-0.1254	0.8631	0.9125
metab_8485	neg	224.0562	1.7885	0.0353	0.2034	0.9927	0.9945
metab_6361	pos	224.0612	0.5983	1.3189	-1.6620	0.0010	0.0077
metab_3390	pos	224.0754	15.3139	0.1863	-0.1757	0.2364	0.3603
metab_6553	pos	224.0754	0.2224	0.3265	-0.2493	0.0201	0.0586
metab_5182	pos	224.0755	3.0572	0.3315	-0.2714	0.3044	0.4362
metab_438	pos	224.0755	2.2519	0.3538	-0.3631	0.1665	0.2769
metab_5642	pos	224.0912	1.9609	0.8471	1.1985	0.0272	0.0735
metab_14701	neg	224.0925	0.8098	0.9302	13.6746	0.0012	0.0094
metab_6966	neg	224.0925	4.3716	0.4324	-0.6256	0.2339	0.3594
metab_13353	neg	224.0925	3.1626	0.3806	-0.5319	0.2437	0.3699
metab_7542	neg	224.0926	2.0516	0.0566	0.0054	0.7723	0.8374
metab_6899	neg	224.0926	1.4427	0.7103	1.1335	0.0553	0.1265
metab_13527	neg	224.0926	2.7624	0.3192	-0.6132	0.4062	0.5338
metab_4900	pos	224.1065	4.1544	0.5152	3.4213	0.5562	0.6737
metab_6305	pos	224.1122	0.6402	1.4199	-2.3449	0.0012	0.0086
metab_374	pos	224.1275	1.7412	1.3553	2.0008	0.0000	0.0009
metab_2315	pos	224.1640	4.3818	0.8686	1.7604	0.0895	0.1748
metab_295	pos	224.1751	1.2306	0.0712	0.0559	0.9088	0.9417
metab_764	pos	224.2002	8.2289	0.2155	-0.2223	0.3733	0.5072
metab_15062	neg	224.8266	0.4307	0.3247	-0.0117	0.0614	0.1368
metab_10965	neg	224.8266	14.1744	0.3607	-0.0414	0.0411	0.1028
metab_11600	neg	224.8266	9.0391	0.3295	0.0563	0.3220	0.4544
metab_12682	neg	224.8266	5.5639	0.3018	-0.0408	0.3204	0.4524
metab_15142	neg	224.9085	0.0197	0.2337	0.0301	0.2099	0.3326
metab_10997	neg	224.9085	14.0927	0.5395	-0.2927	0.0087	0.0360
metab_10946	neg	224.9085	14.2070	0.5282	-0.1497	0.0099	0.0396

metab_6408	pos	224.9864	0.5560	0.0154	0.0066	0.8220	0.8831
metab_13978	neg	225.0039	1.8974	0.5267	3.2777	0.4954	0.6166
metab_14507	neg	225.0402	1.2521	1.1994	-2.2551	0.0118	0.0441
metab_8557	neg	225.0402	1.9439	1.3099	2.2090	0.0000	0.0013
metab_9175	neg	225.0561	3.6479	0.2500	0.0515	0.4369	0.5607
metab_6605	neg	225.0612	0.5851	0.8997	-0.8267	0.0003	0.0042
metab_8522	neg	225.0766	1.8682	1.2148	-1.9119	0.0008	0.0074
metab_13910	neg	225.0797	2.0202	0.4765	1.4394	0.3410	0.4741
metab_223	pos	225.0863	0.6542	0.2068	-0.1985	0.2947	0.4252
metab_6864	neg	225.0878	1.2805	0.6903	1.0058	0.0355	0.0926
metab_13786	neg	225.0882	2.2272	0.0278	0.4427	0.8756	0.9149
metab_2362	pos	225.1116	4.6398	1.3934	11.8578	0.0301	0.0788
metab_5310	pos	225.1116	2.6561	1.6693	3.2836	0.0016	0.0099
metab_9313	neg	225.1130	4.1535	0.2093	1.0832	0.7808	0.8438
metab_13834	neg	225.1131	2.1475	0.7136	0.9459	0.0161	0.0546
metab_13333	neg	225.1131	3.2130	0.9427	1.2200	0.0048	0.0236
metab_302	pos	225.1227	1.3153	0.1694	-0.0996	0.6403	0.7436
metab_5209	pos	225.1227	2.9806	0.4606	-0.7512	0.2451	0.3696
metab_1004	pos	225.1268	5.9800	0.9209	0.8844	0.0379	0.0927
metab_2404	pos	225.1479	4.9413	0.4201	0.1186	0.0037	0.0178
metab_13296	neg	225.1494	3.2972	1.8293	3.8868	0.0022	0.0140
metab_5609	pos	225.1591	2.0062	0.4187	0.1190	0.4349	0.5661
metab_5005	pos	225.1592	3.6987	0.3369	-0.5525	0.2300	0.3536
metab_3264	pos	225.2085	13.9785	0.4175	-0.3150	0.0053	0.0228
metab_11833	neg	225.2222	8.5135	0.8343	1.3703	0.0055	0.0262
metab_11737	neg	225.2222	8.7031	0.1667	0.7824	0.5187	0.6368
metab_7171	neg	225.8970	14.1904	0.3364	-0.0417	0.0723	0.1532
metab_1286	pos	225.9696	0.5703	1.0647	-2.0596	0.0031	0.0160
metab_14302	neg	226.0178	1.4710	0.6666	1.2655	0.1910	0.3108
metab_14215	neg	226.0356	1.5586	0.4810	-0.7234	0.3105	0.4415
metab_9088	neg	226.0543	3.3304	1.4158	-4.1835	0.0333	0.0890
metab_5792	pos	226.0703	1.6272	0.9422	1.1161	0.0330	0.0842
metab_6263	pos	226.0703	0.7801	0.0204	0.0010	0.8557	0.9075
metab_14920	neg	226.0830	0.5431	0.6489	-0.2978	0.2432	0.3695
metab_8109	neg	226.0831	1.1645	1.1859	-3.3022	0.0050	0.0243
metab_6334	pos	226.1067	0.6262	1.8869	6.2854	0.0000	0.0000
metab_6237	pos	226.1067	0.8081	1.8630	3.8174	0.0000	0.0001
metab_8051	neg	226.1082	0.9933	0.9430	-2.7582	0.2343	0.3599
metab_9005	neg	226.1083	3.0432	0.3750	-0.5103	0.4431	0.5671
metab_8475	neg	226.1195	1.7729	1.6198	-2.8243	0.0070	0.0309
metab_14290	neg	226.1195	1.4856	1.2303	-3.6682	0.0231	0.0694
metab_1858	pos	226.1221	2.2665	0.0849	-0.5957	0.7947	0.8629
metab_404	pos	226.1261	2.0062	1.2482	1.7258	0.0001	0.0019
metab_5387	pos	226.1432	2.4530	0.9990	-1.5016	0.0159	0.0493
metab_6187	pos	226.1542	0.8920	0.3922	-0.2432	0.3615	0.4955
metab_3510	pos	226.1583	14.0378	0.1268	0.0115	0.3688	0.5027
metab_2044	pos	226.1585	2.9954	0.0563	-0.3860	0.7420	0.8225
metab_5464	pos	226.1585	2.2665	0.1387	0.0942	0.4402	0.5708

metab_606	pos	226.1795	4.6096	0.0023	-0.1107	0.8919	0.9313
metab_2160	pos	226.1796	3.5011	0.9285	1.1538	0.0401	0.0968
metab_4002	pos	226.2158	8.6132	0.4458	-0.4154	0.0284	0.0757
metab_3876	pos	226.2159	9.1302	0.1657	-0.2893	0.5179	0.6394
metab_4314	pos	226.2159	7.1416	0.2112	-0.2106	0.3348	0.4686
metab_15066	neg	226.8237	0.3798	0.3065	-0.0097	0.0891	0.1788
metab_10960	neg	226.8237	14.1744	0.3555	-0.0392	0.0459	0.1110
metab_7397	neg	226.8237	6.6962	0.4849	-0.2638	0.1170	0.2172
metab_3522	pos	226.9507	14.0228	0.0596	-0.2193	0.8210	0.8825
metab_11147	neg	226.9626	13.8160	0.1476	0.1209	0.4227	0.5488
metab_11091	neg	226.9654	14.0282	0.2666	-0.0027	0.2742	0.4047
metab_3386	pos	226.9743	15.6702	0.4358	-0.3471	0.0037	0.0179
metab_3453	pos	226.9743	14.1389	0.3985	-0.3103	0.0060	0.0250
metab_6511	pos	226.9867	0.4999	1.0210	-1.7374	0.0143	0.0458
metab_6761	neg	227.0194	0.6551	1.1224	2.0980	0.0162	0.0547
metab_6573	neg	227.0194	0.8662	0.9445	1.7261	0.0134	0.0482
metab_6333	pos	227.0421	0.6262	0.9760	1.4588	0.0131	0.0433
metab_6832	neg	227.0672	1.0785	0.2384	0.3154	0.4283	0.5534
metab_2017	pos	227.0809	2.9192	0.2920	0.6491	0.6057	0.7154
metab_9222	neg	227.0826	3.8001	0.0423	0.7428	0.9245	0.9508
metab_1884	pos	227.0908	2.3751	1.0389	1.2826	0.0009	0.0068
metab_13802	neg	227.0923	2.2119	1.1427	2.2061	0.0107	0.0413
metab_8418	neg	227.0924	1.6642	0.1616	0.3613	0.7557	0.8242
metab_5999	pos	227.1020	1.2585	0.4588	0.3869	0.1331	0.2342
metab_5778	pos	227.1020	1.6553	0.8570	0.6391	0.0002	0.0026
metab_7473	neg	227.1035	1.5155	0.0674	0.9904	0.7655	0.8319
metab_5345	pos	227.1174	2.5469	0.5271	1.8134	0.3896	0.5219
metab_12881	neg	227.1286	4.6550	1.2095	2.1386	0.0032	0.0179
metab_7412	neg	227.1287	6.1320	0.0273	0.2215	0.8458	0.8929
metab_6932	neg	227.1287	3.8506	0.9679	1.2833	0.0056	0.0265
metab_6193	pos	227.1383	0.8920	1.0324	1.2863	0.0015	0.0097
metab_8587	neg	227.1399	1.9904	0.4478	0.9068	0.2942	0.4253
metab_1945	pos	227.1537	2.6090	1.2012	-2.2638	0.0015	0.0097
metab_1881	pos	227.1537	2.3751	0.6047	-0.6484	0.0333	0.0846
metab_1983	pos	227.1537	2.7959	0.7382	-1.2410	0.0299	0.0785
metab_1776	pos	227.1539	2.0360	0.0112	0.0116	0.9799	0.9882
metab_12623	neg	227.1650	5.7751	0.2873	0.3884	0.3638	0.4960
metab_13249	neg	227.1651	3.4470	0.9697	2.3786	0.0243	0.0722
metab_3526	pos	227.2055	13.9785	0.2950	-0.1963	0.0745	0.1524
metab_3568	pos	227.2056	11.2519	0.4769	-0.3945	0.0228	0.0640
metab_9811	neg	227.7952	6.8093	0.3828	-0.0548	0.2436	0.3699
metab_13909	neg	228.0335	2.0202	0.0874	0.4704	0.7614	0.8287
metab_13436	neg	228.0335	2.9760	0.0927	0.5146	0.9047	0.9368
metab_8180	neg	228.0511	1.2948	0.5696	-0.7985	0.1843	0.3028
metab_9061	neg	228.0665	3.2473	0.0370	0.2208	0.7773	0.8409
metab_12248	neg	228.0808	7.2069	0.0652	-0.1619	0.8340	0.8835
metab_12037	neg	228.0808	7.9137	0.6894	-0.6758	0.0202	0.0635
metab_1506	pos	228.0860	1.2162	0.6982	0.4842	0.0003	0.0037

metab_14444	neg	228.0875	1.3089	1.0046	1.6108	0.0025	0.0154
metab_13944	neg	228.0875	1.9745	0.1931	0.5601	0.7340	0.8081
metab_7651	neg	228.0875	2.8123	0.2388	0.6929	0.7057	0.7892
metab_6603	neg	228.0875	2.2595	0.2137	-0.0914	0.5438	0.6572
metab_7630	neg	228.0876	2.6678	0.1040	0.5875	0.9304	0.9549
metab_236	pos	228.0971	0.7801	0.5520	-0.3755	0.0569	0.1256
metab_7990	neg	228.0986	0.7958	0.2742	-0.5294	0.3378	0.4710
metab_8126	neg	228.0988	1.2084	1.6408	-5.1314	0.0008	0.0071
metab_544	pos	228.1013	3.5771	1.0568	-1.8398	0.0548	0.1220
metab_5316	pos	228.1014	2.6402	0.2049	-0.2725	0.5320	0.6526
metab_13583	neg	228.1026	2.6357	1.0118	-1.2967	0.0040	0.0210
metab_13683	neg	228.1027	2.4338	1.4294	-4.1554	0.0022	0.0140
metab_9340	neg	228.1029	4.2702	1.9540	-4.6134	0.0007	0.0067
metab_9193	neg	228.1029	3.7148	1.7685	-3.4152	0.0012	0.0098
metab_14280	neg	228.1068	1.5155	0.3273	1.2252	0.6200	0.7214
metab_1639	pos	228.1223	1.5987	1.3405	2.3952	0.0008	0.0064
metab_9426	neg	228.1239	4.6550	0.9499	-1.7306	0.0782	0.1626
metab_13101	neg	228.1321	3.8506	1.1954	3.1048	0.0431	0.1063
metab_5717	pos	228.1335	1.7705	2.9746	-3.4914	0.0006	0.0056
metab_1061	pos	228.1336	1.4860	0.0526	-0.2376	0.8216	0.8830
metab_4986	pos	228.1588	3.7599	0.6663	0.8359	0.2718	0.4003
metab_9477	neg	228.1603	4.9021	0.1463	-0.7213	0.7391	0.8119
metab_9301	neg	228.1603	4.1023	0.2124	-0.0946	0.6282	0.7276
metab_6569	neg	228.1716	0.5286	0.3314	-0.0393	0.4263	0.5518
metab_3974	pos	228.1950	8.6861	0.2158	0.0509	0.3335	0.4678
metab_4749	pos	228.1952	4.8961	0.1649	-0.1851	0.3878	0.5200
metab_4199	pos	228.2315	7.7800	0.3966	-0.3752	0.0545	0.1216
metab_4030	pos	228.2315	8.4651	0.8122	-0.8910	0.0010	0.0075
metab_7808	neg	228.9688	0.5126	0.7697	-1.0143	0.0422	0.1049
metab_6721	neg	229.0117	0.5431	0.7654	-1.1666	0.1081	0.2058
metab_8189	neg	229.0174	1.3089	1.8461	4.5508	0.0300	0.0826
metab_7462	neg	229.0175	1.5885	1.3619	2.8679	0.0043	0.0219
metab_6727	neg	229.0229	0.5571	0.0915	0.4589	0.9408	0.9617
metab_1405	pos	229.0336	0.9060	1.0650	2.4189	0.1064	0.1984
metab_6800	neg	229.0351	0.8803	0.3630	0.6799	0.3840	0.5131
metab_14528	neg	229.0352	1.2084	0.0180	0.0594	0.8655	0.9077
metab_6366	pos	229.0578	0.5983	0.6127	-0.7409	0.0329	0.0839
metab_7830	neg	229.0595	0.5286	1.3274	-2.1022	0.0012	0.0098
metab_13345	neg	229.0617	3.1797	0.2598	0.8727	0.7469	0.8189
metab_5806	pos	229.0713	1.5987	1.4418	10.8569	0.0010	0.0075
metab_7507	neg	229.0716	1.8524	0.6022	0.7105	0.0222	0.0676
metab_14695	neg	229.0827	0.8240	0.4741	1.3483	0.3686	0.5002
metab_6348	pos	229.0872	0.6123	0.7367	-0.3583	0.0609	0.1315
metab_13541	neg	229.1079	2.7318	0.6834	1.0151	0.0464	0.1117
metab_1057	pos	229.1176	1.5284	0.3565	-0.2819	0.3276	0.4614
metab_5592	pos	229.1177	2.0360	0.4441	-0.9426	0.1963	0.3133
metab_7447	neg	229.1192	1.5736	0.8268	1.3194	0.0618	0.1373
metab_5679	pos	229.1328	1.8577	1.1944	3.6497	0.0317	0.0817

metab_2165	pos	229.1330	3.5162	0.9710	-2.8164	0.0014	0.0094
metab_5414	pos	229.1330	2.3908	0.8418	-1.1383	0.0790	0.1594
metab_2349	pos	229.1428	4.5785	1.1600	-2.3640	0.0048	0.0212
metab_4344	pos	229.1428	7.0082	0.3950	-0.9169	0.4214	0.5528
metab_78	pos	229.1429	3.8511	0.8842	0.9887	0.0532	0.1194
metab_5424	pos	229.1429	2.3751	1.2743	1.7378	0.0000	0.0009
metab_2107	pos	229.1429	3.2573	0.6895	1.2142	0.1494	0.2556
metab_9688	neg	229.1443	6.1320	0.2496	0.1213	0.4614	0.5838
metab_12798	neg	229.1443	4.9510	0.1915	0.2348	0.6206	0.7217
metab_13316	neg	229.1443	3.2637	0.1167	-0.6793	0.7956	0.8534
metab_9341	neg	229.1443	4.2870	0.0128	0.2399	0.9730	0.9809
metab_9227	neg	229.1443	3.8333	0.4905	0.7493	0.2107	0.3334
metab_9163	neg	229.1443	3.6142	0.2873	0.9625	0.6178	0.7202
metab_1423	pos	229.1540	0.9761	0.9932	1.7120	0.0089	0.0334
metab_341	pos	229.1540	1.5705	0.0131	-0.1596	0.9768	0.9870
metab_14204	neg	229.1556	1.5736	0.5190	-0.5880	0.3556	0.4884
metab_14095	neg	229.1556	1.7264	0.0131	-0.1803	0.9820	0.9873
metab_12382	neg	229.1806	6.8255	0.2287	-1.7253	0.6693	0.7615
metab_10749	neg	229.9157	14.0117	0.4522	-0.1049	0.0114	0.0432
metab_9168	neg	229.9158	3.6142	0.4353	-0.1487	0.0206	0.0643
metab_7167	neg	229.9204	14.1255	0.3785	-0.0307	0.0326	0.0877
metab_14452	neg	230.0126	1.2948	0.5852	-0.6357	0.1299	0.2347
metab_8750	neg	230.0459	2.3855	1.2587	3.2568	0.0226	0.0685
metab_1768	pos	230.0476	2.0211	0.0592	0.3177	0.8349	0.8914
metab_252	pos	230.0654	0.8920	0.5410	2.0679	0.3562	0.4907
metab_14694	neg	230.0667	0.8240	0.3976	0.5996	0.3071	0.4382
metab_8588	neg	230.0669	1.9904	0.7464	1.6030	0.1932	0.3132
metab_5129	pos	230.0806	3.2274	0.2334	0.7958	0.7975	0.8652
metab_13406	neg	230.0821	3.0260	0.8717	-1.2885	0.0215	0.0663
metab_9078	neg	230.0821	3.2972	0.8051	-1.0455	0.0654	0.1429
metab_13599	neg	230.0821	2.6046	0.0314	0.5466	0.8507	0.8967
metab_13131	neg	230.0821	3.7830	0.8762	-0.9721	0.1384	0.2465
metab_13928	neg	230.0821	1.9904	0.9040	-2.0470	0.1076	0.2049
metab_6356	pos	230.0951	0.6123	1.1754	-1.4461	0.0007	0.0060
metab_2869	pos	230.0951	8.3322	0.9134	-1.1463	0.0024	0.0137
metab_4179	pos	230.0951	7.9118	0.6885	-0.8357	0.0121	0.0410
metab_2955	pos	230.0952	8.8169	0.8830	-1.0843	0.0004	0.0044
metab_2264	pos	230.0953	4.0338	0.7333	-0.8022	0.0036	0.0176
metab_2313	pos	230.0953	4.3672	0.5324	-0.4487	0.0305	0.0795
metab_4971	pos	230.0953	3.8361	0.5596	-0.4890	0.0280	0.0750
metab_5185	pos	230.0953	3.0422	0.5331	-0.4851	0.0096	0.0351
metab_5045	pos	230.0953	3.5465	0.3998	-0.3443	0.0867	0.1709
metab_5675	pos	230.1015	1.8729	0.5274	-0.7179	0.0788	0.1591
metab_14008	neg	230.1032	1.8524	0.8883	2.1476	0.0836	0.1705
metab_14537	neg	230.1032	1.1940	1.2158	2.2122	0.0011	0.0090
metab_13794	neg	230.1032	2.2272	1.0102	-0.9807	0.0383	0.0980
metab_309	pos	230.1128	1.3579	2.2550	5.7894	0.0000	0.0000
metab_610	pos	230.1169	4.7156	1.7046	-4.1091	0.0132	0.0434

metab_2223	pos	230.1170	3.7753	1.5234	-2.7363	0.0041	0.0192
metab_524	pos	230.1170	3.2727	1.6586	-3.6420	0.0014	0.0092
metab_5365	pos	230.1170	2.5003	1.1903	-2.0011	0.0025	0.0138
metab_75	pos	230.1380	1.2162	0.8821	-1.2869	0.0068	0.0272
metab_4876	pos	230.1381	4.2907	0.0800	0.4078	0.9133	0.9434
metab_13622	neg	230.1395	2.5592	0.3148	0.8369	0.5095	0.6288
metab_13772	neg	230.1395	2.2595	0.8152	1.3809	0.0288	0.0807
metab_9247	neg	230.1396	3.9005	0.4152	0.0262	0.4028	0.5309
metab_13508	neg	230.1397	2.7961	0.1494	0.9963	0.8720	0.9121
metab_13301	neg	230.1397	3.2972	0.1521	0.7091	0.8330	0.8829
metab_13189	neg	230.1661	3.6142	0.6352	1.1777	0.2807	0.4113
metab_5335	pos	230.1745	2.5782	0.4523	0.2149	0.1780	0.2917
metab_1367	pos	230.1855	0.7941	0.0555	-0.3589	0.8895	0.9298
metab_2572	pos	230.2472	6.1927	0.3613	-0.0148	0.2258	0.3492
metab_11008	neg	230.7661	14.0771	0.4694	-0.0897	0.0158	0.0540
metab_8254	neg	230.9968	1.4267	0.1858	2.6675	0.8608	0.9041
metab_9213	neg	231.0064	3.7830	0.5517	2.4637	0.4340	0.5581
metab_14689	neg	231.0144	0.8382	1.5288	3.0107	0.0025	0.0151
metab_1345	pos	231.0259	0.6962	1.8706	3.7841	0.0000	0.0001
metab_14921	neg	231.0271	0.5431	0.7434	-1.1842	0.1550	0.2679
metab_14676	neg	231.0313	0.8662	1.2459	2.6158	0.0747	0.1569
metab_168	pos	231.0344	0.4855	0.5764	-0.6864	0.0234	0.0654
metab_13621	neg	231.0661	2.5592	0.8692	-1.1345	0.0493	0.1165
metab_8741	neg	231.0662	2.3533	0.6657	-0.6483	0.1232	0.2255
metab_7612	neg	231.0773	2.5264	0.7199	-0.7032	0.1149	0.2143
metab_13956	neg	231.0872	1.9439	0.6045	1.1003	0.1181	0.2186
metab_14717	neg	231.0983	0.7818	1.2775	2.2234	0.0006	0.0064
metab_9404	neg	231.1025	4.5884	1.3625	-5.7678	0.0876	0.1765
metab_5404	pos	231.1123	2.4224	0.7858	-0.8169	0.0090	0.0336
metab_5459	pos	231.1220	2.2824	0.6010	-0.4286	0.0225	0.0635
metab_8844	neg	231.1236	2.6206	1.0247	1.6990	0.0014	0.0104
metab_8902	neg	231.1236	2.7792	0.7826	1.3881	0.0690	0.1483
metab_13258	neg	231.1237	3.4303	0.9261	1.2830	0.0088	0.0366
metab_9004	neg	231.1237	3.0432	0.8738	1.0631	0.0277	0.0788
metab_346	pos	231.1332	1.5565	0.7958	1.1596	0.0530	0.1192
metab_8048	neg	231.1347	0.9933	0.0302	0.2954	0.8859	0.9234
metab_8112	neg	231.1348	1.1796	0.1121	0.2212	0.7076	0.7907
metab_14297	neg	231.1349	1.4856	0.3792	0.5889	0.3697	0.5012
metab_14062	neg	231.1350	1.7572	0.5518	0.7091	0.2313	0.3566
metab_6357	pos	231.1445	0.5983	1.0184	11.8140	0.0110	0.0383
metab_1970	pos	231.1486	2.7188	0.1266	-0.4081	0.7883	0.8578
metab_2406	pos	231.1585	4.9554	0.1542	-0.2085	0.8219	0.8831
metab_12697	neg	231.1600	5.4844	0.2643	0.3565	0.5501	0.6621
metab_13192	neg	231.1600	3.6142	0.2522	0.6568	0.4015	0.5297
metab_6967	neg	231.1600	4.3549	0.5737	1.3368	0.2944	0.4253
metab_13137	neg	231.1600	3.7658	0.5701	0.9600	0.0356	0.0927
metab_9077	neg	231.1600	3.2972	0.4522	0.5529	0.4221	0.5480
metab_1629	pos	231.1696	1.5705	0.4564	-0.7300	0.3805	0.5128

metab_5741	pos	231.1697	1.7271	0.1895	-0.2353	0.7014	0.7916
metab_4272	pos	231.2100	7.3521	1.2292	-3.2086	0.0156	0.0487
metab_660	pos	231.2101	5.9361	1.3852	-0.5662	0.0775	0.1570
metab_4723	pos	231.2101	4.9413	0.4042	0.1725	0.4863	0.6116
metab_11014	neg	231.8946	14.0771	0.1667	0.0664	0.3689	0.5003
metab_7797	neg	231.9160	0.4975	0.3726	-0.2262	0.2355	0.3610
metab_10976	neg	231.9175	14.1424	0.2772	0.0211	0.1216	0.2235
metab_11056	neg	231.9314	14.0438	0.3140	0.0131	0.0713	0.1517
metab_15136	neg	231.9314	0.0197	0.1075	0.1120	0.5591	0.6704
metab_10731	neg	231.9432	13.9301	0.2819	0.0485	0.1063	0.2031
metab_191	pos	231.9878	0.5420	0.0025	0.0035	0.9135	0.9434
metab_13954	neg	232.0250	1.9439	1.1768	12.9434	0.0097	0.0391
metab_8311	neg	232.0286	1.5012	0.8719	-0.5830	0.0500	0.1173
metab_14594	neg	232.0461	1.0215	0.8365	-0.6690	0.1139	0.2129
metab_281	pos	232.0809	1.1731	0.4899	-0.9802	0.1959	0.3129
metab_6892	neg	232.0825	1.4115	0.2797	0.6356	0.5724	0.6826
metab_6597	neg	232.0825	1.6335	0.2738	-0.0290	0.4758	0.5978
metab_5366	pos	232.0962	2.5003	1.2518	-1.6811	0.0000	0.0001
metab_9033	neg	232.1011	3.1455	0.2744	-0.3676	0.5244	0.6420
metab_5682	pos	232.1172	1.8577	1.0233	1.7677	0.0192	0.0566
metab_6919	neg	232.1188	1.5155	0.5847	1.1282	0.1136	0.2128
metab_6709	neg	232.1301	0.5286	0.2724	1.6461	0.6654	0.7588
metab_12768	neg	232.1341	5.1131	1.2500	-2.1699	0.0361	0.0937
metab_5446	pos	232.1440	2.3292	0.3398	0.8043	0.5089	0.6311
metab_1068	pos	232.1536	1.4289	1.5067	-2.9722	0.0053	0.0229
metab_14965	neg	232.1665	0.5286	0.7582	1.1068	0.0258	0.0750
metab_5028	pos	232.1803	3.6073	0.3789	0.6700	0.4979	0.6211
metab_6497	pos	232.2013	0.4999	0.5432	0.4117	0.0377	0.0922
metab_5887	pos	232.2483	1.4569	1.6286	-3.6595	0.0003	0.0035
metab_15090	neg	232.7637	0.0382	0.1737	0.0083	0.3771	0.5075
metab_11009	neg	232.7637	14.0771	0.4719	-0.0850	0.0128	0.0467
metab_7763	neg	232.8585	0.0753	0.3934	-0.0536	0.0193	0.0615
metab_10955	neg	232.8585	14.1904	0.3234	-0.0284	0.0842	0.1711
metab_13918	neg	232.9680	2.0055	0.1966	0.0000	0.2936	0.4247
metab_8182	neg	233.0124	1.2948	1.1258	3.2378	0.0647	0.1419
metab_13641	neg	233.0431	2.5264	1.2412	-1.5639	0.0000	0.0005
metab_13460	neg	233.0455	2.8922	1.6451	-3.9118	0.0040	0.0209
metab_14656	neg	233.0565	0.8946	1.5610	-2.5150	0.0002	0.0027
metab_14508	neg	233.0665	1.2521	0.6015	1.0615	0.0988	0.1922
metab_8776	neg	233.0754	2.4338	0.8428	0.3906	0.3474	0.4803
metab_6751	neg	233.0776	0.6131	0.4839	-0.6423	0.2319	0.3571
metab_1889	pos	233.0803	2.3908	1.2937	3.1974	0.0246	0.0681
metab_9355	neg	233.0818	4.3380	1.5853	3.8456	0.0049	0.0241
metab_2018	pos	233.0915	2.9192	0.1420	2.7718	0.9574	0.9734
metab_14015	neg	233.1028	1.8371	1.1978	2.1029	0.0029	0.0169
metab_13473	neg	233.1029	2.8606	0.5710	-1.3482	0.2194	0.3432
metab_6262	pos	233.1125	0.7801	1.2399	1.8139	0.0000	0.0007
metab_2135	pos	233.1166	3.3635	1.1566	1.6439	0.0003	0.0038

metab_5427	pos	233.1206	2.3751	0.9004	-1.1169	0.0406	0.0976
metab_1312	pos	233.1237	0.6123	0.9335	1.0535	0.0217	0.0619
metab_2036	pos	233.1279	2.9806	0.3774	1.3290	0.4138	0.5458
metab_8476	neg	233.1393	1.7729	1.3987	2.6766	0.0003	0.0043
metab_13743	neg	233.1393	2.3227	0.7613	0.8430	0.2091	0.3316
metab_260	pos	233.1488	0.9761	0.0807	0.0377	0.7590	0.8353
metab_330	pos	233.1491	1.4860	1.6918	2.8869	0.0004	0.0043
metab_4566	pos	233.1529	5.8597	1.4857	5.0937	0.0001	0.0018
metab_9753	neg	233.1545	6.4709	0.1736	0.1043	0.3204	0.4524
metab_4545	pos	233.2257	5.9504	0.9308	-0.3152	0.1251	0.2238
metab_2609	pos	233.2258	6.6148	1.1004	-1.6046	0.0316	0.0816
metab_13264	neg	234.0408	3.3971	0.6585	1.7229	0.3176	0.4499
metab_1673	pos	234.0755	1.6983	0.9186	2.3746	0.1031	0.1939
metab_5413	pos	234.0755	2.3908	0.6072	0.7059	0.0792	0.1597
metab_7488	neg	234.0770	1.7264	0.4042	0.7648	0.3616	0.4944
metab_13564	neg	234.0771	2.6841	0.2281	0.5743	0.5465	0.6590
metab_6158	pos	234.0867	0.9620	0.0049	0.6559	0.9989	0.9993
metab_6299	pos	234.0965	0.6542	0.9298	-2.1620	0.0929	0.1796
metab_360	pos	234.0965	1.6413	0.2783	-0.3692	0.4763	0.6033
metab_5705	pos	234.1117	1.7845	0.7974	1.8377	0.0742	0.1522
metab_5663	pos	234.1118	1.9169	0.1726	0.1437	0.6587	0.7573
metab_13209	neg	234.1135	3.5813	1.0765	-1.5964	0.0052	0.0251
metab_7427	neg	234.1135	4.1869	0.0883	2.6405	0.7531	0.8228
metab_8437	neg	234.1135	1.6948	0.6328	0.7468	0.4028	0.5309
metab_5643	pos	234.1229	1.9463	1.3017	4.7864	0.0043	0.0197
metab_5440	pos	234.1232	2.3292	0.6471	-0.4520	0.1477	0.2533
metab_334	pos	234.1329	1.5004	0.8392	1.0303	0.0037	0.0177
metab_364	pos	234.1480	1.6983	0.4499	0.1442	0.3622	0.4962
metab_2276	pos	234.1483	4.0939	0.3271	-0.4691	0.3542	0.4888
metab_5526	pos	234.1484	2.1595	0.5608	0.3543	0.3004	0.4315
metab_1803	pos	234.1694	2.1132	1.6771	2.5994	0.0001	0.0012
metab_10815	neg	234.7613	14.0771	0.4847	-0.0974	0.0101	0.0401
metab_11602	neg	234.8198	9.0391	0.3940	0.0032	0.2799	0.4106
metab_10852	neg	234.8555	14.1744	0.3025	-0.0122	0.1044	0.2004
metab_15089	neg	234.8556	0.0382	0.3436	-0.0654	0.1956	0.3159
metab_9012	neg	234.9703	3.0604	1.2847	-2.2557	0.0262	0.0758
metab_8637	neg	234.9837	2.1153	0.4342	7.8295	0.2278	0.3528
metab_7560	neg	235.0248	2.1153	1.7508	0.5511	0.0667	0.1448
metab_14588	neg	235.0281	1.0357	0.7249	4.3948	0.3971	0.5250
metab_6750	neg	235.0457	0.6131	1.0628	2.1961	0.0385	0.0983
metab_13684	neg	235.0611	2.4338	0.6643	-0.8231	0.0842	0.1711
metab_8935	neg	235.0642	2.8440	0.5631	1.3189	0.4032	0.5313
metab_14155	neg	235.0723	1.6335	0.4950	2.6149	0.5046	0.6244
metab_14564	neg	235.0821	1.1069	0.9198	1.8930	0.0333	0.0890
metab_12765	neg	235.0974	5.1131	0.5608	-0.3592	0.0557	0.1270
metab_1098	pos	235.1069	1.2016	0.5361	0.6019	0.1152	0.2102
metab_4255	pos	235.1086	7.4561	0.8786	0.9146	0.0052	0.0225
metab_4232	pos	235.1087	7.6042	0.7026	0.3175	0.1214	0.2190

metab_13528	neg	235.1087	2.7475	0.4078	0.7607	0.4140	0.5403
metab_8395	neg	235.1087	1.6186	0.3748	0.6633	0.2878	0.4195
metab_9133	neg	235.1088	3.5141	0.2104	-0.0223	0.7017	0.7861
metab_6868	neg	235.1185	1.2805	0.9938	1.4148	0.0017	0.0119
metab_13661	neg	235.1186	2.4970	0.8650	1.3651	0.0221	0.0675
metab_1740	pos	235.1322	1.9463	0.8882	1.1485	0.0042	0.0195
metab_614	pos	235.1322	4.8359	0.1101	0.1307	0.7780	0.8501
metab_328	pos	235.1431	1.4569	1.0828	1.8234	0.0065	0.0265
metab_1704	pos	235.1435	1.7705	0.7222	8.9405	0.0215	0.0614
metab_2170	pos	235.1435	3.5465	0.8650	-1.2308	0.2130	0.3340
metab_9141	neg	235.1451	3.5475	0.5529	-1.0536	0.2675	0.3979
metab_4973	pos	235.1534	3.8208	0.3468	-0.2754	0.1055	0.1973
metab_2673	pos	235.1686	7.0082	0.2319	-0.2237	0.5671	0.6832
metab_5602	pos	235.1686	2.0211	1.9886	4.2740	0.0007	0.0062
metab_4742	pos	235.1686	4.9115	1.3836	3.0794	0.0086	0.0323
metab_5542	pos	235.1797	2.1290	0.9941	-2.6879	0.0627	0.1344
metab_5116	pos	235.1799	3.2727	1.9272	-4.2188	0.0000	0.0002
metab_10864	neg	235.9259	14.2390	0.3726	-0.0473	0.0376	0.0967
metab_15056	neg	235.9259	0.4634	0.2690	0.0032	0.1285	0.2330
metab_13543	neg	235.9947	2.7155	0.9512	-0.3086	0.0392	0.0994
metab_477	pos	236.0548	2.7029	0.7435	2.8031	0.2389	0.3632
metab_14399	neg	236.0563	1.3669	0.6816	0.9175	0.0260	0.0755
metab_8916	neg	236.0565	2.8123	0.0216	0.1279	0.9130	0.9426
metab_7493	neg	236.0774	0.5991	0.1034	0.1886	0.8151	0.8685
metab_6410	pos	236.0789	0.5560	0.2873	-0.1915	0.2344	0.3586
metab_5409	pos	236.0912	2.4071	0.4419	-0.5189	0.2097	0.3298
metab_1018	pos	236.0912	2.2045	0.0205	0.1065	0.9654	0.9788
metab_6858	neg	236.0927	1.2663	1.2382	-1.7439	0.0063	0.0288
metab_7643	neg	236.0927	2.7155	0.8437	-0.2920	0.0328	0.0880
metab_12641	neg	236.1052	5.7259	0.9506	1.3672	0.0004	0.0047
metab_5620	pos	236.1107	1.9918	1.7458	4.0795	0.0000	0.0003
metab_282	pos	236.1274	1.0321	0.4599	2.7360	0.4533	0.5827
metab_365	pos	236.1275	1.6983	0.7988	1.2593	0.0201	0.0585
metab_14146	neg	236.1290	1.6335	1.1609	-2.1746	0.0113	0.0430
metab_12861	neg	236.1291	4.7217	0.9538	1.9926	0.1343	0.2410
metab_9171	neg	236.1291	3.6311	0.7363	-0.9731	0.0219	0.0671
metab_1662	pos	236.1386	1.6693	0.6417	-0.6524	0.0388	0.0944
metab_5279	pos	236.1638	2.7653	0.4530	-1.1098	0.2960	0.4268
metab_203	pos	236.1750	0.5983	0.2505	-0.5468	0.6250	0.7311
metab_5543	pos	236.1751	2.1290	2.1086	-5.8428	0.0000	0.0001
metab_1601	pos	236.2114	1.5144	0.5892	0.8775	0.1929	0.3089
metab_10816	neg	236.7588	14.0771	0.4804	-0.0936	0.0116	0.0436
metab_7798	neg	236.9324	0.4975	0.3059	-0.7375	0.5955	0.7021
metab_8639	neg	236.9807	2.1153	0.1965	0.0000	0.2937	0.4247
metab_6409	pos	236.9917	0.5560	1.2976	1.7259	0.0171	0.0521
metab_14657	neg	237.0073	0.8946	0.3584	0.6488	0.5565	0.6681
metab_6495	pos	237.0199	0.4999	0.8982	-1.5229	0.0218	0.0620
metab_8213	neg	237.0439	1.3373	1.3894	-1.6949	0.0013	0.0100

metab_5558	pos	237.0608	2.0973	0.0868	0.0885	0.8003	0.8672
metab_7975	neg	237.0614	0.7818	0.3309	0.0878	0.1808	0.2989
metab_5479	pos	237.0752	2.2361	0.9315	0.9219	0.0119	0.0405
metab_5188	pos	237.0752	3.0264	0.4151	-0.2705	0.5656	0.6820
metab_8952	neg	237.0767	2.9092	1.2334	-1.4305	0.0029	0.0168
metab_6401	pos	237.0822	0.5560	0.1622	-0.0519	0.4896	0.6145
metab_276	pos	237.0862	1.1306	0.1336	0.1053	0.8135	0.8770
metab_8075	neg	237.0879	1.0642	0.2292	0.6306	0.6983	0.7835
metab_14201	neg	237.0881	1.5736	0.1186	0.1952	0.5955	0.7021
metab_13292	neg	237.0881	3.3144	0.1389	0.5366	0.7404	0.8130
metab_8992	neg	237.1132	3.0101	1.3538	2.9126	0.0097	0.0391
metab_1561	pos	237.1226	1.4005	0.0397	-0.2862	0.9165	0.9452
metab_2001	pos	237.1228	2.8568	0.0323	0.1403	0.7921	0.8607
metab_7544	neg	237.1244	2.0516	1.1430	-2.1748	0.0490	0.1159
metab_5029	pos	237.1309	3.5923	0.7127	-1.0337	0.1722	0.2844
metab_2005	pos	237.1479	2.8725	1.0281	1.5123	0.0060	0.0250
metab_327	pos	237.1589	1.4569	1.0012	1.4711	0.0190	0.0562
metab_2806	pos	237.2206	7.9407	0.1417	-0.3039	0.5885	0.7010
metab_2828	pos	237.2207	8.1132	0.4517	-0.5817	0.1636	0.2734
metab_10917	neg	237.8881	14.5179	0.3234	0.4268	0.0809	0.1667
metab_8386	neg	238.0181	1.5885	0.6383	-0.4514	0.1945	0.3146
metab_14963	neg	238.0235	0.5286	0.6069	-0.6226	0.0262	0.0759
metab_14570	neg	238.0354	1.0927	1.8066	4.3401	0.0009	0.0076
metab_7509	neg	238.0357	1.8371	1.5509	3.5366	0.0040	0.0210
metab_8367	neg	238.0357	1.5736	0.3293	0.6274	0.4084	0.5357
metab_14339	neg	238.0720	1.4568	0.0568	0.1806	0.6919	0.7791
metab_7487	neg	238.0720	1.7419	0.2397	-0.2459	0.4550	0.5782
metab_8619	neg	238.0720	2.0670	0.7219	0.9255	0.0131	0.0472
metab_6404	pos	238.0770	0.5560	0.4069	-0.2886	0.1522	0.2588
metab_6857	neg	238.0980	1.2663	2.0660	-4.3051	0.0008	0.0074
metab_13544	neg	238.0981	2.7155	1.0053	-0.3845	0.0293	0.0815
metab_5924	pos	238.1067	1.4005	0.7633	-0.8850	0.0018	0.0108
metab_478	pos	238.1068	2.7188	0.7425	-0.5523	0.0471	0.1091
metab_7581	neg	238.1084	2.3078	0.9081	-0.9545	0.0006	0.0064
metab_13236	neg	238.1084	3.5141	0.9043	-1.9470	0.1491	0.2601
metab_283	pos	238.1179	1.1589	0.8076	2.1227	0.1079	0.2004
metab_14488	neg	238.1195	1.2663	0.7233	2.1032	0.2351	0.3605
metab_5830	pos	238.1541	1.5565	1.0560	-1.3380	0.0029	0.0154
metab_1943	pos	238.1796	2.5938	0.8441	0.8657	0.0495	0.1132
metab_5970	pos	238.1907	1.3153	0.8043	-1.0648	0.0449	0.1053
metab_5883	pos	238.1907	1.4714	0.7230	-0.7965	0.1423	0.2464
metab_4121	pos	238.2159	8.1273	0.5869	-0.6615	0.1113	0.2051
metab_10819	neg	238.7563	14.0771	0.3762	0.0258	0.1455	0.2558
metab_3278	pos	238.9065	14.0378	0.0503	0.0547	0.7726	0.8465
metab_14954	neg	238.9812	0.5286	0.7903	-1.1753	0.0487	0.1156
metab_8253	neg	239.0196	1.4267	0.5436	2.7587	0.3313	0.4640
metab_7932	neg	239.0230	0.6131	0.4464	2.7654	0.6324	0.7306
metab_1748	pos	239.0512	1.9609	1.4157	3.6473	0.0004	0.0042

metab_14093	neg	239.0562	1.7264	0.2098	-0.3352	0.5223	0.6402
metab_14586	neg	239.0673	1.0499	0.3211	0.5007	0.4125	0.5389
metab_13261	neg	239.0717	3.4133	2.1404	7.2753	0.0000	0.0000
metab_368	pos	239.0909	1.6838	0.8415	0.8046	0.0002	0.0023
metab_9251	neg	239.0925	3.9005	1.3111	2.1270	0.0000	0.0010
metab_5822	pos	239.1019	1.5705	0.7510	-1.3356	0.0310	0.0805
metab_6123	pos	239.1019	1.0601	0.3822	0.1524	0.1647	0.2747
metab_1101	pos	239.1020	1.1731	0.6453	-0.6906	0.0027	0.0145
metab_2121	pos	239.1020	3.3032	0.0637	0.0057	0.9455	0.9658
metab_8470	neg	239.1036	1.7572	0.2250	-0.1429	0.4432	0.5672
metab_6862	neg	239.1037	1.2663	0.0556	0.2374	0.8997	0.9328
metab_5063	pos	239.1102	3.4702	1.1181	9.8049	0.0342	0.0862
metab_12683	neg	239.1288	5.5639	0.1434	0.0957	0.4255	0.5511
metab_12784	neg	239.1288	5.0159	0.3201	-0.5813	0.6015	0.7062
metab_13373	neg	239.1288	3.1115	1.7439	3.4562	0.0009	0.0076
metab_12847	neg	239.1289	4.7709	0.0853	0.2225	0.9547	0.9705
metab_6352	pos	239.1382	0.6123	2.5543	6.7097	0.0000	0.0000
metab_5046	pos	239.1384	3.5465	0.6022	-1.1914	0.2374	0.3615
metab_409	pos	239.1386	2.0524	0.3412	-0.8550	0.3200	0.4530
metab_3421	pos	239.1482	14.4245	0.2114	-0.1776	0.1261	0.2250
metab_46	pos	239.1489	1.5284	0.3034	-0.2534	0.2807	0.4101
metab_76	pos	239.1635	3.9577	0.8869	1.3774	0.1027	0.1933
metab_12600	neg	239.1651	5.8896	0.0828	-0.6963	0.8910	0.9267
metab_12443	neg	239.1651	6.5673	1.3146	-2.5708	0.0485	0.1153
metab_12961	neg	239.1652	4.3380	1.1061	1.7089	0.0002	0.0029
metab_3041	pos	239.1788	9.3429	0.5490	0.7982	0.1717	0.2838
metab_3528	pos	239.2241	13.9785	0.4172	-0.3061	0.0037	0.0177
metab_7726	neg	239.9035	0.0197	0.2292	0.0313	0.1974	0.3181
metab_13894	neg	239.9971	2.0358	1.2772	4.4734	0.0324	0.0873
metab_1745	pos	240.0322	1.9463	1.7158	-2.9972	0.0002	0.0026
metab_14104	neg	240.0337	1.7104	1.2445	-1.6054	0.0041	0.0210
metab_9042	neg	240.0432	3.1797	0.3428	-0.0728	0.1254	0.2288
metab_8542	neg	240.0513	1.9125	0.0798	0.0029	0.7244	0.8022
metab_8199	neg	240.0513	1.3231	0.0139	0.1254	0.9648	0.9758
metab_13156	neg	240.0665	3.7148	1.0909	-1.9335	0.0429	0.1061
metab_264	pos	240.0859	1.0180	0.7270	0.9034	0.0348	0.0873
metab_13534	neg	240.0877	2.7475	0.7718	0.8310	0.0006	0.0061
metab_13998	neg	240.0877	1.8682	0.1994	-0.8333	0.6271	0.7269
metab_2007	pos	240.1014	2.8725	0.9752	4.3643	0.0825	0.1645
metab_13465	neg	240.1029	2.8768	1.2759	-1.0367	0.0163	0.0550
metab_1110	pos	240.1222	0.9340	2.0321	4.6690	0.0000	0.0002
metab_7717	neg	240.1241	3.4470	0.8715	1.4420	0.0199	0.0626
metab_14089	neg	240.1241	1.7264	1.3837	4.2388	0.0197	0.0622
metab_1531	pos	240.1335	1.2725	1.1311	1.9156	0.0334	0.0847
metab_8667	neg	240.1354	2.1798	1.2297	-4.0643	0.0329	0.0883
metab_1210	pos	240.1551	0.4855	0.5434	-0.7812	0.1806	0.2947
metab_2052	pos	240.1586	3.0422	0.6686	0.5584	0.0262	0.0712
metab_450	pos	240.1587	2.4071	0.2071	-0.1585	0.6321	0.7371

metab_1456	pos	240.1699	1.0883	0.7227	-0.7863	0.0028	0.0150
metab_2009	pos	240.1742	2.8725	0.7992	-2.2991	0.2350	0.3592
metab_625	pos	240.1951	5.0307	0.3679	0.0669	0.1484	0.2544
metab_4515	pos	240.2315	6.1009	0.7354	5.1280	0.2444	0.3690
metab_3009	pos	240.2316	9.1302	0.0004	-0.0960	0.9183	0.9467
metab_2916	pos	240.2316	8.6132	0.3667	-0.3863	0.1043	0.1956
metab_172	pos	240.9523	0.4999	0.8941	-1.5286	0.0146	0.0466
metab_8079	neg	240.9845	1.0785	1.7816	8.7173	0.0041	0.0212
metab_8276	neg	241.0104	1.4568	1.3939	2.7681	0.0005	0.0053
metab_7615	neg	241.0118	0.5431	0.0303	0.1301	0.9420	0.9621
metab_14544	neg	241.0353	1.1796	0.3639	0.5951	0.2859	0.4174
metab_5448	pos	241.0813	2.3133	0.0304	-0.5523	0.9240	0.9510
metab_1666	pos	241.0814	1.6838	1.2079	2.3202	0.0110	0.0383
metab_14888	neg	241.0927	0.5571	1.1174	-1.1157	0.0000	0.0013
metab_2161	pos	241.0965	3.5011	1.4110	4.8703	0.0611	0.1318
metab_5176	pos	241.0966	3.0726	1.0904	-1.1760	0.0230	0.0643
metab_13313	neg	241.0983	3.2637	0.1092	-0.1392	0.8352	0.8844
metab_9201	neg	241.0984	3.7488	0.4233	-0.7554	0.3544	0.4874
metab_13188	neg	241.1081	3.6311	1.1956	1.8503	0.0006	0.0059
metab_13124	neg	241.1081	3.8001	0.3680	-0.4815	0.2684	0.3984
metab_8740	neg	241.1084	2.3533	1.3259	2.5802	0.0009	0.0081
metab_5953	pos	241.1175	1.3437	0.5745	0.5531	0.0316	0.0815
metab_13923	neg	241.1193	2.0055	0.0770	0.7894	0.9864	0.9903
metab_7559	neg	241.1194	2.1630	0.1456	0.6853	0.8768	0.9157
metab_9513	neg	241.1444	5.1456	0.3853	0.4426	0.3844	0.5133
metab_13377	neg	241.1445	3.0942	1.2577	1.9462	0.0038	0.0202
metab_12974	neg	241.1445	4.3037	0.1201	-0.0591	0.8579	0.9018
metab_1408	pos	241.1539	0.9200	1.0940	1.7140	0.0028	0.0151
metab_5353	pos	241.1693	2.5314	1.0053	-1.2481	0.0019	0.0114
metab_9903	neg	241.1808	7.1437	0.2078	-0.4105	0.6431	0.7399
metab_12460	neg	241.1808	6.4709	0.4616	-0.3654	0.3487	0.4815
metab_5085	pos	241.2031	3.3793	0.1938	0.6677	0.5379	0.6581
metab_7365	neg	241.2172	7.8193	1.1747	2.1417	0.0061	0.0279
metab_11993	neg	241.2172	8.0373	0.4222	1.2925	0.3839	0.5131
metab_10299	neg	241.2173	8.7031	0.0293	0.5266	0.8900	0.9261
metab_3265	pos	241.2211	13.9785	0.3069	-0.2611	0.0614	0.1323
metab_3243	pos	241.2211	12.5404	0.3354	-0.2418	0.0573	0.1259
metab_9463	neg	241.7933	4.8210	0.3877	0.0120	0.3469	0.4798
metab_10939	neg	241.8832	14.2554	0.3952	-0.0819	0.0390	0.0989
metab_7780	neg	241.8840	0.4474	0.2890	-0.0570	0.1437	0.2535
metab_10920	neg	241.8988	14.5008	0.5547	-0.2039	0.0022	0.0139
metab_15146	neg	241.8988	0.0197	0.2870	-0.0074	0.1075	0.2049
metab_10996	neg	241.9464	14.0927	0.3047	-0.0856	0.1157	0.2153
metab_14005	neg	241.9943	1.8524	0.4716	0.0682	0.3963	0.5241
metab_167	pos	241.9989	0.4999	0.8907	-1.4539	0.0143	0.0459
metab_8022	neg	242.0069	0.8946	0.6702	3.3408	0.2414	0.3676
metab_1360	pos	242.0417	0.7801	0.8751	-1.2044	0.1117	0.2056
metab_6169	pos	242.0652	0.9340	0.9708	1.7268	0.0254	0.0696

metab_14374	neg	242.0670	1.4115	0.2919	-0.3355	0.3967	0.5246
metab_14896	neg	242.0796	0.5571	1.0830	1.7288	0.0156	0.0534
metab_10755	neg	242.0798	14.0117	1.2664	1.9627	0.0016	0.0116
metab_2208	pos	242.0805	3.7148	1.3331	-2.7680	0.0204	0.0590
metab_14166	neg	242.0807	1.6028	1.3348	2.3385	0.0006	0.0064
metab_13000	neg	242.0824	4.2203	0.9722	-1.2290	0.0070	0.0308
metab_13267	neg	242.0825	3.3803	0.4436	-0.4622	0.1746	0.2915
metab_8099	neg	242.0862	1.1355	0.7505	0.8456	0.3636	0.4958
metab_169	pos	242.1015	0.1727	0.0920	-0.0649	0.4570	0.5859
metab_1652	pos	242.1017	1.6413	1.0084	1.0256	0.0032	0.0164
metab_8787	neg	242.1034	2.4651	0.6151	-0.7359	0.0916	0.1820
metab_6113	pos	242.1127	1.0741	0.8067	-0.7757	0.0624	0.1338
metab_8053	neg	242.1145	1.0074	1.9306	6.6717	0.0000	0.0001
metab_5754	pos	242.1169	1.6983	1.3724	1.9957	0.0000	0.0006
metab_14099	neg	242.1227	1.7104	0.2489	0.1342	0.5780	0.6870
metab_12754	neg	242.1397	5.1772	0.8495	-1.7141	0.1609	0.2755
metab_13134	neg	242.1398	3.7658	0.4574	0.4693	0.5014	0.6218
metab_5904	pos	242.1492	1.4429	0.7136	1.2132	0.1302	0.2306
metab_74	pos	242.1744	3.5162	0.9194	0.8303	0.0003	0.0038
metab_2115	pos	242.1744	3.2880	1.0009	1.0095	0.0005	0.0046
metab_12896	neg	242.1761	4.6056	0.1002	0.1347	0.6823	0.7716
metab_9025	neg	242.1762	3.1115	0.4660	-0.9140	0.3706	0.5020
metab_13318	neg	242.1762	3.2473	0.6768	-1.1881	0.1896	0.3088
metab_1154	pos	242.1856	0.5280	0.1745	-0.1135	0.4611	0.5890
metab_4648	pos	242.2107	5.3608	0.1172	-0.3917	0.7800	0.8517
metab_2414	pos	242.2107	5.0163	0.1437	-0.1455	0.4973	0.6206
metab_4113	pos	242.2471	8.1707	0.1954	-0.2117	0.4084	0.5412
metab_7629	neg	242.9399	0.5286	1.1165	-1.1305	0.0395	0.1000
metab_6643	neg	242.9435	14.0438	0.3403	-0.0487	0.0532	0.1227
metab_3304	pos	243.0186	14.0948	0.6481	-0.5934	0.0004	0.0043
metab_14332	neg	243.0335	1.4568	0.9873	1.1864	0.0015	0.0111
metab_6902	neg	243.0511	1.4568	1.2889	2.3725	0.0012	0.0096
metab_7976	neg	243.0621	0.7818	0.9230	-0.9204	0.0014	0.0106
metab_6336	pos	243.0734	0.6123	1.0354	2.5086	0.0128	0.0424
metab_13444	neg	243.0773	2.9429	0.3335	-0.5683	0.3531	0.4862
metab_13655	neg	243.0774	2.4970	1.0136	9.5089	0.0450	0.1095
metab_13972	neg	243.0810	1.9125	0.5405	-0.7127	0.2127	0.3357
metab_5250	pos	243.0871	2.8568	0.3712	0.4260	0.1295	0.2296
metab_7616	neg	243.0874	2.5592	0.8113	1.0819	0.0970	0.1900
metab_14287	neg	243.0985	1.5012	0.1600	0.0135	0.4819	0.6039
metab_3352	pos	243.1063	14.4865	0.1547	-0.1750	0.3762	0.5096
metab_1020	pos	243.1123	2.1132	0.0706	-0.0116	0.9455	0.9658
metab_8509	neg	243.1138	1.8205	0.2842	-0.0781	0.5408	0.6549
metab_13450	neg	243.1144	2.9263	1.5264	-2.5699	0.0021	0.0134
metab_8875	neg	243.1238	2.7155	0.0495	0.1754	0.9735	0.9812
metab_9085	neg	243.1238	3.3304	0.8044	1.0602	0.0498	0.1173
metab_13154	neg	243.1238	3.7317	0.3698	0.3600	0.5690	0.6797
metab_331	pos	243.1331	1.4860	0.4943	0.3555	0.1079	0.2004

metab_58	pos	243.1334	2.1595	0.0601	0.1191	0.7587	0.8351
metab_1762	pos	243.1334	2.0062	0.1869	0.6197	0.7948	0.8629
metab_14136	neg	243.1348	1.6491	0.6207	1.5887	0.2330	0.3582
metab_8738	neg	243.1349	2.3533	0.1587	-0.1321	0.7936	0.8521
metab_14067	neg	243.1349	1.7572	0.4602	-1.3799	0.5076	0.6271
metab_7546	neg	243.1350	2.0670	0.3359	0.9917	0.5653	0.6763
metab_14549	neg	243.1462	1.1503	1.1930	10.6327	0.0302	0.0831
metab_4879	pos	243.1486	4.2750	0.3388	0.7309	0.4661	0.5935
metab_2254	pos	243.1486	3.9722	1.0479	3.0241	0.0580	0.1269
metab_12707	neg	243.1601	5.4355	0.8477	1.4592	0.0421	0.1046
metab_9018	neg	243.1602	3.0774	0.8808	2.0437	0.1592	0.2733
metab_5799	pos	243.1694	1.6128	1.4233	12.1486	0.0079	0.0305
metab_5869	pos	243.1695	1.5004	0.9075	11.6028	0.0635	0.1357
metab_382	pos	243.1700	1.7993	1.5543	12.3615	0.0038	0.0180
metab_13997	neg	243.1713	1.8682	0.6230	-0.7699	0.3461	0.4792
metab_13879	neg	243.1714	2.0670	0.3211	-0.7608	0.5291	0.6457
metab_13804	neg	243.1714	2.2119	0.2840	-0.8528	0.5527	0.6646
metab_5959	pos	243.1808	1.3293	1.4011	12.7954	0.0017	0.0107
metab_4709	pos	243.1949	5.0163	0.0379	-0.1274	0.8566	0.9079
metab_12084	neg	243.1965	7.7567	0.3733	0.0139	0.4395	0.5634
metab_2485	pos	243.2099	5.5260	1.1465	-1.8476	0.0168	0.0512
metab_3058	pos	243.2100	9.4667	0.5107	0.1708	0.3867	0.5189
metab_4594	pos	243.2100	5.6934	0.8660	-0.7810	0.1252	0.2238
metab_3689	pos	243.2100	9.9307	0.1145	-0.0284	0.7600	0.8361
metab_2433	pos	243.2100	5.1372	1.1732	4.7224	0.0502	0.1144
metab_2725	pos	243.2100	7.2763	0.8702	-0.8548	0.1372	0.2398
metab_3758	pos	243.2100	9.6968	0.5870	-0.5540	0.1032	0.1939
metab_7758	neg	243.8991	0.0444	0.1898	0.0488	0.3063	0.4374
metab_7183	neg	243.8992	14.1090	0.2044	0.0531	0.2678	0.3981
metab_9418	neg	243.9316	4.6216	0.1922	0.5148	0.3602	0.4931
metab_10719	neg	243.9529	13.8980	0.2469	0.0625	0.1557	0.2689
metab_13846	neg	244.0614	2.1311	0.4498	-0.3279	0.1084	0.2061
metab_8758	neg	244.0614	2.4010	0.4782	-0.3180	0.1301	0.2349
metab_1568	pos	244.0806	1.4146	0.2105	-0.3648	0.6687	0.7660
metab_6088	pos	244.0808	1.1306	0.5764	0.9921	0.2182	0.3403
metab_8629	neg	244.0826	2.0987	0.0876	0.1181	0.8348	0.8841
metab_6877	neg	244.0827	1.3231	0.3210	-0.3349	0.2909	0.4221
metab_8911	neg	244.0827	2.7961	0.3919	0.8984	0.5103	0.6294
metab_54	pos	244.0964	1.9309	0.0347	-0.1726	0.9976	0.9986
metab_7658	neg	244.0979	2.8768	0.4118	-0.5136	0.2686	0.3985
metab_13026	neg	244.0979	4.1193	0.9907	-1.2558	0.0232	0.0698
metab_6371	pos	244.1108	0.5983	0.1357	-0.1006	0.6536	0.7542
metab_1512	pos	244.1172	1.2306	0.2383	0.0328	0.3313	0.4659
metab_454	pos	244.1173	2.4530	1.1206	-1.8098	0.0002	0.0029
metab_14288	neg	244.1190	1.5012	1.1958	1.7986	0.0001	0.0020
metab_8732	neg	244.1191	2.3380	0.3902	0.7543	0.3801	0.5099
metab_13579	neg	244.1269	2.6524	0.7974	-0.7699	0.1062	0.2030
metab_1117	pos	244.1284	0.7801	1.2751	2.2571	0.0009	0.0070

metab_8607	neg	244.1300	2.0358	2.0101	-8.2606	0.0009	0.0076
metab_14353	neg	244.1301	1.4427	0.6267	0.9185	0.1347	0.2417
metab_8024	neg	244.1301	0.9086	0.4903	2.6753	0.5082	0.6276
metab_2119	pos	244.1327	3.3032	0.8364	-0.6987	0.1074	0.2000
metab_49	pos	244.1536	1.6838	0.5813	-0.6378	0.1568	0.2646
metab_14131	neg	244.1553	1.6642	0.2296	1.0286	0.6815	0.7711
metab_12919	neg	244.1554	4.5391	0.6126	-0.4708	0.2658	0.3959
metab_13139	neg	244.1554	3.7658	0.4008	1.7006	0.6027	0.7072
metab_12892	neg	244.1823	4.6056	0.1165	0.2161	0.5407	0.6549
metab_5270	pos	244.1901	2.7959	0.4041	0.2084	0.2938	0.4243
metab_5126	pos	244.1901	3.2420	0.3701	-0.8828	0.5106	0.6325
metab_5775	pos	244.2550	1.6553	0.5733	-0.5935	0.2207	0.3431
metab_14916	neg	244.9109	0.5431	1.2997	-1.7360	0.0003	0.0040
metab_1215	pos	244.9462	0.4855	0.9102	-1.0031	0.0003	0.0035
metab_15018	neg	244.9640	0.5126	0.8785	-1.1339	0.0153	0.0527
metab_13587	neg	244.9859	2.6357	1.0837	4.2206	0.1234	0.2258
metab_3313	pos	244.9974	14.1389	0.3861	-0.2926	0.0062	0.0255
metab_4628	pos	244.9974	5.4809	0.6319	-0.6061	0.0181	0.0541
metab_2378	pos	244.9974	4.7302	0.5785	-0.5242	0.0178	0.0535
metab_4807	pos	244.9974	4.5785	0.5186	-0.4026	0.0047	0.0209
metab_2320	pos	244.9974	4.3818	0.4523	-0.3841	0.0557	0.1235
metab_5107	pos	244.9974	3.3032	0.3266	-0.3122	0.1243	0.2230
metab_4759	pos	244.9974	4.8359	0.5197	-0.4069	0.0604	0.1307
metab_4921	pos	244.9975	4.0479	0.7147	-0.7660	0.0035	0.0174
metab_14650	neg	245.0301	0.9086	0.7574	2.0743	0.1860	0.3047
metab_14918	neg	245.0337	0.5431	2.2563	4.4123	0.0042	0.0215
metab_14922	neg	245.0431	0.5431	0.0599	0.0518	0.7073	0.7906
metab_8523	neg	245.0577	1.8682	1.5864	-3.1012	0.0003	0.0037
metab_15016	neg	245.0629	0.5126	0.4619	0.9257	0.2625	0.3924
metab_6594	neg	245.0667	1.4568	0.6170	0.7303	0.0667	0.1448
metab_1921	pos	245.0803	2.5003	0.5909	0.9625	0.2496	0.3751
metab_8975	neg	245.0821	2.9760	0.5360	-1.1043	0.5917	0.6988
metab_5800	pos	245.0912	1.6128	0.8036	0.8055	0.0067	0.0272
metab_7622	neg	245.0931	2.5893	0.0973	0.5039	0.9059	0.9376
metab_14052	neg	245.0933	1.7729	0.3912	-0.2393	0.3258	0.4580
metab_6066	pos	245.0949	1.1731	0.7286	0.8424	0.0094	0.0344
metab_13739	neg	245.1030	2.3227	1.1492	1.9488	0.0023	0.0145
metab_14627	neg	245.1142	0.9649	0.0921	0.3449	0.7631	0.8298
metab_14279	neg	245.1143	1.5155	0.2128	-0.0985	0.4591	0.5818
metab_8124	neg	245.1143	1.2084	0.1334	0.0150	0.6143	0.7174
metab_1838	pos	245.1278	2.2209	0.2845	-0.4127	0.3684	0.5024
metab_5897	pos	245.1281	1.4429	1.2989	11.5617	0.0023	0.0131
metab_2010	pos	245.1376	2.8877	0.1810	-0.5832	0.5351	0.6557
metab_5319	pos	245.1377	2.6244	0.0612	-0.2040	0.6430	0.7457
metab_13322	neg	245.1394	3.2473	1.2063	2.6141	0.0210	0.0651
metab_9275	neg	245.1395	4.0182	0.3615	1.0828	0.5684	0.6792
metab_12886	neg	245.1395	4.6216	0.5008	0.5274	0.5356	0.6509
metab_1877	pos	245.1489	2.3600	0.2616	-0.4857	0.5012	0.6240

metab_1789	pos	245.1491	2.0828	0.3049	0.0963	0.5570	0.6746
metab_6084	pos	245.1601	1.1447	1.7720	3.8043	0.0008	0.0064
metab_1616	pos	245.1851	1.5284	0.5685	2.0212	0.3624	0.4963
metab_5376	pos	245.1854	2.4850	0.9239	1.2282	0.0222	0.0629
metab_5492	pos	245.1854	2.2209	0.2336	-0.9587	0.5773	0.6909
metab_1782	pos	245.1854	2.0677	0.2325	-0.8097	0.6118	0.7209
metab_3134	pos	245.2257	9.9307	0.0599	0.3635	0.9717	0.9832
metab_666	pos	245.2257	5.9949	0.3297	2.4658	0.5578	0.6749
metab_4378	pos	245.2257	6.9035	0.0087	0.0112	0.9654	0.9788
metab_3762	pos	245.2258	9.6823	0.1007	0.3815	0.9079	0.9410
metab_1166	pos	245.2329	0.5140	0.4426	-0.2732	0.3455	0.4803
metab_10729	neg	245.8946	13.9301	0.2525	0.0290	0.2712	0.4018
metab_7759	neg	245.8947	0.0589	0.1729	0.0572	0.3475	0.4804
metab_10827	neg	245.8947	14.1090	0.1196	0.0849	0.5291	0.6457
metab_7446	neg	246.0443	1.5439	1.4129	-2.1187	0.0032	0.0178
metab_13964	neg	246.0444	1.9280	1.0810	-1.5579	0.0322	0.0869
metab_6795	neg	246.0619	0.8662	0.5154	-0.2926	0.2053	0.3273
metab_1714	pos	246.0661	1.8134	0.9798	-1.3823	0.0285	0.0758
metab_8591	neg	246.0774	2.0055	0.8092	-2.2872	0.1280	0.2322
metab_6534	pos	246.0893	0.4693	0.6872	0.9081	0.0647	0.1376
metab_13985	neg	246.0983	1.8833	0.1469	0.1250	0.6232	0.7236
metab_13778	neg	246.0983	2.2595	0.2338	-0.1133	0.5122	0.6312
metab_14206	neg	246.0983	1.5736	0.8577	-1.0143	0.0670	0.1453
metab_6745	neg	246.1094	0.5991	0.5227	1.9480	0.4354	0.5591
metab_5229	pos	246.1119	2.9192	0.4184	-0.6052	0.2433	0.3680
metab_5374	pos	246.1119	2.4850	0.6846	-0.9056	0.0353	0.0880
metab_5127	pos	246.1119	3.2420	1.1957	-2.7337	0.0151	0.0478
metab_6951	neg	246.1136	4.2203	0.5216	0.8535	0.3896	0.5182
metab_9273	neg	246.1136	4.0020	0.9862	-1.2661	0.0879	0.1770
metab_13281	neg	246.1137	3.3304	0.3557	0.0953	0.3310	0.4639
metab_1359	pos	246.1329	0.7801	0.3587	-0.5667	0.1803	0.2944
metab_8595	neg	246.1346	2.0202	0.7283	1.5516	0.0975	0.1906
metab_245	pos	246.1439	0.8920	0.0140	0.2290	0.9497	0.9676
metab_7828	neg	246.1459	0.5286	0.1290	0.9381	0.7763	0.8402
metab_1657	pos	246.1590	1.6553	0.5402	-0.5898	0.1887	0.3041
metab_1973	pos	246.1596	2.7341	0.6600	1.3838	0.2235	0.3463
metab_384	pos	246.1692	1.8277	1.0007	-1.4140	0.0335	0.0848
metab_7505	neg	246.1711	1.8205	1.1681	-1.5549	0.0072	0.0317
metab_2398	pos	246.2421	4.9267	0.2504	-0.0820	0.2917	0.4219
metab_5687	pos	246.2725	1.8277	1.0496	-1.5457	0.0454	0.1062
metab_10905	neg	246.8476	15.7464	0.5033	-0.1828	0.0046	0.0229
metab_15064	neg	246.8476	0.4134	0.4008	-0.1478	0.0380	0.0975
metab_10840	neg	246.9107	14.1424	0.3588	-0.0475	0.0915	0.1818
metab_13578	neg	246.9829	2.6524	1.4141	6.1361	0.0936	0.1848
metab_8984	neg	247.0016	2.9931	1.4246	4.3266	0.0207	0.0645
metab_1351	pos	247.0207	0.7661	0.3174	0.9595	0.5767	0.6907
metab_7626	neg	247.0612	2.6357	1.0238	-2.9784	0.1002	0.1944
metab_13725	neg	247.0613	2.3533	0.0596	-0.1342	0.9981	0.9985

metab_9089	neg	247.0613	3.3304	1.5074	-4.5370	0.0105	0.0409
metab_8512	neg	247.0725	1.8371	1.3063	2.8619	0.0315	0.0856
metab_9039	neg	247.0811	3.1626	1.0922	4.3880	0.1234	0.2258
metab_13492	neg	247.0816	2.8269	0.1405	2.3715	0.7147	0.7956
metab_1829	pos	247.0958	2.1746	0.9180	-0.8498	0.0476	0.1098
metab_9414	neg	247.0976	4.6056	1.3040	-5.0219	0.0448	0.1091
metab_13310	neg	247.0977	3.2801	0.8346	-1.9647	0.2043	0.3260
metab_5878	pos	247.1069	1.4860	0.4322	0.4958	0.3077	0.4399
metab_1949	pos	247.1071	2.6090	0.1663	0.1856	0.6416	0.7446
metab_8805	neg	247.1085	2.5113	0.6510	-0.2530	0.1462	0.2568
metab_5616	pos	247.1169	2.0062	1.3558	2.3791	0.0002	0.0030
metab_13705	neg	247.1187	2.4010	0.8264	1.0605	0.1084	0.2061
metab_289	pos	247.1281	1.2016	0.1056	-0.0369	0.7889	0.8582
metab_5340	pos	247.1433	2.5627	0.1301	-0.7905	0.7626	0.8383
metab_2004	pos	247.1434	2.8568	0.1896	-0.7441	0.6979	0.7892
metab_8802	neg	247.1551	2.5113	0.8008	0.8898	0.1802	0.2980
metab_13305	neg	247.1551	3.2972	0.5519	2.5452	0.5049	0.6245
metab_5790	pos	247.1645	1.6272	0.3614	0.6059	0.2817	0.4111
metab_6369	pos	247.2009	0.5983	0.7411	-5.2017	0.3620	0.4960
metab_10991	neg	247.9625	14.1090	0.3930	-0.0747	0.0338	0.0898
metab_14378	neg	248.0057	1.3965	2.2598	-5.3493	0.0000	0.0005
metab_14150	neg	248.0600	1.6335	1.4330	-2.7212	0.0007	0.0068
metab_14641	neg	248.0775	0.9368	0.3450	-0.1673	0.1743	0.2913
metab_8795	neg	248.0928	2.4970	0.7881	-1.8241	0.1687	0.2851
metab_7666	neg	248.0928	2.9760	0.3968	-0.4090	0.2944	0.4253
metab_14469	neg	248.0928	1.2805	0.8476	-0.9077	0.0108	0.0416
metab_1465	pos	248.1024	1.1164	0.3068	1.0025	0.5518	0.6697
metab_1723	pos	248.1119	1.8729	0.1064	-0.1410	0.6861	0.7799
metab_37	pos	248.1121	0.6262	0.0913	0.2210	0.7824	0.7912
metab_437	pos	248.1122	2.2665	0.2524	-0.3647	0.4918	0.6159
metab_14451	neg	248.1139	1.2948	0.1124	0.3292	0.6256	0.7257
metab_8085	neg	248.1140	1.0927	0.6959	1.2108	0.0506	0.1184
metab_8469	neg	248.1140	1.7572	0.0314	0.1456	0.7823	0.8446
metab_2291	pos	248.1274	4.2150	0.6042	-0.0037	0.2900	0.4204
metab_5200	pos	248.1275	2.9954	0.9432	-1.2874	0.0151	0.0478
metab_13109	neg	248.1292	3.8333	0.8217	-1.3632	0.0618	0.1373
metab_6945	neg	248.1293	4.1869	0.2094	0.4147	0.6651	0.7585
metab_1626	pos	248.1385	1.5565	1.8124	2.4805	0.0445	0.1045
metab_5269	pos	248.1387	2.8108	0.5561	-0.3121	0.3888	0.5211
metab_256	pos	248.1484	0.9480	0.7429	-1.5236	0.0810	0.1625
metab_5618	pos	248.1485	2.0062	0.8933	1.5559	0.0286	0.0759
metab_1886	pos	248.1638	2.3751	0.1648	-0.9263	0.6685	0.7659
metab_4819	pos	248.1639	4.5335	0.5874	0.4081	0.0825	0.1645
metab_5689	pos	248.1744	1.8277	1.0152	-1.4474	0.0359	0.0891
metab_4735	pos	248.1820	4.9267	0.9432	1.8609	0.0357	0.0887
metab_1344	pos	248.8956	0.6962	0.1669	-0.0753	0.7654	0.8402
metab_6707	neg	248.9059	0.5286	1.2287	-1.5390	0.0005	0.0057
metab_10975	neg	248.9077	14.1424	0.3120	-0.0227	0.0826	0.1688

metab_10699	neg	248.9217	11.5359	0.1885	0.0920	0.2283	0.3533
metab_11178	neg	248.9217	11.1796	0.3409	-0.0455	0.0799	0.1652
metab_11161	neg	248.9217	11.8118	0.3172	-0.0249	0.0881	0.1772
metab_11130	neg	248.9217	13.9301	0.2528	0.0494	0.1855	0.3041
metab_6422	pos	248.9320	0.5420	0.2299	0.2118	0.6812	0.7764
metab_6645	neg	248.9605	14.0282	0.2804	-0.0031	0.2254	0.3501
metab_1290	pos	249.0363	0.5843	1.1769	-2.8612	0.0155	0.0485
metab_14269	neg	249.0385	1.5155	1.1776	-1.1679	0.0014	0.0103
metab_14078	neg	249.0403	1.7419	0.6082	1.0742	0.1483	0.2594
metab_14228	neg	249.0432	1.5439	1.3238	-1.4272	0.0017	0.0119
metab_8066	neg	249.0616	1.0357	0.5746	-0.1051	0.1467	0.2573
metab_14487	neg	249.0616	1.2663	0.4911	-0.3278	0.0757	0.1583
metab_14517	neg	249.0703	1.2236	1.2777	3.3783	0.1933	0.3133
metab_1798	pos	249.0749	2.0973	1.4779	3.4493	0.0038	0.0180
metab_5759	pos	249.0862	1.6838	1.7463	6.2397	0.0007	0.0062
metab_7480	neg	249.0882	1.6797	0.3278	4.0291	0.7903	0.8497
metab_9485	neg	249.1132	4.9349	0.2801	0.0196	0.0935	0.1848
metab_1717	pos	249.1225	1.8277	0.1798	-0.5066	0.5047	0.6272
metab_5865	pos	249.1225	1.5144	0.4026	0.4844	0.2877	0.4183
metab_14047	neg	249.1244	1.7885	0.5705	1.6415	0.1917	0.3114
metab_13858	neg	249.1246	2.0987	0.0313	0.5890	0.9456	0.9644
metab_1744	pos	249.1324	1.9463	1.7749	4.9626	0.0000	0.0001
metab_14446	neg	249.1344	1.2948	1.2361	5.0218	0.0062	0.0285
metab_2636	pos	249.1478	6.8433	1.9381	5.5025	0.0000	0.0001
metab_631	pos	249.1478	5.1372	0.1373	0.5626	0.7561	0.8331
metab_637	pos	249.1479	5.3165	1.0559	1.7193	0.0029	0.0154
metab_5149	pos	249.1479	3.1799	0.2431	0.2801	0.6079	0.7177
metab_5866	pos	249.1590	1.5004	1.3860	3.6393	0.0009	0.0069
metab_4188	pos	249.1630	7.8248	0.4189	0.5315	0.3980	0.5311
metab_785	pos	249.1631	8.5835	0.1642	-0.0381	0.7509	0.8292
metab_3854	pos	249.1631	9.2504	0.6375	0.4194	0.0177	0.0533
metab_3716	pos	249.1631	9.8522	0.9196	0.9747	0.0022	0.0128
metab_3788	pos	249.1631	9.5594	0.9887	1.0740	0.0014	0.0093
metab_27	pos	249.1842	5.6623	0.6278	2.9613	0.4170	0.5489
metab_4471	pos	249.2205	6.3885	0.0392	-0.2637	0.9936	0.9960
metab_638	pos	249.2205	5.3454	0.1633	-0.4450	0.7885	0.8578
metab_2716	pos	249.2205	7.2458	0.0409	-0.3183	0.8640	0.9132
metab_4192	pos	249.2205	7.7946	1.3241	2.9133	0.0028	0.0151
metab_4725	pos	249.2206	4.9413	0.1090	-0.3633	0.8429	0.8982
metab_15077	neg	249.8488	0.1093	0.3455	-0.0422	0.0457	0.1106
metab_10951	neg	249.8488	14.1904	0.4347	-0.1211	0.0207	0.0645
metab_7767	neg	249.9068	0.1425	0.2544	0.0259	0.1226	0.2247
metab_10911	neg	249.9068	14.8664	0.4763	-0.0902	0.0082	0.0346
metab_10889	neg	249.9068	14.5349	0.3343	-0.0486	0.0817	0.1676
metab_10977	neg	249.9781	14.1424	0.3843	-0.0650	0.0377	0.0969
metab_9444	neg	249.9782	4.7546	0.4277	-0.0917	0.0499	0.1173
metab_13635	neg	249.9938	2.5264	1.4559	-2.2198	0.0002	0.0029
metab_8819	neg	250.0332	2.5431	1.6141	-2.5709	0.0000	0.0006

metab_7461	neg	250.0721	1.5885	0.1466	0.1481	0.5388	0.6534
metab_7538	neg	250.0722	2.0202	0.5457	3.1388	0.4663	0.5887
metab_1370	pos	250.0914	0.8081	0.9865	-1.8572	0.0606	0.1310
metab_14852	neg	250.0931	0.5851	0.8491	-1.6872	0.1560	0.2693
metab_506	pos	250.1066	3.0264	0.2841	-0.5150	0.4362	0.5672
metab_434	pos	250.1067	2.2824	0.1020	0.0960	0.8855	0.9275
metab_7597	neg	250.1085	2.4175	0.5517	-0.3278	0.0627	0.1387
metab_13453	neg	250.1086	2.9263	1.0191	-0.9606	0.0012	0.0096
metab_13812	neg	250.1086	2.1956	0.7282	-0.8115	0.0190	0.0607
metab_9109	neg	250.1086	3.4303	0.1309	0.7257	0.5972	0.7035
metab_14051	neg	250.1086	1.7729	1.0137	-1.6180	0.0384	0.0982
metab_8298	neg	250.1086	1.4710	0.4325	-0.5521	0.3098	0.4407
metab_14449	neg	250.1197	1.2948	0.1582	2.1414	0.9323	0.9560
metab_13010	neg	250.1356	4.1869	0.7264	1.9246	0.3874	0.5163
metab_4896	pos	250.1432	4.1853	0.4023	-0.1835	0.4461	0.5762
metab_12284	neg	250.1449	7.0959	0.0897	0.1305	0.6426	0.7394
metab_14026	neg	250.1449	1.8205	1.2105	-3.6625	0.0634	0.1399
metab_5966	pos	250.1641	1.3293	1.5857	-3.4370	0.0009	0.0069
metab_4751	pos	250.1770	4.8809	0.2016	-0.2010	0.2923	0.4227
metab_2368	pos	250.1796	4.6700	1.4678	2.7974	0.0007	0.0061
metab_9920	neg	250.1813	7.2225	0.3437	-1.1794	0.6067	0.7105
metab_6398	pos	250.8070	0.5560	0.9284	-1.3371	0.0182	0.0543
metab_14179	neg	251.0199	1.5885	0.7134	1.6280	0.2877	0.4194
metab_198	pos	251.0521	0.5703	1.4966	-2.5577	0.0003	0.0038
metab_14310	neg	251.0560	1.4710	1.1627	1.8220	0.0007	0.0068
metab_8930	neg	251.0563	2.8440	0.9960	-2.0051	0.0540	0.1242
metab_13615	neg	251.0595	2.5748	1.1759	4.4209	0.0887	0.1781
metab_6421	pos	251.0603	0.5420	0.9299	-1.9415	0.1080	0.2006
metab_6811	neg	251.0772	0.9649	1.0949	-0.9962	0.0000	0.0002
metab_5289	pos	251.0908	2.7188	0.7220	0.8197	0.1204	0.2175
metab_9220	neg	251.0961	3.8001	0.4865	1.7808	0.5410	0.6549
metab_366	pos	251.1017	1.6983	0.5739	3.4737	0.4335	0.5647
metab_14040	neg	251.1037	1.8046	0.9350	1.5623	0.0190	0.0609
metab_8570	neg	251.1037	1.9745	0.5613	1.2545	0.1870	0.3058
metab_9282	neg	251.1037	4.0347	0.2348	0.0587	0.6301	0.7289
metab_14255	neg	251.1038	1.5297	0.0698	0.2235	0.7528	0.8228
metab_6828	neg	251.1135	0.9368	1.4418	2.5724	0.0000	0.0006
metab_14387	neg	251.1148	1.3817	1.0523	1.6222	0.0004	0.0049
metab_520	pos	251.1271	3.2420	1.2644	2.1352	0.0009	0.0069
metab_5707	pos	251.1381	1.7845	1.0842	2.0378	0.0091	0.0336
metab_347	pos	251.1382	1.5705	0.7712	1.2765	0.0420	0.1004
metab_612	pos	251.1383	2.2209	0.5590	0.6380	0.0597	0.1297
metab_6496	pos	251.1594	0.4999	1.1131	-1.8545	0.0459	0.1070
metab_617	pos	251.1635	4.8809	0.9200	1.0940	0.0006	0.0057
metab_4862	pos	251.1635	4.3512	1.3413	5.3058	0.0853	0.1687
metab_521	pos	251.1636	2.4530	1.3622	2.5587	0.0011	0.0081
metab_5333	pos	251.1746	2.5782	0.2216	0.5843	0.7485	0.8285
metab_5572	pos	251.1746	2.0828	0.7651	-1.3658	0.0744	0.1523

metab_3707	pos	251.1788	9.8836	0.9221	1.0195	0.0026	0.0141
metab_4969	pos	251.1845	3.8511	0.2721	-0.2432	0.1681	0.2789
metab_5101	pos	251.1846	3.3337	0.3540	-0.2729	0.0451	0.1058
metab_1948	pos	251.1846	2.6090	0.7472	1.3024	0.1115	0.2053
metab_4657	pos	251.1997	5.3311	0.2007	2.0364	0.7396	0.8212
metab_2073	pos	251.2111	3.1346	0.1904	-0.2396	0.4556	0.5846
metab_2586	pos	251.2361	6.3885	0.5475	0.8733	0.2455	0.3700
metab_3090	pos	251.2362	9.6968	0.7826	-0.5697	0.0646	0.1375
metab_3688	pos	251.2362	9.9307	0.7060	1.2084	0.1397	0.2431
metab_6689	neg	251.8458	0.1093	0.3341	-0.0297	0.0550	0.1260
metab_10866	neg	251.8458	14.2390	0.4131	-0.0999	0.0293	0.0815
metab_14571	neg	252.0514	1.0927	0.2524	0.1673	0.5092	0.6287
metab_7642	neg	252.0515	2.7155	1.6356	4.2523	0.0028	0.0163
metab_3377	pos	252.0702	15.9306	0.2619	-0.2209	0.0882	0.1730
metab_6545	pos	252.0702	0.2886	0.3103	-0.2214	0.0173	0.0523
metab_1518	pos	252.0719	1.2446	0.9014	-1.5326	0.0147	0.0468
metab_55	pos	252.0858	2.0211	0.7851	2.3496	0.1465	0.2517
metab_5922	pos	252.0858	1.4005	0.7926	1.6965	0.0717	0.1487
metab_14144	neg	252.0878	1.6491	0.6297	0.0022	0.0715	0.1519
metab_6247	pos	252.0972	0.7801	0.1874	0.9450	0.7393	0.8211
metab_1376	pos	252.1073	0.8221	1.1905	-1.9058	0.0992	0.1883
metab_1706	pos	252.1222	1.7845	0.5930	-1.1690	0.1136	0.2080
metab_9124	neg	252.1243	3.4969	1.2116	2.3377	0.0339	0.0898
metab_6049	pos	252.1334	1.1875	0.7772	2.6647	0.0915	0.1778
metab_1574	pos	252.1585	1.4289	2.1299	1.7272	0.0209	0.0602
metab_2029	pos	252.1589	2.9652	0.2859	-0.4822	0.3783	0.5113
metab_2190	pos	252.1740	3.6535	0.6195	0.4609	0.0513	0.1161
metab_15110	neg	252.8295	0.0197	0.1578	-0.0069	0.4438	0.5676
metab_10937	neg	252.9163	14.2554	0.3384	-0.0228	0.0675	0.1461
metab_7472	neg	253.0354	1.6642	1.7071	3.6787	0.0001	0.0018
metab_6143	pos	253.0560	1.0040	1.3630	3.8763	0.0136	0.0443
metab_9276	neg	253.0620	4.0182	0.4692	-0.3232	0.2947	0.4254
metab_13385	neg	253.0620	3.0774	0.8112	-0.5563	0.2983	0.4288
metab_1998	pos	253.0700	2.8411	1.1725	-3.7727	0.0355	0.0884
metab_13688	neg	253.0717	2.4338	0.7058	-0.7238	0.0144	0.0505
metab_6838	neg	253.0830	1.1645	0.0739	0.1389	0.7306	0.8061
metab_13547	neg	253.0830	2.7155	0.4511	0.0218	0.3660	0.4980
metab_8702	neg	253.1082	2.2754	0.5704	1.7465	0.4330	0.5573
metab_301	pos	253.1176	1.3013	0.9553	1.1659	0.0014	0.0093
metab_1060	pos	253.1176	1.5004	0.2899	0.1137	0.2601	0.3877
metab_13886	neg	253.1195	2.0516	0.2093	-0.2275	0.6156	0.7185
metab_8246	neg	253.1195	1.4115	0.4640	-0.7148	0.2892	0.4207
metab_315	pos	253.1287	1.3720	0.7942	0.6097	0.0424	0.1009
metab_144	pos	253.1427	4.2301	0.1455	0.3175	0.7321	0.8149
metab_2453	pos	253.1428	5.3014	0.2286	0.5995	0.7525	0.8306
metab_556	pos	253.1428	2.2045	0.9540	1.2963	0.0030	0.0155
metab_9640	neg	253.1446	5.8731	1.2532	-2.7305	0.0059	0.0273
metab_9502	neg	253.1446	5.0812	0.2633	0.5613	0.4649	0.5872

metab_13480	neg	253.1446	2.8440	1.4394	2.7059	0.0006	0.0063
metab_6959	neg	253.1447	4.5717	0.4457	0.7642	0.2006	0.3214
metab_13245	neg	253.1447	3.4800	0.0380	0.4242	0.8045	0.8604
metab_1078	pos	253.1539	1.3153	0.8676	0.9678	0.0767	0.1557
metab_5622	pos	253.1541	1.9918	0.1173	-0.6404	0.7185	0.8050
metab_508	pos	253.1792	3.0877	1.0103	2.3368	0.0693	0.1451
metab_12454	neg	253.1810	6.5033	0.6060	-3.9423	0.4310	0.5558
metab_12069	neg	253.2173	7.7875	1.0958	-0.6592	0.0309	0.0845
metab_11730	neg	253.2174	8.7031	1.1328	-0.5369	0.0232	0.0698
metab_3266	pos	253.2396	13.9785	0.2498	-0.3290	0.2860	0.4162
metab_2644	pos	253.2518	6.8728	1.1271	2.0745	0.0444	0.1044
metab_3119	pos	253.2520	9.8522	0.0448	0.4010	0.9750	0.9853
metab_10410	neg	253.2537	9.1038	0.5999	-0.4698	0.1149	0.2143
metab_10895	neg	253.8429	15.0494	0.4815	-0.1335	0.0092	0.0377
metab_15071	neg	253.8429	0.2955	0.3234	-0.0510	0.0719	0.1526
metab_10875	neg	253.8429	14.3373	0.1555	0.0742	0.4326	0.5571
metab_6112	pos	254.0651	1.0883	0.1876	0.2302	0.7281	0.8123
metab_8268	neg	254.0672	1.4568	1.1220	-2.2158	0.0183	0.0593
metab_14283	neg	254.0863	1.5012	0.6480	2.8073	0.2178	0.3412
metab_5779	pos	254.1015	1.6553	0.6749	-0.5150	0.0223	0.0631
metab_6144	pos	254.1015	1.0040	0.5792	0.4929	0.0347	0.0872
metab_5891	pos	254.1017	1.4569	0.3878	0.2929	0.4039	0.5372
metab_14522	neg	254.1035	1.2236	0.4092	-0.8711	0.5316	0.6478
metab_1028	pos	254.1166	1.9918	0.5030	-0.5098	0.1903	0.3057
metab_12645	neg	254.1187	5.7102	0.4682	-0.2985	0.3589	0.4918
metab_5868	pos	254.1490	1.5004	0.7116	1.0391	0.1480	0.2538
metab_6139	pos	254.1604	1.0180	0.5204	0.4446	0.1497	0.2559
metab_5921	pos	254.1676	1.4005	1.3937	-2.4267	0.0009	0.0071
metab_5514	pos	254.1744	2.1746	1.4278	2.8086	0.0080	0.0307
metab_262	pos	254.1855	0.9900	0.8947	-1.3410	0.0288	0.0765
metab_6439	pos	254.1856	0.5280	1.0837	-1.4784	0.0180	0.0539
metab_2483	pos	254.2107	5.5260	0.3533	0.0718	0.2225	0.3450
metab_3875	pos	254.2469	9.1302	0.1342	0.0290	0.6211	0.7285
metab_2803	pos	254.2470	7.9260	0.3373	-0.3393	0.1242	0.2230
metab_10769	neg	254.9171	14.0282	0.4619	-0.1162	0.3176	0.4499
metab_8304	neg	255.0005	1.4856	1.4503	4.8097	0.0038	0.0202
metab_7490	neg	255.0512	1.4427	0.5595	1.0711	0.2249	0.3495
metab_4933	pos	255.0757	4.0183	0.4356	-0.4900	0.3177	0.4504
metab_2106	pos	255.0757	3.2573	2.3127	7.5407	0.0010	0.0073
metab_13769	neg	255.0778	2.2595	0.0891	0.8732	0.9999	0.9999
metab_1017	pos	255.0856	2.1746	0.6006	-0.8571	0.2306	0.3543
metab_1040	pos	255.0856	1.6693	0.5613	-0.6250	0.1563	0.2640
metab_7525	neg	255.0884	1.8205	0.7079	-1.0994	0.0781	0.1625
metab_5416	pos	255.0951	2.3908	0.6547	-0.4345	0.1782	0.2920
metab_5862	pos	255.0968	1.5144	0.5838	0.5445	0.0612	0.1319
metab_5199	pos	255.0969	2.9954	0.7786	-1.6491	0.1356	0.2380
metab_5295	pos	255.0969	2.7029	0.7819	-1.3195	0.0301	0.0788
metab_5130	pos	255.1121	3.2274	0.6065	0.3981	0.1576	0.2655

metab_8729	neg	255.1139	2.3227	1.3558	-2.6780	0.0117	0.0438
metab_13563	neg	255.1239	2.6841	0.9401	1.2826	0.0136	0.0487
metab_8621	neg	255.1241	2.0670	2.1109	6.5527	0.0000	0.0003
metab_333	pos	255.1330	1.5004	0.7416	0.8834	0.0638	0.1363
metab_5587	pos	255.1332	2.0524	0.3587	0.0770	0.3142	0.4468
metab_13619	neg	255.1351	2.5592	1.1525	1.6021	0.0027	0.0162
metab_8791	neg	255.1351	2.4651	0.3897	0.7596	0.3789	0.5088
metab_481	pos	255.1584	2.8725	0.8830	1.1121	0.0097	0.0353
metab_12496	neg	255.1602	6.3759	1.0999	-2.7375	0.0745	0.1565
metab_9547	neg	255.1603	5.3388	0.1163	-0.0452	0.6990	0.7841
metab_12821	neg	255.1603	4.8865	0.0162	0.7967	0.9867	0.9904
metab_5619	pos	255.1694	1.9918	1.6235	3.1036	0.0016	0.0102
metab_6008	pos	255.1696	1.2306	0.9489	-2.5812	0.0405	0.0975
metab_1991	pos	255.1848	2.8259	1.3759	-1.3034	0.0382	0.0932
metab_2407	pos	255.1900	4.9698	0.8148	0.8468	0.0084	0.0318
metab_4752	pos	255.1947	4.8809	0.0796	-0.2340	0.8546	0.9068
metab_2074	pos	255.2186	3.1495	1.5759	7.2288	0.0060	0.0251
metab_10152	neg	255.2330	8.1774	0.9696	-0.8047	0.0009	0.0076
metab_11867	neg	255.2330	8.4022	0.9869	-0.7693	0.0041	0.0210
metab_10407	neg	255.2330	9.1038	0.8622	-0.4876	0.0032	0.0179
metab_7394	neg	255.8222	6.8255	0.5470	-0.4126	0.1008	0.1951
metab_12770	neg	255.8222	5.0967	0.5155	-0.1742	0.1493	0.2603
metab_12851	neg	255.8223	4.7709	0.5873	-0.2502	0.0808	0.1666
metab_9011	neg	255.8223	3.0604	0.5144	-0.1868	0.1816	0.2997
metab_10949	neg	255.8999	14.1904	0.3629	-0.0598	0.0477	0.1140
metab_10879	neg	255.9347	14.3862	0.6773	-0.3423	0.0048	0.0238
metab_3289	pos	255.9435	14.0525	0.0380	0.1307	0.9110	0.9427
metab_14464	neg	256.0464	1.2805	0.7279	0.8511	0.0228	0.0687
metab_7912	neg	256.0592	0.5991	1.4903	3.9926	0.0033	0.0183
metab_13610	neg	256.0651	2.5893	1.8270	-4.2042	0.0531	0.1227
metab_212	pos	256.0688	0.6542	1.5486	6.0078	0.0134	0.0438
metab_1141	pos	256.0807	0.6123	0.3667	0.3834	0.1325	0.2336
metab_14492	neg	256.0828	1.2663	0.7205	0.9765	0.0117	0.0437
metab_1387	pos	256.0917	0.8500	1.5335	-2.4269	0.0018	0.0109
metab_6839	neg	256.0939	0.7818	1.1471	3.1561	0.0561	0.1277
metab_5039	pos	256.0961	3.5771	1.1357	-2.0867	0.0570	0.1257
metab_12934	neg	256.0981	4.4551	1.3080	-1.9948	0.0105	0.0409
metab_362	pos	256.1170	1.6693	0.0973	-0.6606	0.8655	0.9139
metab_6025	pos	256.1172	1.2162	0.2424	-0.1973	0.5314	0.6520
metab_2505	pos	256.1325	5.6934	0.4735	-0.5982	0.3716	0.5056
metab_9214	neg	256.1343	3.7830	1.7093	-5.7625	0.0000	0.0007
metab_12990	neg	256.1344	4.2367	0.5910	-0.8028	0.0874	0.1761
metab_12509	neg	256.1345	6.3111	1.2507	-2.4718	0.0168	0.0560
metab_515	pos	256.1537	3.1799	0.4101	0.6822	0.3488	0.4838
metab_13057	neg	256.1557	4.0182	0.0991	1.3105	0.9139	0.9428
metab_5621	pos	256.1894	1.9918	1.4322	5.9656	0.0090	0.0336
metab_4905	pos	256.1900	4.1234	1.2062	2.1032	0.0490	0.1126
metab_1951	pos	256.1901	2.6244	1.6911	7.2354	0.0037	0.0179

metab_12548	neg	256.1919	6.1320	0.4857	1.0063	0.1851	0.3037
metab_2531	pos	256.2264	5.8753	0.3643	0.1118	0.1570	0.2647
metab_2906	pos	256.2521	8.5393	0.9594	2.1158	0.0475	0.1097
metab_2903	pos	256.2627	8.5241	0.3435	-0.3572	0.1317	0.2323
metab_5875	pos	256.6931	1.5004	0.0949	-1.8486	0.9094	0.9421
metab_10925	neg	256.9396	14.4028	0.4285	-0.0563	0.0158	0.0540
metab_10966	neg	256.9396	14.1581	0.2033	0.0627	0.3771	0.5075
metab_6272	pos	257.0526	0.7661	0.2318	1.1518	0.5683	0.6843
metab_14919	neg	257.0659	0.5431	1.7203	3.9947	0.0012	0.0096
metab_7949	neg	257.0779	0.6551	0.6531	1.8976	0.2874	0.4191
metab_7996	neg	257.0780	0.8240	0.4336	0.9686	0.2941	0.4253
metab_5584	pos	257.0910	2.0524	0.0722	0.1093	0.9807	0.9884
metab_5466	pos	257.0914	2.2665	0.0195	0.1872	0.9020	0.9371
metab_6598	neg	257.1033	1.6948	0.4800	0.8498	0.2275	0.3524
metab_14714	neg	257.1143	0.7818	0.1497	0.2731	0.7906	0.8498
metab_8778	neg	257.1144	2.4487	0.0440	0.5481	0.9244	0.9508
metab_14527	neg	257.1144	1.2084	0.0151	0.2942	0.9651	0.9759
metab_5452	pos	257.1278	2.3133	0.9073	-1.5768	0.0520	0.1173
metab_13263	neg	257.1298	3.3971	0.3349	-0.5650	0.5032	0.6233
metab_13112	neg	257.1396	3.8333	0.0657	0.0793	0.9810	0.9868
metab_13334	neg	257.1396	3.2130	0.0623	-0.2372	0.9670	0.9768
metab_1490	pos	257.1487	1.1731	1.0720	1.7530	0.0034	0.0170
metab_1979	pos	257.1489	2.7653	1.0170	1.3305	0.0067	0.0271
metab_13819	neg	257.1508	2.1798	0.4202	0.7758	0.4324	0.5570
metab_13509	neg	257.1508	2.7961	0.4468	0.8741	0.3814	0.5112
metab_14335	neg	257.1621	1.4568	1.4429	13.0962	0.0171	0.0566
metab_2081	pos	257.1741	3.1799	1.4859	2.6573	0.0024	0.0134
metab_12745	neg	257.1759	5.2257	0.5181	-0.2461	0.3396	0.4728
metab_12612	neg	257.1759	5.8569	0.1423	-0.0567	0.6439	0.7404
metab_12799	neg	257.1760	4.9510	0.1882	1.4483	0.8093	0.8643
metab_11940	neg	257.2124	8.1623	0.5300	0.9724	0.1072	0.2044
metab_12651	neg	257.8193	5.6776	0.5349	-0.2206	0.0913	0.1818
metab_9505	neg	257.8193	5.0967	0.5247	-0.1679	0.1466	0.2572
metab_12918	neg	257.8193	4.5391	0.6343	-0.2751	0.0600	0.1342
metab_12383	neg	257.8193	6.8093	0.5633	-0.4953	0.1045	0.2006
metab_13438	neg	257.8194	2.9588	0.8838	-0.4299	0.1592	0.2733
metab_11064	neg	257.9191	14.0282	0.2848	-0.0580	0.1688	0.2851
metab_7609	neg	258.0385	0.5431	0.7689	-0.5441	0.0044	0.0221
metab_8128	neg	258.0622	1.2236	0.2850	0.5347	0.4908	0.6122
metab_13784	neg	258.0773	2.2437	0.4181	-0.0192	0.1150	0.2144
metab_13319	neg	258.0773	3.2473	0.6454	-0.7925	0.0701	0.1499
metab_13012	neg	258.0773	4.1869	0.9243	-1.1236	0.0340	0.0900
metab_8880	neg	258.0774	2.7318	0.5981	-0.5836	0.0672	0.1456
metab_8165	neg	258.0985	1.2805	2.0052	4.4576	0.0000	0.0001
metab_13999	neg	258.0985	1.8682	0.7672	-2.4539	0.1716	0.2881
metab_226	pos	258.1092	0.6402	1.2037	1.5715	0.0086	0.0324
metab_12887	neg	258.1137	4.6216	1.2435	-1.9237	0.0119	0.0442
metab_511	pos	258.1328	3.1495	0.6086	0.4873	0.0812	0.1627

metab_8821	neg	258.1349	2.5592	0.3363	1.3618	0.5952	0.7020
metab_6160	pos	258.1437	0.9620	0.5748	-0.5922	0.1256	0.2242
metab_14668	neg	258.1459	0.8803	0.0499	0.8058	0.9706	0.9790
metab_14363	neg	258.1461	1.4427	0.4518	0.9382	0.3241	0.4565
metab_13798	neg	258.1463	2.2119	2.5364	-6.5543	0.0001	0.0024
metab_5617	pos	258.1692	2.0062	1.0526	-1.7251	0.0023	0.0132
metab_13370	neg	258.1714	3.1115	0.1599	-0.2452	0.7706	0.8360
metab_4671	pos	258.2056	5.2573	0.1716	1.0027	0.7268	0.8118
metab_5485	pos	258.2057	2.2209	0.2070	0.4780	0.6360	0.7402
metab_2905	pos	258.2696	8.5393	0.2974	-0.3357	0.1962	0.3132
metab_10787	neg	258.9160	14.0438	0.6484	-0.3143	0.0001	0.0019
metab_6531	pos	258.9890	0.4855	0.7617	-0.9832	0.0095	0.0348
metab_13959	neg	258.9915	1.9280	0.8179	1.7466	0.0965	0.1891
metab_7999	neg	259.0047	0.8240	0.3448	-0.3872	0.5442	0.6574
metab_6863	neg	259.0096	1.2663	0.4839	1.6072	0.4599	0.5825
metab_2077	pos	259.0159	3.1648	0.7837	2.6885	0.2152	0.3365
metab_7614	neg	259.0363	2.5431	0.7938	-0.2681	0.0595	0.1335
metab_5258	pos	259.0595	2.8259	1.7074	11.2747	0.0017	0.0108
metab_14163	neg	259.0613	1.6186	2.1294	-4.9463	0.0000	0.0008
metab_13646	neg	259.0613	2.5113	2.3473	-7.0194	0.0000	0.0000
metab_8531	neg	259.0726	1.8833	0.0845	0.5154	0.9829	0.9881
metab_14253	neg	259.0764	1.5297	0.8562	-0.9789	0.0318	0.0862
metab_7677	neg	259.0788	0.5126	0.6878	-0.6357	0.1014	0.1959
metab_6218	pos	259.0918	0.8500	0.4296	-0.3068	0.0470	0.1091
metab_14659	neg	259.0937	0.8946	0.4675	-0.2712	0.1815	0.2996
metab_13269	neg	259.0978	3.3803	1.2509	-1.6235	0.0036	0.0195
metab_5214	pos	259.1071	2.9652	0.9908	-1.2669	0.0151	0.0477
metab_8896	neg	259.1189	2.7624	1.5584	2.8526	0.0000	0.0010
metab_13851	neg	259.1190	2.1153	1.4581	2.9038	0.0002	0.0034
metab_8458	neg	259.1193	1.7419	1.4373	2.9142	0.0008	0.0074
metab_1118	pos	259.1279	0.7801	0.1006	0.0131	0.7408	0.8221
metab_6028	pos	259.1281	1.2162	0.1611	-0.1186	0.6818	0.7766
metab_8057	neg	259.1300	1.0215	0.0176	0.2356	0.9743	0.9818
metab_8439	neg	259.1301	1.7104	0.2016	-0.0509	0.5762	0.6854
metab_14934	neg	259.1414	0.5286	0.6954	2.0048	0.1969	0.3175
metab_1888	pos	259.1434	2.3908	0.9105	-1.3992	0.0559	0.1239
metab_2142	pos	259.1434	3.3943	0.5211	-0.5632	0.1078	0.2003
metab_13872	neg	259.1552	2.0670	1.9426	3.7671	0.0003	0.0040
metab_12928	neg	259.1553	4.4888	0.9210	1.6801	0.0499	0.1173
metab_9179	neg	259.1554	3.6645	1.5569	2.9933	0.0003	0.0041
metab_1903	pos	259.1645	2.4370	0.7176	-2.1550	0.2448	0.3694
metab_529	pos	259.1646	2.3133	0.3012	-1.1890	0.5272	0.6480
metab_400	pos	259.1750	1.9758	1.1566	1.9426	0.0031	0.0160
metab_5894	pos	259.1758	1.4569	1.5516	3.6720	0.0042	0.0196
metab_9518	neg	259.1916	5.1610	0.5694	2.3427	0.3565	0.4893
metab_9465	neg	259.1916	4.8541	0.5005	4.5677	0.4220	0.5480
metab_12631	neg	259.1916	5.7585	0.0545	0.2923	0.8641	0.9068
metab_5201	pos	259.2009	2.9954	1.9512	4.5818	0.0007	0.0061

metab_650	pos	259.2048	5.6773	0.0020	-0.2702	0.9873	0.9921
metab_984	pos	259.2049	7.0670	0.7060	-0.8396	0.0641	0.1368
metab_4475	pos	259.2049	6.3885	0.1703	-0.5159	0.6936	0.7859
metab_628	pos	259.2049	4.9413	0.1017	-0.3304	0.8383	0.8940
metab_4440	pos	259.2050	6.5395	0.1790	-0.6098	0.6975	0.7890
metab_4849	pos	259.2050	4.3818	0.1065	-0.5438	0.7790	0.8509
metab_10954	neg	259.8779	14.1904	0.3369	-0.0524	0.0814	0.1674
metab_8225	neg	260.0236	1.3669	1.1279	2.6347	0.0287	0.0805
metab_14035	neg	260.0389	1.8046	1.6202	-5.4040	0.0004	0.0050
metab_6360	pos	260.0523	0.5983	0.3723	-0.4174	0.2192	0.3415
metab_13311	neg	260.0568	3.2801	1.0770	3.0450	0.0663	0.1442
metab_5796	pos	260.0732	1.6272	0.4099	-0.5724	0.3792	0.5118
metab_291	pos	260.0759	1.2016	0.2361	0.8394	0.7052	0.7944
metab_1520	pos	260.0909	1.2446	1.4264	4.8870	0.0301	0.0788
metab_1825	pos	260.0911	2.1746	0.5358	-0.6737	0.0385	0.0937
metab_5069	pos	260.0911	3.4549	1.6197	-3.2736	0.0015	0.0097
metab_5327	pos	260.0911	2.6090	0.4218	-0.3991	0.2319	0.3559
metab_13116	neg	260.0931	3.8164	0.9100	-1.5975	0.0487	0.1156
metab_7679	neg	260.0931	3.0942	0.2461	-0.1821	0.3828	0.5125
metab_8706	neg	260.0934	2.2754	0.1092	0.7735	0.7838	0.8459
metab_13965	neg	260.1042	1.9280	2.0812	-3.5943	0.0004	0.0047
metab_5989	pos	260.1121	1.2725	2.2692	5.6113	0.0000	0.0000
metab_8786	neg	260.1142	2.4651	1.2035	-1.9469	0.0052	0.0253
metab_7563	neg	260.1142	2.2272	0.6135	-0.6085	0.1580	0.2718
metab_14917	neg	260.1253	0.5431	1.2542	3.3574	0.0196	0.0619
metab_4898	pos	260.1274	4.1544	0.5524	-1.9313	0.3228	0.4562
metab_9459	neg	260.1294	4.8210	0.3989	0.1784	0.5017	0.6219
metab_14461	neg	260.1334	1.2805	1.4300	5.1773	0.0003	0.0037
metab_6134	pos	260.1485	1.0321	0.8977	-1.8330	0.0375	0.0920
metab_2148	pos	260.1485	3.4399	0.4498	-0.0497	0.3158	0.4483
metab_473	pos	260.1486	2.5627	0.3839	0.7303	0.4042	0.5373
metab_13680	neg	260.1505	2.4487	1.3571	-1.6104	0.0202	0.0634
metab_6202	pos	260.1595	0.8780	0.1183	0.3965	0.8970	0.9339
metab_320	pos	260.1598	1.4289	0.7043	0.9616	0.0735	0.1512
metab_1834	pos	260.1598	2.2045	1.2397	-1.9845	0.0139	0.0451
metab_7822	neg	260.1618	0.5286	1.3201	2.9013	0.0233	0.0701
metab_2173	pos	260.1639	3.5620	1.2404	8.1634	0.1886	0.3041
metab_12680	neg	260.1658	5.5808	0.1231	0.0503	0.8664	0.9083
metab_1933	pos	260.1850	2.5782	1.3017	-2.3589	0.0001	0.0020
metab_5476	pos	260.1850	2.2519	0.8756	-1.0226	0.0350	0.0877
metab_5423	pos	260.1850	2.3908	0.4933	-0.4279	0.0350	0.0877
metab_6161	pos	260.1961	0.9620	0.2467	-0.2299	0.5595	0.6764
metab_7706	neg	260.8630	0.0197	0.1718	0.0723	0.3865	0.5159
metab_7202	neg	260.9164	14.0282	0.3750	-0.0326	0.0184	0.0594
metab_14121	neg	261.0077	1.6797	0.4330	-0.0849	0.4035	0.5316
metab_1260	pos	261.0299	0.5420	0.2459	0.3715	0.5445	0.6638
metab_6719	neg	261.0382	0.5431	0.0855	1.2426	0.8677	0.9092
metab_14055	neg	261.0384	1.7729	0.0484	1.6222	0.8800	0.9180

metab_8375	neg	261.0390	1.5736	0.0509	1.5350	0.9942	0.9952
metab_14329	neg	261.0433	1.4568	0.8483	3.4799	0.1731	0.2897
metab_8176	neg	261.0618	1.2805	1.6245	3.5118	0.0009	0.0078
metab_14190	neg	261.0729	1.5736	0.5680	1.4529	0.3993	0.5273
metab_14774	neg	261.0729	0.6271	0.2344	0.1682	0.3533	0.4864
metab_5299	pos	261.0752	2.6869	1.7663	4.8995	0.0026	0.0143
metab_8700	neg	261.0773	2.2595	1.4207	-2.8646	0.0073	0.0319
metab_1727	pos	261.0859	1.8871	0.4331	0.7214	0.4112	0.5440
metab_446	pos	261.0863	2.3600	0.6146	2.6449	0.2902	0.4206
metab_8520	neg	261.0882	1.8682	1.2822	2.2633	0.0002	0.0030
metab_8361	neg	261.0980	1.5586	1.5247	3.3052	0.0018	0.0122
metab_7476	neg	261.0982	1.6642	1.7766	4.0479	0.0001	0.0017
metab_359	pos	261.1227	1.6413	0.4661	0.3547	0.0867	0.1709
metab_5266	pos	261.1322	2.8108	1.2029	3.2234	0.0288	0.0764
metab_13841	neg	261.1344	2.1311	0.7950	1.0585	0.0053	0.0257
metab_8836	neg	261.1345	2.6046	1.0993	1.6146	0.0023	0.0144
metab_13519	neg	261.1346	2.7792	0.1945	-0.0573	0.7417	0.8138
metab_1677	pos	261.1436	1.7128	0.2834	-0.3220	0.4872	0.6121
metab_207	pos	261.1437	0.6123	0.5762	0.7284	0.1250	0.2238
metab_14542	neg	261.1458	1.1796	1.0892	-3.4129	0.0901	0.1800
metab_4884	pos	261.1479	4.2457	1.6747	5.7687	0.0064	0.0263
metab_6464	pos	261.1550	0.5140	0.5113	1.4908	0.3525	0.4876
metab_5590	pos	261.1585	2.0360	0.9004	-1.2876	0.0772	0.1566
metab_2198	pos	261.1590	3.6835	0.6962	-1.1787	0.1582	0.2665
metab_13575	neg	261.1709	2.6524	0.7333	-0.6360	0.1717	0.2881
metab_5965	pos	261.1801	1.3293	0.4862	-0.5127	0.2633	0.3909
metab_974	pos	261.2205	7.8830	0.1029	-0.1181	0.7774	0.8499
metab_684	pos	261.2206	6.6001	0.9063	-1.2302	0.0037	0.0178
metab_1005	pos	261.2206	5.9649	0.4323	-0.3271	0.3759	0.5094
metab_9399	neg	261.7753	4.5717	0.8544	-0.7137	0.0143	0.0503
metab_15015	neg	261.9544	0.5126	0.7081	-0.7539	0.0336	0.0896
metab_14679	neg	262.0569	0.8662	0.4409	0.8713	0.1896	0.3088
metab_6407	pos	262.0680	0.5560	1.4714	-2.5208	0.0004	0.0039
metab_6607	neg	262.0723	2.8269	0.2111	0.0337	0.5138	0.6328
metab_6080	pos	262.0912	1.1447	0.7702	1.2523	0.0486	0.1118
metab_8029	neg	262.0934	0.9368	1.0949	1.7181	0.0012	0.0095
metab_5183	pos	262.1067	3.0572	0.1691	-0.1609	0.4904	0.6149
metab_5118	pos	262.1068	3.2727	0.9556	-1.4020	0.0065	0.0265
metab_8990	neg	262.1086	3.0101	1.2204	-1.7504	0.0038	0.0203
metab_13352	neg	262.1087	3.1626	0.5127	0.2692	0.3059	0.4371
metab_8853	neg	262.1087	2.6524	0.3065	-0.2135	0.4048	0.5329
metab_1059	pos	262.1102	1.5004	1.2455	1.8055	0.0047	0.0210
metab_13144	neg	262.1122	3.7488	1.5532	-3.5569	0.0050	0.0245
metab_1032	pos	262.1177	1.9463	1.1125	-1.6619	0.0104	0.0368
metab_8640	neg	262.1192	2.1153	0.5855	-1.2217	0.0983	0.1918
metab_1122	pos	262.1277	0.7661	1.1381	-2.3787	0.0706	0.1471
metab_5484	pos	262.1278	2.2361	0.5899	-0.8501	0.1047	0.1962
metab_500	pos	262.1279	2.4690	1.1343	-1.8648	0.0008	0.0064

metab_14474	neg	262.1298	1.2805	0.9444	1.8281	0.0359	0.0932
metab_13731	neg	262.1298	2.3533	0.7644	1.1580	0.0284	0.0800
metab_4763	pos	262.1431	4.8208	0.7799	-0.2332	0.2421	0.3671
metab_2116	pos	262.1431	3.2880	0.2202	0.3574	0.7744	0.8477
metab_12808	neg	262.1450	4.9349	0.6568	-1.4797	0.1627	0.2776
metab_12853	neg	262.1450	4.7546	0.6870	-0.0518	0.2561	0.3847
metab_5578	pos	262.1542	2.0677	0.1402	0.5157	0.8090	0.8737
metab_5088	pos	262.1543	3.3793	0.3462	-0.6708	0.4556	0.5846
metab_1454	pos	262.1641	1.0883	0.1295	-0.0273	0.6638	0.7620
metab_5102	pos	262.1643	3.3183	1.5306	2.9604	0.0006	0.0055
metab_6440	pos	262.1754	0.5280	1.3772	2.6027	0.0307	0.0799
metab_2492	pos	262.1795	5.5864	0.2461	-0.2551	0.6634	0.7617
metab_5225	pos	262.1795	2.9342	1.3407	2.9952	0.1272	0.2265
metab_4686	pos	262.2370	5.1533	0.4260	0.1082	0.2042	0.3236
metab_6682	neg	262.8584	0.0197	0.3555	-0.0448	0.0545	0.1250
metab_10959	neg	262.8584	14.1744	0.3241	-0.0101	0.0880	0.1770
metab_11043	neg	262.9120	14.0438	0.2983	-0.0114	0.0963	0.1889
metab_7864	neg	262.9217	0.5431	0.6679	-1.7995	0.1675	0.2836
metab_7674	neg	262.9303	0.5126	0.7269	-0.7779	0.0256	0.0747
metab_1283	pos	262.9476	0.5703	2.0245	3.8464	0.0000	0.0011
metab_8538	neg	263.0562	1.8974	1.0306	-2.0374	0.1984	0.3191
metab_8257	neg	263.0599	1.4427	1.6333	3.7964	0.0011	0.0092
metab_1113	pos	263.0865	0.8640	0.6286	0.7992	0.2268	0.3499
metab_1619	pos	263.1018	1.5424	0.2922	-0.3168	0.2997	0.4310
metab_5676	pos	263.1019	1.8729	1.3591	2.3363	0.0001	0.0019
metab_14526	neg	263.1035	1.2084	0.2043	1.7428	0.9111	0.9415
metab_8518	neg	263.1039	1.8524	0.5373	-0.9846	0.2065	0.3285
metab_7567	neg	263.1040	2.2119	0.0650	0.3163	0.9569	0.9715
metab_13546	neg	263.1292	2.7155	0.6933	-1.3675	0.1008	0.1951
metab_372	pos	263.1381	1.7128	0.3175	-0.4559	0.3399	0.4742
metab_7523	neg	263.1403	1.9280	0.7203	-0.7563	0.0650	0.1425
metab_14083	neg	263.1403	1.7419	0.9602	-1.0209	0.1084	0.2061
metab_13811	neg	263.1501	2.1956	0.3852	1.3199	0.5803	0.6889
metab_7640	neg	263.1503	2.7155	1.4517	2.9996	0.0039	0.0208
metab_2496	pos	263.1633	5.6322	1.3665	2.5252	0.0050	0.0220
metab_2215	pos	263.1635	3.7447	1.6887	4.5824	0.0048	0.0213
metab_6243	pos	263.2320	0.7941	1.4466	-2.3330	0.0002	0.0029
metab_734	pos	263.2361	7.6332	0.0264	0.0192	0.9587	0.9744
metab_767	pos	263.2362	8.2289	0.3089	-0.2769	0.1313	0.2320
metab_11140	neg	263.7724	13.8980	0.7120	-0.3431	0.0001	0.0020
metab_6386	pos	263.9254	0.5703	0.9780	-1.3848	0.0012	0.0086
metab_1225	pos	263.9325	0.4999	1.2290	-2.3091	0.0012	0.0085
metab_14703	neg	263.9682	0.7958	0.1783	1.2158	0.9290	0.9539
metab_14257	neg	264.0551	1.5297	1.5309	-2.3014	0.0015	0.0111
metab_13482	neg	264.0778	2.8440	0.1250	0.1510	0.6898	0.7775
metab_1694	pos	264.0857	1.7412	0.4007	1.4289	0.5451	0.6644
metab_7605	neg	264.0880	2.4813	1.2461	-1.3525	0.0003	0.0036
metab_6018	pos	264.0974	1.2162	2.3495	10.5303	0.0107	0.0375

metab_219	pos	264.1069	0.7241	0.5522	-0.9651	0.0947	0.1822
metab_14823	neg	264.1089	0.5991	0.5569	-0.1125	0.3887	0.5175
metab_299	pos	264.1223	1.2446	1.2492	-1.8930	0.0068	0.0274
metab_13971	neg	264.1242	1.9125	0.4983	-0.6943	0.2178	0.3412
metab_13053	neg	264.1244	4.0516	1.2160	-1.5769	0.0406	0.1018
metab_13553	neg	264.1244	2.7155	0.1647	0.7076	0.7506	0.8216
metab_5911	pos	264.1257	1.4289	0.1847	0.0474	0.7535	0.8312
metab_3349	pos	264.1271	14.4708	0.1952	-0.1873	0.2506	0.3758
metab_318	pos	264.1333	1.4146	1.3536	2.7720	0.0138	0.0449
metab_285	pos	264.1336	1.1875	1.5226	2.8994	0.0015	0.0098
metab_1537	pos	264.1432	1.2869	1.5118	14.8185	0.0110	0.0383
metab_6138	pos	264.1433	1.0180	0.1537	-0.3849	0.6791	0.7748
metab_5434	pos	264.1434	2.3600	1.1249	1.6232	0.0004	0.0041
metab_4732	pos	264.1587	4.9267	0.0582	-1.2637	0.8538	0.9062
metab_12911	neg	264.1607	4.5553	1.2946	1.9798	0.0020	0.0130
metab_406	pos	264.2162	2.0211	1.9133	3.6862	0.0000	0.0002
metab_15129	neg	264.8742	0.0197	0.4018	-0.0973	0.0345	0.0909
metab_15014	neg	264.9277	0.5126	0.6889	-0.6942	0.0308	0.0843
metab_8820	neg	264.9809	2.5431	1.6236	-2.7742	0.0003	0.0042
metab_6522	pos	265.0149	0.4999	0.9274	-1.4750	0.0079	0.0304
metab_8284	neg	265.0355	1.4568	0.3495	0.7334	0.5153	0.6336
metab_8067	neg	265.0931	1.0357	0.4111	0.6998	0.2784	0.4089
metab_2113	pos	265.1065	3.2727	1.4833	10.2967	0.0079	0.0306
metab_1697	pos	265.1175	1.7554	1.4664	2.1661	0.0006	0.0054
metab_1507	pos	265.1176	1.2162	1.0216	1.0079	0.0012	0.0085
metab_8130	neg	265.1196	1.2236	0.1490	0.2362	0.6609	0.7553
metab_14162	neg	265.1196	1.6186	0.2326	-0.0054	0.3837	0.5131
metab_13424	neg	265.1200	2.9931	0.0935	-0.1082	0.7559	0.8242
metab_3423	pos	265.1257	14.3928	0.1809	-0.1836	0.2808	0.4101
metab_6570	neg	265.1294	1.9439	1.0729	1.7323	0.0013	0.0101
metab_14097	neg	265.1295	1.7104	1.2385	3.2946	0.0050	0.0244
metab_71	pos	265.1428	3.3490	2.0483	4.3556	0.0000	0.0000
metab_2286	pos	265.1428	4.1698	1.6678	4.5606	0.0306	0.0797
metab_13429	neg	265.1448	2.9931	2.0545	5.7419	0.0001	0.0025
metab_9761	neg	265.1481	6.5346	0.2567	0.5763	0.3242	0.4567
metab_11566	neg	265.1482	9.1200	0.7196	-0.3261	0.0001	0.0023
metab_10297	neg	265.1482	8.7031	0.7568	-0.4177	0.0000	0.0005
metab_5657	pos	265.1538	1.9309	0.3748	-0.4943	0.2283	0.3520
metab_2910	pos	265.1577	8.5835	1.0797	1.3941	0.0035	0.0173
metab_5358	pos	265.1652	2.5158	0.0447	0.5516	0.9582	0.9741
metab_4938	pos	265.1791	3.9876	2.1223	5.1913	0.0000	0.0002
metab_5090	pos	265.1791	3.3635	1.1731	1.9419	0.0672	0.1418
metab_5273	pos	265.1903	2.7804	0.1289	-0.3596	0.7954	0.8635
metab_2071	pos	265.1903	3.1346	0.2558	-0.5882	0.6764	0.7725
metab_1198	pos	265.2518	0.3380	0.4853	-0.3757	0.0008	0.0064
metab_3370	pos	265.2518	15.2974	0.4464	-0.3321	0.0029	0.0153
metab_788	pos	265.2518	8.6132	0.3528	-0.3536	0.1042	0.1956
metab_9784	neg	265.7696	6.6632	0.6293	-0.5947	0.0722	0.1530

metab_10747	neg	266.0237	13.9952	0.1165	-0.0263	0.5462	0.6588
metab_7489	neg	266.0673	1.4710	0.2342	0.5843	0.6807	0.7704
metab_8863	neg	266.0674	2.6678	0.9273	1.7340	0.1050	0.2011
metab_9128	neg	266.0857	3.5141	0.2380	-0.0618	0.4585	0.5813
metab_61	pos	266.1016	2.4850	1.2782	-1.8161	0.0009	0.0069
metab_13617	neg	266.1036	2.5748	0.7166	-0.5251	0.0514	0.1197
metab_14440	neg	266.1036	1.3089	1.2276	-1.5857	0.0020	0.0132
metab_12543	neg	266.1036	6.1482	0.2888	-0.0169	0.1575	0.2712
metab_8528	neg	266.1147	1.8682	0.4959	-0.9265	0.3363	0.4694
metab_9035	neg	266.1150	3.1455	0.5858	-0.5092	0.2863	0.4178
metab_12782	neg	266.1190	5.0159	1.6529	-3.2036	0.0011	0.0091
metab_3332	pos	266.1222	14.3019	0.1723	-0.1730	0.2912	0.4215
metab_14	pos	266.1226	0.6123	0.1286	0.2562	0.8942	0.9319
metab_5686	pos	266.1377	1.8432	0.4791	1.3506	0.5121	0.6338
metab_6441	pos	266.1590	0.5280	1.6206	-3.3267	0.0016	0.0104
metab_5417	pos	266.1646	2.3908	0.5002	-1.1715	0.4611	0.5890
metab_604	pos	266.1735	4.6398	0.0182	-0.0725	0.8918	0.9313
metab_1747	pos	266.1739	1.9609	2.6706	6.7541	0.0002	0.0027
metab_4724	pos	266.1743	4.9413	1.9382	5.2512	0.0000	0.0001
metab_2682	pos	266.2472	7.0228	1.4935	-4.6980	0.0006	0.0055
metab_10948	neg	266.9685	14.1904	0.2875	0.0186	0.1311	0.2363
metab_14219	neg	267.0150	1.5439	1.6201	2.3660	0.0283	0.0414
metab_190	pos	267.0583	0.5280	0.8240	-1.2144	0.0202	0.0588
metab_9244	neg	267.0667	3.8839	1.5002	-4.1068	0.0069	0.0306
metab_6775	neg	267.0724	0.6131	0.7990	-0.6561	0.0116	0.0435
metab_6003	pos	267.0968	1.2446	1.4368	2.7466	0.0016	0.0102
metab_9094	neg	267.0990	3.3465	0.4692	-0.4296	0.3775	0.5076
metab_14132	neg	267.0992	1.6642	0.0319	0.3969	0.9366	0.9586
metab_9686	neg	267.1086	6.1157	0.2615	-0.0254	0.1678	0.2840
metab_13669	neg	267.1087	2.4651	0.4533	-0.1159	0.0091	0.0375
metab_10892	neg	267.1087	14.6830	0.2701	-0.0200	0.1826	0.3008
metab_12920	neg	267.1088	4.5219	0.2623	0.0115	0.2141	0.3369
metab_9466	neg	267.1088	4.8541	0.4703	-0.2104	0.0240	0.0715
metab_12678	neg	267.1088	5.5808	0.3815	-0.0696	0.0572	0.1296
metab_13063	neg	267.1089	4.0020	0.1787	0.1463	0.3173	0.4496
metab_8035	neg	267.1090	0.9508	0.4005	1.4577	0.4385	0.5625
metab_5359	pos	267.1216	2.5158	0.4523	1.0191	0.2811	0.4104
metab_4678	pos	267.1219	5.1975	0.3809	-0.3241	0.0503	0.1145
metab_9308	neg	267.1241	4.1193	1.1087	-1.5701	0.0033	0.0181
metab_7721	neg	267.1241	3.5475	0.8815	1.5557	0.0221	0.0673
metab_13791	neg	267.1241	2.2272	0.9805	1.5187	0.0066	0.0295
metab_12979	neg	267.1241	4.2870	0.5632	-0.7764	0.1791	0.2970
metab_7708	neg	267.1241	3.3803	0.5806	0.8505	0.0648	0.1421
metab_9432	neg	267.1242	4.6883	0.1937	0.4150	0.5500	0.6621
metab_3428	pos	267.1249	14.3315	0.1561	-0.1702	0.3404	0.4748
metab_5803	pos	267.1331	1.6128	0.9326	1.0280	0.0005	0.0050
metab_5208	pos	267.1331	2.9806	0.9857	-0.9760	0.1840	0.2986
metab_39	pos	267.1332	1.1875	0.8256	0.9703	0.0042	0.0196

metab_8264	neg	267.1353	1.4427	0.9739	1.4744	0.0192	0.0610
metab_5290	pos	267.1417	2.7188	0.4163	0.8769	0.3335	0.4678
metab_2271	pos	267.1419	4.0479	1.6994	-3.2592	0.0001	0.0011
metab_79	pos	267.1584	4.3371	1.2437	1.8445	0.0002	0.0024
metab_482	pos	267.1584	2.8108	1.5726	3.2753	0.0003	0.0038
metab_12727	neg	267.1604	5.3228	0.1599	0.3141	0.3752	0.5060
metab_9471	neg	267.1604	4.8865	1.0217	1.6765	0.0009	0.0081
metab_12677	neg	267.1604	5.5966	0.2501	0.6345	0.6285	0.7276
metab_12528	neg	267.1605	6.2294	1.1144	1.6898	0.0001	0.0018
metab_7690	neg	267.1605	3.1626	1.2772	2.8723	0.0050	0.0244
metab_9153	neg	267.1605	3.5813	2.0788	5.4679	0.0042	0.0215
metab_13085	neg	267.1605	3.9177	1.6623	4.0792	0.0019	0.0128
metab_5941	pos	267.1694	1.3579	0.8203	0.7996	0.0676	0.1424
metab_756	pos	267.1736	8.1132	0.4343	0.2979	0.1995	0.3175
metab_3835	pos	267.1736	9.3429	0.2840	0.1597	0.4002	0.5337
metab_3888	pos	267.1736	9.0999	0.3268	0.1598	0.3358	0.4695
metab_3961	pos	267.1736	8.7303	0.4328	0.1912	0.1505	0.2570
metab_2456	pos	267.1946	5.3311	0.0470	1.2309	0.9453	0.9658
metab_2420	pos	267.1947	5.0451	1.4147	2.0264	0.0202	0.0588
metab_577	pos	267.1947	4.0939	1.3911	3.8475	0.0581	0.1269
metab_605	pos	267.1947	4.6851	1.3069	2.6578	0.0516	0.1167
metab_557	pos	267.1948	3.7447	2.4506	7.2648	0.0080	0.0307
metab_12254	neg	267.1969	7.1903	1.8790	-4.6198	0.0003	0.0044
metab_12466	neg	267.1972	6.4396	1.5679	-1.3949	0.0230	0.0692
metab_3994	pos	267.2585	8.6132	0.1605	-0.1558	0.4985	0.6215
metab_14882	neg	267.9633	0.5571	1.3029	-1.6072	0.0000	0.0006
metab_8424	neg	268.0290	1.6642	2.0332	-6.4041	0.0000	0.0000
metab_154	pos	268.0508	0.5983	1.2180	-1.5308	0.0004	0.0044
metab_370	pos	268.0807	1.7128	0.5227	1.7740	0.4047	0.5377
metab_14341	neg	268.0830	1.4568	0.7452	1.4547	0.0974	0.1906
metab_13658	neg	268.0830	2.4970	0.3800	0.3698	0.2907	0.4220
metab_13277	neg	268.0830	3.3465	0.2065	-0.0937	0.6354	0.7334
metab_13202	neg	268.0982	3.5813	1.3631	-9.3704	0.0005	0.0057
metab_13016	neg	268.0982	4.1699	0.2493	1.9447	0.6276	0.7272
metab_6234	pos	268.1031	0.8221	1.0841	-1.1481	0.0029	0.0152
metab_1079	pos	268.1171	1.3153	0.5272	0.2716	0.0173	0.0525
metab_5454	pos	268.1323	2.2978	1.7490	-1.4981	0.0164	0.0505
metab_5938	pos	268.1535	1.3720	0.2703	-3.9737	0.7078	0.7965
metab_4786	pos	268.1901	4.6700	1.6374	3.1960	0.0000	0.0007
metab_2857	pos	268.2627	8.2872	0.2948	-0.2855	0.2292	0.3529
metab_5936	pos	268.2707	1.3720	0.3388	-7.5272	0.4200	0.5514
metab_11117	neg	268.8011	13.9952	0.1727	0.1008	0.3772	0.5075
metab_10707	neg	268.8012	13.2913	0.3816	-0.1359	0.0708	0.1510
metab_7735	neg	268.8246	0.0197	0.4933	-0.2110	0.0169	0.0562
metab_1119	pos	268.9452	0.7801	1.1074	-1.3661	0.0042	0.0196
metab_14742	neg	268.9472	0.7254	0.8731	-0.8981	0.0333	0.0890
metab_10829	neg	269.0101	14.1090	0.3196	-0.1932	0.1791	0.2970
metab_6822	neg	269.0102	1.0074	0.5553	-0.4870	0.0499	0.1173

metab_8353	neg	269.0162	1.5439	0.6263	1.3726	0.1817	0.2998
metab_13970	neg	269.0162	1.9125	0.6413	2.0693	0.3077	0.4388
metab_8369	neg	269.0306	1.5736	1.5517	3.0014	0.0007	0.0066
metab_9173	neg	269.0460	3.6479	1.2824	7.2812	0.0267	0.0552
metab_6596	neg	269.0671	1.5012	1.0926	1.6038	0.0033	0.0183
metab_8092	neg	269.0783	1.1212	0.7033	1.4507	0.0941	0.1855
metab_13220	neg	269.0936	3.5475	0.4414	-0.7338	0.2744	0.4047
metab_14130	neg	269.1035	1.6642	0.8119	1.3439	0.1564	0.2697
metab_6232	pos	269.1065	0.8221	1.0702	-1.1465	0.0023	0.0132
metab_6140	pos	269.1123	1.0180	0.4133	0.4501	0.2723	0.4004
metab_13601	neg	269.1146	2.6046	1.1415	1.6890	0.0099	0.0396
metab_2147	pos	269.1279	3.4251	1.5121	-2.1268	0.0309	0.0802
metab_12582	neg	269.1315	5.9705	0.2309	0.0157	0.2258	0.3503
metab_2169	pos	269.1376	3.5465	0.3957	0.8063	0.2783	0.4072
metab_5339	pos	269.1377	2.5782	1.2013	2.3175	0.0022	0.0129
metab_8869	neg	269.1397	2.6986	1.1221	2.1131	0.0035	0.0190
metab_13955	neg	269.1398	1.9439	1.1758	2.4502	0.0036	0.0195
metab_7558	neg	269.1398	2.1956	0.9168	1.7540	0.0211	0.0654
metab_9351	neg	269.1398	4.3207	0.6185	1.0668	0.1583	0.2721
metab_392	pos	269.1484	1.9169	0.6857	0.8090	0.2089	0.3287
metab_249	pos	269.1599	0.8920	0.3887	-0.4668	0.5224	0.6441
metab_2171	pos	269.1641	3.5465	2.6192	-5.9537	0.0000	0.0003
metab_4565	pos	269.1739	5.8753	1.1764	-3.0787	0.0137	0.0446
metab_4754	pos	269.1740	4.8809	0.7247	-1.8737	0.1098	0.2032
metab_4823	pos	269.1740	4.5184	0.7755	-1.7851	0.0980	0.1867
metab_17	pos	269.1740	2.4530	1.5447	3.4868	0.0013	0.0090
metab_9626	neg	269.1761	5.7585	0.1747	1.4511	0.8969	0.9310
metab_12913	neg	269.1761	4.5391	1.4550	2.8254	0.0136	0.0486
metab_12981	neg	269.1762	4.2702	1.2558	2.2222	0.0213	0.0658
metab_9155	neg	269.1762	3.5813	2.6548	7.2239	0.0014	0.0105
metab_5934	pos	269.1852	1.3720	1.3605	-4.5038	0.0263	0.0716
metab_797	pos	269.1889	8.7161	0.1623	-0.0552	0.6706	0.7678
metab_3845	pos	269.1892	9.2815	0.2753	0.2191	0.4450	0.5756
metab_4789	pos	269.2104	4.6398	2.2535	6.4155	0.0114	0.0394
metab_9771	neg	269.2125	6.5989	0.4005	-1.7083	0.6878	0.7759
metab_624	pos	269.7086	5.0163	1.0863	1.5814	0.0169	0.0515
metab_11131	neg	270.0056	13.9301	0.1510	0.1133	0.4301	0.5550
metab_14438	neg	270.0623	1.3089	0.2815	-0.3726	0.3899	0.5185
metab_436	pos	270.0944	2.2519	0.3353	-0.4171	0.3869	0.5190
metab_5710	pos	270.0964	1.7705	0.2254	-0.6301	0.6235	0.7300
metab_13591	neg	270.0968	2.6206	0.6085	-0.2379	0.2743	0.4047
metab_13878	neg	270.0987	2.0670	0.3409	0.4166	0.2118	0.3345
metab_9317	neg	270.1047	4.1699	0.9441	0.3959	0.1008	0.1951
metab_1597	pos	270.1114	1.5004	0.0017	0.2947	0.9063	0.9400
metab_5080	pos	270.1117	3.4098	0.8990	1.5635	0.0689	0.1443
metab_4901	pos	270.1118	4.1544	0.3616	1.6097	0.5444	0.6638
metab_9389	neg	270.1140	4.5391	0.4221	0.7972	0.3414	0.4745
metab_6166	pos	270.1171	0.9480	0.8889	-0.5280	0.0669	0.1414

metab_5831	pos	270.1327	1.5565	0.6311	0.8613	0.0374	0.0917
metab_5724	pos	270.1327	1.7554	0.7623	1.0077	0.0731	0.1508
metab_5408	pos	270.1327	2.4071	0.5478	0.3338	0.1060	0.1979
metab_1449	pos	270.1328	1.0741	1.2236	1.7522	0.0001	0.0013
metab_8441	neg	270.1351	1.7104	2.1932	6.0372	0.0005	0.0056
metab_13417	neg	270.1352	3.0101	0.3228	-0.2248	0.4641	0.5865
metab_5206	pos	270.1478	2.9806	0.5359	-1.0978	0.1907	0.3061
metab_13082	neg	270.1499	3.9177	1.8325	-4.3807	0.0019	0.0128
metab_12852	neg	270.1503	4.7546	1.7516	-4.2151	0.0020	0.0133
metab_12416	neg	270.1503	6.6803	1.1179	-2.1194	0.0250	0.0735
metab_5939	pos	270.1595	1.3720	0.0620	0.3621	0.9259	0.9521
metab_1861	pos	270.1693	2.2978	0.3453	0.3414	0.3344	0.4684
metab_5256	pos	270.1693	2.8411	0.5502	0.4990	0.1980	0.3155
metab_12878	neg	270.1714	4.6550	0.0193	-0.1990	0.9287	0.9539
metab_14964	neg	270.1827	0.5286	1.0860	-1.0388	0.0347	0.0912
metab_2388	pos	270.2057	4.8509	1.3574	12.0264	0.0247	0.0682
metab_12444	neg	270.2076	6.5512	0.6780	1.6024	0.1223	0.2244
metab_9576	neg	270.2078	5.5322	0.2296	0.2553	0.4121	0.5387
metab_3941	pos	270.2784	8.8466	0.6474	-0.5547	0.0008	0.0063
metab_4967	pos	270.6580	3.8511	0.5465	-0.4854	0.1816	0.2959
metab_7682	neg	270.9436	0.4975	0.5054	-0.4460	0.1101	0.2083
metab_8208	neg	271.0153	1.3231	0.5636	0.1357	0.2530	0.3815
metab_8168	neg	271.0481	1.2805	1.2093	3.3275	0.0124	0.0459
metab_232	pos	271.0681	0.7661	1.5724	2.6638	0.0000	0.0004
metab_14223	neg	271.0706	1.5439	0.2036	-0.2845	0.5627	0.6739
metab_7699	neg	271.0728	3.2637	1.4907	3.9721	0.0105	0.0409
metab_9112	neg	271.0728	3.4470	1.7617	11.6862	0.0093	0.0380
metab_7623	neg	271.0805	0.5286	0.3864	1.1273	0.4359	0.5596
metab_7474	neg	271.0827	1.6642	0.4291	-0.3010	0.2113	0.3341
metab_1402	pos	271.0916	0.8920	0.8694	1.7192	0.0676	0.1425
metab_8154	neg	271.0941	1.2663	0.8152	1.4570	0.0479	0.1140
metab_14719	neg	271.1038	0.7818	0.2198	0.6291	0.6887	0.7767
metab_5825	pos	271.1069	1.5705	0.3184	-0.6108	0.4569	0.5859
metab_13058	neg	271.1093	4.0182	1.1368	-1.7002	0.0086	0.0359
metab_9174	neg	271.1094	3.6479	0.9696	-1.0808	0.0079	0.0338
metab_9125	neg	271.1095	3.4969	1.0701	-1.5742	0.0087	0.0361
metab_13260	neg	271.1190	3.4133	0.9815	1.5018	0.1038	0.1995
metab_8344	neg	271.1303	1.5439	0.0547	0.2875	0.9907	0.9933
metab_5346	pos	271.1435	2.5469	0.5277	-0.5994	0.0555	0.1231
metab_13132	neg	271.1458	3.7658	1.9269	-4.3810	0.0000	0.0005
metab_5275	pos	271.1541	2.7804	1.3419	2.6470	0.0048	0.0212
metab_13153	neg	271.1554	3.7317	1.0373	1.9464	0.0295	0.0818
metab_8866	neg	271.1555	2.6841	1.1056	2.0023	0.0101	0.0402
metab_9131	neg	271.1666	3.5141	0.0461	0.5984	0.8651	0.9075
metab_8949	neg	271.1667	2.8922	0.0268	1.2551	0.9844	0.9890
metab_9718	neg	271.1916	6.3269	1.1824	3.4031	0.0092	0.0377
metab_12912	neg	271.1919	4.5553	0.7524	0.7819	0.1525	0.2646
metab_2394	pos	271.2260	4.8961	0.0963	-0.1285	0.7103	0.7983

metab_11836	neg	271.2282	8.5135	0.5337	1.0313	0.0643	0.1414
metab_3480	pos	271.9156	14.0665	0.5129	-0.4604	0.0098	0.0355
metab_9738	neg	272.0359	6.4073	0.4136	1.0452	0.2610	0.3907
metab_12359	neg	272.0362	6.8887	0.9827	1.8198	0.0101	0.0400
metab_7979	neg	272.0779	0.7818	0.0843	0.1789	0.6322	0.7305
metab_6291	pos	272.0869	0.6682	1.0642	1.7607	0.0729	0.1505
metab_9339	neg	272.0932	4.2702	1.5227	-3.0611	0.0129	0.0470
metab_9144	neg	272.0932	3.5641	1.1643	-1.8763	0.0529	0.1223
metab_5582	pos	272.1120	2.0677	0.3157	0.2228	0.1240	0.2225
metab_8822	neg	272.1143	2.5592	0.6642	-1.6965	0.2095	0.3321
metab_1462	pos	272.1232	1.1024	0.5569	0.9741	0.2072	0.3269
metab_2343	pos	272.1273	4.5335	0.3961	0.5685	0.4249	0.5565
metab_8897	neg	272.1295	2.7624	0.6515	-0.9118	0.3183	0.4505
metab_2137	pos	272.1426	3.3793	1.3290	-2.1054	0.0301	0.0789
metab_369	pos	272.1484	1.7128	0.4553	1.4504	0.4196	0.5513
metab_9040	neg	272.1508	3.1797	0.2884	1.7204	0.6953	0.7815
metab_14125	neg	272.1619	1.6642	0.6469	1.2411	0.0472	0.1132
metab_4700	pos	272.1848	5.0610	0.6127	1.4853	0.3567	0.4909
metab_2369	pos	272.1849	4.6700	0.9275	1.0680	0.1555	0.2630
metab_1912	pos	272.1849	2.4690	0.7476	-0.9534	0.0016	0.0102
metab_9512	neg	272.1870	5.1296	1.2883	-1.8076	0.0141	0.0498
metab_6610	neg	272.9593	0.5126	0.8107	-0.9766	0.0181	0.0590
metab_6389	pos	272.9764	0.5703	1.1610	-1.6894	0.0049	0.0215
metab_1307	pos	273.0475	0.5983	1.2760	-3.5483	0.0994	0.1884
metab_8641	neg	273.0885	2.1311	0.0391	0.1770	0.7752	0.8399
metab_6908	neg	273.1095	1.4427	0.0892	0.0332	0.6720	0.7632
metab_6259	pos	273.1183	0.7801	1.1427	-1.4666	0.0096	0.0350
metab_335	pos	273.1223	1.5144	0.7510	0.6072	0.0660	0.1399
metab_5730	pos	273.1225	1.7412	0.7233	0.6058	0.0352	0.0880
metab_5581	pos	273.1226	2.0677	0.7477	-1.2389	0.0949	0.1823
metab_537	pos	273.1227	3.4399	0.3908	-0.4619	0.1108	0.2044
metab_13700	neg	273.1249	2.4010	1.1825	-1.2672	0.1351	0.2420
metab_9126	neg	273.1250	3.4969	0.9557	-1.2754	0.0132	0.0477
metab_13146	neg	273.1250	3.7488	0.1547	1.2657	0.7181	0.7976
metab_13524	neg	273.1345	2.7624	1.3212	1.9862	0.0059	0.0274
metab_8605	neg	273.1348	2.0358	0.9296	1.5447	0.0160	0.0543
metab_6322	pos	273.1435	0.6262	0.1271	-1.1989	0.8054	0.8712
metab_6428	pos	273.1436	0.5280	0.8908	-1.3578	0.0699	0.1459
metab_6057	pos	273.1437	1.1875	0.2224	0.1033	0.5546	0.6724
metab_13942	neg	273.1460	1.9745	0.5119	1.2510	0.2642	0.3943
metab_14978	neg	273.1571	0.5286	0.0234	0.8433	0.8517	0.8970
metab_1746	pos	273.1587	1.9609	0.9448	1.2130	0.0060	0.0250
metab_1987	pos	273.1591	2.8108	0.3133	-0.2802	0.5182	0.6397
metab_5056	pos	273.1591	3.5011	1.0066	-2.5125	0.0346	0.0870
metab_2244	pos	273.1688	3.9114	3.3271	12.3278	0.0000	0.0000
metab_9437	neg	273.1711	4.7217	0.3970	-0.4383	0.4895	0.6110
metab_9135	neg	273.1712	3.5310	1.0733	2.6240	0.0957	0.1881
metab_5240	pos	273.1801	2.8877	0.1459	0.6502	0.7906	0.8593

metab_1875	pos	273.1801	2.3447	0.7190	1.2609	0.1758	0.2890
metab_14091	neg	273.1823	1.7264	0.0182	0.8062	0.8377	0.8864
metab_1217	pos	273.1858	0.4999	1.1344	1.3625	0.0091	0.0337
metab_1817	pos	273.1913	2.1444	1.0329	1.3988	0.0244	0.0676
metab_7826	neg	273.1936	0.5286	1.0164	1.9644	0.0138	0.0491
metab_9665	neg	273.2075	6.0024	1.3349	2.6864	0.0070	0.0311
metab_10237	neg	273.2346	8.5135	0.7906	1.3307	0.0074	0.0323
metab_11740	neg	273.2347	8.7031	0.1204	0.6788	0.6093	0.7128
metab_2395	pos	273.2528	4.8961	0.1519	-0.1664	0.6180	0.7257
metab_936	pos	273.9159	14.0525	0.2291	0.2782	0.2886	0.4191
metab_8745	neg	273.9786	2.3533	0.3700	0.1255	0.4900	0.6114
metab_14651	neg	274.0571	0.9086	0.7349	2.1894	0.2254	0.3501
metab_14305	neg	274.0724	1.4710	0.1140	1.4626	0.7431	0.8149
metab_8912	neg	274.0724	2.7961	0.7329	-0.9146	0.0899	0.1798
metab_1121	pos	274.0912	0.7801	1.1259	-1.7866	0.0093	0.0342
metab_14471	neg	274.0936	1.2805	0.6113	1.0126	0.0795	0.1647
metab_7925	neg	274.1047	0.6131	0.4685	1.0145	0.2970	0.4276
metab_4885	pos	274.1065	4.2457	1.7882	-5.5963	0.0047	0.0209
metab_1731	pos	274.1067	1.9169	0.6786	-0.6228	0.1227	0.2207
metab_9205	neg	274.1089	3.7658	1.3491	-2.1499	0.0107	0.0415
metab_13611	neg	274.1299	2.5893	0.0180	0.3875	0.8984	0.9320
metab_5784	pos	274.1389	1.6413	1.0262	1.2103	0.0006	0.0057
metab_5008	pos	274.1546	3.6987	0.2147	0.0384	0.6108	0.7204
metab_13195	neg	274.1567	3.6142	0.0326	0.4093	0.9349	0.9579
metab_1546	pos	274.1640	1.3437	1.2515	1.9069	0.0002	0.0027
metab_516	pos	274.1643	3.1957	0.8345	1.2142	0.0246	0.0682
metab_6093	pos	274.1751	1.1164	0.4816	0.5354	0.2713	0.3996
metab_343	pos	274.2004	1.5284	1.6826	6.2509	0.0069	0.0275
metab_2231	pos	274.2005	3.8511	0.5234	0.1480	0.2599	0.3876
metab_5096	pos	274.2005	3.3490	0.4574	-1.0786	0.1897	0.3052
metab_5221	pos	274.2005	2.9501	0.2942	-0.2105	0.1331	0.2342
metab_5499	pos	274.2006	2.2045	0.5080	0.3641	0.1125	0.2063
metab_1257	pos	274.2117	0.5280	1.0593	-2.0730	0.0252	0.0693
metab_4633	pos	274.2732	5.4357	0.3460	-0.4005	0.0744	0.1523
metab_4604	pos	274.2732	5.6473	0.2990	-0.3280	0.1768	0.2899
metab_2557	pos	274.2733	6.0561	0.4437	0.0757	0.1248	0.2237
metab_6403	pos	274.9749	0.5560	1.2585	-2.9038	0.0002	0.0031
metab_8719	neg	275.0203	2.3078	0.9029	-2.6232	0.1209	0.2225
metab_13801	neg	275.0678	2.2119	0.5273	-0.5255	0.2298	0.3548
metab_8219	neg	275.0777	1.3517	1.3723	2.6117	0.0013	0.0099
metab_6789	neg	275.0889	0.7818	0.3985	0.0876	0.1659	0.2815
metab_5422	pos	275.0908	2.3908	2.5223	7.7115	0.0000	0.0003
metab_13648	neg	275.0930	2.5113	1.7960	3.9728	0.0048	0.0236
metab_13521	neg	275.0930	2.7624	1.2103	3.0509	0.0104	0.0407
metab_9177	neg	275.0931	3.6479	1.5561	4.3993	0.0048	0.0239
metab_9342	neg	275.0964	4.2870	1.1666	4.4262	0.1400	0.2486
metab_5548	pos	275.1018	2.1290	0.2127	0.6218	0.7358	0.8182
metab_1923	pos	275.1020	2.5158	0.3208	-0.3332	0.3547	0.4894

metab_7621	neg	275.1041	2.6678	0.5272	0.1488	0.1813	0.2994
metab_7580	neg	275.1042	2.3078	0.3145	0.0655	0.3475	0.4804
metab_6910	neg	275.1043	1.4710	0.8031	-0.8872	0.0713	0.1518
metab_14249	neg	275.1140	1.5297	2.6968	6.1532	0.0007	0.0068
metab_265	pos	275.1229	1.1024	1.3052	2.8229	0.0058	0.0242
metab_14891	neg	275.1251	0.5571	0.3129	-0.2131	0.5362	0.6510
metab_209	pos	275.1340	0.6262	1.7394	3.7368	0.0256	0.0701
metab_4994	pos	275.1383	3.7447	0.6285	0.9748	0.3634	0.4971
metab_5420	pos	275.1383	2.3908	0.6449	-0.8997	0.1493	0.2555
metab_13408	neg	275.1504	3.0260	1.6629	3.4216	0.0024	0.0149
metab_13539	neg	275.1504	2.7318	0.8384	1.2425	0.0341	0.0902
metab_12299	neg	275.1690	7.0322	0.3801	-0.6848	0.4070	0.5344
metab_5230	pos	275.1746	2.9192	1.6027	3.3311	0.0054	0.0231
metab_9537	neg	275.1867	5.2741	0.4711	-0.5256	0.4698	0.5922
metab_13097	neg	275.1868	3.8839	1.3860	2.8562	0.0171	0.0566
metab_5748	pos	275.1957	1.7128	0.5323	-0.6529	0.1896	0.3051
metab_2621	pos	275.1997	6.7366	0.4898	-1.1970	0.2085	0.3283
metab_180	pos	275.2071	0.5140	0.9802	1.3412	0.0036	0.0176
metab_15041	neg	275.9063	0.4975	0.4291	-0.0668	0.0603	0.1348
metab_1326	pos	276.0472	0.6402	1.2209	-2.4832	0.0494	0.1131
metab_6896	neg	276.0551	1.4427	1.0774	-3.0043	0.0597	0.1337
metab_322	pos	276.0684	1.4429	1.4984	16.1933	0.0009	0.0069
metab_14653	neg	276.0727	0.9086	0.3309	0.9991	0.3403	0.4734
metab_1777	pos	276.0859	2.0360	0.1597	-0.8813	0.7149	0.8020
metab_7668	neg	276.0880	2.9931	0.1860	0.9639	0.8240	0.8756
metab_1357	pos	276.1007	0.7801	0.1609	0.0975	0.6312	0.7364
metab_8152	neg	276.1090	1.2663	0.7010	1.0075	0.0383	0.0980
metab_5885	pos	276.1214	1.4569	0.3430	0.6685	0.5644	0.6810
metab_5131	pos	276.1223	3.2274	1.1324	-2.0164	0.0049	0.0216
metab_4984	pos	276.1224	3.7753	1.2576	-2.0622	0.0260	0.0710
metab_9506	neg	276.1242	5.1131	1.2636	-2.1207	0.0544	0.1248
metab_13099	neg	276.1244	3.8678	0.5097	0.4806	0.3761	0.5067
metab_5685	pos	276.1335	1.8432	0.1849	0.1499	0.7191	0.8055
metab_6	pos	276.1433	0.9480	1.1640	-2.3989	0.0752	0.1534
metab_8072	neg	276.1454	1.0499	1.0699	-2.2152	0.0587	0.1321
metab_8622	neg	276.1455	2.0670	0.9852	-1.1948	0.0229	0.0691
metab_6903	neg	276.1455	1.4568	1.1669	-2.2490	0.0246	0.0727
metab_2182	pos	276.1700	3.6073	0.0037	0.1441	0.8951	0.9325
metab_1870	pos	276.1799	2.3292	0.9439	1.2969	0.0036	0.0174
metab_2475	pos	276.2132	5.4662	1.6462	4.1135	0.0999	0.1890
metab_1932	pos	276.2428	2.5627	0.4163	0.3227	0.2128	0.3339
metab_3164	pos	276.2678	10.2085	0.3470	-0.3243	0.2632	0.3908
metab_2559	pos	276.2798	6.0705	0.8331	0.6478	0.0090	0.0336
metab_10862	neg	276.8679	14.2070	0.4658	-0.1965	0.0307	0.0841
metab_1120	pos	277.0312	0.7801	0.4303	-0.1990	0.1512	0.2578
metab_5160	pos	277.0389	3.1346	0.4582	1.5033	0.4223	0.5538
metab_14809	neg	277.0600	0.5991	1.3680	3.7825	0.0166	0.0556
metab_6337	pos	277.0676	0.6123	0.2303	0.6314	0.6783	0.7742

metab_8886	neg	277.0720	2.7475	1.6911	3.7590	0.0216	0.0664
metab_14169	neg	277.0757	1.6028	1.8441	4.4082	0.0002	0.0027
metab_8157	neg	277.0833	1.2663	0.5179	1.0130	0.2332	0.3586
metab_456	pos	277.1065	2.5003	1.6494	3.5518	0.0004	0.0041
metab_13517	neg	277.1084	2.7792	0.0837	0.3295	0.9469	0.9650
metab_9375	neg	277.1120	4.4386	0.3243	0.3697	0.4044	0.5326
metab_14565	neg	277.1124	1.1069	0.0907	0.6319	0.9369	0.9586
metab_356	pos	277.1174	1.6272	0.2323	0.2341	0.5346	0.6553
metab_439	pos	277.1176	2.2978	0.4250	-0.3147	0.2410	0.3659
metab_8739	neg	277.1195	2.3533	0.2179	0.4286	0.7130	0.7942
metab_11722	neg	277.1230	8.7031	1.3118	-11.7522	0.0102	0.0403
metab_11817	neg	277.1230	8.5455	1.8090	-12.6566	0.0001	0.0016
metab_14349	neg	277.1294	1.4427	0.7051	1.0937	0.0366	0.0946
metab_14180	neg	277.1303	1.5885	1.5241	4.2819	0.0095	0.0384
metab_1347	pos	277.1386	0.7241	0.3716	-0.7814	0.4519	0.5816
metab_12217	neg	277.1445	7.3180	0.0171	0.8573	0.8929	0.9282
metab_9785	neg	277.1448	6.6632	0.5483	0.6406	0.1033	0.1986
metab_440	pos	277.1539	2.3133	0.2606	-0.1561	0.4238	0.5552
metab_5704	pos	277.1539	1.7845	1.6202	2.9930	0.0002	0.0029
metab_1031	pos	277.1539	1.9169	0.8278	1.1104	0.0073	0.0286
metab_13653	neg	277.1560	2.5113	0.8609	-1.1700	0.0822	0.1683
metab_13744	neg	277.1560	2.3227	0.7689	-0.9798	0.1117	0.2108
metab_4729	pos	277.2154	4.9413	0.2058	-0.4730	0.6321	0.7371
metab_4333	pos	277.2154	7.0670	0.4587	-0.5358	0.1603	0.2690
metab_12479	neg	277.2170	6.4073	0.3232	0.9711	0.3556	0.4884
metab_10900	neg	277.8610	15.9128	0.4385	-0.1274	0.0147	0.0513
metab_14096	neg	278.0672	1.7264	0.4243	0.8438	0.3381	0.4712
metab_7449	neg	278.0673	1.5439	0.0881	0.3691	0.9052	0.9371
metab_2041	pos	278.1017	2.9954	0.1642	0.6947	0.6167	0.7247
metab_6859	neg	278.1037	1.2663	1.2621	-1.7812	0.0083	0.0348
metab_13887	neg	278.1037	2.0516	0.5523	-0.4395	0.1882	0.3073
metab_8847	neg	278.1037	2.6357	0.8182	-0.7292	0.0204	0.0638
metab_14222	neg	278.1149	1.5439	0.5774	1.1510	0.2351	0.3605
metab_1946	pos	278.1211	2.6090	0.7430	-0.2482	0.0819	0.1636
metab_205	pos	278.1226	0.6123	1.2278	-2.1134	0.0559	0.1239
metab_14732	neg	278.1247	0.7678	0.9470	-2.0407	0.1301	0.2349
metab_1457	pos	278.1312	1.1024	0.1559	0.5889	0.8137	0.8771
metab_8596	neg	278.1400	2.0202	0.4112	1.9625	0.5349	0.6503
metab_8891	neg	278.1400	2.7475	0.6298	-1.0767	0.1705	0.2870
metab_12885	neg	278.1400	4.6380	1.2826	-1.8423	0.0159	0.0542
metab_5773	pos	278.1407	1.6693	0.5862	-0.8356	0.0993	0.1883
metab_1254	pos	278.1421	0.5280	0.1230	-0.1575	0.8325	0.8899
metab_6167	pos	278.1484	0.9480	0.9892	-2.2484	0.0853	0.1686
metab_6039	pos	278.1855	1.2016	1.4729	-4.5405	0.0108	0.0378
metab_993	pos	278.2471	6.3885	0.1255	0.0904	0.6757	0.7719
metab_2798	pos	278.2471	7.8683	0.3744	-0.4154	0.0946	0.1821
metab_2040	pos	278.2584	2.9954	0.0434	-0.0840	0.9723	0.9837
metab_6695	neg	278.8535	0.3798	0.0653	0.0986	0.7559	0.8242

metab_7172	neg	278.8535	14.2390	0.3922	-0.0731	0.0465	0.1119
metab_10780	neg	278.8971	14.0438	0.3463	-0.0362	0.0440	0.1077
metab_14971	neg	278.9251	0.5286	1.1462	-1.2196	0.0735	0.1547
metab_6698	neg	278.9445	0.5126	0.5042	-0.1923	0.0388	0.0987
metab_7556	neg	279.0150	2.1311	0.3575	-0.0476	0.6918	0.7791
metab_6766	neg	279.0487	0.6131	0.5015	-0.0079	0.2339	0.3594
metab_14118	neg	279.0513	1.6797	0.8093	1.0568	0.1577	0.2714
metab_8034	neg	279.0722	0.9508	0.5194	2.4564	0.3560	0.4888
metab_8218	neg	279.0850	1.3517	0.8341	1.1956	0.0110	0.0422
metab_13155	neg	279.0878	3.7148	0.2824	-0.2135	0.4575	0.5806
metab_13455	neg	279.0987	2.9092	2.0274	7.2958	0.0011	0.0091
metab_8401	neg	279.0990	1.6335	0.7509	-0.6847	0.0337	0.0896
metab_13949	neg	279.1240	1.9596	0.5601	-0.2765	0.0794	0.1646
metab_8725	neg	279.1241	2.3227	0.7089	-0.6497	0.0307	0.0841
metab_8858	neg	279.1241	2.6678	0.9502	-0.9846	0.0038	0.0203
metab_443	pos	279.1332	2.3600	0.3742	-0.4831	0.3499	0.4852
metab_8704	neg	279.1350	2.2754	0.9030	-0.7308	0.1131	0.2123
metab_9140	neg	279.1352	3.5475	0.9249	-1.4225	0.1431	0.2526
metab_14407	neg	279.1353	1.3517	0.5217	-0.4185	0.2064	0.3284
metab_574	pos	279.1584	4.0479	0.4047	-0.3759	0.1854	0.3003
metab_3879	pos	279.1585	9.1302	0.3011	-0.2313	0.1136	0.2080
metab_5009	pos	279.1585	3.6987	1.3383	2.4453	0.0007	0.0062
metab_530	pos	279.1696	2.3292	0.3847	-0.5098	0.3437	0.4785
metab_5361	pos	279.1696	2.5158	0.5934	-0.9730	0.1246	0.2234
metab_5232	pos	279.2060	2.9028	0.1955	0.4239	0.7281	0.8123
metab_706	pos	279.2310	7.0228	0.4161	-0.3785	0.2186	0.3408
metab_634	pos	279.2311	5.2431	0.4157	0.7337	0.2677	0.3953
metab_992	pos	279.2311	6.4035	0.0781	0.4698	0.7844	0.8552
metab_106	pos	279.2311	9.6968	0.3068	-0.0562	0.4471	0.5769
metab_2946	pos	279.2311	8.7303	0.1259	0.3974	0.6986	0.7896
metab_12013	neg	279.2332	7.9908	0.8753	-0.7681	0.0077	0.0330
metab_7316	neg	279.2332	8.7031	1.2157	-1.3170	0.0001	0.0020
metab_15148	neg	279.8611	0.0136	0.2679	0.0516	0.2000	0.3207
metab_13695	neg	279.9575	2.4010	0.7267	-1.3460	0.0988	0.1922
metab_10872	neg	279.9888	14.2876	0.0656	0.3713	0.8374	0.8863
metab_6878	neg	280.0466	1.2948	0.2359	0.6672	0.6309	0.7294
metab_6572	neg	280.0829	2.4010	0.5058	-0.3338	0.1157	0.2154
metab_6602	neg	280.0830	2.1311	0.1995	0.0179	0.4209	0.5469
metab_6392	pos	280.0912	0.5703	2.0775	4.9624	0.0016	0.0099
metab_14833	neg	280.1039	0.5991	1.0677	-1.6819	0.0713	0.1518
metab_69	pos	280.1173	2.0677	0.2076	-0.2998	0.3668	0.5003
metab_7566	neg	280.1194	2.1798	0.3582	0.9014	0.4119	0.5386
metab_14373	neg	280.1306	1.4115	0.1926	0.7656	0.7129	0.7942
metab_394	pos	280.1536	1.9463	1.3135	2.4120	0.0052	0.0226
metab_6053	pos	280.1648	1.1875	0.1081	0.1630	0.7501	0.8290
metab_1654	pos	280.1757	1.6413	0.3846	-0.3915	0.4285	0.5600
metab_472	pos	280.1797	2.6561	0.3936	-0.4215	0.3705	0.5044
metab_5535	pos	280.1899	2.1444	1.8394	3.1201	0.0055	0.0235

metab_1910	pos	280.1900	2.4530	2.4884	4.1968	0.0022	0.0128
metab_536	pos	280.1901	3.4399	2.4820	6.3544	0.0000	0.0000
metab_4101	pos	280.2627	8.2143	0.3395	-0.3282	0.1250	0.2238
metab_14892	neg	280.9948	0.5571	0.2934	0.8540	0.6300	0.7288
metab_10653	neg	280.9972	10.3687	0.2767	-0.0249	0.3930	0.5214
metab_10586	neg	280.9973	9.9274	0.8309	1.4630	0.0053	0.0255
metab_14213	neg	281.0306	1.5586	0.6310	1.3203	0.3558	0.4885
metab_199	pos	281.0626	0.5703	1.4252	-13.7682	0.0002	0.0026
metab_7973	neg	281.0880	0.7818	1.0476	-1.0167	0.0003	0.0037
metab_9234	neg	281.0938	3.8506	0.6134	-0.5798	0.1716	0.2881
metab_1065	pos	281.0984	1.4714	0.2862	0.2552	0.3801	0.5126
metab_8187	neg	281.0992	1.3089	1.0150	-1.0829	0.0002	0.0033
metab_4680	pos	281.1011	5.1830	0.1610	-0.1980	0.4657	0.5932
metab_5787	pos	281.1122	1.6413	0.9574	-1.3289	0.0141	0.0455
metab_6024	pos	281.1123	1.2162	0.1865	0.0747	0.5579	0.6749
metab_8331	neg	281.1145	1.5297	0.4558	-0.3533	0.1606	0.2751
metab_14554	neg	281.1146	1.1355	0.1903	0.7654	0.8326	0.8827
metab_8086	neg	281.1256	1.0927	0.0588	0.6359	0.8262	0.8776
metab_4578	pos	281.1375	5.7846	0.0907	-0.0333	0.4853	0.6106
metab_13302	neg	281.1398	3.2972	1.9499	4.8359	0.0001	0.0021
metab_9243	neg	281.1398	3.8839	2.3849	6.7561	0.0002	0.0027
metab_1653	pos	281.1487	1.6413	1.2888	1.8722	0.0000	0.0005
metab_5973	pos	281.1487	1.3153	0.1074	0.1440	0.8361	0.8922
metab_2168	pos	281.1487	3.5465	0.1339	-0.7718	0.6813	0.7764
metab_2945	pos	281.1528	8.7161	1.3018	12.1812	0.0061	0.0253
metab_2364	pos	281.1569	4.6398	1.4821	-2.3475	0.0006	0.0053
metab_659	pos	281.1740	5.9049	1.6873	3.3898	0.0044	0.0201
metab_9272	neg	281.1761	3.9850	1.5995	5.1957	0.0016	0.0113
metab_12848	neg	281.1762	4.7709	0.8680	1.9735	0.0936	0.1848
metab_5906	pos	281.1853	1.4289	1.0679	11.6319	0.0154	0.0484
metab_9782	neg	281.2124	6.6480	0.6233	0.2447	0.3117	0.4430
metab_10101	neg	281.2399	7.9908	1.5812	-2.4947	0.0030	0.0171
metab_3657	pos	281.2465	10.1175	0.5710	0.9710	0.3559	0.4905
metab_26	pos	281.2466	5.9949	0.5313	1.7006	0.4131	0.5455
metab_4160	pos	281.2467	7.9835	0.6090	-0.5089	0.1509	0.2574
metab_11849	neg	281.2487	8.4667	0.1466	0.4309	0.7264	0.8030
metab_10419	neg	281.2488	9.1200	0.5745	-0.0380	0.0801	0.1654
metab_15037	neg	281.9178	0.4975	0.3819	-0.1848	0.2354	0.3608
metab_7998	neg	282.0024	0.8240	1.0036	1.7492	0.0245	0.0724
metab_7528	neg	282.0446	1.9596	0.3001	1.6137	0.6541	0.7489
metab_6343	pos	282.0480	0.6123	1.0827	-1.5321	0.0003	0.0033
metab_6898	neg	282.0622	1.5586	0.1110	0.3211	0.8871	0.9243
metab_14575	neg	282.0622	1.0785	0.9548	1.4015	0.0075	0.0325
metab_1301	pos	282.0664	0.5983	0.4624	-0.3718	0.3203	0.4533
metab_14639	neg	282.0736	0.9368	0.7275	5.8204	0.4951	0.6165
metab_1317	pos	282.0841	0.6262	1.2313	4.3175	0.0006	0.0052
metab_7600	neg	282.0883	2.4010	0.0451	0.5312	0.9328	0.9561
metab_1019	pos	282.0965	2.1290	0.2890	-0.2945	0.3538	0.4886

metab_18	pos	282.0965	2.4071	0.4603	-0.5554	0.2044	0.3239
metab_13568	neg	282.0985	2.6678	0.1877	-0.4040	0.6024	0.7069
metab_14200	neg	282.0985	1.5736	0.0271	0.1967	0.7762	0.8402
metab_12945	neg	282.1139	4.3882	1.2608	-2.2048	0.0073	0.0319
metab_208	pos	282.1178	0.6262	1.0988	-1.8615	0.1269	0.2259
metab_1436	pos	282.1187	1.0180	0.0101	-0.0999	0.9962	0.9977
metab_6614	neg	282.1211	0.5286	0.6480	-0.4295	0.0448	0.1091
metab_1997	pos	282.1234	2.8411	0.9793	-1.3770	0.0519	0.1172
metab_379	pos	282.1326	1.7705	0.1025	-0.1124	0.8192	0.8814
metab_59	pos	282.1327	2.1897	0.0730	-0.0643	0.9121	0.9430
metab_5331	pos	282.1328	2.5938	0.8770	-1.0353	0.0020	0.0119
metab_5996	pos	282.1328	1.2585	0.3837	0.0484	0.1422	0.2464
metab_1127	pos	282.1438	0.6262	0.2923	-0.2277	0.6057	0.7154
metab_2911	pos	282.1524	8.5989	0.3533	-0.4123	0.1095	0.2028
metab_4559	pos	282.2056	5.9049	1.2057	2.3211	0.0585	0.1278
metab_5189	pos	282.2057	3.0264	0.6924	1.9558	0.2177	0.3397
metab_3051	pos	282.2664	9.4049	1.0973	1.7031	0.0093	0.0341
metab_789	pos	282.2783	8.6132	0.3884	-0.3811	0.0672	0.1419
metab_3987	pos	282.4053	8.6284	0.4266	-0.4298	0.0545	0.1216
metab_14044	neg	283.0581	1.7885	1.4183	1.0421	0.0406	0.0531
metab_1221	pos	283.0617	0.4999	1.0379	-1.8541	0.0065	0.0264
metab_6808	neg	283.0687	0.9227	0.5036	-1.3745	0.4862	0.6080
metab_7569	neg	283.0730	2.2119	0.0281	0.6292	0.8222	0.8740
metab_8494	neg	283.0942	1.8046	0.3309	0.7047	0.4936	0.6147
metab_6119	pos	283.1028	1.0601	0.7422	-0.6320	0.1493	0.2555
metab_6852	neg	283.1036	0.5991	1.6066	-2.6896	0.0002	0.0036
metab_377	pos	283.1068	1.7705	0.3843	-1.1161	0.4305	0.5619
metab_13649	neg	283.1190	2.5113	0.7710	1.7736	0.0821	0.1683
metab_8698	neg	283.1191	2.2595	0.1864	0.9157	0.7161	0.7964
metab_6091	pos	283.1278	1.1306	0.5089	0.5671	0.0978	0.1864
metab_6199	pos	283.1279	0.8780	1.2797	2.5003	0.0036	0.0176
metab_6914	neg	283.1302	1.4856	0.3252	-0.1806	0.3005	0.4308
metab_270	pos	283.1392	1.0741	0.6424	0.6858	0.1147	0.2098
metab_505	pos	283.1531	3.0422	2.0544	5.2470	0.0000	0.0008
metab_1837	pos	283.1532	2.2209	2.3211	5.3370	0.0000	0.0002
metab_9552	neg	283.1554	5.3546	1.2645	2.0443	0.0000	0.0005
metab_6972	neg	283.1554	4.3380	1.0211	1.5408	0.0001	0.0026
metab_3440	pos	283.1579	14.2127	0.0823	-0.1268	0.6927	0.7851
metab_552	pos	283.1645	2.2209	0.1898	-0.8187	0.6897	0.7828
metab_3965	pos	283.1679	8.7161	1.2413	1.7814	0.0002	0.0024
metab_2310	pos	283.1682	4.3512	0.6938	2.3473	0.2293	0.3529
metab_5849	pos	283.1741	1.5284	0.4558	0.3788	0.0527	0.1186
metab_3430	pos	283.1743	14.3315	0.2155	-0.1793	0.1265	0.2253
metab_1195	pos	283.1743	0.2886	0.1743	-0.1660	0.2479	0.3732
metab_1552	pos	283.1749	1.3579	0.5739	0.5494	0.0734	0.1512
metab_139	pos	283.1895	5.5260	1.2065	6.8196	0.0502	0.1144
metab_12507	neg	283.1916	6.3269	1.2325	-2.9290	0.1027	0.1978
metab_9593	neg	283.1918	5.6128	0.3590	-0.2307	0.5756	0.6849

metab_9545	neg	283.1918	5.3228	0.1816	0.3157	0.7601	0.8280
metab_12832	neg	283.1918	4.8371	1.1243	2.7289	0.0979	0.1912
metab_10148	neg	283.2283	8.1307	1.5851	4.4919	0.0001	0.0019
metab_11621	neg	283.2643	8.9903	0.8206	-0.3466	0.0822	0.1683
metab_6797	neg	284.0180	0.8662	1.9283	-2.5795	0.0003	0.0036
metab_6513	pos	284.0458	0.4999	1.0072	-1.6586	0.0127	0.0423
metab_13673	neg	284.0779	2.4651	1.4839	2.4282	0.0127	0.0465
metab_8939	neg	284.0932	2.8606	0.0112	2.6224	0.8320	0.8822
metab_9105	neg	284.0932	3.4133	0.7600	2.5939	0.2741	0.4046
metab_8665	neg	284.1047	2.1798	0.6800	2.3106	0.3668	0.4985
metab_5450	pos	284.1120	2.3133	0.4529	-1.0414	0.3567	0.4909
metab_1473	pos	284.1120	1.1306	0.6265	0.5618	0.1815	0.2958
metab_1081	pos	284.1233	1.3153	0.3725	1.3000	0.6839	0.7781
metab_8087	neg	284.1255	1.0927	1.1251	2.9825	0.0841	0.1710
metab_8134	neg	284.1256	1.2236	0.1037	0.7100	0.9518	0.9687
metab_1810	pos	284.1271	2.1290	1.5345	10.0188	0.0062	0.0255
metab_4853	pos	284.1273	4.3818	1.4517	-3.0349	0.0049	0.0216
metab_5854	pos	284.1484	1.5284	1.3747	-2.1509	0.0008	0.0064
metab_13115	neg	284.1510	3.8164	0.4511	1.6612	0.5943	0.7011
metab_5962	pos	284.1595	1.3293	1.0674	1.3988	0.0584	0.1276
metab_8466	neg	284.1620	1.7419	0.2265	1.2668	0.6617	0.7557
metab_12323	neg	284.1657	6.9834	0.5249	-0.7515	0.3312	0.4640
metab_5042	pos	284.1847	3.5620	0.5400	0.1807	0.4199	0.5514
metab_4843	pos	284.1849	4.4272	1.0546	1.0020	0.1048	0.1962
metab_2265	pos	284.1849	4.0338	1.4040	1.9271	0.0004	0.0041
metab_2376	pos	284.1849	4.7156	1.1478	1.1866	0.0946	0.1821
metab_9529	neg	284.1870	5.2257	0.2677	-1.0225	0.6129	0.7159
metab_9464	neg	284.1871	4.8371	0.5059	0.6851	0.5948	0.7016
metab_376	pos	284.2112	1.7554	1.0332	-1.2127	0.0026	0.0145
metab_2224	pos	284.2214	3.7908	1.0251	0.4609	0.1682	0.2789
metab_4003	pos	284.2848	8.6132	0.3784	-0.3706	0.0745	0.1524
metab_3853	pos	284.2852	9.2504	0.2149	-0.2520	0.4235	0.5550
metab_3885	pos	284.2939	9.1140	0.3064	-0.3991	0.2658	0.3939
metab_6249	pos	285.0475	0.7801	0.7768	-0.8822	0.0581	0.1269
metab_14330	neg	285.0622	1.4568	0.5508	0.7469	0.1712	0.2877
metab_9027	neg	285.0814	3.1115	1.1587	7.1830	0.1941	0.3143
metab_6176	pos	285.0821	0.9200	0.2760	-1.3611	0.7113	0.7992
metab_1835	pos	285.0862	2.2045	0.2033	-0.3636	0.6675	0.7651
metab_8865	neg	285.0885	2.6841	0.1597	0.3336	0.5387	0.6533
metab_9204	neg	285.0885	3.7658	0.6328	-0.5274	0.2359	0.3613
metab_8718	neg	285.0886	2.3078	0.5903	-0.6250	0.1489	0.2598
metab_15009	neg	285.0946	0.5126	1.4477	-2.4704	0.0171	0.0566
metab_7508	neg	285.0985	1.8205	0.2210	0.3666	0.4405	0.5645
metab_1420	pos	285.1074	0.9620	1.3913	2.2969	0.0037	0.0179
metab_6873	neg	285.1095	1.3373	0.0436	0.3610	0.9290	0.9539
metab_1698	pos	285.1224	1.7554	0.2787	-0.3386	0.5176	0.6393
metab_5060	pos	285.1226	3.5011	1.1579	-1.9311	0.0259	0.0708
metab_6944	neg	285.1249	4.1364	0.6625	-0.6174	0.0915	0.1819

metab_5474	pos	285.1323	2.2519	0.6799	2.0209	0.2327	0.3566
metab_6222	pos	285.1437	0.836	1.7046	3.7113	0.0262	0.0394
metab_484	pos	285.1437	2.7653	0.2711	0.4457	0.6186	0.7261
metab_1640	pos	285.1439	1.5987	0.5162	-0.6280	0.0679	0.1429
metab_8600	neg	285.1457	2.0202	0.5852	0.8432	0.1184	0.2189
metab_5267	pos	285.1530	2.8108	0.3078	-0.2771	0.1751	0.2881
metab_3447	pos	285.1531	14.1820	0.1425	-0.1569	0.4168	0.5488
metab_5438	pos	285.1590	2.3447	0.6062	-0.5725	0.0063	0.0258
metab_2011	pos	285.1688	2.8877	1.8841	13.0128	0.0001	0.0014
metab_1831	pos	285.1688	2.1897	2.3523	7.2709	0.0022	0.0129
metab_8917	neg	285.1711	2.8123	1.4006	2.3776	0.0070	0.0309
metab_12917	neg	285.1711	4.5391	1.2621	2.0485	0.0329	0.0883
metab_8983	neg	285.1712	2.9931	1.3596	3.1213	0.0068	0.0304
metab_13130	neg	285.1712	3.7830	2.1673	5.4759	0.0027	0.0159
metab_1488	pos	285.1800	1.1731	0.4167	-1.0332	0.3805	0.5128
metab_12857	neg	285.1822	4.7384	0.7450	3.1726	0.2097	0.3323
metab_13240	neg	285.1823	3.4969	0.6889	1.2215	0.2080	0.3305
metab_2825	pos	285.1840	8.0988	0.2156	0.1750	0.6608	0.7593
metab_2893	pos	285.1841	8.4504	0.4652	0.3210	0.1797	0.2936
metab_4809	pos	285.2052	4.5785	0.8787	1.1699	0.2569	0.3838
metab_9450	neg	285.2074	4.7709	1.7751	4.0202	0.0269	0.0772
metab_12628	neg	285.2075	5.7751	0.5254	-0.3986	0.2238	0.3481
metab_11669	neg	285.2439	8.8286	0.9339	1.3267	0.0019	0.0127
metab_11809	neg	285.2439	8.5768	0.8832	1.1524	0.0369	0.0953
metab_6693	neg	285.9106	0.4307	0.3495	-0.0938	0.0837	0.1706
metab_7174	neg	285.9106	14.2390	0.3925	-0.0786	0.0319	0.0863
metab_13687	neg	286.0153	2.4338	0.3257	0.1159	0.5147	0.6331
metab_8407	neg	286.0394	1.6335	0.9555	-2.4969	0.0987	0.1922
metab_8717	neg	286.0396	2.3078	0.7728	-0.2329	0.2915	0.4227
metab_14165	neg	286.0724	1.6028	0.2090	0.4824	0.6316	0.7300
metab_13270	neg	286.0725	3.3803	0.8093	-1.1215	0.1088	0.2066
metab_7659	neg	286.0725	2.8768	1.0852	-2.0521	0.0584	0.1317
metab_8272	neg	286.0936	1.4568	0.5949	0.9047	0.0705	0.1506
metab_5245	pos	286.1066	2.8725	0.4855	1.1299	0.4007	0.5339
metab_5075	pos	286.1067	3.4251	1.0538	2.8187	0.0572	0.1258
metab_12863	neg	286.1088	4.7217	1.1997	-1.9291	0.0170	0.0565
metab_9106	neg	286.1089	3.4133	1.2748	3.0911	0.0142	0.0501
metab_14869	neg	286.1146	0.5711	0.2735	-0.1358	0.3706	0.5020
metab_5520	pos	286.1182	2.1746	0.4554	0.5874	0.3552	0.4899
metab_6326	pos	286.1211	0.6262	0.3225	-0.2417	0.2745	0.4027
metab_6207	pos	286.1211	0.8640	0.0002	0.5222	0.8725	0.9196
metab_8309	neg	286.1412	1.5012	0.7498	1.9704	0.1577	0.2714
metab_8214	neg	286.1412	1.3373	0.6093	2.1266	0.3081	0.4390
metab_1687	pos	286.1429	1.7271	1.6686	3.3982	0.0003	0.0038
metab_13291	neg	286.1452	3.3144	1.3116	-3.0127	0.0201	0.0633
metab_12723	neg	286.1453	5.3388	1.1287	-2.4591	0.0350	0.0916
metab_1318	pos	286.1500	0.6262	1.0378	2.8401	0.0325	0.0833
metab_13228	neg	286.1665	3.5310	0.3920	1.2259	0.5429	0.6565

metab_8377	neg	286.1775	1.5736	0.0016	0.2102	0.9789	0.9857
metab_13875	neg	286.1776	2.0670	1.7886	4.2518	0.0003	0.0042
metab_8077	neg	286.1889	1.0642	0.9762	10.7586	0.0151	0.0522
metab_2470	pos	286.2005	5.4056	0.2627	0.1048	0.7113	0.7992
metab_2822	pos	286.2732	8.0700	0.1845	0.5770	0.7302	0.8142
metab_3011	pos	286.3009	9.1302	0.0222	-0.1317	0.8512	0.9044
metab_2924	pos	286.3089	8.6132	0.0529	-0.7801	0.8580	0.9088
metab_29	pos	286.9577	0.4999	0.9035	-1.4224	0.0131	0.0432
metab_8374	neg	287.0235	1.5736	1.0610	-4.2249	0.0871	0.1758
metab_14331	neg	287.0499	1.4568	1.8777	-4.4482	0.0004	0.0044
metab_14113	neg	287.0501	1.6948	1.3528	-2.0580	0.0033	0.0182
metab_1163	pos	287.0567	0.5140	1.0701	1.6435	0.0188	0.0555
metab_7457	neg	287.0777	1.5736	1.1262	1.7863	0.0006	0.0062
metab_7628	neg	287.0852	0.5286	1.6469	3.2929	0.0016	0.0114
metab_14497	neg	287.0888	1.2521	0.5177	0.9508	0.1663	0.2820
metab_6590	neg	287.0985	0.5991	1.3177	-1.6720	0.0000	0.0012
metab_1661	pos	287.1019	1.6693	0.0811	0.1671	0.9701	0.9818
metab_5305	pos	287.1020	2.6869	0.3330	-0.1879	0.3252	0.4591
metab_8636	neg	287.1042	2.1153	0.6028	-0.6062	0.2426	0.3688
metab_275	pos	287.1229	1.1164	0.1039	0.1699	0.8470	0.9016
metab_7498	neg	287.1251	1.8046	0.2270	0.3039	0.5692	0.6798
metab_6874	neg	287.1252	1.3089	0.3861	1.0718	0.3902	0.5186
metab_393	pos	287.1381	1.9309	0.3437	0.2450	0.3002	0.4312
metab_4904	pos	287.1382	4.1387	0.7314	-1.0325	0.0837	0.1662
metab_13070	neg	287.1406	3.9850	0.4150	1.7952	0.5856	0.6936
metab_8979	neg	287.1407	2.9931	1.0003	-1.5819	0.1405	0.2493
metab_5284	pos	287.1478	2.7341	0.3937	0.8679	0.5537	0.6719
metab_13386	neg	287.1502	3.0774	1.4218	3.3330	0.0090	0.0370
metab_8894	neg	287.1504	2.7624	1.1093	2.4241	0.0677	0.1463
metab_8613	neg	287.1505	2.0516	2.0500	4.8247	0.0006	0.0064
metab_6047	pos	287.1593	1.1875	1.1352	3.1082	0.0154	0.0484
metab_13754	neg	287.1615	2.3078	0.3908	1.3038	0.4111	0.5384
metab_5478	pos	287.1703	2.2361	0.6555	1.1936	0.1594	0.2679
metab_4990	pos	287.1747	3.7447	1.9172	-2.2921	0.0283	0.0757
metab_6989	neg	287.1867	5.4844	0.4678	1.1723	0.2965	0.4273
metab_8861	neg	287.1867	2.6678	1.8960	4.9883	0.0020	0.0131
metab_13105	neg	287.1868	3.8506	1.3947	2.9374	0.0020	0.0131
metab_9020	neg	287.1868	3.0942	0.6292	1.9591	0.2924	0.4238
metab_540	pos	287.1957	3.5011	0.7991	0.7470	0.1758	0.2890
metab_12619	neg	287.2231	5.8074	0.0461	0.7993	0.8555	0.8997
metab_9597	neg	287.2231	5.6292	0.6178	1.9356	0.1091	0.2069
metab_12579	neg	287.2231	5.9866	0.6403	1.2338	0.0581	0.1312
metab_9550	neg	287.2231	5.3546	0.2447	2.9669	0.6507	0.7463
metab_12513	neg	287.2232	6.2943	0.3162	0.6346	0.2724	0.4029
metab_7765	neg	287.9077	0.1093	0.3524	-0.0257	0.0476	0.1138
metab_15043	neg	287.9077	0.4807	0.3771	-0.1164	0.0734	0.1546
metab_10947	neg	287.9077	14.2070	0.2811	-0.0301	0.1286	0.2330
metab_13836	neg	287.9743	2.1475	0.7724	1.1540	0.2322	0.3574

metab_8803	neg	288.0187	2.5113	2.0028	6.4128	0.0029	0.0170
metab_13881	neg	288.0517	2.0516	0.7698	-0.9181	0.0787	0.1634
metab_13355	neg	288.0518	3.1455	0.6366	-0.4723	0.1674	0.2836
metab_1650	pos	288.0526	1.6272	0.9944	-3.5115	0.0937	0.1810
metab_14881	neg	288.0605	0.5571	0.5303	1.1438	0.3371	0.4702
metab_14593	neg	288.0728	1.0357	0.4738	0.6453	0.1837	0.3022
metab_8167	neg	288.0729	1.2805	0.7739	-0.6846	0.0072	0.0317
metab_5810	pos	288.0858	1.5987	0.2237	0.0718	0.6187	0.7261
metab_2043	pos	288.0859	2.9954	0.9499	-1.8472	0.0432	0.1022
metab_13845	neg	288.0882	2.1311	0.8757	-1.1263	0.1099	0.2080
metab_7664	neg	288.0882	2.8768	0.4675	-0.5288	0.2933	0.4247
metab_14342	neg	288.0885	1.4568	0.7233	-0.6859	0.0707	0.1509
metab_8413	neg	288.0994	1.6491	0.7581	-0.6059	0.1810	0.2991
metab_6295	pos	288.1003	0.6542	0.5470	-1.3446	0.1843	0.2990
metab_14678	neg	288.1203	0.8662	1.3454	2.6664	0.0006	0.0059
metab_5257	pos	288.1223	2.8259	0.7846	-1.0907	0.0392	0.0951
metab_14168	neg	288.1285	1.6028	0.6431	0.8984	0.0455	0.1103
metab_5615	pos	288.1336	2.0062	0.2317	1.2903	0.8116	0.8753
metab_13793	neg	288.1358	2.2272	0.1759	1.5553	0.7518	0.8220
metab_5228	pos	288.1372	2.9192	1.6634	-2.9469	0.0006	0.0053
metab_8772	neg	288.1455	2.4338	0.6505	1.1170	0.1838	0.3022
metab_1549	pos	288.1545	1.3437	0.8894	2.0265	0.1655	0.2757
metab_6127	pos	288.1545	1.0461	1.1944	4.3484	0.0316	0.0815
metab_8217	neg	288.1568	1.3373	1.7963	3.5417	0.0001	0.0015
metab_12773	neg	288.1608	5.0812	0.0733	-0.0348	0.8092	0.8643
metab_1783	pos	288.1700	2.0677	0.5432	-0.4555	0.3352	0.4689
metab_441	pos	288.1701	2.3133	0.3598	-0.1516	0.4469	0.5768
metab_5631	pos	288.1795	1.9758	1.4547	2.3416	0.0000	0.0008
metab_73	pos	288.1798	3.5311	0.3547	0.4414	0.4815	0.6070
metab_13190	neg	288.1823	3.6142	2.0059	9.6097	0.0062	0.0285
metab_5809	pos	288.1907	1.5987	0.5592	1.3563	0.3634	0.4971
metab_5575	pos	288.1910	2.0677	1.6054	3.6620	0.0038	0.0180
metab_6122	pos	288.2021	1.0601	1.2553	2.4676	0.0092	0.0340
metab_2272	pos	288.2161	4.0634	1.5090	-3.9253	0.0096	0.0348
metab_197	pos	288.2273	0.5560	0.8191	1.0124	0.0080	0.0307
metab_82	pos	288.2525	5.3454	0.5022	2.3953	0.2791	0.4082
metab_4487	pos	288.2889	6.2978	0.0196	0.0288	0.9050	0.9393
metab_4432	pos	288.2889	6.5857	0.0013	0.0262	0.9463	0.9663
metab_11053	neg	288.9368	14.0438	0.3569	-0.0187	0.0292	0.0814
metab_15007	neg	288.9369	0.5126	0.5307	1.3848	0.1702	0.2866
metab_14664	neg	288.9970	0.8803	1.7753	-4.4273	0.0000	0.0002
metab_13309	neg	289.0124	3.2801	0.6557	0.7726	0.0615	0.1369
metab_14923	neg	289.0336	0.5431	1.1877	-2.6067	0.0203	0.0638
metab_8783	neg	289.0725	2.4487	0.9304	-2.6497	0.0918	0.1822
metab_2021	pos	289.0812	2.9342	0.7692	-1.3135	0.1115	0.2053
metab_296	pos	289.1021	1.2306	1.2277	3.1853	0.0435	0.1026
metab_8046	neg	289.1045	0.9933	0.3255	0.7792	0.4285	0.5535
metab_5723	pos	289.1175	1.7554	0.2982	0.2570	0.4942	0.6185

metab_5556	pos	289.1176	2.1132	0.5343	-0.7515	0.1701	0.2814
metab_13721	neg	289.1198	2.3704	1.5951	-4.1812	0.0136	0.0487
metab_5848	pos	289.1271	1.5284	1.7229	4.3152	0.0265	0.0718
metab_310	pos	289.1385	1.3579	0.5843	1.4412	0.3475	0.4824
metab_13940	neg	289.1408	1.9745	0.4099	0.2899	0.4324	0.5570
metab_8379	neg	289.1409	1.5885	0.3516	0.1090	0.3723	0.5036
metab_4878	pos	289.1539	4.2907	0.1119	0.8182	0.9878	0.9924
metab_571	pos	289.1539	3.9577	0.2623	1.7019	0.7266	0.8118
metab_497	pos	289.1539	2.9501	0.7925	-1.3874	0.1843	0.2990
metab_13459	neg	289.1661	2.9092	0.9836	2.4508	0.0914	0.1818
metab_5961	pos	289.1749	1.3293	1.4390	12.5264	0.0014	0.0093
metab_4958	pos	289.1791	3.8967	1.7213	7.2567	0.0041	0.0193
metab_9207	neg	289.2025	3.7658	0.9253	8.5809	0.0643	0.1414
metab_1238	pos	289.2591	0.5140	0.8667	1.7191	0.2210	0.3433
metab_928	pos	289.8882	14.0665	0.0460	0.2189	0.7542	0.8317
metab_8817	neg	289.9209	2.5431	1.4686	-1.8838	0.0039	0.0207
metab_13390	neg	289.9210	3.0604	2.1953	-4.3908	0.0004	0.0051
metab_15020	neg	289.9495	0.5126	0.7225	-0.7659	0.0315	0.0855
metab_8661	neg	289.9712	2.1630	0.0302	0.1547	0.8517	0.8970
metab_6726	neg	289.9952	0.5431	0.0817	0.2370	0.6670	0.7598
metab_14484	neg	290.0155	1.2663	0.4359	-0.3546	0.2049	0.3267
metab_200	pos	290.0628	0.5843	0.7760	-1.9404	0.0910	0.1771
metab_14728	neg	290.0884	0.7818	0.9704	-1.0966	0.0250	0.0735
metab_7440	neg	290.0885	1.5297	0.3464	-0.0066	0.3094	0.4404
metab_5586	pos	290.1014	2.0524	0.4922	-0.5736	0.2259	0.3492
metab_5893	pos	290.1015	1.4569	0.8662	-0.9961	0.1517	0.2583
metab_2006	pos	290.1015	2.8725	0.6718	-1.2659	0.1423	0.2464
metab_8908	neg	290.1038	2.7792	0.9052	-1.1601	0.0595	0.1334
metab_13306	neg	290.1039	3.2801	0.5068	-0.5620	0.1726	0.2892
metab_14057	neg	290.1155	1.7729	0.2916	0.3378	0.5541	0.6660
metab_9664	neg	290.1190	6.0024	1.6686	-3.1663	0.0003	0.0037
metab_14429	neg	290.1249	1.3231	1.2280	1.7912	0.0000	0.0007
metab_8643	neg	290.1249	2.1311	0.7580	-1.3126	0.1914	0.3112
metab_38	pos	290.1378	1.0883	1.0956	-1.1573	0.0040	0.0187
metab_5298	pos	290.1379	2.7029	1.6602	-3.1187	0.0005	0.0045
metab_5736	pos	290.1491	1.7271	1.1413	1.8534	0.0120	0.0407
metab_426	pos	290.1492	2.2045	0.0378	0.5318	0.8790	0.9230
metab_1907	pos	290.1586	2.4370	0.1373	-0.0749	0.8485	0.9023
metab_5993	pos	290.1589	1.2585	1.0745	1.5822	0.0035	0.0172
metab_7519	neg	290.1613	1.8205	1.4613	-1.8679	0.0633	0.1398
metab_12570	neg	290.1765	6.0024	1.3531	-3.1745	0.0286	0.0804
metab_5406	pos	290.1783	2.4071	0.1240	1.3030	0.8653	0.9138
metab_5645	pos	290.1953	1.9463	1.9531	4.3286	0.0000	0.0005
metab_5049	pos	290.2107	3.5311	0.0873	-0.6188	0.9324	0.9573
metab_1235	pos	290.2430	0.5140	2.0897	13.8297	0.0000	0.0008
metab_2578	pos	290.2681	6.2678	0.1273	-0.3289	0.6879	0.7811
metab_1276	pos	290.8465	0.5560	1.0537	-1.2718	0.0487	0.1119
metab_12565	neg	290.8743	6.0346	0.2271	0.4537	0.6225	0.7231

metab_13590	neg	290.9970	2.6206	1.1113	-0.3383	0.0715	0.1519
metab_13536	neg	291.0203	2.7318	1.2528	-0.6892	0.0227	0.0687
metab_8	pos	291.0467	0.9340	0.8787	-0.8847	0.0080	0.0307
metab_487	pos	291.0856	2.8259	2.6599	8.1098	0.0000	0.0001
metab_9010	neg	291.0912	3.0604	1.7544	5.9768	0.0067	0.0299
metab_6912	neg	291.0990	1.4856	0.0815	0.6468	0.9735	0.9812
metab_13652	neg	291.0990	2.5113	0.4265	-0.0873	0.2308	0.3561
metab_1138	pos	291.1289	0.6123	0.8328	2.5170	0.0662	0.1402
metab_5745	pos	291.1330	1.7128	0.6254	0.7238	0.1593	0.2679
metab_5430	pos	291.1332	2.3751	1.1454	-2.0730	0.0008	0.0064
metab_56	pos	291.1333	2.0062	0.0142	-0.2408	0.9024	0.9372
metab_8969	neg	291.1354	2.9588	0.6413	-0.9444	0.0720	0.1526
metab_13666	neg	291.1355	2.4813	0.6959	-1.6410	0.3146	0.4464
metab_9056	neg	291.1356	3.2130	0.3876	2.5679	0.7082	0.7908
metab_13993	neg	291.1389	1.8682	1.1764	-1.3115	0.0842	0.1711
metab_5630	pos	291.1537	1.9758	0.4207	0.2595	0.2934	0.4239
metab_1123	pos	291.1541	0.6262	0.1381	-0.4592	0.6461	0.7486
metab_355	pos	291.1542	1.6128	0.1395	0.1786	0.7527	0.8306
metab_5695	pos	291.1694	1.7993	1.2788	12.0429	0.0178	0.0535
metab_9041	neg	291.1817	3.1797	0.9159	3.5382	0.2681	0.3984
metab_4421	pos	291.1946	6.6606	1.0904	1.2072	0.0005	0.0045
metab_4993	pos	291.1946	3.7447	0.7473	0.5735	0.0479	0.1104
metab_4601	pos	291.1946	5.6623	0.5362	0.3747	0.1438	0.2483
metab_598	pos	291.1946	4.5027	0.8430	0.6690	0.0283	0.0756
metab_4745	pos	291.1946	4.9115	0.5058	0.2052	0.1426	0.2468
metab_5061	pos	291.1947	3.5011	0.7881	0.6182	0.0708	0.1473
metab_7020	neg	291.1970	6.8093	0.2130	-0.4330	0.6044	0.7087
metab_10812	neg	291.8838	14.0771	0.3077	-0.0109	0.1199	0.2213
metab_8818	neg	291.9179	2.5431	1.5241	-2.0056	0.0040	0.0208
metab_194	pos	291.9741	0.5560	1.3053	1.9311	0.0023	0.0130
metab_7531	neg	292.0831	1.9904	0.9414	-2.4543	0.0716	0.1520
metab_8356	neg	292.0833	1.5586	0.9674	-1.4130	0.0069	0.0306
metab_13787	neg	292.1025	2.2272	0.1707	-0.0359	0.6631	0.7568
metab_1754	pos	292.1170	1.9758	0.5392	-0.8799	0.0624	0.1338
metab_2105	pos	292.1171	3.2420	0.9840	-1.5733	0.0112	0.0388
metab_5362	pos	292.1171	2.5003	1.0069	-1.5071	0.0100	0.0359
metab_8967	neg	292.1195	2.9588	0.7952	-0.7523	0.0236	0.0705
metab_12955	neg	292.1195	4.3549	0.7819	1.0602	0.0990	0.1925
metab_9028	neg	292.1195	3.1286	0.8111	-0.5387	0.0191	0.0610
metab_13111	neg	292.1195	3.8333	0.8058	-1.1478	0.1122	0.2115
metab_6114	pos	292.1285	1.0741	1.2775	1.2560	0.1645	0.2745
metab_8340	neg	292.1308	1.5439	0.0054	0.4451	0.8891	0.9256
metab_13771	neg	292.1309	2.2595	1.9551	-3.9654	0.0002	0.0028
metab_8111	neg	292.1309	1.1796	0.1918	0.1153	0.5347	0.6503
metab_12700	neg	292.1345	5.4684	1.2802	-3.4811	0.0393	0.0996
metab_14630	neg	292.1405	0.9649	1.3459	-2.3099	0.1235	0.2259
metab_8554	neg	292.1405	1.9280	0.4853	-0.5255	0.2174	0.3409
metab_8125	neg	292.1669	1.2084	0.2607	-0.4292	0.4746	0.5966

metab_1167	pos	292.2222	0.5140	0.4495	-0.6532	0.2626	0.3904
metab_4976	pos	292.2263	3.8208	0.2711	-0.4413	0.4786	0.6053
metab_7737	neg	292.8692	0.0197	0.3409	-0.0145	0.0816	0.1676
metab_8461	neg	293.0308	1.7419	1.0057	1.9994	0.0211	0.0652
metab_14380	neg	293.0784	1.3965	0.5432	1.1359	0.2895	0.4209
metab_14818	neg	293.0995	0.5991	1.3077	-1.9563	0.0306	0.0841
metab_5169	pos	293.1011	3.1032	1.3330	11.6768	0.0259	0.0707
metab_67	pos	293.1013	2.3908	2.0867	5.2652	0.0001	0.0013
metab_8662	neg	293.1037	2.1798	0.0335	0.4251	0.9552	0.9707
metab_13435	neg	293.1038	2.9760	1.5167	3.0554	0.0073	0.0318
metab_13059	neg	293.1071	4.0182	1.1934	4.8443	0.0296	0.0819
metab_13114	neg	293.1072	3.8164	2.1601	5.2818	0.0048	0.0237
metab_1593	pos	293.1122	1.4860	0.0971	0.5137	0.9276	0.9533
metab_1475	pos	293.1124	1.1447	0.0040	0.0284	0.9329	0.9576
metab_7553	neg	293.1146	2.1153	0.7804	-0.8731	0.0182	0.0592
metab_7494	neg	293.1147	1.7729	1.0114	-1.0216	0.0021	0.0137
metab_8188	neg	293.1149	1.3089	0.7569	-0.7473	0.0323	0.0871
metab_1862	pos	293.1277	2.3133	0.3718	0.8051	0.4066	0.5396
metab_9555	neg	293.1302	5.3713	0.1263	1.0787	0.9468	0.9650
metab_7647	neg	293.1358	0.5431	0.3032	0.3707	0.6187	0.7206
metab_459	pos	293.1488	2.4850	0.3614	-1.0793	0.3833	0.5158
metab_8521	neg	293.1511	1.8682	0.2990	-0.2169	0.4539	0.5773
metab_14103	neg	293.1511	1.7104	0.5215	-0.4389	0.2130	0.3359
metab_1667	pos	293.1596	1.6838	1.7695	9.7235	0.0507	0.1151
metab_5613	pos	293.1599	2.0062	1.1563	-0.7366	0.0477	0.1100
metab_9615	neg	293.1762	5.7259	0.6317	0.7875	0.0029	0.0170
metab_12226	neg	293.1797	7.2865	0.2061	0.6993	0.4534	0.5767
metab_10301	neg	293.1799	8.7031	0.6589	-0.3488	0.0130	0.0471
metab_11549	neg	293.1800	9.1364	0.7036	-0.3102	0.0000	0.0013
metab_181	pos	293.1812	0.5140	1.1871	-1.2503	0.0333	0.0846
metab_5193	pos	293.1851	3.0114	1.3062	3.0861	0.0366	0.0904
metab_137	pos	293.2103	5.6773	0.7241	-0.9786	0.0296	0.0780
metab_12291	neg	293.2127	7.0796	0.6270	-0.4843	0.0654	0.1429
metab_2988	pos	293.2465	9.0112	1.1254	3.5114	0.0143	0.0458
metab_3197	pos	293.2466	10.7001	1.3088	5.4420	0.0512	0.1161
metab_735	pos	293.2466	7.6332	1.3229	1.6405	0.0742	0.1522
metab_3073	pos	293.2466	9.6044	1.4044	4.1984	0.0074	0.0289
metab_3180	pos	293.2466	10.3632	1.0722	2.1363	0.1322	0.2331
metab_12016	neg	293.2490	7.9757	1.7835	-7.3229	0.0015	0.0111
metab_820	pos	293.2831	9.1302	0.0504	-0.1818	0.9373	0.9611
metab_6477	pos	293.9703	0.5140	1.2828	-2.4951	0.0043	0.0198
metab_6373	pos	294.0365	0.5983	0.5583	0.6596	0.0542	0.1212
metab_13397	neg	294.0447	3.0432	1.4051	12.1720	0.0114	0.0432
metab_14347	neg	294.0626	1.4427	0.1561	-0.0697	0.6181	0.7202
metab_6752	neg	294.0834	0.6131	0.6465	-0.1452	0.2787	0.4092
metab_435	pos	294.0964	2.2519	0.3115	-0.2643	0.2098	0.3299
metab_9431	neg	294.0988	4.6883	1.2765	2.6028	0.0055	0.0263
metab_7607	neg	294.0988	2.4970	1.4068	-1.7907	0.0000	0.0000

metab_13761	neg	294.0988	2.2912	1.0668	-1.2697	0.0029	0.0168
metab_9454	neg	294.1140	4.7881	0.9453	-0.4574	0.1548	0.2676
metab_8411	neg	294.1179	1.6491	0.7050	-0.3843	0.0096	0.0388
metab_13293	neg	294.1253	3.2972	1.0194	-1.7481	0.0320	0.0865
metab_5975	pos	294.1327	1.3153	1.3778	-2.2962	0.0045	0.0204
metab_2196	pos	294.1328	3.6684	0.9316	-1.6185	0.0383	0.0934
metab_4859	pos	294.1328	4.3512	1.1620	1.4131	0.0326	0.0833
metab_8816	neg	294.1350	2.5431	1.9129	-2.2186	0.0043	0.0220
metab_7504	neg	294.1350	1.8205	1.4082	-1.8948	0.0290	0.0810
metab_9032	neg	294.1352	3.1455	1.2732	-1.7994	0.0034	0.0187
metab_8286	neg	294.1352	1.4568	1.0904	-1.2651	0.0236	0.0705
metab_344	pos	294.1438	1.5424	0.2066	0.3969	0.6732	0.7697
metab_6067	pos	294.1442	1.1731	0.3008	-0.1137	0.3861	0.5185
metab_8303	neg	294.1463	1.4856	0.8180	-0.0680	0.2134	0.3363
metab_395	pos	294.1529	1.9463	0.2502	-0.7165	0.6893	0.7825
metab_5760	pos	294.1623	1.6838	0.9122	0.5598	0.1164	0.2119
metab_6026	pos	294.1803	1.2162	0.7370	-0.6327	0.1888	0.3041
metab_2020	pos	294.1957	2.9342	0.2285	-0.4097	0.8836	0.9261
metab_127	pos	294.2419	6.4035	0.5190	0.5307	0.0492	0.1128
metab_5112	pos	294.2532	3.2880	0.1413	-0.3026	0.7876	0.8574
metab_4092	pos	294.2782	8.2435	1.0828	-1.7356	0.0497	0.1135
metab_11025	neg	294.8855	14.0610	0.1322	0.0776	0.5130	0.6319
metab_6785	neg	295.0339	0.7958	0.5575	1.7828	0.4008	0.5289
metab_14296	neg	295.0463	1.4856	1.4452	2.6627	0.0008	0.0070
metab_8115	neg	295.0862	1.1796	1.6617	2.4888	0.0437	0.1072
metab_1565	pos	295.0915	1.4005	0.8633	1.6603	0.0903	0.1760
metab_9858	neg	295.0923	7.0162	1.5826	-1.7458	0.0461	0.1113
metab_7466	neg	295.0939	1.5155	0.5117	-0.4067	0.2281	0.3531
metab_14611	neg	295.0939	0.9933	0.4198	2.3024	0.6195	0.7211
metab_5172	pos	295.1071	3.1032	0.9560	-1.6935	0.0734	0.1512
metab_6288	pos	295.1124	0.6822	1.3316	-2.7199	0.0536	0.1203
metab_4574	pos	295.1168	5.7846	0.0913	-0.0277	0.8235	0.8840
metab_13733	neg	295.1190	2.3380	0.8577	2.0183	0.1972	0.3178
metab_9034	neg	295.1192	3.1455	1.4025	2.6424	0.0166	0.0556
metab_9312	neg	295.1220	4.1364	0.3495	5.2046	0.6278	0.7272
metab_1705	pos	295.1280	1.7845	1.0224	-1.3711	0.0015	0.0097
metab_14319	neg	295.1302	1.4568	0.6823	0.8629	0.1344	0.2412
metab_8898	neg	295.1302	2.7624	0.2791	0.0174	0.5999	0.7051
metab_1992	pos	295.1436	2.8259	0.0061	0.4471	0.9486	0.9671
metab_216	pos	295.1491	0.6402	1.3065	-2.1847	0.0475	0.1097
metab_1158	pos	295.1492	0.5280	0.7153	-0.7270	0.1862	0.3013
metab_2282	pos	295.1529	4.1387	1.7610	12.4412	0.0009	0.0072
metab_2157	pos	295.1530	3.5011	2.3315	6.0665	0.0004	0.0043
metab_9257	neg	295.1554	3.9177	2.6699	8.3582	0.0001	0.0016
metab_9393	neg	295.1554	4.5391	1.3922	3.3459	0.0144	0.0505
metab_6949	neg	295.1555	4.2533	0.3678	0.7672	0.3724	0.5037
metab_5674	pos	295.1643	1.8729	0.6226	0.3716	0.0474	0.1097
metab_5410	pos	295.1645	2.4071	0.9297	1.2798	0.0057	0.0241

metab_9699	neg	295.1916	6.1968	0.7148	3.3395	0.2886	0.4202
metab_5070	pos	295.2108	3.4549	0.3361	-0.2619	0.2188	0.3410
metab_1474	pos	295.2119	1.1447	0.6451	-0.6240	0.1705	0.2820
metab_5997	pos	295.2120	1.2585	0.5223	-0.1220	0.3438	0.4785
metab_2691	pos	295.2260	7.0670	0.6531	-0.7793	0.0649	0.1379
metab_4653	pos	295.2260	5.3454	0.0064	-0.1609	0.9972	0.9985
metab_12	pos	295.2260	4.9267	0.0677	-0.2717	0.8887	0.9298
metab_6568	neg	295.2281	7.0162	1.0857	-1.4839	0.0249	0.0732
metab_3021	pos	295.2623	9.1898	0.7061	10.1596	0.1251	0.2238
metab_12304	neg	295.3634	7.0322	1.2343	-1.7252	0.0385	0.0983
metab_10746	neg	295.8201	13.9952	0.1819	0.1635	0.5533	0.6652
metab_6411	pos	296.0651	0.5560	1.2222	1.6129	0.0075	0.0292
metab_7450	neg	296.0795	1.5439	0.0579	0.2179	0.7487	0.8204
metab_13212	neg	296.0828	3.5641	0.1304	0.0144	0.7677	0.8337
metab_13145	neg	296.0965	3.7488	1.3738	-2.3324	0.0081	0.0342
metab_14598	neg	296.1001	1.0215	0.3322	-0.0754	0.4743	0.5964
metab_8799	neg	296.1042	2.4970	2.0299	-3.8492	0.0000	0.0000
metab_8714	neg	296.1045	2.2912	1.3876	-1.9661	0.0256	0.0747
metab_5980	pos	296.1054	1.2869	0.7652	1.4586	0.1459	0.2510
metab_1859	pos	296.1121	2.2824	1.1320	-1.6258	0.0015	0.0099
metab_6920	neg	296.1142	1.5155	0.3295	0.8604	0.3872	0.5163
metab_12812	neg	296.1144	4.9183	1.2818	4.0805	0.0184	0.0594
metab_5978	pos	296.1238	1.3013	0.4809	1.1529	0.3610	0.4950
metab_8122	neg	296.1254	1.1940	0.8829	3.3791	0.1594	0.2735
metab_4766	pos	296.1273	4.7907	1.0072	-0.6656	0.1125	0.2063
metab_6968	neg	296.1293	4.7881	1.2824	-0.8378	0.0347	0.0912
metab_5110	pos	296.1388	3.3032	0.9065	-1.2937	0.1605	0.2692
metab_13356	neg	296.1409	3.1455	1.3607	-2.1702	0.0355	0.0926
metab_62	pos	296.1484	2.5469	1.4292	-2.2050	0.0005	0.0045
metab_2070	pos	296.1485	3.1346	1.1366	-1.7788	0.0031	0.0160
metab_1843	pos	296.1961	2.2361	0.4458	-0.9236	0.6529	0.7536
metab_4361	pos	296.2001	6.9638	0.0218	0.3169	0.9113	0.9427
metab_4965	pos	296.2061	3.8666	0.2457	-0.1803	0.2299	0.3535
metab_2595	pos	296.2218	6.4489	0.8118	1.2094	0.1818	0.2959
metab_2129	pos	296.2424	3.3337	0.2265	-0.2252	0.3970	0.5300
metab_4620	pos	296.2575	5.5414	0.3902	0.5048	0.1193	0.2161
metab_1009	pos	296.2575	5.7846	0.9070	1.5944	0.0134	0.0438
metab_696	pos	296.2575	6.9035	0.4179	-0.3556	0.0249	0.0686
metab_2963	pos	296.2940	8.8759	0.3491	-0.2987	0.1481	0.2539
metab_6917	neg	297.0668	1.5012	1.5894	3.0272	0.0034	0.0188
metab_6769	neg	297.0828	0.6131	0.0036	0.7403	0.9876	0.9908
metab_9156	neg	297.0982	3.5813	2.4799	5.1593	0.0002	0.0034
metab_8652	neg	297.0984	2.1475	0.6874	-0.5163	0.0495	0.1167
metab_6101	pos	297.1073	1.1024	0.2621	0.2212	0.4987	0.6215
metab_5709	pos	297.1074	1.7705	1.0251	1.4973	0.0206	0.0595
metab_14520	neg	297.1097	1.2236	0.2180	-0.1618	0.5834	0.6915
metab_486	pos	297.1326	2.8259	0.1148	-0.4007	0.6856	0.7794
metab_9267	neg	297.1346	3.9677	2.0459	4.7627	0.0000	0.0005

metab_8887	neg	297.1348	2.7475	1.8266	4.0499	0.0001	0.0014
metab_1025	pos	297.1436	2.0062	0.0063	-0.4030	0.9883	0.9924
metab_5979	pos	297.1436	1.3013	1.4776	2.3552	0.0008	0.0066
metab_5465	pos	297.1437	2.2665	0.6722	0.6023	0.0116	0.0399
metab_7515	neg	297.1457	1.8682	0.4931	0.9031	0.3482	0.4812
metab_8389	neg	297.1458	1.6028	0.0637	0.3738	0.9699	0.9787
metab_11134	neg	297.1532	13.9143	0.2284	-0.0279	0.3706	0.5020
metab_11159	neg	297.1532	12.1016	0.3479	-0.0926	0.1823	0.3004
metab_12427	neg	297.1533	6.6157	0.1239	0.3762	0.7996	0.8565
metab_11734	neg	297.1534	8.7031	0.9506	-0.7085	0.0001	0.0023
metab_5016	pos	297.1682	3.6684	1.7404	3.4490	0.0001	0.0019
metab_4881	pos	297.1687	4.2609	2.0598	4.6706	0.0006	0.0057
metab_1796	pos	297.1690	2.0973	1.6568	4.0209	0.0012	0.0083
metab_12763	neg	297.1710	5.1296	0.2475	0.7168	0.5105	0.6295
metab_12883	neg	297.1711	4.6380	1.1816	1.8718	0.0067	0.0301
metab_9218	neg	297.1711	3.8001	1.5594	3.5067	0.0135	0.0484
metab_13227	neg	297.1711	3.5310	1.7180	3.7129	0.0183	0.0593
metab_13388	neg	297.1711	3.0604	1.8567	5.5369	0.0020	0.0133
metab_1758	pos	297.1797	1.9918	0.4894	0.8487	0.4691	0.5962
metab_5158	pos	297.2054	3.1495	0.1120	1.1235	0.9878	0.9924
metab_4644	pos	297.2324	5.3757	0.3123	-0.4435	0.7331	0.8157
metab_983	pos	297.2325	7.0816	0.5858	-0.5163	0.2447	0.3693
metab_9857	neg	297.2344	7.0162	1.1150	-1.5034	0.0307	0.0841
metab_9	pos	297.2415	5.1975	0.4766	1.1769	0.2826	0.4123
metab_4288	pos	297.2415	7.2763	0.6932	-0.6239	0.0924	0.1791
metab_4541	pos	297.2415	5.9649	0.3609	0.5433	0.5733	0.6883
metab_12236	neg	297.2436	7.2377	0.7677	-0.8915	0.1245	0.2274
metab_12459	neg	297.2441	6.4709	0.2366	-0.4390	0.6728	0.7636
metab_8194	neg	298.0572	1.3089	0.2132	-0.2973	0.5915	0.6986
metab_8008	neg	298.0698	0.8662	1.2645	-1.5273	0.0000	0.0004
metab_8329	neg	298.0935	1.5297	0.3000	0.4336	0.4610	0.5836
metab_1066	pos	298.0960	1.4569	0.4521	-0.4065	0.4004	0.5337
metab_7694	neg	298.1088	3.2130	1.3122	-2.8327	0.0184	0.0594
metab_9001	neg	298.1089	3.0260	1.0992	-0.9463	0.1309	0.2361
metab_5518	pos	298.1277	2.1746	1.1873	-1.5677	0.0000	0.0000
metab_306	pos	298.1277	1.3293	0.0095	0.0381	0.9446	0.9655
metab_2024	pos	298.1430	2.9501	1.2146	3.7562	0.0660	0.1400
metab_13142	neg	298.1452	3.7488	1.8179	-6.7482	0.0002	0.0034
metab_1563	pos	298.1574	1.4005	1.2113	-1.7979	0.0001	0.0017
metab_5371	pos	298.1638	2.4850	0.2107	-0.3066	0.4590	0.5879
metab_1083	pos	298.1640	1.3013	0.0753	0.9358	0.9217	0.9493
metab_9229	neg	298.1665	3.8333	0.5712	-0.1493	0.3415	0.4745
metab_1399	pos	298.1752	0.8920	0.4716	-0.1963	0.2644	0.3922
metab_1252	pos	298.1866	0.5280	1.3642	2.5741	0.0295	0.0778
metab_588	pos	298.2005	4.4126	1.5533	3.7593	0.0589	0.1284
metab_5881	pos	298.2333	1.4714	0.1852	0.0802	0.7075	0.7964
metab_10134	neg	298.2394	8.0998	0.3718	0.4034	0.5029	0.6230
metab_4276	pos	298.2732	7.3370	0.9450	-1.1381	0.0002	0.0029

metab_2793	pos	298.2732	7.8097	1.1309	-1.2706	0.0091	0.0338
metab_997	pos	298.2732	6.2978	0.9695	1.7306	0.0176	0.0532
metab_3060	pos	298.3462	9.4981	0.2461	0.1852	0.3502	0.4853
metab_1268	pos	298.9856	0.5420	0.5474	0.6816	0.2611	0.3887
metab_1857	pos	299.0842	2.2665	0.3790	2.5300	0.5934	0.7045
metab_6866	neg	299.0887	1.2948	0.1896	0.4703	0.7349	0.8088
metab_7639	neg	299.0888	2.7155	0.9891	-0.4113	0.0249	0.0732
metab_13825	neg	299.1141	2.1630	0.5163	-0.3924	0.0845	0.1716
metab_271	pos	299.1230	1.0741	1.0217	1.1420	0.0000	0.0010
metab_8693	neg	299.1251	2.2437	0.1960	0.5220	0.7557	0.8242
metab_8162	neg	299.1348	1.2663	0.9922	1.4257	0.2402	0.3663
metab_5561	pos	299.1382	2.0973	0.1942	0.2136	0.6460	0.7486
metab_13275	neg	299.1505	3.3631	1.5186	2.6481	0.0000	0.0005
metab_13448	neg	299.1505	2.9429	1.7269	4.1429	0.0012	0.0096
metab_1460	pos	299.1592	1.1024	0.3349	0.2564	0.2618	0.3896
metab_5084	pos	299.1596	3.3793	1.7531	3.1967	0.0000	0.0005
metab_13351	neg	299.1615	3.1626	0.2936	0.4220	0.5754	0.6848
metab_12815	neg	299.1655	4.9021	0.5429	2.3120	0.3915	0.5198
metab_466	pos	299.1746	2.5627	0.4331	-0.5118	0.1306	0.2311
metab_2230	pos	299.1839	3.8063	1.2489	3.5501	0.0205	0.0594
metab_5078	pos	299.1845	3.4098	1.0947	3.2455	0.0924	0.1791
metab_9433	neg	299.1867	4.7046	0.7797	1.4648	0.2551	0.3835
metab_12560	neg	299.2231	6.0831	0.9497	1.5601	0.0052	0.0251
metab_12278	neg	299.2232	7.1111	1.9334	5.7125	0.0000	0.0001
metab_12400	neg	299.2232	6.7603	1.2081	1.6684	0.0000	0.0003
metab_9717	neg	299.2234	6.3111	1.0705	1.8571	0.0002	0.0028
metab_9924	neg	299.2501	7.2377	0.6328	-1.1392	0.4293	0.5543
metab_4157	pos	299.2570	7.9835	0.0026	0.1828	0.9454	0.9658
metab_3154	pos	299.2571	10.1021	0.8613	0.9403	0.1367	0.2392
metab_122	pos	299.2572	7.5005	1.2483	1.9856	0.0427	0.1015
metab_904	pos	299.2572	9.8362	0.0275	0.4976	0.8632	0.9125
metab_3916	pos	299.2573	8.9656	1.2031	1.8825	0.0160	0.0495
metab_3839	pos	299.2574	9.3117	1.4514	2.3313	0.0022	0.0129
metab_12002	neg	299.2593	8.0069	1.2981	-0.7708	0.0194	0.0616
metab_6633	neg	299.2595	9.1038	0.5721	-0.3696	0.0826	0.1689
metab_2085	pos	299.6789	3.1799	1.2123	-2.1146	0.0107	0.0375
metab_14698	neg	300.0129	0.8098	0.2496	0.5125	0.7047	0.7884
metab_14926	neg	300.0492	0.5431	0.3593	-0.1430	0.3120	0.4433
metab_8419	neg	300.0997	1.6642	0.4012	1.1120	0.4547	0.5779
metab_1584	pos	300.1010	1.4569	0.6140	-0.4439	0.3390	0.4732
metab_396	pos	300.1219	1.9463	0.1554	-0.6506	0.6568	0.7563
metab_13295	neg	300.1245	3.2972	0.5017	-0.7166	0.1799	0.2977
metab_9334	neg	300.1246	4.2367	1.3126	-2.4193	0.0010	0.0087
metab_14856	neg	300.1302	0.5851	0.6249	-0.6198	0.0654	0.1429
metab_1957	pos	300.1341	2.6561	1.2092	-2.1888	0.0388	0.0944
metab_13958	neg	300.1359	1.9280	0.3904	-0.7499	0.2740	0.4046
metab_6201	pos	300.1361	0.8780	0.2841	-0.4086	0.4769	0.6038
metab_1096	pos	300.1434	1.1875	0.8594	1.2744	0.0153	0.0482

metab_13522	neg	300.1458	2.7624	0.0698	0.2883	0.9850	0.9892
metab_1310	pos	300.1544	0.6123	2.4335	14.9500	0.0130	0.0429
metab_14049	neg	300.1567	1.7885	1.0152	2.5102	0.0584	0.1317
metab_2045	pos	300.1587	3.0114	0.8288	2.9572	0.2002	0.3184
metab_12669	neg	300.1610	5.6128	0.3171	-0.5896	0.3817	0.5115
metab_1721	pos	300.1731	1.8577	1.8608	3.5113	0.0001	0.0014
metab_573	pos	300.1797	4.0634	1.0963	1.4113	0.0798	0.1607
metab_2080	pos	300.1799	3.1799	0.7736	0.8953	0.0503	0.1144
metab_9283	neg	300.1821	4.0516	0.3938	0.9266	0.4928	0.6141
metab_13874	neg	300.1932	2.0670	1.0178	1.4139	0.0119	0.0445
metab_13624	neg	300.1932	2.5431	1.5272	2.6407	0.0014	0.0108
metab_8872	neg	300.1933	2.6986	1.7261	3.7975	0.0001	0.0019
metab_14227	neg	300.1934	1.5439	2.0301	5.6264	0.0000	0.0000
metab_4040	pos	300.2886	8.4356	0.3352	-0.2763	0.4720	0.5989
metab_6716	neg	300.8776	0.5286	1.5283	-2.3708	0.0012	0.0096
metab_6278	pos	301.0075	0.7381	0.2344	1.1141	0.6871	0.7805
metab_6437	pos	301.0075	0.5280	0.3633	1.9476	0.4815	0.6070
metab_14152	neg	301.0392	1.6335	0.5913	0.7016	0.2802	0.4108
metab_1132	pos	301.0423	0.6262	0.0062	0.1893	0.9479	0.9669
metab_14423	neg	301.0570	1.3231	0.8514	1.9740	0.1682	0.2845
metab_1164	pos	301.0724	0.5140	0.8371	-1.2856	0.0462	0.1075
metab_6314	pos	301.0787	0.6262	0.8091	1.1525	0.1361	0.2385
metab_8925	neg	301.0833	2.8269	0.1874	0.5185	0.8552	0.8997
metab_13332	neg	301.0834	3.2130	0.8129	-0.7388	0.0809	0.1667
metab_13224	neg	301.0835	3.5310	0.0273	0.7859	0.7908	0.8499
metab_5949	pos	301.0847	1.3437	0.4594	1.2047	0.4465	0.5765
metab_8120	neg	301.1044	1.1940	0.5152	0.9506	0.1597	0.2737
metab_421	pos	301.1176	2.1444	0.6750	-1.0611	0.0472	0.1094
metab_2094	pos	301.1176	3.2274	1.5635	3.1720	0.0001	0.0013
metab_13638	neg	301.1199	2.5264	0.8081	-0.6925	0.2169	0.3406
metab_8958	neg	301.1199	2.9263	0.4419	-0.2667	0.1886	0.3077
metab_13865	neg	301.1200	2.0829	0.6900	0.2772	0.1988	0.3195
metab_14013	neg	301.1201	1.8371	0.1414	-0.0175	0.6503	0.7462
metab_6070	pos	301.1207	1.1589	0.3413	-8.2507	0.4666	0.5939
metab_13751	neg	301.1299	2.3078	3.1797	9.7897	0.0000	0.0000
metab_8390	neg	301.1300	1.6028	1.1017	1.6761	0.0007	0.0068
metab_1095	pos	301.1387	1.2016	0.3110	0.1952	0.2351	0.3592
metab_14140	neg	301.1405	1.6491	0.0775	0.4349	0.9636	0.9750
metab_13941	neg	301.1407	1.9745	0.6946	1.0437	0.0755	0.1580
metab_5755	pos	301.1469	1.6983	1.2563	-2.6202	0.0227	0.0639
metab_6468	pos	301.1498	0.5140	1.1329	3.1077	0.0285	0.0282
metab_5337	pos	301.1539	2.5782	0.5342	-0.7700	0.1898	0.3052
metab_9778	neg	301.1578	6.6320	0.2251	1.5921	0.7232	0.8015
metab_7856	neg	301.1632	0.5286	1.3500	4.0310	0.0105	0.0409
metab_7617	neg	301.1661	2.5748	1.2498	2.8215	0.0094	0.0381
metab_417	pos	301.1748	2.1132	0.2026	0.1268	0.5498	0.6680
metab_8666	neg	301.1770	2.1798	0.4832	0.5875	0.3534	0.4864
metab_8988	neg	301.1772	3.0101	0.2552	0.8723	0.6727	0.7636

metab_8507	neg	301.1773	1.8205	1.1363	2.5707	0.0008	0.0074
metab_2355	pos	301.1790	4.6096	1.7237	6.9662	0.0352	0.0880
metab_15024	neg	301.1997	0.4975	0.5643	1.9905	0.2476	0.3745
metab_9299	neg	301.2024	4.1023	0.5046	1.2806	0.4740	0.5960
metab_4963	pos	301.2113	3.8821	1.0763	3.5588	0.0990	0.1879
metab_12465	neg	301.2383	6.4553	1.4824	2.6902	0.0003	0.0042
metab_12436	neg	301.2386	6.5989	1.6232	3.0317	0.0008	0.0070
metab_10408	neg	301.2660	9.1038	0.6370	-0.4912	0.0804	0.1659
metab_8848	neg	302.0341	2.6357	0.2023	0.2919	0.6441	0.7406
metab_14602	neg	302.0521	1.0074	0.7183	2.4068	0.2214	0.3454
metab_6872	neg	302.0885	1.3089	1.0004	1.5143	0.0009	0.0078
metab_7579	neg	302.1011	0.5711	1.0530	1.5650	0.0503	0.1180
metab_2082	pos	302.1016	3.1799	0.8144	-1.6048	0.0416	0.0996
metab_8881	neg	302.1036	2.7318	1.0040	-1.9807	0.0233	0.0700
metab_1660	pos	302.1125	1.6553	1.1276	3.0791	0.0247	0.0683
metab_4887	pos	302.1378	4.2301	1.2427	-3.5157	0.0135	0.0440
metab_8687	neg	302.1442	2.2272	1.1178	1.6250	0.0161	0.0546
metab_5656	pos	302.1493	1.9309	0.3045	-0.3361	0.4947	0.6186
metab_14325	neg	302.1515	1.4568	0.5747	-0.5715	0.1687	0.2851
metab_13073	neg	302.1614	3.9677	1.5868	3.1165	0.0001	0.0017
metab_380	pos	302.1699	1.7705	1.1459	1.8912	0.0138	0.0449
metab_1087	pos	302.1702	1.2162	0.8439	1.7306	0.1002	0.1895
metab_14127	neg	302.1878	1.6642	0.2018	-0.4629	0.6166	0.7195
metab_2327	pos	302.1892	4.4429	0.4236	-1.1579	0.3334	0.4678
metab_2263	pos	302.1955	4.0338	0.9532	1.1986	0.0413	0.0990
metab_1613	pos	302.2064	1.5284	1.9289	3.3792	0.0000	0.0008
metab_1806	pos	302.2065	2.1290	1.3510	1.5875	0.0053	0.0228
metab_5291	pos	302.2066	2.7188	2.8599	7.1148	0.0000	0.0009
metab_563	pos	302.2318	3.8208	0.5459	0.4421	0.2733	0.4016
metab_2864	pos	302.3044	8.3018	0.6562	-0.9360	0.1730	0.2853
metab_2699	pos	302.3044	7.1119	0.4314	0.1413	0.0497	0.1135
metab_6723	neg	302.8482	0.5431	1.0789	-1.0574	0.0057	0.0268
metab_8259	neg	303.0449	1.4427	1.8473	-4.1073	0.0007	0.0068
metab_14640	neg	303.0836	0.9368	0.5511	0.8797	0.1333	0.2394
metab_14059	neg	303.0991	1.7729	0.8783	-1.0673	0.0748	0.1570
metab_6056	pos	303.1178	1.1875	0.9726	1.5066	0.0296	0.0781
metab_317	pos	303.1178	1.3720	0.3349	1.5317	0.6149	0.7229
metab_8328	neg	303.1200	1.5297	0.0776	0.1518	0.7531	0.8228
metab_8590	neg	303.1200	2.0055	0.3919	-0.1343	0.4096	0.5368
metab_1701	pos	303.1329	1.7705	0.1418	-0.3505	0.7154	0.8023
metab_1026	pos	303.1332	2.0062	0.4847	-0.6383	0.2261	0.3494
metab_13168	neg	303.1356	3.6812	0.8084	-0.4161	0.0654	0.1429
metab_13440	neg	303.1370	2.9588	0.6259	-1.2142	0.2286	0.3536
metab_6065	pos	303.1446	1.1731	0.0670	-0.0279	0.8248	0.8843
metab_13172	neg	303.1447	3.6645	3.0575	10.4429	0.0000	0.0002
metab_13690	neg	303.1461	2.4175	2.2485	5.2021	0.0092	0.0375
metab_8500	neg	303.1563	1.8205	0.0103	0.3056	0.8492	0.8954
metab_13844	neg	303.1563	2.1311	0.3190	0.6406	0.5646	0.6758

metab_1603	pos	303.1692	1.5144	1.2651	-2.0215	0.0007	0.0059
metab_8777	neg	303.1816	2.4487	1.4713	3.4083	0.0021	0.0135
metab_13803	neg	303.1817	2.2119	1.4223	3.3697	0.0032	0.0180
metab_9256	neg	303.1818	3.9177	1.7152	7.4634	0.0065	0.0292
metab_465	pos	303.1858	2.5782	0.0880	1.1062	0.9414	0.9632
metab_561	pos	303.1875	2.1897	0.1149	0.7031	0.8291	0.8875
metab_5202	pos	303.1906	2.9954	0.3484	0.0444	0.4801	0.6065
metab_9419	neg	303.2180	4.6380	0.9289	3.2152	0.1627	0.2776
metab_9582	neg	303.2181	5.5639	0.1636	-0.3117	0.7417	0.8138
metab_4548	pos	303.2310	5.9361	0.0386	0.3084	0.8688	0.9161
metab_7522	neg	303.9890	1.9125	0.6121	1.0953	0.0891	0.1787
metab_12976	neg	304.0654	4.2870	1.8236	-4.2530	0.0001	0.0025
metab_8009	neg	304.0677	0.8662	0.2619	0.1159	0.3829	0.5125
metab_1135	pos	304.0783	0.6262	0.2934	0.5574	0.5075	0.6297
metab_13548	neg	304.0808	2.7155	0.0787	0.4289	0.7324	0.8070
metab_8233	neg	304.0962	1.3817	0.1710	-0.0589	0.7336	0.8079
metab_8611	neg	304.1041	2.0516	0.1281	0.3464	0.6981	0.7834
metab_7561	neg	304.1153	2.1630	0.1163	0.7479	0.7309	0.8063
metab_5287	pos	304.1171	2.7341	1.1851	-2.4626	0.0228	0.0640
metab_5801	pos	304.1171	1.6128	0.8344	3.4501	0.1156	0.2108
metab_13337	neg	304.1193	3.1964	0.5098	-0.1841	0.0802	0.1656
metab_9117	neg	304.1195	3.4639	0.3107	0.1173	0.3719	0.5034
metab_1039	pos	304.1283	1.6693	0.4337	0.9338	0.4105	0.5433
metab_2114	pos	304.1323	3.2880	1.0879	-1.8453	0.0544	0.1215
metab_12497	neg	304.1346	6.3759	1.5542	-2.7821	0.0012	0.0094
metab_12989	neg	304.1346	4.2367	1.3009	-1.5161	0.0000	0.0005
metab_12898	neg	304.1347	4.5884	2.1364	-5.2403	0.0000	0.0001
metab_5077	pos	304.1385	3.4251	0.5206	-1.6113	0.3326	0.4675
metab_14001	neg	304.1402	1.8524	1.9722	6.6195	0.0115	0.0434
metab_1038	pos	304.1467	1.6838	0.9466	-1.4171	0.0095	0.0347
metab_1586	pos	304.1640	1.4569	0.4142	0.3019	0.3651	0.4986
metab_1788	pos	304.1649	2.0828	0.4248	0.0617	0.4463	0.5763
metab_4947	pos	304.1746	3.9421	1.7900	5.3773	0.0000	0.0000
metab_6618	neg	304.1883	0.5286	0.9479	-1.2969	0.0277	0.0788
metab_9756	neg	304.1925	6.4874	0.0943	0.6256	0.7788	0.8423
metab_1022	pos	304.2110	2.0973	1.4344	2.3959	0.0000	0.0004
metab_2087	pos	304.2740	3.2116	0.0280	0.0096	0.8629	0.9125
metab_4279	pos	304.2838	7.3216	0.4933	-0.3957	0.3866	0.5189
metab_3525	pos	304.2989	14.0074	0.2493	-0.2247	0.0945	0.1821
metab_2623	pos	304.2990	6.7516	0.1927	-0.1510	0.2125	0.3336
metab_10914	neg	304.8697	14.5672	0.2034	0.0306	0.2996	0.4300
metab_15038	neg	304.9091	0.4975	0.6149	-0.2497	0.0046	0.0229
metab_6641	neg	304.9145	14.0438	0.1399	0.1512	0.4265	0.5520
metab_6772	neg	304.9919	0.7254	0.6309	-0.4865	0.1638	0.2789
metab_9203	neg	305.0573	3.7658	0.1144	-0.0969	0.7616	0.8288
metab_14343	neg	305.0814	1.4568	0.4038	-1.3159	0.4575	0.5806
metab_468	pos	305.1124	2.5627	0.4672	-0.4219	0.2139	0.3351
metab_1453	pos	305.1334	1.0741	0.9262	1.4717	0.0794	0.1600

metab_6316	pos	305.1445	0.6262	0.3035	0.5975	0.6035	0.7135
metab_1981	pos	305.1488	2.7804	0.5189	-0.7638	0.1161	0.2115
metab_13400	neg	305.1511	3.0432	0.4847	0.4520	0.5145	0.6331
metab_1897	pos	305.1598	2.4224	0.2254	1.6125	0.8228	0.8836
metab_5763	pos	305.1695	1.6838	0.3724	-0.0337	0.4395	0.5702
metab_13677	neg	305.1873	2.4487	1.9889	11.0726	0.0142	0.0500
metab_4762	pos	305.2102	4.8208	1.3583	4.6422	0.0239	0.0666
metab_4795	pos	305.2103	4.6096	1.0688	13.1452	0.1249	0.2238
metab_6683	neg	305.8608	0.0197	0.4191	-0.1323	0.0265	0.0765
metab_6365	pos	306.0066	0.5983	1.4096	-1.9984	0.0000	0.0011
metab_13633	neg	306.0077	2.5264	0.1889	0.5201	0.7060	0.7894
metab_6132	pos	306.0727	1.0321	0.0516	-0.9807	0.8992	0.9348
metab_7940	neg	306.0767	0.6271	0.2228	3.1035	0.7157	0.7962
metab_12392	neg	306.0770	6.7926	0.6999	2.2907	0.2739	0.4045
metab_9359	neg	306.0770	4.3549	1.0614	3.9201	0.0921	0.1826
metab_13287	neg	306.0887	3.3304	0.8624	-0.7897	0.1414	0.2504
metab_13217	neg	306.0979	3.5475	1.0281	-2.6591	0.0556	0.1269
metab_8338	neg	306.0986	1.5297	0.8304	-1.8405	0.0987	0.1922
metab_8882	neg	306.0987	2.7318	0.8033	-1.7796	0.0830	0.1695
metab_14069	neg	306.0987	1.7572	0.7842	-0.9646	0.1241	0.2268
metab_13447	neg	306.0987	2.9429	1.0564	-1.7429	0.0294	0.0816
metab_5927	pos	306.1089	1.3865	0.6545	0.4378	0.2438	0.3684
metab_9456	neg	306.1140	4.7881	1.5728	-2.7382	0.0001	0.0014
metab_9478	neg	306.1141	4.9021	1.6835	-3.0898	0.0003	0.0037
metab_5596	pos	306.1174	2.0360	0.1399	-0.2117	0.6980	0.7892
metab_9019	neg	306.1196	3.0774	0.8685	0.8787	0.1713	0.2877
metab_13339	neg	306.1351	3.1964	0.3772	0.4874	0.5206	0.6384
metab_6186	pos	306.1537	0.8920	0.7768	-3.1406	0.2493	0.3748
metab_9210	neg	306.1716	3.7658	0.8085	-1.2911	0.1404	0.2493
metab_1579	pos	306.1802	1.4429	1.3537	14.2375	0.0100	0.0360
metab_6608	neg	306.2039	0.5286	0.6684	-0.4449	0.1701	0.2866
metab_2624	pos	306.2055	6.7516	0.9929	1.8261	0.0798	0.1607
metab_12719	neg	306.2077	5.3546	0.1638	1.2284	0.7248	0.8022
metab_13062	neg	306.2079	4.0182	1.3408	2.3313	0.0007	0.0066
metab_12407	neg	306.2080	6.7439	1.2593	2.1382	0.0045	0.0227
metab_4395	pos	306.2418	6.8433	1.0864	11.1954	0.0328	0.0839
metab_2761	pos	306.2782	7.5307	0.7737	0.7901	0.1171	0.2128
metab_4364	pos	306.2782	6.9477	0.7843	-1.0243	0.0172	0.0521
metab_4218	pos	306.2783	7.6776	0.5925	0.4712	0.0360	0.0892
metab_3063	pos	306.2783	9.5137	1.0569	-1.3681	0.0012	0.0086
metab_3513	pos	306.8937	14.0378	0.2321	0.1609	0.2429	0.3676
metab_7695	neg	306.9396	0.5126	0.5575	-0.2611	0.0118	0.0441
metab_1278	pos	306.9486	0.5560	0.0588	0.3643	0.8531	0.9056
metab_6236	pos	307.0426	0.8081	0.6437	-0.8801	0.1592	0.2678
metab_13262	neg	307.0730	3.4133	0.4969	-0.4183	0.1895	0.3088
metab_152	pos	307.0778	0.5983	0.5840	-0.6541	0.0963	0.1841
metab_13490	neg	307.0827	2.8269	1.6608	3.1002	0.0010	0.0086
metab_2391	pos	307.0857	4.8809	0.5194	-1.2516	0.4179	0.5498

metab_13052	neg	307.1094	4.0516	0.6500	3.8649	0.5596	0.6708
metab_14855	neg	307.1150	0.5851	0.7385	-0.7067	0.1008	0.1951
metab_12871	neg	307.1225	4.6722	0.0246	1.7225	0.8887	0.9254
metab_9199	neg	307.1227	3.7488	1.5504	3.0691	0.0126	0.0463
metab_13472	neg	307.1229	2.8606	0.2764	-1.0003	0.5333	0.6491
metab_287	pos	307.1281	1.1875	0.7498	0.6552	0.0051	0.0222
metab_8779	neg	307.1303	2.4487	0.2409	0.2909	0.6223	0.7231
metab_8675	neg	307.1303	2.2119	0.1020	0.3490	0.8096	0.8645
metab_4861	pos	307.1508	4.3512	1.1945	1.5565	0.0001	0.0014
metab_7655	neg	307.1514	0.5431	0.9652	-0.4274	0.1768	0.2942
metab_12966	neg	307.1556	4.3207	1.7909	7.2277	0.0021	0.0134
metab_5190	pos	307.1644	3.0264	0.1023	0.1652	0.8540	0.9063
metab_8477	neg	307.1667	1.7729	0.6275	-0.8866	0.1758	0.2929
metab_8941	neg	307.1766	2.8606	2.8439	8.5683	0.0007	0.0068
metab_584	pos	307.1896	4.2457	1.5364	2.2334	0.0012	0.0083
metab_611	pos	307.1897	3.9270	1.3904	2.1088	0.0069	0.0276
metab_12775	neg	307.1917	5.0650	0.0984	-0.1123	0.6800	0.7698
metab_12602	neg	307.1918	5.8896	0.0502	0.4987	0.8449	0.8925
metab_9442	neg	307.1919	4.7384	0.5765	0.5166	0.2575	0.3864
metab_9385	neg	307.1920	4.5054	0.6231	0.6930	0.1766	0.2938
metab_4275	pos	307.2259	7.3370	1.4054	1.9757	0.0022	0.0128
metab_4032	pos	307.2259	8.4651	0.9744	4.7868	0.0299	0.0785
metab_24	pos	307.2259	6.4035	0.7745	-0.5283	0.0980	0.1867
metab_5000	pos	307.2260	3.7148	1.5201	12.0061	0.0090	0.0335
metab_10218	neg	307.2282	8.4336	0.3498	-0.6132	0.3625	0.4949
metab_4178	pos	307.2623	7.9118	1.8154	-3.3599	0.0005	0.0046
metab_10957	neg	307.8562	14.1744	0.6032	-0.2472	0.0014	0.0108
metab_6681	neg	307.8563	0.0197	0.2635	-0.0712	0.2101	0.3328
metab_8812	neg	307.9627	2.5264	1.6585	-2.9534	0.0002	0.0034
metab_14427	neg	308.0782	1.3231	0.8829	-1.8819	0.0504	0.1181
metab_1809	pos	308.0901	2.1290	1.9717	4.8007	0.0000	0.0001
metab_2299	pos	308.0903	4.2907	0.4411	0.8969	0.4381	0.5687
metab_5342	pos	308.0903	2.5627	1.1733	1.5699	0.0051	0.0224
metab_1905	pos	308.0903	2.4370	0.4103	0.7898	0.4944	0.6185
metab_6739	neg	308.0990	0.5991	1.4620	-3.0165	0.0045	0.0226
metab_7591	neg	308.1143	2.3855	1.1739	-1.4167	0.0007	0.0068
metab_13580	neg	308.1143	2.6524	0.2647	-0.1385	0.3891	0.5178
metab_14576	neg	308.1255	1.0785	0.9827	1.5772	0.0817	0.1677
metab_9378	neg	308.1296	4.4722	1.3087	-3.0972	0.0261	0.0758
metab_14041	neg	308.1352	1.8046	1.1433	-1.7270	0.0569	0.1293
metab_9049	neg	308.1414	3.1964	0.4330	0.2919	0.4534	0.5767
metab_517	pos	308.1484	3.3032	0.5441	-0.2725	0.3317	0.4664
metab_2153	pos	308.1751	3.4702	0.6025	0.2429	0.4535	0.5827
metab_1604	pos	308.1848	1.5144	1.2235	9.1460	0.0005	0.0047
metab_31	pos	308.2171	0.5140	0.7187	-0.8431	0.1864	0.3016
metab_4411	pos	308.2211	6.7516	1.3466	1.9395	0.0017	0.0108
metab_2560	pos	308.2211	6.0864	0.8133	11.6340	0.0738	0.1518
metab_626	pos	308.2212	5.0451	0.2937	0.5091	0.5239	0.6450

metab_570	pos	308.2212	4.0479	1.2900	1.9366	0.0003	0.0035
metab_3972	pos	308.2936	8.7008	0.9475	-0.9855	0.0001	0.0020
metab_2889	pos	308.2938	8.4214	1.5336	-2.2286	0.0007	0.0061
metab_15140	neg	308.8643	0.0197	0.3392	-0.0393	0.0700	0.1499
metab_14908	neg	309.0375	0.5431	1.8150	4.5349	0.0023	0.0143
metab_14239	neg	309.0624	1.5297	1.7477	5.1260	0.0053	0.0256
metab_5081	pos	309.0862	3.4098	0.9830	-1.5737	0.0646	0.1374
metab_5552	pos	309.0863	2.1132	0.7526	1.6959	0.2323	0.3561
metab_428	pos	309.0959	2.2045	0.6324	3.3279	0.4065	0.5396
metab_9065	neg	309.0985	3.2473	2.2046	9.3912	0.0014	0.0104
metab_13735	neg	309.1018	2.3380	2.2024	7.2221	0.0001	0.0023
metab_6050	pos	309.1073	1.1875	0.3255	0.3368	0.4381	0.5687
metab_14515	neg	309.1103	1.2377	0.8176	-0.7820	0.0057	0.0269
metab_150	pos	309.1282	0.6402	0.8256	-1.2209	0.0507	0.1151
metab_383	pos	309.1435	1.8577	0.5459	0.2320	0.0928	0.1796
metab_5375	pos	309.1436	2.4850	0.1701	0.1771	0.6175	0.7254
metab_429	pos	309.1436	2.2209	0.1321	-0.0896	0.6474	0.7496
metab_13174	neg	309.1474	3.6645	1.1992	1.8651	0.0185	0.0596
metab_7854	neg	309.1565	0.5286	0.2219	0.4359	0.7247	0.8022
metab_5236	pos	309.1589	2.8877	1.7385	2.9842	0.0432	0.1022
metab_4951	pos	309.1589	3.9270	0.3465	-0.0504	0.7076	0.7964
metab_204	pos	309.1645	0.6682	1.3325	-2.2322	0.0332	0.0845
metab_1168	pos	309.1648	0.5280	1.2376	-1.7875	0.0624	0.1338
metab_12967	neg	309.1714	4.3207	1.5801	4.1343	0.0109	0.0419
metab_9630	neg	309.1714	5.7751	0.0075	0.1775	0.9552	0.9707
metab_12665	neg	309.1715	5.6292	0.0994	0.2568	0.7416	0.8138
metab_9869	neg	309.1744	7.0322	0.2808	0.0090	0.4605	0.5832
metab_12388	neg	309.1746	6.8093	0.3501	0.9408	0.2101	0.3328
metab_378	pos	309.1798	1.7554	0.0764	0.2446	0.8528	0.9055
metab_9328	neg	309.1824	4.2203	1.4680	2.8224	0.0010	0.0088
metab_2533	pos	309.2050	5.9049	1.0143	1.5015	0.0007	0.0061
metab_2432	pos	309.2051	5.1372	0.6004	0.6204	0.0228	0.0640
metab_4427	pos	309.2051	6.6001	1.0583	1.1515	0.0012	0.0083
metab_2303	pos	309.2052	4.3371	0.9218	0.8044	0.0008	0.0064
metab_6673	neg	309.2076	5.6615	0.7395	-0.6547	0.0406	0.1018
metab_5128	pos	309.2262	3.2274	1.1368	2.8474	0.0458	0.1069
metab_679	pos	309.2415	6.5395	1.4653	4.1341	0.0162	0.0500
metab_4197	pos	309.2415	7.7946	1.5067	2.5408	0.0077	0.0299
metab_2703	pos	309.2415	7.1416	1.6820	4.3845	0.0004	0.0042
metab_11896	neg	309.2439	8.3055	0.8012	1.1480	0.0429	0.1060
metab_11762	neg	309.2440	8.6874	1.3069	-2.2201	0.0011	0.0090
metab_11571	neg	309.2440	9.1038	0.8774	-1.0342	0.0046	0.0229
metab_10810	neg	309.8565	14.0771	0.4831	-0.1095	0.0052	0.0251
metab_3402	pos	309.9477	14.6110	0.2940	-0.2638	0.0708	0.1472
metab_3417	pos	309.9478	14.4556	0.4417	-0.3843	0.0497	0.1135
metab_1179	pos	309.9737	0.4999	0.9182	-1.4810	0.0159	0.0494
metab_13625	neg	309.9784	2.5431	1.8107	-2.9980	0.0000	0.0008
metab_1237	pos	310.0364	0.5140	0.5951	-0.6259	0.0366	0.0904

metab_8746	neg	310.0395	2.3704	1.4623	14.8780	0.0059	0.0274
metab_8153	neg	310.0573	1.2663	0.0359	0.1143	0.8725	0.9124
metab_7592	neg	310.0573	2.3855	0.2136	1.7226	0.6451	0.7414
metab_14313	neg	310.0937	1.4710	0.8414	-0.6940	0.0015	0.0108
metab_9357	neg	310.0937	4.3380	1.9230	7.1566	0.0019	0.0127
metab_9157	neg	310.0938	3.5813	1.6753	10.7257	0.0027	0.0161
metab_6851	neg	310.1052	1.2236	1.0107	1.8310	0.0806	0.1662
metab_12781	neg	310.1087	5.0159	1.8177	-3.6902	0.0015	0.0108
metab_6924	neg	310.1089	3.7488	0.6206	0.2594	0.2719	0.4023
metab_14760	neg	310.1146	0.6551	0.2101	1.4050	0.7858	0.8470
metab_14707	neg	310.1146	0.7958	0.2472	1.4356	0.7298	0.8058
metab_5428	pos	310.1276	2.3751	1.1747	-1.5661	0.0000	0.0009
metab_7	pos	310.1276	1.2585	1.3726	-2.2355	0.0136	0.0444
metab_1955	pos	310.1276	2.6561	0.2415	0.3807	0.5915	0.7034
metab_8201	neg	310.1300	1.3231	1.5356	-2.3364	0.0318	0.0862
metab_13859	neg	310.1301	2.0987	0.3197	-0.3087	0.3818	0.5115
metab_6121	pos	310.1387	1.0601	1.2341	1.7053	0.0683	0.1434
metab_14281	neg	310.1407	1.5012	1.2537	4.2328	0.2042	0.3259
metab_6041	pos	310.1571	1.2016	1.6820	3.7639	0.0002	0.0026
metab_1596	pos	310.1638	1.5004	0.3614	-0.7042	0.5401	0.6602
metab_6023	pos	310.1750	1.2162	0.8516	2.2576	0.1639	0.2738
metab_5217	pos	310.1835	2.9501	1.6086	2.8785	0.0008	0.0067
metab_1236	pos	310.1866	0.5140	1.5749	4.3059	0.0006	0.0055
metab_569	pos	310.2010	4.0028	1.7648	3.9062	0.0004	0.0045
metab_1219	pos	310.2227	0.4999	0.9750	-0.9438	0.1631	0.2727
metab_4989	pos	310.2368	3.7599	0.7266	0.9801	0.0632	0.1351
metab_5175	pos	310.2845	3.0877	0.4220	0.5334	0.3194	0.4523
metab_5098	pos	310.2845	3.3337	0.5804	1.0062	0.3538	0.4886
metab_3358	pos	310.3095	14.6110	0.4719	-0.3631	0.0013	0.0091
metab_1191	pos	310.3095	0.2224	0.4981	-0.3907	0.0005	0.0047
metab_960	pos	310.3096	9.1302	0.0803	-0.1847	0.8280	0.8869
metab_4997	pos	310.3097	3.7297	0.6642	-0.5876	0.0014	0.0094
metab_6414	pos	310.8276	0.5420	0.0645	0.0314	0.7763	0.8492
metab_7197	neg	310.9314	14.0282	0.2451	-0.0518	0.2086	0.3310
metab_7957	neg	311.0623	0.6831	0.2597	1.9568	0.8396	0.8880
metab_6591	neg	311.0986	0.6131	0.9186	-0.8865	0.0024	0.0150
metab_13375	neg	311.1045	3.1115	0.8889	-1.3380	0.1083	0.2061
metab_1781	pos	311.1226	2.0524	0.6623	-1.3671	0.2014	0.3201
metab_13253	neg	311.1265	3.4303	0.7936	0.6709	0.4239	0.5496
metab_5393	pos	311.1385	2.4370	0.2010	-0.1453	0.4986	0.6215
metab_13605	neg	311.1406	2.6046	1.0006	-1.2011	0.0168	0.0560
metab_2256	pos	311.1533	3.9876	1.6397	-4.3145	0.0000	0.0008
metab_415	pos	311.1591	2.0973	0.2046	-0.0493	0.6107	0.7204
metab_5951	pos	311.1593	1.3437	1.9228	3.8974	0.0000	0.0001
metab_1046	pos	311.1596	1.6128	1.5548	2.3772	0.0000	0.0000
metab_12319	neg	311.1690	7.0162	0.2521	0.1971	0.4803	0.6026
metab_11744	neg	311.1691	8.7031	0.8636	-0.5399	0.0000	0.0005
metab_9500	neg	311.1868	5.0812	0.3078	-0.2581	0.3730	0.5042

metab_5181	pos	311.1954	3.0572	0.6144	-0.9143	0.3843	0.5170
metab_4891	pos	311.1956	4.2150	1.4689	2.7714	0.0011	0.0080
metab_290	pos	311.1956	1.2016	0.8245	1.1634	0.0300	0.0787
metab_12175	neg	311.2021	7.5087	1.0701	-1.2902	0.0018	0.0122
metab_130	pos	311.2207	6.3277	0.7457	-0.8979	0.0284	0.0757
metab_2474	pos	311.2208	5.4662	0.4844	0.2897	0.1168	0.2125
metab_4755	pos	311.2208	4.8809	0.7554	0.6419	0.0144	0.0459
metab_9567	neg	311.2231	5.4684	0.5531	-0.7409	0.2566	0.3850
metab_12595	neg	311.2232	5.9375	0.6222	-0.5612	0.2393	0.3654
metab_12514	neg	311.2233	6.2943	0.4942	-0.5216	0.2232	0.3473
metab_3893	pos	311.2570	9.0857	0.8429	1.6358	0.0990	0.1879
metab_877	pos	311.2571	10.1647	1.7873	1.8952	0.0392	0.0951
metab_4181	pos	311.2571	7.8978	0.9401	1.1857	0.1184	0.2147
metab_86	pos	311.2571	7.0670	1.0786	2.1322	0.0545	0.1216
metab_901	pos	311.2571	9.9307	1.0735	1.5394	0.0860	0.1697
metab_7693	neg	311.9629	3.1964	0.3689	0.2103	0.5507	0.6627
metab_13913	neg	312.0491	2.0055	0.5810	1.5678	0.3550	0.4879
metab_9706	neg	312.0681	6.2617	0.0451	0.4785	0.8984	0.9320
metab_6592	neg	312.0729	1.3231	0.1400	-0.1409	0.6187	0.7206
metab_5882	pos	312.1068	1.4714	0.7031	-0.6435	0.0813	0.1627
metab_14139	neg	312.1092	1.6491	0.6007	0.8277	0.1055	0.2019
metab_14525	neg	312.1205	1.2084	0.7621	1.5310	0.1623	0.2771
metab_2203	pos	312.1222	3.6987	0.8107	0.1044	0.2259	0.3492
metab_9321	neg	312.1245	4.1869	1.9662	-3.9006	0.0000	0.0007
metab_9143	neg	312.1246	3.5475	0.4547	0.3488	0.3222	0.4546
metab_9415	neg	312.1246	4.6216	1.6928	-2.7781	0.0000	0.0007
metab_2191	pos	312.1333	3.6535	0.1153	0.0819	0.9428	0.9641
metab_1848	pos	312.1338	2.2519	0.5697	-0.8476	0.2397	0.3642
metab_8695	neg	312.1358	2.2437	0.3003	0.4515	0.5796	0.6884
metab_5879	pos	312.1428	1.4860	1.0136	-1.6529	0.0110	0.0383
metab_8924	neg	312.1455	2.8269	0.7836	1.2114	0.0478	0.1140
metab_13987	neg	312.1568	1.8682	0.2619	1.7721	0.7196	0.7989
metab_14197	neg	312.1569	1.5736	0.5610	3.0908	0.5808	0.6894
metab_6363	pos	312.1656	0.5983	1.6343	15.1257	0.0011	0.0082
metab_9358	neg	312.1821	4.3549	0.9732	-0.7584	0.1835	0.3019
metab_2240	pos	312.2164	3.8967	2.1669	5.4473	0.0001	0.0012
metab_4712	pos	312.2166	5.0009	0.6358	0.8334	0.3178	0.4505
metab_4693	pos	312.2524	5.1218	1.2128	1.9331	0.0005	0.0048
metab_2185	pos	312.2524	3.6229	1.2178	2.3208	0.0065	0.0265
metab_2372	pos	312.2524	4.7001	1.3666	2.3502	0.0007	0.0060
metab_5083	pos	312.2525	3.3943	1.4653	2.8433	0.0008	0.0064
metab_10167	neg	312.2548	8.2253	0.6030	-0.1855	0.1249	0.2281
metab_12694	neg	312.2548	5.5002	0.8862	3.0177	0.1171	0.2172
metab_11859	neg	312.2549	8.4336	0.6239	-0.3115	0.1205	0.2220
metab_11763	neg	312.2549	8.6874	0.9385	-0.8220	0.0395	0.0999
metab_975	pos	312.2888	7.7946	0.9888	1.5032	0.0529	0.1189
metab_3880	pos	312.3162	9.1302	0.0849	-0.1998	0.8097	0.8741
metab_3785	pos	312.3251	9.5749	0.4907	-0.5414	0.0761	0.1549

metab_3005	pos	312.3252	9.0999	0.7268	-0.8054	0.0030	0.0155
metab_14980	neg	312.9037	0.5126	1.0491	-1.6763	0.0097	0.0390
metab_7800	neg	312.9170	0.4975	0.0698	0.1664	0.7753	0.8399
metab_3485	pos	312.9420	14.0525	0.5955	-0.6559	0.0111	0.0384
metab_14486	neg	313.0571	1.2663	0.7351	-1.0792	0.1004	0.1946
metab_14106	neg	313.0572	1.6948	0.1947	-0.4788	0.7481	0.8201
metab_8768	neg	313.0721	2.4175	2.0325	-4.8502	0.0000	0.0005
metab_7876	neg	313.0812	0.5571	0.3324	-0.3128	0.5306	0.6470
metab_9017	neg	313.0836	3.0774	0.3802	2.3664	0.5323	0.6484
metab_12494	neg	313.0916	6.3917	0.4006	0.9245	0.2809	0.4113
metab_14415	neg	313.0936	1.3373	0.8034	1.3650	0.0181	0.0590
metab_1841	pos	313.1106	2.2209	0.0037	0.8160	0.9159	0.9450
metab_6891	neg	313.1145	0.5991	0.3729	-0.0080	0.3347	0.4676
metab_5241	pos	313.1174	2.8725	1.0217	-1.6861	0.0688	0.1443
metab_9110	neg	313.1202	3.4303	1.0720	-1.0254	0.1553	0.2684
metab_7529	neg	313.1303	1.9596	0.4349	0.8809	0.3852	0.5143
metab_1090	pos	313.1384	1.2162	1.0676	1.0542	0.0001	0.0016
metab_5328	pos	313.1541	2.6090	0.6515	-0.8363	0.0709	0.1474
metab_550	pos	313.1542	2.2519	0.7669	2.0230	0.1451	0.2501
metab_5525	pos	313.1637	2.1595	1.8148	3.8316	0.0001	0.0013
metab_349	pos	313.1747	1.5705	1.2045	2.0601	0.0072	0.0285
metab_1628	pos	313.1747	1.5565	1.6855	3.6908	0.0005	0.0051
metab_294	pos	313.1749	1.2162	0.8268	0.8097	0.0165	0.0508
metab_9103	neg	313.1772	3.3971	0.1715	-1.1666	0.7893	0.8493
metab_13922	neg	313.1773	1.9904	0.1811	0.9676	0.8058	0.8612
metab_9587	neg	313.2025	5.5808	0.0186	-0.9376	0.9587	0.9726
metab_9208	neg	313.2026	3.7658	0.1142	2.2479	0.9569	0.9715
metab_9383	neg	313.2138	4.5054	0.7292	1.5492	0.2240	0.3483
metab_141	pos	313.2364	5.2868	0.1952	-0.5122	0.6663	0.7644
metab_2295	pos	313.2364	4.2301	1.2034	3.6953	0.1359	0.2382
metab_6623	neg	313.2389	6.4073	0.2695	0.7962	0.4075	0.5350
metab_3032	pos	313.2726	9.2815	0.5767	0.8597	0.2070	0.3269
metab_778	pos	313.2726	8.4356	0.2710	0.0130	0.5728	0.6882
metab_982	pos	313.2727	7.5005	1.1813	-1.4327	0.0000	0.0008
metab_2960	pos	313.2727	8.8466	0.5198	-0.3899	0.1815	0.2958
metab_3121	pos	313.2727	9.8673	0.9128	-1.3861	0.0203	0.0588
metab_4133	pos	313.2727	8.0843	1.0058	-1.1339	0.0005	0.0046
metab_3178	pos	313.2728	10.3326	1.1950	1.8296	0.0571	0.1258
metab_12484	neg	313.3870	6.4073	0.2891	0.8750	0.4085	0.5357
metab_8592	neg	313.9978	2.0055	0.1320	0.7591	0.9082	0.9392
metab_14217	neg	314.0887	1.5586	0.1023	0.2231	0.9137	0.9428
metab_155	pos	314.0907	0.7801	0.1535	0.9391	0.8846	0.9268
metab_14622	neg	314.0996	0.9649	0.1045	0.8788	0.6713	0.7625
metab_13716	neg	314.1151	2.3855	0.1480	0.4461	0.5453	0.6583
metab_14482	neg	314.1362	1.2663	0.8610	-1.4089	0.0908	0.1810
metab_4803	pos	314.1378	4.6096	1.8208	-3.5067	0.0001	0.0014
metab_9451	neg	314.1402	4.7709	1.5585	-4.0714	0.0017	0.0116
metab_13048	neg	314.1403	4.0516	0.7083	-2.2381	0.2905	0.4218

metab_5373	pos	314.1493	2.4850	0.1198	0.3729	0.8896	0.9298
metab_1797	pos	314.1500	2.0973	0.4682	0.4309	0.1262	0.2250
metab_5998	pos	314.1521	1.2585	0.0804	-1.1429	0.8390	0.8945
metab_9016	neg	314.1615	3.0774	0.1541	0.9204	0.7830	0.8452
metab_9195	neg	314.1620	3.7317	1.6209	3.9959	0.0159	0.0542
metab_8372	neg	314.1624	1.5736	0.4325	1.3198	0.4070	0.5344
metab_8584	neg	314.1725	1.9904	1.2808	3.9606	0.0514	0.1197
metab_2294	pos	314.1952	4.2301	0.0816	0.2351	0.9894	0.9930
metab_1827	pos	314.1953	2.1746	1.2680	1.6349	0.0000	0.0006
metab_5133	pos	314.1954	3.2274	1.5292	2.8181	0.0015	0.0098
metab_2334	pos	314.1954	4.4730	0.0568	0.0299	0.9690	0.9811
metab_9390	neg	314.1979	4.5391	0.0850	0.2927	0.9347	0.9578
metab_8826	neg	314.2089	2.5748	1.6165	2.8874	0.0008	0.0074
metab_5242	pos	314.2102	2.8725	1.7541	-3.7332	0.0001	0.0020
metab_5220	pos	314.2317	2.9501	1.9406	5.1115	0.0012	0.0086
metab_2099	pos	314.2318	3.2274	1.6076	5.9401	0.0031	0.0160
metab_2311	pos	314.2319	4.3672	2.5554	8.3344	0.0002	0.0030
metab_616	pos	314.2321	4.8809	1.1107	1.5300	0.0040	0.0188
metab_4058	pos	314.2469	8.3763	1.3609	2.1949	0.0002	0.0024
metab_4908	pos	314.2680	4.0939	0.5676	6.5154	0.3334	0.4678
metab_4672	pos	314.2680	5.2573	0.5624	1.3366	0.1740	0.2867
metab_4626	pos	314.2680	5.5112	1.1533	2.3022	0.0282	0.0754
metab_7817	neg	314.9003	0.5126	1.2869	-2.1008	0.0005	0.0057
metab_1174	pos	314.9524	0.4999	0.9034	-1.8241	0.0571	0.1258
metab_6255	pos	315.0580	0.7801	0.9764	-0.7118	0.0120	0.0406
metab_14877	neg	315.0715	0.5571	0.3905	1.2043	0.6277	0.7272
metab_8603	neg	315.0728	2.0202	1.3333	10.8548	0.0226	0.0686
metab_8674	neg	315.0996	2.1956	0.6659	0.8394	0.0373	0.0960
metab_12356	neg	315.1040	6.8887	1.0155	1.7307	0.0125	0.0459
metab_13799	neg	315.1089	2.2119	1.7085	3.2245	0.0027	0.0160
metab_1445	pos	315.1099	1.0601	0.3435	-0.3281	0.4141	0.5461
metab_13979	neg	315.1201	1.8974	0.5241	-0.9840	0.2213	0.3452
metab_8202	neg	315.1202	1.3231	0.6691	1.1051	0.0494	0.1165
metab_2145	pos	315.1331	3.4251	1.0047	-1.4035	0.0848	0.1679
metab_66	pos	315.1331	2.4224	0.5418	-0.5715	0.0616	0.1326
metab_9030	neg	315.1362	3.1286	0.4715	-0.3074	0.3323	0.4649
metab_13515	neg	315.1455	2.7792	1.7133	3.2140	0.0002	0.0034
metab_8827	neg	315.1457	2.5748	2.0734	5.5043	0.0004	0.0049
metab_13862	neg	315.1565	2.0987	0.1042	0.1426	0.6479	0.7439
metab_8185	neg	315.1678	1.2948	0.6107	3.3068	0.2929	0.4243
metab_8117	neg	315.1679	1.1796	1.0342	4.9418	0.0462	0.1114
metab_5380	pos	315.1695	2.4690	0.8486	-1.1224	0.0095	0.0346
metab_2229	pos	315.1808	3.8063	0.0601	1.6025	0.9411	0.9632
metab_12753	neg	315.1819	5.1772	0.1545	0.1409	0.3841	0.5132
metab_13182	neg	315.1821	3.6479	1.9080	4.3175	0.0002	0.0034
metab_9151	neg	315.1930	3.5813	0.1837	1.0693	0.7690	0.8346
metab_2340	pos	315.2271	4.5184	1.5407	3.0998	0.0164	0.0504
metab_775	pos	315.2310	8.4074	0.6880	1.4634	0.1123	0.2061

metab_2653	pos	315.2311	6.9035	1.3246	1.5910	0.0005	0.0050
metab_9736	neg	315.2446	6.4073	0.2685	0.8002	0.4263	0.5518
metab_6624	neg	315.2542	6.8887	0.7931	1.3646	0.0268	0.0771
metab_9828	neg	315.4032	6.8887	0.9680	1.7365	0.0209	0.0650
metab_7961	neg	316.0078	0.7254	0.0658	0.3014	0.7317	0.8067
metab_13746	neg	316.0818	2.3078	0.3685	0.2778	0.3527	0.4859
metab_7859	neg	316.1153	0.5431	0.7539	-0.6207	0.1876	0.3066
metab_13007	neg	316.1169	4.1869	0.2282	0.4785	0.6420	0.7391
metab_5233	pos	316.1172	2.9028	1.3310	-2.7265	0.0118	0.0402
metab_412	pos	316.1172	2.0524	0.5545	-0.7503	0.0557	0.1235
metab_1899	pos	316.1172	2.4224	0.6575	-1.0615	0.2440	0.3685
metab_8928	neg	316.1193	2.8269	0.9508	-1.2993	0.0178	0.0583
metab_1618	pos	316.1320	1.5424	1.4413	-3.5190	0.0037	0.0178
metab_1108	pos	316.1360	0.9620	0.7033	-1.1585	0.1110	0.2048
metab_14289	neg	316.1516	1.5012	0.1261	0.7179	0.7582	0.8261
metab_4924	pos	316.1534	4.0479	1.4468	-4.5064	0.0216	0.0618
metab_5219	pos	316.1536	2.9501	0.5602	3.3560	0.4558	0.5848
metab_13518	neg	316.1671	2.7792	0.0264	0.4212	0.8778	0.9165
metab_13135	neg	316.1776	3.7658	1.7502	3.7521	0.0049	0.0241
metab_8646	neg	316.1880	2.1311	1.5377	5.1678	0.0025	0.0153
metab_2306	pos	316.2111	4.3371	1.3295	3.4731	0.0001	0.0014
metab_1869	pos	316.2112	2.3292	0.9062	0.9592	0.0022	0.0129
metab_5338	pos	316.2220	2.5782	1.6442	2.6382	0.0028	0.0150
metab_1755	pos	316.2221	1.9758	3.3920	12.7463	0.0000	0.0000
metab_4867	pos	316.2474	4.3371	0.9155	0.4461	0.1735	0.2861
metab_4695	pos	316.6939	5.1076	0.8438	0.8659	0.0184	0.0546
metab_14906	neg	316.8641	0.5431	1.1345	-1.1739	0.0003	0.0043
metab_6684	neg	316.8700	0.0197	0.1673	0.0761	0.4547	0.5779
metab_7205	neg	316.9482	14.0282	0.2575	-0.0249	0.3302	0.4630
metab_183	pos	316.9864	0.5140	1.4711	-3.0345	0.0003	0.0038
metab_8144	neg	317.0340	1.2521	1.9960	4.1829	0.0002	0.0028
metab_8088	neg	317.0992	1.1069	0.6631	1.6400	0.1582	0.2720
metab_7663	neg	317.1071	2.9092	0.5522	1.2197	0.2024	0.3238
metab_9045	neg	317.1147	3.1797	0.4015	-0.2661	0.3357	0.4687
metab_14306	neg	317.1151	1.4710	0.5075	-0.3230	0.1504	0.2618
metab_14149	neg	317.1247	1.6335	0.3635	0.8665	0.5381	0.6530
metab_8388	neg	317.1356	1.6028	0.3066	0.1360	0.3807	0.5105
metab_8579	neg	317.1357	1.9904	0.2905	-0.0544	0.5197	0.6377
metab_12650	neg	317.1431	5.6776	0.9713	-0.1838	0.0442	0.1080
metab_7593	neg	317.1469	0.5431	0.8770	1.4207	0.0183	0.0594
metab_1044	pos	317.1487	1.6413	0.7996	1.1105	0.0476	0.1098
metab_6948	neg	317.1511	4.2367	0.2274	0.2018	0.4149	0.5411
metab_5395	pos	317.1584	2.4370	1.2920	1.9708	0.0014	0.0093
metab_13863	neg	317.1720	2.0987	0.0810	0.3315	0.9404	0.9615
metab_303	pos	317.1810	1.3153	0.7719	3.7194	0.1362	0.2386
metab_13288	neg	317.1973	3.3304	0.4900	1.1867	0.5347	0.6503
metab_5038	pos	317.2061	3.5771	0.1957	0.1906	0.6969	0.7885
metab_5260	pos	317.2063	2.8259	0.4918	1.3263	0.3857	0.5182

metab_986	pos	317.2078	7.0082	0.5731	-0.9371	0.2194	0.3417
metab_4765	pos	317.2102	4.8053	1.0498	13.0460	0.1249	0.2238
metab_2266	pos	317.2103	4.0338	1.3139	5.3019	0.0578	0.1267
metab_5629	pos	317.2170	1.9758	0.5319	0.5485	0.1148	0.2098
metab_4017	pos	317.2467	8.5554	0.1857	0.4349	0.7561	0.8331
metab_7391	neg	317.2602	6.8887	0.8321	1.4401	0.0276	0.0787
metab_12575	neg	317.2604	6.0024	0.2618	2.3438	0.8742	0.9140
metab_4382	pos	317.2679	6.8886	0.8952	2.1601	0.0792	0.1597
metab_7799	neg	317.9172	0.4975	0.1318	0.1781	0.7099	0.7924
metab_14439	neg	318.0290	1.3089	0.3303	-0.0676	0.2368	0.3626
metab_6330	pos	318.0940	0.6262	1.1047	-2.4000	0.0523	0.1178
metab_5403	pos	318.0960	2.4224	0.9507	-1.4598	0.0436	0.1028
metab_13982	neg	318.1102	1.8833	0.5973	-0.2309	0.2228	0.3470
metab_13824	neg	318.1193	2.1630	0.0109	0.5633	0.9571	0.9716
metab_8084	neg	318.1197	1.0785	3.2669	11.2714	0.0000	0.0000
metab_1316	pos	318.1285	0.6262	0.7574	-1.0343	0.0085	0.0322
metab_5259	pos	318.1323	2.8259	0.8857	-1.3033	0.0307	0.0799
metab_5141	pos	318.1328	3.2116	1.1435	-2.3157	0.0702	0.1463
metab_13242	neg	318.1352	3.4800	0.3478	0.5458	0.5899	0.6973
metab_2184	pos	318.1481	3.6073	0.7188	-1.0566	0.1485	0.2545
metab_4939	pos	318.1540	3.9876	0.5583	-2.0573	0.4069	0.5398
metab_5864	pos	318.1649	1.5144	0.0039	0.6404	0.9770	0.9870
metab_12744	neg	318.1712	5.2257	0.7589	-1.0709	0.0917	0.1820
metab_5277	pos	318.1805	2.7804	0.0535	0.3223	0.9950	0.9968
metab_5344	pos	318.1806	2.5627	0.2245	-0.0860	0.5965	0.7069
metab_32	pos	318.2015	0.5140	0.2165	-0.1638	0.4081	0.5409
metab_5534	pos	318.2019	2.1444	2.3790	6.0432	0.0013	0.0088
metab_4955	pos	318.2266	3.9114	1.9417	3.8629	0.0003	0.0038
metab_5139	pos	318.2267	3.2116	1.2543	1.9005	0.0078	0.0302
metab_1733	pos	318.2268	1.9169	1.3613	12.2116	0.0014	0.0093
metab_2674	pos	318.2394	7.0082	0.5467	-0.9996	0.1683	0.2789
metab_2648	pos	318.2396	6.8886	0.1475	0.1263	0.5577	0.6749
metab_4425	pos	318.2396	6.6148	0.0333	0.4557	0.8692	0.9164
metab_746	pos	318.2994	7.8532	0.4319	-0.5567	0.3742	0.5079
metab_2554	pos	318.2994	6.0410	0.3756	0.0303	0.1746	0.2875
metab_627	pos	318.6914	5.0610	0.2130	0.9529	0.7071	0.7961
metab_3444	pos	318.9427	14.1820	0.5660	-0.4664	0.0028	0.0150
metab_14495	neg	319.0582	1.2521	1.5558	11.3199	0.0012	0.0099
metab_14773	neg	319.0788	0.6271	0.6566	1.8924	0.2944	0.4253
metab_14379	neg	319.0863	1.3965	0.3149	0.4478	0.6505	0.7462
metab_7606	neg	319.0938	2.4970	0.3273	0.5122	0.5558	0.6676
metab_7585	neg	319.0939	2.3227	0.5963	0.9708	0.2471	0.3741
metab_7444	neg	319.0946	1.5586	0.1054	0.0787	0.6479	0.7439
metab_6103	pos	319.1127	1.1024	0.9442	2.4866	0.1408	0.2445
metab_14669	neg	319.1148	0.8803	0.7528	1.4889	0.0492	0.1163
metab_13181	neg	319.1225	3.6479	1.7166	6.3914	0.0161	0.0547
metab_6431	pos	319.1322	0.5280	0.4764	-1.8702	0.2076	0.3275
metab_14402	neg	319.1512	1.3517	0.3579	0.0192	0.2404	0.3665

metab_211	pos	319.1602	0.6262	1.3289	2.2235	0.0143	0.0458
metab_2104	pos	319.1644	3.2420	0.8685	-1.0401	0.2198	0.3423
metab_1969	pos	319.1644	2.7188	0.1533	-0.2903	0.7160	0.8027
metab_1893	pos	319.1646	2.4071	0.0446	0.0552	0.7647	0.8400
metab_2292	pos	319.1648	4.2150	1.3578	-1.5180	0.0665	0.1407
metab_13141	neg	319.1667	3.7658	0.2433	0.5887	0.7154	0.7961
metab_13977	neg	319.1765	1.8974	1.8990	4.5014	0.0016	0.0115
metab_14120	neg	319.1766	1.6797	1.4117	3.4037	0.0063	0.0286
metab_9715	neg	319.1914	6.2943	1.2779	11.8575	0.0338	0.0898
metab_13147	neg	319.2129	3.7488	1.1524	5.3955	0.1956	0.3159
metab_4286	pos	319.2245	7.2763	0.0497	0.0970	0.8562	0.9078
metab_4201	pos	319.2257	7.7514	0.2607	0.7263	0.5762	0.6906
metab_4363	pos	319.2258	6.9477	0.6028	1.0544	0.1160	0.2114
metab_4588	pos	319.2258	5.7385	0.9789	1.3071	0.0018	0.0112
metab_4452	pos	319.2258	6.4489	0.5865	1.0506	0.1313	0.2320
metab_4066	pos	319.2259	8.3322	1.0058	1.6794	0.0120	0.0406
metab_3963	pos	319.2622	8.7161	0.3019	-0.6892	0.4109	0.5437
metab_12390	neg	319.8173	6.7926	0.5109	-0.3087	0.0837	0.1706
metab_10722	neg	319.8173	13.8980	0.7131	-0.3448	0.0001	0.0022
metab_12532	neg	319.8173	6.1968	0.4040	-0.2383	0.2894	0.4208
metab_12566	neg	319.8173	6.0346	0.3914	0.0281	0.2654	0.3956
metab_12499	neg	319.8175	6.3593	0.3067	-0.2009	0.4464	0.5699
metab_13642	neg	319.9632	2.5264	1.4117	-2.1688	0.0004	0.0046
metab_13630	neg	319.9996	2.5431	1.0463	-0.9178	0.0016	0.0113
metab_8692	neg	320.0779	2.2437	0.3900	0.0337	0.1482	0.2591
metab_13565	neg	320.0779	2.6678	0.4239	-0.2480	0.2025	0.3239
metab_1350	pos	320.0917	0.7661	1.1017	-2.3445	0.0018	0.0111
metab_14677	neg	320.0924	0.8662	0.7268	1.3731	0.1135	0.2128
metab_7981	neg	320.0990	0.7818	0.4096	-0.3766	0.3434	0.4766
metab_14566	neg	320.1075	1.1069	0.4419	2.1759	0.6102	0.7136
metab_402	pos	320.1230	1.9918	0.2849	-0.1684	0.4549	0.5844
metab_14012	neg	320.1255	1.8371	0.4539	1.0661	0.6504	0.7462
metab_8594	neg	320.1258	2.0055	0.2452	0.6725	0.6216	0.7225
metab_9539	neg	320.1293	5.2899	1.3564	-2.4436	0.0017	0.0119
metab_1484	pos	320.1417	1.1589	0.5588	-0.4433	0.0598	0.1297
metab_7434	neg	320.1508	3.6645	0.5350	-0.2977	0.2684	0.3984
metab_5516	pos	320.1600	2.1746	0.2025	-0.0341	0.5473	0.6659
metab_4646	pos	320.1846	5.3757	0.6190	-0.0195	0.3556	0.4902
metab_2444	pos	320.1848	5.2277	0.5635	-1.1999	0.1817	0.2959
metab_12547	neg	320.1871	6.1320	1.6490	2.9261	0.0001	0.0026
metab_1231	pos	320.2171	0.5140	0.9227	-1.3098	0.1084	0.2012
metab_2656	pos	320.2552	6.9035	0.1568	-0.1146	0.6733	0.7697
metab_4186	pos	320.3058	7.8532	0.4539	-0.4848	0.3775	0.5107
metab_7208	neg	320.8420	13.9952	0.1523	0.1612	0.5928	0.6997
metab_6434	pos	320.8654	0.5280	1.0169	-1.2723	0.0251	0.0690
metab_7760	neg	320.8866	0.0589	0.3658	-0.1542	0.0709	0.1511
metab_7161	neg	320.8866	14.0610	0.5861	-0.1859	0.0006	0.0064
metab_1354	pos	321.0573	0.7801	1.1170	-1.3868	0.0000	0.0009

metab_8506	neg	321.0623	1.8205	1.7672	3.9905	0.0151	0.0523
metab_8277	neg	321.0755	1.4568	0.8458	-0.7050	0.1382	0.2463
metab_1920	pos	321.1073	2.5003	0.3517	0.3645	0.4757	0.6026
metab_5858	pos	321.1073	1.5144	1.2058	1.8490	0.0142	0.0455
metab_8599	neg	321.1097	2.0202	0.1307	0.1256	0.6950	0.7815
metab_8114	neg	321.1191	1.1796	0.3013	-0.0605	0.3944	0.5226
metab_13960	neg	321.1194	1.9280	1.0573	1.5201	0.0022	0.0141
metab_12963	neg	321.1249	4.3380	1.4472	-0.3445	0.0982	0.1916
metab_13348	neg	321.1249	3.1797	1.1184	-1.5856	0.0451	0.1097
metab_160	pos	321.1284	0.8780	0.4020	1.3145	0.4183	0.5501
metab_337	pos	321.1434	1.5284	0.0900	0.0082	0.8078	0.8730
metab_13623	neg	321.1459	2.5592	0.0295	1.1201	0.9139	0.9428
metab_8549	neg	321.1461	1.9280	0.4525	-0.5594	0.4342	0.5582
metab_6714	neg	321.1670	0.5286	0.1694	0.6423	0.7685	0.8342
metab_2219	pos	321.1800	3.7599	0.0670	-0.0426	0.9103	0.9427
metab_397	pos	321.1801	1.9609	1.1322	1.3802	0.0006	0.0052
metab_2178	pos	321.2053	3.5923	2.3279	7.8108	0.0001	0.0017
metab_10093	neg	321.2108	7.9612	0.2204	0.1946	0.5330	0.6489
metab_961	pos	321.2415	8.4504	0.6123	-0.3727	0.0890	0.1741
metab_2695	pos	321.2415	7.0816	0.6025	-0.3962	0.0920	0.1787
metab_7101	neg	321.2437	8.7191	1.6684	-2.5993	0.0000	0.0013
metab_12027	neg	321.2438	7.9454	1.5986	-2.4395	0.0001	0.0016
metab_11874	neg	321.2438	8.3687	0.0579	0.2060	0.9108	0.9413
metab_4631	pos	321.2524	5.4662	1.1111	6.4427	0.1061	0.1980
metab_3793	pos	321.3143	9.5438	0.5570	0.4829	0.0280	0.0750
metab_11144	neg	321.8143	13.8818	0.7168	-0.3525	0.0001	0.0020
metab_9724	neg	321.8143	6.3427	0.4495	-0.2617	0.1791	0.2970
metab_12415	neg	321.8143	6.6803	0.5034	-0.2401	0.0936	0.1848
metab_15135	neg	321.8724	0.0197	0.2514	-0.0201	0.2202	0.3444
metab_14777	neg	322.0449	0.6131	1.4253	-2.0704	0.0104	0.0409
metab_1323	pos	322.0536	0.6402	1.2173	-1.8309	0.0001	0.0014
metab_14129	neg	322.0573	1.6642	0.2744	0.6973	0.6476	0.7437
metab_6918	neg	322.0606	1.5012	0.9945	-1.4126	0.0311	0.0848
metab_8056	neg	322.0699	1.0215	0.2110	-0.9117	0.6997	0.7846
metab_8669	neg	322.0937	2.1956	0.2551	-0.2091	0.3672	0.4988
metab_14046	neg	322.0937	1.7885	0.0906	-0.0768	0.7006	0.7854
metab_8397	neg	322.0939	1.6186	0.0107	0.2223	0.9036	0.9360
metab_1115	pos	322.1055	0.8640	1.2343	1.8900	0.0029	0.0151
metab_272	pos	322.1209	1.1024	0.0920	0.9185	0.7522	0.8303
metab_5487	pos	322.1277	2.2209	0.4609	-0.5897	0.1880	0.3037
metab_13877	neg	322.1299	2.0670	0.7994	0.1441	0.2633	0.3932
metab_8855	neg	322.1299	2.6524	0.4320	-0.1897	0.2542	0.3826
metab_8256	neg	322.1412	1.4427	1.7107	3.2025	0.0004	0.0048
metab_5014	pos	322.1642	3.6684	0.9046	-1.1813	0.0796	0.1604
metab_1521	pos	322.1864	1.2446	1.8362	2.8738	0.0443	0.1042
metab_7644	neg	322.1988	0.5286	0.1947	0.2192	0.5739	0.6836
metab_12536	neg	322.2027	6.1804	0.3400	0.4499	0.5773	0.6865
metab_9526	neg	322.2028	5.2100	1.3377	2.4738	0.0005	0.0057

metab_6493	pos	322.2328	0.4999	0.5433	2.0331	0.3787	0.5115
metab_9821	neg	322.2389	6.8565	1.2335	11.1429	0.0252	0.0739
metab_999	pos	322.2731	6.2978	0.4110	-0.0068	0.4728	0.5997
metab_10739	neg	322.8391	13.9796	0.2057	0.0665	0.4925	0.6138
metab_15122	neg	322.8510	0.0197	0.3471	0.0130	0.0636	0.1403
metab_11057	neg	322.8870	14.0438	0.1945	0.0816	0.2085	0.3309
metab_14834	neg	323.0288	0.5991	0.0880	0.2412	0.8511	0.8969
metab_10999	neg	323.0290	14.0927	0.1295	0.2489	0.4250	0.5507
metab_1409	pos	323.0378	0.9200	0.5758	-1.7705	0.5408	0.6609
metab_12302	neg	323.0600	7.0322	1.1336	-1.9518	0.0980	0.1912
metab_6813	neg	323.0623	0.9933	0.3590	0.4048	0.3620	0.4947
metab_1289	pos	323.0729	0.5843	1.3733	-2.1733	0.0005	0.0048
metab_13211	neg	323.0985	3.5641	0.2688	0.0145	0.3076	0.4388
metab_7177	neg	323.0986	15.6962	0.1732	0.1177	0.3459	0.4790
metab_7770	neg	323.0986	0.2781	0.0341	0.2135	0.8036	0.8596
metab_10906	neg	323.0986	15.4478	0.0980	0.1238	0.6185	0.7204
metab_12105	neg	323.0988	7.6951	0.5724	-0.5999	0.1381	0.2462
metab_13381	neg	323.0988	3.0774	0.2886	0.0730	0.1588	0.2728
metab_11068	neg	323.0988	14.0282	0.2083	0.0285	0.2792	0.4099
metab_13456	neg	323.1173	2.9092	2.0996	5.1893	0.0001	0.0020
metab_13279	neg	323.1174	3.3465	0.4306	1.1688	0.3954	0.5234
metab_8503	neg	323.1177	1.8205	0.5245	1.6979	0.3666	0.4985
metab_13931	neg	323.1252	1.9904	0.0429	0.3724	0.9536	0.9697
metab_8403	neg	323.1255	1.6335	0.1296	0.2171	0.6870	0.7754
metab_7453	neg	323.1350	1.5297	0.4434	1.4200	0.3723	0.5036
metab_4869	pos	323.1381	4.3371	1.2421	-0.9199	0.0733	0.1510
metab_5152	pos	323.1383	3.1799	1.0408	-1.6196	0.0688	0.1443
metab_5788	pos	323.1593	1.6413	0.4723	-1.2826	0.4145	0.5464
metab_609	pos	323.1842	4.6851	0.3079	0.8387	0.4980	0.6211
metab_6955	neg	323.1868	4.3882	1.4740	3.6014	0.0086	0.0358
metab_12731	neg	323.1868	5.2741	0.7402	0.8214	0.0680	0.1467
metab_9601	neg	323.1869	5.6452	0.9353	1.3894	0.0200	0.0630
metab_12530	neg	323.1870	6.2129	0.4567	0.1618	0.4113	0.5384
metab_494	pos	323.1955	2.9652	1.6405	2.7350	0.0055	0.0235
metab_5297	pos	323.1955	2.7029	1.7894	3.7818	0.0002	0.0028
metab_419	pos	323.1956	2.1595	0.9191	0.9816	0.0119	0.0406
metab_9946	neg	323.2230	7.3659	0.2141	0.0649	0.6071	0.7108
metab_12581	neg	323.2231	5.9705	0.9163	2.3558	0.0942	0.1856
metab_9629	neg	323.2232	5.7751	1.1106	2.7910	0.0457	0.1106
metab_2892	pos	323.2480	8.4504	0.6680	-0.4348	0.0792	0.1597
metab_794	pos	323.2569	8.6577	0.6382	-0.6892	0.0505	0.1149
metab_4305	pos	323.2572	7.1701	0.8641	1.3246	0.0474	0.1097
metab_10007	neg	323.2594	7.6323	1.1419	-1.2689	0.0682	0.1472
metab_2525	pos	323.2684	5.8148	1.2638	7.9323	0.0661	0.1400
metab_14240	neg	323.9576	1.5297	1.7616	-2.9406	0.0001	0.0018
metab_9162	neg	323.9628	3.5978	1.8679	-3.6939	0.0003	0.0043
metab_8203	neg	324.0731	1.3231	0.2868	0.1338	0.3836	0.5130
metab_6472	pos	324.0881	0.5140	0.9045	-1.4099	0.0325	0.0833

metab_7573	neg	324.1094	2.2437	0.5649	-0.5210	0.0892	0.1788
metab_5976	pos	324.1180	1.3013	1.2710	4.2680	0.0154	0.0484
metab_532	pos	324.1284	2.2824	0.2657	0.0658	0.6228	0.7295
metab_8062	neg	324.1301	1.0215	0.8593	0.6214	0.2731	0.4038
metab_5314	pos	324.1430	2.6402	0.7052	-1.0844	0.0512	0.1161
metab_1070	pos	324.1543	1.3579	1.4561	2.4784	0.0008	0.0067
metab_14018	neg	324.1567	1.8205	0.2188	0.2801	0.8114	0.8658
metab_266	pos	324.1641	1.0461	0.9971	-2.5149	0.0821	0.1640
metab_1162	pos	324.2120	0.5140	0.4024	-0.3657	0.1407	0.2444
metab_4870	pos	324.2161	4.3216	1.8609	3.3817	0.0000	0.0007
metab_6980	neg	324.2186	5.0318	0.3716	0.7565	0.4676	0.5901
metab_4848	pos	324.2524	4.3818	1.0916	11.4636	0.0039	0.0184
metab_11998	neg	324.2547	8.0069	1.6580	-1.9107	0.0010	0.0088
metab_2634	pos	324.2886	6.8280	0.3820	-0.8232	0.5499	0.6680
metab_2602	pos	324.2887	6.5246	0.3514	1.3508	0.4715	0.5984
metab_4352	pos	324.2887	6.9935	0.8140	-0.8726	0.0743	0.1522
metab_105	pos	324.2887	8.0267	1.0967	-1.4300	0.0103	0.0366
metab_7698	neg	324.8462	0.0197	0.1056	0.0113	0.6123	0.7154
metab_11054	neg	324.8825	14.0438	0.2592	0.0095	0.1080	0.2056
metab_7637	neg	324.9812	2.7155	1.1104	-0.3579	0.0263	0.0759
metab_6798	neg	324.9968	0.8803	1.0466	1.9094	0.0478	0.1140
metab_7654	neg	325.0682	2.8440	0.1251	0.1085	0.6675	0.7603
metab_13729	neg	325.0934	2.3533	1.6159	5.9290	0.0671	0.1455
metab_8158	neg	325.0935	1.2663	0.3685	0.7182	0.4877	0.6095
metab_14642	neg	325.1074	0.9368	0.4125	-1.0683	0.3995	0.5275
metab_1826	pos	325.1174	2.1746	0.9566	1.2079	0.0117	0.0400
metab_4929	pos	325.1176	4.0338	1.6264	12.5126	0.0178	0.0535
metab_9315	neg	325.1201	4.1535	0.2242	-0.8937	0.8432	0.8911
metab_5692	pos	325.1309	1.8134	0.6611	0.4008	0.0226	0.0636
metab_1093	pos	325.1384	1.2016	1.0190	1.0946	0.0004	0.0040
metab_1554	pos	325.1386	1.3720	1.0647	1.1373	0.0023	0.0129
metab_14251	neg	325.1408	1.5297	0.0763	0.2136	0.8140	0.8677
metab_13792	neg	325.1408	2.2272	0.5275	0.5032	0.2552	0.3836
metab_14540	neg	325.1408	1.1796	1.9779	2.8719	0.0188	0.0604
metab_8459	neg	325.1408	1.7419	0.5728	0.5366	0.1908	0.3106
metab_5782	pos	325.1654	1.6413	0.9032	-0.1237	0.2360	0.3598
metab_1590	pos	325.1748	1.4714	1.7985	3.6351	0.0000	0.0006
metab_5576	pos	325.1748	2.0677	1.0329	1.0002	0.0105	0.0372
metab_1077	pos	325.1749	1.3293	0.3081	0.3619	0.3393	0.4735
metab_8680	neg	325.1772	2.2119	1.1663	5.1808	0.0293	0.0816
metab_9937	neg	325.1845	7.3343	0.3672	0.1155	0.2535	0.3819
metab_7314	neg	325.1847	8.7031	0.8529	-0.5334	0.0001	0.0019
metab_2333	pos	325.1989	4.4730	1.3165	1.7084	0.0079	0.0304
metab_4713	pos	325.1994	4.9856	0.1962	0.1304	0.7390	0.8208
metab_5099	pos	325.2000	3.3337	1.9775	3.9873	0.0001	0.0012
metab_4948	pos	325.2000	3.9421	1.7546	2.9766	0.0002	0.0031
metab_12661	neg	325.2025	5.6615	0.4206	0.6492	0.2053	0.3273
metab_7716	neg	325.2025	3.4800	0.5512	1.0557	0.2235	0.3477

metab_12233	neg	325.2386	7.2377	0.1319	-0.2586	0.8813	0.9192
metab_9990	neg	325.2386	7.5702	1.0247	-1.8261	0.0148	0.0515
metab_10168	neg	325.2387	8.2253	1.1150	-1.7369	0.0294	0.0816
metab_9612	neg	325.2388	5.6936	0.6398	4.7423	0.4070	0.5344
metab_2262	pos	325.2477	4.0338	1.9447	3.9284	0.0004	0.0044
metab_2790	pos	325.2727	7.7800	0.3206	1.7626	0.2970	0.4277
metab_632	pos	325.6992	5.1076	0.7062	0.6566	0.0239	0.0666
metab_7704	neg	325.8546	0.0197	0.1958	0.0076	0.3422	0.4753
metab_13722	neg	325.8791	2.3533	1.1029	-3.3781	0.0269	0.0772
metab_11063	neg	325.9066	14.0282	0.3164	-0.0513	0.1204	0.2219
metab_6436	pos	326.0843	0.5280	0.2789	0.2697	0.5889	0.7011
metab_7445	neg	326.0888	1.5297	0.4263	-0.3559	0.1563	0.2697
metab_8862	neg	326.1041	2.6678	0.2710	0.8781	0.5746	0.6843
metab_14789	neg	326.1094	0.6131	0.2879	1.7429	0.6954	0.7815
metab_6287	pos	326.1154	0.6962	1.0244	-1.7442	0.0245	0.0677
metab_9323	neg	326.1184	4.1869	1.0311	3.5552	0.1163	0.2162
metab_5761	pos	326.1223	1.6838	0.2205	-0.1761	0.5924	0.7040
metab_14493	neg	326.1250	1.2663	1.4367	-2.1175	0.0116	0.0436
metab_217	pos	326.1434	0.6402	2.1239	-4.1248	0.0046	0.0206
metab_5253	pos	326.1586	2.8411	0.0849	-0.5757	0.8487	0.9024
metab_13531	neg	326.1615	2.7475	0.1171	1.0218	0.9605	0.9732
metab_13907	neg	326.1724	2.0202	0.0557	1.5875	0.9879	0.9910
metab_14050	neg	326.1727	1.7729	0.4112	1.0258	0.1132	0.2124
metab_9281	neg	326.1981	4.0347	0.6587	2.0243	0.3083	0.4391
metab_12855	neg	326.1989	4.7546	1.2239	2.0812	0.0448	0.1091
metab_5890	pos	326.2064	1.4569	1.4063	2.4424	0.0020	0.0120
metab_4610	pos	326.2316	5.6168	0.0379	0.6783	0.9901	0.9935
metab_9780	neg	326.2343	6.6320	0.3352	3.5665	0.4911	0.6125
metab_11815	neg	326.2705	8.5604	1.2959	-1.4247	0.0027	0.0160
metab_2904	pos	326.3042	8.5241	0.0388	0.0171	0.9651	0.9788
metab_3029	pos	326.3409	9.2658	1.4620	-2.1815	0.0103	0.0367
metab_15132	neg	326.8303	0.0197	0.4062	-0.1065	0.0293	0.0816
metab_14969	neg	326.9018	0.5286	1.2650	-1.7115	0.0112	0.0427
metab_6599	neg	327.0936	0.5991	0.5250	-0.5006	0.1232	0.2255
metab_13251	neg	327.0992	3.4470	0.4550	-0.0652	0.4000	0.5280
metab_7536	neg	327.0997	2.0055	0.3389	-0.2282	0.4138	0.5402
metab_6082	pos	327.1176	1.1447	0.0223	-0.2573	0.9894	0.9930
metab_13513	neg	327.1201	2.7792	1.3684	-2.0334	0.1647	0.2799
metab_8357	neg	327.1202	1.5586	0.0722	-0.0590	0.9209	0.9483
metab_5157	pos	327.1331	3.1495	0.8488	-1.2537	0.2007	0.3192
metab_449	pos	327.1333	2.3908	0.2282	-0.3820	0.3598	0.4936
metab_5569	pos	327.1333	2.0828	0.8306	-1.1216	0.0829	0.1651
metab_14068	neg	327.1356	1.7572	0.4543	-0.2725	0.1350	0.2420
metab_13888	neg	327.1357	2.0516	0.4544	-0.2690	0.1987	0.3194
metab_6729	neg	327.1411	0.5571	0.4029	1.3493	0.4457	0.5693
metab_8951	neg	327.1455	2.8922	0.0631	0.2732	0.9505	0.9678
metab_326	pos	327.1539	1.4569	0.9801	1.2287	0.0216	0.0618
metab_1689	pos	327.1540	1.7271	0.7649	0.4520	0.1523	0.2590

metab_8780	neg	327.1563	2.4487	0.0963	-0.0586	0.7328	0.8074
metab_13367	neg	327.1565	3.1286	0.0899	1.0852	0.8385	0.8872
metab_7860	neg	327.1776	0.5431	0.9111	-1.2072	0.0394	0.0998
metab_13221	neg	327.1818	3.5475	0.2939	0.9558	0.6056	0.7095
metab_13321	neg	327.1818	3.2473	0.5819	1.2581	0.2081	0.3305
metab_1833	pos	327.1908	2.2045	0.9013	1.7000	0.0341	0.0860
metab_1656	pos	327.2001	1.6413	1.1380	2.2670	0.0245	0.0677
metab_9812	neg	327.2003	6.8093	1.1345	-1.2373	0.0143	0.0504
metab_3353	pos	327.2003	14.5016	0.3209	-0.2496	0.0280	0.0750
metab_12531	neg	327.2179	6.2129	0.3760	0.3872	0.3840	0.5131
metab_12702	neg	327.2181	5.4684	0.3348	0.3836	0.3067	0.4378
metab_12764	neg	327.2182	5.1296	0.4091	0.4240	0.2793	0.4099
metab_2552	pos	327.2519	6.0252	0.8516	2.9144	0.1503	0.2566
metab_12417	neg	327.2544	6.6632	0.7494	-0.9460	0.1138	0.2129
metab_9662	neg	327.2544	5.9866	1.2084	-1.5843	0.0155	0.0532
metab_11548	neg	327.2545	9.1522	0.5108	0.4072	0.2955	0.4260
metab_11440	neg	327.2908	9.5478	0.1470	0.1874	0.5881	0.6956
metab_13569	neg	328.0133	2.6678	0.1537	0.4207	0.7373	0.8105
metab_13498	neg	328.0134	2.8123	0.1160	0.6601	0.9121	0.9418
metab_7598	neg	328.0623	2.4175	0.3187	-0.2804	0.5120	0.6310
metab_8444	neg	328.0678	1.7104	1.4490	2.7651	0.0013	0.0101
metab_240	pos	328.0895	0.7941	1.6993	3.3920	0.0006	0.0057
metab_1209	pos	328.1195	0.4855	0.6619	0.8349	0.0422	0.1006
metab_13304	neg	328.1195	3.2972	1.5712	-2.2629	0.0000	0.0002
metab_6860	neg	328.1304	1.2663	0.1373	0.0499	0.5401	0.6543
metab_14214	neg	328.1516	1.5586	0.0391	0.7361	0.7985	0.8556
metab_14388	neg	328.1517	1.3817	0.6902	-0.6480	0.1983	0.3189
metab_1736	pos	328.1678	1.9309	0.0296	-0.3659	0.9864	0.9917
metab_8825	neg	328.1881	2.5748	0.9473	3.2688	0.2070	0.3291
metab_9748	neg	328.1928	6.4236	0.3899	0.1308	0.4173	0.5435
metab_4930	pos	328.2110	4.0338	2.1954	5.0954	0.0002	0.0023
metab_2022	pos	328.2112	2.9342	1.7536	3.2042	0.0022	0.0126
metab_5930	pos	328.2219	1.3865	0.4597	1.0723	0.4804	0.6066
metab_5533	pos	328.2221	2.1444	0.0996	-0.1720	0.8352	0.8915
metab_13732	neg	328.2246	2.3380	0.5690	0.7799	0.3006	0.4309
metab_6445	pos	328.2446	0.5280	0.4305	-0.3034	0.1876	0.3031
metab_2186	pos	328.2472	3.6229	1.5049	3.3989	0.0001	0.0017
metab_4946	pos	328.2475	3.9421	1.7827	3.8585	0.0000	0.0002
metab_10084	neg	328.2497	7.9292	0.1974	0.1288	0.5100	0.6293
metab_12868	neg	328.2498	4.6883	1.8705	4.6586	0.0001	0.0020
metab_2826	pos	328.2836	8.1132	0.9073	2.2538	0.1327	0.2337
metab_4011	pos	328.2837	8.5695	1.1229	-1.5318	0.0127	0.0423
metab_693	pos	328.2837	6.5395	0.5010	0.5776	0.2601	0.3877
metab_2991	pos	328.3200	9.0397	0.3535	0.4829	0.5339	0.6546
metab_4167	pos	328.3200	7.9695	0.8303	-1.2211	0.1426	0.2468
metab_6644	neg	328.9041	14.0282	0.4018	-0.0524	0.0128	0.0466
metab_11136	neg	328.9310	13.9143	0.2534	0.0510	0.1568	0.2702
metab_11155	neg	328.9310	13.0951	0.0958	0.1630	0.7233	0.8015

metab_14968	neg	329.0136	0.5286	0.7672	-0.0585	0.1379	0.2460
metab_12739	neg	329.0737	5.2419	0.2330	-0.1294	0.7044	0.7882
metab_12805	neg	329.0744	4.9349	0.2690	-0.4249	0.6458	0.7421
metab_8076	neg	329.0817	1.0642	0.3068	0.6818	0.5239	0.6414
metab_14889	neg	329.0872	0.5571	0.9289	2.0623	0.1133	0.2126
metab_5847	pos	329.1120	1.5284	0.8158	-0.9736	0.1727	0.2849
metab_538	pos	329.1123	3.4702	1.0492	-2.4163	0.0325	0.0833
metab_5604	pos	329.1124	2.0211	0.3099	-0.4080	0.5493	0.6676
metab_8543	neg	329.1151	1.9125	0.3643	-0.2035	0.2632	0.3932
metab_13835	neg	329.1152	2.1475	0.7587	-0.6453	0.1492	0.2602
metab_13421	neg	329.1152	2.9931	0.3970	-0.3419	0.3082	0.4390
metab_8384	neg	329.1255	1.5885	1.1876	2.0908	0.0023	0.0143
metab_5726	pos	329.1485	1.7554	0.7613	-1.1055	0.0718	0.1488
metab_5588	pos	329.1489	2.0524	0.4863	-0.5282	0.1219	0.2196
metab_13047	neg	329.1512	4.0516	1.4913	-2.9887	0.0000	0.0005
metab_13418	neg	329.1614	3.0101	1.0440	2.2413	0.0356	0.0927
metab_7657	neg	329.1721	2.8768	0.9432	-0.9539	0.0948	0.1867
metab_8362	neg	329.1834	1.5586	1.4565	2.7786	0.0045	0.0227
metab_12985	neg	329.1977	4.2533	1.4115	3.5310	0.0238	0.0710
metab_12263	neg	329.2126	7.1595	0.1995	0.0876	0.7227	0.8011
metab_4882	pos	329.2311	4.2457	1.0780	1.3975	0.0483	0.1110
metab_4590	pos	329.2313	5.7232	0.2069	-0.6467	0.7458	0.8262
metab_12809	neg	329.2338	4.9349	0.1359	-0.0948	0.7925	0.8513
metab_2413	pos	329.2420	5.0009	1.6224	4.5404	0.0369	0.0908
metab_9546	neg	329.3929	5.3228	0.2397	-0.1734	0.6811	0.7707
metab_9479	neg	329.3940	4.9183	0.2881	-0.2880	0.6010	0.7057
metab_14036	neg	329.9687	1.8046	2.1585	-3.8977	0.0000	0.0011
metab_8491	neg	330.0052	1.7885	1.1972	-2.3955	0.0006	0.0064
metab_8537	neg	330.0053	1.8833	2.0253	-11.4104	0.0000	0.0000
metab_6269	pos	330.0575	0.7801	1.1543	-1.8348	0.0086	0.0323
metab_13613	neg	330.0988	2.5748	0.8812	-1.4457	0.1296	0.2345
metab_8824	neg	330.1095	2.5592	0.3831	0.8497	0.4759	0.5979
metab_2117	pos	330.1325	3.3032	1.4505	-2.2901	0.0001	0.0012
metab_9069	neg	330.1352	3.2801	1.0105	-0.8714	0.1183	0.2188
metab_5985	pos	330.1435	1.2725	1.2943	-2.0079	0.0079	0.0305
metab_15028	neg	330.1524	0.4975	0.0147	0.2000	0.9592	0.9727
metab_13957	neg	330.1674	1.9439	0.2042	1.4392	0.7125	0.7941
metab_5073	pos	330.1691	3.4399	0.2371	4.7100	0.8901	0.9300
metab_6530	pos	330.1867	0.4855	0.8460	-0.9323	0.1125	0.2063
metab_5178	pos	330.1902	3.0726	0.4165	0.8087	0.4285	0.5600
metab_1935	pos	330.2010	2.5782	1.2042	2.4808	0.0281	0.0752
metab_13584	neg	330.2039	2.6357	1.5399	3.4240	0.0003	0.0037
metab_4457	pos	330.2055	6.4192	0.0391	0.4961	0.9914	0.9944
metab_6516	pos	330.2071	0.4999	1.4102	2.6677	0.0117	0.0401
metab_587	pos	330.2266	4.3371	1.0030	1.1664	0.0002	0.0023
metab_2202	pos	330.2266	3.6987	1.6308	3.2481	0.0001	0.0017
metab_5246	pos	330.2268	2.8725	1.9846	4.8193	0.0000	0.0006
metab_5449	pos	330.2375	2.3133	0.6759	0.6114	0.1603	0.2690

metab_2086	pos	330.2375	3.1957	2.7656	8.1940	0.0000	0.0005
metab_2452	pos	330.2628	5.3014	0.5085	-0.5885	0.4378	0.5686
metab_4553	pos	330.2629	5.9361	0.5839	-0.7404	0.3735	0.5073
metab_4866	pos	330.2630	4.3371	0.9732	1.3253	0.0122	0.0411
metab_2843	pos	330.2992	8.2143	0.6526	-1.0854	0.1348	0.2367
metab_4417	pos	330.2993	6.7062	0.0780	0.1181	0.9132	0.9434
metab_2832	pos	330.3357	8.1414	1.6338	3.4762	0.0004	0.0043
metab_7201	neg	330.8995	14.0282	0.3719	-0.0481	0.0296	0.0819
metab_8837	neg	331.0286	2.6046	0.1966	0.0000	0.2936	0.4247
metab_8974	neg	331.0288	2.9760	0.9811	1.1619	0.0299	0.0824
metab_14336	neg	331.0399	1.4568	1.8732	-4.5379	0.0017	0.0116
metab_7571	neg	331.0940	2.2272	0.3823	-0.3933	0.4308	0.5556
metab_6045	pos	331.1126	1.1875	1.4921	2.6463	0.0002	0.0026
metab_5625	pos	331.1279	1.9918	0.6210	-0.8879	0.1441	0.2487
metab_13461	neg	331.1308	2.8922	0.2079	0.1256	0.5372	0.6521
metab_8684	neg	331.1310	2.2272	0.6997	-0.1255	0.2984	0.4289
metab_8976	neg	331.1397	2.9760	0.1001	0.3565	0.7234	0.8016
metab_14039	neg	331.1415	1.8046	0.8448	1.6239	0.0835	0.1703
metab_14389	neg	331.1417	1.3817	0.7975	1.8049	0.1331	0.2392
metab_8841	neg	331.1512	2.6046	0.0308	1.4342	0.9064	0.9379
metab_13868	neg	331.1514	2.0829	0.4166	0.0282	0.3307	0.4636
metab_9408	neg	331.1556	4.6056	3.0924	15.5315	0.0021	0.0135
metab_13049	neg	331.1557	4.0516	0.5322	1.5778	0.3269	0.4590
metab_13110	neg	331.1558	3.8333	0.3262	4.3139	0.8617	0.9048
metab_1839	pos	331.1645	2.2209	0.2450	-0.0480	0.5356	0.6562
metab_5254	pos	331.1855	2.8411	1.4083	-2.2083	0.0057	0.0242
metab_4431	pos	331.1871	6.5857	1.3307	1.9108	0.0018	0.0109
metab_13572	neg	331.1877	2.6678	0.2070	-0.0953	0.4578	0.5809
metab_7652	neg	331.1878	2.7961	0.0105	0.0455	0.9699	0.9787
metab_4748	pos	331.1895	4.8961	1.4811	6.9024	0.0856	0.1691
metab_4846	pos	331.1896	4.3972	1.7172	10.6420	0.0612	0.1319
metab_9527	neg	331.1920	5.2100	1.4063	5.6929	0.1081	0.2058
metab_1632	pos	331.1965	1.5705	1.7296	5.2095	0.0003	0.0033
metab_10113	neg	331.2283	8.0228	0.0801	0.0872	0.9132	0.9426
metab_12811	neg	331.2397	4.9349	0.2038	-0.1928	0.7075	0.7906
metab_2514	pos	331.2470	5.7385	1.3440	1.9749	0.0189	0.0560
metab_2447	pos	331.2470	5.2573	1.2319	1.6106	0.0298	0.0785
metab_12740	neg	331.2492	5.2419	0.4287	0.9647	0.2679	0.3982
metab_14545	neg	331.9481	1.1645	0.1518	-0.5451	0.7132	0.7942
metab_7802	neg	331.9600	0.4975	0.1924	0.2653	0.6214	0.7224
metab_14151	neg	332.0083	1.6335	0.4023	-0.1731	0.4278	0.5530
metab_8271	neg	332.0595	1.4568	0.6280	-0.4314	0.3063	0.4374
metab_13889	neg	332.0981	2.0358	0.5485	-1.4431	0.2559	0.3846
metab_14685	neg	332.1103	0.8522	1.2643	-1.6368	0.1351	0.2420
metab_8616	neg	332.1146	2.0516	0.5436	-0.4879	0.2772	0.4079
metab_13933	neg	332.1256	1.9904	0.4409	-0.1524	0.1756	0.2926
metab_13753	neg	332.1257	2.3078	0.7615	-0.8034	0.1787	0.2966
metab_1993	pos	332.1272	2.8259	1.2192	-4.4696	0.1324	0.2333

metab_8052	neg	332.1466	1.0074	0.8559	-1.6414	0.2273	0.3523
metab_2110	pos	332.1484	3.2727	1.1597	-1.5521	0.0472	0.1093
metab_5239	pos	332.1484	2.8877	1.1229	-2.1715	0.0307	0.0799
metab_1739	pos	332.1805	1.9463	0.4083	1.4483	0.4607	0.5888
metab_5401	pos	332.2058	2.4224	1.1221	1.8345	0.0193	0.0568
metab_5031	pos	332.2533	3.5923	0.3936	0.6057	0.6143	0.7227
metab_4191	pos	332.2574	7.8097	1.6457	5.8768	0.0002	0.0023
metab_4467	pos	332.2786	6.4035	0.0284	0.1402	0.9791	0.9882
metab_14966	neg	332.8677	0.5286	1.4617	-1.9660	0.0059	0.0273
metab_3283	pos	332.9151	14.0525	0.4330	1.1228	0.2140	0.3351
metab_13486	neg	333.0444	2.8440	0.6150	3.2447	0.3829	0.5125
metab_8944	neg	333.0553	2.8768	1.1951	11.7777	0.1981	0.3187
metab_14982	neg	333.0597	0.5286	0.3437	-0.0863	0.0998	0.1937
metab_6307	pos	333.0684	0.6402	1.3862	-2.3878	0.0342	0.0863
metab_14523	neg	333.0833	1.2236	0.6568	-0.6417	0.0538	0.1238
metab_14715	neg	333.0943	0.7818	1.1990	-1.8027	0.0248	0.0731
metab_6423	pos	333.1047	0.5420	0.8613	-0.7100	0.1739	0.2865
metab_5500	pos	333.1072	2.2045	0.3986	-0.6821	0.3767	0.5100
metab_13704	neg	333.1097	2.4010	0.1730	0.0812	0.5107	0.6296
metab_8103	neg	333.1242	1.1503	0.8772	2.2335	0.0524	0.1216
metab_12895	neg	333.1252	4.6056	0.5079	2.3049	0.5421	0.6560
metab_1047	pos	333.1282	1.5847	0.3700	0.5624	0.3252	0.4591
metab_14277	neg	333.1306	1.5155	0.2295	-0.0365	0.4518	0.5751
metab_8083	neg	333.1307	1.0785	0.1771	0.8965	0.7818	0.8445
metab_12905	neg	333.1382	4.5884	1.0978	1.8388	0.0162	0.0549
metab_13299	neg	333.1382	3.2972	1.1842	-1.7805	0.0096	0.0388
metab_7713	neg	333.1383	3.4470	0.8811	-1.1670	0.0227	0.0687
metab_339	pos	333.1437	1.5144	0.5478	-0.6905	0.1260	0.2248
metab_13593	neg	333.1461	2.6206	1.7793	-1.6410	0.0101	0.0400
metab_5697	pos	333.1545	1.7993	1.4660	2.6499	0.0278	0.0746
metab_13150	neg	333.1559	3.7317	1.9785	4.9650	0.0003	0.0043
metab_13098	neg	333.1715	3.8678	1.6336	7.3912	0.0495	0.1167
metab_9406	neg	333.1825	4.5884	1.3418	6.7578	0.0581	0.1312
metab_13030	neg	333.1825	4.1023	0.1610	0.3068	0.6708	0.7623
metab_13094	neg	333.1826	3.8839	1.3134	5.3518	0.0195	0.0618
metab_489	pos	333.2010	2.6869	0.2415	-0.3626	0.3525	0.4876
metab_9875	neg	333.2022	7.0322	1.1494	-1.8078	0.1153	0.2147
metab_4512	pos	333.2029	6.1167	0.0058	0.0941	0.9673	0.9801
metab_5227	pos	333.2161	2.9192	2.2292	6.4297	0.0000	0.0008
metab_5811	pos	333.2273	1.5987	1.6043	-2.8204	0.0001	0.0018
metab_7331	neg	333.2292	8.5299	0.2701	-0.0091	0.2377	0.3636
metab_4148	pos	333.2408	8.0419	0.1408	0.2630	0.8114	0.8753
metab_2268	pos	333.2415	4.0338	1.5485	6.1935	0.0049	0.0216
metab_11921	neg	333.2440	8.2099	1.2210	-2.0573	0.0095	0.0386
metab_9531	neg	333.2549	5.2419	0.5646	1.5703	0.2140	0.3369
metab_2508	pos	333.2625	5.6934	0.8799	4.3990	0.1549	0.2622
metab_9786	neg	333.9261	6.6632	0.4965	-0.2743	0.0545	0.1250
metab_14432	neg	334.0339	1.3089	1.2838	3.9063	0.0267	0.0768

metab_8275	neg	334.0565	1.4568	0.4204	-0.4052	0.4181	0.5443
metab_8907	neg	334.0937	2.7792	0.8398	-2.0772	0.0683	0.1472
metab_13627	neg	334.1132	2.5431	0.6846	-0.7415	0.1680	0.2843
metab_5595	pos	334.1276	2.0360	0.2922	-1.5697	0.6575	0.7565
metab_401	pos	334.1385	1.9918	0.0500	0.0947	0.7280	0.8123
metab_8569	neg	334.1415	1.9745	0.7467	1.5616	0.0840	0.1709
metab_4880	pos	334.1427	4.2609	1.2539	-1.7524	0.0086	0.0324
metab_5030	pos	334.1541	3.5923	0.2810	-0.6146	0.5792	0.6923
metab_8950	neg	334.1625	2.8922	0.3185	0.3998	0.6116	0.7149
metab_13032	neg	334.1665	4.1023	0.0906	0.0280	0.7718	0.8370
metab_5829	pos	334.1747	1.5565	1.1330	12.5176	0.0140	0.0452
metab_9783	neg	334.1813	6.6480	0.5309	-2.3479	0.3708	0.5021
metab_5222	pos	334.1906	2.9342	0.9357	-1.3219	0.0487	0.1119
metab_9544	neg	334.2025	5.3228	0.2933	2.0806	0.7222	0.8007
metab_5583	pos	334.2212	2.0524	1.5478	6.7422	0.0026	0.0142
metab_4304	pos	334.2368	7.1701	1.1367	2.8873	0.0119	0.0406
metab_5047	pos	334.2579	3.5311	0.0681	-0.0133	0.9035	0.9383
metab_5177	pos	334.2580	3.0726	0.0211	1.1768	0.9739	0.9846
metab_2596	pos	334.2732	6.4639	0.7036	1.7482	0.3327	0.4675
metab_15112	neg	334.8436	0.0197	0.1502	-0.0252	0.4862	0.6080
metab_7728	neg	334.8639	0.0197	0.3208	-0.0433	0.0992	0.1926
metab_2690	pos	335.0729	7.0526	0.3467	-0.4241	0.2539	0.3800
metab_4356	pos	335.0732	6.9638	0.5295	-0.8717	0.1818	0.2959
metab_6720	neg	335.0753	0.5431	0.4233	0.8433	0.4056	0.5336
metab_8586	neg	335.0888	1.9904	1.6477	2.3999	0.0443	0.1082
metab_8479	neg	335.0892	1.7729	0.9475	1.5768	0.0289	0.0809
metab_8381	neg	335.0896	1.5885	0.4884	-0.1378	0.2977	0.4282
metab_13897	neg	335.1176	2.0358	1.2492	2.4075	0.0049	0.0242
metab_14383	neg	335.1182	1.3817	0.0100	0.4355	0.9445	0.9637
metab_451	pos	335.1228	2.4071	0.8157	-1.0640	0.0213	0.0610
metab_8710	neg	335.1254	2.2912	0.5068	-0.2570	0.1583	0.2721
metab_8850	neg	335.1255	2.6357	0.7561	-0.7811	0.0418	0.1042
metab_14273	neg	335.1262	1.5155	0.4230	0.2490	0.3258	0.4580
metab_6081	pos	335.1371	1.1447	0.6222	3.1696	0.5307	0.6515
metab_4800	pos	335.1385	4.6096	0.3249	2.9585	0.6283	0.7335
metab_8630	neg	335.1447	2.0987	0.0964	1.9379	0.8258	0.8773
metab_2680	pos	335.1505	7.0228	1.1337	-2.2779	0.0706	0.1471
metab_4805	pos	335.1514	4.5944	1.3833	2.0599	0.0256	0.0701
metab_12777	neg	335.1539	5.0484	1.3230	3.2504	0.0162	0.0547
metab_1952	pos	335.1593	2.6244	0.9959	-1.2823	0.0032	0.0163
metab_9051	neg	335.1618	3.2130	0.3842	0.1949	0.3931	0.5214
metab_7624	neg	335.1618	2.6206	0.5835	0.9838	0.1953	0.3157
metab_994	pos	335.2187	6.3885	0.3246	-0.5740	0.4424	0.5730
metab_4551	pos	335.2194	5.9361	0.1399	-0.0196	0.7714	0.8455
metab_4767	pos	335.2207	4.7758	2.1854	5.8178	0.0000	0.0000
metab_9876	neg	335.2231	7.0322	0.3275	0.2508	0.4720	0.5944
metab_12411	neg	335.2340	6.6962	0.4489	-1.8432	0.4501	0.5738
metab_809	pos	335.2570	8.9367	1.4232	2.8851	0.0125	0.0418

metab_4544	pos	335.2680	5.9504	0.8426	2.6585	0.0763	0.1550
metab_2495	pos	335.2681	5.6322	0.6729	2.9539	0.1860	0.3011
metab_6475	pos	335.5219	0.5140	1.2942	2.1716	0.0032	0.0161
metab_14246	neg	335.9584	1.5297	1.3388	-1.7995	0.0012	0.0098
metab_13550	neg	336.0187	2.7155	0.9347	-0.3284	0.0300	0.0826
metab_7510	neg	336.0300	1.8524	1.4158	3.1420	0.0069	0.0306
metab_7853	neg	336.0624	0.5286	1.2788	2.0643	0.0004	0.0050
metab_6131	pos	336.0694	1.0321	0.0535	0.1692	0.8800	0.9237
metab_8358	neg	336.1099	1.5586	0.1301	-0.0118	0.6966	0.7822
metab_13927	neg	336.1201	1.9904	0.9192	1.7751	0.2799	0.4106
metab_13815	neg	336.1284	2.1798	0.2767	0.5303	0.5327	0.6487
metab_7881	neg	336.1417	0.5571	0.2062	0.1202	0.3888	0.5176
metab_14004	neg	336.1459	1.8524	0.8664	-1.0062	0.0040	0.0210
metab_1551	pos	336.1541	1.3437	1.6309	3.2628	0.0001	0.0019
metab_399	pos	336.1543	1.9758	0.9340	1.3593	0.0163	0.0502
metab_14145	neg	336.1568	1.6335	1.1369	-1.3440	0.0009	0.0081
metab_1982	pos	336.1656	2.7804	1.4964	-4.1958	0.0140	0.0452
metab_13175	neg	336.1664	3.6645	1.8608	4.6705	0.0244	0.0724
metab_8296	neg	336.1668	1.4710	1.5670	9.3675	0.0089	0.0367
metab_4820	pos	336.1735	4.5335	1.6160	-3.0742	0.0032	0.0162
metab_597	pos	336.1795	4.0939	0.0361	-0.0910	0.9135	0.9434
metab_2120	pos	336.2160	3.3032	2.2277	7.8705	0.0000	0.0000
metab_2521	pos	336.2515	5.7846	1.4394	2.8233	0.0012	0.0084
metab_4480	pos	336.2522	6.3581	0.0360	0.3447	0.9217	0.9493
metab_5079	pos	336.2525	3.4098	1.1162	-1.0118	0.0513	0.1162
metab_12523	neg	336.2545	6.2456	0.0089	0.0491	0.8720	0.9121
metab_11987	neg	336.2549	8.0532	1.1515	-1.2639	0.0003	0.0043
metab_10294	neg	336.2549	8.7031	1.3540	-1.7103	0.0000	0.0013
metab_2840	pos	336.2887	8.1707	0.6058	-0.5039	0.1046	0.1961
metab_3957	pos	336.3251	8.7590	1.8338	-3.3424	0.0001	0.0016
metab_3868	pos	336.3253	9.1898	0.1531	0.1509	0.5655	0.6820
metab_2816	pos	336.3253	7.9980	0.0210	-0.1739	0.9276	0.9533
metab_13411	neg	336.8953	3.0101	0.7180	-2.4389	0.1550	0.2679
metab_1337	pos	337.0522	0.6682	0.5765	0.7759	0.3697	0.5035
metab_14110	neg	337.0572	1.6948	0.8899	4.9029	0.1258	0.2294
metab_228	pos	337.0885	0.6262	0.3538	1.8620	0.6277	0.7333
metab_8638	neg	337.1046	2.1153	0.6531	-0.4466	0.1271	0.2311
metab_8059	neg	337.1048	1.0215	0.3523	1.2881	0.5256	0.6427
metab_14199	neg	337.1048	1.5736	0.3715	0.7892	0.3789	0.5088
metab_8726	neg	337.1202	2.3227	0.5846	-0.3502	0.2119	0.3347
metab_13327	neg	337.1217	3.2301	0.1211	-0.0675	0.7372	0.8105
metab_5798	pos	337.1313	1.6272	0.5118	-0.5196	0.2347	0.3589
metab_13570	neg	337.1330	2.6678	1.5205	5.5975	0.0274	0.0783
metab_8980	neg	337.1330	2.9931	1.7914	5.4318	0.0052	0.0253
metab_8797	neg	337.1333	2.4970	0.8504	3.3767	0.1724	0.2890
metab_1056	pos	337.1384	1.5144	1.1877	2.3325	0.0102	0.0364
metab_1094	pos	337.1386	1.2016	0.2949	-0.2980	0.1995	0.3175
metab_13728	neg	337.1410	2.3533	0.1168	0.5164	0.7290	0.8050

metab_6718	neg	337.1622	0.5431	0.9551	-0.0123	0.1997	0.3203
metab_2938	pos	337.1663	8.7008	1.0681	-1.2980	0.0000	0.0008
metab_6350	pos	337.1706	0.6123	0.4312	-0.2962	0.4486	0.5782
metab_471	pos	337.1748	2.6244	0.3183	0.4350	0.4526	0.5821
metab_512	pos	337.1749	3.1346	0.1855	-0.0301	0.5415	0.6615
metab_12907	neg	337.2027	4.5717	1.9017	3.7852	0.0016	0.0115
metab_9977	neg	337.2058	7.5239	0.2182	0.3246	0.5956	0.7021
metab_5044	pos	337.2112	3.5465	2.0763	3.7026	0.0023	0.0130
metab_2225	pos	337.2151	3.7908	1.6843	7.2745	0.0033	0.0165
metab_2	pos	337.2342	6.4035	0.2448	0.5476	0.4936	0.6180
metab_1908	pos	337.2365	2.4370	1.7229	12.7616	0.0042	0.0193
metab_12453	neg	337.2385	6.5033	0.1053	0.5236	0.7004	0.7852
metab_12280	neg	337.2387	7.1111	0.4627	-0.0975	0.0900	0.1799
metab_12673	neg	337.2388	5.6128	0.5179	0.1130	0.1147	0.2141
metab_9645	neg	337.2388	5.9217	0.2955	0.2085	0.2915	0.4227
metab_12035	neg	337.2389	7.9137	0.7700	-0.3460	0.0197	0.0622
metab_10274	neg	337.2391	8.6239	1.5510	-2.0851	0.0000	0.0004
metab_2668	pos	337.2567	6.9638	0.0407	0.9831	0.9371	0.9611
metab_4242	pos	337.2727	7.5450	0.5750	0.7320	0.1354	0.2377
metab_85	pos	337.2728	6.9336	0.3594	0.4124	0.4235	0.5550
metab_2542	pos	337.2838	5.9504	1.2707	6.1948	0.0574	0.1261
metab_175	pos	337.9683	0.4999	1.0437	-1.6871	0.0201	0.0584
metab_13813	neg	338.0887	2.1956	0.2470	0.1812	0.3663	0.4982
metab_1646	pos	338.1196	1.6272	1.4341	3.1999	0.0023	0.0131
metab_5495	pos	338.1225	2.2045	0.8996	-1.4165	0.0823	0.1641
metab_8455	neg	338.1253	1.7264	0.8357	-1.0890	0.0696	0.1494
metab_65	pos	338.1518	2.4224	0.2298	-0.3262	0.4595	0.5883
metab_1037	pos	338.1521	1.6838	0.7984	0.5784	0.0658	0.1397
metab_1463	pos	338.1521	1.1164	0.1604	-0.0989	0.4725	0.5994
metab_338	pos	338.1697	1.5144	1.2199	1.6804	0.0000	0.0011
metab_1212	pos	338.2275	0.4855	0.2673	-0.4059	0.6356	0.7399
metab_1001	pos	338.2676	6.2522	0.3490	0.4239	0.2905	0.4209
metab_12045	neg	338.2705	7.8822	0.7614	12.0313	0.0823	0.1685
metab_11825	neg	338.2706	8.5299	0.0381	0.5556	0.9290	0.9539
metab_2835	pos	338.3043	8.1414	1.0984	-0.8383	0.0373	0.0916
metab_3985	pos	338.3045	8.6284	0.3458	0.9832	0.5983	0.7086
metab_3256	pos	338.3404	13.4283	0.2189	0.0455	0.2934	0.4239
metab_4057	pos	338.3407	8.3763	0.3880	-0.3301	0.0260	0.0710
metab_844	pos	338.3408	9.5438	0.1523	0.0692	0.3955	0.5282
metab_2964	pos	338.3408	8.8759	0.1187	-0.1639	0.7573	0.8342
metab_6680	neg	338.8593	0.0197	0.3874	-0.0968	0.0435	0.1071
metab_11032	neg	338.8593	14.0610	0.6847	-0.3458	0.0003	0.0038
metab_1152	pos	338.9636	0.5420	0.0014	1.3839	0.9639	0.9781
metab_14649	neg	338.9891	0.9086	0.9334	4.0864	0.1853	0.3039
metab_14937	neg	339.0559	0.5286	1.4238	10.4427	0.0029	0.0168
metab_14216	neg	339.0727	1.5586	1.3442	2.4159	0.0003	0.0040
metab_7611	neg	339.1047	2.5264	1.9238	-3.5333	0.0000	0.0000
metab_5821	pos	339.1180	1.5705	1.2618	2.5130	0.0080	0.0307

metab_13864	neg	339.1200	2.0829	0.5648	0.7757	0.1574	0.2711
metab_7437	neg	339.1202	1.5155	0.1595	0.4725	0.8278	0.8789
metab_5451	pos	339.1334	2.3133	0.7615	-0.9574	0.1074	0.2000
metab_2097	pos	339.1337	3.2274	0.7646	-0.8189	0.2024	0.3214
metab_9209	neg	339.1354	3.7658	1.3342	-2.2425	0.0049	0.0240
metab_13072	neg	339.1356	3.9677	0.5130	1.1275	0.3097	0.4406
metab_13770	neg	339.1563	2.2595	0.6904	0.9106	0.1423	0.2517
metab_8262	neg	339.1564	1.4427	0.6333	1.1930	0.2623	0.3922
metab_2261	pos	339.1794	4.0183	1.3434	3.1597	0.0135	0.0440
metab_5738	pos	339.1904	1.7271	1.4484	2.5160	0.0027	0.0145
metab_405	pos	339.1905	2.0211	0.9206	0.8270	0.0367	0.0906
metab_10027	neg	339.2002	7.7100	0.4999	-0.0237	0.0642	0.1412
metab_7313	neg	339.2003	8.7031	0.7918	-0.4242	0.0005	0.0054
metab_11832	neg	339.2153	8.5135	0.6577	1.0701	0.0355	0.0925
metab_5027	pos	339.2159	3.6073	2.5033	10.3168	0.0000	0.0006
metab_12380	neg	339.2180	6.8255	0.3537	0.9230	0.3986	0.5264
metab_12836	neg	339.2180	4.8210	1.5642	3.9014	0.0030	0.0170
metab_2587	pos	339.2404	6.4035	0.4250	0.8580	0.3335	0.4678
metab_19	pos	339.2503	6.8886	0.7314	1.1384	0.0445	0.1046
metab_2659	pos	339.2793	6.9336	0.2618	0.2988	0.5369	0.6572
metab_2741	pos	339.2883	7.4119	0.5788	0.7404	0.1888	0.3041
metab_4195	pos	339.2883	7.7946	1.0377	1.8166	0.0297	0.0782
metab_4496	pos	339.2883	6.2522	0.0708	0.3681	0.8483	0.9023
metab_4141	pos	339.2884	8.0700	1.1423	1.8712	0.0117	0.0400
metab_4470	pos	339.2884	6.4035	0.1537	0.4231	0.6856	0.7794
metab_11491	neg	339.2908	9.3329	0.4158	-0.3038	0.2656	0.3958
metab_13468	neg	340.0960	2.8768	0.9704	-1.5984	0.0204	0.0638
metab_5511	pos	340.1019	2.1897	0.4437	-0.1179	0.2335	0.3575
metab_6879	neg	340.1045	1.3089	0.9321	-1.2032	0.0191	0.0610
metab_14621	neg	340.1151	0.9649	1.5272	3.0409	0.0039	0.0205
metab_5559	pos	340.1380	2.0973	0.0183	-0.1524	0.9803	0.9882
metab_1508	pos	340.1385	1.2162	0.5803	-1.7865	0.1938	0.3101
metab_7662	neg	340.1405	2.9263	0.4695	-1.3920	0.3409	0.4741
metab_323	pos	340.1858	1.4429	1.7892	4.4340	0.0002	0.0030
metab_13592	neg	340.1880	2.6206	0.4664	-0.0026	0.3238	0.4565
metab_2379	pos	340.2109	4.7302	1.2392	4.5482	0.0078	0.0301
metab_5252	pos	340.2111	2.8411	1.0810	4.0037	0.2412	0.3660
metab_9520	neg	340.2133	5.1610	1.7621	3.7010	0.0024	0.0150
metab_5903	pos	340.2224	1.4429	0.8256	1.5070	0.1035	0.1945
metab_2445	pos	340.2473	5.2431	0.9383	1.9139	0.1104	0.2040
metab_4661	pos	340.2833	5.3014	0.0844	2.1601	0.8078	0.8730
metab_695	pos	340.2837	6.5246	0.4715	0.1384	0.3788	0.5115
metab_4747	pos	340.2837	4.8961	1.2809	12.3358	0.0043	0.0198
metab_7335	neg	340.2861	8.4505	0.7888	2.0598	0.0559	0.1273
metab_2772	pos	340.3201	7.6626	1.6408	-3.5799	0.0000	0.0011
metab_3789	pos	340.3476	9.5594	0.3487	0.2411	0.0942	0.1816
metab_11146	neg	340.9404	13.8651	0.3343	-0.0148	0.0626	0.1387
metab_6759	neg	341.0392	0.6551	0.8693	-2.7337	0.0695	0.1491

metab_9421	neg	341.1356	4.6380	1.6568	-2.9081	0.0003	0.0040
metab_14112	neg	341.1357	1.6948	0.0675	0.1015	0.8134	0.8673
metab_11675	neg	341.1429	8.8128	0.8730	1.1639	0.0020	0.0130
metab_445	pos	341.1490	2.3447	0.1672	-0.2312	0.5547	0.6724
metab_13092	neg	341.1609	3.9005	2.5133	5.5575	0.0000	0.0006
metab_13363	neg	341.1721	3.1286	0.0565	0.1213	0.8858	0.9234
metab_1659	pos	341.1797	1.6553	0.2554	1.6450	0.7464	0.8266
metab_2322	pos	341.1951	4.3972	0.4931	0.2223	0.6399	0.7435
metab_13586	neg	341.1957	2.6357	1.7258	3.6219	0.0028	0.0163
metab_9624	neg	341.1971	5.7420	0.6312	1.2002	0.1099	0.2080
metab_9250	neg	341.1972	3.9005	1.4642	2.4665	0.0014	0.0108
metab_7709	neg	341.1973	3.3803	1.9065	4.1110	0.0004	0.0049
metab_9445	neg	341.1974	4.7546	0.9051	1.1771	0.0118	0.0440
metab_12941	neg	341.1975	4.4055	0.9155	1.2046	0.0171	0.0566
metab_4897	pos	341.2061	4.1698	0.3055	-0.5831	0.7128	0.8004
metab_572	pos	341.2061	3.9577	0.3453	-0.8062	0.6946	0.7866
metab_9727	neg	341.2329	6.3593	0.2634	0.0244	0.3582	0.4911
metab_12457	neg	341.2329	6.4874	0.2481	0.0899	0.3660	0.4980
metab_12780	neg	341.2336	5.0318	1.6374	4.6892	0.0140	0.0495
metab_12227	neg	341.2336	7.2701	0.6404	0.7995	0.1651	0.2806
metab_9169	neg	341.2337	3.6311	2.1801	6.9221	0.0004	0.0044
metab_9384	neg	341.2338	4.5054	1.5124	6.2127	0.0090	0.0372
metab_5135	pos	341.2425	3.2116	1.5283	9.9028	0.0217	0.0618
metab_4380	pos	341.2559	6.8886	1.4212	3.5213	0.0020	0.0120
metab_9866	neg	341.2697	7.0322	1.0368	1.8303	0.0202	0.0634
metab_4138	pos	341.2953	8.0700	1.1446	2.1784	0.0097	0.0353
metab_3149	pos	341.3040	10.0875	0.9248	1.0642	0.1534	0.2603
metab_4250	pos	341.3040	7.5005	0.6085	2.4928	0.2285	0.3520
metab_3625	pos	341.3041	10.3480	0.9967	1.6706	0.0889	0.1740
metab_3117	pos	341.3042	9.8522	0.0876	0.2540	0.8630	0.9125
metab_3031	pos	341.3043	9.2815	1.4376	2.5110	0.0146	0.0465
metab_4387	pos	341.3043	6.8886	0.9124	1.3372	0.0504	0.1147
metab_10544	neg	341.3064	9.7312	0.2288	0.6027	0.6264	0.7262
metab_12751	neg	342.0878	5.1772	0.5188	0.4074	0.2885	0.4201
metab_6905	neg	342.0882	1.4568	0.3912	-0.3334	0.4348	0.5587
metab_8548	neg	342.1199	1.9280	0.9159	-0.8641	0.0021	0.0136
metab_8251	neg	342.1309	1.4267	0.6697	1.2208	0.1738	0.2906
metab_6031	pos	342.1648	1.2016	1.0318	1.4471	0.0119	0.0406
metab_13855	neg	342.1671	2.0987	0.0266	0.3408	0.8440	0.8919
metab_14045	neg	342.1672	1.7885	0.1239	1.0452	0.8971	0.9312
metab_14827	neg	342.1770	0.5991	0.8196	1.0240	0.0819	0.1681
metab_5463	pos	342.1829	2.2665	0.0118	-0.3549	0.8765	0.9214
metab_1962	pos	342.1832	2.6869	0.0886	-0.1949	0.6561	0.7558
metab_5329	pos	342.2013	2.5938	0.5449	-0.4206	0.2387	0.3630
metab_5385	pos	342.2014	2.4530	0.7590	-1.9880	0.3784	0.5113
metab_4373	pos	342.2052	6.9182	0.0085	-0.1307	0.9446	0.9655
metab_4826	pos	342.2266	4.5027	1.3572	2.2342	0.0008	0.0064
metab_5341	pos	342.2377	2.5627	0.4487	0.4185	0.3716	0.5056

metab_5764	pos	342.2379	1.6838	0.5558	0.6532	0.3064	0.4385
metab_8884	neg	342.2401	2.7318	0.7556	0.7156	0.1343	0.2410
metab_13413	neg	342.2402	3.0101	0.9684	1.4583	0.0740	0.1557
metab_11897	neg	342.2653	8.3055	1.1737	-1.3072	0.0053	0.0257
metab_2970	pos	342.2781	8.9212	0.8059	-1.1991	0.1031	0.1939
metab_10227	neg	342.2926	8.4667	0.7674	11.5595	0.1245	0.2274
metab_4170	pos	342.2992	7.9551	0.3292	-0.0681	0.5553	0.6729
metab_4613	pos	342.2994	5.5864	0.0583	1.2908	0.8683	0.9157
metab_4641	pos	342.2994	5.3913	0.8050	2.5911	0.2198	0.3423
metab_782	pos	342.2994	8.4504	1.3957	3.1525	0.0104	0.0369
metab_4027	pos	342.3357	8.4949	1.4617	-2.4306	0.0023	0.0131
metab_15123	neg	342.8154	0.0197	0.5762	-0.3421	0.0081	0.0342
metab_11055	neg	342.8552	14.0438	0.2760	0.0217	0.1176	0.2178
metab_6841	neg	343.0804	1.1503	1.7451	-3.8225	0.0005	0.0056
metab_6083	pos	343.0983	1.1447	2.1427	-7.2122	0.0000	0.0009
metab_7578	neg	343.1145	2.1630	0.4761	-0.0880	0.0186	0.0597
metab_6897	neg	343.1145	1.4427	0.1811	0.3800	0.6708	0.7623
metab_6733	neg	343.1246	0.5711	1.2235	-2.5383	0.0149	0.0516
metab_1914	pos	343.1281	2.4850	0.2178	0.1339	0.6389	0.7425
metab_8436	neg	343.1414	1.6948	0.0567	0.2462	0.6920	0.7791
metab_6009	pos	343.1491	1.2306	0.8111	0.9115	0.0145	0.0464
metab_7549	neg	343.1511	2.0358	0.5822	0.8381	0.0691	0.1486
metab_13715	neg	343.1513	2.3855	0.1470	0.3366	0.7382	0.8111
metab_9365	neg	343.1555	4.3882	0.4105	3.3970	0.6415	0.7389
metab_5563	pos	343.1644	2.0973	0.9015	0.8846	0.0004	0.0040
metab_13031	neg	343.1768	4.1023	2.2337	4.4533	0.0001	0.0021
metab_9136	neg	343.1878	3.5310	0.8170	-0.6231	0.1821	0.3002
metab_8385	neg	343.1989	1.5885	0.7849	3.2198	0.1879	0.3069
metab_12550	neg	343.2129	6.1320	1.0011	1.3274	0.0038	0.0204
metab_13045	neg	343.2130	4.0685	0.4507	0.4445	0.3437	0.4768
metab_8839	neg	343.2130	2.6046	2.2555	6.9150	0.0000	0.0004
metab_12495	neg	343.2132	6.3759	1.3263	4.2658	0.0054	0.0260
metab_12687	neg	343.2491	5.5322	0.6567	-0.4184	0.0386	0.0984
metab_7322	neg	343.2495	8.7031	1.6096	-2.0264	0.0003	0.0039
metab_12184	neg	343.2857	7.4776	0.9849	1.4357	0.0291	0.0812
metab_11577	neg	343.2858	9.1038	2.1642	14.5077	0.0001	0.0014
metab_4516	pos	343.2947	6.1009	0.2368	3.1660	0.6500	0.7515
metab_9015	neg	344.0975	3.0604	0.3042	-1.0742	0.4512	0.5746
metab_14101	neg	344.1147	1.7104	1.7153	-3.4927	0.0002	0.0030
metab_5655	pos	344.1331	1.9309	1.4196	-2.3849	0.0002	0.0022
metab_242	pos	344.1332	0.8081	1.2516	-1.9983	0.0028	0.0149
metab_6869	neg	344.1465	1.2663	1.3383	-2.5626	0.0063	0.0287
metab_9333	neg	344.1508	4.2367	1.1354	-1.7142	0.0408	0.1020
metab_7845	neg	344.1774	0.5286	0.2401	0.6962	0.6656	0.7589
metab_13951	neg	344.1829	1.9439	0.2412	0.5496	0.6180	0.7202
metab_14122	neg	344.1830	1.6642	0.0326	0.6305	0.8899	0.9260
metab_14268	neg	344.1832	1.5155	0.9749	3.9796	0.0313	0.0852
metab_1695	pos	344.1959	1.7412	1.3928	-4.8102	0.0123	0.0414

metab_4974	pos	344.2059	3.8208	1.9010	4.1351	0.0007	0.0059
metab_5285	pos	344.2060	2.7341	1.5711	2.8207	0.0003	0.0036
metab_519	pos	344.2061	2.4370	1.2547	2.6041	0.0084	0.0319
metab_9082	neg	344.2087	3.3144	1.4620	3.4141	0.0523	0.1214
metab_13225	neg	344.2093	3.5310	0.8954	1.8663	0.2176	0.3410
metab_2118	pos	344.2170	3.3032	1.7806	4.7114	0.0023	0.0132
metab_5795	pos	344.2269	1.6272	1.4259	2.4480	0.0015	0.0097
metab_2430	pos	344.2423	5.1218	0.1374	0.5316	0.7488	0.8287
metab_13074	neg	344.2446	3.9677	2.1790	4.9193	0.0000	0.0005
metab_9336	neg	344.2447	4.2533	1.8663	3.7532	0.0001	0.0022
metab_1984	pos	344.2534	2.7959	0.9459	0.8115	0.0550	0.1224
metab_4550	pos	344.2786	5.9361	1.0724	-1.2909	0.2080	0.3279
metab_4442	pos	344.2786	6.5395	0.5604	1.3967	0.4511	0.5808
metab_4420	pos	344.3150	6.6760	0.1731	0.9612	0.7248	0.8104
metab_4055	pos	344.3150	8.3912	0.5124	-0.4962	0.3748	0.5085
metab_2930	pos	344.3152	8.6434	0.8812	-1.0442	0.1681	0.2789
metab_13184	neg	345.0555	3.6311	0.3446	0.5330	0.2808	0.4113
metab_5180	pos	345.1073	3.0726	0.9298	-1.3469	0.1789	0.2928
metab_14016	neg	345.1097	1.8371	0.2528	-0.0612	0.5311	0.6475
metab_8625	neg	345.1099	2.0829	0.3549	-0.3583	0.4096	0.5368
metab_7541	neg	345.1304	2.0358	0.9275	-0.5788	0.2285	0.3536
metab_14082	neg	345.1305	1.7419	0.6035	-0.8995	0.1452	0.2555
metab_14465	neg	345.1305	1.2805	1.2922	2.1421	0.0016	0.0114
metab_9230	neg	345.1349	3.8333	1.3671	7.7503	0.1434	0.2530
metab_1329	pos	345.1368	0.6402	0.8758	-3.3527	0.0305	0.0796
metab_1669	pos	345.1368	1.6983	1.3364	-2.4925	0.0179	0.0536
metab_1805	pos	345.1439	2.1290	0.6091	-0.8749	0.1326	0.2336
metab_13358	neg	345.1461	3.1455	0.0877	0.6589	0.8456	0.8928
metab_3214	pos	345.1534	11.0226	0.3272	-0.2641	0.0154	0.0483
metab_2220	pos	345.1534	3.7599	0.2152	-0.1594	0.1850	0.2998
metab_3683	pos	345.1535	9.9470	0.3991	-0.3311	0.0329	0.0839
metab_3811	pos	345.1535	9.4667	0.5249	-0.3802	0.0165	0.0508
metab_13883	neg	345.1668	2.0516	0.5755	-0.4419	0.1539	0.2666
metab_8868	neg	345.1670	2.6986	0.2995	0.1130	0.5646	0.6758
metab_9495	neg	345.1709	5.0159	2.1126	9.9044	0.0427	0.1056
metab_9354	neg	345.1712	4.3380	0.2231	2.2310	0.6875	0.7757
metab_13037	neg	345.1713	4.0859	2.2061	10.0017	0.0075	0.0323
metab_5184	pos	345.1800	3.0422	0.3208	-0.6663	0.3743	0.5079
metab_12325	neg	345.2072	6.9688	0.4928	-1.9297	0.4147	0.5410
metab_5920	pos	345.2122	1.4005	1.4921	13.1182	0.0172	0.0523
metab_1641	pos	345.2122	1.5987	0.9074	2.8225	0.0646	0.1374
metab_12999	neg	345.2286	4.2203	0.4933	0.8162	0.2773	0.4079
metab_9194	neg	345.2287	3.7317	0.5338	0.5358	0.2739	0.4045
metab_11506	neg	345.2440	9.2834	0.0411	0.0920	0.9438	0.9634
metab_10700	neg	345.9091	11.6337	0.4208	-0.1074	0.0146	0.0509
metab_13774	neg	346.0240	2.2595	0.3387	-0.2626	0.4131	0.5394
metab_7907	neg	346.0559	0.5991	0.1242	0.3776	0.6438	0.7404
metab_10821	neg	346.0561	14.0927	0.2281	0.3264	0.1457	0.2561

metab_8429	neg	346.0566	1.6797	0.2846	0.4738	0.1586	0.2725
metab_14272	neg	346.1150	1.5155	0.1805	0.0284	0.3870	0.5162
metab_8434	neg	346.1232	1.6948	1.0781	-1.4337	0.0477	0.1140
metab_13526	neg	346.1302	2.7624	1.1817	-1.7221	0.0499	0.1173
metab_12992	neg	346.1454	4.2203	2.1626	-11.4599	0.0000	0.0000
metab_1528	pos	346.1596	1.2585	1.8163	-4.6046	0.0017	0.0104
metab_8097	neg	346.1621	1.1355	0.6619	1.1758	0.1047	0.2008
metab_1581	pos	346.1635	1.4429	0.8317	2.9383	0.1949	0.3115
metab_2293	pos	346.1642	4.2301	1.7027	-3.0113	0.0091	0.0337
metab_10459	neg	346.1776	9.3000	0.4627	-0.3730	0.1783	0.2960
metab_9024	neg	346.1779	3.1115	0.3110	1.0124	0.6512	0.7465
metab_1898	pos	346.1961	2.4224	0.5621	3.1264	0.4814	0.6070
metab_12752	neg	346.2028	5.1772	0.0008	-0.4050	0.9556	0.9708
metab_5497	pos	346.2216	2.2045	1.9678	4.3661	0.0006	0.0055
metab_5095	pos	346.2216	3.3490	1.7514	3.6773	0.0000	0.0005
metab_2374	pos	346.2579	4.7001	0.3791	1.0143	0.5858	0.6991
metab_2297	pos	346.2579	4.2609	1.8851	3.4516	0.0000	0.0008
metab_4945	pos	346.2581	3.9577	2.0778	4.3324	0.0000	0.0003
metab_3918	pos	346.3306	8.9656	0.1153	0.6379	0.7497	0.8288
metab_2491	pos	346.7225	5.5864	0.6313	1.8907	0.4125	0.5453
metab_4701	pos	346.7226	5.0610	1.0105	0.9790	0.0126	0.0422
metab_1229	pos	347.0299	0.5140	1.4096	2.1402	0.0031	0.0160
metab_1143	pos	347.0841	0.6402	0.8667	-1.2146	0.0523	0.1178
metab_14608	neg	347.0995	0.9933	0.1216	1.1614	0.9914	0.9933
metab_8711	neg	347.1172	2.2912	1.1650	2.8129	0.0181	0.0590
metab_8653	neg	347.1173	2.1475	0.7737	1.9925	0.1115	0.2105
metab_6425	pos	347.1204	0.5420	1.2710	-1.7885	0.0432	0.1022
metab_5681	pos	347.1227	1.8577	0.6206	0.4014	0.0911	0.1772
metab_5564	pos	347.1228	2.0973	0.3081	-0.6539	0.3739	0.5077
metab_13884	neg	347.1256	2.0516	0.4711	-0.5222	0.1503	0.2618
metab_8235	neg	347.1258	1.3817	0.8234	-0.6767	0.2187	0.3424
metab_268	pos	347.1439	1.0601	1.9545	4.6548	0.0000	0.0008
metab_7518	neg	347.1462	1.8371	0.3877	-0.0925	0.3760	0.5067
metab_14436	neg	347.1462	1.3089	1.2505	2.3293	0.0043	0.0217
metab_1808	pos	347.1594	2.1290	0.1974	0.2688	0.7014	0.7916
metab_5296	pos	347.1804	2.7029	0.4964	-0.5678	0.3816	0.5140
metab_601	pos	347.1844	4.0788	0.4187	4.2283	0.7280	0.8123
metab_6965	neg	347.1980	4.6216	0.9247	1.9873	0.0677	0.1464
metab_12515	neg	347.1993	6.2775	0.8063	-1.6275	0.1536	0.2661
metab_4944	pos	347.2209	3.9577	1.8149	6.8998	0.0042	0.0193
metab_12253	neg	347.2231	7.1903	0.1816	-0.0164	0.7484	0.8202
metab_9329	neg	347.2347	4.2203	0.5616	1.4477	0.5617	0.6729
metab_9397	neg	347.2443	4.5717	1.1979	2.0059	0.0028	0.0165
metab_4691	pos	347.6940	5.1372	1.1055	2.5221	0.0527	0.1186
metab_10741	neg	347.8610	13.9796	0.1749	0.1197	0.4830	0.6050
metab_12931	neg	348.0438	4.4722	1.4998	2.4764	0.0550	0.1259
metab_14231	neg	348.0495	1.5439	1.8033	7.0938	0.0003	0.0037
metab_8171	neg	348.0495	1.2805	1.2766	12.7591	0.0217	0.0667

metab_151	pos	348.0691	0.6542	1.1698	-2.2448	0.0028	0.0150
metab_12897	neg	348.1245	4.5884	2.0519	-7.0874	0.0000	0.0001
metab_13040	neg	348.1246	4.0685	1.9923	-4.6697	0.0000	0.0005
metab_5767	pos	348.1366	1.6838	1.2008	-1.9393	0.0019	0.0117
metab_5283	pos	348.1435	2.7497	1.2159	-2.1265	0.0131	0.0432
metab_8960	neg	348.1568	2.9263	0.6317	6.3084	0.6325	0.7306
metab_12915	neg	348.1824	4.5391	0.6007	-0.9318	0.2160	0.3393
metab_5168	pos	348.1910	3.1191	0.3148	0.4670	0.5995	0.7097
metab_8877	neg	348.1932	2.7155	1.1771	1.5798	0.0124	0.0459
metab_4706	pos	348.2141	5.0307	0.4119	1.2180	0.4788	0.6055
metab_4520	pos	348.2160	6.0561	1.2095	1.9962	0.0148	0.0471
metab_1941	pos	348.2373	2.5938	1.4979	3.4501	0.0027	0.0149
metab_2049	pos	348.2474	3.0114	0.4093	0.1893	0.4640	0.5914
metab_4841	pos	348.2526	4.4272	1.5543	6.4980	0.0061	0.0254
metab_2298	pos	348.2527	4.2750	1.3161	12.1076	0.0032	0.0161
metab_618	pos	348.2735	4.9267	0.0754	-0.3709	0.8602	0.9102
metab_13752	neg	349.0393	2.3078	0.2330	0.0000	0.2923	0.4237
metab_6821	neg	349.0545	0.9508	1.2015	-1.3169	0.0001	0.0015
metab_14433	neg	349.0683	1.3089	0.4821	1.0210	0.5486	0.6607
metab_13407	neg	349.0935	3.0260	0.4195	-0.0974	0.5386	0.6533
metab_8265	neg	349.1049	1.4427	0.0313	0.4337	0.9227	0.9498
metab_9123	neg	349.1201	3.4969	0.7738	-1.0532	0.1208	0.2223
metab_5456	pos	349.1306	2.2978	0.5784	1.0633	0.1465	0.2517
metab_5536	pos	349.1309	2.1444	0.3882	0.8549	0.3538	0.4886
metab_8801	neg	349.1328	2.5113	1.4439	2.5248	0.0107	0.0413
metab_8709	neg	349.1328	2.2912	1.1318	1.9368	0.0209	0.0650
metab_14266	neg	349.1412	1.5155	0.8989	-0.9976	0.0970	0.1900
metab_13848	neg	349.1507	2.1153	0.1091	0.0674	0.8083	0.8636
metab_1710	pos	349.1748	1.7845	1.8624	12.1376	0.0159	0.0493
metab_13106	neg	349.1773	3.8506	0.5339	0.1542	0.3283	0.4608
metab_4956	pos	349.2001	3.9114	1.9581	8.7760	0.0087	0.0329
metab_12667	neg	349.2024	5.6292	3.2531	9.0347	0.0001	0.0024
metab_9348	neg	349.2025	4.3037	1.5309	16.0285	0.0173	0.0570
metab_9166	neg	349.2025	3.6142	0.9045	3.4209	0.3457	0.4788
metab_2360	pos	349.2112	4.6248	0.6323	0.3977	0.4029	0.5361
metab_9679	neg	349.2152	6.0831	0.0979	1.4047	0.7533	0.8229
metab_4419	pos	349.2358	6.6909	1.7144	4.8539	0.0002	0.0027
metab_715	pos	349.2363	7.1864	0.7393	0.4897	0.0989	0.1879
metab_2204	pos	349.2364	3.6987	1.6788	4.3994	0.0053	0.0230
metab_5109	pos	349.2365	3.3032	1.8094	4.5041	0.0019	0.0113
metab_11920	neg	349.2389	8.2099	0.6867	1.1095	0.1013	0.1957
metab_10041	neg	349.2389	7.7251	0.4706	-1.0245	0.3447	0.4777
metab_9398	neg	349.2501	4.5717	1.5192	2.7457	0.0083	0.0351
metab_4781	pos	349.2576	4.6851	1.3252	4.5538	0.0038	0.0182
metab_15133	neg	349.8673	0.0197	0.2879	0.0050	0.1431	0.2526
metab_9050	neg	349.9365	3.1964	0.3953	0.2210	0.5176	0.6359
metab_8002	neg	350.1096	0.8522	1.2282	-1.0375	0.0191	0.0610
metab_13075	neg	350.1250	3.9508	1.0034	-1.9603	0.0529	0.1224

metab_8435	neg	350.1373	1.6948	0.7241	-0.5485	0.0272	0.0779
metab_8310	neg	350.1456	1.5012	0.0095	0.3917	0.9569	0.9715
metab_8871	neg	350.1514	2.6986	0.7132	-0.5335	0.1716	0.2881
metab_8954	neg	350.1515	2.9092	0.8133	-0.5597	0.1701	0.2866
metab_12316	neg	350.1560	7.0162	1.2868	-2.0373	0.0456	0.1105
metab_8720	neg	350.1569	2.3078	0.2457	0.5308	0.5717	0.6820
metab_7672	neg	350.1609	3.0101	0.4991	-0.5220	0.2150	0.3381
metab_5480	pos	350.1700	2.2361	0.8329	-1.2913	0.2154	0.3368
metab_2093	pos	350.1863	3.2116	0.3877	0.4847	0.4852	0.6106
metab_5432	pos	350.1955	2.3600	0.3834	-0.3724	0.3261	0.4598
metab_5324	pos	350.2061	2.6090	1.0805	1.4246	0.0347	0.0871
metab_1802	pos	350.2170	2.1132	0.6573	-0.8013	0.2433	0.3680
metab_5159	pos	350.2317	3.1495	0.4078	0.9346	0.5572	0.6747
metab_9729	neg	350.2336	6.3759	0.0600	1.4826	0.9364	0.9586
metab_527	pos	350.2528	2.3908	1.5126	3.5276	0.0011	0.0082
metab_4290	pos	350.2679	7.2614	0.5138	0.6697	0.1903	0.3057
metab_4405	pos	350.2680	6.7825	0.1356	-0.2634	0.6528	0.7536
metab_4444	pos	350.2680	6.5246	1.1481	-1.4389	0.0351	0.0879
metab_7340	neg	350.2705	8.1774	1.3305	-1.3493	0.0002	0.0031
metab_11750	neg	350.2705	8.7031	1.5569	-1.8720	0.0001	0.0017
metab_5347	pos	350.2793	2.5469	0.3098	-0.5243	0.4865	0.6117
metab_2873	pos	350.3045	8.3463	1.3555	-1.8996	0.0003	0.0037
metab_3926	pos	350.3407	8.9367	1.9582	-4.2315	0.0000	0.0003
metab_1137	pos	351.0677	0.6123	0.2411	0.0587	0.5064	0.6286
metab_1451	pos	351.1109	1.0741	0.9295	3.5274	0.0589	0.1283
metab_8796	neg	351.1202	2.4970	0.0226	0.2858	0.9298	0.9546
metab_13773	neg	351.1202	2.2595	0.5518	-0.4580	0.2323	0.3574
metab_14221	neg	351.1205	1.5439	0.4161	-0.2519	0.3441	0.4771
metab_12987	neg	351.1356	4.2533	1.6403	-2.5762	0.0002	0.0035
metab_6842	neg	351.1414	0.5991	1.6836	-3.1466	0.0071	0.0311
metab_492	pos	351.1461	2.5158	1.1299	1.8062	0.0113	0.0389
metab_5460	pos	351.1461	2.2824	0.8622	1.3547	0.0342	0.0862
metab_8834	neg	351.1481	2.5893	0.1469	0.9710	0.8867	0.9240
metab_5544	pos	351.1542	2.1290	0.2998	-0.6577	0.4052	0.5383
metab_13832	neg	351.1569	2.1475	0.0259	0.5398	0.9213	0.9486
metab_13681	neg	351.1663	2.4487	1.0686	1.9802	0.0088	0.0365
metab_4970	pos	351.1905	3.8511	0.7564	-0.4740	0.1477	0.2533
metab_4629	pos	351.2134	5.4809	0.4677	0.2352	0.1818	0.2959
metab_4692	pos	351.2136	5.1218	0.5081	0.3257	0.2103	0.3306
metab_586	pos	351.2155	4.2907	1.4047	3.3893	0.0082	0.0312
metab_4392	pos	351.2157	6.8582	1.7426	12.4873	0.0000	0.0002
metab_2108	pos	351.2159	3.2573	1.8131	3.3212	0.0002	0.0028
metab_9886	neg	351.2182	7.0796	0.2847	0.2265	0.5363	0.6510
metab_5737	pos	351.2304	1.7271	1.5867	-1.7678	0.0026	0.0143
metab_12572	neg	351.2307	6.0024	1.2840	3.6719	0.1619	0.2767
metab_692	pos	351.2518	6.5857	0.7663	-0.9249	0.0036	0.0177
metab_2786	pos	351.2519	7.7514	0.1586	0.6578	0.8061	0.8716
metab_4116	pos	351.2519	8.1560	1.0717	3.2787	0.0870	0.1712

metab_599	pos	351.2521	4.5184	1.1268	-2.6442	0.0180	0.0539
metab_10086	neg	351.2545	7.9292	1.3641	-2.4874	0.0010	0.0084
metab_11676	neg	351.2546	8.8128	1.0389	-0.4224	0.0172	0.0569
metab_14557	neg	352.0680	1.1212	0.8331	1.9770	0.2135	0.3364
metab_13632	neg	352.1042	2.5264	0.5268	-0.2165	0.1773	0.2947
metab_9382	neg	352.1195	4.4888	1.1745	-3.5489	0.0572	0.1296
metab_8041	neg	352.1252	0.9649	1.6309	-2.5311	0.0002	0.0033
metab_13779	neg	352.1310	2.2437	0.2543	-0.1861	0.4054	0.5334
metab_9055	neg	352.1311	3.2130	0.7097	2.2571	0.2420	0.3681
metab_6021	pos	352.1315	1.2162	1.2126	2.4343	0.0027	0.0148
metab_2251	pos	352.1382	3.9577	1.1059	-2.1789	0.0601	0.1302
metab_6048	pos	352.1490	1.1875	1.5099	4.5183	0.0020	0.0118
metab_14316	neg	352.1514	1.4568	0.4323	0.4685	0.4119	0.5386
metab_1643	pos	352.1599	1.6272	1.0629	-2.3843	0.0396	0.0958
metab_5445	pos	352.1647	2.3292	0.0784	0.2928	0.7780	0.8501
metab_476	pos	352.1648	2.7029	0.7121	-1.1032	0.0737	0.1516
metab_2016	pos	352.1649	2.9192	0.7087	-0.7291	0.1678	0.2788
metab_5614	pos	352.1675	2.0062	0.7410	1.3484	0.1386	0.2415
metab_5034	pos	352.1677	3.5771	0.5210	-1.6188	0.3636	0.4971
metab_14027	neg	352.2134	1.8205	1.1888	-3.5047	0.0540	0.1242
metab_4443	pos	352.2471	6.5246	0.3301	-0.3378	0.3451	0.4800
metab_590	pos	352.2471	4.2609	0.9462	1.6174	0.0838	0.1662
metab_4983	pos	352.2473	3.7753	0.1738	1.0185	0.8159	0.8791
metab_9726	neg	352.2494	6.3593	0.8173	0.1457	0.2273	0.3523
metab_9824	neg	352.2494	6.8730	1.6230	-1.4631	0.0297	0.0820
metab_11934	neg	352.2770	8.1774	1.5731	-1.8142	0.0002	0.0035
metab_4004	pos	352.2831	8.5989	1.8364	-2.9739	0.0003	0.0031
metab_4413	pos	352.2836	6.7366	0.4047	-1.0949	0.5664	0.6825
metab_966	pos	352.2837	8.1857	1.2426	-1.5329	0.0006	0.0057
metab_10201	neg	352.2857	8.3528	0.9505	-0.7405	0.0397	0.1002
metab_11782	neg	352.2862	8.6406	1.2765	-1.1006	0.0018	0.0122
metab_4150	pos	352.3200	8.0419	0.5313	-0.5374	0.0182	0.0543
metab_3952	pos	352.3200	8.7883	0.0913	0.3010	0.7898	0.8587
metab_4074	pos	352.3201	8.3018	0.2457	-0.1160	0.3556	0.4902
metab_3841	pos	352.3565	9.3117	1.6680	-3.2539	0.0000	0.0003
metab_15085	neg	352.8540	0.0444	0.0458	-0.0027	0.8021	0.8585
metab_10931	neg	352.8542	14.3207	0.2861	-0.0123	0.1712	0.2877
metab_15033	neg	352.8971	0.4975	0.4461	-0.4037	0.2683	0.3984
metab_1267	pos	353.0506	0.5420	0.1226	-0.7432	0.7851	0.8555
metab_201	pos	353.0834	0.6123	0.3318	-0.2098	0.3858	0.5182
metab_7613	neg	353.1179	2.5264	1.1311	-2.3389	0.0189	0.0606
metab_13860	neg	353.1182	2.0987	0.8891	-1.6969	0.1023	0.1973
metab_5837	pos	353.1267	1.5424	0.4142	0.9916	0.4418	0.5724
metab_8986	neg	353.1283	2.9931	1.1504	4.0906	0.0920	0.1824
metab_1627	pos	353.1333	1.5565	0.0843	-0.5123	0.7549	0.8323
metab_8942	neg	353.1371	2.8606	0.6222	0.9667	0.2902	0.4215
metab_11810	neg	353.1431	8.5604	0.9980	-0.5971	0.0015	0.0108
metab_5308	pos	353.1492	2.6561	0.0991	-0.1566	0.7747	0.8477

metab_9233	neg	353.1512	3.8506	0.6068	-0.1103	0.3351	0.4680
metab_10403	neg	353.1643	9.0872	0.7787	-0.4313	0.0018	0.0120
metab_5751	pos	353.1696	1.6983	1.1991	1.3851	0.0097	0.0352
metab_9486	neg	353.1973	4.9349	2.2605	6.1250	0.0003	0.0037
metab_12908	neg	353.1975	4.5717	1.4290	2.6985	0.0002	0.0033
metab_6923	neg	353.1976	3.6311	2.7871	6.3413	0.0007	0.0066
metab_12349	neg	353.2006	6.9046	0.3082	1.1178	0.3674	0.4988
metab_4492	pos	353.2077	6.2678	0.6303	0.7625	0.2492	0.3747
metab_9939	neg	353.2166	7.3343	1.5274	11.3223	0.0003	0.0043
metab_1989	pos	353.2175	2.8108	1.4225	8.3039	0.1509	0.2574
metab_7406	neg	353.2338	6.2456	0.8828	1.2948	0.0004	0.0044
metab_12636	neg	353.2338	5.7420	0.7525	1.0230	0.0017	0.0120
metab_12463	neg	353.2338	6.4709	0.9941	1.4820	0.0001	0.0023
metab_12221	neg	353.2340	7.3180	0.7972	-1.2317	0.0335	0.0894
metab_11672	neg	353.2609	8.8128	1.3117	-0.5359	0.0196	0.0619
metab_2855	pos	353.2677	8.2586	0.5722	0.9385	0.2986	0.4297
metab_84	pos	353.2677	6.6001	1.2269	-1.9577	0.0032	0.0161
metab_3722	pos	353.2677	9.8362	1.1474	-1.6857	0.0050	0.0220
metab_2987	pos	353.2678	9.0112	0.3077	0.1222	0.5922	0.7039
metab_11538	neg	353.2702	9.1858	0.1516	1.0618	0.8475	0.8943
metab_4768	pos	353.2787	4.7758	2.0372	9.4167	0.0150	0.0476
metab_11445	neg	353.3065	9.5314	0.4556	-0.2632	0.1093	0.2071
metab_15130	neg	353.8490	0.0197	0.3233	-0.0692	0.0974	0.1906
metab_14893	neg	354.0726	0.5571	1.0895	1.6617	0.0510	0.1190
metab_5942	pos	354.0778	1.3579	0.2702	-0.5971	0.4893	0.6143
metab_14311	neg	354.0836	1.4710	0.1639	0.9023	0.6376	0.7353
metab_14780	neg	354.1045	0.6131	2.0091	-3.8508	0.0000	0.0010
metab_1336	pos	354.1069	0.6682	0.3973	-1.5500	0.5555	0.6731
metab_5138	pos	354.1440	3.2116	0.1311	0.3387	0.7385	0.8207
metab_432	pos	354.1444	2.2519	0.6476	0.8395	0.0967	0.1848
metab_1505	pos	354.1472	1.2162	1.7464	3.7197	0.0001	0.0013
metab_10256	neg	354.1495	8.5604	0.2819	0.2686	0.4508	0.5742
metab_11581	neg	354.1510	9.0872	0.3385	0.0563	0.2717	0.4023
metab_1527	pos	354.1646	1.2585	1.1250	3.4075	0.0537	0.1204
metab_6190	pos	354.1747	0.8920	1.5499	-4.1706	0.0225	0.0634
metab_9533	neg	354.2299	5.2419	0.9874	5.4691	0.0439	0.1076
metab_5672	pos	354.2374	1.8729	0.2595	-0.1717	0.7091	0.7976
metab_5725	pos	354.2376	1.7554	0.2110	0.4691	0.7598	0.8361
metab_2459	pos	354.2626	5.3608	0.2550	0.4933	0.6549	0.7552
metab_2396	pos	354.2628	4.9115	0.5438	0.3716	0.0924	0.1791
metab_5026	pos	354.2630	3.6073	0.4962	0.8569	0.3478	0.4826
metab_5143	pos	354.2630	3.1957	1.6157	-1.5110	0.0080	0.0307
metab_9765	neg	354.2650	6.5673	0.0312	-0.5477	0.9665	0.9766
metab_3982	pos	354.2990	8.6434	0.9270	-0.9495	0.0403	0.0972
metab_7299	neg	354.3019	8.8608	1.1986	-1.6187	0.0145	0.0507
metab_4705	pos	354.7019	5.0307	0.0320	1.1902	0.9835	0.9899
metab_14406	neg	355.0678	1.3517	1.2586	2.4055	0.0394	0.0998
metab_6754	neg	355.0885	0.6551	0.0121	2.3566	0.9319	0.9560

metab_7562	neg	355.1308	2.1798	0.5570	-0.4659	0.0882	0.1773
metab_8946	neg	355.1309	2.8768	0.6287	-0.8834	0.3498	0.4827
metab_11591	neg	355.1587	9.0872	0.7975	-0.4254	0.0011	0.0089
metab_14286	neg	355.1625	1.5012	0.2452	1.4066	0.5521	0.6641
metab_14466	neg	355.1625	1.2805	0.4851	2.5379	0.4824	0.6044
metab_2232	pos	355.1644	3.8511	0.7456	-0.5204	0.2955	0.4262
metab_1894	pos	355.1646	2.4071	0.6836	1.4356	0.1720	0.2842
metab_483	pos	355.1852	2.7959	0.0899	-0.5658	0.8276	0.8866
metab_5470	pos	355.1853	2.2519	0.4916	-1.2767	0.3466	0.4815
metab_8873	neg	355.1872	2.6986	0.3658	2.1399	0.7299	0.8058
metab_13238	neg	355.1879	3.4969	1.3317	4.0298	0.0653	0.1429
metab_9642	neg	355.2130	5.8731	0.5203	0.6626	0.1721	0.2885
metab_12762	neg	355.2130	5.1296	1.9136	5.9357	0.0006	0.0061
metab_12676	neg	355.2130	5.5966	0.4323	-0.4430	0.1893	0.3086
metab_12713	neg	355.2131	5.3877	0.0375	-0.2721	0.8216	0.8736
metab_9158	neg	355.2132	3.5978	1.9316	4.8062	0.0004	0.0048
metab_2347	pos	355.2367	4.5642	2.7493	6.7764	0.0000	0.0001
metab_4934	pos	355.2370	4.0183	2.4528	6.8397	0.0000	0.0004
metab_2573	pos	355.2468	6.1927	1.6445	2.5868	0.0004	0.0043
metab_2599	pos	355.2468	6.4794	1.4679	2.3787	0.0003	0.0037
metab_9805	neg	355.2494	6.7926	0.6557	1.1557	0.0430	0.1062
metab_9327	neg	355.2495	4.2203	0.4006	1.0765	0.4337	0.5578
metab_9405	neg	355.2495	4.5884	0.9395	2.8183	0.2058	0.3277
metab_9936	neg	355.2604	7.3180	0.9203	0.1435	0.1380	0.2460
metab_689	pos	355.2829	6.7825	0.5450	-0.5565	0.2670	0.3945
metab_978	pos	355.2832	7.6185	1.4233	-1.9170	0.0092	0.0340
metab_11427	neg	355.2858	9.5811	0.3851	1.7939	0.4401	0.5641
metab_4278	pos	355.3098	7.3216	0.6859	-0.3727	0.2032	0.3224
metab_10574	neg	355.3222	9.8947	0.0285	0.3324	0.8484	0.8948
metab_9084	neg	356.1257	3.3144	0.2477	0.7359	0.5704	0.6808
metab_8558	neg	356.1260	1.9439	0.0661	0.8591	0.9354	0.9582
metab_13833	neg	356.1261	2.1475	0.1286	0.4254	0.6735	0.7640
metab_11821	neg	356.1301	8.5299	0.1547	0.0874	0.8243	0.8759
metab_8220	neg	356.1359	1.3517	1.2034	-2.2509	0.0507	0.1186
metab_8612	neg	356.1464	2.0516	0.6524	1.2927	0.1318	0.2374
metab_6938	neg	356.1508	4.0020	1.0321	-3.2474	0.0986	0.1921
metab_293	pos	356.1628	1.2162	0.0717	-0.4517	0.9598	0.9754
metab_13742	neg	356.1831	2.3227	0.3988	-1.7467	0.5227	0.6404
metab_6969	neg	356.2085	4.3380	0.7810	0.9823	0.1737	0.2905
metab_9575	neg	356.2445	5.5164	0.4850	1.7148	0.4779	0.6001
metab_4669	pos	356.2783	5.2573	0.2589	1.6470	0.7028	0.7925
metab_2503	pos	356.2783	5.6773	0.7979	-0.9892	0.0497	0.1135
metab_996	pos	356.2784	6.3277	0.6599	-0.8634	0.0645	0.1373
metab_4798	pos	356.2786	4.6096	0.7483	1.5282	0.1507	0.2572
metab_7085	neg	356.2811	8.4178	1.2016	-1.3593	0.0029	0.0168
metab_7095	neg	356.2811	8.7031	1.4530	-1.9558	0.0010	0.0088
metab_3940	pos	356.3149	8.8618	1.0262	-1.5851	0.0804	0.1615
metab_12962	neg	357.0621	4.3380	0.4616	-0.1123	0.0158	0.0538

metab_6850	neg	357.0808	1.2377	0.5593	0.9090	0.1412	0.2502
metab_8617	neg	357.1105	2.0516	0.6917	-1.1752	0.2541	0.3826
metab_8913	neg	357.1128	2.7961	1.5075	2.7792	0.0015	0.0108
metab_427	pos	357.1437	2.2045	0.2317	0.0976	0.3517	0.4867
metab_14862	neg	357.1516	0.5711	0.5851	-0.7587	0.1536	0.2661
metab_5473	pos	357.1646	2.2519	0.4460	-1.0078	0.4849	0.6103
metab_13467	neg	357.1666	2.8768	0.2786	-0.3725	0.5250	0.6423
metab_533	pos	357.1797	2.2978	0.0204	0.1213	0.9883	0.9924
metab_1813	pos	357.1799	2.1444	0.7735	1.0231	0.1076	0.2002
metab_9266	neg	357.1926	3.9677	0.3668	0.7491	0.4628	0.5853
metab_5052	pos	357.2008	3.5162	2.3039	3.9228	0.0108	0.0377
metab_1966	pos	357.2010	2.7029	0.7691	1.1685	0.1429	0.2470
metab_13396	neg	357.2030	3.0432	1.9859	4.3568	0.0011	0.0089
metab_13896	neg	357.2148	2.0358	0.4313	1.4615	0.5435	0.6571
metab_9494	neg	357.2287	5.0002	0.5284	0.6451	0.2281	0.3531
metab_13004	neg	357.2289	4.2040	1.5541	2.9040	0.0017	0.0119
metab_9253	neg	357.2289	3.9177	1.9605	5.6700	0.0009	0.0077
metab_12238	neg	357.2649	7.2225	1.3057	2.1950	0.0137	0.0488
metab_12451	neg	357.2650	6.5033	1.3603	4.8419	0.0001	0.0024
metab_12658	neg	357.2652	5.6615	0.6316	1.2962	0.1074	0.2047
metab_11337	neg	357.3287	9.8947	0.0225	0.3832	0.8587	0.9023
metab_4673	pos	357.6465	5.2277	1.3882	11.8487	0.0010	0.0074
metab_1924	pos	358.1207	2.5158	0.8348	-1.2332	0.0358	0.0891
metab_5649	pos	358.1388	1.9463	0.4733	0.9672	0.3699	0.5037
metab_6886	neg	358.1514	1.3817	0.2697	-1.0371	0.5690	0.6797
metab_1780	pos	358.1592	2.0524	0.5042	0.7287	0.2367	0.3604
metab_7547	neg	358.1622	2.0987	1.0129	-0.8971	0.0512	0.1193
metab_8487	neg	358.1622	1.7885	0.8422	-0.3761	0.1022	0.1971
metab_9440	neg	358.1664	4.7384	2.0458	-2.8534	0.0570	0.1293
metab_9196	neg	358.1779	3.7317	1.0501	3.0985	0.1138	0.2129
metab_1864	pos	358.1961	2.3133	0.6469	-2.1433	0.3243	0.4581
metab_13496	neg	358.1986	2.8123	0.6625	1.0042	0.1748	0.2918
metab_13789	neg	358.1986	2.2272	0.1700	0.6878	0.7726	0.8374
metab_5776	pos	358.2060	1.6553	1.4661	5.3650	0.0487	0.1119
metab_535	pos	358.2215	3.4251	1.2829	2.0593	0.0024	0.0134
metab_2534	pos	358.2577	5.9049	1.1442	4.4619	0.0194	0.0569
metab_12626	neg	358.2604	5.7751	1.7332	3.8083	0.0027	0.0162
metab_12005	neg	358.2606	7.9908	1.3712	2.4568	0.0002	0.0028
metab_5153	pos	358.2691	3.1648	0.2894	-0.1700	0.6835	0.7779
metab_4120	pos	358.2730	8.1414	0.5612	-0.7366	0.1374	0.2401
metab_2628	pos	358.2731	6.7825	0.3585	-0.1536	0.6270	0.7328
metab_4047	pos	358.2941	8.4074	1.2570	-1.8407	0.0026	0.0144
metab_4079	pos	358.2941	8.2872	1.0160	-1.4929	0.0168	0.0512
metab_21	pos	358.2942	5.7086	0.2939	-0.0177	0.5059	0.6283
metab_2538	pos	358.2942	5.9361	0.9686	-1.0393	0.0554	0.1230
metab_2948	pos	358.3304	8.7444	0.7803	-1.5738	0.1494	0.2556
metab_3028	pos	358.3671	9.2658	1.4632	2.7014	0.0003	0.0037
metab_847	pos	358.3671	9.6199	0.4972	-0.9063	0.2684	0.3961

metab_15012	neg	358.9448	0.5126	0.8717	-1.2397	0.0374	0.0962
metab_8842	neg	358.9742	2.6046	1.0149	-1.2896	0.0124	0.0459
metab_14692	neg	359.0736	0.8240	1.5017	-2.2737	0.0103	0.0404
metab_8289	neg	359.0785	1.4568	0.4205	-1.3225	0.4960	0.6171
metab_14468	neg	359.1099	1.2805	0.3488	0.8437	0.4777	0.5999
metab_8058	neg	359.1101	1.0215	0.9649	1.5530	0.0069	0.0307
metab_2013	pos	359.1229	2.8877	0.1353	-0.0210	0.7304	0.8142
metab_8953	neg	359.1255	2.9092	0.8706	1.1745	0.0000	0.0014
metab_7565	neg	359.1255	2.1798	0.2714	-0.5165	0.5219	0.6397
metab_8559	neg	359.1257	1.9439	0.1654	0.4080	0.7061	0.7894
metab_5553	pos	359.1257	2.1132	0.0741	0.3776	0.7840	0.8551
metab_13197	neg	359.1529	3.5978	0.3318	1.3906	0.4971	0.6178
metab_12890	neg	359.1538	4.6216	1.3220	2.0856	0.0404	0.1016
metab_7635	neg	359.1722	2.6986	1.3813	2.5250	0.0016	0.0115
metab_9206	neg	359.1728	3.7658	0.6805	1.0876	0.2034	0.3250
metab_13555	neg	359.1826	2.6986	0.7888	-0.9833	0.1048	0.2009
metab_9076	neg	359.1828	3.2972	0.3469	0.3975	0.6105	0.7139
metab_12708	neg	359.1901	5.4188	0.2797	-1.0585	0.6286	0.7276
metab_5573	pos	359.1910	2.0677	1.6027	-5.1955	0.0007	0.0060
metab_12792	neg	359.2081	4.9668	0.2256	0.0054	0.7330	0.8075
metab_9115	neg	359.2082	3.4639	1.3205	2.4192	0.0130	0.0472
metab_8867	neg	359.2083	2.6841	2.6289	10.5936	0.0000	0.0002
metab_12556	neg	359.2266	6.0993	1.5137	-2.1382	0.0296	0.0818
metab_12605	neg	359.2267	5.8731	1.4045	-2.1883	0.0897	0.1795
metab_5601	pos	359.2277	2.0211	1.1107	1.7570	0.0231	0.0646
metab_4145	pos	359.2765	8.0419	0.7886	-10.6022	0.0083	0.0315
metab_4576	pos	359.3259	5.7846	1.2655	4.4325	0.0332	0.0845
metab_13191	neg	359.9397	3.6142	1.6346	-3.7571	0.0005	0.0052
metab_13478	neg	360.0407	2.8440	0.7073	-0.6448	0.1740	0.2907
metab_14359	neg	360.1415	1.4427	0.1946	0.1208	0.5795	0.6884
metab_14580	neg	360.1416	1.0642	0.4143	-0.9009	0.3738	0.5048
metab_13366	neg	360.1460	3.1286	1.3241	-1.9023	0.0509	0.1189
metab_6219	pos	360.1489	0.8500	0.1445	0.4900	0.5611	0.6777
metab_8689	neg	360.1570	2.2272	0.1817	1.6359	0.8553	0.8997
metab_13454	neg	360.1672	2.9092	1.9093	10.3562	0.0036	0.0193
metab_5696	pos	360.1754	1.7993	0.9829	-0.8505	0.0829	0.1650
metab_8209	neg	360.1780	1.3231	0.2770	0.0214	0.4852	0.6071
metab_14141	neg	360.1781	1.6491	0.0189	0.3638	0.9473	0.9653
metab_1818	pos	360.1784	2.1595	1.2976	-2.0620	0.0011	0.0079
metab_4774	pos	360.1796	4.7302	1.7759	-3.3922	0.0349	0.0876
metab_12849	neg	360.1821	4.7709	1.2278	-2.0967	0.0377	0.0969
metab_1023	pos	360.1906	2.0973	0.5069	0.4830	0.2131	0.3341
metab_2146	pos	360.1907	3.4251	0.4128	-0.5912	0.1290	0.2291
metab_5496	pos	360.2115	2.2045	0.3190	0.0816	0.5249	0.6460
metab_4660	pos	360.2158	5.3165	0.5787	-0.3187	0.4971	0.6206
metab_4618	pos	360.2159	5.5715	0.3497	0.3393	0.7129	0.8004
metab_5708	pos	360.2227	1.7705	1.2050	7.3861	0.0686	0.1439
metab_5146	pos	360.2372	3.1799	2.0634	4.8433	0.0002	0.0022

metab_13294	neg	360.2396	3.2972	0.3495	-5.1485	0.6616	0.7557
metab_5383	pos	360.2735	2.4530	1.8500	12.8613	0.0015	0.0099
metab_2076	pos	360.2736	3.1648	0.6370	0.8968	0.4118	0.5444
metab_12512	neg	360.2762	6.2943	1.5218	3.3120	0.0046	0.0229
metab_4174	pos	360.3099	7.9407	0.6466	-0.4684	0.2911	0.4215
metab_25	pos	360.3099	6.2226	0.2155	0.5526	0.6348	0.7395
metab_4540	pos	360.3099	5.9649	1.1502	-0.6985	0.1364	0.2387
metab_4051	pos	360.3100	8.3912	0.1002	0.2125	0.8586	0.9092
metab_3561	pos	360.3251	11.4801	0.3023	-0.3131	0.2536	0.3797
metab_2936	pos	360.3252	8.6861	0.6525	-0.6210	0.0001	0.0020
metab_3874	pos	360.3252	9.1451	0.9673	-1.0750	0.0000	0.0002
metab_13298	neg	360.9714	3.2972	0.4474	-0.0810	0.5048	0.6245
metab_3314	pos	360.9894	14.1389	0.6800	-0.6061	0.0006	0.0055
metab_14421	neg	361.0105	1.3231	0.5611	-0.0113	0.2136	0.3364
metab_1542	pos	361.0690	1.3293	1.3143	1.9439	0.0122	0.0413
metab_13760	neg	361.0936	2.2912	1.1803	2.0019	0.0109	0.0419
metab_8551	neg	361.0940	1.9280	1.4744	2.3199	0.0012	0.0098
metab_14053	neg	361.1071	1.7729	0.6957	0.2416	0.4016	0.5298
metab_14996	neg	361.1112	0.5126	0.8788	-1.3006	0.0278	0.0791
metab_261	pos	361.1231	1.0180	1.6334	2.5278	0.0459	0.1071
metab_6843	neg	361.1258	1.1940	0.6002	0.9733	0.1096	0.2076
metab_5647	pos	361.1386	1.9463	0.3401	-0.3562	0.5806	0.6939
metab_422	pos	361.1386	2.1897	0.0141	-0.1695	0.9775	0.9875
metab_496	pos	361.1386	2.9028	0.3218	0.1746	0.1076	0.2002
metab_9053	neg	361.1410	3.2130	0.2495	0.1937	0.5866	0.6943
metab_13290	neg	361.1663	3.3144	1.4162	0.6417	0.0740	0.1557
metab_9231	neg	361.1666	3.8333	0.8147	5.4731	0.3625	0.4949
metab_4792	pos	361.1669	4.6248	1.6899	2.2279	0.0384	0.0935
metab_5860	pos	361.1704	1.5144	1.6229	13.1792	0.0010	0.0075
metab_6930	neg	361.2027	3.8001	2.0388	5.4131	0.0007	0.0068
metab_13244	neg	361.2028	3.4800	1.4520	13.9084	0.0062	0.0284
metab_12774	neg	361.2137	5.0812	0.3712	1.6048	0.6371	0.7351
metab_4912	pos	361.2194	4.0788	0.8670	-1.9924	0.1203	0.2175
metab_9387	neg	361.2237	4.5219	1.2315	1.8019	0.0128	0.0467
metab_13051	neg	361.2238	4.0516	0.8259	1.0689	0.0560	0.1275
metab_12795	neg	361.2238	4.9510	2.1813	5.1487	0.0000	0.0013
metab_9058	neg	361.2238	3.2301	1.0858	1.5559	0.0074	0.0323
metab_12345	neg	361.2601	6.9046	0.9440	1.3419	0.0700	0.1499
metab_13405	neg	361.8382	3.0260	1.4600	-1.9715	0.0007	0.0068
metab_7436	neg	361.9366	3.5978	2.0723	-4.0322	0.0003	0.0041
metab_8931	neg	361.9978	2.8440	0.2930	-0.0447	0.4529	0.5764
metab_8804	neg	362.0466	2.5113	1.0732	-1.5692	0.0243	0.0722
metab_14747	neg	362.0510	0.6973	0.9138	-0.8871	0.0870	0.1756
metab_5946	pos	362.1101	1.3437	2.5210	12.5910	0.0000	0.0000
metab_14457	neg	362.1103	1.2805	1.0995	-1.3383	0.0156	0.0534
metab_7545	neg	362.1361	2.0516	0.8336	-0.7796	0.0049	0.0243
metab_215	pos	362.1370	0.6402	1.7023	-4.0082	0.0049	0.0215
metab_2111	pos	362.1494	3.2727	0.5679	-0.3074	0.2884	0.4190

metab_2072	pos	362.1590	3.1346	1.4236	-2.5239	0.0186	0.0552
metab_9245	neg	362.1934	3.8839	0.3628	1.2114	0.6869	0.7753
metab_8752	neg	362.2092	2.3855	1.1058	12.1430	0.0286	0.0804
metab_2468	pos	362.2889	5.3913	0.3942	-0.9836	0.4322	0.5634
metab_4721	pos	362.2891	4.9554	0.2803	-1.0361	0.5886	0.7010
metab_10064	neg	362.2918	7.8352	0.0127	-0.2875	0.9049	0.9369
metab_4525	pos	362.3256	6.0252	0.7280	0.5317	0.0175	0.0530
metab_7731	neg	362.8757	0.0197	0.1725	0.0608	0.3727	0.5039
metab_11030	neg	362.8757	14.0610	0.2262	0.0490	0.1936	0.3137
metab_8843	neg	362.9506	2.6206	1.1282	-0.4674	0.0516	0.1201
metab_13297	neg	362.9683	3.2972	0.2741	-0.1955	0.6626	0.7564
metab_3457	pos	362.9898	14.1243	0.4553	-0.3779	0.0455	0.1065
metab_6774	neg	362.9975	0.7394	1.5620	-3.9081	0.0009	0.0077
metab_13916	neg	363.0697	2.0055	1.1504	2.0432	0.0604	0.1350
metab_8060	neg	363.0940	1.0215	1.3546	-1.8999	0.0297	0.0822
metab_13750	neg	363.1122	2.3078	0.7102	1.1549	0.0793	0.1644
metab_13995	neg	363.1204	1.8682	1.1266	1.8851	0.0006	0.0059
metab_14581	neg	363.1413	1.0642	2.0107	4.8542	0.0000	0.0010
metab_13398	neg	363.1568	3.0432	1.5410	-1.9908	0.0101	0.0401
metab_13856	neg	363.1593	2.0987	1.6318	-2.7521	0.0002	0.0028
metab_7649	neg	363.1668	2.8123	0.3036	1.0827	0.6875	0.7757
metab_14115	neg	363.1677	1.6797	0.6078	1.7440	0.2985	0.4289
metab_8287	neg	363.1778	1.4568	0.5150	-1.0183	0.5864	0.6943
metab_7660	neg	363.1820	2.9429	1.9034	5.3055	0.0056	0.0265
metab_9044	neg	363.1821	3.1797	0.5258	1.1523	0.4349	0.5588
metab_9165	neg	363.1822	3.6142	0.8138	3.7672	0.3226	0.4551
metab_12995	neg	363.1935	4.2203	0.2841	-0.3781	0.6939	0.7805
metab_2075	pos	363.2159	3.1648	2.4674	7.6095	0.0001	0.0021
metab_2241	pos	363.2160	3.8967	1.7709	14.1624	0.0105	0.0371
metab_12318	neg	363.2160	7.0162	0.7460	-1.3999	0.1308	0.2360
metab_12426	neg	363.2180	6.6157	0.0285	-0.7417	0.9701	0.9788
metab_9591	neg	363.2182	5.5966	1.6230	4.1292	0.0020	0.0131
metab_6934	neg	363.2184	3.8839	1.8682	4.4135	0.0001	0.0025
metab_7426	neg	363.2184	4.1869	1.6389	4.3671	0.0020	0.0133
metab_12951	neg	363.2393	4.3716	1.4336	2.9266	0.0072	0.0315
metab_3524	pos	363.2783	14.0074	0.2103	-0.1903	0.1574	0.2653
metab_2502	pos	363.2785	5.6773	0.0941	-0.1109	0.6558	0.7557
metab_4563	pos	363.2997	5.8753	0.2896	2.7593	0.5308	0.6515
metab_2513	pos	363.7148	5.7232	0.4466	0.9868	0.5769	0.6907
metab_13404	neg	363.8351	3.0260	1.8226	-3.0025	0.0000	0.0002
metab_8794	neg	364.0136	2.4813	1.6495	-2.0877	0.0005	0.0057
metab_1036	pos	364.1312	1.7554	0.0566	0.1508	0.9809	0.9884
metab_1468	pos	364.1315	1.1306	1.1627	-1.5376	0.0000	0.0003
metab_13083	neg	364.1408	3.9177	0.7926	-0.9258	0.2057	0.3276
metab_14218	neg	364.1519	1.5439	0.0631	-0.0177	0.8532	0.8982
metab_8651	neg	364.1521	2.1475	0.0709	0.8951	0.9170	0.9448
metab_8300	neg	364.1521	1.4710	0.1796	0.0188	0.7896	0.8493
metab_8593	neg	364.1883	2.0055	1.0950	-0.8716	0.0809	0.1667

metab_8654	neg	364.1883	2.1475	1.2138	2.6833	0.0234	0.0703
metab_12603	neg	364.2136	5.8731	0.7095	-0.4978	0.2533	0.3818
metab_7006	neg	364.2137	6.3111	0.3630	1.8894	0.6029	0.7073
metab_1929	pos	364.2219	2.5314	0.8149	0.9010	0.0739	0.1518
metab_5429	pos	364.2223	2.3751	2.0458	5.7070	0.0000	0.0005
metab_12447	neg	364.2495	6.5346	0.6665	-0.0421	0.1921	0.3118
metab_4899	pos	364.2684	4.1544	2.4427	12.8073	0.0058	0.0244
metab_989	pos	364.2832	6.4192	0.9169	2.8543	0.0754	0.1537
metab_7351	neg	364.2863	8.0688	1.0044	2.2425	0.0209	0.0650
metab_5364	pos	364.7073	2.5003	1.8536	8.1171	0.0051	0.0222
metab_15150	neg	364.7991	0.0136	0.4615	-0.1006	0.0084	0.0352
metab_11100	neg	364.8779	14.0117	0.3683	-0.0071	0.2944	0.4253
metab_13596	neg	364.9473	2.6206	1.2011	-0.6174	0.0307	0.0841
metab_6275	pos	365.0470	0.7521	1.0889	-0.4725	0.0255	0.0700
metab_1761	pos	365.0829	1.9918	0.4460	2.8227	0.3188	0.4516
metab_13576	neg	365.1148	2.6524	0.5749	-1.4922	0.3567	0.4895
metab_14670	neg	365.1206	0.8662	1.3626	-12.0356	0.0007	0.0066
metab_13736	neg	365.1358	2.3380	0.6704	-0.1436	0.0825	0.1687
metab_13399	neg	365.1359	3.0432	0.1502	-0.0525	0.7110	0.7933
metab_6909	neg	365.1360	1.4710	1.1921	-1.5105	0.0398	0.1005
metab_9073	neg	365.1628	3.2801	0.4562	2.4903	0.4881	0.6097
metab_13476	neg	365.1721	2.8606	0.3127	0.3550	0.3834	0.5129
metab_13365	neg	365.1974	3.1286	1.8207	6.0089	0.0074	0.0323
metab_9517	neg	365.1975	5.1456	1.5817	6.3931	0.0400	0.1008
metab_7433	neg	365.1975	3.6980	1.5490	4.0251	0.0052	0.0254
metab_6359	pos	365.2017	0.5983	1.5332	-4.7440	0.0042	0.0193
metab_6992	neg	365.2103	4.9349	0.0374	0.1025	0.9368	0.9586
metab_4902	pos	365.2315	4.1387	1.6795	4.4556	0.0029	0.0155
metab_2090	pos	365.2315	3.2116	1.7780	4.1173	0.0072	0.0286
metab_2243	pos	365.2315	3.9114	1.5888	3.0108	0.0014	0.0093
metab_12220	neg	365.2329	7.3180	0.1246	-0.4879	0.8118	0.8660
metab_12164	neg	365.2336	7.5397	0.6725	0.5637	0.1445	0.2544
metab_7026	neg	365.2336	6.9525	1.0965	1.3822	0.0313	0.0852
metab_9816	neg	365.2336	6.8255	0.7005	-2.2379	0.2322	0.3574
metab_12078	neg	365.2336	7.7728	0.8828	1.1014	0.0159	0.0541
metab_9216	neg	365.2338	3.8001	1.7282	4.7472	0.0015	0.0108
metab_7423	neg	365.2339	4.2203	1.3745	3.3890	0.0282	0.0797
metab_1602	pos	365.2537	1.5144	1.0033	-1.5755	0.1354	0.2377
metab_711	pos	365.2673	7.0961	0.1310	0.6207	0.8489	0.9025
metab_10326	neg	365.2701	8.7346	2.3114	-8.2398	0.0000	0.0001
metab_2800	pos	365.3152	7.8830	0.6832	2.6995	0.2649	0.3927
metab_4510	pos	365.3153	6.1311	0.8198	13.0932	0.0851	0.1684
metab_6364	pos	366.0786	0.5983	1.3155	-18.6461	0.0031	0.0160
metab_6005	pos	366.0940	1.2446	2.1792	-2.5956	0.0448	0.1052
metab_8181	neg	366.1056	1.2948	1.0567	-1.2910	0.0251	0.0735
metab_8069	neg	366.1407	1.0357	0.2822	0.4694	0.4682	0.5906
metab_13816	neg	366.1471	2.1798	0.4540	-0.1546	0.3409	0.4741
metab_1811	pos	366.1650	2.1444	0.0552	0.3250	0.9160	0.9450

metab_8781	neg	366.1772	2.4487	2.4215	8.4705	0.0000	0.0009
metab_5610	pos	366.2012	2.0062	0.7012	-0.6621	0.0739	0.1518
metab_1812	pos	366.2014	2.1444	0.3529	0.4512	0.3041	0.4359
metab_4485	pos	366.2264	6.3131	1.1703	2.4423	0.0639	0.1363
metab_2221	pos	366.2375	3.7599	0.0864	-0.1295	0.8286	0.8872
metab_4502	pos	366.2628	6.1927	1.0049	-1.2602	0.0870	0.1713
metab_678	pos	366.2629	6.5246	0.6055	-0.2349	0.2378	0.3619
metab_2370	pos	366.2629	4.6851	0.6141	1.0325	0.3531	0.4881
metab_2326	pos	366.2630	4.4429	1.2145	1.2787	0.0862	0.1702
metab_9803	neg	366.2652	6.7764	0.2168	-0.2929	0.6388	0.7364
metab_12317	neg	366.2652	7.0162	1.6319	-2.6512	0.0103	0.0404
metab_9759	neg	366.2653	6.5190	1.4139	-1.5086	0.0347	0.0912
metab_6577	neg	366.2653	7.9137	0.7214	-0.8089	0.0425	0.1055
metab_10293	neg	366.2654	8.7031	1.1852	-1.2102	0.0000	0.0005
metab_10126	neg	366.2921	8.0688	1.2397	4.4407	0.0121	0.0448
metab_2940	pos	366.2989	8.7008	0.9243	3.1333	0.1302	0.2306
metab_4370	pos	366.2990	6.9182	0.8611	3.3355	0.0939	0.1812
metab_969	pos	366.2994	8.0700	0.9972	1.8782	0.0318	0.0819
metab_7330	neg	366.3018	8.5455	1.0460	2.6431	0.0233	0.0701
metab_4180	pos	366.3357	7.9118	1.6644	-2.9773	0.0001	0.0013
metab_3088	pos	366.3721	9.6968	0.2118	-0.1868	0.3302	0.4645
metab_8809	neg	366.9242	2.5264	0.7339	-0.7386	0.2214	0.3454
metab_14741	neg	367.0286	0.7254	1.5250	-4.0025	0.0001	0.0023
metab_13948	neg	367.1012	1.9596	0.6275	1.2087	0.2094	0.3319
metab_6746	neg	367.1016	0.5991	1.4707	4.9101	0.0065	0.0295
metab_13976	neg	367.1149	1.8974	0.4106	-0.0589	0.4347	0.5587
metab_8312	neg	367.1151	1.5012	0.2778	-0.1003	0.4594	0.5819
metab_8895	neg	367.1429	2.7624	1.4414	2.6738	0.0003	0.0037
metab_329	pos	367.1490	1.4714	1.1263	-1.6403	0.0337	0.0853
metab_5661	pos	367.1491	1.9169	0.5441	-1.3101	0.2562	0.3830
metab_444	pos	367.1491	2.3292	0.8159	-0.9328	0.0109	0.0380
metab_8405	neg	367.1519	1.6335	0.4594	0.0924	0.3907	0.5191
metab_11652	neg	367.1587	8.8929	0.8941	3.7013	0.0004	0.0049
metab_8426	neg	367.1606	1.6797	1.4623	2.5476	0.0032	0.0181
metab_9562	neg	367.1665	5.4188	0.8651	-2.3510	0.1996	0.3203
metab_491	pos	367.1854	2.8725	0.4708	-0.1944	0.1552	0.2625
metab_13278	neg	367.1877	3.3465	0.4863	-0.2151	0.4082	0.5355
metab_2131	pos	367.2106	3.3490	0.0548	0.2468	0.9102	0.9427
metab_13199	neg	367.2131	3.5978	3.4442	15.8550	0.0000	0.0009
metab_12940	neg	367.2131	4.4221	1.7902	4.3626	0.0009	0.0081
metab_9037	neg	367.2131	3.1626	2.4378	10.3251	0.0001	0.0023
metab_9536	neg	367.2254	5.2579	1.7808	6.1593	0.0044	0.0223
metab_4503	pos	367.2467	6.1757	0.9270	-1.6051	0.0127	0.0423
metab_4658	pos	367.2470	5.3165	1.0084	-1.4054	0.0178	0.0534
metab_2048	pos	367.2471	3.0114	1.7431	2.9342	0.0061	0.0254
metab_10404	neg	367.2475	9.0872	0.8836	-0.6810	0.0015	0.0109
metab_9751	neg	367.2485	6.4553	0.1012	0.6730	0.8225	0.8743
metab_9716	neg	367.2488	6.3111	0.1493	0.5702	0.6903	0.7777

metab_9692	neg	367.2491	6.1482	0.1367	0.5055	0.6934	0.7802
metab_12229	neg	367.2492	7.2701	0.4645	-0.2156	0.2966	0.4273
metab_9300	neg	367.2494	4.1023	1.9579	5.7048	0.0016	0.0112
metab_9236	neg	367.2494	3.8506	1.6479	8.7602	0.0190	0.0609
metab_10186	neg	367.2495	8.3055	1.8755	-3.2561	0.0001	0.0026
metab_4985	pos	367.2682	3.7599	1.3969	13.5103	0.0411	0.0987
metab_11393	neg	367.3221	9.6986	0.4179	0.7117	0.2778	0.4082
metab_13283	neg	367.9891	3.3304	0.8392	0.6332	0.1702	0.2866
metab_229	pos	368.0133	0.7381	1.3201	-2.0599	0.0002	0.0026
metab_6304	pos	368.0134	0.6402	0.9450	-1.5258	0.0109	0.0381
metab_14229	neg	368.0996	1.5439	0.1857	0.2866	0.6532	0.7482
metab_8149	neg	368.1096	1.2521	0.1528	0.8506	0.8341	0.8835
metab_14781	neg	368.1199	0.6131	1.9658	-3.9407	0.0000	0.0005
metab_14181	neg	368.1464	1.5885	0.2142	0.9472	0.7726	0.8374
metab_9007	neg	368.1466	3.0432	0.3968	-0.3817	0.3677	0.4992
metab_5585	pos	368.1601	2.0524	0.2576	-0.1791	0.5456	0.6647
metab_6060	pos	368.1628	1.1875	0.2396	-0.6625	0.4227	0.5542
metab_14061	neg	368.1830	1.7572	0.8471	1.3987	0.1088	0.2066
metab_1930	pos	368.1991	2.5469	0.2288	-0.9148	0.5416	0.6615
metab_1879	pos	368.2170	2.3600	1.0873	4.5485	0.0478	0.1102
metab_5173	pos	368.2422	3.1032	1.0957	-1.4396	0.1064	0.1984
metab_2143	pos	368.2422	3.4098	0.7163	-0.1516	0.2642	0.3920
metab_12722	neg	368.2439	5.3388	1.0142	0.1528	0.1499	0.2613
metab_9457	neg	368.2444	4.8049	0.6377	-0.2226	0.2402	0.3663
metab_12033	neg	368.2714	7.9137	1.4673	-1.8791	0.0020	0.0130
metab_2482	pos	368.2786	5.5260	0.0174	1.4225	0.9488	0.9671
metab_4407	pos	368.2786	6.7671	0.0697	-0.6184	0.8899	0.9300
metab_4348	pos	368.2786	7.0082	1.7060	-3.0800	0.0030	0.0158
metab_118	pos	368.2787	7.9118	0.5849	-0.7612	0.1809	0.2951
metab_12156	neg	368.2808	7.5541	1.4051	-3.9937	0.0109	0.0419
metab_12405	neg	368.2809	6.7439	0.8284	-1.8941	0.3633	0.4957
metab_12313	neg	368.2810	7.0162	1.2521	-1.8732	0.0360	0.0934
metab_11866	neg	368.2810	8.4022	0.5605	-0.1835	0.1359	0.2429
metab_10252	neg	368.3083	8.5455	1.0000	4.7154	0.0274	0.0783
metab_4020	pos	368.3149	8.5393	1.1830	2.5075	0.0173	0.0523
metab_10375	neg	368.3174	9.0067	1.1411	-1.4631	0.0128	0.0466
metab_973	pos	368.3423	7.9118	1.2575	-1.9974	0.0148	0.0470
metab_3989	pos	368.3513	8.6284	1.2143	-1.7456	0.0110	0.0383
metab_7868	neg	368.8356	0.5431	0.4957	-0.2251	0.1515	0.2632
metab_8810	neg	368.9211	2.5264	0.7092	-0.6888	0.2453	0.3719
metab_10898	neg	369.1041	15.3987	0.2917	-0.0348	0.1114	0.2103
metab_7783	neg	369.1041	0.4634	0.0651	0.1234	0.7306	0.8061
metab_13180	neg	369.1133	3.6479	1.0388	1.8803	0.0334	0.0892
metab_14075	neg	369.1305	1.7419	0.7280	-0.9636	0.2888	0.4204
metab_8927	neg	369.1463	2.8269	0.3614	0.8249	0.5443	0.6574
metab_1064	pos	369.1542	1.4569	0.7985	-1.1384	0.0947	0.1821
metab_1976	pos	369.1563	2.7497	1.2249	2.9528	0.0421	0.1004
metab_14161	neg	369.1782	1.6186	0.1045	1.7439	0.7539	0.8231

metab_13930	neg	369.1782	1.9904	0.4245	2.2957	0.5732	0.6832
metab_13121	neg	369.1924	3.8001	3.1088	13.2792	0.0000	0.0001
metab_2130	pos	369.2015	3.3490	0.6173	-0.2176	0.2803	0.4097
metab_2516	pos	369.2032	5.7385	1.4541	2.0150	0.0004	0.0045
metab_12354	neg	369.2106	6.8887	1.6344	3.5256	0.0027	0.0159
metab_5163	pos	369.2165	3.1346	0.0843	-3.1921	0.8471	0.9016
metab_11969	neg	369.2202	8.0843	0.6445	0.8233	0.4060	0.5337
metab_1013	pos	369.2240	4.8961	1.0447	1.0071	0.0525	0.1182
metab_4893	pos	369.2241	4.1994	1.5145	2.4333	0.0353	0.0881
metab_7701	neg	369.2287	3.2972	1.6391	3.3589	0.0001	0.0022
metab_12721	neg	369.2288	5.3546	1.2885	2.1531	0.0000	0.0005
metab_12927	neg	369.2288	4.5054	1.4846	3.0546	0.0009	0.0079
metab_9286	neg	369.2392	4.0516	2.4487	6.0783	0.0001	0.0026
metab_2278	pos	369.2528	4.1234	2.0325	5.0291	0.0000	0.0003
metab_2615	pos	369.2626	6.6606	1.0970	-1.6609	0.0016	0.0104
metab_13071	neg	369.2650	3.9677	1.6411	11.9606	0.0031	0.0175
metab_11950	neg	369.2760	8.1307	1.2580	-1.2211	0.0039	0.0207
metab_12096	neg	369.2761	7.7251	0.1367	0.4770	0.6964	0.7822
metab_7307	neg	369.3014	8.7031	1.1836	-1.4034	0.0004	0.0051
metab_11576	neg	369.3015	9.1038	0.8273	2.6146	0.1129	0.2123
metab_10540	neg	369.3286	9.6986	0.3465	1.0480	0.5845	0.6925
metab_10605	neg	369.3377	10.0432	0.4875	0.9112	0.2323	0.3574
metab_13280	neg	369.9861	3.3304	0.8204	0.9972	0.1514	0.2632
metab_9108	neg	370.0686	3.4133	0.4514	-0.7087	0.2602	0.3898
metab_1446	pos	370.1420	1.0601	0.5240	-0.5243	0.2720	0.4003
metab_1634	pos	370.1593	1.5847	0.6542	1.4677	0.4603	0.5885
metab_5443	pos	370.1609	2.3292	0.0477	-0.2209	0.7734	0.8467
metab_13840	neg	370.1621	2.1311	0.3480	-0.4141	0.3846	0.5135
metab_14058	neg	370.1622	1.7729	0.1781	0.7005	0.7493	0.8208
metab_5150	pos	370.1882	3.1799	0.8348	-2.3022	0.1061	0.1980
metab_1681	pos	370.1963	1.7128	1.5227	5.4891	0.0024	0.0135
metab_1814	pos	370.2148	2.1444	0.5658	-1.7280	0.3687	0.5026
metab_5351	pos	370.2149	2.5314	0.3700	-1.4051	0.4952	0.6190
metab_11962	neg	370.2390	8.0998	0.0964	0.1682	0.7624	0.8293
metab_2386	pos	370.2579	4.8208	0.0317	0.1960	0.9502	0.9679
metab_10177	neg	370.2602	8.2744	0.3154	-1.0763	0.6521	0.7473
metab_10082	neg	370.2602	7.9137	1.1635	-0.8678	0.0134	0.0481
metab_9628	neg	370.2603	5.7751	0.6258	1.4272	0.1781	0.2958
metab_657	pos	370.2943	5.8907	1.3809	2.9700	0.0072	0.0286
metab_10070	neg	370.2966	7.8657	1.6350	-13.7899	0.0032	0.0180
metab_10355	neg	370.2967	8.9086	1.2490	-1.7178	0.0016	0.0115
metab_2270	pos	370.3055	4.0479	1.2661	12.2837	0.0076	0.0298
metab_3036	pos	370.3095	9.3117	1.1363	-1.8283	0.0322	0.0826
metab_10383	neg	370.3239	9.0230	1.1509	-1.6563	0.0045	0.0226
metab_3908	pos	370.3306	9.0112	0.8243	-1.1336	0.0610	0.1317
metab_4023	pos	370.3611	8.5092	0.9209	-0.7843	0.1315	0.2321
metab_3733	pos	370.3672	9.7899	1.3670	-2.1730	0.0022	0.0128
metab_7575	neg	370.9635	2.2595	0.9412	-0.7679	0.1486	0.2597

metab_7841	neg	371.0465	0.5286	1.0363	4.6011	0.0102	0.0403
metab_5504	pos	371.1230	2.1897	0.6577	-1.0294	0.1662	0.2766
metab_14820	neg	371.1369	0.5991	1.4744	4.9831	0.0269	0.0772
metab_11586	neg	371.1535	9.0872	0.0237	0.3305	0.8952	0.9298
metab_10238	neg	371.1536	8.5135	0.9343	1.6156	0.0016	0.0113
metab_4468	pos	371.1717	6.4035	0.3299	0.5352	0.3498	0.4851
metab_13594	neg	371.1825	2.6206	1.5630	2.5101	0.0040	0.0209
metab_13749	neg	371.1825	2.3078	0.5787	1.3064	0.3314	0.4641
metab_5624	pos	371.1911	1.9918	0.8320	1.8317	0.0893	0.1745
metab_1651	pos	371.1914	1.6272	1.0310	3.9775	0.2103	0.3305
metab_8699	neg	371.1938	2.2595	2.0153	8.0807	0.0000	0.0011
metab_9739	neg	371.1974	6.4073	0.0880	0.6469	0.7508	0.8216
metab_13481	neg	371.2081	2.8440	1.3987	2.3502	0.0005	0.0053
metab_12932	neg	371.2081	4.4722	1.0386	1.5502	0.0045	0.0226
metab_9043	neg	371.2081	3.1797	1.5004	2.7963	0.0009	0.0077
metab_9188	neg	371.2186	3.6980	1.8717	3.5864	0.0102	0.0402
metab_565	pos	371.2207	3.8967	1.6074	3.4779	0.0005	0.0049
metab_4783	pos	371.2209	4.6851	1.7957	6.6945	0.0099	0.0358
metab_13620	neg	371.2301	2.5592	0.8160	1.2443	0.1279	0.2321
metab_81	pos	371.2397	4.5642	1.7897	3.8845	0.0030	0.0158
metab_9564	neg	371.2442	5.4355	0.2896	-0.3452	0.6272	0.7269
metab_9687	neg	371.2444	6.1320	0.0283	0.3393	0.8696	0.9104
metab_9594	neg	371.2444	5.6128	0.6452	0.5813	0.2866	0.4180
metab_11790	neg	371.2444	8.6239	0.6253	-0.5572	0.1694	0.2858
metab_13274	neg	371.2446	3.3631	1.9372	6.1356	0.0007	0.0069
metab_12906	neg	371.2553	4.5717	0.0819	-0.7963	0.9491	0.9667
metab_3064	pos	371.2782	9.5283	1.2961	-1.9339	0.0076	0.0296
metab_126	pos	371.2783	6.0107	1.2284	-1.8046	0.0037	0.0179
metab_4325	pos	371.2783	7.0816	1.0831	-1.6074	0.0263	0.0716
metab_10370	neg	371.2807	8.9736	0.5544	0.0077	0.1613	0.2759
metab_2781	pos	371.2892	7.7208	0.5582	0.4225	0.3661	0.4997
metab_7357	neg	371.3171	8.0228	0.1853	0.3618	0.7011	0.7858
metab_10500	neg	371.3171	9.5478	2.1599	8.8355	0.0001	0.0022
metab_10302	neg	371.3171	8.7031	0.5068	-0.4041	0.1621	0.2769
metab_13210	neg	372.0186	3.5641	1.2880	-2.4839	0.0194	0.0616
metab_8545	neg	372.0516	1.9125	1.0304	4.2959	0.0248	0.0731
metab_14720	neg	372.1057	0.7818	0.2168	-0.5331	0.5295	0.6460
metab_6418	pos	372.2345	0.5420	1.3511	13.6484	0.0134	0.0439
metab_13018	neg	372.2396	4.1535	1.9549	6.9037	0.0127	0.0465
metab_4130	pos	372.2524	8.0988	0.1413	0.1937	0.8941	0.9319
metab_10190	neg	372.2549	8.3055	0.7783	-1.8287	0.1219	0.2239
metab_653	pos	372.2735	5.7695	0.1148	0.5306	0.8973	0.9341
metab_5062	pos	372.2735	3.4857	1.4501	2.8978	0.0006	0.0055
metab_2331	pos	372.2736	4.4730	0.2615	-0.0820	0.5026	0.6250
metab_9709	neg	372.2760	6.2775	1.1187	2.1363	0.0439	0.1076
metab_4549	pos	372.3097	5.9361	1.1538	-1.1034	0.0414	0.0992
metab_964	pos	372.3098	8.3912	0.6179	-1.0110	0.2143	0.3354
metab_4441	pos	372.3098	6.5395	0.6346	1.9505	0.1757	0.2889

metab_129	pos	372.3099	6.3885	1.1436	1.9452	0.0186	0.0552
metab_3900	pos	372.3464	9.0397	0.9302	-1.1607	0.1647	0.2747
metab_7576	neg	372.9604	2.2595	0.7753	-0.6516	0.1034	0.1990
metab_7675	neg	373.0594	0.5126	0.9248	-1.1726	0.0672	0.1457
metab_9318	neg	373.1329	4.1699	0.7279	3.5176	0.1960	0.3163
metab_420	pos	373.1386	2.1290	0.5317	-0.7138	0.1502	0.2565
metab_7589	neg	373.1411	2.3533	1.0764	-1.2863	0.0683	0.1473
metab_13926	neg	373.1415	1.9904	0.3461	0.3326	0.6349	0.7330
metab_5555	pos	373.1749	2.1132	1.0267	1.8525	0.0134	0.0438
metab_13714	neg	373.1773	2.3855	1.7716	-2.8994	0.0072	0.0316
metab_3601	pos	373.1847	10.6685	0.3668	-0.2801	0.0048	0.0215
metab_3132	pos	373.1847	9.9307	0.5559	-0.4526	0.0025	0.0137
metab_4835	pos	373.1848	4.4580	0.1783	-0.1634	0.1413	0.2452
metab_9745	neg	373.1939	6.4073	0.1873	0.8598	0.5564	0.6681
metab_13039	neg	373.1987	4.0685	0.4872	0.8620	0.3585	0.4914
metab_1974	pos	373.2332	2.7341	0.3899	1.0172	0.5484	0.6669
metab_5162	pos	373.2363	3.1346	0.0408	-1.3156	0.8579	0.9088
metab_5043	pos	373.2364	3.5620	0.6768	0.4730	0.1990	0.3169
metab_1938	pos	373.2437	2.5782	0.9774	1.2594	0.0744	0.1523
metab_10532	neg	373.2753	9.6644	0.3258	0.7848	0.5255	0.6427
metab_4018	pos	373.2877	8.5393	1.2980	2.4078	0.0140	0.0452
metab_4137	pos	373.2879	8.0700	0.3632	0.5330	0.4733	0.6000
metab_2526	pos	373.3415	5.8148	0.9784	12.1574	0.0236	0.0659
metab_10952	neg	373.9873	14.1904	1.7592	3.3924	0.0006	0.0059
metab_13764	neg	374.0885	2.2754	0.3488	0.3927	0.5776	0.6866
metab_13507	neg	374.1251	2.7961	1.6213	-3.4795	0.0018	0.0122
metab_11589	neg	374.1327	9.0872	0.7867	-0.4349	0.0018	0.0122
metab_8678	neg	374.1453	2.2119	0.4131	-0.0468	0.5018	0.6219
metab_7448	neg	374.1572	1.5439	0.8876	1.4436	0.0531	0.1226
metab_13162	neg	374.1615	3.6980	1.2745	-2.3901	0.0236	0.0705
metab_8910	neg	374.1725	2.7961	0.4428	0.1522	0.3414	0.4745
metab_14073	neg	374.1936	1.7419	0.1267	0.4583	0.7679	0.8337
metab_9515	neg	374.1980	5.1456	1.0478	3.3898	0.1396	0.2482
metab_1821	pos	374.2097	2.1595	1.0901	-1.3744	0.0155	0.0486
metab_13344	neg	374.2189	3.1797	1.5247	8.4406	0.0267	0.0768
metab_9516	neg	374.2453	5.1456	0.3386	0.0241	0.2432	0.3695
metab_2101	pos	374.2526	3.2420	0.4983	1.1935	0.2861	0.4163
metab_9430	neg	374.2554	4.6722	2.1159	6.2272	0.0085	0.0355
metab_9335	neg	374.2555	4.2367	1.6933	4.5543	0.0111	0.0422
metab_10226	neg	374.2708	8.4667	0.0386	-0.4302	0.9415	0.9617
metab_2418	pos	374.2892	5.0451	0.2990	0.1504	0.2094	0.3294
metab_4630	pos	374.2892	5.4662	0.5128	0.2325	0.0617	0.1327
metab_2345	pos	374.2893	4.5485	0.5378	0.2560	0.0390	0.0948
metab_10028	neg	374.2915	7.7100	0.4169	-0.5864	0.3200	0.4522
metab_3641	pos	374.3044	10.2252	2.0149	4.2993	0.0000	0.0006
metab_4175	pos	374.3255	7.9260	0.6072	-1.5024	0.2358	0.3597
metab_2845	pos	374.3256	8.2143	0.1998	-0.7044	0.6495	0.7511
metab_957	pos	374.3408	9.2354	0.8704	-1.1999	0.0166	0.0508

metab_13747	neg	375.0294	2.3078	0.2297	-0.0490	0.5248	0.6422
metab_8963	neg	375.0558	2.9429	0.5855	8.2855	0.4587	0.5814
metab_8962	neg	375.0659	2.9429	0.6183	10.5731	0.4118	0.5386
metab_12893	neg	375.1201	4.6056	1.3322	-10.2380	0.0070	0.0309
metab_13876	neg	375.1210	2.0670	0.0734	0.0446	0.8022	0.8585
metab_14352	neg	375.1211	1.4427	0.2432	0.3028	0.6949	0.7815
metab_15005	neg	375.1266	0.5126	1.1360	-2.5147	0.0126	0.0463
metab_7503	neg	375.1321	1.8205	0.8257	-0.9198	0.0405	0.1017
metab_8236	neg	375.1413	1.3817	0.5139	-0.7832	0.2361	0.3616
metab_7495	neg	375.1413	1.8046	0.0523	0.2580	0.8981	0.9320
metab_13387	neg	375.1484	3.0604	1.3436	4.0071	0.0235	0.0705
metab_8751	neg	375.1486	2.3855	0.8914	2.8158	0.1133	0.2126
metab_554	pos	375.1544	2.2519	0.4111	-0.5305	0.2982	0.4293
metab_5623	pos	375.1544	1.9918	0.4455	0.5433	0.1549	0.2622
metab_6933	neg	375.1568	3.8678	0.1790	0.3585	0.7169	0.7968
metab_9127	neg	375.1820	3.4969	1.0642	0.0865	0.3467	0.4797
metab_6971	neg	375.1850	4.8865	0.5843	1.0789	0.1797	0.2976
metab_5419	pos	375.1906	2.3908	1.5840	-2.6340	0.0245	0.0677
metab_12660	neg	375.1944	5.6615	0.4782	-0.0669	0.1024	0.1974
metab_7407	neg	375.1945	6.2456	1.7650	-3.0334	0.0009	0.0081
metab_9439	neg	375.2212	4.7217	0.3024	-0.0636	0.6002	0.7051
metab_2896	pos	375.3036	8.4809	0.3272	0.0860	0.2424	0.3674
metab_4128	pos	375.3036	8.1132	0.4740	0.2673	0.0721	0.1493
metab_14543	neg	376.0492	1.1796	1.5161	-1.2573	0.0616	0.1370
metab_8007	neg	376.0667	0.8662	0.2546	0.6000	0.8478	0.8943
metab_7878	neg	376.1015	0.5571	0.7167	-1.6308	0.3486	0.4814
metab_14555	neg	376.1183	1.1355	1.0204	1.9916	0.0177	0.0580
metab_5263	pos	376.1382	2.8108	1.6748	-4.2695	0.0019	0.0115
metab_398	pos	376.1702	1.9463	0.1131	0.3583	0.8828	0.9255
metab_5770	pos	376.1703	1.6693	0.2866	0.4767	0.6792	0.7748
metab_5943	pos	376.1736	1.3579	2.4935	11.0940	0.0088	0.0330
metab_5003	pos	376.1746	3.7148	1.6062	-3.0914	0.0154	0.0484
metab_12996	neg	376.1771	4.2203	1.6128	-2.1879	0.0021	0.0136
metab_9129	neg	376.1880	3.5141	1.0349	2.6789	0.0814	0.1674
metab_6527	pos	376.1920	0.4855	0.3913	-0.5127	0.4027	0.5361
metab_12921	neg	376.2096	4.5219	1.4441	3.8227	0.0191	0.0610
metab_4769	pos	376.2108	4.7758	0.1813	1.2699	0.8183	0.8809
metab_2342	pos	376.2108	4.5335	0.2325	0.6861	0.7584	0.8349
metab_1450	pos	376.2180	1.0741	1.4663	14.2012	0.0040	0.0187
metab_5605	pos	376.2220	2.0211	0.4501	-0.8435	0.3070	0.4392
metab_13330	neg	376.2246	3.2130	0.6621	1.0631	0.1164	0.2164
metab_9000	neg	376.2248	3.0260	0.1676	0.0115	0.7545	0.8233
metab_2047	pos	376.2320	3.0114	1.3080	1.9182	0.0088	0.0329
metab_9704	neg	376.2343	6.2456	0.3019	0.8099	0.4546	0.5779
metab_12483	neg	376.2343	6.4073	0.3170	0.7457	0.3261	0.4583
metab_2434	pos	376.2584	5.1533	0.0253	-0.0301	0.9377	0.9612
metab_11728	neg	376.2630	8.7031	1.2352	6.5696	0.0349	0.0916
metab_1947	pos	376.2686	2.6090	0.0729	0.8631	0.9824	0.9891

metab_4414	pos	376.2837	6.7366	1.4621	-1.2985	0.0148	0.0472
metab_2677	pos	376.2837	7.0082	0.9444	-0.9883	0.0531	0.1194
metab_11723	neg	376.2859	8.7031	1.7402	-4.0905	0.0000	0.0005
metab_10207	neg	376.2862	8.3858	1.5545	-2.3605	0.0037	0.0197
metab_4667	pos	376.3048	5.2717	0.4788	-1.0311	0.2149	0.3362
metab_4727	pos	376.3048	4.9413	0.3541	-0.8354	0.3695	0.5033
metab_741	pos	376.3051	7.7062	0.1519	-0.2817	0.6772	0.7731
metab_7807	neg	376.9108	0.5126	0.6660	-0.7173	0.0573	0.1298
metab_14430	neg	377.0667	1.3231	0.8584	1.3578	0.0311	0.0849
metab_8934	neg	377.0708	2.8440	0.4438	0.8181	0.1104	0.2087
metab_6600	neg	377.0859	0.5851	0.7098	1.8312	0.0427	0.1056
metab_11172	neg	377.0860	11.2769	0.4047	0.5179	0.0326	0.0877
metab_10742	neg	377.0860	13.9952	0.4015	0.5426	0.0322	0.0869
metab_11578	neg	377.0862	9.1038	0.0303	0.5725	0.7379	0.8110
metab_11700	neg	377.0863	8.7346	0.4562	0.6106	0.0752	0.1575
metab_13727	neg	377.0991	2.3533	0.3380	0.5767	0.5232	0.6408
metab_1786	pos	377.1336	2.0677	0.4058	-0.4993	0.4814	0.6070
metab_1891	pos	377.1348	2.3908	0.5823	-1.2835	0.0959	0.1838
metab_8604	neg	377.1360	2.0358	0.8231	-0.8676	0.0185	0.0596
metab_11889	neg	377.1434	8.3055	1.4363	-1.5301	0.0007	0.0068
metab_5693	pos	377.1444	1.8134	0.7378	-1.1699	0.0706	0.1471
metab_8370	neg	377.1470	1.5736	0.6248	6.8063	0.5145	0.6331
metab_1882	pos	377.1614	2.3751	0.1683	0.5463	0.7730	0.8467
metab_9350	neg	377.1642	4.3207	1.4626	2.6207	0.0044	0.0224
metab_11745	neg	377.1643	8.7031	1.0907	-1.0130	0.0000	0.0005
metab_5468	pos	377.1697	2.2519	0.7664	-1.0015	0.0358	0.0890
metab_9302	neg	377.1725	4.1023	1.3212	-1.4191	0.0063	0.0287
metab_7432	neg	377.1726	3.6812	0.0522	0.2837	0.7227	0.8011
metab_2529	pos	377.1850	5.8597	1.5066	-7.0631	0.0082	0.0314
metab_8562	neg	377.1934	1.9439	0.3905	0.8003	0.6476	0.7437
metab_9707	neg	377.1998	6.2617	1.7497	-2.9340	0.0002	0.0035
metab_4988	pos	377.2048	3.7599	0.4372	-0.2466	0.2719	0.4003
metab_2172	pos	377.2049	3.5620	0.1907	0.1592	0.5699	0.6854
metab_9453	neg	377.2081	4.7709	0.4802	-0.6742	0.2115	0.3341
metab_9296	neg	377.2084	4.0859	1.7636	4.7838	0.0004	0.0051
metab_12525	neg	377.2099	6.2129	1.0797	-1.2811	0.0181	0.0590
metab_7016	neg	377.2103	6.7122	1.1247	-1.0391	0.0005	0.0054
metab_9750	neg	377.2105	6.4553	1.3373	-2.5594	0.0470	0.1127
metab_4463	pos	377.2289	6.4035	1.0630	2.0050	0.0211	0.0605
metab_4527	pos	377.3153	6.0252	0.4827	0.8916	0.4103	0.5432
metab_808	pos	377.3194	8.7303	0.1378	-0.0917	0.6967	0.7884
metab_4070	pos	377.3194	8.3322	0.0918	-0.1650	0.7495	0.8288
metab_1270	pos	377.9744	0.5420	0.1677	-0.3108	0.7402	0.8216
metab_13698	neg	378.0503	2.4010	1.0224	-1.1110	0.0277	0.0787
metab_1489	pos	378.0622	1.1731	0.6526	-0.8329	0.2071	0.3269
metab_8828	neg	378.1128	2.5748	0.8074	5.5646	0.2540	0.3825
metab_8394	neg	378.1205	1.6028	0.5657	1.1378	0.4116	0.5385
metab_1470	pos	378.1316	1.1306	2.0030	4.5125	0.0015	0.0096

metab_14521	neg	378.1340	1.2236	1.4284	3.3060	0.0004	0.0047
metab_8735	neg	378.1674	2.3380	0.0981	0.1524	0.8570	0.9011
metab_13161	neg	378.1883	3.6980	0.3204	0.6725	0.6087	0.7123
metab_13028	neg	378.1922	4.1023	0.4577	-0.0440	0.3982	0.5260
metab_14967	neg	378.2250	0.5286	1.0396	-1.0886	0.1229	0.2253
metab_5140	pos	378.2377	3.2116	0.8290	1.2286	0.0674	0.1421
metab_2051	pos	378.2378	3.0264	0.3520	0.0258	0.4438	0.5743
metab_9829	neg	378.2504	6.8887	0.8975	1.4623	0.0421	0.1046
metab_541	pos	378.2630	3.5162	1.3254	3.1972	0.0103	0.0367
metab_9749	neg	378.2648	6.4396	1.2776	3.9743	0.0510	0.1190
metab_12262	neg	378.2649	7.1595	1.6845	0.0278	0.0586	0.1320
metab_988	pos	378.2993	6.9477	0.7194	-0.7183	0.1462	0.2513
metab_7086	neg	378.3018	8.4972	1.0137	-1.0712	0.0119	0.0443
metab_11766	neg	378.3019	8.6874	1.3422	-1.6792	0.0016	0.0112
metab_15036	neg	378.9080	0.4975	0.7672	-0.8378	0.0349	0.0914
metab_7195	neg	378.9188	14.0282	0.1741	-0.0169	0.4331	0.5573
metab_3443	pos	379.0126	14.1820	0.4312	-0.3012	0.0217	0.0619
metab_14842	neg	379.0827	0.5991	0.7939	2.1598	0.0357	0.0929
metab_10705	neg	379.0828	12.7209	0.4307	0.5378	0.0182	0.0591
metab_10483	neg	379.0828	9.4485	0.1628	0.8411	0.5097	0.6290
metab_10565	neg	379.0829	9.8459	0.4506	0.9664	0.1348	0.2417
metab_8091	neg	379.1151	1.1069	2.1797	-3.6199	0.0015	0.0108
metab_14002	neg	379.1152	1.8524	0.1468	0.7744	0.6384	0.7360
metab_8443	neg	379.1153	1.7104	0.4869	-0.0425	0.3335	0.4664
metab_6815	neg	379.1362	0.9792	1.7717	3.6736	0.0000	0.0001
metab_13571	neg	379.1516	2.6678	0.1516	-0.0133	0.6668	0.7598
metab_8961	neg	379.1516	2.9429	0.6398	-0.8999	0.1562	0.2695
metab_11751	neg	379.1588	8.7031	1.0808	-0.9772	0.0000	0.0005
metab_6200	pos	379.1701	0.8780	1.5954	-2.6551	0.0013	0.0089
metab_8228	neg	379.1727	1.3669	1.0530	-0.7832	0.1891	0.3083
metab_13241	neg	379.1761	3.4800	1.0571	2.9763	0.0861	0.1742
metab_592	pos	379.1775	4.3216	1.7735	3.2622	0.0026	0.0142
metab_11555	neg	379.1791	9.1200	0.5528	-0.0506	0.1308	0.2360
metab_5006	pos	379.1853	3.6987	1.5186	-2.1126	0.0038	0.0181
metab_5103	pos	379.1933	3.3183	0.4738	2.3500	0.6135	0.7222
metab_1742	pos	379.2063	1.9463	0.2689	-1.0361	0.6559	0.7557
metab_12511	neg	379.2109	6.2943	0.1862	-1.4323	0.7803	0.8435
metab_12695	neg	379.2111	5.4844	0.1881	-0.4545	0.6680	0.7605
metab_12555	neg	379.2125	6.0993	0.0065	-0.7666	0.9620	0.9740
metab_8859	neg	379.2131	2.6678	2.3811	9.9146	0.0000	0.0005
metab_6612	neg	379.2132	3.2972	2.5486	6.9199	0.0007	0.0066
metab_4208	pos	379.2234	7.7062	0.7890	1.3068	0.2035	0.3226
metab_12409	neg	379.2605	6.7122	1.0843	-2.9372	0.1147	0.2141
metab_7049	neg	379.2760	7.4776	0.7060	-0.5417	0.0093	0.0380
metab_4523	pos	379.3219	6.0252	0.6983	1.4733	0.3000	0.4312
metab_11430	neg	379.3222	9.5811	0.0730	0.2572	0.7614	0.8287
metab_2875	pos	379.3257	8.3463	0.3455	0.3137	0.4868	0.6119
metab_2585	pos	379.3310	6.3885	1.5467	15.8639	0.0179	0.0537

metab_4016	pos	379.3353	8.5554	1.3347	-2.4924	0.0123	0.0414
metab_3804	pos	379.3355	9.4981	0.9856	-1.3926	0.0008	0.0066
metab_13837	neg	380.0080	2.1311	0.2459	-0.1115	0.5906	0.6978
metab_8760	neg	380.0082	2.4010	0.4814	-0.2351	0.2326	0.3578
metab_14080	neg	380.0994	1.7419	0.9676	-1.3705	0.0447	0.1091
metab_13939	neg	380.0994	1.9745	1.0152	-1.4164	0.0406	0.1018
metab_6830	neg	380.1101	0.9368	1.5409	2.4962	0.0288	0.0808
metab_292	pos	380.1473	1.2162	1.3333	3.0386	0.0019	0.0116
metab_14328	neg	380.1474	1.4568	0.7293	-0.2097	0.0491	0.1162
metab_13667	neg	380.1481	2.4651	0.1578	-0.3549	0.7860	0.8471
metab_8330	neg	380.1562	1.5297	1.2985	-1.6070	0.0007	0.0066
metab_14435	neg	380.1565	1.3089	1.2513	-1.8990	0.0058	0.0272
metab_4784	pos	380.1636	4.6700	1.5845	-2.5382	0.0055	0.0235
metab_5323	pos	380.1806	2.6090	0.1355	0.8604	0.8791	0.9230
metab_11788	neg	380.1900	8.6239	0.3274	-0.1270	0.2880	0.4195
metab_9557	neg	380.2446	5.3877	0.7130	2.0884	0.2208	0.3448
metab_9701	neg	380.2448	6.2129	0.6503	-0.0882	0.2543	0.3826
metab_12639	neg	380.2449	5.7259	0.5388	1.4468	0.3842	0.5132
metab_9813	neg	380.2450	6.8093	0.6021	0.9922	0.2383	0.3642
metab_10318	neg	380.2580	8.7191	0.3368	-0.3199	0.5052	0.6248
metab_83	pos	380.2782	6.4192	0.5737	1.7139	0.2081	0.3279
metab_4987	pos	380.2785	3.7599	1.6822	-2.3481	0.0036	0.0177
metab_12343	neg	380.2810	6.9191	1.2531	3.9708	0.0422	0.1049
metab_7358	neg	380.2811	8.0373	1.2713	-1.3895	0.0000	0.0005
metab_11747	neg	380.2811	8.7031	1.4427	-1.8103	0.0000	0.0005
metab_7404	neg	380.2811	6.4073	0.3452	2.0930	0.4296	0.5545
metab_10231	neg	380.3082	8.4972	1.0848	-1.2594	0.0097	0.0390
metab_4182	pos	380.3148	7.8830	1.0610	2.8888	0.1450	0.2500
metab_665	pos	380.3148	6.0252	1.2513	-0.8123	0.0559	0.1239
metab_2898	pos	380.3148	8.4949	0.8696	-1.1719	0.0396	0.0957
metab_7105	neg	380.3176	8.9086	0.6945	-0.4990	0.1729	0.2895
metab_6470	pos	381.0171	0.5140	1.3702	3.2705	0.0003	0.0032
metab_8294	neg	381.0437	1.4710	0.7311	1.5114	0.1825	0.3006
metab_3	pos	381.0783	0.5843	0.1967	0.1575	0.3539	0.4886
metab_13505	neg	381.1198	2.7961	0.9522	-3.6314	0.0759	0.1587
metab_8544	neg	381.1307	1.9125	0.3576	-0.0812	0.4195	0.5456
metab_7555	neg	381.1308	2.1311	0.7133	-0.7205	0.1602	0.2745
metab_8359	neg	381.1309	1.5586	0.0445	0.3739	0.7922	0.8511
metab_14064	neg	381.1310	1.7572	0.0639	0.2978	0.8151	0.8685
metab_6846	neg	381.1311	1.1940	0.4517	-0.3948	0.1478	0.2587
metab_5223	pos	381.1647	2.9342	0.9473	-1.3987	0.1543	0.2615
metab_13990	neg	381.1672	1.8682	0.5660	1.7204	0.1862	0.3050
metab_13250	neg	381.1688	3.4470	0.7869	2.1833	0.4466	0.5701
metab_10418	neg	381.1745	9.1200	0.7308	-0.2639	0.0258	0.0750
metab_7564	neg	381.1773	2.1798	2.0114	5.3904	0.0004	0.0048
metab_6979	neg	381.1824	5.0967	0.0623	1.8411	0.7714	0.8368
metab_12225	neg	381.2054	7.2865	1.0035	-0.9450	0.0432	0.1064
metab_7405	neg	381.2262	6.4073	0.2356	0.6759	0.4069	0.5344

metab_5108	pos	381.2265	3.3032	1.4599	3.6625	0.0313	0.0809
metab_2061	pos	381.2267	3.0877	1.3896	3.0652	0.0259	0.0708
metab_12785	neg	381.2287	5.0002	1.4431	4.4702	0.0052	0.0253
metab_12939	neg	381.2288	4.4221	2.0857	6.2888	0.0007	0.0069
metab_13425	neg	381.2289	2.9931	2.3524	9.6395	0.0000	0.0005
metab_6611	neg	381.2289	3.3304	2.5373	6.6819	0.0010	0.0086
metab_10009	neg	381.2324	7.6323	0.5528	0.1341	0.2381	0.3640
metab_4935	pos	381.2414	4.0028	1.4956	3.4597	0.0121	0.0410
metab_7094	neg	381.2648	8.7031	1.4532	-2.4011	0.0011	0.0092
metab_12858	neg	381.2652	4.7384	0.4852	-0.1373	0.2930	0.4243
metab_10219	neg	381.2653	8.4336	1.8437	-2.9597	0.0002	0.0031
metab_9972	neg	381.2763	7.4927	1.0498	-0.2056	0.0700	0.1499
metab_2751	pos	381.2887	7.4703	0.8432	-0.8804	0.0029	0.0151
metab_897	pos	381.2890	11.0985	0.2842	-0.2189	0.0480	0.1104
metab_2498	pos	381.3102	5.6473	1.6991	5.0889	0.0003	0.0036
metab_3766	pos	381.3142	9.6671	1.1729	2.1621	0.0239	0.0667
metab_2809	pos	381.3143	7.9695	0.0608	-0.1363	0.9818	0.9889
metab_11347	neg	381.3179	9.8620	0.0756	0.3559	0.9327	0.9561
metab_11636	neg	381.3378	8.9417	0.4378	1.2410	0.2060	0.3279
metab_6637	neg	381.3379	9.8620	0.1265	0.1693	0.5697	0.6801
metab_6856	neg	382.1009	1.2663	1.0850	-1.6308	0.0071	0.0313
metab_6172	pos	382.1234	0.9200	0.8647	1.6442	0.2285	0.3520
metab_6174	pos	382.1420	0.9200	1.6752	-4.0471	0.0004	0.0042
metab_13850	neg	382.1623	2.1153	0.2836	0.7067	0.6920	0.7791
metab_14541	neg	382.1721	1.1796	0.8608	2.0825	0.0553	0.1265
metab_13419	neg	382.1776	2.9931	0.8633	1.5162	0.0618	0.1373
metab_4006	pos	382.2028	8.5989	0.0218	-0.1613	0.9655	0.9788
metab_5022	pos	382.2065	3.6384	0.6948	-0.4686	0.4130	0.5454
metab_9473	neg	382.2235	4.8865	0.4246	0.7992	0.5079	0.6274
metab_6956	neg	382.2239	4.4551	1.9480	2.7946	0.0023	0.0145
metab_5104	pos	382.2577	3.3183	0.0201	-0.4844	0.9690	0.9811
metab_608	pos	382.2578	4.0634	1.6861	2.5764	0.0007	0.0061
metab_2506	pos	382.2578	5.6934	1.0853	2.0941	0.0441	0.1038
metab_2207	pos	382.2578	3.7148	0.1265	-0.6834	0.8858	0.9276
metab_4640	pos	382.2578	5.3913	0.8187	2.1934	0.0906	0.1765
metab_12534	neg	382.2601	6.1968	1.1763	-1.0784	0.0659	0.1436
metab_9647	neg	382.2602	5.9217	0.5308	-0.2202	0.4111	0.5384
metab_12403	neg	382.2602	6.7439	1.7852	-2.6875	0.0013	0.0100
metab_12925	neg	382.2603	4.5054	1.5578	11.6223	0.0022	0.0138
metab_12690	neg	382.2603	5.5164	0.8535	0.0717	0.2840	0.4149
metab_9344	neg	382.2604	4.2870	2.0459	7.1923	0.0007	0.0067
metab_4424	pos	382.2940	6.6295	0.2354	2.4443	0.6225	0.7293
metab_4362	pos	382.2941	6.9638	0.9199	2.3678	0.1025	0.1932
metab_9779	neg	382.2967	6.6320	0.9724	12.7504	0.0680	0.1467
metab_10414	neg	382.2967	9.1038	0.9081	-0.8431	0.0729	0.1540
metab_12351	neg	382.2971	6.8887	0.2963	2.0797	0.5344	0.6501
metab_2968	pos	382.3304	8.9060	0.3235	-0.2995	0.5460	0.6649
metab_2546	pos	382.3306	5.9800	1.5546	-2.2234	0.0098	0.0354

metab_11495	neg	382.3331	9.3329	1.1310	-1.6252	0.0097	0.0391
metab_3648	pos	382.4396	10.1793	0.0416	-0.0982	0.7162	0.8028
metab_3705	pos	382.4398	9.8836	0.4602	-0.3547	0.0121	0.0409
metab_7980	neg	383.1140	0.7818	1.2227	-1.5881	0.0432	0.1065
metab_9104	neg	383.1255	3.3971	1.1390	-3.1560	0.0634	0.1400
metab_7684	neg	383.1360	3.1115	0.6466	1.0888	0.0870	0.1756
metab_7443	neg	383.1466	1.5297	0.8627	-0.9006	0.1570	0.2706
metab_9259	neg	383.1618	3.9349	1.0015	-0.8475	0.0398	0.1005
metab_10427	neg	383.1810	9.1200	0.6597	-0.1200	0.0584	0.1317
metab_10501	neg	383.1901	9.5478	0.9567	-0.5157	0.0063	0.0286
metab_13379	neg	383.2083	3.0942	2.2341	5.5569	0.0008	0.0076
metab_4915	pos	383.2144	4.0788	0.9418	-1.7618	0.0822	0.1640
metab_7025	neg	383.2419	6.8887	0.7653	1.1881	0.0084	0.0351
metab_9666	neg	383.2422	6.0024	0.0629	1.8946	0.8685	0.9097
metab_13303	neg	383.2445	3.2972	2.0209	8.0899	0.0001	0.0018
metab_7686	neg	383.2446	3.1115	2.2285	7.2768	0.0001	0.0018
metab_13169	neg	383.2446	3.6812	2.1936	5.8888	0.0000	0.0003
metab_9118	neg	383.2446	3.4800	2.2914	9.0765	0.0000	0.0003
metab_4788	pos	383.2683	4.6544	1.3950	1.9541	0.0012	0.0083
metab_2421	pos	383.2684	5.0610	1.5756	2.5180	0.0000	0.0003
metab_7344	neg	383.2919	8.1153	0.9914	-0.7285	0.0079	0.0338
metab_10465	neg	383.3172	9.3329	2.4408	7.2638	0.0003	0.0037
metab_11926	neg	383.3281	8.1936	1.0784	-0.6042	0.0415	0.1035
metab_11350	neg	383.3443	9.8620	0.1367	0.1676	0.5553	0.6671
metab_11265	neg	383.3535	10.1910	0.0017	0.3906	0.9501	0.9674
metab_2312	pos	383.7159	4.3672	1.1159	-3.4142	0.1455	0.2506
metab_13639	neg	384.0305	2.5264	2.0172	-5.0774	0.0000	0.0003
metab_298	pos	384.1139	1.2446	0.9717	-1.6815	0.0090	0.0336
metab_13823	neg	384.1309	2.1630	1.3993	-1.7281	0.0000	0.0005
metab_8450	neg	384.1424	1.7264	1.1729	1.7117	0.0218	0.0669
metab_391	pos	384.1545	1.8729	1.3492	-2.0545	0.0915	0.1779
metab_12729	neg	384.1604	5.2899	0.4992	-0.2926	0.6121	0.7153
metab_418	pos	384.1754	2.1290	0.2687	0.0993	0.6848	0.7790
metab_8957	neg	384.1797	2.9092	0.4195	1.3736	0.5707	0.6811
metab_1487	pos	384.1854	1.1731	0.2749	0.0164	0.3799	0.5123
metab_1416	pos	384.1855	0.9480	1.0652	-1.7586	0.0249	0.0687
metab_4842	pos	384.2733	4.4272	1.7396	2.6364	0.0006	0.0055
metab_4623	pos	384.2734	5.5112	0.8437	-0.0964	0.1888	0.3041
metab_12481	neg	384.2756	6.4073	0.6210	0.0497	0.2136	0.3364
metab_9654	neg	384.2760	5.9542	0.0541	0.8151	0.9156	0.9440
metab_9578	neg	384.2760	5.5484	0.0678	1.2473	0.9415	0.9617
metab_11908	neg	384.2761	8.2577	1.4902	-1.7590	0.0000	0.0012
metab_2900	pos	384.3097	8.4949	0.0167	0.2009	0.9801	0.9882
metab_3954	pos	384.3103	8.7731	0.4901	1.0505	0.4352	0.5664
metab_11615	neg	384.3124	9.0067	1.3621	-1.8538	0.0040	0.0210
metab_3949	pos	384.3248	8.8169	1.8163	-3.2203	0.0004	0.0040
metab_3061	pos	384.3251	9.5137	1.2503	-2.0396	0.1344	0.2361
metab_4499	pos	384.3464	6.2071	0.9719	-0.2760	0.1128	0.2068

metab_7793	neg	384.9243	0.4975	0.6956	-0.7843	0.0665	0.1444
metab_7157	neg	384.9359	14.0282	0.3241	-0.0803	0.2621	0.3919
metab_1148	pos	385.0398	0.5560	1.0229	-1.7475	0.0108	0.0378
metab_6595	neg	385.0905	1.5155	1.9677	-3.6842	0.0022	0.0137
metab_6267	pos	385.1275	0.7801	0.9334	-1.6340	0.0816	0.1632
metab_7988	neg	385.1382	0.7958	1.6386	3.5773	0.0033	0.0182
metab_4953	pos	385.1749	3.9270	1.2389	-1.3850	0.0271	0.0731
metab_13315	neg	385.2239	3.2637	2.1207	4.7917	0.0022	0.0141
metab_9303	neg	385.2342	4.1023	1.0660	2.9814	0.0280	0.0794
metab_2274	pos	385.2481	4.0634	1.9581	4.4678	0.0000	0.0003
metab_9791	neg	385.2602	6.7278	0.4634	1.7742	0.3440	0.4771
metab_10305	neg	385.2602	8.7031	0.9812	-0.3564	0.0881	0.1772
metab_12452	neg	385.2604	6.5033	0.0423	0.9267	0.9622	0.9741
metab_10369	neg	385.2966	8.9736	1.9196	-2.6825	0.0005	0.0056
metab_11952	neg	385.2983	8.1153	1.2238	-1.1175	0.0028	0.0163
metab_2823	pos	385.3050	8.0988	1.0743	-1.2757	0.0360	0.0892
metab_11386	neg	385.3328	9.7312	1.6888	15.0087	0.0056	0.0265
metab_11906	neg	385.3329	8.2744	0.9438	1.3005	0.0718	0.1524
metab_4108	pos	385.3414	8.1857	1.1351	-0.7785	0.0716	0.1486
metab_11263	neg	385.3600	10.1910	0.1624	0.5806	0.6583	0.7526
metab_1306	pos	386.0246	0.5983	0.5641	-0.4383	0.0014	0.0093
metab_8423	neg	386.1028	1.6642	0.7546	1.7738	0.2316	0.3569
metab_13796	neg	386.1100	2.2119	0.3950	2.3951	0.4698	0.5921
metab_14595	neg	386.1209	1.0215	0.5113	-0.0105	0.2777	0.4082
metab_14458	neg	386.1464	1.2805	1.9919	-3.7496	0.0033	0.0182
metab_5640	pos	386.1524	1.9609	1.2316	-2.2053	0.0032	0.0163
metab_15023	neg	386.1702	0.4975	0.2950	-0.7435	0.7238	0.8019
metab_13870	neg	386.1760	2.0670	0.4699	-0.9704	0.4019	0.5301
metab_6087	pos	386.1907	1.1306	0.2923	-0.4180	0.5844	0.6979
metab_1853	pos	386.2096	2.2665	0.7300	1.2598	0.0571	0.1257
metab_1916	pos	386.2096	2.4850	0.5571	0.7072	0.1052	0.1968
metab_12891	neg	386.2553	4.6216	0.1632	1.4847	0.8539	0.8986
metab_12984	neg	386.2554	4.2702	0.2626	0.6430	0.7612	0.8286
metab_4072	pos	386.2679	8.3166	1.1367	1.9282	0.0278	0.0747
metab_10306	neg	386.2740	8.7191	1.6701	-3.3023	0.0004	0.0049
metab_661	pos	386.2891	5.9504	0.0555	0.8331	0.9631	0.9779
metab_2489	pos	386.2892	5.5715	0.2231	0.8995	0.7776	0.8499
metab_12468	neg	386.2912	6.4396	0.3793	1.3677	0.5181	0.6365
metab_10139	neg	386.2916	8.1153	0.8614	-1.7231	0.0904	0.1805
metab_781	pos	386.3255	8.4809	0.0315	-0.2215	0.9494	0.9675
metab_3019	pos	386.3408	9.1898	1.4841	-2.0590	0.0038	0.0182
metab_3065	pos	386.3619	9.5438	0.8793	-1.9446	0.1435	0.2479
metab_14901	neg	386.8106	0.5431	1.2760	-1.6135	0.0011	0.0093
metab_10775	neg	386.8629	14.0282	0.2421	0.0519	0.2347	0.3601
metab_7902	neg	386.9122	0.5851	0.2145	-0.0094	0.7130	0.7942
metab_7681	neg	386.9399	0.5126	0.8343	-1.1941	0.0446	0.1089
metab_7551	neg	387.1149	0.5711	0.0348	0.1728	0.8928	0.9282
metab_9079	neg	387.1484	3.2972	1.0147	4.8646	0.1798	0.2977

metab_13445	neg	387.1563	2.9429	0.6781	-1.2669	0.2365	0.3622
metab_15032	neg	387.1739	0.4975	1.0947	2.3483	0.0123	0.0456
metab_1633	pos	387.1751	1.5705	0.9544	-2.7231	0.0682	0.1433
metab_11	pos	387.1792	5.1830	0.3366	-0.2893	0.0950	0.1824
metab_8317	neg	387.1889	1.5155	1.6328	2.9889	0.0002	0.0036
metab_12724	neg	387.1918	5.3388	0.2512	-0.4267	0.6702	0.7621
metab_9981	neg	387.2216	7.5239	0.5215	-0.8724	0.2295	0.3547
metab_5900	pos	387.2228	1.4429	1.5219	14.0456	0.0028	0.0150
metab_13185	neg	387.2396	3.6311	1.7528	5.5095	0.0006	0.0063
metab_13343	neg	387.2397	3.1797	1.7564	7.3279	0.0010	0.0086
metab_12352	neg	387.2752	6.8887	0.0645	0.6612	0.8882	0.9251
metab_9909	neg	387.2758	7.1749	0.7960	2.4371	0.1515	0.2632
metab_7414	neg	387.2860	6.0993	0.6244	3.0612	0.4157	0.5419
metab_2189	pos	387.2997	3.6384	1.4613	11.9413	0.0160	0.0495
metab_7959	neg	387.3171	0.6973	0.1545	0.2248	0.9596	0.9727
metab_7903	neg	387.3199	0.5851	0.1176	0.1248	0.9196	0.9471
metab_14710	neg	388.0656	0.7818	1.3790	-2.9296	0.0018	0.0125
metab_6055	pos	388.1593	1.1875	2.0553	-3.8046	0.0148	0.0470
metab_14434	neg	388.1729	1.3089	1.6460	4.0037	0.0008	0.0073
metab_8539	neg	388.1729	1.8974	0.4983	-0.2531	0.3535	0.4864
metab_8430	neg	388.1731	1.6797	0.4933	-0.1168	0.2611	0.3908
metab_9070	neg	388.1883	3.2801	0.4808	-0.1012	0.2377	0.3636
metab_13257	neg	388.1884	3.4303	0.5932	0.0638	0.3301	0.4629
metab_9221	neg	388.1884	3.8001	0.2827	1.3150	0.7369	0.8104
metab_411	pos	388.1887	2.0677	0.1872	-0.6094	0.5420	0.6619
metab_5580	pos	388.2220	2.0677	0.8232	-1.1006	0.0857	0.1693
metab_1255	pos	388.2309	0.5280	1.2020	1.9951	0.0011	0.0081
metab_5194	pos	388.2685	3.0114	1.7589	3.9148	0.0001	0.0014
metab_2358	pos	388.2686	4.6096	1.6831	2.2902	0.0035	0.0174
metab_1953	pos	388.2687	2.6402	2.1735	5.9401	0.0004	0.0045
metab_10002	neg	388.2710	7.6169	0.5166	-0.3420	0.2982	0.4287
metab_12900	neg	388.2714	4.5884	0.9797	3.3071	0.1959	0.3162
metab_4075	pos	388.2822	8.3018	0.0029	0.3164	0.9346	0.9587
metab_3977	pos	388.2829	8.6717	1.3534	-1.6839	0.0018	0.0109
metab_11761	neg	388.2867	8.6874	1.3340	-1.7692	0.0007	0.0068
metab_2696	pos	388.3047	7.0816	1.8618	-3.1292	0.0010	0.0073
metab_676	pos	388.3047	6.4339	0.2558	0.9798	0.6667	0.7647
metab_5204	pos	388.3047	2.9806	2.1349	13.2620	0.0000	0.0001
metab_12098	neg	388.3074	7.7251	1.0250	-1.5997	0.0603	0.1348
metab_6628	neg	388.3074	7.9612	0.9867	-1.3136	0.0845	0.1716
metab_750	pos	388.3410	7.9980	0.8434	-1.0144	0.2169	0.3386
metab_3878	pos	388.3562	9.1302	0.0455	-0.0378	0.7270	0.8118
metab_2417	pos	388.7149	5.0307	0.0559	0.2725	0.9412	0.9632
metab_11039	neg	388.8743	14.0438	0.5065	-0.0987	0.0157	0.0536
metab_8860	neg	388.9299	2.6678	0.3983	-0.4587	0.4067	0.5343
metab_13500	neg	388.9299	2.8123	0.2407	-0.2033	0.5593	0.6705
metab_13805	neg	389.1355	2.1956	0.7916	-1.1645	0.1795	0.2975
metab_7463	neg	389.1357	1.5155	1.0973	-1.1364	0.0000	0.0006

metab_8207	neg	389.1569	1.3231	0.8037	-0.8060	0.0365	0.0945
metab_13431	neg	389.1630	2.9760	1.1411	2.3215	0.0822	0.1683
metab_12964	neg	389.1642	4.3207	1.6424	-1.3639	0.0025	0.0152
metab_2023	pos	389.1692	2.9342	0.5123	-1.0278	0.2424	0.3674
metab_1624	pos	389.1695	1.5424	1.2695	13.7874	0.0206	0.0594
metab_5372	pos	389.1698	2.4850	0.8515	-1.5658	0.0938	0.1810
metab_5304	pos	389.1698	2.6869	0.6289	-1.0975	0.3055	0.4373
metab_12854	neg	389.1745	4.7546	2.1400	-4.7180	0.0000	0.0002
metab_5317	pos	389.1847	2.6244	0.5462	-2.2649	0.5270	0.6480
metab_2440	pos	389.1852	5.1830	0.2380	-0.2555	0.2427	0.3675
metab_2727	pos	389.2828	7.2916	0.8090	1.5857	0.1682	0.2789
metab_7792	neg	389.8871	0.4975	0.5491	-0.3983	0.0741	0.1558
metab_6901	neg	390.1136	1.4568	0.8394	-1.5683	0.0418	0.1042
metab_14628	neg	390.1174	0.9649	0.8651	-1.7764	0.0864	0.1747
metab_8513	neg	390.1204	1.8371	1.6045	-3.0420	0.0002	0.0030
metab_14868	neg	390.1221	0.5711	0.0434	0.2485	0.7260	0.8027
metab_13810	neg	390.1310	2.1956	0.8948	1.3445	0.1474	0.2584
metab_9349	neg	390.1690	4.3037	0.6842	1.2610	0.1641	0.2793
metab_12947	neg	390.1929	4.3716	0.1321	0.1302	0.7818	0.8445
metab_5122	pos	390.2032	3.2573	0.4130	-2.0268	0.4067	0.5397
metab_3596	pos	390.2112	10.7150	0.3851	-0.2896	0.0031	0.0161
metab_3686	pos	390.2112	9.9470	0.6063	-0.5168	0.0002	0.0028
metab_12766	neg	390.2143	5.1131	1.3568	2.0002	0.0010	0.0086
metab_9499	neg	390.2246	5.0484	1.3998	2.7540	0.0111	0.0423
metab_9116	neg	390.2404	3.4639	0.0703	-0.3445	0.8429	0.8910
metab_9941	neg	390.2419	7.3508	1.7872	3.6045	0.0010	0.0084
metab_705	pos	390.2606	7.0082	1.1102	-1.7300	0.0153	0.0482
metab_2647	pos	390.2607	6.8728	0.3313	-1.0200	0.5515	0.6697
metab_995	pos	390.2627	6.3581	0.5003	0.2430	0.3902	0.5226
metab_11553	neg	390.2788	9.1200	1.6376	-2.7396	0.0001	0.0026
metab_470	pos	390.2842	2.6090	1.6244	13.6971	0.0030	0.0158
metab_2937	pos	390.2976	8.6861	0.8610	2.0905	0.1297	0.2299
metab_12088	neg	390.3133	7.7251	1.0059	-2.2019	0.0218	0.0670
metab_4207	pos	390.3204	7.7208	0.9860	-1.5842	0.0999	0.1890
metab_11052	neg	390.8746	14.0438	0.3021	-0.0182	0.1068	0.2039
metab_8918	neg	390.9269	2.8123	0.1971	-0.1607	0.6168	0.7195
metab_13562	neg	390.9269	2.6841	0.4178	-0.4015	0.3873	0.5163
metab_15010	neg	390.9341	0.5126	0.8219	-1.1788	0.0636	0.1403
metab_13726	neg	390.9455	2.3533	1.8424	-1.2192	0.0035	0.0192
metab_14961	neg	391.1131	0.5286	1.3591	-1.7543	0.0287	0.0805
metab_6827	neg	391.1362	1.0357	0.3703	1.9238	0.6670	0.7598
metab_1880	pos	391.1401	2.3600	0.6092	-1.0083	0.0874	0.1717
metab_9200	neg	391.1436	3.7488	1.8414	4.3159	0.0046	0.0229
metab_5506	pos	391.1491	2.1897	0.3429	-0.4328	0.3566	0.4909
metab_8945	neg	391.1522	2.8768	0.2722	0.4017	0.6219	0.7228
metab_5212	pos	391.1755	2.9652	0.8803	2.1135	0.2262	0.3494
metab_13439	neg	391.1791	2.9588	0.5942	0.9249	0.1544	0.2671
metab_12953	neg	391.1897	4.3716	0.5690	-0.9159	0.1837	0.3022

metab_9232	neg	391.1986	3.8333	2.1217	6.3598	0.0003	0.0039
metab_1608	pos	391.2063	1.5284	0.7778	-1.2265	0.3592	0.4934
metab_14230	neg	391.2093	1.5439	0.7134	-1.7805	0.2902	0.4215
metab_513	pos	391.2118	3.1346	0.5725	-2.9652	0.3345	0.4684
metab_10108	neg	391.2262	8.0069	2.1966	-8.0170	0.0000	0.0005
metab_518	pos	391.2464	3.1191	0.2390	-1.3984	0.8150	0.8784
metab_822	pos	391.2834	9.1302	0.4911	-0.3431	0.0360	0.0892
metab_4787	pos	391.2938	4.6544	0.1106	-0.1073	0.8460	0.9007
metab_4330	pos	391.2983	7.0670	0.0113	-0.0623	0.9413	0.9632
metab_4135	pos	391.2984	8.0843	0.5306	0.3283	0.0615	0.1326
metab_3779	pos	391.2985	9.6199	1.0400	1.3093	0.0003	0.0036
metab_955	pos	391.2986	9.3429	0.5779	0.5005	0.0601	0.1302
metab_8722	neg	392.0661	2.3078	1.5523	-2.8793	0.0007	0.0067
metab_10289	neg	392.1092	8.6728	1.9001	-3.5814	0.0000	0.0006
metab_8137	neg	392.1132	1.2377	0.3066	-0.0566	0.3975	0.5255
metab_5905	pos	392.1263	1.4429	1.6159	-3.8200	0.0009	0.0070
metab_8755	neg	392.1362	2.3855	1.7579	-2.9939	0.0000	0.0010
metab_13932	neg	392.1468	1.9904	1.0229	-1.1436	0.0836	0.1705
metab_8308	neg	392.1494	1.4856	1.3387	3.7377	0.0058	0.0272
metab_12904	neg	392.1510	4.5884	1.8682	-3.0059	0.0018	0.0121
metab_1532	pos	392.1804	1.2725	0.9863	0.8727	0.1490	0.2551
metab_2096	pos	392.1832	3.2274	0.7886	-1.0903	0.0413	0.0990
metab_8705	neg	392.1833	2.2754	0.1089	0.3500	0.7603	0.8280
metab_4854	pos	392.2059	4.3672	0.2844	0.1018	0.6642	0.7622
metab_6983	neg	392.2296	5.2257	0.1271	-0.0773	0.8171	0.8701
metab_6991	neg	392.2296	4.9349	0.3194	-0.3356	0.5202	0.6381
metab_12397	neg	392.2448	6.7603	1.6688	7.8224	0.0050	0.0244
metab_5064	pos	392.2533	3.4702	0.4646	-0.0859	0.5213	0.6429
metab_5237	pos	392.2635	2.8877	0.4359	0.4231	0.0757	0.1541
metab_2625	pos	392.2780	6.7516	0.2824	0.8286	0.6795	0.7750
metab_9846	neg	392.2810	6.9525	0.2289	0.4727	0.6788	0.7689
metab_11986	neg	392.2924	8.0532	1.4862	-2.0965	0.0006	0.0062
metab_548	pos	392.2991	3.3943	0.9475	1.0654	0.0197	0.0575
metab_2209	pos	392.2994	3.7148	0.2686	-0.0614	0.5053	0.6277
metab_717	pos	392.3146	7.1864	0.8725	-1.3234	0.0940	0.1813
metab_11767	neg	392.3177	8.6874	1.4984	-2.2718	0.0005	0.0056
metab_3920	pos	392.3300	8.9507	1.4354	3.0896	0.0020	0.0121
metab_6870	neg	393.0945	1.2663	0.3218	-0.0803	0.3276	0.4601
metab_14234	neg	393.1340	1.5439	1.1173	-1.1406	0.0000	0.0007
metab_12285	neg	393.1381	7.0796	0.6571	-0.5276	0.1407	0.2497
metab_13702	neg	393.1590	2.4010	1.5339	3.3614	0.0033	0.0181
metab_9036	neg	393.1592	3.1626	0.8828	2.0647	0.0830	0.1695
metab_8660	neg	393.1593	2.1630	0.7051	1.0095	0.0719	0.1526
metab_5528	pos	393.1645	2.1444	1.0586	-2.0041	0.0520	0.1173
metab_13254	neg	393.1678	3.4470	0.1746	-0.1414	0.5925	0.6996
metab_9152	neg	393.1678	3.5813	0.4091	-0.4856	0.3288	0.4615
metab_14326	neg	393.1887	1.4568	0.0831	-0.4866	0.9104	0.9411
metab_9265	neg	393.1950	3.9677	1.4019	3.1319	0.0043	0.0219

metab_6961	neg	393.1956	4.3882	1.2973	2.7863	0.0093	0.0379
metab_2150	pos	393.1996	3.4549	0.8357	1.2765	0.0544	0.1215
metab_2188	pos	393.1997	3.6384	0.8722	1.2824	0.0741	0.1521
metab_2316	pos	393.2024	4.3818	1.1399	-1.9442	0.0430	0.1020
metab_6990	neg	393.2054	5.4844	1.0531	-1.1923	0.0532	0.1228
metab_1822	pos	393.2121	2.1595	0.3870	1.0630	0.6178	0.7257
metab_1050	pos	393.2222	1.5705	0.4419	-1.7872	0.5579	0.6749
metab_3902	pos	393.2390	9.0254	1.5249	-3.1307	0.0031	0.0159
metab_2819	pos	393.2391	8.0700	1.1633	-1.5926	0.0002	0.0031
metab_10042	neg	393.2764	7.7404	1.4681	-1.6853	0.0002	0.0031
metab_2437	pos	393.2851	5.1686	0.2938	-0.2729	0.1882	0.3038
metab_11501	neg	393.3020	9.3165	0.9609	-0.8786	0.0219	0.0671
metab_4572	pos	393.3101	5.8148	0.9886	3.9421	0.0469	0.1089
metab_2427	pos	393.3101	5.1076	0.9857	2.0961	0.2025	0.3214
metab_2353	pos	393.3104	4.6096	0.8347	1.8047	0.1414	0.2453
metab_12131	neg	393.3127	7.6323	0.9768	-2.3581	0.0590	0.1325
metab_3104	pos	393.3141	9.7899	0.6524	0.6427	0.0155	0.0485
metab_798	pos	393.3142	8.7008	0.0127	-0.2040	0.8327	0.8899
metab_2619	pos	393.3465	6.7217	1.1776	16.2429	0.0852	0.1685
metab_4176	pos	393.3578	7.9260	1.0435	-1.0557	0.1331	0.2342
metab_11061	neg	393.8944	14.0282	0.1783	0.0376	0.4959	0.6170
metab_8798	neg	394.0242	2.4970	1.8168	-3.7160	0.0000	0.0001
metab_14207	neg	394.0446	1.5586	0.1068	0.2930	0.7803	0.8435
metab_14550	neg	394.1256	1.1503	0.9494	-1.3466	0.0420	0.1045
metab_6002	pos	394.1267	1.2446	0.7634	1.1395	0.2268	0.3499
metab_13645	neg	394.1275	2.5113	0.6667	-1.4758	0.3002	0.4303
metab_7031	neg	394.1451	7.0322	0.9213	-1.3962	0.0531	0.1226
metab_1766	pos	394.1599	2.0062	0.9529	-1.4882	0.1114	0.2052
metab_14135	neg	394.1623	1.6491	0.1317	0.4008	0.8678	0.9092
metab_7703	neg	394.1633	3.2972	0.9948	1.8633	0.0515	0.1198
metab_2352	pos	394.1640	4.5944	1.9442	-3.5532	0.0009	0.0068
metab_12899	neg	394.1668	4.5884	1.7987	-3.3536	0.0000	0.0005
metab_7527	neg	394.1725	1.9596	1.6006	-2.6783	0.0026	0.0155
metab_1856	pos	394.1964	2.2665	0.0900	0.0652	0.8175	0.8804
metab_5638	pos	394.2073	1.9609	0.9887	1.9315	0.2049	0.3244
metab_9532	neg	394.2450	5.2419	0.9936	2.6245	0.0881	0.1772
metab_2460	pos	394.2556	5.3608	1.1657	12.1943	0.0306	0.0798
metab_2237	pos	394.2578	3.8821	1.9276	6.7370	0.0031	0.0158
metab_9730	neg	394.2605	6.3759	0.5053	0.2748	0.3399	0.4731
metab_12044	neg	394.2609	7.8984	0.0820	-0.4188	0.8838	0.9215
metab_699	pos	394.2941	6.9638	0.2825	0.2161	0.6283	0.7335
metab_4670	pos	394.2941	5.2573	0.7336	1.9196	0.4040	0.5372
metab_4068	pos	394.2943	8.3322	1.0230	2.3672	0.2109	0.3314
metab_2373	pos	394.2943	4.7001	2.2489	7.9726	0.0000	0.0000
metab_4844	pos	394.2944	4.3972	1.6124	5.1934	0.0577	0.1265
metab_9845	neg	394.2966	6.9525	1.0926	-0.7528	0.1375	0.2454
metab_11983	neg	394.2966	8.0532	0.9210	-1.4809	0.0555	0.1267
metab_11765	neg	394.3239	8.6874	1.5686	-2.4523	0.0003	0.0036

metab_7110	neg	394.3332	9.0713	0.8142	-0.9014	0.0928	0.1838
metab_3113	pos	394.3453	9.8211	0.1926	-0.2459	0.5755	0.6902
metab_13205	neg	394.9554	3.5813	1.6910	-3.1782	0.0010	0.0088
metab_5984	pos	395.1076	1.2725	0.1303	0.4306	0.9218	0.9493
metab_13866	neg	395.1463	2.0829	0.7237	-0.7270	0.1463	0.2568
metab_13962	neg	395.1464	1.9280	0.4844	-0.5972	0.2476	0.3745
metab_14337	neg	395.1464	1.4568	0.6326	-0.7957	0.3026	0.4331
metab_12315	neg	395.1535	7.0162	1.0411	-1.5445	0.0584	0.1317
metab_10308	neg	395.1537	8.7191	0.9725	-0.8153	0.0001	0.0027
metab_6019	pos	395.1551	1.2162	1.7282	4.3198	0.0003	0.0035
metab_8703	neg	395.1561	2.2754	1.3589	4.4913	0.0508	0.1187
metab_3547	pos	395.1665	12.6430	0.3843	-0.2927	0.0251	0.0690
metab_3534	pos	395.1665	13.6662	0.2140	-0.2276	0.3072	0.4394
metab_3230	pos	395.1665	11.5098	0.2228	-0.1994	0.1814	0.2958
metab_3004	pos	395.1666	9.0999	0.8392	-0.7127	0.0044	0.0201
metab_3248	pos	395.1667	12.8805	0.2528	-0.2590	0.0910	0.1771
metab_5036	pos	395.1804	3.5771	0.7762	-1.3120	0.1692	0.2802
metab_12756	neg	395.1974	5.1610	0.8813	1.7674	0.1875	0.3064
metab_7634	neg	395.2080	2.6841	1.6189	4.2648	0.0021	0.0134
metab_13122	neg	395.2081	3.8001	2.6005	11.1196	0.0000	0.0010
metab_7421	neg	395.2082	4.2367	1.8630	4.2768	0.0004	0.0046
metab_13064	neg	395.2183	4.0020	1.7535	4.5905	0.0002	0.0028
metab_2486	pos	395.2183	5.5414	1.0748	-1.8825	0.0624	0.1338
metab_9558	neg	395.2200	5.4034	1.0581	1.2432	0.1388	0.2469
metab_12577	neg	395.2207	5.9866	0.8777	-1.1980	0.0852	0.1727
metab_12112	neg	395.2208	7.6951	0.5970	0.8991	0.1664	0.2821
metab_12241	neg	395.2441	7.2069	1.2367	-2.3176	0.0365	0.0945
metab_11913	neg	395.2444	8.2409	1.0036	-1.0836	0.0321	0.0867
metab_4140	pos	395.2783	8.0700	0.9929	-0.9900	0.0434	0.1024
metab_10265	neg	395.2807	8.6093	1.0661	-0.9055	0.0073	0.0320
metab_10171	neg	395.2916	8.2409	1.2031	-0.8295	0.0234	0.0703
metab_902	pos	395.3301	9.9154	0.7070	0.5993	0.0095	0.0347
metab_10598	neg	395.3533	9.9929	0.8563	1.6771	0.0514	0.1197
metab_3980	pos	395.3732	8.6577	0.1217	0.2865	0.8910	0.9307
metab_1175	pos	395.9590	0.4999	0.1827	-0.4241	0.6495	0.7511
metab_8281	neg	396.0080	1.4568	0.4675	0.7421	0.3751	0.5059
metab_13643	neg	396.0310	2.5264	1.8015	-3.2668	0.0002	0.0029
metab_14601	neg	396.0543	1.0074	0.6076	0.1310	0.1770	0.2943
metab_11829	neg	396.0932	8.5135	0.5490	-0.1304	0.2381	0.3640
metab_11607	neg	396.0933	9.0230	1.3264	3.0934	0.0020	0.0131
metab_14314	neg	396.0939	1.4568	0.4664	-1.6194	0.4232	0.5490
metab_1112	pos	396.1213	0.9200	1.2502	-1.7741	0.0000	0.0010
metab_8145	neg	396.1275	1.2521	1.1124	-1.9761	0.0641	0.1411
metab_12182	neg	396.1600	7.4776	1.8989	-3.1739	0.0002	0.0033
metab_7526	neg	396.2031	1.8205	1.2433	-3.4182	0.0262	0.0759
metab_9474	neg	396.2396	4.8865	0.2789	1.0752	0.5662	0.6773
metab_4966	pos	396.2484	3.8511	1.1975	-0.5889	0.1526	0.2593
metab_7035	neg	396.2516	7.1266	0.9826	-1.3540	0.0159	0.0541

metab_12183	neg	396.2523	7.4776	0.3920	-0.3885	0.3809	0.5108
metab_4478	pos	396.2725	6.3735	0.6759	1.4047	0.2598	0.3876
metab_583	pos	396.2733	4.2301	1.5695	2.7971	0.0021	0.0126
metab_2179	pos	396.2734	3.5923	1.2950	4.4054	0.0430	0.1020
metab_2429	pos	396.2734	5.1076	1.5095	3.3826	0.0030	0.0157
metab_5105	pos	396.2734	3.3183	0.4362	2.2041	0.4843	0.6100
metab_12503	neg	396.2756	6.3427	1.0845	-0.5501	0.1139	0.2129
metab_9826	neg	396.2757	6.8730	1.6900	-2.2815	0.0060	0.0278
metab_10205	neg	396.2758	8.3687	0.3048	0.2574	0.6296	0.7285
metab_4156	pos	396.3097	7.9980	0.3597	-0.2483	0.4907	0.6151
metab_664	pos	396.3099	6.0252	0.2341	1.7785	0.6324	0.7373
metab_4307	pos	396.3099	7.1701	0.0127	-0.2288	0.9792	0.9882
metab_3937	pos	396.3102	8.8759	0.5883	1.3957	0.3510	0.4859
metab_12270	neg	396.3123	7.1437	0.9862	-1.5563	0.1888	0.3079
metab_10401	neg	396.3397	9.0713	0.9491	-1.1510	0.0774	0.1614
metab_11468	neg	396.3487	9.4485	0.8977	-1.1655	0.0957	0.1881
metab_11095	neg	396.8915	14.0282	0.4003	-0.0963	0.0293	0.0815
metab_7636	neg	396.9348	2.7155	1.1213	-0.3347	0.0295	0.0818
metab_13203	neg	396.9547	3.5813	1.4564	-2.4097	0.0042	0.0215
metab_6722	neg	396.9716	0.5431	0.1280	1.4725	0.7408	0.8134
metab_173	pos	397.0419	0.5140	0.8174	-1.1464	0.0050	0.0220
metab_7485	neg	397.1118	1.7264	0.8245	1.7703	0.0846	0.1717
metab_1720	pos	397.1166	1.8432	1.7747	5.7899	0.0093	0.0341
metab_324	pos	397.1595	1.4429	1.0159	-1.8739	0.0460	0.1072
metab_7464	neg	397.1622	1.5439	0.0908	-0.1343	0.7042	0.7882
metab_9927	neg	397.1686	7.2537	0.8367	-1.7315	0.2707	0.4012
metab_10098	neg	397.1692	7.9757	1.8189	-1.7601	0.0022	0.0139
metab_13213	neg	397.1873	3.5641	0.3158	0.1092	0.3139	0.4456
metab_9792	neg	397.2059	6.7278	1.0916	-1.1380	0.0403	0.1014
metab_6988	neg	397.2213	4.9349	0.2337	-0.2826	0.6150	0.7178
metab_7700	neg	397.2237	3.3144	2.3301	6.4590	0.0010	0.0088
metab_13609	neg	397.2238	2.5893	2.8613	12.9641	0.0001	0.0018
metab_12326	neg	397.2264	6.9688	0.3434	1.0677	0.3632	0.4956
metab_2253	pos	397.2341	3.9722	1.9172	5.5401	0.0003	0.0032
metab_4530	pos	397.2342	5.9949	0.7825	-2.2192	0.1520	0.2586
metab_11775	neg	397.2601	8.6728	0.7092	-0.4884	0.1297	0.2346
metab_11943	neg	397.2602	8.1468	1.8319	-3.0253	0.0000	0.0007
metab_2375	pos	397.2842	4.7156	2.5841	8.5128	0.0000	0.0000
metab_10278	neg	397.2871	8.6239	1.1142	-1.4263	0.0054	0.0258
metab_4082	pos	397.2941	8.2872	1.2472	-1.5451	0.0210	0.0605
metab_10037	neg	397.2963	7.7251	1.4960	6.2014	0.0031	0.0177
metab_10377	neg	397.2965	9.0067	0.0570	0.8002	0.8656	0.9077
metab_11965	neg	397.2965	8.0843	0.7497	2.0324	0.1453	0.2556
metab_11651	neg	397.3326	8.9086	0.3896	0.7038	0.1691	0.2853
metab_10300	neg	397.3327	8.7031	0.9767	-1.0070	0.0156	0.0535
metab_7123	neg	397.3328	9.5314	1.7860	5.1974	0.0009	0.0078
metab_7088	neg	397.3328	8.4667	0.9618	-1.2625	0.0250	0.0734
metab_4036	pos	397.3446	8.4504	1.5262	-2.7304	0.0040	0.0189

metab_2834	pos	397.3449	8.1414	1.0161	-2.1734	0.0030	0.0156
metab_807	pos	397.3462	8.9212	0.9088	-1.3621	0.0015	0.0096
metab_11235	neg	397.3689	10.3524	0.4643	0.9815	0.2671	0.3974
metab_2416	pos	397.7202	5.0307	0.7119	-1.0721	0.2909	0.4213
metab_9443	neg	398.0362	4.7384	0.3442	-1.2678	0.5395	0.6540
metab_11828	neg	398.0901	8.5135	0.5518	-0.1355	0.2665	0.3966
metab_14285	neg	398.1029	1.5012	0.7048	1.9281	0.2620	0.3919
metab_6889	neg	398.1102	1.3817	0.3656	-0.5161	0.3361	0.4691
metab_14476	neg	398.1213	1.2663	0.6859	1.5899	0.2689	0.3988
metab_13089	neg	398.1251	3.9005	1.2433	-1.5429	0.0114	0.0430
metab_14997	neg	398.1274	0.5126	0.9568	-1.1736	0.0382	0.0977
metab_14776	neg	398.1308	0.6131	0.9230	-3.5028	0.0371	0.0956
metab_7306	neg	398.1326	8.7191	1.0298	-0.9398	0.0000	0.0013
metab_8524	neg	398.1360	1.8682	0.4276	-0.4144	0.2428	0.3691
metab_13765	neg	398.1452	2.2754	1.1687	-1.7462	0.0007	0.0067
metab_9029	neg	398.1574	3.1286	0.8647	0.1282	0.2211	0.3451
metab_5282	pos	398.1734	2.7497	0.6407	0.6667	0.2900	0.4204
metab_5320	pos	398.2099	2.6244	0.4621	-0.6576	0.0598	0.1297
metab_9708	neg	398.2166	6.2617	0.0541	0.6077	0.8325	0.8827
metab_12475	neg	398.2168	6.4073	0.0192	0.4869	0.9797	0.9859
metab_4757	pos	398.2527	4.8509	1.3811	2.3511	0.0167	0.0509
metab_539	pos	398.2528	3.4857	1.8036	3.1008	0.0001	0.0015
metab_5113	pos	398.2528	3.2880	1.8233	3.1511	0.0006	0.0056
metab_12750	neg	398.2547	5.1772	0.4260	-0.4011	0.5207	0.6385
metab_2752	pos	398.2656	7.4703	0.2230	-0.5106	0.5882	0.7008
metab_10352	neg	398.2707	8.8929	0.5749	0.2522	0.3604	0.4932
metab_4393	pos	398.2890	6.8582	1.8415	-3.9442	0.0000	0.0003
metab_2536	pos	398.2890	5.9208	1.3698	2.2048	0.0181	0.0540
metab_646	pos	398.2891	5.4206	1.1877	2.8150	0.0619	0.1332
metab_545	pos	398.2891	3.5771	1.5957	2.6790	0.0000	0.0009
metab_4856	pos	398.2892	4.3672	2.1718	4.8793	0.0000	0.0007
metab_9637	neg	398.2916	5.8409	1.7603	4.1021	0.0021	0.0134
metab_7200	neg	398.8867	14.0282	0.2903	-0.0051	0.1057	0.2023
metab_8874	neg	398.9317	2.7155	1.1592	-0.4009	0.0272	0.0778
metab_13231	neg	398.9505	3.5141	1.1331	-2.4932	0.0613	0.1364
metab_14972	neg	399.0467	0.5286	0.3632	0.2661	0.4312	0.5560
metab_8663	neg	399.1202	2.1798	1.1959	-1.6778	0.0341	0.0902
metab_8940	neg	399.1203	2.8606	0.6886	-0.5411	0.2287	0.3537
metab_8968	neg	399.1566	2.9588	0.9369	-0.4362	0.1067	0.2038
metab_2455	pos	399.2269	5.3311	1.6521	14.4102	0.0017	0.0108
metab_9543	neg	399.2369	5.3060	0.6830	1.6045	0.1619	0.2767
metab_12404	neg	399.2479	6.7439	0.1802	-0.1341	0.5865	0.6943
metab_11558	neg	399.2754	9.1200	0.5214	-0.2869	0.3312	0.4640
metab_12432	neg	399.2756	6.5989	0.5268	-0.1569	0.1445	0.2544
metab_4770	pos	399.2924	4.7608	1.4330	13.0561	0.0105	0.0371
metab_12006	neg	399.3121	7.9908	0.9090	-1.2066	0.1137	0.2129
metab_2493	pos	399.3207	5.6015	2.1519	2.9887	0.0231	0.0647
metab_4015	pos	399.3248	8.5554	0.3025	-0.0126	0.5879	0.7007

metab_2959	pos	399.3249	8.8318	0.4390	0.0251	0.4456	0.5761
metab_11442	neg	399.3387	9.5314	0.8244	2.5541	0.1504	0.2618
metab_11339	neg	399.3484	9.8947	2.2050	5.9744	0.0005	0.0054
metab_7087	neg	399.3484	8.5135	0.1268	0.2318	0.8300	0.8808
metab_2411	pos	399.7176	4.9856	1.6918	-3.1105	0.0049	0.0215
metab_6341	pos	400.0929	0.6123	1.7830	-5.7350	0.0040	0.0187
metab_13699	neg	400.1255	2.4010	0.0388	1.8795	0.9964	0.9972
metab_11568	neg	400.1474	9.1200	0.7301	-0.2133	0.0291	0.0812
metab_5471	pos	400.1493	2.2519	0.5369	-0.9122	0.2567	0.3836
metab_14425	neg	400.1828	1.3231	0.5532	0.4365	0.3323	0.4649
metab_375	pos	400.1890	1.7412	0.9102	1.1399	0.0136	0.0444
metab_4972	pos	400.2222	3.8361	0.3112	-0.6345	0.5922	0.7039
metab_13160	neg	400.2454	3.6980	1.5650	3.4074	0.0082	0.0346
metab_4903	pos	400.2683	4.1387	0.6411	0.5484	0.1744	0.2872
metab_12668	neg	400.2707	5.6292	0.1370	0.5036	0.7198	0.7990
metab_12826	neg	400.2708	4.8707	0.3010	0.7333	0.6862	0.7746
metab_9370	neg	400.2709	4.4221	0.1998	0.7223	0.7772	0.8409
metab_2858	pos	400.2835	8.2872	0.8116	2.0898	0.1359	0.2382
metab_10223	neg	400.2865	8.4667	1.6396	-1.9794	0.0026	0.0158
metab_136	pos	400.3048	5.8448	1.1042	1.8489	0.0023	0.0129
metab_2163	pos	400.3049	3.5162	1.6725	4.5730	0.0003	0.0033
metab_9722	neg	400.3071	6.3427	2.0286	4.2442	0.0044	0.0224
metab_2540	pos	400.3411	5.9361	1.4940	-1.4936	0.0193	0.0567
metab_4638	pos	400.3411	5.4056	0.6291	-0.7899	0.2498	0.3751
metab_13230	neg	400.9475	3.5141	0.7108	-2.4235	0.2892	0.4207
metab_8573	neg	401.0061	1.9745	0.1739	0.4518	0.6698	0.7618
metab_8575	neg	401.0423	1.9745	0.8209	1.4499	0.0170	0.0566
metab_12685	neg	401.0883	5.5484	0.3361	-0.1044	0.1795	0.2974
metab_13194	neg	401.0883	3.6142	0.3535	-0.0503	0.0676	0.1461
metab_9319	neg	401.0883	4.1869	0.4152	-0.0785	0.0337	0.0896
metab_8576	neg	401.0929	1.9745	1.4713	-2.0206	0.0153	0.0527
metab_8686	neg	401.1296	2.2272	0.3395	0.6394	0.4270	0.5524
metab_5519	pos	401.1334	2.1746	0.9565	-1.5558	0.0336	0.0851
metab_2002	pos	401.1335	2.8568	0.7436	-0.9930	0.1666	0.2770
metab_14500	neg	401.1569	1.2521	1.3094	-3.2134	0.0495	0.1167
metab_5218	pos	401.1699	2.9501	1.1965	-0.9084	0.0336	0.0850
metab_13239	neg	401.1723	3.4969	0.5537	-1.1139	0.3250	0.4574
metab_9057	neg	401.1724	3.2301	0.6098	-0.9727	0.3575	0.4903
metab_13818	neg	401.1832	2.1630	0.0749	0.7180	0.9464	0.9649
metab_8445	neg	401.2047	1.7104	0.1153	-1.0360	0.9113	0.9415
metab_2412	pos	401.2159	4.9856	0.0232	-0.0653	0.8115	0.8753
metab_8905	neg	401.2297	2.7792	2.4609	9.0794	0.0002	0.0028
metab_9980	neg	401.2340	7.5239	0.7616	0.8337	0.0910	0.1812
metab_8833	neg	401.2409	2.5893	0.9033	12.0912	0.0106	0.0412
metab_9554	neg	401.2551	5.3713	0.2682	-0.2326	0.6310	0.7294
metab_667	pos	401.3364	6.1009	0.6277	1.8344	0.3177	0.4504
metab_11335	neg	401.3548	9.8947	1.1012	12.3843	0.0640	0.1409
metab_11834	neg	401.3549	8.5135	0.3993	0.3996	0.4758	0.5978

metab_2748	pos	401.3725	7.4561	0.7928	-1.3598	0.0882	0.1730
metab_14610	neg	402.0979	0.9933	2.2050	5.9895	0.0000	0.0006
metab_7543	neg	402.1310	2.0516	0.6582	-0.3443	0.3609	0.4938
metab_10498	neg	402.1639	9.5314	1.0607	-0.3007	0.0268	0.0771
metab_7890	neg	402.1645	0.5711	1.7158	4.3796	0.0011	0.0091
metab_5281	pos	402.1838	2.7497	0.8127	-2.7882	0.2490	0.3746
metab_5161	pos	402.2027	3.1346	1.0234	-4.2610	0.1616	0.2708
metab_1885	pos	402.2043	2.3751	1.5212	-3.3581	0.0038	0.0182
metab_12834	neg	402.2137	4.8210	1.4937	11.8013	0.0216	0.0664
metab_7685	neg	402.2137	3.1115	0.2096	0.2662	0.8526	0.8977
metab_9930	neg	402.2145	7.2701	1.6509	-3.8030	0.0000	0.0009
metab_5714	pos	402.2326	1.7705	1.6310	7.3884	0.0103	0.0367
metab_12203	neg	402.2421	7.3824	2.1512	-5.0609	0.0000	0.0006
metab_13042	neg	402.2501	4.0685	1.2073	3.6143	0.0729	0.1540
metab_12956	neg	402.2502	4.3549	0.8539	2.5252	0.2267	0.3514
metab_4863	pos	402.2840	4.3371	1.2009	1.3952	0.0095	0.0346
metab_2181	pos	402.2840	3.5923	1.7681	13.2870	0.0001	0.0016
metab_2461	pos	402.2840	5.3608	0.4892	1.0788	0.4799	0.6065
metab_4607	pos	402.2841	5.6322	0.1521	0.4559	0.8526	0.9054
metab_4991	pos	402.2841	3.7447	1.4907	13.5197	0.0029	0.0152
metab_4825	pos	402.2843	4.5027	0.6519	1.0637	0.2762	0.4047
metab_6669	neg	402.2866	5.7102	1.0097	1.1178	0.1478	0.2587
metab_12551	neg	402.2867	6.1320	0.2268	0.7653	0.7335	0.8079
metab_10069	neg	402.2867	7.8657	1.6355	3.3583	0.0016	0.0116
metab_7305	neg	402.3019	8.7191	0.6822	-1.4011	0.2184	0.3420
metab_11641	neg	402.3019	8.9417	1.4559	-1.9923	0.0002	0.0027
metab_9720	neg	402.3127	6.3269	0.9827	11.3881	0.0311	0.0848
metab_128	pos	402.3204	6.3437	1.0782	2.1873	0.0132	0.0434
metab_2813	pos	402.3206	7.9835	0.4698	-1.7096	0.4011	0.5343
metab_10313	neg	402.3220	8.7191	1.6949	-5.5601	0.0003	0.0037
metab_12074	neg	402.3229	7.7875	1.1848	-1.9459	0.0337	0.0896
metab_11972	neg	402.3230	8.0843	1.3754	-2.2794	0.0252	0.0739
metab_780	pos	402.3567	8.4651	0.0485	-0.0797	0.9147	0.9440
metab_4552	pos	402.3568	5.9361	0.1960	-0.0156	0.8029	0.8693
metab_15003	neg	402.9174	0.5126	0.1461	0.4270	0.6416	0.7389
metab_12994	neg	402.9244	4.2203	1.3693	-2.3574	0.0013	0.0103
metab_9113	neg	402.9632	3.4470	1.4736	4.6600	0.0007	0.0070
metab_14625	neg	402.9953	0.9649	0.5273	0.5989	0.0324	0.0873
metab_13259	neg	402.9955	3.4133	0.6072	0.6494	0.0012	0.0098
metab_13001	neg	403.0934	4.2040	0.3811	-0.1090	0.0873	0.1759
metab_5653	pos	403.1418	1.9309	0.2544	0.4186	0.5930	0.7044
metab_430	pos	403.1421	2.2519	0.3459	0.4645	0.4809	0.6070
metab_13820	neg	403.1523	2.1630	0.7730	1.8718	0.1770	0.2944
metab_7548	neg	403.1626	2.0358	0.4227	1.0852	0.4333	0.5574
metab_7513	neg	403.1626	1.8524	0.0135	1.1517	0.8950	0.9298
metab_14081	neg	403.1727	1.7419	1.1280	-1.6195	0.0849	0.1722
metab_2158	pos	403.1854	3.5011	0.6416	-1.2950	0.3273	0.4613
metab_5132	pos	403.1856	3.2274	0.6634	-1.3172	0.3261	0.4598

metab_5523	pos	403.1965	2.1595	0.1725	0.7819	0.7615	0.8373
metab_5658	pos	403.2177	1.9169	0.4953	0.6250	0.4492	0.5788
metab_1944	pos	403.2532	2.5938	2.7241	12.8314	0.0000	0.0000
metab_4785	pos	403.2621	4.6700	2.0062	4.8893	0.0007	0.0061
metab_12841	neg	403.2705	4.8049	1.1462	-2.2170	0.0163	0.0549
metab_3996	pos	403.2811	8.6132	0.9525	-0.7278	0.0289	0.0765
metab_7804	neg	403.9299	0.5126	0.6985	-0.7938	0.0599	0.1341
metab_7906	neg	404.1049	0.5991	0.2362	0.4599	0.2562	0.3847
metab_1429	pos	404.1111	0.9900	2.3657	7.1262	0.0000	0.0003
metab_12355	neg	404.1426	6.8887	0.7919	1.2124	0.0396	0.1000
metab_8673	neg	404.1466	2.1956	0.2700	0.0506	0.5260	0.6429
metab_13900	neg	404.1467	2.0202	0.5089	-0.1395	0.2127	0.3357
metab_648	pos	404.2058	5.1975	0.3384	-0.2773	0.0832	0.1655
metab_6347	pos	404.2061	0.6123	1.2213	5.4266	0.0142	0.0456
metab_1693	pos	404.2132	1.7271	1.6175	4.0629	0.0000	0.0008
metab_5884	pos	404.2160	1.4569	0.6681	1.1786	0.2226	0.3451
metab_12647	neg	404.2450	5.6936	0.2680	2.5968	0.7579	0.8260
metab_2308	pos	404.2636	4.3512	1.4947	2.5063	0.0033	0.0166
metab_4385	pos	404.2764	6.8886	1.2245	4.7725	0.0166	0.0508
metab_10157	neg	404.2815	8.1774	0.2057	-0.3306	0.5460	0.6588
metab_12021	neg	404.2822	7.9612	0.7705	-0.6756	0.0940	0.1853
metab_11617	neg	404.2945	9.0067	1.2364	-1.4149	0.0194	0.0616
metab_6667	neg	404.3022	6.2294	1.1923	0.9941	0.1137	0.2129
metab_6663	neg	404.3023	7.9292	0.5561	-0.1269	0.3366	0.4696
metab_2942	pos	404.3148	8.7161	0.5138	-1.6185	0.3351	0.4689
metab_2977	pos	404.3149	8.9507	1.0731	-1.6844	0.0359	0.0891
metab_747	pos	404.3361	7.7946	0.9940	-1.6079	0.0744	0.1523
metab_14956	neg	404.4965	0.5286	1.7738	2.6813	0.0081	0.0344
metab_9331	neg	404.9214	4.2203	1.4769	-2.8402	0.0009	0.0076
metab_7596	neg	404.9481	2.4010	0.7452	-0.1015	0.1738	0.2906
metab_6100	pos	405.0083	1.1024	1.2996	4.0432	0.0014	0.0092
metab_14915	neg	405.1285	0.5431	0.6755	-0.8352	0.1328	0.2387
metab_14294	neg	405.1309	1.4856	0.0183	0.3699	0.8890	0.9256
metab_5532	pos	405.1650	2.1444	0.3775	0.2618	0.4459	0.5762
metab_410	pos	405.1757	2.0524	0.5284	-0.3393	0.1710	0.2828
metab_13108	neg	405.1935	3.8333	0.8588	-0.3735	0.0969	0.1899
metab_9452	neg	405.2038	4.7709	1.0803	-1.0898	0.0032	0.0178
metab_12335	neg	405.2052	6.9354	1.2836	-1.5520	0.0078	0.0334
metab_13800	neg	405.2247	2.2119	1.0030	-2.2627	0.1932	0.3132
metab_13906	neg	405.2248	2.0202	0.7616	-2.2448	0.3895	0.5182
metab_8508	neg	405.2251	1.8205	0.2401	-1.2550	0.7145	0.7955
metab_6931	neg	405.2287	3.8164	1.4909	5.2287	0.0000	0.0004
metab_2367	pos	405.2778	4.6700	0.5789	1.6577	0.3090	0.4413
metab_2236	pos	405.2779	3.8666	1.3831	3.7112	0.0303	0.0792
metab_10481	neg	405.2803	9.4485	1.2758	2.0545	0.0009	0.0078
metab_14592	neg	406.0914	1.0357	1.6916	-2.8361	0.1895	0.3088
metab_1464	pos	406.1266	1.1164	1.8532	15.9299	0.0008	0.0067
metab_8920	neg	406.1987	2.8123	0.2914	-0.2688	0.4505	0.5741

metab_2888	pos	406.2942	8.4074	1.0711	2.1731	0.0434	0.1024
metab_4102	pos	406.2945	8.1998	0.9129	1.7569	0.0293	0.0773
metab_12250	neg	406.2967	7.2069	0.6105	-0.2322	0.3402	0.4734
metab_4707	pos	406.7255	5.0307	1.1186	-1.0388	0.1231	0.2213
metab_15128	neg	406.7938	0.0197	0.3902	-0.0950	0.0242	0.0720
metab_7595	neg	406.9449	2.4010	0.9204	-0.1980	0.1474	0.2584
metab_14087	neg	407.1436	1.7264	0.3641	1.9488	0.3940	0.5224
metab_9353	neg	407.1749	4.3207	1.0454	4.9842	0.1395	0.2481
metab_9297	neg	407.1834	4.0859	1.1995	-1.2974	0.0009	0.0079
metab_8955	neg	407.1844	2.9092	0.3779	1.2220	0.5217	0.6396
metab_9954	neg	407.2206	7.4300	0.7161	-0.2356	0.0364	0.0944
metab_5498	pos	407.2379	2.2045	1.1174	-2.3403	0.0962	0.1840
metab_1770	pos	407.2379	2.0211	0.6589	-2.2830	0.3761	0.5096
metab_9675	neg	407.2553	6.0510	1.2487	-0.8819	0.0247	0.0728
metab_4526	pos	407.2899	6.0252	0.6838	-0.8247	0.0354	0.0884
metab_7060	neg	407.2920	7.7251	0.8442	-0.0751	0.0797	0.1650
metab_2603	pos	407.2931	6.5704	0.7363	0.7326	0.0193	0.0568
metab_2810	pos	407.2933	7.9835	0.6397	0.4456	0.0931	0.1800
metab_4283	pos	407.2933	7.2916	0.6557	0.5513	0.0400	0.0966
metab_772	pos	407.2935	8.4074	0.7420	0.5541	0.0093	0.0342
metab_7124	neg	407.2960	9.5314	0.9268	1.4062	0.0046	0.0229
metab_2530	pos	407.3259	5.8753	0.6198	2.1201	0.3516	0.4867
metab_10029	neg	407.3279	7.7100	1.8828	-2.7347	0.0020	0.0130
metab_3992	pos	407.5191	8.6132	0.3861	-0.5546	0.2683	0.3959
metab_1441	pos	408.1048	1.0461	0.9435	-3.2315	0.1906	0.3060
metab_13996	neg	408.1417	1.8682	0.1743	-0.1099	0.8426	0.8907
metab_13921	neg	408.1417	2.0055	0.1421	-0.3196	0.6844	0.7733
metab_7486	neg	408.1418	1.7264	0.8841	1.3669	0.1164	0.2164
metab_9159	neg	408.1458	3.5978	1.8336	-3.0365	0.0067	0.0300
metab_5750	pos	408.1577	1.6983	0.2760	1.0902	0.4900	0.6147
metab_7009	neg	408.1616	6.4073	0.2870	0.8107	0.3671	0.4987
metab_8478	neg	408.1781	1.7729	0.5135	-0.5375	0.2778	0.4082
metab_2187	pos	408.1943	3.6384	0.3505	0.7759	0.5328	0.6535
metab_5089	pos	408.2119	3.3635	0.0488	-0.2375	0.9109	0.9427
metab_12839	neg	408.2245	4.8049	0.4713	0.8439	0.3197	0.4520
metab_9326	neg	408.2246	4.2203	1.0754	1.9505	0.0429	0.1060
metab_13158	neg	408.2246	3.6980	0.9492	1.2869	0.1004	0.1946
metab_4539	pos	408.2711	5.9649	0.5580	1.1326	0.4058	0.5389
metab_5155	pos	408.2735	3.1648	2.0514	8.1671	0.0001	0.0014
metab_4954	pos	408.2735	3.9114	1.8778	5.6083	0.0042	0.0193
metab_4992	pos	408.2736	3.7447	1.5859	14.2599	0.0050	0.0220
metab_12519	neg	408.2760	6.2617	0.2595	-0.1704	0.6331	0.7311
metab_10071	neg	408.2764	7.8822	1.3806	-1.4577	0.0003	0.0044
metab_12256	neg	408.3123	7.1903	1.1390	-1.4022	0.0911	0.1815
metab_3912	pos	408.3248	8.9810	1.9734	-4.2993	0.0003	0.0034
metab_3933	pos	408.3463	8.8904	0.6118	0.9878	0.2782	0.4072
metab_4171	pos	408.3463	7.9407	1.6263	-3.2305	0.0002	0.0025
metab_4374	pos	408.3938	6.9182	0.3891	-0.2211	0.2480	0.3733

metab_7725	neg	408.7889	0.0136	0.3723	-0.0752	0.0484	0.1150
metab_9320	neg	408.9714	4.1869	0.7810	-0.5587	0.2173	0.3409
metab_13665	neg	409.1079	2.4813	0.8301	-1.6516	0.2232	0.3473
metab_8614	neg	409.1259	2.0516	0.5446	-0.3990	0.2503	0.3779
metab_9607	neg	409.1329	5.6615	1.4055	-1.9887	0.0059	0.0274
metab_13091	neg	409.1408	3.9005	0.2431	-0.6091	0.6396	0.7370
metab_1690	pos	409.1574	1.7271	1.0174	1.1564	0.0974	0.1858
metab_13678	neg	409.1623	2.4487	0.3883	-0.1943	0.5038	0.6238
metab_14271	neg	409.1623	1.5155	0.8343	-0.3490	0.0752	0.1575
metab_5568	pos	409.1746	2.0828	0.0651	1.1806	0.8984	0.9345
metab_5851	pos	409.1856	1.5284	0.1616	0.2029	0.8004	0.8673
metab_6950	neg	409.2004	4.2367	0.9868	-1.0726	0.0337	0.0896
metab_2641	pos	409.2339	6.8582	0.0291	-0.8280	0.9316	0.9568
metab_11982	neg	409.2367	8.0688	1.0593	-1.0051	0.0001	0.0024
metab_4567	pos	409.2552	5.8597	1.3314	-2.9974	0.0069	0.0275
metab_2591	pos	409.2554	6.4035	0.9321	-0.9662	0.0153	0.0482
metab_11883	neg	409.2597	8.3209	1.2233	-1.0329	0.0095	0.0384
metab_9769	neg	409.2712	6.5829	2.0206	-3.1022	0.0003	0.0040
metab_12549	neg	409.2712	6.1320	1.7189	-0.5517	0.0256	0.0745
metab_10264	neg	409.2967	8.6093	2.0005	-3.4038	0.0001	0.0021
metab_7061	neg	409.2985	7.7251	0.3946	0.1967	0.3197	0.4520
metab_738	pos	409.3059	7.7208	0.5964	-0.4694	0.1434	0.2478
metab_11918	neg	409.3077	8.2253	0.5063	0.3623	0.3187	0.4509
metab_95	pos	409.3087	8.0700	0.1200	-0.0622	0.7507	0.8292
metab_11400	neg	409.3118	9.6644	0.5780	-0.6401	0.0903	0.1803
metab_10492	neg	409.3119	9.5147	0.4425	-0.3024	0.0667	0.1448
metab_2780	pos	409.3412	7.7208	1.5302	-2.3305	0.0040	0.0189
metab_10198	neg	409.3441	8.3373	0.4433	0.2520	0.4393	0.5633
metab_11276	neg	409.3691	10.1401	0.3619	0.8774	0.3564	0.4892
metab_14499	neg	410.0863	1.2521	1.9981	-2.2932	0.0478	0.1140
metab_8261	neg	410.1432	1.4427	1.6220	-3.6188	0.0105	0.0409
metab_1724	pos	410.1547	1.8729	0.5241	-0.0576	0.4637	0.5912
metab_1765	pos	410.1548	2.0062	0.0565	-0.5088	0.9728	0.9838
metab_5032	pos	410.1589	3.5923	1.9289	-3.4560	0.0008	0.0065
metab_13023	neg	410.1615	4.1193	1.8232	-3.0477	0.0005	0.0056
metab_4860	pos	410.1741	4.3512	0.3465	-1.4863	0.5194	0.6409
metab_12340	neg	410.1765	6.9191	1.0005	1.5412	0.0440	0.1076
metab_12909	neg	410.2404	4.5717	1.6673	2.7694	0.0045	0.0225
metab_4797	pos	410.2510	4.6096	1.5307	3.8537	0.0148	0.0471
metab_534	pos	410.2527	3.4098	1.8078	6.0824	0.0000	0.0003
metab_9490	neg	410.2553	4.9668	0.6820	1.2662	0.2288	0.3538
metab_12414	neg	410.2555	6.6803	1.0807	12.5180	0.0148	0.0515
metab_2719	pos	410.2679	7.2614	0.4767	-0.1761	0.5391	0.6592
metab_11875	neg	410.2743	8.3687	1.4104	-2.1342	0.0003	0.0040
metab_4454	pos	410.2874	6.4339	0.6886	1.3403	0.3045	0.4363
metab_2799	pos	410.2886	7.8830	1.6238	-2.1429	0.0029	0.0151
metab_2426	pos	410.2890	5.0928	0.7007	0.3455	0.3330	0.4678
metab_4839	pos	410.2890	4.4429	2.0901	5.4459	0.0002	0.0024

metab_4782	pos	410.2890	4.6851	1.1326	1.3363	0.0851	0.1684
metab_5037	pos	410.2891	3.5771	2.0558	6.8703	0.0002	0.0023
metab_9590	neg	410.2914	5.5966	0.3624	1.0999	0.5887	0.6962
metab_7410	neg	410.2915	6.2129	0.8872	-0.4507	0.1643	0.2795
metab_9631	neg	410.2916	5.7751	0.3325	0.7374	0.6841	0.7731
metab_12573	neg	410.2916	6.0024	0.8104	0.6182	0.2347	0.3601
metab_11878	neg	410.2917	8.3528	0.6752	-0.1922	0.1337	0.2401
metab_10147	neg	410.2919	8.1307	0.8617	1.6750	0.2131	0.3359
metab_12240	neg	410.3190	7.2069	0.6330	-1.3569	0.1993	0.3200
metab_2583	pos	410.3251	6.3277	0.7135	1.1190	0.3446	0.4794
metab_11954	neg	410.3282	8.1153	1.7540	-2.0390	0.0219	0.0671
metab_12202	neg	410.3285	7.3984	1.1766	-2.5240	0.1864	0.3051
metab_2785	pos	410.4091	7.7514	0.3919	1.1016	0.5278	0.6486
metab_13011	neg	410.9683	4.1869	0.6506	-0.3386	0.2654	0.3956
metab_8427	neg	411.0874	1.6797	0.6693	-0.5848	0.3249	0.4574
metab_14745	neg	411.0912	0.6973	0.4230	-0.4174	0.3918	0.5202
metab_1261	pos	411.0975	0.5420	0.7614	-1.2556	0.1281	0.2277
metab_14344	neg	411.1276	1.4568	0.1388	0.1292	0.4695	0.5919
metab_9658	neg	411.1488	5.9542	0.9948	-0.8584	0.1488	0.2598
metab_13136	neg	411.1566	3.7658	1.9497	-2.4752	0.0056	0.0265
metab_1730	pos	411.1666	1.9014	1.3412	-3.6043	0.0004	0.0042
metab_12474	neg	411.1689	6.4073	0.6843	1.3650	0.2137	0.3365
metab_7627	neg	411.1692	2.6986	1.3228	2.6105	0.0020	0.0133
metab_1678	pos	411.2016	1.7128	1.0573	6.9604	0.1019	0.1922
metab_12926	neg	411.2162	4.5054	0.2809	0.3455	0.5891	0.6965
metab_9754	neg	411.2386	6.4709	0.4650	0.5651	0.4665	0.5889
metab_7350	neg	411.2422	8.0688	1.0848	-1.2136	0.0024	0.0150
metab_7096	neg	411.2763	8.7031	1.2886	-1.5137	0.0007	0.0068
metab_10415	neg	411.2764	9.1038	1.3937	-1.9131	0.0035	0.0192
metab_4611	pos	411.2996	5.5864	1.6038	5.5970	0.0003	0.0034
metab_12041	neg	411.3121	7.8984	0.1408	0.5833	0.9012	0.9341
metab_11618	neg	411.3123	9.0067	1.5055	-1.9709	0.0009	0.0080
metab_11693	neg	411.3234	8.7488	0.9616	-0.8062	0.0179	0.0586
metab_11862	neg	411.3235	8.4022	0.9320	2.3539	0.1230	0.2253
metab_816	pos	411.3248	9.0857	0.2285	0.0559	0.4297	0.5614
metab_4069	pos	411.3568	8.3322	0.4939	-0.0453	0.4087	0.5414
metab_10662	neg	411.3850	10.5180	0.1378	0.6326	0.7360	0.8094
metab_9412	neg	412.0499	4.6056	1.1587	-10.8167	0.0023	0.0145
metab_9888	neg	412.1116	7.0796	0.9110	-0.9263	0.0825	0.1688
metab_7454	neg	412.1206	1.5586	1.0277	1.6881	0.0509	0.1189
metab_8838	neg	412.1255	2.6046	2.6668	14.8763	0.0000	0.0002
metab_8724	neg	412.1256	2.3078	1.4441	7.5436	0.0281	0.0794
metab_12476	neg	412.1561	6.4073	0.1822	0.6258	0.5018	0.6219
metab_10247	neg	412.1564	8.5135	1.1979	1.9444	0.0080	0.0340
metab_9235	neg	412.1731	3.8506	0.3874	0.2731	0.4580	0.5810
metab_13451	neg	412.1882	2.9263	0.1399	1.8735	0.8349	0.8841
metab_8318	neg	412.1980	1.5155	1.8945	-15.2435	0.0000	0.0005
metab_5455	pos	412.2080	2.2978	0.2112	0.0171	0.5393	0.6593

metab_2307	pos	412.2684	4.3512	0.7159	0.9698	0.2831	0.4129
metab_2408	pos	412.2684	4.9698	1.0933	1.7424	0.0528	0.1187
metab_2139	pos	412.2685	3.3793	2.0187	4.8598	0.0001	0.0012
metab_549	pos	412.2685	3.6987	1.5632	2.6727	0.0000	0.0003
metab_12824	neg	412.2712	4.8707	0.5980	2.4240	0.5000	0.6206
metab_2465	pos	412.3045	5.3913	0.0416	0.3457	0.9275	0.9533
metab_1000	pos	412.3046	6.2226	0.2742	0.2853	0.6367	0.7408
metab_663	pos	412.3047	5.9949	0.2865	1.0802	0.6389	0.7425
metab_4813	pos	412.3047	4.5642	1.3072	1.7291	0.0342	0.0862
metab_5013	pos	412.3049	3.6835	1.8129	3.9700	0.0014	0.0094
metab_2878	pos	412.3049	8.3609	0.1540	-0.3690	0.7127	0.8004
metab_12489	neg	412.3072	6.4073	0.2778	1.5214	0.6729	0.7636
metab_7078	neg	412.3074	8.2409	0.1364	0.1955	0.6647	0.7581
metab_7425	neg	412.9654	4.1699	0.1860	1.2438	0.8356	0.8846
metab_13797	neg	413.1392	2.2119	1.0884	2.1001	0.0334	0.0892
metab_5895	pos	413.1404	1.4569	0.1278	-0.0986	0.5509	0.6690
metab_12488	neg	413.1641	6.4073	0.4182	1.0623	0.2746	0.4049
metab_9832	neg	413.1851	6.8887	1.3865	3.6723	0.0013	0.0100
metab_13128	neg	413.2336	3.7830	2.1631	6.5182	0.0014	0.0104
metab_3546	pos	413.2647	12.6724	0.1065	-0.2433	0.7551	0.8324
metab_3260	pos	413.2648	13.8445	0.0237	-0.1102	0.9242	0.9510
metab_3535	pos	413.2649	13.5323	0.2979	-0.2677	0.1273	0.2265
metab_10154	neg	413.2663	8.1774	1.3062	-1.2401	0.0007	0.0066
metab_8989	neg	413.2776	3.0101	2.2016	8.1333	0.0000	0.0000
metab_677	pos	413.2871	6.5096	1.0287	-1.2450	0.0062	0.0256
metab_9795	neg	413.2915	6.7439	0.7051	2.9611	0.3776	0.5076
metab_4026	pos	413.3040	8.4949	0.2756	-0.2700	0.5747	0.6896
metab_2971	pos	413.3041	8.9212	1.1111	1.2200	0.0157	0.0488
metab_3938	pos	413.3407	8.8759	0.8008	-0.8777	0.0059	0.0249
metab_11296	neg	413.3640	10.0432	1.8327	13.1039	0.0014	0.0108
metab_10321	neg	413.3642	8.7346	0.6500	0.8266	0.2338	0.3593
metab_10725	neg	413.9087	13.9143	0.2211	0.0694	0.2914	0.4227
metab_174	pos	414.1048	0.4999	0.2200	0.2548	0.4302	0.5617
metab_12187	neg	414.1274	7.4618	1.8913	-4.4947	0.0002	0.0028
metab_7028	neg	414.1275	7.0322	1.2273	-2.1363	0.0289	0.0809
metab_7318	neg	414.1277	8.7031	0.9620	-0.7418	0.0005	0.0057
metab_7618	neg	414.1345	2.5748	1.4912	2.6091	0.0059	0.0273
metab_8421	neg	414.1414	1.6642	0.1002	2.5826	0.9510	0.9681
metab_13895	neg	414.1415	2.0358	1.7827	-2.7917	0.0000	0.0001
metab_8279	neg	414.1460	1.4568	1.0313	1.8397	0.0248	0.0731
metab_5343	pos	414.1653	2.5627	0.3082	0.3562	0.6014	0.7112
metab_5853	pos	414.1791	1.5284	0.7869	-0.8426	0.1024	0.1930
metab_1021	pos	414.2045	2.0973	0.6791	-2.2902	0.2235	0.3463
metab_1927	pos	414.2046	2.5314	0.9390	-2.8630	0.1380	0.2407
metab_4390	pos	414.2139	6.8728	1.0504	1.4579	0.0020	0.0120
metab_13020	neg	414.2617	4.1364	0.5494	2.1588	0.5002	0.6208
metab_9774	neg	414.2657	6.5989	0.1484	-0.5043	0.7646	0.8311
metab_1015	pos	414.2841	4.8509	0.9883	2.1345	0.1359	0.2382

metab_12627	neg	414.2866	5.7751	0.2098	0.7321	0.7041	0.7881
metab_9468	neg	414.2866	4.8707	1.8665	5.7059	0.0049	0.0242
metab_12471	neg	414.3135	6.4236	0.8857	2.2071	0.2432	0.3695
metab_4096	pos	414.3203	8.2289	0.0541	0.2222	0.9782	0.9879
metab_1	pos	414.3204	6.4192	0.2087	0.8789	0.7309	0.8142
metab_639	pos	414.3205	5.3608	0.6880	-1.6356	0.3597	0.4936
metab_9823	neg	414.3230	6.8730	0.6700	1.9740	0.3394	0.4726
metab_9671	neg	414.3233	6.0346	0.0093	4.8314	0.8906	0.9264
metab_12959	neg	415.0677	4.3380	0.4912	-0.2494	0.0266	0.0766
metab_8514	neg	415.1153	1.8371	0.0054	0.1732	0.9454	0.9643
metab_13395	neg	415.1190	3.0432	1.2403	5.0331	0.0057	0.0267
metab_8090	neg	415.1362	1.1069	1.3613	3.4120	0.0149	0.0517
metab_14397	neg	415.1727	1.3669	1.1677	-3.0705	0.1280	0.2322
metab_12360	neg	415.1797	6.8887	1.0051	1.8238	0.0124	0.0458
metab_125	pos	415.2106	5.7846	0.2678	-0.2235	0.1636	0.2734
metab_9711	neg	415.2162	6.2775	1.5810	-1.3134	0.0099	0.0395
metab_10933	neg	415.2260	14.3039	0.8668	1.3011	0.0043	0.0219
metab_5517	pos	415.2327	2.1746	0.1645	0.5919	0.9007	0.9358
metab_13629	neg	415.2343	2.5431	1.8924	15.1129	0.0005	0.0057
metab_13494	neg	415.2346	2.8269	2.4766	7.6452	0.0000	0.0010
metab_9794	neg	415.2434	6.7439	0.1180	-0.1177	0.6891	0.7769
metab_9467	neg	415.2495	4.8541	2.0984	6.4481	0.0007	0.0070
metab_10738	neg	415.2527	13.9796	0.3191	0.0151	0.1812	0.2993
metab_6622	neg	415.2704	6.5989	1.4403	-2.6693	0.0040	0.0210
metab_3012	pos	415.2714	9.1302	0.6214	-0.3894	0.0990	0.1879
metab_11673	neg	415.2858	8.8128	1.3939	-2.6453	0.0124	0.0459
metab_11931	neg	415.2860	8.1774	1.4258	3.3550	0.0017	0.0119
metab_12128	neg	415.2862	7.6323	0.0688	-0.0483	0.9272	0.9530
metab_5195	pos	415.2905	3.0114	2.5611	7.3813	0.0000	0.0000
metab_4212	pos	415.3188	7.6919	0.0012	-0.1786	0.9075	0.9408
metab_2983	pos	415.3203	8.9810	0.3026	-0.5568	0.7103	0.7983
metab_12010	neg	415.3436	7.9908	0.8050	1.1144	0.1503	0.2618
metab_3898	pos	415.3566	9.0556	0.7208	-0.8046	0.0494	0.1131
metab_3847	pos	415.3566	9.2815	0.8258	-0.8008	0.0123	0.0414
metab_8321	neg	416.0203	1.5155	2.1953	-6.1296	0.0000	0.0004
metab_8493	neg	416.0421	1.8046	1.7268	-5.5456	0.0000	0.0002
metab_7290	neg	416.0752	9.0872	0.6273	-0.1066	0.0760	0.1588
metab_6861	neg	416.1204	1.2663	1.3328	-2.0410	0.0019	0.0127
metab_7113	neg	416.1435	9.1200	0.7533	-0.1813	0.0914	0.1818
metab_14350	neg	416.1466	1.4427	0.2091	0.3459	0.6958	0.7818
metab_8620	neg	416.1469	2.0670	0.0294	0.3818	0.8961	0.9305
metab_9237	neg	416.1470	3.8506	0.4895	-0.0010	0.4080	0.5354
metab_5971	pos	416.1474	1.3153	2.2454	7.6476	0.0000	0.0000
metab_63	pos	416.1476	2.5782	1.5938	2.5575	0.0020	0.0118
metab_8480	neg	416.1828	1.7729	1.4869	-2.0462	0.0253	0.0741
metab_1708	pos	416.2116	1.7845	1.4259	14.1258	0.0103	0.0366
metab_1433	pos	416.2378	1.0040	1.4497	-4.8381	0.0171	0.0521
metab_2341	pos	416.2456	4.5184	1.7923	13.9028	0.0027	0.0145

metab_6953	neg	416.2660	4.3882	0.2750	1.1555	0.6098	0.7134
metab_9306	neg	416.2660	4.1193	0.1211	0.3751	0.8712	0.9118
metab_4433	pos	416.2785	6.5857	0.1658	-0.8599	0.7387	0.8208
metab_12155	neg	416.2924	7.5702	1.5713	-1.9583	0.0001	0.0020
metab_11752	neg	416.2945	8.6874	0.1531	-0.6464	0.7323	0.8070
metab_4584	pos	416.2999	5.7695	0.5831	0.9590	0.4596	0.5884
metab_4822	pos	416.2999	4.5184	1.7640	3.6835	0.0002	0.0028
metab_12516	neg	416.3021	6.2775	0.8391	1.3486	0.1216	0.2235
metab_11991	neg	416.3025	8.0373	1.2564	-2.0485	0.0199	0.0627
metab_2934	pos	416.3127	8.6717	1.1834	-1.8777	0.0096	0.0350
metab_11598	neg	416.3175	9.0557	0.9412	-1.6030	0.2700	0.4004
metab_12363	neg	416.3286	6.8730	0.6217	3.5393	0.2105	0.3332
metab_4775	pos	416.3359	4.7156	0.8152	-1.5180	0.1341	0.2357
metab_2300	pos	416.3361	4.3060	0.9429	-1.2336	0.0822	0.1641
metab_20	pos	416.3361	6.8582	0.2101	0.0455	0.6772	0.7731
metab_2246	pos	416.3361	3.9270	1.0246	-1.2017	0.0962	0.1841
metab_682	pos	416.3362	6.6148	1.3152	-2.4677	0.0080	0.0308
metab_7356	neg	416.3386	8.0373	0.0929	4.3878	0.8100	0.8648
metab_3792	pos	416.3876	9.5438	0.0636	-0.0307	0.9476	0.9669
metab_15118	neg	416.7967	0.0197	0.3534	-0.0461	0.1225	0.2247
metab_8659	neg	417.1310	2.1630	1.0927	-1.6746	0.0277	0.0788
metab_14147	neg	417.1313	1.6335	1.1768	-1.9001	0.0289	0.0809
metab_13545	neg	417.1673	2.7155	0.5541	-1.1344	0.3955	0.5235
metab_8431	neg	417.1999	1.6797	1.3876	2.4831	0.0002	0.0028
metab_13357	neg	417.2149	3.1455	1.7478	6.7467	0.0168	0.0561
metab_654	pos	417.2165	5.7846	0.1761	-0.1925	0.4645	0.5919
metab_12458	neg	417.2287	6.4874	1.3601	14.9609	0.0760	0.1588
metab_4228	pos	417.2390	7.6332	0.0480	-0.0758	0.8451	0.9000
metab_13686	neg	417.2501	2.4338	1.3194	13.1471	0.0265	0.0764
metab_7638	neg	417.2502	2.7155	2.1232	14.7395	0.0017	0.0119
metab_12434	neg	417.2761	6.5989	1.6374	-3.4387	0.0044	0.0225
metab_9814	neg	417.2865	6.8255	0.2713	0.4684	0.7391	0.8119
metab_3928	pos	417.2966	8.9212	1.4274	3.5643	0.0205	0.0594
metab_11658	neg	417.2980	8.8608	1.1232	-1.4403	0.0133	0.0478
metab_11779	neg	417.3011	8.6406	0.8683	1.7683	0.0525	0.1217
metab_11588	neg	418.0721	9.0872	0.8715	-0.4319	0.0101	0.0400
metab_8647	neg	418.1150	2.1311	1.8379	-11.8094	0.0057	0.0267
metab_6982	neg	418.1221	5.2100	0.3140	-0.4946	0.5728	0.6828
metab_12803	neg	418.1222	4.9349	0.5045	-0.8885	0.3244	0.4569
metab_225	pos	418.1541	0.6962	0.0833	0.2753	0.6515	0.7527
metab_1784	pos	418.1597	2.0677	0.0941	0.1081	0.8340	0.8909
metab_43	pos	418.1607	1.4289	1.2998	2.1804	0.0179	0.0537
metab_13885	neg	418.1656	2.0516	1.6948	3.8652	0.0016	0.0113
metab_13198	neg	418.2090	3.5978	2.1533	5.5584	0.0002	0.0033
metab_2438	pos	418.2215	5.1830	0.3776	-0.3686	0.0587	0.1280
metab_3598	pos	418.2425	10.7001	0.5346	-0.4411	0.0008	0.0065
metab_4624	pos	418.2426	5.5112	0.3462	-0.2515	0.0286	0.0760
metab_4715	pos	418.2426	4.9856	0.1111	-0.1462	0.5660	0.6822

metab_4836	pos	418.2427	4.4580	0.0890	-0.0194	0.6413	0.7444
metab_9434	neg	418.2607	4.7046	0.7389	0.0011	0.1646	0.2799
metab_2377	pos	418.2788	4.7156	0.1541	0.0231	0.7350	0.8174
metab_2783	pos	418.2948	7.7357	0.8541	1.9952	0.2064	0.3265
metab_10203	neg	418.2970	8.3528	1.6169	-2.1947	0.0000	0.0014
metab_2763	pos	418.3050	7.5743	1.3452	-1.9749	0.0114	0.0393
metab_3848	pos	418.3096	9.2815	0.9938	-1.3973	0.0243	0.0675
metab_12109	neg	418.3180	7.6951	0.9353	-2.4542	0.0730	0.1541
metab_2994	pos	418.3303	9.0556	0.4198	-1.6126	0.4347	0.5660
metab_4355	pos	418.3304	6.9790	1.0635	-1.4617	0.0121	0.0410
metab_2986	pos	418.7935	8.9958	0.3031	-0.8098	0.4957	0.6194
metab_15035	neg	418.8897	0.4975	0.7624	-0.5670	0.0073	0.0319
metab_1842	pos	419.1552	2.2361	1.2128	-2.0686	0.0319	0.0820
metab_12255	neg	419.2210	7.1903	0.8553	1.4151	0.0209	0.0649
metab_4224	pos	419.2455	7.6481	0.1937	-0.2089	0.4043	0.5373
metab_4117	pos	419.2549	8.1560	0.9250	1.3402	0.0098	0.0355
metab_4875	pos	419.2567	4.3060	1.5969	4.1303	0.0017	0.0105
metab_5012	pos	419.2569	3.6835	2.0078	7.5080	0.0038	0.0180
metab_11772	neg	419.2576	8.6728	2.7818	-4.9483	0.0005	0.0056
metab_11891	neg	419.2577	8.3055	1.7314	-5.4337	0.0002	0.0029
metab_9613	neg	419.2771	5.7102	0.6302	1.1572	0.2813	0.4117
metab_4821	pos	419.2896	4.5335	0.7723	0.6311	0.2251	0.3483
metab_11936	neg	419.3021	8.1774	0.9148	-0.6358	0.0265	0.0766
metab_5594	pos	420.1782	2.0360	1.6271	3.5523	0.0006	0.0053
metab_5735	pos	420.1786	1.7271	1.5031	4.9147	0.0004	0.0042
metab_8240	neg	420.1997	1.3817	0.3956	-0.9482	0.4266	0.5520
metab_12872	neg	420.2399	4.6722	0.7148	4.5466	0.4869	0.6087
metab_4461	pos	420.2712	6.4035	0.3000	0.8010	0.6724	0.7693
metab_680	pos	420.2728	6.5704	0.4260	0.0448	0.3952	0.5279
metab_4384	pos	420.2729	6.8886	0.2090	0.3909	0.6085	0.7183
metab_4780	pos	420.2734	4.7001	0.1429	0.5331	0.8179	0.8806
metab_2874	pos	420.3098	8.3463	1.7155	-2.9197	0.0004	0.0043
metab_2774	pos	420.3312	7.6776	1.2255	-2.2498	0.0123	0.0414
metab_2711	pos	420.3463	7.2014	2.0177	-4.2653	0.0000	0.0005
metab_3022	pos	420.3463	9.2049	1.2648	-2.8370	0.0380	0.0928
metab_4909	pos	420.3576	4.0939	0.1620	0.4447	0.8017	0.8685
metab_7757	neg	420.7895	0.0382	0.1397	-0.1106	0.5551	0.6670
metab_7801	neg	420.9199	0.4975	0.1087	-0.1680	0.7221	0.8007
metab_7653	neg	421.0758	0.5286	0.3338	0.5990	0.2038	0.3255
metab_1291	pos	421.1209	0.5843	0.7807	2.0565	0.2071	0.3269
metab_10283	neg	421.1697	8.6568	0.7589	-0.4119	0.0481	0.1144
metab_8293	neg	421.1733	1.4710	0.0180	0.5550	0.9663	0.9765
metab_14562	neg	421.1834	1.1069	1.1698	-2.8357	0.0777	0.1618
metab_14111	neg	421.1841	1.6948	1.4094	-2.6433	0.0136	0.0486
metab_13350	neg	421.2239	3.1626	2.1679	5.7138	0.0000	0.0010
metab_9148	neg	421.2240	3.5641	1.9154	12.1825	0.0001	0.0019
metab_12295	neg	421.2267	7.0469	0.2803	1.4418	0.6939	0.7805
metab_12062	neg	421.2368	7.8193	1.7007	3.1522	0.0014	0.0108

metab_9747	neg	421.2597	6.4236	1.5630	-1.6354	0.0157	0.0536
metab_9674	neg	421.2715	6.0510	0.3750	0.2920	0.4050	0.5331
metab_12526	neg	421.2922	6.2294	1.0008	1.2207	0.1489	0.2598
metab_2490	pos	421.3043	5.5715	0.5867	2.7419	0.1559	0.2634
metab_475	pos	421.3052	2.6869	0.5886	2.0878	0.4130	0.5454
metab_12599	neg	421.3076	5.9060	0.5939	0.9385	0.3522	0.4857
metab_15126	neg	421.7976	0.0197	0.3024	-0.0518	0.1684	0.2849
metab_6764	neg	422.0882	0.6271	1.6850	3.3150	0.0001	0.0024
metab_6837	neg	422.0884	0.7818	1.5618	3.0152	0.0002	0.0028
metab_14590	neg	422.1239	1.0357	1.5550	5.5342	0.0031	0.0177
metab_13937	neg	422.1575	1.9745	0.7396	-0.5770	0.0285	0.0802
metab_13226	neg	422.1728	3.5310	1.1471	-1.5936	0.0458	0.1107
metab_13095	neg	422.1729	3.8839	0.4076	-0.1693	0.4574	0.5806
metab_5932	pos	422.2124	1.3720	0.1724	-0.7283	0.7084	0.7970
metab_11684	neg	422.2707	8.7640	0.6098	2.3023	0.2781	0.4087
metab_2589	pos	422.2872	6.4035	1.1777	1.9761	0.0018	0.0112
metab_2829	pos	422.2892	8.1273	0.9375	1.5715	0.0540	0.1209
metab_7068	neg	422.2921	8.0843	0.5579	0.9853	0.2493	0.3767
metab_10450	neg	422.3067	9.2507	0.8520	-0.5580	0.2451	0.3717
metab_13485	neg	422.9144	2.8440	0.7595	-0.5519	0.1040	0.1999
metab_8032	neg	423.0553	0.9368	0.7818	-0.7400	0.0930	0.1840
metab_1382	pos	423.0877	0.8361	0.4442	0.0879	0.1904	0.3058
metab_14957	neg	423.0911	0.5286	0.0711	0.9440	0.9865	0.9903
metab_14160	neg	423.1097	1.6186	0.6341	4.5868	0.5558	0.6676
metab_8606	neg	423.1779	2.0358	1.0478	-0.8238	0.3214	0.4538
metab_5793	pos	423.1862	1.6272	0.3405	0.8441	0.5604	0.6771
metab_1062	pos	423.1862	1.4714	1.2135	2.1094	0.0411	0.0987
metab_3886	pos	423.1979	9.0999	0.5378	-0.3253	0.0866	0.1707
metab_425	pos	423.2114	2.1897	1.0261	-1.6295	0.1535	0.2605
metab_10221	neg	423.2528	8.4505	0.0004	0.2413	0.9640	0.9752
metab_698	pos	423.2736	6.4192	0.6950	-1.0269	0.0390	0.0947
metab_134	pos	423.2740	5.9800	0.5738	-0.6902	0.2213	0.3438
metab_644	pos	423.2741	5.4662	1.3710	2.5674	0.0256	0.0702
metab_9766	neg	423.2869	6.5829	1.8478	-2.6730	0.0001	0.0021
metab_9670	neg	423.2870	6.0346	1.2480	-1.1828	0.0047	0.0233
metab_4318	pos	423.2884	7.1267	0.7429	0.5180	0.0654	0.1388
metab_10426	neg	423.2913	9.1200	0.4042	0.7953	0.2991	0.4295
metab_11865	neg	423.2918	8.4022	0.3625	-0.3238	0.2547	0.3831
metab_694	pos	423.3091	6.8886	1.6396	5.6119	0.0393	0.0951
metab_11336	neg	423.3100	9.8947	0.1093	0.5493	0.6699	0.7618
metab_7311	neg	423.3118	8.7031	1.1027	-1.1086	0.0008	0.0072
metab_9731	neg	423.3234	6.3759	1.8662	-3.2989	0.0003	0.0037
metab_9641	neg	423.3235	5.8731	1.1734	-0.0838	0.0732	0.1544
metab_10409	neg	423.3274	9.1038	0.6713	-0.3288	0.0029	0.0167
metab_97	pos	423.3437	8.5241	0.1305	-0.3794	0.8397	0.8952
metab_1177	pos	423.9540	0.4999	1.0445	-1.7989	0.0203	0.0588
metab_1356	pos	424.1009	0.7801	1.5419	2.8173	0.0006	0.0053
metab_14504	neg	424.1030	1.2521	1.6121	-2.3527	0.0055	0.0262

metab_14372	neg	424.1367	1.4115	0.6017	0.7033	0.3252	0.4576
metab_14567	neg	424.1469	1.1069	1.1861	-3.1672	0.0563	0.1280
metab_10296	neg	424.1566	8.7031	0.8591	-0.6244	0.0008	0.0074
metab_11510	neg	424.1566	9.2674	1.2264	-1.2140	0.0002	0.0036
metab_8498	neg	424.1832	1.8046	1.8753	-4.6228	0.0011	0.0091
metab_12224	neg	424.2472	7.3017	0.5794	-0.0188	0.1853	0.3039
metab_4753	pos	424.2665	4.8668	1.4542	2.4112	0.0054	0.0233
metab_9757	neg	424.2708	6.5033	0.6295	-0.3102	0.1641	0.2793
metab_9798	neg	424.2711	6.7603	1.1560	-1.1220	0.1004	0.1946
metab_12527	neg	424.2711	6.2294	0.6363	-0.4300	0.2801	0.4108
metab_10325	neg	424.2841	8.7346	1.0119	-1.2845	0.0936	0.1848
metab_4654	pos	424.2861	5.3311	1.6906	3.2254	0.0023	0.0129
metab_4131	pos	424.3047	8.0988	0.5054	0.5700	0.2209	0.3433
metab_10038	neg	424.3075	7.7251	0.8379	-0.9638	0.0345	0.0909
metab_670	pos	424.3410	6.1614	1.0234	-1.1738	0.0720	0.1492
metab_4598	pos	424.3887	5.6773	0.0940	0.0754	0.8961	0.9333
metab_4698	pos	424.3887	5.0928	0.4968	2.1985	0.4598	0.5884
metab_13484	neg	424.9113	2.8440	0.8248	-0.6558	0.1024	0.1974
metab_8793	neg	424.9300	2.4813	1.8566	-1.7924	0.0008	0.0076
metab_13050	neg	424.9664	4.0516	1.8060	-3.2896	0.0006	0.0061
metab_13414	neg	425.1359	3.0101	0.3345	0.4384	0.6725	0.7635
metab_5392	pos	425.1843	2.4370	0.7140	-2.3751	0.2249	0.3481
metab_1774	pos	425.1909	2.0360	0.3117	-0.5750	0.6817	0.7766
metab_8505	neg	425.1940	1.8205	1.6920	-2.5508	0.0087	0.0362
metab_13446	neg	425.2189	2.9429	1.1101	12.2589	0.0221	0.0675
metab_10368	neg	425.2703	8.9574	0.1700	-0.1575	0.6050	0.7094
metab_2553	pos	425.2887	6.0252	1.2267	-1.1879	0.0378	0.0924
metab_2622	pos	425.2888	6.7366	1.3639	-1.8347	0.0023	0.0131
metab_2577	pos	425.2891	6.2372	1.0684	-1.7983	0.0050	0.0218
metab_2481	pos	425.2891	5.5112	0.7117	4.4039	0.3552	0.4899
metab_120	pos	425.2893	7.6776	0.7824	-0.9762	0.0755	0.1537
metab_10164	neg	425.2906	8.2099	1.3090	-2.0674	0.0004	0.0048
metab_12314	neg	425.2910	7.0162	1.6941	-1.8520	0.0140	0.0495
metab_9767	neg	425.3026	6.5829	1.5375	-1.5120	0.0093	0.0378
metab_12524	neg	425.3026	6.2456	0.8102	-2.1794	0.1974	0.3180
metab_1007	pos	425.3037	5.9649	0.3490	-0.0734	0.4180	0.5498
metab_719	pos	425.3040	7.2763	0.1423	-0.1759	0.7607	0.8368
metab_4453	pos	425.3358	6.4339	1.8731	-2.0780	0.0011	0.0082
metab_11692	neg	425.3393	8.7488	0.9555	-0.4284	0.1732	0.2898
metab_11351	neg	425.3642	9.8620	1.7529	7.8997	0.0020	0.0133
metab_10460	neg	425.3644	9.3000	1.5911	2.9382	0.0035	0.0192
metab_11642	neg	425.3644	8.9252	0.2805	1.2253	0.5718	0.6821
metab_100	pos	425.3769	9.0397	1.2167	5.9439	0.0839	0.1665
metab_2901	pos	425.3978	8.5092	0.8301	0.1439	0.2434	0.3680
metab_15124	neg	425.7791	0.0197	0.3345	-0.0817	0.0937	0.1849
metab_10893	neg	426.0227	14.8329	0.5494	0.5759	0.0020	0.0133
metab_14626	neg	426.0228	0.9649	0.5867	0.7938	0.0654	0.1429
metab_14672	neg	426.0653	0.8662	1.6462	2.9381	0.0065	0.0295

metab_1381	pos	426.1318	0.8361	1.1511	-16.6311	0.0033	0.0167
metab_10241	neg	426.1360	8.5135	1.1959	1.9793	0.0001	0.0027
metab_10424	neg	426.1724	9.1200	0.2905	0.2263	0.4280	0.5532
metab_13892	neg	426.1818	2.0358	1.0560	-1.2789	0.0913	0.1818
metab_8406	neg	426.1821	1.6335	1.1781	-2.5130	0.0212	0.0654
metab_5912	pos	426.1862	1.4146	1.2425	3.1348	0.0068	0.0272
metab_13783	neg	426.1904	2.2437	0.0181	-1.8014	0.9819	0.9873
metab_10255	neg	426.1961	8.5604	1.3584	-1.5800	0.0042	0.0215
metab_510	pos	426.2044	2.4530	0.8461	1.0669	0.0070	0.0277
metab_8504	neg	426.2138	1.8205	1.7072	-2.8532	0.0001	0.0021
metab_9363	neg	426.2356	4.3716	1.6008	12.4298	0.0052	0.0253
metab_720	pos	426.2605	7.2916	0.3300	0.1875	0.5406	0.6607
metab_12087	neg	426.2694	7.7404	2.6446	-6.4196	0.0001	0.0022
metab_7066	neg	426.2824	7.9612	0.8928	-1.3238	0.0478	0.1140
metab_543	pos	426.2841	3.5465	2.1052	5.7079	0.0008	0.0065
metab_5117	pos	426.2841	3.2727	2.3419	9.3321	0.0002	0.0027
metab_11803	neg	426.3021	8.6093	1.5932	-2.2009	0.0000	0.0000
metab_2462	pos	426.3196	5.3757	0.9508	0.9748	0.1060	0.1979
metab_12410	neg	426.3230	6.7122	0.0408	1.1035	0.9380	0.9595
metab_700	pos	426.3353	6.9638	0.0986	0.0273	0.8884	0.9295
metab_2722	pos	426.3355	7.2763	0.8732	0.6757	0.0035	0.0174
metab_13670	neg	426.9266	2.4651	1.6793	-1.4996	0.0015	0.0111
metab_9285	neg	426.9633	4.0516	1.8098	-3.3641	0.0000	0.0005
metab_13177	neg	427.1516	3.6645	0.6371	-1.2606	0.2283	0.3533
metab_10311	neg	427.1640	8.7191	0.8987	-0.7054	0.0001	0.0015
metab_9487	neg	427.1649	4.9349	0.5330	0.1987	0.4405	0.5645
metab_8348	neg	427.1725	1.5439	1.6871	-2.9757	0.0060	0.0276
metab_7008	neg	427.1920	6.4073	0.5988	1.2649	0.0988	0.1922
metab_1712	pos	427.2067	1.7993	1.4066	-2.4713	0.0367	0.0904
metab_12802	neg	427.2107	4.9349	0.1244	-0.0324	0.8051	0.8607
metab_5481	pos	427.2330	2.2361	0.0393	1.4236	0.9534	0.9701
metab_9530	neg	427.2339	5.2257	0.0840	0.2204	0.8483	0.8948
metab_7670	neg	427.2342	2.9931	1.5185	14.3661	0.0038	0.0204
metab_4927	pos	427.2585	4.0338	1.3881	12.9336	0.0211	0.0605
metab_2457	pos	427.2656	5.3311	1.1576	-2.1395	0.0620	0.1333
metab_1012	pos	427.2657	4.9115	1.3433	-2.9982	0.0349	0.0876
metab_2344	pos	427.2658	4.5485	1.3685	-3.2227	0.0069	0.0274
metab_11980	neg	427.2715	8.0688	1.2547	-1.4289	0.0001	0.0027
metab_7348	neg	427.2816	8.0843	0.6321	-0.3791	0.0409	0.1024
metab_12693	neg	427.2817	5.5002	0.6784	-0.1877	0.1886	0.3077
metab_9330	neg	427.2859	4.2203	2.1761	4.7884	0.0032	0.0180
metab_2571	pos	427.3051	6.1757	0.0236	0.2932	0.9622	0.9772
metab_1002	pos	427.3163	6.2372	0.3649	-0.3402	0.3844	0.5170
metab_12737	neg	427.3179	5.2579	0.5999	1.6074	0.3018	0.4322
metab_2605	pos	427.3191	6.5704	0.1058	-0.2191	0.6145	0.7227
metab_4637	pos	427.3634	5.4056	0.1505	1.3122	0.7611	0.8371
metab_7291	neg	427.3798	8.9574	0.6557	0.9700	0.2109	0.3336
metab_14270	neg	428.0208	1.5155	2.2445	-3.9855	0.0083	0.0349

metab_1421	pos	428.0355	0.9620	0.8562	0.9650	0.0284	0.0757
metab_6182	pos	428.0780	0.8920	1.2136	3.5887	0.0312	0.0809
metab_14309	neg	428.1316	1.4710	0.8841	-0.9310	0.0133	0.0479
metab_13952	neg	428.1346	1.9439	0.6972	3.1175	0.2048	0.3266
metab_9489	neg	428.1503	4.9510	0.2751	-0.5525	0.5737	0.6836
metab_13826	neg	428.1569	2.1630	2.0067	-3.6406	0.0000	0.0001
metab_13067	neg	428.1657	3.9850	0.3092	-0.1656	0.4066	0.5343
metab_5350	pos	428.1807	2.5314	1.0649	1.9573	0.0247	0.0683
metab_5633	pos	428.1948	1.9758	0.6501	-1.2304	0.1534	0.2603
metab_1645	pos	428.1949	1.6272	1.1197	-1.9746	0.0622	0.1336
metab_5483	pos	428.2037	2.2361	0.2903	-3.3184	0.7208	0.8070
metab_9879	neg	428.2812	7.0469	1.0383	1.6737	0.1139	0.2129
metab_11776	neg	428.2944	8.6728	1.0331	-1.2100	0.0022	0.0139
metab_2317	pos	428.2996	4.3818	0.6257	0.2518	0.2913	0.4215
metab_5213	pos	428.2998	2.9652	1.8788	15.1763	0.0012	0.0083
metab_12733	neg	428.3020	5.2741	0.0530	1.3351	0.9326	0.9561
metab_12778	neg	428.3021	5.0484	0.1692	0.9679	0.8132	0.8672
metab_11624	neg	428.3176	8.9903	1.0832	-1.0997	0.0032	0.0180
metab_685	pos	428.3360	6.6909	0.0839	0.5452	0.8615	0.9115
metab_2561	pos	428.3361	6.1009	0.7332	-0.9441	0.2312	0.3552
metab_12277	neg	428.3386	7.1266	0.4094	1.4341	0.4977	0.6183
metab_4252	pos	428.3510	7.4855	1.2107	-2.4214	0.0138	0.0449
metab_3833	pos	428.3725	9.3429	1.0661	-3.8393	0.1095	0.2029
metab_4917	pos	429.1171	4.0634	0.1780	-0.1827	0.4292	0.5608
metab_6791	neg	429.1256	0.8382	0.1525	-0.0645	0.8525	0.8977
metab_12810	neg	429.1589	4.9349	0.2288	-0.1177	0.6679	0.7605
metab_12725	neg	429.1589	5.3388	0.0990	0.0467	0.8626	0.9054
metab_2195	pos	429.1649	3.6684	0.8161	-2.8602	0.2450	0.3696
metab_1620	pos	429.1749	1.5424	0.5541	1.7483	0.3137	0.4466
metab_11709	neg	429.1955	8.7191	0.7250	-0.9271	0.0886	0.1780
metab_8383	neg	429.1996	1.5885	1.3249	3.1412	0.0007	0.0068
metab_9667	neg	429.2055	6.0024	1.5419	-4.1036	0.0070	0.0308
metab_9190	neg	429.2288	3.6980	1.7461	6.9573	0.0002	0.0033
metab_13525	neg	429.2360	2.7624	2.4100	6.6437	0.0000	0.0002
metab_12728	neg	429.2499	5.3060	1.8414	-2.8953	0.0007	0.0068
metab_12592	neg	429.2499	5.9375	0.4670	-0.0033	0.3296	0.4623
metab_9063	neg	429.2609	3.2473	2.0638	8.3218	0.0002	0.0029
metab_12043	neg	429.2613	7.8984	1.0173	-0.9577	0.0016	0.0112
metab_9804	neg	429.2613	6.7764	0.9994	1.6361	0.1152	0.2146
metab_12506	neg	429.2859	6.3269	0.7019	-1.4944	0.2478	0.3748
metab_12634	neg	429.2866	5.7420	0.0730	1.4251	0.8716	0.9120
metab_7341	neg	429.2871	8.1468	1.6399	-2.8810	0.0010	0.0086
metab_12419	neg	429.3011	6.6480	0.2656	0.3307	0.5719	0.6821
metab_12901	neg	429.3019	4.5884	1.5718	3.1742	0.0098	0.0392
metab_3997	pos	429.3189	8.6132	1.4176	-2.0572	0.0000	0.0008
metab_636	pos	429.3313	5.2717	0.7804	2.2019	0.1890	0.3043
metab_2784	pos	429.3328	7.7357	1.2187	-1.8044	0.0050	0.0220
metab_9632	neg	429.3338	5.7913	0.8544	1.5110	0.2315	0.3568

metab_4062	pos	429.3357	8.3609	0.9563	-1.3065	0.0006	0.0057
metab_2539	pos	429.3790	5.9361	0.1700	1.3774	0.7308	0.8142
metab_11631	neg	429.3861	8.9574	0.8382	1.2031	0.1440	0.2539
metab_7112	neg	430.1141	9.0872	1.0874	-0.8888	0.0013	0.0101
metab_9861	neg	430.1217	7.0162	1.2064	-2.6603	0.0759	0.1587
metab_7901	neg	430.1573	0.5851	0.4044	0.6794	0.3772	0.5075
metab_1304	pos	430.1922	0.5983	1.3868	14.4705	0.0042	0.0196
metab_8229	neg	430.2088	1.3669	0.1965	0.0000	0.2937	0.4247
metab_2441	pos	430.2214	5.1830	0.4681	-0.3765	0.0196	0.0573
metab_12825	neg	430.2261	4.8707	1.2461	2.8894	0.0250	0.0735
metab_9498	neg	430.2272	5.0318	1.3501	4.7279	0.0248	0.0732
metab_13237	neg	430.2450	3.4969	2.0514	8.0216	0.0001	0.0019
metab_9781	neg	430.2603	6.6480	0.6224	-0.7839	0.1880	0.3070
metab_5436	pos	430.2651	2.3447	1.3154	13.1689	0.0039	0.0182
metab_4716	pos	430.2689	4.9698	1.6169	3.5995	0.0057	0.0242
metab_9346	neg	430.2816	4.3037	0.0049	0.7768	0.9794	0.9857
metab_2712	pos	430.2925	7.2014	0.2613	-0.2006	0.6460	0.7486
metab_4339	pos	430.2940	7.0381	1.6805	3.7769	0.0083	0.0315
metab_4656	pos	430.3153	5.3311	0.6054	-0.6347	0.2556	0.3823
metab_4779	pos	430.3153	4.7001	0.2173	-0.4164	0.6905	0.7830
metab_4187	pos	430.3154	7.8389	0.8608	1.5527	0.1881	0.3038
metab_10088	neg	430.3181	7.9454	0.4985	-1.5276	0.2325	0.3577
metab_9548	neg	430.3184	5.3388	0.4273	4.5492	0.6422	0.7391
metab_3911	pos	430.3298	8.9958	0.5762	-0.7297	0.0835	0.1658
metab_11479	neg	430.3332	9.3991	1.4814	-2.3409	0.0001	0.0015
metab_124	pos	430.3517	7.1119	0.3491	1.4130	0.6374	0.7413
metab_10765	neg	430.9280	14.0282	0.1178	0.0335	0.7882	0.8484
metab_9277	neg	431.0729	4.0182	1.9650	4.5272	0.0002	0.0029
metab_14752	neg	431.1049	0.6831	0.2403	-0.1878	0.5626	0.6738
metab_7974	neg	431.1410	0.7818	0.0638	0.7801	0.6690	0.7614
metab_1045	pos	431.1439	1.6413	0.9658	-1.5923	0.0299	0.0785
metab_12924	neg	431.1618	4.5054	1.3484	-3.8486	0.0000	0.0003
metab_12736	neg	431.1727	5.2579	0.0271	1.5931	0.9358	0.9583
metab_13595	neg	431.1973	2.6206	1.6801	3.5974	0.0003	0.0039
metab_12761	neg	431.2114	5.1456	1.2869	3.1624	0.0170	0.0564
metab_12264	neg	431.2208	7.1595	0.9571	1.5012	0.0036	0.0196
metab_12587	neg	431.2211	5.9542	1.8277	-4.6232	0.0054	0.0258
metab_5278	pos	431.2494	2.7653	2.1390	13.0420	0.0000	0.0001
metab_12769	neg	431.2654	5.0967	1.6256	-3.2704	0.0092	0.0375
metab_6999	neg	431.2655	5.8731	1.2562	-2.2871	0.0050	0.0244
metab_9949	neg	431.2805	7.3659	0.5166	-1.6081	0.4131	0.5394
metab_12094	neg	431.2924	7.7251	0.2545	0.9247	0.7418	0.8139
metab_10376	neg	431.3134	9.0067	1.0070	-1.3166	0.0464	0.1117
metab_4127	pos	431.3146	8.1132	0.0555	-0.5373	0.9442	0.9654
metab_2522	pos	431.3470	5.7846	0.7961	1.4082	0.2068	0.3269
metab_6396	pos	431.9978	0.5560	1.2604	2.0340	0.0110	0.0383
metab_7402	neg	432.1383	6.4236	0.0720	0.3676	0.8472	0.8940
metab_353	pos	432.1423	1.5847	3.2653	14.2405	0.0000	0.0000

metab_8664	neg	432.1444	2.1798	0.0730	1.0389	0.9150	0.9437
metab_8212	neg	432.1448	1.3373	1.6378	3.0706	0.0000	0.0007
metab_1300	pos	432.1702	0.5983	0.4757	0.2964	0.1625	0.2721
metab_9062	neg	432.1802	3.2473	1.1140	-1.3636	0.0032	0.0180
metab_1262	pos	432.2209	0.5420	1.6373	17.1222	0.0009	0.0068
metab_4685	pos	432.2371	5.1830	0.3167	-0.3057	0.1254	0.2240
metab_2393	pos	432.2395	4.8809	0.6183	1.0398	0.1724	0.2845
metab_12954	neg	432.2607	4.3549	2.2610	4.5318	0.0002	0.0033
metab_2304	pos	432.2947	4.3371	1.8524	3.7527	0.0005	0.0048
metab_4919	pos	432.2947	4.0634	2.1723	5.6521	0.0000	0.0010
metab_12059	neg	432.2974	7.8193	0.0133	-0.0415	0.9283	0.9539
metab_11593	neg	432.3060	9.0713	1.0665	-1.3561	0.0727	0.1537
metab_1008	pos	432.3310	5.8597	1.1297	-2.2136	0.0047	0.0212
metab_4169	pos	432.3312	7.9551	0.7098	-1.3013	0.2286	0.3521
metab_10228	neg	432.3338	8.4821	1.7498	-2.8731	0.0007	0.0068
metab_12130	neg	432.9957	7.6323	0.4388	0.8993	0.2867	0.4182
metab_14481	neg	433.1107	1.2663	0.5580	-0.2921	0.2039	0.3255
metab_2424	pos	433.1900	5.0771	0.1174	-0.9924	0.7639	0.8396
metab_12129	neg	433.2123	7.6323	0.3318	0.6356	0.3947	0.5229
metab_12757	neg	433.2266	5.1610	0.9269	1.9324	0.0288	0.0807
metab_4315	pos	433.2339	7.1416	1.0513	1.4386	0.0156	0.0487
metab_133	pos	433.2342	5.9361	0.5718	-1.2512	0.2217	0.3442
metab_6627	neg	433.2366	7.5855	0.3377	0.6538	0.3250	0.4574
metab_12625	neg	433.2567	5.7751	1.2253	2.1650	0.1739	0.2907
metab_12870	neg	433.2590	4.6722	1.1909	3.2649	0.0279	0.0792
metab_9787	neg	433.2600	6.6803	1.2116	6.7023	0.1852	0.3038
metab_10214	neg	433.2804	8.4022	1.7847	-2.9170	0.0008	0.0071
metab_6670	neg	433.2811	6.0024	1.3075	-1.5602	0.0014	0.0108
metab_12401	neg	433.2957	6.7439	1.9330	-1.5155	0.0120	0.0445
metab_7036	neg	433.2961	7.1437	0.1658	0.6902	0.5939	0.7008
metab_2760	pos	433.3048	7.5307	0.5189	1.2701	0.3963	0.5291
metab_91	pos	433.3048	7.7062	0.0127	0.0780	0.8514	0.8827
metab_10008	neg	433.4789	7.6323	0.0422	0.2005	0.7943	0.8525
metab_7023	neg	434.1539	6.9046	0.9007	1.3472	0.0066	0.0295
metab_5954	pos	434.1579	1.3437	1.7390	3.2495	0.0001	0.0016
metab_14514	neg	434.1606	1.2377	1.2540	2.4355	0.0004	0.0046
metab_5125	pos	434.1943	3.2420	1.8695	-3.9196	0.0000	0.0010
metab_9095	neg	434.1971	3.3465	1.5364	-1.7120	0.0000	0.0013
metab_8334	neg	434.2299	1.5297	1.6168	-3.4985	0.0002	0.0034
metab_4683	pos	434.2432	5.1830	0.2540	-0.2721	0.2438	0.3684
metab_600	pos	434.2559	4.5642	1.3145	-0.0500	0.1921	0.3080
metab_4227	pos	434.2650	7.6185	1.6046	-14.7536	0.0023	0.0132
metab_4253	pos	434.2651	7.4703	1.3085	-13.9201	0.0060	0.0250
metab_4474	pos	434.2868	6.3885	0.2209	0.7067	0.7309	0.8142
metab_4962	pos	434.2890	3.8821	1.2454	1.4853	0.0006	0.0056
metab_11907	neg	434.2914	8.2577	0.7951	-0.9528	0.0906	0.1807
metab_11435	neg	434.3107	9.5478	1.8303	12.4605	0.0000	0.0013
metab_4529	pos	434.3467	6.0107	1.3626	-2.3292	0.0020	0.0120

metab_13838	neg	434.9905	2.1311	0.4838	-0.3700	0.2382	0.3642
metab_13600	neg	435.0418	2.6046	1.2672	-12.1487	0.0000	0.0010
metab_8771	neg	435.1347	2.4338	0.0966	1.3785	0.7496	0.8208
metab_9099	neg	435.1567	3.3803	1.0721	-1.5210	0.0438	0.1075
metab_13847	neg	435.1890	2.1153	0.0599	0.6565	0.9595	0.9727
metab_89	pos	435.2497	7.5450	0.2247	0.3276	0.5443	0.6638
metab_10120	neg	435.2525	8.0688	1.0324	1.8253	0.0057	0.0270
metab_11028	neg	435.2527	14.0610	1.2488	2.3750	0.0068	0.0304
metab_2613	pos	435.2709	6.6295	0.5795	-0.2861	0.2737	0.4019
metab_9712	neg	435.2716	6.2775	1.9307	7.2867	0.0127	0.0465
metab_4926	pos	435.2845	4.0479	0.8953	1.4628	0.0716	0.1486
metab_7011	neg	435.2967	6.5033	1.1001	-0.9569	0.0024	0.0146
metab_10541	neg	435.3096	9.6986	0.8354	2.1561	0.1379	0.2460
metab_12065	neg	435.3232	7.8026	1.8090	-2.0995	0.0022	0.0140
metab_7079	neg	435.3233	8.2744	1.1259	-0.9973	0.0275	0.0783
metab_10429	neg	435.3251	9.1200	0.2356	-0.2338	0.6395	0.7370
metab_10023	neg	435.3344	7.6951	1.2351	-1.2429	0.0035	0.0191
metab_13530	neg	436.0480	2.7475	0.2303	-0.0409	0.8163	0.8695
metab_8195	neg	436.0936	1.3089	1.1792	-1.5686	0.0003	0.0037
metab_1519	pos	436.1737	1.2446	1.1020	2.1086	0.0049	0.0217
metab_11984	neg	436.1802	8.0532	1.3526	-1.7861	0.0014	0.0106
metab_5094	pos	436.2101	3.3490	1.7754	-2.4995	0.0008	0.0064
metab_12133	neg	436.2451	7.6323	0.3586	0.7046	0.3189	0.4511
metab_643	pos	436.2673	5.3311	0.8353	1.2312	0.0789	0.1593
metab_581	pos	436.2683	4.2150	0.6164	0.5584	0.0883	0.1730
metab_12910	neg	436.2710	4.5553	1.8877	5.4575	0.0004	0.0051
metab_4389	pos	436.3025	6.8728	0.7694	1.8470	0.2218	0.3442
metab_2174	pos	436.3046	3.5620	0.4182	0.1223	0.4164	0.5483
metab_7319	neg	436.3077	8.6874	0.2528	0.2827	0.6712	0.7625
metab_4052	pos	436.3410	8.3912	0.4781	-1.1762	0.3113	0.4439
metab_3399	pos	436.3411	14.6892	0.2865	-0.2146	0.0362	0.0897
metab_6549	pos	436.3411	0.2389	0.2974	-0.2596	0.0554	0.1230
metab_14233	neg	437.0869	1.5439	0.5330	-0.0489	0.2495	0.3770
metab_7530	neg	437.0973	0.5991	1.3932	2.6066	0.0023	0.0145
metab_5142	pos	437.1381	3.2116	0.0886	-0.2122	0.8290	0.8875
metab_1904	pos	437.1479	2.4370	0.0225	1.3339	0.8752	0.9210
metab_7520	neg	437.1561	1.9125	1.1674	-1.1962	0.0004	0.0050
metab_2138	pos	437.1697	3.3793	1.0748	-1.7511	0.0606	0.1310
metab_1792	pos	437.1857	2.0828	1.0689	-1.9264	0.1477	0.2533
metab_4465	pos	437.1924	6.4035	0.1286	0.5908	0.7492	0.8288
metab_1801	pos	437.2015	2.1132	0.1352	0.4927	0.8805	0.9240
metab_12788	neg	437.2490	4.9838	1.4790	-1.7558	0.0155	0.0532
metab_2820	pos	437.2655	8.0700	1.1013	1.8252	0.0119	0.0406
metab_9376	neg	437.2664	4.4722	1.0933	1.7202	0.1125	0.2118
metab_10322	neg	437.2685	8.7346	1.2822	-1.6033	0.0000	0.0005
metab_12539	neg	437.2701	6.1644	2.1504	-3.8367	0.0002	0.0028
metab_12050	neg	437.3022	7.8657	1.7147	-2.4926	0.0053	0.0256
metab_11756	neg	437.3385	8.6874	0.6896	1.7131	0.3017	0.4322

metab_10192	neg	437.3387	8.3055	1.2045	-2.0941	0.0124	0.0459
metab_737	pos	437.3475	7.6776	0.3708	-0.4708	0.4358	0.5669
metab_811	pos	437.3592	9.0112	0.6473	0.9802	0.3952	0.5279
metab_2630	pos	437.3728	6.7975	1.3992	2.3370	0.0001	0.0019
metab_6621	neg	438.1036	6.4073	0.0391	0.5974	0.8400	0.8884
metab_14021	neg	438.1269	1.8205	1.0727	-1.5229	0.0210	0.0650
metab_7387	neg	438.1349	7.0322	1.0625	-1.7828	0.0398	0.1004
metab_10250	neg	438.1958	8.5299	0.2313	1.4499	0.6896	0.7775
metab_9424	neg	438.2138	4.6550	1.9616	-2.8497	0.0005	0.0053
metab_7059	neg	438.2630	7.7251	0.6352	0.9107	0.1158	0.2155
metab_9837	neg	438.2630	6.9191	1.2701	2.0193	0.0012	0.0097
metab_10048	neg	438.2858	7.7728	0.9065	2.7417	0.2663	0.3965
metab_12518	neg	438.2864	6.2617	0.5079	1.2557	0.3469	0.4798
metab_7083	neg	438.2866	8.3373	0.5227	0.9263	0.3240	0.4565
metab_10129	neg	438.3004	8.0843	1.3162	-1.7268	0.0809	0.1667
metab_9420	neg	438.9820	4.6380	1.5093	-2.5535	0.0000	0.0011
metab_6810	neg	439.0758	0.9368	0.0417	0.3761	0.6928	0.7797
metab_14799	neg	439.0864	0.6131	0.1898	0.1121	0.5032	0.6233
metab_5832	pos	439.1000	1.5565	0.6228	-0.2328	0.2605	0.3879
metab_6323	pos	439.1202	0.6262	1.7464	-4.6605	0.0000	0.0001
metab_9134	neg	439.1846	3.5141	0.7893	-0.9422	0.1087	0.2065
metab_13689	neg	439.2091	2.4338	0.9840	-1.7070	0.0429	0.1060
metab_8671	neg	439.2091	2.1956	1.1721	-2.0609	0.0513	0.1196
metab_13616	neg	439.2091	2.5748	1.4122	-1.9203	0.0370	0.0954
metab_9484	neg	439.2472	4.9349	1.4080	-2.5788	0.0258	0.0752
metab_4524	pos	439.2685	6.0252	0.9420	1.2050	0.0392	0.0951
metab_12843	neg	439.2818	4.7881	1.6212	-1.5162	0.0438	0.1075
metab_6977	neg	439.2819	5.0484	1.3128	-1.4089	0.0351	0.0919
metab_4834	pos	439.2831	4.4730	1.2974	2.7155	0.0067	0.0271
metab_4377	pos	439.2833	6.9035	1.5475	2.4191	0.0008	0.0064
metab_10388	neg	439.2858	9.0391	0.4230	-0.1692	0.0940	0.1854
metab_10470	neg	439.2859	9.3494	0.2155	-0.2243	0.5480	0.6603
metab_4009	pos	439.3174	8.5835	0.4232	-1.0082	0.2845	0.4146
metab_4517	pos	439.3309	6.1009	0.1909	0.5183	0.6965	0.7884
metab_4358	pos	439.3525	6.9638	0.4194	-0.5763	0.2406	0.3654
metab_11643	neg	439.3547	8.9252	1.0292	-1.3335	0.0567	0.1290
metab_11764	neg	440.0751	8.6874	1.2132	-1.1573	0.0003	0.0042
metab_8526	neg	440.1498	1.8682	0.0084	3.6627	0.9535	0.9697
metab_13808	neg	440.1696	2.1956	0.2950	0.1725	0.6849	0.7735
metab_13369	neg	440.1832	3.1115	1.0701	-2.1592	0.0496	0.1169
metab_5547	pos	440.2204	2.1290	1.0198	-1.3763	0.0211	0.0607
metab_12813	neg	440.2652	4.9183	0.5618	-0.4682	0.2311	0.3565
metab_90	pos	440.2758	7.7208	0.7739	0.8355	0.0657	0.1394
metab_9905	neg	440.2810	7.1595	1.0763	-0.6544	0.0617	0.1372
metab_10132	neg	440.3024	8.0998	0.0389	0.1864	0.7212	0.8001
metab_718	pos	440.3150	7.2164	0.1056	0.1512	0.9045	0.9391
metab_3233	pos	440.3570	11.6464	0.2885	-0.2701	0.1608	0.2696
metab_3238	pos	440.3570	12.0501	0.0550	-0.1463	0.8767	0.9214

metab_3181	pos	440.3571	10.3632	0.5523	-0.4913	0.0078	0.0302
metab_3226	pos	440.3572	11.2987	0.2214	-0.2209	0.1699	0.2812
metab_3580	pos	440.3573	11.0226	0.1176	-0.1577	0.5678	0.6839
metab_2686	pos	440.3573	7.0381	0.1252	-0.2521	0.6631	0.7614
metab_1222	pos	440.9175	0.4999	0.7993	-3.8174	0.0543	0.1213
metab_9423	neg	440.9789	4.6550	1.6426	-2.9637	0.0002	0.0031
metab_8295	neg	441.0053	1.4710	0.9702	-1.4740	0.1425	0.2519
metab_6799	neg	441.1256	0.8946	1.3706	-1.1822	0.0042	0.0215
metab_5397	pos	441.2224	2.4370	1.3145	-2.2515	0.0160	0.0495
metab_9859	neg	441.2532	7.0162	0.2082	1.5526	0.6264	0.7262
metab_10191	neg	441.2636	8.3055	1.1757	2.5608	0.0525	0.1217
metab_11711	neg	441.2638	8.7191	0.8370	2.7613	0.2129	0.3358
metab_4489	pos	441.2845	6.2824	0.0301	-0.0674	0.8896	0.9298
metab_12699	neg	441.2975	5.4844	0.2280	0.8675	0.7162	0.7964
metab_4438	pos	441.2982	6.5550	0.1179	-0.0715	0.8036	0.8699
metab_80	pos	441.2984	4.6544	0.9297	1.7277	0.0293	0.0774
metab_2631	pos	441.2988	6.8131	0.4806	0.2927	0.1048	0.1962
metab_4044	pos	441.2989	8.4214	0.8023	1.2271	0.0439	0.1033
metab_7312	neg	441.3012	8.7031	0.1174	0.3870	0.7717	0.8370
metab_9944	neg	441.3128	7.3508	1.5419	-3.5430	0.0000	0.0003
metab_12083	neg	441.3130	7.7567	0.0610	0.4739	0.8414	0.8897
metab_4202	pos	441.3311	7.7514	1.5532	4.3096	0.0054	0.0231
metab_9541	neg	441.3338	5.3060	0.1832	0.4052	0.7182	0.7977
metab_12796	neg	441.3339	4.9510	0.0262	1.4054	0.9638	0.9752
metab_10487	neg	441.3378	9.4812	1.3773	-2.8201	0.0125	0.0459
metab_11746	neg	442.0720	8.7031	1.2297	-1.2615	0.0003	0.0037
metab_10421	neg	442.0906	9.1200	0.4252	0.3643	0.3624	0.4949
metab_7951	neg	442.0942	0.6551	0.0774	0.2241	0.9595	0.9727
metab_7516	neg	442.1276	1.8833	1.1686	-1.8502	0.0162	0.0547
metab_11892	neg	442.1910	8.3055	1.4468	-1.7784	0.0028	0.0166
metab_9080	neg	442.2086	3.2972	2.3283	14.7339	0.0000	0.0013
metab_12563	neg	442.2640	6.0510	1.9195	-2.3784	0.0068	0.0303
metab_9611	neg	442.2820	5.6936	0.2938	0.2957	0.5396	0.6540
metab_2458	pos	442.2939	5.3454	0.1772	0.4425	0.6575	0.7565
metab_87	pos	442.2944	7.1416	0.5683	-0.7046	0.1643	0.2742
metab_649	pos	442.2961	5.7385	0.6359	0.7659	0.1101	0.2036
metab_12276	neg	442.2968	7.1266	1.4490	-1.0437	0.0332	0.0889
metab_12209	neg	442.2972	7.3659	1.5980	-1.5997	0.0542	0.1246
metab_12718	neg	442.3175	5.3713	0.2308	0.3422	0.7509	0.8216
metab_9592	neg	442.3177	5.6128	0.1272	0.7375	0.7928	0.8515
metab_2643	pos	442.3780	6.8728	0.4261	0.3981	0.3337	0.4679
metab_2611	pos	442.3781	6.6295	0.1264	-0.0041	0.7028	0.7925
metab_4806	pos	442.3993	4.5944	1.5761	1.5701	0.0555	0.1231
metab_10784	neg	442.8834	14.0438	0.4773	0.9502	0.2132	0.3361
metab_1403	pos	442.9641	0.8920	1.3549	3.6057	0.0000	0.0001
metab_6704	neg	443.1014	0.5126	1.8260	-2.5915	0.0346	0.0911
metab_8068	neg	443.1416	1.0357	1.6525	-13.6070	0.0018	0.0125
metab_8926	neg	443.1467	2.8269	0.6017	0.6742	0.3739	0.5048

metab_8870	neg	443.1467	2.6986	0.4479	-1.0282	0.3940	0.5224
metab_13908	neg	443.1497	2.0202	1.1116	2.0057	0.1202	0.2216
metab_12406	neg	443.2379	6.7439	0.1912	-0.1905	0.5508	0.6628
metab_7014	neg	443.2772	6.6320	0.8758	0.3027	0.1810	0.2991
metab_9270	neg	443.2781	3.9850	0.8250	1.1706	0.1090	0.2068
metab_5165	pos	443.2783	3.1191	2.2845	8.3423	0.0035	0.0173
metab_2180	pos	443.2785	3.5923	2.4580	8.8169	0.0001	0.0018
metab_4874	pos	443.2786	4.3060	2.0310	5.3375	0.0063	0.0258
metab_10117	neg	443.2787	8.0373	0.3882	0.7599	0.6108	0.7140
metab_12110	neg	443.2808	7.6951	0.3759	1.9557	0.6205	0.7217
metab_13159	neg	443.2811	3.6980	2.3195	5.8372	0.0011	0.0092
metab_12467	neg	443.3014	6.4396	1.2659	-1.2076	0.0254	0.0742
metab_12075	neg	443.3019	7.7875	1.0909	-1.2004	0.0095	0.0386
metab_645	pos	443.3123	5.5260	0.1550	-0.0031	0.5238	0.6450
metab_9507	neg	443.3128	5.1131	0.7938	2.5017	0.4060	0.5337
metab_9663	neg	443.3138	6.0024	0.4598	0.8989	0.3943	0.5226
metab_799	pos	443.3144	8.7161	0.8310	0.8189	0.0067	0.0271
metab_6574	neg	443.3171	8.4821	0.1605	0.3287	0.6969	0.7825
metab_10445	neg	443.3172	9.2185	0.1032	0.3639	0.7757	0.8402
metab_5121	pos	443.3224	3.2573	2.0359	13.3813	0.0000	0.0001
metab_4659	pos	443.3467	5.3165	0.3022	0.7650	0.6732	0.7697
metab_2402	pos	443.3469	4.9413	0.0176	1.9605	0.9987	0.9993
metab_12613	neg	443.3493	5.8409	0.5879	1.0401	0.4413	0.5652
metab_11837	neg	443.3745	8.4972	0.5328	0.9506	0.3554	0.4884
metab_11716	neg	444.0689	8.7191	1.2966	-1.4034	0.0003	0.0037
metab_11567	neg	444.0876	9.1200	0.4985	0.2903	0.2872	0.4188
metab_14477	neg	444.1151	1.2663	0.2775	0.7965	0.6512	0.7465
metab_6529	pos	444.1668	0.4855	0.6974	-0.5070	0.1980	0.3155
metab_13822	neg	444.1730	2.1630	1.4829	7.3732	0.0160	0.0543
metab_424	pos	444.1752	2.1746	0.1876	-0.1378	0.6507	0.7520
metab_1763	pos	444.2065	2.0062	1.5007	3.0268	0.0003	0.0032
metab_9912	neg	444.2760	7.1749	1.1979	10.7772	0.0012	0.0096
metab_4850	pos	444.2946	4.3818	2.3493	5.4352	0.0000	0.0002
metab_9698	neg	444.2975	6.1804	0.2679	0.9455	0.7336	0.8079
metab_2735	pos	444.3101	7.3521	0.1934	-1.6060	0.7317	0.8148
metab_4488	pos	444.3117	6.2824	0.4880	1.2034	0.2670	0.3945
metab_12216	neg	444.3126	7.3343	1.4977	-2.5409	0.0795	0.1647
metab_2405	pos	444.3306	4.9554	0.1989	-0.3574	0.8163	0.8793
metab_7241	neg	444.3336	9.8620	0.1243	0.3292	0.6468	0.7430
metab_12657	neg	444.3343	5.6615	0.4186	3.1957	0.5667	0.6777
metab_4235	pos	444.3453	7.5892	1.0059	-1.0700	0.0474	0.1097
metab_2867	pos	444.3458	8.3166	0.9314	1.4423	0.0392	0.0951
metab_2425	pos	444.4149	5.0928	1.2626	1.4530	0.0728	0.1505
metab_3120	pos	444.4191	9.8522	0.0954	-0.3166	0.7169	0.8035
metab_3130	pos	444.8140	9.9154	1.2671	1.8403	0.0005	0.0045
metab_1233	pos	445.0582	0.5140	0.1582	0.1138	0.5860	0.6991
metab_12986	neg	445.1146	4.2533	0.4368	-0.3020	0.1221	0.2242
metab_13080	neg	445.1147	3.9349	0.4045	-0.0922	0.0863	0.1745

metab_8263	neg	445.1405	1.4427	1.6699	2.4745	0.0205	0.0642
metab_14569	neg	445.1570	1.0927	0.4286	-0.0211	0.3667	0.4985
metab_6970	neg	445.2452	4.8707	0.3041	-0.3761	0.4673	0.5897
metab_10268	neg	445.2733	8.6093	3.1355	-4.6038	0.0004	0.0048
metab_12339	neg	445.2814	6.9191	1.3175	-11.8002	0.0054	0.0257
metab_12470	neg	445.2814	6.4236	1.4699	-3.7838	0.1036	0.1992
metab_12648	neg	445.2963	5.6936	0.7834	2.0871	0.1632	0.2781
metab_11725	neg	445.2965	8.7031	1.1927	1.7815	0.0137	0.0488
metab_9211	neg	445.2967	3.7830	1.9127	4.3485	0.0029	0.0168
metab_12011	neg	445.3081	7.9908	0.7997	-0.9036	0.0292	0.0813
metab_92	pos	445.3152	7.9835	1.0342	-1.5141	0.0101	0.0363
metab_11902	neg	445.3179	8.2897	0.4819	0.1323	0.2657	0.3958
metab_5007	pos	445.3265	3.6987	1.7191	4.3447	0.0156	0.0487
metab_669	pos	445.3267	5.9949	0.4689	0.7116	0.5120	0.6338
metab_3988	pos	445.3303	8.6284	0.1424	-0.0506	0.6488	0.7507
metab_3934	pos	445.3305	8.8904	0.0567	-0.1120	0.8924	0.9316
metab_4635	pos	445.3311	5.4056	0.5079	-1.6787	0.3583	0.4927
metab_11844	neg	445.3327	8.4821	0.1585	0.2421	0.7966	0.8541
metab_11477	neg	445.3328	9.3991	1.1111	-1.2853	0.0011	0.0092
metab_4570	pos	445.3625	5.8294	0.8704	1.5934	0.2538	0.3798
metab_6385	pos	445.9518	0.5703	0.0996	0.0028	0.6999	0.7907
metab_8200	neg	446.1243	1.3231	2.1308	5.4406	0.0000	0.0013
metab_14871	neg	446.1520	0.5711	0.1097	0.3260	0.7485	0.8203
metab_13078	neg	446.1577	3.9349	0.9278	-1.1534	0.1369	0.2445
metab_8412	neg	446.2302	1.6491	0.3688	-0.5713	0.4282	0.5534
metab_2439	pos	446.2527	5.1830	0.5388	-0.5278	0.0277	0.0744
metab_4580	pos	446.2528	5.7846	0.2862	-0.3050	0.1528	0.2597
metab_1725	pos	446.2586	1.8729	1.6206	6.5946	0.0048	0.0212
metab_4711	pos	446.2739	5.0009	0.1056	-0.1178	0.6979	0.7892
metab_9369	neg	446.2769	4.4055	1.3232	2.4753	0.0436	0.1071
metab_10137	neg	446.2880	8.1153	0.6212	0.1186	0.1793	0.2973
metab_2567	pos	446.2884	6.1311	0.4752	-0.4734	0.3304	0.4648
metab_2346	pos	446.3102	4.5642	1.6836	2.4123	0.0048	0.0212
metab_9441	neg	446.3131	4.7384	2.1474	6.0854	0.0000	0.0008
metab_722	pos	446.3252	7.3216	1.2372	-2.7514	0.1204	0.2176
metab_7332	neg	446.3488	8.4667	0.7336	1.5348	0.1993	0.3200
metab_11262	neg	446.3495	10.1910	0.4754	0.9899	0.3530	0.4862
metab_2852	pos	446.3831	8.2586	0.2956	-0.1919	0.6054	0.7153
metab_7187	neg	446.9064	14.0438	0.3280	-0.1574	0.1410	0.2498
metab_6740	neg	447.1361	0.6131	1.2391	-1.6092	0.0119	0.0444
metab_13807	neg	447.1530	2.1956	0.1742	1.1256	0.9422	0.9622
metab_12578	neg	447.2159	5.9866	0.2690	-0.4514	0.4898	0.6113
metab_9167	neg	447.2394	3.6142	1.9370	7.5972	0.0001	0.0016
metab_9963	neg	447.2530	7.4465	0.5659	0.6531	0.5485	0.6606
metab_4761	pos	447.2593	4.8359	0.6445	-0.1867	0.3875	0.5197
metab_9644	neg	447.2612	5.8896	1.3084	-3.0183	0.0638	0.1407
metab_9589	neg	447.2618	5.5966	1.6209	-4.6424	0.0244	0.0724
metab_9655	neg	447.2720	5.9542	0.1326	0.2747	0.7215	0.8003

metab_12508	neg	447.2757	6.3111	0.6028	-1.6383	0.3058	0.4371
metab_4494	pos	447.2817	6.2522	2.4143	-2.0186	0.0059	0.0246
metab_4437	pos	447.2819	6.5704	2.8028	-3.8343	0.0004	0.0042
metab_3970	pos	447.2859	8.7008	0.6488	0.6234	0.0499	0.1138
metab_4146	pos	447.3094	8.0419	1.1734	1.5873	0.0098	0.0354
metab_4595	pos	447.3095	5.6934	1.9776	8.1048	0.0183	0.0545
metab_11939	neg	447.3121	8.1623	0.0254	-0.4710	0.9700	0.9787
metab_12982	neg	447.3128	4.2702	1.2030	10.5292	0.0035	0.0190
metab_2336	pos	447.3421	4.4881	0.3580	0.1022	0.5734	0.6883
metab_2387	pos	447.3423	4.8208	0.1442	-0.1947	0.7830	0.8544
metab_3106	pos	447.3458	9.7899	0.3102	-0.2395	0.1627	0.2724
metab_3946	pos	447.3464	8.8169	0.9581	-0.9942	0.0260	0.0710
metab_5972	pos	448.1373	1.3153	1.7587	17.8722	0.0006	0.0056
metab_8118	neg	448.1400	1.1940	1.9355	6.2145	0.0007	0.0067
metab_6281	pos	448.1648	0.7102	0.0240	0.2068	0.9913	0.9944
metab_14028	neg	448.2459	1.8205	0.9035	-0.4758	0.1273	0.2313
metab_12977	neg	448.2462	4.2870	1.4605	2.1489	0.0904	0.1804
metab_12333	neg	448.2475	6.9354	0.9341	-0.4631	0.0710	0.1513
metab_9835	neg	448.2707	6.9046	1.3937	2.5442	0.0057	0.0267
metab_13096	neg	448.2822	3.8839	0.3143	0.4353	0.5063	0.6257
metab_2318	pos	448.2902	4.3818	1.0189	1.9166	0.1283	0.2278
metab_4482	pos	448.3024	6.3277	0.7306	-0.6691	0.3631	0.4969
metab_2371	pos	448.3260	4.6851	1.5110	-4.3289	0.0115	0.0395
metab_13757	neg	449.0061	2.2912	1.4116	-2.3065	0.0036	0.0195
metab_12583	neg	449.2060	5.9542	0.6953	-2.1212	0.2636	0.3936
metab_12791	neg	449.2219	4.9668	0.7238	-0.7876	0.1744	0.2913
metab_9402	neg	449.2222	4.5884	0.6631	-1.2954	0.1327	0.2386
metab_2547	pos	449.2288	5.9800	0.2846	-0.6996	0.4970	0.6206
metab_77	pos	449.2291	3.9876	0.4788	-2.0467	0.3407	0.4751
metab_9648	neg	449.2316	5.9375	0.5537	-0.7887	0.2435	0.3698
metab_12735	neg	449.2321	5.2579	1.3853	-2.3631	0.0015	0.0111
metab_9586	neg	449.2549	5.5808	0.4552	5.4173	0.6826	0.7719
metab_9264	neg	449.2552	3.9508	1.0647	11.7490	0.0135	0.0483
metab_7310	neg	449.2681	8.7031	1.6035	-3.2417	0.0014	0.0105
metab_5123	pos	449.2751	3.2420	1.8889	12.3211	0.0028	0.0151
metab_6617	neg	449.2763	4.5553	1.3563	-2.9216	0.0059	0.0274
metab_4306	pos	449.2880	7.1701	1.4709	-2.5531	0.0262	0.0714
metab_2657	pos	449.2882	6.9182	0.9852	-2.0195	0.0623	0.1338
metab_11847	neg	449.3130	8.4667	1.3699	-1.9159	0.0181	0.0590
metab_7243	neg	449.3254	9.8620	0.3103	0.1144	0.3118	0.4432
metab_10213	neg	449.3396	8.4022	1.3580	-1.6648	0.0117	0.0438
metab_12346	neg	450.1486	6.9046	1.6059	3.0841	0.0014	0.0103
metab_6058	pos	450.1529	1.1875	2.1604	7.1126	0.0014	0.0094
metab_11935	neg	450.1959	8.1774	1.6924	-2.0116	0.0002	0.0027
metab_9964	neg	450.2635	7.4618	0.6148	-0.0603	0.0734	0.1546
metab_12574	neg	450.2707	6.0024	1.1132	-1.2628	0.0116	0.0435
metab_576	pos	450.2952	4.1387	1.0302	1.5787	0.0091	0.0338
metab_2239	pos	450.2953	3.8967	0.5649	0.2884	0.3797	0.5121

metab_2058	pos	450.2953	3.0726	0.3003	2.2613	0.7023	0.7923
metab_12866	neg	450.2976	4.7046	0.3499	-0.0558	0.5057	0.6253
metab_12938	neg	450.2977	4.4221	2.4443	-4.1204	0.0031	0.0175
metab_9521	neg	450.2980	5.1772	0.5917	-0.2069	0.2303	0.3556
metab_7293	neg	450.3020	8.9417	1.0152	-1.4816	0.0477	0.1139
metab_10307	neg	450.3024	8.7191	0.9842	-1.6401	0.0438	0.1074
metab_3939	pos	450.3028	8.8618	1.3146	-5.9887	0.0077	0.0298
metab_4418	pos	450.3179	6.7062	0.1795	1.1655	0.7847	0.8555
metab_9808	neg	450.3230	6.7926	0.0199	-0.4579	0.9576	0.9719
metab_6658	neg	450.3356	8.6874	0.3090	0.5184	0.5834	0.6915
metab_4818	pos	450.3416	4.5485	1.5413	-3.6746	0.0012	0.0086
metab_10561	neg	450.3959	9.8129	0.6886	2.3477	0.1646	0.2799
metab_12289	neg	451.1434	7.0796	0.3662	0.2296	0.4634	0.5859
metab_13582	neg	451.1516	2.6357	0.9627	-1.5355	0.0850	0.1724
metab_10342	neg	451.1802	8.8128	1.0210	-0.1760	0.0488	0.1157
metab_5407	pos	451.2000	2.4071	0.6516	-1.5254	0.3343	0.4683
metab_5166	pos	451.2148	3.1191	1.4506	-2.9513	0.0845	0.1674
metab_4506	pos	451.2445	6.1614	0.1198	1.0551	0.8047	0.8706
metab_2140	pos	451.2468	3.3793	1.8601	6.1311	0.0051	0.0224
metab_12552	neg	451.2477	6.1320	0.5461	1.4954	0.3153	0.4472
metab_9847	neg	451.2478	6.9525	0.7111	2.0187	0.1126	0.2118
metab_12081	neg	451.2820	7.7567	0.0172	-0.8244	0.9704	0.9790
metab_6629	neg	451.2923	8.0373	0.6590	1.4627	0.1145	0.2138
metab_7362	neg	451.3035	7.9612	0.1680	-0.2036	0.5521	0.6641
metab_3973	pos	451.3171	8.6861	0.3909	-0.5066	0.3336	0.4678
metab_11861	neg	451.3179	8.4178	1.7547	-3.8278	0.0006	0.0061
metab_12119	neg	451.3181	7.6793	1.6975	-2.2094	0.0000	0.0010
metab_4568	pos	451.3270	5.8294	0.1587	2.2131	0.8002	0.8672
metab_9636	neg	451.3299	5.8074	0.5347	0.5212	0.3414	0.4745
metab_11667	neg	451.3551	8.8286	0.5496	1.7481	0.2676	0.3979
metab_3921	pos	451.3747	8.9507	0.5014	0.4540	0.4961	0.6197
metab_9241	neg	452.2426	3.8678	0.6335	-1.0902	0.2117	0.3344
metab_13025	neg	452.2427	4.1193	0.1996	-0.3938	0.6056	0.7095
metab_9535	neg	452.2658	5.2579	0.8724	1.9284	0.1353	0.2422
metab_4258	pos	452.2759	7.4561	0.1602	0.0194	0.5541	0.6721
metab_9933	neg	452.2786	7.3017	0.1157	0.2508	0.7856	0.8469
metab_7072	neg	452.2790	8.0998	1.1187	-1.1845	0.0001	0.0025
metab_2450	pos	452.2973	5.2717	0.4583	2.0639	0.4802	0.6065
metab_143	pos	452.2974	5.0009	0.5466	1.3051	0.3884	0.5207
metab_7829	neg	452.3096	0.5286	0.8449	0.1968	0.2777	0.4082
metab_2324	pos	452.3108	4.4126	1.7652	-3.9759	0.0018	0.0109
metab_4776	pos	452.3109	4.7156	0.6316	-0.5676	0.2966	0.4274
metab_12643	neg	452.3136	5.7102	0.1195	-0.1034	0.7621	0.8291
metab_3922	pos	452.3148	8.9507	1.0136	-1.8258	0.0588	0.1283
metab_3966	pos	452.3149	8.7161	0.9494	-2.1720	0.0974	0.1858
metab_11563	neg	452.3176	9.1200	1.8841	-4.2243	0.0005	0.0054
metab_3870	pos	452.3360	9.1747	0.1853	0.2374	0.6004	0.7105
metab_10354	neg	452.3388	8.8929	1.0412	2.0225	0.2129	0.3358

metab_3727	pos	452.4088	9.8049	0.4764	2.1553	0.3640	0.4974
metab_6773	neg	453.1022	0.6131	1.0619	1.8618	0.0007	0.0069
metab_11799	neg	453.1958	8.6093	0.8629	-0.7959	0.0206	0.0645
metab_10185	neg	453.1958	8.3055	1.1989	-1.5271	0.0031	0.0177
metab_11441	neg	453.2321	9.5314	0.1352	0.0924	0.6570	0.7516
metab_2566	pos	453.2608	6.1311	1.3867	4.8650	0.0864	0.1704
metab_9364	neg	453.2617	4.3716	0.4540	1.3536	0.4228	0.5488
metab_607	pos	453.2625	4.5944	1.6148	3.7077	0.0026	0.0144
metab_4655	pos	453.2625	5.3311	0.2791	0.8693	0.5696	0.6853
metab_12438	neg	453.2651	6.5829	1.6341	3.9542	0.0001	0.0027
metab_9610	neg	453.2652	5.6776	1.1151	1.9512	0.0115	0.0434
metab_9309	neg	453.2978	4.1193	0.0934	0.5230	0.9412	0.9617
metab_2666	pos	453.3202	6.9638	1.5611	13.8168	0.0037	0.0177
metab_4042	pos	453.3311	8.4214	1.8476	-15.1099	0.0000	0.0005
metab_4162	pos	453.3312	7.9835	1.8424	-3.3998	0.0006	0.0055
metab_4221	pos	453.3316	7.6626	1.5749	-13.4484	0.0003	0.0038
metab_4571	pos	453.3428	5.8148	0.9411	0.6029	0.1456	0.2508
metab_9669	neg	453.3454	6.0184	0.7737	0.3153	0.2676	0.3979
metab_4511	pos	453.3464	6.1311	0.1701	0.2279	0.5365	0.6569
metab_7733	neg	453.7867	0.0197	0.4286	-0.1731	0.0382	0.0978
metab_12287	neg	454.0547	7.0796	0.3644	-1.3234	0.6402	0.7375
metab_9482	neg	454.0988	4.9349	0.3765	-0.5953	0.4284	0.5535
metab_11733	neg	454.1144	8.7031	1.3295	-1.5520	0.0000	0.0001
metab_13867	neg	454.1294	2.0829	0.2818	2.1276	0.5319	0.6480
metab_6906	neg	454.1408	1.4568	0.3589	0.1858	0.4720	0.5944
metab_14534	neg	454.1834	1.1940	1.2661	-1.6790	0.0132	0.0477
metab_14301	neg	454.1837	1.4710	1.1464	-1.2802	0.0367	0.0948
metab_11657	neg	454.2273	8.8608	1.1493	-1.4471	0.0978	0.1911
metab_9893	neg	454.2600	7.1111	0.4558	0.1779	0.5149	0.6333
metab_2448	pos	454.2789	5.2573	1.2453	2.5448	0.0131	0.0431
metab_22	pos	454.2917	8.0988	1.0799	-1.4470	0.0008	0.0063
metab_4430	pos	454.2920	6.5857	1.2065	1.7429	0.0538	0.1206
metab_2782	pos	454.3617	7.7208	0.3261	-0.3567	0.5273	0.6480
metab_5074	pos	454.3630	3.4251	0.1074	0.6899	0.9006	0.9358
metab_4251	pos	454.3725	7.4855	0.2192	-0.2672	0.4573	0.5862
metab_3231	pos	454.3727	11.5256	0.3205	-0.3107	0.1026	0.1932
metab_3573	pos	454.3728	11.1596	0.4125	-0.3415	0.0252	0.0693
metab_3213	pos	454.3728	10.9933	0.2364	-0.2115	0.1833	0.2978
metab_9031	neg	454.9769	3.1286	1.8348	-3.1948	0.0002	0.0028
metab_8365	neg	455.0981	1.5736	0.2775	0.5267	0.3383	0.4714
metab_10753	neg	455.1028	14.0117	0.4096	0.5044	0.1209	0.2225
metab_1302	pos	455.1148	0.5983	2.0446	-4.2200	0.0003	0.0032
metab_14463	neg	455.1414	1.2805	0.4566	1.1984	0.3267	0.4590
metab_8471	neg	455.1686	1.7572	2.1246	-4.2390	0.0002	0.0029
metab_7832	neg	455.1890	0.5286	0.7132	-1.8886	0.3673	0.4988
metab_2283	pos	455.1999	4.1544	0.9510	2.6800	0.1255	0.2240
metab_10575	neg	455.2476	9.8947	0.2790	0.7412	0.4237	0.5495
metab_2039	pos	455.2492	2.9806	0.4960	1.6980	0.4472	0.5769

metab_13088	neg	455.2772	3.9005	0.9334	-0.5662	0.0777	0.1618
metab_2314	pos	455.2781	4.3818	1.6782	3.8588	0.0020	0.0120
metab_4599	pos	455.2781	5.6773	1.4681	2.8921	0.0024	0.0133
metab_9852	neg	455.2783	6.9688	1.0383	1.4997	0.0014	0.0103
metab_7336	neg	455.2789	8.4022	0.8360	-0.6397	0.0152	0.0524
metab_10420	neg	455.2791	9.1200	0.9184	-0.5257	0.0335	0.0894
metab_12779	neg	455.2807	5.0318	0.1908	0.7402	0.7289	0.8050
metab_9522	neg	455.2808	5.1772	0.3464	0.8236	0.3647	0.4967
metab_12688	neg	455.2809	5.5322	0.1558	0.6671	0.7374	0.8105
metab_12387	neg	455.2810	6.8093	0.3130	0.5266	0.2970	0.4276
metab_7326	neg	455.3133	8.6728	1.0693	-1.4297	0.0049	0.0241
metab_11704	neg	455.3495	8.7191	1.8782	-2.7139	0.0013	0.0102
metab_2568	pos	455.3535	6.1456	0.1670	0.2779	0.5933	0.7045
metab_2962	pos	455.3874	8.8759	1.0749	-1.5742	0.0191	0.0565
metab_9889	neg	456.0518	7.0796	0.5851	-1.0749	0.2174	0.3409
metab_7386	neg	456.0696	7.0162	0.8716	-3.0953	0.3177	0.4500
metab_5570	pos	456.1423	2.0828	0.3729	2.2006	0.4656	0.5932
metab_12486	neg	456.1459	6.4073	0.1274	0.6316	0.6633	0.7568
metab_6156	pos	456.1698	0.9620	1.3357	-13.9437	0.0028	0.0149
metab_1502	pos	456.1962	1.2016	1.0429	-1.9325	0.0254	0.0696
metab_1595	pos	456.1966	1.4860	0.6951	-1.4091	0.0974	0.1858
metab_13919	neg	456.1994	2.0055	0.2727	1.7269	0.8089	0.8641
metab_10217	neg	456.2071	8.4178	1.5553	-2.5853	0.0002	0.0029
metab_5702	pos	456.2145	1.7845	1.0422	4.4171	0.0695	0.1452
metab_12823	neg	456.2610	4.8707	1.6291	3.4708	0.0027	0.0160
metab_2008	pos	456.2701	2.8725	2.6738	10.8162	0.0000	0.0004
metab_2098	pos	456.2705	3.2274	2.1266	14.3714	0.0000	0.0007
metab_12529	neg	456.2763	6.2129	0.7726	-0.1907	0.3084	0.4392
metab_12034	neg	456.2980	7.9137	0.9487	1.5427	0.0278	0.0789
metab_675	pos	456.3094	6.4489	2.0213	6.4230	0.0000	0.0000
metab_12038	neg	456.3338	7.9137	1.9160	-3.4469	0.0002	0.0035
metab_5824	pos	457.1105	1.5705	0.3016	0.2877	0.2998	0.4310
metab_11713	neg	457.1140	8.7191	1.1571	-1.1210	0.0000	0.0002
metab_14697	neg	457.1206	0.8240	0.4208	0.7530	0.1719	0.2882
metab_8342	neg	457.1573	1.5439	0.1284	0.1718	0.8686	0.9097
metab_6372	pos	457.1674	0.5983	0.7652	-0.5105	0.0228	0.0640
metab_1699	pos	457.1811	1.7554	2.0112	-4.3392	0.0001	0.0016
metab_1558	pos	457.1903	1.3865	1.7815	5.5848	0.0025	0.0138
metab_6469	pos	457.2017	0.5140	1.5354	-3.7740	0.1405	0.2442
metab_4024	pos	457.2313	8.4949	0.3085	0.4898	0.4966	0.6203
metab_9538	neg	457.2601	5.2741	1.0710	1.9971	0.0542	0.1246
metab_11718	neg	457.2784	8.7191	0.9472	-0.7691	0.0042	0.0215
metab_10100	neg	457.2818	7.9908	1.4876	-2.0294	0.0000	0.0001
metab_9676	neg	457.2820	6.0670	1.2362	-1.5732	0.0081	0.0343
metab_13065	neg	457.2929	4.0020	0.2103	-0.2271	0.6829	0.7721
metab_12445	neg	457.2958	6.5512	0.9039	-0.8123	0.0218	0.0669
metab_7395	neg	457.2962	6.7439	0.4112	-0.2439	0.2375	0.3634
metab_10295	neg	457.2963	8.7031	0.6655	0.8520	0.0375	0.0965

metab_12568	neg	457.2963	6.0346	0.1415	0.4794	0.8442	0.8920
metab_10179	neg	457.2965	8.2897	0.6780	0.8173	0.0279	0.0791
metab_3913	pos	457.3201	8.9810	0.0999	0.3220	0.9281	0.9537
metab_10394	neg	457.3291	9.0557	0.6313	-0.6438	0.2703	0.4008
metab_7030	neg	458.0668	7.0162	0.9015	-3.2321	0.2809	0.4113
metab_9827	neg	458.1615	6.8887	0.8533	1.4029	0.0119	0.0444
metab_8574	neg	458.1676	1.9745	2.7302	-4.6266	0.0005	0.0053
metab_1990	pos	458.2223	2.8108	2.1697	5.8831	0.0001	0.0015
metab_1819	pos	458.2308	2.1595	0.8888	-0.9492	0.0626	0.1341
metab_4682	pos	458.2525	5.1830	0.1900	-0.3077	0.4600	0.5885
metab_11838	neg	458.2687	8.4972	0.8342	1.9673	0.2153	0.3384
metab_10130	neg	458.2691	8.0843	0.1958	0.5647	0.7323	0.8070
metab_11893	neg	458.2692	8.3055	0.0885	0.2519	0.7535	0.8230
metab_7422	neg	458.2772	4.2040	0.6262	1.0530	0.1241	0.2269
metab_12329	neg	458.2915	6.9525	1.2777	-1.8837	0.0125	0.0459
metab_12819	neg	458.3126	4.8865	0.8195	1.7251	0.0752	0.1576
metab_2975	pos	458.3466	8.9367	0.6485	-0.6488	0.0716	0.1485
metab_7092	neg	458.3495	8.6093	1.3757	-1.9083	0.0056	0.0266
metab_7188	neg	458.8623	14.0438	0.1865	0.0511	0.3960	0.5239
metab_15029	neg	458.8699	0.4975	0.7688	-0.7533	0.0211	0.0654
metab_14585	neg	459.0901	1.0499	0.4167	1.2253	0.4131	0.5394
metab_13005	neg	459.0940	4.2040	0.3962	-0.0593	0.0442	0.1080
metab_8004	neg	459.1367	0.8662	1.5389	-1.9550	0.0009	0.0077
metab_14501	neg	459.1730	1.2521	0.5661	1.4700	0.3229	0.4554
metab_6221	pos	459.1831	0.8361	1.6799	-3.3004	0.0004	0.0043
metab_9569	neg	459.2759	5.4844	0.3099	0.8813	0.4965	0.6173
metab_12337	neg	459.2760	6.9354	0.9256	3.6707	0.1926	0.3124
metab_12655	neg	459.2761	5.6615	1.5442	2.7790	0.0073	0.0318
metab_9806	neg	459.2762	6.7926	0.7788	2.9763	0.2600	0.3896
metab_7354	neg	459.2978	8.0532	1.4100	-2.8205	0.0017	0.0117
metab_655	pos	459.3094	5.8000	0.4174	0.1975	0.3141	0.4468
metab_4114	pos	459.3094	8.1560	0.4920	0.2768	0.2416	0.3666
metab_568	pos	459.3096	3.9577	1.1947	2.4411	0.0107	0.0375
metab_11720	neg	459.3122	8.7191	0.2755	0.3117	0.3978	0.5257
metab_10166	neg	459.3123	8.2253	0.3612	0.3986	0.2716	0.4021
metab_12067	neg	459.3124	7.8026	0.5655	0.5995	0.1466	0.2572
metab_674	pos	459.3782	6.4192	1.2155	1.9016	0.0166	0.0508
metab_14756	neg	460.1313	0.6551	1.3790	-3.7290	0.0375	0.0965
metab_13938	neg	460.1667	1.9745	1.4513	-2.5732	0.0009	0.0081
metab_8399	neg	460.1674	1.6186	2.3218	-3.8104	0.0063	0.0288
metab_14857	neg	460.1677	0.5851	0.2577	-0.1062	0.5862	0.6941
metab_9052	neg	460.1767	3.2130	1.2464	2.1997	0.0010	0.0085
metab_6273	pos	460.2012	0.7661	0.5188	-0.7402	0.4333	0.5646
metab_14530	neg	460.2058	1.1940	1.1525	5.3406	0.0071	0.0312
metab_1728	pos	460.2378	1.9014	1.4087	15.0701	0.0066	0.0269
metab_5442	pos	460.2465	2.3292	0.6664	-1.3625	0.4898	0.6146
metab_4583	pos	460.2682	5.7846	0.2346	-0.2441	0.1854	0.3003
metab_4681	pos	460.2684	5.1830	0.5758	-0.6013	0.0784	0.1584

metab_9643	neg	460.2708	5.8731	1.4405	-3.0012	0.0183	0.0593
metab_12760	neg	460.2755	5.1456	0.1380	0.0035	0.7877	0.8483
metab_10021	neg	460.2838	7.6951	1.8415	-3.7302	0.0000	0.0005
metab_11738	neg	460.2865	8.7031	0.8334	-1.0961	0.0586	0.1320
metab_594	pos	460.2897	4.4272	1.3492	1.8956	0.0060	0.0250
metab_12674	neg	460.3033	5.5966	0.6852	1.8351	0.3464	0.4795
metab_4493	pos	460.3047	6.2678	0.3598	0.4453	0.5490	0.6673
metab_12421	neg	460.3073	6.6480	0.5369	0.1338	0.2472	0.3741
metab_12965	neg	460.3120	4.3207	0.6577	2.9386	0.4185	0.5445
metab_10251	neg	460.3156	8.5455	0.7606	-1.3662	0.0752	0.1575
metab_2740	pos	460.3262	7.3824	0.3520	-0.3650	0.3667	0.5003
metab_4230	pos	460.3265	7.6185	0.0388	0.3270	0.9332	0.9579
metab_12080	neg	460.3281	7.7567	0.0457	-0.5085	0.8337	0.8834
metab_12767	neg	460.3287	5.1131	1.0792	3.4984	0.0788	0.1635
metab_7257	neg	460.3289	9.5314	1.2174	14.3005	0.0495	0.1168
metab_4335	pos	460.3412	7.0526	1.6108	3.2117	0.0003	0.0033
metab_5644	pos	460.7387	1.9463	1.7267	13.6374	0.0001	0.0022
metab_1348	pos	461.0445	0.7241	0.2668	0.5164	0.4300	0.5615
metab_6435	pos	461.0448	0.5280	0.6229	0.7349	0.0739	0.1518
metab_6735	neg	461.0473	0.5711	0.5142	1.9377	0.2419	0.3681
metab_14333	neg	461.1621	1.4568	0.7189	-0.4074	0.2437	0.3699
metab_9149	neg	461.1859	3.5641	0.9766	1.9475	0.1399	0.2486
metab_13806	neg	461.2150	2.1956	0.9033	4.3838	0.2924	0.4238
metab_2734	pos	461.2654	7.3370	1.0324	-3.1876	0.0322	0.0826
metab_12095	neg	461.2683	7.7251	0.2503	1.5132	0.7286	0.8048
metab_11901	neg	461.2684	8.2897	0.3151	0.2379	0.5855	0.6936
metab_12862	neg	461.2773	4.7217	0.8236	1.1385	0.1449	0.2550
metab_9638	neg	461.2883	5.8409	1.6047	3.3961	0.0000	0.0014
metab_6960	neg	461.2911	4.6216	1.2649	2.6583	0.0038	0.0204
metab_13170	neg	461.2917	3.6812	1.8792	4.5058	0.0009	0.0081
metab_9293	neg	461.2917	4.0685	1.9567	5.7354	0.0004	0.0047
metab_12205	neg	461.2923	7.3659	1.0934	2.8379	0.1777	0.2952
metab_4558	pos	461.3243	5.9208	0.0749	-0.5531	0.9601	0.9755
metab_582	pos	461.3253	4.1994	0.5505	0.5658	0.0823	0.1642
metab_12169	neg	461.3274	7.5239	0.1424	0.3019	0.7545	0.8233
metab_10163	neg	461.3284	8.2099	0.1034	0.0578	0.5255	0.6427
metab_6325	pos	462.0569	0.6262	1.5818	5.2216	0.0000	0.0010
metab_234	pos	462.0569	0.7801	1.8447	3.9183	0.0008	0.0065
metab_6809	neg	462.0674	0.9086	1.2392	-1.5011	0.0324	0.0873
metab_13830	neg	462.1192	2.1475	0.3242	1.4515	0.8140	0.8677
metab_1752	pos	462.1791	1.9758	1.1946	-1.8893	0.0078	0.0301
metab_1644	pos	462.1792	1.6272	1.2863	-2.2587	0.0011	0.0078
metab_6296	pos	462.1808	0.6542	0.3836	-0.7626	0.5153	0.6370
metab_2095	pos	462.1894	3.2274	1.2766	2.0943	0.0014	0.0093
metab_13523	neg	462.1922	2.7624	0.6031	-1.7537	0.4484	0.5718
metab_12257	neg	462.2634	7.1749	1.6407	3.5701	0.0005	0.0054
metab_9934	neg	462.2641	7.3180	1.0047	1.5469	0.0022	0.0139
metab_5713	pos	462.2699	1.7705	1.8247	4.7427	0.0545	0.1216

metab_7091	neg	462.2789	8.6239	1.6013	-2.5494	0.0000	0.0001
metab_760	pos	462.2968	8.1857	0.6818	0.5825	0.0159	0.0494
metab_740	pos	462.2970	7.6919	2.4021	-6.4614	0.0000	0.0001
metab_2939	pos	462.2990	8.7008	0.6223	-1.1966	0.1357	0.2381
metab_683	pos	462.3199	6.6295	0.2729	-0.0083	0.5298	0.6505
metab_9878	neg	462.3231	7.0469	0.3762	0.1106	0.4148	0.5410
metab_12517	neg	462.3234	6.2775	0.6988	3.3195	0.4121	0.5387
metab_4200	pos	462.3414	7.7654	1.0153	-2.6859	0.0903	0.1760
metab_11334	neg	462.3445	9.8947	1.1797	12.1663	0.0579	0.1309
metab_7893	neg	463.0442	0.5711	0.2289	1.9036	0.6511	0.7465
metab_1265	pos	463.0689	0.5420	0.9132	-1.4692	0.0345	0.0868
metab_14307	neg	463.1025	1.4710	1.4029	-2.1930	0.0073	0.0319
metab_12704	neg	463.2110	5.4522	0.4795	-0.2333	0.3844	0.5133
metab_9436	neg	463.2119	4.7046	1.0778	-2.2656	0.0403	0.1014
metab_12933	neg	463.2279	4.4722	0.4659	0.0128	0.1700	0.2864
metab_12176	neg	463.2473	7.5087	2.2894	-5.3326	0.0000	0.0005
metab_4007	pos	463.2807	8.5989	0.7753	2.1025	0.1252	0.2238
metab_11691	neg	463.2838	8.7488	1.0610	-1.0193	0.0002	0.0034
metab_10119	neg	463.2840	8.0532	1.4619	-1.6397	0.0112	0.0425
metab_6666	neg	463.2927	7.7875	1.0836	-0.7978	0.0225	0.0683
metab_321	pos	463.3001	1.4289	2.5365	-6.6370	0.0022	0.0128
metab_9723	neg	463.3032	6.3427	1.9175	5.2761	0.0004	0.0047
metab_4801	pos	463.3045	4.6096	2.2244	16.4667	0.0001	0.0013
metab_12048	neg	463.3064	7.8657	0.2334	-0.1533	0.6206	0.7217
metab_9373	neg	463.3073	4.4221	1.7749	5.3314	0.0036	0.0194
metab_7058	neg	463.3075	7.6951	0.1564	-0.0508	0.7518	0.8220
metab_671	pos	463.3254	6.1614	1.0283	2.1187	0.0708	0.1473
metab_688	pos	463.3405	6.8433	0.0944	-0.1295	0.8495	0.9030
metab_4855	pos	463.3414	4.3672	0.1062	0.3199	0.7178	0.8044
metab_11942	neg	463.3443	8.1468	1.1078	-1.6983	0.0021	0.0136
metab_2435	pos	463.3631	5.1533	0.0168	-0.0375	0.8975	0.9342
metab_6173	pos	464.0801	0.9200	1.3161	-1.8892	0.0311	0.0806
metab_6253	pos	464.1152	0.7801	0.2322	0.3146	0.6007	0.7107
metab_14787	neg	464.1411	0.6131	0.3905	0.8820	0.1719	0.2882
metab_9400	neg	464.1831	4.5717	1.5988	-2.3731	0.0013	0.0100
metab_9100	neg	464.2058	3.3803	0.2662	1.1216	0.6889	0.7768
metab_8903	neg	464.2076	2.7792	1.3833	3.0661	0.0162	0.0549
metab_10270	neg	464.2242	8.6093	1.5310	-4.1340	0.0006	0.0064
metab_2705	pos	464.2761	7.1560	0.1803	0.2427	0.7252	0.8108
metab_9178	neg	464.2769	3.6645	1.2412	-1.4083	0.0048	0.0237
metab_12054	neg	464.2790	7.8507	1.1972	1.8323	0.0005	0.0053
metab_131	pos	464.3361	6.2824	1.3024	8.1517	0.1502	0.2565
metab_7309	neg	464.3389	8.7031	0.9355	-1.7995	0.0446	0.1089
metab_11811	neg	464.3578	8.5604	1.9127	-1.7779	0.0162	0.0549
metab_11092	neg	464.8790	14.0282	0.3535	-0.1170	0.0816	0.1676
metab_1149	pos	465.0397	0.5560	0.4665	-0.4546	0.2262	0.3494
metab_14056	neg	465.1455	1.7729	1.1068	1.4533	0.0660	0.1438
metab_13557	neg	465.1883	2.6986	0.6990	-0.3075	0.1603	0.2746

metab_2351	pos	465.1884	4.5785	0.4316	-1.3459	0.3340	0.4682
metab_13068	neg	465.2266	3.9850	1.2895	-3.0383	0.0209	0.0650
metab_9525	neg	465.2267	5.2100	0.8461	-1.2250	0.0271	0.0776
metab_1895	pos	465.2336	2.4071	1.5442	12.0441	0.0580	0.1269
metab_9492	neg	465.2430	4.9838	1.1802	-1.3488	0.0069	0.0306
metab_4697	pos	465.2924	5.0928	0.0612	1.6481	0.9110	0.9427
metab_2476	pos	465.2926	5.4809	0.4853	0.7913	0.4793	0.6060
metab_4337	pos	465.2964	7.0381	0.2117	-0.0428	0.6241	0.7306
metab_4010	pos	465.2964	8.5835	0.4488	0.5349	0.2629	0.3905
metab_4455	pos	465.2967	6.4192	1.0269	1.7993	0.0357	0.0888
metab_9690	neg	465.2976	6.1320	1.2872	2.5714	0.0109	0.0419
metab_11638	neg	465.2977	8.9417	1.2035	-1.1259	0.0001	0.0022
metab_7084	neg	465.3076	8.4022	0.8925	-0.6065	0.0230	0.0694
metab_11971	neg	465.3192	8.0843	0.5008	-0.9772	0.2497	0.3770
metab_12663	neg	465.3227	5.6452	1.1133	5.5087	0.1513	0.2630
metab_12620	neg	465.3351	5.7913	0.3726	0.5357	0.4295	0.5545
metab_985	pos	465.3562	7.0816	1.2370	-2.3120	0.0028	0.0150
metab_3582	pos	465.8372	10.9933	1.9593	6.5713	0.0014	0.0094
metab_9172	neg	466.1624	3.6311	0.0835	-1.3969	0.8196	0.8720
metab_11742	neg	466.1670	8.7031	1.1367	-1.1684	0.0001	0.0015
metab_10466	neg	466.1671	9.3329	1.2222	-1.2053	0.0001	0.0015
metab_10080	neg	466.1909	7.9137	1.2502	-1.4494	0.0006	0.0059
metab_5274	pos	466.2205	2.7804	0.5502	1.5009	0.4369	0.5678
metab_11818	neg	466.2271	8.5455	1.2801	4.3019	0.0115	0.0433
metab_9394	neg	466.2662	4.5553	1.4690	-3.1470	0.0055	0.0263
metab_7342	neg	466.2821	8.1307	0.4969	-0.9333	0.4122	0.5388
metab_7353	neg	466.2833	8.0532	0.6641	-1.6044	0.1791	0.2970
metab_2194	pos	466.2903	3.6684	1.0364	-0.8400	0.1111	0.2049
metab_13056	neg	466.2929	4.0347	0.1271	0.9987	0.8108	0.8654
metab_11961	neg	466.2949	8.0998	0.5015	-0.5863	0.2137	0.3365
metab_12135	neg	466.2950	7.6169	1.4678	5.4358	0.0031	0.0176
metab_10220	neg	466.2952	8.4505	0.0384	0.0362	0.8887	0.9254
metab_11545	neg	466.3107	9.1522	0.9084	0.2817	0.1628	0.2777
metab_640	pos	466.3122	5.3913	0.3363	-0.0335	0.4835	0.6092
metab_2494	pos	466.3126	5.6168	0.2639	0.7695	0.7397	0.8212
metab_10756	neg	466.8742	14.0282	0.3606	-0.1281	0.0986	0.1921
metab_9278	neg	467.0496	4.0182	1.3208	3.2639	0.0093	0.0379
metab_9322	neg	467.2117	4.1869	1.7472	3.4438	0.0181	0.0590
metab_13079	neg	467.2118	3.9349	1.7730	3.2767	0.0287	0.0806
metab_10024	neg	467.2422	7.6951	0.6807	-0.3614	0.0867	0.1751
metab_10538	neg	467.2474	9.6986	0.7859	1.3078	0.0865	0.1748
metab_12653	neg	467.2583	5.6615	0.7954	-1.2449	0.2644	0.3945
metab_9170	neg	467.2964	3.6311	0.0465	0.9346	0.9967	0.9974
metab_2862	pos	467.3121	8.3018	0.8676	-1.2205	0.0101	0.0363
metab_796	pos	467.3122	8.6861	0.4326	-0.6320	0.1057	0.1975
metab_2708	pos	467.3355	7.1701	1.9505	6.1346	0.0000	0.0009
metab_10243	neg	467.3359	8.5135	0.5643	0.2123	0.3908	0.5192
metab_10411	neg	467.3861	9.1038	1.5339	-2.4435	0.0028	0.0167

metab_12482	neg	468.1455	6.4073	0.6895	1.1761	0.1256	0.2291
metab_12520	neg	468.1458	6.2456	0.4516	0.9527	0.3248	0.4574
metab_12480	neg	468.1822	6.4073	0.5387	1.5802	0.3129	0.4445
metab_11560	neg	468.1828	9.1200	0.7144	-0.3074	0.0569	0.1292
metab_9855	neg	468.2374	7.0002	0.0672	0.8627	0.9243	0.9508
metab_11616	neg	468.2428	9.0067	1.0404	-1.4230	0.0147	0.0513
metab_7825	neg	468.2567	0.5286	1.0263	-1.0420	0.1427	0.2523
metab_11992	neg	468.2828	8.0373	0.9987	1.8515	0.0098	0.0393
metab_10230	neg	468.2973	8.4972	0.7422	1.4005	0.1889	0.3080
metab_2259	pos	468.3060	4.0183	0.2148	0.8701	0.6963	0.7883
metab_4301	pos	468.3075	7.2014	0.2280	0.5143	0.4158	0.5477
metab_10330	neg	468.3331	8.7488	0.7600	1.6356	0.2056	0.3276
metab_786	pos	468.3671	8.5835	0.6480	7.0723	0.4228	0.5543
metab_862	pos	468.4045	9.8362	0.7165	-0.9179	0.1488	0.2548
metab_6462	pos	469.0118	0.5140	0.5652	1.9593	0.1392	0.2423
metab_9279	neg	469.0465	4.0182	1.6166	3.8630	0.0009	0.0077
metab_14370	neg	469.1574	1.4115	1.4386	3.7068	0.0023	0.0142
metab_13873	neg	469.1734	2.0670	0.0104	0.2748	0.9303	0.9549
metab_9772	neg	469.1903	6.5989	1.5812	-3.5096	0.0033	0.0181
metab_14970	neg	469.2044	0.5286	0.6693	-1.6202	0.4080	0.5354
metab_2436	pos	469.2563	5.1533	0.9234	1.2682	0.0533	0.1197
metab_2288	pos	469.2576	4.1853	2.4461	6.6530	0.0053	0.0230
metab_546	pos	469.2576	3.5771	2.2191	6.2762	0.0013	0.0087
metab_12611	neg	469.2587	5.8569	0.4210	0.7249	0.1942	0.3144
metab_9802	neg	469.2598	6.7603	1.6569	4.7153	0.0001	0.0018
metab_9600	neg	469.2599	5.6292	1.0136	1.8170	0.0092	0.0377
metab_9371	neg	469.2600	4.4221	1.5186	3.3459	0.0028	0.0163
metab_12245	neg	469.2921	7.2069	0.0955	0.0005	0.8837	0.9215
metab_11715	neg	469.2957	8.7191	0.7249	1.2961	0.0978	0.1911
metab_9851	neg	469.3175	6.9688	1.7699	4.5400	0.0012	0.0098
metab_9683	neg	469.3290	6.0993	0.7670	3.4170	0.2718	0.4023
metab_12732	neg	469.3400	5.2741	0.4869	1.4924	0.4330	0.5573
metab_2563	pos	469.3416	6.1167	1.0554	1.3269	0.0604	0.1307
metab_10349	neg	469.3653	8.8449	1.6686	-3.0502	0.0006	0.0061
metab_9605	neg	470.0493	5.6615	1.1649	-1.3305	0.0129	0.0470
metab_7947	neg	470.1519	0.6551	0.5768	0.9564	0.0593	0.1331
metab_14708	neg	470.1519	0.7958	0.6343	1.0986	0.0353	0.0922
metab_9834	neg	470.1616	6.9046	1.1632	1.4479	0.0623	0.1382
metab_12624	neg	470.1859	5.7751	1.0781	3.2506	0.0371	0.0956
metab_12353	neg	470.1979	6.8887	1.5549	3.0348	0.0047	0.0233
metab_6517	pos	470.2699	0.4999	1.2347	-2.3738	0.0922	0.1790
metab_10034	neg	470.2878	7.7251	0.6911	0.3203	0.1243	0.2271
metab_11857	neg	470.3134	8.4336	0.8480	-0.8630	0.2743	0.4047
metab_2876	pos	470.3617	8.3609	0.8070	-1.0607	0.0111	0.0385
metab_7887	neg	471.0761	0.5711	0.7700	-0.6739	0.0175	0.0575
metab_8106	neg	471.1363	1.1645	0.7033	1.5761	0.0700	0.1499
metab_1890	pos	471.1689	2.3908	1.3749	-2.6617	0.0048	0.0215
metab_6601	neg	471.1726	1.9904	1.3487	2.6799	0.0002	0.0033

metab_5574	pos	471.1863	2.0677	0.0265	0.2153	0.9075	0.9408
metab_12244	neg	471.2329	7.2069	0.0403	-0.0973	0.9715	0.9797
metab_10502	neg	471.2424	9.5478	1.4762	13.5384	0.0222	0.0677
metab_13434	neg	471.2468	2.9760	1.4728	3.6193	0.0411	0.1027
metab_595	pos	471.2731	4.3371	1.4555	2.9728	0.0009	0.0071
metab_7388	neg	471.2753	7.0002	0.5082	0.5177	0.2207	0.3447
metab_9968	neg	471.2756	7.4618	0.5492	0.5949	0.2890	0.4206
metab_12856	neg	471.2758	4.7546	1.2750	2.5036	0.0018	0.0122
metab_6947	neg	471.2758	4.2203	1.2877	2.8325	0.0065	0.0294
metab_10012	neg	471.3075	7.6479	1.4690	-2.3773	0.0081	0.0343
metab_12845	neg	471.3332	4.7881	1.3114	-1.8724	0.0172	0.0569
metab_7375	neg	471.3335	7.3343	0.4745	1.1776	0.4112	0.5384
metab_4666	pos	471.3532	5.2717	0.6937	1.4089	0.2989	0.4299
metab_12621	neg	471.3558	5.7913	0.1967	1.4697	0.7620	0.8291
metab_9606	neg	472.0464	5.6615	1.2362	-1.5209	0.0133	0.0479
metab_12510	neg	472.0648	6.2943	0.7007	-2.2095	0.3141	0.4458
metab_9656	neg	472.0650	5.9542	1.2261	-2.1021	0.1073	0.2045
metab_8230	neg	472.1038	1.3669	1.4365	-2.5170	0.0011	0.0089
metab_12807	neg	472.1406	4.9349	0.2597	-0.3683	0.6240	0.7243
metab_1696	pos	472.1868	1.7554	1.7549	5.6342	0.0249	0.0687
metab_5433	pos	472.2466	2.3600	1.1855	-0.9212	0.0431	0.1021
metab_11914	neg	472.3031	8.2253	0.0823	1.0854	0.8512	0.8969
metab_12576	neg	472.3285	5.9866	1.8609	-2.5941	0.0026	0.0157
metab_6585	neg	472.9253	0.5126	0.9339	-1.4303	0.0444	0.1084
metab_9410	neg	472.9663	4.6056	1.8478	-4.6723	0.0000	0.0006
metab_7806	neg	473.0244	0.5126	0.3211	0.6805	0.4458	0.5693
metab_14412	neg	473.1518	1.3373	0.4236	0.0647	0.2986	0.4290
metab_7897	neg	473.1628	0.5851	0.1184	0.2573	0.6861	0.7746
metab_1917	pos	473.1846	2.4850	1.3919	-2.2689	0.0046	0.0208
metab_8204	neg	473.1896	1.3231	2.3536	5.3893	0.0014	0.0106
metab_8788	neg	473.1929	2.4651	1.4880	-1.9326	0.0230	0.0694
metab_9461	neg	473.2563	4.8210	1.9813	4.5304	0.0046	0.0230
metab_4535	pos	473.2884	5.9800	1.7789	5.0038	0.0022	0.0126
metab_4894	pos	473.2889	4.1994	1.6313	3.7344	0.0015	0.0098
metab_9401	neg	473.2912	4.5884	1.0902	2.0674	0.0031	0.0177
metab_12134	neg	473.2913	7.6323	0.8259	1.0928	0.0196	0.0620
metab_6974	neg	473.2914	4.9510	1.0964	2.0438	0.0030	0.0174
metab_7355	neg	473.3121	8.0532	1.1293	-1.5075	0.0014	0.0104
metab_10249	neg	473.3270	8.5299	1.2423	-1.5317	0.0019	0.0129
metab_2618	pos	473.3938	6.7062	0.6461	1.4057	0.2891	0.4198
metab_7924	neg	473.5920	0.6131	1.7702	3.6003	0.0000	0.0013
metab_12590	neg	474.0622	5.9375	0.8487	-2.1246	0.4211	0.5471
metab_9737	neg	474.0802	6.4073	0.0969	0.6308	0.7013	0.7858
metab_14835	neg	474.1471	0.5991	0.4592	0.9750	0.2171	0.3408
metab_8250	neg	474.1738	1.4267	1.2096	2.3146	0.0033	0.0183
metab_13382	neg	474.1918	3.0774	0.6861	0.8101	0.3534	0.4864
metab_254	pos	474.2169	0.9480	0.1000	-0.3380	0.8801	0.9237
metab_14323	neg	474.2212	1.4568	1.3592	5.2850	0.0034	0.0185

metab_2443	pos	474.2477	5.1975	0.3612	-0.3741	0.2077	0.3275
metab_9923	neg	474.2630	7.2377	1.3022	1.9421	0.0000	0.0000
metab_2442	pos	474.2835	5.1975	0.6547	-0.6534	0.0359	0.0891
metab_2519	pos	474.2836	5.7846	0.4749	-0.4595	0.0432	0.1022
metab_4223	pos	474.3410	7.6481	1.0691	14.0048	0.0284	0.0757
metab_2323	pos	474.3414	4.3972	0.3504	-1.1494	0.4005	0.5337
metab_9411	neg	474.9632	4.6056	2.0500	-4.4330	0.0000	0.0013
metab_14769	neg	475.1309	0.6411	0.0991	0.1609	0.9355	0.9582
metab_8335	neg	475.1468	1.5297	0.3537	-0.2117	0.5583	0.6695
metab_14702	neg	475.1668	0.8098	0.7215	1.1925	0.0758	0.1585
metab_8197	neg	475.1707	1.3089	0.2185	0.6366	0.6238	0.7242
metab_7535	neg	475.2054	2.0055	1.0246	-1.9813	0.1606	0.2751
metab_457	pos	475.2063	2.4850	1.3862	-2.1417	0.0016	0.0099
metab_4289	pos	475.2444	7.2614	0.3383	0.2687	0.4773	0.6042
metab_2662	pos	475.2446	6.9336	0.3096	0.4381	0.6009	0.7109
metab_9413	neg	475.2713	4.6056	1.7213	3.9992	0.0017	0.0116
metab_11317	neg	475.2985	9.9606	2.2238	-4.7838	0.0000	0.0005
metab_620	pos	475.3041	4.9413	1.4029	2.7706	0.0009	0.0069
metab_4479	pos	475.3042	6.3581	1.3299	2.5619	0.0065	0.0264
metab_4300	pos	475.3043	7.2014	0.7459	0.5152	0.0790	0.1594
metab_4810	pos	475.3044	4.5785	1.3659	2.7176	0.0022	0.0128
metab_11664	neg	475.3072	8.8449	1.3012	2.4142	0.0007	0.0069
metab_10236	neg	475.3073	8.5135	1.0443	1.5186	0.0008	0.0071
metab_564	pos	475.3364	3.8821	1.3553	4.5679	0.0914	0.1777
metab_2419	pos	475.3730	5.0451	0.3048	0.6668	0.6332	0.7381
metab_2466	pos	475.3731	5.3913	0.7241	0.8689	0.1660	0.2764
metab_4317	pos	475.4096	7.1267	1.3236	4.0096	0.0160	0.0495
metab_12117	neg	476.0016	7.6793	0.6927	0.7684	0.0006	0.0061
metab_7403	neg	476.0772	6.4073	0.1300	0.6955	0.6422	0.7391
metab_9680	neg	476.0772	6.0831	0.5864	1.9058	0.1384	0.2465
metab_7024	neg	476.0958	6.8887	0.8059	1.2971	0.0171	0.0566
metab_13389	neg	476.1714	3.0604	1.6605	3.3570	0.0019	0.0127
metab_10184	neg	476.2510	8.2897	1.4754	2.1923	0.0096	0.0388
metab_12120	neg	476.2510	7.6793	0.4497	0.4822	0.0749	0.1572
metab_2713	pos	476.2761	7.2314	1.3146	1.7789	0.0000	0.0008
metab_4555	pos	476.2762	5.9361	0.1491	-0.8890	0.6875	0.7808
metab_7371	neg	476.2789	7.6793	0.6947	0.7759	0.0013	0.0099
metab_4907	pos	476.2854	4.0939	2.2673	12.2288	0.0008	0.0067
metab_9579	neg	476.3020	5.5639	0.1105	0.7811	0.7267	0.8032
metab_6986	neg	476.3021	5.3388	0.0541	0.3992	0.9728	0.9808
metab_1749	pos	476.3053	1.9609	1.2928	2.1150	0.0075	0.0294
metab_2756	pos	476.3353	7.5151	0.6785	-1.2813	0.1223	0.2201
metab_2562	pos	476.3837	6.1009	1.1355	5.0644	0.0337	0.0853
metab_12116	neg	476.5577	7.6793	0.5943	0.5886	0.0139	0.0494
metab_8025	neg	477.1004	0.9227	0.1493	0.5393	0.7430	0.8149
metab_14828	neg	477.1464	0.5991	0.8591	-0.7663	0.0226	0.0684
metab_14066	neg	477.1996	1.7572	0.2735	3.3519	0.6123	0.7154
metab_11975	neg	477.2245	8.0688	1.7368	-12.1298	0.0000	0.0000

metab_14395	neg	477.2248	1.3669	0.7654	-10.9281	0.0657	0.1434
metab_2768	pos	477.2611	7.6332	1.0126	-0.9350	0.0307	0.0800
metab_7044	neg	477.2628	7.3343	0.7501	-1.0357	0.0646	0.1417
metab_12331	neg	477.2821	6.9525	0.6213	-0.6200	0.0625	0.1385
metab_4771	pos	477.3201	4.7608	1.4193	2.9097	0.0054	0.0231
metab_5055	pos	477.3204	3.5162	1.5771	3.4140	0.0032	0.0164
metab_4995	pos	477.3204	3.7447	1.2616	2.6432	0.0108	0.0377
metab_12372	neg	477.3206	6.8565	0.8402	1.1655	0.0306	0.0841
metab_11949	neg	477.3212	8.1307	0.4505	-0.8909	0.2683	0.3984
metab_9758	neg	477.3223	6.5033	0.5935	0.9344	0.1273	0.2314
metab_11741	neg	477.3229	8.7031	0.8228	-0.8459	0.0104	0.0407
metab_10390	neg	477.3239	9.0391	0.9583	1.7953	0.0387	0.0985
metab_11785	neg	477.3705	8.6239	1.5985	-2.5523	0.0014	0.0106
metab_4689	pos	477.3787	5.1533	0.1326	-0.1557	0.5175	0.6393
metab_7022	neg	478.0928	6.8887	0.7749	1.3052	0.0247	0.0730
metab_14276	neg	478.1406	1.5155	1.2363	-1.7588	0.0664	0.1443
metab_6075	pos	478.1742	1.1589	1.3041	-1.4170	0.0110	0.0383
metab_2054	pos	478.1843	3.0572	1.7958	3.7607	0.0007	0.0061
metab_11841	neg	478.2276	8.4972	1.2189	-1.5832	0.0103	0.0405
metab_11922	neg	478.2661	8.1936	1.1312	10.3189	0.0000	0.0009
metab_5318	pos	478.2682	2.6244	2.1163	7.0003	0.0027	0.0145
metab_987	pos	478.2918	6.9477	0.4373	-0.4833	0.1156	0.2108
metab_121	pos	478.2918	7.6776	0.7143	0.6135	0.0060	0.0251
metab_7338	neg	478.2949	8.1936	1.2480	1.9876	0.0003	0.0040
metab_12659	neg	478.3141	5.6615	0.9090	2.0345	0.0169	0.0561
metab_2543	pos	478.3151	5.9504	0.3117	1.2605	0.6712	0.7682
metab_12144	neg	478.3397	7.5855	0.8575	-2.2478	0.1717	0.2881
metab_3743	pos	478.3671	9.7586	1.0900	1.7150	0.0140	0.0452
metab_2706	pos	478.7741	7.1560	1.4036	-2.5563	0.0636	0.1358
metab_11048	neg	478.8304	14.0438	0.4927	-0.1204	0.0085	0.0356
metab_13717	neg	479.1248	2.3855	2.7190	11.3531	0.0000	0.0002
metab_9307	neg	479.2228	4.1193	0.5778	-0.3080	0.0536	0.1234
metab_8964	neg	479.2306	2.9429	0.8766	10.7820	0.0588	0.1323
metab_12937	neg	479.2516	4.4221	1.7074	4.4204	0.0056	0.0266
metab_12398	neg	479.2516	6.7603	1.4911	9.2073	0.1104	0.2087
metab_10320	neg	479.2791	8.7346	1.0289	-1.0251	0.0182	0.0592
metab_4602	pos	479.3202	5.6623	0.9093	1.8451	0.0593	0.1289
metab_6625	neg	479.3230	6.1644	0.9416	2.2323	0.0350	0.0917
metab_10188	neg	479.3498	8.3055	1.9000	-3.1758	0.0019	0.0127
metab_8148	neg	480.0668	1.2521	1.6397	-3.2117	0.0038	0.0204
metab_1272	pos	480.0899	0.5560	1.8391	3.7059	0.0006	0.0055
metab_9064	neg	480.2009	3.2473	0.6740	1.3313	0.1745	0.2913
metab_11990	neg	480.2062	8.0373	1.5045	-2.1420	0.0001	0.0017
metab_11647	neg	480.2432	8.9086	0.1388	0.3079	0.8193	0.8718
metab_2717	pos	480.3078	7.2458	1.0155	1.0689	0.0028	0.0150
metab_4112	pos	480.3078	8.1857	1.2548	1.8247	0.0009	0.0069
metab_12003	neg	480.3099	8.0069	0.0891	0.2311	0.7115	0.7934
metab_10333	neg	480.3100	8.7640	0.8697	-0.7099	0.0001	0.0020

metab_2335	pos	480.3244	4.4730	0.6795	0.9412	0.2377	0.3619
metab_2592	pos	480.3307	6.4035	0.6945	2.1091	0.3040	0.4358
metab_7089	neg	480.3337	8.5604	1.4523	-1.7336	0.0003	0.0043
metab_4210	pos	480.3521	7.7062	1.4411	-1.6108	0.0694	0.1451
metab_15111	neg	480.7933	0.0197	0.1566	0.0871	0.3391	0.4724
metab_1394	pos	481.0943	0.8780	0.8407	-0.7238	0.1790	0.2928
metab_14684	neg	481.0964	0.8522	0.8003	1.5722	0.0897	0.1795
metab_447	pos	481.1378	2.3908	1.2762	2.4367	0.0102	0.0365
metab_6213	pos	481.1378	0.8500	0.9373	-1.0977	0.0372	0.0913
metab_6925	neg	481.2385	4.0516	1.0320	-1.1875	0.0021	0.0135
metab_11352	neg	481.2632	9.8620	0.1294	0.4412	0.7542	0.8233
metab_1913	pos	481.2646	2.4690	0.8945	4.6044	0.2393	0.3637
metab_2549	pos	481.2912	5.9949	1.0467	1.1804	0.0580	0.1269
metab_6661	neg	481.2942	8.4667	0.5046	0.1293	0.2388	0.3648
metab_4214	pos	481.3010	7.6776	0.7720	0.7203	0.0099	0.0358
metab_12720	neg	481.3178	5.3546	1.6640	7.5518	0.0084	0.0351
metab_9391	neg	481.3180	4.5391	0.9741	4.9854	0.0726	0.1537
metab_9446	neg	481.3180	4.7546	1.5988	12.9034	0.0074	0.0320
metab_10447	neg	481.4018	9.2350	1.6918	-3.3249	0.0022	0.0140
metab_7741	neg	481.7928	0.0197	0.4075	-0.1932	0.0957	0.1881
metab_12201	neg	482.2533	7.4148	0.8871	1.4077	0.0242	0.0718
metab_4491	pos	482.2862	6.2678	0.3713	0.0591	0.5966	0.7069
metab_9197	neg	482.2879	3.7488	1.0290	2.5993	0.0605	0.1350
metab_10211	neg	482.2975	8.4022	0.8074	-0.4920	0.0533	0.1228
metab_2949	pos	482.3228	8.7590	1.2609	-2.3601	0.0085	0.0322
metab_4231	pos	482.3234	7.6185	1.3546	2.0846	0.0000	0.0007
metab_2410	pos	482.3401	4.9856	1.5401	-2.2433	0.0118	0.0404
metab_784	pos	482.3462	8.5554	1.4268	-2.2938	0.0000	0.0008
metab_7109	neg	482.3492	8.9574	0.8803	-0.3073	0.0493	0.1164
metab_7268	neg	482.3858	9.4156	0.1466	0.3745	0.8693	0.9102
metab_7727	neg	482.7897	0.0197	0.3609	-0.0865	0.0694	0.1491
metab_14184	neg	483.1729	1.5885	1.5949	3.5326	0.0213	0.0659
metab_1493	pos	483.1898	1.1875	0.7605	-2.0391	0.1917	0.3074
metab_13266	neg	483.2063	3.3803	1.9264	12.9712	0.0019	0.0129
metab_11955	neg	483.2176	8.1153	1.1641	-1.1046	0.0239	0.0711
metab_8496	neg	483.2202	1.8046	1.6267	-4.5576	0.0000	0.0000
metab_6937	neg	483.2372	3.9850	0.5267	-1.0298	0.2238	0.3481
metab_9999	neg	483.2726	7.6009	1.7099	-2.6075	0.0000	0.0001
metab_10625	neg	483.2788	10.1910	0.2379	0.1659	0.4878	0.6095
metab_9984	neg	483.2899	7.5397	2.1763	-2.1659	0.0110	0.0421
metab_7324	neg	483.2916	8.6874	0.4235	0.0086	0.2540	0.3825
metab_10425	neg	483.2917	9.1200	0.7269	0.3690	0.2102	0.3328
metab_2532	pos	483.3070	5.8753	0.9836	0.8875	0.0662	0.1402
metab_6632	neg	483.3094	8.9903	0.8403	-0.4477	0.0818	0.1679
metab_2714	pos	483.3212	7.2314	0.8156	1.4141	0.1222	0.2200
metab_7082	neg	483.3234	8.3528	1.3812	-2.0619	0.0102	0.0402
metab_3901	pos	483.3800	9.0397	0.0929	2.7135	0.8645	0.9133
metab_12746	neg	484.1414	5.2100	0.0810	0.3748	0.9252	0.9513

metab_12804	neg	484.1414	4.9349	0.1414	0.0246	0.8152	0.8686
metab_6824	neg	484.1679	1.0215	0.0250	0.5812	0.9889	0.9918
metab_12801	neg	484.1770	4.9349	0.0264	0.4859	0.9855	0.9896
metab_12585	neg	484.2014	5.9542	0.0960	0.9641	0.8863	0.9237
metab_495	pos	484.2356	2.9192	0.3823	1.2014	0.5858	0.6991
metab_9054	neg	484.2380	3.2130	1.3726	3.9235	0.0275	0.0783
metab_725	pos	484.2656	7.3964	0.8260	1.1218	0.0457	0.1067
metab_8410	neg	484.2784	1.6491	1.9812	7.1995	0.0000	0.0001
metab_2212	pos	484.3007	3.7447	1.0325	2.5341	0.0662	0.1402
metab_2612	pos	484.3021	6.6295	0.4387	0.2658	0.4270	0.5586
metab_12371	neg	484.3077	6.8565	0.7251	-0.9984	0.1164	0.2164
metab_10049	neg	484.3400	7.7728	0.5093	-0.1039	0.6586	0.7529
metab_3237	pos	484.3829	11.9758	0.0945	-0.1161	0.6000	0.7102
metab_4338	pos	484.3832	7.0381	0.2736	-0.2153	0.3258	0.4597
metab_3566	pos	484.3833	11.3128	0.2596	-0.2133	0.1212	0.2188
metab_13510	neg	485.1883	2.7961	1.2245	2.6631	0.0014	0.0105
metab_9198	neg	485.2225	3.7488	2.0951	4.6232	0.0012	0.0098
metab_4964	pos	485.2523	3.8666	2.1314	8.6508	0.0017	0.0104
metab_12998	neg	485.2553	4.2203	1.2666	2.9042	0.0026	0.0158
metab_9403	neg	485.2554	4.5884	1.0909	2.3356	0.0129	0.0470
metab_9871	neg	485.2792	7.0322	0.4616	2.8418	0.4570	0.5804
metab_7073	neg	485.2838	8.1153	0.5575	-0.3114	0.0098	0.0392
metab_12211	neg	485.3031	7.3508	1.6835	-3.6863	0.0000	0.0001
metab_4280	pos	485.3095	7.3071	0.4431	0.6929	0.4575	0.5863
metab_4394	pos	485.3221	6.8582	0.0385	-0.1439	0.9242	0.9510
metab_10040	neg	485.3239	7.7251	0.9765	1.4524	0.2017	0.3229
metab_10317	neg	485.3468	8.7191	0.6763	3.1471	0.1980	0.3187
metab_9978	neg	485.3486	7.5239	1.2080	13.1614	0.0142	0.0501
metab_14797	neg	486.1469	0.6131	0.8079	1.6643	0.0498	0.1171
metab_7933	neg	486.1820	0.6131	1.1378	4.2320	0.0011	0.0093
metab_60	pos	486.2256	2.1444	0.1224	-0.7551	0.8556	0.9075
metab_2088	pos	486.2510	3.2116	1.2658	3.0240	0.0546	0.1217
metab_464	pos	486.2620	2.5469	0.0137	-0.9123	0.8992	0.9348
metab_485	pos	486.2624	2.8259	1.0156	0.7705	0.0961	0.1839
metab_656	pos	486.2838	5.7846	0.1075	-0.2040	0.6542	0.7547
metab_4679	pos	486.2840	5.1830	0.1458	0.1285	0.5980	0.7085
metab_5786	pos	486.2911	1.6413	1.8586	5.2314	0.0000	0.0003
metab_10077	neg	486.3076	7.9137	1.4123	2.8177	0.0026	0.0159
metab_4336	pos	486.3178	7.0526	0.0729	-0.0139	0.8748	0.9209
metab_4043	pos	486.3567	8.4214	0.7373	1.7674	0.1120	0.2058
metab_11985	neg	486.3806	8.0532	0.0931	-0.3877	0.8319	0.8822
metab_12902	neg	486.3921	4.5884	1.3005	1.6536	0.0632	0.1396
metab_15013	neg	487.0401	0.5126	0.9122	-1.4041	0.0406	0.1018
metab_6737	neg	487.1785	0.5991	0.9159	1.4021	0.0010	0.0086
metab_7861	neg	487.2147	0.5431	0.3536	0.4549	0.5725	0.6827
metab_13066	neg	487.2395	3.9850	0.2128	0.4606	0.6601	0.7545
metab_547	pos	487.2682	3.5771	2.5298	6.6585	0.0029	0.0154
metab_509	pos	487.2684	3.1495	2.1397	8.3264	0.0000	0.0009

metab_12099	neg	487.2705	7.7100	0.0159	0.2694	0.8287	0.8796
metab_9874	neg	487.2709	7.0322	0.3288	0.3606	0.5199	0.6378
metab_9360	neg	487.2709	4.3716	1.2858	2.6962	0.0018	0.0124
metab_7360	neg	487.2923	7.9908	1.4959	-2.1885	0.0002	0.0033
metab_7399	neg	487.3069	6.5190	1.1620	1.7032	0.0019	0.0126
metab_4164	pos	487.3260	7.9695	0.9786	-1.2966	0.0425	0.1011
metab_2214	pos	487.3480	3.7447	0.3030	2.9563	0.7350	0.8174
metab_11242	neg	487.3798	10.3031	0.5930	-0.3385	0.0236	0.0705
metab_8580	neg	488.1626	1.9904	1.4222	3.0311	0.0003	0.0038
metab_7533	neg	488.1627	0.5991	0.3945	-0.3865	0.3196	0.4519
metab_7081	neg	488.1648	8.3209	1.6943	-2.3784	0.0020	0.0133
metab_8672	neg	488.2256	2.1956	0.0486	0.8705	0.8925	0.9281
metab_12089	neg	488.2327	7.7251	0.6327	-2.1514	0.2255	0.3502
metab_4579	pos	488.2998	5.7846	0.2461	-0.2810	0.4773	0.6042
metab_11681	neg	488.3356	8.7640	0.4399	0.0964	0.4692	0.5916
metab_2907	pos	488.3723	8.5554	0.5301	0.8609	0.3344	0.4683
metab_2640	pos	488.3723	6.8582	0.6814	0.4642	0.0882	0.1730
metab_12771	neg	488.4074	5.0967	1.0844	1.5576	0.0908	0.1810
metab_14962	neg	489.0504	0.5286	0.6421	-0.4800	0.0853	0.1728
metab_6835	neg	489.1473	0.7818	0.6987	-0.6683	0.1764	0.2936
metab_14502	neg	489.1832	1.2521	0.4030	0.8385	0.1698	0.2862
metab_1072	pos	489.2332	1.3437	1.3838	2.2561	0.0007	0.0059
metab_12844	neg	489.2507	4.7881	0.3277	0.6140	0.5569	0.6684
metab_9372	neg	489.2511	4.4221	1.1889	2.8677	0.0337	0.0896
metab_2255	pos	489.2523	3.9876	0.1682	-0.2522	0.6194	0.7268
metab_591	pos	489.2837	4.3818	1.3206	2.1349	0.0001	0.0014
metab_2197	pos	489.2838	3.6835	1.9928	4.9324	0.0009	0.0070
metab_12177	neg	489.2853	7.4927	0.5819	0.6706	0.3483	0.4812
metab_12269	neg	489.2863	7.1437	0.9416	1.1647	0.0748	0.1571
metab_6936	neg	489.2867	3.7830	1.4521	3.1037	0.0005	0.0057
metab_3906	pos	489.3029	9.0112	0.0602	-0.4445	0.8108	0.8749
metab_12501	neg	489.3073	6.3427	1.6020	-2.5978	0.0102	0.0402
metab_6662	neg	489.3075	7.9908	0.8991	-0.8310	0.0159	0.0542
metab_12540	neg	489.3222	6.1644	0.2515	1.0300	0.6436	0.7403
metab_10379	neg	489.3225	9.0067	0.7423	1.0895	0.0488	0.1157
metab_12178	neg	489.3232	7.4927	0.4353	-0.5972	0.3467	0.4797
metab_7811	neg	489.9159	0.5126	0.7658	-1.0037	0.0644	0.1415
metab_9622	neg	490.0754	5.7420	1.0815	1.6000	0.0030	0.0171
metab_12806	neg	490.0756	4.9349	0.3920	-0.6592	0.4209	0.5469
metab_6241	pos	490.1749	0.7941	0.2727	-0.5640	0.7467	0.8269
metab_13493	neg	490.1872	2.8269	1.0411	-0.6872	0.3427	0.4759
metab_6011	pos	490.1907	1.2306	0.5991	-1.1228	0.4625	0.5905
metab_12288	neg	490.2577	7.0796	2.3244	2.5587	0.0231	0.0694
metab_9660	neg	490.2581	5.9866	0.4245	0.2017	0.3717	0.5032
metab_12709	neg	490.2583	5.4188	1.1961	2.0978	0.0165	0.0554
metab_9086	neg	490.2682	3.3304	3.2532	14.5306	0.0000	0.0011
metab_1753	pos	490.2849	1.9758	1.8955	7.8136	0.0007	0.0060
metab_7057	neg	490.2944	7.6793	0.2047	0.3294	0.5875	0.6950

metab_12710	neg	490.3390	5.4034	0.4390	-0.4636	0.4234	0.5492
metab_10367	neg	490.3757	8.9574	0.0882	0.2084	0.9592	0.9727
metab_4136	pos	490.3882	8.0700	0.1562	-0.9108	0.6601	0.7586
metab_6855	neg	491.1161	1.2663	0.3365	0.9398	0.4963	0.6172
metab_9292	neg	491.1204	4.0685	0.4878	-0.1645	0.0277	0.0787
metab_13861	neg	491.1415	2.0987	0.9461	3.5399	0.1937	0.3138
metab_8923	neg	491.1679	2.8269	0.1079	1.1229	0.7963	0.8539
metab_5210	pos	491.1950	2.9652	1.7127	-2.6658	0.0012	0.0083
metab_14629	neg	491.2001	0.9649	3.0362	8.3300	0.0001	0.0017
metab_9915	neg	491.2421	7.2069	0.7220	1.3236	0.1363	0.2435
metab_123	pos	491.2755	7.2164	1.0284	-1.4586	0.0102	0.0364
metab_12197	neg	491.2790	7.4300	1.1676	-1.3515	0.1983	0.3189
metab_12942	neg	491.2876	4.4055	0.4326	0.3607	0.4569	0.5803
metab_531	pos	491.2996	3.3490	1.8972	4.6212	0.0012	0.0083
metab_7339	neg	491.3018	8.3209	0.3512	0.6618	0.2115	0.3341
metab_9760	neg	491.3019	6.5190	0.9501	1.3722	0.0254	0.0742
metab_7037	neg	491.3020	7.1595	0.6902	0.7991	0.1486	0.2597
metab_12873	neg	491.3024	4.6722	1.2098	2.0776	0.0002	0.0028
metab_9900	neg	491.3347	7.1437	0.9423	2.1536	0.0990	0.1925
metab_11951	neg	491.3382	8.1307	0.2527	0.1001	0.5582	0.6695
metab_10327	neg	491.3383	8.7488	0.0269	0.3066	0.9261	0.9520
metab_963	pos	491.3719	8.4214	0.1730	-0.5288	0.7966	0.8643
metab_2979	pos	491.3719	8.9507	0.0570	0.1321	0.9057	0.9398
metab_3821	pos	491.3720	9.3891	0.4269	-0.7456	0.2710	0.3994
metab_2794	pos	491.3723	7.8389	0.5384	1.5042	0.4185	0.5503
metab_7301	neg	491.3862	8.7640	1.7426	-3.1589	0.0001	0.0020
metab_6975	neg	492.0724	4.9349	0.4654	-0.7555	0.3437	0.4768
metab_9621	neg	492.0725	5.7420	1.1333	1.6657	0.0002	0.0027
metab_11513	neg	492.1096	9.2507	0.0445	1.3304	0.7891	0.8492
metab_13762	neg	492.2027	2.2754	1.2991	-1.9747	0.0097	0.0390
metab_8464	neg	492.2109	1.7419	0.3286	0.8681	0.6818	0.7713
metab_8901	neg	492.2319	2.7624	1.7334	12.0180	0.0029	0.0167
metab_9788	neg	492.2373	6.6962	0.9087	-1.0773	0.0564	0.1283
metab_11774	neg	492.2433	8.6728	0.9808	-1.2534	0.0220	0.0673
metab_4537	pos	492.2709	5.9800	0.5421	0.0735	0.2434	0.3680
metab_9649	neg	492.2738	5.9375	0.0992	-0.4725	0.7666	0.8329
metab_2124	pos	492.2811	3.3183	2.7993	14.0210	0.0000	0.0004
metab_2773	pos	492.3071	7.6776	0.4230	0.9688	0.4361	0.5672
metab_4398	pos	492.3074	6.8433	0.1633	0.0366	0.7642	0.8397
metab_3725	pos	492.3825	9.8211	0.7578	0.6047	0.1462	0.2513
metab_13903	neg	493.1941	2.0202	0.6373	-0.4809	0.2410	0.3672
metab_161	pos	493.2129	0.9620	3.5787	14.5090	0.0000	0.0000
metab_13325	neg	493.2452	3.2301	1.1945	5.2869	0.0084	0.0351
metab_12124	neg	493.2583	7.6632	0.8089	1.6842	0.0639	0.1408
metab_12629	neg	493.2586	5.7585	0.9969	-3.3856	0.1437	0.2535
metab_10073	neg	493.2945	7.8822	0.2994	-0.2118	0.6170	0.7196
metab_10159	neg	493.3138	8.1936	0.3030	0.4878	0.5450	0.6580
metab_460	pos	493.3151	2.5003	1.9675	7.5186	0.0022	0.0127

metab_5071	pos	493.3156	3.4549	1.6895	4.0573	0.0010	0.0073
metab_11830	neg	493.3162	8.5135	0.6198	-0.6215	0.0479	0.1141
metab_9366	neg	493.3178	4.3882	1.1008	2.6205	0.0161	0.0546
metab_12711	neg	493.3179	5.4034	1.7905	4.2195	0.0002	0.0027
metab_12828	neg	493.3181	4.8541	1.6341	3.8490	0.0002	0.0031
metab_9225	neg	493.3187	3.8164	1.3061	3.0383	0.0046	0.0230
metab_6656	neg	493.3527	8.6874	1.1299	-1.9826	0.0114	0.0430
metab_10229	neg	493.3659	8.4821	2.4011	-4.9504	0.0010	0.0083
metab_10451	neg	494.1067	9.2507	0.1233	1.2902	0.7037	0.7878
metab_7398	neg	494.1300	6.5989	1.8625	-4.0447	0.0029	0.0168
metab_10187	neg	494.1984	8.3055	1.6429	-2.4343	0.0002	0.0031
metab_12007	neg	494.1986	7.9908	1.6362	-2.8185	0.0005	0.0056
metab_1855	pos	494.2153	2.2665	1.5180	-2.7481	0.0008	0.0067
metab_13055	neg	494.2167	4.0347	0.7079	2.0183	0.1062	0.2030
metab_12327	neg	494.2224	6.9525	0.5223	-0.2813	0.3093	0.4404
metab_7034	neg	494.2536	7.1595	0.5193	-0.0086	0.1697	0.2861
metab_10396	neg	494.2588	9.0557	0.3461	-0.4245	0.4479	0.5713
metab_4505	pos	494.2867	6.1614	0.8490	1.1431	0.0586	0.1278
metab_4303	pos	494.2868	7.1701	1.1436	3.8354	0.0045	0.0204
metab_13129	neg	494.2888	3.7830	0.4544	-0.3546	0.4929	0.6141
metab_9284	neg	494.2896	4.0516	0.5224	-0.2424	0.4188	0.5447
metab_11796	neg	494.2897	8.6093	1.2502	-1.5092	0.0000	0.0006
metab_4722	pos	494.3098	4.9554	1.4162	2.9170	0.0377	0.0923
metab_4273	pos	494.3231	7.3521	0.5469	1.7418	0.1670	0.2776
metab_4110	pos	494.3234	8.1857	1.7624	3.5197	0.0000	0.0011
metab_11732	neg	494.3280	8.7031	0.8139	-0.8167	0.0704	0.1504
metab_952	pos	494.3619	9.4207	0.2532	0.2909	0.5131	0.6346
metab_2811	pos	494.3981	7.9835	0.3316	-0.2271	0.5966	0.7069
metab_15098	neg	494.7901	0.0320	0.3967	-0.1803	0.0634	0.1400
metab_14687	neg	495.1113	0.8522	0.5247	-0.3283	0.0472	0.1132
metab_7937	neg	495.1121	0.6271	0.4291	-0.1973	0.2835	0.4143
metab_6368	pos	495.1232	0.5983	0.8665	-0.7895	0.0103	0.0366
metab_8027	neg	495.1361	0.9227	0.8396	1.8614	0.0764	0.1593
metab_8193	neg	495.1361	1.3089	0.4263	2.2664	0.1831	0.3014
metab_11909	neg	495.1698	8.2409	1.1706	-3.3122	0.0139	0.0494
metab_11798	neg	495.2062	8.6093	0.7792	-0.2738	0.0556	0.1269
metab_1024	pos	495.2073	2.0211	0.2380	-0.0414	0.5712	0.6865
metab_12882	neg	495.2183	4.6380	1.0594	2.6313	0.0567	0.1290
metab_9741	neg	495.2736	6.4073	0.8120	-1.0996	0.0873	0.1760
metab_9703	neg	495.2737	6.2456	1.0546	-1.8148	0.0341	0.0902
metab_12399	neg	495.2740	6.7603	1.4146	-1.7823	0.0039	0.0207
metab_9689	neg	495.2920	6.1320	0.3519	0.4595	0.5749	0.6843
metab_5148	pos	495.3311	3.1799	1.6827	5.5339	0.0067	0.0272
metab_9488	neg	495.3335	4.9510	1.7537	6.7953	0.0164	0.0552
metab_4423	pos	495.3933	6.6454	0.1612	0.2159	0.7150	0.8020
metab_14945	neg	495.4744	0.5286	1.4234	4.3696	0.0095	0.0384
metab_14516	neg	495.6035	1.2236	1.5258	-1.9421	0.0053	0.0257
metab_8234	neg	496.1682	1.3817	2.2526	-5.6944	0.0004	0.0049

metab_14895	neg	496.1789	0.5571	0.3220	0.7172	0.4125	0.5389
metab_4923	pos	496.2296	4.0479	0.7577	3.6072	0.0994	0.1884
metab_10005	neg	496.2326	7.6323	0.7626	1.1846	0.0226	0.0685
metab_13084	neg	496.2327	3.9177	0.8984	-0.9472	0.0336	0.0895
metab_11689	neg	496.2330	8.7488	1.2156	4.2088	0.0116	0.0436
metab_4872	pos	496.2658	4.3216	1.2897	-5.5947	0.0924	0.1791
metab_4308	pos	496.2659	7.1701	0.7352	-0.3917	0.1453	0.2503
metab_6664	neg	496.2687	7.8026	0.6431	-0.1592	0.0274	0.0783
metab_11080	neg	496.2689	14.0282	0.7344	-0.2922	0.0073	0.0319
metab_560	pos	496.3028	3.7908	0.8317	0.7518	0.1152	0.2103
metab_4925	pos	496.3029	4.0479	0.1006	-0.8028	0.8941	0.9319
metab_752	pos	496.3387	7.9980	0.1415	0.1004	0.4820	0.6075
metab_11770	neg	496.3759	8.6728	1.5259	-2.9976	0.0000	0.0013
metab_3787	pos	496.3777	9.5594	0.5023	-1.1878	0.2426	0.3675
metab_14559	neg	497.1157	1.1212	1.5543	3.6969	0.0091	0.0375
metab_255	pos	497.1236	0.9200	0.5911	-0.5453	0.0091	0.0337
metab_13953	neg	497.1886	1.9439	1.3371	2.1544	0.0098	0.0393
metab_13002	neg	497.2338	4.2040	1.9572	-2.2721	0.0039	0.0207
metab_11443	neg	497.2583	9.5314	2.1119	15.4687	0.0003	0.0036
metab_12367	neg	497.3045	6.8730	0.2988	0.2913	0.4836	0.6056
metab_2380	pos	497.3362	4.7608	2.0603	8.0134	0.0001	0.0016
metab_11843	neg	497.3394	8.4821	2.6299	-3.8480	0.0006	0.0061
metab_9639	neg	498.2172	5.8409	1.2424	5.9617	0.0099	0.0394
metab_11970	neg	498.2482	8.0843	1.6320	3.5566	0.0011	0.0091
metab_743	pos	498.2815	7.8097	0.7154	-0.5752	0.0226	0.0636
metab_2392	pos	498.2816	4.8809	0.7267	-2.9814	0.2429	0.3676
metab_12440	neg	498.2845	6.5829	0.9793	1.3208	0.1349	0.2418
metab_10055	neg	498.3192	7.8026	1.8098	-2.1906	0.0013	0.0103
metab_12714	neg	498.3193	5.3877	1.0344	2.6954	0.2606	0.3902
metab_10169	neg	498.3205	8.2253	1.1975	-1.8045	0.0025	0.0153
metab_2273	pos	498.3349	4.0634	1.2228	-1.8165	0.0017	0.0108
metab_3587	pos	498.3988	10.8701	0.2570	-0.1951	0.1036	0.1945
metab_15100	neg	498.7844	0.0259	0.1947	-0.0310	0.5810	0.6895
metab_10727	neg	498.8866	13.9143	0.3386	0.0778	0.1645	0.2798
metab_14992	neg	498.9047	0.5126	0.9312	-1.0457	0.0166	0.0556
metab_6419	pos	499.0003	0.5420	0.4338	0.5575	0.2078	0.3276
metab_6465	pos	499.1570	0.5140	1.1280	-4.8030	0.0160	0.0495
metab_6911	neg	499.1681	1.4710	1.0962	2.0389	0.0329	0.0883
metab_13491	neg	499.1729	2.8269	1.1732	-0.8820	0.0568	0.1291
metab_10240	neg	499.2738	8.5135	0.5111	0.1064	0.4329	0.5572
metab_10576	neg	499.2742	9.8947	1.2170	14.8824	0.0171	0.0566
metab_10257	neg	499.3315	8.5604	1.6984	-3.1510	0.0005	0.0054
metab_12071	neg	499.3393	7.7875	0.1043	1.2847	0.7943	0.8525
metab_11630	neg	499.3649	8.9574	0.9678	1.3327	0.0431	0.1063
metab_15119	neg	499.7802	0.0197	0.2950	-0.0575	0.2409	0.3671
metab_14758	neg	500.1626	0.6551	0.7612	1.5024	0.0525	0.1217
metab_7442	neg	500.1727	1.5297	0.1208	1.0126	0.9353	0.9582
metab_4219	pos	500.2737	7.6776	0.1415	-0.0477	0.3909	0.5229

metab_2608	pos	500.2949	6.6001	0.8707	-2.4507	0.1772	0.2905
metab_2488	pos	500.2974	5.5715	0.5931	0.8981	0.1668	0.2774
metab_5639	pos	500.3068	1.9609	1.5042	13.6393	0.0040	0.0187
metab_3732	pos	500.4662	9.7899	1.0214	-1.8639	0.0244	0.0677
metab_15017	neg	500.9206	0.5126	0.9319	-1.4506	0.0478	0.1140
metab_1342	pos	501.0367	0.6962	1.6879	15.8301	0.0021	0.0125
metab_10572	neg	501.0924	9.8782	0.7558	1.4265	0.0130	0.0472
metab_11169	neg	501.0924	11.3419	0.5154	0.6158	0.0133	0.0480
metab_13969	neg	501.1836	1.9125	1.4477	3.4051	0.0010	0.0086
metab_13285	neg	501.2173	3.3304	1.6551	14.1893	0.0063	0.0288
metab_13422	neg	501.2173	2.9931	2.0003	14.9534	0.0002	0.0034
metab_12132	neg	501.2234	7.6323	0.5214	-0.3624	0.0974	0.1906
metab_6940	neg	501.2504	4.0685	1.8195	4.4413	0.0013	0.0103
metab_12864	neg	501.2868	4.7046	0.1164	1.0637	0.7114	0.7934
metab_4049	pos	501.3028	8.4074	0.0139	-0.8437	0.8678	0.9153
metab_11551	neg	501.3592	9.1364	0.8229	1.6601	0.0260	0.0756
metab_12047	neg	501.3917	7.8822	0.5213	-1.2138	0.3971	0.5250
metab_11880	neg	501.3919	8.3373	0.8136	-1.6129	0.2723	0.4028
metab_7308	neg	501.3919	8.7031	0.9476	-2.5720	0.1442	0.2540
metab_10970	neg	501.9128	14.1581	0.4923	0.5108	0.0039	0.0207
metab_9462	neg	502.0571	4.8210	1.3227	12.1744	0.0183	0.0593
metab_6847	neg	502.1784	0.5991	0.3335	0.7101	0.2680	0.3982
metab_14591	neg	502.1787	1.0357	0.5208	1.6137	0.1728	0.2894
metab_5815	pos	502.2117	1.5847	1.2150	14.3662	0.0267	0.0722
metab_6741	neg	502.2147	0.5991	0.3324	-0.2756	0.5103	0.6294
metab_10361	neg	502.2276	8.9417	1.4506	-2.1180	0.0003	0.0039
metab_13577	neg	502.2350	2.6524	1.0251	4.6433	0.0785	0.1629
metab_4577	pos	502.2795	5.7846	0.0174	-0.0677	0.9518	0.9689
metab_4309	pos	502.2918	7.1560	1.0267	3.4536	0.0230	0.0643
metab_12246	neg	502.3115	7.2069	0.1025	0.0594	0.8757	0.9149
metab_9793	neg	502.3116	6.7439	0.1356	-0.1457	0.6657	0.7589
metab_4582	pos	502.3152	5.7846	0.2539	-0.2433	0.4693	0.5962
metab_6631	neg	502.3315	8.5937	0.9226	6.6903	0.1177	0.2181
metab_4832	pos	502.3517	4.4730	1.5063	5.6260	0.0369	0.0908
metab_10356	neg	502.3521	8.9086	1.1823	-2.0426	0.0283	0.0798
metab_3565	pos	502.3725	11.3742	0.3590	-0.2696	0.0132	0.0434
metab_3137	pos	502.3728	9.9470	0.3498	-0.2766	0.1021	0.1925
metab_4246	pos	502.3730	7.5151	0.3839	-0.3372	0.1170	0.2127
metab_14646	neg	503.1626	0.9227	0.9136	-0.6778	0.0045	0.0226
metab_8170	neg	503.1627	1.2805	0.3487	0.1386	0.4099	0.5370
metab_11937	neg	503.2403	8.1623	0.6964	1.1127	0.0762	0.1590
metab_5002	pos	503.2630	3.7148	1.8689	16.0675	0.0015	0.0099
metab_5124	pos	503.2632	3.2420	2.4183	10.3430	0.0001	0.0019
metab_13200	neg	503.2660	3.5978	1.7754	4.2806	0.0007	0.0066
metab_12446	neg	503.2864	6.5346	0.0599	0.4282	0.8577	0.9017
metab_7005	neg	503.2866	6.2943	0.1795	0.1474	0.6711	0.7625
metab_10279	neg	503.2987	8.6239	1.9167	5.9011	0.0041	0.0210
metab_12656	neg	503.3023	5.6615	0.6199	1.3444	0.1285	0.2330

metab_9496	neg	503.3023	5.0159	0.9596	2.4386	0.1152	0.2146
metab_4067	pos	503.4041	8.3322	0.2886	-1.5607	0.4992	0.6220
metab_15030	neg	503.8677	0.4975	0.6214	-0.5866	0.0897	0.1795
metab_7900	neg	504.1944	0.5851	0.0964	0.3768	0.7252	0.8024
metab_9514	neg	504.2378	5.1456	1.2395	2.7565	0.0269	0.0772
metab_403	pos	504.2640	1.9918	1.8548	4.3520	0.0002	0.0027
metab_2739	pos	504.2709	7.3824	0.2041	0.2857	0.5439	0.6635
metab_9895	neg	504.2740	7.1266	0.5369	0.5936	0.0751	0.1574
metab_12150	neg	504.3104	7.5855	0.6198	0.6977	0.0351	0.0919
metab_686	pos	504.3243	6.7366	0.0850	-0.1731	0.7243	0.8101
metab_9848	neg	504.3276	6.9525	2.1479	-4.5271	0.0084	0.0351
metab_10380	neg	504.3467	9.0067	0.9646	8.0914	0.1934	0.3134
metab_4536	pos	504.3673	5.9800	0.6557	0.5871	0.1848	0.2996
metab_4804	pos	504.3675	4.5944	1.4644	3.0737	0.1028	0.1934
metab_7824	neg	505.0373	0.5286	0.1735	0.2188	0.7252	0.8024
metab_13842	neg	505.1574	2.1311	0.4087	0.0011	0.5146	0.6331
metab_2167	pos	505.2104	3.5311	1.4420	-2.7639	0.0044	0.0201
metab_6613	neg	505.2818	3.6812	1.6535	3.7733	0.0003	0.0039
metab_7408	neg	505.3024	6.2294	0.8971	-1.5478	0.0292	0.0814
metab_7396	neg	505.3025	6.7122	0.6563	-0.4319	0.0713	0.1517
metab_12561	neg	505.3028	6.0670	1.4110	-2.3244	0.0002	0.0035
metab_7381	neg	505.3179	7.1749	0.9210	1.1780	0.0024	0.0148
metab_11646	neg	505.4020	8.9252	1.8130	-3.4453	0.0006	0.0059
metab_14848	neg	506.1552	0.5851	0.8072	-1.8753	0.1458	0.2562
metab_14467	neg	506.1882	1.2805	0.2915	-0.0200	0.6076	0.7113
metab_13537	neg	506.2182	2.7318	0.5358	1.2068	0.2776	0.4082
metab_13276	neg	506.2477	3.3465	1.7866	13.1922	0.0004	0.0048
metab_12341	neg	506.2524	6.9191	1.0240	2.4418	0.0824	0.1686
metab_9882	neg	506.2529	7.0469	0.7771	1.3582	0.1230	0.2253
metab_9573	neg	506.2530	5.5002	0.6599	-0.4884	0.4161	0.5423
metab_748	pos	506.2868	7.9118	1.6820	2.8706	0.0107	0.0375
metab_712	pos	506.2871	7.1267	0.0879	-0.1216	0.8963	0.9334
metab_10094	neg	506.2976	7.9757	0.7395	-0.6963	0.0781	0.1625
metab_976	pos	506.3232	7.6481	0.5219	0.7832	0.2575	0.3845
metab_716	pos	506.3236	7.2164	0.9252	1.3735	0.0052	0.0225
metab_11964	neg	506.3252	8.0998	1.1998	2.0004	0.0000	0.0008
metab_702	pos	506.3397	7.0082	0.9436	-1.1982	0.0002	0.0028
metab_11968	neg	506.3594	8.0843	1.3851	-3.4962	0.0005	0.0055
metab_4422	pos	506.3831	6.6454	0.8182	0.7804	0.1700	0.2813
metab_4514	pos	506.3832	6.1167	1.0290	1.0169	0.0172	0.0523
metab_4564	pos	506.3832	5.8753	0.9317	1.0017	0.0426	0.1012
metab_3704	pos	506.4766	9.8836	0.1910	-0.2142	0.3036	0.4354
metab_14832	neg	507.1571	0.5991	1.0269	-0.9713	0.0086	0.0358
metab_8465	neg	507.1624	1.7419	0.4326	0.6928	0.4854	0.6073
metab_13637	neg	507.1724	2.5264	1.5860	-2.2139	0.0130	0.0471
metab_6803	neg	507.1947	0.9086	2.9400	10.4456	0.0000	0.0001
metab_12097	neg	507.2171	7.7251	0.9438	0.0057	0.0900	0.1799
metab_4296	pos	507.2709	7.2314	1.2288	-0.0511	0.0745	0.1524

metab_7039	neg	507.2734	7.2069	0.6969	-0.3942	0.0323	0.0870
metab_2365	pos	507.2944	4.6544	1.8723	3.8015	0.0000	0.0003
metab_4918	pos	507.2946	4.0634	2.2167	6.1044	0.0001	0.0021
metab_68	pos	507.2947	3.1191	1.6066	4.0065	0.0015	0.0096
metab_12374	neg	507.3332	6.8565	0.0927	0.0696	0.8738	0.9136
metab_11678	neg	507.3652	8.7807	0.1896	-0.7753	0.7024	0.7866
metab_11781	neg	507.3654	8.6406	0.0906	-0.1351	0.8357	0.8846
metab_3943	pos	507.3927	8.8318	1.1143	-0.4449	0.0723	0.1496
metab_3070	pos	507.3934	9.5594	0.2564	-0.1733	0.4134	0.5456
metab_11895	neg	508.1778	8.3055	0.9605	-1.0129	0.0099	0.0394
metab_5986	pos	508.2014	1.2725	0.0138	-0.0381	0.8666	0.9147
metab_10072	neg	508.2016	7.8822	1.6332	-1.9777	0.0009	0.0078
metab_13383	neg	508.2089	3.0774	1.1707	12.0060	0.0455	0.1103
metab_12243	neg	508.2385	7.2069	1.0242	-1.6631	0.1047	0.2007
metab_9685	neg	508.2688	6.1157	1.2634	2.4703	0.0004	0.0050
metab_6981	neg	508.2689	5.2100	0.3058	-0.4764	0.3200	0.4522
metab_11848	neg	508.3137	8.4667	0.3094	0.4415	0.5133	0.6323
metab_2247	pos	508.3257	3.9270	2.2950	14.1812	0.0100	0.0359
metab_2354	pos	508.3260	4.6096	2.4170	9.7522	0.0000	0.0005
metab_2950	pos	508.3391	8.7731	1.0781	1.4644	0.0178	0.0535
metab_2839	pos	508.3391	8.1707	1.9404	3.9382	0.0001	0.0017
metab_4203	pos	508.3391	7.7514	1.9765	4.5647	0.0000	0.0001
metab_11773	neg	508.3413	8.6728	0.4040	-0.8193	0.3674	0.4988
metab_11789	neg	508.4011	8.6239	0.5668	1.3236	0.3918	0.5202
metab_4161	pos	508.4139	7.9835	0.2651	0.0116	0.5314	0.6520
metab_14599	neg	509.1266	1.0215	0.0086	0.2347	0.8686	0.9097
metab_11977	neg	509.1617	8.0688	1.3935	-2.1963	0.0365	0.0945
metab_14090	neg	509.1994	1.7264	1.2504	-0.8098	0.0198	0.0625
metab_11806	neg	509.2215	8.5937	2.2374	-3.8607	0.0001	0.0023
metab_10338	neg	509.2216	8.7807	1.8724	-3.7205	0.0001	0.0021
metab_4297	pos	509.2864	7.2164	0.8624	-1.0885	0.0251	0.0691
metab_9994	neg	509.2891	7.5855	0.3792	0.0755	0.2176	0.3410
metab_10620	neg	509.2943	10.1401	0.0083	0.7572	0.9368	0.9586
metab_5086	pos	509.3103	3.3793	2.2167	8.5699	0.0000	0.0005
metab_7431	neg	509.3137	3.9508	1.7450	4.3234	0.0005	0.0057
metab_7368	neg	509.3485	7.7404	1.2545	-1.9561	0.0249	0.0733
metab_7383	neg	509.3494	7.0959	1.2737	-2.2437	0.0028	0.0163
metab_2897	pos	509.3943	8.4809	0.7044	0.8394	0.2411	0.3659
metab_10206	neg	510.1994	8.3687	1.5526	-5.8271	0.0016	0.0113
metab_7018	neg	510.2631	6.7764	0.0531	-0.2498	0.8799	0.9180
metab_630	pos	510.2815	5.1830	0.0195	-0.6270	0.9487	0.9671
metab_12816	neg	510.2848	4.9021	1.2859	2.2140	0.0012	0.0098
metab_3931	pos	510.3265	8.9060	0.4514	-0.5931	0.6332	0.7381
metab_10371	neg	510.3292	8.9903	0.8522	-0.5219	0.0846	0.1717
metab_4065	pos	510.3546	8.3463	1.4101	2.3550	0.0001	0.0020
metab_2205	pos	510.3777	3.6987	1.7841	14.2447	0.0010	0.0074
metab_3784	pos	510.3935	9.5749	0.1557	-0.1344	0.8307	0.8887
metab_787	pos	510.3936	8.5554	0.3457	-0.3062	0.4603	0.5885

metab_263	pos	511.1394	1.0180	0.0768	-0.2607	0.8063	0.8717
metab_13692	neg	511.2040	2.4175	1.4476	2.4804	0.0000	0.0010
metab_7364	neg	511.2859	7.9137	1.3770	-1.8731	0.0059	0.0273
metab_3917	pos	511.2981	8.9656	0.2022	-0.0136	0.5227	0.6442
metab_10090	neg	511.3393	7.9454	0.1193	1.4867	0.9912	0.9933
metab_4522	pos	511.3519	6.0410	0.5668	1.5594	0.3586	0.4929
metab_13614	neg	512.1017	2.5748	1.3017	4.0452	0.0051	0.0250
metab_14863	neg	512.1026	0.5711	0.1163	0.1545	0.7165	0.7966
metab_12431	neg	512.1721	6.5989	1.9079	-7.2448	0.0002	0.0028
metab_12948	neg	512.2146	4.3716	1.6024	13.4726	0.0004	0.0050
metab_9740	neg	512.2319	6.4073	0.9715	2.0929	0.2198	0.3437
metab_2627	pos	512.2761	6.7825	0.0580	-0.2762	0.9893	0.9930
metab_12418	neg	512.2786	6.6632	0.7137	1.0154	0.0813	0.1672
metab_9850	neg	512.2787	6.9688	0.7076	-1.3047	0.2031	0.3246
metab_7042	neg	512.2998	7.2069	0.4601	0.5364	0.1685	0.2849
metab_12717	neg	512.3002	5.3713	1.9127	4.1307	0.0010	0.0082
metab_11870	neg	512.3354	8.3858	1.5124	-2.1106	0.0095	0.0384
metab_3014	pos	512.3725	9.1605	0.2624	-0.0720	0.6378	0.7417
metab_4285	pos	512.4086	7.2763	0.4862	0.4276	0.3613	0.4953
metab_14744	neg	513.0869	0.7114	0.5406	-0.3970	0.2881	0.4195
metab_6747	neg	513.1230	0.6131	0.2228	0.1982	0.5578	0.6692
metab_4851	pos	513.2812	4.3818	2.5190	10.6602	0.0000	0.0008
metab_9988	neg	513.2830	7.5541	1.8807	-6.4745	0.0000	0.0006
metab_12730	neg	513.2844	5.2741	1.2365	3.8926	0.0743	0.1562
metab_9096	neg	513.3078	3.3465	1.8645	4.9295	0.0001	0.0018
metab_10162	neg	513.3189	8.1936	0.4213	-1.1694	0.4336	0.5578
metab_4037	pos	513.3210	8.4504	1.5419	2.3904	0.0250	0.0689
metab_2500	pos	513.3674	5.6623	1.5582	-2.1211	0.0013	0.0089
metab_9092	neg	514.1872	3.3304	0.7884	1.9003	0.2732	0.4038
metab_1942	pos	514.2166	2.5938	1.3450	-1.8769	0.0183	0.0545
metab_12364	neg	514.2484	6.8730	1.0070	2.7300	0.0979	0.1912
metab_4359	pos	514.2917	6.9638	0.3637	-0.9070	0.4523	0.5818
metab_2614	pos	514.2919	6.6454	1.3065	1.9472	0.0048	0.0214
metab_4645	pos	514.3135	5.3757	2.4923	13.3769	0.0000	0.0004
metab_11855	neg	514.3143	8.4336	1.9131	-13.8515	0.0013	0.0103
metab_3948	pos	514.3289	8.8169	0.3911	0.5661	0.2961	0.4268
metab_9713	neg	514.3386	6.2943	0.7147	2.9872	0.3181	0.4504
metab_11666	neg	514.3510	8.8286	0.3511	1.5467	0.5428	0.6564
metab_4434	pos	514.3930	6.5704	0.5633	0.3479	0.0361	0.0895
metab_10758	neg	514.8938	14.0282	0.1036	0.0142	0.6724	0.7635
metab_13915	neg	515.1624	2.0055	1.0446	2.9104	0.0279	0.0791
metab_8081	neg	515.1986	1.0785	1.0702	4.5669	0.0168	0.0560
metab_14824	neg	515.2103	0.5991	0.3000	-0.5088	0.5921	0.6991
metab_7889	neg	515.2210	0.5711	0.8331	1.5442	0.0761	0.1589
metab_12492	neg	515.2998	6.3917	1.0808	4.7814	0.1298	0.2346
metab_3891	pos	515.3005	9.0857	1.9345	-7.5850	0.0001	0.0012
metab_9523	neg	515.3133	5.1772	0.2985	1.6988	0.6551	0.7498
metab_8572	neg	515.3205	1.9745	1.8339	14.0798	0.0000	0.0002

metab_3822	pos	515.3363	9.3891	0.3290	2.1028	0.5935	0.7045
metab_7285	neg	515.3862	9.1693	2.1714	-4.0871	0.0000	0.0013
metab_2617	pos	515.4409	6.6909	0.8033	1.5928	0.1923	0.3082
metab_10194	neg	516.1088	8.3209	2.0605	-6.6484	0.0000	0.0001
metab_4557	pos	516.2691	5.9208	0.1238	-0.7370	0.9245	0.9512
metab_11813	neg	516.3105	8.5604	1.3200	-1.5015	0.0003	0.0041
metab_12686	neg	516.3180	5.5484	1.2551	3.8666	0.0640	0.1410
metab_2520	pos	516.3309	5.7846	0.1595	-0.1423	0.7378	0.8200
metab_4091	pos	516.3673	8.2435	1.6045	4.9194	0.0043	0.0196
metab_4077	pos	516.4402	8.3018	1.0871	-1.7138	0.0055	0.0235
metab_6706	neg	516.8982	0.5126	1.0349	-1.3065	0.0707	0.1509
metab_1338	pos	517.0111	0.6822	0.3773	-0.3235	0.0999	0.1890
metab_13193	neg	517.0995	3.6142	0.5254	-0.1973	0.0137	0.0489
metab_9650	neg	517.2188	5.9375	0.4356	-1.3779	0.3833	0.5129
metab_2580	pos	517.2412	6.2978	1.1624	12.9818	0.0227	0.0639
metab_9189	neg	517.2456	3.6980	2.0513	8.4221	0.0002	0.0030
metab_9524	neg	517.2812	5.1939	2.1088	4.1096	0.0051	0.0246
metab_4811	pos	517.2964	4.5642	1.1236	1.1329	0.1461	0.2512
metab_14782	neg	518.1727	0.6131	0.1081	0.3888	0.6256	0.7257
metab_12338	neg	518.2531	6.9354	0.5148	1.0790	0.2227	0.3470
metab_2754	pos	518.2868	7.4855	0.3464	1.0025	0.3789	0.5115
metab_2555	pos	518.2872	6.0410	0.7180	1.7809	0.1599	0.2685
metab_12596	neg	518.2893	5.9217	0.1524	-2.0935	0.7721	0.8373
metab_10013	neg	518.2895	7.6632	0.3257	0.4176	0.2102	0.3328
metab_12265	neg	518.2897	7.1595	0.8006	1.0243	0.0356	0.0927
metab_13458	neg	518.2992	2.9092	2.4557	7.2603	0.0000	0.0002
metab_2707	pos	518.3230	7.1701	1.3304	2.1575	0.0000	0.0006
metab_4817	pos	518.3467	4.5485	1.7816	4.3354	0.0058	0.0245
metab_566	pos	518.3468	3.8511	1.8930	4.5040	0.0001	0.0018
metab_884	pos	518.4920	10.3632	1.8528	-3.5963	0.0000	0.0003
metab_14490	neg	519.1111	1.2663	0.2411	0.0210	0.4638	0.5861
metab_7926	neg	519.1571	0.6131	0.8597	-1.1016	0.0651	0.1427
metab_11868	neg	519.2273	8.3858	0.9738	-0.6494	0.0340	0.0900
metab_13127	neg	519.2612	3.7830	2.2363	6.1985	0.0003	0.0037
metab_9347	neg	519.2972	4.3037	1.0535	2.3724	0.0202	0.0634
metab_12014	neg	519.3178	7.9757	2.7376	-5.1232	0.0006	0.0062
metab_11637	neg	519.3286	8.9417	1.3259	-1.2416	0.0010	0.0083
metab_10346	neg	519.3425	8.8286	0.6427	-0.7018	0.0472	0.1132
metab_12204	neg	519.9506	7.3824	0.6872	1.1271	0.1217	0.2236
metab_4256	pos	520.0225	7.4561	1.0756	1.4454	0.0002	0.0027
metab_2764	pos	520.0225	7.5892	0.5370	0.4291	0.0676	0.1425
metab_8033	neg	520.1676	0.9508	0.5192	-1.2196	0.4629	0.5853
metab_4368	pos	520.2658	6.9336	0.6443	0.9000	0.1848	0.2996
metab_4592	pos	520.2659	5.7086	0.2477	-0.4824	0.4692	0.5962
metab_6626	neg	520.2687	7.3824	0.5717	1.0259	0.1428	0.2524
metab_11066	neg	520.2688	14.0282	0.4953	0.9173	0.1846	0.3031
metab_9710	neg	520.2772	6.2775	0.2905	-0.4299	0.4613	0.5838
metab_12126	neg	520.3045	7.6479	1.3155	3.6134	0.0774	0.1614

metab_12609	neg	520.3049	5.8731	0.1129	-1.1489	0.7934	0.8519
metab_731	pos	520.3063	7.5892	0.6891	0.4691	0.0104	0.0369
metab_4106	pos	520.3063	8.1857	0.8250	1.1086	0.1255	0.2241
metab_5235	pos	520.3117	2.9028	2.1580	7.9136	0.0000	0.0002
metab_980	pos	520.3387	7.5892	0.7570	0.6984	0.0020	0.0121
metab_2332	pos	520.3624	4.4730	1.4922	3.6333	0.0122	0.0412
metab_10180	neg	520.3762	8.2897	2.0195	-2.8335	0.0167	0.0558
metab_3163	pos	520.4349	10.1941	0.6222	-0.5431	0.0011	0.0078
metab_108	pos	520.4715	9.9154	0.6005	0.7052	0.0614	0.1323
metab_9929	neg	520.5855	7.2701	0.7076	1.2492	0.1078	0.2053
metab_4239	pos	520.6553	7.5743	0.7159	0.6096	0.0210	0.0603
metab_11093	neg	520.9110	14.0282	0.2231	-0.0662	0.4792	0.6015
metab_8248	neg	521.1565	1.4115	0.0210	0.4617	0.9887	0.9917
metab_14837	neg	521.1729	0.5991	0.4700	-0.2149	0.2261	0.3506
metab_9616	neg	521.2534	5.7259	1.7149	-3.4410	0.0034	0.0185
metab_13208	neg	521.2767	3.5813	1.7662	3.9537	0.0034	0.0187
metab_12273	neg	521.3120	7.1266	1.1839	1.9431	0.0237	0.0707
metab_7718	neg	521.3126	3.4969	1.1296	2.4862	0.0108	0.0417
metab_12391	neg	521.3126	6.7926	1.1775	1.7097	0.0040	0.0210
metab_6927	neg	521.3128	3.7488	0.9657	2.0930	0.0229	0.0692
metab_6962	neg	521.3129	4.6380	0.3432	1.2370	0.4879	0.6095
metab_4448	pos	521.3186	6.4794	0.6592	-2.9775	0.2824	0.4121
metab_10208	neg	521.3606	8.3858	2.1636	-4.1289	0.0000	0.0009
metab_3858	pos	521.3613	9.2206	1.4807	-2.3861	0.0508	0.1152
metab_3737	pos	521.4556	9.7899	1.0994	1.7150	0.0116	0.0399
metab_2747	pos	521.6593	7.4415	0.6048	0.7868	0.0776	0.1571
metab_4142	pos	521.8202	8.0560	0.4229	0.0579	0.5002	0.6230
metab_6790	neg	522.1834	0.8240	0.3569	-0.0931	0.5022	0.6222
metab_13759	neg	522.2101	2.2912	0.4732	-0.1440	0.3522	0.4857
metab_8391	neg	522.2212	1.6028	1.4221	13.4930	0.0003	0.0037
metab_9228	neg	522.2488	3.8333	0.8564	1.8569	0.1228	0.2251
metab_11349	neg	522.2666	9.8620	0.3092	0.7884	0.3774	0.5076
metab_2175	pos	522.2737	3.5620	0.9047	2.6781	0.2794	0.4085
metab_88	pos	522.2815	7.2614	0.4812	0.6454	0.2215	0.3439
metab_10050	neg	522.2846	7.7875	1.3348	2.6943	0.0063	0.0288
metab_4416	pos	522.2899	6.7217	1.9071	-3.9624	0.0004	0.0041
metab_7003	neg	522.2929	6.2456	0.7631	-1.4013	0.1010	0.1953
metab_4744	pos	522.2986	4.9115	0.6022	-0.9192	0.1163	0.2118
metab_2321	pos	522.2987	4.3972	0.0688	0.0593	0.7822	0.8538
metab_4922	pos	522.3015	4.0479	0.2765	1.1069	0.6404	0.7436
metab_2846	pos	522.3260	8.2289	0.0176	-1.2012	0.8669	0.9148
metab_116	pos	522.3544	8.0988	1.2035	1.9953	0.0002	0.0027
metab_4890	pos	522.3780	4.2301	1.3381	3.3236	0.0187	0.0555
metab_6587	neg	523.0477	0.5571	0.7150	-0.3712	0.0062	0.0286
metab_8582	neg	523.1337	1.9904	1.5838	4.3469	0.0012	0.0094
metab_7467	neg	523.1677	1.5155	1.1456	-1.4727	0.0011	0.0089
metab_8888	neg	523.2040	2.7475	0.6206	-0.0221	0.2966	0.4273
metab_10006	neg	523.2320	7.6323	0.7138	1.2839	0.0962	0.1889

metab_9609	neg	523.2687	5.6776	1.5370	-2.3161	0.0222	0.0676
metab_7352	neg	523.2688	8.0843	1.2541	-1.8587	0.0253	0.0741
metab_11876	neg	523.2698	8.3528	1.3428	-1.7403	0.0123	0.0456
metab_12952	neg	523.2915	4.3716	0.9443	1.2219	0.0005	0.0056
metab_12424	neg	523.3012	6.6320	1.4904	-1.0087	0.0859	0.1738
metab_6994	neg	523.3126	5.6615	0.5442	1.6283	0.2308	0.3561
metab_7400	neg	523.3281	6.4874	0.8300	1.1236	0.0152	0.0524
metab_12562	neg	523.3282	6.0670	1.0689	1.5984	0.0013	0.0099
metab_9561	neg	523.3283	5.4188	1.4469	4.3431	0.0129	0.0470
metab_12973	neg	523.3287	4.3037	1.1554	2.9037	0.0235	0.0704
metab_6958	neg	523.3289	4.5219	0.9798	2.2887	0.0347	0.0912
metab_2961	pos	523.3339	8.8618	0.8933	-0.0715	0.2727	0.4009
metab_4237	pos	523.3469	7.5892	0.7349	0.7131	0.0030	0.0155
metab_10281	neg	523.3626	8.6406	0.3172	-1.1896	0.5011	0.6217
metab_4616	pos	523.3729	5.5715	0.1926	0.2810	0.7881	0.8577
metab_10526	neg	523.4376	9.6477	0.4150	1.2092	0.2650	0.3953
metab_14393	neg	524.1630	1.3669	0.9504	-1.5872	0.0918	0.1822
metab_14245	neg	524.1897	1.5297	0.2734	1.0453	0.6145	0.7174
metab_6231	pos	524.1961	0.8221	0.2052	-0.2911	0.6667	0.7647
metab_749	pos	524.2975	7.9260	1.2988	2.4025	0.0097	0.0353
metab_596	pos	524.3146	4.4580	0.3360	-0.3767	0.2089	0.3287
metab_6987	neg	524.3167	4.9838	1.5085	-1.9577	0.0015	0.0109
metab_7393	neg	524.3233	6.8565	0.1399	0.0573	0.7982	0.8554
metab_3978	pos	524.3696	8.6717	0.6500	-0.7253	0.0125	0.0418
metab_10123	neg	525.2475	8.0688	0.9804	3.8787	0.0227	0.0687
metab_7420	neg	525.2851	5.8569	0.4929	-1.2247	0.5163	0.6346
metab_4287	pos	525.2882	7.2763	0.0397	0.3238	0.8400	0.8954
metab_13029	neg	525.3085	4.1023	1.4137	3.2580	0.0020	0.0133
metab_9719	neg	525.3433	6.3269	0.6495	-0.3953	0.1389	0.2471
metab_9799	neg	525.3435	6.7603	0.6649	-0.7305	0.0237	0.0707
metab_12564	neg	525.3436	6.0510	0.9795	-1.0439	0.0129	0.0469
metab_2824	pos	525.3629	8.0988	1.2574	2.3155	0.0004	0.0042
metab_2449	pos	525.3675	5.2573	1.4584	14.6708	0.0006	0.0056
metab_11717	neg	525.3708	8.7191	2.2379	-4.4566	0.0000	0.0004
metab_10374	neg	525.3805	8.9903	1.4956	3.7959	0.0146	0.0510
metab_12386	neg	525.3915	6.8093	0.9397	2.2505	0.1479	0.2588
metab_11359	neg	525.4533	9.8129	0.8786	1.9430	0.0543	0.1247
metab_7888	neg	526.1185	0.5711	0.9417	-1.0613	0.0020	0.0130
metab_8047	neg	526.1784	0.9933	0.3947	0.6704	0.2407	0.3670
metab_8422	neg	526.1958	1.6642	0.6901	-0.6359	0.1653	0.2809
metab_10266	neg	526.2273	8.6093	1.3953	-1.7213	0.0000	0.0001
metab_12413	neg	526.2479	6.6962	0.7114	1.8335	0.2680	0.3982
metab_9700	neg	526.2585	6.1968	0.4914	0.8408	0.4437	0.5676
metab_9269	neg	526.2798	3.9850	0.1283	-0.3380	0.8340	0.8835
metab_12138	neg	526.3160	7.6169	1.4813	2.6849	0.0001	0.0023
metab_623	pos	526.3298	4.9856	1.3776	-2.0714	0.0050	0.0218
metab_12691	neg	526.3328	5.5164	1.0371	-1.1167	0.0676	0.1461
metab_12664	neg	526.3329	5.6452	1.1819	-1.0610	0.0320	0.0865

metab_10258	neg	526.3505	8.5768	1.4996	-2.3502	0.0006	0.0063
metab_3824	pos	526.3882	9.3891	0.6953	-0.8226	0.1944	0.3108
metab_7159	neg	526.8497	14.0438	0.1743	0.0159	0.4451	0.5689
metab_6417	pos	527.0695	0.5420	0.7726	1.7469	0.1326	0.2336
metab_6589	neg	527.1388	0.5991	0.7399	1.0835	0.0074	0.0323
metab_13893	neg	527.1795	2.0358	0.6083	-0.9431	0.2188	0.3424
metab_13713	neg	527.1891	2.3855	0.6986	-0.5847	0.1926	0.3124
metab_11628	neg	527.3056	8.9574	0.3659	0.7196	0.6017	0.7063
metab_10359	neg	527.3384	8.9252	1.5932	3.2741	0.0007	0.0068
metab_4596	pos	527.3831	5.6934	1.2827	-1.8136	0.0051	0.0222
metab_9396	neg	528.1358	4.5553	1.6129	-4.4218	0.0136	0.0486
metab_14519	neg	528.1579	1.2236	0.7050	2.3272	0.2970	0.4276
metab_11622	neg	528.2429	8.9903	1.1756	-1.4099	0.0174	0.0574
metab_1612	pos	528.2440	1.5284	0.9334	3.0139	0.0197	0.0576
metab_12274	neg	528.2637	7.1266	1.5019	3.2762	0.0630	0.1392
metab_12441	neg	528.2740	6.5829	1.1619	1.6912	0.0082	0.0346
metab_2252	pos	528.2923	3.9722	0.1387	-1.8210	0.8820	0.9248
metab_10102	neg	528.3184	7.9908	0.9401	-0.9164	0.0524	0.1215
metab_2497	pos	528.3458	5.6473	0.9610	-1.1960	0.1065	0.1984
metab_2883	pos	528.4037	8.4074	0.4503	1.1961	0.3340	0.4682
metab_3795	pos	528.4064	9.5283	1.0515	-1.6134	0.0006	0.0052
metab_3236	pos	528.4086	11.8273	0.2521	-0.1276	0.3125	0.4453
metab_4353	pos	528.4091	6.9935	0.0246	-0.1339	0.9380	0.9612
metab_3589	pos	528.4091	10.8080	0.1910	-0.1817	0.1783	0.2921
metab_6770	neg	529.1891	0.7114	1.2255	2.1942	0.0070	0.0311
metab_4149	pos	529.3158	8.0419	1.6700	2.9828	0.0017	0.0106
metab_2604	pos	529.3159	6.5704	0.0069	-1.4758	0.8905	0.9303
metab_7090	neg	529.3503	8.5937	0.4439	5.7404	0.5315	0.6478
metab_7104	neg	529.3525	8.8608	0.8488	0.8250	0.1970	0.3175
metab_4410	pos	529.3774	6.7516	1.3147	-1.3700	0.0168	0.0512
metab_773	pos	529.3883	8.3763	0.7800	-2.6906	0.1917	0.3074
metab_8205	neg	530.1453	1.3231	1.4660	14.1862	0.0027	0.0163
metab_8221	neg	530.1732	1.3517	0.1573	-0.5276	0.7761	0.8402
metab_6758	neg	530.1736	0.6551	0.0969	0.1786	0.7992	0.8562
metab_8540	neg	530.2096	1.8974	0.0761	1.3684	0.9834	0.9884
metab_6133	pos	530.2429	1.0321	1.8478	-7.5329	0.0004	0.0045
metab_5402	pos	530.2433	2.4224	1.7773	3.4260	0.0001	0.0016
metab_12546	neg	530.2898	6.1482	1.1643	1.6875	0.0021	0.0135
metab_12449	neg	530.2899	6.5190	1.0354	1.5248	0.0196	0.0619
metab_13167	neg	530.2983	3.6812	1.7912	8.7946	0.0042	0.0215
metab_4446	pos	530.3240	6.4948	0.8001	-3.3511	0.1974	0.3149
metab_2685	pos	530.4189	7.0381	0.6665	-0.9321	0.0327	0.0837
metab_2908	pos	530.4191	8.5554	0.1314	0.1560	0.7493	0.8288
metab_7898	neg	531.2047	0.5851	0.6672	-0.5594	0.0164	0.0552
metab_7788	neg	531.2527	0.4807	0.4024	-0.4545	0.5012	0.6217
metab_8226	neg	531.2567	1.3669	0.4462	-10.1239	0.0454	0.1102
metab_9820	neg	531.2738	6.8565	0.1365	0.3246	0.8051	0.8607
metab_10096	neg	531.3189	7.9757	1.2557	-1.2608	0.0065	0.0292

metab_11612	neg	531.3666	9.0067	0.8694	2.7220	0.2585	0.3878
metab_3924	pos	531.4033	8.9367	0.8662	-1.9477	0.0874	0.1717
metab_3960	pos	531.4034	8.7303	1.2395	-2.9885	0.1232	0.2214
metab_3827	pos	531.4035	9.3736	1.2419	-2.8500	0.0176	0.0531
metab_7013	neg	532.1035	6.5989	1.5955	-4.8175	0.0005	0.0055
metab_13532	neg	532.1979	2.7475	1.0058	3.3731	0.1425	0.2520
metab_12222	neg	532.3048	7.3017	0.6302	1.2776	0.2649	0.3951
metab_2199	pos	532.3118	3.6835	1.9760	13.9633	0.0015	0.0099
metab_9379	neg	532.3137	4.4722	1.1666	3.0260	0.0428	0.1059
metab_4302	pos	532.3191	7.2014	0.2499	0.2888	0.5336	0.6543
metab_2681	pos	532.3830	7.0228	0.3854	0.1381	0.0821	0.1640
metab_886	pos	532.5079	10.4704	0.5291	-0.8344	0.2969	0.4276
metab_11090	neg	532.8668	14.0282	0.3702	-0.1771	0.0952	0.1874
metab_6793	neg	533.0668	0.8662	1.2778	-1.4631	0.0032	0.0180
metab_12167	neg	533.1619	7.5397	0.3656	0.6620	0.4113	0.5384
metab_10552	neg	533.1622	9.7470	0.0351	0.2565	0.9748	0.9821
metab_6768	neg	533.1729	0.6131	1.1964	1.9156	0.0000	0.0007
metab_12715	neg	533.2742	5.3713	1.3891	3.8274	0.2280	0.3530
metab_12422	neg	533.3127	6.6320	0.4475	5.2027	0.7020	0.7861
metab_3081	pos	533.4555	9.6521	0.4156	-0.0455	0.3532	0.4882
metab_14988	neg	534.1648	0.5126	1.0490	-0.8937	0.0342	0.0903
metab_9652	neg	534.2096	5.9375	0.6849	-2.0317	0.2344	0.3599
metab_5286	pos	534.2106	2.7341	1.1642	3.7167	0.0511	0.1158
metab_9625	neg	534.2483	5.7585	0.1397	0.0652	0.8057	0.8612
metab_12198	neg	534.2843	7.4300	0.5465	-0.1521	0.0008	0.0073
metab_2535	pos	534.3181	5.9208	0.1594	-1.2651	0.8363	0.8922
metab_4942	pos	534.3417	3.9722	2.4166	7.2998	0.0007	0.0058
metab_5114	pos	534.3421	3.2880	1.7088	6.1767	0.0018	0.0108
metab_109	pos	534.4868	10.0715	0.1734	0.2865	0.7207	0.8070
metab_11079	neg	534.8620	14.0282	0.3597	-0.0962	0.1413	0.2503
metab_14633	neg	535.0381	0.9508	1.6879	2.4249	0.0150	0.0518
metab_14958	neg	535.0382	0.5286	1.7074	2.8881	0.0045	0.0227
metab_6215	pos	535.0795	0.8500	0.6781	-0.6699	0.0060	0.0250
metab_7492	neg	535.1723	1.7572	1.4038	2.9571	0.0238	0.0709
metab_10121	neg	535.1782	8.0688	1.3027	2.2302	0.0194	0.0616
metab_8712	neg	535.2558	2.2912	1.1801	13.6204	0.0117	0.0438
metab_9046	neg	535.2571	3.1797	2.8025	10.9750	0.0000	0.0012
metab_13140	neg	535.2922	3.7658	1.5624	3.8217	0.0016	0.0114
metab_9938	neg	535.3041	7.3343	0.7036	1.3013	0.1071	0.2042
metab_12208	neg	535.3493	7.3659	0.6269	0.3831	0.3202	0.4523
metab_10089	neg	535.3759	7.9454	0.9708	-1.9227	0.0403	0.1014
metab_11886	neg	535.3759	8.3209	0.6871	-2.2448	0.0262	0.0758
metab_11768	neg	535.3762	8.6728	0.8040	-1.0604	0.1329	0.2390
metab_3122	pos	535.4254	9.8673	0.5019	0.3090	0.0298	0.0785
metab_11398	neg	535.4378	9.6644	1.0758	2.7868	0.0532	0.1228
metab_3692	pos	535.4711	9.9307	0.2817	0.4726	0.3849	0.5173
metab_14931	neg	536.0416	0.5286	0.9021	3.3478	0.0171	0.0566
metab_5024	pos	536.1390	3.6229	0.6354	-0.6807	0.0267	0.0722

metab_14579	neg	536.1626	1.0642	0.6063	-0.8622	0.2305	0.3558
metab_3146	pos	536.1640	10.0715	0.1122	0.0740	0.6787	0.7746
metab_11344	neg	536.2457	9.8620	0.2165	0.0807	0.4715	0.5940
metab_4587	pos	536.2609	5.7540	0.3290	-0.0200	0.4948	0.6186
metab_12642	neg	536.2638	5.7259	0.0254	0.2946	0.9928	0.9946
metab_4281	pos	536.2969	7.3071	0.2242	-0.3012	0.3587	0.4929
metab_9817	neg	536.3001	6.8411	0.0885	-0.1635	0.8968	0.9310
metab_10075	neg	536.3030	7.8984	0.3549	-0.0935	0.1917	0.3114
metab_135	pos	536.3337	5.8753	0.3768	-1.6409	0.4273	0.5588
metab_2361	pos	536.3572	4.6398	2.0996	4.9784	0.0029	0.0154
metab_776	pos	536.3704	8.4074	1.5396	3.4384	0.0007	0.0059
metab_2872	pos	536.3710	8.3322	1.7121	3.7065	0.0000	0.0009
metab_6825	neg	537.1217	1.0215	0.3026	-0.2270	0.4125	0.5389
metab_7953	neg	537.1224	0.6691	0.3314	-0.3719	0.3486	0.4814
metab_13991	neg	537.1693	1.8682	1.6952	-3.0022	0.0016	0.0115
metab_13179	neg	537.1822	3.6479	2.1018	-6.7519	0.0000	0.0000
metab_9087	neg	537.2723	3.3304	2.3172	16.4729	0.0001	0.0022
metab_12259	neg	537.2841	7.1749	1.1428	-1.5321	0.0477	0.1140
metab_5226	pos	537.2845	2.9192	0.2179	0.2898	0.7863	0.8566
metab_9215	neg	537.3079	3.8001	1.2212	2.7405	0.0055	0.0262
metab_12063	neg	537.3207	7.8026	1.6676	4.4446	0.0014	0.0106
metab_7108	neg	537.3525	8.9574	1.3547	-2.3861	0.0009	0.0081
metab_7282	neg	537.3529	9.1858	2.3647	-4.9011	0.0000	0.0010
metab_4035	pos	537.3890	8.4504	0.3975	-0.7925	0.4460	0.5762
metab_3819	pos	537.4507	9.4049	1.4393	3.3934	0.0043	0.0199
metab_7132	neg	537.4533	9.7965	0.9390	1.5402	0.0110	0.0422
metab_3157	pos	537.4866	10.1331	0.8105	1.1646	0.0456	0.1067
metab_3199	pos	537.5342	10.7305	0.4230	-0.3044	0.0111	0.0383
metab_5944	pos	538.1985	1.3579	1.8750	-5.2529	0.0025	0.0137
metab_4554	pos	538.2766	5.9361	0.8035	2.1459	0.1842	0.2990
metab_7418	neg	538.2801	5.8896	0.7943	2.1036	0.1287	0.2331
metab_474	pos	538.2934	2.6719	0.0636	-0.6615	0.9865	0.9917
metab_2183	pos	538.2935	3.6073	0.6493	-1.2722	0.2812	0.4105
metab_2260	pos	538.2936	4.0183	0.8873	-1.1491	0.0561	0.1240
metab_6943	neg	538.2964	4.1193	0.2654	-0.0400	0.3548	0.4877
metab_12884	neg	538.3001	4.6380	0.4349	0.1165	0.1990	0.3196
metab_12249	neg	538.3157	7.2069	1.6919	5.5513	0.0029	0.0170
metab_9940	neg	538.3164	7.3508	0.3547	1.3755	0.3834	0.5129
metab_2664	pos	538.3498	6.9477	0.5458	0.2942	0.3743	0.5079
metab_5100	pos	538.3731	3.3337	1.8967	4.3712	0.0047	0.0209
metab_11509	neg	538.3909	9.2674	1.3562	-2.9581	0.0022	0.0140
metab_13790	neg	539.0278	2.2272	0.5978	-0.2414	0.0055	0.0264
metab_10990	neg	539.0278	14.1090	0.2441	-0.0684	0.2711	0.4017
metab_14079	neg	539.0278	1.7419	0.7069	-0.4912	0.0071	0.0311
metab_8113	neg	539.0279	1.1796	0.7869	-0.5130	0.0008	0.0075
metab_6141	pos	539.1342	1.0180	0.1749	-0.2919	0.6288	0.7338
metab_6801	neg	539.1621	0.8946	0.3059	1.4070	0.4815	0.6037
metab_5025	pos	539.1946	3.6229	1.9627	-3.7080	0.0003	0.0034

metab_13256	neg	539.2354	3.4303	1.0748	5.6422	0.0407	0.1020
metab_12837	neg	539.2672	4.8049	0.2564	0.0852	0.4584	0.5813
metab_9154	neg	539.2869	3.5813	1.6205	4.7605	0.0059	0.0275
metab_714	pos	539.2970	7.1701	1.1631	-2.3778	0.0177	0.0533
metab_12127	neg	539.3006	7.6479	0.7527	-0.2528	0.1746	0.2915
metab_13117	neg	539.3239	3.8164	1.0280	2.2540	0.0164	0.0552
metab_7715	neg	539.3240	3.4639	1.2966	3.0820	0.0061	0.0279
metab_7298	neg	539.3864	8.8608	2.0393	-3.7964	0.0000	0.0010
metab_859	pos	539.4661	9.7899	1.5118	2.9126	0.0067	0.0271
metab_10587	neg	539.4691	9.9433	1.2871	2.2289	0.0060	0.0276
metab_10135	neg	540.2277	8.0998	0.5904	2.0255	0.5396	0.6540
metab_12944	neg	540.2593	4.3882	0.9500	-4.4214	0.2880	0.4195
metab_2610	pos	540.2925	6.6148	1.5258	6.1347	0.0075	0.0294
metab_4560	pos	540.2928	5.9049	0.9223	4.7538	0.2285	0.3520
metab_9602	neg	540.3029	5.6615	0.6228	1.7351	0.1744	0.2913
metab_578	pos	540.3092	4.1234	0.4944	-0.5464	0.0753	0.1535
metab_2477	pos	540.3097	5.4809	1.2333	1.7882	0.0183	0.0545
metab_6939	neg	540.3119	4.0516	1.2174	-1.5608	0.0007	0.0068
metab_12456	neg	540.3188	6.4874	1.1588	1.9425	0.0026	0.0156
metab_7359	neg	540.3315	8.0069	0.0910	0.1653	0.8798	0.9179
metab_12601	neg	540.3663	5.8896	1.2545	2.2533	0.1797	0.2976
metab_3851	pos	540.3836	9.2658	0.1548	-0.1474	0.7256	0.8111
metab_5154	pos	540.3891	3.1648	1.6248	4.3666	0.0173	0.0523
metab_3198	pos	540.5339	10.7150	1.5669	-3.1536	0.0002	0.0028
metab_15008	neg	540.8998	0.5126	1.0368	-1.6117	0.0521	0.1209
metab_14638	neg	541.1183	0.9368	1.5401	-4.8947	0.0001	0.0018
metab_10235	neg	541.1408	8.4972	1.2110	-11.4258	0.0031	0.0177
metab_14658	neg	541.1892	0.8946	1.6519	3.4514	0.0139	0.0493
metab_12696	neg	541.2231	5.4844	0.5615	0.8443	0.5738	0.6836
metab_4328	pos	541.2761	7.0670	0.3913	0.3875	0.4681	0.5954
metab_9588	neg	541.2850	5.5966	1.5341	2.5901	0.0000	0.0007
metab_12559	neg	541.2946	6.0831	1.3941	-2.1970	0.0026	0.0157
metab_7040	neg	541.3029	7.2225	0.1920	0.3386	0.7841	0.8460
metab_9574	neg	541.3063	5.5164	0.8785	2.6865	0.1868	0.3056
metab_13265	neg	541.3393	3.3971	1.4419	3.3199	0.0176	0.0579
metab_13471	neg	541.3393	2.8606	1.1792	6.0783	0.0347	0.0912
metab_9634	neg	541.3864	5.7913	1.2265	4.8556	0.0846	0.1717
metab_14479	neg	542.2098	1.2663	1.5113	4.1398	0.0394	0.0998
metab_9896	neg	542.2221	7.1266	1.0268	-0.8711	0.1261	0.2296
metab_9559	neg	542.2430	5.4034	0.2571	1.9512	0.7545	0.8233
metab_12670	neg	542.2434	5.6128	0.3564	2.2149	0.6288	0.7277
metab_11084	neg	542.2509	14.0282	0.5392	1.0588	0.1901	0.3095
metab_12818	neg	542.2747	4.8865	1.5367	-4.4307	0.0316	0.0857
metab_2606	pos	542.3036	6.5857	0.5252	0.3263	0.2642	0.3920
metab_12542	neg	542.3186	6.1644	1.1020	2.4712	0.0347	0.0912
metab_728	pos	542.3203	7.4703	0.3202	-0.6544	0.3754	0.5092
metab_13286	neg	542.3208	3.3304	1.2226	5.9160	0.0793	0.1644
metab_567	pos	542.3249	4.0479	1.1295	-1.6449	0.0013	0.0089

metab_4050	pos	542.4195	8.4074	0.5111	1.5049	0.2888	0.4193
metab_2745	pos	542.4237	7.4415	0.5966	-0.7640	0.0415	0.0993
metab_3557	pos	542.4248	11.6464	0.3449	-0.3573	0.1160	0.2114
metab_3224	pos	542.4251	11.2221	0.2110	-0.1776	0.2366	0.3604
metab_3654	pos	542.4924	10.1331	1.2863	-12.8146	0.0081	0.0310
metab_1328	pos	543.1309	0.6402	0.3613	-1.3016	0.4460	0.5762
metab_10054	neg	543.2266	7.7875	1.3902	-1.6910	0.0046	0.0231
metab_12012	neg	543.2271	7.9908	1.2658	-1.3193	0.0057	0.0268
metab_12412	neg	543.2583	6.6962	1.4144	-2.2637	0.0019	0.0128
metab_13329	neg	543.2741	3.2130	2.0273	9.0738	0.0007	0.0069
metab_11814	neg	543.3299	8.5604	1.1188	-1.0397	0.0017	0.0116
metab_6812	neg	544.2254	0.9508	1.8176	-3.8890	0.0109	0.0419
metab_4154	pos	544.2635	7.9980	0.3578	0.0622	0.5464	0.6652
metab_10016	neg	544.2666	7.6793	0.4655	0.5497	0.0165	0.0553
metab_11077	neg	544.2666	14.0282	1.5675	3.6330	0.0034	0.0185
metab_2885	pos	544.3986	8.4074	0.7649	1.1879	0.2339	0.3579
metab_7247	neg	544.4594	9.7965	1.1387	-1.6541	0.0463	0.1116
metab_11050	neg	544.8223	14.0438	0.3389	-0.1298	0.0974	0.1906
metab_6743	neg	545.1843	0.5991	0.4908	-0.5827	0.1813	0.2994
metab_14248	neg	545.1998	1.5297	0.2937	0.5685	0.4851	0.6071
metab_9067	neg	545.2052	3.2637	1.1534	-3.3534	0.0404	0.1016
metab_14437	neg	545.2360	1.3089	1.5669	-2.9867	0.0008	0.0070
metab_11842	neg	545.2424	8.4821	0.5197	0.6136	0.3616	0.4944
metab_12230	neg	545.2532	7.2701	1.5724	2.6160	0.0091	0.0375
metab_12053	neg	545.3336	7.8507	2.7910	-10.3113	0.0000	0.0000
metab_10365	neg	545.3481	8.9574	0.2983	0.7887	0.3867	0.5161
metab_10298	neg	545.3493	8.7031	0.5843	0.9305	0.1041	0.2000
metab_7334	neg	545.3494	8.4505	0.7303	1.2271	0.0432	0.1064
metab_8017	neg	546.1684	0.8946	0.3362	0.7008	0.3239	0.4565
metab_14604	neg	546.2047	1.0074	0.5834	1.7365	0.1228	0.2251
metab_11925	neg	546.2826	8.1936	1.1102	1.6455	0.0043	0.0219
metab_7070	neg	546.2827	8.0688	0.5999	3.2196	0.0483	0.1150
metab_3968	pos	546.3521	8.7008	1.1256	-3.7035	0.0070	0.0279
metab_2755	pos	546.3982	7.4855	0.2408	-0.3854	0.3501	0.4853
metab_3564	pos	546.3988	11.3883	0.3153	-0.2334	0.0191	0.0563
metab_3715	pos	546.3989	9.8522	0.5382	-0.4056	0.0075	0.0293
metab_3079	pos	546.4872	9.6358	1.1099	1.3497	0.0003	0.0032
metab_10783	neg	546.8180	14.0438	0.4125	-0.2077	0.0508	0.1186
metab_10022	neg	547.2683	7.6951	1.3420	2.3957	0.0106	0.0412
metab_4717	pos	547.3259	4.9698	0.1753	-1.9831	0.6839	0.7781
metab_11455	neg	547.4379	9.4812	1.3094	-1.1506	0.0027	0.0160
metab_3118	pos	547.4712	9.8522	1.1237	1.8852	0.0165	0.0507
metab_1335	pos	548.0766	0.6682	0.8589	1.1557	0.0980	0.1867
metab_9407	neg	548.2504	4.5884	1.0136	3.0553	0.1130	0.2123
metab_12283	neg	548.2643	7.0959	0.5145	0.8969	0.1982	0.3189
metab_5562	pos	548.2905	2.0973	1.6432	14.0277	0.0020	0.0121
metab_12377	neg	548.3001	6.8411	0.5261	1.2368	0.3156	0.4475
metab_12213	neg	548.3002	7.3508	0.6617	0.9141	0.0381	0.0977

metab_9969	neg	548.3003	7.4776	0.0810	0.0904	0.8881	0.9251
metab_4094	pos	548.3695	8.2289	0.6587	1.1619	0.0503	0.1144
metab_11639	neg	548.3753	8.9417	0.2346	1.4198	0.6776	0.7678
metab_3531	pos	548.4650	13.8585	0.2005	0.0035	0.2362	0.3601
metab_3509	pos	548.4653	14.0378	0.1590	-0.0006	0.3147	0.4473
metab_3551	pos	548.4654	12.2734	0.1640	-0.1845	0.4952	0.6190
metab_3253	pos	548.4655	13.2651	0.0186	-0.0811	0.9202	0.9484
metab_3242	pos	548.4655	12.4952	0.0229	-0.0890	0.8156	0.8789
metab_3533	pos	548.4656	13.6960	0.1659	-0.2695	0.4458	0.5762
metab_3543	pos	548.4657	12.9103	0.3382	-0.2507	0.0565	0.1249
metab_110	pos	548.5026	10.2085	0.1928	-0.1253	0.4749	0.6019
metab_3083	pos	548.5027	9.6671	0.7184	1.1629	0.0890	0.1741
metab_1273	pos	549.0010	0.5560	0.8914	-1.1002	0.0410	0.0986
metab_9534	neg	549.1046	5.2579	0.3531	-0.0609	0.0935	0.1848
metab_12594	neg	549.1571	5.9375	0.3009	-0.7580	0.6222	0.7230
metab_7913	neg	549.1680	0.5991	0.5635	0.7188	0.0725	0.1535
metab_9119	neg	549.2715	3.4800	2.3937	7.0940	0.0006	0.0064
metab_9395	neg	549.2882	4.5553	2.8079	13.6514	0.0016	0.0113
metab_11406	neg	549.4535	9.6477	0.4631	0.1905	0.2625	0.3924
metab_3651	pos	549.4869	10.1647	0.2974	0.7061	0.4368	0.5677
metab_14250	neg	550.0801	1.5297	0.1750	-0.0838	0.6557	0.7503
metab_6042	pos	550.1260	1.2016	0.3448	-0.4917	0.3572	0.4916
metab_9352	neg	550.2668	4.3207	2.1524	4.2953	0.0085	0.0355
metab_12153	neg	550.2795	7.5702	2.0676	4.3126	0.0022	0.0141
metab_9618	neg	550.2800	5.7259	0.5642	-1.6997	0.2943	0.4253
metab_9563	neg	550.2802	5.4188	1.1238	2.6329	0.0169	0.0561
metab_4254	pos	550.3114	7.4703	0.4689	-0.5984	0.2351	0.3592
metab_723	pos	550.3120	7.3370	0.6695	-0.7763	0.0450	0.1055
metab_9887	neg	550.3155	7.0796	3.0168	7.7288	0.0008	0.0074
metab_12239	neg	550.3160	7.2225	0.9181	1.5945	0.0374	0.0962
metab_7363	neg	550.3161	7.8657	1.0283	1.6268	0.0026	0.0155
metab_2156	pos	550.3367	3.4857	2.7445	9.9384	0.0007	0.0060
metab_3971	pos	550.3858	8.7008	0.3296	0.1953	0.2896	0.4202
metab_11384	neg	550.4843	9.7312	1.7212	7.3594	0.0147	0.0513
metab_9789	neg	551.1884	6.6962	0.9979	2.2429	0.0964	0.1891
metab_13173	neg	551.2357	3.6645	2.1626	9.5161	0.0019	0.0128
metab_13164	neg	551.2874	3.6980	1.9892	4.9246	0.0012	0.0096
metab_12234	neg	551.2999	7.2377	0.6734	1.5698	0.1465	0.2572
metab_10362	neg	551.3302	8.9417	2.3584	-7.7303	0.0025	0.0152
metab_7413	neg	551.3320	6.1482	0.2957	-0.3071	0.3064	0.4375
metab_845	pos	551.4661	9.5899	0.2427	0.3708	0.4455	0.5761
metab_7234	neg	551.4691	9.9767	0.2788	0.7096	0.3017	0.4322
metab_883	pos	551.5023	10.3480	1.4358	2.7573	0.0226	0.0636
metab_11960	neg	552.2046	8.0998	1.5680	-2.4210	0.0004	0.0049
metab_12223	neg	552.2054	7.3017	0.5758	0.9090	0.1211	0.2228
metab_12617	neg	552.2591	5.8074	1.3075	2.5145	0.0333	0.0891
metab_12814	neg	552.2593	4.9183	0.4219	0.5110	0.2335	0.3589
metab_9427	neg	552.2823	4.6722	1.7653	4.0853	0.0006	0.0061

metab_4688	pos	552.3286	5.1533	0.4663	-2.2175	0.6274	0.7331
metab_4889	pos	552.3289	4.2301	0.4966	-3.7798	0.5024	0.6249
metab_7062	neg	552.3318	7.7567	1.1928	2.0096	0.0008	0.0072
metab_2155	pos	552.3519	3.4857	1.6479	4.2537	0.0006	0.0055
metab_3780	pos	552.4969	9.6044	1.1220	1.3665	0.1869	0.3023
metab_3095	pos	552.4974	9.7283	0.6697	2.5867	0.2770	0.4057
metab_15125	neg	552.7236	0.0197	0.2648	-0.0226	0.1936	0.3137
metab_1341	pos	553.0551	0.6962	0.3748	-0.2608	0.5426	0.6622
metab_14667	neg	553.1898	0.8803	1.0980	3.3008	0.0573	0.1297
metab_7568	neg	553.2378	2.2119	1.0114	1.7507	0.0966	0.1895
metab_9598	neg	553.2793	5.6292	1.4026	-4.0901	0.0189	0.0607
metab_7676	neg	553.3032	3.1797	1.5459	3.7620	0.0013	0.0099
metab_7300	neg	553.3481	8.8286	0.6500	-0.4630	0.0092	0.0378
metab_11877	neg	553.3870	8.3528	0.1263	2.2212	0.9677	0.9772
metab_11473	neg	553.4483	9.4322	1.0582	1.7152	0.0098	0.0393
metab_3691	pos	553.4818	9.9307	0.5044	0.7906	0.1313	0.2320
metab_7142	neg	553.4848	10.0749	0.8959	1.4939	0.0066	0.0298
metab_14705	neg	554.1132	0.7958	0.7019	1.2084	0.0114	0.0430
metab_14498	neg	554.1521	1.2521	0.7614	-0.6387	0.3133	0.4449
metab_9491	neg	554.2753	4.9668	0.8154	1.1535	0.1475	0.2585
metab_1988	pos	554.2922	2.8108	1.8478	7.3248	0.0017	0.0104
metab_9422	neg	554.2949	4.6550	1.0431	1.7362	0.0102	0.0402
metab_9958	neg	554.3031	7.4465	0.7155	0.9256	0.0053	0.0254
metab_12148	neg	554.3034	7.5855	0.1851	0.2256	0.5614	0.6726
metab_4696	pos	554.3443	5.1076	0.2920	0.3837	0.5487	0.6672
metab_10200	neg	554.3469	8.3528	1.1221	1.7917	0.0019	0.0128
metab_5087	pos	554.3679	3.3793	2.2573	6.8945	0.0010	0.0076
metab_12535	neg	554.3821	6.1804	1.3280	3.4613	0.0031	0.0175
metab_11679	neg	554.3825	8.7807	1.0102	-1.5250	0.0077	0.0331
metab_834	pos	554.4922	9.4049	1.0728	3.0563	0.0809	0.1623
metab_6679	neg	554.7197	0.0197	0.3759	-0.0668	0.0516	0.1202
metab_1271	pos	554.9670	0.5560	0.7192	-0.7223	0.0074	0.0291
metab_7875	neg	555.0376	0.5571	1.1395	-1.4713	0.0176	0.0579
metab_5379	pos	555.1896	2.4690	1.9612	-4.5022	0.0001	0.0021
metab_558	pos	555.2504	2.2045	0.8229	0.9275	0.2293	0.3529
metab_6922	neg	555.2759	3.6311	1.4996	-2.7624	0.0007	0.0066
metab_726	pos	555.2911	7.4269	1.7620	-3.6762	0.0001	0.0019
metab_13659	neg	555.3184	2.4970	1.9941	5.7372	0.0001	0.0025
metab_13392	neg	555.3191	3.0604	0.8809	1.9665	0.0504	0.1181
metab_2836	pos	555.3666	8.1560	0.6094	-0.5028	0.2722	0.4004
metab_867	pos	555.4947	9.9470	1.1242	2.3710	0.0673	0.1421
metab_3152	pos	555.4976	10.0875	0.7757	3.0266	0.0386	0.0941
metab_14836	neg	556.1654	0.5991	1.2804	2.2181	0.0015	0.0108
metab_8761	neg	556.1943	2.4010	0.7197	-1.3453	0.1833	0.3017
metab_12305	neg	556.2453	7.0322	1.4024	-1.8321	0.0002	0.0036
metab_5068	pos	556.3040	3.4549	0.6886	-0.8775	0.0723	0.1497
metab_4886	pos	556.3041	4.2457	0.5178	0.7533	0.1003	0.1895
metab_2389	pos	556.3043	4.8668	1.2351	1.8989	0.0054	0.0233

metab_4790	pos	556.3044	4.6398	1.1043	1.6274	0.0034	0.0171
metab_8856	neg	556.3080	2.6524	0.5525	-1.8257	0.3714	0.5028
metab_13514	neg	556.3082	2.7792	0.4825	-1.3450	0.4507	0.5742
metab_2382	pos	556.3401	4.7758	0.9986	-0.8450	0.0326	0.0833
metab_11792	neg	556.4018	8.6093	0.2628	0.3508	0.8270	0.8783
metab_3161	pos	556.5078	10.1793	1.3828	1.3577	0.0682	0.1433
metab_3618	pos	556.5286	10.4092	1.0548	-1.3718	0.0259	0.0707
metab_6506	pos	556.8964	0.4999	0.6086	-3.3324	0.0937	0.1809
metab_7863	neg	557.0089	0.5431	0.1557	0.0749	0.6686	0.7611
metab_14533	neg	557.2207	1.1940	1.6341	4.1402	0.0273	0.0780
metab_13204	neg	557.2488	3.5813	0.7782	2.4965	0.0946	0.1863
metab_14365	neg	557.2725	1.4267	1.7650	-12.8698	0.0003	0.0044
metab_12294	neg	557.2734	7.0629	0.2204	-0.1651	0.4463	0.5699
metab_12137	neg	557.3811	7.6169	0.7129	3.7283	0.5980	0.7038
metab_2954	pos	557.4553	8.8169	0.6322	-0.6562	0.1003	0.1895
metab_10616	neg	557.4578	10.1250	1.4899	-2.3495	0.0281	0.0794
metab_5224	pos	557.8028	2.9342	2.4237	8.0262	0.0003	0.0037
metab_12644	neg	558.2458	5.7102	0.0895	0.2095	0.8016	0.8580
metab_2684	pos	558.2580	7.0381	1.4110	-2.2570	0.0016	0.0103
metab_7043	neg	558.2814	7.3017	0.5747	-0.3515	0.3186	0.4508
metab_13246	neg	558.2928	3.4639	0.9374	2.9844	0.0914	0.1818
metab_12545	neg	558.3015	6.1482	0.5233	-0.1841	0.1284	0.2328
metab_1980	pos	558.3198	2.7804	0.9181	-1.9025	0.0369	0.0909
metab_5115	pos	558.3199	3.2880	0.8603	-1.4684	0.0747	0.1526
metab_12261	neg	558.3806	7.1595	0.0069	-0.1486	0.9032	0.9358
metab_2919	pos	558.4140	8.6132	0.4798	0.1713	0.5592	0.6763
metab_9377	neg	559.2701	4.4722	0.6898	2.7019	0.2504	0.3780
metab_9970	neg	559.3043	7.4776	0.2932	2.1511	0.7216	0.8003
metab_11654	neg	559.3397	8.8929	0.1746	-2.0794	0.7611	0.8286
metab_9807	neg	559.3758	6.7926	0.0354	0.5336	0.9932	0.9948
metab_2989	pos	559.4711	9.0254	0.4737	0.6939	0.1976	0.3151
metab_860	pos	559.4711	9.7435	0.8097	-0.7741	0.0203	0.0588
metab_10651	neg	559.4737	10.3687	0.4528	0.2654	0.6082	0.7119
metab_13929	neg	560.1993	1.9904	2.2497	-7.8418	0.0000	0.0003
metab_8186	neg	560.2204	1.3089	0.1256	0.6912	0.5849	0.6929
metab_12420	neg	560.2325	6.6480	0.2004	0.3269	0.6752	0.7656
metab_9651	neg	560.2617	5.9375	0.0695	-0.7161	0.8626	0.9054
metab_12064	neg	560.2997	7.8026	2.2667	-5.4081	0.0000	0.0001
metab_4508	pos	560.3141	6.1456	0.0018	-0.1894	0.9745	0.9850
metab_4267	pos	560.3339	7.3824	0.8371	2.4500	0.0737	0.1516
metab_2222	pos	560.3354	3.7599	0.6933	-1.5033	0.3155	0.4481
metab_12140	neg	560.3356	7.6009	1.6403	6.6047	0.0063	0.0287
metab_11845	neg	560.3363	8.4667	1.7118	-4.3115	0.0000	0.0006
metab_14890	neg	561.0039	0.5571	1.0640	-1.0836	0.0015	0.0108
metab_14196	neg	561.2306	1.5736	0.3288	1.0809	0.6129	0.7159
metab_11851	neg	561.2565	8.4505	0.2471	0.1221	0.3137	0.4454
metab_2329	pos	561.2829	4.4580	0.1182	1.1042	0.6813	0.7764
metab_10324	neg	561.3447	8.7346	0.3731	-1.4130	0.4038	0.5320

metab_10519	neg	561.4537	9.6147	0.7359	1.2605	0.2074	0.3296
metab_3023	pos	561.4866	9.2049	1.8954	6.7405	0.0003	0.0032
metab_113	pos	561.4867	9.9154	0.2379	0.4392	0.4482	0.5780
metab_7424	neg	561.9302	4.1869	0.7779	-0.3968	0.1179	0.2184
metab_7382	neg	562.2794	7.0959	0.5909	1.0396	0.1756	0.2926
metab_3607	pos	562.2846	10.5629	0.4637	-0.3671	0.0039	0.0185
metab_4719	pos	562.2846	4.9698	0.3197	-0.2813	0.0304	0.0794
metab_3685	pos	562.2848	9.9470	0.6581	-0.5426	0.0029	0.0154
metab_9904	neg	562.3153	7.1595	1.3229	2.2064	0.0008	0.0071
metab_12869	neg	562.3395	4.6883	2.1743	6.3241	0.0007	0.0068
metab_732	pos	562.3474	7.5892	0.1492	0.5022	0.7958	0.8637
metab_11613	neg	562.3887	9.0067	1.2289	2.4952	0.0186	0.0597
metab_14816	neg	563.1772	0.5991	0.9925	1.7329	0.0227	0.0687
metab_6796	neg	563.1834	0.8662	0.6081	-0.0210	0.0570	0.1293
metab_4323	pos	563.2969	7.1119	1.5141	3.4334	0.0002	0.0030
metab_783	pos	563.3476	8.5393	2.2703	-8.2969	0.0068	0.0274
metab_11348	neg	563.4171	9.8620	0.3955	1.3188	0.4047	0.5328
metab_3655	pos	563.4563	10.1331	0.6027	-0.5350	0.0098	0.0355
metab_11364	neg	563.4690	9.7965	1.7326	3.2490	0.0014	0.0103
metab_3155	pos	563.5022	10.1175	0.4434	0.5706	0.2724	0.4005
metab_13008	neg	563.9271	4.1869	0.8002	-0.6703	0.0192	0.0612
metab_12143	neg	563.9738	7.5855	0.3733	0.1995	0.3766	0.5072
metab_12196	neg	563.9751	7.4300	1.2876	2.0575	0.0009	0.0080
metab_9623	neg	564.2588	5.7420	1.0230	2.0769	0.0385	0.0983
metab_4791	pos	564.3084	4.6248	0.3099	-0.2759	0.3813	0.5138
metab_9480	neg	564.3119	4.9183	0.1398	1.7665	0.8931	0.9282
metab_9993	neg	564.3313	7.5855	0.5917	0.7978	0.0550	0.1260
metab_10989	neg	564.3315	14.1090	1.0160	1.5034	0.0066	0.0297
metab_11504	neg	564.3460	9.3000	0.5759	-0.5466	0.1449	0.2550
metab_7135	neg	564.4400	9.8620	1.6025	6.0701	0.0207	0.0645
metab_856	pos	564.4975	9.7586	1.0593	1.2185	0.0000	0.0008
metab_9998	neg	564.6890	7.5855	0.5542	0.5744	0.2799	0.4106
metab_9966	neg	564.6894	7.4618	1.2650	2.2965	0.0002	0.0029
metab_14624	neg	565.0484	0.9649	2.5052	6.6585	0.0000	0.0002
metab_10865	neg	565.0484	14.2390	2.1362	5.0519	0.0000	0.0002
metab_14977	neg	565.0485	0.5286	2.1923	5.2656	0.0000	0.0001
metab_6391	pos	565.1031	0.5703	0.7217	1.0386	0.0629	0.1347
metab_9911	neg	565.2792	7.1749	0.6213	-0.0414	0.1119	0.2110
metab_7017	neg	565.3083	6.7439	0.0773	0.1037	0.6670	0.7598
metab_13069	neg	565.3394	3.9677	0.8833	11.7638	0.0289	0.0809
metab_11826	neg	565.4482	8.5135	0.6405	0.8157	0.3240	0.4565
metab_10456	neg	565.4483	9.2834	0.2295	0.9838	0.5470	0.6593
metab_3572	pos	565.5653	11.1749	0.0041	-0.1029	0.8871	0.9285
metab_14529	neg	566.1241	1.2084	0.5349	-0.3427	0.0860	0.1739
metab_1011	pos	566.3250	4.9267	0.2833	0.8501	0.5761	0.6906
metab_7346	neg	566.3471	8.0998	1.0820	2.0551	0.0010	0.0083
metab_11254	neg	566.4794	10.2225	2.2267	-3.8902	0.0007	0.0068
metab_3097	pos	566.5134	9.7586	0.1686	0.1815	0.5519	0.6697

metab_10142	neg	566.7077	8.1153	1.2640	2.4548	0.0067	0.0299
metab_14462	neg	567.2050	1.2805	0.7660	0.7749	0.1630	0.2779
metab_3007	pos	567.2398	9.1140	0.5340	-0.3477	0.1103	0.2038
metab_3714	pos	567.2399	9.8522	0.0679	0.3745	0.8474	0.9018
metab_3791	pos	567.2400	9.5438	0.4547	0.8642	0.3002	0.4312
metab_9743	neg	567.2586	6.4073	2.2809	-13.3340	0.0000	0.0000
metab_12092	neg	567.2754	7.7251	0.6862	0.8327	0.2482	0.3752
metab_12271	neg	567.2946	7.1437	1.5910	-2.8237	0.0017	0.0117
metab_12654	neg	567.3020	5.6615	0.5676	-0.2215	0.2973	0.4279
metab_9693	neg	567.3270	6.1644	0.2272	-0.2435	0.3913	0.5197
metab_11424	neg	567.4639	9.5979	0.2336	0.5199	0.4990	0.6196
metab_3650	pos	567.4979	10.1647	0.3994	1.0398	0.3530	0.4881
metab_1500	pos	568.1364	1.2016	0.3622	-0.4790	0.3439	0.4786
metab_11682	neg	568.2719	8.7640	1.5536	3.0765	0.0030	0.0174
metab_9185	neg	568.2770	3.6980	1.1056	1.8393	0.0006	0.0063
metab_9409	neg	568.2772	4.6056	1.9873	4.7774	0.0010	0.0088
metab_9646	neg	568.2814	5.9217	0.0498	-2.5059	0.8883	0.9252
metab_10104	neg	568.2872	7.9908	2.2299	-7.1245	0.0001	0.0018
metab_2732	pos	568.3014	7.3370	1.3462	-3.1187	0.0061	0.0251
metab_4143	pos	568.3386	8.0560	0.4239	0.9533	0.4783	0.6050
metab_2177	pos	568.3465	3.5771	1.5811	4.8927	0.0115	0.0395
metab_10284	neg	568.3619	8.6728	0.2171	0.1432	0.5576	0.6691
metab_729	pos	568.3668	7.3824	1.1346	-1.8617	0.0030	0.0155
metab_11645	neg	568.3974	8.9252	1.6956	-3.0517	0.0023	0.0145
metab_7948	neg	569.1365	0.6551	1.1013	2.0749	0.0041	0.0214
metab_14680	neg	569.1366	0.8662	1.0796	1.9494	0.0016	0.0113
metab_8553	neg	569.1569	1.9280	0.2466	1.2680	0.7815	0.8444
metab_8096	neg	569.1845	1.1212	0.4024	-0.0746	0.3702	0.5017
metab_8333	neg	569.2209	1.5297	0.5337	1.9986	0.3427	0.4759
metab_9038	neg	569.2459	3.1626	1.4277	4.7854	0.0151	0.0521
metab_12373	neg	569.2739	6.8565	1.3871	-1.8321	0.0001	0.0026
metab_6964	neg	569.2978	4.6380	1.0260	1.6270	0.0020	0.0131
metab_4316	pos	569.3068	7.1267	1.7030	-5.3068	0.0116	0.0398
metab_4501	pos	569.4297	6.1927	0.4800	-1.0200	0.3595	0.4936
metab_10527	neg	569.4778	9.6477	0.9501	1.8557	0.0406	0.1018
metab_7908	neg	570.1982	0.5991	1.1781	-1.1844	0.0032	0.0180
metab_9181	neg	570.2921	3.6812	1.3129	2.8165	0.0013	0.0103
metab_9006	neg	570.2931	3.0432	2.1245	6.5234	0.0005	0.0056
metab_4758	pos	570.3384	4.8359	1.4637	-3.5505	0.0019	0.0114
metab_4949	pos	570.3392	3.9421	0.1087	-2.6770	0.9284	0.9538
metab_2363	pos	570.3613	4.6398	0.2328	-0.6076	0.4947	0.6186
metab_2319	pos	570.3616	4.3818	0.6673	0.6565	0.0515	0.1165
metab_11230	neg	570.4664	10.3687	1.8312	-3.1835	0.0037	0.0200
metab_6604	neg	571.0924	0.7818	1.0113	-0.7860	0.1170	0.2172
metab_14866	neg	571.1407	0.5711	0.3487	0.7365	0.3281	0.4607
metab_5652	pos	571.1690	1.9309	0.4467	1.3336	0.4209	0.5523
metab_14693	neg	571.2003	0.8240	0.0937	0.6290	0.8927	0.9282
metab_8173	neg	571.2003	1.2805	0.3569	2.2931	0.4642	0.5865

metab_12199	neg	571.2892	7.4300	1.4458	-2.1596	0.0000	0.0003
metab_12389	neg	571.3761	6.7926	1.6566	12.4712	0.0008	0.0071
metab_12056	neg	571.3969	7.8352	0.3711	2.1652	0.7080	0.7908
metab_10276	neg	571.3974	8.6239	1.4735	4.3382	0.0522	0.1212
metab_11582	neg	571.4010	9.0872	0.0751	1.0083	0.8395	0.8880
metab_7272	neg	571.4377	9.3661	0.1541	1.2079	0.9139	0.9428
metab_11399	neg	571.4379	9.6644	1.5663	-2.1616	0.0189	0.0606
metab_3895	pos	571.4709	9.0857	0.5986	0.6095	0.2664	0.3943
metab_7736	neg	571.7096	0.0197	0.1252	0.1207	0.5424	0.6562
metab_14546	neg	572.1842	1.1645	0.9693	2.9549	0.0491	0.1162
metab_13022	neg	572.2308	4.1193	0.4211	-1.6695	0.5361	0.6510
metab_9540	neg	572.2402	5.2899	2.4576	-5.2910	0.0055	0.0264
metab_4978	pos	572.2818	3.8063	0.1268	-0.1956	0.8046	0.8706
metab_9254	neg	572.3085	3.9177	1.0255	1.8742	0.0102	0.0403
metab_5144	pos	572.3412	3.1957	1.5599	7.7888	0.0211	0.0606
metab_10405	neg	572.3760	9.0872	1.3835	-2.0906	0.0037	0.0201
metab_9942	neg	572.3962	7.3508	0.4508	-1.6440	0.4304	0.5553
metab_3234	pos	572.4351	11.6911	0.1997	-0.1763	0.3471	0.4819
metab_3560	pos	572.4352	11.5098	0.2005	-0.2496	0.4319	0.5632
metab_3227	pos	572.4353	11.3742	0.1776	-0.2703	0.4064	0.5395
metab_4354	pos	572.4355	6.9790	0.3191	0.1795	0.2071	0.3269
metab_7227	neg	572.4904	10.0924	1.4289	-2.2125	0.0126	0.0462
metab_3627	pos	572.5239	10.3169	0.5055	-0.4717	0.2603	0.3878
metab_8282	neg	573.2356	1.4568	1.6593	-4.8888	0.0081	0.0342
metab_9183	neg	573.2692	3.6812	1.4660	2.9567	0.0032	0.0180
metab_13104	neg	573.2750	3.8506	0.4126	-0.1272	0.1462	0.2568
metab_12875	neg	573.3075	4.6550	0.4596	2.0005	0.5604	0.6717
metab_11677	neg	573.3706	8.7965	3.1855	-6.9501	0.0000	0.0010
metab_4014	pos	573.3768	8.5554	0.0509	0.6357	0.9849	0.9909
metab_761	pos	573.3776	8.1857	0.0165	0.2321	0.9140	0.9438
metab_2789	pos	573.4094	7.7800	1.0014	2.4227	0.2264	0.3497
metab_768	pos	573.4501	8.2586	0.6551	1.0333	0.2357	0.3597
metab_7273	neg	573.4534	9.3494	0.2895	0.9844	0.6706	0.7623
metab_2972	pos	573.4861	8.9367	1.4818	-2.9083	0.0117	0.0399
metab_3776	pos	573.4865	9.6358	0.3025	-0.4321	0.3739	0.5077
metab_3694	pos	573.4867	9.9307	0.3859	0.6891	0.3904	0.5227
metab_8404	neg	574.1999	1.6335	0.1710	0.2582	0.7490	0.8206
metab_12026	neg	574.3260	7.9454	1.8864	-3.9568	0.0041	0.0213
metab_11757	neg	574.3965	8.6874	1.5520	-2.2624	0.0022	0.0139
metab_3646	pos	574.5181	10.1941	0.4019	1.1536	0.2914	0.4216
metab_6728	neg	575.0194	0.5571	0.7727	-0.5369	0.0117	0.0437
metab_14596	neg	575.0776	1.0215	0.3216	-0.2363	0.4221	0.5480
metab_9935	neg	575.1729	7.3180	0.5500	0.6615	0.3000	0.4303
metab_2233	pos	575.2886	3.8511	0.3989	-0.6045	0.3216	0.4548
metab_6659	neg	575.3298	8.5455	1.6675	-5.2220	0.0087	0.0360
metab_10547	neg	575.4691	9.7470	0.8704	-0.4766	0.0090	0.0371
metab_898	pos	575.5021	10.1647	0.0427	0.3942	0.9701	0.9818
metab_3753	pos	575.5021	9.7283	1.3268	-1.9541	0.0000	0.0002

metab_3190	pos	575.5022	10.5777	0.7829	-0.8210	0.0000	0.0010
metab_13647	neg	576.1878	2.5113	0.5252	0.9857	0.3617	0.4945
metab_12121	neg	576.2040	7.6793	0.6202	0.6591	0.0008	0.0072
metab_12330	neg	576.2044	6.9525	0.2232	0.0203	0.5683	0.6792
metab_9583	neg	576.2279	5.5639	0.3134	1.5430	0.6200	0.7214
metab_12290	neg	576.2951	7.0796	0.4962	0.8489	0.1951	0.3154
metab_12505	neg	576.3314	6.3269	1.1733	2.8317	0.0093	0.0378
metab_9971	neg	576.3316	7.4927	0.3989	1.0076	0.1821	0.3002
metab_2669	pos	576.4088	6.9790	0.1763	0.1020	0.3343	0.4683
metab_3611	pos	576.5333	10.5161	1.6380	3.8694	0.0055	0.0235
metab_12214	neg	577.1879	7.3343	1.0723	-1.3347	0.0837	0.1705
metab_12165	neg	577.2071	7.5397	0.9566	1.2093	0.0009	0.0081
metab_2479	pos	577.2760	5.5112	1.5360	-2.8544	0.0160	0.0496
metab_12943	neg	577.2821	4.3882	0.3428	1.3761	0.5830	0.6912
metab_13556	neg	577.3002	2.6986	1.0455	14.0556	0.0369	0.0953
metab_5356	pos	577.3024	2.5158	1.6199	13.2926	0.0037	0.0178
metab_6997	neg	577.3241	5.6776	2.3485	-3.8977	0.0050	0.0243
metab_2976	pos	577.4811	8.9507	0.3577	-0.4455	0.4137	0.5458
metab_107	pos	577.4817	9.6823	0.3651	-0.2344	0.2864	0.4166
metab_3838	pos	577.4817	9.3278	0.9257	1.1801	0.0062	0.0254
metab_11332	neg	577.4847	9.9108	0.0921	0.5279	0.8747	0.9143
metab_3211	pos	577.5176	10.9772	0.5975	0.6966	0.0228	0.0640
metab_882	pos	577.5180	10.3169	0.4282	1.0428	0.3210	0.4540
metab_6844	neg	578.1944	0.5991	0.6581	-1.1658	0.2354	0.3608
metab_2724	pos	578.2012	7.2763	0.8547	1.3650	0.0629	0.1347
metab_8876	neg	578.2101	2.7155	0.6200	0.6414	0.3371	0.4702
metab_10158	neg	578.2205	8.1936	1.4174	2.4536	0.0004	0.0050
metab_13420	neg	578.2399	2.9931	1.4295	5.3034	0.0200	0.0631
metab_4324	pos	578.3077	7.0816	0.7403	1.3522	0.1863	0.3014
metab_7417	neg	578.3113	5.9217	0.1529	-1.1748	0.7510	0.8216
metab_7295	neg	578.3488	8.9252	1.2598	-2.3009	0.0096	0.0388
metab_10436	neg	578.3818	9.1693	1.8579	-5.0948	0.0000	0.0001
metab_3020	pos	578.4170	9.1898	0.8778	-1.6612	0.1515	0.2582
metab_3098	pos	578.5128	9.7586	1.1929	0.7636	0.1938	0.3101
metab_831	pos	578.5131	9.3590	0.5880	-0.7926	0.2405	0.3653
metab_7978	neg	579.1785	0.7818	0.1292	0.3636	0.7873	0.8480
metab_10215	neg	579.2856	8.4022	0.2568	0.3486	0.6023	0.7069
metab_708	pos	579.2916	7.0526	0.6549	-0.7662	0.0404	0.0974
metab_7067	neg	579.3268	7.9908	1.2309	-1.7428	0.0044	0.0224
metab_11864	neg	579.3304	8.4022	0.0140	-0.9022	0.9534	0.9697
metab_5577	pos	579.3344	2.0677	1.5326	15.3467	0.0001	0.0021
metab_11475	neg	579.3617	9.3991	1.6098	-12.6106	0.0021	0.0136
metab_12210	neg	579.3902	7.3508	1.3812	13.0028	0.0022	0.0137
metab_3803	pos	579.4971	9.5137	1.3109	1.9198	0.0011	0.0081
metab_11293	neg	579.5004	10.0593	0.2384	0.6996	0.4457	0.5693
metab_7943	neg	580.1820	0.6411	0.2240	0.4251	0.6523	0.7475
metab_10254	neg	580.2592	8.5604	1.4425	-1.5680	0.0014	0.0104
metab_7419	neg	580.3265	5.8731	0.5042	-1.6930	0.3396	0.4728

metab_11860	neg	580.3624	8.4178	1.9143	4.5894	0.0388	0.0987
metab_9967	neg	580.3706	7.4618	1.8724	-14.0406	0.0000	0.0005
metab_3075	pos	580.4328	9.6199	1.0374	-1.7062	0.0210	0.0603
metab_3656	pos	580.5288	10.1331	0.9243	-1.5660	0.0535	0.1201
metab_8065	neg	581.0384	1.0357	0.4614	-0.3653	0.1410	0.2498
metab_8345	neg	581.1600	1.5439	1.4386	-2.8451	0.0054	0.0258
metab_4245	pos	581.3066	7.5307	0.3844	0.4469	0.3483	0.4832
metab_9892	neg	581.3108	7.1111	0.7433	1.1063	0.0299	0.0825
metab_12149	neg	581.3221	7.5855	0.1531	0.1977	0.6228	0.7233
metab_6575	neg	581.3429	8.8286	0.6398	-0.3408	0.0066	0.0298
metab_2598	pos	581.4300	6.4794	1.4157	2.6221	0.0010	0.0076
metab_3668	pos	581.5135	10.0715	0.1022	-0.1862	0.8021	0.8688
metab_10645	neg	581.5164	10.3524	0.8449	1.4409	0.0140	0.0497
metab_7209	neg	581.5280	10.7359	0.4739	-0.2121	0.0126	0.0464
metab_14362	neg	582.1917	1.4427	1.0816	-1.8259	0.0106	0.0412
metab_8031	neg	582.2163	0.9368	0.1674	9.5962	0.6322	0.7305
metab_7210	neg	582.5126	10.5844	1.7364	-3.1212	0.0002	0.0033
metab_3182	pos	582.5445	10.3942	0.0712	-0.0859	0.8773	0.9216
metab_11081	neg	582.8815	14.0282	0.0334	0.0274	0.9368	0.9586
metab_2672	pos	583.3231	6.9935	1.0350	1.2582	0.0802	0.1614
metab_4322	pos	583.3233	7.1119	0.7902	0.8781	0.0420	0.1004
metab_11963	neg	583.3380	8.0998	0.9791	1.7330	0.0017	0.0119
metab_9677	neg	583.3455	6.0670	0.1330	-0.5880	0.8535	0.8984
metab_7283	neg	583.3588	9.1858	1.8303	-3.2373	0.0000	0.0001
metab_11911	neg	583.4590	8.2409	0.8804	2.3634	0.1086	0.2064
metab_1573	pos	584.2039	1.4289	1.1376	-2.2421	0.0070	0.0279
metab_13207	neg	584.2720	3.5813	2.1611	6.3621	0.0018	0.0123
metab_13371	neg	584.2724	3.1115	1.8469	13.6741	0.0007	0.0068
metab_7047	neg	584.3005	7.4465	1.0388	-1.4233	0.0907	0.1808
metab_11197	neg	584.5269	10.7190	1.7251	-5.9657	0.0039	0.0207
metab_12275	neg	585.3442	7.1266	0.3276	-0.0702	0.1312	0.2364
metab_10244	neg	585.4744	8.5135	0.6824	3.5932	0.0583	0.1314
metab_9416	neg	586.2876	4.6216	1.0299	1.4678	0.0020	0.0134
metab_12949	neg	586.2877	4.3716	1.0050	1.4138	0.0028	0.0163
metab_12439	neg	586.2969	6.5829	0.0791	-0.2307	0.9105	0.9411
metab_12072	neg	586.3160	7.7875	0.2920	0.0832	0.5749	0.6843
metab_9947	neg	586.3710	7.3659	0.1442	3.8798	0.9659	0.9763
metab_10462	neg	586.4489	9.3165	0.6578	3.8316	0.2203	0.3444
metab_3218	pos	586.4510	11.0985	0.1909	-0.1763	0.2895	0.4202
metab_3782	pos	586.5028	9.5899	0.2716	0.2937	0.5598	0.6764
metab_10633	neg	586.5067	10.2384	0.4418	-1.3545	0.3410	0.4741
metab_6702	neg	586.9062	0.5126	1.0338	-1.6746	0.0464	0.1117
metab_7813	neg	587.0046	0.5126	0.2275	0.4225	0.5187	0.6368
metab_14577	neg	587.1951	1.0785	0.5222	0.3283	0.2894	0.4208
metab_12789	neg	587.3130	4.9838	1.6217	-2.1699	0.0461	0.1113
metab_11916	neg	587.3366	8.2253	0.0622	-0.4496	0.9991	0.9993
metab_9762	neg	587.3713	6.5346	1.0225	2.3339	0.0394	0.0998
metab_11731	neg	587.4327	8.7031	1.4303	-1.9279	0.0661	0.1439

metab_8701	neg	588.2174	2.2595	0.0444	0.4883	0.9839	0.9887
metab_7834	neg	588.2268	0.5286	1.2355	3.4504	0.0371	0.0956
metab_12260	neg	588.2954	7.1595	1.2728	2.4507	0.0046	0.0230
metab_4270	pos	588.3846	7.3673	0.7899	3.3264	0.4114	0.5442
metab_2812	pos	589.3713	7.9835	0.0112	0.5253	0.9250	0.9515
metab_2886	pos	589.4450	8.4074	1.1572	1.6271	0.0321	0.0826
metab_2818	pos	589.4451	8.0419	0.6038	2.7158	0.5106	0.6325
metab_7102	neg	589.4484	8.8286	0.4190	0.0749	0.2865	0.4180
metab_11283	neg	589.4802	10.0924	1.1772	-1.9155	0.0163	0.0549
metab_2998	pos	589.4813	9.0857	0.9439	0.8762	0.1545	0.2616
metab_3072	pos	589.4814	9.5899	0.7011	-0.9297	0.0261	0.0711
metab_3033	pos	589.4817	9.2968	1.1351	1.6806	0.0353	0.0881
metab_14553	neg	590.0334	1.1355	0.6846	-0.6712	0.0954	0.1877
metab_12247	neg	590.3108	7.2069	0.8230	1.4063	0.0268	0.0769
metab_2409	pos	590.3158	4.9698	0.2758	-0.1406	0.4635	0.5912
metab_2469	pos	590.3159	5.4056	0.2730	-0.2860	0.0926	0.1793
metab_3196	pos	590.3161	10.7001	0.5151	-0.3987	0.0002	0.0029
metab_3690	pos	590.3161	9.9307	0.6287	-0.5143	0.0088	0.0330
metab_3169	pos	590.3161	10.2404	0.7964	-0.9315	0.0084	0.0317
metab_2744	pos	590.4250	7.4415	0.2299	-0.3444	0.3907	0.5227
metab_3574	pos	590.4251	11.1297	0.3454	-0.2771	0.0369	0.0908
metab_6284	pos	591.0114	0.6962	0.2246	-0.0631	0.7431	0.8236
metab_12219	neg	591.1679	7.3180	0.3467	1.4421	0.5798	0.6884
metab_9953	neg	591.2052	7.4148	0.8268	-1.4848	0.1516	0.2633
metab_12049	neg	591.3392	7.8657	2.0300	-4.4189	0.0000	0.0006
metab_2884	pos	591.4608	8.4074	0.9911	1.2628	0.0093	0.0342
metab_11721	neg	591.4641	8.7191	0.7455	-0.4652	0.0432	0.1064
metab_3713	pos	591.4971	9.8673	0.7867	-1.0265	0.0103	0.0366
metab_12591	neg	592.1994	5.9375	0.2335	-1.0054	0.6951	0.7815
metab_14854	neg	592.2102	0.5851	0.8438	-1.5024	0.2464	0.3734
metab_12306	neg	592.2900	7.0322	1.3937	2.7253	0.0093	0.0379
metab_5505	pos	592.3164	2.1897	1.4811	13.2017	0.0141	0.0455
metab_12279	neg	592.3254	7.1111	2.3738	14.5746	0.0000	0.0004
metab_11917	neg	592.3626	8.2253	0.4176	1.1285	0.2059	0.3279
metab_4053	pos	592.3982	8.3912	0.0089	0.0162	0.9505	0.9681
metab_3823	pos	592.4327	9.3891	0.1495	1.4047	0.6138	0.7224
metab_9511	neg	593.1309	5.1296	0.4194	-0.1294	0.0494	0.1166
metab_8346	neg	593.1636	1.5439	0.1385	0.1333	0.8130	0.8671
metab_14643	neg	593.1944	0.9368	0.9922	-0.7311	0.0014	0.0103
metab_7015	neg	593.2747	6.6157	0.9928	-1.5396	0.0288	0.0806
metab_7010	neg	593.3062	6.5033	0.0944	0.1636	0.9286	0.9539
metab_12633	neg	593.3063	5.7420	0.3986	-1.0394	0.3779	0.5079
metab_9809	neg	593.3063	6.8093	0.1415	-0.9103	0.7255	0.8025
metab_2454	pos	593.3338	5.3165	0.5730	0.9025	0.3297	0.4640
metab_2422	pos	593.3339	5.0610	0.0704	1.1357	0.9682	0.9808
metab_777	pos	593.4763	8.4214	0.7090	1.2098	0.1084	0.2012
metab_11634	neg	593.4798	8.9574	0.2423	0.7977	0.4054	0.5334
metab_2042	pos	593.8135	2.9954	1.2467	2.1773	0.0413	0.0991

metab_14568	neg	594.1688	1.1069	0.2613	0.7017	0.8043	0.8603
metab_4313	pos	594.3022	7.1560	1.6919	2.7855	0.0085	0.0320
metab_9976	neg	594.3050	7.5087	1.5880	3.4329	0.0062	0.0284
metab_12703	neg	594.3061	5.4522	0.5341	-1.9823	0.4213	0.5473
metab_9997	neg	594.3424	7.5855	0.4960	0.0191	0.3870	0.5162
metab_12192	neg	594.3431	7.4465	0.9810	1.3480	0.0170	0.0565
metab_3728	pos	594.4486	9.8049	0.5322	1.0575	0.2936	0.4241
metab_10614	neg	594.4680	10.1082	1.5299	-1.9865	0.0011	0.0093
metab_3087	pos	594.5081	9.6968	0.7873	-0.6868	0.0294	0.0777
metab_7214	neg	594.5114	10.4679	0.6294	-0.5607	0.2346	0.3601
metab_10764	neg	594.8377	14.0282	0.2806	0.3459	0.4180	0.5443
metab_9844	neg	594.9017	6.9354	0.6571	0.3206	0.2952	0.4256
metab_9890	neg	594.9028	7.0796	0.5369	-0.8617	0.2258	0.3503
metab_8098	neg	595.1996	1.1355	0.2996	0.1589	0.3710	0.5023
metab_4952	pos	595.2357	3.9270	2.0022	-3.4881	0.0006	0.0053
metab_4625	pos	595.2870	5.5112	1.4226	-1.9226	0.0542	0.1212
metab_9840	neg	595.2897	6.9354	0.3029	-0.1665	0.3930	0.5214
metab_10842	neg	595.2899	14.1581	0.3280	0.3174	0.5699	0.6803
metab_12675	neg	595.2899	5.5966	2.1671	4.7455	0.0035	0.0189
metab_12597	neg	595.3012	5.9217	0.0035	-1.5387	0.9794	0.9857
metab_6579	neg	595.3221	6.1644	0.3985	-0.4675	0.1725	0.2890
metab_3042	pos	595.4324	9.3590	0.2250	1.3897	0.6461	0.7486
metab_23	pos	595.4910	8.6861	0.8144	1.2696	0.0559	0.1239
metab_11469	neg	595.4952	9.4485	1.1016	1.8655	0.0005	0.0057
metab_12292	neg	595.6776	7.0629	0.7682	-0.8190	0.0655	0.1430
metab_9838	neg	595.6777	6.9191	0.4936	0.1747	0.4253	0.5510
metab_10056	neg	596.1941	7.8026	1.1035	-0.5131	0.0251	0.0735
metab_14038	neg	596.2933	1.8046	1.7547	-13.0633	0.0000	0.0000
metab_9519	neg	596.3214	5.1610	0.1623	-2.0390	0.8027	0.8589
metab_11534	neg	596.3945	9.1858	0.6026	-0.5953	0.1171	0.2172
metab_7222	neg	596.4902	10.1910	0.0722	0.7067	0.9620	0.9740
metab_10439	neg	596.4989	9.1858	0.6544	1.3238	0.0611	0.1362
metab_3698	pos	596.5233	9.9154	0.1541	0.1654	0.7517	0.8300
metab_7851	neg	596.5484	0.5286	1.0530	10.3053	0.0440	0.1076
metab_14735	neg	597.0485	0.7537	1.0740	-2.8977	0.0212	0.0656
metab_697	pos	597.3023	6.9336	0.3482	-0.5007	0.3650	0.4986
metab_7389	neg	597.3044	6.9834	0.6263	1.4281	0.0833	0.1699
metab_7048	neg	597.3055	7.4465	0.5065	0.9732	0.2290	0.3540
metab_12610	neg	597.3170	5.8731	0.5918	-2.6219	0.3527	0.4859
metab_9733	neg	597.3367	6.3917	1.4208	-2.6749	0.0024	0.0150
metab_10074	neg	597.3377	7.8984	0.9876	-1.3830	0.0102	0.0402
metab_829	pos	597.4487	9.3429	0.3595	0.3462	0.4814	0.6070
metab_3944	pos	597.4864	8.8318	1.1985	-3.3925	0.0233	0.0650
metab_10649	neg	597.4865	10.3687	1.4417	-2.2537	0.0082	0.0345
metab_3830	pos	597.4875	9.3590	0.5440	-1.3334	0.1622	0.2716
metab_99	pos	597.5072	8.9212	0.8884	1.2746	0.0183	0.0544
metab_12008	neg	598.2101	7.9908	0.6389	-0.0411	0.1665	0.2823
metab_14767	neg	598.2109	0.6411	0.9823	3.5544	0.1152	0.2146

metab_9503	neg	598.3373	5.0967	0.2615	0.4681	0.5327	0.6487
metab_4794	pos	598.3504	4.6096	1.6997	-14.4040	0.0047	0.0209
metab_12888	neg	598.3546	4.6216	2.3831	-4.2161	0.0002	0.0027
metab_9469	neg	598.3546	4.8707	1.4962	-2.9321	0.0019	0.0128
metab_4134	pos	598.3783	8.0843	0.7436	-0.8701	0.1251	0.2238
metab_10673	neg	598.5180	10.7359	0.0049	0.2039	0.9846	0.9891
metab_3696	pos	598.5389	9.9154	0.1648	0.4904	0.8025	0.8690
metab_12679	neg	599.2654	5.5808	1.9076	-1.9545	0.0024	0.0150
metab_2698	pos	599.3182	7.0961	0.2530	0.1476	0.5230	0.6444
metab_7080	neg	599.3533	8.1936	1.8194	-3.6015	0.0000	0.0010
metab_10615	neg	599.4653	10.1082	1.1897	-1.6872	0.0158	0.0540
metab_839	pos	599.5019	9.6044	1.4034	-2.5633	0.0000	0.0011
metab_3638	pos	599.5021	10.2404	0.8713	-1.2068	0.0009	0.0069
metab_9553	neg	600.2970	5.3713	2.1740	-5.0945	0.0210	0.0652
metab_4342	pos	600.3043	7.0228	0.9152	-1.0690	0.0138	0.0447
metab_2102	pos	600.3091	3.2420	0.7677	0.6146	0.0560	0.1240
metab_13412	neg	600.3134	3.0101	0.4045	-1.1354	0.4858	0.6077
metab_12282	neg	600.3145	7.0959	0.4918	0.5504	0.3289	0.4615
metab_12188	neg	600.3241	7.4618	0.2545	1.4273	0.6091	0.7127
metab_12616	neg	600.3530	5.8239	0.5675	0.8824	0.3255	0.4579
metab_4802	pos	600.3665	4.6096	2.0925	-3.8610	0.0007	0.0062
metab_7149	neg	600.5219	10.4016	1.4309	-2.5730	0.0053	0.0255
metab_3600	pos	600.5551	10.6685	0.8462	-1.2183	0.0526	0.1184
metab_7196	neg	600.8545	14.0282	0.4042	-0.1202	0.0625	0.1385
metab_7954	neg	601.1386	0.6691	0.4221	-0.9425	0.4728	0.5951
metab_9311	neg	601.2923	4.1364	0.7572	-0.7777	0.0423	0.1050
metab_9268	neg	601.2924	3.9677	0.6774	-0.1239	0.0783	0.1627
metab_13462	neg	601.3212	2.8922	1.7475	4.9469	0.0007	0.0066
metab_7117	neg	601.3754	9.3165	0.1125	0.0308	0.6822	0.7716
metab_10391	neg	601.3760	9.0391	0.3179	0.3043	0.5776	0.6866
metab_11574	neg	601.4478	9.1038	1.9066	4.6931	0.0001	0.0015
metab_11758	neg	601.4481	8.6874	1.5607	3.5212	0.0006	0.0060
metab_887	pos	601.5178	10.5313	0.1231	-0.1974	0.6758	0.7719
metab_14995	neg	602.1405	0.5126	1.3709	-1.3341	0.0136	0.0487
metab_4647	pos	602.3093	5.3757	1.7655	-6.7708	0.0335	0.0849
metab_9620	neg	602.3129	5.7420	1.8877	-2.4460	0.0105	0.0409
metab_9572	neg	602.3132	5.5002	1.5071	-3.3429	0.0275	0.0783
metab_502	pos	602.3254	2.9954	0.2058	-1.4444	0.8238	0.8841
metab_11819	neg	602.3491	8.5455	1.9914	-5.7907	0.0013	0.0102
metab_10339	neg	602.3493	8.7965	1.6862	-6.3583	0.0135	0.0484
metab_11659	neg	602.3822	8.8608	2.1157	-3.7765	0.0028	0.0163
metab_7221	neg	602.5010	10.1743	0.4466	0.0557	0.4164	0.5424
metab_3751	pos	602.5193	9.7283	1.5494	-2.2237	0.0012	0.0085
metab_11076	neg	602.8502	14.0282	0.4067	-0.2647	0.1286	0.2331
metab_6748	neg	603.1554	0.6131	0.1516	1.6374	0.7127	0.7942
metab_9187	neg	603.2510	3.6980	1.4772	3.9053	0.0019	0.0128
metab_12018	neg	603.2851	7.9757	0.7615	-0.6201	0.0963	0.1889
metab_13081	neg	603.3080	3.9349	1.2969	-1.7324	0.0003	0.0040

metab_12108	neg	603.3168	7.6951	1.7532	-3.7912	0.0022	0.0138
metab_11994	neg	603.3311	8.0228	0.3731	-0.9887	0.7153	0.7961
metab_12402	neg	603.3314	6.7439	0.2457	-0.1781	0.8184	0.8710
metab_5238	pos	603.3335	2.8877	1.2561	5.6053	0.0205	0.0594
metab_7264	neg	603.3915	9.4485	0.6271	-0.5766	0.0196	0.0619
metab_11422	neg	603.4405	9.5979	0.6966	1.3771	0.1450	0.2552
metab_10573	neg	603.5006	9.8782	0.0207	0.3225	0.9751	0.9823
metab_3210	pos	603.5333	10.9614	0.8115	-0.9717	0.0083	0.0316
metab_1284	pos	604.0427	0.5703	1.0004	-1.4928	0.0069	0.0277
metab_8039	neg	604.0710	0.9649	0.6921	-0.1327	0.3102	0.4411
metab_10877	neg	604.0711	14.3693	0.1984	0.0365	0.6611	0.7554
metab_14914	neg	604.0713	0.5431	0.3173	1.2625	0.6395	0.7370
metab_9926	neg	604.1991	7.2537	0.7849	0.9460	0.0236	0.0705
metab_9287	neg	604.2752	4.0516	1.5540	-0.2422	0.0358	0.0930
metab_5251	pos	604.3040	2.8411	0.2858	0.2019	0.5731	0.6882
metab_724	pos	604.3833	7.1267	0.3608	-0.2837	0.0315	0.0814
metab_4295	pos	604.4558	7.2314	1.5401	16.1446	0.0028	0.0149
metab_11280	neg	604.4956	10.1082	2.0695	-3.7696	0.0001	0.0022
metab_9182	neg	605.2070	3.6812	1.8683	7.6347	0.0003	0.0038
metab_7814	neg	605.2168	0.5126	0.8607	2.2747	0.1224	0.2246
metab_12212	neg	605.2458	7.3508	0.0821	0.1291	0.8650	0.9075
metab_10225	neg	605.3009	8.4667	0.3935	0.4964	0.4893	0.6109
metab_4076	pos	605.3666	8.3018	0.2838	1.5996	0.7413	0.8224
metab_11947	neg	605.3710	8.1307	0.0614	0.5665	0.9798	0.9859
metab_12055	neg	605.3823	7.8352	0.0666	0.3119	0.9161	0.9443
metab_7255	neg	605.4067	9.6316	1.5587	-2.7216	0.0000	0.0006
metab_10116	neg	605.4430	8.0373	0.6270	1.7946	0.1844	0.3029
metab_11736	neg	605.4432	8.7031	0.7747	1.0472	0.0694	0.1491
metab_11302	neg	605.4760	10.0090	0.1156	1.6894	0.9600	0.9729
metab_6814	neg	606.0754	0.9649	1.3944	2.2555	0.0001	0.0020
metab_7656	neg	606.0755	0.5286	1.6564	3.0581	0.0001	0.0016
metab_7032	neg	606.3419	7.0629	0.2408	0.4163	0.5932	0.7001
metab_7053	neg	606.3424	7.5855	0.1224	0.2968	0.6535	0.7484
metab_9925	neg	606.3807	7.2377	0.8271	-1.9816	0.1297	0.2346
metab_3783	pos	606.4483	9.5899	0.9806	-1.1926	0.1101	0.2035
metab_7148	neg	606.4886	10.3194	0.4657	-0.5313	0.2504	0.3779
metab_3750	pos	606.5084	9.7283	0.0420	1.1978	0.9837	0.9901
metab_10636	neg	606.5122	10.2707	1.6335	-3.1756	0.0006	0.0062
metab_12235	neg	607.1986	7.2377	0.4911	-0.1483	0.2247	0.3492
metab_13989	neg	607.2779	1.8682	0.6119	11.1360	0.1298	0.2346
metab_641	pos	607.3493	5.3913	0.0598	0.0500	0.9483	0.9670
metab_629	pos	607.3494	5.1076	0.6460	0.6167	0.0456	0.1066
metab_11804	neg	607.3856	8.5937	0.1718	0.4125	0.8698	0.9105
metab_7248	neg	607.4227	9.7965	2.2106	-5.1504	0.0000	0.0009
metab_11881	neg	607.4591	8.3373	0.1638	0.5259	0.6729	0.7636
metab_803	pos	607.4920	8.8318	1.0909	2.5272	0.0832	0.1655
metab_3710	pos	607.4920	9.8673	1.1378	-1.9360	0.0100	0.0360
metab_6153	pos	608.0875	0.9761	1.5937	2.7213	0.0007	0.0058

metab_5715	pos	608.0876	1.7705	1.5828	2.5660	0.0012	0.0086
metab_6840	neg	608.2052	0.5991	0.5375	-1.0209	0.4182	0.5443
metab_12000	neg	608.3205	8.0069	0.0856	-0.0658	0.7540	0.8231
metab_9902	neg	608.3223	7.1437	1.5493	2.5697	0.0027	0.0159
metab_12146	neg	608.3580	7.5855	0.6449	1.1565	0.0921	0.1826
metab_11831	neg	608.3765	8.5135	1.4353	3.0766	0.0007	0.0069
metab_2920	pos	608.4143	8.6132	0.6311	1.1737	0.3505	0.4855
metab_899	pos	608.5236	9.9307	0.2035	0.3131	0.6014	0.7112
metab_14793	neg	609.1893	0.6131	0.8790	-0.6542	0.0021	0.0136
metab_10033	neg	609.2141	7.7100	1.2208	-1.0381	0.0111	0.0422
metab_9580	neg	609.2690	5.5639	1.0943	-1.7762	0.0183	0.0594
metab_721	pos	609.3374	7.1267	0.6696	-0.8069	0.0070	0.0279
metab_3552	pos	609.3376	12.1687	0.1850	-0.2280	0.2879	0.4184
metab_3244	pos	609.3378	12.5834	0.0989	0.0157	0.6118	0.7209
metab_3247	pos	609.3379	12.7765	0.0312	-0.0390	0.7318	0.8148
metab_3532	pos	609.3381	13.8445	0.0941	-0.0365	0.4486	0.5782
metab_3887	pos	609.3385	9.0999	0.7419	-0.6063	0.0281	0.0752
metab_2802	pos	609.4710	7.9118	1.2500	1.8595	0.0045	0.0205
metab_11835	neg	609.4745	8.5135	0.5609	1.2921	0.1668	0.2827
metab_3114	pos	609.5077	9.8362	0.5343	-0.5913	0.1379	0.2407
metab_101	pos	609.5078	9.0556	0.2030	0.6245	0.5946	0.7054
metab_10687	neg	609.5585	11.1796	0.3929	-0.0419	0.0124	0.0459
metab_3635	pos	610.1827	10.2712	0.0242	0.1396	0.9865	0.9917
metab_11071	neg	610.2381	14.0282	0.6182	1.4927	0.1919	0.3117
metab_12076	neg	610.3154	7.7728	1.3460	2.8166	0.0130	0.0470
metab_11959	neg	610.3370	8.0998	0.9711	1.5964	0.0027	0.0160
metab_11584	neg	610.3918	9.0872	1.1069	-0.9610	0.0077	0.0330
metab_6638	neg	610.5057	10.3524	0.9145	1.9648	0.0107	0.0414
metab_3159	pos	610.5387	10.1647	2.1498	2.0336	0.0346	0.0871
metab_238	pos	611.0606	0.7801	1.3293	-1.6510	0.0172	0.0522
metab_6771	neg	611.0640	0.7254	1.0744	-1.1993	0.0430	0.1062
metab_6781	neg	611.1454	0.7818	0.2642	0.7292	0.3745	0.5054
metab_286	pos	611.1707	1.1875	0.7949	-1.7306	0.2800	0.4093
metab_4619	pos	611.2816	5.5565	1.2058	-2.6242	0.0020	0.0121
metab_12759	neg	611.2847	5.1456	2.0986	-2.1938	0.0281	0.0794
metab_12692	neg	611.2847	5.5164	1.0221	-1.2928	0.0401	0.1010
metab_9107	neg	611.2906	3.4133	1.7914	4.6442	0.0222	0.0676
metab_12748	neg	611.3170	5.1939	0.1500	-0.4550	0.6366	0.7346
metab_12103	neg	611.3289	7.7100	1.0061	1.4347	0.0294	0.0816
metab_4209	pos	611.3808	7.7062	0.6189	-0.8917	0.3205	0.4535
metab_7252	neg	611.4459	9.7470	0.7859	-0.3120	0.0291	0.0812
metab_11753	neg	611.4488	8.6874	0.9082	2.8475	0.0896	0.1795
metab_2830	pos	611.4868	8.1273	1.5924	2.9067	0.0014	0.0094
metab_10290	neg	611.4899	8.6874	0.7950	1.4667	0.0652	0.1427
metab_102	pos	611.5236	9.2504	0.4217	0.8716	0.2755	0.4040
metab_9965	neg	612.3063	7.4618	1.0447	-1.0139	0.0184	0.0594
metab_7376	neg	612.3081	7.3017	0.7778	-0.5386	0.0507	0.1186
metab_6256	pos	613.1577	0.7801	0.6453	1.0630	0.0986	0.1875

metab_14366	neg	613.2484	1.4267	1.4947	-13.3726	0.0124	0.0459
metab_4617	pos	613.2972	5.5715	0.0792	1.0547	0.9623	0.9772
metab_12684	neg	613.2998	5.5639	0.6468	-0.4417	0.3063	0.4374
metab_9916	neg	613.3325	7.2069	0.2566	-0.7742	0.5682	0.6792
metab_804	pos	613.4430	8.8318	0.3055	-0.0603	0.4503	0.5800
metab_7238	neg	613.4618	9.9108	0.0691	0.6280	0.9336	0.9568
metab_800	pos	613.4815	8.7303	1.3033	-2.6231	0.0289	0.0766
metab_7106	neg	613.5058	8.9903	1.2562	2.1619	0.0082	0.0345
metab_6942	neg	614.2781	4.0859	0.3562	0.1924	0.5792	0.6881
metab_12742	neg	614.2876	5.2257	2.3477	-10.8011	0.0009	0.0076
metab_9493	neg	614.3125	4.9838	1.6975	-2.3998	0.0046	0.0230
metab_2729	pos	614.3187	7.3071	0.5255	-0.3105	0.2366	0.3604
metab_4372	pos	614.3289	6.9182	0.3392	-0.3694	0.5902	0.7021
metab_9258	neg	614.3317	3.9349	0.8411	-2.8904	0.1707	0.2871
metab_12019	neg	614.3320	7.9757	1.9600	-4.1774	0.0031	0.0176
metab_2227	pos	614.3460	3.8063	2.0331	-4.4152	0.0005	0.0047
metab_7435	neg	614.3495	3.6311	1.9520	-4.2991	0.0000	0.0010
metab_8747	neg	614.3892	2.3704	1.8441	14.0291	0.0000	0.0006
metab_3200	pos	614.5128	10.7305	0.2219	0.5977	0.7094	0.7979
metab_870	pos	614.5338	9.9470	0.7555	0.6782	0.0619	0.1332
metab_3817	pos	614.5343	9.4357	0.8980	1.1356	0.0131	0.0432
metab_11072	neg	614.8054	14.0282	0.1274	-0.0190	0.4957	0.6169
metab_815	pos	615.4584	9.0857	0.3976	-0.2292	0.2358	0.3597
metab_10480	neg	615.4641	9.4322	0.2820	0.2936	0.6537	0.7486
metab_10609	neg	615.4781	10.0593	0.5432	1.0558	0.1380	0.2460
metab_909	pos	615.4971	9.6358	1.4361	-2.8883	0.0023	0.0132
metab_2985	pos	615.5160	8.9958	1.7022	3.0201	0.0031	0.0159
metab_3016	pos	615.5377	9.1747	0.6260	1.2820	0.1870	0.3024
metab_8676	neg	616.2337	2.2119	0.7430	1.4249	0.2469	0.3740
metab_9501	neg	616.2919	5.0812	0.7506	1.9672	0.1871	0.3059
metab_6954	neg	616.2920	4.3716	0.8939	-0.6261	0.0784	0.1628
metab_4168	pos	616.3444	7.9695	2.1922	-5.0656	0.0005	0.0047
metab_5023	pos	616.3616	3.6384	1.9422	-4.2153	0.0001	0.0016
metab_448	pos	616.4017	2.3600	1.5114	14.3210	0.0003	0.0034
metab_3043	pos	616.5501	9.3736	1.0855	0.6551	0.1180	0.2141
metab_14979	neg	617.0603	0.5286	1.8273	6.1462	0.0000	0.0005
metab_12042	neg	617.3701	7.8984	0.4193	-0.2456	0.3747	0.5054
metab_11944	neg	617.3703	8.1468	0.1422	0.1036	0.6760	0.7663
metab_11739	neg	617.3703	8.7031	0.0489	0.3492	0.8169	0.8700
metab_10212	neg	617.3704	8.4022	0.3772	-0.2432	0.3256	0.4579
metab_3035	pos	617.4740	9.3117	0.2023	0.4839	0.5094	0.6315
metab_11405	neg	617.4794	9.6477	0.4086	0.1247	0.3457	0.4788
metab_863	pos	617.5116	9.8522	1.1936	-1.9903	0.0127	0.0423
metab_9931	neg	618.2153	7.2865	1.0298	1.5282	0.0164	0.0551
metab_7915	neg	618.2255	0.5991	1.0394	-2.7445	0.1152	0.2146
metab_14351	neg	618.2623	1.4427	1.7500	-4.4732	0.0004	0.0049
metab_4857	pos	618.3043	4.3672	0.6587	-0.3098	0.3707	0.5045
metab_6976	neg	618.3077	5.0002	0.3757	1.5981	0.3623	0.4949

metab_9368	neg	618.3082	4.4055	0.9281	0.4017	0.3343	0.4672
metab_2471	pos	618.3465	5.4056	0.0146	0.0958	0.9815	0.9887
metab_4233	pos	618.3698	7.6042	0.4122	-0.3765	0.3702	0.5041
metab_4163	pos	618.3708	7.9835	0.2865	-0.6834	0.3156	0.4481
metab_7223	neg	618.5116	10.1743	0.5984	1.1709	0.2013	0.3223
metab_8147	neg	619.2730	1.2521	1.1727	-3.7437	0.0678	0.1464
metab_11979	neg	619.3199	8.0688	1.6274	-5.5610	0.1488	0.2598
metab_2556	pos	619.3308	6.0410	0.7002	1.1194	0.1741	0.2867
metab_10341	neg	619.3861	8.8128	0.1386	0.0035	0.6543	0.7490
metab_7287	neg	619.3861	9.1522	0.0192	0.3999	0.9320	0.9560
metab_10315	neg	619.4222	8.7191	0.1452	-0.2607	0.6973	0.7829
metab_7235	neg	619.4565	9.9606	0.6995	1.2136	0.0276	0.0787
metab_3769	pos	619.4906	9.6521	0.5982	0.8569	0.1521	0.2588
metab_11266	neg	619.4918	10.1743	0.2437	1.0835	0.7410	0.8135
metab_3593	pos	619.5187	10.7459	0.4114	0.1702	0.0597	0.1296
metab_7045	neg	620.1940	7.3824	0.4801	0.9239	0.2108	0.3335
metab_7970	neg	620.2416	0.7678	1.2027	-2.0816	0.1536	0.2661
metab_13014	neg	620.2692	4.1699	1.7484	-2.9236	0.0001	0.0017
metab_9853	neg	620.3017	6.9834	0.3638	0.9411	0.4964	0.6173
metab_9975	neg	620.3203	7.5087	0.5135	-0.8261	0.3555	0.4884
metab_10025	neg	620.3577	7.6951	1.2407	2.6465	0.0184	0.0594
metab_11467	neg	620.3818	9.4485	0.3877	-0.3790	0.1655	0.2812
metab_4194	pos	620.3864	7.7946	1.3231	-2.2177	0.0007	0.0059
metab_2879	pos	620.3869	8.3763	1.0659	-1.6154	0.0055	0.0235
metab_11794	neg	620.3900	8.6093	1.5579	-2.6435	0.0002	0.0028
metab_2665	pos	620.4350	6.9638	0.3815	0.2367	0.1148	0.2098
metab_3759	pos	620.5229	9.6968	0.8995	-0.6119	0.0680	0.1431
metab_11221	neg	620.5271	10.4510	1.6987	3.9887	0.0021	0.0134
metab_1585	pos	621.2432	1.4569	1.5968	14.7961	0.0056	0.0238
metab_4357	pos	621.2985	6.9638	0.2005	0.5316	0.6918	0.7843
metab_11281	neg	621.4860	10.1082	2.0743	-12.9390	0.0000	0.0000
metab_12369	neg	622.1699	6.8565	0.1916	-0.6291	0.7789	0.8423
metab_12036	neg	622.2106	7.9137	1.7456	3.0316	0.0108	0.0417
metab_7911	neg	622.2206	0.5991	1.1003	-1.6639	0.0538	0.1238
metab_9146	neg	622.2564	3.5641	1.5569	14.2057	0.0078	0.0334
metab_701	pos	622.3143	6.9790	0.1640	0.5576	0.7490	0.8287
metab_12637	neg	622.3232	5.7259	1.5105	-2.4938	0.0141	0.0498
metab_12154	neg	622.3364	7.5702	0.6693	-0.4743	0.1583	0.2721
metab_10210	neg	622.3732	8.3858	1.1788	2.4321	0.0435	0.1071
metab_4220	pos	622.3786	7.6776	0.7135	-0.8852	0.0487	0.1119
metab_4124	pos	622.4013	8.1273	1.0479	-1.4675	0.0013	0.0089
metab_3986	pos	622.4021	8.6284	1.5836	-2.9875	0.0000	0.0008
metab_11536	neg	622.4096	9.1858	1.3048	-1.8472	0.0029	0.0170
metab_3761	pos	622.5392	9.6968	1.0904	-1.1639	0.0036	0.0174
metab_6734	neg	623.1119	0.5711	0.4311	0.8287	0.3417	0.4746
metab_14721	neg	623.2047	0.7818	1.1447	-1.0601	0.0000	0.0005
metab_12086	neg	623.4535	7.7404	0.8769	1.3532	0.0584	0.1317
metab_11905	neg	623.4538	8.2744	1.5264	2.5168	0.0078	0.0332

metab_2978	pos	623.5232	8.9507	1.2968	2.3746	0.0098	0.0355
metab_7938	neg	624.2079	0.6271	1.3450	-1.4694	0.0010	0.0083
metab_12606	neg	624.3165	5.8731	0.7151	-3.0518	0.3741	0.5050
metab_4111	pos	624.3929	8.1857	0.0476	0.2862	0.9979	0.9987
metab_4123	pos	624.4098	8.1273	0.2105	0.6020	0.6825	0.7770
metab_10518	neg	624.4258	9.6147	0.5666	-0.5793	0.3689	0.5003
metab_7269	neg	624.4778	9.3991	0.9074	2.5371	0.1002	0.1944
metab_3834	pos	624.5187	9.3429	0.7865	1.3769	0.1316	0.2322
metab_7225	neg	624.5218	10.1401	0.7104	-0.8842	0.0646	0.1418
metab_3129	pos	624.5545	9.9154	0.7030	-0.3972	0.0996	0.1888
metab_3595	pos	624.6389	10.7305	0.5486	-0.4528	0.0009	0.0070
metab_14652	neg	625.1855	0.9086	0.6069	-1.7575	0.4889	0.6105
metab_9943	neg	625.3271	7.3508	0.6774	-1.6105	0.1688	0.2851
metab_12237	neg	625.3273	7.2225	0.4628	0.9191	0.3094	0.4404
metab_11974	neg	625.3455	8.0843	0.4618	-0.2034	0.1920	0.3117
metab_681	pos	625.3932	6.5857	0.4438	1.1375	0.5351	0.6557
metab_10061	neg	625.4691	7.8352	1.0413	1.5050	0.0013	0.0103
metab_4126	pos	626.4251	8.1132	0.3406	1.0692	0.7891	0.8582
metab_4039	pos	626.4978	8.4356	0.4655	0.8694	0.3289	0.4630
metab_3802	pos	626.5342	9.5137	1.7269	2.6731	0.0177	0.0532
metab_10657	neg	626.5377	10.4016	0.2027	0.3212	0.7181	0.7976
metab_11179	neg	626.5492	11.1796	0.2582	-0.0673	0.2809	0.4113
metab_12829	neg	627.2799	4.8541	1.1269	-2.1101	0.0179	0.0586
metab_10400	neg	627.3689	9.0713	0.0560	0.4870	0.7771	0.8409
metab_11580	neg	627.4410	9.1038	0.7972	-0.3796	0.0174	0.0574
metab_11701	neg	627.4415	8.7346	0.6700	-0.4428	0.0938	0.1850
metab_817	pos	627.4582	9.1140	1.4161	1.8063	0.0428	0.1015
metab_10143	neg	627.4850	8.1307	1.5891	2.8016	0.0002	0.0030
metab_11236	neg	627.4964	10.3524	1.1821	2.6330	0.0015	0.0108
metab_2792	pos	627.8661	7.7946	0.5052	1.9540	0.5251	0.6461
metab_14973	neg	628.0529	0.5286	2.0905	5.1089	0.0001	0.0015
metab_2891	pos	628.5138	8.4214	1.0144	1.7952	0.0683	0.1435
metab_853	pos	628.5498	9.7283	2.4506	9.7946	0.0008	0.0063
metab_11192	neg	628.5530	10.7689	1.9234	-5.3204	0.0000	0.0001
metab_14955	neg	629.0503	0.5286	1.6591	5.6251	0.0002	0.0035
metab_9497	neg	629.2956	5.0159	1.6768	-3.2616	0.0001	0.0024
metab_12840	neg	629.2956	4.8049	1.1458	-1.6061	0.0283	0.0800
metab_10474	neg	629.4371	9.3824	1.5562	-2.8156	0.0101	0.0400
metab_2899	pos	629.4378	8.4949	0.0280	0.0081	0.9873	0.9921
metab_11633	neg	629.4562	8.9574	0.3802	0.0067	0.3768	0.5074
metab_10457	neg	629.4570	9.3000	0.1267	0.7100	0.6210	0.7221
metab_3003	pos	629.4748	9.0999	0.4770	-0.4166	0.1092	0.2024
metab_4061	pos	629.4763	8.3609	0.5893	-2.8534	0.3356	0.4694
metab_2870	pos	629.4967	8.3322	0.7679	2.4435	0.1723	0.2845
metab_9830	neg	629.5006	6.8887	1.1520	2.1815	0.0687	0.1480
metab_1165	pos	630.0568	0.5140	1.6679	2.7308	0.0012	0.0085
metab_9289	neg	630.3076	4.0516	1.4527	-1.9337	0.0009	0.0078
metab_7075	neg	630.3787	8.1468	0.6567	0.7519	0.1544	0.2671

metab_11423	neg	630.4607	9.5979	0.3632	0.8329	0.2546	0.3830
metab_6653	neg	630.4877	9.3494	0.8637	-0.7388	0.0720	0.1526
metab_3975	pos	630.5289	8.6861	1.3779	2.6241	0.0088	0.0329
metab_9260	neg	631.3223	3.9349	1.4710	-3.7471	0.0138	0.0490
metab_3892	pos	631.4328	9.0857	0.2440	0.0921	0.6118	0.7209
metab_2926	pos	631.4534	8.6284	0.0865	0.2300	0.7772	0.8498
metab_2965	pos	631.4914	8.8904	0.8724	-1.2599	0.0413	0.0990
metab_6749	neg	632.2051	0.6411	1.1209	-1.4112	0.0031	0.0175
metab_6777	neg	632.2053	0.7678	0.9454	-1.0905	0.0405	0.1017
metab_14023	neg	632.2781	1.8205	0.9951	-1.3244	0.0519	0.1206
metab_9392	neg	632.2870	4.5391	0.2377	0.8799	0.7180	0.7976
metab_9959	neg	632.3180	7.4465	0.1354	0.0826	0.6531	0.7482
metab_3209	pos	632.3626	10.9161	0.2603	-0.2158	0.1430	0.2471
metab_4118	pos	632.3903	8.1414	1.1284	1.2277	0.0218	0.0621
metab_4319	pos	632.4145	7.1267	0.1946	0.3255	0.5611	0.6777
metab_3929	pos	632.5445	8.9060	1.1605	3.7093	0.0086	0.0323
metab_9932	neg	633.2130	7.2865	1.1398	3.9890	0.0438	0.1075
metab_10081	neg	633.3195	7.9137	0.1267	-0.1393	0.7872	0.8480
metab_11888	neg	633.3221	8.3055	0.0496	0.4947	0.9942	0.9952
metab_10127	neg	633.3651	8.0843	0.3753	0.0540	0.4583	0.5813
metab_12322	neg	633.3656	7.0002	0.0992	0.5249	0.9624	0.9741
metab_4022	pos	633.4688	8.5241	0.2370	0.5026	0.5595	0.6764
metab_11239	neg	633.5071	10.3194	0.0457	-0.0887	0.8303	0.8810
metab_943	pos	633.5075	9.5899	1.0115	-1.3832	0.0223	0.0631
metab_14635	neg	634.2573	0.9508	1.2789	-1.8982	0.1166	0.2167
metab_12980	neg	634.3026	4.2870	0.2155	0.9168	0.5902	0.6974
metab_12880	neg	634.3027	4.6550	0.0788	0.8102	0.8011	0.8577
metab_7345	neg	634.3352	8.0998	1.0949	2.1316	0.0231	0.0695
metab_673	pos	634.3659	6.3581	0.6326	0.8312	0.2477	0.3729
metab_12139	neg	634.3702	7.6009	0.6670	-1.0894	0.1466	0.2572
metab_12207	neg	634.3703	7.3659	0.7467	-1.3991	0.2043	0.3260
metab_6650	neg	634.5194	10.6686	1.1515	-2.0350	0.0192	0.0610
metab_3621	pos	634.5391	10.3783	1.4243	-2.6624	0.0007	0.0061
metab_9653	neg	635.1808	5.9375	0.4048	-2.0726	0.5190	0.6370
metab_4615	pos	635.2788	5.5715	1.2666	-2.2117	0.0345	0.0869
metab_7041	neg	635.3804	7.2377	1.0041	-1.2714	0.0097	0.0390
metab_11929	neg	635.3810	8.1936	0.1052	0.1162	0.7245	0.8022
metab_11264	neg	635.5013	10.1910	1.2120	2.2850	0.0150	0.0520
metab_857	pos	635.5227	9.7586	1.0334	-1.3256	0.0064	0.0261
metab_13766	neg	636.1451	2.2754	1.5084	3.7188	0.0073	0.0318
metab_9961	neg	636.2596	7.4465	0.6745	0.7774	0.0761	0.1589
metab_10000	neg	636.2601	7.6009	0.1425	-0.4531	0.7996	0.8565
metab_10202	neg	636.2602	8.3528	0.5960	1.1919	0.1735	0.2903
metab_10045	neg	636.2608	7.7567	0.4827	0.0192	0.3800	0.5098
metab_9868	neg	636.2806	7.0322	1.3494	2.9627	0.0312	0.0851
metab_9714	neg	636.2968	6.2943	1.2369	4.2762	0.0794	0.1645
metab_585	pos	636.3146	4.2301	0.9767	2.5137	0.1681	0.2789
metab_2660	pos	636.3815	6.9336	0.4154	0.3917	0.3244	0.4582

metab_2880	pos	636.3817	8.3763	0.1101	0.1691	0.9084	0.9413
metab_2801	pos	636.3818	7.8978	0.4292	-0.5875	0.2738	0.4019
metab_4268	pos	636.3822	7.3824	0.6275	-0.9537	0.1380	0.2407
metab_12111	neg	636.3847	7.6951	0.7563	-0.9610	0.0625	0.1386
metab_10477	neg	636.4268	9.3991	0.0125	1.3736	0.8209	0.8731
metab_10469	neg	636.4493	9.3494	0.1123	1.3244	0.9447	0.9638
metab_868	pos	636.5546	9.9470	0.0560	0.2444	0.8758	0.9214
metab_7993	neg	637.1510	0.8098	0.6050	2.0772	0.3788	0.5088
metab_9880	neg	637.2099	7.0469	0.8033	-0.5634	0.1274	0.2315
metab_12258	neg	637.2099	7.1749	1.5265	-2.0443	0.0065	0.0294
metab_9801	neg	637.3475	6.7603	1.4106	3.0356	0.0179	0.0585
metab_11656	neg	637.3545	8.8770	1.9786	-4.8309	0.0001	0.0027
metab_11915	neg	637.3603	8.2253	1.2850	4.7372	0.1095	0.2075
metab_10114	neg	637.3607	8.0228	0.2473	0.9683	0.8237	0.8754
metab_7337	neg	637.3967	8.3373	0.1189	0.0478	0.6924	0.7794
metab_7114	neg	637.4694	9.1038	0.0073	0.6640	0.9676	0.9772
metab_11714	neg	637.5062	8.7191	1.3449	2.5639	0.0190	0.0607
metab_11220	neg	637.5172	10.4510	1.3947	5.8952	0.0017	0.0117
metab_8347	neg	638.1950	1.5439	0.5219	1.4448	0.3264	0.4586
metab_10140	neg	638.2772	8.1153	1.2759	2.4794	0.0025	0.0152
metab_9261	neg	638.2801	3.9349	1.7784	-2.5287	0.0031	0.0176
metab_12393	neg	638.3125	6.7926	0.6714	2.0520	0.2714	0.4019
metab_9907	neg	638.3129	7.1595	0.5245	-0.4892	0.3759	0.5066
metab_2527	pos	638.3286	5.8294	1.1576	-1.5002	0.1902	0.3056
metab_12158	neg	638.3319	7.5541	1.2112	-1.6038	0.0184	0.0594
metab_12433	neg	638.3467	6.5989	1.6144	2.8471	0.0021	0.0135
metab_2574	pos	638.3731	6.2071	0.6863	3.1140	0.2664	0.3943
metab_2600	pos	638.3732	6.4948	1.5100	-1.3205	0.0467	0.1084
metab_736	pos	638.3974	7.6776	0.3578	-0.4742	0.2225	0.3450
metab_11381	neg	638.4650	9.7470	0.7598	-0.2636	0.0251	0.0736
metab_11488	neg	638.5017	9.3494	0.1642	0.7277	0.7809	0.8438
metab_2984	pos	638.5332	8.9958	0.1220	0.4285	0.6680	0.7655
metab_3828	pos	638.5335	9.3736	0.3827	-0.1772	0.2278	0.3513
metab_3594	pos	638.6544	10.7305	0.4985	-0.4535	0.0035	0.0173
metab_14786	neg	639.1997	0.6131	0.6517	-0.1379	0.1484	0.2594
metab_4833	pos	639.3411	4.4730	1.0200	2.8607	0.2020	0.3210
metab_138	pos	639.3572	5.4504	1.0886	3.0094	0.1105	0.2040
metab_7038	neg	639.3756	7.1903	0.4336	1.5011	0.5512	0.6632
metab_10107	neg	639.4047	8.0069	0.7481	-0.8123	0.0294	0.0816
metab_7329	neg	639.4114	8.5455	1.0701	-1.4398	0.0042	0.0214
metab_11502	neg	639.4854	9.3000	1.7488	3.2310	0.0062	0.0285
metab_3620	pos	639.4942	10.3783	1.8647	-14.0721	0.0000	0.0000
metab_11632	neg	639.5218	8.9574	1.7220	2.8950	0.0101	0.0400
metab_14304	neg	640.1642	1.4710	1.2419	13.5094	0.0332	0.0889
metab_13917	neg	640.2470	2.0055	1.3904	13.0296	0.0173	0.0572
metab_4406	pos	640.3246	6.7825	0.3044	1.1914	0.6184	0.7260
metab_9856	neg	640.3283	7.0002	0.3907	1.4794	0.5417	0.6557
metab_981	pos	640.3445	7.5450	1.2203	-1.8721	0.0257	0.0703

metab_11981	neg	640.3474	8.0688	0.6889	-0.4444	0.2063	0.3283
metab_2943	pos	640.4131	8.7161	1.0373	-1.5650	0.0009	0.0069
metab_2804	pos	640.4132	7.9407	0.9703	-1.3274	0.0010	0.0077
metab_6576	neg	640.4167	8.8286	0.7839	-0.5617	0.0012	0.0095
metab_10579	neg	640.4805	9.9108	0.0160	0.6612	0.9535	0.9697
metab_3837	pos	640.5499	9.3278	0.8367	1.0886	0.0149	0.0473
metab_10018	neg	641.3144	7.6793	1.2186	-1.5344	0.0963	0.1890
metab_12544	neg	641.3280	6.1482	0.3574	-0.2582	0.3319	0.4645
metab_13009	neg	641.3389	4.1869	0.9645	-1.3143	0.0245	0.0724
metab_9449	neg	641.3390	4.7709	0.7741	-0.7886	0.1502	0.2616
metab_14031	neg	641.3667	1.8205	1.3617	-3.1491	0.0115	0.0434
metab_11912	neg	641.4195	8.2409	0.7492	-0.9932	0.0475	0.1136
metab_12365	neg	641.4641	6.8730	1.2054	2.4429	0.0393	0.0996
metab_11274	neg	641.5122	10.1401	0.7254	-0.9421	0.0974	0.1906
metab_7286	neg	641.5375	9.1858	1.1145	1.9502	0.0079	0.0338
metab_8337	neg	642.1434	1.5297	1.6987	-3.7374	0.0000	0.0001
metab_14750	neg	642.1664	0.6831	0.4489	0.0636	0.5198	0.6378
metab_8733	neg	642.1799	2.3380	1.8937	6.7630	0.0000	0.0007
metab_6904	neg	642.2749	1.4568	2.0088	-4.2037	0.0002	0.0031
metab_4139	pos	642.3599	8.0700	0.7883	-0.7836	0.2042	0.3236
metab_10083	neg	642.3770	7.9137	0.3472	1.3492	0.6793	0.7692
metab_11743	neg	642.3966	8.7031	1.3390	-1.8982	0.0001	0.0017
metab_3951	pos	642.4286	8.8169	0.8523	-1.0121	0.0004	0.0042
metab_11850	neg	642.4310	8.4505	1.5805	-3.7458	0.0030	0.0172
metab_6655	neg	642.4323	9.1858	1.5978	-2.5637	0.0000	0.0002
metab_7280	neg	642.4881	9.2185	1.7137	3.1336	0.0105	0.0409
metab_11288	neg	642.4961	10.0749	0.1340	0.0013	0.6254	0.7257
metab_4041	pos	642.5288	8.4214	0.4604	1.3063	0.3780	0.5110
metab_3774	pos	642.5294	9.6358	1.2998	-2.1258	0.0049	0.0218
metab_7263	neg	642.5313	9.4812	0.6155	-1.5063	0.4491	0.5725
metab_7128	neg	642.5323	9.6316	1.2975	0.1008	0.0838	0.1706
metab_3805	pos	642.5649	9.4981	2.4431	2.7631	0.0241	0.0669
metab_11948	neg	643.3492	8.1307	0.6370	-0.6152	0.1505	0.2618
metab_2381	pos	643.3511	4.7758	0.4503	-0.6756	0.3153	0.4481
metab_11251	neg	643.4351	10.2384	0.3939	-0.2026	0.2520	0.3802
metab_10312	neg	643.4360	8.7191	0.9300	1.2693	0.2712	0.4018
metab_11397	neg	643.4569	9.6814	0.7435	-0.2480	0.0259	0.0754
metab_11222	neg	643.5277	10.4016	0.5489	0.8546	0.1941	0.3143
metab_5444	pos	644.1922	2.3292	1.2906	14.2169	0.0126	0.0421
metab_6736	neg	644.2181	0.5851	1.1995	1.9356	0.0303	0.0833
metab_6926	neg	644.3417	3.7317	1.3053	-1.1110	0.0730	0.1541
metab_7270	neg	644.4179	9.3661	0.1297	1.6308	0.8721	0.9121
metab_3864	pos	644.4444	9.2049	1.6506	-3.0107	0.0000	0.0001
metab_7267	neg	644.5049	9.4156	0.8220	2.6786	0.0732	0.1544
metab_3100	pos	644.5445	9.7738	0.9599	-1.0289	0.0728	0.1505
metab_14788	neg	644.6507	0.6131	1.8254	4.0008	0.0003	0.0039
metab_9186	neg	645.2905	3.6980	1.5116	-3.4090	0.0058	0.0271
metab_10087	neg	645.3471	7.9292	1.2606	-4.8153	0.0045	0.0227

metab_11824	neg	645.4520	8.5299	0.8426	1.5688	0.0621	0.1379
metab_3956	pos	645.4682	8.7590	1.7087	4.5448	0.0071	0.0280
metab_1398	pos	646.0432	0.8920	1.6568	2.7297	0.0010	0.0073
metab_2206	pos	646.3535	3.6987	0.7838	3.1014	0.2321	0.3560
metab_10580	neg	646.4758	9.9108	0.4346	0.2493	0.2990	0.4294
metab_11592	neg	646.4804	9.0713	0.4031	-0.9559	0.5840	0.6921
metab_825	pos	646.5602	9.2504	0.7570	1.0456	0.0441	0.1038
metab_7886	neg	646.9643	0.5571	1.0338	-12.7957	0.0026	0.0156
metab_9921	neg	647.3304	7.2225	0.1690	-0.2564	0.7706	0.8360
metab_9960	neg	647.3690	7.4465	1.3691	2.2373	0.0000	0.0002
metab_3581	pos	647.4571	11.0078	0.2953	0.1463	0.0762	0.1549
metab_11686	neg	647.4679	8.7640	1.2230	2.1020	0.0081	0.0342
metab_802	pos	647.4845	8.7731	0.8870	1.5271	0.1362	0.2386
metab_4172	pos	647.4868	7.9407	0.7381	-1.4185	0.2081	0.3279
metab_7000	neg	648.3142	5.8731	1.0001	-4.2221	0.1800	0.2978
metab_9504	neg	648.3795	5.0967	0.8126	1.1395	0.0176	0.0579
metab_7284	neg	648.4986	9.1693	0.6917	0.8176	0.1480	0.2589
metab_14853	neg	649.2320	0.5851	0.9266	-1.0869	0.1202	0.2217
metab_7370	neg	649.3448	7.6793	0.7449	-0.5859	0.0337	0.0896
metab_12215	neg	649.3450	7.3343	0.3988	-0.2906	0.2544	0.3828
metab_12622	neg	649.3604	5.7913	0.1474	1.1019	0.9166	0.9446
metab_12378	neg	649.3605	6.8411	0.0184	0.9989	0.8912	0.9268
metab_9910	neg	649.3612	7.1749	0.7141	1.8930	0.2822	0.4129
metab_4614	pos	649.3957	5.5715	1.3086	3.8393	0.0161	0.0497
metab_4097	pos	649.4637	8.2143	0.8652	1.2817	0.1236	0.2221
metab_753	pos	649.4637	8.0419	0.3914	0.5958	0.4983	0.6213
metab_12485	neg	649.4670	6.4073	0.4645	1.2483	0.3185	0.4508
metab_5179	pos	649.8720	3.0726	2.2516	6.4430	0.0000	0.0003
metab_2056	pos	650.1227	3.0726	2.2845	6.1725	0.0000	0.0005
metab_6929	neg	650.1297	3.7830	1.3929	13.1968	0.0188	0.0604
metab_14813	neg	650.2160	0.5991	1.4448	-4.2241	0.0227	0.0687
metab_9870	neg	650.2953	7.0322	1.0944	3.3560	0.0527	0.1221
metab_12662	neg	650.3643	5.6452	0.2205	0.0055	0.5258	0.6429
metab_11431	neg	650.4418	9.5811	0.8595	-0.3782	0.2113	0.3341
metab_6652	neg	650.5151	9.3661	0.7995	0.6519	0.1688	0.2851
metab_3724	pos	650.5342	9.8362	1.4234	-2.4475	0.0011	0.0079
metab_10767	neg	650.8692	14.0282	0.1053	0.0923	0.7559	0.8242
metab_6252	pos	651.1135	0.7801	1.0550	1.8372	0.0543	0.1213
metab_9508	neg	651.1366	5.1131	0.3352	-0.0590	0.2880	0.4195
metab_8677	neg	651.2060	2.2119	0.2426	0.8222	0.6005	0.7053
metab_7076	neg	651.3593	8.1774	0.2743	0.7564	0.3738	0.5048
metab_10047	neg	651.3749	7.7728	0.1778	0.0218	0.6229	0.7233
metab_9584	neg	651.3757	5.5639	0.0499	-0.3371	0.9778	0.9848
metab_7033	neg	651.3759	7.0959	0.1299	0.3851	0.8897	0.9260
metab_9955	neg	651.3763	7.4300	0.3407	-0.0848	0.4426	0.5665
metab_11315	neg	651.3946	9.9767	0.5683	1.3860	0.1385	0.2465
metab_10350	neg	651.4124	8.8608	0.0526	0.4603	0.8201	0.8725
metab_962	pos	651.4792	8.4809	1.8045	2.4565	0.0233	0.0650

metab_7321	neg	651.4862	8.6874	2.0995	4.4358	0.0003	0.0039
metab_12608	neg	652.3115	5.8731	1.2235	-1.5739	0.1161	0.2159
metab_4605	pos	652.3763	5.6473	0.2382	-0.2869	0.7420	0.8225
metab_4447	pos	652.3764	6.4794	0.6418	0.4864	0.2429	0.3676
metab_2702	pos	652.3764	7.1416	0.5018	0.4440	0.1628	0.2724
metab_4521	pos	652.3765	6.0410	0.0030	-0.8908	0.8765	0.9214
metab_12630	neg	652.3800	5.7585	0.2592	-0.2670	0.3555	0.4884
metab_12462	neg	652.3800	6.4709	0.3259	0.4108	0.3632	0.4956
metab_10345	neg	652.4442	8.8286	0.2094	0.2518	0.6539	0.7487
metab_3220	pos	652.6701	11.1749	0.0605	-0.2087	0.7022	0.7923
metab_6753	neg	653.2156	0.6131	1.1249	-1.2634	0.0007	0.0065
metab_14506	neg	653.2577	1.2521	2.1625	-3.3970	0.0025	0.0152
metab_12950	neg	653.3400	4.3716	0.1740	-0.0404	0.7339	0.8080
metab_10057	neg	653.3914	7.8193	0.0234	0.1557	0.8512	0.8969
metab_7343	neg	653.3915	8.1153	0.0806	0.1568	0.7266	0.8031
metab_12357	neg	653.4976	6.8887	1.4283	2.6217	0.0042	0.0215
metab_7134	neg	653.5007	9.8620	1.6358	-2.3485	0.0195	0.0618
metab_12960	neg	654.3083	4.3380	1.3413	6.9712	0.1823	0.3005
metab_9836	neg	654.3271	6.9191	0.0829	-0.1398	0.8718	0.9121
metab_12614	neg	654.3272	5.8409	1.0014	-1.1631	0.1428	0.2523
metab_4449	pos	654.3923	6.4794	0.0075	-0.1120	0.8777	0.9219
metab_4600	pos	654.3923	5.6773	0.3555	-0.8815	0.3232	0.4567
metab_4401	pos	654.3925	6.8131	0.4689	-0.9361	0.1839	0.2986
metab_4249	pos	654.3928	7.5151	0.0512	-0.1223	0.9937	0.9960
metab_6580	neg	654.3958	6.1644	0.3744	-0.3540	0.1672	0.2834
metab_10340	neg	654.4600	8.8128	0.5828	-0.3379	0.0892	0.1789
metab_10648	neg	654.4966	10.3524	1.4809	4.6246	0.0049	0.0241
metab_4048	pos	654.5288	8.4074	0.2256	0.7077	0.5769	0.6907
metab_3712	pos	654.5654	9.8673	1.0962	-1.8336	0.0107	0.0375
metab_3062	pos	654.5658	9.5137	1.0123	2.5489	0.0900	0.1756
metab_3212	pos	654.6018	10.9933	1.7686	-4.2184	0.0006	0.0056
metab_14860	neg	655.1017	0.5711	0.4065	0.0567	0.2469	0.3740
metab_12706	neg	655.3988	5.4355	0.0206	-0.0923	0.9443	0.9637
metab_3076	pos	655.4899	9.6358	1.5680	-2.9187	0.0005	0.0049
metab_4569	pos	656.3395	5.8294	0.9933	-1.1637	0.2151	0.3365
metab_12379	neg	656.3589	6.8255	2.3482	6.4635	0.0000	0.0003
metab_4632	pos	656.3847	5.4662	0.6853	1.3400	0.1065	0.1984
metab_4634	pos	656.4057	5.4357	0.6313	0.8221	0.1314	0.2320
metab_4213	pos	656.4076	7.6919	0.6876	-0.8149	0.0092	0.0339
metab_668	pos	656.4078	6.1009	0.3627	-0.6328	0.2244	0.3477
metab_703	pos	656.4078	7.0228	0.6988	-0.7922	0.0034	0.0168
metab_6578	neg	656.4114	6.3917	1.5199	-2.8297	0.0002	0.0033
metab_11516	neg	656.4751	9.2507	0.2881	0.7413	0.2799	0.4106
metab_2890	pos	656.5447	8.4214	0.7408	1.3881	0.0981	0.1868
metab_11086	neg	656.8863	14.0282	0.2476	-0.0787	0.4851	0.6071
metab_11460	neg	657.3115	9.4647	1.0007	-1.5334	0.0062	0.0286
metab_3056	pos	657.4476	9.4667	1.7160	3.5818	0.0181	0.0541
metab_11223	neg	657.4512	10.4016	1.2126	2.0652	0.0023	0.0142

metab_10364	neg	657.4788	8.9574	0.1583	0.2379	0.8102	0.8649
metab_11490	neg	657.5066	9.3494	0.4875	-0.3476	0.2152	0.3384
metab_3649	pos	657.5071	10.1793	1.2775	-1.7489	0.0030	0.0155
metab_11287	neg	657.5076	10.0749	0.2495	1.0954	0.7862	0.8472
metab_12122	neg	658.3580	7.6632	0.8220	12.8487	0.0389	0.0987
metab_7320	neg	658.3916	8.6874	1.1099	-1.0929	0.0007	0.0066
metab_119	pos	658.4234	7.9118	0.6528	-0.7856	0.0156	0.0487
metab_10286	neg	658.4835	8.6728	0.7222	4.4507	0.2745	0.4048
metab_3953	pos	658.5599	8.7731	1.1247	14.5472	0.0191	0.0563
metab_810	pos	658.5602	9.0112	1.3608	2.8515	0.0073	0.0286
metab_10847	neg	658.9869	14.1744	1.3569	2.1814	0.0001	0.0023
metab_4484	pos	659.4270	6.3277	1.7299	-3.6598	0.0002	0.0030
metab_11193	neg	659.4669	10.7689	1.6717	3.4872	0.0001	0.0021
metab_6973	neg	659.4750	4.9349	0.4650	0.3580	0.5748	0.6843
metab_11458	neg	659.5212	9.4812	0.4840	-0.3820	0.3839	0.5131
metab_10522	neg	659.5223	9.6316	0.8499	0.5709	0.2566	0.3850
metab_3629	pos	659.5231	10.3026	1.4595	-2.2854	0.0022	0.0128
metab_11967	neg	660.3903	8.0843	0.5113	0.7726	0.3896	0.5182
metab_3583	pos	660.3940	10.9772	0.2588	-0.1925	0.1039	0.1950
metab_4105	pos	660.4392	8.1998	1.5630	-2.7942	0.0000	0.0007
metab_10535	neg	660.4472	9.6814	0.8004	-0.2235	0.1265	0.2302
metab_3910	pos	660.5758	8.9958	1.4265	4.2975	0.0001	0.0022
metab_3442	pos	660.9987	14.1972	2.1502	4.0121	0.0010	0.0073
metab_9899	neg	661.2771	7.1437	0.4356	-0.1254	0.1633	0.2782
metab_11033	neg	661.2773	14.0610	0.5004	-0.2432	0.0503	0.1180
metab_10110	neg	661.3260	8.0069	0.2603	0.6184	0.5125	0.6315
metab_7051	neg	661.3831	7.5087	1.0678	3.2030	0.0824	0.1686
metab_11662	neg	661.4654	8.8449	0.9151	2.7681	0.0957	0.1881
metab_7245	neg	661.4830	9.8459	0.5347	-0.5319	0.2159	0.3393
metab_10397	neg	661.4837	9.0713	1.0117	2.7138	0.0744	0.1563
metab_11201	neg	661.5388	10.6686	0.9948	-2.1215	0.0335	0.0894
metab_871	pos	661.5751	9.9782	1.2538	1.6612	0.0224	0.0634
metab_11195	neg	661.5787	10.7359	0.0270	0.4799	0.8758	0.9149
metab_8156	neg	662.1585	1.2663	0.9033	-0.8202	0.0013	0.0100
metab_11561	neg	662.4234	9.1200	1.2690	-0.2765	0.0540	0.1242
metab_11045	neg	662.8255	14.0438	0.5530	0.6718	0.1556	0.2687
metab_9951	neg	663.1755	7.3824	0.4279	0.7236	0.2083	0.3308
metab_2700	pos	663.2889	7.1119	0.3522	-0.2073	0.3732	0.5072
metab_10062	neg	663.2927	7.8352	0.6132	-0.2937	0.0487	0.1156
metab_11034	neg	663.2931	14.0610	0.6295	-0.2653	0.0014	0.0103
metab_12569	neg	663.3239	6.0184	0.5020	0.7761	0.0948	0.1867
metab_3633	pos	663.4525	10.2870	0.6217	-0.5441	0.0003	0.0033
metab_3919	pos	663.5153	8.9507	1.0375	2.4636	0.0421	0.1005
metab_3158	pos	663.5910	10.1492	1.4821	2.4302	0.0197	0.0576
metab_10691	neg	663.5945	11.2289	1.7233	3.7152	0.0015	0.0111
metab_10681	neg	663.5946	10.9673	0.1798	0.8635	0.6614	0.7556
metab_7378	neg	664.3472	7.2225	1.3637	3.0760	0.0033	0.0181
metab_2956	pos	664.4105	8.8169	0.8589	-0.8147	0.0024	0.0133

metab_11232	neg	664.4266	10.3524	0.6205	1.8129	0.1542	0.2669
metab_10309	neg	664.4935	8.7191	1.1011	2.1974	0.0895	0.1794
metab_3078	pos	664.5500	9.6358	1.6345	-2.9405	0.0033	0.0167
metab_864	pos	664.5502	9.8362	1.4928	-2.3947	0.0001	0.0020
metab_10589	neg	664.5531	9.9433	0.8432	5.6800	0.1597	0.2737
metab_11771	neg	664.5534	8.6728	0.7461	0.0304	0.1712	0.2877
metab_6742	neg	665.1458	0.6271	0.4291	0.5453	0.0235	0.0704
metab_12324	neg	665.2919	6.9688	1.5187	2.0050	0.0695	0.1491
metab_6671	neg	665.3400	5.9866	0.4992	-0.3043	0.3161	0.4482
metab_12743	neg	665.3553	5.2257	1.1346	2.5980	0.0643	0.1414
metab_9568	neg	665.3555	5.4684	1.0815	5.1511	0.0729	0.1540
metab_4321	pos	665.4586	7.1119	0.9347	3.7019	0.1761	0.2893
metab_12029	neg	665.4646	7.9292	1.1145	12.9221	0.0151	0.0523
metab_1041	pos	665.6443	1.6693	1.1056	-0.8171	0.0286	0.0759
metab_9918	neg	666.0611	7.2069	0.1131	0.5068	0.7426	0.8146
metab_6928	neg	666.1240	3.6812	1.4555	13.2493	0.0122	0.0454
metab_8631	neg	666.2980	2.0987	2.1831	-6.8289	0.0000	0.0000
metab_12303	neg	666.3270	7.0322	0.7359	-0.3512	0.1185	0.2191
metab_10014	neg	666.3352	7.6793	0.6556	-0.7341	0.0970	0.1900
metab_7401	neg	666.3432	6.5033	1.0608	-0.8902	0.0348	0.0914
metab_826	pos	666.5290	9.2658	1.3562	-2.1013	0.0031	0.0158
metab_813	pos	666.5290	9.0556	0.8237	1.2005	0.0384	0.0935
metab_3222	pos	666.6857	11.1899	0.1717	-0.1839	0.3112	0.4438
metab_12537	neg	667.3537	6.1804	0.4770	0.7742	0.3204	0.4524
metab_9681	neg	667.3689	6.0831	0.1358	0.5840	0.6732	0.7638
metab_12030	neg	667.3691	7.9292	0.7604	-0.5548	0.2038	0.3255
metab_10004	neg	667.3696	7.6169	0.2029	0.1541	0.6445	0.7410
metab_7416	neg	667.3710	5.9375	0.1119	0.8577	0.9513	0.9683
metab_9825	neg	667.3710	6.8730	0.1262	0.7544	0.9194	0.9471
metab_12783	neg	667.3713	5.0159	0.4270	1.1027	0.5302	0.6468
metab_12726	neg	667.3713	5.3228	0.5135	0.8773	0.2715	0.4020
metab_9599	neg	667.3713	5.6292	0.4067	1.2216	0.5977	0.7038
metab_11313	neg	667.4364	9.9767	0.7603	-0.8001	0.1042	0.2001
metab_801	pos	667.4528	8.7731	1.1513	-2.0599	0.0014	0.0093
metab_11413	neg	667.4725	9.6316	1.6129	-2.9979	0.0032	0.0180
metab_2859	pos	667.5128	8.2872	0.5667	0.6952	0.4901	0.6147
metab_11523	neg	667.5532	9.2185	1.2873	1.9060	0.0375	0.0965
metab_14675	neg	668.1751	0.8662	1.6554	-1.6677	0.0025	0.0151
metab_8141	neg	668.2420	1.2521	1.4140	-1.5290	0.0378	0.0971
metab_13474	neg	668.2873	2.8606	2.3843	8.9545	0.0000	0.0006
metab_1794	pos	668.3096	2.0973	2.0405	-4.7608	0.0000	0.0003
metab_7374	neg	668.3424	7.5397	1.3416	-2.5229	0.0053	0.0257
metab_10153	neg	668.3493	8.1774	0.2276	0.1819	0.7046	0.7884
metab_4205	pos	668.3707	7.7357	1.0250	-1.4104	0.0104	0.0368
metab_4586	pos	668.3712	5.7540	1.3057	1.6521	0.0031	0.0159
metab_10199	neg	668.4390	8.3373	1.0397	3.3718	0.1102	0.2084
metab_10271	neg	668.4395	8.6093	1.6495	3.8015	0.0005	0.0056
metab_10618	neg	668.5118	10.1401	0.9659	-1.3951	0.0759	0.1587

metab_3799	pos	668.5445	9.5283	1.0630	-1.4649	0.0173	0.0523
metab_11087	neg	668.8422	14.0282	0.2777	-0.0999	0.2907	0.4220
metab_7985	neg	669.0695	0.7818	1.1989	-3.3625	0.0011	0.0093
metab_7012	neg	669.3866	6.5989	0.2282	0.5895	0.7244	0.8022
metab_7367	neg	669.3869	7.7567	0.0306	0.0759	0.9317	0.9560
metab_9974	neg	669.3871	7.5087	0.0903	0.5726	0.7597	0.8276
metab_10632	neg	669.4513	10.2384	0.3144	0.0395	0.1772	0.2946
metab_98	pos	669.4686	8.7444	1.5407	-2.9798	0.0112	0.0387
metab_11663	neg	669.4959	8.8449	1.4246	3.0996	0.0395	0.0998
metab_11456	neg	669.4961	9.4812	0.6383	-0.5002	0.1577	0.2714
metab_11526	neg	669.5073	9.2185	1.3531	2.3931	0.0216	0.0665
metab_4031	pos	669.5283	8.4651	0.4891	0.5078	0.4610	0.5890
metab_11478	neg	669.5690	9.3991	1.3450	2.0666	0.0214	0.0660
metab_5249	pos	670.2990	2.8568	1.7880	16.7131	0.0005	0.0050
metab_13347	neg	670.3029	3.1797	1.8507	4.6599	0.0007	0.0067
metab_13501	neg	670.3033	2.8123	1.7238	6.2836	0.0220	0.0673
metab_9509	neg	670.3217	5.1131	1.0243	-3.2581	0.3043	0.4352
metab_4244	pos	670.3549	7.5307	1.1580	-2.8168	0.0333	0.0846
metab_10138	neg	670.3821	8.1153	0.3865	0.2888	0.4607	0.5833
metab_4184	pos	670.3866	7.8532	1.6184	-14.2931	0.0000	0.0000
metab_4376	pos	670.3867	6.9035	0.7582	0.9521	0.0598	0.1297
metab_4435	pos	670.3868	6.5704	0.4427	0.2186	0.3729	0.5070
metab_4663	pos	670.3870	5.2868	0.8472	0.7155	0.0365	0.0903
metab_4498	pos	670.3871	6.2226	0.0809	-1.0731	0.9651	0.9788
metab_12293	neg	670.3900	7.0629	0.0559	0.0473	0.9942	0.9952
metab_12533	neg	670.3907	6.1968	0.0131	0.1526	0.8719	0.9121
metab_9577	neg	670.3909	5.5322	0.3169	-0.2301	0.2464	0.3734
metab_12749	neg	670.3911	5.1939	0.2206	-0.2165	0.3688	0.5003
metab_9957	neg	671.2160	7.4300	1.9452	-14.0811	0.0000	0.0000
metab_12800	neg	671.3509	4.9349	0.1507	0.3191	0.9055	0.9373
metab_12381	neg	671.4002	6.8255	0.8728	-1.1623	0.0616	0.1369
metab_10490	neg	671.4308	9.4984	2.2184	5.2443	0.0188	0.0603
metab_849	pos	671.4626	9.6521	0.6199	-1.0958	0.1818	0.2959
metab_11210	neg	671.4671	10.5679	0.4341	-0.2477	0.0693	0.1489
metab_3962	pos	671.4842	8.7303	1.4635	-2.6428	0.0101	0.0361
metab_11356	neg	671.5120	9.8459	0.7207	-0.6477	0.0478	0.1140
metab_10393	neg	671.5125	9.0557	1.5643	3.4060	0.0132	0.0475
metab_542	pos	671.8434	3.5311	3.0043	6.5761	0.0007	0.0061
metab_14671	neg	672.2368	0.8662	0.4717	-0.1326	0.3694	0.5009
metab_5151	pos	672.3148	3.1799	1.9688	4.8641	0.0009	0.0070
metab_5268	pos	672.3151	2.8108	1.9069	9.0171	0.0054	0.0231
metab_8991	neg	672.3187	3.0101	1.6257	4.2558	0.0075	0.0325
metab_4845	pos	672.3783	4.3972	0.6602	0.7966	0.3534	0.4884
metab_4931	pos	672.3792	4.0183	1.1440	2.5504	0.0413	0.0990
metab_4193	pos	672.4019	7.7946	0.2789	-0.2909	0.6427	0.7455
metab_2687	pos	672.4028	7.0526	0.4838	0.2281	0.1049	0.1964
metab_2570	pos	672.4028	6.1614	0.1166	0.0337	0.8992	0.9348
metab_4428	pos	672.4028	6.6001	0.6558	0.7296	0.0283	0.0755

metab_4591	pos	672.4028	5.7086	0.3409	0.0819	0.4057	0.5388
metab_4677	pos	672.4029	5.1975	0.0116	-0.2526	0.8849	0.9270
metab_12671	neg	672.4067	5.6128	1.1214	-1.6025	0.0014	0.0108
metab_10248	neg	672.4706	8.5299	0.9415	1.7194	0.0146	0.0509
metab_2995	pos	672.5757	9.0556	1.1486	4.6842	0.0817	0.1634
metab_11466	neg	673.4462	9.4647	0.8677	1.4982	0.0901	0.1800
metab_4462	pos	673.4612	6.4035	0.4778	1.2565	0.3630	0.4969
metab_3191	pos	673.4788	10.5777	0.3932	-0.4943	0.1179	0.2140
metab_10680	neg	673.4814	10.9673	0.6392	0.8507	0.0117	0.0439
metab_838	pos	673.5000	9.4981	1.0694	-1.3756	0.0086	0.0324
metab_7277	neg	673.5270	9.2507	1.5198	3.0770	0.0255	0.0744
metab_771	pos	673.5496	8.3763	0.5413	-0.5522	0.3323	0.4671
metab_6586	neg	674.0503	0.5286	1.4808	2.4779	0.0001	0.0018
metab_504	pos	674.3295	2.9954	0.0223	-0.0001	0.8928	0.9317
metab_9066	neg	674.3526	3.2637	1.9466	12.6846	0.0000	0.0002
metab_1003	pos	674.4183	6.1614	0.1986	-0.3835	0.3851	0.5175
metab_2709	pos	674.4184	7.2014	0.3598	-0.5034	0.1655	0.2757
metab_7304	neg	674.4857	8.7640	1.3678	2.5227	0.0021	0.0135
metab_3907	pos	674.5029	9.0112	0.5379	1.1734	0.4910	0.6152
metab_11394	neg	674.5368	9.6814	0.5870	2.6554	0.3911	0.5195
metab_10548	neg	675.3945	9.7470	0.6156	-0.0614	0.1482	0.2591
metab_2480	pos	675.4213	5.5112	0.4413	-0.7900	0.1791	0.2928
metab_11544	neg	675.5174	9.1693	0.9453	1.2456	0.0250	0.0735
metab_2944	pos	675.5655	8.7161	0.0947	0.2088	0.8314	0.8893
metab_12286	neg	676.2205	7.0796	0.7503	1.1379	0.1988	0.3195
metab_2109	pos	676.3651	3.2573	1.0947	12.6916	0.0103	0.0367
metab_991	pos	676.4341	6.4035	1.3775	-2.4252	0.0001	0.0011
metab_2771	pos	676.4341	7.6481	1.5177	-14.4431	0.0001	0.0020
metab_4257	pos	676.4343	7.4561	1.8520	-3.5900	0.0013	0.0090
metab_3185	pos	676.4898	10.4092	1.4760	3.4596	0.0104	0.0368
metab_10438	neg	676.5004	9.1858	1.0928	1.7840	0.0134	0.0482
metab_11648	neg	676.5012	8.9252	1.3275	2.3556	0.0033	0.0182
metab_3741	pos	676.5193	9.7738	1.2240	-1.6347	0.0194	0.0569
metab_848	pos	676.5482	9.6358	1.4386	-2.6005	0.0187	0.0554
metab_12716	neg	677.2718	5.3713	1.1941	-1.5507	0.0044	0.0221
metab_13186	neg	677.3449	3.6311	2.5931	-5.8895	0.0000	0.0007
metab_11331	neg	677.4106	9.9108	0.1234	1.0144	0.7131	0.7942
metab_11823	neg	677.4623	8.5299	0.9505	1.5528	0.1310	0.2363
metab_11481	neg	677.5327	9.3824	0.1066	0.2474	0.7823	0.8446
metab_10363	neg	677.5336	8.9417	1.6554	9.7419	0.0386	0.0983
metab_12193	neg	678.3085	7.4465	0.8940	1.2092	0.0049	0.0241
metab_12145	neg	678.3088	7.5855	0.0755	-0.0618	0.8215	0.8736
metab_12835	neg	678.3205	4.8210	1.3082	-1.4235	0.0181	0.0591
metab_12914	neg	678.3206	4.5391	1.5836	-2.3067	0.0056	0.0265
metab_11272	neg	678.4408	10.1401	1.0292	-1.5075	0.0277	0.0787
metab_3723	pos	678.5654	9.8362	1.6355	-3.2451	0.0000	0.0011
metab_3680	pos	678.6002	9.9470	1.4877	7.5375	0.0300	0.0787
metab_11279	neg	679.4262	10.1082	0.4191	1.7725	0.4952	0.6165

metab_11760	neg	679.4777	8.6874	1.0944	1.7907	0.0435	0.1069
metab_10563	neg	679.5161	9.8459	1.2433	-2.0105	0.0315	0.0855
metab_836	pos	679.5859	9.4667	0.6425	0.8192	0.1062	0.1980
metab_9842	neg	680.2671	6.9354	0.2948	-0.1017	0.3769	0.5074
metab_9668	neg	680.3145	6.0184	0.3279	0.7981	0.3814	0.5112
metab_12024	neg	680.3241	7.9612	3.4826	12.0895	0.0000	0.0000
metab_13017	neg	680.3385	4.1535	1.4395	4.4997	0.1068	0.2039
metab_12477	neg	680.3959	6.4073	0.3815	1.6817	0.4647	0.5871
metab_3630	pos	680.4787	10.2870	0.8524	-1.0298	0.0125	0.0417
metab_3820	pos	680.5445	9.4049	1.4811	-4.7081	0.0000	0.0004
metab_818	pos	680.5800	9.0112	0.4303	0.7678	0.5145	0.6361
metab_7158	neg	680.7974	14.0282	0.2842	-0.0183	0.2009	0.3218
metab_7099	neg	681.4518	8.7191	0.9909	-1.6040	0.0244	0.0723
metab_10372	neg	681.4935	8.9903	1.4578	2.8093	0.0098	0.0392
metab_11487	neg	681.5687	9.3494	0.9251	2.9070	0.0353	0.0922
metab_912	pos	681.6017	9.6199	1.1721	1.3911	0.0299	0.0786
metab_14959	neg	681.9869	0.5286	1.7648	3.1884	0.0004	0.0046
metab_2487	pos	682.3183	5.5565	0.6079	1.1350	0.4200	0.5514
metab_7384	neg	682.3218	7.0796	0.8993	-0.7518	0.0672	0.1456
metab_9661	neg	682.3300	5.9866	0.4124	-0.1113	0.4983	0.6189
metab_2280	pos	682.3507	4.1387	1.3371	4.2904	0.0571	0.1258
metab_2762	pos	682.3535	7.5450	1.0023	-14.7069	0.0224	0.0633
metab_13036	neg	682.3542	4.0859	1.1371	2.5789	0.2347	0.3601
metab_2588	pos	682.4084	6.4035	0.6675	1.2922	0.2126	0.3337
metab_4056	pos	682.5234	8.3763	1.0131	-1.9526	0.1122	0.2061
metab_3796	pos	682.5602	9.5283	2.9925	-3.8245	0.0023	0.0132
metab_900	pos	682.6327	11.4499	1.6011	-2.9204	0.0000	0.0003
metab_6668	neg	683.3504	5.4684	0.9482	2.1850	0.0372	0.0957
metab_11224	neg	683.4665	10.3849	1.5788	3.0223	0.0016	0.0116
metab_10505	neg	683.5237	9.5478	0.1352	0.3184	0.9256	0.9516
metab_10493	neg	683.5840	9.5147	1.0526	2.7223	0.0080	0.0340
metab_6277	pos	683.9985	0.7381	1.8715	4.7613	0.0000	0.0003
metab_34	pos	683.9993	0.5280	1.9187	4.1599	0.0003	0.0034
metab_3613	pos	684.2013	10.4865	0.2984	0.7535	0.4711	0.5981
metab_7992	neg	684.2358	0.8098	1.4052	-1.6988	0.0409	0.1024
metab_713	pos	684.3342	7.1416	0.8762	-1.0431	0.0891	0.1742
metab_10010	neg	684.3373	7.6479	0.3825	0.3983	0.5813	0.6896
metab_12170	neg	684.3375	7.5239	0.1150	0.7963	0.7820	0.8445
metab_2275	pos	684.3658	4.0788	1.3738	3.9711	0.0262	0.0714
metab_2969	pos	684.5395	8.9212	0.9556	-1.3828	0.0347	0.0872
metab_10782	neg	685.2749	14.0438	0.6088	-0.3023	0.0127	0.0465
metab_6672	neg	685.3668	5.9542	1.3863	2.7119	0.0106	0.0412
metab_4466	pos	685.4171	6.4035	0.8489	1.4367	0.1596	0.2682
metab_11505	neg	685.4459	9.3000	0.2771	-0.1706	0.4729	0.5951
metab_10531	neg	685.4469	9.6644	0.5558	0.6563	0.5357	0.6509
metab_4021	pos	685.4631	8.5241	2.6949	-5.1993	0.0004	0.0041
metab_10676	neg	685.4824	10.7689	0.6541	0.8954	0.1003	0.1944
metab_11614	neg	685.4834	9.0067	0.8559	-1.1712	0.1596	0.2737

metab_11559	neg	685.4843	9.1200	0.3384	0.3762	0.6634	0.7568
metab_13631	neg	686.2976	2.5431	1.9050	6.9835	0.0000	0.0005
metab_8716	neg	686.2977	2.3078	1.8780	6.8939	0.0000	0.0006
metab_13961	neg	686.2978	1.9280	1.2734	13.7396	0.0075	0.0326
metab_4248	pos	686.3486	7.5151	0.2307	0.8297	0.6577	0.7565
metab_12435	neg	686.3769	6.5989	0.0244	0.7761	0.9243	0.9508
metab_3846	pos	687.4568	9.2815	1.6331	-2.9764	0.0059	0.0248
metab_10524	neg	687.4619	9.6477	0.1038	-0.0531	0.7645	0.8311
metab_2927	pos	687.4780	8.6284	1.2298	-2.0950	0.0086	0.0324
metab_7246	neg	687.4972	9.8294	0.7515	-0.4468	0.1583	0.2721
metab_6654	neg	687.5067	9.2185	0.4490	-0.4313	0.1218	0.2237
metab_7909	neg	688.1729	0.5991	0.2618	0.5765	0.2222	0.3462
metab_1734	pos	688.3086	1.9169	1.3408	13.3497	0.0079	0.0305
metab_1863	pos	688.3095	2.3133	2.0770	7.8106	0.0000	0.0007
metab_5348	pos	688.3096	2.5469	2.7037	8.0207	0.0003	0.0031
metab_9249	neg	688.3327	3.9005	1.2948	-2.9448	0.2093	0.3319
metab_10063	neg	688.4653	7.8352	1.4532	2.3940	0.0036	0.0195
metab_10495	neg	688.4841	9.5314	0.8853	-0.9367	0.0503	0.1179
metab_3661	pos	688.4904	10.1021	0.1866	0.8740	0.6899	0.7829
metab_11228	neg	688.4908	10.3687	1.4003	2.0629	0.0506	0.1184
metab_11597	neg	688.5022	9.0557	1.7401	3.4607	0.0148	0.0513
metab_7131	neg	688.5522	9.7470	0.1455	2.0492	0.9016	0.9343
metab_7204	neg	688.5664	11.0007	2.0367	-4.2953	0.0002	0.0030
metab_12320	neg	689.3435	7.0002	1.2052	-1.6944	0.0294	0.0816
metab_11665	neg	689.3727	8.8286	0.6641	0.5210	0.2969	0.4276
metab_3734	pos	689.4729	9.7899	0.4549	1.4244	0.5387	0.6589
metab_3935	pos	689.4947	8.8904	1.1546	-1.7161	0.0052	0.0226
metab_2245	pos	690.3446	3.9114	1.2691	-3.4507	0.1683	0.2789
metab_9682	neg	690.3724	6.0993	0.7171	-0.7940	0.0260	0.0755
metab_4916	pos	690.4127	4.0634	1.0971	1.7123	0.0591	0.1287
metab_2473	pos	690.4129	5.4357	0.5245	-0.8559	0.2661	0.3941
metab_2575	pos	690.4129	6.2071	0.7327	0.7198	0.1180	0.2142
metab_10557	neg	690.4804	9.7804	0.7944	1.6605	0.3402	0.4734
metab_10145	neg	690.4806	8.1307	2.2544	6.8520	0.0000	0.0000
metab_3605	pos	690.5054	10.5777	0.4471	-0.5227	0.0779	0.1576
metab_10449	neg	690.5165	9.2507	1.1601	2.8987	0.0744	0.1564
metab_6648	neg	690.5820	11.5691	0.8704	-2.2809	0.1684	0.2848
metab_9596	neg	691.2513	5.6128	0.1642	0.6201	0.8065	0.8620
metab_11674	neg	691.3893	8.8128	0.6347	-0.2349	0.0833	0.1699
metab_11694	neg	691.4806	8.7488	1.5053	-2.6137	0.0173	0.0571
metab_3894	pos	691.5088	9.0857	1.2895	-1.7047	0.0046	0.0208
metab_11797	neg	691.5118	8.6093	0.8546	-1.0121	0.2045	0.3263
metab_12297	neg	692.2151	7.0469	0.8769	1.9280	0.1200	0.2214
metab_4609	pos	692.4296	5.6168	1.2227	-2.0906	0.0036	0.0176
metab_3584	pos	692.5205	10.9614	1.0757	1.7060	0.0063	0.0257
metab_3717	pos	692.5800	9.8362	1.9138	-3.7793	0.0002	0.0026
metab_7390	neg	693.2559	6.9354	0.1782	-0.0600	0.8070	0.8624
metab_7369	neg	693.3349	7.6793	0.7270	-0.4316	0.1596	0.2737

metab_10366	neg	693.4044	8.9574	0.2532	0.5348	0.6900	0.7776
metab_10463	neg	693.4051	9.3165	0.5784	1.3847	0.1211	0.2228
metab_10509	neg	693.4053	9.5811	1.3692	3.0816	0.0169	0.0561
metab_766	pos	693.4473	8.2728	0.8744	-1.3687	0.1953	0.3121
metab_10601	neg	693.4512	10.0090	0.0137	0.1293	0.8770	0.9158
metab_7100	neg	693.4961	8.7346	1.4463	-2.4868	0.0201	0.0631
metab_907	pos	693.5994	9.7283	0.2039	1.5879	0.7011	0.7915
metab_14785	neg	694.1613	0.6131	0.4233	2.8374	0.2490	0.3764
metab_13233	neg	694.3159	3.5141	1.6193	-3.0016	0.0049	0.0243
metab_3904	pos	694.5600	9.0254	0.8781	-0.6168	0.3837	0.5163
metab_3047	pos	694.5602	9.3891	1.5270	-2.5118	0.0019	0.0117
metab_12344	neg	695.2148	6.9191	0.5442	0.3365	0.3760	0.5067
metab_9884	neg	695.2148	7.0629	0.6838	-0.6549	0.0548	0.1255
metab_3604	pos	695.4609	10.5777	0.6988	-0.9652	0.0301	0.0788
metab_3586	pos	695.4609	10.8847	0.4108	-0.6929	0.1896	0.3051
metab_830	pos	695.4632	9.3590	0.5954	-1.4708	0.1763	0.2895
metab_7146	neg	695.4670	10.2384	0.4524	-0.3820	0.1023	0.1973
metab_7126	neg	695.5118	9.5979	1.0005	-0.9764	0.0172	0.0569
metab_10485	neg	695.5833	9.4647	1.1901	1.8575	0.0154	0.0531
metab_903	pos	695.6174	9.8673	1.4176	2.7466	0.0263	0.0715
metab_14757	neg	696.1895	0.6551	1.3665	2.2709	0.0181	0.0591
metab_2162	pos	696.3276	3.5162	1.5358	-2.8118	0.0349	0.0875
metab_7385	neg	696.3380	7.0469	0.5281	0.0272	0.3019	0.4323
metab_874	pos	696.5392	10.0715	0.1614	0.2289	0.7475	0.8275
metab_946	pos	696.5757	9.5594	1.3994	-2.1443	0.0005	0.0051
metab_7027	neg	697.2307	6.9834	0.5787	1.5467	0.1916	0.3114
metab_3775	pos	697.4772	9.6358	0.2356	-0.1220	0.7236	0.8096
metab_880	pos	697.4788	10.2404	0.4312	-0.5893	0.1078	0.2003
metab_10663	neg	697.4814	10.5343	0.2341	0.3734	0.3443	0.4773
metab_7253	neg	697.5997	9.6316	1.2715	1.8607	0.0210	0.0651
metab_12672	neg	698.3169	5.6128	0.7884	1.0192	0.2891	0.4206
metab_4998	pos	698.3455	3.7297	1.6218	7.7772	0.0274	0.0738
metab_7688	neg	698.3493	3.1797	0.4773	-0.6767	0.3076	0.4388
metab_9097	neg	698.3494	3.3631	1.3018	4.0114	0.0106	0.0411
metab_9973	neg	698.3537	7.4927	1.3861	-2.8361	0.0130	0.0470
metab_12649	neg	698.3853	5.6776	0.8835	-2.4292	0.0953	0.1875
metab_7019	neg	698.3859	6.8093	0.4782	-1.5715	0.4114	0.5384
metab_13044	neg	699.3455	4.0685	0.5705	1.5652	0.2296	0.3547
metab_10413	neg	699.4621	9.1038	1.8574	-2.9096	0.0110	0.0420
metab_11452	neg	699.4622	9.4984	1.6713	5.9883	0.0297	0.0820
metab_10277	neg	699.4625	8.6239	1.0733	-1.4899	0.0187	0.0601
metab_3610	pos	699.4929	10.5313	0.2407	0.1168	0.2869	0.4172
metab_11188	neg	699.4971	10.9673	0.2195	0.2924	0.5014	0.6218
metab_11327	neg	699.5243	9.9274	0.2572	0.4269	0.4921	0.6135
metab_3653	pos	699.5798	10.1492	1.0935	-0.4587	0.1670	0.2776
metab_12635	neg	700.3322	5.7420	2.0446	13.9078	0.0001	0.0024
metab_12701	neg	700.3402	5.4684	1.1805	2.5706	0.0355	0.0926
metab_514	pos	700.3612	3.1648	1.8390	3.8555	0.0095	0.0346

metab_9091	neg	700.3645	3.3304	1.2500	7.3755	0.0796	0.1648
metab_9691	neg	700.4012	6.1482	0.4071	-0.4679	0.1799	0.2977
metab_11754	neg	700.4024	8.6874	1.6692	-3.1565	0.0000	0.0005
metab_3046	pos	700.4898	9.3891	1.0433	1.6923	0.1590	0.2675
metab_11253	neg	700.4935	10.2384	1.1057	1.5787	0.0032	0.0180
metab_11416	neg	700.4937	9.6147	0.5064	0.8943	0.3330	0.4658
metab_112	pos	700.5342	9.9154	0.3002	0.1978	0.3802	0.5126
metab_2059	pos	701.0498	3.0726	1.1828	5.2536	0.0220	0.0625
metab_12348	neg	701.3396	6.9046	0.9208	1.0228	0.0034	0.0185
metab_5321	pos	701.3608	2.6090	1.3180	12.5227	0.0103	0.0367
metab_11649	neg	701.4421	8.9086	0.7254	1.7277	0.1884	0.3075
metab_11579	neg	701.4779	9.1038	1.2350	-1.8412	0.0032	0.0180
metab_1844	pos	701.8556	2.2361	1.7267	14.9871	0.0044	0.0200
metab_2079	pos	701.8618	3.1799	2.8258	15.6502	0.0000	0.0000
metab_12586	neg	702.3565	5.9542	1.1825	3.0499	0.0282	0.0795
metab_5293	pos	702.3769	2.7188	1.9700	9.3117	0.0038	0.0181
metab_9132	neg	702.3811	3.5141	0.3761	-0.3770	0.2885	0.4201
metab_6665	neg	702.4170	7.8984	0.8773	-0.9752	0.0057	0.0269
metab_3637	pos	702.5052	10.2404	1.2211	1.6511	0.0010	0.0076
metab_10661	neg	702.5089	10.5180	1.7521	3.4690	0.0002	0.0035
metab_7154	neg	702.5821	11.2122	1.5243	-3.0604	0.0017	0.0117
metab_12114	neg	703.2646	7.6793	0.8762	-3.1728	0.0340	0.0900
metab_11668	neg	703.4129	8.8286	0.3011	0.0202	0.2024	0.3238
metab_11496	neg	703.4567	9.3329	0.1777	-0.4917	0.6225	0.7231
metab_11690	neg	703.4916	8.7488	1.1295	-1.3707	0.0468	0.1125
metab_12268	neg	704.3042	7.1437	1.1566	-1.1763	0.0148	0.0514
metab_10003	neg	704.3582	7.6169	1.9807	-3.7516	0.0007	0.0069
metab_1999	pos	704.3920	2.8411	1.5288	5.5229	0.0677	0.1426
metab_4808	pos	704.3922	4.5785	1.4613	2.1168	0.0011	0.0078
metab_2238	pos	704.3924	3.8821	0.2899	0.5779	0.6235	0.7300
metab_4852	pos	704.3924	4.3818	1.3444	2.0430	0.0045	0.0202
metab_5053	pos	704.3928	3.5162	0.3160	-0.2797	0.3911	0.5231
metab_6630	neg	704.4329	8.1936	1.7880	-3.3736	0.0001	0.0022
metab_11524	neg	704.4966	9.2185	0.6273	-0.8904	0.0701	0.1499
metab_3189	pos	704.5202	10.5313	1.1603	1.7815	0.0036	0.0176
metab_10441	neg	705.4288	9.2025	2.1762	-4.3519	0.0001	0.0023
metab_11472	neg	705.4715	9.4322	0.0737	0.4168	0.8452	0.8926
metab_2865	pos	705.4896	8.3018	1.2222	-2.2893	0.0153	0.0481
metab_14511	neg	706.1686	1.2377	1.3634	-4.9166	0.0144	0.0506
metab_9989	neg	706.3193	7.5541	1.3593	-13.7874	0.0090	0.0370
metab_2144	pos	706.3919	3.4098	0.8479	-0.9411	0.0632	0.1351
metab_1978	pos	706.4081	2.7653	1.4019	4.8630	0.0771	0.1566
metab_8283	neg	707.2529	1.4568	1.0784	-0.6311	0.0803	0.1657
metab_10242	neg	707.4509	8.5135	1.2875	2.5510	0.0569	0.1293
metab_11872	neg	707.4753	8.3687	2.0860	-5.1663	0.0001	0.0022
metab_765	pos	707.5052	8.3018	0.4191	-0.8174	0.3904	0.5227
metab_3094	pos	707.6169	9.7129	1.5286	3.0164	0.0030	0.0156
metab_9913	neg	708.3830	7.1903	0.5266	-1.4834	0.3179	0.4502

metab_7103	neg	708.4043	8.8286	1.1527	-1.3140	0.0007	0.0068
metab_11683	neg	708.4711	8.7640	1.0496	-2.0497	0.0607	0.1354
metab_2658	pos	708.4874	6.9182	0.0388	0.1377	0.7783	0.8503
metab_10659	neg	708.4886	10.4679	0.8163	-0.5612	0.1721	0.2885
metab_3173	pos	708.5103	10.2870	0.8632	-1.1022	0.0294	0.0777
metab_852	pos	708.5392	9.6358	0.1755	0.0630	0.6882	0.7813
metab_8146	neg	709.2335	1.2521	1.5625	-4.1667	0.0036	0.0195
metab_10239	neg	709.3994	8.5135	0.7690	1.5640	0.1160	0.2158
metab_4081	pos	709.4423	8.2872	0.3957	-1.1037	0.6037	0.7135
metab_11485	neg	709.4465	9.3661	0.1180	-0.5128	0.7243	0.8022
metab_4033	pos	709.5204	8.4651	0.2930	-0.3475	0.6351	0.7397
metab_12312	neg	710.3534	7.0162	0.5424	0.0162	0.2898	0.4212
metab_11260	neg	710.4690	10.1910	0.3212	1.4477	0.3797	0.5096
metab_2850	pos	710.4720	8.2435	0.1719	-1.3769	0.7536	0.8312
metab_11333	neg	710.4779	9.8947	0.5911	-0.3811	0.2174	0.3409
metab_11695	neg	710.4878	8.7488	1.0233	-1.2611	0.0600	0.1342
metab_3082	pos	710.5549	9.6671	0.4684	-0.6308	0.1574	0.2652
metab_14885	neg	710.9523	0.5571	0.6889	-0.4929	0.0390	0.0990
metab_9585	neg	711.2103	5.5808	1.2994	-1.8318	0.0205	0.0642
metab_11858	neg	711.4534	8.4336	0.1723	-0.6991	0.8598	0.9034
metab_3077	pos	711.4565	9.6358	0.1484	-0.4521	0.7580	0.8347
metab_3932	pos	711.4569	8.8904	0.1876	-1.4304	0.7554	0.8327
metab_779	pos	711.4569	8.4504	0.1214	0.0251	0.8727	0.9196
metab_833	pos	711.4579	9.3736	0.1249	-0.5397	0.8169	0.8799
metab_11483	neg	711.4620	9.3661	0.6158	-1.2234	0.1619	0.2767
metab_7296	neg	711.5075	8.9252	0.9121	-1.1672	0.0193	0.0613
metab_11583	neg	711.5787	9.0872	1.5919	2.7287	0.0016	0.0114
metab_14975	neg	712.0058	0.5286	1.1568	1.8366	0.0001	0.0015
metab_9897	neg	712.3327	7.1266	1.0475	-1.6110	0.0520	0.1208
metab_9854	neg	712.3330	6.9834	0.3898	0.7848	0.5610	0.6721
metab_3829	pos	712.4893	9.3590	0.7072	-1.0555	0.1212	0.2188
metab_3706	pos	712.4894	9.8836	0.5333	-0.5933	0.2576	0.3845
metab_11284	neg	712.4942	10.0924	0.0837	0.0426	0.6966	0.7822
metab_7229	neg	712.5373	10.0593	0.0137	0.1961	0.9811	0.9868
metab_11623	neg	713.4315	8.9903	1.5788	2.4764	0.0093	0.0380
metab_3832	pos	713.4730	9.3429	0.5570	-0.6607	0.3600	0.4938
metab_7254	neg	713.4770	9.6316	0.5776	-0.9804	0.1920	0.3117
metab_2403	pos	714.3983	4.9413	0.1501	0.2058	0.7732	0.8467
metab_10331	neg	714.4799	8.7488	1.3962	13.9930	0.0133	0.0480
metab_843	pos	714.5051	9.5594	0.1979	-0.4282	0.6521	0.7532
metab_3639	pos	714.5055	10.2404	0.4400	-0.5685	0.0749	0.1529
metab_11226	neg	714.5092	10.3849	0.6186	-0.7142	0.0469	0.1126
metab_3670	pos	714.5497	10.0715	0.0938	-0.0812	0.8737	0.9202
metab_9755	neg	715.3756	6.4709	0.0651	0.0903	0.7350	0.8088
metab_12385	neg	715.3768	6.8093	0.4057	-1.2249	0.4636	0.5860
metab_10650	neg	715.4272	10.3687	2.5768	-7.1466	0.0000	0.0001
metab_11372	neg	715.5121	9.7639	0.6209	-0.6436	0.1311	0.2363
metab_6657	neg	715.5369	8.7031	0.2487	0.4284	0.5376	0.6524

metab_7153	neg	715.5846	11.0007	1.5604	-3.1309	0.0013	0.0102
metab_13041	neg	716.3357	4.0685	0.4803	2.0547	0.3926	0.5211
metab_13223	neg	716.3600	3.5310	1.1843	4.8859	0.0333	0.0890
metab_12425	neg	716.3950	6.6157	0.5812	1.1424	0.2138	0.3365
metab_11376	neg	716.3975	9.7470	0.9194	-0.6508	0.0727	0.1537
metab_4360	pos	716.4286	6.9638	0.1320	0.0454	0.9143	0.9438
metab_10473	neg	716.4888	9.3824	0.9497	1.6634	0.1512	0.2630
metab_885	pos	716.5210	10.3942	0.5039	-0.7460	0.0653	0.1387
metab_10670	neg	716.5241	10.7023	0.6069	0.6709	0.0652	0.1427
metab_6649	neg	716.5977	11.4384	1.6482	-3.2072	0.0002	0.0028
metab_9684	neg	717.3915	6.0993	0.5057	-0.4929	0.0783	0.1627
metab_3721	pos	717.5249	9.8362	0.9993	1.2855	0.0236	0.0658
metab_11839	neg	717.5557	8.4972	0.9929	2.7075	0.0820	0.1681
metab_1936	pos	718.3712	2.5782	1.7725	8.7245	0.0106	0.0373
metab_5164	pos	718.3717	3.1191	1.8437	6.8253	0.0026	0.0143
metab_13416	neg	718.3757	3.0101	2.1745	6.8723	0.0001	0.0018
metab_13123	neg	718.3759	3.8001	1.8661	5.5481	0.0003	0.0036
metab_7377	neg	718.4119	7.2069	0.4699	-0.7765	0.2248	0.3494
metab_3195	pos	718.5351	10.7001	0.6543	0.4718	0.0198	0.0577
metab_14826	neg	719.2029	0.5991	1.2659	4.0124	0.0148	0.0515
metab_10079	neg	719.4074	7.9137	1.1594	-1.6577	0.0084	0.0352
metab_3579	pos	719.4605	11.0382	0.3669	-0.5902	0.2439	0.3685
metab_3612	pos	719.4605	10.4865	0.8141	-1.1330	0.0290	0.0767
metab_11894	neg	719.4874	8.3055	0.0717	0.1470	0.9937	0.9950
metab_2663	pos	720.3098	6.9336	0.9575	-4.6998	0.0392	0.0951
metab_2228	pos	720.3872	3.8063	1.8073	5.5713	0.0002	0.0024
metab_507	pos	720.3874	3.0422	1.5930	3.2306	0.0001	0.0017
metab_11354	neg	720.4851	9.8459	1.6717	-3.8924	0.0008	0.0075
metab_11924	neg	721.4233	8.1936	2.1446	-6.1344	0.0000	0.0003
metab_7303	neg	721.4673	8.7640	0.8036	0.8701	0.0022	0.0138
metab_7328	neg	721.5046	8.5604	0.3390	0.6378	0.5385	0.6533
metab_188	pos	721.9551	0.5420	1.9599	4.0989	0.0002	0.0030
metab_9695	neg	722.3834	6.1644	0.4957	-0.6295	0.1406	0.2494
metab_2025	pos	722.4024	2.9501	1.6106	5.5317	0.0110	0.0383
metab_2211	pos	722.4024	3.7297	0.1863	0.1743	0.6967	0.7884
metab_4941	pos	722.4026	3.9722	0.9091	2.1774	0.1239	0.2225
metab_2103	pos	722.4027	3.2420	1.2711	14.2601	0.0143	0.0457
metab_5352	pos	722.4027	2.5314	1.6382	15.5967	0.0031	0.0159
metab_5489	pos	722.4028	2.2209	1.7049	6.3112	0.0073	0.0286
metab_11255	neg	722.5149	10.2070	2.4258	-6.4439	0.0010	0.0084
metab_10189	neg	723.4258	8.3055	0.0592	-0.7392	0.9164	0.9445
metab_10688	neg	723.5358	11.1796	0.0377	0.2301	0.8548	0.8994
metab_3059	pos	723.5388	9.4825	0.3248	0.7298	0.5768	0.6907
metab_905	pos	723.5537	9.8049	2.8416	7.7467	0.0000	0.0001
metab_10539	neg	723.6160	9.6986	1.5851	2.8040	0.0039	0.0206
metab_14503	neg	724.2178	1.2521	0.9143	-1.1903	0.0469	0.1127
metab_11234	neg	724.4854	10.3524	0.9955	2.1987	0.0079	0.0336
metab_11306	neg	724.4936	9.9929	0.8534	1.2322	0.0115	0.0434

metab_3179	pos	724.5707	10.3480	0.4123	1.0723	0.4826	0.6081
metab_10314	neg	724.5747	8.7191	1.0939	3.8977	0.0341	0.0902
metab_11903	neg	725.4412	8.2897	0.5148	-0.8190	0.4162	0.5423
metab_10564	neg	725.6313	9.8459	1.7275	2.4545	0.0058	0.0273
metab_5040	pos	725.7506	3.5620	1.3206	13.3714	0.0208	0.0600
metab_7392	neg	726.3487	6.9354	0.8945	-0.1949	0.1028	0.1978
metab_3745	pos	726.5495	9.7586	0.4969	0.3620	0.0912	0.1774
metab_10173	neg	727.4569	8.2577	0.5388	-0.8936	0.3252	0.4576
metab_12093	neg	727.5011	7.7251	1.2427	-2.5228	0.1129	0.2123
metab_6708	neg	727.9930	0.5286	1.5536	2.5760	0.0017	0.0118
metab_10357	neg	728.4963	8.9252	0.9800	-1.1840	0.0111	0.0422
metab_10267	neg	728.4964	8.6093	0.8528	-0.8205	0.0719	0.1526
metab_3067	pos	728.5212	9.5438	1.4760	12.9341	0.0524	0.1181
metab_3128	pos	728.5222	9.8994	0.3251	-1.8462	0.7247	0.8104
metab_851	pos	728.5645	9.6968	0.5228	-0.5028	0.0865	0.1706
metab_879	pos	728.5657	10.2085	0.3553	-0.3225	0.1823	0.2964
metab_6639	neg	728.6422	12.1668	0.3808	-1.9963	0.5971	0.7034
metab_10386	neg	729.4715	9.0391	0.2347	0.0540	0.5607	0.6719
metab_11308	neg	729.4719	9.9929	0.7199	-0.5152	0.0108	0.0416
metab_11522	neg	729.4989	9.2185	0.5766	-1.0636	0.1442	0.2540
metab_10155	neg	729.5160	8.1774	0.1079	0.6260	0.9064	0.9379
metab_11176	neg	729.6011	11.2122	1.2783	-2.6954	0.0030	0.0171
metab_10260	neg	730.4753	8.5937	0.1418	1.6386	0.8007	0.8575
metab_3069	pos	730.5006	9.5594	0.4849	0.2465	0.5016	0.6243
metab_7125	neg	730.5043	9.5979	0.4542	-0.4145	0.2785	0.4091
metab_3672	pos	730.5364	10.0088	0.5571	0.6842	0.1100	0.2035
metab_3623	pos	730.5365	10.3632	0.6427	0.9627	0.2245	0.3477
metab_6907	neg	731.2797	1.4568	1.1703	-1.2146	0.0093	0.0379
metab_11408	neg	731.4214	9.6316	1.9036	-4.4509	0.0001	0.0018
metab_2992	pos	731.4833	9.0397	0.6605	1.7505	0.2210	0.3433
metab_10468	neg	731.4851	9.3494	0.1391	0.4771	0.7524	0.8225
metab_10559	neg	731.4864	9.7965	0.6847	-0.7120	0.0827	0.1690
metab_7333	neg	731.5328	8.4972	0.3720	1.0029	0.5260	0.6429
metab_14822	neg	732.2130	0.5991	0.3264	1.5801	0.4099	0.5370
metab_10626	neg	732.4889	10.1910	0.9819	1.7012	0.0139	0.0493
metab_3084	pos	732.5160	9.6823	0.3857	1.1085	0.5012	0.6240
metab_3781	pos	732.5598	9.6044	0.7397	0.9470	0.1810	0.2953
metab_9777	neg	733.3862	6.6157	0.9243	1.7230	0.0284	0.0801
metab_13809	neg	734.3708	2.1956	1.7923	13.4898	0.0000	0.0013
metab_11207	neg	734.4633	10.5844	0.5135	-0.3708	0.0462	0.1115
metab_7136	neg	734.6081	9.8947	0.6096	-3.5818	0.3662	0.4982
metab_12267	neg	735.2343	7.1437	1.1586	-1.6140	0.0159	0.0542
metab_12541	neg	735.4021	6.1644	0.2939	-0.2407	0.2395	0.3656
metab_9917	neg	735.4024	7.2069	0.5797	-1.4370	0.2863	0.4178
metab_7274	neg	735.5066	9.3661	0.8182	-0.3042	0.1405	0.2493
metab_1954	pos	736.3801	2.6402	1.0562	13.6545	0.0191	0.0563
metab_1830	pos	736.3819	2.1897	1.6958	15.8464	0.0002	0.0025
metab_10682	neg	736.4775	10.9840	0.6537	0.8572	0.0149	0.0516

metab_94	pos	736.4894	8.2143	0.3049	-1.1368	0.6737	0.7701
metab_3174	pos	736.5378	10.2870	0.5014	-0.4508	0.2769	0.4055
metab_7193	neg	736.8297	14.0282	0.3787	-0.1682	0.0936	0.1848
metab_13346	neg	737.3582	3.1797	1.1195	-2.7189	0.0647	0.1419
metab_3140	pos	737.4741	9.9935	0.3347	0.6705	0.4128	0.5454
metab_10569	neg	737.4776	9.8620	1.2457	-1.6821	0.0000	0.0004
metab_10499	neg	737.5222	9.5314	0.6971	-0.7063	0.1108	0.2093
metab_9843	neg	738.1960	6.9354	0.4423	0.2640	0.4375	0.5614
metab_9985	neg	738.2574	7.5397	1.5911	-4.9901	0.0026	0.0157
metab_827	pos	738.5053	9.2968	0.3804	-1.2973	0.3596	0.4936
metab_7226	neg	738.5092	10.1082	0.3842	-0.3178	0.2209	0.3448
metab_1424	pos	739.1816	0.9761	1.2712	2.4234	0.0192	0.0565
metab_5147	pos	739.3692	3.1799	1.2428	-3.8119	0.0684	0.1437
metab_10668	neg	739.4563	10.5844	0.6008	-0.6101	0.1102	0.2084
metab_7236	neg	739.4924	9.9433	1.3234	-3.1257	0.0255	0.0743
metab_11450	neg	739.5130	9.5147	0.8944	-0.7752	0.0024	0.0150
metab_10558	neg	739.5521	9.7965	1.5992	3.5218	0.0000	0.0012
metab_9841	neg	740.3644	6.9354	0.5374	0.3928	0.3544	0.4874
metab_875	pos	740.5211	10.1175	0.3212	-0.4881	0.2267	0.3499
metab_10644	neg	740.5237	10.3524	0.2669	0.3828	0.2757	0.4059
metab_11378	neg	740.5243	9.7470	0.5124	-0.0696	0.1357	0.2427
metab_10111	neg	741.4360	8.0069	0.4126	-0.1484	0.5245	0.6420
metab_10399	neg	741.4726	9.0713	0.8041	-0.2797	0.1192	0.2204
metab_947	pos	741.5244	9.5283	0.4974	-0.8223	0.1243	0.2230
metab_3624	pos	742.5355	10.3632	0.3378	0.1364	0.1115	0.2053
metab_10671	neg	742.5400	10.7190	0.1793	-0.1264	0.6740	0.7644
metab_10640	neg	742.5417	10.3194	0.5237	-0.6603	0.1561	0.2693
metab_10520	neg	742.5475	9.6316	0.6885	0.9510	0.0446	0.1089
metab_8749	neg	743.3607	2.3855	1.2704	15.8842	0.0082	0.0346
metab_10193	neg	743.3960	8.3209	0.8817	-0.9901	0.0572	0.1296
metab_7093	neg	743.4516	8.7031	0.6801	-1.2794	0.3468	0.4797
metab_12057	neg	743.4518	7.8352	0.9416	-2.0948	0.2276	0.3526
metab_11404	neg	743.4880	9.6477	1.7813	-2.7299	0.0056	0.0266
metab_10604	neg	743.5081	10.0264	0.8723	-2.7476	0.3001	0.4303
metab_10697	neg	743.6168	11.4384	1.6565	-3.1791	0.0001	0.0018
metab_3025	pos	744.4798	9.2354	1.2285	1.8234	0.0406	0.0976
metab_10622	neg	744.4836	10.1575	1.0854	1.7626	0.0026	0.0155
metab_7198	neg	744.4836	14.0282	1.2752	2.2244	0.0007	0.0068
metab_12046	neg	744.4904	7.8822	0.8219	-1.8573	0.2807	0.4113
metab_7237	neg	744.5283	9.9274	0.3032	0.5063	0.3738	0.5048
metab_3652	pos	744.5523	10.1647	1.3147	1.8078	0.0000	0.0010
metab_11204	neg	744.5558	10.6182	1.6444	3.3019	0.0227	0.0687
metab_855	pos	744.5603	9.7586	0.5805	0.4781	0.0493	0.1130
metab_528	pos	745.3715	2.3908	2.6832	17.3486	0.0000	0.0001
metab_10216	neg	745.4671	8.4178	0.2688	-0.4816	0.4867	0.6085
metab_11320	neg	745.5050	9.9606	1.3027	-2.0091	0.0002	0.0028
metab_832	pos	745.5195	9.3891	0.3493	0.8204	0.5212	0.6428
metab_11437	neg	745.5243	9.5478	1.8207	3.5983	0.0105	0.0409

metab_10702	neg	745.6326	12.1668	0.0387	-2.2176	0.9320	0.9560
metab_3520	pos	746.4939	14.0228	1.0800	3.4885	0.0096	0.0350
metab_878	pos	746.4952	10.1647	1.3168	2.0807	0.0009	0.0071
metab_11217	neg	746.4978	10.5015	1.3111	2.5596	0.0006	0.0061
metab_11508	neg	746.4988	9.2834	0.1641	0.2118	0.8487	0.8950
metab_10181	neg	746.5056	8.2897	0.0571	0.9893	0.9045	0.9366
metab_3615	pos	746.5663	10.4552	1.7608	3.4326	0.0000	0.0001
metab_3746	pos	746.5755	9.7586	0.0762	0.0992	0.7840	0.8551
metab_10329	neg	747.4816	8.7488	0.3118	0.3489	0.6056	0.7095
metab_10182	neg	748.4285	8.2897	0.5090	2.9113	0.6002	0.7051
metab_3188	pos	748.5090	10.4865	2.2798	4.9847	0.0006	0.0056
metab_104	pos	748.5104	9.2658	0.4083	-0.3026	0.4894	0.6143
metab_10610	neg	748.5151	10.0749	0.1873	0.0694	0.5158	0.6342
metab_11812	neg	748.5225	8.5604	0.6278	1.0800	0.3725	0.5037
metab_11421	neg	749.4313	9.5979	1.0148	-1.4744	0.0740	0.1557
metab_11403	neg	750.4573	9.6477	0.0496	1.1158	0.9566	0.9715
metab_10233	neg	750.4938	8.4972	1.5922	3.2942	0.0780	0.1624
metab_893	pos	750.5530	10.2870	0.4407	-0.3674	0.0276	0.0742
metab_13133	neg	751.3907	3.7658	0.6666	-0.0119	0.1957	0.3160
metab_9617	neg	752.3635	5.7259	0.6488	1.3635	0.2311	0.3564
metab_1865	pos	752.3791	2.3133	2.2196	6.7537	0.0000	0.0011
metab_10562	neg	752.4878	9.8294	0.6937	0.7644	0.0423	0.1050
metab_10458	neg	752.4880	9.3000	0.0843	-0.2254	0.8967	0.9310
metab_4383	pos	752.5135	6.8886	0.4410	0.3835	0.1605	0.2691
metab_1232	pos	753.2450	0.5140	1.5650	5.6331	0.0002	0.0026
metab_9819	neg	753.3511	6.8411	1.5859	4.8340	0.0002	0.0031
metab_10556	neg	754.4704	9.7804	0.9484	-1.0297	0.0245	0.0725
metab_3844	pos	754.5003	9.2968	0.5330	-0.5241	0.3765	0.5098
metab_11759	neg	754.5036	8.6874	1.2684	-2.7272	0.0309	0.0846
metab_10455	neg	754.5041	9.2834	0.4719	-1.1293	0.2760	0.4063
metab_3110	pos	754.5359	9.8049	0.3044	-0.6038	0.4780	0.6048
metab_3666	pos	754.5373	10.0875	0.2580	-0.6123	0.6363	0.7405
metab_10528	neg	755.4513	9.6477	0.0089	0.0200	0.9934	0.9949
metab_10373	neg	755.4862	8.9903	1.2820	-4.9429	0.1250	0.2282
metab_10545	neg	755.6054	9.7312	1.1337	5.1860	0.0284	0.0800
metab_10597	neg	756.4834	9.9929	0.8757	-0.5182	0.0220	0.0672
metab_842	pos	756.5158	9.5438	0.1111	-0.6344	0.8199	0.8819
metab_11439	neg	756.5181	9.5478	0.2262	0.0090	0.6463	0.7426
metab_3673	pos	756.5519	10.0088	0.4003	0.6478	0.2160	0.3376
metab_11247	neg	756.5633	10.2707	1.1748	-3.0079	0.0814	0.1674
metab_892	pos	756.5959	10.5161	0.2250	-0.1390	0.4756	0.6025
metab_12328	neg	757.3416	6.9525	0.5047	3.3534	0.3079	0.4389
metab_9810	neg	757.3421	6.8093	0.6336	3.3867	0.2684	0.3984
metab_7002	neg	757.3749	5.9705	0.0405	0.1895	0.9463	0.9649
metab_7239	neg	757.6209	9.8620	0.9784	4.4390	0.0753	0.1576
metab_11252	neg	758.4634	10.2384	0.6167	-0.3582	0.0244	0.0723
metab_11514	neg	758.4638	9.2507	1.7788	3.4409	0.0022	0.0140
metab_3818	pos	758.4953	9.4207	0.2985	-0.4889	0.3768	0.5100

metab_3206	pos	758.4963	10.8080	0.7781	-0.8752	0.1239	0.2225
metab_11238	neg	758.4988	10.3355	0.4959	-0.0544	0.0476	0.1138
metab_11096	neg	758.4989	14.0282	0.3594	0.0409	0.1206	0.2222
metab_3037	pos	758.5293	9.3117	0.2402	-0.0982	0.6702	0.7675
metab_7230	neg	758.5436	10.0593	0.1578	0.4800	0.7209	0.7999
metab_3628	pos	758.5678	10.3169	0.2269	-0.0501	0.4102	0.5432
metab_11464	neg	758.5794	9.4647	1.8523	2.6763	0.0320	0.0865
metab_9891	neg	759.4461	7.0959	0.3510	-1.2911	0.6464	0.7426
metab_10497	neg	759.5394	9.5314	1.0862	2.8191	0.1752	0.2921
metab_14284	neg	760.3445	1.5012	1.3785	4.7285	0.0064	0.0290
metab_11517	neg	760.4784	9.2507	0.9865	1.5243	0.0429	0.1060
metab_3626	pos	760.5107	10.3480	0.2121	-0.0515	0.4433	0.5739
metab_7151	neg	760.5121	10.7023	0.7254	1.4712	0.0189	0.0606
metab_3842	pos	760.5471	9.3117	1.1875	1.1837	0.0363	0.0898
metab_7275	neg	760.5573	9.3329	0.6792	1.4286	0.1316	0.2369
metab_3192	pos	760.5819	10.6239	0.7985	1.1068	0.0166	0.0508
metab_11411	neg	760.5956	9.6316	1.6613	2.8029	0.0054	0.0258
metab_12298	neg	761.4620	7.0469	0.8850	-1.5130	0.1958	0.3162
metab_11587	neg	761.4985	9.0872	1.0405	-2.8477	0.1123	0.2115
metab_11329	neg	761.5184	9.9274	0.5285	0.7458	0.1293	0.2340
metab_11341	neg	761.5496	9.8782	1.0743	2.3860	0.0112	0.0426
metab_1598	pos	762.3556	1.5004	0.9951	3.2841	0.0520	0.1173
metab_9694	neg	762.4020	6.1644	0.3528	-0.3622	0.2297	0.3548
metab_3048	pos	762.4900	9.3891	0.5657	3.4484	0.6482	0.7502
metab_10442	neg	762.5017	9.2025	0.0923	0.6166	0.9113	0.9415
metab_7950	neg	763.1924	0.6551	0.3757	1.0142	0.2949	0.4254
metab_10065	neg	763.3225	7.8507	0.9482	-1.2217	0.1298	0.2346
metab_10017	neg	763.3225	7.6793	0.5427	-0.4961	0.2542	0.3826
metab_13034	neg	763.4099	4.0859	1.1658	-2.2992	0.0590	0.1325
metab_11216	neg	763.4559	10.5015	0.7006	-0.7499	0.0491	0.1162
metab_11248	neg	763.4587	10.2548	0.9513	-1.3269	0.0335	0.0894
metab_3599	pos	763.5295	10.6845	1.0598	2.0834	0.0038	0.0180
metab_854	pos	763.5462	9.8049	1.4525	2.1021	0.0050	0.0220
metab_3044	pos	764.5045	9.3736	1.3500	13.4254	0.0609	0.1315
metab_10328	neg	764.5098	8.7488	0.3652	0.2801	0.5650	0.6761
metab_3634	pos	764.5719	10.2870	0.5257	-0.4134	0.0017	0.0105
metab_8138	neg	765.2094	1.2377	1.5959	-4.8525	0.0137	0.0488
metab_9381	neg	765.3528	4.4888	2.0431	-5.4842	0.0001	0.0017
metab_3241	pos	765.6080	12.4065	0.3597	-0.1292	0.6263	0.7320
metab_10549	neg	766.5033	9.7470	1.0337	-2.0847	0.0209	0.0650
metab_9160	neg	767.2946	3.5978	0.7677	1.6357	0.0326	0.0877
metab_13720	neg	768.1844	2.3704	1.6481	-2.8769	0.0019	0.0129
metab_10170	neg	768.4828	8.2409	0.1472	-1.0145	0.8307	0.8813
metab_866	pos	768.5526	9.9307	0.9584	0.9150	0.0005	0.0048
metab_11244	neg	768.5542	10.2875	0.9828	1.3826	0.0012	0.0098
metab_11200	neg	768.5559	10.6686	0.9750	1.4320	0.0015	0.0108
metab_13374	neg	769.2742	3.1115	0.7427	0.3338	0.1808	0.2989
metab_11702	neg	769.4655	8.7191	0.9242	2.1141	0.0893	0.1789

metab_7111	neg	769.4671	9.0872	0.2840	0.2359	0.5093	0.6287
metab_10533	neg	769.5034	9.6814	1.3356	-2.0807	0.0015	0.0112
metab_11361	neg	769.5621	9.7965	2.5635	14.8195	0.0000	0.0000
metab_8849	neg	770.2002	2.6357	1.9371	-15.3516	0.0017	0.0120
metab_7077	neg	770.4989	8.2099	0.3569	-0.8308	0.5480	0.6603
metab_11625	neg	770.4990	8.9736	0.2407	0.0307	0.6031	0.7074
metab_5171	pos	771.2851	3.1032	0.7261	-0.3943	0.2183	0.3404
metab_10666	neg	771.3923	10.5679	0.6676	-0.5497	0.0112	0.0427
metab_3763	pos	771.5146	9.6823	1.9130	-3.8071	0.0008	0.0064
metab_10582	neg	771.5183	9.9274	1.0011	-1.0298	0.0020	0.0133
metab_11338	neg	771.5783	9.8947	1.8508	4.8369	0.0002	0.0033
metab_7266	neg	772.4798	9.4156	0.3469	0.4586	0.3603	0.4931
metab_11594	neg	772.4798	9.0713	0.5702	-0.3834	0.1985	0.3191
metab_7240	neg	772.4928	9.8620	0.7213	-1.0312	0.0530	0.1225
metab_10628	neg	772.5016	10.2070	0.4875	-0.2020	0.1421	0.2514
metab_2980	pos	772.5104	8.9656	0.5182	-0.1904	0.3596	0.4936
metab_3873	pos	772.5444	9.1605	0.0067	-1.2366	0.9408	0.9632
metab_3808	pos	772.5457	9.4825	0.0478	-1.0799	0.9683	0.9808
metab_11012	neg	772.5593	14.0771	0.4120	-0.2504	0.1819	0.3000
metab_6651	neg	772.5594	10.2070	0.3889	-0.0396	0.1207	0.2222
metab_3614	pos	772.5830	10.4704	1.4699	2.2108	0.0000	0.0005
metab_10627	neg	773.1305	10.1910	0.3093	0.2137	0.3782	0.5082
metab_5995	pos	773.1657	1.2585	0.8736	3.2560	0.0117	0.0400
metab_11186	neg	773.4070	10.9840	0.6094	0.9170	0.0378	0.0969
metab_11543	neg	773.5147	9.1693	0.8258	0.9552	0.0281	0.0795
metab_11278	neg	773.5347	10.1250	0.2254	-0.1404	0.5747	0.6843
metab_12521	neg	773.5425	6.2456	0.8198	-3.3892	0.0259	0.0754
metab_10599	neg	773.5945	9.9929	0.0177	0.1978	0.9387	0.9601
metab_3165	pos	773.6230	10.2085	0.9897	-1.1850	0.0290	0.0766
metab_11489	neg	774.4580	9.3494	0.6514	-1.2766	0.1773	0.2947
metab_3053	pos	774.4923	9.4357	1.2298	1.9343	0.0081	0.0310
metab_10479	neg	774.4937	9.4322	0.0070	0.0866	0.9577	0.9719
metab_6640	neg	774.4940	14.0282	0.1414	0.0513	0.6695	0.7616
metab_11184	neg	774.4941	11.0007	0.0329	0.4874	0.9668	0.9768
metab_3718	pos	774.5067	9.8362	0.1996	-0.0526	0.8272	0.8863
metab_951	pos	774.5619	9.4825	0.1207	-0.8289	0.7648	0.8400
metab_3207	pos	774.5976	10.8232	0.8921	1.4695	0.0221	0.0627
metab_9818	neg	775.3848	6.8411	0.2576	0.6053	0.7677	0.8337
metab_11289	neg	775.5344	10.0749	0.3767	0.7008	0.3666	0.4985
metab_12376	neg	775.5742	6.8411	0.8376	1.9722	0.1754	0.2925
metab_13243	neg	776.3449	3.4800	1.9269	4.1902	0.0686	0.1479
metab_10360	neg	776.4732	8.9252	2.0278	5.3051	0.0004	0.0048
metab_3790	pos	776.5061	9.5594	0.7452	2.4011	0.2052	0.3247
metab_6636	neg	776.5536	9.5979	0.6744	1.2625	0.1274	0.2315
metab_9763	neg	777.4565	6.5346	0.6217	-2.2753	0.4163	0.5423
metab_10655	neg	777.5063	10.3849	0.2011	-0.2460	0.5290	0.6457
metab_10647	neg	777.5463	10.3524	0.3138	0.5888	0.3610	0.4938
metab_12366	neg	777.5891	6.8730	1.1556	1.7235	0.0054	0.0257

metab_11269	neg	778.5175	10.1575	1.9436	4.2604	0.0000	0.0006
metab_7251	neg	778.5252	9.6986	0.7031	1.0143	0.0203	0.0637
metab_13359	neg	779.4055	3.1455	0.6212	-4.1356	0.4814	0.6037
metab_7271	neg	779.4493	9.3661	0.8764	-1.8168	0.0711	0.1515
metab_11194	neg	779.5200	10.7359	1.3397	2.3164	0.0056	0.0266
metab_6615	neg	779.9636	0.5286	1.7614	3.2815	0.0005	0.0052
metab_970	pos	780.4788	8.0123	0.9607	-2.1921	0.0926	0.1793
metab_11205	neg	780.4807	10.6182	0.6945	-0.3017	0.0038	0.0202
metab_11088	neg	780.4810	14.0282	0.4586	-0.0631	0.0587	0.1321
metab_11357	neg	780.4830	9.8294	1.2464	-0.8812	0.0627	0.1387
metab_8502	neg	780.4875	1.8205	1.2756	-1.9279	0.0230	0.0694
metab_3852	pos	780.5151	9.2658	1.1781	1.4635	0.0000	0.0004
metab_3693	pos	780.5157	9.9307	0.8920	0.8502	0.0000	0.0010
metab_3575	pos	780.5165	11.1144	0.2197	-0.5275	0.5790	0.6922
metab_11367	neg	780.5205	9.7965	0.6743	-1.2175	0.0962	0.1889
metab_959	pos	780.5507	9.2206	0.2280	-1.4441	0.5761	0.6906
metab_861	pos	780.5509	9.8362	0.7010	0.5878	0.0281	0.0752
metab_6760	neg	781.2030	0.6691	0.0420	0.3643	0.6958	0.7818
metab_13326	neg	781.3477	3.2301	1.2659	-1.6466	0.0078	0.0333
metab_10771	neg	781.4843	14.0282	0.4683	-0.0602	0.0586	0.1320
metab_10530	neg	781.5460	9.6644	0.0472	0.3189	0.9434	0.9631
metab_3147	pos	781.9841	10.0715	0.1659	-0.1968	0.6385	0.7423
metab_13441	neg	782.2694	2.9588	1.1589	1.0537	0.0461	0.1113
metab_3267	pos	782.4916	14.0074	0.4735	-0.2510	0.2224	0.3450
metab_3577	pos	782.4926	11.0839	0.4308	-0.2615	0.1120	0.2058
metab_3013	pos	782.4947	9.1451	1.1775	-2.0129	0.0044	0.0201
metab_10603	neg	782.4988	10.0264	1.2379	-1.3476	0.0000	0.0005
metab_7203	neg	782.4990	14.0282	1.1094	-1.1678	0.0001	0.0019
metab_3736	pos	782.5289	9.7899	0.2067	-0.7328	0.5935	0.7045
metab_11515	neg	782.5328	9.2507	0.0724	-0.2683	0.7839	0.8459
metab_873	pos	782.5676	10.0403	0.1812	-0.0515	0.4414	0.5721
metab_14941	neg	783.1143	0.5286	1.9104	3.8695	0.0269	0.0771
metab_3669	pos	783.1546	10.0403	0.1432	-0.0844	0.6244	0.7308
metab_10517	neg	783.4823	9.6147	1.1593	-2.2798	0.1338	0.2402
metab_14355	neg	784.1513	1.4427	1.0425	1.7566	0.0064	0.0292
metab_2030	pos	784.2801	2.9652	1.1336	0.5804	0.0573	0.1259
metab_10115	neg	784.4783	8.0228	0.5628	1.9714	0.3479	0.4808
metab_910	pos	784.5093	14.0074	1.2350	-1.5775	0.0004	0.0045
metab_3143	pos	784.5108	10.0088	1.2422	-1.6201	0.0000	0.0003
metab_11241	neg	784.5119	10.3194	0.6537	-0.1292	0.0342	0.0903
metab_948	pos	784.5431	9.5137	0.0348	-0.0188	0.9561	0.9722
metab_11250	neg	785.4638	10.2384	0.2669	-0.5757	0.6376	0.7353
metab_10348	neg	785.4978	8.8449	1.0956	-2.1042	0.0661	0.1439
metab_10759	neg	785.5150	14.0282	0.7381	-0.2276	0.0175	0.0575
metab_3747	pos	785.5427	9.7435	0.9594	-3.3960	0.1788	0.2926
metab_7259	neg	785.5526	9.4812	1.1141	1.9963	0.0015	0.0109
metab_3172	pos	785.5861	10.2870	0.7244	0.7585	0.0115	0.0395
metab_7317	neg	785.6423	8.6728	1.7687	-3.1848	0.0015	0.0111

metab_3175	pos	786.5234	10.3026	0.7304	-0.5166	0.0313	0.0809
metab_11198	neg	786.5290	10.7190	0.6625	-0.2210	0.0545	0.1250
metab_7139	neg	786.5301	9.9767	0.6591	0.8941	0.0312	0.0852
metab_953	pos	786.5615	9.3590	0.5357	0.6718	0.4675	0.5948
metab_10646	neg	786.5744	10.3524	0.6099	1.2751	0.1626	0.2775
metab_889	pos	786.5975	10.6538	0.3293	0.3181	0.2858	0.4162
metab_10525	neg	787.3875	9.6477	0.6074	-0.2917	0.3064	0.4374
metab_7669	neg	788.0308	0.5126	1.5065	2.3744	0.0005	0.0056
metab_10204	neg	788.5095	8.3687	0.2583	-0.1643	0.6834	0.7726
metab_11585	neg	788.5150	9.0872	0.1612	-0.4933	0.7081	0.7908
metab_3597	pos	788.5413	10.7001	1.2749	-1.0731	0.0218	0.0621
metab_3089	pos	788.5423	9.6968	1.5947	-3.4096	0.0271	0.0731
metab_11380	neg	788.5533	9.7470	0.5129	0.6921	0.1270	0.2310
metab_3772	pos	788.5753	9.6358	0.3818	-0.5046	0.5226	0.6442
metab_3215	pos	788.6129	11.0382	0.5233	-0.4303	0.1820	0.2961
metab_6110	pos	789.1598	1.0883	1.0172	3.0693	0.0057	0.0240
metab_248	pos	789.1604	0.8640	0.7190	-1.6128	0.0594	0.1291
metab_11259	neg	789.5495	10.2070	0.1136	0.1850	0.5678	0.6789
metab_9696	neg	790.3425	6.1644	0.1408	-0.1654	0.6106	0.7139
metab_10422	neg	790.4894	9.1200	0.8130	0.9026	0.0326	0.0877
metab_774	pos	790.5207	8.3912	0.2636	-0.7146	0.7417	0.8225
metab_10310	neg	790.5227	8.7191	1.0138	1.0205	0.1351	0.2420
metab_3867	pos	790.5565	9.1898	0.3906	-1.0149	0.6222	0.7293
metab_11840	neg	790.5614	8.4972	1.4240	3.2935	0.0019	0.0128
metab_7250	neg	790.5688	9.7639	0.1910	0.4462	0.5438	0.6572
metab_10514	neg	791.5292	9.5979	2.0433	3.5313	0.0281	0.0794
metab_3884	pos	792.5003	9.1140	1.4198	1.7035	0.0059	0.0246
metab_7216	neg	792.5321	10.3524	0.2401	0.1958	0.4406	0.5645
metab_10232	neg	792.5765	8.4972	1.9862	6.2969	0.0001	0.0025
metab_10513	neg	793.5448	9.5979	1.1407	1.9792	0.0439	0.1076
metab_11900	neg	794.4833	8.2897	2.0293	4.0822	0.0016	0.0115
metab_11471	neg	794.5202	9.4322	1.2692	2.6932	0.0058	0.0270
metab_10631	neg	795.3924	10.2384	0.7783	-0.7818	0.0076	0.0328
metab_10175	neg	795.4443	8.2577	1.1109	-2.3746	0.2639	0.3939
metab_9595	neg	795.4676	5.6128	0.6934	-1.9353	0.2973	0.4279
metab_11303	neg	795.5191	10.0090	0.3901	-0.2095	0.1250	0.2281
metab_745	pos	795.5834	7.8389	0.0498	0.3483	0.9946	0.9967
metab_6711	neg	795.9681	0.5286	0.9500	2.5054	0.0553	0.1265
metab_9013	neg	796.3710	3.0604	0.6505	13.7950	0.1321	0.2378
metab_13143	neg	796.3714	3.7488	1.2601	11.9812	0.0299	0.0825
metab_11482	neg	796.4417	9.3661	1.1437	-2.0420	0.0205	0.0642
metab_7120	neg	796.4773	9.4322	0.4212	-0.4477	0.2631	0.3932
metab_11547	neg	796.4792	9.1522	0.2992	-0.3027	0.2677	0.3981
metab_2990	pos	796.5111	9.0254	0.5501	-0.1340	0.3734	0.5073
metab_10358	neg	796.5138	8.9252	0.0288	-0.9732	0.9831	0.9882
metab_11511	neg	796.5141	9.2674	0.8327	0.9251	0.0077	0.0330
metab_4388	pos	796.5395	6.8728	0.5864	0.4780	0.1533	0.2603
metab_3861	pos	796.5462	9.2206	0.2260	-0.8035	0.7133	0.8007

metab_11212	neg	797.4057	10.5343	0.2124	0.0297	0.3735	0.5047
metab_2055	pos	798.3814	3.0572	1.8604	12.9962	0.0136	0.0444
metab_7137	neg	798.4488	9.9274	1.9329	0.6647	0.0689	0.1482
metab_762	pos	798.4890	8.1998	0.0823	0.9763	0.9376	0.9611
metab_7289	neg	798.4938	9.1364	0.9043	-1.3012	0.0434	0.1069
metab_3815	pos	798.5624	9.4506	0.1481	-0.9504	0.6905	0.7830
metab_3187	pos	798.5975	10.4397	1.1282	1.4050	0.0051	0.0223
metab_12180	neg	799.4497	7.4927	0.2007	0.1295	0.6059	0.7096
metab_12031	neg	799.4498	7.9292	0.2778	0.3898	0.5006	0.6211
metab_10515	neg	799.5144	9.6147	1.2650	-2.6273	0.0123	0.0455
metab_2710	pos	800.4494	7.2014	0.0493	0.0596	0.8304	0.8886
metab_103	pos	800.5061	9.3278	0.5221	-0.2883	0.4189	0.5506
metab_7140	neg	800.5460	9.9929	0.4439	1.0362	0.1996	0.3203
metab_7150	neg	800.5902	10.5180	0.2422	0.0901	0.3880	0.5170
metab_10560	neg	801.0828	9.7965	0.0966	0.1283	0.7019	0.7861
metab_7141	neg	801.0830	10.0264	0.1533	-0.0099	0.5295	0.6460
metab_10058	neg	801.4931	7.8193	1.0117	-2.6711	0.0809	0.1667
metab_11277	neg	801.5073	10.1250	0.7165	-0.8615	0.0768	0.1602
metab_3812	pos	801.5798	9.4667	0.0815	-0.7308	0.8868	0.9283
metab_6660	neg	802.4687	8.3209	0.8110	-0.8453	0.0473	0.1132
metab_944	pos	802.5239	9.5749	2.5860	-1.5686	0.0332	0.0845
metab_10611	neg	802.5335	10.0749	0.2256	0.2400	0.5449	0.6580
metab_7147	neg	802.5618	10.3194	0.3461	0.4216	0.2547	0.3830
metab_3755	pos	802.5894	9.7129	0.1208	-0.6909	0.7935	0.8618
metab_11325	neg	803.5245	9.9274	0.8111	1.5171	0.1168	0.2170
metab_11541	neg	803.5680	9.1693	2.0872	7.6358	0.0000	0.0003
metab_770	pos	804.4806	8.3322	0.6521	-0.8226	0.1197	0.2165
metab_11089	neg	804.4809	14.0282	1.1248	-1.1465	0.0000	0.0008
metab_10602	neg	804.4811	10.0090	2.5499	-5.3226	0.0002	0.0028
metab_7327	neg	804.4839	8.6406	1.3101	-1.8291	0.0059	0.0274
metab_11846	neg	804.5036	8.4667	1.2942	3.0287	0.0132	0.0477
metab_10461	neg	804.5418	9.3165	0.9971	1.2003	0.0060	0.0275
metab_7213	neg	804.5765	10.6351	0.7777	1.4228	0.0289	0.0809
metab_11085	neg	805.4845	14.0282	1.2255	-1.3964	0.0000	0.0005
metab_11465	neg	805.4894	9.4647	1.5314	-2.6935	0.0002	0.0031
metab_10623	neg	805.5369	10.1575	1.4779	2.5970	0.0001	0.0023
metab_10550	neg	805.5447	9.7470	0.4967	0.6571	0.1779	0.2955
metab_11412	neg	805.5449	9.6316	0.6926	0.9974	0.0278	0.0791
metab_3550	pos	806.4903	12.4360	1.4711	-2.1472	0.0002	0.0030
metab_3246	pos	806.4905	12.7317	1.5669	-2.4798	0.0003	0.0033
metab_10761	neg	806.4909	14.0282	0.7974	-0.4781	0.0077	0.0330
metab_3225	pos	806.4916	11.2827	1.3180	-1.7420	0.0001	0.0014
metab_2932	pos	806.4942	8.6577	1.8347	-3.5123	0.0001	0.0017
metab_10613	neg	806.4950	10.1082	0.9609	-0.9651	0.0003	0.0037
metab_6634	neg	806.4984	9.2185	0.7473	0.9472	0.0813	0.1672
metab_11318	neg	806.5210	9.9606	1.6217	-2.6446	0.0001	0.0025
metab_3681	pos	806.5299	9.9470	2.4317	-6.1834	0.0000	0.0005
metab_3632	pos	806.5644	10.2870	0.8117	0.9641	0.0161	0.0496

metab_6390	pos	807.1547	0.5703	0.2036	-0.2684	0.7011	0.7915
metab_11373	neg	807.5592	9.7639	0.5394	0.9667	0.1026	0.1977
metab_11820	neg	808.4994	8.5455	0.9339	1.1355	0.0196	0.0619
metab_11388	neg	808.5191	9.7152	0.7870	-1.3600	0.1307	0.2359
metab_10512	neg	809.4905	9.5979	1.0941	-1.2447	0.0040	0.0208
metab_2909	pos	810.5101	8.5554	1.4190	1.7136	0.0035	0.0174
metab_11389	neg	810.5294	9.7152	0.1690	-0.0278	0.4963	0.6172
metab_11297	neg	810.5299	10.0432	1.1732	-2.3103	0.0129	0.0469
metab_881	pos	810.5992	10.2870	0.0100	-0.0729	0.8092	0.8738
metab_11484	neg	811.3871	9.3661	0.8292	-1.5172	0.1248	0.2278
metab_7115	neg	811.5140	9.1364	0.9502	1.7434	0.0477	0.1139
metab_11258	neg	811.5701	10.2070	0.3849	-0.0149	0.2438	0.3699
metab_5852	pos	812.1763	1.5284	1.3428	2.4068	0.0388	0.0944
metab_7989	neg	812.2690	0.7958	0.7810	1.7071	0.0237	0.0707
metab_14754	neg	812.2693	0.6691	0.8140	1.6803	0.0280	0.0792
metab_11286	neg	812.4622	10.0749	0.3517	-1.3369	0.7881	0.8484
metab_10118	neg	812.4737	8.0532	0.9566	-1.5481	0.1062	0.2030
metab_11611	neg	812.5098	9.0230	0.8870	1.0994	0.0423	0.1050
metab_3903	pos	812.5406	9.0254	0.3169	-1.0224	0.6704	0.7676
metab_759	pos	812.5411	8.1560	0.0413	-1.8124	0.8940	0.9319
metab_11328	neg	812.5461	9.9274	0.7866	1.1261	0.0244	0.0723
metab_10683	neg	812.5732	11.0007	1.7039	-3.6493	0.0006	0.0064
metab_3603	pos	812.6140	10.5923	0.5160	0.3874	0.1609	0.2696
metab_7892	neg	813.1932	0.5711	0.6247	-0.8839	0.1500	0.2615
metab_10521	neg	813.4009	9.6316	0.7014	-0.4165	0.2949	0.4254
metab_11396	neg	813.5304	9.6814	0.4716	-0.4513	0.4116	0.5385
metab_9678	neg	814.4333	6.0831	0.0746	0.9685	0.9611	0.9736
metab_10654	neg	814.4348	10.3849	0.7053	-0.8166	0.0274	0.0783
metab_11996	neg	814.4887	8.0228	1.3307	-1.5502	0.0677	0.1463
metab_11871	neg	814.4889	8.3858	0.6103	1.4551	0.4433	0.5672
metab_11542	neg	814.5219	9.1693	0.6528	0.7450	0.2128	0.3357
metab_3967	pos	814.5570	8.7161	0.1354	-0.8317	0.8414	0.8966
metab_93	pos	814.5570	8.1273	0.8554	-2.1737	0.1458	0.2509
metab_12147	neg	814.5615	7.5855	1.0070	-1.4957	0.1123	0.2115
metab_11267	neg	814.5632	10.1743	1.5544	2.8575	0.0000	0.0002
metab_11162	neg	814.5884	11.5850	0.7719	-2.3510	0.2076	0.3299
metab_3216	pos	814.6294	11.0382	0.7726	1.2850	0.0993	0.1884
metab_7958	neg	815.7090	0.6973	2.0159	8.3213	0.0003	0.0041
metab_4518	pos	816.4440	6.0864	0.4798	1.1677	0.5782	0.6916
metab_9768	neg	816.4482	6.5829	0.1047	0.1396	0.7495	0.8208
metab_6571	neg	816.4487	5.9705	0.2545	-0.1291	0.5071	0.6266
metab_6995	neg	816.4488	5.7420	0.2953	-0.1601	0.4318	0.5564
metab_6646	neg	816.4580	14.0117	0.8012	-0.4381	0.0075	0.0325
metab_2882	pos	816.5001	8.3912	0.6410	4.3901	0.3638	0.4973
metab_7297	neg	816.5051	8.8770	0.4823	-0.4472	0.2983	0.4288
metab_7228	neg	816.5334	10.1401	0.0087	0.1917	0.9589	0.9727
metab_805	pos	816.5716	8.8759	0.6914	0.4881	0.0754	0.1537
metab_10141	neg	816.5774	8.1153	0.1329	0.2191	0.7929	0.8515

metab_12191	neg	817.5794	7.4465	0.4334	-0.5610	0.3949	0.5231
metab_11215	neg	817.5807	10.5180	0.7323	0.9768	0.0310	0.0847
metab_7602	neg	817.9201	0.5431	1.5857	2.7801	0.0020	0.0131
metab_6713	neg	818.1436	0.5286	2.4864	8.9065	0.0000	0.0001
metab_10019	neg	818.3955	7.6793	0.8351	2.8930	0.0346	0.0911
metab_4436	pos	818.4589	6.5704	0.2044	-0.1236	0.6252	0.7312
metab_4589	pos	818.4598	5.7385	0.2842	-0.2321	0.5263	0.6474
metab_7409	neg	818.4631	6.1968	1.5772	-2.7986	0.0016	0.0112
metab_3074	pos	818.5537	9.6044	1.1682	-4.2748	0.0671	0.1418
metab_7121	neg	818.5568	9.4812	0.1451	-0.2972	0.8396	0.8880
metab_3871	pos	818.5860	9.1747	1.1396	1.3923	0.0047	0.0210
metab_14867	neg	819.1586	0.5711	0.5365	-0.2363	0.0985	0.1921
metab_10511	neg	819.5044	9.5979	0.3340	-0.2290	0.2768	0.4073
metab_4236	pos	820.4755	7.5892	0.6000	-0.6896	0.2138	0.3350
metab_3860	pos	820.5441	9.2206	0.1818	-0.9965	0.6212	0.7285
metab_2795	pos	822.4895	7.8532	1.6707	-3.1148	0.0011	0.0081
metab_7118	neg	822.4922	9.2834	0.4399	-1.3476	0.3524	0.4859
metab_11322	neg	822.5297	9.9433	1.3676	-2.6059	0.0013	0.0103
metab_12091	neg	822.5512	7.7251	0.9014	-1.0893	0.1634	0.2784
metab_3711	pos	822.5619	9.8673	0.7047	0.9072	0.0215	0.0614
metab_7119	neg	823.5551	9.3329	0.5667	1.2089	0.2073	0.3295
metab_11418	neg	824.5099	9.6147	1.3107	-2.5976	0.0314	0.0855
metab_7244	neg	824.5458	9.8459	0.4447	0.5753	0.1871	0.3059
metab_3675	pos	824.5767	9.9935	0.5218	0.6175	0.0958	0.1836
metab_11995	neg	824.5906	8.0228	1.9989	-2.8743	0.0003	0.0037
metab_7219	neg	826.4812	10.2070	0.8461	-0.7356	0.0097	0.0391
metab_7279	neg	826.5241	9.1858	0.2615	-1.1727	0.6343	0.7325
metab_10384	neg	826.5245	9.0230	1.1811	1.7424	0.0325	0.0876
metab_11298	neg	826.5619	10.0432	0.1820	0.2437	0.5040	0.6240
metab_3642	pos	826.5912	10.2252	1.0682	1.6833	0.0164	0.0505
metab_3085	pos	826.5934	9.6823	0.1228	-0.1739	0.8247	0.8843
metab_10174	neg	827.3817	8.2577	0.0204	-0.0159	0.9467	0.9650
metab_11827	neg	828.5046	8.5135	1.4011	3.0635	0.0190	0.0608
metab_11710	neg	828.5049	8.7191	1.0817	2.2129	0.0873	0.1759
metab_11246	neg	828.5762	10.2875	0.7173	1.1219	0.0246	0.0727
metab_10568	neg	828.5954	9.8620	0.1710	0.4057	0.7858	0.8470
metab_11295	neg	829.1135	10.0432	0.0863	0.1014	0.7950	0.8531
metab_7914	neg	829.2955	0.5991	1.2464	3.8064	0.0001	0.0025
metab_10453	neg	829.4886	9.2674	1.4818	-2.4831	0.0001	0.0022
metab_11434	neg	829.5250	9.5649	1.5645	-3.8432	0.0042	0.0215
metab_3092	pos	830.5303	9.7129	2.0580	-6.1917	0.0006	0.0055
metab_4206	pos	830.5519	7.7208	0.4000	-3.3821	0.5470	0.6658
metab_11486	neg	830.5545	9.3494	1.4605	3.1185	0.0110	0.0420
metab_7212	neg	830.5919	10.6351	0.2970	0.6294	0.3648	0.4968
metab_3183	pos	831.7033	10.3942	0.9863	1.4287	0.0651	0.1383
metab_10149	neg	832.4994	8.1468	0.6440	-0.7509	0.1137	0.2129
metab_11521	neg	832.5290	9.2185	0.3004	-0.5608	0.3868	0.5161
metab_11670	neg	832.5349	8.8128	0.3514	0.3352	0.6354	0.7334

metab_4151	pos	832.5655	8.0123	0.0539	-0.4627	0.9807	0.9884
metab_4088	pos	832.5673	8.2586	0.5593	-0.3191	0.3756	0.5092
metab_10684	neg	832.6079	11.0331	0.2093	0.4867	0.5254	0.6427
metab_3925	pos	833.5149	8.9367	1.5795	-3.2757	0.0082	0.0312
metab_10543	neg	833.5206	9.7312	1.3368	-1.7318	0.0000	0.0001
metab_888	pos	833.7200	10.5629	1.8010	2.8716	0.0042	0.0193
metab_4477	pos	834.4542	6.3885	1.5040	2.3352	0.0802	0.1614
metab_7021	neg	834.4590	6.8565	0.6251	-0.0956	0.2947	0.4254
metab_10389	neg	834.5524	9.0391	0.1251	-0.0320	0.8751	0.9145
metab_2928	pos	834.5818	8.6284	0.4719	0.0134	0.3274	0.4613
metab_12141	neg	834.5879	7.6009	1.1336	2.3159	0.0037	0.0197
metab_12194	neg	834.5881	7.4465	1.4478	2.9909	0.0028	0.0167
metab_11299	neg	835.0432	10.0264	0.0906	0.1833	0.7856	0.8469
metab_7127	neg	835.0434	9.7639	0.1650	0.1292	0.4787	0.6010
metab_7302	neg	835.4991	8.7640	0.4265	-0.4944	0.3682	0.4998
metab_908	pos	835.5305	9.7283	1.5281	-2.5012	0.0000	0.0003
metab_3184	pos	835.5937	10.4092	1.6608	-3.4281	0.0019	0.0114
metab_3202	pos	835.7359	10.7614	1.9208	4.2122	0.0000	0.0003
metab_2638	pos	836.4703	6.8582	0.0872	-0.0019	0.8124	0.8760
metab_4538	pos	836.4704	5.9800	0.0976	-0.0604	0.7745	0.8477
metab_11316	neg	836.5377	9.9767	0.9835	-0.8125	0.0017	0.0118
metab_806	pos	836.5389	8.8904	0.1256	-1.0700	0.8745	0.9207
metab_11459	neg	836.5450	9.4812	0.0019	-0.1360	0.9869	0.9904
metab_11498	neg	836.5669	9.3165	1.3704	4.4670	0.0012	0.0097
metab_7323	neg	836.6271	8.6874	2.1569	-3.0176	0.0007	0.0069
metab_8266	neg	837.2226	1.4427	0.4039	-0.9596	0.3591	0.4919
metab_10555	neg	837.5374	9.7639	1.1246	-1.4751	0.0011	0.0091
metab_7143	neg	838.4345	10.1082	0.3748	-0.2130	0.2231	0.3473
metab_11449	neg	838.4346	9.5147	1.5136	-2.7397	0.0047	0.0234
metab_4500	pos	838.4837	6.2071	1.4382	-2.5221	0.0110	0.0383
metab_10432	neg	838.5250	9.1364	0.5324	-2.2563	0.4154	0.5416
metab_11368	neg	838.5256	9.7804	1.8582	-4.5721	0.0262	0.0759
metab_10508	neg	838.5268	9.5811	0.8999	-1.4421	0.0215	0.0663
metab_4312	pos	838.5583	7.1560	1.1535	-1.2246	0.0804	0.1615
metab_10581	neg	838.5617	9.9274	0.6722	0.8471	0.0100	0.0397
metab_11319	neg	839.4180	9.9606	1.5515	-5.1163	0.0241	0.0716
metab_11233	neg	840.4490	10.3524	0.1614	0.3332	0.4872	0.6089
metab_10750	neg	840.4583	14.0117	1.2487	-1.1934	0.0062	0.0283
metab_10444	neg	840.5405	9.2185	0.2556	-0.3544	0.6634	0.7568
metab_3866	pos	840.5716	9.1898	0.6412	0.3388	0.2709	0.3993
metab_11166	neg	840.6038	11.4384	1.6643	-3.2262	0.0001	0.0016
metab_3585	pos	840.6454	10.9614	0.5061	-1.5319	0.4270	0.5586
metab_10035	neg	841.5356	7.7251	0.6443	-0.0082	0.2825	0.4132
metab_10675	neg	842.4637	10.7359	0.6967	-0.7080	0.1815	0.2996
metab_11528	neg	842.5566	9.2185	0.2133	-0.8185	0.5994	0.7048
metab_11158	neg	842.6193	12.1834	0.2292	-1.6805	0.7500	0.8212
metab_13664	neg	843.3463	2.4813	1.2368	14.3727	0.0190	0.0609
metab_7156	neg	843.4768	11.2769	0.7612	-0.2800	0.0067	0.0299

metab_6647	neg	843.4769	13.9952	0.7340	-0.2948	0.0272	0.0779
metab_11275	neg	843.5517	10.1401	0.0315	0.0883	0.9682	0.9775
metab_7379	neg	844.4433	7.1903	0.1267	0.2877	0.6618	0.7557
metab_10484	neg	844.5707	9.4647	0.1607	-0.1972	0.7255	0.8025
metab_10590	neg	845.4289	9.9606	1.8650	-3.7934	0.0005	0.0058
metab_11699	neg	845.5196	8.7346	1.7432	-8.1894	0.1429	0.2524
metab_11245	neg	845.5663	10.2875	0.6427	0.9744	0.0291	0.0813
metab_3665	pos	845.5764	10.0875	1.2445	-2.3397	0.0114	0.0392
metab_10537	neg	846.5301	9.6986	1.6237	-3.3427	0.0029	0.0169
metab_2671	pos	846.5465	6.9790	0.2615	-2.3077	0.5863	0.6992
metab_11369	neg	846.5837	9.7804	0.4586	0.4083	0.4654	0.5877
metab_10272	neg	846.6105	8.6093	1.8006	-4.6397	0.0000	0.0000
metab_11787	neg	847.4987	8.6239	1.3215	-1.7911	0.0219	0.0671
metab_10669	neg	847.5822	10.6518	0.1460	0.3893	0.6001	0.7051
metab_11225	neg	847.7038	10.3849	1.1510	2.0052	0.0147	0.0513
metab_7194	neg	848.4684	14.0282	0.4702	-0.0224	0.0999	0.1937
metab_9815	neg	848.4940	6.8255	1.1716	-1.5552	0.0831	0.1697
metab_10146	neg	848.5224	8.1307	1.4302	-3.8923	0.1201	0.2215
metab_4369	pos	848.5621	6.9336	0.0327	-2.5482	0.9024	0.9372
metab_9734	neg	848.5665	6.3917	0.4388	-0.1829	0.5274	0.6443
metab_12522	neg	848.5665	6.2456	0.2388	1.5277	0.6904	0.7777
metab_14137	neg	849.2228	1.6491	0.5505	1.9738	0.3628	0.4953
metab_7294	neg	849.5148	8.9417	1.2098	-1.9888	0.0214	0.0660
metab_11182	neg	849.5981	11.0331	0.0618	0.3106	0.7700	0.8356
metab_3616	pos	849.6097	10.4250	0.9245	-2.3928	0.1168	0.2124
metab_11209	neg	849.7192	10.5679	1.6813	3.0910	0.0007	0.0068
metab_8501	neg	851.2385	1.8205	0.6207	1.3940	0.3683	0.4999
metab_6941	neg	851.2907	4.0859	0.1749	0.3801	0.6496	0.7455
metab_11417	neg	852.5398	9.6147	1.2787	-2.4578	0.0046	0.0230
metab_3752	pos	852.5575	9.7283	1.5904	-2.6671	0.0000	0.0002
metab_11777	neg	852.5623	8.6568	0.6515	0.3331	0.4204	0.5465
metab_10677	neg	852.7383	10.7689	1.9603	5.0159	0.0000	0.0001
metab_602	pos	853.3016	4.0634	0.1990	0.1516	0.5545	0.6724
metab_10592	neg	853.4342	9.9606	1.3485	-3.6523	0.0704	0.1504
metab_11601	neg	853.5360	9.0391	0.7523	2.1059	0.2399	0.3660
metab_3806	pos	853.6890	9.4981	0.2222	-0.0538	0.5856	0.6991
metab_11503	neg	854.4299	9.3000	0.2966	-1.2129	0.5764	0.6856
metab_11438	neg	854.5205	9.5478	0.2378	0.3208	0.5813	0.6896
metab_11562	neg	854.5568	9.1200	1.4472	1.9979	0.0313	0.0852
metab_3801	pos	854.5899	9.5137	2.3138	-4.3664	0.0001	0.0017
metab_7960	neg	855.2034	0.7114	0.0868	0.1207	0.6857	0.7743
metab_4216	pos	855.4987	7.6776	1.9244	-6.9312	0.0022	0.0129
metab_11499	neg	855.5033	9.3165	2.8778	-7.8932	0.0000	0.0004
metab_11257	neg	855.5966	10.2070	0.3745	0.7150	0.2313	0.3565
metab_896	pos	855.7047	10.1941	0.4946	-0.3641	0.3098	0.4423
metab_9059	neg	856.3686	3.2301	2.4745	-6.1439	0.0000	0.0002
metab_10617	neg	856.4455	10.1250	1.3567	-3.8028	0.0872	0.1759
metab_10151	neg	856.5354	8.1623	0.4100	-2.4940	0.5391	0.6536

metab_11610	neg	856.5354	9.0230	0.5505	-0.2906	0.4275	0.5527
metab_11203	neg	856.6080	10.6013	0.4440	0.7236	0.2170	0.3406
metab_3773	pos	856.7078	9.6358	1.8842	3.7255	0.0002	0.0022
metab_795	pos	857.5148	8.6577	2.2940	-6.2883	0.0000	0.0004
metab_10488	neg	857.5197	9.4984	1.6231	-2.6014	0.0000	0.0000
metab_13533	neg	858.4011	2.7475	2.1467	7.5006	0.0000	0.0014
metab_11231	neg	858.4243	10.3687	0.7124	-0.2714	0.0030	0.0171
metab_7074	neg	858.5510	8.1307	0.2262	-0.6503	0.4981	0.6188
metab_11655	neg	858.5514	8.8929	0.2220	-0.2992	0.6835	0.7726
metab_6286	pos	859.1258	0.6962	0.4961	-0.3991	0.1499	0.2561
metab_950	pos	859.5304	9.4981	1.8006	-3.3918	0.0000	0.0002
metab_3153	pos	859.5943	10.1021	1.5025	-3.7792	0.0007	0.0062
metab_3608	pos	859.7354	10.5474	1.2590	1.7413	0.0435	0.1027
metab_1975	pos	860.4113	2.7497	2.4241	10.1011	0.0000	0.0003
metab_11199	neg	860.4362	10.7023	0.6604	1.5893	0.0582	0.1313
metab_10494	neg	860.5390	9.5314	1.1868	-1.6573	0.0029	0.0168
metab_10351	neg	860.5639	8.8770	0.7093	0.8273	0.0406	0.1018
metab_10629	neg	860.6094	10.2070	0.0923	0.5512	0.8600	0.9035
metab_11379	neg	861.5480	9.7470	1.1509	-1.2433	0.0038	0.0204
metab_7601	neg	861.9535	0.5431	1.1264	2.3891	0.0460	0.1112
metab_11256	neg	862.4553	10.2070	0.7595	-0.5118	0.0354	0.0925
metab_10619	neg	862.5495	10.1401	1.3850	-2.8753	0.0254	0.0742
metab_945	pos	862.5501	9.5283	1.5397	-2.6508	0.0020	0.0119
metab_10433	neg	862.5816	9.1693	1.1944	1.9440	0.0001	0.0016
metab_3054	pos	862.5930	9.4506	2.0376	-4.1522	0.0000	0.0005
metab_11312	neg	863.5581	9.9767	0.9828	-1.3573	0.0085	0.0355
metab_3742	pos	863.5600	9.7586	1.5116	-2.4828	0.0031	0.0158
metab_10245	neg	863.6263	8.5135	1.2373	2.7389	0.0340	0.0900
metab_7054	neg	864.4691	7.5702	1.1212	-1.5029	0.0221	0.0674
metab_7428	neg	865.3063	4.1364	0.7388	-0.3941	0.0172	0.0569
metab_11887	neg	865.4667	8.3209	1.0506	-1.4959	0.0145	0.0507
metab_11778	neg	865.5092	8.6568	1.2416	-2.2975	0.0370	0.0954
metab_3754	pos	865.5674	9.7129	2.0461	-7.0108	0.0011	0.0079
metab_11282	neg	865.5732	10.0749	0.4154	-0.1262	0.1420	0.2513
metab_12102	neg	865.5934	7.7100	0.6679	-0.8294	0.2312	0.3565
metab_2057	pos	866.1594	3.0726	2.3813	6.2968	0.0000	0.0003
metab_11377	neg	866.4290	9.7470	1.6490	-4.2859	0.0131	0.0473
metab_11291	neg	866.4657	10.0593	0.8336	-1.4872	0.1909	0.3107
metab_7145	neg	866.5691	10.2070	0.9164	-0.8288	0.0032	0.0179
metab_3619	pos	866.7413	10.3942	1.0710	1.6489	0.0381	0.0929
metab_2279	pos	867.3172	4.1387	0.6339	-0.5663	0.0547	0.1220
metab_11107	neg	867.4759	14.0117	1.3255	-1.4559	0.0025	0.0154
metab_11420	neg	867.5012	9.5979	1.5650	-14.2570	0.0137	0.0488
metab_14851	neg	868.2352	0.5851	0.8775	-1.2194	0.0567	0.1290
metab_10507	neg	868.5722	9.5811	1.7287	-2.6244	0.0101	0.0400
metab_11309	neg	868.5723	9.9929	0.1066	0.1246	0.7643	0.8310
metab_3228	pos	868.6764	11.4034	0.1105	-0.2139	0.9181	0.9467
metab_891	pos	868.7567	10.5629	1.6234	2.5817	0.0065	0.0264

metab_11392	neg	869.4291	9.6986	1.6807	-3.4198	0.0037	0.0200
metab_10122	neg	869.4965	8.0688	1.2046	3.0651	0.0449	0.0572
metab_3859	pos	869.5771	9.2206	0.5470	-0.5480	0.3723	0.5063
metab_13093	neg	870.5321	3.8839	1.2551	12.2904	0.0023	0.0145
metab_10643	neg	870.5507	10.3355	0.0563	0.1689	0.7515	0.8218
metab_10634	neg	870.5841	10.2384	0.0870	0.5655	0.7060	0.7894
metab_11786	neg	870.6107	8.6239	2.3929	-4.4018	0.0000	0.0006
metab_11407	neg	871.0012	9.6316	0.2350	0.3809	0.3531	0.4862
metab_11323	neg	871.4423	9.9274	1.7499	-3.1601	0.0006	0.0065
metab_7349	neg	871.5108	8.0843	0.0043	0.6463	0.9536	0.9697
metab_7144	neg	871.7042	10.1401	0.3447	0.3195	0.4626	0.5850
metab_3201	pos	871.7756	10.7459	1.5353	5.3460	0.0005	0.0045
metab_10757	neg	872.4682	14.0282	1.0944	-1.0812	0.0001	0.0018
metab_12101	neg	872.5060	7.7100	1.3589	1.7197	0.0487	0.1156
metab_14394	neg	873.2230	1.3669	0.5069	-7.9707	0.1787	0.2966
metab_8932	neg	873.3949	2.8440	2.4244	9.2040	0.0000	0.0000
metab_10760	neg	873.4721	14.0282	1.2702	-1.4172	0.0002	0.0028
metab_7315	neg	873.5145	8.7031	1.5456	-3.0612	0.0033	0.0185
metab_3658	pos	873.6087	10.1175	0.5188	-1.1672	0.2950	0.4256
metab_3778	pos	873.6945	9.6199	1.0887	2.5284	0.0409	0.0984
metab_3709	pos	873.7136	9.8673	1.7643	3.6367	0.0006	0.0056
metab_9137	neg	873.9122	3.5310	2.5597	7.1150	0.0000	0.0000
metab_10478	neg	874.4192	9.4156	0.0540	0.0772	0.8444	0.8921
metab_11069	neg	874.4783	14.0282	0.8366	-0.4507	0.0901	0.1800
metab_7232	neg	874.5468	9.9929	0.7247	-0.6013	0.1092	0.2070
metab_835	pos	874.6046	9.4506	1.6715	15.5364	0.0000	0.0008
metab_11227	neg	874.7226	10.3687	0.5409	1.3526	0.2371	0.3629
metab_5255	pos	875.4053	2.8411	1.6668	16.6660	0.0001	0.0011
metab_11945	neg	875.5432	8.1307	0.9289	-3.2723	0.2125	0.3354
metab_10664	neg	875.7350	10.5343	1.2472	2.1112	0.0103	0.0405
metab_5054	pos	875.9230	3.5162	2.7912	8.8587	0.0000	0.0000
metab_7292	neg	876.5335	8.9417	0.8925	-1.5790	0.0437	0.1073
metab_10506	neg	876.5388	9.5649	1.4736	-4.7441	0.0313	0.0852
metab_3807	pos	876.5573	9.4981	1.7353	-3.3758	0.0000	0.0004
metab_10176	neg	876.5617	8.2744	0.1526	-0.4485	0.8316	0.8821
metab_7217	neg	876.5618	10.2875	1.6196	-3.4062	0.0021	0.0134
metab_11644	neg	876.5748	8.9252	1.2711	-1.9968	0.0004	0.0045
metab_6952	neg	877.3429	4.3380	0.6096	0.8427	0.0916	0.1820
metab_11712	neg	877.5360	8.7191	1.2534	-3.3280	0.0262	0.0759
metab_7361	neg	877.5456	7.9908	0.9432	-0.4563	0.0465	0.1119
metab_10052	neg	877.5457	7.7875	0.9853	-0.3078	0.0714	0.1519
metab_7429	neg	878.3017	4.0685	0.3160	0.5723	0.3884	0.5172
metab_11500	neg	878.5422	9.3165	0.4241	0.0125	0.5280	0.6447
metab_3171	pos	878.5729	10.2712	1.9244	-4.9431	0.0002	0.0028
metab_10667	neg	878.5756	10.5679	1.7552	-3.7464	0.0116	0.0435
metab_10275	neg	878.5774	8.6239	0.9017	0.6261	0.1361	0.2433
metab_2973	pos	878.5897	8.9367	1.9651	-3.7936	0.0010	0.0074
metab_10674	neg	878.7541	10.7359	1.6066	3.1056	0.0014	0.0106

metab_4871	pos	879.3536	4.3371	0.7145	0.8191	0.0528	0.1187
metab_12640	neg	879.4443	5.7259	0.0328	-0.2063	0.8276	0.8788
metab_3797	pos	879.5749	9.5283	1.6696	-3.3350	0.0016	0.0101
metab_4920	pos	880.3124	4.0634	0.3281	0.3394	0.3463	0.4812
metab_11366	neg	880.4451	9.7965	1.1131	-2.7216	0.0862	0.1742
metab_3606	pos	880.5853	10.5629	1.4671	-4.3031	0.0028	0.0150
metab_11304	neg	882.4234	10.0090	1.7874	-2.9880	0.0000	0.0005
metab_10504	neg	882.5492	9.5478	0.6777	0.9982	0.1481	0.2591
metab_11305	neg	882.5514	9.9929	1.3732	-2.4375	0.0007	0.0068
metab_11191	neg	883.4266	10.8354	1.5810	-1.9501	0.0003	0.0041
metab_11956	neg	883.5197	8.1153	1.1577	-2.0336	0.0484	0.1150
metab_3671	pos	883.6878	10.0403	0.3497	-0.0996	0.3565	0.4909
metab_14846	neg	884.3019	0.5851	1.0541	-13.6664	0.0012	0.0095
metab_7218	neg	884.5414	10.2070	0.3625	0.0149	0.2686	0.3986
metab_10446	neg	884.5672	9.2185	0.8917	1.2478	0.0417	0.1039
metab_12104	neg	884.5780	7.7100	0.5209	0.5782	0.3379	0.4710
metab_11189	neg	884.6397	10.9510	0.6428	-1.5218	0.3637	0.4959
metab_11307	neg	885.5622	9.9929	0.6190	-0.5636	0.0698	0.1497
metab_11448	neg	885.6839	9.5147	0.5663	2.0463	0.2398	0.3659
metab_13027	neg	886.3430	4.1193	0.7659	-0.4113	0.0105	0.0409
metab_7071	neg	886.5232	8.0843	0.9758	-1.5473	0.0473	0.1133
metab_7220	neg	886.5365	10.2070	0.3850	-0.0417	0.1613	0.2759
metab_10486	neg	886.5449	9.4812	0.2028	-0.8293	0.6800	0.7698
metab_12004	neg	886.6065	7.9908	1.5508	-3.2426	0.0000	0.0008
metab_11444	neg	887.6992	9.5314	0.3431	0.9029	0.2807	0.4113
metab_7430	neg	888.3221	3.9850	0.6387	-0.2550	0.0420	0.1045
metab_4906	pos	888.3538	4.1093	0.7009	-0.6024	0.0267	0.0722
metab_7347	neg	888.5378	8.0843	1.2634	-1.5514	0.0003	0.0042
metab_111	pos	888.6195	10.2404	1.1760	1.4118	0.0003	0.0037
metab_10015	neg	889.5092	7.6793	1.6513	-2.5825	0.0260	0.0755
metab_4940	pos	890.3326	3.9876	0.5588	-0.4650	0.1001	0.1892
metab_7249	neg	890.7180	9.8129	1.4374	2.6637	0.0006	0.0060
metab_3647	pos	890.7410	10.1941	0.5281	-0.3704	0.2437	0.3684
metab_10091	neg	891.5221	7.9612	0.8048	0.3138	0.3240	0.4565
metab_11854	neg	891.5236	8.4505	1.2234	-1.3283	0.0579	0.1309
metab_7261	neg	892.5568	9.4812	0.7364	-1.2516	0.2838	0.4147
metab_12336	neg	892.5592	6.9354	0.9113	-4.0915	0.2557	0.3842
metab_2720	pos	892.5689	7.2614	1.5953	-7.1626	0.0033	0.0164
metab_6993	neg	893.3376	4.2040	0.3782	1.0990	0.5263	0.6430
metab_4284	pos	893.5709	7.2763	1.1379	-13.8824	0.0142	0.0456
metab_3660	pos	893.7186	10.1021	1.3357	-1.9814	0.0132	0.0434
metab_11211	neg	894.5494	10.5511	0.3052	0.1932	0.4057	0.5336
metab_4292	pos	894.5841	7.2458	2.3886	-5.4681	0.0002	0.0029
metab_3609	pos	894.7726	10.5313	1.0338	1.5524	0.0370	0.0911
metab_10638	neg	896.5628	10.2875	0.8255	1.3833	0.0226	0.0685
metab_11863	neg	896.5878	8.4022	0.3351	0.8048	0.5168	0.6351
metab_9659	neg	897.4549	5.9705	0.4516	-0.3645	0.3015	0.4320
metab_10607	neg	898.4928	10.0432	0.2210	0.1371	0.3806	0.5104

metab_10612	neg	898.5814	10.0749	0.0071	0.2401	0.9526	0.9693
metab_10689	neg	898.6556	11.1796	1.3397	3.4738	0.0081	0.0343
metab_10516	neg	899.4399	9.6147	1.8955	-6.1469	0.0026	0.0157
metab_10454	neg	899.5786	9.2674	0.0360	0.0970	0.9159	0.9441
metab_9101	neg	900.4323	3.3803	2.0167	7.7998	0.0000	0.0011
metab_10600	neg	900.5615	10.0090	1.6576	-3.3023	0.0032	0.0180
metab_13086	neg	901.3177	3.9177	0.5995	-0.1802	0.0816	0.1676
metab_9262	neg	901.4804	3.9349	0.9415	12.9163	0.0236	0.0705
metab_11074	neg	901.5076	14.0282	1.5622	-2.4998	0.0000	0.0005
metab_11603	neg	901.6784	9.0391	1.9954	9.7110	0.0015	0.0108
metab_7130	neg	902.5305	9.7152	0.3808	-0.4039	0.1924	0.3122
metab_3144	pos	902.5730	10.0088	1.9165	-4.1478	0.0038	0.0182
metab_949	pos	902.5733	9.4981	1.5448	-3.4696	0.0007	0.0058
metab_10635	neg	902.5750	10.2548	1.8094	-3.4420	0.0180	0.0589
metab_7910	neg	903.2725	0.5991	0.3662	1.7625	0.3768	0.5074
metab_4957	pos	903.3279	3.9114	0.4405	-0.2253	0.2254	0.3487
metab_4950	pos	903.4902	3.9270	1.1258	3.1100	0.0470	0.1090
metab_7281	neg	903.6939	9.2185	1.2129	2.3943	0.0031	0.0175
metab_10554	neg	904.5465	9.7639	0.1995	0.5324	0.5762	0.6854
metab_3800	pos	904.7212	9.5137	0.4938	1.5656	0.3589	0.4931
metab_7276	neg	905.7097	9.3329	1.0471	2.1220	0.0300	0.0826
metab_10578	neg	906.5402	9.8947	0.4580	-0.7544	0.2604	0.3901
metab_3760	pos	906.7367	9.6968	0.0487	0.2688	0.8454	0.9002
metab_3571	pos	906.7727	11.1749	1.2091	-1.7753	0.0144	0.0462
metab_13060	neg	907.3685	4.0182	0.3342	0.4954	0.5280	0.6447
metab_12061	neg	907.5200	7.8193	1.9414	-4.0785	0.0005	0.0058
metab_7262	neg	907.7245	9.4812	1.4880	2.7072	0.0064	0.0290
metab_3726	pos	908.7513	9.8211	1.1712	1.6669	0.0018	0.0111
metab_4932	pos	909.3750	4.0183	0.7461	-0.4974	0.0875	0.1719
metab_840	pos	909.7260	9.5137	0.6981	1.2879	0.2331	0.3570
metab_12125	neg	910.5225	7.6632	0.7691	1.2068	0.0461	0.1114
metab_11525	neg	910.5442	9.2185	0.2603	-0.8094	0.5276	0.6444
metab_11419	neg	910.7445	9.6147	1.8034	3.0928	0.0041	0.0211
metab_850	pos	911.6917	9.7129	0.4369	0.8612	0.3024	0.4339
metab_3093	pos	911.7506	9.7129	2.6565	14.1429	0.0015	0.0097
metab_3677	pos	911.7708	9.9782	1.5937	3.9861	0.0000	0.0002
metab_14825	neg	912.3149	0.5991	1.3324	-1.2049	0.0147	0.0513
metab_4222	pos	912.5330	7.6626	0.6948	0.9137	0.0920	0.1787
metab_10489	neg	912.5603	9.4984	1.4105	-2.6162	0.0034	0.0187
metab_956	pos	912.6197	9.2504	0.1265	-0.0871	0.7306	0.8142
metab_7155	neg	912.6712	11.4070	0.3911	-0.0120	0.6245	0.7248
metab_11208	neg	912.7157	10.5679	1.4118	3.6164	0.0007	0.0068
metab_9983	neg	913.5318	7.5397	1.3205	2.8541	0.0263	0.0760
metab_4241	pos	914.5399	7.5450	0.9770	1.4445	0.1197	0.2165
metab_4107	pos	914.5477	8.1857	0.7559	3.1172	0.0644	0.1372
metab_11214	neg	914.5679	10.5180	0.3890	-0.0236	0.2344	0.3599
metab_11451	neg	915.4792	9.4984	1.7801	-3.5064	0.0002	0.0029
metab_10030	neg	915.5492	7.7100	1.2377	1.7769	0.0274	0.0783

metab_11978	neg	915.5565	8.0688	1.1597	5.1285	0.0071	0.0315
metab_3684	pos	915.7204	9.9470	1.6503	2.8932	0.0020	0.0121
metab_11078	neg	916.4559	14.0282	0.5241	0.1124	0.1489	0.2598
metab_11240	neg	916.5386	10.3194	0.1980	0.2130	0.5260	0.6429
metab_11527	neg	916.5567	9.2185	1.1959	-2.8173	0.0465	0.1119
metab_755	pos	916.5604	8.1132	0.2935	0.1316	0.5867	0.6994
metab_11342	neg	916.5858	9.8782	0.8253	1.6564	0.0708	0.1510
metab_14872	neg	917.1349	0.5571	0.6342	-0.2782	0.0627	0.1387
metab_14743	neg	917.1361	0.7254	0.4994	-0.2662	0.0529	0.1223
metab_7211	neg	918.5525	10.6351	0.9303	1.7437	0.0106	0.0411
metab_958	pos	918.5674	9.2206	1.5342	-4.0405	0.0748	0.1527
metab_6635	neg	918.5720	9.4647	0.5982	-1.9758	0.4315	0.5562
metab_463	pos	919.4888	2.5314	0.8261	3.9371	0.2666	0.3943
metab_7231	neg	920.5522	10.0264	1.6288	-3.1154	0.0003	0.0041
metab_7192	neg	920.5524	14.0282	2.0737	-5.6049	0.0003	0.0037
metab_12079	neg	920.5877	7.7728	0.7795	-0.4920	0.1031	0.1983
metab_10095	neg	920.5879	7.9757	0.4897	0.0234	0.2385	0.3645
metab_10378	neg	921.7046	9.0067	1.8420	3.5746	0.0003	0.0042
metab_10160	neg	922.6031	8.1936	0.1134	1.8515	0.8827	0.9206
metab_10224	neg	922.6039	8.4667	0.8072	2.4895	0.1729	0.2895
metab_3040	pos	922.7313	9.3278	1.8619	3.9459	0.0009	0.0071
metab_7199	neg	923.5071	14.0282	0.0285	0.0650	0.8517	0.8970
metab_9764	neg	923.5134	6.5512	1.4169	-3.3333	0.0168	0.0560
metab_11383	neg	923.6781	9.7312	0.7004	2.4182	0.3062	0.4374
metab_7288	neg	923.7199	9.1364	2.3758	4.1460	0.0036	0.0195
metab_12115	neg	924.5386	7.6793	1.1951	13.5905	0.0334	0.0892
metab_3039	pos	924.7468	9.3278	1.2224	2.0403	0.0197	0.0576
metab_11453	neg	925.5080	9.4984	1.8104	-2.9382	0.0000	0.0007
metab_7242	neg	926.6147	9.8620	1.0511	2.0845	0.0037	0.0199
metab_10036	neg	926.6249	7.7251	0.7267	1.2814	0.2359	0.3613
metab_3809	pos	926.7621	9.4825	1.4833	2.3099	0.0081	0.0308
metab_823	pos	927.6863	9.1898	1.1765	3.2016	0.0330	0.0842
metab_13021	neg	928.3022	4.1364	0.8301	-0.5596	0.0253	0.0741
metab_7258	neg	928.5572	9.4984	1.9154	-4.0288	0.0012	0.0094
metab_10112	neg	928.5694	8.0069	0.0291	0.0873	0.9938	0.9950
metab_11433	neg	929.4500	9.5649	1.4231	-15.5194	0.0163	0.0549
metab_10128	neg	929.5670	8.0843	0.2419	-0.0328	0.5103	0.6294
metab_11167	neg	929.6615	11.4070	0.4437	0.0405	0.6179	0.7202
metab_828	pos	929.7021	9.3278	0.9295	1.6774	0.0960	0.1838
metab_872	pos	929.7416	9.9470	0.2791	-0.4753	0.5866	0.6994
metab_7366	neg	930.5125	7.8193	0.1694	-0.0675	0.6517	0.7469
metab_11301	neg	930.5722	10.0090	1.5418	-7.1976	0.0139	0.0494
metab_3588	pos	930.7727	10.8542	1.2188	-1.9930	0.0309	0.0803
metab_3744	pos	930.7900	9.7586	2.1024	3.1967	0.0031	0.0159
metab_967	pos	931.5751	8.0843	0.6995	-2.8118	0.2359	0.3597
metab_837	pos	931.7167	9.4825	1.3153	1.9735	0.0235	0.0656
metab_10353	neg	932.5508	8.8929	0.9991	-2.5252	0.2174	0.3409
metab_10144	neg	932.5517	8.1307	1.2680	-0.9931	0.1039	0.1997

metab_11385	neg	933.4448	9.7312	1.7668	-3.3866	0.0000	0.0011
metab_12342	neg	933.5326	6.9191	0.0567	0.8975	0.9299	0.9546
metab_913	pos	933.7320	9.6199	1.7103	2.3087	0.0117	0.0400
metab_10694	neg	933.7412	11.2769	1.4431	-2.0036	0.0104	0.0407
metab_11273	neg	934.7003	10.1401	0.5803	0.5788	0.3001	0.4303
metab_894	pos	935.7281	10.8542	1.4395	-2.4868	0.0070	0.0279
metab_10434	neg	936.5468	9.1693	0.9972	-0.7504	0.1203	0.2218
metab_906	pos	937.7535	9.7435	0.9792	3.2982	0.0359	0.0891
metab_11326	neg	938.4872	9.9274	1.1563	1.9486	0.0001	0.0023
metab_11355	neg	938.5236	9.8459	1.1439	2.1798	0.0125	0.0460
metab_10011	neg	938.5544	7.6479	1.0346	1.8679	0.0453	0.1099
metab_10665	neg	938.7317	10.5511	0.7347	3.0499	0.1528	0.2649
metab_11082	neg	939.5017	14.0282	0.2715	-0.1488	0.3268	0.4590
metab_7847	neg	939.5801	0.5286	0.7507	11.5447	0.0499	0.1173
metab_9356	neg	940.3389	4.3380	0.5968	0.8558	0.1398	0.2484
metab_10763	neg	940.4581	14.0282	1.1862	-1.2333	0.0014	0.0105
metab_7224	neg	940.5385	10.1401	0.0645	0.1073	0.8675	0.9092
metab_3765	pos	940.7783	9.6823	1.0532	4.1930	0.0893	0.1745
metab_13043	neg	941.2965	4.0685	0.3472	0.5579	0.3662	0.4982
metab_11447	neg	942.4970	9.5147	2.4660	-5.3396	0.0000	0.0012
metab_10637	neg	942.5517	10.2875	0.4258	0.7327	0.1527	0.2648
metab_865	pos	942.5672	9.8673	0.7476	-1.4042	0.1583	0.2666
metab_7233	neg	942.5767	9.9767	0.8443	-1.1576	0.0463	0.1116
metab_3017	pos	942.7566	9.1747	2.6332	7.7027	0.0000	0.0001
metab_3735	pos	942.7933	9.7899	2.4539	4.6468	0.0042	0.0195
metab_12152	neg	943.5359	7.5702	0.4186	-0.2745	0.4114	0.5384
metab_758	pos	943.6353	8.0843	1.9890	3.3361	0.0156	0.0264
metab_841	pos	943.7174	9.5283	1.2120	3.6449	0.0476	0.1098
metab_11370	neg	944.5515	9.7804	2.1571	-4.5453	0.0032	0.0180
metab_876	pos	944.7503	10.1331	1.8495	-2.7854	0.0009	0.0071
metab_3123	pos	944.7525	9.8836	1.7231	3.3393	0.0013	0.0087
metab_11202	neg	945.5710	10.6518	0.2214	0.0133	0.6738	0.7643
metab_11480	neg	945.5801	9.3824	0.9279	-0.2329	0.1264	0.2302
metab_4129	pos	945.6498	8.0988	2.2911	6.2194	0.0001	0.0013
metab_3000	pos	945.6967	9.0857	1.9413	3.5593	0.0010	0.0074
metab_3767	pos	945.7336	9.6671	1.3920	3.3892	0.0214	0.0612
metab_11391	neg	946.4549	9.6986	1.8543	-7.2589	0.0008	0.0073
metab_3105	pos	946.5620	9.7899	2.3697	-6.7700	0.0000	0.0008
metab_3166	pos	946.7662	10.2252	1.4640	-2.2514	0.0224	0.0634
metab_3006	pos	947.7120	9.1140	1.8210	3.7916	0.0010	0.0074
metab_10529	neg	948.7249	9.6477	2.1728	6.5542	0.0000	0.0005
metab_9310	neg	949.3383	4.1193	1.0626	-0.9364	0.0050	0.0244
metab_3124	pos	949.7081	9.8836	1.7153	5.2666	0.0021	0.0122
metab_13378	neg	950.4315	3.0942	2.0275	15.4762	0.0001	0.0021
metab_11904	neg	950.5619	8.2744	1.1809	-5.1061	0.1088	0.2066
metab_10503	neg	950.6947	9.5478	1.1417	2.4047	0.0147	0.0513
metab_9271	neg	951.3182	3.9850	0.7711	-0.4543	0.0640	0.1410
metab_2065	pos	952.4413	3.1032	1.6596	15.7908	0.0011	0.0082

metab_10001	neg	952.5694	7.6169	0.9471	1.2341	0.0001	0.0021
metab_9982	neg	954.5124	7.5397	0.7504	0.6892	0.2563	0.3848
metab_4229	pos	954.5808	7.6185	0.9327	1.3007	0.0057	0.0241
metab_3719	pos	954.6569	9.8362	0.9296	-2.8045	0.1387	0.2415
metab_11928	neg	955.5836	8.1936	1.6967	3.5500	0.0001	0.0015
metab_12107	neg	955.6244	7.6951	0.1948	0.2430	0.6881	0.7762
metab_10051	neg	956.5275	7.7875	1.8309	2.5084	0.0286	0.0803
metab_10482	neg	956.5337	9.4485	0.1829	-0.2342	0.7097	0.7923
metab_10269	neg	956.5449	8.6093	0.8287	-1.3869	0.0831	0.1696
metab_10343	neg	956.5451	8.8128	0.7603	-0.5965	0.2258	0.3503
metab_3777	pos	956.8087	9.6199	1.4047	3.1628	0.0299	0.0786
metab_10491	neg	957.4451	9.5147	2.3902	-6.0668	0.0000	0.0006
metab_4109	pos	957.5904	8.1857	2.0952	3.7601	0.0010	0.0076
metab_10032	neg	957.5926	7.7100	2.2193	4.7044	0.0024	0.0150
metab_7152	neg	957.7410	10.8522	1.4235	-2.1109	0.0025	0.0151
metab_11346	neg	957.8138	9.8620	0.4526	0.3181	0.4684	0.5908
metab_744	pos	958.5379	7.7946	0.8487	3.3729	0.1333	0.2344
metab_10542	neg	958.5484	9.7152	0.7930	1.2808	0.1565	0.2698
metab_2957	pos	958.5549	8.8169	0.8337	-1.3176	0.1208	0.2183
metab_11535	neg	958.5698	9.1858	0.9974	-1.2257	0.0977	0.1909
metab_10496	neg	959.4590	9.5314	2.0196	-4.0517	0.0011	0.0090
metab_10686	neg	959.7557	11.1147	1.6597	-1.8887	0.0064	0.0290
metab_11653	neg	960.5467	8.8929	1.4284	-2.5230	0.0116	0.0435
metab_3814	pos	960.7466	9.4506	0.3135	2.3575	0.6355	0.7399
metab_9906	neg	961.5022	7.1595	1.0614	1.8801	0.0826	0.1689
metab_3720	pos	962.7621	9.8362	0.7311	-0.6299	0.2045	0.3239
metab_3135	pos	964.7775	9.9470	0.6194	-0.5927	0.3145	0.4471
metab_10534	neg	966.5875	9.6814	2.2103	-4.9503	0.0015	0.0108
metab_11529	neg	966.6897	9.2025	2.0073	6.6598	0.0001	0.0020
metab_12958	neg	967.3378	4.3380	0.4995	0.8708	0.3783	0.5083
metab_12118	neg	967.5799	7.6793	1.1866	2.3111	0.0248	0.0731
metab_3125	pos	967.7170	9.8836	1.1100	-0.7561	0.0957	0.1834
metab_7129	neg	967.7451	9.6814	0.9700	5.6483	0.1953	0.3157
metab_11429	neg	968.4984	9.5811	2.5746	-3.6559	0.0133	0.0478
metab_11494	neg	968.7053	9.3329	1.2520	2.5397	0.0186	0.0597
metab_9962	neg	969.5947	7.4465	0.4715	1.9021	0.3253	0.4577
metab_3697	pos	969.7329	9.9154	0.4822	-0.6261	0.2991	0.4302
metab_7133	neg	969.7614	9.8129	1.2070	6.3315	0.0204	0.0639
metab_7260	neg	970.7199	9.4812	2.0714	3.4667	0.0047	0.0235
metab_10067	neg	971.6109	7.8507	0.6590	1.9906	0.0740	0.1557
metab_12001	neg	971.6111	8.0069	0.3324	-0.0283	0.3446	0.4777
metab_11270	neg	971.6298	10.1401	0.2486	1.1528	0.7513	0.8218
metab_9901	neg	972.5399	7.1437	1.5430	-3.8091	0.0115	0.0434
metab_11401	neg	972.5822	9.6477	1.7134	-6.0430	0.0002	0.0027
metab_7063	neg	973.5543	7.8026	0.1328	0.6336	0.7537	0.8231
metab_971	pos	973.6224	7.9980	1.0541	1.2875	0.0040	0.0188
metab_10133	neg	973.6261	8.0998	0.0165	0.4306	0.9131	0.9426
metab_11474	neg	974.5836	9.4156	1.0150	-1.8624	0.0451	0.1097

metab_10679	neg	974.7315	10.8522	1.4855	-2.2797	0.0102	0.0402
metab_11371	neg	974.7470	9.7639	2.1497	4.9056	0.0000	0.0005
metab_12957	neg	975.3124	4.3380	0.4624	1.1631	0.3900	0.5185
metab_742	pos	975.5642	7.8097	0.6611	1.4319	0.1123	0.2061
metab_12969	neg	977.3700	4.3207	1.3165	-1.5276	0.0004	0.0044
metab_11415	neg	977.7284	9.6147	1.5698	5.1249	0.0005	0.0059
metab_3836	pos	978.7570	9.3278	1.0634	4.2285	0.1233	0.2215
metab_2301	pos	979.3792	4.3060	1.4663	-2.3573	0.0004	0.0040
metab_3235	pos	980.2739	11.8118	1.2058	15.6379	0.0542	0.1212
metab_13126	neg	980.3335	3.8001	1.2010	-1.2310	0.0001	0.0022
metab_3764	pos	980.7716	9.6823	0.6816	1.0633	0.2988	0.4299
metab_4980	pos	982.3436	3.8063	1.2099	-1.4850	0.0000	0.0007
metab_954	pos	983.7121	9.3429	0.8316	2.2626	0.2528	0.3788
metab_10103	neg	984.6398	7.9908	1.0997	-1.6819	0.0450	0.1096
metab_11755	neg	984.7881	8.6874	1.3676	-3.2187	0.0158	0.0540
metab_12978	neg	985.4118	4.2870	1.4520	-1.7050	0.0103	0.0404
metab_3840	pos	985.7268	9.3117	0.3962	0.4173	0.5895	0.7014
metab_8011	neg	986.1477	0.8803	1.9331	3.8009	0.0007	0.0068
metab_14739	neg	986.1477	0.7537	1.8306	3.0795	0.0006	0.0063
metab_14800	neg	986.3341	0.5991	1.1926	15.6456	0.0010	0.0086
metab_9735	neg	986.6515	6.3917	0.1499	0.2886	0.6075	0.7113
metab_9705	neg	986.6520	6.2456	0.3006	0.8050	0.5728	0.6828
metab_13003	neg	987.3907	4.2040	1.5980	-2.4383	0.0047	0.0233
metab_12493	neg	988.6659	6.3917	1.4406	-2.3186	0.0003	0.0039
metab_7122	neg	989.7299	9.4812	0.9337	1.3270	0.0719	0.1526
metab_14518	neg	991.2117	1.2236	1.5260	-2.2816	0.0024	0.0150
metab_10672	neg	991.7018	10.7190	1.2319	-1.4582	0.0292	0.0814
metab_7138	neg	991.7451	9.9606	0.6496	0.2158	0.4245	0.5502
metab_13148	neg	993.3287	3.7488	0.9431	-0.7955	0.0043	0.0221
metab_2213	pos	995.3385	3.7447	0.9691	-1.1306	0.0071	0.0283
metab_7056	neg	995.6118	7.6479	1.0463	1.4490	0.0001	0.0017
metab_8420	neg	995.9525	1.6642	1.2155	-0.2675	0.0340	0.0900
metab_12123	neg	997.5548	7.6632	1.3322	1.6547	0.0426	0.1056
metab_12166	neg	997.5551	7.5397	1.0636	0.9214	0.1096	0.2076
metab_4240	pos	997.6223	7.5743	1.1631	1.3999	0.0000	0.0009
metab_12025	neg	997.6258	7.9612	1.6084	3.3137	0.0000	0.0002
metab_4979	pos	999.3699	3.8063	1.5925	-2.3749	0.0061	0.0253
metab_730	pos	999.5645	7.5307	0.9922	0.5854	0.1386	0.2415
metab_10567	neg	1000.6148	9.8620	1.6150	-3.0534	0.0080	0.0340
metab_11168	neg	1000.7386	11.3904	1.4173	-3.1504	0.0039	0.0207
metab_13268	neg	1001.4799	3.3803	1.3669	15.2837	0.0001	0.0018
metab_10053	neg	1001.5759	7.7875	1.0131	3.7728	0.0279	0.0792
metab_4196	pos	1001.5796	7.7946	1.6081	2.6198	0.0106	0.0372
metab_9956	neg	1001.5848	7.4300	1.7869	4.1957	0.0002	0.0032
metab_11463	neg	1002.5933	9.4647	1.8978	-8.6414	0.0043	0.0220
metab_13163	neg	1003.3498	3.6980	0.8466	-0.5333	0.0005	0.0056
metab_9952	neg	1003.5292	7.3984	1.0341	1.3071	0.1090	0.2068
metab_754	pos	1003.5923	8.0560	0.9435	4.1738	0.0335	0.0849

metab_10291	neg	1004.8143	8.6874	0.0705	0.3798	0.9117	0.9416
metab_5010	pos	1005.3595	3.6987	0.9232	-0.8820	0.0001	0.0014
metab_7116	neg	1005.7252	9.2185	1.1377	-0.3942	0.2038	0.3255
metab_13038	neg	1006.3854	4.0859	1.1618	-1.2143	0.0005	0.0057
metab_11519	neg	1006.7269	9.2350	0.3018	1.5159	0.7305	0.8061
metab_11457	neg	1007.6508	9.4812	2.1689	3.4647	0.0096	0.0387
metab_10593	neg	1007.6959	9.9767	1.3050	-1.9512	0.0236	0.0705
metab_575	pos	1008.3954	4.0788	1.2722	-1.7211	0.0039	0.0184
metab_12009	neg	1008.6405	7.9908	0.6053	-0.5424	0.2995	0.4300
metab_10588	neg	1008.7382	9.9433	0.4073	0.0680	0.4437	0.5676
metab_11410	neg	1009.6652	9.6316	2.2272	4.1175	0.0028	0.0163
metab_12017	neg	1010.6550	7.9757	0.0217	1.5021	0.9412	0.9617
metab_9948	neg	1013.5864	7.3659	1.0949	4.5994	0.0575	0.1302
metab_11999	neg	1015.6007	8.0069	0.2834	1.1949	0.4441	0.5677
metab_12066	neg	1015.6019	7.8026	0.2292	0.3893	0.6577	0.7521
metab_4155	pos	1015.6686	7.9980	1.1335	2.0253	0.0215	0.0616
metab_10430	neg	1015.7222	9.1200	0.3631	1.5007	0.5480	0.6603
metab_13187	neg	1016.3449	3.6311	0.9981	-0.8526	0.0005	0.0052
metab_10109	neg	1017.6162	8.0069	1.4148	4.3968	0.0069	0.0307
metab_2815	pos	1017.6833	7.9980	1.6391	3.7863	0.0021	0.0124
metab_13206	neg	1021.4078	3.5813	1.2137	-1.2190	0.0000	0.0007
metab_2176	pos	1023.4163	3.5771	1.3469	-1.9143	0.0000	0.0007
metab_4914	pos	1025.4218	4.0788	1.4024	-2.1144	0.0037	0.0178
metab_2769	pos	1025.6528	7.6481	1.3053	1.8884	0.0022	0.0128
metab_12231	neg	1028.5487	7.2537	0.1450	0.3209	0.8207	0.8730
metab_1807	pos	1029.5212	2.1290	1.1331	14.6093	0.0027	0.0147
metab_5545	pos	1029.7227	2.1290	1.0158	14.8949	0.0064	0.0261
metab_12163	neg	1031.5475	7.5397	0.0395	-0.2892	0.9151	0.9437
metab_11957	neg	1032.6250	8.0998	1.2477	-13.1270	0.0015	0.0110
metab_7372	neg	1037.5824	7.6632	0.4648	-0.4070	0.3643	0.4963
metab_7046	neg	1039.6017	7.4465	0.9858	1.6751	0.0007	0.0068
metab_979	pos	1039.6690	7.5892	1.0581	1.3785	0.0137	0.0447
metab_4263	pos	1041.6106	7.4269	1.2108	2.2217	0.0031	0.0158
metab_12073	neg	1041.6170	7.7875	1.8293	4.9114	0.0004	0.0049
metab_96	pos	1041.6829	8.0419	1.4721	3.0453	0.0030	0.0157
metab_2718	pos	1043.5546	7.2614	0.8161	1.1486	0.1254	0.2240
metab_4115	pos	1043.6444	8.1560	0.0402	-0.9645	0.8240	0.8841
metab_751	pos	1043.6964	7.9551	2.1614	6.3219	0.0001	0.0011
metab_968	pos	1043.6967	8.0843	1.6737	4.1073	0.0010	0.0074
