# Supplementary material for: Nutritional Characterization and Untargeted Metabolomics of Oyster Mushroom Produced Using Astragalus membranaceus var. mongolicus Stems and Leaves as Substrates
Source: Front Plant Sci. 2022 Feb 3;13:802801. doi: 10.3389/fpls.2022.802801 (PMC8853653; doi:10.3389/fpls.2022.802801)
Supplement: Supplementary file 4 [file Table_2.pdf]

Table S2 Metabolites annotated with HMDB and Pubchem databases.

**Metabolite_ID**	**Retention_ time**	**Apex_ m/z**	**Mode**	**Putative_ Metabolite**	**Putative_ Formula**	**Molecular _Weight**	**Pubchem_ ID**	**Pubchem_ Class**	**Pubchem_ Sub_class**	**HMDB_ID**	**HMDB_name**
metab_6274	0.7661	72.0813	pos	3-Buten-1-amine	C4H9N	71.0735	443732	-	-	-	-
metab_221	0.7941	84.0447	pos	L-Threonine	C4H9NO3	119.0582	6288	Carboxylic acids and derivatives	Amino acids, peptides, and analogues	HMDB0000167	L-Threonine
metab_1297	0.5983	86.0603	pos	2-Pyrrolidinone	C4H7NO	85.0528	12025	Pyrrolidines	Pyrrolidones	HMDB0002039	2-Pyrrolidinone
metab_6375	0.5983	87.0443	pos	Isocrotonic acid	C4H6O2	86.0368	643792	Fatty Acyls	Fatty acids and conjugates	LMFA01030194; HMDB0034439	Isocrotonic acid
metab_1524	1.2585	100.0758	pos	2,4-Dimethyl-3-oxazoline	C5H9NO	99.0684	53654	Azolines	Oxazolines	HMDB0040518	2,5-Dihydro-2,4-dimethyloxazole
metab_14033	1.8205	101.0596	neg	Valerate	C5H10O2	102.0680	114781	-	-	-	-
metab_14260	1.5297	101.0596	neg	Valerate	C5H10O2	102.0680	114781	-	-	-	-
metab_14736	0.7537	103.0024	neg	Malonic acid	C3H4O4	104.0109	867	-	-	-	-
metab_1147	0.5843	104.1070	pos	Choline	C5H14NO	103.1001	305	-	-	-	-
metab_1670	1.6983	105.0336	pos	Malonic acid	C3H4O4	104.0109	867	Carboxylic acids and derivatives	Dicarboxylic acids and derivatives	HMDB0000691	Propanedioic acid
metab_5867	1.5144	105.0699	pos	Styrene	C8H8	104.0630	7501	-	-	-	-
metab_5990	1.2725	107.0492	pos	Benzaldehyde	C7H6O	106.0419	240	Benzene and substituted derivatives	Benzoyl derivatives	HMDB0006115	Benzaldehyde
metab_6554	0.2224	108.0444	pos	3-Pyridinecarboxaldehyde	C6H5NO	107.0371	10371	-	-	-	-
metab_5877	1.5004	109.0648	pos	m-Cresol	C7H8O	108.0575	342	Phenols	Cresols	HMDB0002048; HMDB0001858	m-Cresol;p-Cresol
metab_4095	8.2289	109.1011	pos	4-Vinylcyclohexene	C8H12	108.0942	7499	-	-	-	-
metab_4728	4.9413	109.1011	pos	4-Vinylcyclohexene	C8H12	108.0942	7499	-	-	-	-
metab_5439	2.3447	109.1012	pos	4-Vinylcyclohexene	C8H12	108.0942	7499	-	-	-	-

metab_6757	0.6411	111.0075	neg	2-Furoate	C5H4O3	112.0160	5460151	-	-	-	-
metab_6778	0.7818	111.0075	neg	2-Furoate	C5H4O3	112.0160	5460151	-	-	-	-
metab_2921	8.6132	111.0804	pos	2,4-Heptadienal	C7H10O	110.0736	5283321	-	-	-	-
metab_3991	8.6132	111.1168	pos	(4E)-2,3-Dimethylhexa-1,4-diene	C8H14	110.1099	5368910	-	-	-	-
metab_1311	0.6123	112.0505	pos	Cytosine	C4H5N3O	111.0436	597	-	-	-	-
metab_8071	1.0499	114.0549	neg	2-Amino-5-hydroxypentanoic acid	C5H11NO3	133.0739	95562	Carboxylic acids and derivatives	Amino acids, peptides, and analogues	HMDB0031658	L-2-Amino-5-hydroxypentanoic acid
metab_1074	1.3293	115.0389	pos	cis-Acetylacrylic acid	C5H6O3	114.0319	5281015	-	-	-	-
metab_5945	1.3579	115.0866	pos	L-Prolinamide	C5H10N2O	114.0793	111306	-	-	-	-
metab_8691	2.2437	116.0494	neg	Indole	C8H7N	117.0580	798	-	-	-	-
metab_14241	1.5297	116.0494	neg	Indole	C8H7N	117.0580	798	-	-	-	-
metab_146	0.6123	116.0705	pos	Proline	C5H9NO2	115.0636	145742	-	-	-	-
metab_6831	0.8662	117.0181	neg	Succinic acid	C4H6O4	118.0265	1110	-	-	-	-
metab_14858	0.5851	118.0498	neg	L-Homoserine	C4H9NO3	119.0582	12647	-	-	-	-
metab_5857	1.5284	118.0650	pos	Phenylacetonitrile	C8H7N	117.0581	8794	-	-	-	-
metab_5857	1.5284	118.0650	pos	Indole	C8H7N	117.0580	798	-	-	-	-
metab_1088	1.2725	118.0652	pos	Phenylacetonitrile	C8H7N	117.0581	8794	-	-	-	-
metab_1088	1.2725	118.0652	pos	Indole	C8H7N	117.0580	798	-	-	-	-
metab_6310	0.6402	118.0861	pos	Valine	C5H11NO2	117.0790	6287	Carboxylic acids and derivatives	Amino acids, peptides, and analogues	HMDB0000883	L-Valine
metab_6310	0.6402	118.0861	pos	Norvaline	C5H11NO2	117.0792	65098	Carboxylic acids and derivatives	Amino acids, peptides, and analogues	HMDB0000883	L-Valine
metab_2518	5.7846	119.0854	pos	alpha-Methylstyrene	C9H10	118.0783	7407	Benzene and substituted derivatives	Phenylpropenes	HMDB0059899	alpha-Methylstyrene

metab_3877	9.1302	121.0282	pos	4-Hydroxybenzaldehyde	C7H6O2	120.0213	126	-	-	-	-
metab_5076	3.4251	121.0283	pos	4-Hydroxybenzaldehyde	C7H6O2	120.0213	126	-	-	-	-
metab_1965	2.7029	121.0283	pos	4-Hydroxybenzaldehyde	C7H6O2	120.0213	126	-	-	-	-
metab_8023	0.9086	121.0283	neg	4-Hydroxybenzaldehyde	C7H6O2	122.0367	126	-	-	-	-
metab_8618	2.0670	121.0284	neg	4-Hydroxybenzaldehyde	C7H6O2	122.0367	126	-	-	-	-
metab_13354	3.1626	121.0284	neg	4-Hydroxybenzaldehyde	C7H6O2	122.0367	126	-	-	-	-
metab_6244	0.7941	121.0395	pos	4-Hydroxybenzaldehyde	C7H6O2	120.0213	126	Organooxygen compounds	Carbohydrates and carbohydrate conjugates	HMDB0011718	P-Formylphenol
metab_1085	1.2725	121.0645	pos	Phenylacetaldehyde	C8H8O	120.0577	998	-	-	-	-
metab_4684	5.1830	121.0646	pos	Phenylacetaldehyde	C8H8O	120.0577	998	-	-	-	-
metab_1397	0.8920	121.0646	pos	Phenylacetaldehyde	C8H8O	120.0577	998	Benzene and substituted derivatives	Phenylacetaldehydes	HMDB0006236; HMDB0041610	Phenylacetaldehyde;(2,2-Diethoxyethyl)benzene
metab_14726	0.7818	122.0236	neg	Isonicotinic acid	C6H5NO2	123.0320	5922	-	-	-	-
metab_6248	0.7801	123.0515	pos	Isonicotineamide	C6H6N2O	122.0482	15074	-	-	-	-
metab_6270	0.7801	123.0551	pos	Isonicotinamide	C6H6N2O	122.0482	15074	Pyridines and derivatives	Pyridinecarboxylic acids and derivatives	HMDB0001406	Niacinamide
metab_13216	3.5641	124.0393	neg	6-Oxopiperidine-2-carboxylic acid	C6H9NO3	143.0582	3014237	Carboxylic acids and derivatives	Amino acids, peptides, and analogues	HMDB0061705	6-Oxopiperidine-2-carboxylic acid
metab_8042	0.9792	125.0233	neg	5-Hydroxymethylfurfural	C6H6O3	126.0318	237332	-	-	-	-
metab_8042	0.9792	125.0233	neg	4-Hydroxy-6-methyl-2-pyrone	C6H6O3	126.0318	54675757	-	-	-	-
metab_8644	2.1311	125.0961	neg	2-Octenal	C8H14O	126.1044	5283324	-	-	-	-
metab_13340	3.1964	125.0961	neg	2-Octenal	C8H14O	126.1044	5283324	-	-	-	-
metab_158	0.8361	127.0387	pos	5-Hydroxymethylfurfural	C6H6O3	126.0318	237332	-	-	-	-
metab_158	0.8361	127.0387	pos	4-Hydroxy-6-methyl-2-pyrone	C6H6O3	126.0318	54675757	-	-	-	-
metab_158	0.8361	127.0387	pos	4-Hydroxy-6-methyl-2-pyrone	C6H6O3	126.0318	54675757	-	-	-	-

metab_117	14.5961	127.0387	pos	5-Hydroxymethylfurfural	C6H6O3	126.0318	237332	-	-	-	-
metab_117	14.5961	127.0387	pos	4-Hydroxy-6-methyl-2-pyrone	C6H6O3	126.0318	54675757	-	-	-	-
metab_28	0.2713	127.0387	pos	5-Hydroxymethylfurfural	C6H6O3	126.0318	237332	-	-	-	-
metab_28	0.2713	127.0387	pos	4-Hydroxy-6-methyl-2-pyrone	C6H6O3	126.0318	54675757	-	-	-	-
metab_13427	2.9931	127.0754	neg	Cyclohexanecarboxylic acid	C7 H12 O2	128.0837	7413	-	-	-	-
metab_8715	2.3078	127.0754	neg	Cyclohexanecarboxylic acid	C7 H12 O2	128.0837	7413	-	-	-	-
metab_7482	1.7104	127.0754	neg	Cyclohexanecarboxylic acid	C7 H12 O2	128.0837	7413	-	-	-	-
metab_7987	0.7958	128.0342	neg	L-4-Hydroxyglutamate semialdehyde	C5H9NO4	147.0532	440851	Carboxylic acids and derivatives	Amino acids, peptides, and analogues	HMDB0006556	L-4-Hydroxyglutamate semialdehyde
metab_6254	0.7801	128.1068	pos	N-Cyclohexylformamide	C7H13NO	127.0998	13017	-	-	-	-
metab_1075	1.3293	128.1068	pos	N-Cyclohexylformamide	C7H13NO	127.0998	13017	-	-	-	-
metab_5412	2.4071	128.1068	pos	N-Cyclohexylformamide	C7H13NO	127.0998	13017	-	-	-	-
metab_3295	14.0665	128.1431	pos	Coniine	C8H17N	127.1363	441072	-	-	-	-
metab_3419	14.4245	128.1431	pos	Coniine	C8H17N	127.1363	441072	-	-	-	-
metab_2028	2.9501	128.1432	pos	Coniine	C8H17N	127.1363	441072	-	-	-	-
metab_10902	15.9795	129.0546	neg	6-Oxohexanoic acid	C6H10O3	130.0630	440918	-	-	-	-
metab_10902	15.9795	129.0546	neg	Ethyl acetoacetate	C6H10O3	130.0631	8868	-	-	-	-
metab_7785	0.4634	129.0546	neg	6-Oxohexanoic acid	C6H10O3	130.0630	440918	-	-	-	-
metab_7785	0.4634	129.0546	neg	Ethyl acetoacetate	C6H10O3	130.0631	8868	-	-	-	-
metab_7539	2.0202	129.0546	neg	6-Oxohexanoic acid	C6H10O3	130.0630	440918	-	-	-	-
metab_7539	2.0202	129.0546	neg	Ethyl acetoacetate	C6H10O3	130.0631	8868	-	-	-	-
metab_1442	1.0460	129.0908	pos	Cyclohexanecarboxylic acid	C7 H12 O2	128.0837	7413	Carboxylic acids and derivatives	Carboxylic acids	HMDB0031342	Cyclohexanoic acid
metab_1544	1.3293	129.0908	pos	Cyclohexanecarboxylic acid	C7 H12 O2	128.0837	7413	Carboxylic acids and derivatives	Carboxylic acids	HMDB0031342	Cyclohexanoic acid

metab_1325	0.6402	130.0456	pos	3-Methylindole	C9H9N	129.058	6736	Indoles and derivatives		HMDB0000466	b-Methylindole
metab_220	0.7941	130.0496	pos	Glutamic acid	C5H9NO4	147.0532	33032	Carboxylic acids and derivatives	Amino acids, peptides, and analogues	HMDB0060475; HMDB0003339; HMDB0000148	DL-Glutamate;D-Glutamic acid;L-Glutamic acid
metab_6297	0.6542	130.0606	pos	L-Pipecolate	C6H11NO2	129.0791	439227	Carboxylic acids and derivatives	Amino acids, peptides, and analogues	HMDB0000716	L-(-)-Pipecolic acid
metab_5842	1.5424	130.0649	pos	3-Methylindole	C9H9N	129.0580	6736	-	-	-	-
metab_503	3.0114	130.0649	pos	3-Methylindole	C9H9N	129.0580	6736	-	-	-	-
metab_1136	0.6123	130.0860	pos	l-Pipecolic acid	C6H11NO2	129.0791	439227	-	-	-	-
metab_1171	0.5140	130.0860	pos	l-Pipecolic acid	C6H11NO2	129.0791	439227	-	-	-	-
metab_14619	0.9792	130.0863	neg	Leucine	C6H13NO2	131.0947	6106	-	-	-	-
metab_8547	1.9280	130.0863	neg	Leucine	C6H13NO2	131.0947	6106	-	-	-	-
metab_13706	2.4010	130.0863	neg	Leucine	C6H13NO2	131.0947	6106	-	-	-	-
metab_14417	1.3373	131.0339	neg	Glutaric acid	C5H8O4	132.0423	743	Carboxylic acids and derivatives	Dicarboxylic acids and derivatives	HMDB0000661	Glutaric acid
metab_14897	0.5571	131.0451	neg	Asparagine	C4H8N2O3	132.0535	6267	Carboxylic acids and derivatives	Amino acids, peptides, and analogues	HMDB0000168	L-Asparagine
metab_6450	0.5280	131.1289	pos	Agmatine	C5H14N4	130.1218	199	Organonitrogen compounds	Guanidines	HMDB0001432	Agmatine
metab_14843	0.5991	132.0292	neg	Aspartic acid	C4H7NO4	133.0375	5960	Carboxylic acids and derivatives	Amino acids, peptides, and analogues	HMDB0000191	L-Aspartic acid
metab_5856	1.5284	132.0805	pos	3-Methylindole	C9H9N	131.0735	6736	Indoles and derivatives	Indoles	HMDB0000466	3-Methylindole
metab_5856	1.5284	132.0805	pos	2-Methylbenzyl cyanide	C9H9N	131.0736	31155	Indoles and derivatives	Indoles	HMDB0000466	3-Methylindole
metab_1422	0.9761	132.1016	pos	Leucine	C6H13NO2	131.0947	6106	Carboxylic acids and derivatives	Amino acids, peptides, and analogues	HMDB0000172; HMDB0000557	L-Isoleucine;L-Alloisoleucine
metab_1422	0.9761	132.1016	pos	l-Isoleucine	C6H13NO2	131.0946	6306	Carboxylic acids and	Amino acids, peptides,	HMDB0000172;	L-Isoleucine;L-Alloisoleucine

								derivatives	and analogues	HMDB0000557	
metab_1379	0.8220	132.1380	pos	Leucine	C6H13NO2	131.0947	6106	Carboxylic acids and derivatives	Indoles	HMDB0000687	(2S)-2-amino-4-Methylpentanoic acid
metab_6762	0.6411	133.0131	neg	D-Malate	C4H6O5	134.0215	92824	Fatty Acyls	Fatty acids and conjugates	HMDB0031518	D-Malic acid
metab_1355	0.7801	133.0314	pos	Tetrahydrothiophene-2-carboxylic acid	C5H8O2S	132.0245	443066	-	-	-	-
metab_6151	0.9761	133.0491	pos	Glutaric acid	C5H8O4	132.0423	743	-	-	-	-
metab_7991	0.8098	133.0494	neg	1-Deoxy-D-xylulose	C5H10O4	134.0579	9548569	-	-	-	-
metab_4575	5.7846	133.0645	pos	Cinnamaldehyde	C9H8O	132.0576	637511	-	-	-	-
metab_1160	0.5280	133.0969	pos	D-Ornithine	C5H12N2O2	132.0899	71082	Carboxylic acids and derivatives	Amino acids, peptides, and analogues	HMDB0003374	D-Ornithine
metab_1572	1.4146	133.0969	pos	Cinnamaldehyde	C9H8O	132.0576	637511	Cinnamaldehydes	Eicosanoids	HMDB0003441	3-Phenylacrylaldehyde
metab_4347	7.0082	133.1009	pos	1,2,3,4-Tetrahydronaphthalene	C10H12	132.0940	8404	-	-	-	-
metab_5388	2.4530	133.1010	pos	1-Methyl-4-(prop-1-en-2-yl)benzene	C10H12	132.0939	62385	Benzene and substituted derivatives	Phenylpropenes	HMDB0029641	p-Mentha-1,3,5,8-tetraene
metab_5388	2.4530	133.1010	pos	1,2,3,4-Tetrahydronaphthalene	C10H12	132.0940	8404	Benzene and substituted derivatives	Phenylpropenes	HMDB0029641	p-Mentha-1,3,5,8-tetraene
metab_14345	1.4568	134.0462	neg	Adenine	C5H5N5	135.0546	190	-	-	-	-
metab_14844	0.5991	135.0288	neg	(2R,3R)-2,3,4-Trihydroxybutanoic acid	C4H8O5	136.0372	2781043	Organooxygen compounds	Carbohydrates and carbohydrate conjugates	HMDB0000613	Erythronic acid
metab_4756	4.8509	135.0801	pos	4-Allylphenol	C9H10O	134.0732	68148	-	-	-	-
metab_4581	5.7846	135.0801	pos	4-Allylphenol	C9H10O	134.0732	68148	-	-	-	-
metab_4350	7.0082	135.1165	pos	(-)-Perillyl alcohol	C10H16O	134.1095	369312	Prenol lipids	Monoterpenoids	HMDB0003634	Perillyl alcohol
metab_2917	8.6132	135.1166	pos	(-)-Perillyl alcohol	C10H16O	134.1095	369312	-	-	-	-

metab_4737	4.9267	135.1166	pos	(-)-Perillyl alcohol	C10H16O	134.1095	369312	-	-	-	-
metab_8835	2.6046	136.0394	neg	Anthranilic acid	C7H7NO2	137.0477	227	Pyridines and derivatives	-	HMDB0060722	2-Pyridylacetic acid
metab_7586	2.3380	136.0394	neg	Anthranilic acid	C7H7NO2	137.0477	227	-	-	-	-
metab_7586	2.3380	136.0394	neg	Trigonelline	C7H7NO2	137.0477	5570	Alkaloids and derivatives		HMDB0000875	Trigonelline
metab_14258	1.5297	136.0394	neg	Anthranilic acid	C7H7NO2	137.0477	227	-	-	-	-
metab_14084	1.7419	136.0394	neg	Anthranilic acid	C7H7NO2	137.0477	227	-	-	-	-
metab_1372	0.8221	136.0615	pos	Adenine	C5H5N5	135.0546	190	-	-	-	-
metab_5896	1.4569	136.0615	pos	Adenine	C5H5N5	135.0546	190	-	-	-	-
metab_7496	1.7729	137.0234	neg	4-Hydroxybenzoic acid	C7H6O3	138.0317	135	-	-	-	-
metab_14924	0.5431	137.0346	neg	Urocanic acid	C6H6N2O2	138.0429	736715	Azoles	Imidazoles	HMDB0000301	Urocanic acid
metab_589	4.3371	137.0594	pos	2-(4-Hydroxyphenyl)ethanol	C8H10O2	136.0525	10393	-	-	-	-
metab_5482	2.2361	137.0595	pos	2-(4-Hydroxyphenyl)ethanol	C8H10O2	136.0525	10393	-	-	-	-
metab_8038	0.9649	138.0186	neg	4-Nitrophenol	C6H5NO3	139.0270	980	Pyridines and derivatives	Pyridinecarboxylic acids and derivatives	HMDB0013188	3-Hydroxypicolinic acid
metab_8511	1.8371	138.0187	neg	4-Nitrophenol	C6H5NO3	139.0270	980	-	-	-	-
metab_7683	3.1115	138.0187	neg	4-Nitrophenol	C6H5NO3	139.0270	980	-	-	-	-
metab_6362	0.5983	138.0503	pos	Trigonelline	C7H7NO2	137.0477	5570	Alkaloids and derivatives		HMDB0000875	Trigonelline
metab_6362	0.5983	138.0503	pos	Anthranilic acid	C7H7NO2	137.0477	227	Benzene and substituted derivatives	Fatty acids and conjugates	HMDB0001123	Anthranilic acid, monosodium salt
metab_6362	0.5983	138.0503	pos	Trigonelline	C7H7NO2	137.0477	5570	Alkaloids and derivatives		HMDB0000875	Trigonelline
metab_1296	0.5983	138.0546	pos	Trigonelline	C7H7NO2	137.0477	5570	-	-	-	-
metab_8400	1.6335	138.0551	neg	3-Amino-2,3-dihydrobenzoic acid	C7H9NO2	139.0634	3445	-	-	-	-
metab_2777	7.6776	139.0750	pos	(2E,4E,6E)-7-Hydroxy-4-methylhepta-2,4,6-trienal	C8H10O2	138.0681	70678836	-	-	-	-

metab_4547	5.9361	139.0750	pos	(2E,4E,6E)-7-Hydroxy-4-methylhepta-2,4,6-trienal	C8H10O2	138.0681	70678836	-	-	-	-
metab_4731	4.9267	139.0750	pos	(2E,4E,6E)-7-Hydroxy-4-methylhepta-2,4,6-trienal	C8H10O2	138.0681	70678836	-	-	-	-
metab_5015	3.6684	139.0751	pos	(2E,4E,6E)-7-Hydroxy-4-methylhepta-2,4,6-trienal	C8H10O2	138.0681	70678836	-	-	-	-
metab_1741	1.9463	139.0751	pos	(2E,4E,6E)-7-Hydroxy-4-methylhepta-2,4,6-trienal	C8H10O2	138.0681	70678836	-	-	-	-
metab_1793	2.0828	139.0751	pos	(2E,4E,6E)-7-Hydroxy-4-methylhepta-2,4,6-trienal	C8H10O2	138.0681	70678836	-	-	-	-
metab_4450	6.4639	139.1114	pos	5-Isopropylbicyclo[3.1.0]hexan-2-one	C9H14O	138.1045	92784	Carbonyl compounds	Ketones	HMDB0035229	5-Isopropylbicyclo[3.1.0]hexan-2-one
metab_1365	0.7941	140.0338	pos	3-Nitrophenol	C6H5NO3	139.0269	11137	-	-	-	-
metab_235	0.7941	142.0495	pos	Gentianaine	C6H7NO3	141.0427	135438604	-	-	-	-
metab_314	1.3865	142.0859	pos	Arecaidine	C7H11NO2	141.0790	10355	-	-	HMDB0030352	Arecaidine
metab_3406	14.5638	142.1223	pos	Pelletierine	C8H15NO	141.1155	92987	-	-	-	-
metab_3998	8.6132	142.1223	pos	Pelletierine	C8H15NO	141.1155	92987	-	-	-	-
metab_4098	8.2143	142.1223	pos	Pelletierine	C8H15NO	141.1155	92987	-	-	-	-
metab_1102	1.1875	142.1223	pos	Pelletierine	C8H15NO	141.1155	92987	-	-	-	-
metab_2210	3.7148	142.1224	pos	Pelletierine	C8H15NO	141.1155	92987	-	-	-	-
metab_433	2.2519	142.1224	pos	Pelletierine	C8H15NO	141.1155	92987	-	-	-	-
metab_13391	3.0604	143.0705	neg	7-Oxoheptanoic acid	C7H12O3	144.0786	169732	-	-	-	-
metab_14232	1.5439	143.0705	neg	7-Oxoheptanoic acid	C7H12O3	144.0786	169732	-	-	-	-
metab_9298	4.0859	143.1068	neg	Octanoic acid	C8 H16 O2	144.1150	379	-	-	-	-
metab_9048	3.1964	143.1069	neg	Octanoic acid	C8 H16 O2	144.1150	379	-	-	-	-

metab_1246	0.5140	143.1176	pos	Cycloheptanecarboxylic acid	C8H14O2	142.0993	15091	-	-	-	-
metab_5467	2.2519	143.1257	pos	2-n-Propyl-3-pentenoic acid	C8H14O2	142.0994	24892812	Fatty Acyls	Fatty acid esters	HMDB0013903	(3Z)-2-Propylpent-3-enoic acid
metab_1435	1.0180	144.0652	pos	2-Hydroxymethylclavam	C6H9NO3	143.0583	3050259	-	-	-	-
metab_14637	0.9508	144.0656	neg	L-Allysine	C6H11NO3	145.0739	160603	-	-	-	-
metab_14236	1.5439	144.0657	neg	L-Allysine	C6H11NO3	145.0739	160603	Carboxylic acids and derivatives	Amino acids, peptides, and analogues	HMDB0032862	2-Amino-4-ethoxy-3-hydroxybutanoic acid
metab_5668	1.9014	144.1015	pos	(2R)-1,1-Dimethylpyrrolidin-1-ium-2-carboxylate	C7H13NO2	143.0947	7016562	-	-	-	-
metab_4996	3.7297	144.1380	pos	Pseudoconhydrine	C8H17NO	143.1310	120641	-	-	-	-
metab_2339	4.5027	144.1380	pos	Pseudoconhydrine	C8H17NO	143.1310	120641	-	-	-	-
metab_7983	0.7818	145.0397	neg	Coumarin	C9H6O2	148.0525	323	Coumarins and derivatives	Benzoic acids and derivatives	HMDB0001218	2H-1-Benzopyran-2-one
metab_8516	1.8524	145.0497	neg	Adipic acid	C6H10O4	146.0579	196	Fatty Acyls	Fatty acids and conjugates	HMDB0000448; LMFA01170048	Adipic acid
metab_1685	1.7271	145.0837	pos	Cyclohexylammonium ion	C6H14N+	100.1126	1549093	Organonitrogen compounds	Cyclohexylamines	HMDB0062716	Cyclohexylammonium
metab_14293	1.4856	145.0861	neg	7-Hydroxyheptanoic acid	C7H14O3	146.0943	138016	-	-	-	-
metab_13222	3.5475	145.0861	neg	7-Hydroxyheptanoic acid	C7H14O3	146.0943	138016	-	-	-	-
metab_5276	2.7804	146.0597	pos	Indole-3-carboxaldehyde	C9H7NO	145.0527	10256	-	-	-	-
metab_6351	0.6123	146.1171	pos	4-Trimethylammoniobutanoate	C7H15NO2	145.1104	134	-	-	-	-
metab_5817	1.5847	146.1171	pos	4-Trimethylammoniobutanoate	C7H15NO2	145.1104	134	-	-	-	-
metab_1799	2.0973	146.1172	pos	4-Trimethylammoniobutanoate	C7H15NO2	145.1104	134	-	-	-	-
metab_6492	0.5140	146.1648	pos	Spermidine	C7H19N3	145.1579	1102	Organonitrogen compounds	Amines	HMDB0001257	Spermidine
metab_2235	3.8511	147.0437	pos	Coumarin	C9H6O2	146.0367	323	-	-	-	-

metab_5145	3.1799	147.0437	pos	Coumarin	C9H6O2	146.0367	323	-	-	-	-
metab_5530	2.1444	147.0437	pos	Coumarin	C9H6O2	146.0367	323	-	-	-	-
metab_14473	1.2805	147.0443	neg	p-Coumaraldehyde	C9H8O2	148.0524	641301	-	-	-	-
metab_14473	1.2805	147.0443	neg	Coumarin	C9H6O2	148.0525	323	-	-	-	-
metab_5690	1.8134	147.0549	pos	Coumarin	C9H6O2	148.0525	323	Coumarins and derivatives	Benzoic acids and derivatives	HMDB0001218	2H-1-Benzopyran-2-one
metab_6301	0.6542	147.0760	pos	Glutamine	C5H10N2O3	146.0691	5961	Carboxylic acids and derivatives	Amino acids, peptides, and analogues	HMDB0000641; HMDB0003423	L-Glutamine;D-Glutamine
metab_1743	1.9463	147.0800	pos	7-Hydroxyheptanoic acid	C7H14O3	146.0943	138016	-	-	-	-
metab_1743	1.9463	147.0800	pos	Coumarin	C9H6O2	148.0525	323	Coumarins and derivatives	Benzoic acids and derivatives	HMDB0001218	2H-1-Benzopyran-2-one
metab_14798	0.6131	149.0445	neg	L-Arabinose	C5H10O5	150.0527	439195	-	-	-	-
metab_70	1.2725	149.0593	pos	p-Coumaraldehyde	C9H8O2	148.0524	641301	-	-	-	-
metab_2918	8.6132	149.1321	pos	Ectocarpen	C11H16	148.1251	12491370	-	-	-	-
metab_4346	7.0082	149.1321	pos	Ectocarpen	C11H16	148.1251	12491370	-	-	-	-
metab_132	6.2522	149.1321	pos	Ectocarpen	C11H16	148.1251	12491370	-	-	-	-
metab_4675	5.2129	149.1321	pos	Ectocarpen	C11H16	148.1251	12491370	-	-	-	-
metab_1010	4.9267	149.1322	pos	Ectocarpen	C11H16	148.1251	12491370	-	-	-	-
metab_11098	14.0117	150.0413	neg	Guanine	C5 H5 N5 O	151.0493	135398634	-	-	-	-
metab_8683	2.2272	151.0392	neg	Vanillin	C8H8O3	152.0473	1183	-	-	-	-
metab_14034	1.8205	151.0392	neg	Vanillin	C8H8O3	152.0473	1183	-	-	-	-
metab_7441	1.5297	151.0392	neg	Vanillin	C8H8O3	152.0473	1183	-	-	-	-
metab_8448	1.7104	151.0426	neg	Vanillin	C8H8O3	152.0473	1183	Phenols	Fatty amides	HMDB0012308	4-Hydroxy-3-methoxybenzaldehyde (acd/name 4.0)
metab_14838	0.5991	151.0603	neg	Xylitol	C5H12O5	152.0685	6912	Organooxygen	Carbohydrates and	HMDB0000508	Ribitol

								compounds	carbohydrate conjugates		
metab_6225	0.8361	152.0563	pos	Guanine	C5H5N5O	151.0494	135398634	Imidazopyrimidines	Purines and purine derivatives	HMDB0000132	Guanine
metab_1756	1.9918	152.0703	pos	2-Amino-2-phenylacetic acid	C8H9NO2	151.0633	3866	Carboxylic acids and derivatives	Amino acids, peptides, and analogues	HMDB0002210	2-Phenylglycine
metab_5050	3.5162	152.1430	pos	2-Methyl-3-amylpyrrole	C10H17N	151.1360	3082128	-	-	-	-
metab_14375	1.4115	153.0185	neg	2,5-Dihydroxybenzoic acid	C7H6O4	154.0266	3469	Benzene and substituted derivatives	Benzoic acids and derivatives	HMDB0000152	Gentisic acid
metab_14375	1.4115	153.0185	neg	2,3-Dihydroxybenzoic acid	C7H6O4	154.0266	19	Benzene and substituted derivatives	Benzoic acids and derivatives	HMDB0000152	Gentisic acid
metab_1759	1.9918	153.0907	pos	3-Isopropylcatechol	C9H12O2	152.0837	16498	-	-	-	-
metab_1849	2.2519	153.0907	pos	3-Isopropylcatechol	C9H12O2	152.0837	16498	-	-	-	-
metab_5969	1.3153	155.0334	pos	Diethylphosphoric acid	C4H11O4P	154.0394	654	Organic phosphoric acids and derivatives	Glycerophosphocholines	HMDB0012209	Diethylphosphate
metab_6079	1.1447	155.0334	pos	Diethylphosphoric acid	C4H11O4P	154.0394	654	Organic phosphoric acids and derivatives	Glycerophosphocholines	HMDB0012209	Diethylphosphate
metab_13911	2.0202	155.0341	neg	(2S)-2-Isopropyl-3-oxosuccinate	C7H10O5	174.0528	5462259	Keto acids and derivatives	Short-chain keto acids and derivatives	HMDB0012149	2-Isopropyl-3-oxosuccinate
metab_6085	1.1306	155.0446	pos	Diethyl hydrogen phosphate	C4H11O4P	154.0394	654	-	-	-	-
metab_5820	1.5705	155.0447	pos	Diethyl hydrogen phosphate	C4H11O4P	154.0394	654	-	-	-	-
metab_14655	0.8946	155.0816	neg	Nonane-4,6-dione	C9H16O2	156.115	26454	-	-	-	-
metab_6921	3.6142	155.1069	neg	Nonane-4,6-dione	C9H16O2	156.1150	26454	-	-	-	-
metab_6449	0.5280	156.0764	pos	Histidine	C6H9N3O2	155.0695	6274	Carboxylic acids and derivatives	Amino acids, peptides, and analogues	HMDB0000177	L-Histidine
metab_5727	1.7554	156.1015	pos	Arecoline	C8H13NO2	155.0946	2230	-	-	-	-
metab_3999	8.6132	156.1379	pos	N-Methylpelletierine	C9H17NO	155.1310	1548928	-	-	-	-

metab_763	8.2143	156.1379	pos	N-Methylpelletierine	C9H17NO	155.1310	1548928	-	-	-	-
metab_1557	1.3865	156.1380	pos	N-Methylpelletierine	C9H17NO	155.1310	1548928	-	-	-	-
metab_1795	2.0973	156.1380	pos	N-Methylpelletierine	C9H17NO	155.1310	1548928	-	-	-	-
metab_5307	2.6719	156.1380	pos	N-Methylpelletierine	C9H17NO	155.1310	1548928	-	-	-	-
metab_5106	3.3183	156.1380	pos	N-Methylpelletierine	C9H17NO	155.1310	1548928	-	-	-	-
metab_5947	1.3437	157.0491	pos	4-Methyl-3-oxoadipate-enol-lactone	C7H8O4	156.0421	44123583	-	-	-	-
metab_1447	1.0601	157.0492	pos	4-Methyl-3-oxoadipate-enol-lactone	C7H8O4	156.0421	44123583	-	-	-	-
metab_5469	2.2519	157.0492	pos	4-Methyl-3-oxoadipate-enol-lactone	C7H8O4	156.0421	44123583	-	-	-	-
metab_1703	1.7705	157.0603	pos	4-Methyl-3-oxoadipate-enol-lactone	C7H8O4	156.0421	44123583	-	-	-	-
metab_8765	2.4175	157.0862	neg	4-Oxo-2-propylpentanoic acid	C8H14O3	158.0942	3731686	-	-	-	-
metab_8565	1.9596	157.0862	neg	4-Oxo-2-propylpentanoic acid	C8H14O3	158.0942	3731686	-	-	-	-
metab_4585	5.7540	157.1220	pos	Nonane-4,6-dione	C9H16O2	156.115	26454	-	-	-	-
metab_3567	11.2827	158.1534	pos	2,2,6,6-Tetramethyl-4-piperidinol	C9 H19 N O	157.1466	75471	-	-	-	-
metab_2601	6.4948	158.1534	pos	2,2,6,6-Tetramethyl-4-piperidinol	C9 H19 N O	157.1466	75471	-	-	-	-
metab_3280	14.0378	158.1535	pos	2,2,6,6-Tetramethyl-4-piperidinol	C9 H19 N O	157.1466	75471	-	-	-	-
metab_3729	9.7899	158.1536	pos	2,2,6,6-Tetramethyl-4-piperidinol	C9 H19 N O	157.1466	75471	-	-	-	-
metab_4740	4.9267	158.1536	pos	2,2,6,6-Tetramethyl-4-piperidinol	C9 H19 N O	157.1466	75471	-	-	-	-

metab_7524	1.9125	159.0654	neg	3-Methyladipic acid	C7H12O4	160.0736	12292	Fatty Acyls	Fatty acids and conjugates	HMDB0000555	3-Methyladipic acid
metab_7524	1.9125	159.0654	neg	Pimelic acid	C7H12O4	160.0735	385	Fatty Acyls	Fatty acids and conjugates	HMDB0000555	3-Methyladipic acid
metab_6130	1.0461	159.0760	pos	4-Methylene-L-glutamine	C6H10N2O3	158.0691	439401	-	-	-	-
metab_345	1.5284	159.0912	pos	1,5-Naphthalenediamine	C10H10N2	158.0843	16720	-	-	-	-
metab_13986	1.8682	159.1018	neg	(R)-2-Hydroxycaprylic acid	C8H16O3	160.1099	5312860	-	-	-	-
metab_8845	2.6206	159.1019	neg	(R)-2-Hydroxycaprylic acid	C8H16O3	160.1099	5312860	-	-	-	-
metab_9374	4.4386	159.1019	neg	(R)-2-Hydroxycaprylic acid	C8H16O3	160.1099	5312860	-	-	-	-
metab_9240	3.8678	159.1019	neg	(R)-2-Hydroxycaprylic acid	C8H16O3	160.1099	5312860	-	-	-	-
metab_6313	0.6262	159.1206	pos	1,5-Naphthalenediamine	C10H10N2	158.0843	16720	-	-	-	-
metab_6313	0.6262	159.1206	pos	Nicotyrine	C10H10N2	158.0843	10249	-	-	-	-
metab_6257	0.7801	160.1076	pos	delta-Guanidinovaleric acid	C6H13N3O2	159.1008	160464	-	-	-	-
metab_6268	0.7801	160.1328	pos	3-Aminooctanoic acid	C8H17NO2	159.1259	295606	-	-	-	-
metab_14647	0.9227	161.0447	neg	Meglutol	C6H10O5	162.0528	1662	Fatty Acyls	Fatty acids and conjugates	HMDB0059737; HMDB0000355	3-Methyl-3-hydroxypentanedioate;3-Hydroxymethylglutaric acid
metab_9138	3.5310	161.0599	neg	trans-2-Phenylcyclopropanecarboxylic acid	C10H10O2	162.0681	237413	-	-	-	-
metab_13529	2.7475	161.0599	neg	trans-2-Phenylcyclopropanecarboxylic acid	C10H10O2	162.0681	237413	-	-	-	-
metab_9098	3.3631	161.0600	neg	trans-2-Phenylcyclopropanecarboxylic acid	C10H10O2	162.0681	237413	-	-	-	-
metab_6616	1.1940	161.0811	neg	Oleandrose	C7H14O4	162.0891	5461155	-	-	-	-
metab_14134	1.6642	161.0811	neg	Oleandrose	C7H14O4	162.0891	5461155	-	-	-	-
metab_373	1.7271	161.1069	pos	L-alpha-Amino-1H-pyrrole-1-	C10H16N2O2	196.1212	15800938	Carboxylic acids and	Amino acids, peptides,	HMDB0040551	L-alpha-Amino-1H-pyrrole-1-hexan

				hexanoic acid				derivatives	and analogues		oic acid
metab_5354	2.5314	162.0910	pos	4,7-Octadienoylglycine	C10H15NO3	197.1052	131802977	Carboxylic acids and derivatives	Amino acids, peptides, and analogues	HMDB0094797	4,7-octadienoylglycine
metab_156	0.5983	162.1120	pos	Levocarnitine	C7H15NO3	161.1052	10917	Organonitrogen compounds	Quaternary ammonium salts	HMDB0000062	L-Carnitine
metab_8731	2.3380	163.0393	neg	4-Hydroxycinnamic acid	C9H8O3	164.0474	637542	Cinnamic acids and derivatives	Hydroxycinnamic acids and derivatives	HMDB0001713	m-Coumaric acid
metab_709	7.0670	163.1114	pos	Valerophenone	C11H14O	162.1044	66093	-	-	-	-
metab_619	4.9267	163.1114	pos	Valerophenone	C11H14O	162.1044	66093	-	-	-	-
metab_1503	1.2016	163.1225	pos	Nicotine	C10H14N2	162.1156	89594	-	-	-	-
metab_5908	1.4289	163.1226	pos	Nicotine	C10H14N2	162.1156	89594	-	-	-	-
metab_13607	2.6046	164.0345	neg	2-(Formylamino)benzoic acid	C8H7NO3	165.0426	101399	-	-	-	-
metab_14475	1.2805	164.0709	neg	Phenylalanine	C9H11NO2	165.0790	6140	Carboxylic acids and derivatives	Amino acids, peptides, and analogues	HMDB0000159	L-Phenylalanine
metab_1082	1.3153	164.1064	pos	Eicosapentaenoic acid	C10H13NO	163.0996	446284	-	-	-	-
metab_2100	3.2420	164.1067	pos	Eicosapentaenoic acid	C10H13NO	163.0996	446284	-	-	-	-
metab_5231	2.9192	164.1067	pos	Eicosapentaenoic acid	C10H13NO	163.0996	446284	Organooxygen compounds	Alcohols and polyols	HMDB0003072	Quinic acid
metab_8541	1.9125	165.0185	neg	Terephthalic acid	C8H6O4	166.0265	7489	-	-	-	-
metab_14171	1.6028	165.0186	neg	Terephthalic acid	C8H6O4	166.0265	7489	-	-	-	-
metab_14845	0.5991	165.0396	neg	D-Erythrose	C4H8O4	120.0423	94176	Organooxygen compounds	Carbohydrates and carbohydrate conjugates	HMDB0002649	Erythrose
metab_14845	0.5991	165.0396	neg	L-Lyxonic acid	C5H10O6	166.0476	644110	Organooxygen compounds	Carbohydrates and carbohydrate conjugates	HMDB0002649	Erythrose
metab_10639	10.3031	165.0400	neg	L-Lyxonic acid	C5H10O6	166.0476	644110	-	-	-	-

metab_11314	9.9767	165.0400	neg	L-Lyxonic acid	C5H10O6	166.0476	644110	-	-	-	-
metab_11507	9.2834	165.0401	neg	L-Lyxonic acid	C5H10O6	166.0476	644110	-	-	-	-
metab_243	0.8221	165.0542	pos	Coumarinic acid	C9H8O3	164.0473	5280841	Cinnamic acids and derivatives	Hydroxycinnamic acids and derivatives	HMDB0134028; HMDB0062655; HMDB0002641	3-(2-hydroxyphenyl)prop-2-enoic acid;Cis-2-coumarate;2-Hydroxycinnamic acid
metab_7610	2.5264	165.0549	neg	L-(-)-3-Phenyllactic acid	C9H10O3	166.0628	444718	Phenylpropanoic acids	-	HMDB0000779	Phenyllactic acid
metab_4546	5.9504	165.1270	pos	4-Pentylphenol	C11H16O	164.1201	26975	-	-	-	-
metab_72	1.2725	166.0858	pos	Phenylalanine	C9H11NO2	165.0790	6140	-	-	-	-
metab_1220	0.4999	166.1134	pos	3-Methylguanine	C6H7N5O	165.065	76292	Imidazopyrimidines		HMDB0001566	7-dihydro-3-Methyl-2-amino-3-6H-purin-6-one
metab_5901	1.4429	166.1222	pos	3-Methylguanine	C6H7N5O	165.065	76292	Imidazopyrimidines		HMDB0001566	7-dihydro-3-Methyl-2-amino-3-6H-purin-6-one
metab_381	1.7845	167.0335	pos	Terephthalic acid	C8H6O4	166.0265	7489	Benzene and substituted derivatives	O-methylated isoflavonoids	HMDB0002428	TPA
metab_6820	0.9933	167.0341	neg	2',4',6'-Trihydroxyacetophenone	C8H8O4	168.0422	68073	-	-	-	-
metab_6820	0.9933	167.0341	neg	2,6-Dimethoxy-1,4-benzoquinone	C8H8O4	168.0422	68262	-	-	-	-
metab_6820	0.9933	167.0341	neg	Vanillate	C8H8O4	168.0421	54675858	-	-	-	-
metab_8831	2.5893	167.0342	neg	2',4',6'-Trihydroxyacetophenone	C8H8O4	168.0422	68073	-	-	-	-
metab_8831	2.5893	167.0342	neg	2,6-Dimethoxy-1,4-benzoquinone	C8H8O4	168.0422	68262	-	-	-	-
metab_8831	2.5893	167.0342	neg	Vanillate	C8H8O4	168.0421	54675858	-	-	-	-
metab_14202	1.5736	167.0342	neg	5-Methoxysalicylic acid	C8H8O4	168.0423	75787	Benzene and substituted derivatives	Benzoic acids and derivatives	HMDB0001868	5-Methoxysalicylic acid

metab_14202	1.5736	167.0342	neg	2',4',6'-Trihydroxyacetophenone	C8H8O4	168.0422	68073	Benzene and substituted derivatives	Benzoic acids and derivatives	HMDB0001868	5-Methoxysalicylic acid
metab_14202	1.5736	167.0342	neg	2,6-Dimethoxy-1,4-benzoquinone	C8H8O4	168.0422	68262	Benzene and substituted derivatives	Benzoic acids and derivatives	HMDB0001868	5-Methoxysalicylic acid
metab_14202	1.5736	167.0342	neg	Vanillate	C8H8O4	168.0421	54675858	Benzene and substituted derivatives	Benzoic acids and derivatives	HMDB0001868	5-Methoxysalicylic acid
metab_1486	1.1589	167.0698	pos	4-Ipomeanol	C9H12O3	166.0630	36284	-	-	-	-
metab_1486	1.1589	167.0698	pos	Homovanillin	C9H10O3	166.0629	151276	-	-	-	-
metab_4606	5.6322	167.0699	pos	4-Ipomeanol	C9H12O3	166.0630	36284	-	-	-	-
metab_4606	5.6322	167.0699	pos	Homovanillin	C9H10O3	166.0629	151276	-	-	-	-
metab_1750	1.9609	167.0699	pos	4-Ipomeanol	C9H12O3	166.0630	36284	-	-	-	-
metab_1750	1.9609	167.0699	pos	Homovanillin	C9H10O3	166.0629	151276	-	-	-	-
metab_5807	1.5987	167.0699	pos	4-Ipomeanol	C9H12O3	166.0630	36284	-	-	-	-
metab_5807	1.5987	167.0699	pos	Homovanillin	C9H10O3	166.0629	151276	-	-	-	-
metab_4674	5.2129	167.1426	pos	3,3,5,5-Tetramethylnorbornan-2-one	C11H18O	166.1357	46173325	-	-	-	-
metab_2929	8.6284	167.1426	pos	3,3,5,5-Tetramethylnorbornan-2-one	C11H18O	166.1357	46173325	-	-	-	-
metab_4104	8.1857	167.1427	pos	3,3,5,5-Tetramethylnorbornan-2-one	C11H18O	166.1357	46173325	-	-	-	-
metab_8387	1.6028	169.0135	neg	Gallic acid	C7H6O5	170.0215	370	Benzene and substituted derivatives	Benzoic acids and derivatives	HMDB0005807	Gallic acid
metab_5818	1.5847	169.0966	pos	3,4-Dihydroxy-2-hydroxymethyl-1-pyrrolidinepropanamide	C8H16N2O4	204.1110	85303757	Pyrrolidines	N-alkylpyrrolidines	HMDB0039948	3,4-Dihydroxy-2-hydroxymethyl-1-pyrrolidinepropanamide
metab_1883	2.3751	169.1220	pos	Geranic acid	C10H16O2	168.1150	5275520	-	-	-	-
metab_5265	2.8108	169.1220	pos	Geranic acid	C10H16O2	168.1150	5275520	-	-	-	-

metab_6946	4.2203	169.1226	neg	9-Decenoic acid	C10H18O2	170.1307	61743	-	-	-	-
metab_9093	3.3465	169.1227	neg	9-Decenoic acid	C10H18O2	170.1307	61743	-	-	-	-
metab_12842	4.7881	169.1227	neg	9-Decenoic acid	C10H18O2	170.1307	61743	-	-	-	-
metab_13087	3.9005	169.1227	neg	9-Decenoic acid	C10H18O2	170.1307	61743	-	-	-	-
metab_1124	0.6262	170.0919	pos	N-Methyl-L-histidine	C7H11N3O2	169.0852	90638	-	-	-	-
metab_5813	1.5987	170.1170	pos	Piperidione	C9H15NO2	169.1101	6465	-	-	-	-
metab_480	2.7497	170.1172	pos	Piperidione	C9H15NO2	169.1101	6465	-	-	-	-
metab_5477	2.2519	170.1172	pos	Piperidione	C9H15NO2	169.1101	6465	-	-	-	-
metab_4000	8.6132	170.1534	pos	Tetryl	C10H19NO	169.1465	10178	-	-	-	-
metab_4937	3.9876	170.1535	pos	Tetryl	C10H19NO	169.1465	10178	-	-	-	-
metab_2844	8.2143	170.1535	pos	Tetryl	C10H19NO	169.1465	10178	-	-	-	-
metab_6040	1.2016	171.0647	pos	2,5-Hexadienoic acid, 3-methoxy-5-methyl-4-oxo-	C8H10O4	170.0579	1268111	-	-	-	-
metab_7587	2.3533	171.0656	neg	4,7-Dioxooctanoic acid	C8H12O4	172.0736	244084	-	-	-	-
metab_8440	1.7104	171.0656	neg	4,7-Dioxooctanoic acid	C8H12O4	172.0736	244084	-	-	-	-
metab_13428	2.9931	171.0656	neg	4,7-Dioxooctanoic acid	C8H12O4	172.0736	244084	-	-	-	-
metab_14037	1.8046	171.1019	neg	9-Oxononanoic acid	C9H16O3	172.1099	75704	-	-	-	-
metab_9752	6.4709	171.1019	neg	9-Oxononanoic acid	C9H16O3	172.1099	75704	-	-	-	-
metab_8785	2.4487	171.1019	neg	9-Oxononanoic acid	C9H16O3	172.1099	75704	-	-	-	-
metab_9604	5.6615	171.1019	neg	9-Oxononanoic acid	C9H16O3	172.1099	75704	-	-	-	-
metab_9438	4.7217	171.1020	neg	9-Oxononanoic acid	C9H16O3	172.1099	75704	-	-	-	-
metab_9314	4.1535	171.1020	neg	9-Oxononanoic acid	C9H16O3	172.1099	75704	-	-	-	-
metab_13272	3.3803	171.1020	neg	9-Oxononanoic acid	C9H16O3	172.1099	75704	-	-	-	-
metab_13157	3.7148	171.1020	neg	9-Oxononanoic acid	C9H16O3	172.1099	75704	-	-	-	-
metab_7678	3.0432	171.1020	neg	9-Oxononanoic acid	C9H16O3	172.1099	75704	-	-	-	-

metab_13707	2.4010	172.0972	neg	Acetylleucine	C8H15NO3	173.1052	1995	Carboxylic acids and derivatives	Amino acids, peptides, and analogues	HMDB0011756	N-Acetylleucine
metab_13707	2.4010	172.0972	neg	N-Acetyl-L-leucine	C8H15NO3	173.1052	70912	Carboxylic acids and derivatives	Amino acids, peptides, and analogues	HMDB0011756	N-Acetylleucine
metab_1594	1.4860	172.1326	pos	Gabapentin	C9H17NO2	171.1258	3446	-	-	-	-
metab_6120	1.0601	172.1327	pos	Gabapentin	C9H17NO2	171.1258	3446	-	-	-	-
metab_1939	2.5938	172.1328	pos	Gabapentin	C9H17NO2	171.1258	3446	-	-	-	-
metab_1874	2.3447	172.1328	pos	Gabapentin	C9H17NO2	171.1258	3446	-	-	-	-
metab_4573	5.8148	172.1691	pos	Decanamide	C10 H21 N O	171.1622	75347	-	-	-	-
metab_14727	0.7818	173.0084	neg	Aconitic acid	C6H6O6	174.0164	309	Carboxylic acids and derivatives	Tricarboxylic acids and derivatives	HMDB0000958; HMDB0000072	trans-Aconitic acid;cis-Aconitic acid
metab_8078	1.0785	173.0812	neg	Suberic acid	C8H14O4	174.0891	10457	-	-	-	-
metab_8800	2.5113	173.0812	neg	Suberic acid	C8H14O4	174.0891	10457	-	-	-	-
metab_14839	0.5991	173.0923	neg	N-Acetylornithine	C7H14N2O3	174.1004	439232	Carboxylic acids and derivatives	Amino acids, peptides, and analogues	HMDB0003357	N-Acetylornithine
metab_2597	6.4639	173.1168	pos	9-Oxononanoic acid	C9H16O3	172.1099	75704	-	-	-	-
metab_621	4.9413	173.1168	pos	9-Oxononanoic acid	C9H16O3	172.1099	75704	-	-	LMFA01050232	
metab_1871	2.3292	173.1169	pos	9-Oxononanoic acid	C9H16O3	172.1099	75704	-	-	-	-
metab_13019	4.1364	173.1176	neg	2-Hydroxynonanoic acid	C9H18O3	174.1255	5282897	-	-	-	-
metab_9429	4.6722	173.1176	neg	2-Hydroxynonanoic acid	C9H18O3	174.1255	5282897	-	-	-	-
metab_1369	0.7941	173.1392	pos	9-Oxononanoic acid	C9H16O3	172.1099	75704	-	-	-	-
metab_6823	0.9792	174.0764	neg	Calystegin B2	C7H13NO4	175.0843	443000	-	-	-	-
metab_6027	1.2162	174.1120	pos	N-Acetyl-L-leucine	C8H15NO3	173.1052	70912	-	-	-	-
metab_6258	0.7801	174.1120	pos	N-Acetyl-L-leucine	C8H15NO3	173.1052	70912	-	-	-	-
metab_5411	2.4071	174.1121	pos	N-Acetyl-L-leucine	C8H15NO3	173.1052	70912	-	-	-	-

metab_6208	0.8640	174.1232	pos	Indospicine	C7H15N3O2	173.1164	108010	-	-	-	-
metab_6205	0.8640	174.1484	pos	9-Aminononanoic acid	C9H19NO2	173.1416	136877	-	-	-	-
metab_1455	1.0883	174.1484	pos	9-Aminononanoic acid	C9H19NO2	173.1416	136877	-	-	-	-
metab_5302	2.6869	174.1485	pos	9-Aminononanoic acid	C9H19NO2	173.1416	136877	-	-	-	-
metab_7500	1.8046	175.0605	neg	2-Isopropylmalic acid	C7H12O5	176.0685	77	Fatty Acyls	Fatty acids and conjugates	HMDB0000402	2-Isopropylmalic acid
metab_4968	3.8511	175.1114	pos	Capillanol	C12H14O	174.1044	5315669	-	-	-	-
metab_1153	0.5420	175.1185	pos	Arginine	C6H14N4O2	175.1195	6322	Carboxylic acids and derivatives	Amino acids, peptides, and analogues	HMDB0062762; HMDB0000517	L-argininium(1+);L-Arginine
metab_1562	1.4005	175.1224	pos	alpha-Methyltryptamine	C11H14N2	174.1157	9287	-	-	-	-
metab_4089	8.2435	175.1477	pos	1,1,6-Trimethyltetralin	C13H18	174.1409	68057	-	-	-	-
metab_4532	5.9949	175.1477	pos	1,1,6-Trimethyltetralin	C13H18	174.1409	68057	-	-	-	-
metab_1146	0.5983	176.0103	pos	2-Amino-5-chloro-cis,cis-muconic 6-semialdehyde	C6H6ClNO3	175.0036	25245566	-	-	-	-
metab_5196	3.0114	176.0702	pos	Indole-3-acetic acid	C10H9NO2	175.0633	802	Indoles and derivatives	Indolyl carboxylic acids and derivatives	HMDB0000197	Indoleacetic acid
metab_3369	15.1520	177.0541	pos	7-Methoxycoumarin	C10H8O3	176.0473	10748	-	-	-	-
metab_170	0.2713	177.0542	pos	7-Methoxycoumarin	C10H8O3	176.0473	10748	-	-	-	-
metab_13738	2.3227	177.0551	neg	(3S)-3-Methyl-2-oxo-3-phenylpropanoate	C10H10O3	178.0629	71581073	-	-	-	-
metab_13380	3.0774	177.0551	neg	(3S)-3-Methyl-2-oxo-3-phenylpropanoate	C10H10O3	178.0629	71581073	-	-	-	-
metab_652	4.9267	177.1270	pos	Benzenepropanal, 4-(1-methylethyl)-	C12H16O	176.1201	62654	Prenol lipids	Monoterpenoids	HMDB0036171	3-(4-Isopropylphenyl)propanal
metab_13488	2.8440	178.0504	neg	4-Acetamidobenzoic acid	C9H9NO3	179.0582	19266	-	-	-	-

metab_1016	2.1897	178.0859	pos	Plantagonine	C10H11NO2	177.0790	12300213	Pyridines and derivatives	Pyridinecarboxylic acids and derivatives	HMDB0033482	Plantagonine
metab_7517	1.8682	179.0343	neg	Caffeic acid	C9H8O4	180.0423	689043	Cinnamic acids and derivatives	Hydroxycinnamic acids and derivatives	HMDB0001964	Caffeic acid
metab_6744	0.6131	179.0554	neg	d-Galactofuranose	C6H12O6	180.0634	15560222	Organooxygen compounds	Alcohols and polyols	HMDB0000211	myo-Inositol
metab_6069	1.1589	179.0698	pos	(3S)-2-Oxo-3-phenylbutanoate	C10H10O3	178.0629	71581073	-	-	-	-
metab_12988	4.2367	179.0706	neg	1-Propanone, 1-(3,4-dihydroxyphenyl)-2-methyl-	C10H12O3	180.0786	21632	-	-	-	-
metab_13271	3.3631	179.0707	neg	1-Propanone, 1-(3,4-dihydroxyphenyl)-2-methyl-	C10H12O3	180.0786	21632	-	-	-	-
metab_14187	1.5736	179.0707	neg	U 0521	C10H12O3	180.0786	21632	-	-	-	-
metab_8948	2.8768	179.0707	neg	1-Propanone, 1-(3,4-dihydroxyphenyl)-2-methyl-	C10H12O3	180.0786	21632	-	-	-	-
metab_4351	7.0082	179.1426	pos	4-Hexylphenol	C12H18O	178.1356	17132	Benzene and substituted derivatives	-	HMDB0031568	4-Methyl-1-phenyl-2-pentanol
metab_662	5.9361	179.1426	pos	4-Hexylphenol	C12H18O	178.1356	17132	-	-	-	-
metab_2894	8.4504	179.1426	pos	4-Hexylphenol	C12H18O	178.1356	17132	-	-	-	-
metab_651	4.9413	179.1426	pos	4-Hexylphenol	C12H18O	178.1356	17132	-	-	-	-
metab_14696	0.8240	180.0659	neg	Tyrosine	C9H11NO3	181.0739	6057	-	-	-	-
metab_3375	15.7352	180.0862	pos	D-Glucosamine	C6H13NO5	179.0807	439213	-	-	-	-
metab_1086	1.2725	180.0873	pos	D-Glucosamine	C6H13NO5	179.0807	439213	-	-	-	-
metab_5747	1.7128	180.1015	pos	Salsolinol	C10 H13 N	179.0945	91588	-	-	-	-

					O2						
metab_5698	1.7993	180.1378	pos	(-)-Salsolinol	C10H13NO2	179.0946	91588	Tetrahydroisoquinolines		HMDB0042012	(-)-Salsolinol
metab_6588	0.5711	181.0710	neg	Galactitol	C6H14O6	182.0790	11850	Organooxygen compounds	Carbohydrates and carbohydrate conjugates	HMDB0000107; HMDB0011632	Galactitol;L-Iditol
metab_244	0.8221	182.0807	pos	Tyrosine	C9H11NO3	181.0739	6057	-	-	-	-
metab_1617	1.5424	183.0760	pos	Azatyrosine	C8H10N2O3	182.0692	10986910	-	-	-	-
metab_1617	1.5424	183.0760	pos	Triethyl phosphate	C6H15O4P	182.0707	6535	-	-	-	-
metab_9702	6.2456	183.1384	neg	gamma-Undecalactone	C11H20O2	184.1462	7714	-	-	-	-
metab_12558	6.0831	183.1384	neg	gamma-Undecalactone	C11H20O2	184.1462	7714	-	-	-	-
metab_6963	4.6550	183.1384	neg	gamma-Undecalactone	C11H20O2	184.1462	7714	-	-	-	-
metab_2743	7.4415	184.0729	pos	Phosphocholine	C5H14NO4P	184.0739	1014	Organonitrogen compounds	Quaternary ammonium salts	HMDB0001565	Phosphorylcholine
metab_8857	2.6678	184.0972	neg	5-Hepteneoylglycine	C9H15NO3	185.1052	131802911	Carboxylic acids and derivatives	Amino acids, peptides, and analogues	HMDB0094731	5-Hepteneoylglycine
metab_3409	14.5016	184.1328	pos	Acetylpseudotropine	C10H17NO2	183.1259	103008	-	-	-	-
metab_6551	0.2224	184.1328	pos	Acetylpseudotropine	C10H17NO2	183.1259	103008	-	-	-	-
metab_1092	1.2016	184.1328	pos	Acetylpseudotropine	C10H17NO2	183.1259	103008	-	-	-	-
metab_4840	4.4429	184.1329	pos	Acetylpseudotropine	C10H17NO2	183.1259	103008	-	-	-	-
metab_4960	3.8967	184.1329	pos	Acetylpseudotropine	C10H17NO2	183.1259	103008	-	-	-	-
metab_5956	1.3293	184.1329	pos	Acetylpseudotropine	C10H17NO2	183.1259	103008	-	-	-	-
metab_4001	8.6132	184.1691	pos	Tecostanine	C11H21NO	183.1622	120773	-	-	-	-
metab_4099	8.2143	184.1692	pos	Tecostanine	C11H21NO	183.1622	120773	-	-	-	-
metab_9476	4.8865	185.1176	neg	10-Oxocaprate	C10H18O3	186.1256	19734156	-	-	-	-
metab_9476	4.8865	185.1176	neg	3-Oxodecanoate	C10H18O3	186.1256	22078585	-	-	-	-
metab_12738	5.2419	185.1177	neg	10-Oxocaprate	C10H18O3	186.1256	19734156	-	-	-	-

metab_12738	5.2419	185.1177	neg	3-Oxodecanoate	C10H18O3	186.1256	22078585	-	-	-	-
metab_12971	4.3037	185.1177	neg	10-Oxocaprate	C10H18O3	186.1256	19734156	-	-	-	-
metab_12971	4.3037	185.1177	neg	3-Oxodecanoate	C10H18O3	186.1256	22078585	-	-	-	-
metab_9111	3.4470	185.1177	neg	10-Oxocaprate	C10H18O3	186.1256	19734156	-	-	-	-
metab_9111	3.4470	185.1177	neg	3-Oxodecanoate	C10H18O3	186.1256	22078585	-	-	-	-
metab_8823	2.5592	185.1178	neg	10-Oxocaprate	C10H18O3	186.1256	19734156	-	-	-	-
metab_8823	2.5592	185.1178	neg	3-Oxodecanoate	C10H18O3	186.1256	22078585	-	-	-	-
metab_1402	1.8205	186.0554	neg	3,7,4'-Trihydroxyflavone	C15H10O5	270.0527	5281611	Flavonoids	Flavones	HMDB0034004	4',7-Dihydroxyflavonol
metab_14024	1.8205	186.0554	neg	3-Indoleacrylate	C11H9NO2	187.0632	5375048	-	-	-	-
metab_316	1.3720	186.1119	pos	Pseudoecgonine	C9H15NO3	185.1052	443845	-	-	-	-
metab_431	2.2665	186.1121	pos	Pseudoecgonine	C9H15NO3	185.1052	443845	-	-	-	-
metab_7969	0.7678	187.0717	neg	N-Acetyl-L-glutamine	C7H12N2O4	188.0797	182230	-	-	-	-
metab_1715	1.8277	187.0960	pos	2-(Hydroxymethyl)-6-(propan-2-yloxy)oxane-3,4,5-triol	C9H18O6	222.1103	15613266	Organooxygen compounds	Carbohydrates and carbohydrate conjugates	HMDB0032705	Isopropyl beta-D-glucoside
metab_9047	3.1964	187.0970	neg	Azelaic acid	C9H16O4	188.1049	2266	-	-	-	-
metab_9047	3.1964	187.0970	neg	Azelaic acid	C9H16O4	188.1049	2266	-	-	-	-
metab_13430	2.9760	187.1082	neg	6-Acetamido-3-aminohexanoic acid	C8H16N2O3	188.1161	440139	-	-	-	-
metab_13430	2.9760	187.1082	neg	Ile-gly	C8H16N2O3	188.1161	6992869	-	-	-	-
metab_8302	1.4856	187.1082	neg	6-Acetamido-3-aminohexanoic acid	C8H16N2O3	188.1161	440139	-	-	-	-
metab_8302	1.4856	187.1082	neg	Ile-gly	C8H16N2O3	188.1161	6992869	-	-	-	-
metab_1030	1.9309	187.1324	pos	(5S)-6-Hydroxy-5-isopropenyl-2-methylhexanoate	C10H18O3	186.1255	443191	-	-	-	-
metab_9551	5.3546	187.1334	neg	10-Hydroxydecanoic acid	C10 H20 O3	188.1413	74300	-	-	-	-

metab_9633	5.7913	187.1334	neg	10-Hydroxydecanoic acid	C10 H20 O3	188.1413	74300	-	-	-	-
metab_12922	4.5219	187.1334	neg	10-Hydroxydecanoic acid	C10 H20 O3	188.1413	74300	-	-	-	-
metab_6786	0.8098	188.0558	neg	N-Acetyl-L-glutamic acid	C7H11NO5	189.0637	70914	Carboxylic acids and derivatives	Amino acids, peptides, and analogues	HMDB0001138	N-Acetylglutamic acid
metab_1055	1.5284	188.0701	pos	Indole-3-acrylic acid	C11H9NO2	187.0632	5375048	-	-	-	-
metab_6565	0.0278	188.0701	pos	Indole-3-acrylic acid	C11H9NO2	187.0632	5375048	-	-	-	-
metab_6061	1.1875	188.1276	pos	(E)-2-Butenyl-4-methyl-threonine	C9H17NO3	187.1208	5282047	-	-	-	-
metab_1686	1.7271	188.1640	pos	Decanohydroxamic acid	C10H21NO2	187.1572	142752	-	-	-	-
metab_7608	2.5113	189.0763	neg	(R)-3-[(R)-3-Hydroxybutanoyloxy]butanoic acid	C8H14O5	190.0842	5459971	-	-	-	-
metab_14400	1.3669	189.0763	neg	(R)-3-[(R)-3-Hydroxybutanoyloxy]butanoic acid	C8H14O5	190.0842	5459971	-	-	-	-
metab_5874	1.5004	189.1227	pos	Ile-gly	C8H16N2O3	188.1161	6992869	-	-	-	-
metab_1131	0.6262	189.1228	pos	Ile-gly	C8H16N2O3	188.1161	6992869	-	-	-	-
metab_6175	0.9200	189.1228	pos	Ile-gly	C8H16N2O3	188.1161	6992869	-	-	-	-
metab_1800	2.1132	189.1230	pos	Ile-gly	C8H16N2O3	188.1161	6992869	-	-	-	-
metab_6382	0.5843	189.1341	pos	Tilarginine Acetate	C7H16N4O2	188.1273	135242	Carboxylic acids and derivatives	Amino acids, peptides, and analogues	HMDB0029416	L-Targinine
metab_6448	0.5280	189.1593	pos	N6,N6,N6-Trimethyl-L-lysine	C9H20N2O2	188.1525	440120	-	-	-	-
metab_4652	5.3454	189.1633	pos	Neryl butyrate	C14H24O2	224.1776	5352162	Fatty Acyls	Fatty alcohol esters	HMDB0038259	Neryl butyrate
metab_14770	0.6411	191.0191	neg	Isocitric acid	C6H8O7	192.0270	1198	Carboxylic acids and derivatives	Tricarboxylic acids and derivatives	HMDB0001874; HMDB0000193	D-threo-Isocitric acid;Isocitric acid
metab_14770	0.6411	191.0191	neg	3-Deoxy-D-threo-hex-2-ulosaric acid	C6H8O7	192.0272	6323338	Carboxylic acids and derivatives	Tricarboxylic acids and derivatives	HMDB0001874; HMDB0000193	D-threo-Isocitric acid;Isocitric acid

metab_14770	0.6411	191.0191	neg	Citric acid	C6H8O7	192.0272	311	Carboxylic acids and derivatives	Tricarboxylic acids and derivatives	HMDB0001874; HMDB0000193	D-threo-Isocitric acid;Isocitric acid
metab_6779	0.7818	191.0191	neg	3-Deoxy-D-threo-hex-2-ulosaric acid	C6H8O7	192.0272	6323338	-	-	-	-
metab_6779	0.7818	191.0191	neg	Citric acid	C6H8O7	192.0272	311	-	-	-	-
metab_9074	3.2801	191.0554	neg	Quinic acid	C7H12O6	192.0632	6508	-	-	-	-
metab_9481	4.9183	191.0555	neg	Quinic acid	C7H12O6	192.0632	6508	-	-	-	-
metab_12504	6.3269	191.0555	neg	Quinic acid	C7H12O6	192.0632	6508	-	-	-	-
metab_1130	0.6262	191.1021	pos	(2R,6S)-2,6-Diaminoheptanedioic acid	C7H14N2O4	190.0954	99290	Carboxylic acids and derivatives	Amino acids, peptides, and analogues	HMDB0001370	Diaminopimelic acid
metab_5892	1.4569	192.0650	pos	5-Phenyl-1,3-oxazinane-2,4-dione	C10H9NO3	191.0582	21350393	Benzene and substituted derivatives	-	HMDB0060400	5-Phenyl-1,3-oxazinane-2,4-dione
metab_6092	1.1306	192.0650	pos	5-Phenyl-1,3-oxazinane-2,4-dione	C10H9NO3	191.0582	21350393	-	-	-	-
metab_44	1.3720	192.1013	pos	Ephedroxane	C11H13NO2	191.0946	161171	-	-	-	-
metab_1967	2.7188	192.1015	pos	Ephedroxane	C11H13NO2	191.0946	161171	-	-	-	-
metab_6181	0.9060	192.1015	pos	Ephedroxane	C11H13NO2	191.0946	161171	-	-	-	-
metab_5097	3.3337	192.1095	pos	Ephedroxane	C11H13NO2	191.0946	161171	-	-	-	-
metab_5607	2.0062	192.1379	pos	Diethyltoluamide	C12H17NO	191.1309	4284	-	-	-	-
metab_13483	2.8440	193.0501	neg	5-Hydroxyconiferaldehyde	C10H10O4	194.0579	5282094	-	-	-	-
metab_6030	1.2015	193.0605	pos	Quinate	C7H12O6	192.0634	6508	-	-	-	-
metab_1816	2.1444	193.0686	pos	Sinapyl alcohol	C11H14O4	192.0787	5280507	Phenols	Methoxyphenols	HMDB0013070	Sinapyl alcohol
metab_5244	2.8725	193.0856	pos	Sinapyl alcohol	C11H14O4	192.0787	5280507	-	-	-	-
metab_2607	6.6001	193.1219	pos	Sedanonic acid lactone	C12H16O2	192.1150	10932278	-	-	-	-
metab_2550	5.9949	193.1583	pos	4-Heptylphenol	C13H20O	192.1513	16143	-	-	-	-

metab_145	4.8961	193.1583	pos	4-Heptylphenol	C13H20O	192.1513	16143	-	-	-	-
metab_4476	6.3885	193.1583	pos	4-Heptylphenol	C13H20O	192.1513	16143	-	-	-	-
metab_14072	1.7419	194.0454	neg	N-Acetyl-5-aminosalicylic acid	C9H9NO4	195.0532	65512	Benzene and substituted derivatives	Benzoic acids and derivatives	HMDB0060602	N-acetyl-5-aminosalicylic acid
metab_14259	1.5297	195.0293	neg	2-(2-Hydroxy-4-methoxyphenyl)-2-oxoacetic acid	C9H8O5	196.0372	5044587	Phenols	Methoxyphenols	HMDB0137120	2-(2-hydroxy-4-methoxyphenyl)-2-oxoacetic acid
metab_7532	0.5991	195.0504	neg	Gluconic acid	C6H12O7	196.0583	10690	Organooxygen compounds	Carbohydrates and carbohydrate conjugates	HMDB0000283	D-Ribose
metab_11705	8.7191	195.0505	neg	Gluconic acid	C6H12O7	196.0583	10690	-	-	-	-
metab_7502	1.8046	195.0657	neg	2,3-Dihydroxy-p-cumate	C10H12O4	196.0736	54675852	-	-	-	-
metab_13252	3.4470	195.0658	neg	2,3-Dihydroxy-p-cumate	C10H12O4	196.0736	54675852	-	-	-	-
metab_14186	1.5736	195.0958	neg	Ethyl(E,Z)-decadienoate	C12H20O2	196.1464	5281162	Fatty Acyls	Fatty acid esters	HMDB0036599	Ethyl (2E,4E)-2,4-decadienoate
metab_14107	1.6948	195.1021	neg	Geranyl acetate	C12H20O2	196.1464	1549026	-	-	-	-
metab_6116	1.0741	195.1124	pos	3-Acetyl-7-methyl-1H,2H,4H,5H,6H,7H,7aH-pyrrolo[3,2-b]pyridin-2-one	C10H14N2O2	194.1055	136927122	Pyrrolopyridines	-	HMDB0041440	Laccarin
metab_4490	6.2824	195.1375	pos	Cnidilide	C12H18O2	194.1307	160710	-	-	-	-
metab_4738	4.9267	195.1375	pos	Neocnidilide	C12H18O2	194.1307	3083857	Lactones	Gamma butyrolactones	HMDB0034450	Neocnidilide
metab_4738	4.9267	195.1375	pos	Cnidilide	C12H18O2	194.1307	160710	Lactones	Gamma butyrolactones	HMDB0034450	Neocnidilide
metab_9867	7.0322	195.1385	neg	Ethyl (2E,4Z)-deca-2,4-dienoate	C12H20O2	196.1464	5281162	-	-	-	-
metab_9867	7.0322	195.1385	neg	Geranyl acetate	C12H20O2	196.1464	1549026	-	-	-	-
metab_12797	4.9510	195.1385	neg	Ethyl (2E,4Z)-deca-2,4-dienoate	C12H20O2	196.1464	5281162	-	-	-	-
metab_12797	4.9510	195.1385	neg	Geranyl acetate	C12H20O2	196.1464	1549026	-	-	-	-

metab_9581	5.5639	195.1385	neg	Ethyl (2E,4Z)-deca-2,4-dienoate	C12H20O2	196.1464	5281162	-	-	-	-
metab_9581	5.5639	195.1385	neg	Geranyl acetate	C12H20O2	196.1464	1549026	-	-	-	-
metab_5191	3.0114	196.1078	pos	alpha-[3-(Nitrosoamino)propyl]-3-pyridinemethanol	C9H13N3O2	195.1009	53297442	-	-	-	-
metab_5355	2.5158	196.1409	pos	alpha-[3-(Nitrosoamino)propyl]-3-pyridinemethanol	C9H13N3O2	195.1009	53297442	Pyridines and derivatives		HMDB0062443	alpha-3-(Nitrosoamino)propyl]-3-pyridinemethanol
metab_5757	1.6838	197.0806	pos	2,3-Dihydroxy-p-cumate	C10H12O4	196.0736	54675852	-	-	-	-
metab_6447	0.5280	198.1232	pos	Nitrilacarb	C9H15N3O2	197.1164	9570637	-	-	-	-
metab_14661	0.8946	199.0719	neg	Serinyl-Hydroxyproline	C8H14N2O5	218.0903	69043006	Carboxylic acids and derivatives	Amino acids, peptides, and analogues	HMDB0029040	Serinyl-Hydroxyproline
metab_6076	1.1589	199.1073	pos	H-Thr-pro-OH	C9H16N2O4	216.1110	14647614	Carboxylic acids and derivatives	Amino acids, peptides, and analogues	HMDB0029069	Threoninyl-Proline
metab_12747	5.2100	199.1334	neg	11-Hydroxy-2-undecenoic acid	C11H20O3	200.1413	86289764	-	-	-	-
metab_12876	4.6550	199.1335	neg	11-Hydroxy-2-undecenoic acid	C11H20O3	200.1413	86289764	-	-	-	-
metab_13323	3.2301	199.1335	neg	11-Hydroxy-2-undecenoic acid	C11H20O3	200.1413	86289764	-	-	-	-
metab_13394	3.0432	199.1335	neg	11-Hydroxy-2-undecenoic acid	C11H20O3	200.1413	86289764	-	-	-	-
metab_13151	3.7317	199.1336	neg	11-Hydroxy-2-undecenoic acid	C11H20O3	200.1413	86289764	-	-	-	-
metab_14074	1.7419	200.1005	neg	L-2-amino-8-oxodecanoate	C10H19NO3	201.1365	91820288	-	-	-	-
metab_5732	1.7412	200.1276	pos	Ecgonine methyl ester	C10H17NO3	199.1208	104904	-	-	-	-
metab_5732	1.7412	200.1276	pos	N-[(3s)-2-Oxotetrahydrofuran-	C10H17NO3	199.1208	10058590	-	-	-	-

				3-Yl]hexanamide							
metab_5651	1.9463	200.1277	pos	Ecgonine methyl ester	C10H17NO3	199.1208	104904	-	-	-	-
metab_5651	1.9463	200.1277	pos	N-[(3s)-2-Oxotetrahydrofuran-3-Yl]hexanamide	C10H17NO3	199.1208	10058590	-	-	-	-
metab_9075	3.2972	200.1287	neg	L-2-Amino-8-oxodecanoate	C10H19NO3	201.1365	91820288	-	-	-	-
metab_5808	1.5987	200.1640	pos	11-Nitro-1-Undecene	C11H21NO2	199.1571	543829	-	-	-	-
metab_4999	3.7297	200.1641	pos	11-Nitro-1-Undecene	C11H21NO2	199.1571	543829	-	-	-	-
metab_5271	2.7959	200.1641	pos	11-Nitro-1-Undecene	C11H21NO2	199.1571	543829	-	-	-	-
metab_1772	2.0211	200.1642	pos	11-Nitro-1-Undecene	C11H21NO2	199.1571	543829	-	-	-	-
metab_4391	6.8728	200.2004	pos	Dodecanamide	C12H25NO	199.1935	14256	-	-	-	-
metab_12487	6.4073	201.1127	neg	Sebacic acid	C10H18O4	202.1207	5192	-	-	-	-
metab_14029	1.8205	201.1127	neg	Sebacic acid	C10H18O4	202.1207	5192	-	-	-	-
metab_6935	3.8506	201.1128	neg	Sebacic acid	C10H18O4	202.1207	5192	-	-	-	-
metab_14298	1.4856	201.1240	neg	Ala-ile	C9H18N2O3	202.1319	7408079	-	-	-	-
metab_1606	1.5144	201.1594	pos	UNII-UOM92D5748	C11H20O3	200.1413	86289764	-	-	-	-
metab_8037	0.9649	202.1080	neg	N-Lactoyl-Leucine	C9H17NO4	203.1158	57329455	Carboxylic acids and derivatives	Amino acids, peptides, and analogues	HMDB0062176	N-lactoyl-Leucine
metab_5111	3.3032	202.1434	pos	L-2-Amino-8-oxodecanoate	C10H19NO3	201.1365	91820288	-	-	-	-
metab_5349	2.5314	202.1797	pos	11-Aminoundecanoic acid	C11H23NO2	201.1728	17083	-	-	-	-
metab_8326	1.5297	203.0822	neg	Tryptophan	C11H12N2O2	204.0899	6305	Indoles and derivatives	Indolyl carboxylic acids and derivatives	HMDB0000929	L-Tryptophan
metab_14094	1.7264	203.0920	neg	Diethyl (2S,3R)-2-methyl-3-hydroxysuccinate	C9H16O5	204.0999	440396	-	-	-	-
metab_8657	2.1630	203.0920	neg	Diethyl	C9H16O5	204.0999	440396	-	-	-	-

				(2S,3R)-2-methyl-3-hydroxysuccinate							
metab_1319	0.6262	203.1021	pos	Proclavaminic acid	C8H14N2O4	202.0955	194953	-	-	-	-
metab_6338	0.6122	203.1134	pos	IPA imine	C11H10N2O2	202.0743	5599	-	-	-	-
metab_4864	4.3371	203.1427	pos	alpha-Amylcinnamaldehyde	C14H18O	202.1359	31209	-	-	-	-
metab_6374	0.5983	203.1497	pos	N,N-Dimethylarginine	C8H18N4O2	202.1430	123831	Carboxylic acids and derivatives	Amino acids, peptides, and analogues	HMDB0001539	Asymmetric dimethylarginine
metab_1234	0.5140	203.2226	pos	Thermospermine	C10H26N4	202.2158	194365	-	-	-	-
metab_7620	2.6046	204.0662	neg	Indole-3-lactic acid	C11H11NO3	205.0739	92904	Indoles and derivatives	Indolyl carboxylic acids and derivatives	HMDB0000671	Indolelactic acid
metab_3396	14.8330	204.0861	pos	N-Acetyl-L-2-aminoadipic acid	C8H13NO5	203.0794	443992	-	-	-	-
metab_2637	6.8433	204.0862	pos	N-Acetyl-L-2-aminoadipic acid	C8H13NO5	203.0794	443992	-	-	-	-
metab_6481	0.5140	204.0862	pos	N-Acetyl-L-2-aminoadipic acid	C8H13NO5	203.0794	443992	-	-	-	-
metab_2330	4.4580	204.0862	pos	N-Acetyl-L-2-aminoadipic acid	C8H13NO5	203.0794	443992	-	-	-	-
metab_1964	2.7029	204.1015	pos	Shihunine	C12H13NO2	203.0946	168974	-	-	-	-
metab_6620	1.3231	204.1237	neg	Pantothenol	C9H19NO4	205.1315	131204	-	-	-	-
metab_13983	1.8833	205.0139	neg	4-Fluoromuconolactone	C6H5FO4	160.0172	9543392	Dihydrofurans	Furanones	HMDB0060386	4-Fluoromuconolactone
metab_8015	0.8946	205.0349	neg	(1R,2S)-1-Hydroxybutane-1,2,4-tricarboxylic acid	C7H10O7	206.0427	5460287	Keto acids and derivatives	Gamma-keto acids and derivatives	HMDB0039447	2-Methyl-4-oxopentanedioic acid
metab_13183	3.6311	205.0350	neg	(1R,2S)-1-Hydroxybutane-1,2,4-tricarboxylic acid	C7H10O7	206.0427	5460287	-	-	-	-
metab_13755	2.2912	205.0350	neg	(1R,2S)-1-Hydroxybutane-1,2,	C7H10O7	206.0427	5460287	-	-	-	-

				4-tricarboxylic acid							
metab_1374	0.8221	205.0813	pos	Glu-Gly	C7H12N2O5	204.0745	6427052	-	-	-	-
metab_1054	1.5284	205.0966	pos	Tryptophan	C11H12N2O2	204.0899	6305	-	-	-	-
metab_8808	2.5264	206.0818	neg	N-Acetyl-D-phenylalanine	C11H13NO3	207.0895	101184	-	-	-	-
metab_45	1.3153	206.1382	pos	Dexpanthenol	C9H19NO4	205.1315	131204	Fatty Acyls	Fatty amides	HMDB0004231	Pantothenol
metab_387	1.8577	207.0648	pos	Scoparone	C11H10O4	206.0580	8417	-	-	-	-
metab_7710	3.3803	207.0661	neg	Sinapaldehyde	C11H12O4	208.0736	5280802	-	-	-	-
metab_140	5.3454	207.1738	pos	L-Menthyl (R,S)-3-hydroxybutyrate	C14H26O3	242.1882	10220444	Prenol lipids	Monoterpenoids	HMDB0032370	L-Menthyl (R,S)-3-hydroxybutyrate
metab_14105	1.7104	208.0612	neg	4-Oxo-4-(pyridin-3-yl)butanal	C9H9NO2	163.0633	115084	Organooxygen compounds	Carbonyl compounds	HMDB0062406	4-Oxo-1-(3-pyridyl)-1-butanone
metab_6124	1.0601	208.0962	pos	Rhexifoline	C11H13NO3	207.0895	185301	-	-	-	-
metab_1926	2.5314	208.0964	pos	N-Acetyl-L-phenylalanine	C11H13NO3	207.0895	74839	Carboxylic acids and derivatives	Amino acids, peptides, and analogues	HMDB0000512	N-Acetyl-L-phenylalanine
metab_6776	0.6131	209.0662	neg	L-Glycero-D-Manno-Heptose	C7H14O7	210.0740	21120522	-	-	-	-
metab_8892	2.7624	209.0816	neg	Ethyl 2,4-dihydroxy-3,6-dimethylbenzoate	C11H14O4	210.0892	3084545	-	-	-	-
metab_2126	3.3337	209.1167	pos	Coronafacic acid	C12H16O3	208.1098	114969	-	-	-	-
metab_490	2.8725	209.1168	pos	Coronafacic acid	C12H16O3	208.1098	114969	-	-	-	-
metab_9510	5.1131	209.1179	neg	(+)-7-Iso-Jasmonic acid	C12H18O3	210.1257	7251183	-	-	-	-
metab_9510	5.1131	209.1179	neg	Jasmonic acid	C12H18O3	210.1254	5281166	-	-	-	-
metab_5968	1.3293	209.1278	pos	4-Dimethylamino-L-phenylalanine	C11H16N2O2	208.1212	3081973	-	-	-	-
metab_14870	0.5711	211.0819	neg	Perseitol	C7H16O7	212.0896	441436	Organooxygen	Carbohydrates and	HMDB0033750	D-Glycero-D-galacto-heptitol

								compounds	carbohydrate conjugates		
metab_8627	2.0987	211.0973	neg	3-Hydroxy-p-mentha-1,8-dien-7-al	C10H14O2	166.0994	85247137	Prenol lipids	Monoterpenoids	HMDB0041584	3-Hydroxy-p-mentha-1,8-dien-7-al
metab_2517	5.7540	211.1323	pos	Jasmonic acid	C12H18O3	210.1255	5281166	-	-	-	-
metab_4703	5.0451	211.1323	pos	Jasmonic acid	C12H18O3	210.1255	5281166	-	-	-	-
metab_9483	4.9349	211.1335	neg	7-Oxo-11-Dodecenoic acid	C12H20O3	212.1411	4374065	-	-	-	-
metab_13364	3.1286	211.1336	neg	7-Oxo-11-Dodecenoic acid	C12H20O3	212.1411	4374065	-	-	-	-
metab_13561	2.6841	211.1336	neg	7-Oxo-11-Dodecenoic acid	C12H20O3	212.1411	4374065	-	-	-	-
metab_2428	5.1076	211.1436	pos	(+)-7-Isojasmonic acid	C12H18O3	210.1254	7251183	-	-	-	-
metab_2467	5.3913	211.1687	pos	(3E,5E)-Tridecadienoic acid	C13H22O2	210.1619	5312396	-	-	-	-
metab_559	2.1897	211.1688	pos	(3E,5E)-Tridecadienoic acid	C13H22O2	210.1619	5312396	-	-	-	-
metab_1129	0.6262	212.0911	pos	Enicoflavine	C10H13NO4	211.0845	5281564	-	-	-	-
metab_1129	0.6262	212.0911	pos	Methyldopa	C10H13NO4	211.0845	38853	-	-	-	-
metab_231	0.8920	212.0911	pos	Enicoflavine	C10H13NO4	211.0845	5281564	-	-	-	-
metab_231	0.8920	212.0911	pos	Methyldopa	C10H13NO4	211.0845	38853	-	-	-	-
metab_1100	1.1731	212.0912	pos	Enicoflavine	C10H13NO4	211.0845	5281564	-	-	-	-
metab_1100	1.1731	212.0912	pos	Methyldopa	C10H13NO4	211.0845	38853	-	-	-	-
metab_390	1.9169	212.0912	pos	Enicoflavine	C10H13NO4	211.0845	5281564	-	-	-	-
metab_390	1.9169	212.0912	pos	Methyldopa	C10H13NO4	211.0845	38853	-	-	-	-
metab_8737	2.3533	212.0925	neg	Kainic acid	C10H15NO4	213.1001	10255	-	-	-	-
metab_14007	1.8524	212.0925	neg	Isovalerylglutamic acid	C10H17NO5	231.1107	133383	Carboxylic acids and derivatives	Amino acids, peptides, and analogues	HMDB0000726	Isovalerylglutamic acid
metab_284	1.1731	212.1024	pos	Zalcitabine	C9H13N3O3	211.0957	24066	-	-	-	-
metab_1580	1.4429	212.1066	pos	Zalcitabine	C9H13N3O3	211.0957	24066	Pyrimidine nucleosides	Pyrimidine 2',3'-dideoxyribonucleosi	HMDB0015078	HIVID roche

									des		
metab_1419	0.9480	212.1275	pos	Methoxamine	C11H17NO3	211.1208	6082	-	-	-	-
metab_53	1.7705	212.1276	pos	Methoxamine	C11H17NO3	211.1208	6082	-	-	-	-
metab_580	4.0939	212.1640	pos	Elaeokanine C	C12H21NO2	211.1571	442855	-	-	-	-
metab_2384	4.7907	212.1640	pos	Methoxamine	C11H17NO3	211.1208	6082	Benzene and substituted derivatives	Methoxybenzenes	HMDB0014861	Methoxamine hydrochloride
metab_2384	4.7907	212.1640	pos	Elaeokanine C	C12H21NO2	211.1571	442855	-	-	-	-
metab_5418	2.3908	212.1640	pos	Elaeokanine C	C12H21NO2	211.1571	442855	-	-	-	-
metab_2914	8.6132	212.2003	pos	3-Aminotridec-2-en-4-one	C13H25NO	211.1934	91819972	-	-	-	-
metab_4084	8.2728	212.2004	pos	3-Aminotridec-2-en-4-one	C13H25NO	211.1934	91819972	-	-	-	-
metab_308	1.3437	213.0752	pos	4-Hydroxymethyl-3-methoxyphenoxyacetic acid	C10H12O5	212.0685	134310	-	-	-	-
metab_13006	4.2040	213.1129	neg	2-[1-(Carboxymethyl)-3-methylcyclohexyl]acetic acid	C11H18O4	214.1206	220370	-	-	-	-
metab_14102	1.7104	213.1130	neg	2-[1-(Carboxymethyl)-3-methylcyclohexyl]acetic acid	C11H18O4	214.1206	220370	-	-	-	-
metab_4739	4.9267	213.1480	pos	12-Oxo-9Z-dodecenoic acid	C12H20O3	212.1410	9548789	-	-	LMFA01060182	
metab_4739	4.9267	213.1480	pos	(+)-Cucurbic acid	C12H20O3	212.1411	5281159	-	-	LMFA01060182	
metab_4739	4.9267	213.1480	pos	7-Oxo-11-Dodecenoic acid	C12H20O3	212.1411	4374065	-	-	LMFA01060182	
metab_1915	2.4850	213.1480	pos	12-Oxo-9Z-dodecenoic acid	C12H20O3	212.1410	9548789	-	-	-	-
metab_1915	2.4850	213.1480	pos	(+)-Cucurbic acid	C12H20O3	212.1411	5281159	-	-	-	-
metab_1915	2.4850	213.1480	pos	7-Oxo-11-Dodecenoic acid	C12H20O3	212.1411	4374065	-	-	-	-
metab_5635	1.9609	213.1481	pos	12-Oxo-9Z-dodecenoic acid	C12H20O3	212.1410	9548789	-	-	-	-
metab_5635	1.9609	213.1481	pos	7-Oxo-11-Dodecenoic acid	C12H20O3	212.1411	4374065	-	-	-	-
metab_5635	1.9609	213.1481	pos	Cucurbic acid	C12H20O3	212.1411	5281159	-	-	-	-

metab_9528	5.2100	213.1492	neg	3-Oxododecanoic acid	C12H22O3	214.1570	439717	-	-	-	-
metab_12593	5.9375	213.1493	neg	3-Oxododecanoic acid	C12H22O3	214.1570	439717	-	-	-	-
metab_12846	4.7709	213.1493	neg	3-Oxododecanoic acid	C12H22O3	214.1570	439717	-	-	-	-
metab_13854	2.1153	214.1082	neg	2-Amino-9,10-epoxy-8-oxode canoic acid	C10H17NO4	215.1159	443587	-	-	-	-
metab_8396	1.6186	214.1082	neg	2-Amino-9,10-epoxy-8-oxode canoic acid	C10H17NO4	215.1159	443587	-	-	-	-
metab_469	2.5627	214.1433	pos	N-Heptanoylhomoserine lactone	C11H19NO3	213.1364	443437	-	-	-	-
metab_12584	5.9542	214.1445	neg	Ethyl butylacetylaminopropionate	C11H21NO3	215.1520	104150	-	-	-	-
metab_13033	4.1023	214.1446	neg	Ethyl butylacetylaminopropionate	C11H21NO3	215.1520	104150	-	-	-	-
metab_2576	6.2226	214.2523	pos	Tetradecylamine	C14H31N	213.2454	16217	-	-	-	-
metab_6957	4.4386	215.1286	neg	Undecanedioic acid	C11H20O4	216.1361	15816	-	-	-	-
metab_13891	2.0358	215.1287	neg	Undecanedioic acid	C11H20O4	216.1361	15816	-	-	-	-
metab_1333	0.6542	215.1384	pos	2,2'-(3-methylcyclohexane-1,1 -diyl)diacetic acid	C11H18O4	214.1206	220370	-	-	-	-
metab_8136	1.2377	215.1398	neg	Val-val	C10H20N2O3	216.1474	107475	-	-	-	-
metab_8556	1.9439	215.1398	neg	Val-val	C10H20N2O3	216.1474	107475	-	-	-	-
metab_8258	1.4427	215.1399	neg	Val-val	C10H20N2O3	216.1474	107475	-	-	-	-
metab_8457	1.7419	215.1399	neg	Val-val	C10H20N2O3	216.1474	107475	-	-	-	-
metab_4730	4.9267	215.1637	pos	3-Oxododecanoic acid	C12H22O3	214.1568	439717	-	-	-	-
metab_4858	4.3512	215.1637	pos	3-Oxododecanoic acid	C12H22O3	214.1568	439717	-	-	-	-
metab_9822	6.8565	215.1649	neg	12-Hydroxydodecanoic acid	C12 H24 O3	216.1724	79034	-	-	-	-

metab_5805	1.6128	216.1225	pos	2-Amino-8-oxo-9,10-epoxy-decanoic acid	C10H17NO4	215.1158	16061039	-	-	LMFA01060176	
metab_13612	2.5893	216.1238	neg	Propionylcarnitine	C10H19NO4	217.1314	107738	-	-	-	-
metab_13785	2.2437	216.1238	neg	Propionylcarnitine	C10H19NO4	217.1314	107738	-	-	-	-
metab_5513	2.1746	216.1589	pos	Ethyl butylacetylaminopropionate	C11H21NO3	215.1520	104150	-	-	-	-
metab_4911	4.0939	216.1589	pos	Ethyl butylacetylaminopropionate	C11H21NO3	215.1520	104150	-	-	-	-
metab_7942	0.6411	217.0826	neg	gamma-Glutamylalanine	C8H14N2O5	218.0903	440103	Carboxylic acids and derivatives	Amino acids, peptides, and analogues	HMDB0006248	gamma-Glutamylalanine
metab_9549	5.3388	217.1078	neg	3-Hydroxysebacic acid	C10H18O5	218.1155	3017884	-	-	-	-
metab_13511	2.7961	217.1078	neg	3-Hydroxysebacic acid	C10H18O5	218.1155	3017884	-	-	-	-
metab_14278	1.5155	217.1079	neg	3-Hydroxysebacic acid	C10H18O5	218.1155	3017884	-	-	-	-
metab_14361	1.4427	217.1190	neg	Lysopine	C9H18N2O4	218.1266	193187	-	-	-	-
metab_5082	3.3943	217.1329	pos	undecanedioic acid	C11H20O4	216.1361	15816	Fatty Acyls		HMDB0000888	1,11-Undecanedioic acid
metab_40	1.2162	217.1541	pos	Val-val	C10H20N2O3	216.1474	107475	-	-	-	-
metab_1738	1.9463	217.1541	pos	Val-val	C10H20N2O3	216.1474	107475	-	-	-	-
metab_5902	1.4429	217.1542	pos	Val-val	C10H20N2O3	216.1474	107475	-	-	-	-
metab_14681	0.8662	218.0666	neg	N-(1-Deoxy-1-fructosyl)glycine	C8H15NO7	237.0849	131752250	Organooxygen compounds	Carbohydrates and carbohydrate conjugates	HMDB0037848	N-(1-Deoxy-1-fructosyl)glycine
metab_6619	1.3089	218.1031	neg	Pantothenic acid	C9H17NO5	219.1107	6613	Alcohols and polyols	Polyols	HMDB0000210	Pantothenic acid
metab_280	1.0461	218.1381	pos	Propionylcarnitine	C10H19NO4	217.1314	107738	-	-	-	-
metab_2651	6.8886	218.1381	pos	Propionylcarnitine	C10H19NO4	217.1314	107738	-	-	-	-
metab_3373	15.6702	218.1381	pos	Propionylcarnitine	C10H19NO4	217.1314	107738	-	-	-	-
metab_2537	5.9208	218.1381	pos	Propionylcarnitine	C10H19NO4	217.1314	107738	-	-	-	-

metab_467	2.5938	218.1382	pos	Propionylcarnitine	C10H19NO4	217.1314	107738	-	-	-	-
metab_8763	2.4010	219.0860	neg	Ascaroside C3	C9H16O6	220.0948	44202063	-	-	-	-
metab_8155	1.2663	219.0871	neg	Ascaroside C3	C9H16O6	220.0948	44202063	-	-	-	-
metab_13899	2.0358	220.0612	neg	5-Hydroxyindole-3-acetaldehyde	C10H9NO2	175.0633	74688	Indoles and derivatives	Hydroxyindoles	HMDB0004073	5-Hydroxyindoleacetaldehyde
metab_5701	1.7845	220.1074	pos	D-Pantothenic acid	C9H17NO5	219.1107	6613	-	-	-	-
metab_0	1.3153	220.1173	pos	Pantothenic acid	C9H17NO5	219.1107	6613	-	-	-	-
metab_7619	0.5431	221.0599	neg	Cystathionine	C7H14N2O4S	222.0674	834	Carboxylic acids and derivatives	Amino acids, peptides, and analogues	HMDB0000099	L-Cystathionine
metab_12867	4.7046	221.0816	neg	Dillapiol	C12H14O4	222.0892	10231	-	-	-	-
metab_14123	1.6642	221.0816	neg	Dillapiol	C12H14O4	222.0892	10231	-	-	-	-
metab_14274	1.5155	221.0817	neg	Dillapiol	C12H14O4	222.0892	10231	-	-	-	-
metab_1104	1.0321	221.0912	pos	5-Hydroxytryptophan	C11H12N2O3	220.0848	144	-	-	-	-
metab_1393	0.8780	221.0913	pos	5-Hydroxytryptophan	C11H12N2O3	220.0848	144	-	-	-	-
metab_1080	1.3153	221.0914	pos	5-Hydroxytryptophan	C11H12N2O3	220.0848	144	-	-	-	-
metab_8378	1.5885	221.0930	neg	Glycyl-L-phenylalanine	C11H14N2O3	222.1005	92953	-	-	-	-
metab_8378	1.5885	221.0930	neg	Phe-gly	C11H14N2O3	222.1005	6992304	-	-	-	-
metab_13	4.8809	221.1530	pos	(6R,8Z)-6-Hydroxy-3-oxotetradecenoic acid	C14H24O4	256.1675	134819281	-	-	HMDB0062363	(6R,8Z)-6-Hydroxy-3-oxotetradecenoic acid
metab_8166	1.2805	222.0769	neg	N-Acetyl-L-tyrosine	C11H13NO4	223.0845	68310	Carboxylic acids and derivatives	Amino acids, peptides, and analogues	HMDB0000866	N-Acetyl-L-tyrosine
metab_14261	1.5297	222.0769	neg	N-Acetyl-L-tyrosine	C11H13NO4	223.0845	68310	Carboxylic acids and derivatives	Amino acids, peptides, and analogues	HMDB0000866	N-Acetyl-L-tyrosine
metab_8852	2.6524	223.0609	neg	2-Benzylmalic acid	C11H12O5	224.0685	152197	-	-	-	-
metab_8852	2.6524	223.0609	neg	Sinapic acid	C11H12O5	224.0686	637775	-	-	-	-

metab_9145	3.5641	223.0610	neg	2-Benzylmalic acid	C11H12O5	224.0685	152197	-	-	-	-
metab_9145	3.5641	223.0610	neg	Sinapic acid	C11H12O5	224.0686	637775	-	-	-	-
metab_13734	2.3380	223.0645	neg	Trinexapac	C11H12O5	224.0721	14371531	-	-	-	-
metab_8101	1.1355	223.0646	neg	Trinexapac	C11H12O5	224.0721	14371531	-	-	-	-
metab_5816	1.5847	223.1071	pos	Glycyl-L-phenylalanine	C11H14N2O3	222.1005	92953	-	-	-	-
metab_5816	1.5847	223.1071	pos	Phe-gly	C11H14N2O3	222.1005	6992304	-	-	-	-
metab_1526	1.2585	223.1071	pos	Glycyl-L-phenylalanine	C11H14N2O3	222.1005	92953	-	-	-	-
metab_1526	1.2585	223.1071	pos	Phe-gly	C11H14N2O3	222.1005	6992304	-	-	-	-
metab_2726	7.2763	223.1686	pos	Rishitin	C14H22O2	222.1618	108064	-	-	-	-
metab_2633	6.8280	223.1686	pos	Rishitin	C14H22O2	222.1618	108064	-	-	-	-
metab_10	5.2717	223.1686	pos	Rishitin	C14H22O2	222.1618	108064	-	-	-	-
metab_14701	0.8098	224.0925	neg	Aciclovir	C8H11N5O3	225.0861	135398513	-	-	-	-
metab_764	8.2289	224.2002	pos	Pellitorine	C14H25NO	223.1935	5318516	-	-	-	-
metab_2655	6.9035	224.2003	pos	Pellitorine	C14H25NO	223.1935	5318516	-	-	-	-
metab_6605	0.5851	225.0612	neg	1,3,7-Trimethyl-5-hydroxyisourate	C8H10N4O4	226.0703	118796883	Carboxylic acids and derivatives	Amino acids, peptides, and analogues	HMDB0039222	N-(gamma-Glutamyl)ethanolamine
metab_8522	1.8682	225.0766	neg	Genipin	C11H14O5	226.0843	442424	-	-	-	-
metab_2362	4.6398	225.1116	pos	2-Benzylmalic acid	C11H12O5	224.0685	152197	-	-	-	-
metab_2362	4.6398	225.1116	pos	Sinapic acid	C11H12O5	224.0686	637775	Cinnamic acids and derivatives	Hydroxycinnamic acids and derivatives	HMDB0032616	(e)-3-(4-Hydroxy-3,5-dimethoxyphenyl)-2-propenoate
metab_5310	2.6561	225.1116	pos	Diplodiol	C12H16O4	224.1049	129639	Pyrans	Pyranones and derivatives	HMDB0030680	Diplosporin
metab_2404	4.9413	225.1479	pos	Methyl (+)-7-isojasmonate	C13H20O3	224.1412	6427970	-	-	-	-
metab_11833	8.5135	225.2222	neg	Pentadecanal	C15H30O	226.2297	17697	-	-	-	-
metab_11737	8.7031	225.2222	neg	Pentadecanal	C15H30O	226.2297	17697	-	-	-	-

metab_9222	3.8001	227.0826	neg	(1R,10As)-1,4,10,10a-tetrahydrophenazine-1-carboxylate	C13H12N2O2	228.0900	119058154	-	-	-	-
metab_7473	1.5155	227.1035	neg	(S)-Atpa	C10H16N2O4	228.1111	447407	-	-	-	-
metab_5345	2.5469	227.1174	pos	d-Phenylalanyl-l-proline	C14H18N2O3	262.1317	162656	Carboxylic acids and derivatives	Amino acids, peptides, and analogues	HMDB0011177	Phenylalanylproline
metab_12881	4.6550	227.1286	neg	Traumatic acid	C12H20O4	228.1363	5283028	Fatty Acyls	Fatty acids and conjugates	HMDB0000933; LMFA01170002	Traumatic acid
metab_7412	6.1320	227.1287	neg	Traumatic acid	C12H20O4	228.1363	5283028	-	-	-	-
metab_6932	3.8506	227.1287	neg	Traumatic acid	C12H20O4	228.1363	5283028	-	-	-	-
metab_8587	1.9904	227.1399	neg	Isoleucyl-Proline	C11H20N2O3	228.1474	444876	-	-	-	-
metab_13249	3.4470	227.1651	neg	Myristic acid	C14H28O2	228.2091	11005	Fatty Acyls	Amino acids, peptides, and analogues	HMDB0000806	Myristic acid
metab_13944	1.9745	228.0875	neg	3,4,5,6-Tetrahydrohippurate	C9H13NO3	183.0895	149048	Carboxylic acids and derivatives	Amino acids, peptides, and analogues	HMDB0061679	3,4,5,6-Tetrahydrohippuric acid
metab_6603	2.2595	228.0875	neg	O-Malonylcarnitine	C10H17NO6	247.1056	71464475	Fatty Acyls	Fatty acid esters	LMFA07070080; HMDB0002095	Malonylcarnitine
metab_544	3.5771	228.1013	pos	2,2-Diphenylglycine	C14H13NO2	227.0946	18289	-	-	-	-
metab_9426	4.6550	228.1239	neg	(R)-Crotonylcarnitine	C11H19NO4	229.1315	90659893	-	-	-	-
metab_13101	3.8506	228.1321	neg	crotonyl-L-carnitine	C11H19NO4	229.1315	90659893	-	-	-	-
metab_5717	1.7705	228.1335	pos	Asn-Ile	C10H19N3O4	245.1376	17805101	Carboxylic acids and derivatives	Amino acids, peptides, and analogues	HMDB0028734	Asparaginyl-Isoleucine
metab_5717	1.7705	228.1335	pos	3-Acetamino-6-isobutyl-2,5-dioxopiperazine	C10H17N3O3	227.1270	11413522	Carboxylic acids and derivatives	Amino acids, peptides, and analogues	HMDB0028734	Asparaginyl-Isoleucine
metab_1061	1.4860	228.1336	pos	3-Acetamino-6-isobutyl-2,5-dioxopiperazine	C10H17N3O3	227.1270	11413522	-	-	-	-
metab_4986	3.7599	228.1588	pos	N-(2-Oxotetrahydrofuran-3-Y	C12H21NO3	227.1520	6914579	-	-	-	-

				L)octanamide							
metab_9477	4.9021	228.1603	neg	N-Decanoylglycine	C12H23NO3	229.1676	1712391	-	-	-	-
metab_9301	4.1023	228.1603	neg	N-Decanoylglycine	C12H23NO3	229.1676	1712391	-	-	-	-
metab_5806	1.5987	229.0713	pos	Depdecin	C11H16O5	228.0997	5283267	-	-	-	-
metab_14695	0.8240	229.0827	neg	(2S,3'S)-alpha-Amino-2-carboxy-5-oxo-1-pyrrolidinebutanoic acid	C9H14N2O5	230.0903	14101166	Carboxylic acids and derivatives	Amino acids, peptides, and analogues	HMDB0039110	(2S,3'S)-alpha-Amino-2-carboxy-5-oxo-1-pyrrolidinebutanoic acid
metab_13541	2.7318	229.1079	neg	(1'R)-Nepetalic acid	C10H16O3	184.1099	12313272	Prenol lipids	Monoterpenoids	HMDB0036117	(1'R)-Nepetalic acid
metab_1057	1.5284	229.1176	pos	(S)-Atpa	C10H16N2O4	228.1111	447407	Peptidomimetics	Hybrid peptides	HMDB0011166	L-beta-aspartyl-L-leucine
metab_1057	1.5284	229.1176	pos	L-beta-Aspartyl-L-leucine	C10H18N2O5	246.1216	3549397	Peptidomimetics	Hybrid peptides	HMDB0011166	L-beta-aspartyl-L-leucine
metab_5592	2.0360	229.1177	pos	(S)-Atpa	C10H16N2O4	228.1111	447407	-	-	-	-
metab_5679	1.8577	229.1328	pos	Traumatic Acid	C12H20O4	228.1362	5283028	Fatty Acyls	Fatty acids and conjugates	HMDB0000933	2-Dodecendioate
metab_9688	6.1320	229.1443	neg	Dodecanedioic acid	C12H22O4	230.1517	12736	-	-	-	-
metab_12798	4.9510	229.1443	neg	Dodecanedioic acid	C12H22O4	230.1517	12736	Fatty Acyls	Fatty acids and conjugates	HMDB0033724; LMFA01030036	Undecylenic acid
metab_13316	3.2637	229.1443	neg	Dodecanedioic acid	C12H22O4	230.1517	12736	-	-	-	-
metab_9341	4.2870	229.1443	neg	Dodecanedioic acid	C12H22O4	230.1517	12736	-	-	-	-
metab_9227	3.8333	229.1443	neg	Dodecanedioic acid	C12H22O4	230.1517	12736	-	-	-	-
metab_9163	3.6142	229.1443	neg	Dodecanedioic acid	C12H22O4	230.1517	12736	-	-	-	-
metab_1423	0.9761	229.1540	pos	Prolylisoleucine	C11H20N2O3	228.1473	7079601	-	-	-	-
metab_341	1.5705	229.1540	pos	Prolylisoleucine	C11H20N2O3	228.1473	7079601	-	-	-	-
metab_14204	1.5736	229.1556	neg	Ile-val	C11H22N2O3	230.1630	449407	-	-	-	-
metab_14204	1.5736	229.1556	neg	Val-Leu	C11H22N2O3	230.1630	6993118	-	-	-	-
metab_14095	1.7264	229.1556	neg	Ile-val	C11H22N2O3	230.1630	449407	-	-	-	-

metab_14095	1.7264	229.1556	neg	Val-Leu	C11H22N2O3	230.1630	6993118	-	-	-	-
metab_2955	8.8169	230.0952	pos	Ergothioneine	C9H15N3O2S	229.0885	5351619	Carboxylic acids and derivatives	Amino acids, peptides, and analogues	HMDB0003045	Ergothioneine
metab_14008	1.8524	230.1032	neg	2-Hepteneoylglycine	C9H15NO3	185.1052	131802908	Carboxylic acids and derivatives	Amino acids, peptides, and analogues	HMDB0094728	2-Hepteneoylglycine
metab_13794	2.2272	230.1032	neg	Suberylglycine	C10H17NO5	231.1107	6453952	Carboxylic acids and derivatives	Amino acids, peptides, and analogues	HMDB0000953	Suberylglycine
metab_13622	2.5592	230.1395	neg	N-(Tert-Butoxycarbonyl)-L-leucine	C11H21NO4	231.1470	83170	-	-	-	-
metab_13622	2.5592	230.1395	neg	O-Butanoylcarnitine	C11H21NO4	231.1470	439829	-	-	-	-
metab_13772	2.2595	230.1395	neg	N-(Tert-Butoxycarbonyl)-L-leucine	C11H21NO4	231.1470	83170	-	-	-	-
metab_13772	2.2595	230.1395	neg	O-Butanoylcarnitine	C11H21NO4	231.1470	439829	-	-	-	-
metab_9247	3.9005	230.1396	neg	N-(Tert-Butoxycarbonyl)-L-leucine	C11H21NO4	231.1470	83170	-	-	-	-
metab_9247	3.9005	230.1396	neg	O-Butanoylcarnitine	C11H21NO4	231.1470	439829	-	-	-	-
metab_13508	2.7961	230.1397	neg	N-(Tert-Butoxycarbonyl)-L-leucine	C11H21NO4	231.1470	83170	-	-	-	-
metab_13508	2.7961	230.1397	neg	O-Butanoylcarnitine	C11H21NO4	231.1470	439829	-	-	-	-
metab_13301	3.2972	230.1397	neg	N-(Tert-Butoxycarbonyl)-L-leucine	C11H21NO4	231.1470	83170	-	-	-	-
metab_13301	3.2972	230.1397	neg	O-Butanoylcarnitine	C11H21NO4	231.1470	439829	-	-	-	-
metab_5335	2.5782	230.1745	pos	N-Decanoylglycine	C12H23NO3	229.1676	1712391	-	-	-	-
metab_2572	6.1927	230.2472	pos	Xestoaminol C	C14H31NO	229.2403	14756407	-	-	LMSP01080033	
metab_9213	3.7830	231.0064	neg	Piperonyl acetate	C10H10O4	194.0579	9473	Benzene and substituted derivatives	Benzyloxycarbonyls	HMDB0032614	Piperonyl acetate

metab_13956	1.9439	231.0872	neg	5-(2-Methylpropyl)tetrahydro-2-oxo-3-furancarboxylic acid	C9H14O4	186.0892	131751114	Lactones	Gamma butyrolactones	HMDB0030988	5-(2-Methylpropyl)tetrahydro-2-oxo-3-furancarboxylic acid
metab_8844	2.6206	231.1236	neg	1,2-Dibutyrin	C11H20O5	232.1311	10177014	-	-	-	-
metab_8902	2.7792	231.1236	neg	1,2-Dibutyrin	C11H20O5	232.1311	10177014	-	-	-	-
metab_13258	3.4303	231.1237	neg	1,2-Dibutyrin	C11H20O5	232.1311	10177014	-	-	-	-
metab_9004	3.0432	231.1237	neg	1,2-Dibutyrin	C11H20O5	232.1311	10177014	Prenol lipids	Monoterpenoids	HMDB0038978	(1beta,2beta,5beta)-p-Menth-3-ene-1,2,5-triol
metab_8048	0.9933	231.1347	neg	Thr-Leu	C10H20N2O4	232.1423	7021828	-	-	-	-
metab_8112	1.1796	231.1348	neg	Thr-Leu	C10H20N2O4	232.1423	7021828	-	-	-	-
metab_14297	1.4856	231.1349	neg	Thr-Leu	C10H20N2O4	232.1423	7021828	-	-	-	-
metab_14062	1.7572	231.1350	neg	Thr-Leu	C10H20N2O4	232.1423	7021828	-	-	-	-
metab_12697	5.4844	231.1600	neg	3,12-Dihydroxylauric acid	C12H24O4	232.1675	18408222	-	-	-	-
metab_13192	3.6142	231.1600	neg	3,12-Dihydroxylauric acid	C12H24O4	232.1675	18408222	-	-	-	-
metab_6967	4.3549	231.1600	neg	3,12-Dihydroxylauric acid	C12H24O4	232.1675	18408222	-	-	-	-
metab_13137	3.7658	231.1600	neg	3,12-Dihydroxylauric acid	C12H24O4	232.1675	18408222	-	-	-	-
metab_9077	3.2972	231.1600	neg	3,12-Dihydroxylauric acid	C12H24O4	232.1675	18408222	-	-	-	-
metab_1629	1.5705	231.1696	pos	Ile-val	C11H22N2O3	230.1630	449407	-	-	-	-
metab_1629	1.5705	231.1696	pos	Val-Leu	C11H22N2O3	230.1630	6993118	-	-	-	-
metab_5741	1.7271	231.1697	pos	Ile-val	C11H22N2O3	230.1630	449407	-	-	-	-
metab_5741	1.7271	231.1697	pos	Val-Leu	C11H22N2O3	230.1630	6993118	-	-	-	-
metab_1068	1.4289	232.1536	pos	N-(Tert-Butoxycarbonyl)-L-leucine	C11H21NO4	231.1470	83170	-	-	-	-
metab_1068	1.4289	232.1536	pos	O-Butanoylcarnitine	C11H21NO4	231.1470	439829	-	-	-	-
metab_9355	4.3380	233.0818	neg	2-[2,4-Dihydroxy-3-(3-methylbut-2-en-1-yl)phenyl]-2-hydro	C13H16O5	252.0998	131839409	Phenols	Benzenediols	HMDB0137142	2-[2,4-dihydroxy-3-(3-methylbut-2-en-1-yl)phenyl]-2-hydroxyacetic

				xyacetic acid							acid
metab_14015	1.8371	233.1028	neg	(3R)-3-{[(2R,3R,5R,6S)-3,5-Dihydroxy-6-methyltetrahydro-2H-pyran-2-yl]oxy}butanoic acid	C10H18O6	234.1108	86289642	-	-	-	-
metab_13473	2.8606	233.1029	neg	(3R)-3-{[(2R,3R,5R,6S)-3,5-Dihydroxy-6-methyltetrahydro-2H-pyran-2-yl]oxy}butanoic acid	C10H18O6	234.1108	86289642	-	-	-	-
metab_6262	0.7801	233.1125	pos	N2-Succinyl-L-ornithine	C9H16N2O5	232.1059	127370	Carboxylic acids and derivatives	Amino acids, peptides, and analogues	HMDB0001199	N2-Succinyl-L-ornithine
metab_13743	2.3227	233.1393	neg	(1R,2R,4R,8R)-p-Menthane-2,8,9-triol	C10H20O3	188.1412	73809747	Prenol lipids	Monoterpenoids	HMDB0033574	(1R,2R,4R,8R)-p-Menthane-2,8,9-triol
metab_4566	5.8597	233.1529	pos	3,12-dihydroxydodecanoic acid	C12H24O4	232.1675	18408222	-	-	-	-
metab_9753	6.4709	233.1545	neg	Confertifolin	C15H22O2	234.1618	442187	-	-	-	-
metab_4545	5.9504	233.2257	pos	1,8,11,14-Heptadecatetraene, (Z,Z,Z)-	C17H28	232.2190	5352710	-	-	-	-
metab_2609	6.6148	233.2258	pos	1,8,11,14-Heptadecatetraene, (Z,Z,Z)-	C17H28	232.2190	5352710	-	-	-	-
metab_6299	0.6542	234.0965	pos	N-(1-Deoxy-1-fructosyl)alanine	C9H17NO7	251.1005	101039147	-	-	HMDB0038662	N-(1-Deoxy-1-fructosyl)alanine
metab_7427	4.1869	234.1135	neg	Pandamarilactam 3x	C13H17NO3	235.1208	100951965	Dihydrofurans	Furanones	HMDB0033610	Pandamarilactam 3x
metab_14155	1.6335	235.0723	neg	6-Hydroxy-1H-indole-3-acetamide	C10H10N2O2	190.0742	286538	Indoles and derivatives	Hydroxyindoles	HMDB0031173	6-Hydroxy-1H-indole-3-acetamide
metab_13528	2.7475	235.1087	neg	DL-Alanyl-DL-phenylalanine	C12H16N2O3	236.1159	2080	-	-	-	-

metab_13528	2.7475	235.1087	neg	Phe-ala	C12H16N2O3	236.1159	5488196	-	-	-	-
metab_8395	1.6186	235.1087	neg	DL-Alanyl-DL-phenylalanine	C12H16N2O3	236.1159	2080	-	-	-	-
metab_8395	1.6186	235.1087	neg	Phe-ala	C12H16N2O3	236.1159	5488196	-	-	-	-
metab_9133	3.5141	235.1088	neg	DL-Alanyl-DL-phenylalanine	C12H16N2O3	236.1159	2080	-	-	-	-
metab_9133	3.5141	235.1088	neg	Phe-ala	C12H16N2O3	236.1159	5488196	-	-	-	-
metab_1704	1.7705	235.1435	pos	H-Thr-Leu-OH	C10H20N2O4	232.1423	7021828	-	-	-	-
metab_2673	7.0082	235.1686	pos	Confertifolin	C15H22O2	234.1618	442187	-	-	-	-
metab_5602	2.0211	235.1686	pos	Confertifolin	C15H22O2	234.1618	442187	-	-	-	-
metab_4742	4.9115	235.1686	pos	Confertifolin	C15H22O2	234.1618	442187	-	-	-	-
metab_5542	2.1290	235.1797	pos	Lidocaine	C14H22N2O	234.1731	3676	-	-	-	-
metab_5116	3.2727	235.1799	pos	Lidocaine	C14H22N2O	234.1731	3676	-	-	-	-
metab_14399	1.3669	236.0563	neg	(2-Oxo-2,3-dihydro-1H-indol-3-yl)acetic acid	C10H9NO3	191.0582	3080590	Indoles and derivatives	Indolyl carboxylic acids and derivatives	HMDB0035514	xi-2,3-Dihydro-2-oxo-1H-indole-3-acetic acid
metab_7493	0.5991	236.0774	neg	N-Acetyl-D-glucosaminate	C8H15NO7	237.0849	16126799	-	-	-	-
metab_5409	2.4071	236.0912	pos	Benzylmalonic acid	C10H10O4	194.0579	12031	Phenylpropanoic acids	-	HMDB0142175	2-benzylpropanedioic acid
metab_5620	1.9918	236.1107	pos	CHEBI:133389	C10H21NOS2	235.1054	44237117	-	-	-	-
metab_8919	2.8123	237.0405	neg	3,4-Bis(methoxycarbonyl)benzoic acid	C11H10O6	238.0478	610016	-	-	-	-
metab_7975	0.7818	237.0614	neg	Xanthopterin-B2	C9H10N4O4	238.0694	439706	Organooxygen compounds	Carbohydrates and carbohydrate conjugates	HMDB0131294	3,4,5-trihydroxy-6-(2-hydroxyethoxy)oxane-2-carboxylic acid
metab_7975	0.7818	237.0614	neg	3,4,5-Trihydroxy-6-(2-hydroxyethoxy)oxane-2-carboxylic acid	C8H14O8	238.0689	131836625	Organooxygen compounds	Carbohydrates and carbohydrate conjugates	HMDB0131294	3,4,5-trihydroxy-6-(2-hydroxyethoxy)oxane-2-carboxylic acid
metab_8952	2.9092	237.0767	neg	Swerilactone L, (rel)-	C12H14O5	238.0842	53483970	-	-	-	-
metab_276	1.1306	237.0862	pos	N-Formyl-D-kynurenine	C11H12N2O4	236.0796	11954215	Carboxylic acids and	Amino acids, peptides,	HMDB0029445	L-Agaridoxin

								derivatives	and analogues		
metab_276	1.1306	237.0862	pos	L-Agaridoxin	C11H14N2O5	254.0903	131750870	Carboxylic acids and derivatives	Amino acids, peptides, and analogues	HMDB0029445	L-Agaridoxin
metab_1561	1.4005	237.1226	pos	DL-Alanyl-DL-phenylalanine	C12H16N2O3	236.1159	2080	Carboxylic acids and derivatives	Amino acids, peptides, and analogues	HMDB0029443	L-Pyridosine
metab_1561	1.4005	237.1226	pos	Phe-ala	C12H16N2O3	236.1159	5488196	Carboxylic acids and derivatives	Amino acids, peptides, and analogues	HMDB0029443	L-Pyridosine
metab_1561	1.4005	237.1226	pos	L-Pyridosine	C12H18N2O4	254.1267	15664012	Carboxylic acids and derivatives	Amino acids, peptides, and analogues	HMDB0029443	L-Pyridosine
metab_2001	2.8568	237.1228	pos	DL-Alanyl-DL-phenylalanine	C12H16N2O3	236.1159	2080	-	-	-	-
metab_2001	2.8568	237.1228	pos	Phe-ala	C12H16N2O3	236.1159	5488196	-	-	-	-
metab_2005	2.8725	237.1479	pos	4-Heptyloxybenzoic acid	C14H20O3	236.1410	85154	-	-	-	-
metab_2806	7.9407	237.2206	pos	(10E,12Z)-Hexadeca-10,12-dienal	C16H28O	236.2138	10922550	-	-	-	-
metab_2828	8.1132	237.2207	pos	(10E,12Z)-Hexadeca-10,12-dienal	C16H28O	236.2138	10922550	-	-	-	-
metab_478	2.7188	238.1068	pos	N-Lactoyl-phenylalanine	C12H15NO4	237.1001	11075454	Carboxylic acids and derivatives	Amino acids, peptides, and analogues	HMDB0062175	N-lactoyl-Phenylalanine
metab_283	1.1589	238.1179	pos	N-Acetyl-D-glucosaminate	C8H15NO7	237.0849	16126799	-	-	-	-
metab_1943	2.5938	238.1796	pos	Piroctone	C14H23NO2	237.1728	50259	-	-	-	-
metab_1748	1.9609	239.0512	pos	3,4-Bis(methoxycarbonyl)benzoic acid	C11H10O6	238.0478	610016	-	-	-	-
metab_14093	1.7264	239.0562	neg	2-(3-Carboxypropionyl)-6-hydroxy-cyclohexa-2,4-diene carboxylic acid	C11H12O6	240.0635	5287432	-	-	-	-
metab_5063	3.4702	239.1102	pos	Swerilactone L	C12H14O5	238.0842	53483970	-	-	-	-

metab_12683	5.5639	239.1288	neg	3-[(3As,4S,5R,7aS)-5-hydroxy-7a-methyl-1,5-dioxo-octahydroinden-4-yl]propanoic acid	C13H20O4	240.1362	86290215	-	-	-	-
metab_12784	5.0159	239.1288	neg	3-[(3As,4S,5R,7aS)-5-hydroxy-7a-methyl-1,5-dioxo-octahydroinden-4-yl]propanoic acid	C13H20O4	240.1362	86290215	-	-	-	-
metab_13373	3.1115	239.1288	neg	3-[(3As,4S,5R,7aS)-5-hydroxy-7a-methyl-1,5-dioxo-octahydroinden-4-yl]propanoic acid	C13H20O4	240.1362	86290215	-	-	-	-
metab_12847	4.7709	239.1289	neg	3-[(3As,4S,5R,7aS)-5-hydroxy-7a-methyl-1,5-dioxo-octahydroinden-4-yl]propanoic acid	C13H20O4	240.1362	86290215	-	-	-	-
metab_3421	14.4245	239.1482	pos	Pentaethylene glycol	C10 H22 O6	238.1415	62551	-	-	-	-
metab_46	1.5284	239.1489	pos	Pentaethylene glycol	C10 H22 O6	238.1415	62551	-	-	-	-
metab_1531	1.2725	240.1335	pos	Lysylglutamic acid	C11H21N3O5	275.1481	7010502	Carboxylic acids and derivatives	Amino acids, peptides, and analogues	HMDB0028950	Lysyl-Glutamate
metab_13923	2.0055	241.1193	neg	Glutamylisoleucine	C11H20N2O5	260.1372	9813855	Carboxylic acids and derivatives	Amino acids, peptides, and analogues	HMDB0028822	Glutamylisoleucine
metab_9513	5.1456	241.1444	neg	2-Isocapryloyl-3R-hydroxymethyl-gamma-butyrolactone	C13H22O4	242.1519	119102	-	-	-	-
metab_13377	3.0942	241.1445	neg	2-Isocapryloyl-3R-hydroxymethyl-gamma-butyrolactone	C13H22O4	242.1519	119102	-	-	-	-
metab_12974	4.3037	241.1445	neg	2-Isocapryloyl-3R-hydroxymethyl-gamma-butyrolactone	C13H22O4	242.1519	119102	-	-	-	-
metab_1408	0.9200	241.1539	pos	Pirbuterol	C12H20N2O3	240.1472	4845	-	-	-	-
metab_7365	7.8193	241.2172	neg	Pentadecanoic acid	C15H30O2	242.2247	13849	-	-	-	-

metab_11993	8.0373	241.2172	neg	Pentadecanoic acid	C15H30O2	242.2247	13849	-	-	-	-
metab_10299	8.7031	241.2173	neg	Pentadecanoic acid	C15H30O2	242.2247	13849	-	-	-	-
metab_8053	1.0074	242.1145	neg	L-alanylglycyl-L-proline	C10H17N3O4	243.1219	51389804	-	-	-	-
metab_12754	5.1772	242.1397	neg	3-Hydroxy-N-[(3S)-tetrahydro -2-oxo-3-furanyl]-octanamide	C12H21NO4	243.1470	66769741	-	-	-	-
metab_13134	3.7658	242.1398	neg	3-Hydroxy-N-[(3S)-tetrahydro -2-oxo-3-furanyl]-octanamide	C12H21NO4	243.1470	66769741	-	-	-	-
metab_12896	4.6056	242.1761	neg	N-Undecanoylglycine	C13H25NO3	243.1833	454092	-	-	-	-
metab_9025	3.1115	242.1762	neg	N-Undecanoylglycine	C13H25NO3	243.1833	454092	-	-	-	-
metab_13318	3.2473	242.1762	neg	N-Undecanoylglycine	C13H25NO3	243.1833	454092	-	-	-	-
metab_7976	0.7818	243.0621	neg	Uridine	C9H12N2O6	244.0695	6029	Pyrimidine nucleosides	-	HMDB0000296	Uridine
metab_13655	2.4970	243.0774	neg	Uridine	C9H12N2O6	244.0695	6029	Pyrimidine nucleosides	Pyrimidines and pyrimidine derivatives	HMDB0000296	1-b-D-Ribofuranosyl-2,4(1H,3H)-p yrimidinedione
metab_8875	2.7155	243.1238	neg	Pandangolide 1	C12H20O5	244.1310	11557800	-	-	-	-
metab_9085	3.3304	243.1238	neg	Pandangolide 1	C12H20O5	244.1310	11557800	Fatty Acyls	Fatty acids and conjugates	HMDB0032472	Polyethylene, oxidized
metab_13154	3.7317	243.1238	neg	Pandangolide 1	C12H20O5	244.1310	11557800	-	-	-	-
metab_14549	1.1503	243.1462	neg	Pandangolide 1	C12H20O5	244.131	11557800	-	-	-	-
metab_12707	5.4355	243.1601	neg	Tridecanedioic acid	C13H24O4	244.1675	10458	Fatty Acyls	Fatty acids and conjugates	HMDB0002327	1,11-Undecanedicarboxylic acid
metab_9018	3.0774	243.1602	neg	Tridecanedioic acid	C13H24O4	244.1675	10458	-	-	-	-
metab_5799	1.6128	243.1694	pos	A Factor	C13H22O4	242.1519	119102	-	-	-	-
metab_5869	1.5004	243.1695	pos	A Factor	C13H22O4	242.1519	119102	-	-	-	-
metab_382	1.7993	243.1700	pos	A Factor	C13H22O4	242.1519	119102	-	-	-	-
metab_13997	1.8682	243.1713	neg	Ile-Leu	C12H24N2O3	244.1786	7019083	-	-	-	-

metab_13879	2.0670	243.1714	neg	Ile-Leu	C12H24N2O3	244.1786	7019083	-	-	-	-
metab_13804	2.2119	243.1714	neg	Ile-Leu	C12H24N2O3	244.1786	7019083	-	-	-	-
metab_5959	1.3293	243.1808	pos	A Factor	C13H22O4	242.1519	119102	-	-	-	-
metab_12084	7.7567	243.1965	neg	2-Hydroxytetradecanoic acid	C14H28O3	244.2038	1563	Fatty Acyls	Fatty acids and conjugates	HMDB0002261; LMFA01050484	2-Hydroxymyristic acid
metab_12084	7.7567	243.1965	neg	alpha-Hydroxy myristic acid	C14H28O3	244.2038	656737	Fatty Acyls	Fatty acids and conjugates	HMDB0002261; LMFA01050484	2-Hydroxymyristic acid
metab_2433	5.1372	243.2100	pos	Pentadecylic acid	C15H30O2	242.2247	13849	Fatty Acyls	Fatty acids and conjugates	HMDB0000826	Pentadecanoic acid
metab_1117	0.7801	244.1284	pos	L-Alanylglycyl-L-proline	C10H17N3O4	243.1219	51389804	-	-	-	-
metab_8607	2.0358	244.1300	neg	Gly-gly-ile	C10H19N3O4	245.1375	7020346	-	-	-	-
metab_14353	1.4427	244.1301	neg	Gly-gly-ile	C10H19N3O4	245.1375	7020346	-	-	-	-
metab_8024	0.9086	244.1301	neg	Gly-gly-ile	C10H19N3O4	245.1375	7020346	-	-	-	-
metab_49	1.6838	244.1536	pos	3-Hydroxy-N-[(3S)-tetrahydro -2-oxo-3-furanyl]-octanamide	C12H21NO4	243.1469	66769741	-	-	-	-
metab_14131	1.6642	244.1553	neg	Isovalerylcarnitine	C12H23NO4	245.1626	6426851	-	-	-	-
metab_12919	4.5391	244.1554	neg	Isovalerylcarnitine	C12H23NO4	245.1626	6426851	-	-	-	-
metab_13139	3.7658	244.1554	neg	Isovalerylcarnitine	C12H23NO4	245.1626	6426851	-	-	-	-
metab_5270	2.7959	244.1901	pos	N-Undecanoylglycine	C13H25NO3	243.1833	454092	-	-	-	-
metab_5126	3.2420	244.1901	pos	N-Undecanoylglycine	C13H25NO3	243.1833	454092	-	-	-	-
metab_1921	2.5003	245.0803	pos	Piceatannol	C14H12O4	244.0736	667639	-	-	-	-
metab_7622	2.5893	245.0931	neg	N-Acetyl-DL-tryptophan	C13H14N2O3	246.1004	2002	-	-	-	-
metab_14052	1.7729	245.0933	neg	N-Acetyl-DL-tryptophan	C13H14N2O3	246.1004	2002	-	-	-	-
metab_14627	0.9649	245.1142	neg	2,4-Bis(acetylamino)-2,4,6-tri deoxy-D-mannopyranose	C10H18N2O5	246.1215	46833331	-	-	-	-

metab_14279	1.5155	245.1143	neg	2,4-Bis(acetylamino)-2,4,6-trideoxy-D-mannopyranose	C10H18N2O5	246.1215	46833331	-	-	-	-
metab_8124	1.2084	245.1143	neg	Glutamylvaline	C10H18N2O5	246.1216	433630	Carboxylic acids and derivatives	Amino acids, peptides, and analogues	HMDB0028832	Glutamylvaline
metab_8124	1.2084	245.1143	neg	2,4-Bis(acetylamino)-2,4,6-trideoxy-D-mannopyranose	C10H18N2O5	246.1215	46833331	Carboxylic acids and derivatives	Amino acids, peptides, and analogues	HMDB0028832	Glutamylvaline
metab_5897	1.4429	245.1281	pos	Pandangolide 1	C12H20O5	244.1309	11557800	-	-	-	-
metab_2010	2.8877	245.1376	pos	Pandangolide 1	C12H20O5	244.1309	11557800	-	-	-	-
metab_5319	2.6244	245.1377	pos	Pandangolide 1	C12H20O5	244.1309	11557800	-	-	-	-
metab_13322	3.2473	245.1394	neg	Ascaroside C6	C12H22O5	246.1467	16066475	Hydroxy acids and derivatives	Medium-chain hydroxy acids and derivatives	HMDB0032662; LMFA01050436	(S)-9-Hydroxy-10-undecenoic acid
metab_9275	4.0182	245.1395	neg	Ascaroside C6	C12H22O5	246.1467	16066475	-	-	-	-
metab_12886	4.6216	245.1395	neg	Ascaroside C6	C12H22O5	246.1467	16066475	-	-	-	-
metab_1616	1.5284	245.1851	pos	6-(6-Aminohexanamido)hexanoate	C12H24N2O3	244.1785	5460073	-	-	-	-
metab_1616	1.5284	245.1851	pos	Ile-Leu	C12H24N2O3	244.1786	7019083	-	-	-	-
metab_5376	2.4850	245.1854	pos	6-(6-Aminohexanamido)hexanoate	C12H24N2O3	244.1785	5460073	-	-	-	-
metab_5376	2.4850	245.1854	pos	Ile-Leu	C12H24N2O3	244.1786	7019083	-	-	-	-
metab_5492	2.2209	245.1854	pos	6-(6-Aminohexanamido)hexanoate	C12H24N2O3	244.1785	5460073	-	-	-	-
metab_5492	2.2209	245.1854	pos	Ile-Leu	C12H24N2O3	244.1786	7019083	-	-	-	-
metab_1782	2.0677	245.1854	pos	6-(6-Aminohexanamido)hexanoate	C12H24N2O3	244.1785	5460073	-	-	-	-
metab_1782	2.0677	245.1854	pos	Ile-Leu	C12H24N2O3	244.1786	7019083	-	-	-	-
metab_13985	1.8833	246.0983	neg	D-1-[(3-Carboxypropyl)amino	C10H19NO7	265.1162	131752417	-	-	HMDB0038663	D-1-[(3-Carboxypropyl)amino]-1-d

				]-1-deoxyfructose							eoxyfructose
metab_5127	3.2420	246.1119	pos	3-(4-Hydroxy-3-methoxyphenyl)-N-(4-oxobutyl)prop-2-enimidic acid	C14H17NO4	263.1158	131839454	Cinnamic acids and derivatives	Hydroxycinnamic acids and derivatives	HMDB0139920	3-(4-hydroxy-3-methoxyphenyl)-N-(4-oxobutyl)prop-2-enimidic acid
metab_8595	2.0202	246.1346	neg	3-Hydroxybutyrylcarnitine	C11H21NO5	247.1420	71464477	-	-	-	-
metab_384	1.8277	246.1692	pos	2-Methylbutyroylcarnitine	C12H23NO4	245.1627	6426901	Fatty Acyls	Fatty acid esters	HMDB0000378; LMFA07070034	2-Methylbutyroylcarnitine
metab_384	1.8277	246.1692	pos	Isovalerylcarnitine	C12H23NO4	245.1626	6426851	Fatty Acyls	Fatty acid esters	HMDB0000378; LMFA07070034	2-Methylbutyroylcarnitine
metab_9039	3.1626	247.0811	neg	H-Val-Met-OH	C10H20N2O3S	248.1194	6993039	-	-	-	-
metab_1949	2.6090	247.1071	pos	N-Acetyl-DL-tryptophan	C13H14N2O3	246.100442	2002	-	-	-	-
metab_289	1.2016	247.1281	pos	2,4-Bis(acetylamino)-2,4,6-trideoxy-D-mannopyranose	C10H18N2O5	246.1214	46833331	-	-	-	-
metab_13305	3.2972	247.1551	neg	2-Hydroxyundecanoic acid	C11H22O3	202.1569	5282899	Fatty Acyls	Fatty acids and conjugates	HMDB0059736	2-Hydroxyundecanoate
metab_437	2.2665	248.1122	pos	Valinopine	C10H17NO6	247.1056	15284427	-	-	-	-
metab_6945	4.1869	248.1293	neg	2-Pentanamido-3-phenylpropanoic acid	C14H19NO3	249.1365	13890535	Carboxylic acids and derivatives	Amino acids, peptides, and analogues	HMDB0094646	2-Pentanamido-3-phenylpropanoic acid
metab_14517	1.2235	249.0703	neg	1-Deoxy-11beta-hydroxypentalenate	C15H22O3	250.1567	70679015	-	-	-	-
metab_5759	1.6838	249.0862	pos	Asp-Asp	C8H12N2O7	248.0643	471583	-	-	-	-
metab_1744	1.9463	249.1324	pos	H-Val-Met-OH	C10H20N2O3S	248.1194	6993039	-	-	-	-
metab_14446	1.2948	249.1344	neg	Xanthoxin	C15H22O3	250.1569	5282222	-	-	-	-

metab_631	5.1372	249.1478	pos	3,14-Dihydroxy-11,13-dihydro costunolide	C15H22O4	266.1518	131751828	Prenol lipids	Terpene lactones	HMDB0035647	3,14-Dihydroxy-11,13-dihydrocostu nolide
metab_27	5.6623	249.1842	pos	(4Z,7Z,10Z,13Z)-Hexadeca-4, 7,10,13-tetraenoic acid	C16H24O2	248.1774	5312433	-	-	-	-
metab_7461	1.5885	250.0721	neg	N-Feruloylglycine	C12H13NO5	251.0793	5280527	-	-	-	-
metab_7538	2.0202	250.0722	neg	N-Feruloylglycine	C12H13NO5	251.0793	5280527	-	-	-	-
metab_12284	7.0959	250.1449	neg	Furmecyclox	C14H21NO3	251.1523	43359	-	-	-	-
metab_14026	1.8205	250.1449	neg	Furmecyclox	C14H21NO3	251.1523	43359	-	-	-	-
metab_12450	6.5033	250.1449	neg	Furmecyclox	C14H21NO3	251.1523	43359	-	-	-	-
metab_8930	2.8440	251.0563	neg	Phenylglucuronide	C12H14O7	270.0740	119239	Organooxygen compounds	Carbohydrates and carbohydrate conjugates	HMDB0059806	Phenylglucuronide
metab_14040	1.8046	251.1037	neg	3-Hydroxyhexobarbital	C12H16N2O4	252.1110	439898	-	-	-	-
metab_14040	1.8046	251.1037	neg	Phenylalanylserine	C12H16N2O4	252.1109	193508	-	-	-	-
metab_8570	1.9745	251.1037	neg	3-Hydroxyhexobarbital	C12H16N2O4	252.1110	439898	-	-	-	-
metab_8570	1.9745	251.1037	neg	Phenylalanylserine	C12H16N2O4	252.1109	193508	-	-	-	-
metab_9282	4.0347	251.1037	neg	3-Hydroxyhexobarbital	C12H16N2O4	252.1110	439898	-	-	-	-
metab_9282	4.0347	251.1037	neg	Phenylalanylserine	C12H16N2O4	252.1109	193508	-	-	-	-
metab_14255	1.5297	251.1038	neg	3-Hydroxyhexobarbital	C12H16N2O4	252.1110	439898	-	-	-	-
metab_14255	1.5297	251.1038	neg	Phenylalanylserine	C12H16N2O4	252.1109	193508	-	-	-	-
metab_5707	1.7845	251.1381	pos	N-Caffeoylputrescine	C13H18N2O3	250.1317	5280559	Phenols	Benzenediols	HMDB0029876	Paucine
metab_617	4.8809	251.1635	pos	1-Deoxy-11beta-hydroxypenta lenate	C15H22O3	250.1567	70679015	-	-	-	-
metab_4862	4.3512	251.1635	pos	1-Deoxy-11beta-hydroxypenta lenate	C15H22O3	250.1567	70679015	Carbonyl compounds	Acyloins	HMDB0030917	1-Hydroxyacorenone
metab_521	2.4530	251.1636	pos	1-Deoxy-11beta-hydroxypenta	C15H22O3	250.1567	70679015	-	-	-	-

				lenate							
metab_12705	5.4355	251.1653	neg	Juvenile hormone III acid	C15H24O3	252.1726	23724771	-	-	-	-
metab_14571	1.0927	252.0514	neg	N-Pyruvoyl-5-methoxy-3-hydroxyanthranilate	C11H11NO6	253.0588	443220	-	-	-	-
metab_7642	2.7155	252.0515	neg	N-Pyruvoyl-5-methoxy-3-hydroxyanthranilate	C11H11NO6	253.0588	443220	-	-	-	-
metab_55	2.0211	252.0858	pos	N-Feruloylglycine	C12H13NO5	251.0793	5280527	-	-	-	-
metab_5922	1.4005	252.0858	pos	N-Feruloylglycine	C12H13NO5	251.0793	5280527	-	-	-	-
metab_14144	1.6491	252.0878	neg	N-Lactoyl-Tyrosine	C12H15NO5	253.0950	22167491	Carboxylic acids and derivatives	Amino acids, peptides, and analogues	HMDB0062177	N-lactoyl-Tyrosine
metab_1376	0.8221	252.1073	pos	5'-Deoxyadenosine	C10H13N5O3	251.1018	439182	-	-	-	-
metab_6049	1.1875	252.1334	pos	5'-Deoxyadenosine	C10H13N5O3	251.1018	439182	5'-deoxyribonucleosides	Amino acids, peptides, and analogues	HMDB0001983	5'-Deoxyadenosine
metab_1574	1.4289	252.1585	pos	Furmecyclox	C14H21NO3	251.1521	43359	-	-	-	-
metab_2029	2.9652	252.1589	pos	Furmecyclox	C14H21NO3	251.1521	43359	-	-	-	-
metab_6143	1.0040	253.0560	pos	S-(4-Methylthiobutylthiohydroximoyl)-L-cysteine	C8H16N2O3S2	252.0595	139031736	-	-	-	-
metab_8702	2.2754	253.1082	neg	(Z)-3-Oxo-2-(2-pentenyl)-1-cyclopenteneacetic acid	C12H16O3	208.1099	14262444	Organooxygen compounds	Carbonyl compounds	HMDB0030197	(Z)-3-Oxo-2-(2-pentenyl)-1-cyclopenteneacetic acid
metab_301	1.3013	253.1176	pos	3-Hydroxyhexobarbital	C12H16N2O4	252.1110	439898	-	-	-	-
metab_301	1.3013	253.1176	pos	Phenylalanylserine	C12H16N2O4	252.1109	193508	-	-	-	-
metab_1060	1.5004	253.1176	pos	3-Hydroxyhexobarbital	C12H16N2O4	252.1110	439898	-	-	-	-
metab_1060	1.5004	253.1176	pos	Phenylalanylserine	C12H16N2O4	252.1109	193508	-	-	-	-
metab_8246	1.4115	253.1195	neg	2-Formyl-5-(hydroxymethyl)pyrrole-1-norleucine	C12H18N2O4	254.1267	122228	Carboxylic acids and derivatives	Amino acids, peptides, and analogues	HMDB0033143	Pyrraline

metab_12069	7.7875	253.2173	neg	Hexadecenoic acid	C16H30O2	254.2247	5282743	-	-	-	-
metab_12069	7.7875	253.2173	neg	Palmitoleic acid	C16H30O2	254.2247	445638	-	-	-	-
metab_11730	8.7031	253.2174	neg	Hexadecenoic acid	C16H30O2	254.2247	5282743	-	-	-	-
metab_11730	8.7031	253.2174	neg	Palmitoleic acid	C16H30O2	254.2247	445638	-	-	-	-
metab_2644	6.8728	253.2518	pos	(9Z)-Cycloheptadec-9-en-1-ol	C17H32O	252.2452	5364514	-	-	-	-
metab_3119	9.8522	253.2520	pos	(9Z)-Cycloheptadec-9-en-1-ol	C17H32O	252.2452	5364514	-	-	-	-
metab_14283	1.5012	254.0863	neg	HC Blue No.1	C11H17N3O4	255.1218	17734	-	-	-	-
metab_2803	7.9260	254.2470	pos	Palmitoleamide	C16H31NO	253.2406	56936054	-	-	LMFA08010010	
metab_481	2.8725	255.1584	pos	Aspergillide B	C14H22O4	254.1515	24766619	-	-	-	-
metab_12821	4.8865	255.1603	neg	3-Hydroxytetradecanedioic acid	C14H26O5	274.1780	20848956	Fatty Acyls	Fatty acids and conjugates	HMDB0000394	3-Hydroxytetradecanedioic acid
metab_2074	3.1495	255.2186	pos	(9Z)-Hexadecenoic acid	C16H30O2	254.2247	445638	Fatty Acyls	Isoflav-2-enes	HMDB0003229	cis-9-Hexadecenoic acid
metab_10152	8.1774	255.2330	neg	2-Hexyldecanoic acid	C16H32O2	256.2404	32912	-	-	-	-
metab_11867	8.4022	255.2330	neg	2-Hexyldecanoic acid	C16H32O2	256.2404	32912	-	-	-	-
metab_10407	9.1038	255.2330	neg	2-Hexyldecanoic acid	C16H32O2	256.2404	32912	-	-	-	-
metab_212	0.6542	256.0688	pos	Nicotinate D-ribonucleoside	C11H13NO6	255.074	161234	Organooxygen compounds	Hydroxysteroids	HMDB0006809	Nicotinic acid D-ribonucleoside
metab_1141	0.6123	256.0807	pos	D-Ribosylnicotinic acid	C11H13NO6	255.0741	161234	-	-	-	-
metab_6839	0.7818	256.0939	neg	Pyro-L-glutaminyl-L-glutamin e	C10H15N3O5	257.1012	14889607	Carboxylic acids and derivatives	Amino acids, peptides, and analogues	HMDB0039229	Pyro-L-glutaminyl-L-glutamine
metab_2505	5.6934	256.1325	pos	(2E)-Piperamide-C5:1	C16H19NO3	273.1365	12073743	-	-	HMDB0038834	(2E)-Piperamide-C5:1
metab_12548	6.1320	256.1919	neg	N-Lauroylglycine	C14H27NO3	257.1989	346152	-	-	-	-
metab_2906	8.5392	256.2521	pos	Palmitic amide	C16H33NO	255.256	69421	Fatty Acyls	Amines	HMDB0012273	Amide 16
metab_2903	8.5241	256.2627	pos	Hexadecanamide	C16H33NO	255.2560	69421	Fatty Acyls	Fatty amides	HMDB0012273	Palmitic amide
metab_7949	0.6551	257.0779	neg	Spongothymidine	C10H14N2O6	258.0854	65049	-	-	-	-

metab_7996	0.8240	257.0780	neg	Spongothymidine	C10H14N2O6	258.0854	65049	-	-	-	-
metab_8778	2.4487	257.1144	neg	(2S,2'S)-Pyrosaccharopine	C11H18N2O5	258.1216	131752421	-	-	HMDB0038676	(2S,2'S)-Pyrosaccharopine
metab_14527	1.2084	257.1144	neg	gamma-L-Glutamyl-L-pipecolic acid	C11H18N2O5	258.1216	69247902	Carboxylic acids and derivatives	Amino acids, peptides, and analogues	HMDB0038614	gamma-L-Glutamyl-L-pipecolic acid
metab_5452	2.3133	257.1278	pos	Phenylbutyrylglutamine	C15H20N2O4	292.1423	9943917	Carboxylic acids and derivatives	Amino acids, peptides, and analogues	HMDB0011687	Phenylbutyrylglutamine
metab_13334	3.2130	257.1396	neg	(+)-Cucurbic acid	C12H20O3	212.1412	5281159	Fatty Acyls	Lineolic acids and derivatives	HMDB0029388	Cucurbic acid
metab_14335	1.4568	257.1621	neg	Tetradecanedioic acid	C14 H26 O4	258.1833	13185	Fatty Acyls	Fatty acids and conjugates	HMDB0000872	1,12-Dodecanedicarboxylate
metab_12745	5.2257	257.1759	neg	Tetradecanedioic acid	C14 H26 O4	258.1833	13185	Fatty Acyls	Fatty acids and conjugates	LMFA01170018; HMDB0000872	Tetradecanedioic acid
metab_12612	5.8569	257.1759	neg	Tetradecanedioic acid	C14 H26 O4	258.1833	13185	-	-	-	-
metab_12799	4.9510	257.1760	neg	Tetradecanedioic acid	C14 H26 O4	258.1833	13185	-	-	-	-
metab_11940	8.1623	257.2124	neg	15-Hydroxypentadecanoic acid	C15H30O3	258.2197	78360	Fatty Acyls	Fatty acids and conjugates	HMDB0061657	3-hydroxypentadecanoic acid
metab_8165	1.2805	258.0985	neg	N-(1-Deoxy-1-fructosyl)proline	C11H19NO7	277.1162	131752381	Carboxylic acids and derivatives	Amino acids, peptides, and analogues	HMDB0038493	N-(1-Deoxy-1-fructosyl)proline
metab_14668	0.8803	258.1459	neg	Leu-Ala-Gly	C11H21N3O4	259.1532	9834954	-	-	-	-
metab_14363	1.4427	258.1461	neg	Leu-Ala-Gly	C11H21N3O4	259.1532	9834954	-	-	-	-
metab_13798	2.2119	258.1463	neg	Leu-Ala-Gly	C11H21N3O4	259.1532	9834954	-	-	-	-
metab_13370	3.1115	258.1714	neg	L-Hexanoylcarnitine	C13H25NO4	259.1784	3246938	-	-	-	-
metab_4671	5.2573	258.2056	pos	N-Lauroylglycine	C14H27NO3	257.1989	346152	-	-	-	-
metab_5485	2.2209	258.2057	pos	N-Lauroylglycine	C14H27NO3	257.1989	346152	-	-	-	-
metab_6863	1.2663	259.0096	neg	Ifosfamide	NA	260.0232	3690	Oxazaphosphinanes	Isofamides	HMDB0015312	Ifosfamidum

metab_5258	2.8259	259.0595	pos	Spongothymidine	C10H14N2O6	258.0854	65049	-	-	-	-
metab_14163	1.6186	259.0613	neg	8-(1,2-Dihydroxypropan-2-yl)-9-hydroxy-2H,8H,9H-furo[2,3-h]chromen-2-one	C14H14O6	278.0790	131835237	Coumarins and derivatives	Furanocoumarins	HMDB0128947	8-(1,2-dihydroxypropan-2-yl)-9-hydroxy-2H,8H,9H-furo[2,3-h]chromen-2-one
metab_13851	2.1153	259.1190	neg	5-Hexyltetrahydro-2-oxo-3-furancarboxylic acid	C11H18O4	214.1205	10375946	Lactones	Gamma butyrolactones	HMDB0030984	5-Hexyltetrahydro-2-oxo-3-furancarboxylic acid
metab_12928	4.4888	259.1553	neg	3-Oxododecanoic acid	C12H22O3	214.1569	439717	Keto acids and derivatives	Medium-chain keto acids and derivatives	HMDB0010727	3-Oxododecanoic acid
metab_12631	5.7585	259.1916	neg	Tridecanoic acid	C13H26O2	214.1933	12530	Fatty Acyls	Fatty acids and conjugates	HMDB0000910; LMFA01010013	Tridecanoic acid
metab_650	5.6773	259.2048	pos	13-Apo-beta-carotenone	C18H26O	258.1982	5363697	-	-	-	-
metab_984	7.0670	259.2049	pos	13-Apo-beta-carotenone	C18H26O	258.1982	5363697	-	-	-	-
metab_4475	6.3885	259.2049	pos	13-Apo-beta-carotenone	C18H26O	258.1982	5363697	-	-	-	-
metab_628	4.9413	259.2049	pos	13-Apo-beta-carotenone	C18H26O	258.1982	5363697	-	-	-	-
metab_4440	6.5395	259.2050	pos	13-Apo-beta-carotenone	C18H26O	258.1982	5363697	-	-	-	-
metab_4849	4.3818	259.2050	pos	13-Apo-beta-carotenone	C18H26O	258.1982	5363697	-	-	-	-
metab_6360	0.5983	260.0523	pos	3-Amino-3-deoxy-6-O-phosphono-D-glucopyranose	C6H14NO8P	259.0460	443710	-	-	-	-
metab_13965	1.9280	260.1042	neg	Asparaginyl-phenylalanine	C13H17N3O4	279.1219	18218184	Carboxylic acids and derivatives	Amino acids, peptides, and analogues	HMDB0028738	Asparaginyl-Phenylalanine
metab_9459	4.8210	260.1294	neg	Santonine, oxime	C15H19NO3	261.1365	5351540	-	-	-	-
metab_6134	1.0321	260.1485	pos	2-Hydroxydecanedioic acid	C10H18O5	218.1154	128458	Hydroxy acids and derivatives	Medium-chain hydroxy acids and derivatives	HMDB0000424	2-Hydroxydecanedioic acid
metab_320	1.4289	260.1598	pos	Lysopine	C9H18N2O4	218.1267	193187	-	-	-	-
metab_6161	0.9620	260.1961	pos	Lys-Leu	C12H25N3O3	259.1895	7016103	-	-	-	-
metab_1260	0.5420	261.0299	pos	Ifosfamide	NA	260.0232	3690	-	-	-	-

metab_13841	2.1311	261.1344	neg	Etoglucid	C12H22O6	262.1410	16058	-	-	-	-
metab_8836	2.6046	261.1345	neg	Etoglucid	C12H22O6	262.1410	16058	-	-	-	-
metab_13519	2.7792	261.1346	neg	Undecanedioic acid	C11H20O4	216.1362	15816	Fatty Acyls	Fatty acids and conjugates	LMFA01170007; HMDB0000888	Undecanedioic acid
metab_13519	2.7792	261.1346	neg	Etoglucid	C12H22O6	262.1410	16058	Fatty Acyls	Fatty acids and conjugates	LMFA01170007; HMDB0000888	Undecanedioic acid
metab_1677	1.7128	261.1436	pos	gamma-Glu-leu	C11H20N2O5	260.1372	151023	Carboxylic acids and derivatives	Amino acids, peptides, and analogues	HMDB0011171	gamma-Glutamylleucine
metab_4884	4.2457	261.1479	pos	K-GG	C10H20N4O4	260.1484	70679036	-	-	-	-
metab_6464	0.5140	261.1550	pos	N(6)-(Glycylglycyl)-L-lysine	C10H20N4O4	260.1484	70679036	-	-	-	-
metab_974	7.8830	261.2205	pos	5-(1-Oxopropan-2-yl)isolongifol-5-ene	C18H28O	260.2138	24755571	-	-	-	-
metab_684	6.6001	261.2206	pos	5-(1-Oxopropan-2-yl)isolongifol-5-ene	C18H28O	260.2138	24755571	-	-	LMFA05000063	
metab_1005	5.9649	261.2206	pos	5-(1-Oxopropan-2-yl)isolongifol-5-ene	C18H28O	260.2138	24755571	-	-	-	-
metab_13144	3.7488	262.1122	neg	7-Mercaptoheptanoylthreonine	C11H21NO4S	263.1192	24892802	-	-	-	-
metab_1032	1.9463	262.1177	pos	Imazapyr	C13H15N3O3	261.1113	54738	-	-	-	-
metab_1122	0.7661	262.1277	pos	1-[(1-Carboxy2-methylpropyl) amino]-1-deoxyfructose, 9CI	C11H21NO7	279.1318	131752247	Carboxylic acids and derivatives	Amino acids, peptides, and analogues	HMDB0037844	N-(1-Deoxy-1-fructosyl)valine
metab_4686	5.1533	262.2370	pos	2-Amino-1,3,4-tetradecanetriol	C14H31NO3	261.2302	85781307	-	-	-	-
metab_5020	3.6384	262.2370	pos	2-Amino-1,3,4-tetradecanetriol	C14H31NO3	261.2302	85781307	-	-	-	-
metab_1113	0.8640	263.0865	pos	Asp-glu	C9H14N2O7	262.0800	151403	-	-	-	-
metab_372	1.7128	263.1381	pos	Phe-Pro	C14H18N2O3	262.1316	7020642	-	-	-	-
metab_7523	1.9280	263.1403	neg	Phe-Val	C14H20N2O3	264.1473	7359122	-	-	-	-

metab_7523	1.9280	263.1403	neg	Val-phe	C14H20N2O3	264.1473	6993120	-	-	-	-
metab_14083	1.7419	263.1403	neg	Phe-Val	C14H20N2O3	264.1473	7359122	-	-	-	-
metab_14083	1.7419	263.1403	neg	Val-phe	C14H20N2O3	264.1473	6993120	-	-	-	-
metab_7640	2.7155	263.1503	neg	Hexyl glucoside	C12H24O6	264.1573	4462283	Fatty Acyls	Fatty acyl glycosides	HMDB0031688	Hexyl glucoside
metab_734	7.6332	263.2361	pos	4-Dodecylphenol	C18H30O	262.2295	66030	-	-	-	-
metab_767	8.2289	263.2362	pos	4-Dodecylphenol	C18H30O	262.2295	66030	-	-	-	-
metab_14257	1.5297	264.0551	neg	Endalin	C9H12FN3O3	229.0863	355450	Pyrimidine nucleosides	Pyrimidine 2',3'-dideoxyribonucleosides	HMDB0041887	Endalin
metab_6018	1.2162	264.0974	pos	7-Mercaptoheptanoylthreonine	C11H21NO4S	263.1192	24892802	-	-	-	-
metab_13053	4.0516	264.1244	neg	Ritalinic acid	C13H17NO2	219.1259	86863	Organonitrogen compounds	Amines	HMDB0042008	Ritalinic acid
metab_8067	1.0357	265.0931	neg	Dehydrodeoxycoformycin	C11H14N4O4	266.1002	439866	-	-	-	-
metab_2113	3.2727	265.1065	pos	Thiamine	C12H16N4OS	264.1043	1130	Diazines	Estrane steroids	HMDB0000235	Bequin
metab_8130	1.2236	265.1196	neg	gamma-Glutamyltyramine	C13H18N2O4	266.1268	10214674	-	-	-	-
metab_8130	1.2236	265.1196	neg	Phe-Thr	C13H18N2O4	266.1266	10445608	-	-	-	-
metab_14162	1.6186	265.1196	neg	gamma-Glutamyltyramine	C13H18N2O4	266.1268	10214674	-	-	-	-
metab_14162	1.6186	265.1196	neg	Phe-Thr	C13H18N2O4	266.1266	10445608	-	-	-	-
metab_13424	2.9931	265.1200	neg	gamma-Glutamyltyramine	C13H18N2O4	266.1268	10214674	-	-	-	-
metab_13424	2.9931	265.1200	neg	Phe-Thr	C13H18N2O4	266.1266	10445608	-	-	-	-
metab_71	3.3490	265.1428	pos	Hirsutic acid C	C15H20O4	264.1361	442376	-	-	-	-
metab_2286	4.1698	265.1428	pos	Hirsutic acid C	C15H20O4	264.1361	442376	-	-	-	-
metab_5657	1.9309	265.1538	pos	Phe-Val	C14H20N2O3	264.1473	7359122	-	-	-	-
metab_5657	1.9309	265.1538	pos	Val-phe	C14H20N2O3	264.1473	6993120	-	-	-	-
metab_788	8.6132	265.2518	pos	9,12,15-Octadecatrien-1-ol	C18H32O	264.2453	5367327	-	-	LMFA05000216	

metab_14219	1.5439	267.0150	neg	Formononetin	C16H12O4	268.0737	5280378	Isoflavonoids	Flavones	HMDB0005808	7-Hydroxy-3-(4-methoxyphenyl)-4H-chromen-4-one
metab_9244	3.8839	267.0667	neg	Formononetin	C16H12O4	268.0737	5280378	-	-	-	-
metab_6775	0.6131	267.0724	neg	2-O-(alpha-D-Glucopyranosyl)-D-glycerate	C9H16O9	268.0795	53262367	-	-	-	-
metab_6003	1.2446	267.0968	pos	Dinex	C12H14N2O5	266.0902	8540	-	-	-	-
metab_1852	2.2519	267.0969	pos	Dinex	C12H14N2O5	266.0902	8540	-	-	-	-
metab_9686	6.1157	267.1086	neg	Pentostatin	C11H16N4O4	268.1160	439693	-	-	-	-
metab_13669	2.4651	267.1087	neg	Pentostatin	C11H16N4O4	268.1160	439693	-	-	-	-
metab_10892	14.6830	267.1087	neg	Pentostatin	C11H16N4O4	268.1160	439693	-	-	-	-
metab_14730	0.7678	267.1088	neg	Pentostatin	C11H16N4O4	268.1160	439693	-	-	-	-
metab_12920	4.5219	267.1088	neg	Pentostatin	C11H16N4O4	268.1160	439693	-	-	-	-
metab_9466	4.8541	267.1088	neg	Pentostatin	C11H16N4O4	268.1160	439693	-	-	-	-
metab_12678	5.5808	267.1088	neg	Pentostatin	C11H16N4O4	268.1160	439693	-	-	-	-
metab_13063	4.0020	267.1089	neg	Pentostatin	C11H16N4O4	268.1160	439693	-	-	-	-
metab_8035	0.9508	267.1090	neg	Pentostatin	C11H16N4O4	268.1160	439693	-	-	-	-
metab_5803	1.6128	267.1331	pos	Phe-Thr	C13H18N2O4	266.1266	10445608	-	-	-	-
metab_5208	2.9806	267.1331	pos	Phe-Thr	C13H18N2O4	266.1266	10445608	-	-	-	-
metab_39	1.1875	267.1332	pos	Phe-Thr	C13H18N2O4	266.1266	10445608	-	-	-	-
metab_79	4.3371	267.1584	pos	Mukaadial	C15H22O4	266.1515	159088	-	-	-	-
metab_79	4.3371	267.1584	pos	Xanthoxic acid	C15H22O4	266.1516	5282223	-	-	-	-
metab_482	2.8108	267.1584	pos	Mukaadial	C15H22O4	266.1515	159088	-	-	-	-
metab_482	2.8108	267.1584	pos	Xanthoxic acid	C15H22O4	266.1516	5282223	-	-	-	-
metab_12727	5.3228	267.1604	neg	Dihydroartemisinic acid hydroperoxide	C15H24O4	268.1673	91820389	-	-	-	-

metab_9471	4.8865	267.1604	neg	Dihydroartemisinic acid hydroperoxide	C15H24O4	268.1673	91820389	-	-	-	-
metab_12677	5.5966	267.1604	neg	Dihydroartemisinic acid hydroperoxide	C15H24O4	268.1673	91820389	Fatty Acyls	Fatty alcohols	HMDB0240213	Prostaglandin lactone-diol
metab_12677	5.5966	267.1604	neg	(3Ar,4R,5R,6aS)-5-Hydroxy-4-((S,E)-3-hydroxyoct-1-en-1-yl)hexahydro-2H-cyclopenta[b]furan-2-one	C15H24O4	268.1675	12895812	Fatty Acyls	Fatty alcohols	HMDB0240213	Prostaglandin lactone-diol
metab_12528	6.2294	267.1605	neg	Dihydroartemisinic acid hydroperoxide	C15H24O4	268.1673	91820389	Prenol lipids	Sesquiterpenoids	HMDB0037605	(10betaH,11xi)-11-Hydroxy-13-nor-6-eremophilen-8-one
metab_12528	6.2294	267.1605	neg	(10Betah,11xi)-11-Hydroxy-13-nor-6-eremophilen-8-one	C14H22O2	222.1620	85186102	Prenol lipids	Sesquiterpenoids	HMDB0037605	(10betaH,11xi)-11-Hydroxy-13-nor-6-eremophilen-8-one
metab_7690	3.1626	267.1605	neg	Dihydroartemisinic acid hydroperoxide	C15H24O4	268.1673	91820389	-	-	-	-
metab_9153	3.5813	267.1605	neg	Dihydroartemisinic acid hydroperoxide	C15H24O4	268.1673	91820389	-	-	-	-
metab_13085	3.9177	267.1605	neg	Dihydroartemisinic acid hydroperoxide	C15H24O4	268.1673	91820389	-	-	-	-
metab_2456	5.3311	267.1946	pos	Juvenile hormone III	C16H26O3	266.1880	5281523	-	-	-	-
metab_2420	5.0451	267.1947	pos	Juvenile hormone III	C16H26O3	266.1880	5281523	-	-	-	-
metab_577	4.0939	267.1947	pos	Juvenile hormone III	C16H26O3	266.1880	5281523	-	-	-	-
metab_605	4.6851	267.1947	pos	Juvenile hormone III	C16H26O3	266.1880	5281523	-	-	-	-
metab_557	3.7447	267.1948	pos	Juvenile hormone III	C16H26O3	266.188	5281523	-	-	-	-
metab_14341	1.4568	268.0830	neg	3-Hydroxyphenylpropionylglycine	C11H13NO4	223.0845	131802906	Carboxylic acids and derivatives	Amino acids, peptides, and analogues	HMDB0094724	3-hydroxyphenylpropionylglycine
metab_9173	3.6479	269.0460	neg	Genistein	C15H10O5	270.0527	5280961	Isoflavonoids	Hydroxysteroids	HMDB0003217	Genestein

metab_9173	3.6479	269.0460	neg	3,7,4'-Trihydroxyflavone	C15H10O5	270.0527	5281611	Flavonoids	Flavones	HMDB0034004	4',7-Dihydroxyflavonol
metab_9173	3.6479	269.0460	neg	3,7,4'-Trihydroxyflavone	C15H10O5	270.0527	5281611	-	-	-	-
metab_249	0.8920	269.1599	pos	Histidylleucine	C12H20N4O3	268.1534	189008	-	-	-	-
metab_4565	5.8753	269.1739	pos	Dihydroartemisinic acid hydroperoxide	C15H24O4	268.1672	91820389	-	-	-	-
metab_4754	4.8809	269.1740	pos	Dihydroartemisinic acid hydroperoxide	C15H24O4	268.1672	91820389	-	-	-	-
metab_4823	4.5184	269.1740	pos	Dihydroartemisinic acid hydroperoxide	C15H24O4	268.1672	91820389	-	-	-	-
metab_17	2.4530	269.1740	pos	Dihydroartemisinic acid hydroperoxide	C15H24O4	268.1672	91820389	-	-	-	-
metab_4789	4.6398	269.2104	pos	dihydroartemisinic acid hydroperoxide	C15H24O4	268.1672	11043771	-	-	-	-
metab_9771	6.5989	269.2125	neg	16-Oxohexadecanoic acid	C16H30O3	270.2195	15931626	-	-	-	-
metab_12444	6.5512	270.2076	neg	N-Tridecanoylglycine	C15H29NO3	271.2145	45357453	-	-	-	-
metab_9576	5.5322	270.2078	neg	N-Tridecanoylglycine	C15H29NO3	271.2145	45357453	-	-	-	-
metab_3941	8.8466	270.2784	pos	N-(13-Methyltetradecyl)acetamide	C17H35NO	269.2717	47346	-	-	-	-
metab_5825	1.5705	271.1069	pos	Valyl-Methionine	C10H20N2O3S	248.1195	6993039	Carboxylic acids and derivatives	Amino acids, peptides, and analogues	HMDB0029133	Valyl-Methionine
metab_13153	3.7317	271.1554	neg	4,5-Dihydrovmifoliol	C13H22O3	226.1569	23136584	Prenol lipids	Sesquiterpenoids	HMDB0040615	4,5-Dihydrovomifoliol
metab_8949	2.8922	271.1667	neg	Caprolactam	C6H11NO	113.0841	7768	Lactams	Caprolactams	HMDB0062769	Epsilon-caprolactam
metab_9718	6.3269	271.1916	neg	5-Tetradecenoic acid	C14H26O2	226.1933	5282740	Fatty Acyls	Fatty acids and conjugates	HMDB0000499	5-Tetradecenoic acid
metab_11836	8.5135	271.2282	neg	16-Hydroxyhexadecanoic acid	C16H32O3	272.2351	10466	Fatty Acyls	Fatty acids and conjugates	HMDB0031057	2-Hydroxyhexadecanoic acid

metab_11836	8.5135	271.2282	neg	2-Hydroxyhexadecanoic acid	C16H32O3	272.2353	92836	Fatty Acyls	Fatty acids and conjugates	HMDB0031057	2-Hydroxyhexadecanoic acid
metab_9339	4.2702	272.0932	neg	L-Thyronine	C15H15NO4	273.1001	5461103	Carboxylic acids and derivatives	Amino acids, peptides, and analogues	HMDB0000667	L-Thyronine
metab_5581	2.0677	273.1226	pos	(2S,4R)-4-(9H-Pyrido[3,4-b]indol-1-yl)-1,2,4-butanetriol	C15H16N2O3	272.1161	15394713	Harmala alkaloids	-	HMDB0035191	(2S,4R)-4-(9H-Pyrido[3,4-b]indol-1-yl)-1,2,4-butanetriol
metab_9437	4.7217	273.1711	neg	11-Hydroxy-9-tridecenoic acid	C13H24O3	228.1725	5318367	Fatty Acyls	Fatty acids and conjugates	LMFA01050437; HMDB0035881	11-Hydroxy-9-tridecenoic acid
metab_6093	1.1164	274.1751	pos	(2S)-6-[[(2R,3R)-3-Methyl-2-amino-5-oxopentanoyl]amino]-2-aminohexanoic acid	C12H23N3O4	273.1690	71448982	-	-	-	-
metab_343	1.5284	274.2004	pos	(2R,3R)-3-Methylglutamyl-5-semialdehyde-N6-lysine	C12H23N3O4	273.169	71448982	-	-	-	-
metab_4633	5.4357	274.2732	pos	Hexadecasphinganine	C16H35NO2	273.2666	656816	-	-	-	-
metab_4604	5.6473	274.2732	pos	Hexadecasphinganine	C16H35NO2	273.2666	656816	-	-	-	-
metab_2557	6.0561	274.2733	pos	Hexadecasphinganine	C16H35NO2	273.2666	656816	-	-	LMSP01040001	
metab_5422	2.3908	275.0908	pos	3,4,4',7-Tetrahydroxyflavan	C15H14O5	274.0841	13886894	Flavonoids	Flavans	HMDB0040828	(2S,3S,4R)-3,4,4',7-Tetrahydroxyflavan
metab_14891	0.5571	275.1251	neg	L-Saccharopine	C11H20N2O6	276.1321	160556	Carboxylic acids and derivatives	Amino acids, peptides, and analogues	HMDB0000279	Saccharopine
metab_5420	2.3908	275.1383	pos	4-((6-Methoxyquinolin-8-yl)amino)pentanoic acid	C15H18N2O3	274.1317	127542	-	-	-	-
metab_13408	3.0260	275.1504	neg	p-Coumaroylagmatine	C14H20N4O2	276.1575	5280691	Organooxygen compounds	Ethers	HMDB0030143	Talaromycin A
metab_13539	2.7318	275.1504	neg	p-Coumaroylagmatine	C14H20N4O2	276.1575	5280691	-	-	-	-
metab_9537	5.2741	275.1867	neg	3-[[5-Methyl-2-(1-methylethyl	C13H26O3	230.1882	5362595	Prenol lipids	Monoterpenoids	HMDB0036133	3-[[5-Methyl-2-(1-methylethyl)cycl

				)cyclohexyl]oxy]propane-1,2-diol							ohexyl]oxy]-1,2-propanediol
metab_2621	6.7366	275.1997	pos	17-Hydroxy-estr-5(10)-en-3-one	C18H26O2	274.1930	235672	-	-	-	-
metab_1326	0.6402	276.0472	pos	2-Phthalimidoglutaric acid	C13H11NO6	275.0408	92225	-	-	-	-
metab_1326	0.6402	276.0472	pos	D-Glucosaminic acid 6-phosphate	C6H14NO9P	275.0408	71728452	-	-	-	-
metab_322	1.4429	276.0684	pos	D-Glucosaminate-6-phosphate	C6H14NO9P	275.0408	71728452	-	-	-	-
metab_9506	5.1131	276.1242	neg	1-(m-Methoxycinnamoyl)pyrrolidine	C14H17NO2	231.1259	5373710	-	-	HMDB0038839	1-(m-Methoxycinnamoyl)pyrrolidine
metab_5685	1.8432	276.1335	pos	L-Alanyl-L-tryptophan	C14H17N3O3	275.1270	85362	-	-	-	-
metab_5685	1.8432	276.1335	pos	Trp-Ala	C14H17N3O3	275.1270	6993401	-	-	-	-
metab_6	0.9480	276.1433	pos	O-Glutaroyl-L-carnitine	C12H21NO6	275.1369	71317118	-	-	-	-
metab_2182	3.6073	276.1700	pos	Phenylalanyllysine	C15H23N3O3	293.1739	15607819	Carboxylic acids and derivatives	Amino acids, peptides, and analogues	HMDB0029000	Phenylalanyl-Lysine
metab_8886	2.7475	277.0720	neg	3-(1,2-Dihydroxybut-3-en-1-yl)-1H-isochromen-1-one	C13H12O4	232.0736	131836270	Isocoumarins and derivatives	-	HMDB0130065	3-(1,2-dihydroxybut-3-en-1-yl)-1H-isochromen-1-one
metab_13517	2.7792	277.1084	neg	2beta,9xi-Dihydroxy-8-oxo-1(10),4,11(13)-germacratrien-12,6alpha-olide	C15H18O5	278.1154	131752027	Prenol lipids	Terpene lactones	HMDB0036662	2beta,9xi-Dihydroxy-8-oxo-1(10),4,11(13)-germacratrien-12,6alpha-olide
metab_14349	1.4427	277.1294	neg	N-Benzoyl-D-arginine	C13H18N4O3	278.1366	656500	-	-	-	-
metab_14349	1.4427	277.1294	neg	Pentoxifylline	C13H18N4O3	278.1366	4740	-	-	-	-
metab_14180	1.5885	277.1303	neg	N-Benzoyl-D-arginine	C13H18N4O3	278.1366	656500	-	-	-	-
metab_14180	1.5885	277.1303	neg	Pentoxifylline	C13H18N4O3	278.1366	4740	-	-	-	-
metab_12217	7.3180	277.1445	neg	Bis(2-ethylhexyl) phthalate	C16H22O4	278.1519	8343	-	-	-	-
metab_9785	6.6632	277.1448	neg	Bis(2-ethylhexyl) phthalate	C16H22O4	278.1519	8343	-	-	-	-

metab_13653	2.5113	277.1560	neg	Phe-Ile	C15H22N2O3	278.1630	7010566	-	-	-	-
metab_13653	2.5113	277.1560	neg	Ile-Phe	C15H22N2O3	278.1630	7009596	-	-	-	-
metab_13744	2.3227	277.1560	neg	Phe-Ile	C15H22N2O3	278.1630	7010566	-	-	-	-
metab_13744	2.3227	277.1560	neg	Ile-Phe	C15H22N2O3	278.1630	7009596	-	-	-	-
metab_4729	4.9413	277.2154	pos	Nandrolone	C18H26O2	276.2088	9904	-	-	LMFA01030537	
metab_4729	4.9413	277.2154	pos	Stearidonic acid	C18H28O2	276.2086	5312508	-	-	LMFA01030537	
metab_4729	4.9413	277.2154	pos	9(S)-HOTrE	C18H30O3	276.2087	6439873	-	-	LMFA01030537	
metab_4333	7.0670	277.2154	pos	Nandrolone	C18H26O2	276.2088	9904	-	-	LMFA01030540	
metab_4333	7.0670	277.2154	pos	Stearidonic acid	C18H28O2	276.2086	5312508	-	-	LMFA01030540	
metab_4333	7.0670	277.2154	pos	9(S)-HOTrE	C18H30O3	276.2087	6439873	-	-	LMFA01030540	
metab_12479	6.4073	277.2170	neg	gamma-Linolenic acid	C18H30O2	278.2244	5280933	-	-	-	-
metab_14222	1.5439	278.1149	neg	L-Asparaginyl-L-phenylalanine	C13H17N3O4	279.1220	11471482	-	-	-	-
metab_14222	1.5439	278.1149	neg	Phe-Asn	C13H17N3O4	279.1220	10468817	-	-	-	-
metab_12885	4.6380	278.1400	neg	Thalictroidine	C14H19NO2	233.1416	10489834	Organooxygen compounds	Carbonyl compounds	HMDB0034684	Thalictroidine
metab_993	6.3885	278.2471	pos	Obscuraminol A	C18H31NO	277.2404	10062076	-	-	-	-
metab_2798	7.8683	278.2471	pos	Obscuraminol A	C18H31NO	277.2404	10062076	-	-	LMSP01080036	
metab_13455	2.9092	279.0987	neg	L-Aspartyl-L-phenylalanine	C13H16N2O5	280.1059	93078	-	-	-	-
metab_13455	2.9092	279.0987	neg	H-PHE-ASP-OH	C13H16N2O5	280.1059	6992643	-	-	-	-
metab_8401	1.6335	279.0990	neg	L-Aspartyl-L-phenylalanine	C13H16N2O5	280.1059	93078	-	-	-	-
metab_8401	1.6335	279.0990	neg	Phe-Asp	C13H16N2O5	280.1059	6992643	-	-	-	-
metab_13949	1.9596	279.1240	neg	(+)-8'-Hydroxyabscisic acid	C15H20O5	280.1312	11954194	-	-	-	-
metab_8725	2.3227	279.1241	neg	(+)-8'-Hydroxyabscisic acid	C15H20O5	280.1312	11954194	Cinnamic acids and derivatives	Cinnamic acids	HMDB0133171	(2Z)-3-hydroxy-2-(phenylmethylidene)heptanoic acid

metab_8858	2.6678	279.1241	neg	(+)-8'-Hydroxyabscisic acid	C15H20O5	280.1312	11954194	Cinnamic acids and derivatives	Cinnamic acids	HMDB0133174	(2Z)-6-hydroxy-2-(phenylmethylidene)heptanoic acid
metab_8704	2.2754	279.1350	neg	Tyrosyl-Valine	C14H20N2O4	280.1423	7009560	-	-	-	-
metab_9140	3.5475	279.1352	neg	Tyrosyl-Valine	C14H20N2O4	280.1423	7009560	-	-	-	-
metab_14407	1.3517	279.1353	neg	Tyrosyl-Valine	C14H20N2O4	280.1423	7009560	-	-	-	-
metab_530	2.3292	279.1696	pos	Ile-Phe	C15H22N2O3	278.1632	7009596	-	-	-	-
metab_5361	2.5158	279.1696	pos	Ile-Phe	C15H22N2O3	278.1632	7009596	-	-	-	-
metab_706	7.0228	279.2310	pos	12,13-DiHOME	C18H34O4	314.2457	10236635	Fatty Acyls	Fatty acids and conjugates	HMDB0004705	12,13-DHOME
metab_706	7.0228	279.2310	pos	Linolenic acid	C18H30O2	278.2244	5280934	Fatty Acyls	Fatty acids and conjugates	HMDB0004705	12,13-DHOME
metab_706	7.0228	279.2310	pos	gamma-Linolenic acid	C18H30O2	278.2244	5280933	Fatty Acyls	Fatty acids and conjugates	HMDB0004705	12,13-DHOME
metab_634	5.2431	279.2311	pos	Linolenic acid	C18H30O2	278.2244	5280934	-	-	-	-
metab_634	5.2431	279.2311	pos	gamma-Linolenic acid	C18H30O2	278.2244	5280933	-	-	-	-
metab_992	6.4035	279.2311	pos	Linolenic acid	C18H30O2	278.2244	5280934	-	-	-	-
metab_992	6.4035	279.2311	pos	gamma-Linolenic acid	C18H30O2	278.2244	5280933	-	-	-	-
metab_106	9.6968	279.2311	pos	Linolenic acid	C18H30O2	278.2244	5280934	-	-	-	-
metab_106	9.6968	279.2311	pos	gamma-Linolenic acid	C18H30O2	278.2244	5280933	-	-	-	-
metab_2946	8.7303	279.2311	pos	Linolenic acid	C18H30O2	278.2244	5280934	-	-	-	-
metab_2946	8.7303	279.2311	pos	gamma-Linolenic acid	C18H30O2	278.2244	5280933	-	-	-	-
metab_12013	7.9908	279.2332	neg	Linoleic acid	C18H32O2	280.2403	5280450	Fatty Acyls	Lineolic acids and derivatives	LMFA01030120; HMDB0006270; HMDB0000673	Linoelaidic acid;Linoleic acid
metab_7316	8.7031	279.2332	neg	Linoleic acid	C18H32O2	280.2403	5280450	-	-	-	-

metab_14833	0.5991	280.1039	neg	N-(1-Deoxy-1-fructosyl)threonine	C10H19NO8	281.1111	131752246	Organooxygen compounds	Carbohydrates and carbohydrate conjugates	HMDB0037843	N-(1-Deoxy-1-fructosyl)threonine
metab_4101	8.2143	280.2627	pos	Linoleamide	C18H33NO	279.2560	6435901	Fatty Acyls	Fatty amides	HMDB0062656; LMFA08010008	Linoleamide
metab_7973	0.7818	281.0880	neg	1-Methylinosine	C11H14N4O5	282.0964	65095	Purine nucleosides	-	HMDB0002721	1-Methylinosine
metab_1065	1.4714	281.0984	pos	H-Met-met-OH	C10H20N2O3S2	280.0920	6993082	-	-	-	-
metab_4680	5.1830	281.1011	pos	H-Met-met-OH	C10H20N2O3S2	280.0920	6993082	-	-	-	-
metab_5787	1.6413	281.1122	pos	Phe-Asp	C13H16N2O5	280.1059	6992643	-	-	-	-
metab_6024	1.2162	281.1123	pos	Phe-Asp	C13H16N2O5	280.1059	6992643	-	-	-	-
metab_4578	5.7846	281.1375	pos	Hymenoflorin	C15H20O5	280.1308	442262	-	-	-	-
metab_13302	3.2972	281.1398	neg	Hymenoxon	C15H22O5	282.1468	42295	Prenol lipids	Sesquiterpenoids	HMDB0040778	Eremopetasidione
metab_9243	3.8839	281.1398	neg	Hymenoxon	C15H22O5	282.1468	42295	Prenol lipids	Terpene lactones	HMDB0034983	Cynaratriol
metab_9243	3.8839	281.1398	neg	(3R,3Ar,6aR,8S,9S,9aR,9bR)-3,8-dihydroxy-3-(hydroxymethyl)-9-methyl-6-methylidene-4,5,6a,7,8,9,9a,9b-octahydro-3aH-azuleno[4,5-b]furan-2-one	C15H22O5	282.1467	12149911	Prenol lipids	Terpene lactones	HMDB0034983	Cynaratriol
metab_1653	1.6413	281.1487	pos	Tyrosyl-Valine	C14H20N2O4	280.1423	7009560	-	-	-	-
metab_5973	1.3153	281.1487	pos	Tyrosyl-Valine	C14H20N2O4	280.1423	7009560	-	-	-	-
metab_2168	3.5465	281.1487	pos	Tyrosyl-Valine	C14H20N2O4	280.1423	7009560	-	-	-	-
metab_2945	8.7161	281.1528	pos	Tyr Val	C14H20N2O4	280.1423	7009560	-	-	-	-
metab_659	5.9049	281.1740	pos	Chissonox 201	C16H24O4	280.1672	61094	-	-	-	-
metab_5906	1.4289	281.1853	pos	3,4-Epoxy-6-methylcyclohexylmethyl-3,4-epoxy-6-methylcy	C16H24O4	280.1672	61094	-	-	-	-

				clo-hexanecarboxylate							
metab_3657	10.1175	281.2466	pos	13-(Cyclopent-2-enyl)tridecanoic acid	C18H32O2	280.2397	72853	-	-	-	-
metab_26	5.9949	281.2466	pos	13-(Cyclopent-2-enyl)tridecanoic acid	C18H32O2	280.2397	72853	-	-	-	-
metab_4160	7.9835	281.2467	pos	13-(Cyclopent-2-enyl)tridecanoic acid	C18H32O2	280.2397	72853	-	-	-	-
metab_11849	8.4667	281.2487	neg	trans-Vaccenic acid	C18H34O2	282.2560	5281127	Fatty Acyls	Fatty acids and conjugates	HMDB0010737	(R)-3-Hydroxy-Octadecanoic acid
metab_11849	8.4667	281.2487	neg	(R)-3-Hydroxy-Octadecanoic acid	C18H36O3	300.2664	5312838	Fatty Acyls	Fatty acids and conjugates	HMDB0010737	(R)-3-Hydroxy-Octadecanoic acid
metab_10419	9.1200	281.2488	neg	trans-Vaccenic acid	C18H34O2	282.2560	5281127	-	-	-	-
metab_6343	0.6123	282.0480	pos	Kathon 930	C11H17Cl2NOS	281.0413	91688	-	-	-	-
metab_14639	0.9368	282.0736	neg	Guanosine	C10H13N5O5	283.0845	135398635	-	-	-	-
metab_18	2.4071	282.0965	pos	N-4-Hydroxyphenylacetylglutamic acid	C13H15NO6	281.0899	440731	-	-	-	-
metab_208	0.6262	282.1178	pos	1-Methyladenosine	C11H15N5O4	281.1124	27476	-	-	-	-
metab_208	0.6262	282.1178	pos	2'-O-Methyladenosine	C11H15N5O4	281.1124	102213	-	-	-	-
metab_1436	1.0180	282.1187	pos	1-Methyladenosine	C11H15N5O4	281.1124	27476	Purine nucleosides	-	HMDB0004326	2'-O-Methyladenosine
metab_1436	1.0180	282.1187	pos	2'-O-Methyladenosine	C11H15N5O4	281.1124	102213	Purine nucleosides	-	HMDB0004326	2'-O-Methyladenosine
metab_3051	9.4049	282.2664	pos	Oleamide	C18H35NO	281.2716	5283387	Fatty Acyls		HMDB0002117	Armid O
metab_789	8.6132	282.2783	pos	Oleamide	C18H35NO	281.2716	5283387	Fatty Acyls	Fatty amides	HMDB0002117; LMFA08010004	Oleamide
metab_14044	1.7885	283.0581	neg	Biochanin A	C16H12O5	284.0681	5280373	Isoflavonoids	Methoxyphenols	HMDB0002338	Biochanin A
metab_14044	1.7885	283.0581	neg	Glycitein	C16H12O5	284.0682	5317750	Isoflavonoids	Amino acids, peptides,	HMDB0005781	7-Hydroxy-3-(4-hydroxyphenyl)-6-

									and analogues		methoxy-4H-1-benzopyran-4-one
metab_14044	1.7885	283.0581	neg	Acacetin	$C_{16}H_{12}O_5$	284.0686	5280442	-	-	-	-
metab_14044	1.7885	283.0581	neg	Biochanin A	$C_{16}H_{12}O_5$	284.0681	5280373	Isoflavonoids	Methoxyphenols	HMDB0002338	Biochanin A
metab_14044	1.7885	283.0581	neg	Acacetin	$C_{16}H_{12}O_5$	284.0686	5280442	-	-	-	-
metab_14044	1.7885	283.0581	neg	Glycitein	$C_{16}H_{12}O_5$	284.0682	5317750	Isoflavonoids	Amino acids, peptides, and analogues	HMDB0005781	7-Hydroxy-3-(4-hydroxyphenyl)-6-methoxy-4H-1-benzopyran-4-one
metab_6808	0.9227	283.0687	neg	Xanthosine	$C_{10}H_{12}N_4O_6$	284.0757	64959	Purine nucleosides	-	HMDB0000299	Xanthosine
metab_6852	0.5991	283.1036	neg	Coformycin	$C_{11}H_{16}N_4O_5$	284.1108	25447	-	-	-	-
metab_505	3.0422	283.1531	pos	Artemisinin	$C_{15}H_{22}O_5$	282.1461	68827	-	-	-	-
metab_1837	2.2209	283.1532	pos	Artemisinin	$C_{15}H_{22}O_5$	282.1461	68827	-	-	-	-
metab_6972	4.3380	283.1554	neg	alpha-Dihydroartemisinin	$C_{15}H_{24}O_5$	284.1624	11358077	Prenol lipids	Sesquiterpenoids	HMDB0060593	Alpha-dihydroartemisinin
metab_139	5.5260	283.1895	pos	Hymenoxon	$C_{15}H_{22}O_5$	282.1468	42295	-	-	-	-
metab_12507	6.3269	283.1916	neg	(3S,4S,6R,7S)-1,10-Bisaboladiene-3,4-diol	$C_{15}H_{26}O_2$	238.1933	14633005	Prenol lipids	Sesquiterpenoids	HMDB0031383	(3S,4S,6R,7S)-1,10-Bisaboladiene-3,4-diol
metab_9593	5.6128	283.1918	neg	Tricyclohumuladiol	$C_{15}H_{26}O_2$	238.1933	15647027	Prenol lipids	Sesquiterpenoids	HMDB0036730	Tricyclohumuladiol
metab_11621	8.9903	283.2643	neg	9,10,12,13-Tetradeuteriooctadeca-9,12-dienoic acid	$C_{18}H_{28}D_4O_2$	284.2717	53394405	-	-	-	-
metab_1810	2.1290	284.1271	pos	Ala-Gly-His	$C_{11}H_{17}N_5O_4$	283.1266	71464564	-	-	-	-
metab_5854	1.5284	284.1484	pos	Hydroxyisovaleroyl carnitine	$C_{12}H_{23}NO_5$	261.1576	57357187	Fatty Acyls	Fatty acid esters	LMFA07070073; HMDB0062555	hydroxyisovaleroyl carnitine
metab_3885	9.1140	284.2939	pos	Sphinganine	$C_{18}H_{39}NO_2$	301.2981	91486	Organonitrogen compounds	Amines	LMSP01020001; HMDB0000269	Sphinganine
metab_4119	8.1414	284.3303	pos	Cetrimonium	$C_{19}H_{41}N$	283.3236	2681	-	-	-	-
metab_8865	2.6841	285.0885	neg	2-(2-(1H-Indol-3-yl)acetamido)pentanedioic acid	$C_{15}H_{16}N_2O_5$	304.1059	25200809	Carboxylic acids and derivatives	Amino acids, peptides, and analogues	HMDB0038665	L-N-(1H-Indol-3-ylacetyl)glutamic acid

metab_1420	0.9620	285.1074	pos	Calycosin	C16H12O5	284.068473	5280448	-	-	-	-
metab_6222	0.8360	285.1437	pos	Biochanin A	C16H12O5	284.0681	5280373	Isoflavonoids	Methoxyphenols	HMDB0002338	Biochanin A
metab_6222	0.8360	285.1437	pos	Glycitein	C16H12O5	284.0682	5317750	Isoflavonoids	Amino acids, peptides, and analogues	HMDB0005781	7-Hydroxy-3-(4-hydroxyphenyl)-6-methoxy-4H-1-benzopyran-4-one
metab_6222	0.8360	285.1437	pos	Acacetin	C16H12O5	284.0686	5280442	-	-	-	-
metab_8600	2.0202	285.1457	neg	Abacavir	C14H18N6O	286.1527	441300	-	-	-	-
metab_8474	1.7572	285.1458	neg	Abacavir	C14H18N6O	286.1527	441300	-	-	-	-
metab_1831	2.1896	285.1688	pos	Dihydroartemisinin	C15H24O5	284.1621	3000518	-	-	-	-
metab_2011	2.8877	285.1688	pos	Merulin D	C15H24O5	284.1621	70698134	-	-	-	-
metab_2011	2.8877	285.1688	pos	Artenimol	C15H24O5	284.1622	6918483	-	-	-	-
metab_1831	2.1897	285.1688	pos	Artenimol	C15H24O5	284.1622	6918483	-	-	-	-
metab_1831	2.1897	285.1688	pos	Merulin D	C15H24O5	284.1621	70698134	-	-	-	-
metab_9450	4.7709	285.2074	neg	Hexadecanedioic acid	C16H30O4	286.2146	10459	-	-	-	-
metab_12628	5.7751	285.2075	neg	Hexadecanedioic acid	C16H30O4	286.2146	10459	-	-	-	-
metab_11669	8.8286	285.2439	neg	2-Methoxyhexadecanoic acid	C17H34O3	286.2509	656825	Fatty Acyls	Fatty acids and conjugates	HMDB0061663	12-hydroxyheptadecanoic acid
metab_11669	8.8286	285.2439	neg	12-Hydroxyheptadecanoic acid	C17H34O3	286.2508	15110021	Fatty Acyls	Fatty acids and conjugates	HMDB0061663	12-hydroxyheptadecanoic acid
metab_11809	8.5768	285.2439	neg	2-Methoxyhexadecanoic acid	C17H34O3	286.2509	656825	-	-	-	-
metab_8077	1.0641	286.1889	neg	Arg Leu	C12H25N5O3	287.1956	6992563	-	-	-	-
metab_1163	0.5140	287.0567	pos	Kaempferol	C15H10O6	286.047738	5280863	Flavonoids	Isoflav-2-enes	HMDB0005801	Populnetin
metab_13070	3.9850	287.1406	neg	Hydroxypelenolide	C15H24O3	252.1725	131752028	Prenol lipids	Terpene lactones	HMDB0036663	Hydroxypelenolide
metab_13386	3.0774	287.1502	neg	Asc-DeltaC8	C14H24O6	288.1575	86289665	-	-	HMDB0038736	(3S,5R,6R,7E)-3,5,6-Trihydroxy-7-megastigmen-9-one

metab_8894	2.7624	287.1504	neg	Asc-DeltaC8	C14H24O6	288.1575	86289665	-	-	-	-
metab_8613	2.0516	287.1505	neg	Asc-DeltaC8	C14H24O6	288.1575	86289665	-	-	-	-
metab_6047	1.1875	287.1593	pos	Abacavir	C14H18N6O	286.1527	441300	-	-	-	-
metab_6989	5.4844	287.1867	neg	6-Hydroxypentadecanedioic acid	C15H28O5	288.1937	131751198	Fatty Acyls	Fatty acids and conjugates	HMDB0031885	6-Hydroxypentadecanedioic acid
metab_12619	5.8074	287.2231	neg	7(14)-Farnesene-9,12-diol	C15H30O2	242.2246	131753009	Prenol lipids	Sesquiterpenoids	HMDB0040998	7(14)-Farnesene-9,12-diol
metab_12619	5.8074	287.2231	neg	10,16-Dihydroxyhexadecanoic acid	C16H32O4	288.2302	441449	Prenol lipids	Sesquiterpenoids	HMDB0040998	7(14)-Farnesene-9,12-diol
metab_9597	5.6292	287.2231	neg	10,16-Dihydroxyhexadecanoic acid	C16H32O4	288.2302	441449	-	-	-	-
metab_12579	5.9866	287.2231	neg	10,16-Dihydroxyhexadecanoic acid	C16H32O4	288.2302	441449	-	-	-	-
metab_9550	5.3546	287.2231	neg	10,16-Dihydroxyhexadecanoic acid	C16H32O4	288.2302	441449	Fatty Acyls	Fatty acids and conjugates	HMDB0037798	(S)-10,16-Dihydroxyhexadecanoic acid
metab_12513	6.2943	287.2232	neg	10,16-Dihydroxyhexadecanoic acid	C16H32O4	288.2302	441449	-	-	-	-
metab_8217	1.3373	288.1568	neg	Leu-Ala-Ser	C12H23N3O5	289.1638	23650514	-	-	-	-
metab_5809	1.5987	288.1907	pos	Glycyl-valyl-leucine	C13H25N3O4	287.1845	9857120	-	-	-	-
metab_5809	1.5987	288.1907	pos	Leu-Val-Gly	C13H25N3O4	287.1844	71464648	-	-	-	-
metab_5575	2.0677	288.1910	pos	Glycyl-valyl-leucine	C13H25N3O4	287.1845	9857120	-	-	-	-
metab_5575	2.0677	288.1910	pos	Leu-Val-Gly	C13H25N3O4	287.1844	71464648	-	-	-	-
metab_6122	1.0601	288.2021	pos	Arginyl-Leucine	C12H25N5O3	287.1956	6992563	-	-	-	-
metab_2272	4.0634	288.2161	pos	Octanoylcarnitine	C15H29NO4	287.2097	123701	-	-	-	-
metab_82	5.3454	288.2525	pos	Prosopinine	C16H33NO3	287.2458	42608371	-	-	-	-
metab_13459	2.9092	289.1661	neg	8-(3'R,5'R-Dihydroxy-6'S-met	C14H26O6	290.1732	86289774	-	-	-	-

				hyl-(2H)-tetrahydropyran-2'-yloxy)-octanoic acid						
metab_5961	1.3293	289.1749	pos	Estriol	C18H24O3	288.1723	5756	Steroids and steroid derivatives	Hydroxysteroids	HMDB0000153	(16alpha,17beta)-Estra-1,3,5(10)-triene-3,16,17-triol
metab_4958	3.8967	289.1791	pos	Estriol	C18H24O3	288.1723	5756	-	-	-	-
metab_4958	3.8967	289.1791	pos	3-Methoxyestra-2,5(10)-dien-17beta-ol	C19H28O2	288.2087	101963	-	-	-	-
metab_9207	3.7658	289.2025	neg	4-Androstenediol	C19H30O2	290.2243	136297	Steroids and steroid derivatives	O-methylated isoflavonoids	HMDB0005849	Androst-4-ene-3beta,17beta-diol
metab_14728	0.7818	290.0884	neg	N-Acetyl-Neuraminic Acid	C11H19NO9	309.1060	439197	Organooxygen compounds	Carbohydrates and carbohydrate conjugates	HMDB0000230	N-Acetylneuraminic acid
metab_5049	3.5311	290.2107	pos	Hydroxy-gamma-Sanshool	C18H27NO2	289.2042	14135317	-	-	-	-
metab_1235	0.5140	290.2430	pos	Leu-Ala-Ser	C12H23N3O5	289.1637	23650514	-	-	-	-
metab_487	2.8259	291.0856	pos	Plumericin	C15H14O6	290.0787	5281545	-	-	-	-
metab_5430	2.3751	291.1332	pos	Piperalol	C15H24O3	252.1725	101317814	Organooxygen compounds	Alcohols and polyols	HMDB0035767	Piperalol
metab_5695	1.7993	291.1694	pos	4-Androstenediol	C19H30O2	290.2243	136297	Steroids and steroid derivatives	O-methylated isoflavonoids	HMDB0005849	Androst-4-ene-3beta,17beta-diol
metab_9041	3.1797	291.1817	neg	B-Octylglucoside	C14H28O6	292.1887	62852	Glycerolipids	Monoradylglycerols	HMDB0072866	MG(10:0/0:0/0:0)
metab_4421	6.6606	291.1946	pos	8-(4-Oxo-5-pent-2-enylcyclopent-2-en-1-yl)octanoic acid	C18H28O3	290.1879	188366	-	-	-	-
metab_4421	6.6606	291.1946	pos	ML-236C	C18H26O3	290.1878	173650	-	-	-	-
metab_4993	3.7447	291.1946	pos	8-(4-Oxo-5-pent-2-enylcyclopent-2-en-1-yl)octanoic acid	C18H28O3	290.1879	188366	-	-	-	-
metab_4993	3.7447	291.1946	pos	ML-236C	C18H26O3	290.1878	173650	-	-	-	-
metab_4601	5.6623	291.1946	pos	8-(4-Oxo-5-pent-2-enylcyclop	C18H28O3	290.1879	188366	-	-	-	-

				ent-2-en-1-yl)octanoic acid							
metab_4601	5.6623	291.1946	pos	ML-236C	C18H26O3	290.1878	173650	-	-	-	-
metab_598	4.5027	291.1946	pos	8-(4-Oxo-5-pent-2-enylcyclopent-2-en-1-yl)octanoic acid	C18H28O3	290.1879	188366	-	-	-	-
metab_598	4.5027	291.1946	pos	ML-236C	C18H26O3	290.1878	173650	-	-	-	-
metab_4745	4.9115	291.1946	pos	8-(4-Oxo-5-pent-2-enylcyclopent-2-en-1-yl)octanoic acid	C18H28O3	290.1879	188366	-	-	-	-
metab_4745	4.9115	291.1946	pos	ML-236C	C18H26O3	290.1878	173650	-	-	-	-
metab_5061	3.5011	291.1947	pos	8-(4-Oxo-5-pent-2-enylcyclopent-2-en-1-yl)octanoic acid	C18H28O3	290.1879	188366	-	-	-	-
metab_5061	3.5011	291.1947	pos	ML-236C	C18H26O3	290.1878	173650	-	-	-	-
metab_7020	6.8093	291.1970	neg	Colnelenic acid	C18H28O3	292.2040	6441679	-	-	-	-
metab_7020	6.8093	291.1970	neg	12-Oxo-phytodienoic acid	C18H28O3	292.2034	5280411	-	-	-	-
metab_7020	6.8093	291.1970	neg	9-OxoOTrE	C18H28O3	292.2036	11380794	-	-	-	-
metab_6114	1.0741	292.1285	pos	H-Trp-ser-OH	C14H17N3O4	291.1219	7009660	-	-	-	-
metab_8340	1.5439	292.1308	neg	Glutaminylphenylalanine	C14H19N3O4	293.1375	57288566	-	-	-	-
metab_13771	2.2595	292.1309	neg	Glutaminylphenylalanine	C14H19N3O4	293.1375	57288566	-	-	-	-
metab_8111	1.1796	292.1309	neg	Glutaminylphenylalanine	C14H19N3O4	293.1376	57288566	Carboxylic acids and derivatives	Amino acids, peptides, and analogues	HMDB0028804	Glutaminylphenylalanine
metab_8111	1.1796	292.1309	neg	Glutaminylphenylalanine	C14H19N3O4	293.1375	57288566	Carboxylic acids and derivatives	Amino acids, peptides, and analogues	HMDB0028804	Glutaminylphenylalanine
metab_14630	0.9649	292.1405	neg	N-(1-Deoxy-1-fructosyl)leucine	C12H23NO7	293.1475	131752244	Carboxylic acids and derivatives	Amino acids, peptides, and analogues	HMDB0037840	N-(1-Deoxy-1-fructosyl)leucine
metab_5169	3.1032	293.1011	pos	N4-Acetylsulfadiazine	C12H12N4O3S	292.0559	64952	-	-	-	-

metab_13059	4.0182	293.1071	neg	Aspartame	C14H18N2O5	294.1214	134601	Carboxylic acids and derivatives	Purines and purine derivatives	HMDB0001894	Asp-phe-ome
metab_7553	2.1153	293.1146	neg	Aspartame	C14H18N2O5	294.1216	134601	-	-	-	-
metab_7553	2.1153	293.1146	neg	Phenylalanylglutamate	C14H18N2O5	294.1215	151134	-	-	-	-
metab_7494	1.7729	293.1147	neg	Aspartame	C14H18N2O5	294.1216	134601	Carboxylic acids and derivatives	Amino acids, peptides, and analogues	HMDB0029106	Tyrosyl-Hydroxyproline
metab_7494	1.7729	293.1147	neg	Phenylalanylglutamate	C14H18N2O5	294.1215	151134	Carboxylic acids and derivatives	Amino acids, peptides, and analogues	HMDB0029106	Tyrosyl-Hydroxyproline
metab_8188	1.3089	293.1149	neg	Aspartame	C14H18N2O5	294.1216	134601	-	-	-	-
metab_8188	1.3089	293.1149	neg	Phenylalanylglutamate	C14H18N2O5	294.1215	151134	-	-	-	-
metab_8521	1.8682	293.1511	neg	Tyrosylleucine	C15H22N2O4	294.1579	87071	-	-	-	-
metab_8521	1.8682	293.1511	neg	Tyr-Ile	C15H22N2O4	294.1579	7019110	-	-	-	-
metab_14103	1.7104	293.1511	neg	Tyrosylleucine	C15H22N2O4	294.1579	87071	-	-	-	-
metab_14103	1.7104	293.1511	neg	Tyr-Ile	C15H22N2O4	294.1579	7019110	-	-	-	-
metab_1667	1.6838	293.1596	pos	B-Octylglucoside	C14H28O6	292.1887	62852	-	-	-	-
metab_9615	5.7259	293.1762	neg	Gingerol	C17H26O4	294.1830	442793	Prenol lipids	Sesquiterpenoids	HMDB0036640	Furanofukinin
metab_9615	5.7259	293.1762	neg	Phytuberin	C17H26O4	294.1834	442387	Prenol lipids	Sesquiterpenoids	HMDB0036640	Furanofukinin
metab_9615	5.7259	293.1762	neg	Embelin	C17H26O4	294.1831	3218	Prenol lipids	Sesquiterpenoids	HMDB0036640	Furanofukinin
metab_12226	7.2865	293.1797	neg	Embelin	C17H26O4	294.1867	3218	-	-	-	-
metab_10301	8.7031	293.1799	neg	Embelin	C17H26O4	294.1867	3218	-	-	-	-
metab_11549	9.1364	293.1800	neg	Embelin	C17H26O4	294.1867	3218	-	-	-	-
metab_137	5.6773	293.2103	pos	9,10-Epoxyoctadecatrienoic acid	C18H28O3	292.2035	23724711	-	-	LMFA02000112	
metab_137	5.6773	293.2103	pos	12-Oxo-phytodienoic acid	C18H28O3	292.2034	5280411	-	-	LMFA02000112	
metab_137	5.6773	293.2103	pos	9-OxoOTrE	C18H28O3	292.2036	11380794	-	-	LMFA02000112	

metab_137	5.6773	293.2103	pos	(9Z,11E,13S,15Z)-13-Hydroperoxyoctadeca-9,11,15-trienoic acid	C18H30O4	292.2036	5497123	-	-	LMFA02000112	
metab_12291	7.0796	293.2127	neg	(9Z,11E,13S,15Z)-13-Hydroxyoctadeca-9,11,15-trienoic acid	C18H30O3	294.2198	16061072	Fatty Acyls	Lineolic acids and derivatives	LMFA02000252; HMDB0004668	13-OxoODE
metab_12291	7.0796	293.2127	neg	9-OxoODE	C18H30O3	294.2193	9839084	Fatty Acyls	Lineolic acids and derivatives	LMFA02000252; HMDB0004668	13-OxoODE
metab_12291	7.0796	293.2127	neg	9(S)-HOTrE	C18H30O3	294.2198	6439873	Fatty Acyls	Lineolic acids and derivatives	LMFA02000252; HMDB0004668	13-OxoODE
metab_2988	9.0112	293.2465	pos	9(Z),11(E),13(E)-Octadecatrienoic Acid methyl ester	C19H32O2	292.2399	21718552	-	-	-	-
metab_3197	10.7001	293.2466	pos	Methyl eleostearate	C19H32O2	292.2399	21718552	-	-	-	-
metab_735	7.6332	293.2466	pos	Methyl eleostearate	C19H32O2	292.2399	21718552	-	-	-	-
metab_3073	9.6044	293.2466	pos	Methyl eleostearate	C19H32O2	292.2399	21718552	-	-	-	-
metab_3180	10.3632	293.2466	pos	Methyl eleostearate	C19H32O2	292.2399	21718552	-	-	-	-
metab_12016	7.9757	293.2490	neg	14-Methylhexadecanoic acid	C17H34O2	294.2563	22207	-	-	-	-
metab_820	9.1302	293.2831	pos	(8Z,11Z,14Z)-Icosatrien-1-ol	C20H36O	292.2764	86289279	-	-	-	-
metab_13761	2.2912	294.0988	neg	Tyramine glucuronide	C14H19NO7	313.1162	193088	Organooxygen compounds	Carbohydrates and carbohydrate conjugates	HMDB0010328	Tyramine glucuronide
metab_9454	4.7881	294.1140	neg	Lansiumamide A	C17H15NO	249.1154	11414008	Cinnamic acids and derivatives	Cinnamic acid amides	HMDB0038574	Lansiumamide A
metab_344	1.5424	294.1438	pos	Glutaminylphenylalanine	C14H19N3O4	293.1375	57288566	-	-	-	-
metab_6067	1.1731	294.1442	pos	Glutaminylphenylalanine	C14H19N3O4	293.1375	57288566	Carboxylic acids and derivatives	Amino acids, peptides, and analogues	HMDB0029004	Phenylalanyl-Serine
metab_395	1.9463	294.1529	pos	Methyl	C12H23NO7	293.1474	10493930	-	-	-	-

				4,6-dideoxy-4-(3-deoxy-L-glycero-tetronamido)-2-O-methyl-alpha-D-mannopyranoside							
metab_6026	1.2162	294.1803	pos	Lysylphenylalanine	C15H23N3O3	293.1739	151410	-	-	-	-
metab_9312	4.1364	295.1220	neg	(1S,9R,10R,12S)-12-Hydroxy-7,15-diazatetracyclo[7.7.1.02,7.010,15]heptadeca-2,4-dien-6-one	C15H20N2O2	260.1525	442943	Lupin alkaloids	Anagyrine-type alkaloids	HMDB0033481	Baptifoline
metab_1705	1.7845	295.1280	pos	Aspartame	C14H18N2O5	294.1214	134601	-	-	-	-
metab_1705	1.7845	295.1280	pos	Phenylalanylglutamate	C14H18N2O5	294.1215	151134	-	-	-	-
metab_2282	4.1387	295.1529	pos	H-Ile-Tyr-OH	C15H22N2O4	294.158	7408195	-	-	-	-
metab_5674	1.8729	295.1643	pos	Ile-tyr	C15H22N2O4	294.1580	7408195	-	-	-	-
metab_5674	1.8729	295.1643	pos	Tyrosylleucine	C15H22N2O4	294.1579	87071	-	-	-	-
metab_5674	1.8729	295.1643	pos	Tyr-Ile	C15H22N2O4	294.1579	7019110	-	-	-	-
metab_5410	2.4071	295.1645	pos	Ile-tyr	C15H22N2O4	294.1580	7408195	-	-	-	-
metab_5410	2.4071	295.1645	pos	Tyrosylleucine	C15H22N2O4	294.1579	87071	-	-	-	-
metab_5410	2.4071	295.1645	pos	Tyr-Ile	C15H22N2O4	294.1579	7019110	-	-	-	-
metab_2691	7.0670	295.2260	pos	17-Hydroxylinolenic acid	C18H30O3	294.2193	10708957	Fatty Acyls	Lineolic acids and derivatives	LMFA02000251; LMFA02000274; HMDB0004669	9-OxoODE
metab_2691	7.0670	295.2260	pos	(9Z,11E,13S,15Z)-13-Hydroxyoctadeca-9,11,15-trienoic acid	C18H30O3	294.2192	16061072	Fatty Acyls	Lineolic acids and derivatives	LMFA02000251; LMFA02000274; HMDB0004669	9-OxoODE
metab_2691	7.0670	295.2260	pos	9-OxoODE	C18H30O3	294.2192	9839084	Fatty Acyls	Lineolic acids and derivatives	LMFA02000251; LMFA02000274; HMDB0004669	9-OxoODE

metab_2691	7.0670	295.2260	pos	9(S)-HOTrE	C18H30O3	294.2192	6439873	Fatty Acyls	Lineolic acids and derivatives	LMFA02000251; LMFA02000274; HMDB0004669	9-OxoODE
metab_4653	5.3454	295.2260	pos	17-Hydroxylinolenic acid	C18H30O3	294.2193	10708957	Fatty Acyls	Fatty acids and conjugates	HMDB0031088	12,13-Epoxy-9,15-octadecadienoic acid
metab_4653	5.3454	295.2260	pos	(9Z,11E,13S,15Z)-13-Hydroxyoctadeca-9,11,15-trienoic acid	C18H30O3	294.2192	16061072	Fatty Acyls	Fatty acids and conjugates	HMDB0031088	12,13-Epoxy-9,15-octadecadienoic acid
metab_4653	5.3454	295.2260	pos	9-OxoODE	C18H30O3	294.2192	9839084	Fatty Acyls	Fatty acids and conjugates	HMDB0031088	12,13-Epoxy-9,15-octadecadienoic acid
metab_4653	5.3454	295.2260	pos	9(S)-HOTrE	C18H30O3	294.2192	6439873	Fatty Acyls	Fatty acids and conjugates	HMDB0031088	12,13-Epoxy-9,15-octadecadienoic acid
metab_12	4.9267	295.2260	pos	Pinellic acid	C18H34O5	330.2406	9858729	Fatty Acyls	Fatty acids and conjugates	HMDB0004708; LMFA02000014	9,12,13-TriHOME
metab_12	4.9267	295.2260	pos	17-Hydroxylinolenic acid	C18H30O3	294.2193	10708957	Fatty Acyls	Fatty acids and conjugates	HMDB0004708; LMFA02000014	9,12,13-TriHOME
metab_12	4.9267	295.2260	pos	(9Z,11E,13S,15Z)-13-Hydroxyoctadeca-9,11,15-trienoic acid	C18H30O3	294.2192	16061072	Fatty Acyls	Fatty acids and conjugates	HMDB0004708; LMFA02000014	9,12,13-TriHOME
metab_12	4.9267	295.2260	pos	9-OxoODE	C18H30O3	294.2192	9839084	Fatty Acyls	Fatty acids and conjugates	HMDB0004708; LMFA02000014	9,12,13-TriHOME
metab_12	4.9267	295.2260	pos	9(S)-HOTrE	C18H30O3	294.2192	6439873	Fatty Acyls	Fatty acids and conjugates	HMDB0004708; LMFA02000014	9,12,13-TriHOME
metab_6568	7.0162	295.2281	neg	(9S,10E,12Z)-9-Hydroxyoctadeca-10,12-dienoic acid	C18H32O3	296.2352	5312830	-	-	-	-
metab_6568	7.0162	295.2281	neg	Vernolic acid	C18H32O3	296.2352	5356421	-	-	-	-

metab_3021	9.1898	295.2623	pos	Sterculic acid	C19H34O2	294.2557	12921	-	-	-	-
metab_6411	0.5560	296.0651	pos	Choline Alfoscerate	C8H20NO6P	791.5829	657272	Glycerophospholipids	Glycerophosphocholines	HMDB0011228; HMDB0011221; HMDB0011222; HMDB0011223; HMDB0011224; HMDB0008753; HMDB0011226; HMDB0011227; HMDB0008488; HMDB0008489; HMDB0008161; HMDB0008160; HMDB0008162; HMDB0008619; HMDB0008360; HMDB0011290; HMDB0008685; HMDB0008687; HMDB0008721; HMDB0008720; HMDB0008817; HMDB0008027; HMDB0007962; HMDB0007963; HMDB0008260;	PC(P-16:0/22:5(7Z,10Z,13Z,16Z,19Z));PC(P-16:0/20:4(8Z,11Z,14Z,17Z));PC(P-16:0/20:5(5Z,8Z,11Z,14Z,17Z));PC(P-16:0/22:0);PC(P-16:0/22:1(13Z));PC(22:6(4Z,7Z,10Z,13Z,16Z,19Z)/P-18:1(11Z));PC(P-16:0/22:4(7Z,10Z,13Z,16Z));PC(P-16:0/22:5(4Z,7Z,10Z,13Z,16Z));PC(20:4(8Z,11Z,14Z,17Z)/P-16:0);PC(20:4(8Z,11Z,14Z,17Z)/P-18:0);PC(18:2(9Z,12Z)/P-18:1(11Z));PC(18:2(9Z,12Z)/P-18:0);PC(18:2(9Z,12Z)/P-18:1(9Z));PC(22:2(13Z,16Z)/P-16:0);PC(20:2(11Z,14Z)/P-18:1(9Z));PC(P-18:1(11Z)/22:1(13Z));PC(22:5(4Z,7Z,10Z,13Z,16Z)/P-16:0);PC(22:5(4Z,7Z,10Z,13Z,16Z)/P-18:1(11Z));PC(22:5(7Z,10Z,13Z,16Z,19Z)/P-18:1(9Z));PC(22:5(7Z,10Z,13Z,16Z,19Z)/P-18:1(11Z));PC(24:1(15Z)/P-16:0);PC(16:1(9Z)/P-16:0);PC(15:0/P-18:0);PC(15:0/P-18:1(11Z));PC(18:4(6Z,9Z,12Z,15Z)/P-18:1(11Z));PC(18:4(6Z,9Z,12Z,15Z)/P-18:1(9Z));PC(P-16:0/22:2(13Z,16Z));PC(22:

										HMDB0008261; HMDB0011225; HMDB0008587; HMDB0008586; HMDB0008060; HMDB0008061; HMDB0008062; HMDB0008063; HMDB0008589; HMDB0008588; HMDB0008785; HMDB0007895; HMDB0007896; HMDB0008786; HMDB0008622; HMDB0008620; HMDB0008621; HMDB0008455; HMDB0008820; HMDB0008457; HMDB0008193; HMDB0008194; HMDB0008195; HMDB0008784; HMDB0008458; HMDB0008357;	1(13Z)/P-18:0);PC(22:1(13Z)/P-16:0);PC(18:0/P-16:0);PC(18:0/P-18:0);PC(18:0/P-18:1(11Z));PC(18:0/P-18:1(9Z));PC(22:1(13Z)/P-18:1(9Z));PC(22:1(13Z)/P-18:1(11Z));PC(24:0/P-18:0);PC(14:0/P-16:0);PC(14:0/P-18:0);PC(24:0/P-18:1(11Z));PC(22:2(13Z,16Z)/P-18:1(9Z));PC(22:2(13Z,16Z)/P-18:0);PC(22:2(13Z,16Z)/P-18:1(11Z));PC(20:4(5Z,8Z,11Z,14Z)/P-16:0);PC(24:1(15Z)/P-18:1(9Z));PC(20:4(5Z,8Z,11Z,14Z)/P-18:1(11Z));PC(18:3(6Z,9Z,12Z)/P-18:0);PC(18:3(6Z,9Z,12Z)/P-18:1(11Z));PC(18:3(6Z,9Z,12Z)/P-18:1(9Z));PC(24:0/P-16:0);PC(20:4(5Z,8Z,11Z,14Z)/P-18:1(9Z));PC(20:2(11Z,14Z)/P-16:0);PC(20:0/P-18:1(11Z));PC(20:0/P-18:0);PC(20:2(11Z,14Z)/P-18:0);PC(20:2(11Z,14Z)/P-18:1(11Z));PC(20:0/P-18:1(9Z));PC(18:4(6Z,9Z,12Z,15Z)/P-18:0);PC(18:4(6Z,9Z,12Z,15Z)/P-16:0);PC(20:5(5Z,8Z,11Z,14Z,17Z)/P-16:0);PC(20:5(5Z,8Z,11Z,14Z,17Z)/P-18:1(11Z));PC(20:5(5Z,8Z,11Z,14Z,17Z)/P-18:1

										HMDB0008293; HMDB0008292; HMDB0008358; HMDB0008359; HMDB0008294; HMDB0008259; HMDB0008258; HMDB0008521; HMDB0008523; HMDB0008524; HMDB0008819; HMDB0008818; HMDB0008653; HMDB0008652; HMDB0008718; HMDB0008719; HMDB0008654; HMDB0008327; HMDB0008326; HMDB0008325; HMDB0008128; HMDB0008127; HMDB0008456; HMDB0008093; HMDB0008030; HMDB0000086;	(9Z));PC(24:1(15Z)/P-18:1(11Z));PC(24:1(15Z)/P-18:0);PC(22:4(7Z,10Z,13Z,16Z)/P-18:0);PC(22:4(7Z,10Z,13Z,16Z)/P-16:0);PC(22:5(7Z,10Z,13Z,16Z,19Z)/P-16:0);PC(22:5(7Z,10Z,13Z,16Z,19Z)/P-18:0);PC(22:4(7Z,10Z,13Z,16Z)/P-18:1(11Z));PC(20:1(11Z)/P-18:1(9Z));PC(20:1(11Z)/P-18:1(11Z));PC(20:1(11Z)/P-18:0);PC(18:1(9Z)/P-18:1(11Z));PC(18:1(9Z)/P-18:0);PC(20:4(5Z,8Z,11Z,14Z)/P-18:0);PC(18:1(11Z)/P-16:0);PC(16:1(9Z)/P-18:1(9Z));Glycerophosphocholine;PC(24:0/P-18:1(9Z));PC(18:3(9Z,12Z,15Z)/P-18:1(9Z));PC(20:3(8Z,11Z,14Z)/P-18:1(11Z));PC(20:3(8Z,11Z,14Z)/P-18:1(9Z));PC(18:3(9Z,12Z,15Z)/P-18:0);PC(18:3(9Z,12Z,15Z)/P-18:1(11Z));PC(20:3(8Z,11Z,14Z)/P-16:0);PC(18:3(9Z,12Z,15Z)/P-16:0);PC(20:3(8Z,11Z,14Z)/P-18:0);PC(14:1(9Z)/P-18:1(9Z));PC(14:1(9Z)/P-18:1(11Z));PC(16:0/P-18:0);PC(16:0/P-16:0);PC(16:0/P-18:1(9Z));PC(16:0/P-18:1(11Z));PC(20:3(5Z,8Z,11Z)/P-18:1(11

										HMDB0008787; HMDB0008228; HMDB0008424; HMDB0008425; HMDB0008226; HMDB0008227; HMDB0008422; HMDB0008225; HMDB0008423; HMDB0007931; HMDB0007930; HMDB0007995; HMDB0007994; HMDB0007997; HMDB0007996; HMDB0008392; HMDB0008393; HMDB0008390; HMDB0008391; HMDB0008291; HMDB0008095; HMDB0008094; HMDB0008096; HMDB0008554; HMDB0008555; HMDB0008556;	Z));PC(20:3(5Z,8Z,11Z)/P-18:1(9Z));PC(20:3(5Z,8Z,11Z)/P-16:0);PC(20:3(5Z,8Z,11Z)/P-18:0);PC(20:0/P-16:0);PC(18:1(11Z)/P-18:1(11Z));PC(18:1(11Z)/P-18:0);PC(18:1(11Z)/P-18:1(9Z));PC(22:0/P-16:0);PC(22:0/P-18:0);PC(22:0/P-18:1(11Z));PC(22:0/P-18:1(9Z));PC(20:4(8Z,11Z,14Z,17Z)/P-18:1(9Z));PC(20:4(8Z,11Z,14Z,17Z)/P-18:1(11Z));PC(14:1(9Z)/P-16:0);PC(14:1(9Z)/P-18:0);PC(18:2(9Z,12Z)/P-16:0)

										HMDB0008557; HMDB0008491; HMDB0008490; HMDB0007928; HMDB0007929; HMDB0008159	
metab_14598	1.0215	296.1001	neg	2-Methylguanosine	C11H15N5O5	297.1074	135501934	-	-	-	-
metab_14598	1.0215	296.1001	neg	Nelarabine	C11H15N5O5	297.1073	3011155	-	-	-	-
metab_12812	4.9183	296.1144	neg	Phenyl 2-acetamido-2-deoxy-alpha-D-glucopyranoside	C14H19NO6	297.1212	82399	-	-	-	-
metab_486	2.8259	297.1326	pos	Deoxynivalenol	C15H20O6	296.1259	40024	-	-	-	-
metab_11134	13.9143	297.1532	neg	6-Benzylamino-2-(2-hydroxyethylamino)-7-methylpurine	C15H18N6O	298.1604	512292	-	-	-	-
metab_11159	12.1016	297.1532	neg	6-Benzylamino-2-(2-hydroxyethylamino)-7-methylpurine	C15H18N6O	298.1604	512292	-	-	-	-
metab_12427	6.6157	297.1533	neg	6-Benzylamino-2-(2-hydroxyethylamino)-7-methylpurine	C15H18N6O	298.1604	512292	-	-	-	-
metab_11734	8.7031	297.1534	neg	6-Benzylamino-2-(2-hydroxyethylamino)-7-methylpurine	C15H18N6O	298.1604	512292	-	-	-	-
metab_4881	4.2609	297.1687	pos	Alhpa-tocopheronic acid	C16H24O5	296.1624	52929835	-	-	LMPR02020062	
metab_9	5.1975	297.2415	pos	(9Z,11E,13S)-13-Hydroxyoctadeca-9,11-dienoic acid	C18H32O3	296.2348	6443013	Fatty Acyls	Fatty acids and conjugates	HMDB0034295; LMFA02000147	Floionolic acid
metab_9	5.1975	297.2415	pos	Vernolic acid	C18H32O3	296.2352	5356421	Fatty Acyls	Fatty acids and conjugates	HMDB0034295; LMFA02000147	Floionolic acid
metab_4288	7.2763	297.2415	pos	(9Z,11E,13S)-13-Hydroxyocta	C18H32O3	296.2348	6443013	-	-	LMFA02000187	

				deca-9,11-dienoic acid							
metab_4288	7.2763	297.2415	pos	Vernolic acid	C18H32O3	296.2352	5356421	-	-	LMFA02000187	
metab_4541	5.9649	297.2415	pos	(9Z,11E,13S)-13-Hydroxyoctadeca-9,11-dienoic acid	C18H32O3	296.2348	6443013	-	-	LMFA02000182	
metab_4541	5.9649	297.2415	pos	Vernolic acid	C18H32O3	296.2352	5356421	-	-	LMFA02000182	
metab_12236	7.2377	297.2436	neg	18-Hydroxyoleate	C18H34O3	298.2510	86289578	Fatty Acyls	Fatty alcohols	HMDB0013813	13-Heptadecyn-1-ol
metab_12236	7.2377	297.2436	neg	Ricinoleic acid	C18H34O3	298.2509	643684	Fatty Acyls	Fatty alcohols	HMDB0013813	13-Heptadecyn-1-ol
metab_12236	7.2377	297.2436	neg	9,10-Epoxystearic acid	C18H34O3	298.2510	15868	Fatty Acyls	Fatty alcohols	HMDB0013813	13-Heptadecyn-1-ol
metab_12236	7.2377	297.2436	neg	13-Heptadecyn-1-ol	C17H32O	252.2453	557439	Fatty Acyls	Fatty alcohols	HMDB0013813	13-Heptadecyn-1-ol
metab_12459	6.4709	297.2441	neg	18-Hydroxyoleate	C18H34O3	298.2510	86289578	-	-	-	-
metab_12459	6.4709	297.2441	neg	Ricinoleic acid	C18H34O3	298.2509	643684	-	-	-	-
metab_12459	6.4709	297.2441	neg	9,10-Epoxystearic acid	C18H34O3	298.2510	15868	-	-	-	-
metab_1066	1.4569	298.0960	pos	5'-Deoxy-5'-methylthioadenosine	C11H15N5O3S	297.0895	439176	-	-	-	-
metab_5518	2.1746	298.1277	pos	Phenyl 2-acetamido-2-deoxy-alpha-D-glucopyranoside	C14H19NO6	297.1212	82399	-	-	-	-
metab_306	1.3293	298.1277	pos	Phenyl 2-acetamido-2-deoxy-alpha-D-glucopyranoside	C14H19NO6	297.1212	82399	-	-	-	-
metab_4276	7.3370	298.2732	pos	3-Ketosphingosine	C18H35NO2	297.2666	9839212	-	-	-	-
metab_4276	7.3370	298.2732	pos	Cassine	C18H35NO2	297.2668	193405	-	-	-	-
metab_2793	7.8097	298.2732	pos	3-Ketosphingosine	C18H35NO2	297.2666	9839212	-	-	-	-
metab_2793	7.8097	298.2732	pos	Cassine	C18H35NO2	297.2668	193405	-	-	-	-
metab_997	6.2978	298.2732	pos	3-Ketosphingosine	C18H35NO2	297.2666	9839212	-	-	-	-

metab_997	6.2978	298.2732	pos	Cassine	C18H35NO2	297.2668	193405	-	-	-	-
metab_1857	2.2665	299.0842	pos	(S)-a-Amino-2,5-dihydro-5-oxo-4-isoxazolepropanoic acid N2-glucoside	C12H18N2O9	334.1012	131750866	Organooxygen compounds	Carbohydrates and carbohydrate conjugates	HMDB0029404	(S)-a-Amino-2,5-dihydro-5-oxo-4-isoxazolepropanoic acid N2-glucoside
metab_13825	2.1630	299.1141	neg	Salidroside	C14H20O7	300.1210	159278	-	-	-	-
metab_2230	3.8062	299.1839	pos	Iso-Olomoucine	C15H18N6O	298.1604	512292	-	-	-	-
metab_5078	3.4098	299.1845	pos	Iso-Olomoucine	C15H18N6O	298.1604	512292	-	-	-	-
metab_4157	7.9835	299.2570	pos	9,10-Epoxystearic acid	C18H34O3	298.2510	15868	-	-	-	-
metab_3154	10.1021	299.2571	pos	9,10-Epoxystearic acid	C18H34O3	298.2510	15868	-	-	-	-
metab_122	7.5005	299.2572	pos	9,10-Epoxystearic acid	C18H34O3	298.2510	15868	-	-	-	-
metab_904	9.8362	299.2572	pos	9,10-Epoxystearic acid	C18H34O3	298.2510	15868	-	-	-	-
metab_3916	8.9656	299.2573	pos	9,10-Epoxystearic acid	C18H34O3	298.2510	15868	-	-	-	-
metab_3839	9.3117	299.2574	pos	9,10-Epoxystearic acid	C18H34O3	298.2510	15868	-	-	-	-
metab_12002	8.0069	299.2593	neg	(R)-10-Hydroxystearate	C18H36O3	300.2666	5459807	Fatty Acyls	Fatty acids and conjugates	HMDB0061662	13-hydroxyoctadecanoic acid
metab_12002	8.0069	299.2593	neg	2-Hydroxyoctadecanoic acid	C18H36O3	300.2666	69417	Fatty Acyls	Fatty acids and conjugates	HMDB0061662	13-hydroxyoctadecanoic acid
metab_12002	8.0069	299.2593	neg	13-Hydroxyoctadecanoic acid	C18H36O3	300.2664	5282911	Fatty Acyls	Fatty acids and conjugates	HMDB0061662	13-hydroxyoctadecanoic acid
metab_6633	9.1038	299.2595	neg	(R)-10-Hydroxystearate	C18H36O3	300.2666	5459807	-	-	LMFA02000120	
metab_6633	9.1038	299.2595	neg	2-Hydroxyoctadecanoic acid	C18H36O3	300.2666	69417	-	-	LMFA02000120	
metab_13874	2.0670	300.1932	neg	Leu-Leu-Gly	C14H27N3O4	301.2001	7019089	-	-	-	-
metab_13874	2.0670	300.1932	neg	Val-Leu-Ala	C14H27N3O4	301.2001	25157726	-	-	-	-
metab_13624	2.5431	300.1932	neg	Leu-Leu-Gly	C14H27N3O4	301.2001	7019089	-	-	-	-
metab_13624	2.5431	300.1932	neg	Val-Leu-Ala	C14H27N3O4	301.2001	25157726	-	-	-	-

metab_8872	2.6986	300.1933	neg	Leu-Leu-Gly	C14H27N3O4	301.2001	7019089	-	-	-	-
metab_8872	2.6986	300.1933	neg	Val-Leu-Ala	C14H27N3O4	301.2001	25157726	-	-	-	-
metab_14227	1.5439	300.1934	neg	Leu-Leu-Gly	C14H27N3O4	301.2001	7019089	-	-	-	-
metab_14227	1.5439	300.1934	neg	Val-Leu-Ala	C14H27N3O4	301.2001	25157726	-	-	-	-
metab_14227	1.5439	300.1934	neg	Gly Leu Leu	C14H27N3O4	301.1999	13037660	-	-	-	-
metab_14227	1.5439	300.1934	neg	Gly Ile Ile	C14H27N3O4	301.2	145455641	-	-	-	-
metab_4040	8.4356	300.2886	pos	Palmitoylethanolamide	C18H37NO2	299.2824	4671	Carboximidic acids and derivatives	Carboximidic acids	HMDB0002100; LMFA08040013; LMFA08040057	Palmitoylethanolamide
metab_5949	1.3437	301.0847	pos	Rhamnocitrin	C16H12O6	300.0631	5320946	-	-	-	-
metab_5949	1.3437	301.0847	pos	Pratensein	C16H12O6	300.0631	5281803	Isoflavonoids	O-methylated isoflavonoids	HMDB0030617	Pratensein
metab_13751	2.3078	301.1299	neg	Isomucronulatol	C17H18O5	302.115424	602152	Isoflavonoids	O-methylated isoflavonoids	HMDB0033189	Astraisoflavan
metab_6468	0.5140	301.1498	pos	(-)-Sparticarpin	C17H16O5	300.0996	442823	-	-	-	-
metab_6468	0.5140	301.1498	pos	Astrapterocarpan	C17H16O5	300.0996	14077830	-	-	-	-
metab_6468	0.5140	301.1498	pos	Astrapterocarpan	C17H16O5	300.099774	14077830	-	-	-	-
metab_7856	0.5286	301.1632	neg	Tributyrin	C15H26O6	302.1727	6050	Glycerolipids	Triradylcglycerols	HMDB0031094	Tributyryl glyceride
metab_7617	2.5748	301.1661	neg	Tributyrin	C15H26O6	302.1735	6050	-	-	-	-
metab_8507	1.8205	301.1773	neg	Abietic acid	C20H30O2	302.2246	10569	-	-	-	-
metab_9299	4.1023	301.2024	neg	Arg-Lys	C12H26N6O3	302.2066	6427006	Carboxylic acids and derivatives	Amino acids, peptides, and analogues	HMDB0028714	Arginyl-Lysine
metab_4963	3.8821	301.2113	pos	Dehydroabietic acid	C20H28O2	300.2091	94391	-	-	-	-
metab_7579	0.5711	302.1011	neg	5-Methylcytidine	C10H15N3O5	257.1012	92918	Pyrimidine nucleosides	-	HMDB0000982	5-Methylcytidine
metab_5656	1.9309	302.1493	pos	3-[3-[(2R,3S)-3-Hydroxy-2-pi	C16H19N3O3	301.1425	9851692	-	-	-	-

				peridinyl]-2-oxopropyl]-4(3H) -quinazolinone							
metab_1613	1.5284	302.2064	pos	Glycyl-L-leucyl-L-leucine	C14H27N3O4	301.1999	13037660	-	-	-	-
metab_1613	1.5284	302.2064	pos	Gly-Ile-Ile	C14H27N3O4	301.2000	145455641	-	-	-	-
metab_1613	1.5284	302.2064	pos	Leu-Ala-Val	C14H27N3O4	301.2000	54565935	-	-	-	-
metab_1613	1.5284	302.2064	pos	Val-Ala-Ile	C14H27N3O4	301.2000	145458821	-	-	-	-
metab_1613	1.5284	302.2064	pos	Leu-Leu-Gly	C14H27N3O4	301.2000	7019089	-	-	-	-
metab_1613	1.5284	302.2064	pos	Val-Leu-Ala	C14H27N3O4	301.2001	25157726	-	-	-	-
metab_1806	2.1290	302.2065	pos	Glycyl-L-leucyl-L-leucine	C14H27N3O4	301.1999	13037660	-	-	-	-
metab_1806	2.1290	302.2065	pos	Gly-Ile-Ile	C14H27N3O4	301.2000	145455641	-	-	-	-
metab_1806	2.1290	302.2065	pos	Leu-Ala-Val	C14H27N3O4	301.2000	54565935	-	-	-	-
metab_1806	2.1290	302.2065	pos	Val-Ala-Ile	C14H27N3O4	301.2000	145458821	-	-	-	-
metab_1806	2.1290	302.2065	pos	Leu-Leu-Gly	C14H27N3O4	301.2000	7019089	-	-	-	-
metab_1806	2.1290	302.2065	pos	Val-Leu-Ala	C14H27N3O4	301.2001	25157726	-	-	-	-
metab_5291	2.7188	302.2066	pos	Gly Leu Leu	C14H27N3O4	301.1999	13037660	-	-	-	-
metab_5291	2.7188	302.2066	pos	Gly-Ile-Ile	C14H27N3O4	301.2000	145455641	-	-	-	-
metab_5291	2.7188	302.2066	pos	Leu-Ala-Val	C14H27N3O4	301.2000	54565935	-	-	-	-
metab_5291	2.7188	302.2066	pos	Val-Ala-Ile	C14H27N3O4	301.2000	145458821	-	-	-	-
metab_5291	2.7188	302.2066	pos	Leu-Leu-Gly	C14H27N3O4	301.2000	7019089	-	-	-	-
metab_5291	2.7188	302.2066	pos	Val-Leu-Ala	C14H27N3O4	301.2001	25157726	-	-	-	-
metab_6065	1.1731	303.1446	pos	Histidylphenylalanine	C15H18N4O3	302.1378	152198	-	-	-	-
metab_9256	3.9177	303.1818	neg	16beta,17beta-Dihydroxy-16-methylestr-4-en-3-one	C19H28O3	304.2035	11954109	-	-	-	-
metab_9582	5.5639	303.2181	neg	Ethyl 3-hydroxytridecanoate	C15H30O3	258.2195	575914	Fatty Acyls	Fatty alcohols	HMDB0059866	Ethyl 3-hydroxytridecanoate
metab_1039	1.6693	304.1283	pos	N-(Indol-3-ylacetyl)glutamine	C15H17N3O4	303.1219	25200879	Carboxylic acids and	Amino acids, peptides,	HMDB0013240	Indoleacetyl glutamine

								derivatives	and analogues		
metab_5077	3.4251	304.1385	pos	Anserine	C10H16N4O3	240.1222	112072	Peptidomimetics	Hybrid peptides	HMDB0000194	Anserine
metab_6183	0.8920	304.1492	pos	Nicotianamine	C12H21N3O6	303.1428	9882882	-	-	-	-
metab_1586	1.4569	304.1640	pos	N(6)-[(Indol-3-yl)acetyl]-L-lysine	C16H21N3O3	303.1581	161240	-	-	-	-
metab_1788	2.0828	304.1649	pos	N(6)-[(Indol-3-yl)acetyl]-L-lysine	C16H21N3O3	303.1581	161240	-	-	-	-
metab_4947	3.9421	304.1746	pos	Leu-Thr-Ala	C13H25N3O5	303.1793	71464637	-	-	-	-
metab_5940	1.3579	304.1858	pos	Leu-Thr-Ala	C13H25N3O5	303.1793	71464637	-	-	-	-
metab_9756	6.4874	304.1925	neg	Capsaicin	C18H27NO3	305.1989	1548943	-	-	-	-
metab_6316	0.6262	305.1445	pos	D-Nopaline	C11H20N4O6	304.1382	108012	-	-	-	-
metab_13400	3.0432	305.1511	neg	Cyclo(L-leucyl-L-phenylalanyl)	C15H20N2O2	260.1525	7076347	Carboxylic acids and derivatives	Amino acids, peptides, and analogues	HMDB0094673	Cyclo(Leu-Phe)
metab_4762	4.8208	305.2102	pos	16beta,17beta-Dihydroxy-16-methylestr-4-en-3-one	C19H28O3	304.2035	11954109	-	-	-	-
metab_4762	4.8208	305.2102	pos	7alpha-Hydroxytestosterone	C19H28O3	304.2037	65541	-	-	-	-
metab_4795	4.6096	305.2103	pos	7alpha-Hydroxytestosterone	C19H28O3	304.2037	65541	-	-	-	-
metab_4795	4.6096	305.2103	pos	16-alpha-Methyl-16-beta-hydroxy-19-nortestosterone	C19H28O3	304.2035	11954109	-	-	-	-
metab_7940	0.6271	306.0767	neg	N-Acetyl-b-neuraminic acid	C11H19NO9	307.0842	316	-	-	-	-
metab_12392	6.7926	306.0770	neg	Glutathione	C10H17N3O6S	307.0838	124886	Carboxylic acids and derivatives	Amino acids, peptides, and analogues	HMDB0000125; HMDB0062697	Glutathione;Glutathionate(1-)
metab_13339	3.1964	306.1351	neg	Monocrotaline	C16H23NO6	325.1525	9415	Pyrrolizines	-	HMDB0034363	Monocrotaline
metab_1579	1.4429	306.1802	pos	6'-Oxolividamine	C12H23N3O6	305.1586	121231420	-	-	-	-
metab_2624	6.7516	306.2055	pos	Capsaicin	C18H27NO3	305.1989	1548943	-	-	-	-

metab_12719	5.3546	306.2077	neg	Betaxolol	C18H29NO3	307.2147	2369	Phenols	Fatty acids and conjugates	HMDB0014341	Boots brand OF betaxolol hydrochloride
metab_13062	4.0182	306.2079	neg	N-[[3-Hydroxy-2-(2-pentenyl) cyclopentyl]acetyl]isoleucine	C18H31NO4	325.2253	131753085	Carboxylic acids and derivatives	Amino acids, peptides, and analogues	HMDB0041248	N-[[3-Hydroxy-2-(2-pentenyl)cyclopentyl]acetyl]isoleucine
metab_4395	6.8433	306.2418	pos	Capsaicin	C18H27NO3	305.1989	1548943	Phenols	Fatty amides	HMDB0002227	epsilon-Capsaicin
metab_14855	0.5851	307.1150	neg	Nopalinic acid	C10H18N2O6	262.1165	4474580	Carboxylic acids and derivatives	Amino acids, peptides, and analogues	HMDB0029437	Nopalinic acid
metab_7655	0.5431	307.1514	neg	1-[(5-Amino-5-carboxypentyl) amino]-1-deoxyfructose	C12H24N2O7	308.1584	131751634	Organooxygen compounds	Carbohydrates and carbohydrate conjugates	HMDB0034879	1-[(5-Amino-5-carboxypentyl)amino]-1-deoxyfructose
metab_12966	4.3207	307.1556	neg	Ubenimex	C16H24N2O4	308.1735	72172	-	-	-	-
metab_584	4.2457	307.1896	pos	Compactin diol lactone	C18H26O4	306.1828	173651	-	-	-	-
metab_611	3.9270	307.1897	pos	Compactin diol lactone	C18H26O4	306.1828	173651	-	-	-	-
metab_12775	5.0650	307.1917	neg	5-O-Methylembelin	C18H28O4	308.1984	171489	-	-	-	-
metab_12775	5.0650	307.1917	neg	Soraphen O	C18H28O4	308.1990	49787023	-	-	-	-
metab_12602	5.8896	307.1918	neg	5-O-Methylembelin	C18H28O4	308.1984	171489	Organooxygen compounds	Carbonyl compounds	HMDB0036177	3,5,5-Trimethyl-1,2-cyclohexanedione
metab_12602	5.8896	307.1918	neg	Soraphen O	C18H28O4	308.1990	49787023	Organooxygen compounds	Carbonyl compounds	HMDB0036177	3,5,5-Trimethyl-1,2-cyclohexanedione
metab_9442	4.7384	307.1919	neg	5-O-Methylembelin	C18H28O4	308.1984	171489	-	-	-	-
metab_9442	4.7384	307.1919	neg	Soraphen O	C18H28O4	308.1990	49787023	-	-	-	-
metab_9385	4.5054	307.1920	neg	5-O-Methylembelin	C18H28O4	308.1984	171489	-	-	-	-
metab_9385	4.5054	307.1920	neg	Soraphen O	C18H28O4	308.1990	49787023	-	-	-	-
metab_4275	7.3370	307.2259	pos	Androst-5-ene-3beta,17beta,19-triol	C19H30O3	306.2192	256730	-	-	-	-
metab_4032	8.4651	307.2259	pos	Androst-5-ene-3beta,17beta,19-triol	C19H30O3	306.2192	256730	-	-	-	-

metab_24	6.4035	307.2259	pos	Androst-5-ene-3beta,17beta,19-triol	C19H30O3	306.2192	256730	-	-	-	-
metab_5000	3.7147	307.2260	pos	Androst-5-ene-3beta,17beta,19-triol	C19H30O3	306.2192	256730	-	-	-	-
metab_10218	8.4336	307.2282	neg	Obtusilactone A	C19H32O3	308.2351	6442492	Tetrahydrofurans	-	HMDB0030960	Obtusilactone A
metab_10218	8.4336	307.2282	neg	(10E,12Z,15Z)-9-Hydroxy-10,12,15-octadecatrienoic acid methyl ester	C19H32O3	308.2354	10244787	Tetrahydrofurans	-	HMDB0030960	Obtusilactone A
metab_4178	7.9118	307.2623	pos	11,14,17-Eicosatrienoic acid	C20H34O2	306.2557	5312529	-	-	-	-
metab_4178	7.9118	307.2623	pos	Cembra-2,7,11-triene-4,6-diol	C20H34O2	306.2556	11109567	-	-	-	-
metab_1809	2.1290	308.0901	pos	NA	C11H19NO9	307.0842	NA	-	-	-	-
metab_2299	4.2907	308.0903	pos	NA	C11H19NO9	307.0842	NA	-	-	-	-
metab_5342	2.5627	308.0903	pos	NA	C11H19NO9	307.0842	NA	-	-	-	-
metab_1905	2.4370	308.0903	pos	NA	C11H19NO9	307.0842	NA	-	-	-	-
metab_5091	3.3490	308.0907	pos	NA	C11H19NO9	307.0842	NA	-	-	-	-
metab_1604	1.5144	308.1848	pos	Tebuconazole	C16H22ClN3O	307.1452	86102	-	-	-	-
metab_2560	6.0864	308.2211	pos	Betaxolol	C18H29NO3	307.2147	2369	Phenols	Tyrosols and derivatives	HMDB0014341	Boots brand OF betaxolol hydrochloride
metab_4411	6.7516	308.2211	pos	Betaxolol	C18H29NO3	307.2147	2369	-	-	-	-
metab_626	5.0451	308.2212	pos	Betaxolol	C18H29NO3	307.2147	2369	-	-	-	-
metab_570	4.0479	308.2212	pos	Betaxolol	C18H29NO3	307.2147	2369	-	-	-	-
metab_5081	3.4098	309.0862	pos	Flazin	C17H12N2O4	308.0797	5377686	Harmala alkaloids	-	HMDB0033459	Flazine
metab_14515	1.2377	309.1103	neg	NA	C14H18N2O6	310.1165	NA	Carboxylic acids and derivatives	Amino acids, peptides, and analogues	HMDB0028831	Glutamyltyrosine

metab_5236	2.8877	309.1589	pos	Isopropalin	C15H23N3O4	309.1688	36606	-	-	-	-
metab_9869	7.0322	309.1744	neg	Bestatin hydrochloride	C16H24N2O4	310.1817	11957481	-	-	-	-
metab_12388	6.8093	309.1746	neg	Bestatin hydrochloride	C16H24N2O4	310.1817	11957481	-	-	-	-
metab_378	1.7554	309.1798	pos	Ubenimex	C16H24N2O4	308.1735	72172	-	-	-	-
metab_2533	5.9049	309.2050	pos	5-O-Methylembelin	C18H28O4	308.1984	171489	-	-	-	-
metab_2533	5.9049	309.2050	pos	Soraphen O	C18H28O4	308.1986	49787023	-	-	-	-
metab_2432	5.1372	309.2051	pos	5-O-Methylembelin	C18H28O4	308.1984	171489	-	-	-	-
metab_2432	5.1372	309.2051	pos	Soraphen O	C18H28O4	308.1986	49787023	-	-	-	-
metab_4427	6.6001	309.2051	pos	5-O-Methylembelin	C18H28O4	308.1984	171489	Fatty Acyls	Lineolic acids and derivatives	HMDB0033243	Corchorifatty acid D
metab_4427	6.6001	309.2051	pos	Soraphen O	C18H28O4	308.1986	49787023	Fatty Acyls	Lineolic acids and derivatives	HMDB0033243	Corchorifatty acid D
metab_4427	6.6001	309.2051	pos	Corchorifatty acid D	C18H28O4	308.1988	9861431	Fatty Acyls	Lineolic acids and derivatives	HMDB0033243	Corchorifatty acid D
metab_2303	4.3371	309.2052	pos	5-O-Methylembelin	C18H28O4	308.1984	171489	-	-	-	-
metab_2303	4.3371	309.2052	pos	Soraphen O	C18H28O4	308.1986	49787023	-	-	-	-
metab_11997	8.0069	309.2075	neg	(8E,10S,12Z,15Z)-10-Hydroperoxyoctadeca-8,12,15-trienoic acid	C18H30O4	310.2147	71728395	-	-	-	-
metab_11997	8.0069	309.2075	neg	9-Oxo-11-(3-pentyloxiran-2-YL)undec-10-enoic acid	C18H30O4	310.2147	53394018	-	-	-	-
metab_11997	8.0069	309.2075	neg	(9Z,11E,13S,15Z)-13-Hydroperoxyoctadeca-9,11,15-trienoic acid	C18H30O4	310.2147	5497123	-	-	-	-
metab_11997	8.0069	309.2075	neg	(9R,10E,12Z,15Z)-9-Hydroperoxyoctadeca-10,12,15-trienoat	C18H30O4	310.2147	56927761	-	-	-	-

				e							
metab_6673	5.6615	309.2076	neg	(8E,10S,12Z,15Z)-10-Hydroperoxyoctadeca-8,12,15-trienoic acid	C18H30O4	310.2147	71728395	Fatty Acyls	Fatty acids and conjugates	HMDB0013623	12(13)Ep-9-KODE
metab_6673	5.6615	309.2076	neg	9-Oxo-11-(3-pentyloxiran-2-YL)undec-10-enoic acid	C18H30O4	310.2147	53394018	Fatty Acyls	Fatty acids and conjugates	HMDB0013623	12(13)Ep-9-KODE
metab_6673	5.6615	309.2076	neg	(9Z,11E,13S,15Z)-13-Hydroperoxyoctadeca-9,11,15-trienoic acid	C18H30O4	310.2147	5497123	Fatty Acyls	Fatty acids and conjugates	HMDB0013623	12(13)Ep-9-KODE
metab_6673	5.6615	309.2076	neg	(9R,10E,12Z,15Z)-9-Hydroperoxyoctadeca-10,12,15-trienoate	C18H30O4	310.2147	56927761	Fatty Acyls	Fatty acids and conjugates	HMDB0013623	12(13)Ep-9-KODE
metab_679	6.5395	309.2415	pos	(10E,12Z,15Z)-9-Hydroxy-10,12,15-octadecatrienoic acid methyl ester	C19H32O3	308.2348	10244787	-	-	-	-
metab_4197	7.7946	309.2415	pos	(10E,12Z,15Z)-9-Hydroxy-10,12,15-octadecatrienoic acid methyl ester	C19H32O3	308.2348	10244787	-	-	LMFA01070012	
metab_2703	7.1416	309.2415	pos	Methyl (10E,12Z,15Z)-9-hydroxyoctadeca-10,12,15-trienoate	C19H32O3	308.2348	10244787	-	-	-	-
metab_11896	8.3055	309.2439	neg	Methoprene	C19H34O3	310.2511	5366546	Fatty Acyls	Fatty alcohols	HMDB0031060	(R)-2-Hydroxysterculic acid
metab_11896	8.3055	309.2439	neg	2-Hydroxy-8-(2-octylcycloprop-1-en-1-yl)octanoic acid	C19H34O3	310.2508	131751126	Fatty Acyls	Fatty alcohols	HMDB0031060	(R)-2-Hydroxysterculic acid
metab_11762	8.6874	309.2440	neg	Methoprene	C19H34O3	310.2511	5366546	-	-	-	-
metab_11571	9.1038	309.2440	neg	Methoprene	C19H34O3	310.2511	5366546	-	-	-	-

metab_14313	1.4710	310.0937	neg	Dopamine glucuronide	C14H19NO8	329.1111	3082490	Organooxygen compounds	Carbohydrates and carbohydrate conjugates	HMDB0010329	Dopamine glucuronide
metab_14760	0.6551	310.1146	neg	NA	C12H17N5O5	311.1229	92919	-	-	-	-
metab_14707	0.7958	310.1146	neg	NA	C12H17N5O5	311.1229	92919	-	-	-	-
metab_7	1.2585	310.1276	pos	N-(1-Deoxy-1-fructosyl)phenylalanine	C15H21NO7	327.1318	101039148	Carboxylic acids and derivatives	Amino acids, peptides, and analogues	HMDB0037846	N-(1-Deoxy-1-fructosyl)phenylalanine
metab_6121	1.0601	310.1387	pos	Tyrosyl-Glutamine	C14H19N3O5	309.1324	7020179	-	-	-	-
metab_6023	1.2162	310.1750	pos	Isopropalin	C15H23N3O4	309.1688	36606	-	-	-	-
metab_1236	0.5140	310.1866	pos	Isopropalin	C15H23N3O4	309.1688	36606	-	-	-	-
metab_4989	3.7599	310.2368	pos	(S,Z)-N-(2-Oxotetrahydrofuran-3-yl)tetradec-9-enamide	C18H31NO3	309.2304	35028743	-	-	-	-
metab_3358	14.6110	310.3095	pos	(1Z)-N-Ethyloctadec-9-enimidate	C20H39NO	309.3028	53442596	-	-	-	-
metab_3358	14.6110	310.3095	pos	Oleoyl ethylamide	C20 H39 N O	309.3029	44270319	-	-	-	-
metab_1191	0.2224	310.3095	pos	(1Z)-N-Ethyloctadec-9-enimidate	C20H39NO	309.3028	53442596	-	-	-	-
metab_1191	0.2224	310.3095	pos	Oleoyl ethylamide	C20 H39 N O	309.3029	44270319	-	-	-	-
metab_960	9.1302	310.3096	pos	(1Z)-N-Ethyloctadec-9-enimidate	C20H39NO	309.3028	53442596	-	-	-	-
metab_960	9.1302	310.3096	pos	Oleoyl ethylamide	C20 H39 N O	309.3029	44270319	-	-	-	-
metab_4997	3.7297	310.3097	pos	(1Z)-N-Ethyloctadec-9-enimidate	C20H39NO	309.3028	53442596	-	-	-	-
metab_4997	3.7297	310.3097	pos	Oleoyl ethylamide	C20 H39 N O	309.3029	44270319	-	-	-	-
metab_13605	2.6046	311.1406	neg	Phenylalanylphenylalanine	C18H20N2O3	312.1474	65104	Carboxylic acids and derivatives	Amino acids, peptides, and analogues	HMDB0013302	Phenylalanylphenylalanine
metab_12319	7.0162	311.1690	neg	Triptophenolide	C20H24O3	312.1762	173273	-	-	-	-

metab_11744	8.7031	311.1691	neg	Triptophenolide	C20H24O3	312.1762	173273	-	-	-	-
metab_9500	5.0812	311.1868	neg	Sancuso	C18H24N4O	312.1940	5284566	Fatty Acyls	Fatty acids and conjugates	HMDB0112085	3,4-Dimethyl-5-pentyl-2-furanpentanoic acid
metab_130	6.3277	311.2207	pos	(9R,10E,12Z,15Z)-9-Hydroperoxyoctadeca-10,12,15-trienoate	C18H30O4	310.2140	56927761	-	-	-	-
metab_2474	5.4662	311.2208	pos	(9R,10E,12Z,15Z)-9-Hydroperoxyoctadeca-10,12,15-trienoate	C18H30O4	310.2140	56927761	-	-	-	-
metab_4755	4.8809	311.2208	pos	(9R,10E,12Z,15Z)-9-Hydroperoxyoctadeca-10,12,15-trienoate	C18H30O4	310.2140	56927761	-	-	-	-
metab_9567	5.4684	311.2231	neg	9(S)-Hpode	C18H32O4	312.2304	9548877	-	-	-	-
metab_9567	5.4684	311.2231	neg	13(S)-Hpode	C18H32O4	312.2297	5280720	-	-	-	-
metab_12595	5.9375	311.2232	neg	9(S)-Hpode	C18H32O4	312.2304	9548877	Fatty Acyls	Lineolic acids and derivatives	HMDB0003871	13-L-Hydroperoxylinoleic acid
metab_12595	5.9375	311.2232	neg	13(S)-Hpode	C18H32O4	312.2297	5280720	Fatty Acyls	Lineolic acids and derivatives	HMDB0003871	13-L-Hydroperoxylinoleic acid
metab_12514	6.2943	311.2233	neg	9(S)-Hpode	C18H32O4	312.2304	9548877	Fatty Acyls	Lineolic acids and derivatives	HMDB0006940	9(S)-HPODE
metab_12514	6.2943	311.2233	neg	13(S)-Hpode	C18H32O4	312.2297	5280720	Fatty Acyls	Lineolic acids and derivatives	HMDB0006940	9(S)-HPODE
metab_877	10.1647	311.2571	pos	Methyl 13-hydroxyoctadeca-9,11-dienoate	C19H34O3	310.2508	31868	-	-	-	-
metab_12694	5.5002	312.2548	neg	(+)-Prosopinine	C18H35NO3	313.2613	442654	-	-	-	-

metab_10167	8.2253	312.2548	neg	(+)-Prosopinine	C18H35NO3	313.2613	442654	-	-	-	-
metab_11859	8.4336	312.2549	neg	(+)-Prosopinine	C18H35NO3	313.2613	442654	-	-	-	-
metab_11763	8.6874	312.2549	neg	(+)-Prosopinine	C18H35NO3	313.2613	442654	-	-	-	-
metab_3785	9.5749	312.3251	pos	2,6-Dimethyl-4-tetradecylmorpholine	C20H41NO	311.3185	14993215	-	-	-	-
metab_3005	9.0999	312.3252	pos	2,6-Dimethyl-4-tetradecylmorpholine	C20H41NO	311.3185	14993215	-	-	-	-
metab_8768	2.4175	313.0721	neg	3,7-Dihydroxy-3',4'-dimethoxyflavone	C17H14O6	314.0794	5378832	-	-	-	-
metab_14415	1.3373	313.0936	neg	2-Hydroxyphenylacetic acid O-b-D-glucoside	C14H18O8	314.1002	76503563	Organooxygen compounds	Carbohydrates and carbohydrate conjugates	HMDB0038333	2-Hydroxyphenylacetic acid O-b-D-glucoside
metab_5328	2.6090	313.1541	pos	Phenylalanylphenylalanine	C18H20N2O3	312.1472	65104	-	-	-	-
metab_550	2.2519	313.1542	pos	Phenylalanylphenylalanine	C18H20N2O3	312.1472	65104	-	-	-	-
metab_1628	1.5565	313.1747	pos	TRIPTOPHENOLIDE	C20H24O3	312.1762	173273	-	-	-	-
metab_2295	4.2301	313.2364	pos	(9Z,11E)-(13S)-13-Hydroperoxyoctadeca-9,11-dienoic acid	C18H32O4	312.2297	5280720	Fatty Acyls		HMDB0003871	13S-Hydroperoxy-9Z,11E-octadecadienoic acid
metab_141	5.2868	313.2364	pos	13(S)-Hpode	C18H32O4	312.2297	5280720	-	-	-	-
metab_6623	6.4073	313.2389	neg	(Z)-(7S,8S)-Dihydroxyoctadeca-9-enoate	C18H34O4	314.2460	5281030	Fatty Acyls	Fatty acids and conjugates	HMDB0031679; HMDB0004704; LMFA02000229	(9xi,10xi,12xi)-9,10-Dihydroxy-12-octadecenoic acid;9,10-DHOME
metab_6623	6.4073	313.2389	neg	(12Z)-9,10-Dihydroxyoctadec-12-enoic acid	C18H34O4	314.2459	9966640	Fatty Acyls	Fatty acids and conjugates	HMDB0031679; HMDB0004704; LMFA02000229	(9xi,10xi,12xi)-9,10-Dihydroxy-12-octadecenoic acid;9,10-DHOME
metab_6623	6.4073	313.2389	neg	12,13-DiHOME	C18H34O4	314.2459	10236635	Fatty Acyls	Fatty acids and conjugates	HMDB0031679; HMDB0004704; LMFA02000229	(9xi,10xi,12xi)-9,10-Dihydroxy-12-octadecenoic acid;9,10-DHOME

metab_3032	9.2815	313.2726	pos	(2E)-19-Hydroxynonadec-2-enoic acid	C19H36O3	312.2660	86289805	-	-	-	-
metab_778	8.4356	313.2726	pos	(2E)-19-Hydroxynonadec-2-enoic acid	C19H36O3	312.2660	86289805	-	-	-	-
metab_982	7.5005	313.2727	pos	(2E)-19-Hydroxynonadec-2-enoic acid	C19H36O3	312.2660	86289805	Glycerolipids	Monoradylglycerols	HMDB0011564; LMGL01010009	MG(16:0/0:0/0:0)
metab_2960	8.8466	313.2727	pos	(2E)-19-Hydroxynonadec-2-enoic acid	C19H36O3	312.2660	86289805	-	-	-	-
metab_3121	9.8673	313.2727	pos	(2E)-19-Hydroxynonadec-2-enoic acid	C19H36O3	312.2660	86289805	-	-	-	-
metab_4133	8.0843	313.2727	pos	(2E)-19-Hydroxynonadec-2-enoic acid	C19H36O3	312.2660	86289805	-	-	-	-
metab_3178	10.3326	313.2728	pos	(2E)-19-Hydroxynonadec-2-enoic acid	C19H36O3	312.2660	86289805	-	-	-	-
metab_155	0.7801	314.0907	pos	(S)-5'-Deoxy-5'-(methylsulfinyl)adenosine	C11H15N5O4S	313.0845	13342874	5'-deoxyribonucleosides	-	HMDB0033662	(S)-5'-Deoxy-5'-(methylsulfinyl)adenosine
metab_13716	2.3855	314.1151	neg	Bestim	C16H19N3O5	333.1325	3038501	Carboxylic acids and derivatives	Amino acids, peptides, and analogues	HMDB0029160	gamma-Glutamyltryptophan
metab_4803	4.6096	314.1378	pos	2,5-Dihydroxy-4-(2-hydroxyphenyl)-5-(methylamino)-3-phenylpentanoic acid	C18H21NO5	331.1420	131838515	Stilbenes	-	HMDB0134817	2,5-dihydroxy-4-(2-hydroxyphenyl)-5-(methylamino)-3-phenylpentanoic acid
metab_5998	1.2585	314.1521	pos	(S)-3-Amino-4-oxo-4-(((R)-1-oxo-1-((2,2,4,4-tetramethylthietan-3-yl)amino)propan-2-yl)amino)butanoic acid	C14H25N3O4S	331.1566	64763	Carboxylic acids and derivatives	Amino acids, peptides, and analogues	HMDB0037324	Alitame
metab_2099	3.2274	314.2318	pos	Armepavine	C19H23NO3	313.1675	442169	-	-	-	-

metab_4672	5.2573	314.2680	pos	(+)-Prosopinine	C18H35NO3	313.2613	442654	-	-	-	-
metab_4626	5.5112	314.2680	pos	(+)-Prosopinine	C18H35NO3	313.2613	442654	-	-	-	-
metab_4908	4.0939	314.2680	pos	(+)-Prosopinine	C18H35NO3	313.2613	442654	-	-	-	-
metab_14877	0.5571	315.0715	neg	5-Hydroxy-2-[3,4,5-trihydroxy -6-(hydroxymethyl)oxan-2-yl] oxybenzoate	C13H16O9	316.0799	54726828	-	-	-	-
metab_8603	2.0202	315.0728	neg	5-Hydroxy-2-[3,4,5-trihydroxy -6-(hydroxymethyl)oxan-2-yl] oxybenzoate	C13H16O9	316.0799	54726828	-	-	-	-
metab_8603	2.0202	315.0728	neg	Isorhamnetin	C16H12O7	316.058	5281654	Flavonoids	Benzoic acids and derivatives	HMDB0002655	Isorhamnetin
metab_13799	2.2119	315.1089	neg	2-(3,4-Dihydroxyphenyl)-ethyl -O-beta-D-glucopyranoside	C14H20O8	316.1163	5316821	-	-	-	-
metab_8117	1.1796	315.1679	neg	geranyl beta-D-glucoside	C16H28O6	316.1887	10710515	-	-	-	-
metab_12753	5.1772	315.1819	neg	Geranyl beta-D-glucopyranoside	C16H28O6	316.1887	10710515	-	-	-	-
metab_13182	3.6479	315.1821	neg	Geranyl beta-D-glucopyranoside	C16H28O6	316.1887	10710515	Prenol lipids	Terpene glycosides	HMDB0060004	Menthol-glucoronide
metab_13182	3.6479	315.1821	neg	Menthol-glucoronide	C16H28O6	316.1886	124202108	Prenol lipids	Terpene glycosides	HMDB0060004	Menthol-glucoronide
metab_4298	7.2014	315.2308	pos	Progesterone	C21 H30 O2	314.2243	5994	-	-	-	-
metab_775	8.4074	315.2310	pos	Progesterone	C21 H30 O2	314.2243	5994	-	-	-	-
metab_2653	6.9035	315.2311	pos	Progesterone	C21 H30 O2	314.2243	5994	-	-	-	-
metab_6624	6.8887	315.2542	neg	9,10-Dihydroxystearic acid	C18H36O4	316.2614	89377	-	-	-	-
metab_13135	3.7658	316.1776	neg	Ile Ser Val	C14H27N3O5	317.1948	145456273	-	-	-	-
metab_8646	2.1311	316.1880	neg	Leu-Val-Ser	C14H27N3O5	317.1949	9858135	-	-	-	-
metab_8646	2.1311	316.1880	neg	Ile-Ser-Val	C14H27N3O5	317.1948	145456273	-	-	-	-

metab_2306	4.3371	316.2111	pos	H-LEU-LEU-ALA-OH	C15H29N3O4	315.2158	7020110	-	-	-	-
metab_5338	2.5782	316.2220	pos	Ala-Leu-Leu	C15H29N3O4	315.2157	129011284	-	-	-	-
metab_5338	2.5782	316.2220	pos	Ile-Ala-Ile	C15H29N3O4	315.2157	145456039	-	-	-	-
metab_5338	2.5782	316.2220	pos	Leu-Leu-Ala	C15H29N3O4	315.2158	7020110	-	-	-	-
metab_1755	1.9758	316.2221	pos	Ile-Ala-Ile	C15H29N3O4	315.2157	145456039	-	-	-	-
metab_1755	1.9758	316.2221	pos	Leu-Leu-Ala	C15H29N3O4	315.2158	7020110	-	-	-	-
metab_1755	1.9758	316.2221	pos	Ala Leu Leu	C15H29N3O4	315.2157	129011284	-	-	-	-
metab_986	7.0082	317.2078	pos	Isosteviol	C20H30O3	316.2012	99514	-	-	-	-
metab_4765	4.8053	317.2102	pos	ISOSTEVIOL	C20H30O3	316.2012	99514	-	-	-	-
metab_2266	4.0338	317.2103	pos	Isosteviol	C20H30O3	316.2012	99514	-	-	-	-
metab_8084	1.0784	318.1197	neg	H-TRP-ASP-OH	C15H17N3O5	319.1169	7019108	-	-	-	-
metab_4939	3.9876	318.1540	pos	Arginyl-Valine	C11H23N5O3	273.1801	6992654	Carboxylic acids and derivatives	Amino acids, peptides, and analogues	HMDB0028722	Arginyl-Valine
metab_5277	2.7804	318.1805	pos	Isoleucyl-Tryptophan	C17H23N3O3	317.1738	7019085	-	-	-	-
metab_5277	2.7804	318.1805	pos	Trp-Leu	C17H23N3O3	317.1739	6997510	-	-	-	-
metab_5344	2.5627	318.1806	pos	Isoleucyl-Tryptophan	C17H23N3O3	317.1738	7019085	-	-	-	-
metab_5344	2.5627	318.1806	pos	Trp-Leu	C17H23N3O3	317.1739	6997510	-	-	-	-
metab_32	0.5140	318.2015	pos	Ile-Ser-Val	C14H27N3O5	317.1948	145456273	-	-	-	-
metab_32	0.5140	318.2015	pos	Leu-Val-Ser	C14H27N3O5	317.1949	9858135	-	-	-	-
metab_5534	2.1444	318.2019	pos	Ile-Ser-Val	C14H27N3O5	317.1948	145456273	-	-	-	-
metab_5534	2.1444	318.2019	pos	Leu-Val-Ser	C14H27N3O5	317.1949	9858135	-	-	-	-
metab_1733	1.9168	318.2268	pos	ISOSTEVIOL	C20H30O3	316.2012	99514	-	-	-	-
metab_746	7.8532	318.2994	pos	Phytosphingosine	C18H39NO3	317.2927	122121	-	-	-	-
metab_2554	6.0410	318.2994	pos	Phytosphingosine	C18H39NO3	317.2927	122121	-	-	-	-
metab_14495	1.2521	319.0582	neg	Coptisine	C19H14NO4	320.0919	72322	-	-	-	-

metab_1350	0.7661	320.0917	pos	Decarine	C19H13NO4	319.0842	179640	-	-	-	-
metab_402	1.9918	320.1230	pos	Trp-Asp	C15H17N3O5	319.1169	7019108	-	-	-	-
metab_8506	1.8205	321.0623	neg	2-[[2-(3-Hydroxy-2-Oxo-1H-Indol-3-Yl)Acetyl]Amino]Butanedioate	C14H14N2O7	322.0803	25200421	-	-	-	-
metab_1920	2.5003	321.1073	pos	7-Methylguanosine	C11H16N5O5+	297.1073	135445750	Purine nucleosides	-	HMDB0005862; HMDB0001107	2-Methylguanosine;7-Methylguanosine
metab_10093	7.9612	321.2108	neg	(8)-Gingerol	C19H30O4	322.2180	168114	-	-	-	-
metab_961	8.4504	321.2415	pos	20-Hydroxyeicosatetraenoic acid	C20H32O3	320.2348	5283157	-	-	-	-
metab_2695	7.0816	321.2415	pos	20-Hydroxyeicosatetraenoic acid	C20H32O3	320.2348	5283157	-	-	-	-
metab_1115	0.8640	322.1055	pos	S-Methylglutathione	C11H19N3O6S	321.0998	115260	-	-	-	-
metab_1521	1.2446	322.1864	pos	Phe-arg	C15H23N5O3	321.1800	150903	-	-	-	-
metab_12536	6.1804	322.2027	neg	(-)-Jasmonoyl-L-isoleucine	C18H29NO4	323.2098	5497150	-	-	-	-
metab_9526	5.2100	322.2028	neg	(-)-Jasmonoyl-L-isoleucine	C18H29NO4	323.2098	5497150	-	-	-	-
metab_999	6.2978	322.2731	pos	alpha-Linolenoyl ethanolamide	C20 H35 N O2	321.2665	5283449	-	-	-	-
metab_14834	0.5991	323.0288	neg	Uridine-5'-monophosphate	C9H13N2O9P	324.0359	6030	-	-	-	-
metab_13931	1.9904	323.1252	neg	1-Iodotetradecane	C14H29I	324.1319	29507	-	-	-	-
metab_8403	1.6335	323.1255	neg	1-Iodotetradecane	C14H29I	324.1319	29507	-	-	-	-
metab_4869	4.3371	323.1381	pos	3,6-Dihydroxy-4,5-diphenyl-2,3,4,5-tetrahydropyridin-2-one	C17H15NO3	281.1052	131838498	Stilbenes	-	HMDB0134800	3,6-dihydroxy-4,5-diphenyl-2,3,4,5-tetrahydropyridin-2-one
metab_6955	4.3882	323.1868	neg	Dinor-PGE2	C18H28O5	324.1936	52921876	-	-	-	-
metab_12731	5.2741	323.1868	neg	Dinor-PGE2	C18H28O5	324.1936	52921876	-	-	-	-

metab_9601	5.6452	323.1869	neg	Dinor-PGE2	C18H28O5	324.1936	52921876	-	-	-	-
metab_12530	6.2129	323.1870	neg	Dinor-PGE2	C18H28O5	324.1936	52921876	-	-	-	-
metab_9946	7.3659	323.2230	neg	Dihydromonacolin L acid	C19H32O4	324.2298	86289733	-	-	-	-
metab_12581	5.9705	323.2231	neg	Dihydromonacolin L acid	C19H32O4	324.2298	86289733	-	-	-	-
metab_9629	5.7751	323.2232	neg	Dihydromonacolin L acid	C19H32O4	324.2298	86289733	-	-	-	-
metab_10007	7.6323	323.2594	neg	13-(3-Pentyloxiran-2-yl)tridec-5-enoic acid	C20H36O3	324.2665	53394468	-	-	-	-
metab_10304	8.7031	323.2597	neg	13-(3-Pentyloxiran-2-yl)tridec-5-enoic acid	C20H36O3	324.2665	53394468	-	-	-	-
metab_2525	5.8148	323.2684	pos	N-(4-Aminobutyl)-3-aminopropionic acid	C7H17N2O2+	161.1290	161506	Carboxylic acids and derivatives	Amino acids, peptides, and analogues	HMDB0006078	Putreanine
metab_5976	1.3013	324.1180	pos	Rutacridone epoxide	C19H17NO4	323.1155	5281850	Quinolines and derivatives	Benzoquinolines	HMDB0033853	Rutacridon-epoxide
metab_4848	4.3818	324.2524	pos	Linoleamide MEA	C20H37NO2	323.2822	5283446	Organonitrogen compounds	Phosphate esters	HMDB0012252	Linoleamide mea
metab_2634	6.8280	324.2886	pos	Linoleoyl ethanolamide	C20H37NO2	323.2822	5283446	-	-	-	-
metab_2602	6.5246	324.2887	pos	Linoleoyl ethanolamide	C20H37NO2	323.2822	5283446	-	-	-	-
metab_4352	6.9935	324.2887	pos	Linoleoyl ethanolamide	C20H37NO2	323.2822	5283446	-	-	-	-
metab_105	8.0267	324.2887	pos	Linoleoyl ethanolamide	C20H37NO2	323.2822	5283446	-	-	-	-
metab_13729	2.3533	325.0934	neg	6-O-p-Coumaroyl-D-glucose	C15H18O8	326.1002	13915664	Cinnamic acids and derivatives	Hydroxycinnamic acids and derivatives	HMDB0039169	6-O-p-Coumaroyl-D-glucose
metab_7922	0.5991	325.1144	neg	Scillabiose	C12H22O10	326.1215	20056675	-	-	-	-
metab_4929	4.0338	325.1176	pos	Tetradecyl iodide	C14H29I	324.1319	29507	-	-	-	-
metab_1093	1.2016	325.1384	pos	1-Iodotetradecane	C14H29I	324.1319	29507	-	-	-	-
metab_1554	1.3720	325.1386	pos	1-Iodotetradecane	C14H29I	324.1319	29507	-	-	-	-

metab_8680	2.2119	325.1772	neg	CHEBI:59965	C15H26N4O4	326.1941	46878529	-	-	-	-
metab_4713	4.9856	325.1994	pos	Dinor-PGE2	C18H28O5	324.1936	52921876	-	-	-	-
metab_5099	3.3337	325.2000	pos	Dinor-PGE2	C18H28O5	324.1936	52921876	-	-	-	-
metab_4948	3.9421	325.2000	pos	Dinor-PGE2	C18H28O5	324.1936	52921876	-	-	-	-
metab_12661	5.6615	325.2025	neg	2,3-Dinor-8-epi-prostaglandin F2alpha	C18H30O5	326.2091	9548881	-	-	-	-
metab_7716	3.4800	325.2025	neg	2,3-Dinor-8-epi-prostaglandin F2alpha	C18H30O5	326.2091	9548881	-	-	-	-
metab_2582	6.2978	325.2366	pos	6-Decylubiquinol	C19H32O4	324.2298	11954189	-	-	-	-
metab_2582	6.2978	325.2366	pos	Dihydromonacolin L acid	C19H32O4	324.2298	86289733	-	-	-	-
metab_9990	7.5702	325.2386	neg	6Z,9Z-Octadecadienoic acid	C18H32O2	280.2402	5312483	Fatty Acyls	Lineolic acids and derivatives	HMDB0062238; LMFA01030332	6Z,9Z-octadecadienoic acid
metab_4610	5.6168	326.2316	pos	Bisoprolol	C18H31NO4	325.2253	2405	-	-	-	-
metab_9780	6.6320	326.2343	neg	10-nitro-9E-octadecenoic acid	C18H33NO4	327.2407	24836820	Fatty Acyls	Fatty acids and conjugates	HMDB0062737	(e)-10-Nitrooctadec-9-enoic acid
metab_2904	8.5241	326.3042	pos	n-Oleoylethanolamine	C20H39NO2	325.2980	5283454	-	-	-	-
metab_1656	1.6413	327.2001	pos	Glyoxal-lysine dimer	C15H26N4O4	326.1941	46878529	-	-	-	-
metab_3353	14.5016	327.2003	pos	Glyoxal-lysine dimer	C15H26N4O4	326.1941	46878529	-	-	-	-
metab_12531	6.2129	327.2179	neg	Corchorifatty acid F	C18H32O5	328.2250	44559173	Fatty Acyls	Lineolic acids and derivatives	HMDB0035919	Corchorifatty acid F
metab_12531	6.2129	327.2179	neg	2,3-Dinor-8-epi-prostaglandin F1alpha	C18H32O5	328.2251	9548882	Fatty Acyls	Lineolic acids and derivatives	HMDB0035919	Corchorifatty acid F
metab_12702	5.4684	327.2181	neg	2,3-Dinor-8-epi-prostaglandin F1alpha	C18H32O5	328.2251	9548882	-	-	-	-
metab_12764	5.1296	327.2182	neg	2,3-Dinor-8-epi-prostaglandin F1alpha	C18H32O5	328.2251	9548882	-	-	-	-

metab_12417	6.6632	327.2544	neg	2-[(9Z)-Hexadecenoyl]glycerol	C19H36O4	328.2616	53480960	-	-	-	-
metab_9662	5.9866	327.2544	neg	2-[(9Z)-Hexadecenoyl]glycerol	C19H36O4	328.2616	53480960	-	-	-	-
metab_11548	9.1522	327.2545	neg	Avocadene 1-acetate	C19H36O4	328.2614	3624980	Fatty Acyls	Fatty alcohols	HMDB0031043; LMFA05000640	Avocadene 1-acetate
metab_11548	9.1522	327.2545	neg	2-[(9Z)-Hexadecenoyl]glycerol	C19H36O4	328.2616	53480960	Fatty Acyls	Fatty alcohols	HMDB0031043; LMFA05000640	Avocadene 1-acetate
metab_11440	9.5478	327.2908	neg	12-Hydroxyicosanoic acid	C20H40O3	328.2977	115074	Fatty Acyls	Fatty acids and conjugates	HMDB0061664	12-hydroxyicosanoic acid
metab_11440	9.5478	327.2908	neg	(2S)-2-Hydroxyphytanic acid	C20H40O3	328.2980	6326763	Fatty Acyls	Fatty acids and conjugates	HMDB0061664	12-hydroxyicosanoic acid
metab_1209	0.4855	328.1195	pos	Terrestriamide	C18H17NO5	327.1105	5321824	-	-	-	-
metab_13304	3.2972	328.1195	neg	p-Coumaroyltyramine	C17H17NO3	283.1208	5372945	Cinnamic acids and derivatives	Hydroxycinnamic acids and derivatives	HMDB0039521	N-(p-Hydroxyphenyl)ethyl p-hydroxycinnamide
metab_2186	3.6229	328.2472	pos	10-nitro-9E-octadecenoic acid	C18H33NO4	327.2407	24836820	Fatty Acyls	Fatty acids and conjugates	HMDB0062737	(e)-10-Nitrooctadec-9-enoic acid
metab_4946	3.9421	328.2475	pos	10-Nitro-9E-octadecenoic acid	C18H33NO4	327.2407	24836820	-	-	-	-
metab_2991	9.0397	328.3200	pos	N,N-Dimethylsphingosine	C20H41NO2	327.3135	5282309	-	-	-	-
metab_4167	7.9695	328.3200	pos	N,N-Dimethylsphingosine	C20H41NO2	327.3135	5282309	-	-	-	-
metab_5726	1.7554	329.1485	pos	Tyrosylphenylalanine	C18H20N2O4	328.1421	3421919	-	-	-	-
metab_5588	2.0524	329.1489	pos	Tyrosylphenylalanine	C18H20N2O4	328.1421	3421919	-	-	-	-
metab_4882	4.2457	329.2311	pos	2,3-Dinor-8-epi-prostaglandin F1alpha	C18H32O5	328.2246	9548882	-	-	-	-
metab_4590	5.7232	329.2313	pos	2,3-Dinor-8-epi-prostaglandin F1alpha	C18H32O5	328.2246	9548882	-	-	-	-

metab_12809	4.9349	329.2338	neg	9,10,13-Trihydroxy-11-octadecenoic acid	C18H34O5	330.2408	5282965	Fatty Acyls	Fatty acids and conjugates	HMDB0004710	9,10,13-TriHOME
metab_12809	4.9349	329.2338	neg	Pinellic acid	C18H34O5	330.2408	9858729	Fatty Acyls	Fatty acids and conjugates	HMDB0004710	9,10,13-TriHOME
metab_13584	2.6357	330.2039	neg	Ser-Ile-Leu	C15H29N3O5	331.2106	44230708	-	-	-	-
metab_2086	3.1957	330.2375	pos	Val Val Leu	C16H31N3O4	329.2314	44230570	-	-	-	-
metab_5449	2.3133	330.2375	pos	Val-Val-Leu	C16H31N3O4	329.2314	44230570	-	-	-	-
metab_2452	5.3014	330.2628	pos	3-[(4,8-Dimethylnonanoyl)oxy]-4-(trimethylazaniumyl)butanoate	C18H35NO4	329.2562	53477801	-	-	-	-
metab_4553	5.9361	330.2629	pos	3-[(4,8-Dimethylnonanoyl)oxy]-4-(trimethylazaniumyl)butanoate	C18H35NO4	329.2562	53477801	-	-	-	-
metab_4866	4.3371	330.2630	pos	3-[(4,8-Dimethylnonanoyl)oxy]-4-(trimethylazaniumyl)butanoate	C18H35NO4	329.2562	53477801	-	-	-	-
metab_2843	8.2143	330.2992	pos	Dihydroceramide	C19H39NO3	329.2930	16755624	Carboxylic acids and derivatives	Carboxylic acid derivatives	LMSP02020000; HMDB0006752	Dihydroceramide
metab_9408	4.6056	331.1556	neg	(2S,3S)-2-Hydroxytridecane-1,2,3-tricarboxylate	C16H28O7	332.1849	5458468	-	-	-	-
metab_4431	6.5857	331.1871	pos	Carnosic acid	C20H28O4	330.1804	65126	-	-	-	-
metab_4748	4.8961	331.1895	pos	Carnosic acid	C20H28O4	330.1804	65126	-	-	-	-
metab_4846	4.3972	331.1896	pos	Carnosic acid	C20H28O4	330.1804	65126	-	-	-	-
metab_1632	1.5705	331.1965	pos	(2S,3S)-2-Hydroxytridecane-1,2,3-tricarboxylate	C16H28O7	332.1849	5458468	-	-	-	-
metab_10113	8.0228	331.2283	neg	8,13-Abietadien-18-al	C20H30O	286.2297	14241162	Prenol lipids	Diterpenoids	HMDB0038709	8,13-Abietadien-18-al

metab_2514	5.7385	331.2470	pos	5,8,12-Trihydroxy-9-octadecenoic acid	C18H34O5	330.2406	5312877	Fatty Acyls	Fatty acids and conjugates	HMDB0030936; LMFA01050543; LMFA02000221	5,8,12-Trihydroxy-9-octadecenoic acid
metab_12740	5.2419	331.2492	neg	9,10,13-Trihydroxystearic acid	C18H36O5	332.2563	45359277	Fatty Acyls	Fatty acids and conjugates	HMDB0030935	9,10,13-Trihydroxystearic acid
metab_13486	2.8440	333.0444	neg	a-L-Threo-4-Hex-4-enopyranuronosyl-D-galacturonic acid	C12H16O12	352.0642	3390357	Organooxygen compounds	Carbohydrates and carbohydrate conjugates	HMDB0039720	a-L-threo-4-Hex-4-enopyranuronosyl-D-galacturonic acid
metab_14523	1.2236	333.0833	neg	Glucosylisomaltol	C12H16O8	288.0845	9835588	Organooxygen compounds	Carbohydrates and carbohydrate conjugates	HMDB0038341	Glucosylisomaltol
metab_6423	0.5420	333.1047	pos	Tosyl-L-lysine chloromethyl ketone	C14H21ClN2O3S	332.0958	73094	-	-	-	-
metab_5697	1.7993	333.1545	pos	Trp-Ala-Gly	C16H20N4O4	332.1483	11990218	-	-	-	-
metab_9406	4.5884	333.1825	neg	(3R)-10-[(2R,3R,5R,6S)-3,5-dihydroxy-6-methyloxan-2-yl]oxy-3-hydroxydecanoic acid	C16H30O7	334.1994	86289846	-	-	-	-
metab_13094	3.8838	333.1826	neg	(3R)-10-[(2R,3R,5R,6S)-3,5-dihydroxy-6-methyloxan-2-yl]oxy-3-hydroxydecanoic acid	C16H30O7	334.1994	86289846	-	-	-	-
metab_4512	6.1167	333.2029	pos	15-Keto-13,14-dihydroprostaglandin A2	C20H30O4	332.1962	5283042	-	-	-	-
metab_4512	6.1167	333.2029	pos	Prostaglandin B2	C20H30O4	332.1961	5280881	-	-	-	-
metab_4148	8.0419	333.2408	pos	21-Hydroxypregnenolone	C21H32O3	332.2349	247020	-	-	-	-
metab_2268	4.0338	333.2415	pos	21-Hydroxypregnenolone	C21H32O3	332.2349	247020	Steroids and steroid derivatives	Lineolic acids and derivatives	HMDB0004026	5-Pregnen-3b,21-diol-20-one
metab_401	1.9918	334.1385	pos	Oglufanide	C16H19N3O5	333.1324	100094	-	-	-	-
metab_401	1.9918	334.1385	pos	Trp-glu	C16H19N3O5	333.1323	7009663	-	-	-	-

metab_8569	1.9745	334.1415	neg	Hydroxylated N-acetyl desmethyl frovatriptan	C15H19N3O3	289.1426	131770052	Indoles and derivatives	Carbazoles	HMDB0061151	Hydroxylated N-acetyl desmethyl frovatriptan
metab_13032	4.1023	334.1665	neg	Hyoscyamine	C17H23NO3	289.1678	154417	Tropane alkaloids	-	HMDB0014568	Hyoscyamine
metab_5583	2.0524	334.2212	pos	CARBETAPENTANE	C20H31NO3	333.2276	2562	-	-	-	-
metab_4304	7.1701	334.2368	pos	Carbetapentane	C20H31NO3	333.2276	2562	-	-	-	-
metab_2680	7.0228	335.1505	pos	Pimobendan	C19H18N4O2	334.1440	4823	-	-	-	-
metab_4805	4.5944	335.1514	pos	Pimobendan	C19H18N4O2	334.1440	4823	-	-	-	-
metab_12777	5.0484	335.1539	neg	Smardaesidin G	C19H28O5	336.1933	56599466	-	-	-	-
metab_994	6.3885	335.2187	pos	[1-(1,1,2,2,3,3,3-Heptadeuteriopropyl)-2-methylindol-3-yl]-naphthalen-1-ylmethanone	C23H14D7NO	334.2117	53394756	-	-	-	-
metab_4551	5.9361	335.2194	pos	[1-(1,1,2,2,3,3,3-Heptadeuteriopropyl)-2-methylindol-3-yl]-naphthalen-1-ylmethanone	C23H14D7NO	334.2117	53394756	-	-	LMFA01170034	
metab_4767	4.7758	335.2207	pos	JWH 015-d7	C23H14D7NO	334.2117	53394756	-	-	-	-
metab_809	8.9367	335.2570	pos	3alpha,6alpha-Dihydroxy-5beta-pregnan-20-one	C21H34O3	334.2506	101774	-	-	-	-
metab_809	8.9367	335.2570	pos	Tetrahydrodeoxycorticosterone	C21H34O3	334.2506	101771	-	-	-	-
metab_1551	1.3437	336.1541	pos	Isopentenyl adenosine	C15H21N5O4	335.1591	24405	-	-	-	-
metab_1982	2.7804	336.1656	pos	Riboprine	C15H21N5O4	335.1591	24405	-	-	-	-
metab_8296	1.4710	336.1668	neg	Kyotorphin	C15H23N5O4	337.1735	123804	Carboxylic acids and derivatives	Hydroxysteroids	HMDB0005768	L-Tyrosyl-L-arginine
metab_9977	7.5239	337.2058	neg	Idebenone	C19H30O5	338.2130	3686	-	-	-	-
metab_2225	3.7908	337.2151	pos	Smardaesidin G	C19H28O5	336.1933	56599466	-	-	-	-

metab_2	6.4035	337.2342	pos	(12Z)-9,10-Dihydroxyoctadec-12-enoic acid	C18H34O4	314.2457	9966640	Fatty Acyls	Fatty acids and conjugates	HMDB0004704	9,10-DHOME
metab_1908	2.4370	337.2365	pos	Smardaesidin G	C19H28O5	336.1933	56599466	-	-	-	-
metab_12453	6.5033	337.2385	neg	5,6-Dihydroxy-8Z,11Z,14Z-eicosatrienoic acid	C20H34O4	338.2461	5283142	-	-	-	-
metab_12280	7.1111	337.2387	neg	5,6-Dihydroxy-8Z,11Z,14Z-eicosatrienoic acid	C20H34O4	338.2461	5283142	-	-	-	-
metab_12673	5.6128	337.2388	neg	5,6-Dihydroxy-8Z,11Z,14Z-eicosatrienoic acid	C20H34O4	338.2461	5283142	-	-	-	-
metab_9645	5.9217	337.2388	neg	5,6-Dihydroxy-8Z,11Z,14Z-eicosatrienoic acid	C20H34O4	338.2461	5283142	-	-	-	-
metab_12035	7.9137	337.2389	neg	5,6-Dihydroxy-8Z,11Z,14Z-eicosatrienoic acid	C20H34O4	338.2461	5283142	-	-	-	-
metab_10274	8.6239	337.2391	neg	5,6-Dihydroxy-8Z,11Z,14Z-eicosatrienoic acid	C20H34O4	338.2461	5283142	-	-	-	-
metab_4242	7.5450	337.2727	pos	2alpha-(Hydroxymethyl)-17-methyl-5alpha-androstane-3beta,17beta-diol	C21H36O3	336.2663	243356	Fatty Acyls	Fatty acids and conjugates	HMDB0002177	Cis-8,11,14,17-Eicosatetraenoic acid
metab_85	6.9336	337.2728	pos	2alpha-(Hydroxymethyl)-17-methyl-5alpha-androstane-3beta,17beta-diol	C21H36O3	336.2663	243356	-	-	-	-
metab_2542	5.9504	337.2838	pos	1-Eicosanol	C20H42O	298.3236	12404	Fatty Acyls	Fatty alcohols	HMDB0011619	Arachidyl alcohol
metab_13813	2.1956	338.0887	neg	2-Methoxyacetaminophen glucuronide	C15H19NO9	357.1060	121596203	Organooxygen compounds	Carbohydrates and carbohydrate conjugates	HMDB0240215	2-Methoxyacetaminophen glucuronide
metab_319	1.4429	338.1797	pos	Kyotorphin	C15H23N5O4	337.1735	123804	-	-	-	-
metab_1001	6.2522	338.2676	pos	N-Oleoylglycine	C20H37NO3	337.2588	6436908	-	-	-	-

metab_12045	7.8822	338.2705	neg	N-Oleoyl Glycine	C20H37NO3	339.2746	6436908	Carboxylic acids and derivatives	Methoxyphenols	HMDB0013631	N-(9Z-Octadecenoyl)-glycine
metab_3256	13.4283	338.3404	pos	6-cis-Docosenamide	C22H43NO	337.3342	44584605	-	-	-	-
metab_4057	8.3763	338.3407	pos	6-cis-Docosenamide	C22H43NO	337.3342	44584605	-	-	-	-
metab_844	9.5438	338.3408	pos	6-cis-Docosenamide	C22H43NO	337.3342	44584605	-	-	-	-
metab_2964	8.8759	338.3408	pos	6-cis-Docosenamide	C22H43NO	337.3342	44584605	-	-	-	-
metab_5821	1.5705	339.1180	pos	Demethoxycurcumin	C20H18O5	338.115	5469424	-	-	-	-
metab_5027	3.6073	339.2159	pos	Monacolin J acid	C19H30O5	338.2091	9840816	-	-	-	-
metab_12380	6.8255	339.2180	neg	3alpha-Hydroxy-3,5-dihydromonacolin L acid	C19H32O5	340.2251	195046	-	-	-	-
metab_12836	4.8210	339.2180	neg	3alpha-Hydroxy-3,5-dihydromonacolin L acid	C19H32O5	340.2251	195046	Prenol lipids	Sesquiterpenoids	HMDB0036832	Sterebin D
metab_12836	4.8210	339.2180	neg	Sterebin D	C18H30O3	294.2195	14396288	Prenol lipids	Sesquiterpenoids	HMDB0036832	Sterebin D
metab_12307	7.0162	339.2182	neg	3alpha-Hydroxy-3,5-dihydromonacolin L acid	C19H32O5	340.2251	195046	-	-	-	-
metab_19	6.8886	339.2503	pos	(+)-15,16-Dihydroxyoctadecanoic acid	C18H36O4	316.2614	5312766	Fatty Acyls	Fatty acids and conjugates	HMDB0031008	(+)-15,16-Dihydroxyoctadecanoic acid
metab_2741	7.4119	339.2883	pos	Glycidyl oleate	C21H38O3	338.2820	5354568	-	-	-	-
metab_4195	7.7946	339.2883	pos	Glycidyl oleate	C21H38O3	338.2820	5354568	-	-	-	-
metab_4496	6.2522	339.2883	pos	Glycidyl oleate	C21H38O3	338.2820	5354568	-	-	-	-
metab_4141	8.0700	339.2884	pos	Glycidyl oleate	C21H38O3	338.2820	5354568	Fatty Acyls	Fatty acids and conjugates	HMDB0060039; LMFA01030159	11,14,17-Eicosatrienoic acid
metab_4470	6.4035	339.2884	pos	Glycidyl oleate	C21H38O3	338.2820	5354568	-	-	-	-
metab_2445	5.2431	340.2473	pos	Tumonoic acid A	C19H33NO4	339.2408	10854587	-	-	-	-
metab_4661	5.3014	340.2833	pos	N-Oleoylglycine	C20H37NO3	339.2746	6436908	-	-	-	-

metab_695	6.5246	340.2837	pos	N-Oleoylglycine	C20H37NO3	339.2746	6436908	-	-	-	-
metab_4747	4.8960	340.2837	pos	N-Oleoyl Glycine	C20H37NO3	339.2746	6436908	Carboxylic acids and derivatives	Methoxyphenols	HMDB0013631	N-(9Z-Octadecenoyl)-glycine
metab_7335	8.4505	340.2861	neg	N-Acetylsphingosine	C20H39NO3	341.2927	5497136	-	-	-	-
metab_13092	3.9005	341.1609	neg	3,8-Dihydroxy-6-methoxy-7(11)-eremophilen-12,8-olide	C16H24O5	296.1624	85265509	Prenol lipids	Terpene lactones	HMDB0041551	3,8-Dihydroxy-6-methoxy-7(11)-eremophilen-12,8-olide
metab_13363	3.1286	341.1721	neg	N-[(2S)-3-Hydroxy-1-[[1-[2-(hydroxymethyl)oxiran-2-yl]-4-methyl-1-oxopentan-2-yl]amino]-1-oxobutan-2-yl]-3-methylbutanamide	C16H26N2O6	342.1788	9798121	-	-	-	-
metab_13586	2.6357	341.1957	neg	2,3-Dinor-6-oxoprostaglandin F1alpha	C18H30O6	342.2045	5283084	-	-	-	-
metab_9624	5.7420	341.1971	neg	2,3-Dinor-6-oxoprostaglandin F1alpha	C18H30O6	342.2045	5283084	-	-	-	-
metab_9250	3.9005	341.1972	neg	2,3-Dinor-6-oxoprostaglandin F1alpha	C18H30O6	342.2045	5283084	-	-	-	-
metab_7709	3.3803	341.1973	neg	2,3-Dinor-6-oxoprostaglandin F1alpha	C18H30O6	342.2045	5283084	-	-	-	-
metab_9445	4.7546	341.1974	neg	2,3-Dinor-6-oxoprostaglandin F1alpha	C18H30O6	342.2045	5283084	-	-	-	-
metab_12941	4.4055	341.1975	neg	2,3-Dinor-6-oxoprostaglandin F1alpha	C18H30O6	342.2045	5283084	-	-	-	-
metab_5135	3.2116	341.2425	pos	3alpha-Hydroxy-3,5-dihydromonacolin L acid	C19H32O5	340.2251	195046	-	-	-	-
metab_4380	6.8886	341.2559	pos	Dimethisterone	C23H32O2	340.2403	6607	-	-	-	-

metab_9866	7.0322	341.2697	neg	cis-10-Nonadecenoic acid	C19H36O2	296.2715	5312513	Fatty Acyls	Fatty acids and conjugates	HMDB0013622; LMFA01030362	10Z-Nonadecenoic acid
metab_10544	9.7312	341.3064	neg	Selachyl alcohol	C21H42O3	342.3136	5282282	-	-	-	-
metab_8548	1.9280	342.1199	neg	De-O-methylsimmondsin	C15H23NO9	361.1373	5375609	Organooxygen compounds	Carbohydrates and carbohydrate conjugates	HMDB0041208	De-O-methylsimmondsin
metab_5341	2.5627	342.2377	pos	Pro-Leu-Ile	C17H31N3O4	341.2315	145457506	-	-	-	-
metab_5764	1.6838	342.2379	pos	Pro-Leu-Ile	C17H31N3O4	341.2315	145457506	-	-	-	-
metab_11897	8.3055	342.2653	neg	Palmitoleoyl Ethanolamide	C18H35NO2	297.2668	9835868	Organonitrogen compounds	Amines	HMDB0013648	Palmitoleoyl Ethanolamide
metab_10227	8.4666	342.2926	neg	C2 Dihydroceramide	C20H41NO3	343.3083	6610273	-	-	-	-
metab_4170	7.9551	342.2992	pos	N-Acetylsphingosine	C20H39NO3	341.2926	5497136	-	-	-	-
metab_4266	7.3824	342.2992	pos	N-Acetylsphingosine	C20H39NO3	341.2926	5497136	-	-	-	-
metab_4613	5.5864	342.2994	pos	N-Acetylsphingosine	C20H39NO3	341.2926	5497136	-	-	-	-
metab_4641	5.3913	342.2994	pos	N-acetylsphingosine	C20H39NO3	341.2926	5497136	-	-	-	-
metab_782	8.4504	342.2994	pos	N-Acetylsphingosine	C20H39NO3	341.2926	5497136	-	-	-	-
metab_9365	4.3882	343.1555	neg	[(10E)-7,9-Dihydroxy-10-methyl-3,6-dimethylidene-2-oxo-4,5,7,8,9,11a-hexahydro-3aH-cyclodeca[b]furan-4-yl] (E)-2-methylbut-2-enoate	C20H26O6	362.1729	6438562	Prenol lipids	Terpene lactones	HMDB0034475	Hydroxyisonobilin
metab_8385	1.5885	343.1989	neg	Asn Val Ile	C15H28N4O5	344.2058	145454322	-	-	-	-
metab_12550	6.1320	343.2129	neg	11R-(3'R,5'R-Dihydroxy-6'S-methyl-(2H)-tetrahydropyran-2'-yloxy)-2E-dodecenoic acid	C18H32O6	344.2201	86289690	-	-	-	-
metab_8839	2.6046	343.2130	neg	(E,11R)-11-[(2R,3R,5R,6S)-3,5-dihydroxy-6-methyloxan-2-y	C18H32O6	344.2201	86289690	-	-	-	-

				l]oxydodec-2-enoic acid							
metab_13045	4.0685	343.2130	neg	11R-(3'R,5'R-Dihydroxy-6'S-methyl-(2H)-tetrahydropyran-2'-yloxy)-2E-dodecenoic acid	C18H32O6	344.2201	86289690	-	-	-	-
metab_12495	6.3758	343.2132	neg	(E,11R)-11-[(2R,3R,5R,6S)-3,5-dihydroxy-6-methyloxan-2-yl]oxydodec-2-enoic acid	C18H32O6	344.2201	86289690	-	-	-	-
metab_12687	5.5322	343.2491	neg	Tianshic acid methyl ester	C19H36O5	344.2564	11702937	-	-	-	-
metab_7322	8.7031	343.2495	neg	Tianshic acid methyl ester	C19H36O5	344.2564	11702937	-	-	-	-
metab_12184	7.4776	343.2857	neg	10,20-Dihydroxyeicosanoic acid	C20H40O4	344.2927	14259007	Fatty Acyls	Fatty acids and conjugates	HMDB0031923	10,20-Dihydroxyeicosanoic acid
metab_11577	9.1038	343.2858	neg	Methyl stearate	C19H38O2	298.2872	8201	Fatty Acyls	Fatty acid esters	HMDB0034154	Methyl stearate
metab_13951	1.9439	344.1829	neg	Glu-Val-Val	C15H27N3O6	345.1899	71464666	-	-	-	-
metab_14122	1.6642	344.1830	neg	Glu-Val-Val	C15H27N3O6	345.1899	71464666	-	-	-	-
metab_14268	1.5155	344.1832	neg	Glu-Val-Val	C15H27N3O6	345.1899	71464666	-	-	-	-
metab_1984	2.7959	344.2534	pos	Ile-Val-Ile	C17H33N3O4	343.2470	53765802	-	-	-	-
metab_4550	5.9361	344.2786	pos	Lauroylcarnitine	C19H37NO4	343.2721	168381	-	-	-	-
metab_4442	6.5395	344.2786	pos	Lauroylcarnitine	C19H37NO4	343.2721	168381	-	-	-	-
metab_4420	6.6760	344.3150	pos	N-Acetylsphinganine	C20H41NO3	343.3084	6610273	-	-	-	-
metab_4055	8.3912	344.3150	pos	N-Acetylsphinganine	C20H41NO3	343.3084	6610273	-	-	-	-
metab_2930	8.6434	344.3152	pos	N-Acetylsphinganine	C20H41NO3	343.3084	6610273	-	-	-	-
metab_8868	2.6986	345.1670	neg	Cibaric acid	C18H28O5	324.1937	6438613	Lineolic acids and derivatives	-	LMFA02000289; HMDB0038580	Cibaric acid
metab_9495	5.0159	345.1709	neg	Blumealactone A	C20H28O6	364.1886	14021255	Prenol lipids	Terpene lactones	HMDB0036665	Blumealactone A
metab_9354	4.3380	345.1712	neg	Gibberellin A74	C20H28O6	364.1886	131752448	-	-	HMDB0038746	Gibberellin A74

metab_1641	1.5987	345.2122	pos	13,14-dehydro-15-cyclohexyl Carbaprostacyclin	C21H30O4	344.1961	10088987	-	-	-	-
metab_5920	1.4005	345.2122	pos	13,14-dehydro-15-cyclohexyl Carbaprostacyclin	C21H30O4	344.1961	10088987	-	-	-	-
metab_5920	1.4005	345.2122	pos	Asn-Val-Ile	C15H28N4O5	344.2058	145454322	-	-	-	-
metab_1641	1.5987	345.2122	pos	Asn-Val-Ile	C15H28N4O5	344.2058	145454322	-	-	-	-
metab_12999	4.2203	345.2286	neg	12-[(3,6-Dideoxy-alpha-L-arabino-hexopyranosyl)oxy]lauric acid	C18H34O6	346.2356	86289780	-	-	-	-
metab_9194	3.7317	345.2287	neg	12-[(3,6-Dideoxy-alpha-L-arabino-hexopyranosyl)oxy]lauric acid	C18H34O6	346.2356	86289780	Glycerolipids	Monoradylglycerols	HMDB0011531	MG(0:0/14:1(9Z)/0:0)
metab_1898	2.4224	346.1961	pos	Glu-Val-Val	C15H27N3O6	345.1899	71464666	-	-	-	-
metab_5681	1.8577	347.1227	pos	Miraxanthin-V	C17H18N2O6	346.1165	135438594	-	-	-	-
metab_601	4.0788	347.1844	pos	Ala-Glu-Lys	C14H26N4O6	346.1755	10337675	Prenol lipids	Terpene lactones	HMDB0036690	3-Epinobilin
metab_4944	3.9577	347.2209	pos	Cortexolone	C21H30O4	346.2117	440707	Steroids and steroid derivatives		HMDB0000015	Substance S, reichstein's
metab_2298	4.2750	348.2527	pos	O-Arachidonoyl Ethanolamine	C22H37NO2	347.2797	5712057	Fatty Acyls	Amino acids, peptides, and analogues	HMDB0013655	O-Arachidonoyl Ethanolamine
metab_13752	2.3078	349.0393	neg	{3-[(2E)-3-Phenylprop-2-enoyl]phenyl}oxidanesulfonic acid	C15H12O5S	304.0405	131839036	Linear 1,3-diarylpropanoids	Chalcones and dihydrochalcones	HMDB0135448	{3-[(2E)-3-phenylprop-2-enoyl]phenyl}oxidanesulfonic acid
metab_1710	1.7845	349.1748	pos	Schizonepetoside E	C16H28O8	348.1785	46173963	-	-	-	-
metab_4956	3.9114	349.2001	pos	17alpha,21-Dihydroxypregnenolone	C21H32O4	348.2296	192735	Steroids and steroid derivatives	Androstane steroids	HMDB0006762	3b,17a,21-Trihydroxypregn-5-en-20-one
metab_9348	4.3037	349.2025	neg	11beta-Hydroxytestosterone	C19H28O3	304.2038	114920	Steroids and steroid derivatives	Androstane steroids	HMDB0012533; HMDB0060339	11beta-Hydroxytestosterone;11beta,17beta-Dihydroxy-4-androsten-3-on

											e
metab_9166	3.6142	349.2025	neg	8-Iso-15-keto-PGE2	C20H30O5	350.2093	16061134	Fatty Acyls	Eicosanoids	LMFA03110009; HMDB0002341	8-iso-15-keto-PGE2
metab_4419	6.6909	349.2358	pos	17alpha,21-Dihydroxypregnenolone	C21H32O4	348.2298	192735	-	-	-	-
metab_4419	6.6909	349.2358	pos	5-trans U-44069	C21H34O4	348.2274	91746438	-	-	-	-
metab_715	7.1864	349.2363	pos	17alpha,21-Dihydroxypregnenolone	C21H32O4	348.2298	192735	-	-	-	-
metab_715	7.1864	349.2363	pos	5-trans U-44069	C21H34O4	348.2274	91746438	-	-	-	-
metab_2204	3.6987	349.2364	pos	17alpha,21-Dihydroxypregnenolone	C21H32O4	348.2298	192735	-	-	-	-
metab_2204	3.6987	349.2364	pos	5-trans U-44069	C21H34O4	348.2274	91746438	-	-	-	-
metab_5109	3.3032	349.2365	pos	17alpha,21-Dihydroxypregnenolone	C21H32O4	348.2298	192735	-	-	-	-
metab_5109	3.3032	349.2365	pos	5-trans U-44069	C21H34O4	348.2274	91746438	-	-	-	-
metab_9729	6.3759	350.2336	neg	3-[(4,8-Dimethylnonanoyl)oxy]-4-(trimethylazaniumyl)butanoate	C18H35NO4	329.2566	53477801	Fatty Acyls	Fatty acid esters	LMFA07070046; HMDB0006202	4,8 Dimethylnonanoyl carnitine
metab_1137	0.6123	351.0677	pos	Acetamide, N-(4-(((2,6-dimethoxy-4-pyrimidinyl)amino)sulfonyl)phenyl)-	C14H16N4O5S	350.0614	473359	-	-	-	-
metab_1451	1.0741	351.1109	pos	Fagaronine	C21H19NO4	350.139	40305	-	-	-	-
metab_8796	2.4970	351.1202	neg	Cotinine-glucuronide	C16H20N2O7	352.1274	3398121	-	-	-	-
metab_13773	2.2595	351.1202	neg	Cotinine-glucuronide	C16H20N2O7	352.1274	3398121	-	-	-	-
metab_14221	1.5439	351.1205	neg	Cotinine-glucuronide	C16H20N2O7	352.1274	3398121	-	-	-	-

metab_492	2.5158	351.1461	pos	Fagaronine	C21H19NO4	350.1390	40305	-	-	-	-
metab_5460	2.2824	351.1461	pos	Fagaronine	C21H19NO4	350.1390	40305	-	-	-	-
metab_13681	2.4487	351.1663	neg	1-[(2R,3R,4S,5S,6R)-3,4,5-Trihydroxy-6-(hydroxymethyl)oxan-2-yl]oxyoctan-3-one	C14H26O7	306.1679	129885032	Fatty Acyls	Fatty acyl glycosides	HMDB0031315	1-(beta-D-Glucopyranosyloxy)-3-octanone
metab_4629	5.4809	351.2134	pos	Tebufenozide	C22H28N2O2	350.2068	91773	-	-	-	-
metab_4692	5.1218	351.2136	pos	Tebufenozide	C22H28N2O2	350.2068	91773	-	-	-	-
metab_586	4.2907	351.2155	pos	5S-Hydroperoxy-18R-hydroxy-6E,8Z,11Z,14Z,16E-eicosapentaenoic acid	C20H30O5	350.2091	46174067	Fatty Acyls	Eicosanoids	HMDB0012503; LMFA03020044	10,11-dihydro-20-trihydroxy-leukotriene B4
metab_586	4.2907	351.2155	pos	Tebufenozide	C22H28N2O2	350.2068	91773	Fatty Acyls	Eicosanoids	HMDB0012503; LMFA03020044	10,11-dihydro-20-trihydroxy-leukotriene B4
metab_4392	6.8582	351.2157	pos	Tebufenozide	C22H28N2O2	350.2068	91773	-	-	-	-
metab_4392	6.8582	351.2157	pos	5S-Hydroperoxy-18R-HEPE	C20H30O5	350.2091	46174067	-	-	-	-
metab_2108	3.2573	351.2159	pos	5S-Hydroperoxy-18R-hydroxy-6E,8Z,11Z,14Z,16E-eicosapentaenoic acid	C20H30O5	350.2091	46174067	-	-	-	-
metab_2108	3.2573	351.2159	pos	Tebufenozide	C22H28N2O2	350.2068	91773	-	-	-	-
metab_692	6.5857	351.2518	pos	Tetrahydrocorticosterone	C21H34O4	350.2455	65553	Fatty Acyls	Eicosanoids	HMDB0014573	Carboprost Tromethamine
metab_692	6.5857	351.2518	pos	Tetrahydrodeoxycortisol	C21H34O4	350.2448	65555	Fatty Acyls	Eicosanoids	HMDB0014573	Carboprost Tromethamine
metab_2786	7.7514	351.2519	pos	Tetrahydrocorticosterone	C21H34O4	350.2455	65553	-	-	-	-
metab_2786	7.7514	351.2519	pos	Tetrahydrodeoxycortisol	C21H34O4	350.2448	65555	-	-	-	-
metab_4116	8.1560	351.2519	pos	Tetrahydrocorticosterone	C21H34O4	350.2455	65553	-	-	-	-
metab_4116	8.1560	351.2519	pos	Tetrahydrodeoxycortisol	C21H34O4	350.2448	65555	-	-	-	-
metab_599	4.5184	351.2521	pos	Tetrahydrocorticosterone	C21H34O4	350.2455	65553	-	-	-	-

metab_599	4.5184	351.2521	pos	Tetrahydrodeoxycortisol	C21H34O4	350.2448	65555	-	-	-	-
metab_11676	8.8128	351.2546	neg	Sagittariol	C20H34O2	306.2559	101603339	Prenol lipids	Diterpenoids	HMDB0036835	Sagittariol
metab_6048	1.1875	352.1490	pos	Palmatine	C21H21NO4	351.1466	19009	-	-	-	-
metab_1643	1.6272	352.1599	pos	Zeatin riboside	C15H21N5O5	351.1543	6440982	Purine nucleosides	-	HMDB0030388	9-(beta-D-Ribofuranosyl)zeatin
metab_5445	2.3292	352.1647	pos	Aniflorine	C20H21N3O3	351.1579	442881	-	-	-	-
metab_476	2.7029	352.1648	pos	Aniflorine	C20H21N3O3	351.1579	442881	-	-	-	-
metab_2016	2.9192	352.1649	pos	Aniflorine	C20H21N3O3	351.1579	442881	-	-	-	-
metab_11782	8.6406	352.2862	neg	Tetradecanoylcarnitine	C21H41NO4	371.3036	53477791	Fatty Acyls	Fatty acid esters	LMFA07070107; HMDB0005066	Tetradecanoylcarnitine
metab_4150	8.0419	352.3200	pos	N-cis-11,14-Eicosadienoyl ethanolamine	C22H41NO2	351.3134	5283444	-	-	-	-
metab_3952	8.7883	352.3200	pos	N-cis-11,14-Eicosadienoyl ethanolamine	C22H41NO2	351.3134	5283444	-	-	-	-
metab_4074	8.3018	352.3201	pos	N-cis-11,14-Eicosadienoyl ethanolamine	C22H41NO2	351.3134	5283444	-	-	-	-
metab_9939	7.3343	353.2166	neg	3,3-Difluoro-5alpha-androstan -17beta-yl acetate	C21H32F2O2	354.2384	243754	-	-	-	-
metab_1989	2.8108	353.2175	pos	Clidinium	C22H26NO3	352.1922	2784	-	-	-	-
metab_2855	8.2586	353.2677	pos	Montanol	C21H36O4	352.2612	11953925	-	-	-	-
metab_84	6.6001	353.2677	pos	Montanol	C21H36O4	352.2612	11953925	-	-	-	-
metab_3722	9.8362	353.2677	pos	Montanol	C21H36O4	352.2612	11953925	-	-	-	-
metab_2987	9.0112	353.2678	pos	Montanol	C21H36O4	352.2612	11953925	-	-	-	-
metab_11538	9.1858	353.2702	neg	Sclareol	C20H36O2	308.2715	163263	Prenol lipids	Diterpenoids	HMDB0036827; LMPR0104030010	Sclareol

metab_4768	4.7758	353.2787	pos	Montanol	C21H36O4	352.261	11953925	-	-	-	-
metab_14311	1.4710	354.0836	neg	Indoxyl glucuronide	C14H15NO7	309.0849	2733785	Organooxygen compounds	Carbohydrates and carbohydrate conjugates	HMDB0010319	Indoxyl glucuronide
metab_1505	1.2162	354.1472	pos	Methionyl-Tyrosine	C14H20N2O4S	312.1144	7009558	Carboxylic acids and derivatives	Amino acids, peptides, and analogues	HMDB0028985	Methionyl-Tyrosine
metab_2459	5.3608	354.2626	pos	Methyl tumonoate A	C20H35NO4	353.2563	10498274	-	-	-	-
metab_2396	4.9115	354.2628	pos	Methyl tumonoate A	C20H35NO4	353.2563	10498274	-	-	-	-
metab_5026	3.6073	354.2630	pos	Methyl tumonoate A	C20H35NO4	353.2563	10498274	-	-	-	-
metab_5143	3.1957	354.2630	pos	Methyl tumonoate A	C20H35NO4	353.2563	10498274	-	-	-	-
metab_12762	5.1296	355.2130	neg	Estradiol valerate	C23H32O3	356.2354	13791	-	-	-	-
metab_9158	3.5978	355.2132	neg	(E)-4-[(1R,2S,3S,4R,8As)-2,3,4-trihydroxy-2,5,5,8a-tetramethyl-3,4,4a,6,7,8-hexahydro-1H-naphthalen-1-yl]but-3-en-2-one	C18H30O4	310.2144	71694416	Prenol lipids	Sesquiterpenoids	HMDB0035337	Sterebin A
metab_2573	6.1927	355.2468	pos	Prostaglandin D1	C20H34O5	354.2403	5280936	-	-	-	-
metab_2599	6.4794	355.2468	pos	Prostaglandin D1	C20H34O5	354.2403	5280936	-	-	-	-
metab_689	6.7825	355.2829	pos	Methyl O-acetylricinoleate	C21H38O4	354.2767	5282107	-	-	-	-
metab_978	7.6185	355.2832	pos	Methyl O-acetylricinoleate	C21H38O4	354.2767	5282107	-	-	-	-
metab_10574	9.8947	355.3222	neg	22-Hydroxydocosanoate	C22H44O3	356.3293	17802799	-	-	-	-
metab_9266	3.9677	357.1926	neg	19-Hydroxy-2,3-dinor-6-oxoprostaglandin F1alpha	C18H30O7	358.1996	71581025	-	-	-	-
metab_5052	3.5162	357.2008	pos	CHEBI:73270	C16H28N4O5	356.2059	42641527	-	-	-	-
metab_13496	2.8123	358.1986	neg	Leu-Leu-Asp	C16H29N3O6	359.2061	49864613	-	-	-	-
metab_13496	2.8123	358.1986	neg	Glu-Ile-Val	C16H29N3O6	359.2056	71464664	-	-	-	-

metab_13496	2.8123	358.1986	neg	Val-Ile-Glu	C16H29N3O6	359.2056	145458950	-	-	-	-
metab_13789	2.2272	358.1986	neg	Leu-Leu-Asp	C16H29N3O6	359.2061	49864613	-	-	-	-
metab_13789	2.2272	358.1986	neg	Glu-Ile-Val	C16H29N3O6	359.2056	71464664	-	-	-	-
metab_13789	2.2272	358.1986	neg	Val-Ile-Glu	C16H29N3O6	359.2056	145458950	-	-	-	-
metab_2534	5.9048	358.2577	pos	(9Z)-3-hydroxydodecenoylcarnitine	C19H35NO5	357.2512	71464579	-	-	-	-
metab_5153	3.1648	358.2691	pos	Ile-Leu-Leu	C18H35N3O4	357.2624	49864303	-	-	-	-
metab_2538	5.9361	358.2942	pos	N-Palmitoyl threonine	C20H39NO4	357.2879	14239842	-	-	LMFA08020107	
metab_3028	9.2658	358.3671	pos	1-Decanol	C10H22O	158.1671	8174	Fatty Acyls	Fatty alcohols	HMDB0011624	Decyl alcohol
metab_8953	2.9092	359.1255	neg	Nicotine glucuronide	C16H22N2O6	338.1478	3035848	Organooxygen compounds	Carbohydrates and carbohydrate conjugates	HMDB0001272	Nicotine glucuronide
metab_9076	3.2972	359.1828	neg	Eremopetasitenin C1	C21H30O6	378.2042	131751301	Prenol lipids	Terpene lactones	HMDB0032770	Eremopetasitenin C1
metab_4576	5.7846	359.3259	pos	CHEBI:79294	C21H42O4	358.3081	86289872	-	-	-	-
metab_4774	4.7302	360.1796	pos	Ovalicin	C16H24O5	296.1624	10957430	Carbonyl compounds	Ketones	HMDB0038120	Ovalicin
metab_5496	2.2045	360.2115	pos	Asp-Leu-Leu	C16H29N3O6	359.2056	14934265	-	-	-	-
metab_5496	2.2045	360.2115	pos	Ile-Asp-Ile	C16H29N3O6	359.2053	145456083	-	-	-	-
metab_5496	2.2045	360.2115	pos	Glu-Ile-Val	C16H29N3O6	359.2056	71464664	-	-	-	-
metab_5496	2.2045	360.2115	pos	Val-Ile-Glu	C16H29N3O6	359.2056	145458950	-	-	-	-
metab_5708	1.7705	360.2227	pos	Asp Leu Leu	C16H29N3O6	359.2056	14934265	-	-	-	-
metab_5383	2.4530	360.2735	pos	3-hydroxylauroylcarnitine	C19H37NO5	359.267	71464535	-	-	-	-
metab_2076	3.1648	360.2736	pos	3-Hydroxydodecanoylcarnitine	C19H37NO5	359.2670	71464535	-	-	-	-
metab_13760	2.2912	361.0936	neg	6-Hydroxy-2-(4-hydroxyphenyl)-7,8-dimethoxy-3,4-dihydro-2H-1-benzopyran-4-one	C17H16O6	316.0947	131837228	Flavonoids	O-methylated flavonoids	HMDB0133294	6-hydroxy-2-(4-hydroxyphenyl)-7,8-dimethoxy-3,4-dihydro-2H-1-benzopyran-4-one
metab_5860	1.5144	361.1704	pos	L-Tryptophan-L-arginine	C17H24N6O3	360.1898	25186251	-	-	-	-

metab_13244	3.4800	361.2028	neg	Hydrocortisone	C21H30O5	362.2062	5754	Steroids and steroid derivatives	Hydroxysteroids	HMDB0000063	Cetacort
metab_9387	4.5219	361.2237	neg	(3R)-12-[(3,6-Dideoxy-alpha-L-arabino-hexopyranosyl)oxy]-3-hydroxylauric acid	C18H34O7	362.2307	86289848	-	-	-	-
metab_13051	4.0516	361.2238	neg	(3R)-12-[(3,6-Dideoxy-alpha-L-arabino-hexopyranosyl)oxy]-3-hydroxylauric acid	C18H34O7	362.2307	86289848	-	-	-	-
metab_9058	3.2301	361.2238	neg	(3R)-12-[(3,6-Dideoxy-alpha-L-arabino-hexopyranosyl)oxy]-3-hydroxylauric acid	C18H34O7	362.2307	86289848	-	-	-	-
metab_12795	4.9510	361.2238	neg	(3R)-12-[(2R,3R,5R,6S)-3,5-dihydroxy-6-methyloxan-2-yl]oxy-3-hydroxydodecanoic acid	C18H34O7	362.2307	86289848	-	-	-	-
metab_14747	0.6973	362.0510	neg	Guanosine-5'-monophosphate	C11 H10 N9 O4 P	363.0583	135398631	-	-	-	-
metab_5946	1.3437	362.1101	pos	N(6)-methyl-AMP	C11H16N5O7 P	361.0786	16760025	-	-	-	-
metab_4525	6.0252	362.3256	pos	2-Amino-1,3,4,5-eicosanetetrol	C20H43NO4	361.3188	122391237	-	-	-	-
metab_2075	3.1648	363.2159	pos	Hydrocortisone	C21H30O5	362.2066	5754	-	-	-	-
metab_2241	3.8967	363.2160	pos	Hydrocortisone	C21H30O5	362.2062	5754	Steroids and steroid derivatives	Hydroxysteroids	HMDB0000063	Cetacort
metab_7426	4.1869	363.2184	neg	Tetrahydrocortisone	C21H32O5	364.2250	5866	Steroids and steroid derivatives	Hydroxysteroids	HMDB0000903	Tetrahydrocortisone
metab_4899	4.1544	364.2684	pos	N-cis-octadec-9Z-enoyl-L-Ho	C22H39NO3	363.2747	71684636	-	-	-	-

				moserine lactone							
metab_989	6.4192	364.2832	pos	5(6)-EpETrE-EA	C22H37NO3	363.2746	16061181	-	-	-	-
metab_989	6.4192	364.2832	pos	NA	C22H39NO3	363.2747	NA	-	-	-	-
metab_7351	8.0688	364.2863	neg	(Z,Z)-4-((1-Oxo-9,12-octadeca dienyl)amino)butanoic acid	C22H39NO3	365.2930	6438152	Carboxylic acids and derivatives	Amino acids, peptides, and analogues	HMDB0062334	N-Linoleoyl GABA
metab_1761	1.9918	365.0829	pos	Atovaquone	C22H19ClO3	364.0771	74989	-	-	-	-
metab_13736	2.3380	365.1358	neg	(4S,6R)-p-Mentha-1,8-diene-6,7-diol 7-glucoside	C16H26O7	330.1679	85382765	Prenol lipids	Terpene glycosides	HMDB0039056	(4S,6R)-p-Mentha-1,8-diene-6,7-diol 7-glucoside
metab_6909	1.4710	365.1360	neg	(2R,3R,4S,5R,6S)-2-(Hydroxymethyl)-6-[4-(1H-imidazol-2-ylmethyl)-2-methoxyphenoxy]oxane-3,4,5-triol	C17H22N2O7	366.1427	100927770	Organooxygen compounds	Carbohydrates and carbohydrate conjugates	HMDB0033108	Semilepidinoside B
metab_6992	4.9349	365.2103	neg	1-Tetradecylglycerone 3-phosphate(2-)	C17H35O6P	366.2172	86289246	-	-	-	-
metab_4902	4.1387	365.2315	pos	11beta,17alpha,21-Trihydroxy pregnenolone	C21H32O5	364.2245	11966170	-	-	-	-
metab_2090	3.2116	365.2315	pos	11beta,17alpha,21-Trihydroxy pregnenolone	C21H32O5	364.2245	11966170	-	-	-	-
metab_2243	3.9114	365.2315	pos	11beta,17alpha,21-Trihydroxy pregnenolone	C21H32O5	364.2245	11966170	-	-	-	-
metab_12220	7.3180	365.2329	neg	Cortolone	C21H34O5	366.2405	160499	-	-	-	-
metab_12220	7.3180	365.2329	neg	Tetrahydrocortisol	C21H34O5	366.2405	5864	-	-	-	-
metab_9570	5.4844	365.2335	neg	Cortolone	C21H34O5	366.2405	160499	-	-	-	-
metab_9570	5.4844	365.2335	neg	Tetrahydrocortisol	C21H34O5	366.2405	5864	-	-	-	-
metab_12164	7.5397	365.2336	neg	Cortolone	C21H34O5	366.2405	160499	-	-	-	-
metab_12164	7.5397	365.2336	neg	Tetrahydrocortisol	C21H34O5	366.2405	5864	-	-	-	-

metab_7026	6.9525	365.2336	neg	Cortolone	C21H34O5	366.2405	160499	-	-	-	-
metab_7026	6.9525	365.2336	neg	Tetrahydrocortisol	C21H34O5	366.2405	5864	-	-	-	-
metab_9816	6.8255	365.2336	neg	Cortolone	C21H34O5	366.2405	160499	-	-	-	-
metab_9816	6.8255	365.2336	neg	Tetrahydrocortisol	C21H34O5	366.2405	5864	-	-	-	-
metab_12078	7.7728	365.2336	neg	Cortolone	C21H34O5	366.2405	160499	-	-	-	-
metab_12078	7.7728	365.2336	neg	Tetrahydrocortisol	C21H34O5	366.2405	5864	-	-	-	-
metab_9216	3.8001	365.2338	neg	Ucriol	C20H32O3	320.2351	12315549	Prenol lipids	Diterpenoids	HMDB0036705	Ucriol
metab_9216	3.8001	365.2338	neg	Cortolone	C21H34O5	366.2405	160499	Prenol lipids	Diterpenoids	HMDB0036705	Ucriol
metab_9216	3.8001	365.2338	neg	Tetrahydrocortisol	C21H34O5	366.2405	5864	Prenol lipids	Diterpenoids	HMDB0036705	Ucriol
metab_7423	4.2203	365.2339	neg	Cortolone	C21H34O5	366.2405	160499	-	-	-	-
metab_7423	4.2203	365.2339	neg	Tetrahydrocortisol	C21H34O5	366.2405	5864	-	-	-	-
metab_711	7.0961	365.2673	pos	16,16-Dimethyl-PGE1	C22H38O5	364.2613	5283058	-	-	-	-
metab_4510	6.1311	365.3153	pos	16,16-dimethyl-PGE1	C22H38O5	364.2613	5283058	-	-	-	-
metab_9759	6.5190	366.2653	neg	alpha-Linolenoyl ethanolamide	C20H35NO2	321.2668	5283449	Organonitrogen compounds	Amines	HMDB0013624	Alpha-Linolenoyl ethanolamide
metab_7330	8.5455	366.3018	neg	N-Oleoyl-4-aminobutyric acid	C22H41NO3	367.3086	16759340	Carboxylic acids and derivatives	Amino acids, peptides, and analogues	HMDB0062335; LMFA08020104	N-Oleoyl GABA
metab_11652	8.8929	367.1587	neg	4'-O-Methylxanthohumol	C22H24O5	368.1623	10959555	Linear 1,3-diarylpropanoids	Chalcones and dihydrochalcones	HMDB0038815	2',4-Dihydroxy-4',6'-dimethoxy-3'-prenylchalcone
metab_2131	3.3490	367.2106	pos	Methoxyfenozide	C22H28N2O3	366.2015	105010	-	-	-	-
metab_13199	3.5978	367.2131	neg	20-Hydroxy-PGE2	C20H32O6	368.2199	5283034	Fatty Acyls	Eicosanoids	HMDB0003247; LMFA03010014	20-Hydroxy-PGE2
metab_13199	3.5978	367.2131	neg	Prostaglandin G2	C20H32O6	368.2196	5280883	Fatty Acyls	Fatty acids and conjugates	HMDB0003235	9,11-Epidioxy-15-hydroperoxy-prosta-5,13-dien-1-Oic acid
metab_9037	3.1626	367.2131	neg	Prostaglandin G2	C20H32O6	368.2196	5280883	Fatty Acyls	Fatty acids and	HMDB0003235	9,11-Epidioxy-15-hydroperoxy-pros

									conjugates		ta-5,13-dien-1-Oic acid
metab_9536	5.2579	367.2254	neg	Cortol	C21H36O5	368.2558	246873	Steroids and steroid derivatives	Hydroxysteroids	HMDB0003180	alpha-Cortol
metab_4503	6.1757	367.2467	pos	JWH 018 4-hydroxyindole metabolite-d9	C24H14D9NO2	366.2382	53394760	-	-	-	-
metab_4503	6.1757	367.2467	pos	Cortolone	C21H34O5	366.2405	160499	-	-	-	-
metab_4503	6.1757	367.2467	pos	Tetrahydrocortisol	C21H34O5	366.2405	5864	-	-	-	-
metab_4658	5.3165	367.2470	pos	JWH 018 4-hydroxyindole metabolite-d9	C24H14D9NO2	366.2382	53394760	-	-	-	-
metab_4658	5.3165	367.2470	pos	Cortolone	C21H34O5	366.2405	160499	-	-	-	-
metab_4658	5.3165	367.2470	pos	Tetrahydrocortisol	C21H34O5	366.2405	5864	-	-	-	-
metab_2048	3.0114	367.2471	pos	JWH 018 4-hydroxyindole metabolite-d9	C24H14D9NO2	366.2382	53394760	-	-	-	-
metab_2048	3.0114	367.2471	pos	Cortolone	C21H34O5	366.2405	160499	-	-	-	-
metab_2048	3.0114	367.2471	pos	Tetrahydrocortisol	C21H34O5	366.2405	5864	-	-	-	-
metab_10404	9.0872	367.2475	neg	Carboprost	C21H36O5	368.2565	5281075	-	-	-	-
metab_9751	6.4553	367.2485	neg	Carboprost	C21H36O5	368.2565	5281075	-	-	-	-
metab_9716	6.3111	367.2488	neg	Carboprost	C21H36O5	368.2565	5281075	-	-	-	-
metab_9692	6.1482	367.2491	neg	Carboprost	C21H36O5	368.2565	5281075	-	-	-	-
metab_12229	7.2701	367.2492	neg	Carboprost	C21H36O5	368.2565	5281075	-	-	-	-
metab_9236	3.8506	367.2494	neg	Carboprost	C21H36O5	368.2565	5281075	-	-	-	-
metab_9300	4.1023	367.2494	neg	Carboprost	C21H36O5	368.2565	5281075	Steroids and steroid derivatives	Hydroxysteroids	HMDB0003180	Cortol
metab_9300	4.1023	367.2494	neg	Cortol	C21H36O5	368.2563	246873	Steroids and steroid derivatives	Hydroxysteroids	HMDB0003180	Cortol

metab_10186	8.3055	367.2495	neg	Carboprost	C21H36O5	368.2565	5281075	-	-	-	-
metab_4985	3.7599	367.2682	pos	Cortolone	C21H34O5	366.2401	160499	Steroids and steroid derivatives	Flavones	HMDB0003128	Cortolone, (3alpha,5alpha,20S)-isomer
metab_5585	2.0524	368.1601	pos	Tyrosyl-tryptophan	C20H21N3O4	367.1532	7021832	-	-	-	-
metab_10252	8.5455	368.3083	neg	Aplidiasphingosine	C22H43NO3	369.3241	42608352	-	-	-	-
metab_4456	6.4192	369.1714	pos	4'-O-Methylxanthohumol	C22H24O5	368.1623	10959555	-	-	-	-
metab_13121	3.8001	369.1924	neg	CHEBI:66791	C20H26N4O3	370.1989	135438508	-	-	-	-
metab_7701	3.2972	369.2287	neg	6-Keto-prostaglandin F1alpha	C20H34O6	370.2356	5280888	-	-	-	-
metab_7701	3.2972	369.2287	neg	Thromboxane B2	C20H34O6	370.2357	5283137	-	-	-	-
metab_12721	5.3546	369.2288	neg	6-Keto-prostaglandin F1alpha	C20H34O6	370.2356	5280888	-	-	-	-
metab_12721	5.3546	369.2288	neg	Thromboxane B2	C20H34O6	370.2357	5283137	-	-	-	-
metab_12927	4.5054	369.2288	neg	6-Keto-prostaglandin F1alpha	C20H34O6	370.2356	5280888	-	-	-	-
metab_12927	4.5054	369.2288	neg	Thromboxane B2	C20H34O6	370.2357	5283137	-	-	-	-
metab_9286	4.0516	369.2392	neg	Thromboxane B2	C20H34O6	370.2357	5283137	Fatty Acyls	Eicosanoids	HMDB0003252	b2, Thromboxane
metab_2615	6.6606	369.2626	pos	Cortol	C21H36O5	368.2558	246873	-	-	-	-
metab_2615	6.6606	369.2626	pos	Carboprost	C21H36O5	368.2560	5281075	-	-	-	-
metab_13071	3.9677	369.2650	neg	1-Naphthylacetylspermine	C22H34N4O	370.2722	129695	-	-	-	-
metab_10605	10.0432	369.3377	neg	2-Hydroxytricosanoic acid	C23H46O3	370.3449	152915	-	-	-	-
metab_1681	1.7128	370.1963	pos	Corydaline	C22H27NO4	369.1937	101301	-	-	-	-
metab_657	5.8907	370.2943	pos	O-[(9Z)-Tetradecenoyl]-L-carnitine	C21H39NO4	369.2878	90659872	-	-	-	-
metab_5001	3.7148	370.2943	pos	O-[(9Z)-Tetradecenoyl]-L-carnitine	C21H39NO4	369.2878	90659872	-	-	-	-
metab_2270	4.0479	370.3055	pos	(Z)-myristoleoylcarnitine	C21H39NO4	369.2878	90659872	-	-	-	-
metab_3908	9.0112	370.3306	pos	Aplidiasphingosine	C22H43NO3	369.3241	42608352	-	-	LMFA08020112	

metab_4783	4.6851	371.2209	pos	CHEBI:66791	C20H26N4O3	370.1989	135438508	-	-	-	-
metab_9564	5.4355	371.2442	neg	8,8a-Deoxyoleandolide	C20H36O6	372.2515	193898	-	-	-	-
metab_9687	6.1320	371.2444	neg	8,8a-Deoxyoleandolide	C20H36O6	372.2515	193898	-	-	-	-
metab_9594	5.6128	371.2444	neg	8,8a-Deoxyoleandolide	C20H36O6	372.2515	193898	-	-	-	-
metab_11790	8.6239	371.2444	neg	8,8a-Deoxyoleandolide	C20H36O6	372.2515	193898	-	-	-	-
metab_13274	3.3631	371.2446	neg	8,8a-Deoxyoleandolide	C20H36O6	372.2514	193898	-	-	-	-
metab_3064	9.5283	371.2782	pos	1-Naphthylacetylspermine	C22H34N4O	370.2718	129695	-	-	-	-
metab_126	6.0107	371.2783	pos	1-Naphthylacetylspermine	C22H34N4O	370.2718	129695	-	-	-	-
metab_4325	7.0816	371.2783	pos	1-Naphthylacetylspermine	C22H34N4O	370.2718	129695	Prenol lipids	Diterpenoids	HMDB0035379	Sterebin E
metab_4325	7.0816	371.2783	pos	Sterebin E	C20H34O4	338.2457	131751729	Prenol lipids	Diterpenoids	HMDB0035379	Sterebin E
metab_6418	0.5420	372.2345	pos	7-O-Acetylsalutaridinol	C21H25NO5	371.1731	5460163	-	-	-	-
metab_6418	0.5420	372.2345	pos	Isoandrocymbine	C21H25NO5	371.1731	46173814	-	-	-	-
metab_4549	5.9361	372.3097	pos	Myristoylcarnitine	C21H41NO4	371.3033	6426854	-	-	-	-
metab_964	8.3912	372.3098	pos	Myristoylcarnitine	C21H41NO4	371.3033	6426854	-	-	-	-
metab_4441	6.5395	372.3098	pos	Myristoylcarnitine	C21H41NO4	371.3033	6426854	-	-	-	-
metab_2797	7.8532	372.3098	pos	Myristoylcarnitine	C21H41NO4	371.3033	6426854	-	-	-	-
metab_129	6.3885	372.3099	pos	Myristoylcarnitine	C21H41NO4	371.3033	6426854	-	-	-	-
metab_2526	5.8148	373.3415	pos	8,8a-Deoxyoleandolide	C20H36O6	372.2515	193898	-	-	-	-
metab_11589	9.0872	374.1327	neg	Haloperidol	C21H23ClFNO2	375.1396	3559	-	-	-	-
metab_7448	1.5439	374.1572	neg	Glu-Asp-Ile	C15H25N3O8	375.1644	71464589	-	-	-	-
metab_7503	1.8205	375.1321	neg	Riboflavin	C17H20N4O6	376.1380	493570	-	-	-	-
metab_9127	3.4969	375.1820	neg	Yucalexin P15	C20H26O4	330.1831	131752048	Prenol lipids	Diterpenoids	HMDB0036753	Yucalexin P15
metab_4128	8.1132	375.3036	pos	(3beta,22E,24R)-3-Hydroxyergosta-5,8,22-trien-7-one	C28H42O2	410.3185	131751258	Steroids and steroid derivatives	Ergostane steroids	HMDB0032106	(3beta,22E,24R)-3-Hydroxyergosta-5,8,22-trien-7-one

metab_8007	0.8662	376.0667	neg	NA	C11H16N5O8P	377.0739	440190	-	-	-	-
metab_5943	1.3579	376.1736	pos	Glu-Asp-Ile	C15H25N3O8	375.1644	71464589	-	-	-	-
metab_6600	0.5851	377.0859	neg	Inulobiose	C12H22O11	342.1162	439552	Carbohydrates and carbohydrate conjugates	Glycosyl compounds	HMDB0029898	Inulobiose
metab_11889	8.3055	377.1434	neg	Abafungin	C21H22N4OS	378.1500	159326	-	-	-	-
metab_5693	1.8134	377.1444	pos	Riboflavin	C17H20N4O6	376.1380	493570	-	-	-	-
metab_8370	1.5736	377.1470	neg	1,5-Dihydroriboflavin	C17H22N4O6	378.1531	14080393	-	-	-	-
metab_4070	8.3322	377.3194	pos	(3beta,22E,24R)-Ergosta-4,6,8(14),22-tetraen-3-ol	C28H42O	394.3236	131753018	Steroids and steroid derivatives	Ergostane steroids	HMDB0041050	(3beta,22E,24R)-Ergosta-4,6,8(14),22-tetraen-3-ol
metab_14842	0.5991	379.0827	neg	5,8-Dihydroxy-3-(4-hydroxybenzyl)-7-methoxy-4-chromanone 8-acetate	C19H18O7	358.1053	21627907	Homoisoflavonoids	Homoisoflavans	HMDB0037252	5,8-Dihydroxy-3-(4-hydroxybenzyl)-7-methoxy-4-chromanone 8-acetate
metab_8961	2.9429	379.1516	neg	2,3-Dinor-6-keto-prostaglandin F1 a	C18H30O6	342.2042	53477747	Fatty Acyls	Eicosanoids	HMDB0002277	2,3-Dinor-6-keto-prostaglandin F1 a
metab_8859	2.6678	379.2131	neg	Bicyclo-PGE2	C20H30O4	334.2144	5283043	Fatty Acyls	Eicosanoids	LMFA03010034; HMDB0060054	bicyclo-PGE2
metab_2585	6.3885	379.3310	pos	1-Arachidonoylglycerol	C23H38O4	378.2767	5282281	-	-	-	-
metab_4016	8.5554	379.3353	pos	Ergosterol	C28H44O	396.3392	444679	Steroids and steroid derivatives	Ergostane steroids	LMST01030093; HMDB0000878	Ergosterol
metab_292	1.2162	380.1473	pos	Angoline	C22H21NO5	379.1418	189060	-	-	-	-
metab_8330	1.5297	380.1562	neg	cis-Zeatin-O-glucoside	C16H23N5O6	381.1638	5280589	-	-	-	-
metab_14435	1.3089	380.1565	neg	cis-Zeatin-O-glucoside	C16H23N5O6	381.1638	5280589	-	-	-	-
metab_2898	8.4949	380.3148	pos	N-Linoleoyl valine	C23H41NO3	379.3086	52922069	-	-	LMFA08020121	
metab_3	0.5843	381.0783	pos	Sucrose	C12H22O11	342.1162	5988	Organooxygen compounds	Carbohydrates and carbohydrate conjugates	HMDB0000258	Sucrose

metab_3	0.5843	381.0783	pos	Resorcinolnaphthalein	C24H14O5	380.0720	337218	Organooxygen compounds	Carbohydrates and carbohydrate conjugates	HMDB0000258	Sucrose
metab_10418	9.1200	381.1745	neg	Melleolide	C23H28O6	400.1886	158276	Prenol lipids	Sesquiterpenoids	HMDB0035689	Melleolide
metab_5108	3.3032	381.2265	pos	Nervosanin A	C21H32O6	380.2194	70698018	-	-	-	-
metab_2061	3.0877	381.2267	pos	Nervosanin A	C21H32O6	380.2194	70698018	-	-	-	-
metab_12785	5.0002	381.2287	neg	Sarcostin	C21H34O6	382.2353	46173994	-	-	-	-
metab_13425	2.9931	381.2289	neg	17,18-DiHETE	C20H32O4	336.2301	16061120	Fatty Acyls	Eicosanoids	LMFA03060078; HMDB0010211	17,18-DiHETE
metab_13425	2.9931	381.2289	neg	Lys Pro His	C17H28N6O4	382.2392	145456757	-	-	-	-
metab_10009	7.6323	381.2324	neg	Lys-Pro-His	C17H28N6O4	382.2392	145456757	-	-	-	-
metab_6856	1.2663	382.1009	neg	Succinyladenosine	C14H17N5O8	383.1075	20849086	-	-	-	-
metab_4362	6.9638	382.2941	pos	PGF2alpha dimethyl amide	C22H39NO4	381.2879	5283075	-	-	LMFA03010074	
metab_9779	6.6320	382.2967	neg	N-Oleoyl threonine	C22H41NO4	383.3039	52922063	-	-	-	-
metab_10414	9.1038	382.2967	neg	N-Oleoyl threonine	C22H41NO4	383.3039	52922063	-	-	-	-
metab_12351	6.8887	382.2971	neg	N-Oleoyl threonine	C22H41NO4	383.3039	52922063	-	-	-	-
metab_10501	9.5478	383.1901	neg	Bortezomib	C19H25BN4O4	384.1971	387447	-	-	-	-
metab_11265	10.1910	383.3535	neg	2-Hydroxytetracosanoic acid	C24H48O3	384.3603	102430	Fatty Acyls	Fatty acids and conjugates	HMDB0039540	Cerebronic acid
metab_298	1.2446	384.1139	pos	Succinyladenosine	C14H17N5O8	383.1075	20849086	-	-	-	-
metab_4842	4.4272	384.2733	pos	13-Hydroxy-9-methoxy-10-oxo-11-octadecenoic acid	C19H34O5	342.2406	6366720	Fatty Acyls	Lineolic acids and derivatives	HMDB0040901	13-Hydroxy-9-methoxy-10-oxo-11-octadecenoic acid
metab_2900	8.4949	384.3097	pos	2-Arachidonoyl glycerol-d5	C23H33D5O4	383.3009	52922087	-	-	-	-
metab_6267	0.7801	385.1275	pos	S-Adenosyl-L-homocysteine	C14H20N6O5S	384.1216	439155	5'-deoxyribonucleosides	5'-deoxy-5'-thionucleosides	HMDB0000939	S-Adenosylhomocysteine

metab_9791	6.7278	385.2602	neg	6-Deoxyerythronolide B	C21H38O6	386.2668	121904	Prenol lipids	Sesquiterpenoids	HMDB0038018	Cryptomeridiol 11-rhamnoside
metab_9791	6.7278	385.2602	neg	CryptoMeridiol 11-rhaMnoside	C21H38O6	386.2668	11731806	Prenol lipids	Sesquiterpenoids	HMDB0038018	Cryptomeridiol 11-rhamnoside
metab_10305	8.7031	385.2602	neg	6-Deoxyerythronolide B	C21H38O6	386.2668	121904	-	-	-	-
metab_12452	6.5033	385.2604	neg	6-Deoxyerythronolide B	C21H38O6	386.2668	121904	-	-	-	-
metab_2823	8.0988	385.3050	pos	N-Palmitoyl glutamine	C21H40N2O4	384.2988	52922071	-	-	LMFA08020127	
metab_11386	9.7312	385.3328	neg	10-Undecen-1-OL	C11H22O	170.1671	8185	Fatty Acyls	Fatty alcohols	LMFA05000587; HMDB0031016	10-Undecen-1-ol
metab_661	5.9504	386.2891	pos	O-[(9Z)-3-Hydroxytetradec-9-enoyl]carnitine	C21H39NO5	385.2823	86289104	-	-	-	-
metab_2489	5.5715	386.2892	pos	O-[(9Z)-3-Hydroxytetradec-9-enoyl]carnitine	C21H39NO5	385.2823	86289104	-	-	-	-
metab_9079	3.2972	387.1484	neg	9,20-Didehydro-17-hydroxy-2 2-norajmalan-16-carboxylic acid methyl ester	C21H24N2O3	352.1787	5382842	Corynanthean-type alkaloids	-	HMDB0030389	Quebrachidine
metab_11	5.1830	387.1792	pos	Burseran	C22H26O6	386.1726	11101102	-	-	-	-
metab_5900	1.4429	387.2228	pos	Citroside B	C19H30O8	386.1938	14312562	-	-	-	-
metab_12352	6.8887	387.2752	neg	15-(3'R,5'R-Dihydroxy-6'S-methyl-(2H)-tetrahydropyran-2'-yloxy)-pentadecanoic acid	C21H40O6	388.2823	86289786	-	-	-	-
metab_9909	7.1749	387.2758	neg	15-(3'R,5'R-Dihydroxy-6'S-methyl-(2H)-tetrahydropyran-2'-yloxy)-pentadecanoic acid	C21H40O6	388.2823	86289786	-	-	-	-
metab_2189	3.6384	387.2997	pos	6-Deoxyerythronolide B	C21H38O6	386.2668	121904	-	-	-	-
metab_1624	1.5424	389.1695	pos	Tuberonic acid glucoside	C18H28O9	388.1736	5281204	-	-	-	-
metab_12953	4.3716	391.1897	neg	13,14-Dihydro-lipoxin A4	C20H34O5	354.2406	53481469	Fatty Acyls	Eicosanoids	HMDB0012563;	13,14-Dihydro- lipoxin A4

										LMFA03050024	
metab_518	3.1191	391.2464	pos	Leu-Met-Lys	C17H34N4O4S1	390.2381	25123936	-	-	-	-
metab_518	3.1191	391.2464	pos	Lys-Ile-Met	C17H34N4O4S1	390.2379	145456690	-	-	-	-
metab_8705	2.2754	392.1833	neg	Glu-Phe-Val	C19H27N3O6	393.1900	71464665	-	-	-	-
metab_13975	1.8974	392.1834	neg	Glu-Phe-Val	C19H27N3O6	393.1900	71464665	-	-	-	-
metab_9846	6.9525	392.2810	neg	Anandamide	C22H37NO2	347.2824	5281969	Organonitrogen compounds	Amines	LMFA08040056; HMDB0004080; LMFA08040001	Anandamide
metab_14234	1.5439	393.1340	neg	n-Acetylserotonin glucuronide	C18H22N2O8	394.1376	29971054	Organooxygen compounds	Carbohydrates and carbohydrate conjugates	HMDB0060833	N-Acetylserotonin glucuronide
metab_12285	7.0796	393.1381	neg	Pyridin-3-ylmethyl {4-[(2-amino-4-fluorophenyl)carbamoyl]benzyl}carbamate	C21H19FN4O3	394.1450	118221163	-	-	-	-
metab_9152	3.5813	393.1678	neg	5,7-Megastigmadien-9-ol glucoside	C19H32O6	356.2199	131753017	Fatty Acyls	Fatty acyl glycosides	HMDB0041044	5,7-Megastigmadien-9-ol glucoside
metab_6961	4.3882	393.1956	neg	12,20-Dioxo-leukotriene B4	C20H28O5	348.1937	122164848	Fatty Acyls	Eicosanoids	HMDB0060094	12,20-Dioxo-leukotriene B4
metab_1822	2.1595	393.2121	pos	Leu-Phe-Asn	C19H28N4O5	392.2056	71464635	-	-	-	-
metab_10042	7.7404	393.2764	neg	2-Hydroxydocosanoic acid	C22H44O3	356.3290	193484	Fatty Acyls	Fatty acids and conjugates	HMDB0061660	2(R)-hydroxydocosanoic acid
metab_2353	4.6096	393.3104	pos	Auda	C23H40N2O3	392.3039	10069117	-	-	-	-
metab_3104	9.7899	393.3141	pos	gamma-Tocotrienol	C28H42O2	410.3185	5282349	Prenol lipids	Quinone and hydroquinone lipids	HMDB0012958; LMPR02020057	Gamma-Tocotrienol
metab_1856	2.2665	394.1964	pos	Glu-Phe-Val	C19H27N3O6	393.1900	71464665	-	-	-	-
metab_12315	7.0162	395.1535	neg	Mirabegron	C21H24N4O2	396.1605	9865528	-	-	-	-

					S						
metab_10308	8.7191	395.1537	neg	Mirabegron	C21H24N4O2S	396.1605	9865528	-	-	-	-
metab_6019	1.2162	395.1551	pos	SCHEMBL16892136	C21H19FN4O3	394.145	118221163	-	-	-	-
metab_7634	2.6841	395.2080	neg	20-Oxoleukotriene B4	C20H30O5	350.2093	6449839	Fatty Acyls	Eicosanoids	LMFA03020064; HMDB0012641	20-oxo-leukotriene B4
metab_9558	5.4034	395.2200	neg	(9S,10S)-10-Hydroxy-9-(phosphonooxy)octadecanoic acid	C18H37O7P	396.2279	23724633	Fatty Acyls	Fatty acids and conjugates	HMDB0059632	(9S,10S)-10-hydroxy-9-(phosphonooxy)octadecanoate
metab_12577	5.9866	395.2207	neg	(9S,10S)-10-Hydroxy-9-(phosphonooxy)octadecanoic acid	C18H37O7P	396.2279	23724633	-	-	-	-
metab_12112	7.6951	395.2208	neg	(9S,10S)-10-Hydroxy-9-(phosphonooxy)octadecanoic acid	C18H37O7P	396.2279	23724633	-	-	-	-
metab_11913	8.2409	395.2444	neg	Ascorbyl palmitate	C22H38O7	414.2618	54680660	Fatty Acyls	Fatty acid esters	LMFA07010788; HMDB0039883	Ascorbyl palmitate
metab_5105	3.3183	396.2734	pos	Dinoprost	C20H34O5	354.2406	5280363	Fatty Acyls	Eicosanoids	HMDB0001139; LMFA03010002	Prostaglandin F2a
metab_12326	6.9688	397.2264	neg	His-Leu-Gln	C17H28N6O5	398.2341	46851994	-	-	-	-
metab_2455	5.3311	399.2269	pos	His Leu Gln	C17H28N6O5	398.2341	46851994	-	-	-	-
metab_11558	9.1200	399.2754	neg	16-(3'R,5'R-Dihydroxy-6'S-methyl-(2H)-tetrahydropyran-2'-yloxy)-2E-hexadecenoic acid	C22H40O6	400.2828	86289787	-	-	-	-
metab_12432	6.5989	399.2756	neg	16-(3'R,5'R-Dihydroxy-6'S-methyl-(2H)-tetrahydropyran-2'-yloxy)-2E-hexadecenoic acid	C22H40O6	400.2828	86289787	-	-	-	-
metab_11339	9.8947	399.3484	neg	Tricosanoic acid	C23H46O2	354.3498	17085	Fatty Acyls	Fatty acids and	HMDB0001160;	Tricosanoic acid

									conjugates	LMFA01010023	
metab_9722	6.3427	400.3071	neg	Pristanoylglycine	C21H41NO3	355.3086	53481664	Prenol lipids	Diterpenoids	HMDB0013303	Pristanoylglycine
metab_9319	4.1869	401.0883	neg	5,7-Dihydroxy-2-(4-hydroxyphenyl)-8-(3,4,5-trihydroxyoxan-2-yl)-4H-chromen-4-one	C20H18O9	402.0951	74977436	Flavonoids	Flavonoid glycosides	HMDB0127225	5,7-dihydroxy-2-(4-hydroxyphenyl)-8-(3,4,5-trihydroxyoxan-2-yl)-4H-chromen-4-one
metab_13818	2.1630	401.1832	neg	JWH 200 7-hydroxyindole metabolite	C25H24N2O3	402.1892	91701367	-	-	-	-
metab_8833	2.5893	401.2409	neg	Ala-Leu-Leu-Ser	C18H34N4O6	402.2481	71464518	-	-	-	-
metab_9554	5.3713	401.2551	neg	Erythronolide B	C21H38O7	402.2620	441113	-	-	-	-
metab_4863	4.3371	402.2840	pos	Myriocin	C21H39NO6	401.2777	6438394	-	-	-	-
metab_2181	3.5923	402.2840	pos	Myriocin	C21H39NO6	401.2776	6438394	-	-	-	-
metab_2461	5.3608	402.2840	pos	Myriocin	C21H39NO6	401.2777	6438394	-	-	-	-
metab_4607	5.6322	402.2841	pos	Myriocin	C21H39NO6	401.2777	6438394	-	-	-	-
metab_4991	3.7447	402.2841	pos	Myriocin	C21H39NO6	401.2777	6438394	-	-	-	-
metab_4825	4.5027	402.2843	pos	Myriocin	C21H39NO6	401.2777	6438394	-	-	-	-
metab_7548	2.0358	403.1626	neg	Glu-Ala-Trp	C19H24N4O6	404.1685	10237737	-	-	-	-
metab_7513	1.8524	403.1626	neg	Glu-Ala-Trp	C19H24N4O6	404.1685	10237737	-	-	-	-
metab_1944	2.5938	403.2532	pos	Ala-Leu-Leu-Ser	C18H34N4O6	402.2478	71464518	-	-	-	-
metab_12841	4.8049	403.2705	neg	3R-Hydroxy-15-(3'R,5'R-dihydroxy-6'S-methyl-(2H)-tetrahydropyran-2'-yloxy)-pentadecanoic acid	C21H40O7	404.2776	86289851	Fatty Acyls	Eicosanoids	HMDB0005076	13,14-Dihydro PGF-1a
metab_12841	4.8049	403.2705	neg	3R-Hydroxy-9R-(3'R,5'R-dihydroxy-6'S-methyl-(2H)-tetrahydropyran-2'-yloxy)-decanoic acid	C21H40O7	404.2772	86289816	Fatty Acyls	Eicosanoids	HMDB0005076	13,14-Dihydro PGF-1a

metab_648	5.1975	404.2058	pos	20-Hydroxy-fusarin	C22H29NO6	403.1993	91820337	-	-	-	-
metab_6347	0.6123	404.2061	pos	20-Hydroxy-fusarin	C22H29NO6	403.1993	91820337	-	-	-	-
metab_1693	1.7271	404.2132	pos	20-hydroxy-fusarin	C22H29NO6	403.1993	91820337	-	-	-	-
metab_6931	3.8164	405.2287	neg	Lys Pro Tyr	C20H30N4O5	406.233	7408735	-	-	-	-
metab_9872	7.0322	405.2420	neg	Nandrolone phenpropionate	C27H34O3	406.2490	229455	-	-	-	-
metab_9954	7.4300	407.2206	neg	1-(9Z-Hexadecenoyl)-glycero-3-phosphate	C19H37O7P	408.2277	52929751	Glycerophospholipids	Glycerophosphates	HMDB0062323; LMGP10050016	1-(9Z-hexadecenoyl)-glycero-3-phosphate
metab_9954	7.4300	407.2206	neg	1-Palmitoylglycerone 3-phosphate	C19H37O7P	408.2279	167650	Glycerophospholipids	Glycerophosphates	HMDB0062323; LMGP10050016	1-(9Z-hexadecenoyl)-glycero-3-phosphate
metab_7124	9.5314	407.2960	neg	Ganodosterone	C28H40O2	408.3028	13916720	Steroids and steroid derivatives	Ergostane steroids	HMDB0039148	Ganodosterone
metab_5851	1.5284	409.1856	pos	Vernoflexuoside	C21H28O8	408.1783	442320	-	-	-	-
metab_5851	1.5284	409.1856	pos	Vernoflexuoside	C21H28O8	408.1795	442320	-	-	-	-
metab_11982	8.0688	409.2367	neg	1-Palmitoylglycerol 3-phosphate	C19H39O7P	410.2436	89566	-	-	-	-
metab_4567	5.8597	409.2552	pos	Lys-Val-Tyr	C20H32N4O5	408.2486	145456823	-	-	-	-
metab_4567	5.8597	409.2552	pos	Val-Lys-Tyr	C20H32N4O5	408.2486	10454143	-	-	-	-
metab_2591	6.4035	409.2554	pos	Lys-Val-Tyr	C20H32N4O5	408.2486	145456823	-	-	-	-
metab_2591	6.4035	409.2554	pos	Val-Lys-Tyr	C20H32N4O5	408.2486	10454143	-	-	-	-
metab_10264	8.6093	409.2967	neg	Sorbitan oleate	C24H44O6	428.3138	5385498	Fatty Acyls	Fatty acid esters	LMFA07011018; HMDB0029886	Sorbitan oleate
metab_95	8.0700	409.3087	pos	5,9-Epidioxy-3-hydroxyergost-7-en-6-one	C28H44O4	444.3240	85218115	Steroids and steroid derivatives	Ergostane steroids	HMDB0032667	5,9-Epidioxy-3-hydroxyergost-7-en-6-one
metab_11276	10.1401	409.3691	neg	(2Z,6E)-3,7,11,15,19-Pentamethyl-2,6-eicosadien-1-ol	C25H48O	364.3705	57418214	Prenol lipids	Sesterterpenoids	HMDB0035154	(2Z,6E)-3,7,11,15,19-Pentamethyl-2,6-eicosadien-1-ol

metab_14344	1.4568	411.1276	neg	N6-Carbamoyl-L-threonyladenosine	C15H20N6O8	412.1343	161466	-	-	-	-
metab_4611	5.5864	411.2996	pos	1-Palmitoyl Lysophosphatidic Acid	C19H39O7P	410.2436	89566	-	-	-	-
metab_11618	9.0067	411.3123	neg	2-(3,4-Dihydroxyoxolan-2-yl)-2-hydroxyethyl octadecanoate	C24H46O6	430.3294	3793749	Fatty Acyls	Fatty acid esters	HMDB0029888; LMFA07011020	Sorbitan stearate
metab_816	9.0857	411.3248	pos	Ercalcitriol	C28H44O3	428.3290	9547243	Steroids and steroid derivatives	Vitamin D and derivatives	HMDB0006225; LMST03010040	Ercalcitriol
metab_5895	1.4569	413.1404	pos	N6-Carbamoyl-L-threonyladenosine	C15H20N6O8	412.1343	161466	-	-	-	-
metab_8989	3.0101	413.2776	neg	(E)-17-[(2R,3R,5R,6S)-3,5-dihydroxy-6-methyloxan-2-yl]oxyheptadec-2-enoic acid	C23H42O6	414.2986	86289789	-	-	-	-
metab_9795	6.7439	413.2915	neg	17-(3'R,5'R-Dihydroxy-6'S-methyl-(2H)-tetrahydropyran-2'-yloxy)-2E-heptadecenoic acid	C23H42O6	414.2986	86289789	-	-	-	-
metab_3938	8.8759	413.3407	pos	Amasterol	C28H44O2	412.3341	74951786	Steroids and steroid derivatives	Ergostane steroids	HMDB0030054	Amasterol
metab_10321	8.7346	413.3642	neg	20-Tetracosene-1,18-diol	C24H48O2	368.3654	71346384	Fatty Acyls	Fatty alcohols	HMDB0040887	20-Tetracosene-1,18-diol
metab_1927	2.5314	414.2046	pos	Citrulline	C6H13N3O3	175.0957	9750	Carboxylic acids and derivatives	Amino acids, peptides, and analogues	HMDB0000904	Citrulline
metab_4096	8.2289	414.3203	pos	3-Hydroxypalmitoleoylcarnitine	C23H43NO5	413.3141	71464549	-	-	LMFA08020090	
metab_1	6.4192	414.3204	pos	3-Hydroxypalmitoleoylcarnitine	C23H43NO5	413.3141	71464549	-	-	-	-
metab_639	5.3608	414.3205	pos	3-Hydroxypalmitoleoylcarnitin	C23H43NO5	413.3141	71464549	-	-	-	-

				e							
metab_8090	1.1069	415.1362	neg	Lophotoxin	C22H24O8	416.1454	108179	-	-	-	-
metab_125	5.7846	415.2106	pos	Magnoshinin	C24H30O6	414.2039	135783	-	-	-	-
metab_13629	2.5431	415.2343	neg	Distigmine	C22H32N4O4	416.2414	3116	-	-	-	-
metab_13494	2.8269	415.2346	neg	Distigmine	C22H32N4O4	416.2414	3116	-	-	-	-
metab_12458	6.4874	417.2287	neg	4,4'-(Diphenylethenylidene)bis [N,N-dimethylbenzenamine]	C30H30N2	418.2426	253783	-	-	-	-
metab_12458	6.4874	417.2287	neg	9'-Carboxy-gamma-tocotrienol	C23H32O4	372.2301	53481537	Prenol lipids	Monoterpenoids	HMDB0012869	9'-Carboxy-gamma-tocotrienol
metab_13686	2.4338	417.2501	neg	Erythronolide A	C21H38O8	418.2564	3082261	-	-	-	-
metab_7638	2.7155	417.2502	neg	Erythronolide A	C21H38O8	418.2564	3082261	-	-	-	-
metab_9814	6.8255	417.2865	neg	(3R,15R)-15-[(3,6-Dideoxy-alpha-L-arabino-hexopyranosyl)oxy]-3-hydroxypalmitic acid	C22H42O7	418.2935	86289823	-	-	-	-
metab_1784	2.0677	418.1597	pos	NA	C20H23N3O7	417.1536	NA	-	-	-	-
metab_2438	5.1830	418.2215	pos	NA	C23H31NO6	417.2148	54707875	-	-	-	-
metab_2994	9.0556	418.3303	pos	N-Arachidonoyl isoleucine	C26H43NO3	417.3243	52922064	-	-	LMFA08020111	
metab_7653	0.5286	421.0758	neg	Trehalose-6-phosphate	C12H23O14P	422.0825	122336	Organooxygen compounds	Carbohydrates and carbohydrate conjugates	HMDB0001124	Trehalose 6-phosphate
metab_12062	7.8193	421.2368	neg	2,2-Dibutyl-3-(4-methoxyphenyl)-4-methyl-2H-1-benzopyran-7-ol acetate	C27H34O4	422.2440	255271	-	-	-	-
metab_9674	6.0510	421.2715	neg	13'-Carboxy-gamma-tocotrienol	C28H40O4	440.2927	53481464	Prenol lipids	Quinone and hydroquinone lipids	HMDB0012558	13'-Carboxy-gamma-tocotrienol
metab_4371	6.9182	421.2938	pos	Hippolide G	C25H40O5	420.2873	53355994	-	-	-	-
metab_11684	8.7640	422.2707	neg	3-Hydroxytetradecanoylcarnitine	C21H41NO5	387.2985	71464541	Fatty Acyls	Fatty acid esters	HMDB0061640	3-hydroxytetradecanoyl carnitine

metab_12472	6.4073	423.2517	neg	1-Heptadecanoyl-glycero-3-phosphate	C20H41O7P	424.2588	44575057	-	-	-	-
metab_10221	8.4505	423.2528	neg	1-Heptadecanoyl-glycero-3-phosphate	C20H41O7P	424.2588	44575057	-	-	-	-
metab_698	6.4192	423.2736	pos	Lovastatin acid	C24H38O6	422.2665	64727	-	-	-	-
metab_134	5.9800	423.2740	pos	Lovastatin acid	C24H38O6	422.2665	64727	-	-	-	-
metab_644	5.4662	423.2741	pos	Lovastatin acid	C24H38O6	422.2665	64727	-	-	-	-
metab_9670	6.0346	423.2870	neg	2-Hydroxy-1-(hydroxymethyl)ethyl icosanoate	C23H46O4	386.3396	537294	Endocannabinoids	-	HMDB0011542	MG(0:0/20:0/0:0)
metab_694	6.8886	423.3091	pos	19-(3-methyl-butanoyloxy)-villanovane-13alpha,17-diol	C25H42O5	422.303	42608223	-	-	-	-
metab_1356	0.7801	424.1009	pos	S-(1,2-Dicarboxyethyl)glutathione	C14H21N3O10S	423.0948	9802383	-	-	-	-
metab_12224	7.3017	424.2472	neg	1-Tetradecanoyl-sn-glycero-3-phosphoethanolamine	C19H40NO7P	425.2542	9547070	Glycerophospholipids	Glycerophosphoethanolamines	HMDB0011500	LysoPE(14:0/0:0)
metab_13177	3.6645	427.1516	neg	Honyucitrin	C25H26O5	406.1780	44257865	Flavonoids	Flavones	HMDB0033536; LMPK12110410	Honyucitrin
metab_4927	4.0338	427.2585	pos	Methylcarbamyl PAF C-8	C18H39N2O7P	426.2382	124663	-	-	-	-
metab_11980	8.0688	427.2715	neg	Prostaglandin F2alpha 1-glyceryl ester	C23H40O7	428.2776	24778485	-	-	-	-
metab_9190	3.6980	429.2288	neg	Ala-Leu-Leu-Asp	C19H34N4O7	430.2428	71464517	-	-	-	-
metab_1304	0.5983	430.1922	pos	periglaucine D	C23H27NO7	429.1789	24879471	-	-	-	-
metab_7974	0.7818	431.1410	neg	(6a,11b,16a,17a)-6,9-Difluoro-11,17-dihydroxy-16-methyl-3-oxoandrosta-1,4-diene-17-carb	C21H26F2O5	396.1748	9822089	Steroids and steroid derivatives	Androstane steroids	HMDB0061092	fluticasone 17beta-carboxylic acid

				oxylic acid							
metab_5278	2.7653	431.2494	pos	Ala-Leu-Leu-Asp	C19H34N4O7	430.2428	71464517	-	-	-	-
metab_6627	7.5855	433.2366	neg	1-Oleoylglycerone 3-phosphate	C21H39O7P	434.2433	5280612	Glycerophospholipids	Glycerophosphates	HMDB0007852	LysoPA(0:0/18:2(9Z,12Z))
metab_5954	1.3437	434.1579	pos	Cyclic N-Acetylserotonin glucuronide	C18H20N2O8	392.1220	131769960	Organooxygen compounds	Carbohydrates and carbohydrate conjugates	HMDB0060812	cyclic N-Acetylserotonin glucuronide
metab_89	7.5450	435.2497	pos	1-Oleoylglycerone 3-phosphate	C21H39O7P	434.2433	5280612	Glycerophospholipids	Glycerophosphates	LMGP10050017; HMDB0007856	LysoPA(18:2(9Z,12Z)/0:0)
metab_12133	7.6323	436.2451	neg	Bimatoprost	C25H37NO4	415.2723	5311027	Fatty Acyls	Eicosanoids	HMDB0015041	Bimatoprost
metab_9376	4.4722	437.2664	neg	3-Hydroxy-10'-apo-b,y-carote nal	C27H36O2	392.2715	87443544	Prenol lipids	Sesterterpenoids	HMDB0039019	3-Hydroxy-10'-apo-b,y-carotenal
metab_7059	7.7251	438.2630	neg	3-Hydroxypentadecanoyl carnitine	C22H43NO5	401.3141	131770394	Fatty Acyls	Fatty acid esters	HMDB0061641	3-hydroxypentadecanoyl carnitine
metab_9484	4.9349	439.2472	neg	Scoparic acid A	C27H36O5	440.2544	44584621	-	-	-	-
metab_4524	6.0252	439.2685	pos	(-)-Fusicoplagin A	C24H38O7	438.2596	42608251	-	-	-	-
metab_9859	7.0162	441.2532	neg	L-Lysyl-L-phenylalanyl-L-phe nylalanine	C24H32N4O4	442.2604	71372064	-	-	-	-
metab_12699	5.4844	441.2975	neg	Desmethyl tocotrienol	C27H40O2	396.3028	6857436	Prenol lipids	Quinone and hydroquinone lipids	HMDB0030008	d-Tocotrienol
metab_4202	7.7514	441.3311	pos	Scoparic acid A	C27H36O5	440.2544	44584621	-	-	-	-
metab_9541	5.3060	441.3338	neg	3beta,5alpha,6beta-Trihydroxy cholestane	C27H48O3	420.3603	91498	Steroids and steroid derivatives	Cholestane steroids	HMDB0003990	3b,5a,6b-Cholestanetriol
metab_2982	8.9656	441.3716	pos	Soyasapogenol C	C30H48O2	440.3653	3083637	-	-	-	-
metab_2458	5.3454	442.2939	pos	3-(4-Hydroxyphenyl)-2-[[(9Z, 12Z,15Z)-octadeca-9,12,15-tri enoyl]amino]propanoic acid	C27H39NO4	441.2879	9867951	-	-	-	-

metab_9592	5.6128	442.3177	neg	Glucosylsphingosine	C24H47NO7	461.3353	5280570	Sphingolipids	Glycosphingolipids	HMDB0000596	Glucosylsphingosine
metab_5165	3.1191	443.2783	pos	Lys Phe Phe	C24H32N4O4	442.2604	71372064	-	-	-	-
metab_2180	3.5923	443.2785	pos	2-glyceryl-prostaglandin G2	C23H38O8	442.2572	91666454	Fatty Acyls	Eicosanoids	HMDB0062591	1,3-Dihydroxypropan-2-yl (5Z,9S,11R,13E,15S)-15-hydroperoxy-9,11-epidioxyprosta-5,13-dien-1-Oate
metab_799	8.7161	443.3144	pos	(3beta,5alpha,9alpha,22E,24R)-5,9-Epidioxy-3-hydroxyergosta-7,22-dien-6-one	C28H42O4	442.3083	131751275	Steroids and steroid derivatives	Ergostane steroids	HMDB0032666	(3beta,5alpha,9alpha,22E,24R)-5,9-Epidioxy-3-hydroxyergosta-7,22-dien-6-one
metab_6574	8.4821	443.3171	neg	1,24,25-Trihydroxyergocalciferol	C28H44O4	444.3240	9547253	Steroids and steroid derivatives	Vitamin D and derivatives	HMDB0006227	1-a,24R,25-Trihydroxyvitamin D2
metab_13822	2.1630	444.1730	neg	glipizide	C21H27N5O4S	445.1799	3478	Benzene and substituted derivatives	Benzenesulfonamides	HMDB0015200	CP 28,720
metab_8263	1.4427	445.1405	neg	Calycosin 7-O-Glucoside	C22H22O10	446.121297	5318267	-	-	-	-
metab_14871	0.5711	446.1520	neg	Dihydroxyfumitremorgin C	C22H25N3O5	411.1794	14019526	Indoles and derivatives	Pyridoindoles	HMDB0038581	Dihydroxyfumitremorgin C
metab_9963	7.4465	447.2530	neg	16alpha,17alpha-Dihydroxyprogesterone acetophenide	C29H36O4	448.2588	102011	-	-	-	-
metab_4595	5.6933	447.3095	pos	MC-207,110	C25H30N6O2	446.2433	443301	-	-	-	-
metab_7310	8.7031	449.2681	neg	Wilforol B	C29H38O4	450.2746	10366522	-	-	-	-
metab_5123	3.2420	449.2751	pos	Kaempferol-3-O-glucoside	C21H20O11	448.1011	5282102	Flavonoids	Flavonoid glycosides	HMDB0037429	3-O-b-D-Glucopyranosyloxy-4',5,7-trihydroxyflavone
metab_5123	3.2420	449.2751	pos	16alpha,17alpha-Dihydroxyprogesterone acetophenide	C29H36O4	448.2588	102011	-	-	-	-
metab_9964	7.4618	450.2635	neg	1-(9Z-Hexadecenoyl)-glycero-3-phosphoethanolamine	C21H42NO7P	451.2699	52925129	Glycerophospholipids	Glycerophosphoethanolamines	HMDB0011504	LysoPE(16:1(9Z)/0:0)
metab_4418	6.7062	450.3179	pos	3alpha,7alpha,12beta-Trihydro	C24H40O5	408.2876	5283869	Steroids and steroid	Bile acids, alcohols and	HMDB0000312	3a,7a,12b-Trihydroxy-5b-cholanoic

				xy-5beta-cholanic acid				derivatives	derivatives		acid
metab_9933	7.3017	452.2786	neg	LysoPE(0:0/16:0)	C21H44NO7P	453.2853	53480922	-	-	-	-
metab_7072	8.0998	452.2790	neg	LysoPE(0:0/16:0)	C21H44NO7P	453.2853	53480922	Glycerophospholipids	Glycerophosphoethanolamines	HMDB0011503	LysoPE(16:0/0:0)
metab_2450	5.2717	452.2973	pos	O-Hexadecanedioyl-L-carnitine	C23H43NO6	429.3090	44256593	Fatty Acyls	Fatty acid esters	HMDB0000712; LMFA07070007	Hexadecanedioic acid mono-L-carnitine ester
metab_3922	8.9507	452.3148	pos	N-Oleoyl phenylalanine	C27H43NO3	429.3243	52922059	Carboxylic acids and derivatives	Amino acids, peptides, and analogues	LMFA08020092; HMDB0062336	N-Oleoyl phenylalanine
metab_9364	4.3716	453.2617	neg	20,21,22,23-Tetrahydro-23-oxoazadirone	C28H38O5	454.2702	52952011	-	-	-	-
metab_12438	6.5829	453.2651	neg	CHEBI:67291	C28H38O5	454.2702	52952011	-	-	-	-
metab_2666	6.9638	453.3202	pos	lucialdehyde B	C30H44O3	452.3287	10343868	-	-	-	-
metab_2731	7.3216	454.2917	pos	LysoPE(0:0/16:0)	C21H44NO7P	453.2853	53480922	-	-	-	-
metab_22	8.0988	454.2917	pos	LysoPE(0:0/16:0)	C21H44NO7P	453.2853	53480922	Glycerophospholipids	Glycerophosphoethanolamines	LMGP02050002; HMDB0011503	LysoPE(16:0/0:0)
metab_4430	6.5857	454.2920	pos	LysoPE(0:0/16:0)	C21H44NO7P	453.2853	53480922	-	-	-	-
metab_2645	6.8728	454.2922	pos	LysoPE(0:0/16:0)	C21H44NO7P	453.2853	53480922	-	-	-	-
metab_8365	1.5736	455.0981	neg	Flavin mononucleotide	C17 H21 N4 O9 P	456.1051	643976	-	-	-	-
metab_1302	0.5983	455.1148	pos	Neurodazine	C27H21ClN2O3	454.1090	16112820	-	-	-	-
metab_8471	1.7572	455.1686	neg	Kushenol G	C25H28O8	456.1766	44259516	-	-	-	-
metab_8471	1.7572	455.1686	neg	Methotrexate	C20H22N8O5	456.1766	126941	-	-	-	-
metab_2314	4.3818	455.2781	pos	Withanolide B	C28H38O5	454.2719	14236711	Steroids and steroid derivatives	Steroid lactones	HMDB0030020	Withanolide B
metab_2568	6.1456	455.3535	pos	Glycyrrhetaldehyde	C30H46O3	454.3444	70698333	-	-	-	-

metab_10523	9.6316	455.3536	neg	Oleanolic acid	C30H48O3	456.3609	10494	-	-	-	-
metab_2962	8.8759	455.3874	pos	6-Hydroxy-8-heptacosanone	C27H54O2	410.4124	131751814	Fatty Acyls	Fatty alcohols	HMDB0035617	6-Hydroxy-8-heptacosanone
metab_6372	0.5983	457.1674	pos	Lysylpyridinoline	C18H28N4O7	412.1958	105071	Carboxylic acids and derivatives	Amino acids, peptides, and analogues	HMDB0000569	Deoxypyridinoline
metab_1558	1.3865	457.1903	pos	Kushenol G	C25H28O8	456.1766	44259516	-	-	-	-
metab_10179	8.2897	457.2965	neg	Calcipotriol	C27H40O3	412.2977	5288783	Steroids and steroid derivatives	Vitamin D and derivatives	HMDB0015567	Calcipotriol
metab_2975	8.9367	458.3466	pos	O-(17-Carboxyheptadecanoyl) carnitine	C25H47NO6	457.3402	71464574	-	-	-	-
metab_11720	8.7191	459.3122	neg	(1R,3R,6R,8S,12S,15R,16R)-15-(7-Hydroxy-6-methylhept-1-en-2-yl)-16-methylpentacyclo[9.7.0.01,3.03,8.012,16]octadec-10-ene-6,12-diol	C27H42O3	414.3134	101626006	Steroids and steroid derivatives	Hydroxysteroids	HMDB0041583	Setariol
metab_14530	1.1940	460.2058	neg	Pimozide	C28H29F2N3O	461.2262	16362	Benzene and substituted derivatives	Diphenylmethanes	HMDB0015232	Orap
metab_10021	7.6951	460.2838	neg	3-[(9E,12E)-Octadeca-9,12-dienoyloxy]-4-(trimethylazaniumyl)butanoate	C25H45NO4	423.3349	53477834	Fatty Acyls	Fatty acid esters	LMFA07070078; HMDB0006461	Linoelaidyl carnitine
metab_2740	7.3824	460.3262	pos	Palmitoyl glucuronide	C22H42O7	418.2931	161223	Fatty Acyls	Fatty acyl glycosides	HMDB0010331	Palmitoyl glucuronide
metab_13806	2.1956	461.2150	neg	Arg-Lys-Cys-Gly	C17H34N8O5S	462.2383	71464608	-	-	-	-
metab_10163	8.2099	461.3284	neg	3beta-Hydroxy-5-cholestenoate	C27H44O3	416.3290	53481407	Steroids and steroid derivatives	Bile acids, alcohols and derivatives	HMDB0012453	3 beta-Hydroxy-5-cholestenoate
metab_2095	3.2274	462.1894	pos	Trp-Glu-Gln	C21H27N5O7	461.1836	145458356	-	-	-	-
metab_12704	5.4522	463.2110	neg	Na-Hexanoyl-Nb-inosityltrypt	C23H32N2O8	464.2159	131752663	Carboxylic acids and	Amino acids, peptides,	HMDB0039501	Na-Hexanoyl-Nb-inosityltryptophan

				ophan				derivatives	and analogues		
metab_12176	7.5087	463.2473	neg	(-)-(6Z,12E,2S,3S,4R,5R,9S,11S,15R)-3-Cinnamoyloxylathyra-6,12-diene-5,15-diol-14-one	C29H36O5	464.2547	53355696	-	-	-	-
metab_12176	7.5087	463.2473	neg	(+)-(12E,2S,3S,4R,5R,6R,9S,11S,15R)-3-Cinnamoyloxy-5,6-epoxylathyr-12-en-15-ol-14-one	C29H36O5	464.2546	53355585	-	-	-	-
metab_13317	3.2473	463.2483	neg	(-)-(6Z,12E,2S,3S,4R,5R,9S,11S,15R)-3-Cinnamoyloxylathyra-6,12-diene-5,15-diol-14-one	C29H36O5	464.2547	53355696	-	-	-	-
metab_13317	3.2473	463.2483	neg	(+)-(12E,2S,3S,4R,5R,6R,9S,11S,15R)-3-Cinnamoyloxy-5,6-epoxylathyr-12-en-15-ol-14-one	C29H36O5	464.2546	53355585	-	-	-	-
metab_4801	4.6096	463.3045	pos	ponasterone A	C27H44O6	462.2955	115127	-	-	-	-
metab_13068	3.9850	465.2266	neg	Dolichyl b-D-glucosyl phosphate	C21H39O9P	466.2332	22833557	Prenol lipids	Sesquiterpenoids	HMDB0001054	Dolichyl b-D-glucosyl phosphate
metab_1895	2.4071	465.2336	pos	[(2S,3R,4S,5S,6R)-3,4,5-trihydroxy-6-(hydroxymethyl)oxan-2-yl] (E,8R)-8-[(2R,3R,5R,6S)-3,5-dihydroxy-6-methyloxan-2-yl]oxynon-2-enoate	C21H36O11	464.2261	86289875	-	-	-	-
metab_4010	8.5835	465.2964	pos	1-(11Z-Eicosenoyl)-glycero-3-phosphate	C23H45O7P	464.2903	52929759	-	-	LMGP10050026	

metab_4301	7.2014	468.3075	pos	1-Myristoyl-sn-glycero-3-phosphocholine	C22H46NO7P	467.3012	460604	-	-	LMGP01050012	
metab_786	8.5835	468.3671	pos	O-[(3R,11Z,14Z)-3-Hydroxyicosadienoyl]carnitine	C27H49NO5	467.3612	118796906	-	-	-	-
metab_10034	7.7251	470.2878	neg	Glycodeoxycholic acid	C26H43NO5	449.3141	3035026	Steroids and steroid derivatives	Bile acids, alcohols and derivatives	HMDB0000631; LMST05030006	Deoxycholic acid glycine conjugate
metab_12856	4.7546	471.2758	neg	Ixocarpanolide	C28H40O6	472.2825	14605184	Steroids and steroid derivatives	Steroid lactones	HMDB0033899	Ixocarpanolide
metab_6947	4.2203	471.2758	neg	Vamonolide	C28H40O6	472.2825	13939886	Steroids and steroid derivatives	Steroid lactones	HMDB0037379	Vamonolide
metab_8210	1.3231	472.1595	neg	Leucovorin	C20H23N7O7	473.1661	135403648	-	-	-	-
metab_8204	1.3231	473.1896	neg	Picrasin A	C26H34O8	474.2257	185611	-	-	-	-
metab_9401	4.5884	473.2912	neg	(1R,2S,4S,6R,7S,8R,9S,12S,13S,16S,18R)-16-Hydroxy-7,9,13-trimethyl-5'-methylidenespiro[5-oxapentacyclo[10.8.0.02,9.04,8.013,18]icosane-6,2'-oxane]-10-one	C27H40O4	428.2927	10502675	Prenol lipids	Triterpenoids	HMDB0035506	Schidigeragenin B
metab_12134	7.6323	473.2913	neg	25-Hydroxyvitamin d3-26,23-lactone	C27H40O4	428.2927	131769836	Steroids and steroid derivatives	Vitamin D and derivatives	HMDB0060126	25-Hydroxyvitamin D3-26,23-lactone
metab_6974	4.9510	473.2914	neg	NA	C28H42O6	474.2981	NA	Steroids and steroid derivatives	Steroid lactones	LMPR0104300001;HMDB0030085	Pubescenol
metab_9923	7.2377	474.2630	neg	Ulipristal acetate	C30H37NO4	475.2691	130904	Prenol lipids	Terpene glycosides	HMDB0041085	Sambutoxin
metab_4810	4.5785	475.3044	pos	Riesling acetal	C13H22O3	226.1569	57473083	Tetrahydrofurans	-	HMDB0037562	Riesling acetal
metab_11664	8.8449	475.3072	neg	(25R)-3beta-Hydroxycholest-5	C27H42O4	430.3083	91828282	Steroids and steroid	Bile acids, alcohols and	HMDB0062613	(25R)-3beta-hydroxycholest-5-en-7-

				-en-7-one-26-oate				derivatives	derivatives		one-26-oate
metab_10236	8.5135	475.3073	neg	(1S)-1,25-Dihydroxy-24-oxocalciol	C27H42O4	430.3083	5283703	Steroids and steroid derivatives	Vitamin D and derivatives	HMDB0060128	24-Oxo-1alpha,25-dihydroxyvitamin D3
metab_2713	7.2314	476.2761	pos	Ulipristal acetate	C30H37NO4	475.2691	130904	-	-	-	-
metab_4555	5.9361	476.2762	pos	PE(18:3(6Z,9Z,12Z)/0:0)	C23H42NO7P	475.2699	52925136	Glycerophospholipids	Glycerophosphoethanolamines	HMDB0011508	LysoPE(18:3(6Z,9Z,12Z)/0:0)
metab_4555	5.9361	476.2762	pos	Ulipristal acetate	C30H37NO4	475.2691	130904	Glycerophospholipids	Glycerophosphoethanolamines	HMDB0011508	LysoPE(18:3(6Z,9Z,12Z)/0:0)
metab_7371	7.6793	476.2789	neg	Aminopotentidine	C26H35N7O2	477.2854	164435	-	-	-	-
metab_7371	7.6793	476.2789	neg	LysoPE(0:0/18:2(9Z,12Z))	C23H44NO7P	477.2854	53480926	-	-	-	-
metab_4907	4.0939	476.2854	pos	Ulipristal acetate	C30H37NO4	475.2691	130904	-	-	-	-
metab_1749	1.9609	476.3053	pos	Netilmicin	C21H41N5O7	475.2995	441306	-	-	-	-
metab_14066	1.7572	477.1996	neg	Kanokoside B	C21H34O12	478.2061	46173906	-	-	-	-
metab_11922	8.1936	478.2661	neg	Glycerophospho-N-Oleoyl Ethanolamine	C23H46NO7P	479.3017	16123469	-	-	-	-
metab_987	6.9477	478.2918	pos	Aminopotentidine	C26H35N7O2	477.2854	164435	-	-	-	-
metab_987	6.9477	478.2918	pos	LysoPE(0:0/18:2(9Z,12Z))	C23H44NO7P	477.2854	53480926	-	-	-	-
metab_121	7.6776	478.2918	pos	Aminopotentidine	C26H35N7O2	477.2854	164435	-	-	-	-
metab_121	7.6776	478.2918	pos	LysoPE(0:0/18:2(9Z,12Z))	C23H44NO7P	477.2854	53480926	-	-	-	-
metab_7338	8.1936	478.2949	neg	Glycerophospho-N-Oleoyl Ethanolamine	C23H46NO7P	479.3017	16123469	-	-	-	-
metab_4112	8.1857	480.3078	pos	1-(9Z-Octadecenoyl)-sn-glycero-3-phosphoethanolamine	C23H46NO7P	479.3012	9547071	-	-	LMGP02050004	
metab_12201	7.4148	482.2533	neg	Trimethylsilyl l-Alanine	C6H15NO2Si	161.0872	11008231	Carboxylic acids and derivatives	Amino acids, peptides, and analogues	HMDB0094699	Trimethylsilyl l-Alanine

metab_6937	3.9850	483.2372	neg	Retinyl beta-glucuronide	C26H38O7	462.2618	6440956	Prenol lipids	Terpene glycosides	HMDB0010340	Retinyl beta-glucuronide
metab_9999	7.6009	483.2726	neg	Stigmatellin Y	C29H40O6	484.2800	5282078	-	-	-	-
metab_3901	9.0397	483.3800	pos	2-Hydroxy-3-methoxyestrone	C25H47NO5	300.1725	53480676	Hydroxy acids and derivatives	Beta hydroxy acids and derivatives	HMDB0011195	2-Hydroxy-3-methoxyestrone
metab_2212	3.7447	484.3007	pos	1-Hexadecyl-glycero-3-phosphoserine	C22H46NO8P	483.2961	52926303	-	-	LMGP03060003	
metab_4043	8.4214	486.3567	pos	Isokobusone	C14H22O2	222.1620	3860435	Organooxygen compounds	Alcohols and polyols	HMDB0036791	Isokobusone
metab_9360	4.3716	487.2709	neg	Muzanzagenin	C27H38O5	442.2719	10836928	Steroids and steroid derivatives	Oxosteroids	HMDB0032601	Muzanzagenin
metab_591	4.3818	489.2837	pos	3alpha,7alpha,12alpha-Trihydroxy-5beta-cholestan-26-oic acid	C27H46O5	450.3345	122312	Steroids and steroid derivatives	Bile acids, alcohols and derivatives	HMDB0000601	Coprocholic acid
metab_2197	3.6835	489.2838	pos	2,3-Dihydrowithanolide E	C28H40O7	488.2774	131751517	Steroids and steroid derivatives	Steroid lactones	HMDB0034057	2,3-Dihydrowithanolide E
metab_531	3.3490	491.2996	pos	CID 9983050	C28H42O7	490.2931	9983050	Prenol lipids	Sesquiterpenoids	HMDB0041587	Macrocarpal I
metab_12873	4.6722	491.3024	neg	Spirotaccagenin	C27H42O5	446.3032	14539254	Prenol lipids	Triterpenoids	HMDB0034424	Spirotaccagenin
metab_4273	7.3521	494.3231	pos	1-(9Z-Hexadecenoyl)-sn-glycero-3-phosphocholine	C24H48NO7P	493.3168	24779461	Glycerophospholipids	Glycerophosphocholines	HMDB0010383	LysoPC(16:1(9Z)/0:0)
metab_9488	4.9510	495.3335	neg	Polypodine B	C27H44O8	496.3034	441833	-	-	-	-
metab_10005	7.6323	496.2326	neg	14alpha-Hydroxypaxilline	C27H33NO5	451.2359	85141768	Naphthopyrans	-	HMDB0040978	14alpha-Hydroxypaxilline
metab_752	7.9980	496.3387	pos	1-O-Palmitoyl-sn-glycero-3-phosphocholine	C24H50NO7P	495.3322	460603	-	-	-	-
metab_752	7.9980	496.3387	pos	NA	C24H50NO7P	495.3323	NA	-	-	-	-
metab_11443	9.5314	497.2583	neg	(14alpha,17beta,20S,22R)-14,20-Epoxy-17-hydroxy-1-oxow	C28H36O5	452.2563	73699089	Steroids and steroid derivatives	Steroid lactones	HMDB0032685	(14alpha,17beta,20S,22R)-14,20-Epoxy-17-hydroxy-1-oxowitha-3,5,24-

				itha-3,5,24-trienolide							trienolide
metab_2380	4.7607	497.3362	pos	Polypodine B	C27H44O8	496.3034	441833	-	-	-	-
metab_10576	9.8947	499.2742	neg	Euglobal IVa	C28H38O5	454.2719	131750946	Benzopyrans	1-benzopyrans	HMDB0030035	Euglobal IVa
metab_11630	8.9574	499.3649	neg	Cryptocaryol E	C28H52O7	500.3720	54585961	-	-	-	-
metab_4582	5.7846	502.3152	pos	Lucidenic acid N	C27H40O6	460.2825	21592283	Prenol lipids	Triterpenoids	HMDB0038352	Lucidenic acid N
metab_14646	0.9227	503.1626	neg	Panose	C18H32O16	504.1696	94448	-	-	-	-
metab_8170	1.2805	503.1627	neg	Panose	C18H32O16	504.1696	94448	-	-	-	-
metab_13200	3.5978	503.2660	neg	Lucidenic acid A	C27H38O6	458.2668	14109375	Prenol lipids	Triterpenoids	HMDB0037611	Lucidenic acid A
metab_9496	5.0159	503.3023	neg	Pubesenolide	C28H42O5	458.3032	72999858	Steroids and steroid derivatives	Steroid lactones	HMDB0033728	Pubesenolide
metab_13842	2.1311	505.1574	neg	Methyl salicylate O-[rhamnosyl-(1->6)-glucoside]	C20H28O12	460.1581	131751380	Organooxygen compounds	Carbohydrates and carbohydrate conjugates	HMDB0033138	Methyl salicylate O-[rhamnosyl-(1|right|6)-glucoside]
metab_12251	7.1903	505.2587	neg	Amoritin	C31H38O6	506.2652	42608008	-	-	-	-
metab_6613	3.6812	505.2818	neg	(5alpha,6beta,14alpha,20R,22R)-5,6,14,20,27-Pentahydroxy-1-oxowith-24-enolide	C28H42O8	506.2880	78385401	Steroids and steroid derivatives	Steroid lactones	HMDB0033198	(5alpha,6beta,14alpha,20R,22R)-5,6,14,20,27-Pentahydroxy-1-oxowith-24-enolide
metab_7408	6.2294	505.3024	neg	1,2-Dioctanoyl-3-beta-d-galactosyl-sn-glycerol	C25H46O10	506.3098	68445225	-	-	-	-
metab_7396	6.7122	505.3025	neg	1,2-Dioctanoyl-3-beta-d-galactosyl-sn-glycerol	C25H46O10	506.3098	68445225	-	-	-	-
metab_12561	6.0670	505.3028	neg	1,2-Dioctanoyl-3-beta-d-galactosyl-sn-glycerol	C25H46O10	506.3098	68445225	-	-	-	-
metab_68	3.1191	507.2947	pos	Bicyclo(4.1.0)heptan-3-one, 1-methyl-4-(1-methylethylidene)-7-(3-oxobutyl)-,	C15H22O2	234.1620	153845	Prenol lipids	Sesquiterpenoids	LMPR0103400001;HMDB0033253	Curcumenone

				(1S,6R,7R)-							
metab_12374	6.8565	507.3332	neg	Polyporusterone F	C28H46O5	462.3345	44575603	Steroids and steroid derivatives	Bile acids, alcohols and derivatives	HMDB0038499	Polyporusterone F
metab_4203	7.7514	508.3391	pos	1-(9Z-Heptadecenoyl)-glycero-3-phosphocholine	C25H50NO7P	507.3325	24779451	-	-	LMGP01050126	
metab_9994	7.5855	509.2891	neg	Longirostrerone B	C31H42O6	510.2959	56833383	Glycerophospholipids	Glycerophosphates	HMDB0062305; LMGP10050026	1-(11Z-eicosenoyl)-glycero-3-phosphate
metab_5086	3.3793	509.3103	pos	[(2S,3R,4S,5S,6R)-3,4,5-trihydroxy-6-(hydroxymethyl)oxan-2-yl] (11R)-11-[(2R,3R,5R,6S)-3,5-dihydroxy-6-methyloxan-2-yl]oxydodecanoate	C24H44O11	508.2887	86289879	-	-	-	-
metab_7383	7.0959	509.3494	neg	Trihydroxycoprostanoic acid	C28H48O5	464.3502	122166	Steroids and steroid derivatives	Cholestane steroids	HMDB0002163; LMST01010246; HMDB0000601	Trihydroxycoprostanoic acid;Coprocholic acid
metab_4065	8.3463	510.3546	pos	1-Heptadecanoyl-sn-glycero-3-phosphocholine	C25H52NO7P	509.3473	24779463	-	-	-	-
metab_2205	3.6987	510.3777	pos	1-heptadecanoyl-sn-glycero-3-phosphocholine	C25H52NO7P	509.3473	24779463	Glycerophospholipids	Carbonyl compounds	HMDB0012108	LysoPC(17:0)
metab_12717	5.3713	512.3002	neg	1-Heptadecanoyl-sn-glycero-3-phosphoethanolamine	C22H46NO7P	467.3012	52925149	Glycerophospholipids	Glycerophosphoethanolamines	HMDB0061691	1-Heptadecanoylglycerophosphoethanolamine
metab_8081	1.0785	515.1986	neg	Formononetin 7-O-glucoside-6"-O-malonate	C25H24O12	516.1266	23724663	-	-	-	-
metab_2580	6.2978	517.2412	pos	hesperadin	C29H32N4O3S	516.2219	135421442	-	-	-	-

metab_2580	6.2978	517.2412	pos	Formononetin 7-O-glucoside-6"-O-malonate	C25H24O12	516.1266	23724663	-	-	-	-
metab_12338	6.9354	518.2531	neg	LysoPE(0:0/18:4(6Z,9Z,12Z,15Z))	C23H40NO7P	473.2542	53480929	Glycerophospholipids	Glycerophosphoethanolamines	HMDB0011480; LMGP02050044	LysoPE(0:0/18:4(6Z,9Z,12Z,15Z))
metab_12265	7.1595	518.2897	neg	Vignatic acid B	C27H41N3O7	519.2945	85261327	Carboxylic acids and derivatives	Amino acids, peptides, and analogues	HMDB0033617	Vignatic acid B
metab_2707	7.1701	518.3230	pos	LysoPC(18:3(9Z,12Z,15Z))	C26H48NO7P	517.3168	24779469	Glycerophospholipids	Glycerophosphocholines	HMDB0010388	LysoPC(18:3(9Z,12Z,15Z))
metab_2707	7.1701	518.3230	pos	LysoPC(18:3(9Z,12Z,15Z))	C26H48NO7P	517.3168	24779469	Glycerophospholipids	Glycerophosphocholines	HMDB0010388	LysoPC(18:3(9Z,12Z,15Z))
metab_11066	14.0282	520.2688	neg	LysoPE(0:0/18:3(9Z,12Z,15Z))	C23H42NO7P	475.2699	53480928	Glycerophospholipids	Glycerophosphoethanolamines	LMGP02050043; HMDB0011479	LysoPE(0:0/18:3(9Z,12Z,15Z))
metab_980	7.5892	520.3387	pos	2-Linoleoyl-sn-glycero-3-phosphocholine	C26H50NO7P	519.3325	71768169	-	-	-	-
metab_13208	3.5813	521.2767	neg	Lucidenic acid G	C27H40O7	476.2774	14109386	Prenol lipids	Triterpenoids	HMDB0035599	Lucidenic acid G
metab_10050	7.7875	522.2846	neg	1-(9Z-Octadecenoyl)-sn-glycero-3-phosphoserine	C24H46NO9P	523.2910	9547099	-	-	LMGP03050001	
metab_116	8.0988	522.3544	pos	LysoPC(18:1(11Z))	C26H52NO7P	521.3481	53480465	-	-	-	-
metab_7467	1.5155	523.1677	neg	Isoglobotriaose	C19H34O15	502.1898	53477863	Fatty Acyls	Fatty acyl glycosides	HMDB0006598	Isoglobotriaose
metab_12952	4.3716	523.2915	neg	Erinacine D	C27H42O7	478.2931	9912965	Prenol lipids	Diterpenoids	HMDB0031896	Erinacine D
metab_9561	5.4188	523.3283	neg	CID 10814524	C28H46O6	478.3294	10814524	Steroids and steroid derivatives	Bile acids, alcohols and derivatives	HMDB0038495	Polyporusterone A
metab_4237	7.5892	523.3469	pos	Carpaine	C28H50N2O4	478.3771	442630	Macrolides and analogues	-	HMDB0030272	Carpaine
metab_3978	8.6717	524.3696	pos	2-Octadecanoyl-sn-glycero-3-phosphocholine	C26H54NO7P	523.3637	24779491	-	-	-	-
metab_12138	7.6169	526.3160	neg	NA	C23H48NO7P	481.3168	24779458	Glycerophospholipids	Glycerophosphocholines	HMDB0010381	LysoPC(15:0)
metab_2883	8.4074	528.4037	pos	3,7-Dihydroxy-25-methoxycuc	C31H50O4	486.3709	14807340	Steroids and steroid	Cucurbitacins	HMDB0039362	3,7-Dihydroxy-25-methoxycucurbit

				urbita-5,23-dien-19-al				derivatives			a-5,23-dien-19-al
metab_10096	7.9757	531.3189	neg	3-O-(alpha-L-Olivosyl)erythronolide B	C27H48O10	532.3253	56927754	-	-	-	-
metab_2535	5.9208	534.3181	pos	LysoPE(20:4(8Z,11Z,14Z,17Z)/0:0)	C25H44NO7P	501.2855	53480952	Glycerophospholipids	Glycerophosphoethanolamines	HMDB0011518; LMGP02050067	LysoPE(20:4(8Z,11Z,14Z,17Z)/0:0)
metab_13140	3.7658	535.2922	neg	Corchorosol A	C29H44O9	536.2985	4254782	Steroids and steroid derivatives	Steroid lactones	HMDB0041137	Corchorosol A
metab_9938	7.3343	535.3041	neg	1-(13Z,16Z-Docosadienoyl)-glycero-3-phosphate	C25H47O7P	490.3059	52929763	Glycerophospholipids	Glycerophosphates	HMDB0114751; HMDB0062306; LMGP10050030	LysoPA(22:2(13Z,16Z)/0:0);1-(13Z,16Z-docosadienoyl)-glycero-3-phosphate
metab_3692	9.9307	535.4711	pos	Palmitic acid	C16H32O2	256.2402	985	Fatty Acyls	Fatty acids and conjugates	HMDB0000220; LMFA01010001	Palmitic acid
metab_2872	8.3322	536.3710	pos	CHEBI:68080	C28H49N5O5	535.372	53355999	-	-	-	-
metab_7132	9.7965	537.4533	neg	1-Myristoyl-2-palmitoleoyl-sn-glycerol	C33H62O5	538.4597	53477950	Glycerolipids	Diradylglycerols	HMDB0007012	DG(14:0/16:1(9Z)/0:0)
metab_9940	7.3508	538.3164	neg	1-(9Z-Hexadecenoyl)-sn-glycero-3-phosphocholine	C24H48NO7P	493.3168	24779461	Glycerophospholipids	Glycerophosphocholines	HMDB0010383	LysoPC(16:1(9Z)/0:0)
metab_10587	9.9433	539.4691	neg	1,2-Dipentadecanoyl-sn-glycerol	C33H64O5	540.4754	18642216	Glycerolipids	Diradylglycerols	LMGL02010326;HMDB0007068	DG(15:0/15:0/0:0)
metab_6939	4.0516	540.3119	neg	LysoPC(20:5(5Z,8Z,11Z,14Z,17Z))	C28H48NO7P	541.3168	11757087	Glycerophospholipids	Glycerophosphocholines	HMDB0010397	LysoPC(20:5(5Z,8Z,11Z,14Z,17Z))
metab_7359	8.0069	540.3315	neg	1-Palmitoyl-sn-glycero-3-phosphocholine	C24H50NO7P	495.3325	460602	Glycerophospholipids	Glycerophosphocholines	HMDB0010382; HMDB0240262	LysoPC(16:0);LysoPC(0:0/16:0)
metab_10054	7.7875	543.2266	neg	10-Deacetylbaccatin III	C29H36O10	544.2320	154272	-	-	-	-
metab_12012	7.9908	543.2271	neg	10-Deacetylbaccatin III	C29H36O10	544.2320	154272	-	-	-	-
metab_12213	7.3508	548.3002	neg	LysoPE(20:3(11Z,14Z,17Z)/0:	C25H46NO7P	503.3012	53480950	Glycerophospholipids	Glycerophosphoethanola	LMGP02050065;	LysoPE(20:3(11Z,14Z,17Z)/0:0)

				0)					mines	HMDB0011514	
metab_110	10.2085	548.5026	pos	N-(2R-Hydroxyhexadecanoyl)-2S-amino-9-methyl-4E,8E-octadecadiene-1,3R-diol	C35H67NO4	565.5070	131752781	Sphingolipids	Ceramides	HMDB0040132	N-(2R-Hydroxyhexadecanoyl)-2S-amino-9-methyl-4E,8E-octadecadiene-1,3R-diol
metab_13164	3.6980	551.2874	neg	Desglucocheirotoxol	C29H44O10	552.2934	12309172	Steroids and steroid derivatives	Steroid lactones	HMDB0033828	Desglucocheirotoxol
metab_7234	9.9767	551.4691	neg	1-Pentadecanoyl-2-(9Z-hexadecenoyl)-sn-glycerol	C34H64O5	552.4754	53478003	Glycerolipids	Diradylglycerols	HMDB0007070	DG(15:0/16:1(9Z)/0:0)
metab_883	10.3480	551.5023	pos	1,2-Dipalmitoyl-sn-glycerol	C35H68O5	568.5067	644078	Glycerolipids	Diradylglycerols	HMDB0007098; LMGL02010009	DG(16:0/16:0/0:0)
metab_2155	3.4857	552.3519	pos	Janthitrem C	C37H47NO4	569.3505	156106	Naphthopyrans	-	HMDB0040684	Janthitrem C
metab_3691	9.9307	553.4818	pos	1-(9Z-Pentadecenoyl)-2-hexadecanoyl-sn-glycerol	C34H64O5	552.4754	56936366	-	-	LMGL02010450	
metab_7142	10.0749	553.4848	neg	1-Pentadecanoyl-2-hexadecanoyl-sn-glycerol	C34H66O5	554.4910	14275229	Glycerolipids	Diradylglycerols	HMDB0007069	DG(15:0/16:0/0:0)
metab_558	2.2045	555.2504	pos	Pantethine	C22H42N4O8S2	554.2444	452306	Carboxylic acids and derivatives	Amino acids, peptides, and analogues	HMDB0003828	D-Pantethine
metab_726	7.4269	555.2911	pos	Acrovestone	C32H42O8	554.2854	159969	-	-	-	-
metab_4790	4.6398	556.3044	pos	3-Hydroxydodecanedioic acid	C12H22O5	246.1467	16663321	Hydroxy acids and derivatives	Medium-chain hydroxy acids and derivatives	HMDB0000413	3-Hydroxydodecanedioic acid
metab_2954	8.8169	557.4553	pos	Punicic acid	C18H30O2	278.2246	5281126	Lineolic acids and derivatives	-	LMFA01030146; HMDB0030963	Punicic acid
metab_2989	9.0254	559.4711	pos	1-Pentadecanoyl-2-(9Z,12Z,15Z-octadecatrienoyl)-sn-glycerol	C36H64O5	576.4754	14275371	Fatty Acyls	Lineolic acids and derivatives	HMDB0007076	DG(15:0/18:3(9Z,12Z,15Z)/0:0)
metab_9904	7.1595	562.3153	neg	LysoPC(18:3(6Z,9Z,12Z))	C26H48NO7P	517.3168	52924045	Glycerophospholipids	Glycerophosphocholines	HMDB0010387	LysoPC(18:3(6Z,9Z,12Z))

metab_9993	7.5855	564.3313	neg	1-Linoleoyl-sn-glycero-3-phosphocholine	C26H50NO7P	519.3325	11005824	Glycerophospholipids	Glycerophosphocholines	HMDB0010386	LysoPC(18:2(9Z,12Z))
metab_9911	7.1749	565.2792	neg	NA	C24H36N8O8	566.2864	NA	-	-	-	-
metab_9883	7.0469	565.2799	neg	NA	C24H36N8O8	566.2864	NA	-	-	-	-
metab_11424	9.5979	567.4639	neg	Muricatacin	C17H32O3	284.2351	10016749	-	-	HMDB0038685; LMFA05000682	Muricatacin
metab_10284	8.6728	568.3619	neg	1-Stearoyl-sn-glycero-3-phosphocholine	C26H54NO7P	507.3689	497299	Glycerophospholipids	Glycerophosphocholines	HMDB0013122; HMDB0010384; HMDB0011128	LysoPC(P-18:0);LysoPC(18:0);LysoPC(0:0/18:0)
metab_12199	7.4300	571.2892	neg	1-Hexadecanoyl-sn-glycero-3-phospho-(1'-myo-inositol)	C25H49O12P	572.2964	71296207	Glycerophospholipids	Glycerophosphoinositols	HMDB0061695	1-Palmitoylglycerophosphoinositol
metab_12350	6.8887	571.2895	neg	1-Hexadecanoyl-sn-glycero-3-phospho-(1'-myo-inositol)	C25H49O12P	572.2964	71296207	-	-	-	-
metab_2972	8.9367	573.4861	pos	DG(16:0/18:3(9Z,12Z,15Z)/0:0)	C37H66O5	590.4910	9543700	Fatty Acyls	Lineolic acids and derivatives	HMDB0007105	DG(16:0/18:3(9Z,12Z,15Z)/0:0)
metab_3776	9.6358	573.4865	pos	DG(18:3(9Z,12Z,15Z)/16:0/0:0)	C37H66O5	590.4910	53478143	Fatty Acyls	Lineolic acids and derivatives	HMDB0007301	DG(18:3(9Z,12Z,15Z)/16:0/0:0)
metab_3694	9.9307	573.4867	pos	Artemoin A	C35H66O4	550.4961	129011028	Fatty Acyls	Fatty alcohols	HMDB0033604	Artemoin A
metab_10547	9.7470	575.4691	neg	DG(15:0/18:3(6Z,9Z,12Z)/0:0)	C36H64O5	576.4754	53478005	Fatty Acyls	Lineolic acids and derivatives	HMDB0007075	DG(15:0/18:3(6Z,9Z,12Z)/0:0)
metab_898	10.1647	575.5021	pos	1-Linoleoyl-2-palmitoyl-sn-glycerol	C37H68O5	592.5067	53478101	Fatty Acyls	Lineolic acids and derivatives	HMDB0007243	DG(18:2(9Z,12Z)/16:0/0:0)
metab_3753	9.7283	575.5021	pos	1-Palmitoyl-2-linoleoyl-sn-glycerol	C37H68O5	592.5067	9543695	Fatty Acyls	Lineolic acids and derivatives	HMDB0007103	DG(16:0/18:2(9Z,12Z)/0:0)
metab_12121	7.6793	576.2040	neg	NA	C35H33NO8	595.2206	NA	Quinolines and derivatives	Benzoquinolines	HMDB0040386	Acrimarine J

metab_107	9.6823	577.4817	pos	1-(9Z-Pentadecenoyl)-2-(9Z,12Z-octadecadienoyl)-sn-glycerol	C36H64O5	576.4754	56936373	-	-	LMGL02010457	
metab_11332	9.9108	577.4847	neg	1-Pentadecanoyl-2-(9Z,12Z-octadecadienoyl)-sn-glycerol	C36H66O5	578.4910	14275369	Fatty Acyls	Lineolic acids and derivatives	HMDB0007074	DG(15:0/18:2(9Z,12Z)/0:0)
metab_3211	10.9772	577.5176	pos	1-Hexadecanoyl-2-(11Z-octadecenoyl)-sn-glycerol	C37H70O5	594.5223	9543972	Glycerolipids	Diradylglycerols	LMGL02010307;HMDB0007101	DG(16:0/18:1(11Z)/0:0)
metab_11293	10.0593	579.5004	neg	1-Pentadecanoyl-3-vaccenoyl-sn-glycerol	C36H68O5	580.5067	131801716	Glycerolipids	Diradylglycerols	HMDB0055989	DG(15:0/0:0/18:1n7)
metab_3656	10.1331	580.5288	pos	N-(2-Hydroxy-eicosanoyl)-4E,6E-hexadecasphingadienine	C36H69NO4	579.5227	70698976	-	-	LMSP02010089	
metab_3182	10.3942	582.5445	pos	N-(2-Hydroxy-eicosanoyl)-hexadecasphing-4-enine-1-phosphoethanolamine	C36H71NO4	581.5383	70699065	-	-	LMSP02010084	
metab_12789	4.9838	587.3130	neg	Tyr-Ile	C15H22N2O4	294.1580	7019110	Carboxylic acids and derivatives	Amino acids, peptides, and analogues	HMDB0029108	Tyrosyl-Isoleucine
metab_11721	8.7191	591.4641	neg	Diepomuricanin A	C35H62O4	546.4648	73826292	Fatty Acyls	Fatty alcohols	HMDB0040921	Diepomuricanin A
metab_3713	9.8673	591.4971	pos	1-Palmitoleoyl-2-linoleoyl-sn-glycerol	C37H66O5	590.4912	9543699	-	-	LMGL02010032	
metab_9511	5.1296	593.1309	neg	Tiliroside	C30H26O13	594.1383	5320686	-	-	-	-
metab_14643	0.9368	593.1944	neg	2-Phenyl-3-(2-furyl)prop-2-enal	C13H10O2	198.0681	6435876	Benzene and substituted derivatives	Phenylacetaldehydes	HMDB0036185	3-(2-Furanyl)-2-phenyl-2-propenal
metab_11634	8.9574	593.4798	neg	(2S)-4-[(Z,13R,14R)-13,14-Dihydroxytriacont-17-enyl]-2-methyl-2H-furan-5-one	C35H64O4	548.4805	101729791	Fatty Acyls	Fatty alcohols	HMDB0031168	Cohibin A
metab_9840	6.9354	595.2897	neg	Salannin	C34H44O9	596.2971	6437066	-	-	-	-

metab_10842	14.1581	595.2899	neg	Salannin	C34H44O9	596.2971	6437066	-	-	-	-
metab_12675	5.5966	595.2899	neg	Salannin	C34H44O9	596.2971	6437066	-	-	-	-
metab_9566	5.4522	595.2917	neg	Salannin	C34H44O9	596.2971	6437066	-	-	-	-
metab_7389	6.9834	597.3044	neg	NA	C27H51O12P	598.3118	86289645	Glycerophospholipids	Glycerophosphoinositols	HMDB0061693	1-Oleoylglycerophosphoinositol
metab_12610	5.8731	597.3170	neg	p-Coumaroylagmatine	C14H20N4O2	276.1586	5280691	Cinnamic acids and derivatives	Hydroxycinnamic acids and derivatives	HMDB0033460	4-Hydroxycinnamoylagmatine
metab_829	9.3429	597.4487	pos	DG(15:0/18:4(6Z,9Z,12Z,15Z)/0:0)	C36H62O5	574.4597	53478006	Fatty Acyls	Lineolic acids and derivatives	HMDB0007077	DG(15:0/18:4(6Z,9Z,12Z,15Z)/0:0)
metab_3830	9.3590	597.4875	pos	DG(18:1(11Z)/18:4(6Z,9Z,12Z,15Z)/0:0)	C39H66O5	614.4910	53478068	Fatty Acyls	Lineolic acids and derivatives	HMDB0007193	DG(18:1(11Z)/18:4(6Z,9Z,12Z,15Z)/0:0)
metab_3638	10.2404	599.5021	pos	DG(18:1(11Z)/18:3(9Z,12Z,15Z)/0:0)	C39H68O5	616.5067	53478067	Fatty Acyls	Lineolic acids and derivatives	HMDB0007192	DG(18:1(11Z)/18:3(9Z,12Z,15Z)/0:0)
metab_7656	0.5286	606.0755	neg	Uridine diphosphate-N-acetylglucosamine	C17H27N3O17P2	607.0816	10705	Pyrimidine nucleotides	Pyrimidine nucleotide sugars	HMDB0000290	Uridine diphosphate-N-acetylglucosamine
metab_3783	9.5899	606.4483	pos	LysoPC(24:1(15Z))	C32H64NO7P	605.4420	53480477	Glycerophospholipids	Glycerophosphocholines	HMDB0010406; LMGP01050144	LysoPC(24:1(15Z))
metab_11881	8.3373	607.4591	neg	DG(14:0/18:3(6Z,9Z,12Z)/0:0)	C35H62O5	562.4597	53477953	Fatty Acyls	Lineolic acids and derivatives	HMDB0007017	DG(14:0/18:3(6Z,9Z,12Z)/0:0)
metab_803	8.8318	607.4920	pos	(2S)-2-Methyl-4-[(8R,15S,17R)-8,15,17-trihydroxy-17-[(2R,5R)-5-[(1R)-1-hydroxyundecyl]oxolan-2-yl]heptadecyl]-2H-furan-5-one	C37H68O7	624.4965	10258405	Fatty Acyls	Fatty alcohols	HMDB0035389	Asitrilobin D
metab_11835	8.5135	609.4745	neg	cis-Solamin	C35H64O5	564.4754	11376469	Fatty Acyls	Fatty alcohols	HMDB0032732	cis-Solamin
metab_286	1.1875	611.1707	pos	Okanin 4-methyl ether	C31H30O13	610.1647	42607578	-	-	-	-

				4'-O-(6"-O-p-coumaroylglucoside)							
metab_10290	8.6874	611.4899	neg	1-Palmitoyl-3-palmitoleoyl-sn-glycerol	C35H66O5	566.4910	131801738	Glycerolipids	Diradylglycerols	HMDB0056015	DG(16:0/0:0/16:1n7)
metab_800	8.7303	613.4815	pos	2,3-Dilinolenoyl-sn-glycerol	C39H64O5	612.4751	71768122	-	-	-	-
metab_4168	7.9695	616.3444	pos	(6r)-6-[(3s,10s,13r,14r,15r,17r)-12-Acetoxy-3,15-dihydroxy-4,4,10,13,14-pentamethyl-7,11-dioxo-2,3,5,6,12,15,16,17-octahydro-1h-cyclopenta[a]phenanthren-17-yl]-2-methyl-4-oxo-heptanoic acid	C32H46O9	574.3142	471001	Prenol lipids	Triterpenoids	HMDB0033024	Ganoderic acid alpha
metab_4857	4.3672	618.3043	pos	Methionyl-Lysine	C11H23N3O3S	277.1460	7016112	Carboxylic acids and derivatives	Amino acids, peptides, and analogues	HMDB0028978	Methionyl-Lysine
metab_3769	9.6521	619.4906	pos	Ricinoleic acid	C18H34O3	298.2508	643684	Fatty Acyls	Fatty acids and conjugates	HMDB0034297; LMFA02000184	Ricinoleic acid
metab_2879	8.3763	620.3869	pos	3beta,15alpha-Diacetoxylanosta-8,24-dien-26-oic acid	C34H52O6	556.3764	131751991	Prenol lipids	Triterpenoids	HMDB0036442	3beta,15alpha-Diacetoxylanosta-8,24-dien-26-oic acid
metab_11974	8.0843	625.3455	neg	Lansioside C	C35H56O7	588.4026	73816952	Organooxygen compounds	Carbohydrates and carbohydrate conjugates	HMDB0035103	Lansioside C
metab_10061	7.8352	625.4691	neg	(2S)-2-Methyl-4-[12-[(2S,5R)-5-[(1R)-1,6,7-trihydroxytetradecyl]oxolan-2-yl]dodecyl]-2H-furan-5-one	C35H64O6	580.4703	10076949	Fatty Acyls	Fatty alcohols	HMDB0039453	Muricin H
metab_11580	9.1038	627.4410	neg		C40H62O4	606.4648	131819631	-	-	-	-
metab_11929	8.1936	635.3810	neg	(24R)-5b,8b-Epidioxyergosta-	C34H54O8	590.3819	56673984	Steroids and steroid	Steroidal glycosides	HMDB0037956	(24R)-5b,8b-Epidioxyergosta-6,22E

				6,22E-dien-3b-ol 3-glucoside				derivatives			-dien-3b-ol 3-glucoside
metab_4268	7.3824	636.3822	pos	Ganoderic acid Ma	C34H52O7	572.3713	131751707	Prenol lipids	Triterpenoids	HMDB0035329	Ganoderic acid Ma
metab_7337	8.3373	637.3967	neg	CID 70685270	C34H56O8	592.3975	70685270	Steroids and steroid derivatives	Steroidal glycosides	HMDB0039054	Tuberoside
metab_736	7.6776	638.3974	pos	L-Stercobilinogen	C33H48N4O6	596.3574	440783	Tetrapyrroles and derivatives	Bilirubins	HMDB0004157	L-Urobilinogen
metab_12365	6.8730	641.4641	neg	(2S)-4-[(2R)-2-Hydroxy-12-[(2R,5S)-5-[(1S)-1,8,9-trihydroxytetradecyl]oxolan-2-yl]dodecyl]-2-methyl-2H-furan-5-one	C35H64O7	596.4652	11124830	Fatty Acyls	Fatty alcohols	HMDB0036977	Muricin A
metab_4139	8.0700	642.3599	pos	1-Stearoyl-sn-glycero-3-phospho-1D-myo-inositol	C27H53O12P	600.3275	71581135	Glycerophospholipids	Glycerophosphoinositols	HMDB0061696	1-Stearoylglycerophosphoinositol
metab_11686	8.7640	647.4679	neg	1-Pentadecanoyl-2-(4Z,7Z,10Z,13Z,16Z,19Z-docosahexaenoyl)-sn-glycerol	C40H66O5	626.4910	53478021	Glycerolipids	Diradylglycerols	HMDB0007092	DG(15:0/22:6(4Z,7Z,10Z,13Z,16Z,19Z)/0:0)
metab_802	8.7731	647.4845	pos	Cholesteryl 6-O-hexanoyl-beta-D-galactoside	C39H66O7	646.4781	51351692	-	-	-	-
metab_4172	7.9407	647.4868	pos	Cholesteryl 6-O-hexanoyl-beta-D-galactoside	C39H66O7	646.4781	51351692	-	-	-	-
metab_4614	5.5715	649.3957	pos	gypsogenate-28-beta-D-glucoside	C36H56O10	648.3873	25244752	-	-	-	-
metab_10057	7.8193	653.3914	neg	(1R,2S,4S,6R,7S,8R,9S,12S,13S,18S)-6-Methoxy-7,9,13-trimethyl-6-[(3S)-3-methyl-4-[(2	C34H56O9	608.3924	102444970	Steroids and steroid derivatives	Steroidal glycosides	HMDB0030337	Torvoside G

				R,3R,4S,5S,6R)-3,4,5-trihydroxy-6-(hydroxymethyl)oxan-2-yl]oxybutyl]-5-oxapentacyclo[10.8.0.02,9.04,8.013,18]icosan-16-one							
metab_3076	9.6358	655.4899	pos	[(2S)-2-[9-(3,4-Dimethyl-5-propylfuran-2-yl)nonanoyloxy]-3-hydroxypropyl] 11-(3,4-dimethyl-5-propylfuran-2-yl)undecanoate	C41H68O7	672.4965	131823030	Fatty Acyls	Fatty acids and conjugates	HMDB0116375	DG(11D3/9D3/0:0)
metab_11223	10.4016	657.4512	neg	1-Pentadecanoyl-2-(9Z,12Z-octadecadienoyl)-glycero-3-phosphate	C36H67O8P	658.4574	52929505	Glycerophospholipids	Glycerophosphates	LMGP10010915; HMDB0114815	PA(15:0/18:2(9Z,12Z))
metab_801	8.7731	667.4528	pos	NA	C34H67O10P	666.4469	9547119	-	-	-	-
metab_3799	9.5283	668.5445	pos	1-Hexadecyl-2-(9Z-octadecenoyl)-sn-glycero-3-phosphoethanolamine	C39H78NO7P	703.5516	42607455	Glycerophospholipids	Glycerophosphoethanolamines	HMDB0011157	PE(P-16:0e/18:1(9Z))
metab_13347	3.1797	670.3029	neg	Leukotriene C4	C30H47N3O9S	625.3033	5280493	Carboxylic acids and derivatives	Amino acids, peptides, and analogues	LMFA03020003; HMDB0001198	Leukotriene C4
metab_11210	10.5679	671.4671	neg	1-Hexadecanoyl-2-(9Z,12Z-octadecadienoyl)-sn-glycero-3-phosphate	C37H69O8P	672.4730	9547167	Glycerophospholipids	Glycerophosphates	HMDB0007860; LMGP10010023	PA(16:0/18:2(9Z,12Z))
metab_3962	8.7303	671.4842	pos	Glyceryl 2-pentadecanoate	C18H36O4	316.2614	537297	Glycerolipids	Monoradylglycerols	HMDB0011532	MG(0:0/15:0/0:0)
metab_4428	6.6001	672.4028	pos	Mitiglinide	C19H25NO3	315.1834	121891	Phenylpropanoic acids	-	HMDB0015382	Mitiglinide
metab_10680	10.9673	673.4814	neg	1-Hexadecanoyl-2-(11Z-octadecenoyl)-sn-glycero-3-phospha	C37H71O8P	674.4887	9547158	Glycerophospholipids	Glycerophosphates	HMDB0007858; LMGP10010007	PA(16:0/18:1(11Z))

				te							
metab_13631	2.5431	686.2976	neg	S-(PGA2)-glutathione	C30H47N3O10S	641.2982	53481602	Carboxylic acids and derivatives	Amino acids, peptides, and analogues	HMDB0013062	S-(PGA2)-glutathione
metab_5348	2.5469	688.3096	pos	Buprenorphine Glucuronide	C35H49NO10	643.3356	131769995	Organooxygen compounds	Carbohydrates and carbohydrate conjugates	HMDB0060928	Buprenorphine glucuronide
metab_10063	7.8352	688.4653	neg	Spirolide D	C43H65NO7	707.4761	10699960	Azepines	-	HMDB0030493	Spirolide D
metab_3935	8.8904	689.4947	pos	PA(20:3(5Z,8Z,11Z)/18:1(11Z))	C41H73O8P	724.5043	131821991	Glycerophospholipids	Glycerophosphates	HMDB0115124	PA(20:3(5Z,8Z,11Z)/18:1(11Z))
metab_9884	7.0629	695.2148	neg	Sesamolinol 4'-O-b-D-glucosyl (1->6)-O-b-D-glucoside	C32H40O17	696.2265	131750843	Organooxygen compounds	Carbohydrates and carbohydrate conjugates	HMDB0029299	Sesamolinol 4'-O-b-D-glucosyl (1|right|6)-O-b-D-glucoside
metab_7146	10.2384	695.4670	neg	PA(18:2(9Z,12Z)/18:2(9Z,12Z))	C39H69O8P	696.4730	46891867	Glycerophospholipids	Glycerophosphates	LMGP10010957; HMDB0031092	1,2-Di-(9Z,12Z-octadecadienoyl)-sn-glycero-3-phosphate
metab_10485	9.4647	695.5833	neg	1-Behenoyl-3-palmitoleoyl-sn-glycerol	C41H78O5	650.5849	131801802	Glycerolipids	Diradylglycerols	HMDB0056090	DG(22:0/0:0/16:1n7)
metab_10663	10.5343	697.4814	neg	PA(18:1(11Z)/18:2(9Z,12Z))	C39H71O8P	698.4887	131821910	Glycerophospholipids	Glycerophosphates	HMDB0114902	PA(18:1(11Z)/18:2(9Z,12Z))
metab_13044	4.0685	699.3455	neg	Elatoside G	C36H56O11	664.3823	85149756	Prenol lipids	Terpene glycosides	HMDB0041347	Elatoside G
metab_11253	10.2384	700.4935	neg	1-Pentadecanoyl-2-(9Z,12Z-octadecadienoyl)-glycero-3-phosphoethanolamine	C38H72NO8P	701.4996	52924929	Glycerophospholipids	Glycerophosphoethanolamines	HMDB0008895; LMGP02011232	PE(15:0/18:2(9Z,12Z))
metab_5321	2.6090	701.3608	pos	Chalcomycin	C35H56O14	700.3641	6436271	-	-	-	-
metab_11884	8.3209	707.4016	neg	Pouoside H	C38H60O12	708.4094	56926618	-	-	-	-
metab_7229	10.0593	712.5373	neg	Araliacerebroside	C40H77NO10	731.5547	131751463	Sphingolipids	Glycosphingolipids	HMDB0033621	Araliacerebroside
metab_3670	10.0715	714.5497	pos	Glucocerebrosides	C40H75NO9	713.5442	10169092	-	-	-	-
metab_885	10.3942	716.5210	pos	1-Palmitoyl-2-linoleoyl PE	C39H74NO8P	715.5152	131172	-	-	-	-
metab_10670	10.7023	716.5241	neg	1-Hexadecanoyl-2-(11Z-octad	C39H76NO8P	717.5309	9546726	Glycerophospholipids	Glycerophosphoethanola	LMGP02010010;	PE(16:0/18:1(11Z))

				ecenoyl)-sn-glycero-3-phosphoethanolamine					mines	HMDB0008926	
metab_3745	9.7586	726.5495	pos	Cerebroside A	C41H75NO9	725.544183	100925207	-	-	-	-
metab_3672	10.0088	730.5364	pos	NA	C40H76NO8P	729.5309	24778624	Glycerophospholipids	Glycerophosphocholines	LMGP01010496; HMDB0007874	PC(14:0/18:2(9Z,12Z))
metab_1424	0.9761	739.1816	neg	Rhamnocitrin 3-(5'''-p-coumarylapiosyl)-(1->2)-glucoside	C36H36O17	740.196	44259551	-	-	-	-
metab_10644	10.3524	740.5237	neg	PE(18:1(11Z)/18:2(9Z,12Z))	C41H76NO8P	741.5309	53479626	Glycerophospholipids	Glycerophosphoethanolamines	HMDB0009027	PE(18:1(11Z)/18:2(9Z,12Z))
metab_10640	10.3194	742.5417	neg	PE-NMe2(16:0/18:2(9Z,12Z))	C41H78NO8P	743.5465	131821053	Glycerophospholipids	Glycerophosphoethanolamines	HMDB0113943	PE-NMe2(16:0/18:2(9Z,12Z))
metab_7198	14.0282	744.4836	neg	1-(9Z,12Z-Octadecadienoyl)-2-pentadecanoyl-glycero-3-phosphoserine	C39H72NO10P	745.4894	52925454	Glycerophospholipids	Glycerophosphoserines	LMGP03010344; HMDB0112425	PS(18:2(9Z,12Z)/15:0)
metab_7237	9.9274	744.5283	neg	Lucyobroside	C39H73NO9	699.5285	131751236	Sphingolipids	Glycosphingolipids	HMDB0031983	Lucyobroside
metab_855	9.7586	744.5603	pos	Termitomycesphin A	C41H77NO10	743.5547	42608351	-	-	LMSP01080015	
metab_11217	10.5015	746.4978	neg	1-(9Z,12Z-Octadecadienoyl)-2-pentadecanoyl-glycero-3-phosphoethanolamine	C38H72NO8P	701.4996	52924358	Glycerophospholipids	Glycerophosphoethanolamines	LMGP02010657; HMDB0009087	PE(18:2(9Z,12Z)/15:0)
metab_10610	10.0749	748.5151	neg	Soyacerebroside I	C40H75NO9	713.5442	11104507	Sphingolipids	Glycosphingolipids	HMDB0032677	Soyacerebroside I
metab_3110	9.8049	754.5359	pos	2-[[(E,2S,3R)-2-[[(E)-Hexadec-9-enoyl]amino]-3-hydroxynonadec-11-enoxy]-hydroxyphosphoryl]oxyethyl-trimethylazan	C40H80N2O6P+	715.5754	124202091	Organonitrogen compounds	Quaternary ammonium salts	HMDB0029216	SM C16:1

				ium							
metab_10597	9.9929	756.4834	neg	PE(18:3(9Z,12Z,15Z)/16:1(9Z))	C39H70NO8P	711.4839	52924417	Glycerophospholipids	Glycerophosphoethanolamines	LMGP02010716; HMDB0009155	PE(18:3(9Z,12Z,15Z)/16:1(9Z))
metab_3673	10.0088	756.5519	pos	PE(18:3(9Z,12Z,15Z)/P-18:1(11Z))	C41H74NO7P	723.5203	53479695	Glycerophospholipids	Glycerophosphoethanolamines	HMDB0009182	PE(18:3(9Z,12Z,15Z)/P-18:1(11Z))
metab_11096	14.0282	758.4989	neg	PE(18:2(9Z,12Z)/16:1(9Z))	C39H72NO8P	713.4996	52924360	Glycerophospholipids	Glycerophosphoethanolamines	HMDB0009089; LMGP02010659	PE(18:2(9Z,12Z)/16:1(9Z))
metab_3628	10.3169	758.5678	pos	1-Hexadecanoyl-2-(9Z,12Z-octadecadienoyl)-sn-glycero-3-phosphocholine	C42H80NO8P	757.5622	5287971	Glycerophospholipids	Glycerophosphocholines	HMDB0007973; LMGP01010594	PC(16:0/18:2(9Z,12Z))
metab_10533	9.6814	769.5034	neg	PG(18:1(11Z)/18:3(9Z,12Z,15Z))	C42H75O10P	770.5098	53480621	Glycerophospholipids	Glycerophosphoglycerols	HMDB0010622	PG(18:1(11Z)/18:3(9Z,12Z,15Z))
metab_3808	9.4825	772.5457	pos	PE-NMe2(14:0/20:4(8Z,11Z,14Z,17Z))	C41H74NO8P	739.5152	131820998	Glycerophospholipids	Glycerophosphoethanolamines	HMDB0113888	PE-NMe2(14:0/20:4(8Z,11Z,14Z,17Z))
metab_6651	10.2070	772.5594	neg	CID 11498616	C41H77NO9	727.5598	11498616	Sphingolipids	Glycosphingolipids	HMDB0035990	Cerebroside B
metab_10603	10.0264	782.4988	neg	PE(18:2(9Z,12Z)/18:3(9Z,12Z,15Z))	C41H72NO8P	737.4996	52924366	Glycerophospholipids	Glycerophosphoethanolamines	LMGP02010665; HMDB0009095	PE(18:2(9Z,12Z)/18:3(9Z,12Z,15Z))
metab_7203	14.0282	782.4990	neg	1-(9Z-Tetradecenoyl)-2-eicosanoyl-glycero-3-phosphoserine	C40H76NO10P	761.5207	52925237	Glycerophospholipids	Glycerophosphoserines	HMDB0112277; LMGP03010127	PS(14:1(9Z)/20:0)
metab_873	10.0403	782.5676	pos	1,2-Dilinoleoyl-SN-glycero-3-phosphocholine	C44H80NO8P	781.5622	5288075	Glycerophospholipids	Glycerophosphocholines	HMDB0008138; LMGP01010937	PC(18:2(9Z,12Z)/18:2(9Z,12Z))
metab_14941	0.5286	783.1143	neg	Astragaloside III	C41H68O14	784.4614	441905	-	-	-	-
metab_14941	0.5286	783.1143	neg	Astragaloside IV	C41H68O14	784.4614	13943297	-	-	-	-
metab_11241	10.3194	784.5119	neg	PS(18:1(9Z)/18:2(9Z,12Z))	C42H76NO10P	785.5207	52926065	Glycerophospholipids	Glycerophosphoserines	HMDB0012391; LMGP03010958	PS(18:1(9Z)/18:2(9Z,12Z))
metab_11259	10.2070	789.5495	neg	Finasteride	C23H36N2O2	372.2777	57363	Steroids and steroid	Androstane steroids	HMDB0001984	Finasteride

								derivatives			
metab_11511	9.2674	796.5141	neg	PE-NMe(18:2(9Z,12Z)/18:3(9Z,12Z,15Z))	C42H74NO8P	751.5152	25243956	Glycerophospholipids	Glycerophosphoethanolamines	HMDB0113174	PE-NMe(18:2(9Z,12Z)/18:3(9Z,12Z,15Z))
metab_3861	9.2206	796.5462	pos	PE-NMe2(18:3(6Z,9Z,12Z)/18:3(6Z,9Z,12Z))	C43H74NO8P	763.5152	131821211	Glycerophospholipids	Glycerophosphoethanolamines	HMDB0114103	PE-NMe2(18:3(6Z,9Z,12Z)/18:3(6Z,9Z,12Z))
metab_3815	9.4506	798.5624	pos	16:0/12-Hete PC	C44H80NO9P	797.5571	52929777	-	-	LMGP20010002	
metab_7140	9.9929	800.5460	neg	PE-NMe(18:1(11Z)/18:2(9Z,12Z))	C42H78NO8P	755.5465	131820231	Glycerophospholipids	Glycerophosphoethanolamines	HMDB0113116	PE-NMe(18:1(11Z)/18:2(9Z,12Z))
metab_6660	8.3209	802.4687	neg	Tacrolimus	C44H69NO12	803.4820	445643	Macrolide lactams	-	LMPK04000003; HMDB0015002	Tacrolimus
metab_770	8.3322	804.4806	pos	PS(18:3(6Z,9Z,12Z)/20:5(5Z,8Z,11Z,14Z,17Z))	C44H70NO10P	803.4737	52925500	Glycerophospholipids	Glycerophosphoserines	HMDB0112459; LMGP03010390	PS(18:3(6Z,9Z,12Z)/20:5(5Z,8Z,11Z,14Z,17Z))
metab_7213	10.6351	804.5765	neg	Phophatidylethanolamine(15:0/22:1)	C42H82NO8P	759.5778	53479578	Glycerophospholipids	Glycerophosphoethanolamines	HMDB0008908	PE(15:0/22:1(13Z))
metab_2932	8.6577	806.4942	pos	1-Hexadecanoyl-2-(9Z-octadecenoyl)-sn-glycero-3-phosphoserine	C40H76NO10P	761.5207	5283499	Glycerophospholipids	Glycerophosphoserines	LMGP03010024; HMDB0012357	PS(16:0/18:1(9Z))
metab_11297	10.0432	810.5299	neg	PE(20:3(5Z,8Z,11Z)/18:2(9Z,12Z))	C43H76NO8P	765.5309	53479750	Glycerophospholipids	Glycerophosphoethanolamines	HMDB0009324	PE(20:3(5Z,8Z,11Z)/18:2(9Z,12Z))
metab_11996	8.0228	814.4887	neg	PS(20:4(8Z,11Z,14Z,17Z)/15:0)	C41H72NO10P	769.4894	131819867	Glycerophospholipids	Glycerophosphoserines	HMDB0112656	PS(20:4(8Z,11Z,14Z,17Z)/15:0)
metab_93	8.1273	814.5570	pos	PS(18:2(9Z,12Z)/20:1(11Z))	C44H80NO10P	813.5520	52925467	Glycerophospholipids	Glycerophosphoserines	HMDB0112430; LMGP03010357	PS(18:2(9Z,12Z)/20:1(11Z))
metab_12147	7.5855	814.5615	neg	1-(9Z,12Z-Octadecadienoyl)-2-eicosanoyl-glycero-3-phosphoserine	C44H82NO10P	815.5676	52925466	Glycerophospholipids	Glycerophosphoserines	HMDB0112429; LMGP03010356	PS(18:2(9Z,12Z)/20:0)

metab_3860	9.2206	820.5441	pos	PE-NMe2(18:4(6Z,9Z,12Z,15Z)/20:4(5Z,8Z,11Z,14Z))	C45H74NO8P	787.5152	131821277	Glycerophospholipids	Glycerophosphoethanolamines	HMDB0114169	PE-NMe2(18:4(6Z,9Z,12Z,15Z)/20:4(5Z,8Z,11Z,14Z))
metab_2795	7.8532	822.4895	pos	PS(DiMe(11,3)/DiMe(9,3))	C44H74NO12P	839.4949	134159852	Glycerophospholipids	Glycerophosphoserines	HMDB0061555	PS(DiMe(11,3)/DiMe(9,3))
metab_3711	9.8673	822.5619	pos	PE-NMe2(18:3(6Z,9Z,12Z)/20:4(8Z,11Z,14Z,17Z))	C45H76NO8P	789.5309	131821220	Glycerophospholipids	Glycerophosphoethanolamines	HMDB0114112	PE-NMe2(18:3(6Z,9Z,12Z)/20:4(8Z,11Z,14Z,17Z))
metab_7244	9.8459	824.5458	neg	PC(18:2(9Z,12Z)/18:3(6Z,9Z,12Z))	C44H78NO8P	779.5465	52922729	-	-	LMGP01011625; HMDB0008140	PC(18:2(9Z,12Z)/18:3(6Z,9Z,12Z))
metab_3675	9.9935	824.5767	pos	PE-NMe2(18:1(11Z)/20:5(5Z,8Z,11Z,14Z,17Z))	C45H78NO8P	791.5465	131821137	Glycerophospholipids	Glycerophosphoethanolamines	HMDB0114028	PE-NMe2(18:1(11Z)/20:5(5Z,8Z,11Z,14Z,17Z))
metab_11298	10.0432	826.5619	neg	PC(18:1(11Z)/18:3(6Z,9Z,12Z))	C44H80NO8P	781.5622	53478725	Glycerophospholipids	Glycerophosphocholines	LMGP01012150; HMDB0008073	PC(18:1(11Z)/18:3(6Z,9Z,12Z))
metab_11246	10.2875	828.5762	neg	PC(18:1(11Z)/18:2(9Z,12Z))	C44H82NO8P	783.5778	53478723	Glycerophospholipids	Glycerophosphocholines	LMGP01012149; HMDB0008072	PC(18:1(11Z)/18:2(9Z,12Z))
metab_7914	0.5991	829.2955	neg	Astragaloside IV	C41H68O14	784.4609	13943297	-	-	-	-
metab_7914	0.5991	829.2955	neg	Astragaloside III	C41H68O14	784.4614	441905	-	-	-	-
metab_7914	0.5991	829.2955	neg	Astragaloside IV	C41H68O14	784.4614	13943297	-	-	-	-
metab_4088	8.2586	832.5673	pos	Prostaglandin E2 Ethanolamide	C22H37NO5	395.2672	5283119	Fatty Acyls	Eicosanoids	HMDB0013038	Prostaglandin E2 ethanolamide
metab_10543	9.7312	833.5206	neg	1-Hexadecanoyl-2-(9Z,12Z-octadecadienoyl)-glycero-3-phospho-(1'-myo-inositol)	C43H79O13P	834.5258	46891796	Glycerophospholipids	Glycerophosphoinositols	LMGP06010959; HMDB0009784	PI(16:0/18:2(9Z,12Z))
metab_10581	9.9274	838.5617	neg	NA	C45H80NO8P	793.5622	52924408	Glycerophospholipids	Glycerophosphoethanolamines	HMDB0009140; LMGP02010707	PE(18:3(6Z,9Z,12Z)/22:2(13Z,16Z))
metab_10537	9.6986	846.5301	neg	PE-NMe(18:2(9Z,12Z)/22:6(4Z,7Z,10Z,13Z,16Z,19Z))	C46H76NO8P	801.5309	131820303	Glycerophospholipids	Glycerophosphoethanolamines	HMDB0113190	PE-NMe(18:2(9Z,12Z)/22:6(4Z,7Z,10Z,13Z,16Z,19Z))

metab_10488	9.4984	857.5197	neg	PI(18:1(11Z)/18:3(6Z,9Z,12Z))	C45H79O13P	858.5258	53480073	Glycerophospholipids	Glycerophosphoinositols	HMDB0009827	PI(18:1(11Z)/18:3(6Z,9Z,12Z))
metab_11655	8.8929	858.5514	neg	PS(18:3(9Z,12Z,15Z)/20:0)	C44H80NO10P	813.5520	52925523	Glycerophospholipids	Glycerophosphoserines	LMGP03010413; HMDB0112471	PS(18:3(9Z,12Z,15Z)/20:0)
metab_950	9.4981	859.5304	pos	PI(18:1(11Z)/18:3(9Z,12Z,15Z))	C45H79O13P	858.5258	53480074	Glycerophospholipids	Glycerophosphoinositols	HMDB0009828	PI(18:1(11Z)/18:3(9Z,12Z,15Z))
metab_10351	8.8770	860.5639	neg	1-Hexadecanoyl-2-(13Z,16Z-docosadienoyl)-glycero-3-phosphoserine	C44H82NO10P	815.5676	52925310	Glycerophospholipids	Glycerophosphoserines	LMGP03010200; HMDB0112351	PS(16:0/22:2(13Z,16Z))
metab_11107	14.0117	867.4759	neg	Astrasieversianin IV	C45H72O16	868.4827	13996685	-	-	-	-
metab_3171	10.2712	878.5729	pos	Cavipetin D	C25H38O5	418.2719	14527064	Prenol lipids	Diterpenoids	HMDB0030365	Cavipetin D
metab_9262	3.9349	901.4804	neg	Torvoside H	C45H74O18	902.4867	70697829	-	-	-	-
metab_4950	3.9270	903.4902	pos	Torvoside H	C45H74O18	902.4867	70697829	-	-	-	-
metab_3039	9.3278	924.7468	pos	1-2-Di-tetracosanoyl-sn-glycero-3-phosphoserine	C54H106NO10P	959.7554	131820027	Glycerophospholipids	Glycerophosphoserines	HMDB0112908	PS(24:0/24:0)
metab_758	8.0843	943.6353	pos	Soyasaponin I	C48H78O18	942.5179	122097	-	-	-	-
metab_11928	8.1936	955.5836	neg	25-Acetylvulgaroside	C27H42O7	478.2931	85163499	Prenol lipids	Sesterterpenoids	HMDB0041365	25-Acetylvulgaroside
metab_2213	3.7447	995.3385	pos	Idarubicin	C26H27NO9	497.1686	42890	Anthracyclines	-	HMDB0015308	Idarubicin
metab_12166	7.5397	997.5551	neg	Lucidenic acid H	C27H40O7	476.2774	14109387	Prenol lipids	Triterpenoids	HMDB0035908	Lucidenic acid H
metab_12025	7.9612	997.6258	neg	Polyporusterone B	C28H44O6	476.3138	15168041	Steroids and steroid derivatives	Bile acids, alcohols and derivatives	HMDB0038496	Polyporusterone B
metab_751	7.9551	1043.6964	pos	1-(9Z-Octadecenoyl)-sn-glycero-3-phosphocholine	C26H52NO7P	521.3481	16081932	Glycerophospholipids	Glycerophosphocholines	HMDB0002815	LysoPC(18:1(9Z))
