# Supplementary material for: Nutritional Characterization and Untargeted Metabolomics of Oyster Mushroom Produced Using Astragalus membranaceus var. mongolicus Stems and Leaves as Substrates
Source: Front Plant Sci. 2022 Feb 3;13:802801. doi: 10.3389/fpls.2022.802801 (PMC8853653; doi:10.3389/fpls.2022.802801)
Supplement: Supplementary file 5 [file Table_3.pdf]

Table S3 Differential metabolites between AMM and control group.

Metab_ID	Retention_ Time	Apex_ m/z	Mode	VIP_OPLS-DA	Log2_ Fold_ Change	P_Value	Putative_Metabolite_ from_AMM_data	Metab_id_in_ AMM_herbs	KEGG	Phytochem Classfication	Putative_Metabolite_ HMDB	Putative_ Formula	Molecular_ Weight	HMDB_ID
metab_1297	0.5983	86.0603	pos	1.0213	1.5545	0.0007	2-Pyrrolidinone	NA	NA	NA	2-Pyrrolidinone	C4H7NO	85.0528	HMDB0002039
metab_6375	0.5983	87.0443	pos	1.0965	1.6656	0.0002	Isocrotonic acid	NA	NA	NA	Isocrotonic acid	C4H6O2	86.0368	LMFA01030194; HMDB0034439
metab_14033	1.8205	101.0596	neg	1.0820	-1.2486	0.0013	Valerate	NA	NA	NA	Valerate	C5H10O2	102.0680	-
metab_5867	1.5144	105.0699	pos	1.3639	-2.0453	0.0001	Styrene	NA	NA	NA	Styrene	C8H8	104.0630	-
metab_5877	1.5004	109.0648	pos	1.3520	1.8227	0.0016	m-Cresol	NA	NA	NA	m-Cresol	C7H8O	108.0575	HMDB0002048; HMDB0001858
metab_6831	0.8662	117.0181	neg	1.4965	-2.0013	0.0001	Succinic acid	NA	NA	NA	Succinic acid	C4H6O4	118.0265	-
metab_1442	1.0461	129.0908	pos	1.8574	8.0032	0.0023	Cyclohexanecarboxylic acid	AMM0089	NA	NA	Cyclohexanecarboxyli c acid	C7 H12 O2	128.0837	HMDB0031342
metab_1544	1.3293	129.0908	pos	1.5267	5.1232	0.0081	Cyclohexanecarboxylic acid	AMM0089	NA	NA	Cyclohexanecarboxyli c acid	C7 H12 O2	128.0837	HMDB0031342
metab_1325	0.6402	130.0456	pos	1.0734	1.8521	0.0065	3-Methylindole	AMM0093	NA	NA	3-Methylindole	C9H9N	129.058	HMDB0000466
metab_6297	0.6542	130.0606	pos	2.2610	15.7830	0.0000	L-Pipecolate	AMM0095	C00408||C07500||C13696||C03969||C05936	Alkaloids	L-Pipecolate	C6H11NO2	129.0791	HMDB0000716
metab_1379	0.8221	132.1380	pos	1.3541	3.4011	0.0013	Leucine	AMM0108	NA	NA	Leucine	C6H13NO2	131.0947	HMDB0000687
metab_1355	0.7801	133.0314	pos	1.5577	-2.8569	0.0162	Tetrahydrothiophene-2-carboxylic acid	NA	C11074	NA	Tetrahydrothiophene-2-carboxylic acid	C5H8O2S	132.0245	-
metab_5388	2.4530	133.1010	pos	1.1600	1.8204	0.0008	1-Methyl-4-(prop-1-en-2-yl)benzene	NA	NA	NA	1-Methyl-4-(prop-1-en-2-yl)benzene	C10H12	132.0939	HMDB0029641

metab_5388	2.4530	133.1010	pos	1.1600	1.8204	0.0008	1,2,3,4-Tetrahydronaphthalene	NA	NA	NA	1,2,3,4-Tetrahydronaphthalene	C10H12	132.0940	HMDB0029641
metab_14844	0.5991	135.0288	neg	1.1360	-1.3432	0.0001	(2R,3R)-2,3,4-Trihydroxybutanoic acid	NA	NA	NA	(2R,3R)-2,3,4-Trihydroxybutanoic acid	C4H8O5	136.0372	HMDB0000613
metab_589	4.3371	137.0594	pos	1.0114	1.1442	0.0001	2-(4-Hydroxyphenyl)ethanol	NA	NA	NA	2-(4-Hydroxyphenyl)ethanol	C8H10O2	136.0525	-
metab_5482	2.2361	137.0595	pos	1.2428	1.9961	0.0008	2-(4-Hydroxyphenyl)ethanol	NA	NA	NA	2-(4-Hydroxyphenyl)ethanol	C8H10O2	136.0525	-
metab_6362	0.5983	138.0503	pos	1.9558	6.2072	0.0000	Trigonelline	AMM0136	NA	Alkaloids	Trigonelline	C7H7NO2	137.0477	HMDB0000875
metab_6362	0.5983	138.0503	pos	1.9558	6.2072	0.0000	Anthranilic acid	AMM0137	NA	Alkaloids	Anthranilic acid	C7H7NO2	137.0477	HMDB0001123
metab_1296	0.5983	138.0546	pos	1.9745	4.0936	0.0000	Trigonelline	AMM0136	NA	Alkaloids	Trigonelline	C7H7NO2	137.0477	-
metab_5015	3.6684	139.0751	pos	1.3348	2.2467	0.0016	(2E,4E,6E)-7-Hydroxy-4-methylhepta-2,4,6-trienal	AMM0143	C20694||C11480||C06728||C06044||C13638||C07084||C03352	NA	(2E,4E,6E)-7-Hydroxy-4-methylhepta-2,4,6-trienal	C8H10O2	138.0681	-
metab_1741	1.9463	139.0751	pos	1.1977	1.7246	0.0001	(2E,4E,6E)-7-Hydroxy-4-methylhepta-2,4,6-trienal	NA	NA	NA	(2E,4E,6E)-7-Hydroxy-4-methylhepta-2,4,6-trienal	C8H10O2	138.0681	-
metab_4450	6.4639	139.1114	pos	1.3196	-2.0173	0.0005	5-Isopropylbicyclo[3.1.0]hexan-2-one	NA	NA	NA	5-Isopropylbicyclo[3.1.0]hexan-2-one	C9H14O	138.1045	HMDB0035229
metab_1365	0.7941	140.0338	pos	1.0134	1.5810	0.0035	3-Nitrophenol	NA	NA	NA	3-Nitrophenol	C6H5NO3	139.0269	-
metab_9298	4.0859	143.1068	neg	1.5923	-5.1737	0.0041	Caprylic acid	AMM0182	NA	NA	Octanoic acid	C8 H16 O2	144.1150	-
metab_5467	2.2519	143.1257	pos	1.4094	2.6731	0.0001	2-n-Propyl-3-pentenoic acid	AMM0165	C16654||C16648||C12104||C16653||C19757||C12297||C19318	NA	2-n-Propyl-3-pentenoic acid	C8H14O2	142.0994	HMDB0013903

metab_7983	0.7818	145.0397	neg	1.2966	2.6070	0.0438	Coumarin	AMM0187	C05851	Phenylpropanoids	Coumarin	C9H6O2	146.0367	HMDB0001218
metab_8516	1.8524	145.0497	neg	1.2035	-1.7005	0.0010	Adipic acid	NA	NA	NA	Adipic acid	C6H10O4	146.0579	HMDB0000448; LMFA01170048
metab_14293	1.4856	145.0861	neg	1.0236	1.7717	0.0022	7-Hydroxyheptanoic acid	NA	NA	NA	7-Hydroxyheptanoic acid	C7H14O3	146.0943	-
metab_6351	0.6123	146.1171	pos	1.2095	-2.2676	0.0015	4-Trimethylammoniobutanoate	NA	NA	NA	4-Trimethylammoniobutanoate	C7H15NO2	145.1104	-
metab_5690	1.8134	147.0549	pos	1.8774	1.3112	0.0129	Coumarin	AMM0200	NA	Phenylpropanoids	Coumarin	C9H6O2	146.0367	HMDB0001218
metab_1743	1.9463	147.0800	pos	1.5387	10.4956	0.0033	7-Hydroxyheptanoic acid	AMM0191	NA	NA	7-Hydroxyheptanoic acid	C7H14O3	146.0943	-
metab_1743	1.9463	147.0800	pos	1.5387	10.4956	0.0033	Coumarin	AMM0200	NA	Phenylpropanoids	Coumarin	C9H6O2	146.0367	HMDB0001218
metab_8448	1.7104	151.0426	neg	1.0478	8.1916	0.0313	Vanillin	AMM0226	NA	NA	Vanillin	C8H8O3	152.0473	HMDB0012308
metab_14838	0.5991	151.0603	neg	1.2609	-2.4977	0.0068	Xylitol	NA	NA	NA	Xylitol	C5H12O5	152.0685	HMDB0000508
metab_1849	2.2519	153.0907	pos	1.0091	1.0903	0.0001	3-Isopropylcatechol	NA	NA	NA	3-Isopropylcatechol	C9H12O2	152.0837	-
metab_5969	1.3153	155.0334	pos	1.0558	3.3198	0.0132	Diethylphosphoric acid	AMM0235	C06608	NA	Diethylphosphoric acid	C4H11O4P	154.0394	HMDB0012209
metab_6079	1.1447	155.0334	pos	1.8190	11.9245	0.0000	Diethylphosphoric acid	AMM0235	C06608	NA	Diethylphosphoric acid	C4H11O4P	154.0394	HMDB0012209
metab_13911	2.0202	155.0341	neg	1.3356	3.4259	0.0087	(2S)-2-Isopropyl-3-oxosuccinate	NA	NA	NA	(2S)-2-Isopropyl-3-oxosuccinate	C7H10O5	174.0528	HMDB0012149
metab_6921	3.6142	155.1069	neg	1.2632	-2.0118	0.0144	Nonane-4,6-dione	NA	NA	NA	Nonane-4,6-dione	C9H16O2	156.1150	-
metab_5727	1.7554	156.1015	pos	1.0202	1.1546	0.0000	Arecoline	NA	NA	NA	Arecoline	C8H13NO2	155.0946	-

metab_1557	1.3865	156.1380	pos	1.1961	1.3400	0.0135	N-Methylpelletierine	NA	NA	NA	N-Methylpelletierine	C9H17NO	155.1310	-
metab_5947	1.3437	157.0491	pos	1.4053	4.4005	0.0050	4-Methyl-3-oxoadipate-enol-lactone	AMM0247	C18311||C04558||C06210||C04559||C06760||C04171||C20710||C06321||C04112	NA	4-Methyl-3-oxoadipate-enol-lactone	C7H8O4	156.0421	-
metab_1447	1.0601	157.0492	pos	1.7093	3.7787	0.0000	4-Methyl-3-oxoadipate-enol-lactone	AMM0247	C18311||C04558||C06210||C04559||C06760||C04171||C20710||C06321||C04112	NA	4-Methyl-3-oxoadipate-enol-lactone	C7H8O4	156.0421	-
metab_4585	5.7540	157.1220	pos	1.3158	4.4550	0.0060	Nonane-4,6-dione	AMM0248	C02445||C08471||C08501	NA	Nonane-4,6-dione	C9H16O2	156.115	-
metab_6130	1.0461	159.0760	pos	1.1513	-2.7582	0.0121	4-Methylene-L-glutamine	NA	NA	NA	4-Methylene-L-glutamine	C6H10N2O3	158.0691	-
metab_13986	1.8682	159.1018	neg	1.6778	3.6701	0.0002	(R)-2-Hydroxycaprylic acid	AMM0276	NA	NA	(R)-2-Hydroxycaprylic acid	C8H16O3	160.1099	-
metab_6313	0.6262	159.1206	pos	2.3695	11.2103	0.0000	Nicotyrine	AMM0257	C10161||C19463	Alkaloids	Nicotyrine	C10H10N2	158.0843	-
metab_6313	0.6262	159.1206	pos	2.3695	11.2103	0.0000	1,5-Naphthalenediamine	AMM0256	C19463||C10161	Alkaloids	1,5-Naphthalenediamine	C10H10N2	158.0843	-
metab_13607	2.6046	164.0345	neg	1.0321	2.2043	0.0164	2-(Formylamino)benzoic acid	NA	NA	NA	2-(Formylamino)benzoic acid	C8H7NO3	165.0426	-
metab_14845	0.5991	165.0396	neg	1.0645	-1.1464	0.0011	D-Erythrose	NA	NA	NA	D-Erythrose	C4H8O4	120.0423	HMDB0002649
metab_14845	0.5991	165.0396	neg	1.0645	-1.1464	0.0011	L-Lyxonic acid	NA	NA	NA	L-Lyxonic acid	C5H10O6	166.0476	HMDB0002649
metab_7610	2.5264	165.0549	neg	1.3804	-2.0191	0.0001	L-(-)-3-Phenyllactic acid	NA	NA	NA	L-(-)-3-Phenyllactic acid	C9H10O3	166.0628	HMDB0000779

metab_1220	0.4999	166.1134	pos	1.2658	11.0414	0.0020	3-Methylguanine	AMM0305	C02230||C02242	NA	3-Methylguanine	C6H7N5O	165.065	HMDB0001566
metab_5901	1.4429	166.1222	pos	1.9923	7.0942	0.0000	3-Methylguanine	AMM0305	C02230||C02242	NA	3-Methylguanine	C6H7N5O	165.065	HMDB0001566
metab_381	1.7845	167.0335	pos	1.9193	5.9000	0.0000	Terephthalic acid	AMM0307	NA	NA	Terephthalic acid	C8H6O4	166.0265	HMDB0002428
metab_1750	1.9609	167.0699	pos	1.4762	2.5812	0.0000	3-Methoxy-4-hydroxyphenylacetaldehyde	AMM0313	C05581||C11380||C10712||C02201||C02363||C03080||C12206||C01456||C01198||C01744||C11457||C05607	Phenylpropanoids	Homovanillin	C9H10O3	166.0629	-
metab_1750	1.9609	167.0699	pos	1.4762	2.5812	0.0000	4-Ipomeanol	AMM0314	NA	Phenylpropanoids	4-Ipomeanol	C9H12O3	166.0630	-
metab_5807	1.5987	167.0699	pos	1.6625	3.1792	0.0000	3-Methoxy-4-hydroxyphenylacetaldehyde	AMM0313	C05581||C11380||C10712||C02201||C02363||C03080||C12206||C01456||C01198||C01744||C11457||C05607	Phenylpropanoids	Homovanillin	C9H10O3	166.0629	-
metab_5807	1.5987	167.0699	pos	1.6625	3.1792	0.0000	4-Ipomeanol	AMM0314	NA	Phenylpropanoids	4-Ipomeanol	C9H12O3	166.0630	-
metab_8387	1.6028	169.0135	neg	1.3627	3.6197	0.0113	Gallic acid	NA	NA	NA	Gallic acid	C7H6O5	170.0215	HMDB0005807
metab_5818	1.5847	169.0966	pos	1.1225	-1.5625	0.0008	3,4-Dihydroxy-2-hydroxymethyl-1-pyrrolidinepropanamide	NA	NA	NA	3,4-Dihydroxy-2-hydroxymethyl-1-pyrrolidinepropanamide	C8H16N2O4	204.1110	HMDB0039948
metab_1883	2.3751	169.1220	pos	1.1429	1.9005	0.0011	Geranic acid	NA	NA	NA	Geranic acid	C10H16O2	168.1150	-

metab_5265	2.8108	169.1220	pos	1.1794	1.7305	0.0003	Geranic acid	NA	NA	NA	Geranic acid	C10H16O2	168.1150	-
metab_9093	3.3465	169.1227	neg	1.6318	3.1745	0.0000	9-Decenoic acid	AMM0344	NA	Terpenoids	9-Decenoic acid	C10H18O2	170.1307	-
metab_13087	3.9005	169.1227	neg	1.3291	1.8813	0.0298	9-Decenoic acid	AMM0344	NA	Terpenoids	9-Decenoic acid	C10H18O2	170.1307	-
metab_5813	1.5987	170.1170	pos	1.1959	1.6978	0.0001	Piperidione	NA	NA	NA	Piperidione	C9H15NO2	169.1101	-
metab_4937	3.9876	170.1535	pos	1.2514	2.8628	0.0270	Nitramine	AMM0333	C10163||C10773	Alkaloids	Tetryl	C10H19NO	169.1465	-
metab_8440	1.7104	171.0656	neg	1.2604	2.4457	0.0010	4,7-Dioxooctanoic acid	NA	NA	NA	4,7-Dioxooctanoic acid	C8H12O4	172.0736	-
metab_14839	0.5991	173.0923	neg	1.1765	-1.1192	0.0001	N-Acetylornithine	NA	NA	NA	N-Acetylornithine	C7H14N2O3	174.1004	HMDB0003357
metab_2597	6.4639	173.1168	pos	1.0944	-1.4302	0.0196	9-Oxononanoic acid	NA	NA	NA	9-Oxononanoic acid	C9H16O3	172.1099	-
metab_6205	0.8640	174.1484	pos	1.7536	12.3818	0.0001	9-Aminononanoic acid	AMM0368	NA	NA	9-Aminononanoic acid	C9H19NO2	173.1416	-
metab_1455	1.0883	174.1484	pos	1.1751	-1.4308	0.0000	9-Aminononanoic acid	NA	NA	NA	9-Aminononanoic acid	C9H19NO2	173.1416	-
metab_7500	1.8046	175.0605	neg	1.6310	-2.3874	0.0000	2-Isopropylmalic acid	NA	NA	NA	2-Isopropylmalic acid	C7H12O5	176.0685	HMDB0000402
metab_4968	3.8511	175.1114	pos	1.4182	1.8681	0.0007	Capillanol	AMM0375	C17783	NA	Capillanol	C12H14O	174.1044	-
metab_1153	0.5420	175.1185	pos	1.8539	4.0089	0.0003	Arginine	NA	C00062	NA	Arginine	C6H14N4O2	175.1195	HMDB0062762; HMDB0000517
metab_1146	0.5983	176.0103	pos	2.2748	5.3634	0.0000	2-Amino-5-chloromuconate 6-semialdehyde	AMM0379	C20670	NA	2-Amino-5-chloro-cis, cis-muconic 6-semialdehyde	C6H6ClNO3	175.0036	-
metab_7517	1.8682	179.0343	neg	1.0909	-1.5844	0.0016	Caffeic acid	NA	NA	NA	Caffeic acid	C9H8O4	180.0423	HMDB0001964
metab_14187	1.5736	179.0707	neg	1.7316	4.2455	0.0000	U 0521	AMM0411	C15475||C20343||C00590	Phenylpropanoids	U 0521	C10H12O3	180.0786	-

metab_5698	1.7993	180.1378	pos	2.4619	14.9117	0.0000	(-)-Salsolinol	AMM0405	C09642||C20895||C07591||C10146||C17235||C14579||C18771||C18887	Alkaloids	(-)-Salsolinol	C10H13NO2	179.0946	HMDB0042012
metab_12558	6.0831	183.1384	neg	1.0267	2.4220	0.0176	gamma-Undecalactone	NA	NA	NA	gamma-Undecalactone	C11H20O2	184.1462	-
metab_6963	4.6550	183.1384	neg	1.4072	2.9074	0.0030	gamma-Undecalactone	NA	NA	NA	gamma-Undecalactone	C11H20O2	184.1462	-
metab_2743	7.4415	184.0729	pos	1.0687	1.4665	0.0001	Phosphocholine	NA	NA	NA	Phosphocholine	C5H14NO4P	184.0739	HMDB0001565
metab_5956	1.3293	184.1329	pos	1.0197	10.5881	0.0146	Acetylpseudotropine	AMM0430	C12453	Alkaloids	Acetylpseudotropine	C10H17NO2	183.1259	-
metab_14024	1.8205	186.0554	neg	1.8080	6.8423	0.0046	3-Indoleacrylate	AMM0457	C21283||C06559	NA	3-Indoleacrylate	C11H9NO2	187.0632	-
metab_316	1.3720	186.1119	pos	1.0251	1.3193	0.0002	Pseudoecgonine	NA	NA	NA	Pseudoecgonine	C9H15NO3	185.1052	-
metab_1030	1.9309	187.1324	pos	1.8779	3.4666	0.0000	(5S)-6-Hydroxy-5-isopropenyl-2-methylhexanoate	AMM0447	C11420||C11417||C20714||C02217||C17620||C13945	NA	(5S)-6-Hydroxy-5-isopropenyl-2-methylhexanoate	C10H18O3	186.1255	-
metab_7608	2.5113	189.0763	neg	1.2542	-1.7511	0.0006	(R)-3-[(R)-3-Hydroxybutanoyloxy]butanoic acid	NA	NA	NA	(R)-3-[(R)-3-Hydroxybutanoyloxy]butanoic acid	C8H14O5	190.0842	-
metab_5892	1.4569	192.0650	pos	1.0691	-2.3104	0.0123	5-Phenyl-1,3-oxazinane-2,4-dione	NA	NA	NA	5-Phenyl-1,3-oxazinane-2,4-dione	C10H9NO3	191.0582	HMDB0060400
metab_5097	3.3337	192.1095	pos	1.4664	3.6640	0.0140	Ephedroxane	AMM0482	C17902	NA	Ephedroxane	C11H13NO2	191.0946	-

metab_6030	1.2016	193.0605	pos	1.0226	3.0036	0.0208	Quinate	AMM0490	C00296||C21282||C01295	NA	Quinate	C7H12O6	192.0634	-
metab_1816	2.1444	193.0686	pos	1.4003	9.5876	0.0055	Sinapyl alcohol	AMM0491	NA	Phenylpropanoids	Sinapyl alcohol	C11H14O4	192.0787	HMDB0013070
metab_5244	2.8725	193.0856	pos	1.6213	3.4697	0.0001	Sinapyl alcohol	AMM0491	NA	Phenylpropanoids	Sinapyl alcohol	C11H14O4	192.0787	-
metab_4476	6.3885	193.1583	pos	1.2114	1.8864	0.0001	4-Heptylphenol	NA	NA	NA	4-Heptylphenol	C13H20O	192.1513	-
metab_14259	1.5297	195.0293	neg	1.7665	4.4031	0.0003	2-(2-Hydroxy-4-methoxyphenyl)-2-oxoacetic acid	NA	NA	NA	2-(2-Hydroxy-4-methoxyphenyl)-2-oxoacetic acid	C9H8O5	196.0372	HMDB0137120
metab_11705	8.7191	195.0505	neg	1.0365	-1.0483	0.0003	Gluconic acid	NA	NA	NA	Gluconic acid	C6H12O7	196.0583	-
metab_9867	7.0322	195.1385	neg	1.1133	-1.5198	0.0355	Ethyl (2E,4Z)-deca-2,4-dienoate	NA	NA	NA	Ethyl (2E,4Z)-deca-2,4-dienoate	C12H20O2	196.1464	-
metab_9867	7.0322	195.1385	neg	1.1133	-1.5198	0.0355	Geranyl acetate	NA	NA	NA	Geranyl acetate	C12H20O2	196.1464	-
metab_5355	2.5158	196.1409	pos	1.1976	8.7095	0.0157	alpha-[3-(Nitrosoamino)propyl]-3-pyridinemethanol	AMM0513	C19581	NA	alpha-[3-(Nitrosoamino)propyl]-3-pyridinemethanol	C9H13N3O2	195.1009	HMDB0062443
metab_5757	1.6838	197.0806	pos	1.2245	2.9722	0.0063	2,3-Dihydroxy-p-cumate	AMM0515	C06580||C10664||C10684||C10726||C17455||C12205||C07351||C16778	Phenylpropanoids	2,3-Dihydroxy-p-cumate	C10H12O4	196.0736	-
metab_13323	3.2301	199.1335	neg	1.0917	1.6832	0.0225	11-Hydroxy-2-undecenoic acid	NA	NA	NA	11-Hydroxy-2-undecenoic acid	C11H20O3	200.1413	-
metab_14074	1.7419	200.1005	neg	1.3227	5.1561	0.0089	L-2-amino-8-oxodecanoa	AMM0558	NA	NA	L-2-amino-8-oxodeca	C10H19N	201.1365	-

							te				noate	O3		
metab_14029	1.8205	201.1127	neg	1.0114	1.9908	0.0060	Sebacic acid	NA	NA	NA	Sebacic acid	C10H18O4	202.1207	-
metab_1319	0.6262	203.1021	pos	1.1699	1.2163	0.0001	Proclavaminic acid	NA	NA	NA	Proclavaminic acid	C8H14N2O4	202.0955	-
metab_6338	0.6123	203.1134	pos	1.0250	12.0484	0.0192	IPA imine	AMM0567	C21124||C06732||C10744	Alkaloids	IPA imine	C11H10N2O2	202.0743	-
metab_4864	4.3371	203.1427	pos	1.4218	1.9838	0.0002	alpha-Amylcinnamaldehyde	NA	NA	NA	alpha-Amylcinnamaldehyde	C14H18O	202.1359	-
metab_1234	0.5140	203.2226	pos	1.0640	1.6828	0.0024	Thermospermine	NA	NA	NA	Thermospermine	C10H26N4	202.2158	-
metab_7620	2.6046	204.0662	neg	1.0527	-1.2465	0.0036	Indole-3-lactic acid	NA	NA	NA	Indole-3-lactic acid	C11H11NO3	205.0739	HMDB0000671
metab_3396	14.8330	204.0861	pos	1.1286	1.3033	0.0000	N-Acetyl-L-2-aminoadipic acid	NA	NA	NA	N-Acetyl-L-2-aminoadipic acid	C8H13NO5	203.0794	-
metab_2637	6.8433	204.0862	pos	1.6385	2.7719	0.0002	N2-Acetyl-L-aminoadipate	AMM0586	C12986	NA	N-Acetyl-L-2-aminoadipic acid	C8H13NO5	203.0794	-
metab_2330	4.4580	204.0862	pos	1.0630	1.1897	0.0003	N-Acetyl-L-2-aminoadipic acid	NA	NA	NA	N-Acetyl-L-2-aminoadipic acid	C8H13NO5	203.0794	-
metab_1964	2.7029	204.1015	pos	1.0137	-1.2915	0.0039	Shihunine	NA	NA	NA	Shihunine	C12H13NO2	203.0946	-
metab_1374	0.8221	205.0813	pos	1.1476	-2.0044	0.0366	Glu-Gly	NA	NA	NA	Glu-Gly	C7H12N2O5	204.0745	-
metab_45	1.3153	206.1382	pos	1.4114	7.2501	0.0226	Pantothenol	AMM0599	C05944	NA	Dexpanthenol	C9H19NO4	205.1315	HMDB0004231
metab_6776	0.6131	209.0662	neg	1.1920	1.7978	0.0002	L-Glycero-D-Manno-Heptose	NA	NA	NA	L-Glycero-D-Manno-Heptose	C7H14O7	210.0740	-
metab_8892	2.7624	209.0816	neg	1.1598	-1.8838	0.0056	Ethyl	NA	NA	NA	Ethyl	C11H14O4	210.0892	-

							2,4-dihydroxy-3,6-dimet hylbenzoate				2,4-dihydroxy-3,6-dim ethylbenzoate			
metab_490	2.8725	209.1168	pos	1.2243	1.8240	0.0002	Coronafacic acid	NA	NA	NA	Coronafacic acid	C12H16O3	208.1098	-
metab_14870	0.5711	211.0819	neg	1.3117	-1.8713	0.0002	Perseitol	NA	NA	NA	Perseitol	C7H16O7	212.0896	HMDB0033750
metab_8627	2.0987	211.0973	neg	1.9888	4.2540	0.0000	3-Hydroxy-p-mentha-1,8 -dien-7-al	NA	NA	NA	3-Hydroxy-p-mentha-1,8-dien-7-al	C10H14O2	166.0994	HMDB0041584
metab_2517	5.7540	211.1323	pos	1.3003	2.0500	0.0085	Jasmonic acid	AMM0625	NA	NA	Jasmonic acid	C12H18O3	210.1255	-
metab_4703	5.0451	211.1323	pos	1.0154	1.0320	0.0023	Jasmonic acid	NA	NA	NA	Jasmonic acid	C12H18O3	210.1255	-
metab_1129	0.6262	212.0911	pos	2.0712	4.9190	0.0000	Methyldopa anhydrous	AMM0631	C07194||C20801||C09946	NA	Methyldopa	C10H13NO4	211.0845	-
metab_1129	0.6262	212.0911	pos	2.0712	4.9190	0.0000	Enicoflavine	AMM0630	C09946||C07194||C20801	NA	Enicoflavine	C10H13NO4	211.0845	-
metab_231	0.8920	212.0911	pos	1.2741	1.8500	0.0000	Enicoflavine	NA	NA	NA	Enicoflavine	C10H13NO4	211.0845	-
metab_231	0.8920	212.0911	pos	1.2741	1.8500	0.0000	Methyldopa	NA	NA	NA	Methyldopa	C10H13NO4	211.0845	-
metab_1580	1.4429	212.1066	pos	1.0240	1.4169	0.0454	Zalcitabine	AMM0632	C07207	NA	Zalcitabine	C9H13N3O3	211.0957	HMDB0015078
metab_308	1.3437	213.0752	pos	1.7286	3.6957	0.0000	4-Hydroxymethyl-3-met hoxyphenoxyacetic acid	NA	NA	NA	4-Hydroxymethyl-3-m ethoxyphenoxyacetic acid	C10H12O5	212.0685	-
metab_1915	2.4850	213.1480	pos	1.1407	1.3993	0.0003	12-Oxo-9Z-dodecenoic acid	NA	NA	NA	12-Oxo-9Z-dodecenoi c acid	C12H20O3	212.1410	-
metab_1915	2.4850	213.1480	pos	1.1407	1.3993	0.0003	(+)-Cucurbic acid	NA	NA	NA	(+)-Cucurbic acid	C12H20O3	212.1411	-
metab_1915	2.4850	213.1480	pos	1.1407	1.3993	0.0003	7-Oxo-11-Dodecenoic acid	NA	NA	NA	7-Oxo-11-Dodecenoic acid	C12H20O3	212.1411	-

metab_5635	1.9609	213.1481	pos	1.4874	4.1031	0.0027	Cucurbic acid	AMM0652	C08482||C16311||C16309||C08509	NA	Cucurbic acid	C12H20O3	212.1411	-
metab_5635	1.9609	213.1481	pos	1.4874	4.1031	0.0027	12-Oxo-9Z-dodecenoic acid	NA	NA	NA	12-Oxo-9Z-dodecenoic acid	C12H20O3	212.1410	-
metab_5635	1.9609	213.1481	pos	1.4874	4.1031	0.0027	7-Oxo-11-Dodecenoic acid	NA	NA	NA	7-Oxo-11-Dodecenoic acid	C12H20O3	212.1411	-
metab_1333	0.6542	215.1384	pos	1.7225	5.4112	0.0000	2,2'-(3-methylcyclohexane-1,1-diyl)diacetic acid	AMM0674	NA	NA	2,2'-(3-methylcyclohexane-1,1-diyl)diacetic acid	C11H18O4	214.1206	-
metab_5513	2.1746	216.1589	pos	1.2660	1.8870	0.0000	Ethyl butylacetylaminopropionate	NA	NA	NA	Ethyl butylacetylaminopropionate	C11H21NO3	215.1520	-
metab_9549	5.3388	217.1078	neg	1.3090	2.9427	0.0062	3-Hydroxysebacic acid	AMM0719	NA	NA	3-Hydroxysebacic acid	C10H18O5	218.1155	-
metab_14278	1.5155	217.1079	neg	1.2865	2.2053	0.0000	3-Hydroxysebacic acid	NA	NA	NA	3-Hydroxysebacic acid	C10H18O5	218.1155	-
metab_40	1.2162	217.1541	pos	1.0853	1.0381	0.0001	Val-val	NA	NA	NA	Val-val	C10H20N2O3	216.1474	-
metab_8155	1.2663	219.0871	neg	1.1229	1.9107	0.0009	Ascaroside C3	NA	NA	NA	Ascaroside C3	C9H16O6	220.0948	-
metab_14123	1.6642	221.0816	neg	1.6986	-5.0300	0.0000	Dillapiole	AMM0742	C10449||C10429||C20225||C14175||C19228	NA	Dillapiol	C12H14O4	222.0892	-
metab_14274	1.5155	221.0817	neg	2.1394	-5.6268	0.0001	Dillapiole	AMM0742	C10449||C10429||C20225||C14175||C19228	NA	Dillapiol	C12H14O4	222.0892	-
metab_1104	1.0321	221.0912	pos	1.0166	1.5500	0.0282	5-Hydroxytryptophan	NA	NA	NA	5-Hydroxytryptophan	C11H12N2O3	220.0848	-
metab_9145	3.5641	223.0610	neg	1.1508	1.7603	0.0034	2-Benzylmalic acid	NA	NA	NA	2-Benzylmalic acid	C11H12O5	224.0685	-

metab_9145	3.5641	223.0610	neg	1.1508	1.7603	0.0034	Sinapic acid	NA	NA	NA	Sinapic acid	C11H12O5	224.0686	-
metab_8522	1.8682	225.0766	neg	1.2148	-1.9119	0.0008	Genipin	NA	NA	NA	Genipin	C11H14O5	226.0843	-
metab_2362	4.6398	225.1116	pos	1.3934	11.8578	0.0301	2-Benzylmalic acid	AMM0754	C20653||C20654||C00482	Phenylpropanoids	2-Benzylmalic acid	C11H12O5	224.0685	-
metab_2362	4.6398	225.1116	pos	1.3934	11.8578	0.0301	Sinapic acid	AMM0755	NA	Phenylpropanoids	Sinapic acid	C11H12O5	224.0686	HMDB0032616
metab_5310	2.6561	225.1116	pos	1.6693	3.2836	0.0016	Diplodiol	NA	NA	NA	Diplodiol	C12H16O4	224.1049	HMDB0030680
metab_12881	4.6550	227.1286	neg	1.2095	2.1386	0.0032	Traumatic acid	NA	NA	NA	Traumatic acid	C12H20O4	228.1363	HMDB0000933; LMFA01170002
metab_13101	3.8506	228.1321	neg	1.1954	3.1048	0.0431	crotonyl-L-carnitine	AMM0817	NA	NA	crotonyl-L-carnitine	C11H19NO4	229.1315	-
metab_5717	1.7705	228.1335	pos	2.9746	-3.4914	0.0006	3-acetamino-6-isobutyl-2,5-dioxopiperazine	AMM0789	NA	NA	3-Acetamino-6-isobutyl-2,5-dioxopiperazine	C10H17N3O3	227.1270	HMDB0028734
metab_5717	1.7705	228.1335	pos	2.9746	-3.4914	0.0006	Asparaginyl-Isoleucine	NA	NA	NA	Asn-Ile	C10H19N3O4	245.1376	HMDB0028734
metab_5806	1.5987	229.0713	pos	1.4418	10.8569	0.0010	Depdecin	AMM0800	NA	NA	Depdecin	C11H16O5	228.0997	-
metab_5679	1.8577	229.1328	pos	1.1944	3.6497	0.0317	Traumatic Acid	AMM0805	NA	NA	Traumatic Acid	C12H20O4	228.1362	HMDB0000933
metab_8844	2.6206	231.1236	neg	1.0247	1.6990	0.0014	1,2-Dibutyrin	NA	NA	NA	1,2-Dibutyrin	C11H20O5	232.1311	-
metab_1068	1.4289	232.1536	pos	1.5067	-2.9722	0.0053	N-(Tert-Butoxycarbonyl)-L-leucine	NA	NA	NA	N-(Tert-Butoxycarbonyl)-L-leucine	C11H21NO4	231.1470	-
metab_1068	1.4289	232.1536	pos	1.5067	-2.9722	0.0053	O-Butanoylcarnitine	NA	NA	NA	O-Butanoylcarnitine	C11H21NO4	231.1470	-
metab_9355	4.3380	233.0818	neg	1.5853	3.8456	0.0049	2-[2,4-Dihydroxy-3-(3-methylbut-2-en-1-yl)phenyl]-2-hydroxyacetic acid	NA	NA	NA	2-[2,4-Dihydroxy-3-(3-methylbut-2-en-1-yl)phenyl]-2-hydroxyacetic acid	C13H16O5	252.0998	HMDB0137142

metab_14015	1.8371	233.1028	neg	1.1978	2.1029	0.0029	(3R)-3-{[(2R,3R,5R,6S)-3,5-Dihydroxy-6-methyltetrahydro-2H-pyran-2-yl]oxy}butanoic acid	NA	NA	NA	(3R)-3-{[(2R,3R,5R,6S)-3,5-Dihydroxy-6-methyltetrahydro-2H-pyran-2-yl]oxy}butanoic acid	C10H18O6	234.1108	-
metab_6262	0.7801	233.1125	pos	1.2399	1.8139	0.0000	N2-Succinyl-L-ornithine	NA	NA	NA	N2-Succinyl-L-ornithine	C9H16N2O5	232.1059	HMDB0001199
metab_4566	5.8597	233.1529	pos	1.4857	5.0937	0.0001	3,12-dihydroxydodecanoic acid	AMM0857	NA	NA	3,12-dihydroxydodecanoic acid	C12H24O4	232.1675	-
metab_2609	6.6148	233.2258	pos	1.1004	-1.6046	0.0316	1,8,11,14-Heptadecatetraene, (Z,Z,Z)-	NA	NA	NA	1,8,11,14-Heptadecatetraene, (Z,Z,Z)-	C17H28	232.2190	-
metab_5602	2.0211	235.1686	pos	1.9886	4.2740	0.0007	Confertifolin	AMM0868	NA	Terpenoids	Confertifolin	C15H22O2	234.1618	-
metab_4742	4.9115	235.1686	pos	1.3836	3.0794	0.0086	Confertifolin	AMM0868	NA	Terpenoids	Confertifolin	C15H22O2	234.1618	-
metab_5116	3.2727	235.1799	pos	1.9272	-4.2188	0.0000	Lidocaine	AMM0871	NA	Alkaloids	Lidocaine	C14H22N2O	234.1731	-
metab_5620	1.9918	236.1107	pos	1.7458	4.0795	0.0000	CHEBI:133389	AMM0873	NA	NA	CHEBI:133389	C10H21NOS2	235.1054	-
metab_8952	2.9092	237.0767	neg	1.2334	-1.4305	0.0029	Swerilactone L, (rel)-	NA	NA	NA	Swerilactone L, (rel)-	C12H14O5	238.0842	-
metab_2005	2.8725	237.1479	pos	1.0281	1.5123	0.0060	4-Heptyloxybenzoic acid	NA	NA	NA	4-Heptyloxybenzoic acid	C14H20O3	236.1410	-
metab_1748	1.9609	239.0512	pos	1.4157	3.6473	0.0004	3,4-Bis(methoxycarbonyl)benzoic acid	AMM0896	NA	NA	3,4-Bis(methoxycarbonyl)benzoic acid	C11H10O6	238.0478	-
metab_5063	3.4702	239.1102	pos	1.1181	9.8049	0.0342	Swerilactone L	AMM0901	NA	NA	Swerilactone L	C12H14O5	238.0842	-
metab_13373	3.1115	239.1288	neg	1.7439	3.4562	0.0009	5OH-HIP	AMM0920	NA	NA	3-[(3As,4S,5R,7aS)-5-hydroxy-7a-methyl-1,5-dioxo-octahydroinde	C13H20O4	240.1362	-

											n-4-yl]propanoic acid			
metab_1531	1.2725	240.1335	pos	1.1311	1.9156	0.0334	Lysylglutamic acid	NA	NA	NA	Lysylglutamic acid	C11H21N3O5	275.1481	HMDB0028950
metab_13377	3.0942	241.1445	neg	1.2577	1.9462	0.0038	2-Isocapryloyl-3R-hydroxymethyl-gamma-butyrolactone	NA	NA	NA	2-Isocapryloyl-3R-hydroxymethyl-gamma-butyrolactone	C13H22O4	242.1519	-
metab_1408	0.9200	241.1539	pos	1.0940	1.7140	0.0028	Pirbuterol	NA	NA	NA	Pirbuterol	C12H20N2O3	240.1472	-
metab_7365	7.8193	241.2172	neg	1.1747	2.1417	0.0061	Pentadecanoic acid	NA	NA	NA	Pentadecanoic acid	C15H30O2	242.2247	-
metab_8053	1.0074	242.1145	neg	1.9306	6.6717	0.0000	L-alanylglycyl-L-proline	AMM0956	NA	NA	L-alanylglycyl-L-proline	C10H17N3O4	243.1219	-
metab_13655	2.4970	243.0774	neg	1.0136	9.5089	0.0450	Uridine	AMM0968	NA	NA	Uridine	C9H12N2O6	244.0695	HMDB0000296
metab_14549	1.1503	243.1462	neg	1.1930	10.6327	0.0302	Pandangolide 1	AMM0974	NA	NA	Pandangolide 1	C12H20O5	244.131	-
metab_5799	1.6128	243.1694	pos	1.4233	12.1486	0.0079	A Factor	AMM0949	NA	NA	A Factor	C13H22O4	242.1519	-
metab_382	1.7993	243.1700	pos	1.5543	12.3615	0.0038	A Factor	AMM0949	NA	NA	A Factor	C13H22O4	242.1519	-
metab_5959	1.3293	243.1808	pos	1.4011	12.7954	0.0017	A Factor	AMM0949	NA	NA	A Factor	C13H22O4	242.1519	-
metab_1117	0.7801	244.1284	pos	1.2751	2.2571	0.0009	L-Alanylglycyl-L-proline	NA	NA	NA	L-Alanylglycyl-L-proline	C10H17N3O4	243.1219	-
metab_8607	2.0358	244.1300	neg	2.0101	-8.2606	0.0009	GLY-GLY-ILE	AMM0992	NA	NA	Gly-gly-ile	C10H19N3O4	245.1375	-
metab_5897	1.4429	245.1281	pos	1.2989	11.5617	0.0023	Pandangolide 1	AMM0972	NA	NA	Pandangolide 1	C12H20O5	244.1309	-
metab_13322	3.2473	245.1394	neg	1.2063	2.6141	0.0210	Ascaroside C6	NA	NA	NA	Ascaroside C6	C12H22O5	246.1467	HMDB0032662; LMFA01050436
metab_5127	3.2420	246.1119	pos	1.1957	-2.7337	0.0151	3-(4-Hydroxy-3-methoxyphenyl)-N-(4-oxobutyl)p	NA	NA	NA	3-(4-Hydroxy-3-methoxyphenyl)-N-(4-oxobu	C14H17NO4	263.1158	HMDB0139920

							rop-2-enimidic acid				tyl)prop-2-enimidic acid			
metab_384	1.8277	246.1692	pos	1.0007	-1.4140	0.0335	2-Methylbutyroylcarnitine	NA	NA	NA	2-Methylbutyroylcarnitine	C12H23NO4	245.1627	HMDB0000378; LMFA07070034
metab_384	1.8277	246.1692	pos	1.0007	-1.4140	0.0335	Isovalerylcarnitine	NA	NA	NA	Isovalerylcarnitine	C12H23NO4	245.1626	HMDB0000378; LMFA07070034
metab_5759	1.6838	249.0862	pos	1.7463	6.2397	0.0007	Asp-Asp	AMM1024	NA	NA	Asp-Asp	C8H12N2O7	248.0643	-
metab_1744	1.9463	249.1324	pos	1.7749	4.9626	0.0000	H-Val-Met-OH	AMM1026	NA	NA	H-Val-Met-OH	C10H20N2O3S	248.1194	-
metab_14446	1.2948	249.1344	neg	1.2361	5.0218	0.0062	Xanthoxin	AMM1043	C13453||C09748||C09711||C20403||C09295||C20405||C13456||C07020||C14716||C09689	Terpenoids	Xanthoxin	C15H22O3	250.1569	-
metab_5707	1.7845	251.1381	pos	1.0842	2.0378	0.0091	N-Caffeoylputrescine	NA	NA	NA	N-Caffeoylputrescine	C13H18N2O3	250.1317	HMDB0029876
metab_521	2.4530	251.1636	pos	1.3622	2.5587	0.0011	1-Deoxy-11beta-hydroxypentalenate	NA	NA	NA	1-Deoxy-11beta-hydroxypentalenate	C15H22O3	250.1567	-
metab_7642	2.7155	252.0515	neg	1.6356	4.2523	0.0028	N-Pyruvoyl-5-methoxy-3-hydroxyanthranilate	AMM1075	C11467	NA	N-Pyruvoyl-5-methoxy-3-hydroxyanthranilate	C11H11NO6	253.0588	-
metab_1574	1.4289	252.1585	pos	2.1299	1.7272	0.0209	Furmecyclox	AMM1047	C18912	NA	Furmecyclox	C14H21NO3	251.1521	-
metab_6143	1.0040	253.0560	pos	1.3630	3.8763	0.0136	S-(4-Methylthiobutylthio	AMM1058	C17242	NA	S-(4-Methylthiobutylt	C8H16N2	252.0595	-

							hydroximoyl)-L-cysteine				hiohydroximoyl)-L-cysteine	O3S2		
metab_2644	6.8728	253.2518	pos	1.1271	2.0745	0.0444	(9Z)-Cycloheptadec-9-en-1-ol	NA	NA	NA	(9Z)-Cycloheptadec-9-en-1-ol	C17H32O	252.2452	-
metab_2074	3.1495	255.2186	pos	1.5759	7.2288	0.0060	(9Z)-Hexadecenoic acid	AMM1100	C08362	Fatty acids related compounds	(9Z)-Hexadecenoic acid	C16H30O2	254.2247	HMDB0003229
metab_212	0.6542	256.0688	pos	1.5486	6.0078	0.0134	Nicotinate D-ribonucleoside	AMM1104	NA	NA	Nicotinate D-ribonucleoside	C11H13NO6	255.074	HMDB0006809
metab_14335	1.4568	257.1621	neg	1.4429	13.0962	0.0171	Tetradecanedioic acid	AMM1140	NA	NA	Tetradecanedioic acid	C14 H26 O4	258.1833	HMDB0000872
metab_8165	1.2805	258.0985	neg	2.0052	4.4576	0.0000	N-(1-Deoxy-1-fructosyl)proline	NA	NA	NA	N-(1-Deoxy-1-fructosyl)proline	C11H19NO7	277.1162	HMDB0038493
metab_13798	2.2119	258.1463	neg	2.5364	-6.5543	0.0001	Leu-Ala-Gly	AMM1150	NA	NA	Leu-Ala-Gly	C11H21N3O4	259.1532	-
metab_5258	2.8259	259.0595	pos	1.7074	11.2747	0.0017	Spongothymidine	AMM1137	C16744||C05131	NA	Spongothymidine	C10H14N2O6	258.0854	-
metab_14163	1.6186	259.0613	neg	2.1294	-4.9463	0.0000	8-(1,2-Dihydroxypropan-2-yl)-9-hydroxy-2H,8H,9H-furo[2,3-h]chromen-2-one	NA	NA	NA	8-(1,2-Dihydroxypropan-2-yl)-9-hydroxy-2H,8H,9H-furo[2,3-h]chromen-2-one	C14H14O6	278.0790	HMDB0128947
metab_13851	2.1153	259.1190	neg	1.4581	2.9038	0.0002	5-Hexyltetrahydro-2-oxo-3-furancarboxylic acid	NA	NA	NA	5-Hexyltetrahydro-2-oxo-3-furancarboxylic acid	C11H18O4	214.1205	HMDB0030984
metab_13965	1.9280	260.1042	neg	2.0812	-3.5943	0.0004	Asparaginyl-phenylalanine	NA	NA	NA	Asparaginyl-phenylalanine	C13H17N3O4	279.1219	HMDB0028738

metab_8836	2.6046	261.1345	neg	1.0993	1.6146	0.0023	Etoglucid	NA	NA	NA	Etoglucid	C12H22O6	262.1410	-
metab_4884	4.2457	261.1479	pos	1.6747	5.7687	0.0064	K-GG	AMM1160	NA	NA	K-GG	C10H20N4O4	260.1484	-
metab_13144	3.7488	262.1122	neg	1.5532	-3.5569	0.0050	7-Mercaptoheptanoylthreonine	AMM1190	C16594	NA	7-Mercaptoheptanoylthreonine	C11H21NO4S	263.1192	-
metab_1032	1.9463	262.1177	pos	1.1125	-1.6619	0.0104	Imazapyr	NA	NA	NA	Imazapyr	C13H15N3O3	261.1113	-
metab_7640	2.7155	263.1503	neg	1.4517	2.9996	0.0039	Hexyl glucoside	NA	NA	NA	Hexyl glucoside	C12H24O6	264.1573	HMDB0031688
metab_14257	1.5297	264.0551	neg	1.5309	-2.3014	0.0015	Endalin	NA	NA	NA	Endalin	C9H12FN3O3	229.0863	HMDB0041887
metab_6018	1.2162	264.0974	pos	2.3495	10.5303	0.0107	7-Mercaptoheptanoylthreonine	AMM1190	C16594	NA	7-Mercaptoheptanoylthreonine	C11H21NO4S	263.1192	-
metab_13053	4.0516	264.1244	neg	1.2160	-1.5769	0.0406	Ritalinic acid	NA	NA	NA	Ritalinic acid	C13H17NO2	219.1259	HMDB0042008
metab_2113	3.2727	265.1065	pos	1.4833	10.2967	0.0079	Thiamine	AMM1195	NA	NA	Thiamine	C12H16N4OS	264.1043	HMDB0000235
metab_71	3.3490	265.1428	pos	2.0483	4.3556	0.0000	Hirsutic acid C	AMM1198	C09683||C11060||C20399||C09540||C09304||C20402||C09542||C09355||C09456||C09688||C09531||C09530||C09453||C09349||C09470||C09600||C09481||C09708||C09498	Terpenoids	Hirsutic acid C	C15H20O4	264.1361	-

									||C10766||C09291||C09381||C09574||C09506					
metab_2286	4.1698	265.1428	pos	1.6678	4.5606	0.0306	Hirsutic acid C	AMM1198	C09683||C11060||C20399||C09540||C09304||C20402||C09542||C09355||C09456||C09688||C09531||C09530||C09453||C09349||C09470||C09600||C09481||C09708||C09498||C10766||C09291||C09381||C09574||C09506	Terpenoids	Hirsutic acid C	C15H20O4	264.1361	-
metab_14219	1.5439	267.0150	neg	1.6201	2.3660	0.0283	Formononetin	AMM1236	NA	Flavonoids	Formononetin	C16H12O4	268.0737	HMDB0005808
metab_9244	3.8839	267.0667	neg	1.5002	-4.1068	0.0069	Formononetin	AMM1236	NA	Flavonoids	Formononetin	C16H12O4	268.0737	-
metab_6003	1.2446	267.0968	pos	1.4368	2.7466	0.0016	Dinex	NA	NA	NA	Dinex	C12H14N2O5	266.0902	-
metab_79	4.3371	267.1584	pos	1.2437	1.8445	0.0002	Mukaadial	NA	NA	NA	Mukaadial	C15H22O4	266.1515	-
metab_79	4.3371	267.1584	pos	1.2437	1.8445	0.0002	Xanthoxic acid	NA	NA	NA	Xanthoxic acid	C15H22O4	266.1516	-
metab_482	2.8108	267.1584	pos	1.5726	3.2753	0.0003	Mukaadial	NA	NA	NA	Mukaadial	C15H22O4	266.1515	-
metab_482	2.8108	267.1584	pos	1.5726	3.2753	0.0003	Xanthoxic acid	NA	NA	NA	Xanthoxic acid	C15H22O4	266.1516	-
metab_9471	4.8865	267.1604	neg	1.0217	1.6765	0.0009	Dihydroartemisinic acid hydroperoxide	NA	NA	NA	Dihydroartemisinic acid hydroperoxide	C15H24O4	268.1673	-

metab_12528	6.2294	267.1605	neg	1.1144	1.6898	0.0001	Dihydroartemisinic acid hydroperoxide	NA	NA	NA	Dihydroartemisinic acid hydroperoxide	C15H24O4	268.1673	HMDB0037605
metab_12528	6.2294	267.1605	neg	1.1144	1.6898	0.0001	(10Betah,11xi)-11-Hydroxy-13-nor-6-eremophilen-8-one	NA	NA	NA	(10Betah,11xi)-11-Hydroxy-13-nor-6-eremophilen-8-one	C14H22O2	222.1620	HMDB0037605
metab_7690	3.1626	267.1605	neg	1.2772	2.8723	0.0050	Dihydroartemisinic acid hydroperoxide	NA	NA	NA	Dihydroartemisinic acid hydroperoxide	C15H24O4	268.1673	-
metab_9153	3.5813	267.1605	neg	2.0788	5.4679	0.0042	dihydroartemisinic acid hydroperoxide	AMM1246	NA	NA	Dihydroartemisinic acid hydroperoxide	C15H24O4	268.1673	-
metab_13085	3.9177	267.1605	neg	1.6623	4.0792	0.0019	dihydroartemisinic acid hydroperoxide	AMM1246	NA	NA	Dihydroartemisinic acid hydroperoxide	C15H24O4	268.1673	-
metab_2420	5.0451	267.1947	pos	1.4147	2.0264	0.0202	Juvenile hormone III	AMM1224	C09694||C09693||C04834	Terpenoids	Juvenile hormone III	C16H26O3	266.1880	-
metab_557	3.7447	267.1948	pos	2.4506	7.2648	0.0080	Juvenile hormone III	AMM1224	C09694||C09693||C04834	Terpenoids	Juvenile hormone III	C16H26O3	266.188	-
metab_9173	3.6479	269.0460	neg	1.2824	7.2812	0.0267	3,7,4'-Trihydroxyflavone	AMM1264	NA	Flavonoids	3,7,4'-Trihydroxyflavone	C15H10O5	270.0527	HMDB0034004
metab_9173	3.6479	269.0460	neg	1.2824	7.2812	0.0267	Genistein	NA	NA	Flavonoids_pubchem	Genistein	C15H10O5	270.0527	HMDB0003217
metab_4565	5.8753	269.1739	pos	1.1764	-3.0787	0.0137	Dihydroartemisinic acid hydroperoxide	NA	NA	NA	Dihydroartemisinic acid hydroperoxide	C15H24O4	268.1672	-
metab_17	2.4530	269.1740	pos	1.5447	3.4868	0.0013	dihydroartemisinic acid hydroperoxide	AMM1243	NA	NA	Dihydroartemisinic acid hydroperoxide	C15H24O4	268.1672	-
metab_4789	4.6398	269.2104	pos	2.2535	6.4155	0.0114	dihydroartemisinic acid hydroperoxide	AMM1243	NA	NA	dihydroartemisinic acid hydroperoxide	C15H24O4	268.1672	-
metab_13153	3.7317	271.1554	neg	1.0373	1.9464	0.0295	4,5-Dihydrovmifoliol	NA	NA	NA	4,5-Dihydrovmifoliol	C13H22O3	226.1569	HMDB0040615

metab_9718	6.3269	271.1916	neg	1.1824	3.4031	0.0092	5-Tetradecenoic acid	NA	NA	NA	5-Tetradecenoic acid	C14H26O2	226.1933	HMDB0000499
metab_9339	4.2702	272.0932	neg	1.5227	-3.0611	0.0129	L-Thyronine	NA	NA	NA	L-Thyronine	C15H15N O4	273.1001	HMDB0000667
metab_343	1.5284	274.2004	pos	1.6826	6.2509	0.0069	(2R,3R)-3-Methylglutam yl-5-semialdehyde-N6-ly sine	AMM1306	C20279	NA	(2R,3R)-3-Methylglut amyl-5-semialdehyde-N6-lysine	C12H23N3 O4	273.169	-
metab_5422	2.3908	275.0908	pos	2.5223	7.7115	0.0000	3,4,4',7-Tetrahydroxyflav an	NA	NA	NA	3,4,4',7-Tetrahydroxyf lavan	C15H14O5	274.0841	HMDB0040828
metab_13408	3.0260	275.1504	neg	1.6629	3.4216	0.0024	p-Coumaroylagmatine	AMM1360	C04498	NA	p-Coumaroylagmatine	C14H20N4 O2	276.1575	HMDB0030143
metab_1326	0.6402	276.0472	pos	1.2209	-2.4832	0.0494	2-Phthalimidoglutaric acid	NA	NA	NA	2-Phthalimidoglutaric acid	C13H11N O6	275.0408	-
metab_1326	0.6402	276.0472	pos	1.2209	-2.4832	0.0494	D-Glucosaminic acid 6-phosphate	NA	NA	NA	D-Glucosaminic acid 6-phosphate	C6H14NO 9P	275.0408	-
metab_322	1.4429	276.0684	pos	1.4984	16.1933	0.0009	D-Glucosaminate-6-phos phate	AMM1332	C20589	NA	D-Glucosaminate-6-ph osphate	C6H14NO 9P	275.0408	-
metab_8886	2.7475	277.0720	neg	1.6911	3.7590	0.0216	3-(1,2-Dihydroxybut-3-e n-1-yl)-1H-isochromen-1 -one	NA	NA	NA	3-(1,2-Dihydroxybut-3 -en-1-yl)-1H-isochrom en-1-one	C13H12O4	232.0736	HMDB0130065
metab_14180	1.5885	277.1303	neg	1.5241	4.2819	0.0095	N-Benzoyl-D-arginine	AMM1394	C03001||C07424	NA	N-Benzoyl-D-arginine	C13H18N4 O3	278.1366	-
metab_14180	1.5885	277.1303	neg	1.5241	4.2819	0.0095	Pentoxifylline	AMM1395	C07424||C03001	NA	Pentoxifylline	C13H18N4 O3	278.1366	-
metab_12885	4.6380	278.1400	neg	1.2826	-1.8423	0.0159	Thalictroidine	NA	NA	NA	Thalictroidine	C14H19N O2	233.1416	HMDB0034684
metab_13455	2.9092	279.0987	neg	2.0274	7.2958	0.0011	H-PHE-ASP-OH	AMM1440	NA	NA	H-PHE-ASP-OH	C13H16N2	280.1059	-

												O5		
metab_13455	2.9092	279.0987	neg	2.0274	7.2958	0.0011	L-Aspartyl-L-phenylalanine	NA	NA	NA	L-Aspartyl-L-phenylalanine	C13H16N2O5	280.1059	-
metab_7316	8.7031	279.2332	neg	1.2157	-1.3170	0.0001	Linoleic acid	NA	NA	NA	Linoleic acid	C18H32O2	280.2403	-
metab_7973	0.7818	281.0880	neg	1.0476	-1.0167	0.0003	1-Methylinosine	NA	NA	NA	1-Methylinosine	C11H14N4O5	282.0964	HMDB0002721
metab_13302	3.2972	281.1398	neg	1.9499	4.8359	0.0001	Hymenoxon	AMM1485	C09482||C15971||C09538	Terpenoids	Hymenoxon	C15H22O5	282.1468	HMDB0040778
metab_9243	3.8839	281.1398	neg	2.3849	6.7561	0.0002	Cynaratriol	NA	NA	NA	(3R,3Ar,6aR,8S,9S,9aR,9bR)-3,8-dihydroxy-3-(hydroxymethyl)-9-methyl-6-methylidene-4,5,6a,7,8,9,9a,9b-octahydro-3aH-azuleno[4,5-b]furan-2-one	C15H22O5	282.1467	HMDB0034983
metab_9243	3.8839	281.1398	neg	2.3849	6.7561	0.0002	Hymenoxon	AMM1485	C09482||C15971||C09538	Terpenoids	Hymenoxon	C15H22O5	282.1468	HMDB0034983
metab_1653	1.6413	281.1487	pos	1.2888	1.8722	0.0000	Tyrosyl-Valine	NA	NA	NA	Tyrosyl-Valine	C14H20N2O4	280.1423	-
metab_2945	8.7161	281.1528	pos	1.3018	12.1812	0.0061	Tyr Val	AMM1447	NA	NA	Tyr Val	C14H20N2O4	280.1423	-
metab_659	5.9049	281.1740	pos	1.6873	3.3898	0.0044	3,4-Epoxy-6-methylcyclohexylmethyl-3,4-epoxy-6-methylcyclo-hexanecarboxylate	AMM1449	C19417||C17748	NA	Chissonox 201	C16H24O4	280.1672	-
metab_5906	1.4289	281.1853	pos	1.0679	11.6319	0.0154	3,4-Epoxy-6-methylcycl	AMM1449	C19417||C17748	NA	3,4-Epoxy-6-methylcy	C16H24O4	280.1672	-

							ohexylmethyl-3,4-epoxy-6-methylcyclo-hexanecarboxylate				clohexylmethyl-3,4-epoxy-6-methylcyclo-hexanecarboxylate			
metab_6343	0.6123	282.0480	pos	1.0827	-1.5321	0.0003	Kathon 930	NA	NA	NA	Kathon 930	C11H17Cl2NOS	281.0413	-
metab_3051	9.4049	282.2664	pos	1.0973	1.7031	0.0093	Oleamide	AMM1476	C19670||C18786	NA	Oleamide	C18H35NO	281.2716	HMDB0002117
metab_14044	1.7885	283.0581	neg	1.4183	1.0421	0.0406	Biochanin A	AMM1509	NA	Flavonoids	Biochanin A	C16H12O5	284.0681	HMDB0002338
metab_14044	1.7885	283.0581	neg	1.4183	1.0421	0.0406	Glycitein	AMM1510	NA	Flavonoids	Glycitein	C16H12O5	284.0682	HMDB0005781
metab_14044	1.7885	283.0581	neg	1.4183	1.0421	0.0406	Acacetin	AMM1512	NA	Polyketides	Acacetin	C16H12O5	284.0686	-
metab_6852	0.5991	283.1036	neg	1.6066	-2.6896	0.0002	Coformycin	NA	NA	NA	Coformycin	C11H16N4O5	284.1108	-
metab_505	3.0422	283.1531	pos	2.0544	5.2470	0.0000	Qing Hau Sau	AMM1484	C09538||C09482||C15971	Terpenoids	Artemisinin	C15H22O5	282.1461	-
metab_1837	2.2209	283.1532	pos	2.3211	5.3370	0.0000	Qing Hau Sau	AMM1484	C09538||C09482||C15971	Terpenoids	Artemisinin	C15H22O5	282.1461	-
metab_6972	4.3380	283.1554	neg	1.0211	1.5408	0.0001	alpha-Dihydroartemisinin	NA	NA	NA	alpha-Dihydroartemisinin	C15H24O5	284.1624	HMDB0060593
metab_1810	2.1290	284.1271	pos	1.5345	10.0188	0.0062	Ala-Gly-His	AMM1503	NA	NA	Ala-Gly-His	C11H17N5O4	283.1266	-
metab_5854	1.5284	284.1484	pos	1.3747	-2.1509	0.0008	Hydroxyisovaleroyl carnitine	NA	NA	NA	Hydroxyisovaleroyl carnitine	C12H23NO5	261.1576	LMFA07070073; HMDB0062555
metab_1420	0.9620	285.1074	pos	1.3913	2.2969	0.0037	Calycosin	NA	NA	Flavonoids_pubchem	Calycosin	C16H12O5	284.068473	-
metab_6222	0.836	285.1437	pos	1.7046	3.7113	0.0262	Biochanin A	AMM1509	NA	Flavonoids	Biochanin A	C16H12O5	284.0681	HMDB0002338
metab_6222	0.836	285.1437	pos	1.7046	3.7113	0.0262	Glycitein	AMM1510	NA	Flavonoids	Glycitein	C16H12O5	284.0682	HMDB0005781

metab_6222	0.836	285.1437	pos	1.7046	3.7113	0.0262	Acacetin	AMM1512	NA	Polyketides	Acacetin	C16H12O5	284.0686	-
metab_2011	2.8877	285.1688	pos	1.8841	13.0128	0.0001	CHEBI:69050	AMM1516	NA	Terpenoids_pubchem	Artenimol	C15H24O5	284.1622	-
metab_2011	2.8877	285.1688	pos	1.8841	13.0128	0.0001	Dihydroartemisinin	AMM1521	NA	Terpenoids_pubchem	Merulin D	C15H24O5	284.1621	-
metab_1831	2.1897	285.1688	pos	2.3523	7.2709	0.0022	Artenimol	NA	NA	NA	Artenimol	C15H24O5	284.1622	-
metab_1831	2.1897	285.1688	pos	2.3523	7.2709	0.0022	Merulin D	NA	NA	NA	Merulin D	C15H24O5	284.1621	-
metab_1831	2.1897	285.1688	pos	2.3523	7.2709	0.0022	Dihydroartemisinin	AMM1514	NA	Terpenoids_pubchem	Dihydroartemisinin	C15H24O5	284.1621	-
metab_9450	4.7709	285.2074	neg	1.7751	4.0202	0.0269	Hexadecanedioic acid	AMM1545	NA	NA	Hexadecanedioic acid	C16H30O4	286.2146	-
metab_1163	0.5140	287.0567	pos	1.0701	1.6435	0.0188	Kaempferol	NA	NA	Flavonoids	Kaempferol	C15H10O6	286.047738	HMDB0005801
metab_13386	3.0774	287.1502	neg	1.4218	3.3330	0.0090	Asc-DeltaC8	NA	NA	NA	Asc-DeltaC8	C14H24O6	288.1575	HMDB0038736
metab_8613	2.0516	287.1505	neg	2.0500	4.8247	0.0006	asc-DeltaC8	AMM1570	NA	NA	Asc-DeltaC8	C14H24O6	288.1575	-
metab_6047	1.1875	287.1593	pos	1.1352	3.1082	0.0154	Abacavir	NA	NA	NA	Abacavir	C14H18N6O	286.1527	-
metab_8217	1.3373	288.1568	neg	1.7963	3.5417	0.0001	Leu-Ala-Ser	AMM1586	NA	NA	Leu-Ala-Ser	C12H23N3O5	289.1638	-
metab_5575	2.0677	288.1910	pos	1.6054	3.6620	0.0038	Leu-Val-Gly	AMM1548	NA	NA	Leu-Val-Gly	C13H25N3O4	287.1844	-
metab_5575	2.0677	288.1910	pos	1.6054	3.6620	0.0038	Gly Val Leu	AMM1549	NA	NA	Glycyl-valyl-leucine	C13H25N3O4	287.1845	-
metab_6122	1.0601	288.2021	pos	1.2553	2.4676	0.0092	Arginyl-Leucine	NA	NA	NA	Arginyl-Leucine	C12H25N5O3	287.1956	-
metab_2272	4.0634	288.2161	pos	1.5090	-3.9253	0.0096	Octanoylcarnitine	NA	NA	NA	Octanoylcarnitine	C15H29NO4	287.2097	-

metab_5961	1.3293	289.1749	pos	1.4390	12.5264	0.0014	Estriol	AMM1573	C05141||C05301||C14209||C03935||C15385	NA	Estriol	C18H24O3	288.1723	HMDB0000153
metab_4958	3.8967	289.1791	pos	1.7213	7.2567	0.0041	Estriol	AMM1573	C05141||C05301||C14209||C03935||C15385	NA	Estriol	C18H24O3	288.1723	-
metab_4958	3.8967	289.1791	pos	1.7213	7.2567	0.0041	3-Methoxyestra-2,5(10)-dien-17beta-ol	AMM1578	C14682||C14491||C01227||C15335||C15258||C00535||C15395||C15377||C03772	Terpenoids	3-Methoxyestra-2,5(10)-dien-17beta-ol	C19H28O2	288.2087	-
metab_1235	0.5140	290.2430	pos	2.0897	13.8297	0.0000	Leu-Ala-Ser	AMM1585	NA	NA	Leu-Ala-Ser	C12H23N3O5	289.1637	-
metab_487	2.8259	291.0856	pos	2.6599	8.1098	0.0000	Plumericin	AMM1596	C09796||C09030||C09736||C05907||C17590||C03648||C09511||C08739||C08742	Terpenoids	Plumericin	C15H14O6	290.0787	-
metab_5430	2.3751	291.1332	pos	1.1454	-2.0730	0.0008	Piperalol	NA	NA	NA	Piperalol	C15H24O3	252.1725	HMDB0035767
metab_5695	1.7993	291.1694	pos	1.2788	12.0429	0.0178	4-Androstenediol	AMM1611	C14210||C04295||C15442||C15326||C14932||C05293||C01986||C07635	NA	4-Androstenediol	C19H30O2	290.2243	HMDB0005849
metab_4421	6.6606	291.1946	pos	1.0904	1.2072	0.0005	8-(4-Oxo-5-pent-2-enylcyclopent-2-en-1-yl)octan	NA	NA	NA	8-(4-Oxo-5-pent-2-enylcyclopent-2-en-1-yl)o	C18H28O3	290.1879	-

							oic acid				ctanoic acid			
metab_4421	6.6606	291.1946	pos	1.0904	1.2072	0.0005	ML-236C	NA	NA	NA	ML-236C	C18H26O3	290.1878	-
metab_13771	2.2595	292.1309	neg	1.9551	-3.9654	0.0002	Gln Phe	AMM1669	NA	NA	Glutaminylphenylalanine	C14H19N3O4	293.1375	-
metab_5169	3.1032	293.1011	pos	1.3330	11.6768	0.0259	N4-Acetylsulfadiazine	AMM1647	NA	NA	N4-Acetylsulfadiazine	C12H12N4O3S	292.0559	-
metab_13059	4.0182	293.1071	neg	1.1934	4.8443	0.0296	Aspartame	AMM1685	C11045	NA	Aspartame	C14H18N2O5	294.1214	HMDB0001894
metab_7494	1.7729	293.1147	neg	1.0114	-1.0216	0.0021	Aspartame	NA	NA	NA	Aspartame	C14H18N2O5	294.1216	HMDB0029106
metab_7494	1.7729	293.1147	neg	1.0114	-1.0216	0.0021	Phenylalanylglutamate	NA	NA	NA	Phenylalanylglutamate	C14H18N2O5	294.1215	HMDB0029106
metab_2988	9.0112	293.2465	pos	1.1254	3.5114	0.0143	9(Z),11(E),13(E)-Octadecatrienoic Acid methyl ester	AMM1663	NA	NA	9(Z),11(E),13(E)-Octadecatrienoic Acid methyl ester	C19H32O2	292.2399	-
metab_3073	9.6044	293.2466	pos	1.4044	4.1984	0.0074	9(Z),11(E),13(E)-Octadecatrienoic Acid methyl ester	AMM1663	NA	NA	Methyl eleostearate	C19H32O2	292.2399	-
metab_12016	7.9757	293.2490	neg	1.7835	-7.3229	0.0015	14-methyl palmitic acid	AMM1709	NA	Fatty acids related compounds	14-Methylhexadecanoic acid	C17H34O2	294.2563	-
metab_13761	2.2912	294.0988	neg	1.0668	-1.2697	0.0029	Tyramine glucuronide	NA	NA	NA	Tyramine glucuronide	C14H19NO7	313.1162	HMDB0010328
metab_1705	1.7845	295.1280	pos	1.0224	-1.3711	0.0015	Aspartame	NA	NA	NA	Aspartame	C14H18N2O5	294.1214	-
metab_1705	1.7845	295.1280	pos	1.0224	-1.3711	0.0015	Phenylalanylglutamate	NA	NA	NA	Phenylalanylglutamate	C14H18N2	294.1215	-

												O5		
metab_2282	4.1387	295.1529	pos	1.7610	12.4412	0.0009	H-Ile-Tyr-OH	AMM1693	NA	NA	H-Ile-Tyr-OH	C15H22N2 O4	294.158	-
metab_6568	7.0162	295.2281	neg	1.0857	-1.4839	0.0249	(9S,10E,12Z)-9-Hydroxy octadeca-10,12-dienoic acid	NA	NA	NA	(9S,10E,12Z)-9-Hydro xyoctadeca-10,12-dien oic acid	C18H32O3	296.2352	-
metab_6568	7.0162	295.2281	neg	1.0857	-1.4839	0.0249	Vernolic acid	NA	NA	NA	Vernolic acid	C18H32O3	296.2352	-
metab_6411	0.5560	296.0651	pos	1.2222	1.6129	0.0075	Choline Alfoscerate	NA	NA	NA	Choline Alfoscerate	C8H20NO 6P	791.5829	HMDB0011228; HMDB0011221; HMDB0011222; HMDB0011223; HMDB0011224; HMDB0008753; HMDB0011226; HMDB0011227; HMDB0008488; HMDB0008489; HMDB0008161; HMDB0008160; HMDB0008162; HMDB0008619; HMDB0008360; HMDB0011290; HMDB0008685; HMDB0008687; HMDB0008721;

														HMDB0008720;<br>HMDB0008817;<br>HMDB0008027;<br>HMDB0007962;<br>HMDB0007963;<br>HMDB0008260;<br>HMDB0008261;<br>HMDB0011225;<br>HMDB0008587;<br>HMDB0008586;<br>HMDB0008060;<br>HMDB0008061;<br>HMDB0008062;<br>HMDB0008063;<br>HMDB0008589;<br>HMDB0008588;<br>HMDB0008785;<br>HMDB0007895;<br>HMDB0007896;<br>HMDB0008786;<br>HMDB0008622;<br>HMDB0008620;<br>HMDB0008621;<br>HMDB0008455;<br>HMDB0008820;<br>HMDB0008457;

														HMDB0008193;<br>HMDB0008194;<br>HMDB0008195;<br>HMDB0008784;<br>HMDB0008458;<br>HMDB0008357;<br>HMDB0008293;<br>HMDB0008292;<br>HMDB0008358;<br>HMDB0008359;<br>HMDB0008294;<br>HMDB0008259;<br>HMDB0008258;<br>HMDB0008521;<br>HMDB0008523;<br>HMDB0008524;<br>HMDB0008819;<br>HMDB0008818;<br>HMDB0008653;<br>HMDB0008652;<br>HMDB0008718;<br>HMDB0008719;<br>HMDB0008654;<br>HMDB0008327;<br>HMDB0008326;<br>HMDB0008325;

														HMDB0008128;<br>HMDB0008127;<br>HMDB0008456;<br>HMDB0008093;<br>HMDB0008030;<br>HMDB0000086;<br>HMDB0008787;<br>HMDB0008228;<br>HMDB0008424;<br>HMDB0008425;<br>HMDB0008226;<br>HMDB0008227;<br>HMDB0008422;<br>HMDB0008225;<br>HMDB0008423;<br>HMDB0007931;<br>HMDB0007930;<br>HMDB0007995;<br>HMDB0007994;<br>HMDB0007997;<br>HMDB0007996;<br>HMDB0008392;<br>HMDB0008393;<br>HMDB0008390;<br>HMDB0008391;<br>HMDB0008291;

														HMDB0008095; HMDB0008094; HMDB0008096; HMDB0008554; HMDB0008555; HMDB0008556; HMDB0008557; HMDB0008491; HMDB0008490; HMDB0007928; HMDB0007929; HMDB0008159
metab_12812	4.9183	296.1144	neg	1.2818	4.0805	0.0184	Phenyl 2-acetamido-2-deoxy-alpha-D-glucopyranoside	AMM1757	NA	NA	Phenyl 2-acetamido-2-deoxy-alpha-D-glucopyranoside	C14H19NO6	297.1212	-
metab_4881	4.2609	297.1687	pos	2.0598	4.6706	0.0006	Alhpa-tocopheronic acid	NA	NA	NA	Alhpa-tocopheronic acid	C16H24O5	296.1624	LMPR02020062
metab_5518	2.1746	298.1277	pos	1.1873	-1.5677	0.0000	Phenyl 2-acetamido-2-deoxy-alpha-D-glucopyranoside	NA	NA	NA	Phenyl 2-acetamido-2-deoxy-alpha-D-glucopyranoside	C14H19NO6	297.1212	-
metab_2793	7.8097	298.2732	pos	1.1309	-1.2706	0.0091	3-Ketosphingosine	NA	NA	NA	3-Ketosphingosine	C18H35NO2	297.2666	-
metab_2793	7.8097	298.2732	pos	1.1309	-1.2706	0.0091	Cassine	NA	NA	NA	Cassine	C18H35NO2	297.2668	-

metab_2230	3.8063	299.1839	pos	1.2489	3.5501	0.0205	Iso-Olomoucine	AMM1772	NA	NA	Iso-Olomoucine	C15H18N6O	298.1604	-
metab_122	7.5005	299.2572	pos	1.2483	1.9856	0.0427	9,10-Epoxystearic acid	NA	NA	NA	9,10-Epoxystearic acid	C18H34O3	298.2510	-
metab_3916	8.9656	299.2573	pos	1.2031	1.8825	0.0160	9,10-Epoxystearic acid	NA	NA	NA	9,10-Epoxystearic acid	C18H34O3	298.2510	-
metab_3839	9.3117	299.2574	pos	1.4514	2.3313	0.0022	cis-9,10-Epoxystearic acid	AMM1781	NA	Fatty acids related compounds	9,10-Epoxystearic acid	C18H34O3	298.2510	-
metab_13874	2.0670	300.1932	neg	1.0178	1.4139	0.0119	Leu-Leu-Gly	NA	NA	NA	Leu-Leu-Gly	C14H27N3O4	301.2001	-
metab_13874	2.0670	300.1932	neg	1.0178	1.4139	0.0119	Val-Leu-Ala	NA	NA	NA	Val-Leu-Ala	C14H27N3O4	301.2001	-
metab_13624	2.5431	300.1932	neg	1.5272	2.6407	0.0014	Val-Leu-Ala	AMM1831	NA	NA	Val-Leu-Ala	C14H27N3O4	301.2001	-
metab_13624	2.5431	300.1932	neg	1.5272	2.6407	0.0014	Leu-Leu-Gly	AMM1833	NA	NA	Leu-Leu-Gly	C14H27N3O4	301.2001	-
metab_8872	2.6986	300.1933	neg	1.7261	3.7975	0.0001	Val Leu Ala	AMM1831	NA	NA	Val-Leu-Ala	C14H27N3O4	301.2001	-
metab_8872	2.6986	300.1933	neg	1.7261	3.7975	0.0001	H-LEU-LEU-GLY-OH	AMM1833	NA	NA	Leu-Leu-Gly	C14H27N3O4	301.2001	-
metab_14227	1.5439	300.1934	neg	2.0301	5.6264	0.0000	Gly Leu Leu	AMM1826	NA	NA	Gly Leu Leu	C14H27N3O4	301.1999	-
metab_14227	1.5439	300.1934	neg	2.0301	5.6264	0.0000	Gly Ile Ile	AMM1827	NA	NA	Gly Ile Ile	C14H27N3O4	301.2	-
metab_14227	1.5439	300.1934	neg	2.0301	5.6264	0.0000	Val-Leu-Ala	AMM1831	NA	NA	Val-Leu-Ala	C14H27N3O4	301.2001	-
metab_14227	1.5439	300.1934	neg	2.0301	5.6264	0.0000	Leu-Leu-Gly	AMM1833	NA	NA	Leu-Leu-Gly	C14H27N3	301.2001	-

												O4		
metab_13751	2.3078	301.1299	neg	3.1797	9.7897	0.0000	Isomucronulatol	NA	NA	Flavonoids_pubchem	Isomucronulatol	C17H18O5	302.115424	HMDB0033189
metab_6468	0.5140	301.1498	pos	1.1329	3.1077	0.0285	(-)-Sparticarpin	AMM1813	C10531||C09734||C10461||C10539||C17047||C17416||C10251||C17503	Flavonoids	(-)-Sparticarpin	C17H16O5	300.0996	-
metab_6468	0.5140	301.1498	pos	1.1329	3.1077	0.0285	Astrapterocarpan	NA	NA	NA	Astrapterocarpan	C17H16O5	300.099774	-
metab_7856	0.5286	301.1632	neg	1.3500	4.0310	0.0105	Tributyrin	AMM1844	C13870	NA	Tributyrin	C15H26O6	302.1727	HMDB0031094
metab_7617	2.5748	301.1661	neg	1.2498	2.8215	0.0094	Tributyrin	NA	NA	NA	Tributyrin	C15H26O6	302.1735	-
metab_8507	1.8205	301.1773	neg	1.1363	2.5707	0.0008	Abietic acid	AMM1847	NA	Terpenoids	Abietic acid	C20H30O2	302.2246	-
metab_1613	1.5284	302.2064	pos	1.9289	3.3792	0.0000	Gly Leu Leu	AMM1826	NA	NA	Glycyl-L-leucyl-L-leucine	C14H27N3O4	301.1999	-
metab_1613	1.5284	302.2064	pos	1.9289	3.3792	0.0000	Gly Ile Ile	AMM1827	NA	NA	Gly-Ile-Ile	C14H27N3O4	301.2000	-
metab_1613	1.5284	302.2064	pos	1.9289	3.3792	0.0000	Leu Ala Val	AMM1828	NA	NA	Leu-Ala-Val	C14H27N3O4	301.2000	-
metab_1613	1.5284	302.2064	pos	1.9289	3.3792	0.0000	Val Ala Ile	AMM1829	NA	NA	Val-Ala-Ile	C14H27N3O4	301.2000	-
metab_1613	1.5284	302.2064	pos	1.9289	3.3792	0.0000	H-LEU-LEU-GLY-OH	AMM1830	NA	NA	Leu-Leu-Gly	C14H27N3O4	301.2000	-
metab_1613	1.5284	302.2064	pos	1.9289	3.3792	0.0000	Val Leu Ala	AMM1831	NA	NA	Val-Leu-Ala	C14H27N3O4	301.2001	-
metab_1806	2.1290	302.2065	pos	1.3510	1.5875	0.0053	Glycyl-L-leucyl-L-leucine	NA	NA	NA	Glycyl-L-leucyl-L-leucine	C14H27N3O4	301.1999	-

metab_1806	2.1290	302.2065	pos	1.3510	1.5875	0.0053	Gly-Ile-Ile	NA	NA	NA	Gly-Ile-Ile	C14H27N3O4	301.2000	-
metab_1806	2.1290	302.2065	pos	1.3510	1.5875	0.0053	Leu-Ala-Val	NA	NA	NA	Leu-Ala-Val	C14H27N3O4	301.2000	-
metab_1806	2.1290	302.2065	pos	1.3510	1.5875	0.0053	Val-Ala-Ile	NA	NA	NA	Val-Ala-Ile	C14H27N3O4	301.2000	-
metab_1806	2.1290	302.2065	pos	1.3510	1.5875	0.0053	Leu-Leu-Gly	NA	NA	NA	Leu-Leu-Gly	C14H27N3O4	301.2000	-
metab_1806	2.1290	302.2065	pos	1.3510	1.5875	0.0053	Val-Leu-Ala	NA	NA	NA	Val-Leu-Ala	C14H27N3O4	301.2001	-
metab_5291	2.7188	302.2066	pos	2.8599	7.1148	0.0000	Gly Leu Leu	AMM1826	NA	NA	Gly Leu Leu	C14H27N3O4	301.1999	-
metab_5291	2.7188	302.2066	pos	2.8599	7.1148	0.0000	Gly Ile Ile	AMM1827	NA	NA	Gly-Ile-Ile	C14H27N3O4	301.2000	-
metab_5291	2.7188	302.2066	pos	2.8599	7.1148	0.0000	Leu Ala Val	AMM1828	NA	NA	Leu-Ala-Val	C14H27N3O4	301.2000	-
metab_5291	2.7188	302.2066	pos	2.8599	7.1148	0.0000	Val Ala Ile	AMM1829	NA	NA	Val-Ala-Ile	C14H27N3O4	301.2000	-
metab_5291	2.7188	302.2066	pos	2.8599	7.1148	0.0000	H-LEU-LEU-GLY-OH	AMM1830	NA	NA	Leu-Leu-Gly	C14H27N3O4	301.2000	-
metab_5291	2.7188	302.2066	pos	2.8599	7.1148	0.0000	Val Leu Ala	AMM1831	NA	NA	Val-Leu-Ala	C14H27N3O4	301.2001	-
metab_9256	3.9177	303.1818	neg	1.7152	7.4634	0.0065	16beta,17beta-Dihydroxy-16-methylestr-4-en-3-one	AMM1864	C15144||C14600||C14649||C14627||C05139||C18075||C05294||C052	NA	16beta,17beta-Dihydroxy-16-methylestr-4-en-3-one	C19H28O3	304.2035	-

									91||C14497||C21296||C18045||C14671||C15332||C14912||C15416||C15286					
metab_4947	3.9421	304.1746	pos	1.7900	5.3773	0.0000	Leu-Thr-Ala	AMM1855	NA	NA	Leu-Thr-Ala	C13H25N3O5	303.1793	-
metab_4762	4.8208	305.2102	pos	1.3583	4.6422	0.0239	7alpha-Hydroxytestosterone	AMM1866	C05291||C05294||C18075||C14627||C14497||C15144||C21296||C14600||C14649||C18045||C05139||C15332||C15286||C15416||C14671||C14912	NA	7alpha-Hydroxytestosterone	C19H28O3	304.2037	-
metab_4762	4.8208	305.2102	pos	1.3583	4.6422	0.0239	16beta,17beta-Dihydroxy-16-methylestr-4-en-3-one	AMM1864	C15144||C14600||C14649||C14627||C05139||C18075||C05294||C05291||C14497||C21296||C18045||C14671||C15332||C14912||C15416||C15286	NA	16beta,17beta-Dihydroxy-16-methylestr-4-en-3-one	C19H28O3	304.2035	-
metab_1579	1.4429	306.1802	pos	1.3537	14.2375	0.0100	6'-Oxolividamine	AMM1872	C21256||C21257	NA	6'-Oxolividamine	C12H23N3	305.1586	-

												O6		
metab_13062	4.0182	306.2079	neg	1.3408	2.3313	0.0007	N-[[3-Hydroxy-2-(2-pentenyl)cyclopentyl]acetyl]isoleucine	NA	NA	NA	N-[[3-Hydroxy-2-(2-pentenyl)cyclopentyl]acetyl]isoleucine	C18H31NO4	325.2253	HMDB0041248
metab_4395	6.8433	306.2418	pos	1.0864	11.1954	0.0328	Capsaicin	AMM1874	NA	Amino acid related compounds	Capsaicin	C18H27NO3	305.1989	HMDB0002227
metab_12966	4.3207	307.1556	neg	1.7909	7.2277	0.0021	Ubenimex	AMM1929	C00732	NA	Ubenimex	C16H24N2O4	308.1735	-
metab_584	4.2457	307.1896	pos	1.5364	2.2334	0.0012	Compactin diol lactone	AMM1895	C14183||C20203||C14300	Amino acid related compounds	Compactin diol lactone	C18H26O4	306.1828	-
metab_611	3.9270	307.1897	pos	1.3904	2.1088	0.0069	Compactin diol lactone	NA	NA	NA	Compactin diol lactone	C18H26O4	306.1828	-
metab_4275	7.3370	307.2259	pos	1.4054	1.9757	0.0022	Androst-5-ene-3beta,17beta,19-triol	AMM1898	C15097||C14606||C07346||C15536||C15136	NA	Androst-5-ene-3beta,17beta,19-triol	C19H30O3	306.2192	-
metab_5000	3.7148	307.2260	pos	1.5201	12.0061	0.0090	Androst-5-ene-3beta,17beta,19-triol	AMM1898	C15097||C14606||C07346||C15536||C15136	NA	Androst-5-ene-3beta,17beta,19-triol	C19H30O3	306.2192	-
metab_4178	7.9118	307.2623	pos	1.8154	-3.3599	0.0005	2,7,11-Cembratrien-4,6-diol	AMM1906	C09072||C16522||C03242||C13273||C15058||C15399||C15376	Terpenoids	11,14,17-Eicosatrienoic acid	C20H34O2	306.2557	-
metab_4178	7.9118	307.2623	pos	1.8154	-3.3599	0.0005	Icosatrienoic acid	AMM1908	C16522||C03242||C09072||C13273	Terpenoids	Cembra-2,7,11-triene-4,6-diol	C20H34O2	306.2556	-

									||C15058||C15399||C15376					
metab_1809	2.1290	308.0901	pos	1.9717	4.8007	0.0000	N-Acetyl-b-neuraminic acid	AMM1912	NA	NA	NA	C11H19NO9	307.0842	-
metab_1604	1.5144	308.1848	pos	1.2235	9.1460	0.0005	Tebuconazole	AMM1915	C18489	NA	Tebuconazole	C16H22ClN3O	307.1452	-
metab_4411	6.7516	308.2211	pos	1.3466	1.9395	0.0017	Betaxolol	NA	NA	NA	Betaxolol	C18H29NO3	307.2147	-
metab_570	4.0479	308.2212	pos	1.2900	1.9366	0.0003	Betaxolol	NA	NA	NA	Betaxolol	C18H29NO3	307.2147	-
metab_5236	2.8877	309.1589	pos	1.7385	2.9842	0.0432	Isopropalin	AMM1954	C19063	NA	Isopropalin	C15H23N3O4	309.1688	-
metab_2533	5.9049	309.2050	pos	1.0143	1.5015	0.0007	5-O-Methylembelin	NA	NA	NA	5-O-Methylembelin	C18H28O4	308.1984	-
metab_2533	5.9049	309.2050	pos	1.0143	1.5015	0.0007	Soraphen O	NA	NA	NA	Soraphen O	C18H28O4	308.1986	-
metab_4427	6.6001	309.2051	pos	1.0583	1.1515	0.0012	5-O-Methylembelin	NA	NA	NA	5-O-Methylembelin	C18H28O4	308.1984	HMDB0033243
metab_4427	6.6001	309.2051	pos	1.0583	1.1515	0.0012	Soraphen O	NA	NA	NA	Soraphen O	C18H28O4	308.1986	HMDB0033243
metab_4427	6.6001	309.2051	pos	1.0583	1.1515	0.0012	Corchorifatty acid D	NA	NA	NA	Corchorifatty acid D	C18H28O4	308.1988	HMDB0033243
metab_679	6.5395	309.2415	pos	1.4653	4.1341	0.0162	Methyl (10E,12Z,15Z)-9-hydroxyoctadeca-10,12,15-trienoate	AMM1936	NA	NA	(10E,12Z,15Z)-9-Hydroxy-10,12,15-octadecatrienoic acid methyl ester	C19H32O3	308.2348	-
metab_4197	7.7946	309.2415	pos	1.5067	2.5408	0.0077	(10E,12Z,15Z)-9-Hydroxy-10,12,15-octadecatrienoic acid methyl ester	NA	NA	NA	(10E,12Z,15Z)-9-Hydroxy-10,12,15-octadecatrienoic acid methyl ester	C19H32O3	308.2348	LMFA01070012
metab_2703	7.1416	309.2415	pos	1.6820	4.3845	0.0004	Methyl	AMM1936	NA	NA	Methyl	C19H32O3	308.2348	-

							(10E,12Z,15Z)-9-hydroxyoctadeca-10,12,15-trienoate				(10E,12Z,15Z)-9-hydroxyoctadeca-10,12,15-trienoate			
metab_11762	8.6874	309.2440	neg	1.3069	-2.2201	0.0011	Methoprene	NA	NA	NA	Methoprene	C19H34O3	310.2511	-
metab_7	1.2585	310.1276	pos	1.3726	-2.2355	0.0136	N-(1-Deoxy-1-fructosyl)phenylalanine	NA	NA	NA	N-(1-Deoxy-1-fructosyl)phenylalanine	C15H21NO7	327.1318	HMDB0037846
metab_1236	0.5140	310.1866	pos	1.5749	4.3059	0.0006	Isopropalin	AMM1954	C19063	NA	Isopropalin	C15H23N3O4	309.1688	-
metab_13605	2.6046	311.1406	neg	1.0006	-1.2011	0.0168	Phenylalanylphenylalanine	NA	NA	NA	Phenylalanylphenylalanine	C18H20N2O3	312.1474	HMDB0013302
metab_877	10.1647	311.2571	pos	1.7873	1.8952	0.0392	Methyl 13-hydroxyoctadeca-9,11-dienoate	NA	NA	NA	Methyl 13-hydroxyoctadeca-9,11-dienoate	C19H34O3	310.2508	-
metab_8768	2.4175	313.0721	neg	2.0325	-4.8502	0.0000	3,7-Dihydroxy-3',4'-dimethoxyflavone	AMM2046	NA	Polyketides	3,7-Dihydroxy-3',4'-dimethoxyflavone	C17H14O6	314.0794	-
metab_1628	1.5565	313.1747	pos	1.6855	3.6908	0.0005	TRIPTOPHENOLIDE	AMM2009	NA	NA	TRIPTOPHENOLIDE	C20H24O3	312.1762	-
metab_982	7.5005	313.2727	pos	1.1813	-1.4327	0.0000	(2E)-19-Hydroxynonadec-2-enoic acid	NA	NA	NA	(2E)-19-Hydroxynonadec-2-enoic acid	C19H36O3	312.2660	HMDB0011564; LMGL01010009
metab_4133	8.0843	313.2727	pos	1.0058	-1.1339	0.0005	(2E)-19-Hydroxynonadec-2-enoic acid	NA	NA	NA	(2E)-19-Hydroxynonadec-2-enoic acid	C19H36O3	312.2660	-
metab_4803	4.6096	314.1378	pos	1.8208	-3.5067	0.0001	2,5-Dihydroxy-4-(2-hydroxyphenyl)-5-(methylamino)-3-phenylpentanoic acid	NA	NA	NA	2,5-Dihydroxy-4-(2-hydroxyphenyl)-5-(methylamino)-3-phenylpentanoic acid	C18H21NO5	331.1420	HMDB0134817
metab_2099	3.2274	314.2318	pos	1.6076	5.9401	0.0031	Armepavine	AMM2035	C09342||C09565||C07537||C11780	Alkaloids	Armepavine	C19H23NO3	313.1675	-

									‖C09423‖C15078					
metab_4626	5.5112	314.2680	pos	1.1533	2.3022	0.0282	(+)-Prosopinine	NA	NA	NA	(+)-Prosopinine	C18H35NO3	313.2613	-
metab_8603	2.0202	315.0728	neg	1.3333	10.8548	0.0226	Isorhamnetin	AMM2073	NA	Flavonoids	Isorhamnetin	C16H12O7	316.058	HMDB0002655
metab_8603	2.0202	315.0728	neg	1.3333	10.8548	0.0226	5-Hydroxy-2-[3,4,5-trihydroxy-6-(hydroxymethyl)oxan-2-yl]oxybenzoate	NA	NA	NA	5-Hydroxy-2-[3,4,5-trihydroxy-6-(hydroxymethyl)oxan-2-yl]oxybenzoate	C13H16O9	316.0799	-
metab_13799	2.2119	315.1089	neg	1.7085	3.2245	0.0027	dopaol beta-D-glucoside	AMM2075	NA	NA	2-(3,4-Dihydroxyphenyl)-ethyl-O-beta-D-glucopyranoside	C14H20O8	316.1163	-
metab_8117	1.1796	315.1679	neg	1.0342	4.9418	0.0462	geranyl beta-D-glucoside	AMM2078	NA	NA	geranyl beta-D-glucoside	C16H28O6	316.1887	-
metab_13182	3.6479	315.1821	neg	1.9080	4.3175	0.0002	geranyl beta-D-glucoside	AMM2078	NA	NA	Geranyl beta-D-glucopyranoside	C16H28O6	316.1887	HMDB0060004
metab_13182	3.6479	315.1821	neg	1.9080	4.3175	0.0002	Menthol-glucoronide	NA	NA	NA	Menthol-glucoronide	C16H28O6	316.1886	HMDB0060004
metab_2653	6.9035	315.2311	pos	1.3246	1.5910	0.0005	Progesterone	NA	NA	NA	Progesterone	C21 H30 O2	314.2243	-
metab_13135	3.7658	316.1776	neg	1.7502	3.7521	0.0049	Ile Ser Val	AMM2100	NA	NA	Ile Ser Val	C14H27N3O5	317.1948	-
metab_8646	2.1311	316.1880	neg	1.5377	5.1678	0.0025	Ile Ser Val	AMM2100	NA	NA	Ile-Ser-Val	C14H27N3O5	317.1948	-
metab_8646	2.1311	316.1880	neg	1.5377	5.1678	0.0025	Leu-Val-Ser	AMM2101	NA	NA	Leu-Val-Ser	C14H27N3O5	317.1949	-

metab_2306	4.3371	316.2111	pos	1.3295	3.4731	0.0001	H-LEU-LEU-ALA-OH	AMM2060	NA	NA	H-LEU-LEU-ALA-OH	C15H29N3O4	315.2158	-
metab_5338	2.5782	316.2220	pos	1.6442	2.6382	0.0028	Ile Ala Ile	AMM2058	NA	NA	Ile-Ala-Ile	C15H29N3O4	315.2157	-
metab_5338	2.5782	316.2220	pos	1.6442	2.6382	0.0028	Ala Leu Leu	AMM2059	NA	NA	Ala-Leu-Leu	C15H29N3O4	315.2157	-
metab_5338	2.5782	316.2220	pos	1.6442	2.6382	0.0028	H-LEU-LEU-ALA-OH	AMM2060	NA	NA	Leu-Leu-Ala	C15H29N3O4	315.2158	-
metab_1755	1.9758	316.2221	pos	3.3920	12.7463	0.0000	Ile Ala Ile	AMM2058	NA	NA	Ile-Ala-Ile	C15H29N3O4	315.2157	-
metab_1755	1.9758	316.2221	pos	3.3920	12.7463	0.0000	Ala Leu Leu	AMM2059	NA	NA	Ala Leu Leu	C15H29N3O4	315.2157	-
metab_1755	1.9758	316.2221	pos	3.3920	12.7463	0.0000	H-LEU-LEU-ALA-OH	AMM2060	NA	NA	Leu-Leu-Ala	C15H29N3O4	315.2158	-
metab_8084	1.0785	318.1197	neg	3.2669	11.2714	0.0000	H-TRP-ASP-OH	AMM2122	NA	NA	H-TRP-ASP-OH	C15H17N3O5	319.1169	-
metab_5534	2.1444	318.2019	pos	2.3790	6.0432	0.0013	Ile Ser Val	AMM2100	NA	NA	Ile-Ser-Val	C14H27N3O5	317.1948	-
metab_5534	2.1444	318.2019	pos	2.3790	6.0432	0.0013	Leu-Val-Ser	AMM2101	NA	NA	Leu-Val-Ser	C14H27N3O5	317.1949	-
metab_1733	1.9169	318.2268	pos	1.3613	12.2116	0.0014	ISOSTEVIOL	AMM2080	NA	NA	ISOSTEVIOL	C20H30O3	316.2012	-
metab_14495	1.2521	319.0582	neg	1.5558	11.3199	0.0012	Coptisine	AMM2133	C16938	NA	Coptisine	C19H14NO4	320.0919	-
metab_1350	0.7661	320.0917	pos	1.1017	-2.3445	0.0018	Decarine	NA	NA	NA	Decarine	C19H13NO4	319.0842	-
metab_8506	1.8205	321.0623	neg	1.7672	3.9905	0.0151	2-[[2-(3-Hydroxy-2-Oxo-	AMM2164	NA	NA	2-[[2-(3-Hydroxy-2-O	C14H14N2	322.0803	-

							1H-Indol-3-Yl)Acetyl]Amino]Butanedioate				xo-1H-Indol-3-Yl)Acetyl]Amino]Butanedioate	O7		
metab_1115	0.8640	322.1055	pos	1.2343	1.8900	0.0029	S-Methylglutathione	NA	NA	NA	S-Methylglutathione	C11H19N3O6S	321.0998	-
metab_1521	1.2446	322.1864	pos	1.8362	2.8738	0.0443	H-Phe-Arg-OH	AMM2154	NA	NA	Phe-arg	C15H23N5O3	321.1800	-
metab_9526	5.2100	322.2028	neg	1.3377	2.4738	0.0005	(-)-Jasmonoyl-L-isoleucine	NA	NA	NA	(-)-Jasmonoyl-L-isoleucine	C18H29NO4	323.2098	-
metab_6955	4.3882	323.1868	neg	1.4740	3.6014	0.0086	Dinor-PGE2	AMM2209	NA	NA	Dinor-PGE2	C18H28O5	324.1936	-
metab_9629	5.7751	323.2232	neg	1.1106	2.7910	0.0457	Dihydromonacolin L acid	NA	NA	NA	Dihydromonacolin L acid	C19H32O4	324.2298	-
metab_5976	1.3013	324.1180	pos	1.2710	4.2680	0.0154	Rutacridone epoxide	AMM2184	C10739||C05175	Alkaloids	Rutacridone epoxide	C19H17NO4	323.1155	HMDB0033853
metab_4848	4.3818	324.2524	pos	1.0916	11.4636	0.0039	Linoleamide MEA	AMM2190	NA	NA	Linoleamide MEA	C20H37NO2	323.2822	HMDB0012252
metab_105	8.0267	324.2887	pos	1.0967	-1.4300	0.0103	Linoleoyl ethanolamide	NA	NA	NA	Linoleoyl ethanolamide	C20H37NO2	323.2822	-
metab_4929	4.0338	325.1176	pos	1.6264	12.5126	0.0178	Tetradecyl iodide	AMM2203	NA	NA	Tetradecyl iodide	C14H29I	324.1319	-
metab_1093	1.2016	325.1384	pos	1.0190	1.0946	0.0004	1-Iodotetradecane	NA	NA	NA	1-Iodotetradecane	C14H29I	324.1319	-
metab_1554	1.3720	325.1386	pos	1.0647	1.1373	0.0023	1-Iodotetradecane	NA	NA	NA	1-Iodotetradecane	C14H29I	324.1319	-
metab_8680	2.2119	325.1772	neg	1.1663	5.1808	0.0293	CHEBI:59965	AMM2233	NA	NA	CHEBI:59965	C15H26N4O4	326.1941	-
metab_5099	3.3337	325.2000	pos	1.9775	3.9873	0.0001	Dinor-PGE2	AMM2209	NA	NA	Dinor-PGE2	C18H28O5	324.1936	-
metab_4948	3.9421	325.2000	pos	1.7546	2.9766	0.0002	Dinor-PGE2	AMM2209	NA	NA	Dinor-PGE2	C18H28O5	324.1936	-
metab_9990	7.5702	325.2386	neg	1.0247	-1.8261	0.0148	6Z,9Z-Octadecadienoic	NA	NA	NA	6Z,9Z-Octadecadienoi	C18H32O2	280.2402	HMDB0062238;

							acid				c acid			LMFA01030332
metab_1656	1.6413	327.2001	pos	1.1380	2.2670	0.0245	Glyoxal-lysine dimer	NA	NA	NA	Glyoxal-lysine dimer	C15H26N4 O4	326.1941	-
metab_9662	5.9866	327.2544	neg	1.2084	-1.5843	0.0155	2-[(9Z)-Hexadecenoyl]gl ycerol	NA	NA	NA	2-[(9Z)-Hexadecenoyl]glycerol	C19H36O4	328.2616	-
metab_13304	3.2972	328.1195	neg	1.5712	-2.2629	0.0000	p-Coumaroyltyramine	NA	NA	NA	p-Coumaroyltyramine	C17H17N O3	283.1208	HMDB0039521
metab_2186	3.6229	328.2472	pos	1.5049	3.3989	0.0001	10-nitro-9E-octadecenoic acid	AMM2258	NA	NA	10-nitro-9E-octadecen oic acid	C18H33N O4	327.2407	HMDB0062737
metab_4946	3.9421	328.2475	pos	1.7827	3.8585	0.0000	10-nitro-9E-octadecenoic acid	AMM2259	NA	NA	10-Nitro-9E-octadecen oic acid	C18H33N O4	327.2407	-
metab_4882	4.2457	329.2311	pos	1.0780	1.3975	0.0483	2,3-Dinor-8-epi-prostagl andin F1alpha	NA	NA	NA	2,3-Dinor-8-epi-prosta glandin F1alpha	C18H32O5	328.2246	-
metab_13584	2.6357	330.2039	neg	1.5399	3.4240	0.0003	Ser Ile Leu	AMM2329	NA	NA	Ser-Ile-Leu	C15H29N3 O5	331.2106	-
metab_2086	3.1957	330.2375	pos	2.7656	8.1940	0.0000	Val Val Leu	AMM2292	NA	NA	Val Val Leu	C16H31N3 O4	329.2314	-
metab_9408	4.6056	331.1556	neg	3.0924	15.5315	0.0021	(2S,3S)-2-Hydroxytridec ane-1,2,3-tricarboxylate	AMM2350	C04655	NA	(2S,3S)-2-Hydroxytrid ecane-1,2,3-tricarboxy late	C16H28O7	332.1849	-
metab_4431	6.5857	331.1871	pos	1.3307	1.9108	0.0018	Carnosic acid	NA	NA	NA	Carnosic acid	C20H28O4	330.1804	-
metab_1632	1.5705	331.1965	pos	1.7296	5.2095	0.0003	(2S,3S)-2-Hydroxytridec ane-1,2,3-tricarboxylate	AMM2350	C04655	NA	(2S,3S)-2-Hydroxytrid ecane-1,2,3-tricarboxy late	C16H28O7	332.1849	-
metab_2514	5.7385	331.2470	pos	1.3440	1.9749	0.0189	5,8,12-Trihydroxy-9-octa decenoic acid	NA	NA	NA	5,8,12-Trihydroxy-9-o ctadecenoic acid	C18H34O5	330.2406	HMDB0030936; LMFA01050543;

														LMFA02000221
metab_5697	1.7993	333.1545	pos	1.4660	2.6499	0.0278	Trp-Ala-Gly	AMM2346	NA	NA	Trp-Ala-Gly	C16H20N4O4	332.1483	-
metab_13094	3.8839	333.1826	neg	1.3134	5.3518	0.0195	(3R)-10-[(2R,3R,5R,6S)-3,5-dihydroxy-6-methyloxan-2-yl]oxy-3-hydroxydecanoic acid	AMM2391	NA	NA	(3R)-10-[(2R,3R,5R,6S)-3,5-dihydroxy-6-methyloxan-2-yl]oxy-3-hydroxydecanoic acid	C16H30O7	334.1994	-
metab_2268	4.0338	333.2415	pos	1.5485	6.1935	0.0049	21-Hydroxypregnenolone	AMM2354	C05485||C15405||C05138||C04518||C06390||C15011||C15401||C15022||C18040||C18038||C07393||C15384||C13803||C15355	NA	21-Hydroxypregnenolone	C21H32O3	332.2349	HMDB0004026
metab_5583	2.0524	334.2212	pos	1.5478	6.7422	0.0026	CARBETAPENTANE	AMM2367	NA	NA	CARBETAPENTANE	C20H31NO3	333.2276	-
metab_4304	7.1701	334.2368	pos	1.1367	2.8873	0.0119	Carbetapentane	AMM2367	NA	NA	Carbetapentane	C20H31NO3	333.2276	-
metab_4805	4.5944	335.1514	pos	1.3833	2.0599	0.0256	pimobendan	AMM2389	NA	NA	Pimobendan	C19H18N4O2	334.1440	-
metab_12777	5.0484	335.1539	neg	1.3230	3.2504	0.0162	Smardaesidin G	AMM2443	NA	Terpenoids_pubchem	Smardaesidin G	C19H28O5	336.1933	-
metab_4767	4.7758	335.2207	pos	2.1854	5.8178	0.0000	JWH 015-d7	AMM2392	NA	NA	JWH 015-d7	C23H14D7NO	334.2117	-
metab_809	8.9367	335.2570	pos	1.4232	2.8851	0.0125	3alpha,6alpha-Dihydroxy	NA	NA	NA	3alpha,6alpha-Dihydro	C21H34O3	334.2506	-

							-5beta-pregnan-20-one				xy-5beta-pregnan-20-one			
metab_809	8.9367	335.2570	pos	1.4232	2.8851	0.0125	Tetrahydrodeoxycorticosterone	NA	NA	NA	Tetrahydrodeoxycorticosterone	C21H34O3	334.2506	-
metab_1551	1.3437	336.1541	pos	1.6309	3.2628	0.0001	Isopentenyl adenosine	AMM2421	C16427||C17700	NA	Isopentenyl adenosine	C15H21N5O4	335.1591	-
metab_1982	2.7804	336.1656	pos	1.4964	-4.1958	0.0140	Isopentenyl adenosine	AMM2421	C16427||C17700	NA	Riboprine	C15H21N5O4	335.1591	-
metab_8296	1.4710	336.1668	neg	1.5670	9.3675	0.0089	Kyotorphin	AMM2460	C02993	NA	Kyotorphin	C15H23N5O4	337.1735	HMDB0005768
metab_2225	3.7908	337.2151	pos	1.6843	7.2745	0.0033	Smardaesidin G	AMM2443	NA	Terpenoids_pubchem	Smardaesidin G	C19H28O5	336.1933	-
metab_1908	2.4370	337.2365	pos	1.7229	12.7616	0.0042	Smardaesidin G	AMM2443	NA	Terpenoids_pubchem	Smardaesidin G	C19H28O5	336.1933	-
metab_10274	8.6239	337.2391	neg	1.5510	-2.0851	0.0000	5,6-Dihydroxy-8Z,11Z,14Z-eicosatrienoic acid	NA	NA	NA	5,6-Dihydroxy-8Z,11Z,14Z-eicosatrienoic acid	C20H34O4	338.2461	-
metab_5821	1.5705	339.1180	pos	1.2618	2.5130	0.0080	Demethoxycurcumin	AMM2479	C17742||C11581||C10049||C11576||C10542||C10422||C01701||C15511||C10360||C18783	Flavonoids	Demethoxycurcumin	C20H18O5	338.115	-
metab_5027	3.6073	339.2159	pos	2.5033	10.3168	0.0000	Monacolin J acid	AMM2488	C20854||C18880||C09725	Terpenoids	Monacolin J acid	C19H30O5	338.2091	-
metab_12836	4.8210	339.2180	neg	1.5642	3.9014	0.0030	3alpha-Hydroxy-3,5-dihy	NA	NA	NA	3alpha-Hydroxy-3,5-di	C19H32O5	340.2251	HMDB0036832

							dromonacolin L acid				hydromonacolin L acid			
metab_12836	4.8210	339.2180	neg	1.5642	3.9014	0.0030	Sterebin D	NA	NA	NA	Sterebin D	C18H30O3	294.2195	HMDB0036832
metab_4195	7.7946	339.2883	pos	1.0377	1.8166	0.0297	Glycidyl oleate	NA	NA	NA	Glycidyl oleate	C21H38O3	338.2820	-
metab_4141	8.0700	339.2884	pos	1.1423	1.8712	0.0117	Glycidyl oleate	NA	NA	NA	Glycidyl oleate	C21H38O3	338.2820	HMDB0060039; LMFA01030159
metab_4747	4.8961	340.2837	pos	1.2809	12.3358	0.0043	N-Oleoyl Glycine	AMM2515	NA	NA	N-Oleoyl Glycine	C20H37NO3	339.2746	HMDB0013631
metab_13092	3.9005	341.1609	neg	2.5133	5.5575	0.0000	3,8-Dihydroxy-6-methoxy-7(11)-eremophilen-12,8-olide	NA	NA	NA	3,8-Dihydroxy-6-methoxy-7(11)-eremophilen-12,8-olide	C16H24O5	296.1624	HMDB0041551
metab_13586	2.6357	341.1957	neg	1.7258	3.6219	0.0028	2,3-Dkpgf1alpha	AMM2566	NA	NA	2,3-Dinor-6-oxoprostaglandin F1alpha	C18H30O6	342.2045	-
metab_9250	3.9005	341.1972	neg	1.4642	2.4665	0.0014	2,3-Dkpgf1alpha	AMM2566	NA	NA	2,3-Dinor-6-oxoprostaglandin F1alpha	C18H30O6	342.2045	-
metab_7709	3.3803	341.1973	neg	1.9065	4.1110	0.0004	2,3-Dkpgf1alpha	AMM2566	NA	NA	2,3-Dinor-6-oxoprostaglandin F1alpha	C18H30O6	342.2045	-
metab_5135	3.2116	341.2425	pos	1.5283	9.9028	0.0217	3alpha-Hydroxy-3,5-dihydromonacolin L acid	AMM2526	C20852	NA	3alpha-Hydroxy-3,5-dihydromonacolin L acid	C19H32O5	340.2251	-
metab_4380	6.8886	341.2559	pos	1.4212	3.5213	0.0020	Dimethisterone	AMM2529	C07628||C14883||C15186	NA	Dimethisterone	C23H32O2	340.2403	-
metab_9866	7.0322	341.2697	neg	1.0368	1.8303	0.0202	cis-10-Nonadecenoic acid	NA	NA	NA	cis-10-Nonadecenoic acid	C19H36O2	296.2715	HMDB0013622; LMFA01030362
metab_11897	8.3055	342.2653	neg	1.1737	-1.3072	0.0053	Palmitoleoyl Ethanolamide	NA	NA	NA	Palmitoleoyl Ethanolamide	C18H35NO2	297.2668	HMDB0013648

metab_782	8.4504	342.2994	pos	1.3957	3.1525	0.0104	N-Acetylsphingosine	NA	NA	NA	N-Acetylsphingosine	C20H39NO3	341.2926	-
metab_12550	6.1320	343.2129	neg	1.0011	1.3274	0.0038	11R-(3'R,5'R-Dihydroxy-6'S-methyl-(2H)-tetrahydropyran-2'-yloxy)-2E-dodecenoic acid	NA	NA	NA	11R-(3'R,5'R-Dihydroxy-6'S-methyl-(2H)-tetrahydropyran-2'-yloxy)-2E-dodecenoic acid	C18H32O6	344.2201	-
metab_8839	2.6046	343.2130	neg	2.2555	6.9150	0.0000	(E,11R)-11-[(2R,3R,5R,6S)-3,5-dihydroxy-6-methyloxan-2-yl]oxydodec-2-enoic acid	AMM2605	NA	NA	(E,11R)-11-[(2R,3R,5R,6S)-3,5-dihydroxy-6-methyloxan-2-yl]oxydodec-2-enoic acid	C18H32O6	344.2201	-
metab_12495	6.3759	343.2132	neg	1.3263	4.2658	0.0054	(E,11R)-11-[(2R,3R,5R,6S)-3,5-dihydroxy-6-methyloxan-2-yl]oxydodec-2-enoic acid	AMM2605	NA	NA	(E,11R)-11-[(2R,3R,5R,6S)-3,5-dihydroxy-6-methyloxan-2-yl]oxydodec-2-enoic acid	C18H32O6	344.2201	-
metab_7322	8.7031	343.2495	neg	1.6096	-2.0264	0.0003	Tianshic acid methyl ester	NA	NA	NA	Tianshic acid methyl ester	C19H36O5	344.2564	-
metab_11577	9.1038	343.2858	neg	2.1642	14.5077	0.0001	Methyl stearate	NA	NA	NA	Methyl stearate	C19H38O2	298.2872	HMDB0034154
metab_9495	5.0159	345.1709	neg	2.1126	9.9044	0.0427	Blumealactone A	NA	NA	NA	Blumealactone A	C20H28O6	364.1886	HMDB0036665
metab_5920	1.4005	345.2122	pos	1.4921	13.1182	0.0172	13,14-dehydro-15-cyclohexyl Carbaprostacyclin	AMM2600	NA	NA	13,14-dehydro-15-cyclohexyl Carbaprostacyclin	C21H30O4	344.1961	-
metab_5920	1.4005	345.2122	pos	1.4921	13.1182	0.0172	Asn-Val-Ile	NA	NA	NA	Asn-Val-Ile	C15H28N4O5	344.2058	-
metab_4944	3.9577	347.2209	pos	1.8149	6.8998	0.0042	Cortexolone	AMM2639	NA	NA	Cortexolone	C21H30O4	346.2117	HMDB0000015
metab_2298	4.2750	348.2527	pos	1.3161	12.1076	0.0032	O-Arachidonoyl Ethanolamine	AMM2660	NA	NA	O-Arachidonoyl Ethanolamine	C22H37NO2	347.2797	HMDB0013655

metab_1710	1.7845	349.1748	pos	1.8624	12.1376	0.0159	Schizonepetoside E	AMM2675	C17638	NA	Schizonepetoside E	C16H28O8	348.1785	-
metab_4956	3.9114	349.2001	pos	1.9581	8.7760	0.0087	17alpha,21-Dihydroxypregnenolone	AMM2681	C05487||C05478||C05475||C15685||C14598	NA	17alpha,21-Dihydroxypregnenolone	C21H32O4	348.2296	HMDB0006762
metab_9348	4.3037	349.2025	neg	1.5309	16.0285	0.0173	11beta-Hydroxytestosterone	NA	C18075	NA	11beta-Hydroxytestosterone	C19H28O3	304.2038	HMDB0012533; HMDB0060339
metab_4419	6.6909	349.2358	pos	1.7144	4.8539	0.0002	17alpha,21-Dihydroxypregnenolone	AMM2682	C05487||C05478||C05475||C14598||C15685	NA	17alpha,21-Dihydroxypregnenolone	C21H32O4	348.2298	-
metab_4419	6.6909	349.2358	pos	1.7144	4.8539	0.0002	5-trans U-44069	AMM2678	NA	NA	5-trans U-44069	C21H34O4	348.2274	-
metab_2204	3.6987	349.2364	pos	1.6788	4.3994	0.0053	17alpha,21-Dihydroxypregnenolone	AMM2682	C05487||C05478||C05475||C14598||C15685	NA	17alpha,21-Dihydroxypregnenolone	C21H32O4	348.2298	-
metab_2204	3.6987	349.2364	pos	1.6788	4.3994	0.0053	5-trans U-44069	AMM2678	NA	NA	5-trans U-44069	C21H34O4	348.2274	-
metab_5109	3.3032	349.2365	pos	1.8094	4.5041	0.0019	17alpha,21-Dihydroxypregnenolone	AMM2682	C05487||C05478||C05475||C14598||C15685	NA	17alpha,21-Dihydroxypregnenolone	C21H32O4	348.2298	-
metab_5109	3.3032	349.2365	pos	1.8094	4.5041	0.0019	5-trans U-44069	AMM2678	NA	NA	5-trans U-44069	C21H34O4	348.2274	-
metab_492	2.5158	351.1461	pos	1.1299	1.8062	0.0113	Fagaronine	NA	NA	NA	Fagaronine	C21H19NO4	350.1390	-
metab_13681	2.4487	351.1663	neg	1.0686	1.9802	0.0088	1-[(2R,3R,4S,5S,6R)-3,4,5-Trihydroxy-6-(hydroxymethyl)oxan-2-yl]oxyoctan-3-one	NA	NA	NA	1-[(2R,3R,4S,5S,6R)-3,4,5-Trihydroxy-6-(hydroxymethyl)oxan-2-yl]oxyoctan-3-one	C14H26O7	306.1679	HMDB0031315
metab_586	4.2907	351.2155	pos	1.4047	3.3893	0.0082	5S-Hydroperoxy-18R-hydroxy-6E,8Z,11Z,14Z,16	NA	NA	NA	5S-Hydroperoxy-18R-hydroxy-6E,8Z,11Z,14	C20H30O5	350.2091	HMDB0012503; LMFA03020044

							E-eicosapentaenoic acid				Z,16E-eicosapentaenoic acid			
metab_586	4.2907	351.2155	pos	1.4047	3.3893	0.0082	Tebufenozide	NA	NA	NA	Tebufenozide	C22H28N2O2	350.2068	HMDB0012503; LMFA03020044
metab_4392	6.8582	351.2157	pos	1.7426	12.4873	0.0000	Tebufenozide	NA	NA	NA	Tebufenozide	C22H28N2O2	350.2068	-
metab_4392	6.8582	351.2157	pos	1.7426	12.4873	0.0000	5S-Hydroperoxy-18R-HEPE	AMM2729	C18176||C13802||C06439||C18848||C04707||C04758||C04835||C20214||C18171	Terpenoids	5S-Hydroperoxy-18R-HEPE	C20H30O5	350.2091	-
metab_2108	3.2573	351.2159	pos	1.8131	3.3212	0.0002	Tebufenozide	AMM2726	NA	NA	Tebufenozide	C22H28N2O2	350.2068	-
metab_2108	3.2573	351.2159	pos	1.8131	3.3212	0.0002	5S-Hydroperoxy-18R-HEPE	AMM2729	C18176||C13802||C06439||C18848||C04707||C04758||C04835||C20214||C18171	Terpenoids	5S-Hydroperoxy-18R-hydroxy-6E,8Z,11Z,14Z,16E-eicosapentaenoic acid	C20H30O5	350.2091	-
metab_599	4.5184	351.2521	pos	1.1268	-2.6442	0.0180	Tetrahydrocorticosterone	NA	NA	NA	Tetrahydrocorticosterone	C21H34O4	350.2455	-
metab_599	4.5184	351.2521	pos	1.1268	-2.6442	0.0180	Tetrahydrodeoxycortisol	NA	NA	NA	Tetrahydrodeoxycortisol	C21H34O4	350.2448	-
metab_6048	1.1875	352.1490	pos	1.5099	4.5183	0.0020	Palmatine	AMM2746	NA	Alkaloids	Palmatine	C21H21NO4	351.1466	-
metab_1643	1.6272	352.1599	pos	1.0629	-2.3843	0.0396	Zeatin riboside	NA	NA	NA	Zeatin riboside	C15H21N5O5	351.1543	HMDB0030388

metab_11782	8.6406	352.2862	neg	1.2765	-1.1006	0.0018	Tetradecanoylcarnitine	NA	NA	NA	Tetradecanoylcarnitine	C21H41NO4	371.3036	LMFA07070107; HMDB0005066
metab_9939	7.3343	353.2166	neg	1.5274	11.3223	0.0003	3,3-Difluoro-5alpha-androstan-17beta-yl acetate	AMM2843	C14477	NA	3,3-Difluoro-5alpha-androstan-17beta-yl acetate	C21H32F2O2	354.2384	-
metab_84	6.6001	353.2677	pos	1.2269	-1.9577	0.0032	Montanol	NA	NA	NA	Montanol	C21H36O4	352.2612	-
metab_3722	9.8362	353.2677	pos	1.1474	-1.6857	0.0050	Montanol	NA	NA	NA	Montanol	C21H36O4	352.2612	-
metab_4768	4.7758	353.2787	pos	2.0372	9.4167	0.0150	Montanol	AMM2791	C09137	Terpenoids	Montanol	C21H36O4	352.261	-
metab_1505	1.2162	354.1472	pos	1.7464	3.7197	0.0001	Methionyl-Tyrosine	NA	NA	NA	Methionyl-Tyrosine	C14H20N2O4S	312.1144	HMDB0028985
metab_5143	3.1957	354.2630	pos	1.6157	-1.5110	0.0080	Methyl tumonoate A	AMM2825	NA	NA	Methyl tumonoate A	C20H35NO4	353.2563	-
metab_12762	5.1296	355.2130	neg	1.9136	5.9357	0.0006	Estradiol valerate	AMM2886	C12859||C14503	NA	Estradiol valerate	C23H32O3	356.2354	-
metab_9158	3.5978	355.2132	neg	1.9316	4.8062	0.0004	(E)-4-[(1R,2S,3S,4R,8As)-2,3,4-trihydroxy-2,5,5,8a-tetramethyl-3,4,4a,6,7,8-hexahydro-1H-naphthalen-1-yl]but-3-en-2-one	NA	NA	NA	(E)-4-[(1R,2S,3S,4R,8As)-2,3,4-trihydroxy-2,5,5,8a-tetramethyl-3,4,4a,6,7,8-hexahydro-1H-naphthalen-1-yl]but-3-en-2-one	C18H30O4	310.2144	HMDB0035337
metab_2573	6.1927	355.2468	pos	1.6445	2.5868	0.0004	Prostaglandin D1	AMM2844	C06438||C04741||C13809||C14782||C14814||C14811||C14809	NA	Prostaglandin D1	C20H34O5	354.2403	-
metab_2599	6.4794	355.2468	pos	1.4679	2.3787	0.0003	Prostaglandin D1	NA	NA	NA	Prostaglandin D1	C20H34O5	354.2403	-
metab_978	7.6185	355.2832	pos	1.4233	-1.9170	0.0092	Methyl O-acetylricinoleate	NA	NA	NA	Methyl O-acetylricinoleate	C21H38O4	354.2767	-

metab_5052	3.5162	357.2008	pos	2.3039	3.9228	0.0108	CHEBI:73270	AMM2881	NA	NA	CHEBI:73270	C16H28N4O5	356.2059	-
metab_2534	5.9049	358.2577	pos	1.1442	4.4619	0.0194	(9Z)-3-hydroxydodecenoylcarnitine	AMM2905	NA	NA	(9Z)-3-hydroxydodecenoylcarnitine	C19H35NO5	357.2512	-
metab_3028	9.2658	358.3671	pos	1.4632	2.7014	0.0003	1-Decanol	NA	NA	NA	1-Decanol	C10H22O	158.1671	HMDB0011624
metab_4576	5.7846	359.3259	pos	1.2655	4.4325	0.0332	CHEBI:79294	AMM2928	NA	NA	CHEBI:79294	C21H42O4	358.3081	-
metab_4774	4.7302	360.1796	pos	1.7759	-3.3922	0.0349	Ovalicin	NA	C09674	NA	Ovalicin	C16H24O5	296.1624	HMDB0038120
metab_5383	2.4530	360.2735	pos	1.8500	12.8613	0.0015	3-hydroxylauroylcarnitine	AMM2942	NA	NA	3-hydroxylauroylcarnitine	C19H37NO5	359.267	-
metab_13760	2.2912	361.0936	neg	1.1803	2.0019	0.0109	6-Hydroxy-2-(4-hydroxyphenyl)-7,8-dimethoxy-3,4-dihydro-2H-1-benzopyran-4-one	NA	NA	NA	6-Hydroxy-2-(4-hydroxyphenyl)-7,8-dimethoxy-3,4-dihydro-2H-1-benzopyran-4-one	C17H16O6	316.0947	HMDB0133294
metab_5860	1.5144	361.1704	pos	1.6229	13.1792	0.0010	L-Tryptophan-L-arginine	AMM2953	NA	NA	L-Tryptophan-L-arginine	C17H24N6O3	360.1898	-
metab_13244	3.4800	361.2028	neg	1.4520	13.9084	0.0062	Hydrocortisone	AMM2994	NA	NA	Hydrocortisone	C21H30O5	362.2062	HMDB0000063
metab_9387	4.5219	361.2237	neg	1.2315	1.8019	0.0128	(3R)-12-[(3,6-Dideoxy-alpha-L-arabino-hexopyranosyl)oxy]-3-hydroxylauric acid	NA	NA	NA	(3R)-12-[(3,6-Dideoxy-alpha-L-arabino-hexopyranosyl)oxy]-3-hydroxylauric acid	C18H34O7	362.2307	-
metab_12795	4.9510	361.2238	neg	2.1813	5.1487	0.0000	(3R)-12-[(2R,3R,5R,6S)-3,5-dihydroxy-6-methyloxan-2-yl]oxy-3-hydroxydodecanoic acid	AMM2997	NA	NA	(3R)-12-[(2R,3R,5R,6S)-3,5-dihydroxy-6-methyloxan-2-yl]oxy-3-hydroxydodecanoic acid	C18H34O7	362.2307	-
metab_9058	3.2301	361.2238	neg	1.0858	1.5559	0.0074	(3R)-12-[(3,6-Dideoxy-al	NA	NA	NA	(3R)-12-[(3,6-Dideoxy	C18H34O7	362.2307	-

							pha-L-arabino-hexopyranosyl)oxy]-3-hydroxylauric acid				-alpha-L-arabino-hexopyranosyl)oxy]-3-hydroxylauric acid			
metab_5946	1.3437	362.1101	pos	2.5210	12.5910	0.0000	N(6)-methyl-AMP	AMM2959	NA	NA	N(6)-methyl-AMP	C11H16N5O7P	361.0786	-
metab_2075	3.1648	363.2159	pos	2.4674	7.6095	0.0001	Hydrocortisone	AMM2995	NA	NA	Hydrocortisone	C21H30O5	362.2066	-
metab_2241	3.8967	363.2160	pos	1.7709	14.1624	0.0105	Hydrocortisone	AMM2994	NA	NA	Hydrocortisone	C21H30O5	362.2062	HMDB0000063
metab_7426	4.1869	363.2184	neg	1.6389	4.3671	0.0020	Tetrahydrocortisone	NA	C05470	NA	Tetrahydrocortisone	C21H32O5	364.2250	HMDB0000903
metab_4899	4.1544	364.2684	pos	2.4427	12.8073	0.0058	N-cis-octadec-9Z-enoyl-L-Homoserine lactone	AMM3011	NA	NA	N-cis-octadec-9Z-enoyl-L-Homoserine lactone	C22H39NO3	363.2747	-
metab_7351	8.0688	364.2863	neg	1.0044	2.2425	0.0209	(Z,Z)-4-((1-Oxo-9,12-octadecadienyl)amino)butanoic acid	NA	NA	NA	(Z,Z)-4-((1-Oxo-9,12-octadecadienyl)amino)butanoic acid	C22H39NO3	365.2930	HMDB0062334
metab_6909	1.4710	365.1360	neg	1.1921	-1.5105	0.0398	(2R,3R,4S,5R,6S)-2-(Hydroxymethyl)-6-[4-(1H-imidazol-2-ylmethyl)-2-methoxyphenoxy]oxane-3,4,5-triol	NA	NA	NA	(2R,3R,4S,5R,6S)-2-(Hydroxymethyl)-6-[4-(1H-imidazol-2-ylmethyl)-2-methoxyphenoxy]oxane-3,4,5-triol	C17H22N2O7	366.1427	HMDB0033108
metab_4902	4.1387	365.2315	pos	1.6795	4.4556	0.0029	11beta,17alpha,21-Trihydroxypregnenolone	AMM3024	C05489||C05471||C05470||C05474	NA	11beta,17alpha,21-Trihydroxypregnenolone	C21H32O5	364.2245	-
metab_2090	3.2116	365.2315	pos	1.7780	4.1173	0.0072	11beta,17alpha,21-Trihydroxypregnenolone	AMM3024	C05489||C05471||C05470||C05474	NA	11beta,17alpha,21-Trihydroxypregnenolone	C21H32O5	364.2245	-
metab_2243	3.9114	365.2315	pos	1.5888	3.0108	0.0014	11beta,17alpha,21-Trihydroxypregnenolone	AMM3024	C05489||C05471||C05470||C05474	NA	11beta,17alpha,21-Trihydroxypregnenolone	C21H32O5	364.2245	-
metab_7026	6.9525	365.2336	neg	1.0965	1.3822	0.0313	Cortolone	NA	NA	NA	Cortolone	C21H34O5	366.2405	-

metab_7026	6.9525	365.2336	neg	1.0965	1.3822	0.0313	Tetrahydrocortisol	NA	NA	NA	Tetrahydrocortisol	C21H34O5	366.2405	-
metab_9216	3.8001	365.2338	neg	1.7282	4.7472	0.0015	Urocortisol	AMM3063	C05472||C05481	NA	Tetrahydrocortisol	C21H34O5	366.2405	HMDB0036705
metab_9216	3.8001	365.2338	neg	1.7282	4.7472	0.0015	Cortolone	AMM3064	C05481||C05472	NA	Cortolone	C21H34O5	366.2405	HMDB0036705
metab_9216	3.8001	365.2338	neg	1.7282	4.7472	0.0015	Ucriol	NA	NA	NA	Ucriol	C20H32O3	320.2351	HMDB0036705
metab_7423	4.2203	365.2339	neg	1.3745	3.3890	0.0282	Cortolone	NA	NA	NA	Cortolone	C21H34O5	366.2405	-
metab_7423	4.2203	365.2339	neg	1.3745	3.3890	0.0282	Tetrahydrocortisol	NA	NA	NA	Tetrahydrocortisol	C21H34O5	366.2405	-
metab_9759	6.5190	366.2653	neg	1.4139	-1.5086	0.0347	alpha-Linolenoyl ethanolamide	NA	NA	NA	alpha-Linolenoyl ethanolamide	C20H35NO2	321.2668	HMDB0013624
metab_7330	8.5455	366.3018	neg	1.0460	2.6431	0.0233	N-Oleoyl-4-aminobutyric acid	NA	NA	NA	N-Oleoyl-4-aminobutyric acid	C22H41NO3	367.3086	HMDB0062335; LMFA08020104
metab_13199	3.5978	367.2131	neg	3.4442	15.8550	0.0000	Prostaglandin G2	AMM3097	C05956||C12202||C05964||C05962||C17653||C17655	NA	Prostaglandin G2	C20H32O6	368.2196	HMDB0003235
metab_13199	3.5978	367.2131	neg	3.4442	15.8550	0.0000	20-Hydroxy-PGE2	NA	NA	NA	20-Hydroxy-PGE2	C20H32O6	368.2199	HMDB0003247; LMFA03010014
metab_9037	3.1626	367.2131	neg	2.4378	10.3251	0.0001	Prostaglandin G2	AMM3097	C05956||C12202||C05964||C05962||C17653||C17655	NA	Prostaglandin G2	C20H32O6	368.2196	HMDB0003235
metab_9536	5.2579	367.2254	neg	1.7808	6.1593	0.0044	Cortol	AMM3101	C05482||C06872	NA	Cortol	C21H36O5	368.2558	HMDB0003180
metab_4658	5.3165	367.2470	pos	1.0084	-1.4054	0.0178	JWH 018 4-hydroxyindole metabolite-d9	NA	NA	NA	JWH 018 4-hydroxyindole metabolite-d9	C24H14D9NO2	366.2382	-
metab_4658	5.3165	367.2470	pos	1.0084	-1.4054	0.0178	Cortolone	NA	NA	NA	Cortolone	C21H34O5	366.2405	-
metab_4658	5.3165	367.2470	pos	1.0084	-1.4054	0.0178	Tetrahydrocortisol	NA	NA	NA	Tetrahydrocortisol	C21H34O5	366.2405	-

metab_2048	3.0114	367.2471	pos	1.7431	2.9342	0.0061	Urocortisol	AMM3063	C05472||C05481	NA	Tetrahydrocortisol	C21H34O5	366.2405	-
metab_2048	3.0114	367.2471	pos	1.7431	2.9342	0.0061	Cortolone	AMM3060	C05481||C05472	NA	Cortolone	C21H34O5	366.2405	-
metab_2048	3.0114	367.2471	pos	1.7431	2.9342	0.0061	JWH 018 4-hydroxyindole metabolite-d9	AMM3054	NA	NA	JWH 018 4-hydroxyindole metabolite-d9	C24H14D9NO2	366.2382	-
metab_9300	4.1023	367.2494	neg	1.9579	5.7048	0.0016	Cortol	NA	C05482	NA	Cortol	C21H36O5	368.2563	HMDB0003180
metab_9300	4.1023	367.2494	neg	1.9579	5.7048	0.0016	Carboprost	AMM3108	C06872||C05482	NA	Carboprost	C21H36O5	368.2565	HMDB0003180
metab_9236	3.8506	367.2494	neg	1.6479	8.7602	0.0190	Carboprost	AMM3108	C06872||C05482	NA	Carboprost	C21H36O5	368.2565	-
metab_10186	8.3055	367.2495	neg	1.8755	-3.2561	0.0001	Carboprost	AMM3108	C06872||C05482	NA	Carboprost	C21H36O5	368.2565	-
metab_4985	3.7599	367.2682	pos	1.3969	13.5103	0.0411	Cortolone	AMM3055	C05481||C05472	NA	Cortolone	C21H34O5	366.2401	HMDB0003128
metab_10252	8.5455	368.3083	neg	1.0000	4.7154	0.0274	Aplidiasphingosine	AMM3121	NA	NA	Aplidiasphingosine	C22H43NO3	369.3241	-
metab_13121	3.8001	369.1924	neg	3.1088	13.2792	0.0000	CHEBI:66791	AMM3122	NA	NA	CHEBI:66791	C20H26N4O3	370.1989	-
metab_7701	3.2972	369.2287	neg	1.6391	3.3589	0.0001	6-Keto-prostaglandin F1alpha	AMM3124	C05961||C05963	NA	6-Keto-prostaglandin F1alpha	C20H34O6	370.2356	-
metab_7701	3.2972	369.2287	neg	1.6391	3.3589	0.0001	Thromboxane B2	AMM3125	C05963||C05961	NA	Thromboxane B2	C20H34O6	370.2357	-
metab_12721	5.3546	369.2288	neg	1.2885	2.1531	0.0000	6-Keto-prostaglandin F1alpha	NA	NA	NA	6-Keto-prostaglandin F1alpha	C20H34O6	370.2356	-
metab_12721	5.3546	369.2288	neg	1.2885	2.1531	0.0000	Thromboxane B2	NA	NA	NA	Thromboxane B2	C20H34O6	370.2357	-
metab_12927	4.5054	369.2288	neg	1.4846	3.0546	0.0009	6-Keto-prostaglandin F1alpha	NA	NA	NA	6-Keto-prostaglandin F1alpha	C20H34O6	370.2356	-
metab_12927	4.5054	369.2288	neg	1.4846	3.0546	0.0009	Thromboxane B2	NA	NA	NA	Thromboxane B2	C20H34O6	370.2357	-
metab_9286	4.0516	369.2392	neg	2.4487	6.0783	0.0001	Thromboxane B2	AMM3125	C05963||C05961	NA	Thromboxane B2	C20H34O6	370.2357	HMDB0003252
metab_2615	6.6606	369.2626	pos	1.0970	-1.6609	0.0016	Cortol	NA	NA	NA	Cortol	C21H36O5	368.2558	-

metab_2615	6.6606	369.2626	pos	1.0970	-1.6609	0.0016	Carboprost	NA	NA	NA	Carboprost	C21H36O5	368.2560	-
metab_13071	3.9677	369.2650	neg	1.6411	11.9606	0.0031	1-Naphthylacetylspermine	AMM3129	C13674	NA	1-Naphthylacetylspermine	C22H34N4O	370.2722	-
metab_1681	1.7128	370.1963	pos	1.5227	5.4891	0.0024	Corydaline	AMM3115	C15530||C08714	Alkaloids	Corydaline	C22H27NO4	369.1937	-
metab_657	5.8907	370.2943	pos	1.3809	2.9700	0.0072	(Z)-myristoleoylcarnitine	AMM3120	NA	NA	O-[(9Z)-Tetradecenoyl]-L-carnitine	C21H39NO4	369.2878	-
metab_2270	4.0479	370.3055	pos	1.2661	12.2837	0.0076	(Z)-myristoleoylcarnitine	AMM3120	NA	NA	(Z)-myristoleoylcarnitine	C21H39NO4	369.2878	-
metab_4783	4.6851	371.2209	pos	1.7957	6.6945	0.0099	CHEBI:66791	AMM3122	NA	NA	CHEBI:66791	C20H26N4O3	370.1989	-
metab_13274	3.3631	371.2446	neg	1.9372	6.1356	0.0007	8,8a-Deoxyoleandolide	AMM3152	C11989	NA	8,8a-Deoxyoleandolide	C20H36O6	372.2514	-
metab_3064	9.5283	371.2782	pos	1.2961	-1.9339	0.0076	1-Naphthylacetylspermine	NA	NA	NA	1-Naphthylacetylspermine	C22H34N4O	370.2718	-
metab_126	6.0107	371.2783	pos	1.2284	-1.8046	0.0037	1-Naphthylacetylspermine	NA	NA	NA	1-Naphthylacetylspermine	C22H34N4O	370.2718	-
metab_4325	7.0816	371.2783	pos	1.0831	-1.6074	0.0263	1-Naphthylacetylspermine	NA	NA	NA	1-Naphthylacetylspermine	C22H34N4O	370.2718	HMDB0035379
metab_4325	7.0816	371.2783	pos	1.0831	-1.6074	0.0263	Sterebin E	NA	NA	NA	Sterebin E	C20H34O4	338.2457	HMDB0035379
metab_6418	0.5420	372.2345	pos	1.3511	13.6484	0.0134	7-O-Acetylsalutaridinol	AMM3140	C05322||C16708||C11250||C10569||C16713||C09371	Alkaloids	7-O-Acetylsalutaridinol	C21H25NO5	371.1731	-
metab_6418	0.5420	372.2345	pos	1.3511	13.6484	0.0134	Isoandrocymbine	AMM3139	C16708||C10569||C11250||C05322	Alkaloids	Isoandrocymbine	C21H25NO5	371.1731	-

									‖C16713‖C09371					
metab_4549	5.9361	372.3097	pos	1.1538	-1.1034	0.0414	Myristoylcarnitine	NA	NA	NA	Myristoylcarnitine	C21H41NO4	371.3033	-
metab_129	6.3885	372.3099	pos	1.1436	1.9452	0.0186	Myristoylcarnitine	NA	NA	NA	Myristoylcarnitine	C21H41NO4	371.3033	-
metab_5943	1.3579	376.1736	pos	2.4935	11.0940	0.0088	Glu-Asp-Ile	AMM3192	NA	NA	Glu-Asp-Ile	C15H25N3O8	375.1644	-
metab_11889	8.3055	377.1434	neg	1.4363	-1.5301	0.0007	Abafungin	NA	NA	NA	Abafungin	C21H22N4OS	378.1500	-
metab_8859	2.6678	379.2131	neg	2.3811	9.9146	0.0000	Bicyclo-PGE2	NA	NA	NA	Bicyclo-PGE2	C20H30O4	334.2144	LMFA03010034; HMDB0060054
metab_2585	6.3885	379.3310	pos	1.5467	15.8639	0.0179	1-Arachidonoylglycerol	AMM3246	C13857‖C13856	NA	1-Arachidonoylglycerol	C23H38O4	378.2767	-
metab_4016	8.5554	379.3353	pos	1.3347	-2.4924	0.0123	Ergosterol	NA	NA	NA	Ergosterol	C28H44O	396.3392	LMST01030093; HMDB0000878
metab_292	1.2162	380.1473	pos	1.3333	3.0386	0.0019	Angoline	NA	NA	NA	Angoline	C22H21NO5	379.1418	-
metab_8330	1.5297	380.1562	neg	1.2985	-1.6070	0.0007	cis-Zeatin-O-glucoside	NA	NA	NA	cis-Zeatin-O-glucoside	C16H23N5O6	381.1638	-
metab_14435	1.3089	380.1565	neg	1.2513	-1.8990	0.0058	cis-Zeatin-O-glucoside	NA	NA	NA	cis-Zeatin-O-glucoside	C16H23N5O6	381.1638	-
metab_5108	3.3032	381.2265	pos	1.4599	3.6625	0.0313	CHEBI:67681	AMM3265	NA	Terpenoids_pubchem	Nervosanin A	C21H32O6	380.2194	-
metab_2061	3.0877	381.2267	pos	1.3896	3.0652	0.0259	CHEBI:67681	AMM3265	NA	Terpenoids_pubchem	Nervosanin A	C21H32O6	380.2194	-

metab_12785	5.0002	381.2287	neg	1.4431	4.4702	0.0052	Sarcostin	AMM3282	C17770	NA	Sarcostin	C21H34O6	382.2353	-
metab_13425	2.9931	381.2289	neg	2.3524	9.6395	0.0000	Lys Pro His	AMM3284	NA	NA	Lys Pro His	C17H28N6O4	382.2392	-
metab_13425	2.9931	381.2289	neg	2.3524	9.6395	0.0000	17,18-DiHETE	NA	NA	NA	17,18-DiHETE	C20H32O4	336.2301	LMFA03060078; HMDB0010211
metab_6856	1.2663	382.1009	neg	1.0850	-1.6308	0.0071	Succinyladenosine	NA	NA	NA	Succinyladenosine	C14H17N5O8	383.1075	-
metab_4842	4.4272	384.2733	pos	1.7396	2.6364	0.0006	13-Hydroxy-9-methoxy-10-oxo-11-octadecenoic acid	NA	NA	NA	13-Hydroxy-9-methoxy-10-oxo-11-octadecenoic acid	C19H34O5	342.2406	HMDB0040901
metab_2823	8.0988	385.3050	pos	1.0743	-1.2757	0.0360	N-Palmitoyl glutamine	NA	NA	NA	N-Palmitoyl glutamine	C21H40N2O4	384.2988	LMFA08020127
metab_11386	9.7312	385.3328	neg	1.6888	15.0087	0.0056	10-Undecen-1-OL	NA	NA	NA	10-Undecen-1-OL	C11H22O	170.1671	LMFA05000587; HMDB0031016
metab_5900	1.4429	387.2228	pos	1.5219	14.0456	0.0028	Citroside B	AMM3332	NA	Terpenoids_pubchem	Citroside B	C19H30O8	386.1938	-
metab_2189	3.6384	387.2997	pos	1.4613	11.9413	0.0160	6-Deoxyerythronolide B	AMM3338	C03240	NA	6-Deoxyerythronolide B	C21H38O6	386.2668	-
metab_1624	1.5424	389.1695	pos	1.2695	13.7874	0.0206	Tuberonic acid glucoside	AMM3352	C08558	NA	Tuberonic acid glucoside	C18H28O9	388.1736	-
metab_14234	1.5439	393.1340	neg	1.1173	-1.1406	0.0000	n-Acetylserotonin glucuronide	NA	NA	NA	n-Acetylserotonin glucuronide	C18H22N2O8	394.1376	HMDB0060833
metab_6961	4.3882	393.1956	neg	1.2973	2.7863	0.0093	12,20-Dioxo-leukotriene B4	NA	NA	NA	12,20-Dioxo-leukotriene B4	C20H28O5	348.1937	HMDB0060094
metab_10042	7.7404	393.2764	neg	1.4681	-1.6853	0.0002	2-Hydroxydocosanoic acid	NA	NA	NA	2-Hydroxydocosanoic acid	C22H44O3	356.3290	HMDB0061660

metab_6019	1.2162	395.1551	pos	1.7282	4.3198	0.0003	SCHEMBL16892136	AMM3430	NA	NA	SCHEMBL16892136	C21H19F N4O3	394.145	-
metab_7634	2.6841	395.2080	neg	1.6189	4.2648	0.0021	20-Oxoleukotriene B4	NA	NA	NA	20-Oxoleukotriene B4	C20H30O5	350.2093	LMFA03020064; HMDB0012641
metab_11913	8.2409	395.2444	neg	1.0036	-1.0836	0.0321	Ascorbyl palmitate	NA	NA	NA	Ascorbyl palmitate	C22H38O7	414.2618	LMFA07010788; HMDB0039883
metab_2455	5.3311	399.2269	pos	1.6521	14.4102	0.0017	His Leu Gln	AMM3472	NA	NA	His Leu Gln	C17H28N6 O5	398.2341	-
metab_11339	9.8947	399.3484	neg	2.2050	5.9744	0.0005	Tricosanoic acid	NA	NA	NA	Tricosanoic acid	C23H46O2	354.3498	HMDB0001160; LMFA01010023
metab_9722	6.3427	400.3071	neg	2.0286	4.2442	0.0044	Pristanoylglycine	NA	NA	NA	Pristanoylglycine	C21H41N O3	355.3086	HMDB0013303
metab_4863	4.3371	402.2840	pos	1.2009	1.3952	0.0095	Myriocin	NA	NA	NA	Myriocin	C21H39N O6	401.2777	-
metab_2181	3.5923	402.2840	pos	1.7681	13.2870	0.0001	Myriocin	AMM3495	C19914	NA	Myriocin	C21H39N O6	401.2776	-
metab_4991	3.7447	402.2841	pos	1.4907	13.5197	0.0029	Myriocin	AMM3496	C19914	NA	Myriocin	C21H39N O6	401.2777	-
metab_1944	2.5938	403.2532	pos	2.7241	12.8314	0.0000	Ala-Leu-Leu-Ser	AMM3509	NA	NA	Ala-Leu-Leu-Ser	C18H34N4 O6	402.2478	-
metab_12841	4.8049	403.2705	neg	1.1462	-2.2170	0.0163	3R-Hydroxy-15-(3'R,5'R -dihydroxy-6'S-methyl-(2H)-tetrahydropyran-2'-y loxy)-pentadecanoic acid	NA	NA	NA	3R-Hydroxy-15-(3'R,5 'R-dihydroxy-6'S-meth yl-(2H)-tetrahydropyra n-2'-yloxy)-pentadeca noic acid	C21H40O7	404.2776	HMDB0005076
metab_12841	4.8049	403.2705	neg	1.1462	-2.2170	0.0163	3R-Hydroxy-9R-(3'R,5'R	NA	NA	NA	3R-Hydroxy-9R-(3'R,	C21H40O7	404.2772	HMDB0005076

							-dihydroxy-6'S-methyl-(2H)-tetrahydropyran-2'-yloxy)-decanoic acid				5'R-dihydroxy-6'S-methyl-(2H)-tetrahydropyran-2'-yloxy)-decanoic acid			
metab_6347	0.6123	404.2061	pos	1.2213	5.4266	0.0142	20-Hydroxy-fusarin	NA	NA	NA	20-Hydroxy-fusarin	C22H29NO6	403.1993	-
metab_1693	1.7271	404.2132	pos	1.6175	4.0629	0.0000	20-hydroxy-fusarin	AMM3523	NA	NA	20-hydroxy-fusarin	C22H29NO6	403.1993	-
metab_6931	3.8164	405.2287	neg	1.4909	5.2287	0.0000	Lys Pro Tyr	AMM3563	NA	NA	Lys Pro Tyr	C20H30N4O5	406.233	-
metab_11982	8.0688	409.2367	neg	1.0593	-1.0051	0.0001	1-Palmitoylglycerol 3-phosphate	NA	NA	NA	1-Palmitoylglycerol 3-phosphate	C19H39O7P	410.2436	-
metab_4567	5.8597	409.2552	pos	1.3314	-2.9974	0.0069	Lys-Val-Tyr	NA	NA	NA	Lys-Val-Tyr	C20H32N4O5	408.2486	-
metab_4567	5.8597	409.2552	pos	1.3314	-2.9974	0.0069	Val-Lys-Tyr	NA	NA	NA	Val-Lys-Tyr	C20H32N4O5	408.2486	-
metab_10264	8.6093	409.2967	neg	2.0005	-3.4038	0.0001	Sorbitan oleate	NA	NA	NA	Sorbitan oleate	C24H44O6	428.3138	LMFA07011018; HMDB0029886
metab_4611	5.5864	411.2996	pos	1.6038	5.5970	0.0003	1-Palmitoyl Lysophosphatidic Acid	AMM3606	NA	NA	1-Palmitoyl Lysophosphatidic Acid	C19H39O7P	410.2436	-
metab_11618	9.0067	411.3123	neg	1.5055	-1.9709	0.0009	2-(3,4-Dihydroxyoxolan-2-yl)-2-hydroxyethyl octadecanoate	NA	NA	NA	2-(3,4-Dihydroxyoxolan-2-yl)-2-hydroxyethyl octadecanoate	C24H46O6	430.3294	HMDB0029888; LMFA07011020
metab_8989	3.0101	413.2776	neg	2.2016	8.1333	0.0000	(E)-17-[(2R,3R,5R,6S)-3,5-dihydroxy-6-methylox	AMM3631	NA	NA	(E)-17-[(2R,3R,5R,6S)-3,5-dihydroxy-6-meth	C23H42O6	414.2986	-

							an-2-yl]oxyheptadec-2-enoic acid				yloxan-2-yl]oxyheptadec-2-enoic acid			
metab_8090	1.1069	415.1362	neg	1.3613	3.4120	0.0149	Lophotoxin	AMM3647	C13680‖C10544	Phenylpropanoids	Lophotoxin	C22H24O8	416.1454	-
metab_13629	2.5431	415.2343	neg	1.8924	15.1129	0.0005	Distigmine	AMM3655	C16823‖C17864	NA	Distigmine	C22H32N4O4	416.2414	-
metab_13494	2.8269	415.2346	neg	2.4766	7.6452	0.0000	Distigmine	AMM3655	C16823‖C17864	NA	Distigmine	C22H32N4O4	416.2414	-
metab_13686	2.4338	417.2501	neg	1.3194	13.1471	0.0265	Erythronolide A	AMM3668	NA	NA	Erythronolide A	C21H38O8	418.2564	-
metab_7638	2.7155	417.2502	neg	2.1232	14.7395	0.0017	Erythronolide A	AMM3668	NA	NA	Erythronolide A	C21H38O8	418.2564	-
metab_12062	7.8193	421.2368	neg	1.7007	3.1522	0.0014	2,2-Dibutyl-3-(4-methoxyphenyl)-4-methyl-2H-1-benzopyran-7-ol acetate	AMM3733	C15061	NA	2,2-Dibutyl-3-(4-methoxyphenyl)-4-methyl-2H-1-benzopyran-7-ol acetate	C27H34O4	422.2440	-
metab_644	5.4662	423.2741	pos	1.3710	2.5674	0.0256	Lovastatin acid	AMM3738	C21130	NA	Lovastatin acid	C24H38O6	422.2665	-
metab_9670	6.0346	423.2870	neg	1.2480	-1.1828	0.0047	2-Hydroxy-1-(hydroxymethyl)ethyl icosanoate	NA	NA	NA	2-Hydroxy-1-(hydroxymethyl)ethyl icosanoate	C23H46O4	386.3396	HMDB0011542
metab_694	6.8886	423.3091	pos	1.6396	5.6119	0.0393	19-(3-methyl-butanoyloxy)-villanovane-13alpha,17-diol	AMM3739	NA	NA	19-(3-methyl-butanoyloxy)-villanovane-13alpha,17-diol	C25H42O5	422.303	-
metab_1356	0.7801	424.1009	pos	1.5419	2.8173	0.0006	S-(1,2-Dicarboxyethyl)glutathione	NA	NA	NA	S-(1,2-Dicarboxyethyl)glutathione	C14H21N3O10S	423.0948	-
metab_4927	4.0338	427.2585	pos	1.3881	12.9336	0.0211	Methylcarbamyl PAF C-8	AMM3772	NA	NA	Methylcarbamyl PAF C-8	C18H39N2O7P	426.2382	-
metab_11980	8.0688	427.2715	neg	1.2547	-1.4289	0.0001	Prostaglandin F2alpha	NA	NA	NA	Prostaglandin F2alpha	C23H40O7	428.2776	-

							1-glyceryl ester				1-glyceryl ester			
metab_9190	3.6980	429.2288	neg	1.7461	6.9573	0.0002	Ala-Leu-Leu-Asp	AMM3809	NA	NA	Ala-Leu-Leu-Asp	C19H34N4O7	430.2428	-
metab_1304	0.5983	430.1922	pos	1.3868	14.4705	0.0042	periglaucine D	AMM3796	NA	NA	periglaucine D	C23H27NO7	429.1789	-
metab_5278	2.7653	431.2494	pos	2.1390	13.0420	0.0000	Ala-Leu-Leu-Asp	AMM3809	NA	NA	Ala-Leu-Leu-Asp	C19H34N4O7	430.2428	-
metab_5954	1.3437	434.1579	pos	1.7390	3.2495	0.0001	Cyclic N-Acetylserotonin glucuronide	NA	NA	NA	Cyclic N-Acetylserotonin glucuronide	C18H20N2O8	392.1220	HMDB0060812
metab_9484	4.9349	439.2472	neg	1.4080	-2.5788	0.0258	Scoparic acid A	NA	NA	NA	Scoparic acid A	C27H36O5	440.2544	-
metab_4202	7.7514	441.3311	pos	1.5532	4.3096	0.0054	Scoparic acid A	AMM3915	NA	Terpenoids_pubchem	Scoparic acid A	C27H36O5	440.2544	-
metab_5165	3.1191	443.2783	pos	2.2845	8.3423	0.0035	Lys Phe Phe	AMM3938	NA	NA	Lys Phe Phe	C24H32N4O4	442.2604	-
metab_2180	3.5923	443.2785	pos	2.4580	8.8169	0.0001	2-glyceryl-prostaglandin G2	AMM3937	NA	NA	2-glyceryl-prostaglandin G2	C23H38O8	442.2572	HMDB0062591
metab_13822	2.1630	444.1730	neg	1.4829	7.3732	0.0160	glipizide	AMM3959	NA	NA	glipizide	C21H27N5O4S	445.1799	HMDB0015200
metab_8263	1.4427	445.1405	neg	1.6699	2.4745	0.0205	Calycosin 7-O-Glucoside	NA	NA	Flavonoids_pubchem	Calycosin 7-O-Glucoside	C22H22O10	446.121297	-
metab_4595	5.6934	447.3095	pos	1.9776	8.1048	0.0183	MC-207,110	AMM3974	C11602	NA	MC-207,110	C25H30N6O2	446.2433	-
metab_7310	8.7031	449.2681	neg	1.6035	-3.2417	0.0014	Wilforol B	NA	NA	NA	Wilforol B	C29H38O4	450.2746	-
metab_5123	3.2420	449.2751	pos	1.8889	12.3211	0.0028	Kaempferol-3-O-glucoside	AMM3986	NA	Flavonoids	Kaempferol-3-O-glucoside	C21H20O11	448.1011	HMDB0037429

metab_5123	3.2420	449.2751	pos	1.8889	12.3211	0.0028	16alpha,17alpha-Dihydroxyprogesterone acetophenide	AMM3990	C14674	NA	16alpha,17alpha-Dihydroxyprogesterone acetophenide	C29H36O4	448.2588	-
metab_7072	8.0998	452.2790	neg	1.1187	-1.1845	0.0001	LysoPE(0:0/16:0)	NA	NA	NA	LysoPE(0:0/16:0)	C21H44NO7P	453.2853	HMDB0011503
metab_12438	6.5829	453.2651	neg	1.6341	3.9542	0.0001	CHEBI:67291	AMM4037	NA	Terpenoids_pubchem	CHEBI:67291	C28H38O5	454.2702	-
metab_2666	6.9638	453.3202	pos	1.5611	13.8168	0.0037	lucialdehyde B	AMM4021	NA	Terpenoids_pubchem	lucialdehyde B	C30H44O3	452.3287	-
metab_22	8.0988	454.2917	pos	1.0799	-1.4470	0.0008	LysoPE(0:0/16:0)	NA	NA	NA	LysoPE(0:0/16:0)	C21H44NO7P	453.2853	LMGP02050002; HMDB0011503
metab_1302	0.5983	455.1148	pos	2.0446	-4.2200	0.0003	Neurodazine	AMM4030	NA	NA	Neurodazine	C27H21ClN2O3	454.1090	-
metab_8471	1.7572	455.1686	neg	2.1246	-4.2390	0.0002	Kushenol G	AMM4056	NA	Flavonoids_pubchem	Kushenol G	C25H28O8	456.1766	-
metab_8471	1.7572	455.1686	neg	2.1246	-4.2390	0.0002	Methotrexate	AMM4055	NA	NA	Methotrexate	C20H22N8O5	456.1766	-
metab_2314	4.3818	455.2781	pos	1.6782	3.8588	0.0020	Withanolide B	NA	NA	NA	Withanolide B	C28H38O5	454.2719	HMDB0030020
metab_2962	8.8759	455.3874	pos	1.0749	-1.5742	0.0191	6-Hydroxy-8-heptacosanone	NA	NA	NA	6-Hydroxy-8-heptacosanone	C27H54O2	410.4124	HMDB0035617
metab_1558	1.3865	457.1903	pos	1.7815	5.5848	0.0025	Kushenol G	AMM4056	NA	Flavonoids_pubchem	Kushenol G	C25H28O8	456.1766	-
metab_14530	1.1940	460.2058	neg	1.1525	5.3406	0.0071	Pimozide	AMM4100	C07566	NA	Pimozide	C28H29F2N3O	461.2262	HMDB0015232
metab_10021	7.6951	460.2838	neg	1.8415	-3.7302	0.0000	3-[(9E,12E)-Octadeca-9,12-dienoyloxy]-4-(trimet	NA	NA	NA	3-[(9E,12E)-Octadeca-9,12-dienoyloxy]-4-(tr	C25H45NO4	423.3349	LMFA07070078; HMDB0006461

							hylazaniumyl)butanoate				imethylazaniumyl)butanoate			
metab_2095	3.2274	462.1894	pos	1.2766	2.0943	0.0014	Trp-Glu-Gln	NA	NA	NA	Trp-Glu-Gln	C21H27N5O7	461.1836	-
metab_12176	7.5087	463.2473	neg	2.2894	-5.3326	0.0000	CHEBI:67690	AMM4119	NA	Terpenoids_pubchem	(+)-(12E,2S,3S,4R,5R,6R,9S,11S,15R)-3-Cinnamoyloxy-5,6-epoxylathyr-12-en-15-ol-14-one	C29H36O5	464.2546	-
metab_12176	7.5087	463.2473	neg	2.2894	-5.3326	0.0000	CHEBI:67698	AMM4120	NA	Terpenoids_pubchem	(-)-(6Z,12E,2S,3S,4R,5R,9S,11S,15R)-3-Cinnamoyloxylathyra-6,12-diene-5,15-diol-14-one	C29H36O5	464.2547	-
metab_4801	4.6096	463.3045	pos	2.2244	16.4667	0.0001	ponasterone A	AMM4109	NA	NA	ponasterone A	C27H44O6	462.2955	-
metab_13068	3.9850	465.2266	neg	1.2895	-3.0383	0.0209	Dolichyl b-D-glucosyl phosphate	NA	NA	NA	Dolichyl b-D-glucosyl phosphate	C21H39O9P	466.2332	HMDB0001054
metab_12856	4.7546	471.2758	neg	1.2750	2.5036	0.0018	Ixocarpanolide	NA	NA	NA	Ixocarpanolide	C28H40O6	472.2825	HMDB0033899
metab_6947	4.2203	471.2758	neg	1.2877	2.8325	0.0065	Vamonolide	NA	NA	NA	Vamonolide	C28H40O6	472.2825	HMDB0037379
metab_8204	1.3231	473.1896	neg	2.3536	5.3893	0.0014	Picrasin A	AMM4173	C17049||C10671	Terpenoids	Picrasin A	C26H34O8	474.2257	-
metab_9401	4.5884	473.2912	neg	1.0902	2.0674	0.0031	(1R,2S,4S,6R,7S,8R,9S,12S,13S,16S,18R)-16-Hydroxy-7,9,13-trimethyl-5'-methylidenespiro[5-oxapentacyclo[10.8.0.02,9.04,8.013,18]icosane-6,2'-	NA	NA	NA	(1R,2S,4S,6R,7S,8R,9S,12S,13S,16S,18R)-16-Hydroxy-7,9,13-trimethyl-5'-methylidenespiro[5-oxapentacyclo[10.8.0.02,9.04,8.013,18	C27H40O4	428.2927	HMDB0035506

							oxane]-10-one				]icosane-6,2'-oxane]-10-one			
metab_6974	4.9510	473.2914	neg	1.0964	2.0438	0.0030	Pubescenol	NA	NA	NA	Pubescenol	C28H42O6	474.2981	LMPR0104300001;HMDB0030085
metab_9923	7.2377	474.2630	neg	1.3022	1.9421	0.0000	Ulipristal acetate	NA	NA	NA	Ulipristal acetate	C30H37NO4	475.2691	HMDB0041085
metab_4810	4.5785	475.3044	pos	1.3659	2.7176	0.0022	Riesling acetal	NA	NA	NA	Riesling acetal	C13H22O3	226.1569	HMDB0037562
metab_11664	8.8449	475.3072	neg	1.3012	2.4142	0.0007	(25R)-3beta-Hydroxycholest-5-en-7-one-26-oate	NA	NA	NA	(25R)-3beta-Hydroxycholest-5-en-7-one-26-oate	C27H42O4	430.3083	HMDB0062613
metab_10236	8.5135	475.3073	neg	1.0443	1.5186	0.0008	(1S)-1,25-Dihydroxy-24-oxocalciol	NA	NA	NA	(1S)-1,25-Dihydroxy-24-oxocalciol	C27H42O4	430.3083	HMDB0060128
metab_2713	7.2314	476.2761	pos	1.3146	1.7789	0.0000	Ulipristal acetate	NA	NA	NA	Ulipristal acetate	C30H37NO4	475.2691	-
metab_4907	4.0939	476.2854	pos	2.2673	12.2288	0.0008	Ulipristal acetate	AMM4182	NA	NA	Ulipristal acetate	C30H37NO4	475.2691	-
metab_1749	1.9609	476.3053	pos	1.2928	2.1150	0.0075	Netilmicin	AMM4183	C07657	NA	Netilmicin	C21H41N5O7	475.2995	-
metab_11922	8.1936	478.2661	neg	1.1312	10.3189	0.0000	Glycerophospho-N-Oleoyl Ethanolamine	AMM4217	NA	NA	Glycerophospho-N-Oleoyl Ethanolamine	C23H46NO7P	479.3017	-
metab_7338	8.1936	478.2949	neg	1.2480	1.9876	0.0003	Glycerophospho-N-Oleoyl Ethanolamine	NA	NA	NA	Glycerophospho-N-Oleoyl Ethanolamine	C23H46NO7P	479.3017	-
metab_4112	8.1857	480.3078	pos	1.2548	1.8247	0.0009	1-(9Z-Octadecenoyl)-sn-glycero-3-phosphoethanolamine	NA	NA	NA	1-(9Z-Octadecenoyl)-sn-glycero-3-phosphoethanolamine	C23H46NO7P	479.3012	LMGP02050004

metab_9999	7.6009	483.2726	neg	1.7099	-2.6075	0.0000	Stigmatellin Y	NA	NA	NA	Stigmatellin Y	C29H40O6	484.2800	-
metab_9360	4.3716	487.2709	neg	1.2858	2.6962	0.0018	Muzanzagenin	NA	NA	NA	Muzanzagenin	C27H38O5	442.2719	HMDB0032601
metab_591	4.3818	489.2837	pos	1.3206	2.1349	0.0001	3alpha,7alpha,12alpha-Trihydroxy-5beta-cholestan-26-oic acid	NA	NA	NA	3alpha,7alpha,12alpha-Trihydroxy-5beta-cholestan-26-oic acid	C27H46O5	450.3345	HMDB0000601
metab_2197	3.6835	489.2838	pos	1.9928	4.9324	0.0009	2,3-Dihydrowithanolide E	NA	NA	NA	2,3-Dihydrowithanolide E	C28H40O7	488.2774	HMDB0034057
metab_531	3.3490	491.2996	pos	1.8972	4.6212	0.0012	Macrocarpal I	NA	NA	NA	CID 9983050	C28H42O7	490.2931	HMDB0041587
metab_12873	4.6722	491.3024	neg	1.2098	2.0776	0.0002	Spirotaccagenin	NA	NA	NA	Spirotaccagenin	C27H42O5	446.3032	HMDB0034424
metab_9488	4.9510	495.3335	neg	1.7537	6.7953	0.0164	Polypodine B	AMM4312	C08834||C16500	Terpenoids	Polypodine B	C27H44O8	496.3034	-
metab_11443	9.5314	497.2583	neg	2.1119	15.4687	0.0003	(14alpha,17beta,20S,22R)-14,20-Epoxy-17-hydroxy-1-oxowitha-3,5,24-trienolide	NA	NA	NA	(14alpha,17beta,20S,22R)-14,20-Epoxy-17-hydroxy-1-oxowitha-3,5,24-trienolide	C28H36O5	452.2563	HMDB0032685
metab_2380	4.7608	497.3362	pos	2.0603	8.0134	0.0001	Polypodine B	AMM4312	C08834||C16500	Terpenoids	Polypodine B	C27H44O8	496.3034	-
metab_10576	9.8947	499.2742	neg	1.2170	14.8824	0.0171	Euglobal IVa	NA	NA	NA	Euglobal IVa	C28H38O5	454.2719	HMDB0030035
metab_13200	3.5978	503.2660	neg	1.7754	4.2806	0.0007	Lucidenic acid A	NA	NA	NA	Lucidenic acid A	C27H38O6	458.2668	HMDB0037611
metab_6613	3.6812	505.2818	neg	1.6535	3.7733	0.0003	(5alpha,6beta,14alpha,20R,22R)-5,6,14,20,27-Pentahydroxy-1-oxowith-24-enolide	NA	NA	NA	(5alpha,6beta,14alpha,20R,22R)-5,6,14,20,27-Pentahydroxy-1-oxowith-24-enolide	C28H42O8	506.2880	HMDB0033198
metab_12561	6.0670	505.3028	neg	1.4110	-2.3244	0.0002	1,2-Dioctanoyl-3-beta-d-galactosyl-sn-glycerol	NA	NA	NA	1,2-Dioctanoyl-3-beta-d-galactosyl-sn-glycerol	C25H46O10	506.3098	-
metab_68	3.1191	507.2947	pos	1.6066	4.0065	0.0015	Bicyclo(4.1.0)heptan-3-one,	NA	C17492	NA	Bicyclo(4.1.0)heptan-3-one,	C15H22O2	234.1620	LMPR0103400001;HMDB00332

							1-methyl-4-(1-methylethylidene)-7-(3-oxobutyl)-, (1S,6R,7R)-				1-methyl-4-(1-methylethylidene)-7-(3-oxobutyl)-, (1S,6R,7R)-			53
metab_4203	7.7514	508.3391	pos	1.9765	4.5647	0.0000	1-(9Z-Heptadecenoyl)-glycero-3-phosphocholine	NA	NA	NA	1-(9Z-Heptadecenoyl)-glycero-3-phosphocholine	C25H50NO7P	507.3325	LMGP01050126
metab_5086	3.3793	509.3103	pos	2.2167	8.5699	0.0000	[(2S,3R,4S,5S,6R)-3,4,5-trihydroxy-6-(hydroxymethyl)oxan-2-yl] (11R)-11-[(2R,3R,5R,6S)-3,5-dihydroxy-6-methyloxan-2-yl]oxydodecanoate	AMM4380	NA	NA	[(2S,3R,4S,5S,6R)-3,4,5-trihydroxy-6-(hydroxymethyl)oxan-2-yl] (11R)-11-[(2R,3R,5R,6S)-3,5-dihydroxy-6-methyloxan-2-yl]oxydodecanoate	C24H44O11	508.2887	-
metab_7383	7.0959	509.3494	neg	1.2737	-2.2437	0.0028	Trihydroxycoprostanoic acid	NA	NA	NA	Trihydroxycoprostanoic acid	C28H48O5	464.3502	HMDB0002163; LMST01010246; HMDB0000601
metab_4065	8.3463	510.3546	pos	1.4101	2.3550	0.0001	1-Heptadecanoyl-sn-glycero-3-phosphocholine	NA	NA	NA	1-Heptadecanoyl-sn-glycero-3-phosphocholine	C25H52NO7P	509.3473	-
metab_2205	3.6987	510.3777	pos	1.7841	14.2447	0.0010	1-heptadecanoyl-sn-glycero-3-phosphocholine	AMM4386	NA	NA	1-heptadecanoyl-sn-glycero-3-phosphocholine	C25H52NO7P	509.3473	HMDB0012108
metab_12717	5.3713	512.3002	neg	1.9127	4.1307	0.0010	1-Heptadecanoyl-sn-glycero-3-phosphoethanolamine	NA	NA	NA	1-Heptadecanoyl-sn-glycero-3-phosphoethanolamine	C22H46NO7P	467.3012	HMDB0061691
metab_8081	1.0785	515.1986	neg	1.0702	4.5669	0.0168	Formononetin	AMM4421	NA	Flavonoids	Formononetin	C25H24O1	516.1266	-

							7-O-glucoside-6''-O-malonate				7-O-glucoside-6''-O-malonate	2		
metab_2580	6.2978	517.2412	pos	1.1624	12.9818	0.0227	Formononetin 7-O-glucoside-6''-O-malonate	AMM4421	NA	Flavonoids	Formononetin 7-O-glucoside-6''-O-malonate	C25H24O12	516.1266	-
metab_2580	6.2978	517.2412	pos	1.1624	12.9818	0.0227	hesperadin	AMM4423	NA	NA	hesperadin	C29H32N4O3S	516.2219	-
metab_2707	7.1701	518.3230	pos	1.3304	2.1575	0.0000	LysoPC(18:3(9Z,12Z,15Z))	NA	NA	NA	LysoPC(18:3(9Z,12Z,15Z))	C26H48NO7P	517.3168	HMDB0010388
metab_13208	3.5813	521.2767	neg	1.7662	3.9537	0.0034	Lucidenic acid G	NA	NA	NA	Lucidenic acid G	C27H40O7	476.2774	HMDB0035599
metab_10050	7.7875	522.2846	neg	1.3348	2.6943	0.0063	1-(9Z-Octadecenoyl)-sn-glycero-3-phosphoserine	NA	NA	NA	1-(9Z-Octadecenoyl)-sn-glycero-3-phosphoserine	C24H46NO9P	523.2910	LMGP03050001
metab_116	8.0988	522.3544	pos	1.2035	1.9953	0.0002	LysoPC(18:1(11Z))	NA	NA	NA	LysoPC(18:1(11Z))	C26H52NO7P	521.3481	-
metab_7467	1.5155	523.1677	neg	1.1456	-1.4727	0.0011	Isoglobotriaose	NA	NA	NA	Isoglobotriaose	C19H34O15	502.1898	HMDB0006598
metab_9561	5.4188	523.3283	neg	1.4469	4.3431	0.0129	Polyporusterone A	NA	NA	NA	CID 10814524	C28H46O6	478.3294	HMDB0038495
metab_12138	7.6169	526.3160	neg	1.4813	2.6849	0.0001	LysoPC(15:0)	NA	NA	NA	NA	C23H48NO7P	481.3168	HMDB0010381
metab_10096	7.9757	531.3189	neg	1.2557	-1.2608	0.0065	3-O-(alpha-L-Olivosyl)erythronolide B	NA	NA	NA	3-O-(alpha-L-Olivosyl)erythronolide B	C27H48O10	532.3253	-
metab_13140	3.7658	535.2922	neg	1.5624	3.8217	0.0016	Corchorosol A	NA	NA	NA	Corchorosol A	C29H44O9	536.2985	HMDB0041137
metab_2872	8.3322	536.3710	pos	1.7121	3.7065	0.0000	CHEBI:68080	AMM4519	NA	NA	CHEBI:68080	C28H49N5O5	535.372	-
metab_10587	9.9433	539.4691	neg	1.2871	2.2289	0.0060	1,2-Dipentadecanoyl-sn-	NA	NA	NA	1,2-Dipentadecanoyl-s	C33H64O5	540.4754	LMGL02010326;

							glycerol				n-glycerol			HMDB0007068
metab_6939	4.0516	540.3119	neg	1.2174	-1.5608	0.0007	LysoPC(20:5(5Z,8Z,11Z,14Z,17Z))	NA	NA	NA	LysoPC(20:5(5Z,8Z,11Z,14Z,17Z))	C28H48NO7P	541.3168	HMDB0010397
metab_10054	7.7875	543.2266	neg	1.3902	-1.6910	0.0046	10-Deacetylbaccatin III	NA	NA	NA	10-Deacetylbaccatin III	C29H36O10	544.2320	-
metab_12012	7.9908	543.2271	neg	1.2658	-1.3193	0.0057	10-Deacetylbaccatin III	NA	NA	NA	10-Deacetylbaccatin III	C29H36O10	544.2320	-
metab_13164	3.6980	551.2874	neg	1.9892	4.9246	0.0012	Desglucocheirotoxol	NA	NA	NA	Desglucocheirotoxol	C29H44O10	552.2934	HMDB0033828
metab_883	10.3480	551.5023	pos	1.4358	2.7573	0.0226	1,2-Dipalmitoyl-sn-glycerol	NA	C00165	NA	1,2-Dipalmitoyl-sn-glycerol	C35H68O5	568.5067	HMDB0007098; LMGL02010009
metab_2155	3.4857	552.3519	pos	1.6479	4.2537	0.0006	Janthitrem C	NA	C20601	NA	Janthitrem C	C37H47NO4	569.3505	HMDB0040684
metab_726	7.4269	555.2911	pos	1.7620	-3.6762	0.0001	Acrovestone	NA	NA	NA	Acrovestone	C32H42O8	554.2854	-
metab_4790	4.6398	556.3044	pos	1.1043	1.6274	0.0034	3-Hydroxydodecanedioic acid	NA	NA	NA	3-Hydroxydodecanedioic acid	C12H22O5	246.1467	HMDB0000413
metab_9904	7.1595	562.3153	neg	1.3229	2.2064	0.0008	LysoPC(18:3(6Z,9Z,12Z))	NA	NA	NA	LysoPC(18:3(6Z,9Z,12Z))	C26H48NO7P	517.3168	HMDB0010387
metab_12199	7.4300	571.2892	neg	1.4458	-2.1596	0.0000	1-Hexadecanoyl-sn-glycero-3-phospho-(1'-myo-inositol)	NA	NA	NA	1-Hexadecanoyl-sn-glycero-3-phospho-(1'-myo-inositol)	C25H49O12P	572.2964	HMDB0061695
metab_2972	8.9367	573.4861	pos	1.4818	-2.9083	0.0117	DG(16:0/18:3(9Z,12Z,15Z)/0:0)	NA	NA	NA	DG(16:0/18:3(9Z,12Z,15Z)/0:0)	C37H66O5	590.4910	HMDB0007105
metab_3753	9.7283	575.5021	pos	1.3268	-1.9541	0.0000	1-Palmitoyl-2-linoleoyl-sn-glycerol	NA	NA	NA	1-Palmitoyl-2-linoleoyl-sn-glycerol	C37H68O5	592.5067	HMDB0007103
metab_12789	4.9838	587.3130	neg	1.6217	-2.1699	0.0461	Tyr-Ile	NA	NA	NA	Tyr-Ile	C15H22N2	294.1580	HMDB0029108

												O4		
metab_12675	5.5966	595.2899	neg	2.1671	4.7455	0.0035	Salannin	AMM4755	C08780	Terpenoids	Salannin	C34H44O9	596.2971	-
metab_7656	0.5286	606.0755	neg	1.6564	3.0581	0.0001	Uridine diphosphate-N-acetylglucosamine	NA	NA	NA	Uridine diphosphate-N-acetylglucosamine	C17H27N3O17P2	607.0816	HMDB0000290
metab_800	8.7303	613.4815	pos	1.3033	-2.6231	0.0289	2,3-Dilinolenoyl-sn-glycerol	NA	NA	NA	2,3-Dilinolenoyl-sn-glycerol	C39H64O5	612.4751	-
metab_4168	7.9695	616.3444	pos	2.1922	-5.0656	0.0005	(6r)-6-[(3s,10s,13r,14r,15r,17r)-12-Acetoxy-3,15-dihydroxy-4,4,10,13,14-pentamethyl-7,11-dioxo-2,3,5,6,12,15,16,17-octahydro-1h-cyclopenta[a]phenanthren-17-yl]-2-methyl-4-oxo-heptanoic acid	NA	NA	NA	(6r)-6-[(3s,10s,13r,14r,15r,17r)-12-Acetoxy-3,15-dihydroxy-4,4,10,13,14-pentamethyl-7,11-dioxo-2,3,5,6,12,15,16,17-octahydro-1h-cyclopenta[a]phenanthren-17-yl]-2-methyl-4-oxo-heptanoic acid	C32H46O9	574.3142	HMDB0033024
metab_2879	8.3763	620.3869	pos	1.0659	-1.6154	0.0055	3beta,15alpha-Diacetoxylanosta-8,24-dien-26-oic acid	NA	NA	NA	3beta,15alpha-Diacetoxylanosta-8,24-dien-26-oic acid	C34H52O6	556.3764	HMDB0036442
metab_10061	7.8352	625.4691	neg	1.0413	1.5050	0.0013	(2S)-2-Methyl-4-[12-[(2S,5R)-5-[(1R)-1,6,7-trihydroxytetradecyl]oxolan-2-yl]dodecyl]-2H-furan-5-one	NA	NA	NA	(2S)-2-Methyl-4-[12-[(2S,5R)-5-[(1R)-1,6,7-trihydroxytetradecyl]oxolan-2-yl]dodecyl]-2H-furan-5-one	C35H64O6	580.4703	HMDB0039453
metab_12365	6.8730	641.4641	neg	1.2054	2.4429	0.0393	(2S)-4-[(2R)-2-Hydroxy-12-[(2R,5S)-5-[(1S)-1,8,	NA	NA	NA	(2S)-4-[(2R)-2-Hydroxy-12-[(2R,5S)-5-[(1S	C35H64O7	596.4652	HMDB0036977

							9-trihydroxytetradecyl]oxolan-2-yl]dodecyl]-2-methyl-2H-furan-5-one				)-1,8,9-trihydroxytetradecyl]oxolan-2-yl]dodecyl]-2-methyl-2H-furan-5-one			
metab_11686	8.7640	647.4679	neg	1.2230	2.1020	0.0081	1-Pentadecanoyl-2-(4Z,7Z,10Z,13Z,16Z,19Z-docosahexaenoyl)-sn-glycerol	NA	NA	NA	1-Pentadecanoyl-2-(4Z,7Z,10Z,13Z,16Z,19Z-docosahexaenoyl)-sn-glycerol	C40H66O5	626.4910	HMDB0007092
metab_4614	5.5715	649.3957	pos	1.3086	3.8393	0.0161	gypsogenate-28-beta-D-glucoside	AMM4910	NA	NA	gypsogenate-28-beta-D-glucoside	C36H56O10	648.3873	-
metab_3076	9.6358	655.4899	pos	1.5680	-2.9187	0.0005	[(2S)-2-[9-(3,4-Dimethyl-5-propylfuran-2-yl)nonanoyloxy]-3-hydroxypropyl] 11-(3,4-dimethyl-5-propylfuran-2-yl)undecanoate	NA	NA	NA	[(2S)-2-[9-(3,4-Dimethyl-5-propylfuran-2-yl)nonanoyloxy]-3-hydroxypropyl] 11-(3,4-dimethyl-5-propylfuran-2-yl)undecanoate	C41H68O7	672.4965	HMDB0116375
metab_11223	10.4016	657.4512	neg	1.2126	2.0652	0.0023	1-Pentadecanoyl-2-(9Z,12Z-octadecadienoyl)-glycero-3-phosphate	NA	NA	NA	1-Pentadecanoyl-2-(9Z,12Z-octadecadienoyl)-glycero-3-phosphate	C36H67O8P	658.4574	LMGP10010915; HMDB0114815
metab_801	8.7731	667.4528	pos	1.1513	-2.0599	0.0014	PG(16:0/12:0)	NA	NA	NA	PG(16:0/12:0)	C34H67O10P	666.4469	-
metab_3799	9.5283	668.5445	pos	1.0630	-1.4649	0.0173	1-Hexadecyl-2-(9Z-octadecenoyl)-sn-glycero-3-phosphoethanolamine	NA	NA	NA	1-Hexadecyl-2-(9Z-octadecenoyl)-sn-glycero-3-phosphoethanolamine	C39H78NO7P	703.5516	HMDB0011157

metab_13347	3.1797	670.3029	neg	1.8507	4.6599	0.0007	Leukotriene C4	NA	C02166	NA	Leukotriene C4	C30H47N3O9S	625.3033	LMFA03020003; HMDB0001198
metab_3962	8.7303	671.4842	pos	1.4635	-2.6428	0.0101	Glyceryl 2-pentadecanoate	NA	NA	NA	Glyceryl 2-pentadecanoate	C18H36O4	316.2614	HMDB0011532
metab_13631	2.5431	686.2976	neg	1.9050	6.9835	0.0000	S-(PGA2)-glutathione	NA	NA	NA	S-(PGA2)-glutathione	C30H47N3O10S	641.2982	HMDB0013062
metab_5348	2.5469	688.3096	pos	2.7037	8.0207	0.0003	Buprenorphine Glucuronide	NA	NA	NA	Buprenorphine Glucuronide	C35H49NO10	643.3356	HMDB0060928
metab_10063	7.8352	688.4653	neg	1.4532	2.3940	0.0036	Spirolide D	NA	NA	NA	Spirolide D	C43H65NO7	707.4761	HMDB0030493
metab_3935	8.8904	689.4947	pos	1.1546	-1.7161	0.0052	PA(20:3(5Z,8Z,11Z)/18:1(11Z))	NA	NA	NA	PA(20:3(5Z,8Z,11Z)/18:1(11Z))	C41H73O8P	724.5043	HMDB0115124
metab_10485	9.4647	695.5833	neg	1.1901	1.8575	0.0154	1-Behenoyl-3-palmitoleyl-sn-glycerol	NA	NA	NA	1-Behenoyl-3-palmitoleoyl-sn-glycerol	C41H78O5	650.5849	HMDB0056090
metab_11253	10.2384	700.4935	neg	1.1057	1.5787	0.0032	1-Pentadecanoyl-2-(9Z,12Z-octadecadienoyl)-glycero-3-phosphoethanolamine	NA	NA	NA	1-Pentadecanoyl-2-(9Z,12Z-octadecadienoyl)-glycero-3-phosphoethanolamine	C38H72NO8P	701.4996	HMDB0008895; LMGP02011232
metab_5321	2.6090	701.3608	pos	1.3180	12.5227	0.0103	Chalcomycin	AMM5079	C15678	NA	Chalcomycin	C35H56O14	700.3641	-
metab_1424	0.9761	739.1816	pos	1.2712	2.4234	0.0192	Rhamnocitrin 3-(5'''-p-coumarylapiosyl)-(1->2)-glucoside	AMM5191	NA	Flavonoids_pubchem	Rhamnocitrin 3-(5'''-p-coumarylapiosyl)-(1->2)-glucoside	C36H36O17	740.196	-
metab_7198	14.0282	744.4836	neg	1.2752	2.2244	0.0007	1-(9Z,12Z-Octadecadienoyl)-2-pentadecanoyl-glycero-3-phosphoserine	NA	NA	NA	1-(9Z,12Z-Octadecadienoyl)-2-pentadecanoyl-glycero-3-phosphose	C39H72NO10P	745.4894	LMGP03010344; HMDB0112425

											rine			
metab_11217	10.5015	746.4978	neg	1.3111	2.5596	0.0006	1-(9Z,12Z-Octadecadienoyl)-2-pentadecanoyl-glycero-3-phosphoethanolamine	NA	NA	NA	1-(9Z,12Z-Octadecadienoyl)-2-pentadecanoyl-glycero-3-phosphoethanolamine	C38H72NO8P	701.4996	LMGP02010657; HMDB0009087
metab_10533	9.6814	769.5034	neg	1.3356	-2.0807	0.0015	PG(18:1(11Z)/18:3(9Z,12Z,15Z))	NA	NA	NA	PG(18:1(11Z)/18:3(9Z,12Z,15Z))	C42H75O10P	770.5098	HMDB0010622
metab_10603	10.0264	782.4988	neg	1.2379	-1.3476	0.0000	PE(18:2(9Z,12Z)/18:3(9Z,12Z,15Z))	NA	NA	NA	PE(18:2(9Z,12Z)/18:3(9Z,12Z,15Z))	C41H72NO8P	737.4996	LMGP02010665; HMDB0009095
metab_7203	14.0282	782.4990	neg	1.1094	-1.1678	0.0001	1-(9Z-Tetradecenoyl)-2-eicosanoyl-glycero-3-phosphoserine	NA	NA	NA	1-(9Z-Tetradecenoyl)-2-eicosanoyl-glycero-3-phosphoserine	C40H76NO10P	761.5207	HMDB0112277; LMGP03010127
metab_14941	0.5286	783.1143	neg	1.9104	3.8695	0.0269	Astragaloside IV	AMM5250	NA	Terpenoids	Astragaloside IV	C41H68O14	784.4614	-
metab_14941	0.5286	783.1143	neg	1.9104	3.8695	0.0269	Astragaloside III	NA	NA	Terpenoids	Astragaloside III	C41H68O14	784.4614	-
metab_2932	8.6577	806.4942	pos	1.8347	-3.5123	0.0001	1-Hexadecanoyl-2-(9Z-octadecenoyl)-sn-glycero-3-phosphoserine	NA	NA	NA	1-Hexadecanoyl-2-(9Z-octadecenoyl)-sn-glycero-3-phosphoserine	C40H76NO10P	761.5207	LMGP03010024; HMDB0012357
metab_11297	10.0432	810.5299	neg	1.1732	-2.3103	0.0129	PE(20:3(5Z,8Z,11Z)/18:2(9Z,12Z))	NA	NA	NA	PE(20:3(5Z,8Z,11Z)/18:2(9Z,12Z))	C43H76NO8P	765.5309	HMDB0009324
metab_2795	7.8532	822.4895	pos	1.6707	-3.1148	0.0011	PS(DiMe(11,3)/DiMe(9,3))	NA	NA	NA	PS(DiMe(11,3)/DiMe(9,3))	C44H74NO12P	839.4949	HMDB0061555
metab_7914	0.5991	829.2955	neg	1.2464	3.8064	0.0001	Astragaloside IV	AMM5250	NA	Terpenoids	Astragaloside IV	C41H68O14	784.4609	-
metab_7914	0.5991	829.2955	neg	1.2464	3.8064	0.0001	Astragaloside III	NA	NA	Terpenoids	Astragaloside III	C41H68O1	784.4614	-

												4		
metab_10543	9.7312	833.5206	neg	1.3368	-1.7318	0.0000	1-Hexadecanoyl-2-(9Z,12Z-octadecadienoyl)-glycero-3-phospho-(1'-myo-inositol)	NA	NA	NA	1-Hexadecanoyl-2-(9Z,12Z-octadecadienoyl)-glycero-3-phospho-(1'-myo-inositol)	C43H79O13P	834.5258	LMGP06010959; HMDB0009784
metab_10537	9.6986	846.5301	neg	1.6237	-3.3427	0.0029	PE-NMe(18:2(9Z,12Z)/22:6(4Z,7Z,10Z,13Z,16Z,19Z))	NA	NA	NA	PE-NMe(18:2(9Z,12Z)/22:6(4Z,7Z,10Z,13Z,16Z,19Z))	C46H76NO8P	801.5309	HMDB0113190
metab_10488	9.4984	857.5197	neg	1.6231	-2.6014	0.0000	PI(18:1(11Z)/18:3(6Z,9Z,12Z))	NA	NA	NA	PI(18:1(11Z)/18:3(6Z,9Z,12Z))	C45H79O13P	858.5258	HMDB0009827
metab_950	9.4981	859.5304	pos	1.8006	-3.3918	0.0000	PI(18:1(11Z)/18:3(9Z,12Z,15Z))	NA	NA	NA	PI(18:1(11Z)/18:3(9Z,12Z,15Z))	C45H79O13P	858.5258	HMDB0009828
metab_11107	14.0117	867.4759	neg	1.3255	-1.4559	0.0025	Astrasieversianin IV	NA	NA	NA	Astrasieversianin IV	C45H72O16	868.4827	-
metab_3171	10.2712	878.5729	pos	1.9244	-4.9431	0.0002	Cavipetin D	NA	NA	NA	Cavipetin D	C25H38O5	418.2719	HMDB0030365
metab_4950	3.9270	903.4902	pos	1.1258	3.1100	0.0470	Torvoside H	AMM5381	NA	Terpenoids_pubchem	Torvoside H	C45H74O18	902.4867	-
metab_3039	9.3278	924.7468	pos	1.2224	2.0403	0.0197	1-2-Di-tetracosanoyl-sn-glycero-3-phosphoserine	NA	NA	NA	1-2-Di-tetracosanoyl-sn-glycero-3-phosphoserine	C54H106NO10P	959.7554	HMDB0112908
metab_758	8.0843	943.6353	pos	1.9890	3.3361	0.0156	Soyasaponin I	AMM5415	C08983||C08963||C08923||C08958||C08791	Terpenoids	Soyasaponin I	C48H78O18	942.5179	-
metab_11928	8.1936	955.5836	neg	1.6967	3.5500	0.0001	25-Acetylvulgaroside	NA	NA	NA	25-Acetylvulgaroside	C27H42O7	478.2931	HMDB0041365
metab_12025	7.9612	997.6258	neg	1.6084	3.3137	0.0000	Polyporusterone B	NA	NA	NA	Polyporusterone B	C28H44O6	476.3138	HMDB0038496
metab_751	7.9551	1043.696	pos	2.1614	6.3219	0.0001	1-(9Z-Octadecenoyl)-sn-	NA	C04230	NA	1-(9Z-Octadecenoyl)-s	C26H52N	521.3481	HMDB0002815

		4					glycero-3-phosphocholine				n-glycero-3-phosphocholine	O7P		
