# Supplementary material for: Nutritional Characterization and Untargeted Metabolomics of Oyster Mushroom Produced Using Astragalus membranaceus var. mongolicus Stems and Leaves as Substrates
Source: Front Plant Sci. 2022 Feb 3;13:802801. doi: 10.3389/fpls.2022.802801 (PMC8853653; doi:10.3389/fpls.2022.802801)
Supplement: Supplementary file 6 [file Table_4.pdf]

Table S4 Differential metabolites identified as amino acid for comparison bewteen AMM and control group.

Metab_ID	Retention_Time	Apex_m/z	Mode	VIP	Log2(Fold_Change)	P_Value	Putative_Metabolite	Putative_Formula	Pubchem_ID
**Glutamine**									
metab_2095	3.23	462.19	pos	1.28	2.09	0.00	Trp-Glu-Gln	C21H27N5O7	145458356
metab_5943	1.36	376.17	pos	2.49	11.09	0.01	Glu-Asp-Ile	C15H25N3O8	71464589
**Glycine**									
metab_1613	1.53	302.21	pos	1.93	3.38	0.00	Gly Ile Ile	C14H27N3O4	145455641
metab_1806	2.13	302.21	pos	1.35	1.59	0.01	Glycyl-L-leucyl-L-leucine	C14H27N3O4	13037660
metab_5575	2.07	288.19	pos	1.61	3.66	0.00	Gly Val Leu	C13H25N3O4	9857120
**Alanine**									
metab_5291	2.72	302.21	pos	2.86	7.11	0.00	Leu Ala Val	C14H27N3O4	54565935
metab_13624	2.54	300.19	neg	1.53	2.64	0.00	Val-Leu-Ala	C14H27N3O4	25157726
metab_5697	1.80	333.15	pos	1.47	2.65	0.03	Trp-Ala-Gly	C16H20N4O4	11990218
**Valine**									
metab_5534	2.14	318.20	pos	2.38	6.04	0.00	Leu-Val-Ser	C14H27N3O5	9858135
metab_8872	2.70	300.19	neg	1.73	3.80	0.00	Val Leu Ala	C14H27N3O4	25157726
metab_40	1.22	217.15	pos	1.09	1.04	0.00	Val-val	C10H20N2O3	107475
**Leucine and isoleucine**									
metab_1755	1.98	316.22	pos	3.39	12.75	0.00	Ile Ala Ile	C15H29N3O4	145456039
metab_5338	2.58	316.22	pos	1.64	2.64	0.00	Ile Ala Ile	C15H29N3O4	145456039
metab_14227	1.54	300.19	neg	2.03	5.63	0.00	Gly Ile Ile	C14H27N3O4	145455641
metab_1944	2.59	403.25	pos	2.72	12.83	0.00	Ala-Leu-Leu-Ser	C18H34N4O6	71464518
metab_13874	2.07	300.19	neg	1.02	1.41	0.01	Leu-Leu-Gly	C14H27N3O4	7019089
metab_2306	4.34	316.21	pos	1.33	3.47	0.00	H-LEU-LEU-ALA-OH	C15H29N3O4	7020110

**Histidine**									
metab_1810	2.13	284.13	pos	1.53	10.02	0.01	Ala-Gly-His	C11H17N5O4	71464564
metab_2455	5.33	399.23	pos	1.65	14.41	0.00	His Leu Gln	C17H28N6O5	46851994
metab_13425	2.99	381.23	neg	2.35	9.64	0.00	Lys Pro His	C17H28N6O4	145456757
**Arginine**									
metab_1153	0.54	175.12	pos	1.85	4.01	0.00	Arginine	C6H14N4O2	6322
metab_5860	1.51	361.17	pos	1.62	13.18	0.00	L-Tryptophan-L-arginine	C17H24N6O3	25186251
metab_6122	1.06	288.20	pos	1.26	2.47	0.01	Arginyl-Leucine	C12H25N5O3	6992563
**Methionine**									
metab_1744	1.95	249.13	pos	1.77	4.96	0.00	H-Val-Met-OH	C10H20N2O3S	6993039
