# Supplementary material for: Nutritional Characterization and Untargeted Metabolomics of Oyster Mushroom Produced Using Astragalus membranaceus var. mongolicus Stems and Leaves as Substrates
Source: Front Plant Sci. 2022 Feb 3;13:802801. doi: 10.3389/fpls.2022.802801 (PMC8853653; doi:10.3389/fpls.2022.802801)
Supplement: Supplementary file 7 [file Table_5.pdf]

Table S5. Physicochemical analysis of the substrates from AMM and control group.

	Substrates from AMM group	Substrates from control group
cellulose (mg/g)	203.7	219.4
hemicellulose (mg/g)	254.2	276.7
lignin (%)	15.95*	11.55
Amount of C (g/Kg)	509.9*	418.4
Amount of N (g/Kg)	11*	8.4
K (g/kg)	8.2*	4.9
Na (g/kg)	0.2	0.1
Ca (g/kg)	9.9*	5.3
Mg (g/kg)	1.9*	0.9
S (g/kg)	4.9*	0.7
P (g/kg)	3.1*	0.8
Fe (mg/kg)	507*	275
Mn (mg/kg)	50*	223
Zn (mg/kg)	25	19
B (mg/kg)	10.3	14.3
Cu (mg/kg)	3.7*	9.9
Mo (mg/kg)	1.2*	0.3
Water holding capacity (mm)	0.041	0.04
Porosity (%)	8.1	8.0
Venting quality (%)	5.3	5.9
Total density Mg/m^3	0.18	0.11
